# *Bacteroidaceae*, *Bacteroides*, and *Veillonella*: emerging protectors against Graves’ disease

**DOI:** 10.3389/fcimb.2024.1288222

**Published:** 2024-02-09

**Authors:** Siyuan Liu, Fan Li, Yunjia Cai, Linan Ren, Lin Sun, Xiaokun Gang, Guixia Wang

**Affiliations:** ^1^ Department of Endocrinology and Metabolism, The First Hospital of Jilin University, Jilin, Changchun, China; ^2^ Department of Gastroenterology, The First Hospital of Jilin University, Jilin, Changchun, China

**Keywords:** Graves’ disease, gut microbiota, autoimmune disease, Mendelian randomization, causal effect

## Abstract

**Background:**

Graves’ disease (GD) is the most common cause of hyperthyroidism, and its pathogenesis remains incompletely elucidated. Numerous studies have implicated the gut microbiota in the development of thyroid disorders. This study employs Mendelian randomization analysis to investigate the characteristics of gut microbiota in GD patients, aiming to offer novel insights into the etiology and treatment of Graves’ disease.

**Methods:**

Two-sample Mendelian randomization (MR) analysis was employed to assess the causal relationship between Graves’ disease and the gut microbiota composition. Gut microbiota data were sourced from the international consortium MiBioGen, while Graves’ disease data were obtained from FINNGEN. Eligible single nucleotide polymorphisms (SNPs) were selected as instrumental variables. Multiple analysis methods, including inverse variance-weighted (IVW), MR-Egger regression, weighted median, weighted mode, and MR-RAPS, were utilized. Sensitivity analyses were conducted employing MR-Egger intercept test, Cochran’s Q test, and leave-one-out analysis as quality control measures.

**Results:**

The Mendelian randomization study conducted in a European population revealed a decreased risk of Graves’ disease associated with *Bacteroidaceae* (Odds ratio (OR) [95% confidence interval (CI)]: 0.89 [0.89 ~ 0.90], adjusted *P* value: <0.001), *Bacteroides* (OR: [95% CI]: 0.555 [0.437 ~ 0.706], adjusted *P* value: <0.001), and *Veillonella* (OR [95% CI]: 0.632 [0.492 ~ 0.811], adjusted *P* value: 0.016). No significant evidence of heterogeneity, or horizontal pleiotropy was detected. Furthermore, the preliminary MR analysis identified 13 bacterial species including *Eubacterium brachy* group and *Family XIII AD3011* group, exhibiting significant associations with Graves’ disease onset, suggesting potential causal effects.

**Conclusion:**

A causal relationship exists between gut microbiota and Graves’ disease. *Bacteroidaceae*, *Bacteroides*, and *Veillonella* emerge as protective factors against Graves’ disease development. Prospective probiotic supplementation may offer a novel avenue for adjunctive treatment in the management of Graves’ disease in the future.

## 1 Introduction

Graves’ disease(GD) is an autoimmune disorder and the most prevalent cause of hyperthyroidism, with an age peak of onset between 30 and 50 years (Smith and Hegedus, 2016). The incidence of GD varies by region and gender (Tellez et al., 1992). Clinical manifestations of GD encompass symptoms of hyperthyroidism, such as weight loss, palpitations, fatigue, and tremor, with some patients displaying thyroid-associated orbitopathy (Smith and Hegedus, 2016). Skin manifestations and clubbing of fingers are observed in 1-4% of GD patients, often accompanied by severe thyroid-associated orbitopathy (Fatourechi, 2012). Graves’ Disease can affect multiple systems throughout the body, significantly impacting patients’ quality of life, and in severe cases, may lead to life-threatening events such as thyrotoxic crisis. The exact etiology of GD remains elusive, although it is widely accepted that a combination of genetic, autoimmune, and environmental factors contributes to its development (Lee et al., 2015; Rayman, 2019).

The adult gut harbors 50 bacterial phyla and about 100–1000 bacterial species, encoding genes that are 150 times the size of the human genome (Gill et al., 2006; Adak and Khan, 2019). These gut microbiota play pivotal roles in maintaining host nutrition, metabolism, and immune equilibrium (Kho and Lal, 2018). Dysregulation of the gut ecosystem may contribute to autoimmune and metabolic disorders, including inflammatory bowel disease (Nishino et al., 2018), irritable bowel syndrome (Cao, 2018), obesity (Schwiertz et al., 2010), chronic kidney disease (Sircana et al., 2019), and cardiovascular disease (Jie et al., 2017). In recent years, the thyroid-gut axis has garnered increasing research attention, although its precise mechanisms remain to be fully elucidated (Lerner et al., 2017).

Alterations in gut microbiota composition could potentially exert influences on thyroid function. Gong et al. conducted a meta-analysis revealing reduced abundance of beneficial bacteria such as *Bifidobacterium* and *Lactobacillus* in autoimmune thyroid diseases (AITD), accompanied by a significant increase in detrimental microbial groups like *Bacteroides fragilis* (Gong et al., 2021). Yang et al. demonstrated elevated abundance of *Firmicutes*, *Proteobacteria* and *Actinobacillus* in GD patients compared to controls, with a notably higher *Firmicutes/Bacteroidetes* ratio (Yang et al., 2019). Biscarini et al. identified significant differences in the composition of the gut microbiota in GD/GO mouse models developed at various centers, suggesting a potential association with TSHR-induced disease heterogeneity (Masetti et al., 2018). To further confirm a causal relationship, Moshkelgosha et al. subsequently modified the gut microbiota based on this finding to determine its role in thyroid autoimmunity and ultimately demonstrated the critical role of gut microbiota in the development of TSHR-induced diseases (Moshkelgosha et al., 2021). This discovery was validated in a multicenter study conducted by Biscarini et al. (2023). Moreover, a prospective study similarly indicated distinctive features in the gut microbiota of GD patients, which might be linked to imbalances in the immune system and gut microbiota (Deng et al., 2023). These studies provide compelling evidence for a causal relationship between gut microbiota and GD.

Controversies persist regarding the mechanisms by which gut microbiota influence GD. The impact of gut microbiota on the thyroid could potentially occur through several main avenues: Intestinal dysbiosis leading to impaired gut barrier function and increased intestinal permeability, facilitating antigen entry into circulation and immune system activation (Cayres et al., 2021). Antibodies in circulation may react with bacterial antigens, enhancing the activation of inflammatory foci within the thyroid (Tomasello et al., 2015). In addition, Short Chain Fatty Acids (SCFAs) are the principal products of dietary fiber fermentation by gut microbiota, including acetate, propionate, and butyrate. Beyond regulating intestinal functions, SCFAs also exert regulatory effects on host metabolism and immune responses (den Besten et al., 2013). After absorption by intestinal epithelial cells, SCFAs partly regulate intestinal function within the cells, while the remainder exerts systemic effects through the bloodstream, modulating glucose and fat metabolism, as well as immune system regulation (Kim, 2021), including inhibition of histone deacetylase (HDAC) (Licciardi et al., 2011), G protein-coupled receptor (GPR) signaling, and acetyl-CoA production and metabolic integration. Relevant research suggests that SCFAs can enhance immune responses, and elevated SCFA levels have been linked to improved colitis (Scheppach et al., 1992; Ananthakrishnan et al., 2013), autoimmune neuroinflammation (Haghikia et al., 2016), renal inflammation (Andrade-Oliveira et al., 2015), and atherosclerosis. For instance, butyrate (a type of SCFA) can reduce TNF-α and IL-6 levels, and inhibit NLRP10 inflammasome activation through GPR3A (Pan et al., 2019).

Hence, this study employs Two-sample Mendelian randomization to investigate the causal relationship between gut microbiota and GD, aiming to provide evidence for the pathogenesis of GD and offer insights into its future treatment. Genetic variants (SNPs) associated with gut microbiota were selected as instrumental variables (IVs). Gut microbiota data were sourced from the international consortium MiBioGen, while Graves’ disease data were obtained from FINNGEN. Multiple analysis methods, including inverse variance-weighted (IVW), MR-Egger regression, weighted median, weighted mode, and MR-RAPS, were utilized. Sensitivity analyses were conducted employing MR-Egger intercept test, Cochran’s Q test, and leave-one-out analysis as quality control measures.

## 2 Methods

### 2.1 Data source

In this study, the gut microbiota’s genome-wide association study (GWAS) data were obtained from the international consortium MiBioGen. This database harmonized 16S rRNA gene sequencing profiles and genotyping data of 18,340 participants across 24 cohorts from the United States, Canada, Israel, South Korea, Germany, Denmark, the Netherlands, Belgium, Sweden, Finland, and the United Kingdom. It conducted a large-scale, multi-ethnic, whole-genome meta-analysis of autosomal human genetic variation and its association with gut microbiota composition (Kurilshikov et al., 2021). The GWAS dataset for GD was obtained from FINNGEN, a Finnish database comprising samples from 281,683 individuals, with 1,828 individuals having Graves’ disease and 279,855 individuals in the control group (Kurki et al., 2023). Given the disparities in racial and population stratification, populations of both exposure and outcome datasets were of European ancestry, encompassing both males and females, thereby mitigating biases due to population and gender stratification (Emdin et al., 2017). Detailed information on the dataset is provided in **Table 1**.

**Table 1:** Detailed information on the dataset.

Contribution	Trait	Year	Case	Sample size	SNP	Consortium	Author	Gender	Population
Exposures	Abundance of 150 Gut microbiota	2021	14306	14306	5729268	MiBioGen	Kurilshikov A	Males and Females	European
Outcome	Graves’ disease	2023	1828	281683	20167370	FinnGen	FinnGen	Males and Females	European

### 2.2 Study design

**Figure 1:**
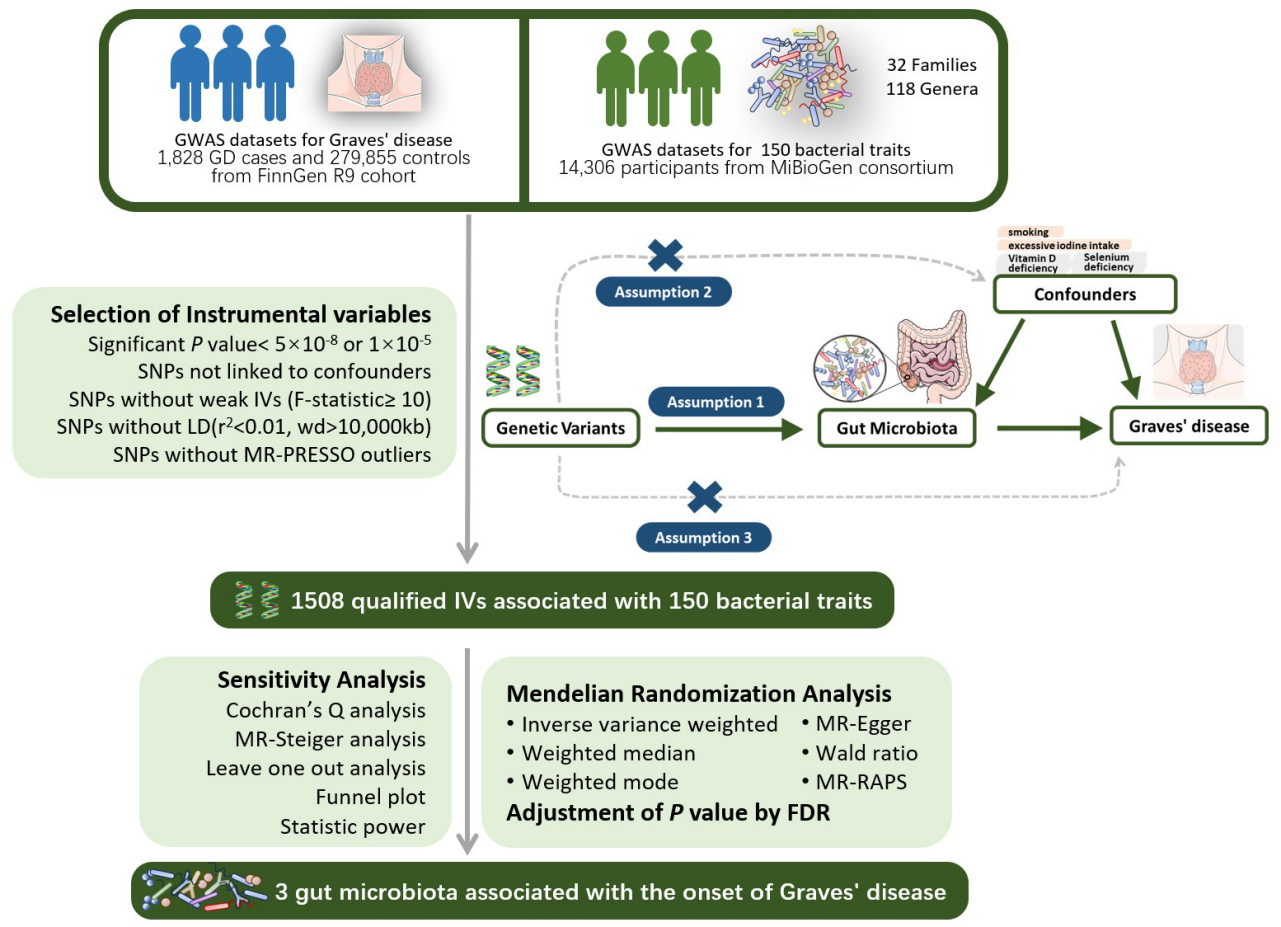
The design of the two-sample MR study for the association of Gut Microbiota on Graves’ disease. GWAS, Genome-wide association study; LD, linkage disequi-librium; MR-PRESSO, Mendelian Randomization Pleiotropy RESidual Sum and Outlier, a method test the pleiotropic biases in the SNPs and correct the pleiotropic effects; MR, Mendelian randomization; SNP, single nucleotide polymorphism, as instrumental variables for the exposures and outcomes; FDR, false discovery rate.

Two-sample Mendelian randomization (TSMR) is a genetic-level causal study that utilizes GWAS summary data from two distinct datasets. In one-sample Mendelian randomization studies where both the exposure and outcome datasets originate from the same database, the likelihood of false positives increases due to the “winner’s curse” and the increased probability of weak instrumental variables (Jiang et al., 2023). Conversely, in two-sample Mendelian randomization analyses where the genetic associations with exposure and outcome are derived from independent samples, the opportunistic correlations vary between datasets. This variation independently influences the associations with exposure and outcome, with the bias due to weaker instruments tending towards zero. In this study, we employed a two-sample Mendelian randomization (MR) approach to explore the causal impact of gut microbiota on GD, as illustrated in **Figure 1**. Genetic variants (SNPs) linked to gut microbiota were chosen as instrumental variables (IVs). Significant instrumental variables will be extracted from the gut microbiome dataset, and their associations with GD will be identified in the GD dataset. Subsequently, a range of MR analytical methods will be applied for causal investigation. Additionally, various sensitivity analyses will be conducted to assess the robustness of the results.

MR analysis must satisfy the following three assumptions: 1) The relevance assumption: A strong association between instrumental variables and the exposure factor (*P*<5×10^-8^ or *P*<1×10^-5^) is presumed; 2) The independence assumption: Instrumental variables must be independent of confounding factors influencing exposure and outcome; 3) The exclusion restriction assumption: Instrumental variables must affect the outcome variable solely through the exposure factor and not directly.

### 2.3 Selection of instrumental variables

Based on the aforementioned study design and assumptions, we initially extracted SNPs significantly associated with the exposure at a genome-wide significance level (alpha=5×10^-8^). For batches where extraction was not feasible, we reduced the threshold to alpha=1×10^-5^ to ensure an adequate number of SNPs. We set r^2^ < 0.01 and kb=10000 as the criteria for removing linkage disequilibrium (LD), ensuring the independence of SNPs and mitigating the risk of multicollinearity. The strength indicator F value (F=β^2^/se^2^) was calculated for each SNP, with F<10 considered weak instrumental variables that fail to satisfy assumption one and were thus excluded (Burgess et al., 2017). We extracted outcome data from the outcome dataset based on significant SNP and performed data matching. Ambiguous and palindromic SNPs were excluded during the matching process. The PhenoScanner database (Staley et al., 2016) was employed to search for each SNP, excluding those associated with confounding factors such as smoking (Wiersinga, 2013; Antonelli et al., 2020) and excessive iodine intake (Burgi, 2010). The MR-PRESSO test was utilized to detect outliers with horizontal pleiotropy, leading to the exclusion of outlier SNPs. MR-Steiger analysis was applied to assess the causal direction of all SNPs and SNPs with erroneous directions were removed (Li et al., 2022). Finally, we employed Bonferroni correction (*P* < 0.05/n, n referring to the number of remaining SNPs) to eliminate SNPs directly associated with the outcome. Following the rigorous screening outlined above, the remaining SNPs were deemed qualified instrumental variables.

### 2.4 MR analysis

We employed a variety of MR methods to calculate the causal relationship between gut microbiota and Graves’ disease, including IVW method, MR-Egger regression, weighted median, weighted mode, and MR-PAPS method.

Firstly, the effect size of each SNP on causal estimation was assessed using the Wald ratio, which represents the ratio of the individual SNP’s impact on the outcome to its impact on the risk factor under the assumption of linearity (Thomas and Conti, 2004). The IVW method is a weighted linear regression without an intercept term, where the slope parameter represents the causal estimate, and the weight is the inverse of the genetic association variance divided by the squared standard error of the outcome (Burgess et al., 2013). Following Mendelian randomization guidelines, we opted for the multiplicative random-effects model as the primary analysis in the absence of horizontal pleiotropy and heterogeneity (Burgess et al., 2019). Weighted median and weighted mode methods are common consensus methods. They compute the causal effect based on the majority valid assumption and the plurality valid assumption, respectively (Bowden et al., 2016a; Burgess et al., 2019). When there is heterogeneity between SNPs, both the weighted median and IVW methods need to support the significant conclusion. The MR-Egger method is similar to the IVW method but includes an intercept term in the regression model (Burgess et al., 2017). The MR-Egger method provides consistent causal effect estimates under the assumption of internal stability and unbiasedness. The intercept term in MR-Egger also offers a test for horizontal pleiotropy among IVs. In case of horizontal pleiotropy among SNPs, we employed the MR-Egger method as the primary analysis.MR-RAPS method estimates causal effects using a probabilistic profile likelihood function assuming that pleiotropy follows a normal distribution centered at zero with a location variance, enhancing robustness against outliers (Zhao et al., 2020). Finally, a False Discovery Rate (FDR) correction was applied to MR results. Causal relationships were inferred based on significant *P*-values < 0.05.

### 2.5 Sensitivity analysis

Cochran’s Q test was employed to assess heterogeneity among IVs, considering SNPs with Q test P-value < 0.05 as heterogeneous. Furthermore, we used the MR-Steiger model to validate the overall direction of estimates for result robustness (Hemani et al., 2017). To ascertain the influence of strong effect SNPs, we conducted a leave-one-out sensitivity test. The Instrument Strength Independent of Direct Effect (InSIDE) assumption (Burgess and Thompson, 2017) and the No Measurement Error (NoME) hypothesis (Bowden et al., 2016b) must be satisfied for MR-Egger regression. We constructed a funnel plot and calculated the I^2^ statistic to validate these assumptions. A correction for causal estimates is required when I^2^ < 90% and the primary analytical method is MR-Egger (Bowden et al., 2016b). Sample size analysis is particularly crucial for determining whether negative results truly indicate the absence of a causal relationship. We calculated the statistical power following Burgess’s method (Burgess, 2014).

### 2.6 Visualization

Scatter plots and regression curve plots were generated for each set of MR analyses in this study, along with forest plots illustrating SNP effects. These visualizations will be presented in the results. Additionally, a circular heatmap and forest plot were constructed for the overall results. A Manhattan plot was created for significant gut microbiome GWAS data to elucidate relevant SNP information. Several figures were partly generated using Servier Medical Art (smart.servier.com), provided by Servier, licensed under a Creative Commons Attribution 3.0 unported license.

### 2.7 Statistical analysis software

All statistical analyses and visualizations in this study were conducted using R software (version 4.1.2) with packages including “TwoSampleMR,” “MR-PRESSO,” “mr.raps,” “forestploter,” and several foundational R packages.

## 3 Results

### 3.1 Selection of instrumental variables

Initially, a total of 1918 SNPs related to gut microbiome were screened, and no weak instruments with F<10 were identified. 97 SNPs were excluded due to missing data in the outcome dataset, 294 SNPs were removed as ambiguous or palindromic SNPs during dataset integration, and 6 SNPs were deleted based on PhenoScanner search indicating associations with confounders such as smoking and vitamin D deficiency. MR-PRESSO test identified 11 SNPs with horizontal pleiotropy. After Bonferroni correction, 2 SNPs directly related to the outcome were removed.

Ultimately, 1508 eligible SNPs were included in the study.

### 3.2 Altered abundance of gut microbiota affects GD incidence

**Figure 2:**
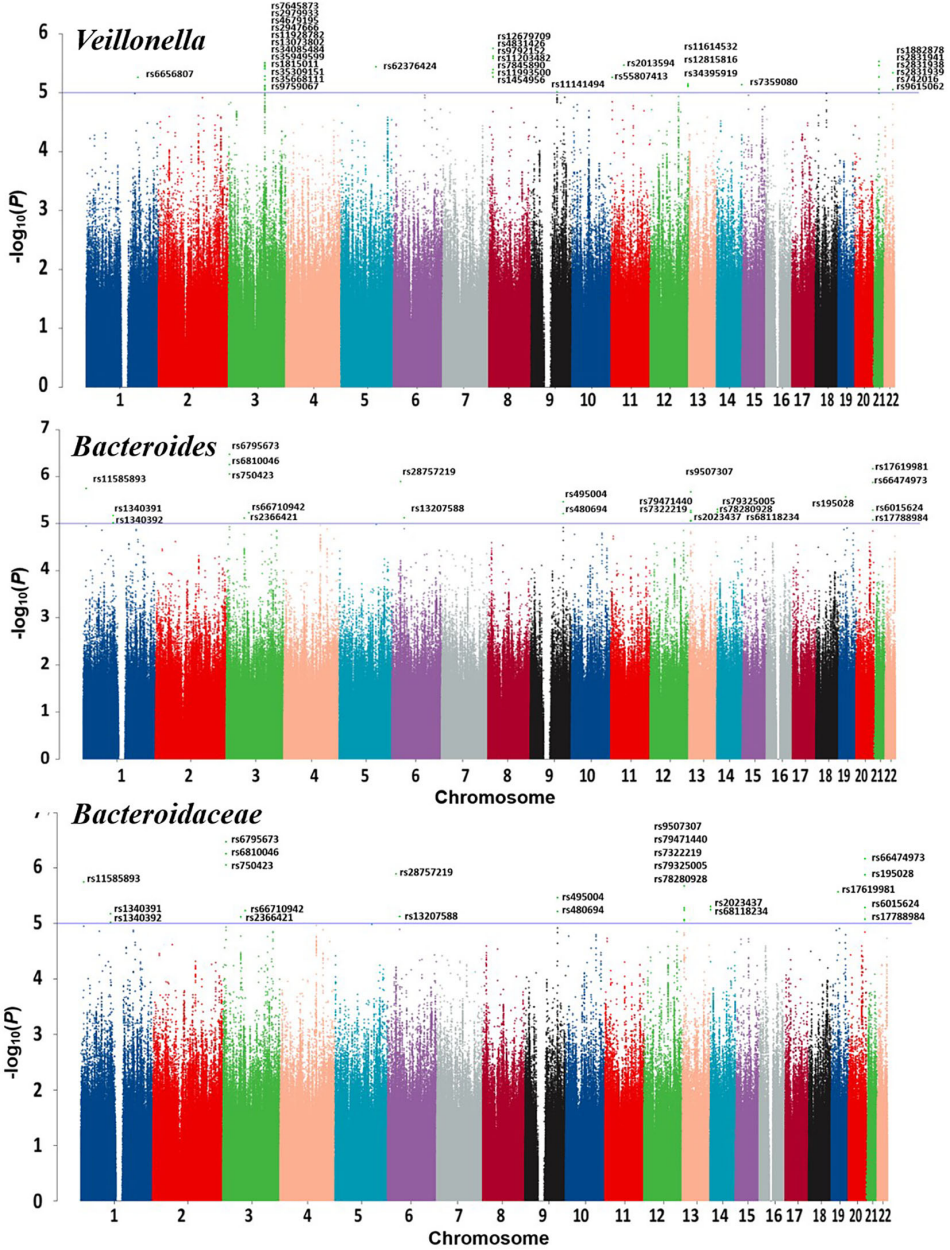
Manhattan plots of GWAS datasets of Veillonella and Bacteroides.

**Figure 3:**
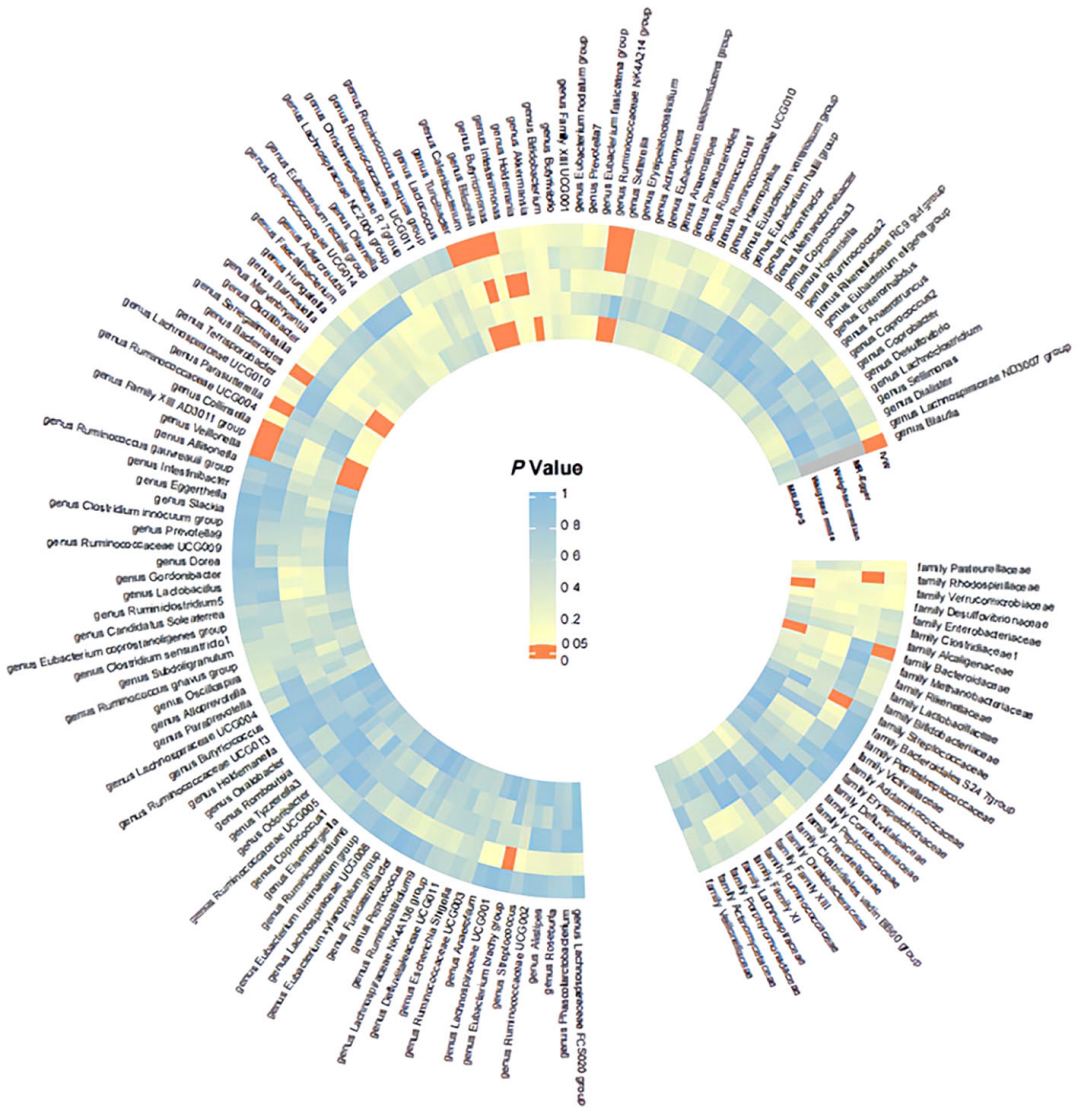
Significance Heatmap of MR Analysis. IVW, inverse variance weighted; MR, Mendelian randomization; RAPS, Robust Adjusted Profile Score.

**Figure 4:**
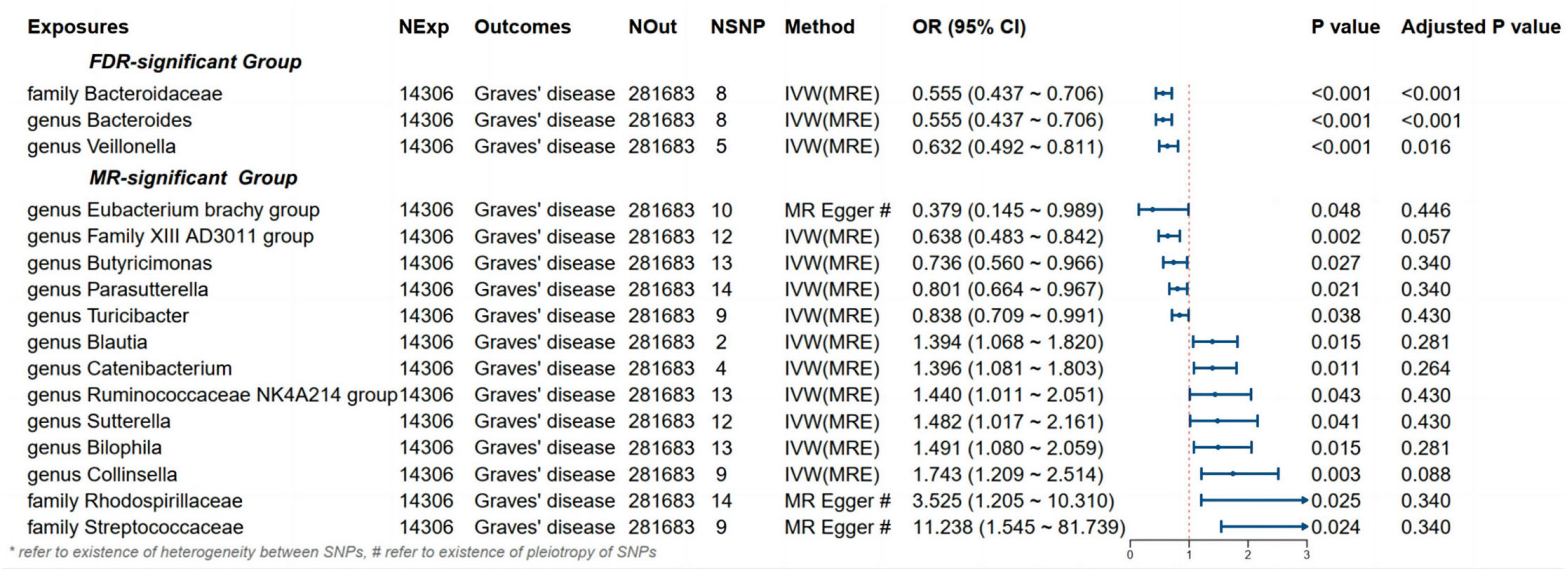
Results and forest plot of the significant MR analysis. IVW(MRE), inverse variance weighted (multiplicative random effects model); CI, confidence interval; NExp, sample size of exposure dataset; NOut, sample size of outcome dataset; NSNP, number of SNP included in MR analysis; * refer to existence of heterogeneity of SNPs, # refer to existence of pleiotropy between SNPs.

Detailed information regarding the MR study is summarized in the **Supplementary Material**. In this study, taxonomic families or genera of 150 gut microbiota were included for analysis. The number of SNPs per type of gut microbiota ranged from 1 to 40. **Supplementary Information** provides detailed IVs information for the 150 gut microbiota. Manhattan plots (**Figure 2**) were generated for significant genera, providing clarity on relevant SNP information. Through MR analysis, we identified 3 gut microbiota family/genera with a protective effect on GD incidence, while 13 genera were found to potentially influence GD incidence. Results of the MR analysis are depicted in the circular heatmap (**Figure 3**) and forest plot (**Figure 4**).

### 3.3 Positive results

MR analysis revealed a significant causal effect of the family *Bacteroidaceae*, genus *Bacteroides*, and genus *Veillonella* on GD incidence. The family *Bacteroidaceae* (OR (95%CI): 0.89 (0.89 ~ 0.90), adjusted *P* value: <0.001), the genus *Bacteroides* (OR (95%CI): 0.555 (0.437 ~ 0.706), adjusted *P* value: <0.001), and the genus *Veillonella* (OR (95%CI): 0.632 (0.492 ~ 0.811), adjusted *P* value: 0.016) were associated with reduced risk of GD incidence (**Figure 4**). Cochran’s Q-test and MR-Egger intercept showed no evidence of potential heterogeneity or pleiotropy bias in our findings (all *P* values > 0.05, **Table 2**).

**Table 2:** Sensitivity Analysis of the significant MR analysis.

Batch	Exposures	Outcomes	Q from IVW	Pval_Q from IVW	Q from MR-Egger	Pval_Q from MR-Egger	I2 for MR-Egger	Pval of Pleotropy	Dirction from MR-Steiger
4	family Bacteroidaceae	Graves’ disease	2.071	0.956	1.596	0.953	0.963	0.516	TRUE
42	genus Bacteroides	Graves’ disease	2.071	0.956	1.596	0.953	0.963	0.516	TRUE
150	genus Veillonella	Graves’ disease	1.374	0.849	0.826	0.843	0.760	0.513	TRUE
70	genus Eubacterium brachy group	Graves’ disease	13.732	0.132	7.714	0.462	0.962	0.040	TRUE
82	genus Family XIII AD3011 group	Graves’ disease	7.240	0.779	6.417	0.779	0.936	0.385	TRUE
48	genus Butyricimonas	Graves’ disease	9.905	0.624	7.575	0.751	0.958	0.155	TRUE
115	genus Parasutterella	Graves’ disease	7.010	0.902	6.940	0.862	0.842	0.796	TRUE
148	genus Turicibacter	Graves’ disease	2.401	0.966	0.837	0.997	0.905	0.251	TRUE
46	genus Blautia	Graves’ disease	–	–	–	–	0.869	–	TRUE
51	genus Catenibacterium	Graves’ disease	2.351	0.503	0.686	0.710	0.000	0.326	TRUE
126	genus Ruminococcaceae NK4A214 group	Graves’ disease	13.807	0.313	10.980	0.445	0.942	0.121	TRUE
146	genus Sutterella	Graves’ disease	14.648	0.199	11.134	0.347	0.958	0.106	TRUE
45	genus Bilophila	Graves’ disease	11.778	0.464	11.035	0.440	0.964	0.408	TRUE
55	genus Collinsella	Graves’ disease	5.961	0.652	5.904	0.551	0.969	0.819	TRUE
26	family Rhodospirillaceae	Graves’ disease	10.540	0.649	4.934	0.960	0.904	0.036	TRUE
29	family Streptococcaceae	Graves’ disease	14.633	0.067	6.333	0.501	0.974	0.024	TRUE

MR analysis with less than 3 SNPs are not available for Cochran’s Q test (marked “-”). IVW refer to inverse variance weighted method.

### 3.4 Other results

Despite not passing FDR multiple testing correction, 13 bacterial types, including the *Eubacterium brachy group*, showed significant results in the initial MR analysis, indicating potential causal effects on GD incidence (**Figure 4**). Among them, *Eubacterium brachy* group (OR [95%CI]: 0.379 (0.145 ~ 0.989), *P* value: 0.048), *Family XIII AD3011* group (OR [95%CI]: 0.638 (0.483 ~ 0.842), *P* value: 0.002), *Butyricimonas* (OR [95%CI]: 0.736 (0.560 ~ 0.966), *P* value: 0.027), *Parasutterella* (OR [95%CI]: 0.801 (0.664 ~ 0.967), *P* value: 0.021) and *Turicibacter* (OR [95%CI]: 0.838 (0.709 ~ 0.991), *P* value: 0.038) showed a potential reduced risk of GD incidence. *Bilophila* (OR [95%CI]: 1.394 (1.068 ~ 1.820), *P* value: 0.015), *Catenibacterium* (OR [95%CI]: 1.396 (1.081 ~ 1.803), *P* value: 0.011), *Ruminococcaceae NK4A214* group (OR [95%CI]: 1.440 (1.011 ~ 2.051), *P* value: 0.043), *Sutterella* (OR [95%CI]: 1.482 (1.017 ~ 2.161), *P* value: 0.041), *Blautia* (OR [95%CI]: 1.491 (1.080 ~ 2.059), *P* value: 0.015), *Collinsella* (OR [95%CI]: 1.743 (1.209 ~ 2.514), *P* value: 0.003), *Rhodospirillaceae* (OR [95%CI]: 3.525 (1.205 ~ 10.310), *P* value: 0.025) and *Streptococcaceae* (OR [95%CI]: 11.238 (1.545 ~ 81.739), *P* value: 0.024) showed a potential increased risk of GD incidence. According to Cochran’s Q test, there is no evidence of heterogeneity (All *P* values > 0.05, **Table 2**).

However, based on the MR-Egger intercept, *Eubacterium brachy* group (MR-Egger intercept *P* value: 0.040), *Rhodospirillaceae* (MR-Egger intercept *P* value: 0.036) and *Streptococcaceae* (MR-Egger intercept *P* value: 0.024) exhibit pleiotropy, and MR-Egger will be used as the primary analytical method.

## 4 Discussion

Although previous studies have consistently demonstrated the impact of gut microbiota on Graves’ disease, there is still controversy regarding changes in gut microbiota abundance in GD patients. Most studies indicate a significant decrease in gut microbiota abundance in GD patients compared to healthy individuals, this aligns with our findings. For instance, Deng et al. analyzed the gut microbiota of Graves’ disease patients and controls and found that GD patients exhibited lower gut bacterial diversity (Deng et al., 2023). However, some studies have reported no significant changes in gut microbiota abundance and diversity in GD patients (Yang et al., 2019; Chang et al., 2021), which requires further investigation for confirmation. The *Firmicutes* and *Bacteroidetes* ratio (F/B) is believed to play a significant role in maintaining normal gut homeostasis. Variations in the F/B ratio are indicative of gut ecological imbalance and, to some extent, reflect human health status. An increased F/B ratio may suggest dysbiosis in obese patients (Magne et al., 2020), while a decreased F/B ratio is observed in certain autoimmune diseases such as systemic lupus erythematosus and inflammatory bowel disease (De Luca and Shoenfeld, 2019; Stojanov et al., 2020). A meta-analysis indicated alterations in the diversity and abundance of certain gut microbiota in patients with autoimmune thyroid disease (AITD) compared to control groups, including a decrease in Firmicutes abundance and an increase in Bacteroidetes abundance (i.e., decreased F/B ratio) (Gong et al., 2021); Studies by El-Zawawy et al. (2021), and Su et al. (2020) also suggest a decreased abundance of Firmicutes and an increased abundance of *Bacteroidetes* in GD patients, associated with a lower F/B ratio. However, Yang et al. (2019) found no significant differences in gut microbiota abundance between GD patients and controls.

Mendelian randomization studies can establish definite causal relationships in the absence of confounding factors. In our study, we identified a decreased risk of GD with the increased abundance of *Bacteroides* in *Bacteroidetes* and *Veillonella* in *Firmicutes*.

Previous research has yielded varying results regarding specific changes in gut microbiota among GD patients. Hafiz et al (Ishaq et al., 2018). recruited 27 GD patients and 11 healthy controls, collected and analyzed fecal samples, and found significantly increased relative abundances of *Prevotellaceae* and *Pasteurellaceae* in GD patients compared to controls, while *Enterobacteriaceae*, *Veillonellaceae*, and *Rikenellaceae* were significantly decreased in GD patients. At the genus level, GD patients exhibited significant increases in *Prevotella 9* and *Haemophilus*, while *Alistipes* and *Faecalibacterium* were significantly decreased. Yang et al. (2019) found higher abundances of *Oribacterium*, *Lactobacillus*, *Aggregatibacter*, and *Mogibacterium* genera in GD patients compared to healthy controls. Additionally, GD patients exhibited higher counts of *Bacteroides* and *Lactobacillus*. Chen et al. found significantly higher relative abundances of *Lactobacillus*, *Veillonella*, and *Streptococcus* in GD patients (Jiang et al., 2021). Due to limited sample sizes in the aforementioned studies, related MR research is gradually emerging. A two-sample MR analysis investigating the causal relationship between gut microbiota and GD identified *Deltaproteobacteria* and *Mollicutes* classes, as well as *Ruminococcus torques* group, *Oxalobacter*, and *Ruminococcaceae ucg011* as risk factors for GD (Cao et al., 2023). *Streptococcaceae* and *Lachnospiraceae* were protective factors (Cao et al., 2023). It is worth noting that the gut microbiota GWAS dataset used in this study was based on European populations, while the GD GWAS database was based on Asian populations. In contrast to the above findings, our study conducted a two-sample MR analysis using GWAS data from European populations for both exposure and outcome, effectively mitigating the impact of population stratification on conclusions, and confirming that *Bacteroidaceae*, *Bacteroides*, and *Veillonella* were protective factors against GD development.

In current research on factors influencing Graves’ disease through the gut microbiota, increasing attention is being paid to the immunomodulatory effects of short-chain fatty acids (SCFAs). SCFAs can serve as an energy source for epithelial cells, maintain intestinal barrier integrity, and reduce gut permeability and circulating lipopolysaccharide levels (Koh et al., 2016). Su et al. (2020) and a prospective study (Deng et al., 2023) both found reduced levels of propionate and butyrate in GD patients, thereby corroborating the notion that decreased SCFAs production in GD patients promotes disease occurrence and progression.

SCFAs are typically produced by certain subtypes of *Firmicutes* and *Bacteroidetes* phyla (Kim, 2021). Members of the *Bacteroidetes* (Gram-negative), *Firmicutes* (Gram-positive), and *Actinobacteria* (Gram-positive) phyla have the highest abundance in the gut. *Bacteroidetes* primarily produce acetate and propionate, while *Firmicutes* generate butyrate as a major end-product of metabolism (Fenneman et al., 2020). The genera mentioned in this study, *Bacteroides* and *Veillonella*, belong to the *Bacteroidetes* and *Firmicutes* phyla, respectively, and both are capable of producing SCFAs. Therefore, the significant decline in the abundance of these three bacteria may lead to reduced SCFAs production, subsequently resulting in impaired intestinal barrier function and dysbiosis (Lapidot et al., 2021).

The mechanism by which SCFAs impact the immune system primarily involves the modulation of innate and adaptive lymphocytes (Kim, 2021). On one hand, SCFAs increase the activity of type 3 innate lymphoid cells (ILC3s) while suppressing the activity of type 2 innate lymphoid cells (ILC2s). Ffar3 signaling in ILC2s and ILC3s triggers PI3K, AKT, and mTOR activity, promoting cell proliferation and activation. On the other hand, under normal conditions, there’s a balance between Th17 and T regulatory cells (Tregs). Excessive Th17 increase and reduced Tregs disrupt the Th17/Tregs balance, leading to GD development. SCFAs are involved in the pathogenesis and progression of GD through the immunomodulation of Th17/Tregs and cytokines (Kohling et al., 2017). Dietary fiber metabolite SCFAs, such as butyrate can directly promote the proliferation of CD4 regulatory T cells (Tregs) under normal circumstances (Arpaia et al., 2013). Tregs regulate intestinal homeostasis and control inflammation by expressing the transcription factor Foxp3 (Smith et al., 2013). For instance, SCFAs from fecal bacteria with chloroform-resistant phenotypes were identified as major bacterial metabolites responsible for inducing Tregs and suppressing inflammation in a colitis mouse model (Atarashi et al., 2013). Furthermore, SCFAs can indirectly regulate Treg proliferation and function through the modulation of antigen-presenting cells (APCs), such as dendritic cells (DCs) and macrophages (Mendoza-Leon et al., 2023). Butyrate can signal through the SCFA receptor GPR109a in macrophages, promoting the expansion of Tregs by DCs (Singh et al., 2014). In addition, SCFAs, especially butyrate, can have a significant effect on B cell function. For instance, intestinal macrophages treated with butyrate can induce the generation of regulatory B cells producing IL-10 (Foh et al., 2022). Overall, SCFAs support the effector functions of lymphocytes to defend against microbial pathogens and cancer, playing a significant role in GD development.

This study indicates that *Bacteroidaceae*, *Bacteroides*, and *Veillonella* are associated with a reduced risk of GD development, serving as protective factors against GD. The genus Bacteroides consists of more than 20 anaerobic, non-spore-forming, Gram-negative rods, and belongs to the *Bacteroideaceae* family. The impact of *Bacteroides* on autoimmune diseases is gradually being investigated. Mazmanian et al. (2005) demonstrated that *Bacteroides fragilis*-derived polysaccharides (PSA) can activate CD4+ T cells, with PSA-stimulated CD4+ T cells producing interleukin-10 (IL-10), which acts to prevent abscess formation and other inflammatory responses. Furthermore, PSA Production by B. fragilis corrects TH1/TH2 imbalance, directs Lymphoid Organogenesis, and promotes development and maturation of the immune system. In previous studies, an increase in the abundance of *Bacteroides* in GD patients was reported, whereas this study indicates the opposite trend. This discrepancy could be attributed to regional or population differences, an inverse causal relationship and insufficient sample size, warranting further research for validation. The family *Bacteroidaceae* comprises *Acetofilamentum*, *Acetothermus*, *Bacteroides*, *Capsularis*, and *Phocaeicola*. In this study, within the family *Bacteroidaceae*, significant findings were observed exclusively within the genus *Bacteroides*, leading us to posit that the impact of *Bacteroidaceae* on GD appears to be attributed to the genus *Bacteroides*. Nevertheless, given the limited existing literature on the relationship between *Bacteroidaceae* and GD, further in-depth research is warranted. *Veillonella*, belonging to the phylum *Firmicutes*, are anaerobic Gram-negative cocci that typically occur in pairs or short chains. They lack flagella, spores, or capsules (Delwiche et al., 1985) and are commonly associated with inflammatory conditions such as periodontitis, bacteremia, and pneumonia (Marriott et al., 2007; Shah et al., 2008). Members of the genus *Veillonella* utilize short-chain organic acids, particularly lactate, as an energy source rather than carbohydrates or amino acids, subsequently producing acetate and propionate (Mashima et al., 2021). It is commonly found in the oral cavity, gastrointestinal tract, and vagina. There is still debate regarding this bacterium. Deng et al. suggested an increased abundance of *Veillonella* in GD patients (Deng et al., 2023), while this study aligns with previous reports indicating lower levels of *Veillonella* in GD patients(**Table 3**).

**Table 3:** Summary of the Mechanisms of Action of Gut Microbiota on Graves’ Disease.

Gut Microbiota	Mechanism	PMID of Reference
**Bacteroidaceae and Bacteroides**	Bacteroidaceae/Bacteroides, by producing SCFAs (acetate and propionate), modulate innate and adaptive lymphocytes, thereby contributing to the pathogenesis of GD.	32412045, 33850311
**Veillonella**	Veillonella is reported to be an opportunistic pathogen in various inflammatory diseases. Although similar evidence of GD has been found in observational studies, its mechanism of action remains unclear.	27326455, 18547858, 17108070, 37485373

Furthermore, it is noteworthy that despite not maintaining statistical significance after adjusting *P*-values, there exists a protective trend towards decreased GD occurrence for *Eubacterium brachy* group, *Family XIII AD3011* group, *Butyricimonas*, *Parasutterella*, and *Turicibacter*. Conversely, a hazardous trend towards increased GD occurrence is observed for *Blautia*, *Catenibacterium*, *Ruminococcaceae NK4A214* group, *Sutterella*, *Bilophila*, *Collinsella*, *Rhodospirillaceae*, and *Streptococcaceae*. However, further validation is warranted.

Presently, there are three main approaches for GD treatment: radioactive iodine (RAI) therapy, anti-thyroid drugs (ATDs), and surgical intervention. However, each method is accompanied by its respective adverse effects (Smith and Hegedus, 2016). Therefore, elucidating the pathogenesis of GD may facilitate the identification of novel therapeutic targets. Probiotics, as living organisms, modulate the gut microbiome in various ways to enhance gut health (Guo et al., 2021). Ingesting probiotics alters the composition of the gut microbiome, aiding in the prevention of the progression of autoimmune diseases.

Zhang et al. found that *Lactobacillus* alleviates inflammatory episodes in lupus-prone female mice (Zhang et al., 2014). Clusters IV and XIVa of the genus *Clostridium* improve IBD in a colitis model through inducing Treg cells and increasing Foxp3 transcription factor expression (Atarashi et al., 2011). In animal models, adjusting the gut microbiome through probiotic supplementation appears to ameliorate SLE symptoms and associated cardiovascular and renal complications (Guo et al., 2021). Despite the numerous favorable outcomes seen when various probiotic strains were used to counteract various autoimmune diseases in animal models, human clinical data remain limited. This limitation might, at least in part, result from poorly designed study protocols that fail to account for the interplay between diseases and dysbiosis (Kim et al., 2016). Therefore, clinical trials employing probiotics should meticulously consider alterations in microbial composition and their impacts on autoimmune diseases. This study aims to offer novel insights into future GD treatment.

Our study possesses several strengths. Firstly, we utilized the MR analysis method to assess the associations between various gut microbiota abundances and GD risk, mitigating potential confounding factors. The ample sample size affords us sufficient power to estimate the causal effect of gut microbiota on GD. Secondly, we conducted an investigation into 150 distinct families and genera of gut microbiota. Given the substantial variations among different phyla, classes, and orders of microbiota, we exclusively utilized data pertaining to families and genera for more precise conclusions.

However, this study is not without limitations. Firstly, some batches of SNP data still exhibited pleiotropy after MR-PRESSO correction, potentially undermining the robustness of MR conclusions. Moreover, our study population was predominantly of European descent, thereby limiting the generalizability of findings to broader populations. Further research is warranted to explore the impact of gut microbiota abundance on GD occurrence across different ethnic groups. As we employed summary data rather than individual-level data, stratified analysis by variables such as gender was unattainable. The statistical power of correlation-based conclusions for some batches in this study was relatively low, potentially elevating the likelihood of type II errors. The assumption of linearity in the causal relationship, inherent in the method of ratio estimation, prevents this study from excluding potential non-linear associations between gut microbiota and GD susceptibility. Lastly, although bacteria are the primary constituents of the intestinal microbiome, viruses, fungi, and archaea also inhabit the gut. Their interactions with the gut microbiota and GD remain largely unknown, necessitating further research. Nevertheless, it is crucial to note that as long as the SNPs utilized in this study satisfy the three assumptions of instrumental variables, the resultant MR conclusions remain valid.

## 5 Conclusion

There is a causal relationship between gut microbiota abundance and GD. *Bacteroidaceae*, *Bacteroides*, and *Veillonella* serve as protective factors against GD occurrence. Thirteen bacterial strains, including *Eubacterium brachy* group, potentially exert causal influences on GD occurrence. Probiotics may offer a novel avenue for adjunctive therapy in future GD treatment.

## Data availability statement

Publicly available datasets were analyzed in this study. This data can be found here: https://mibiogen.gcc.rug.nl/. https://www.finngen.fi/.

## Author contributions

SL: Writing – original draft. FL: Writing – original draft. YC: Investigation, Writing – review & editing. LR: Writing – review & editing. LS: Supervision, Writing – review & editing. XG: Supervision, Writing – review & editing. GW: Funding acquisition, Supervision, Writing – review & editing.
