# Supplementary material for: Bacteroidaceae, Bacteroides, and Veillonella: emerging protectors against Graves’ disease
Source: Front Cell Infect Microbiol. 2024 Feb 9;14:1288222. doi: 10.3389/fcimb.2024.1288222 (PMC10884117; doi:10.3389/fcimb.2024.1288222)

Batch 1 : Gut microbiota abundance (family Acidaminococcaceae id.2166) on Graves' disease

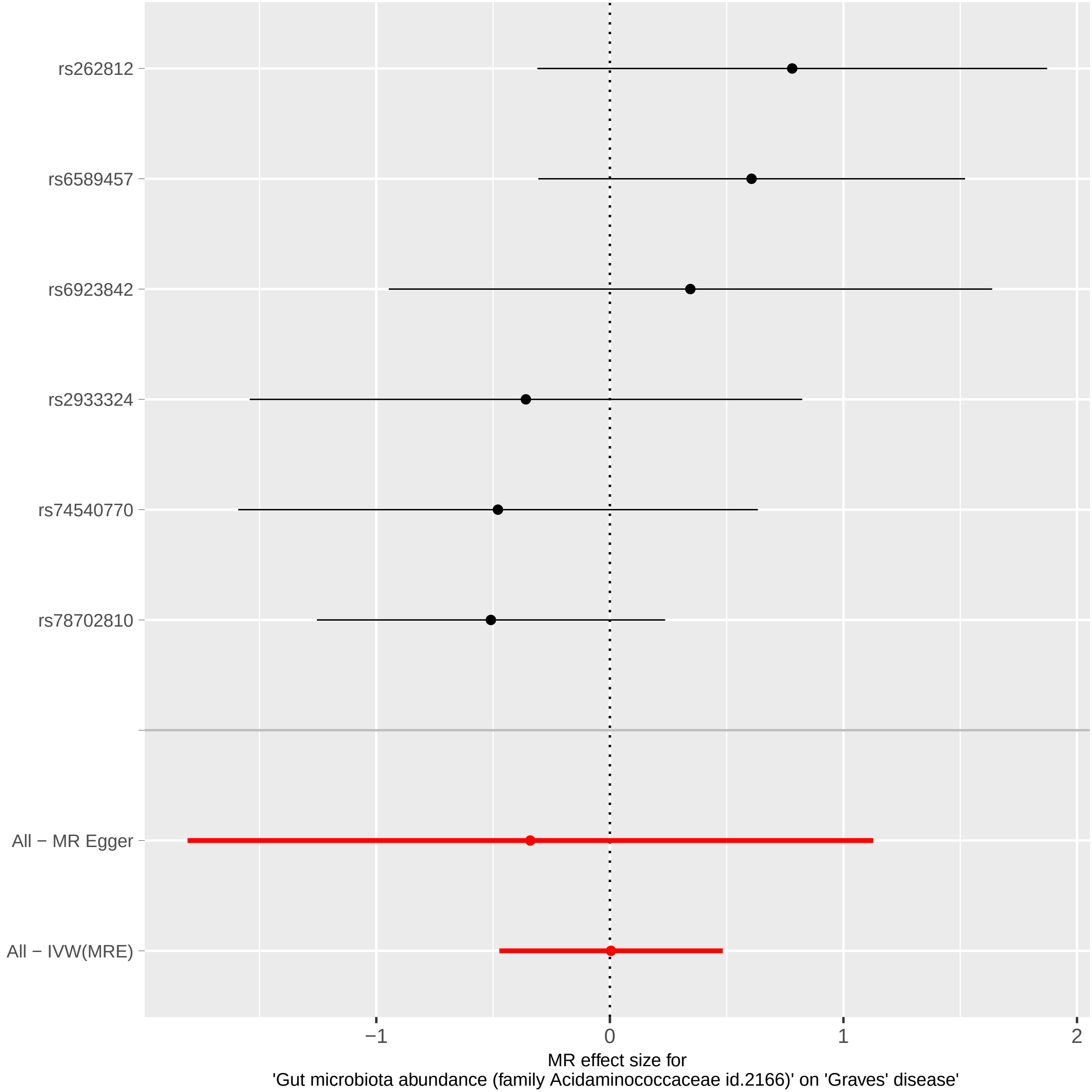

Batch 2 : Gut microbiota abundance (family Actinomycetaceae id.421) on Graves' disease

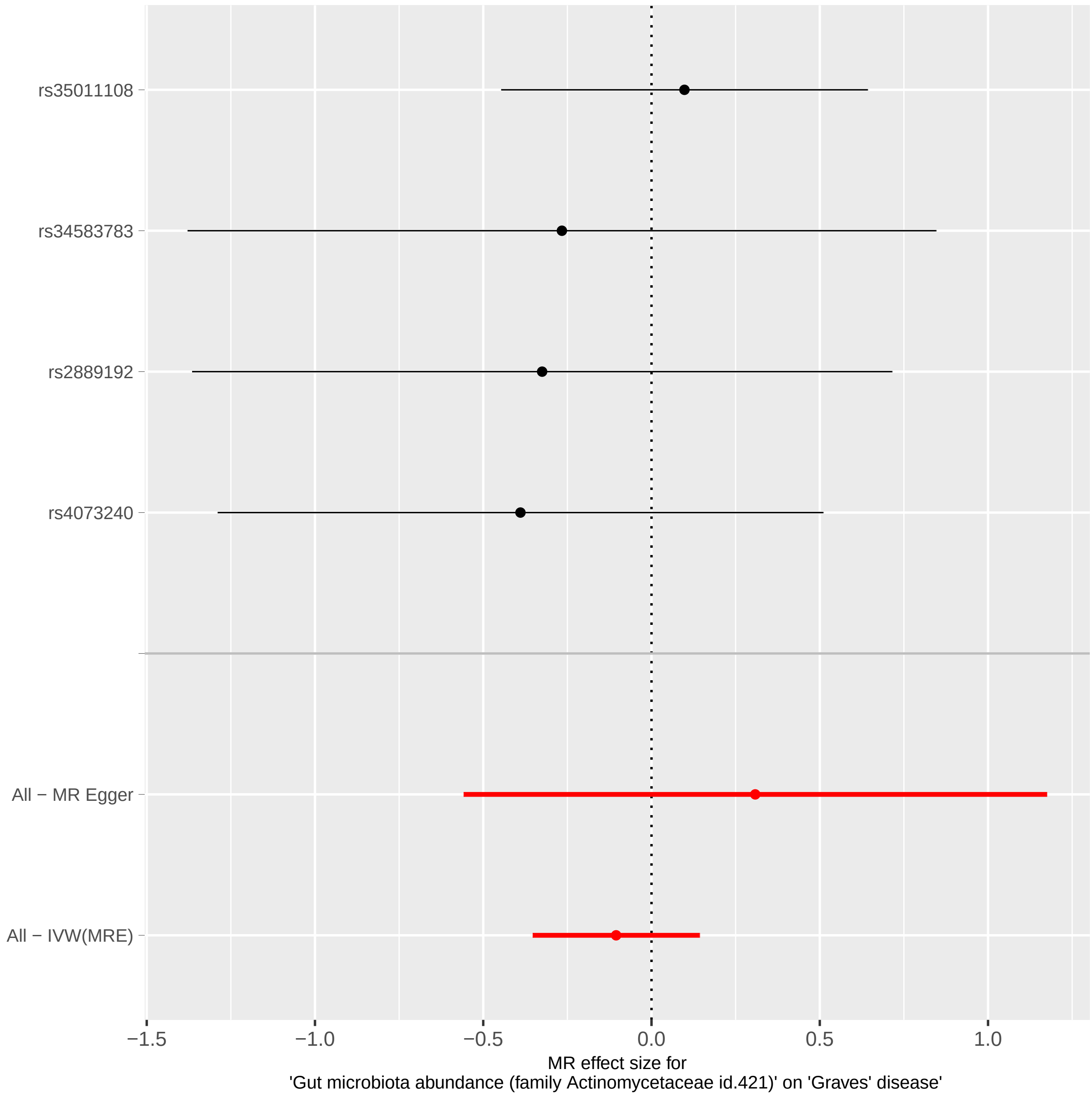

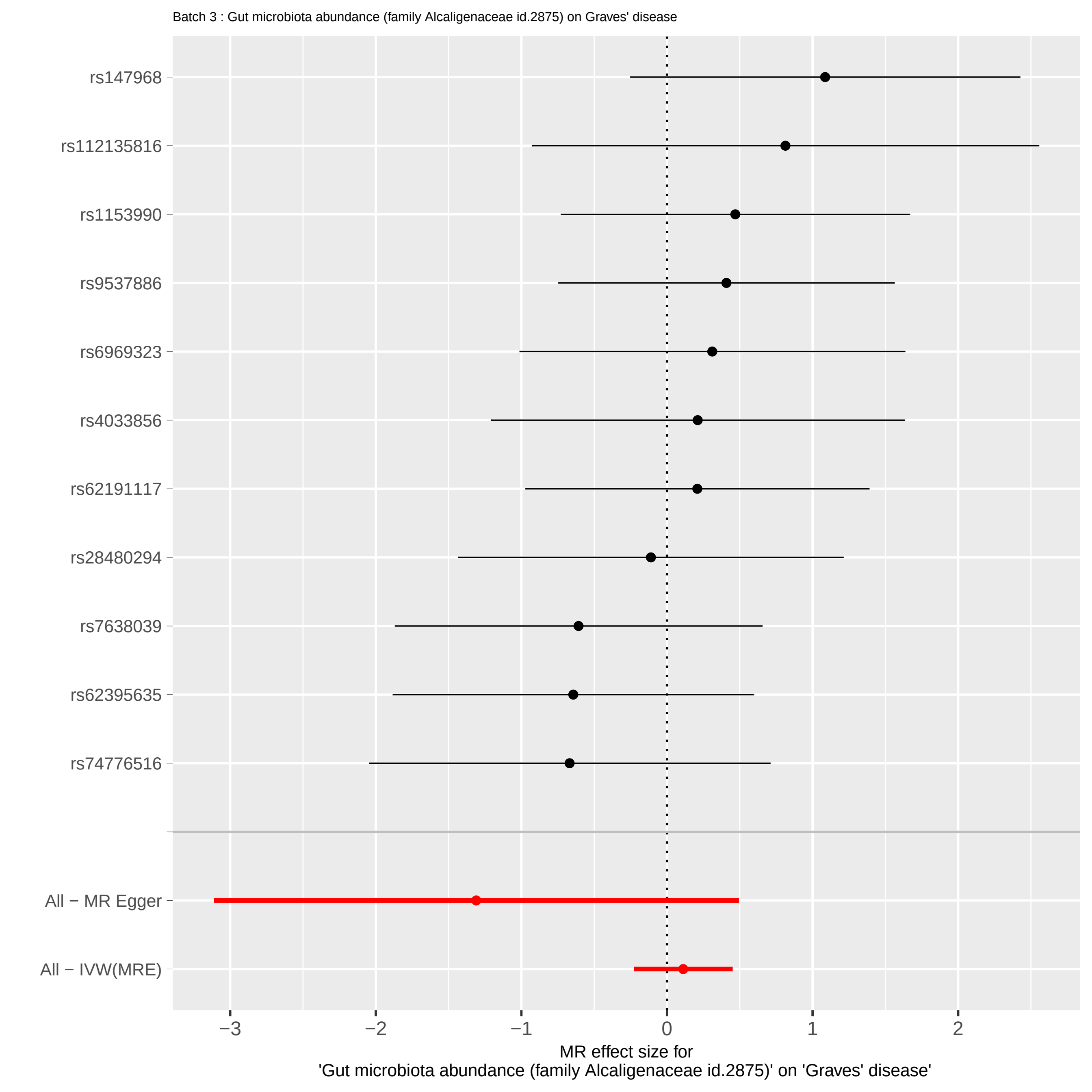

Batch 3 : Gut microbiota abundance (family Alcaligenaceae id.2875) on Graves' disease
rs147968
rs112135816
rs1153990
rs9537886
rs6969323
rs4033856
rs62191117
rs28480294
rs7638039
rs62395635
rs74776516
All − MR Egger
All − IVW(MRE)
−3
−2
−1
0
1
2
MR effect size for
'Gut microbiota abundance (family Alcaligenaceae id.2875)' on 'Graves' disease'

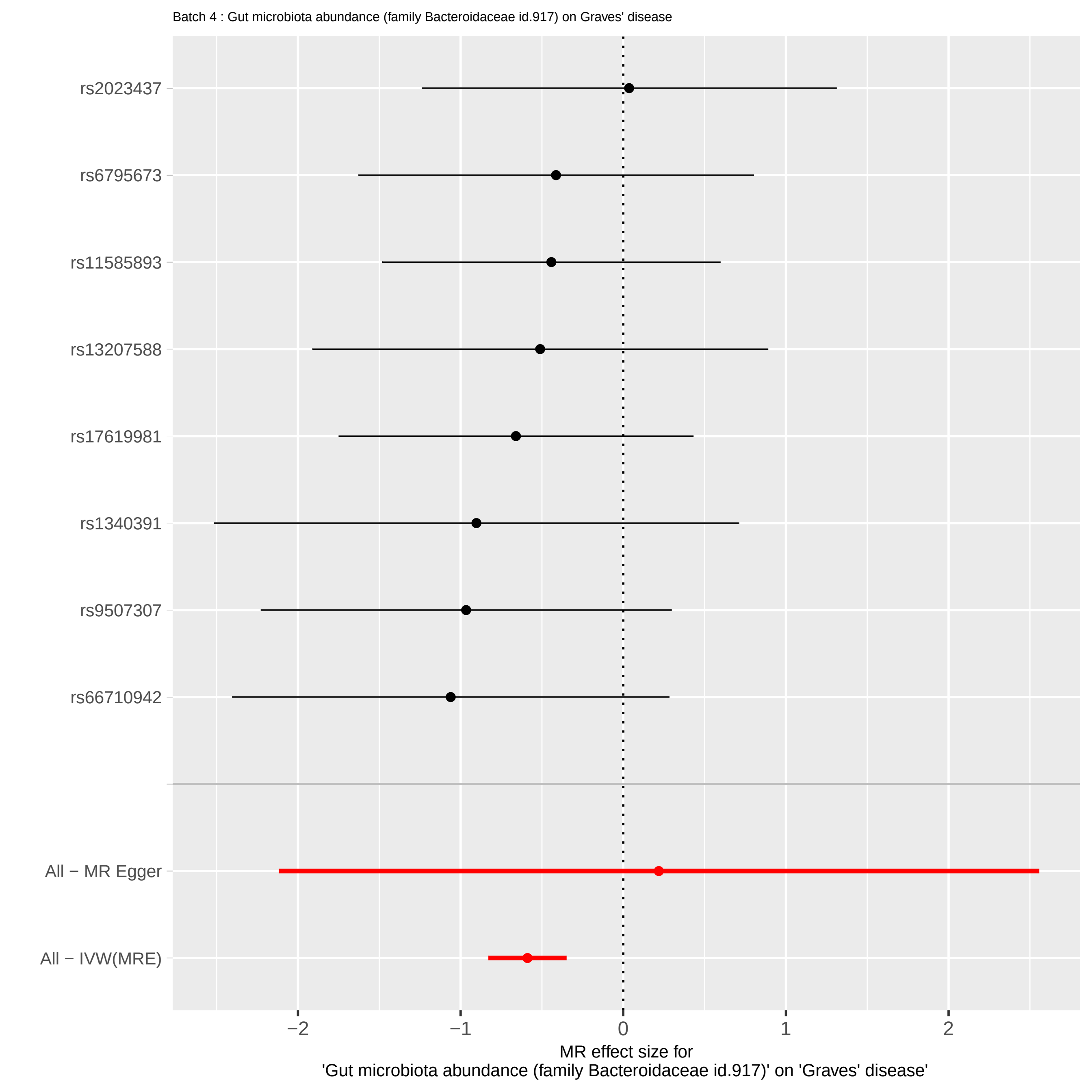
Batch 4 : Gut microbiota abundance (family Bacteroidaceae id.917) on Graves' disease
rs2023437
rs6795673
rs11585893
rs13207588
rs17619981
rs1340391
rs9507307
rs66710942
All − MR Egger
All − IVW(MRE)
−2
−1
0
1
2
MR effect size for
'Gut microbiota abundance (family Bacteroidaceae id.917)' on 'Graves' disease'

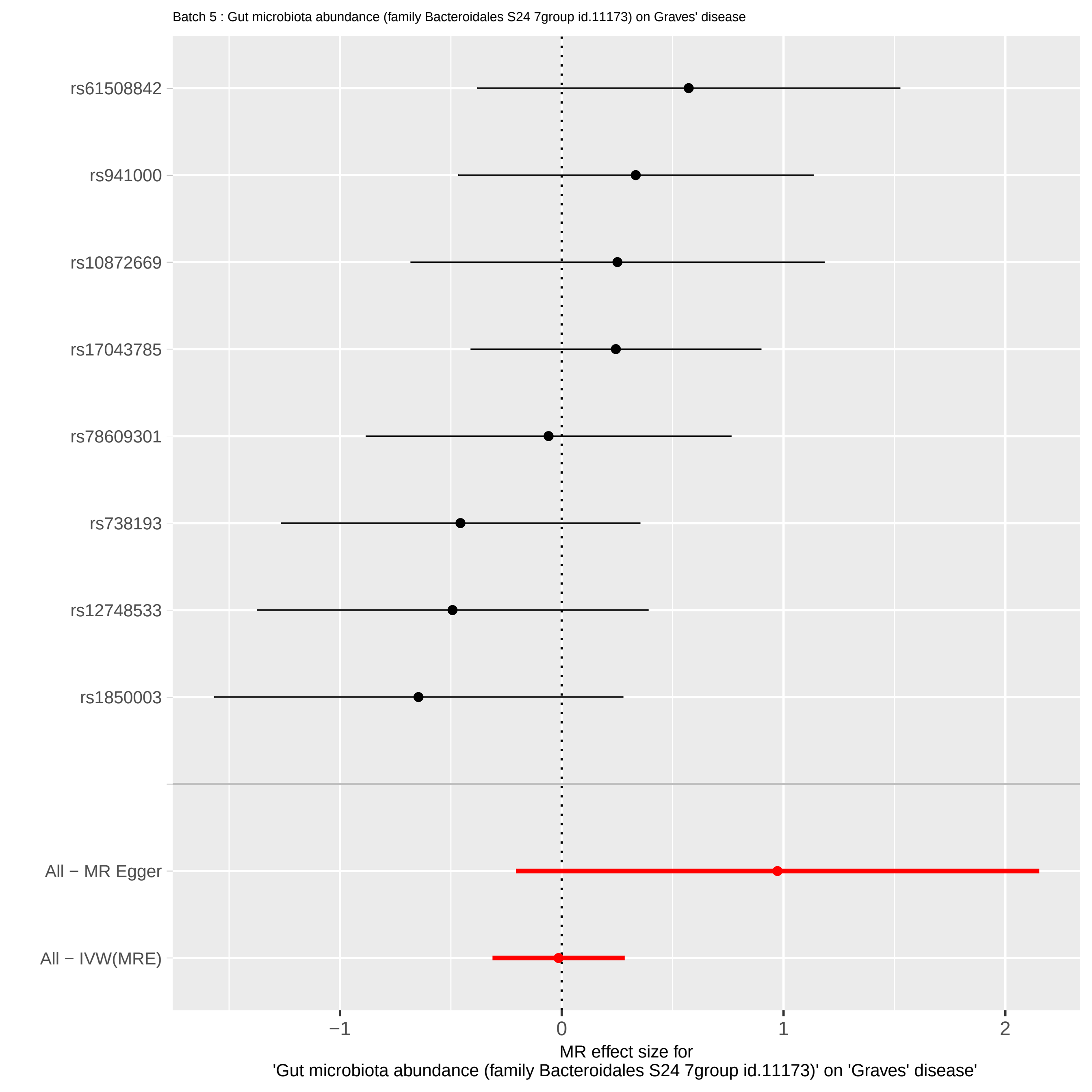

Batch 5 : Gut microbiota abundance (family Bacteroidales S24 7group id.11173) on Graves' disease
rs61508842
rs941000
rs10872669
rs17043785
rs78609301
rs738193
rs12748533
rs1850003
All − MR Egger
All − IVW(MRE)
−1
0
1
2
MR effect size for
'Gut microbiota abundance (family Bacteroidales S24 7group id.11173)' on 'Graves' disease'

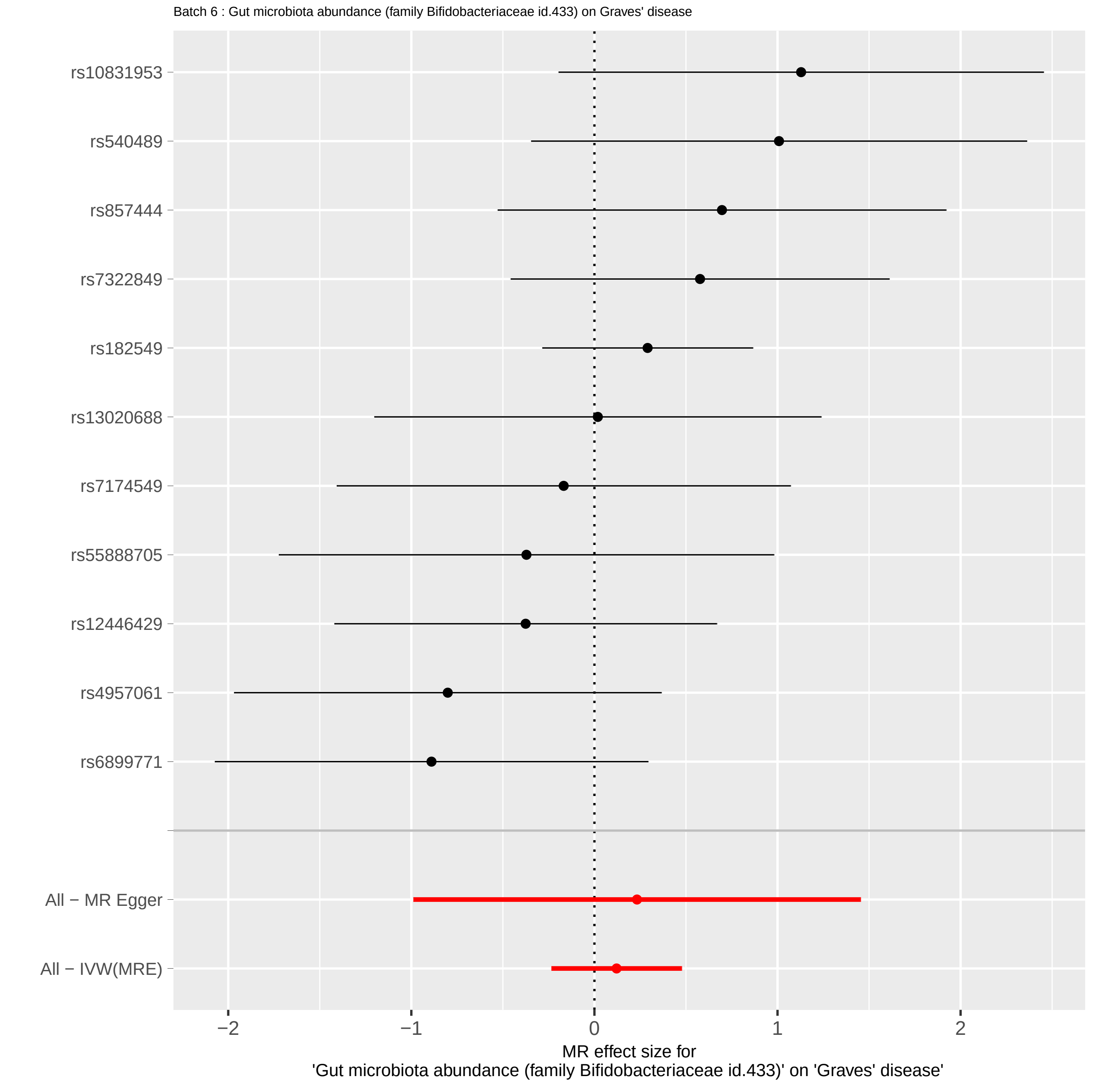

Batch 6 : Gut microbiota abundance (family Bifidobacteriaceae id.433) on Graves' disease
rs10831953
rs540489
rs857444
rs7322849
rs182549
rs13020688
rs7174549
rs55888705
rs12446429
rs4957061
rs6899771
All − MR Egger
All − IVW(MRE)
−2
−1
0
1
2
MR effect size for
'Gut microbiota abundance (family Bifidobacteriaceae id.433)' on 'Graves' disease'

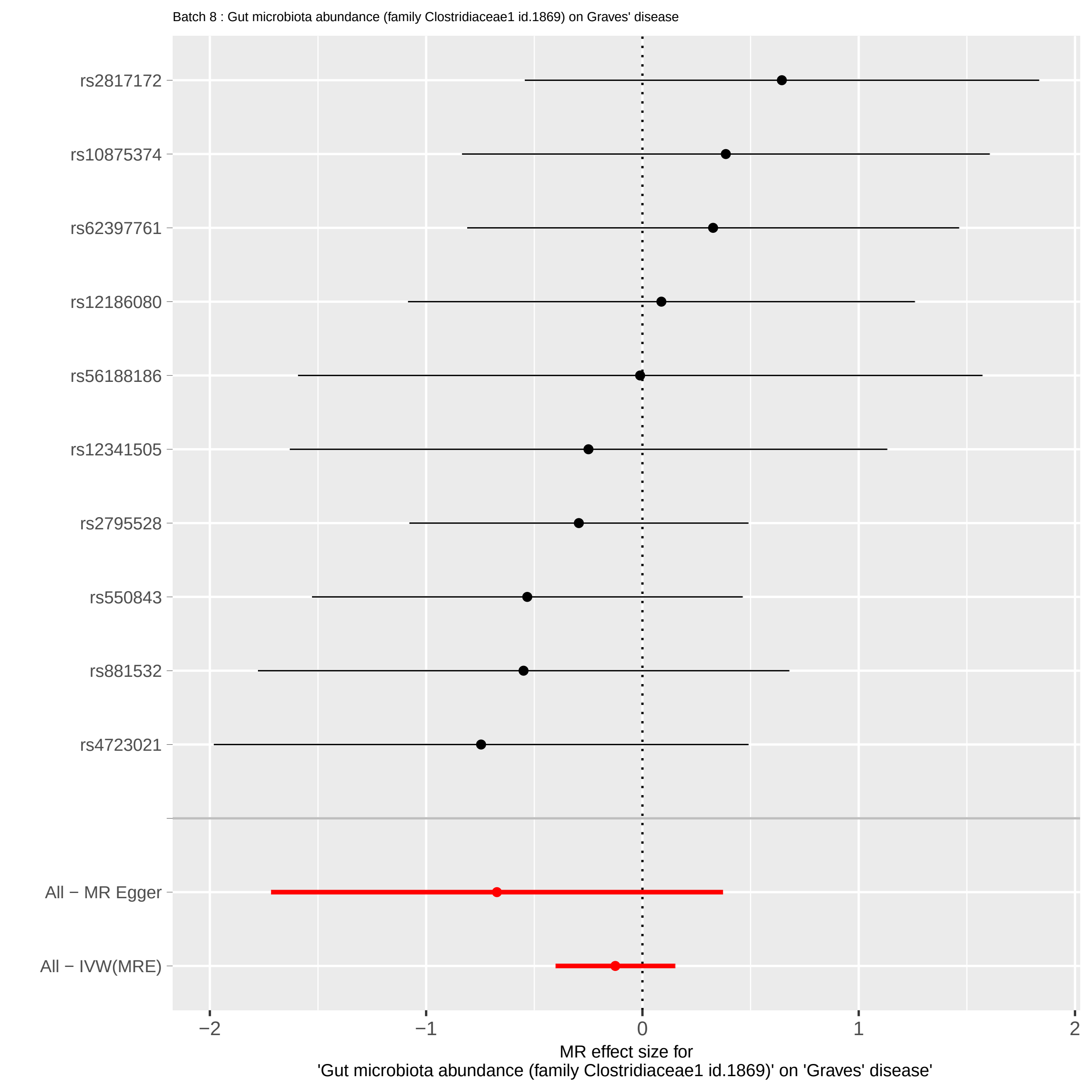

Batch 8 : Gut microbiota abundance (family Clostridiaceae1 id.1869) on Graves' disease
rs2817172
rs10875374
rs62397761
rs12186080
rs56188186
rs12341505
rs2795528
rs550843
rs881532
rs4723021
All − MR Egger
All − IVW(MRE)
−2
−1
0
1
2
MR effect size for
'Gut microbiota abundance (family Clostridiaceae1 id.1869)' on 'Graves' disease'

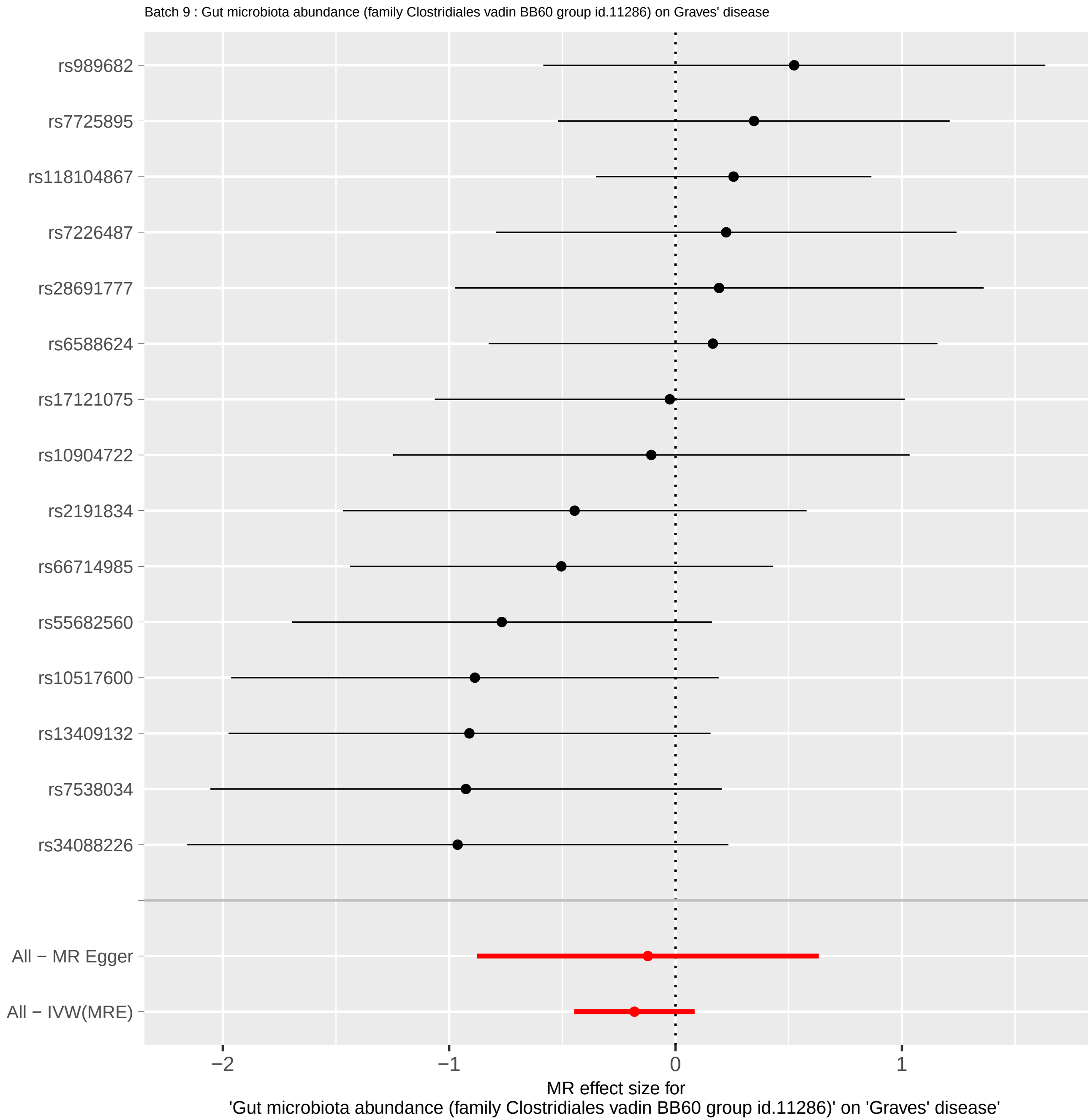
Batch 9 : Gut microbiota abundance (family Clostridiales vadin BB60 group id.11286) on Graves' disease
rs989682
rs7725895
rs118104867
rs7226487
rs28691777
rs6588624
rs17121075
rs10904722
rs2191834
rs66714985
rs55682560
rs10517600
rs13409132
rs7538034
rs34088226
All − MR Egger
All − IVW(MRE)
−2
−1
0
1
MR effect size for
'Gut microbiota abundance (family Clostridiales vadin BB60 group id.11286)' on 'Graves' disease'

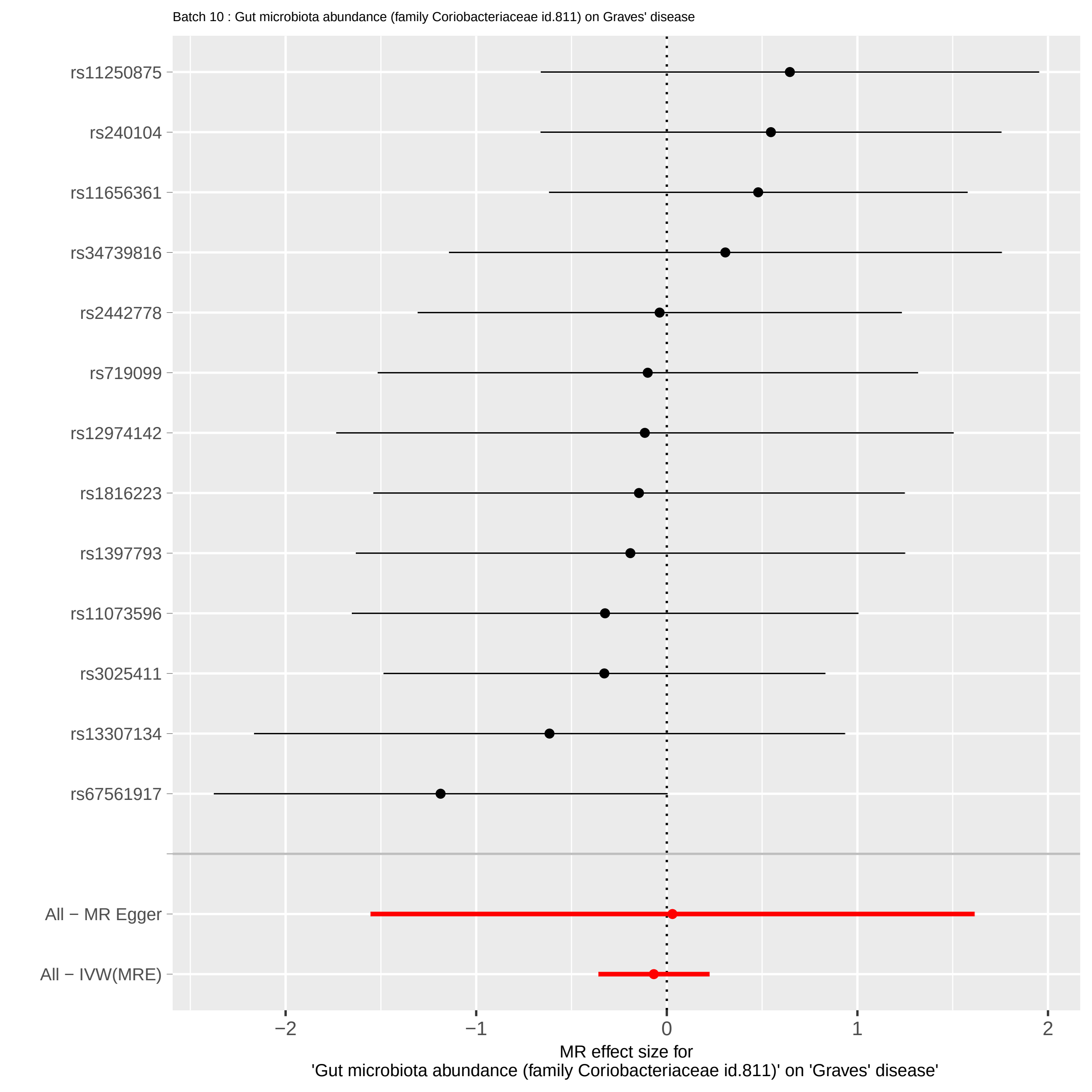
Batch 10 : Gut microbiota abundance (family Coriobacteriaceae id.811) on Graves' disease
rs11250875
rs240104
rs11656361
rs34739816
rs2442778
rs719099
rs12974142
rs1816223
rs1397793
rs11073596
rs3025411
rs13307134
rs67561917
All − MR Egger
All − IVW(MRE)
−2
−1
0
1
2
MR effect size for
'Gut microbiota abundance (family Coriobacteriaceae id.811)' on 'Graves' disease'

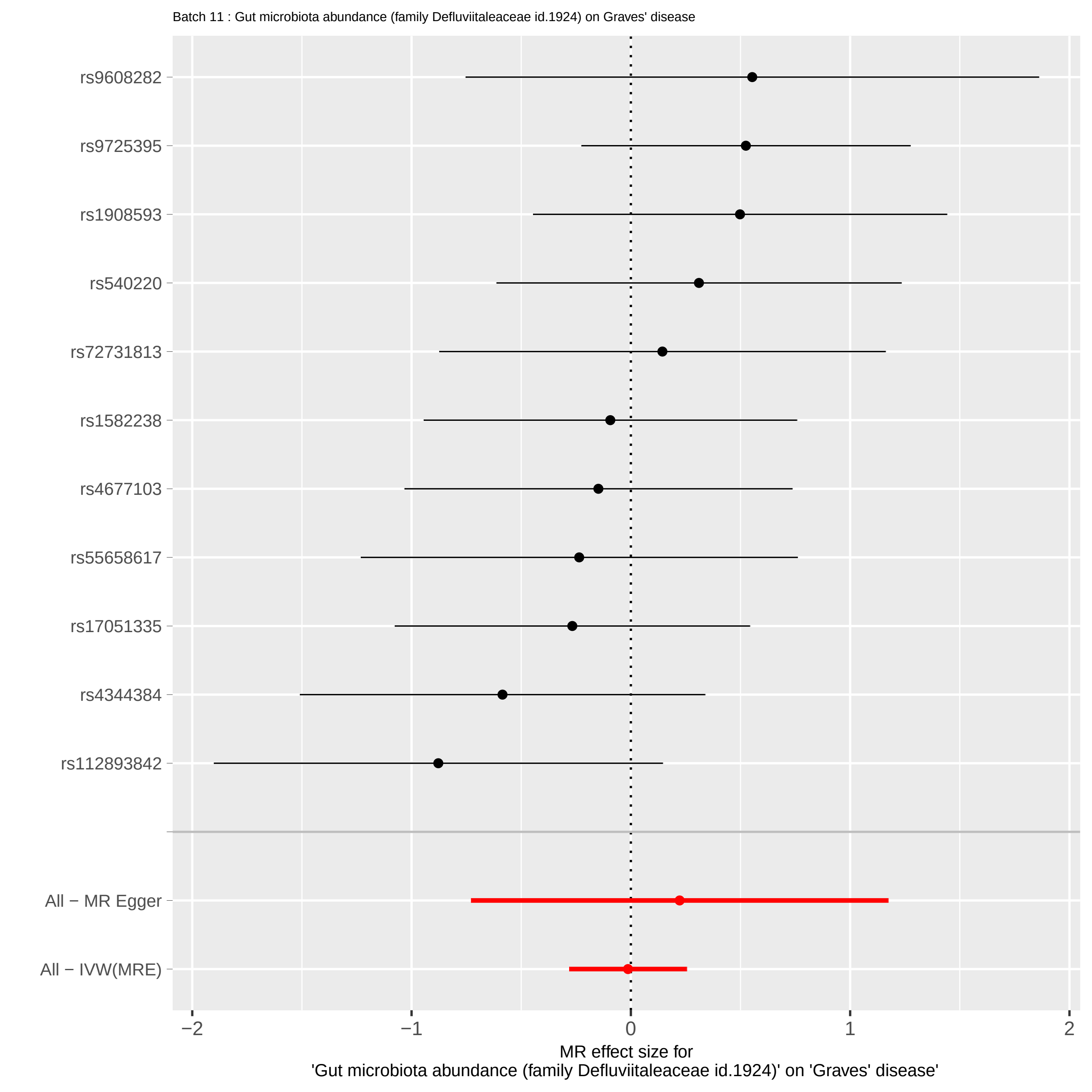
Batch 11 : Gut microbiota abundance (family Defluviitaleaceae id.1924) on Graves' disease
rs9608282
rs9725395
rs1908593
rs540220
rs72731813
rs1582238
rs4677103
rs55658617
rs17051335
rs4344384
rs112893842
All − MR Egger
All − IVW(MRE)
−2
−1
0
1
2
MR effect size for
'Gut microbiota abundance (family Defluviitaleaceae id.1924)' on 'Graves' disease'

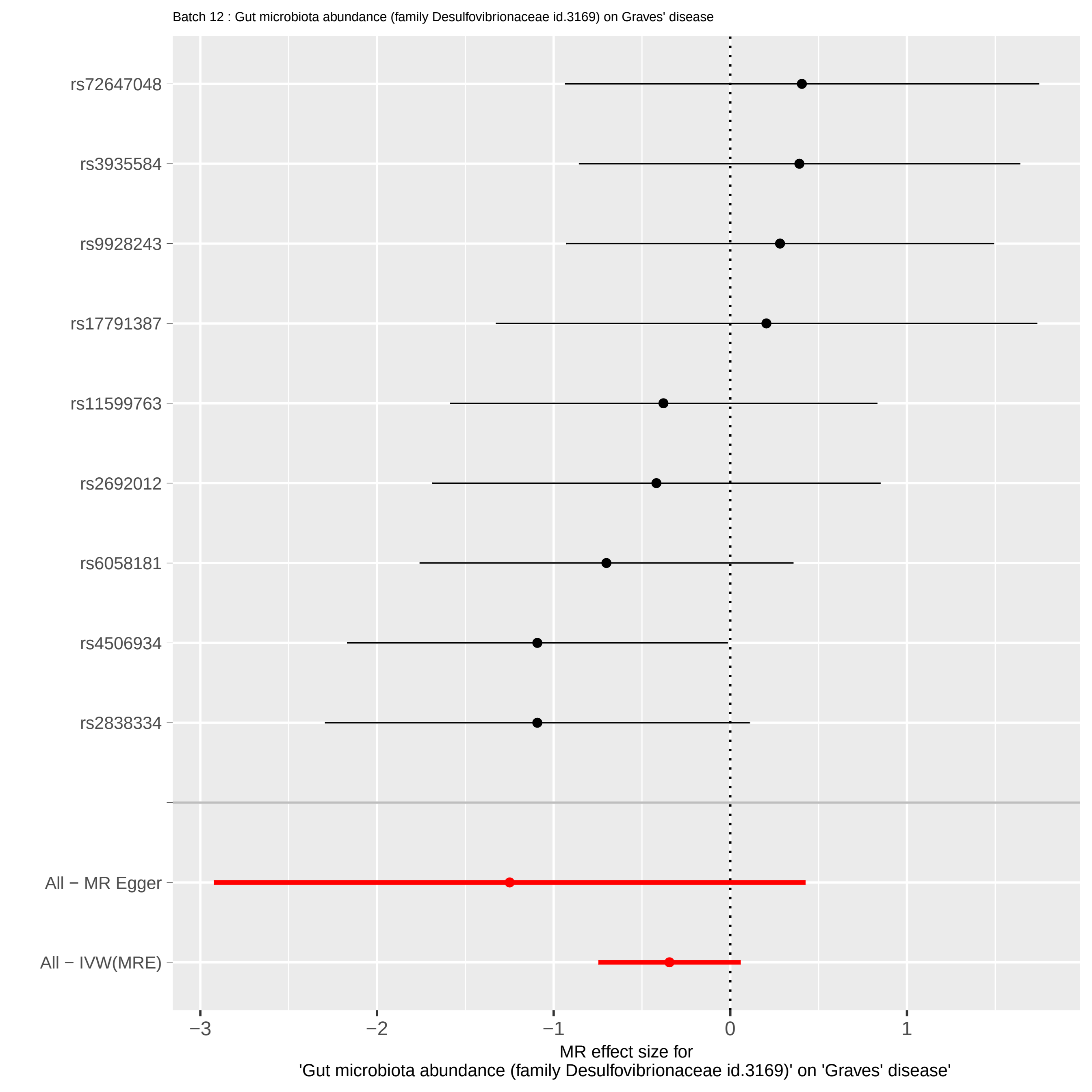

Batch 12 : Gut microbiota abundance (family Desulfovibrionaceae id.3169) on Graves' disease
rs72647048
rs3935584
rs9928243
rs17791387
rs11599763
rs2692012
rs6058181
rs4506934
rs2838334
All − MR Egger
All − IVW(MRE)
−3
−2
−1
0
1
MR effect size for
'Gut microbiota abundance (family Desulfovibrionaceae id.3169)' on 'Graves' disease'

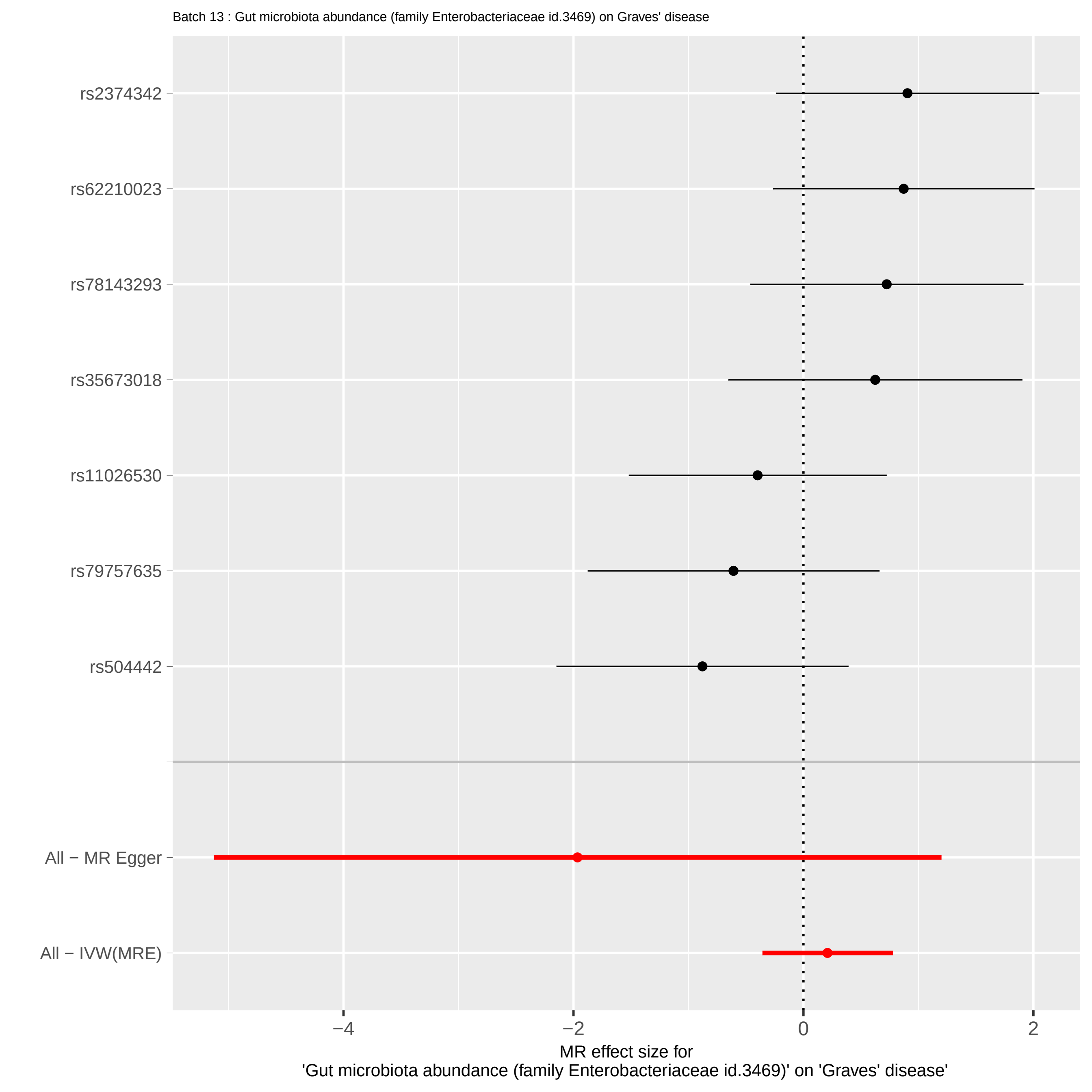

Batch 13 : Gut microbiota abundance (family Enterobacteriaceae id.3469) on Graves' disease
rs2374342
rs62210023
rs78143293
rs35673018
rs11026530
rs79757635
rs504442
All − MR Egger
All − IVW(MRE)
−4
−2
0
2
MR effect size for
'Gut microbiota abundance (family Enterobacteriaceae id.3469)' on 'Graves' disease'

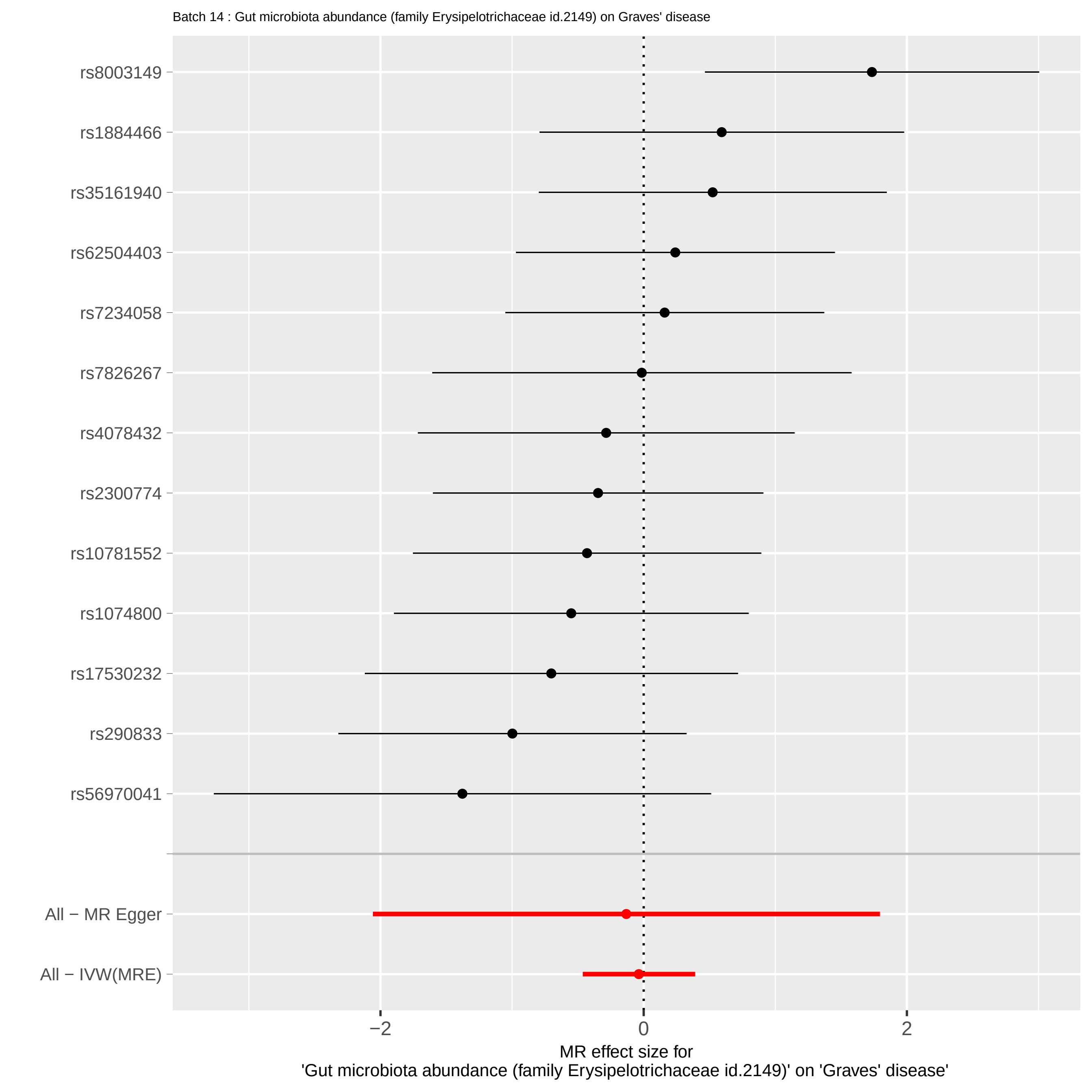
Batch 14 : Gut microbiota abundance (family Erysipelotrichaceae id.2149) on Graves' disease
rs8003149
rs1884466
rs35161940
rs62504403
rs7234058
rs7826267
rs4078432
rs2300774
rs10781552
rs1074800
rs17530232
rs290833
rs56970041
All − MR Egger
All − IVW(MRE)
−2
0
2
MR effect size for
'Gut microbiota abundance (family Erysipelotrichaceae id.2149)' on 'Graves' disease'

Batch 15 : Gut microbiota abundance (family Family XI id.1936) on Graves' disease

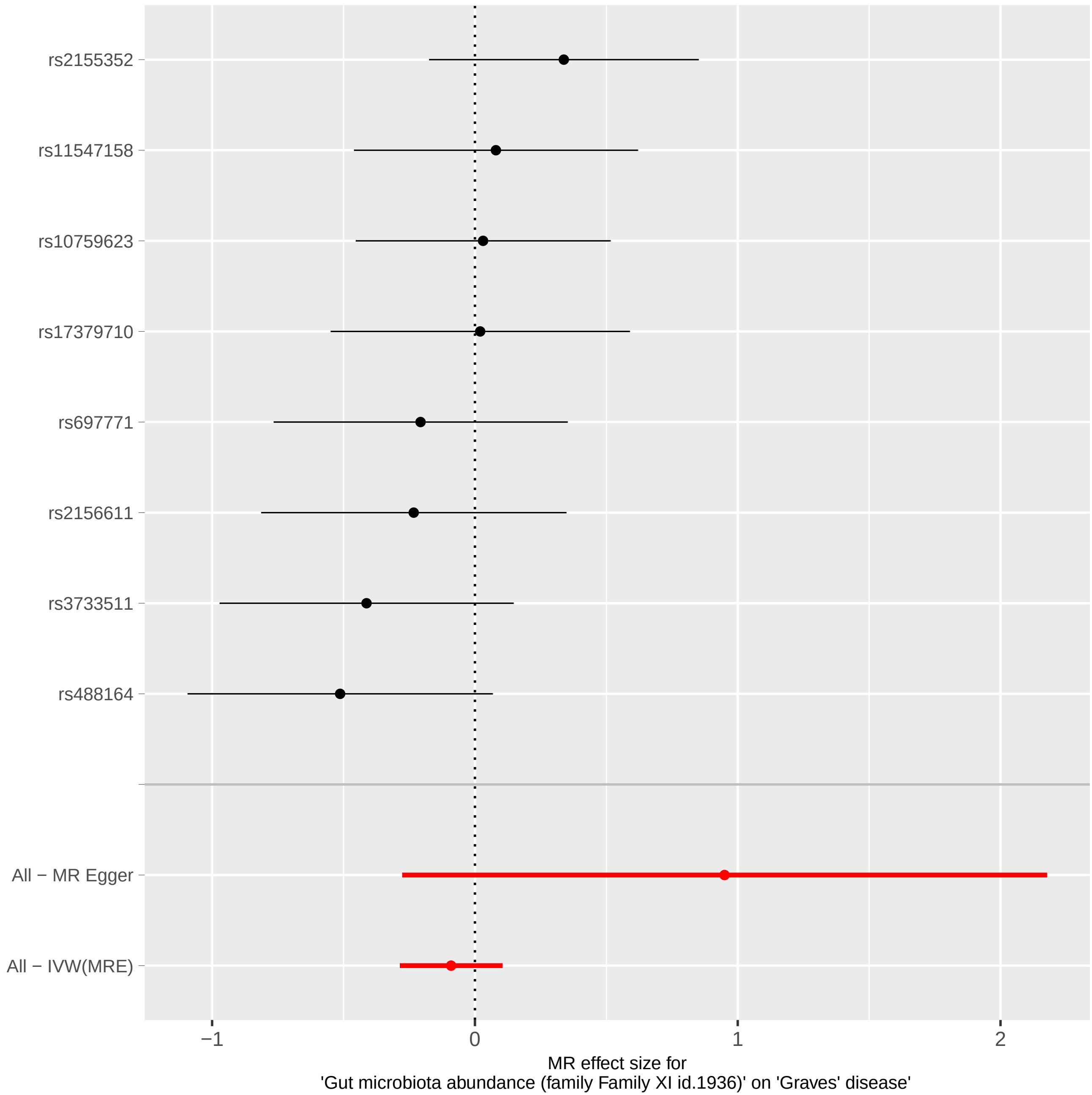

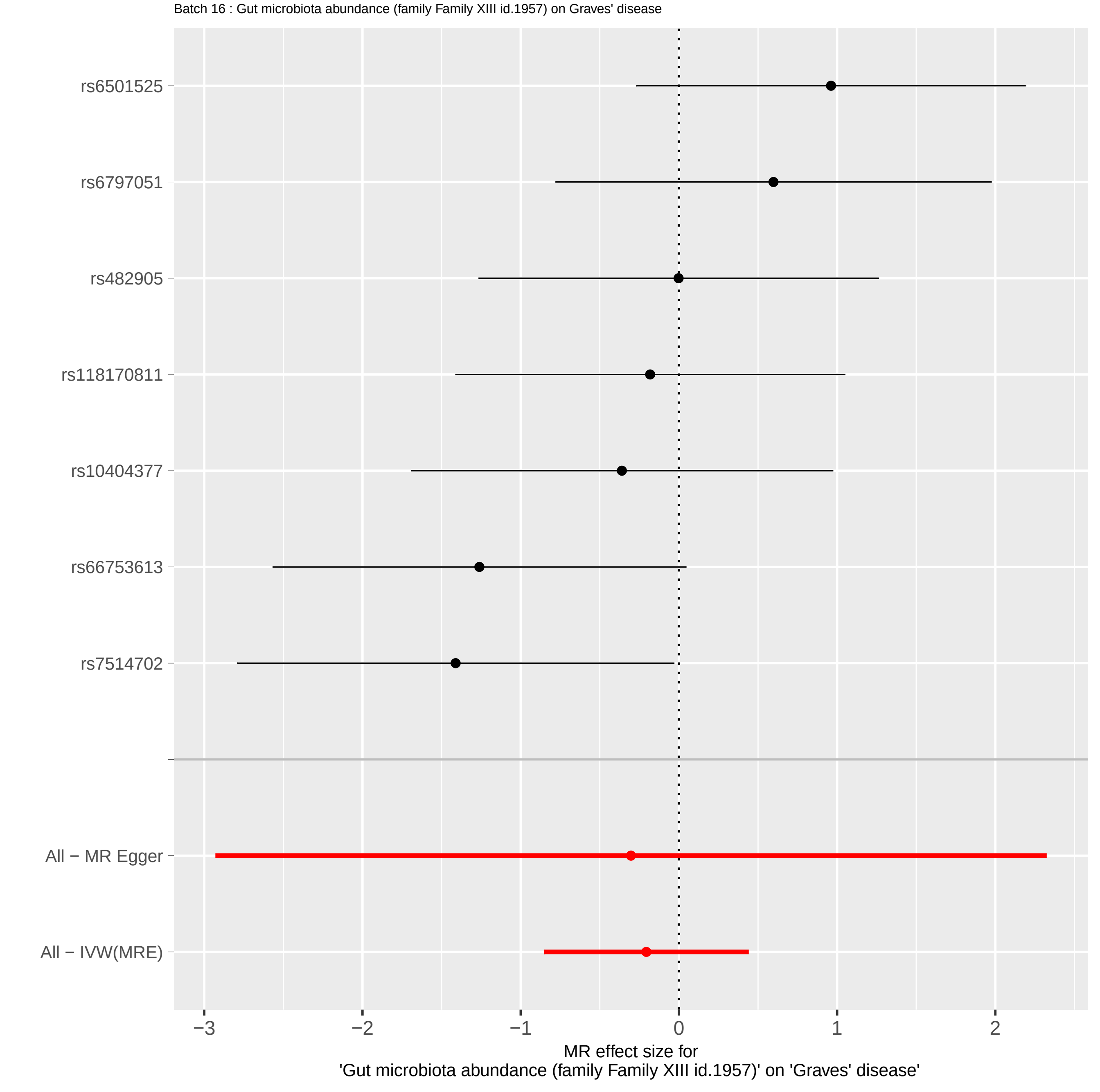
Batch 16 : Gut microbiota abundance (family Family XIII id.1957) on Graves' disease
rs6501525
rs6797051
rs482905
rs118170811
rs10404377
rs66753613
rs7514702
All − MR Egger
All − IVW(MRE)
−3
−2
−1
0
1
2
MR effect size for
'Gut microbiota abundance (family Family XIII id.1957)' on 'Graves' disease'

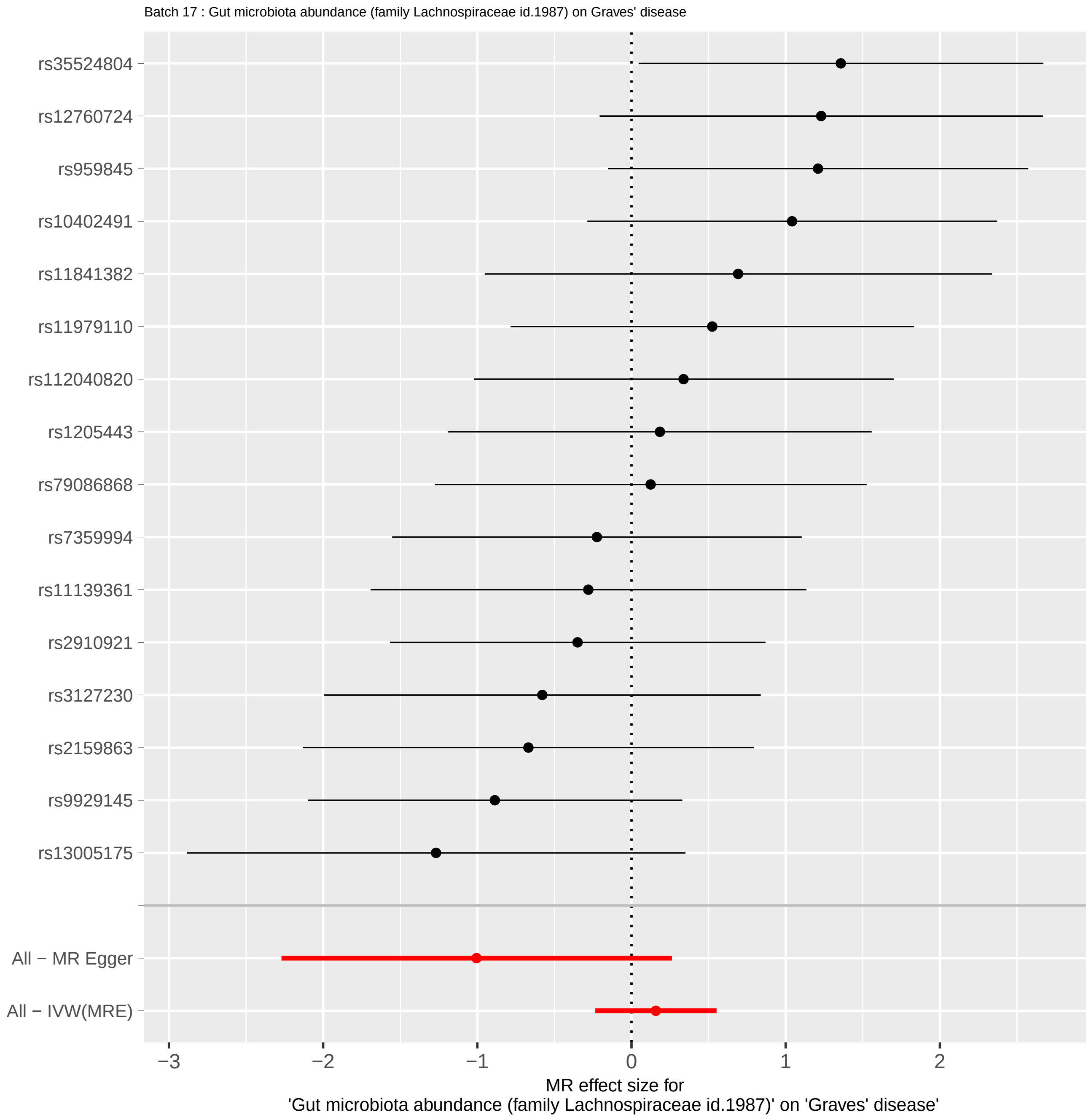

Batch 17 : Gut microbiota abundance (family Lachnospiraceae id.1987) on Graves' disease
rs35524804
rs12760724
rs959845
rs10402491
rs11841382
rs11979110
rs112040820
rs1205443
rs79086868
rs7359994
rs11139361
rs2910921
rs3127230
rs2159863
rs9929145
rs13005175
All − MR Egger
All − IVW(MRE)
−3
−2
−1
0
1
2
MR effect size for
'Gut microbiota abundance (family Lachnospiraceae id.1987)' on 'Graves' disease'

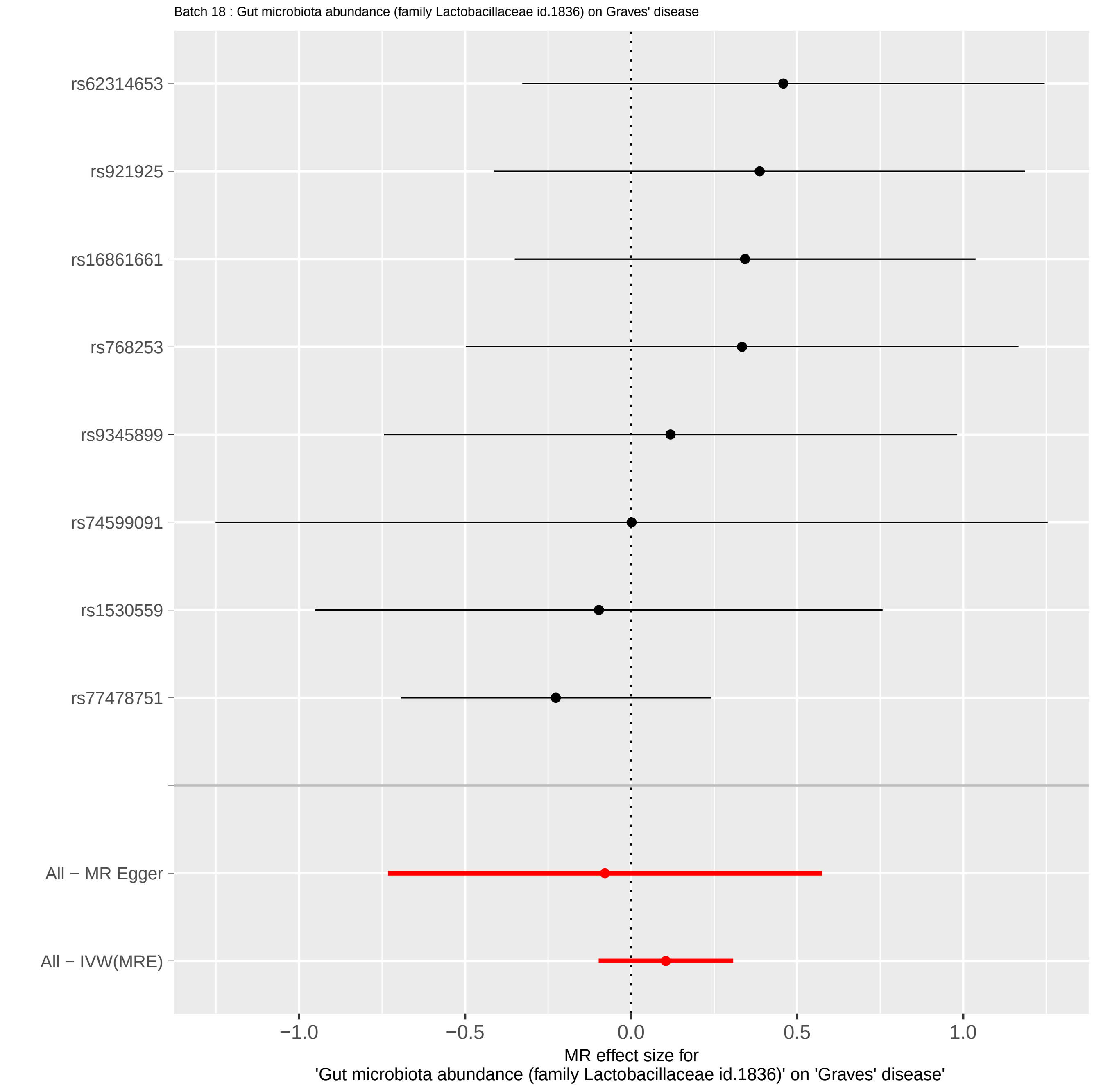
Batch 18 : Gut microbiota abundance (family Lactobacillaceae id.1836) on Graves' disease
rs62314653
rs921925
rs16861661
rs768253
rs9345899
rs74599091
rs1530559
rs77478751
All − MR Egger
All − IVW(MRE)
−1.0
−0.5
0.0
0.5
1.0
MR effect size for
'Gut microbiota abundance (family Lactobacillaceae id.1836)' on 'Graves' disease'

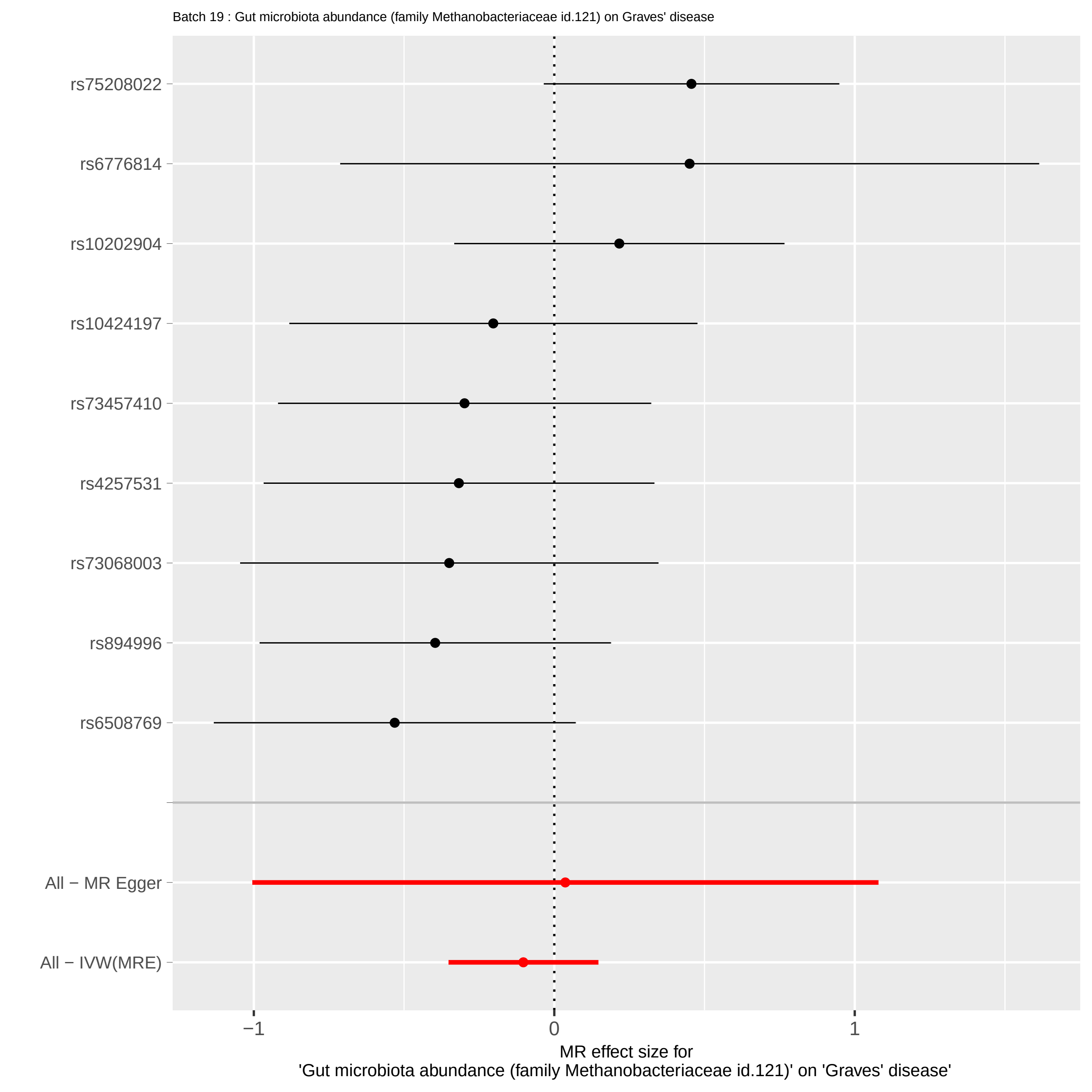

Batch 19 : Gut microbiota abundance (family Methanobacteriaceae id.121) on Graves' disease
rs75208022
rs6776814
rs10202904
rs10424197
rs73457410
rs4257531
rs73068003
rs894996
rs6508769
All − MR Egger
All − IVW(MRE)
−1
0
1
MR effect size for
'Gut microbiota abundance (family Methanobacteriaceae id.121)' on 'Graves' disease'

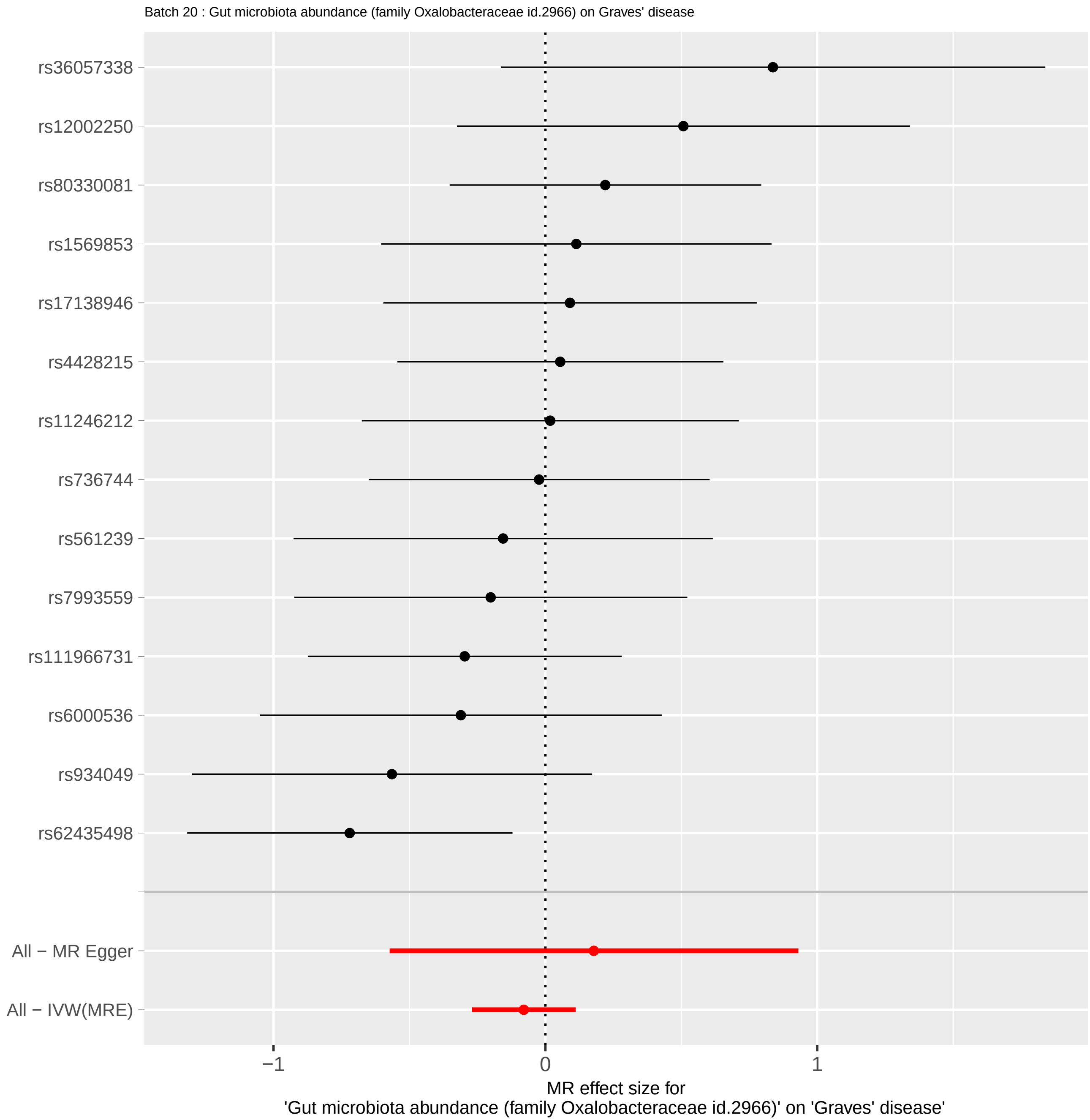

Batch 20 : Gut microbiota abundance (family Oxalobacteraceae id.2966) on Graves' disease
rs36057338
rs12002250
rs80330081
rs1569853
rs17138946
rs4428215
rs11246212
rs736744
rs561239
rs7993559
rs111966731
rs6000536
rs934049
rs62435498
All − MR Egger
All − IVW(MRE)
−1
0
1
MR effect size for
'Gut microbiota abundance (family Oxalobacteraceae id.2966)' on 'Graves' disease'

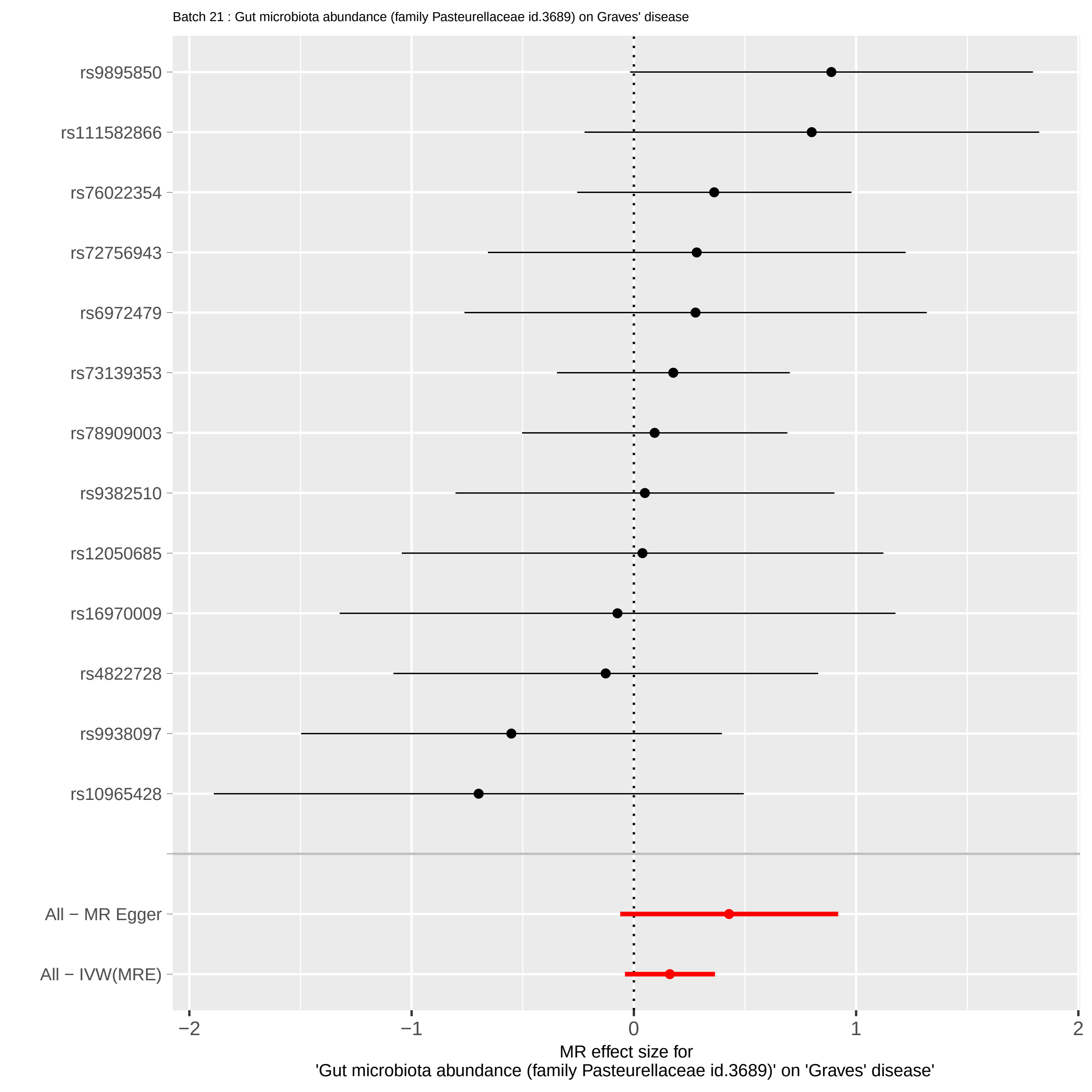
Batch 21 : Gut microbiota abundance (family Pasteurellaceae id.3689) on Graves' disease
rs9895850
rs111582866
rs76022354
rs72756943
rs6972479
rs73139353
rs78909003
rs9382510
rs12050685
rs16970009
rs4822728
rs9938097
rs10965428
All − MR Egger
All − IVW(MRE)
−2
−1
0
1
2
MR effect size for
'Gut microbiota abundance (family Pasteurellaceae id.3689)' on 'Graves' disease

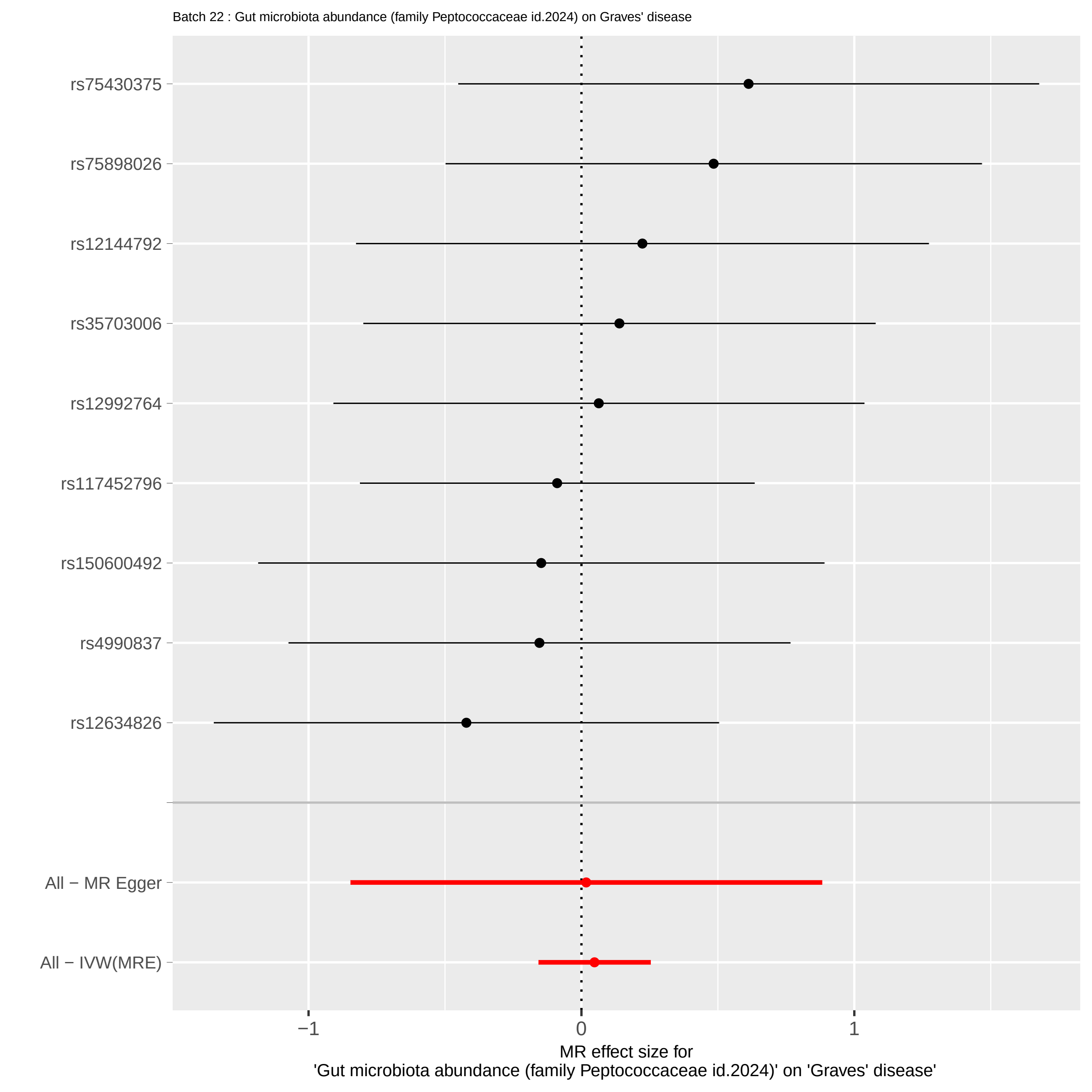

Batch 22 : Gut microbiota abundance (family Peptococcaceae id.2024) on Graves' disease
rs75430375
rs75898026
rs12144792
rs35703006
rs12992764
rs117452796
rs150600492
rs4990837
rs12634826
All − MR Egger
All − IVW(MRE)
−1
0
1
MR effect size for
'Gut microbiota abundance (family Peptococcaceae id.2024)' on 'Graves' disease'

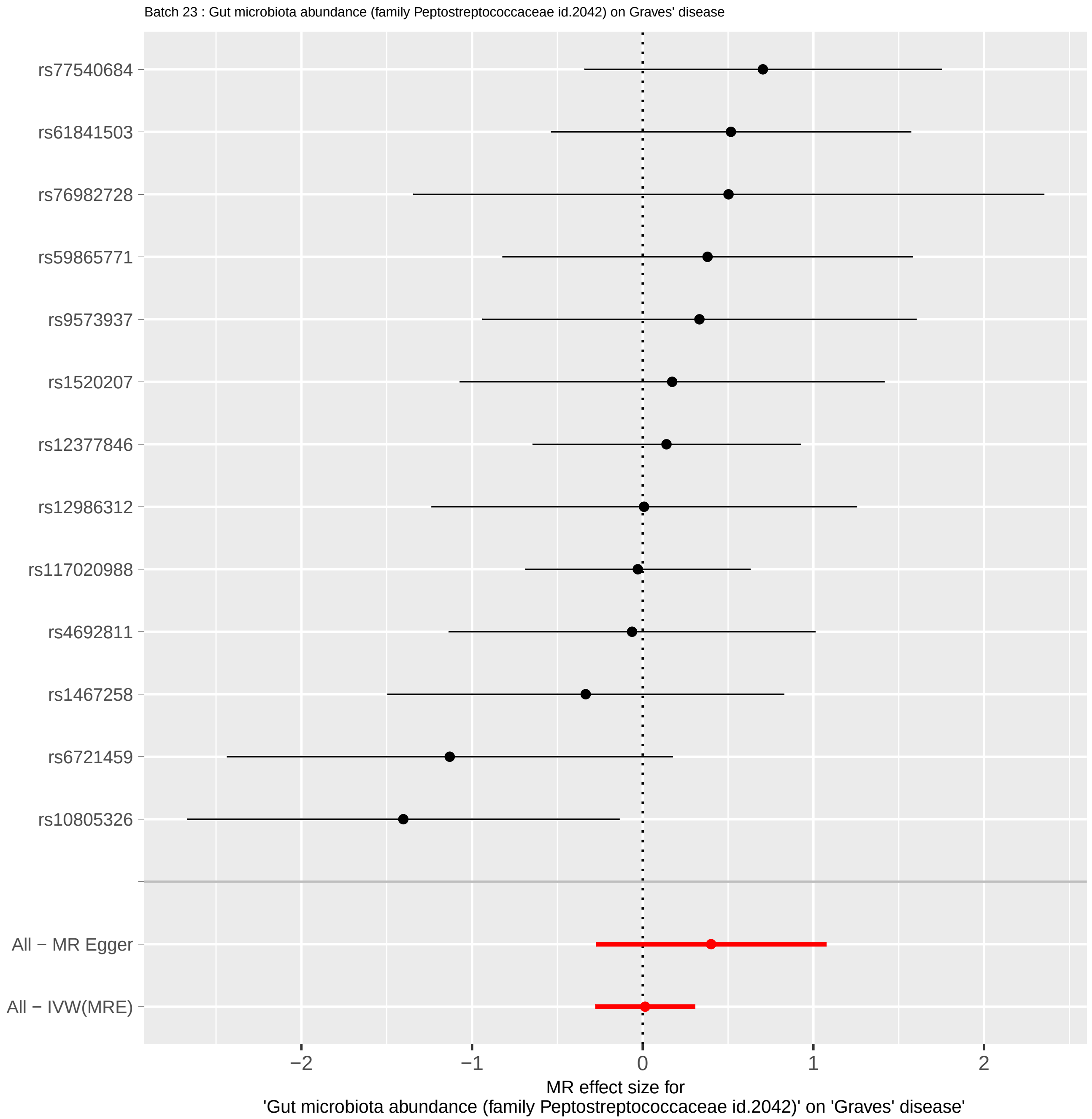
Batch 23 : Gut microbiota abundance (family Peptostreptococcaceae id.2042) on Graves' disease
rs77540684
rs61841503
rs76982728
rs59865771
rs9573937
rs1520207
rs12377846
rs12986312
rs117020988
rs4692811
rs1467258
rs6721459
rs10805326
All − MR Egger
All − IVW(MRE)
−2
−1
0
1
2
MR effect size for
'Gut microbiota abundance (family Peptostreptococcaceae id.2042)' on 'Graves' disease'

Batch 24 : Gut microbiota abundance (family Porphyromonadaceae id.943) on Graves' disease

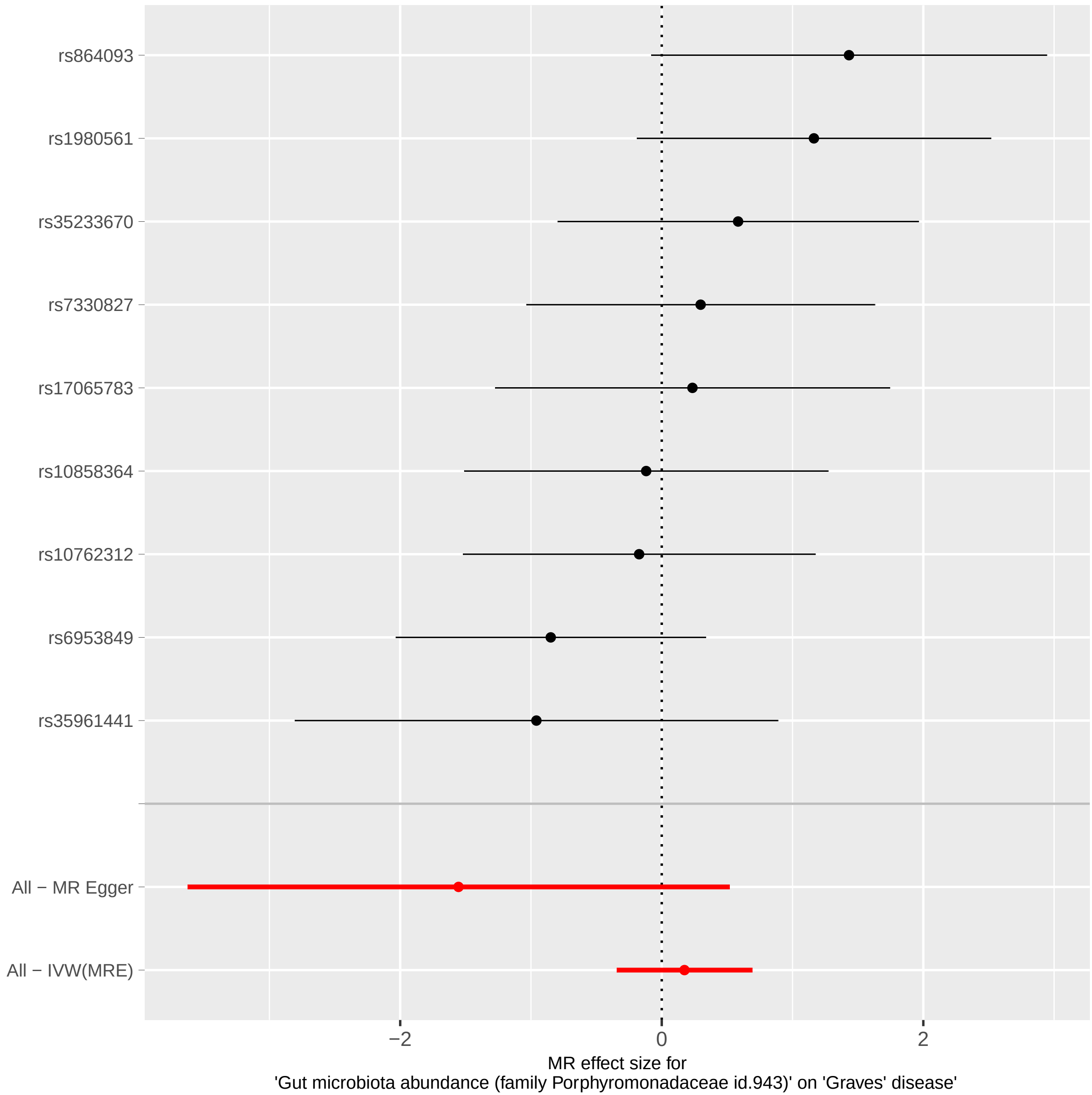

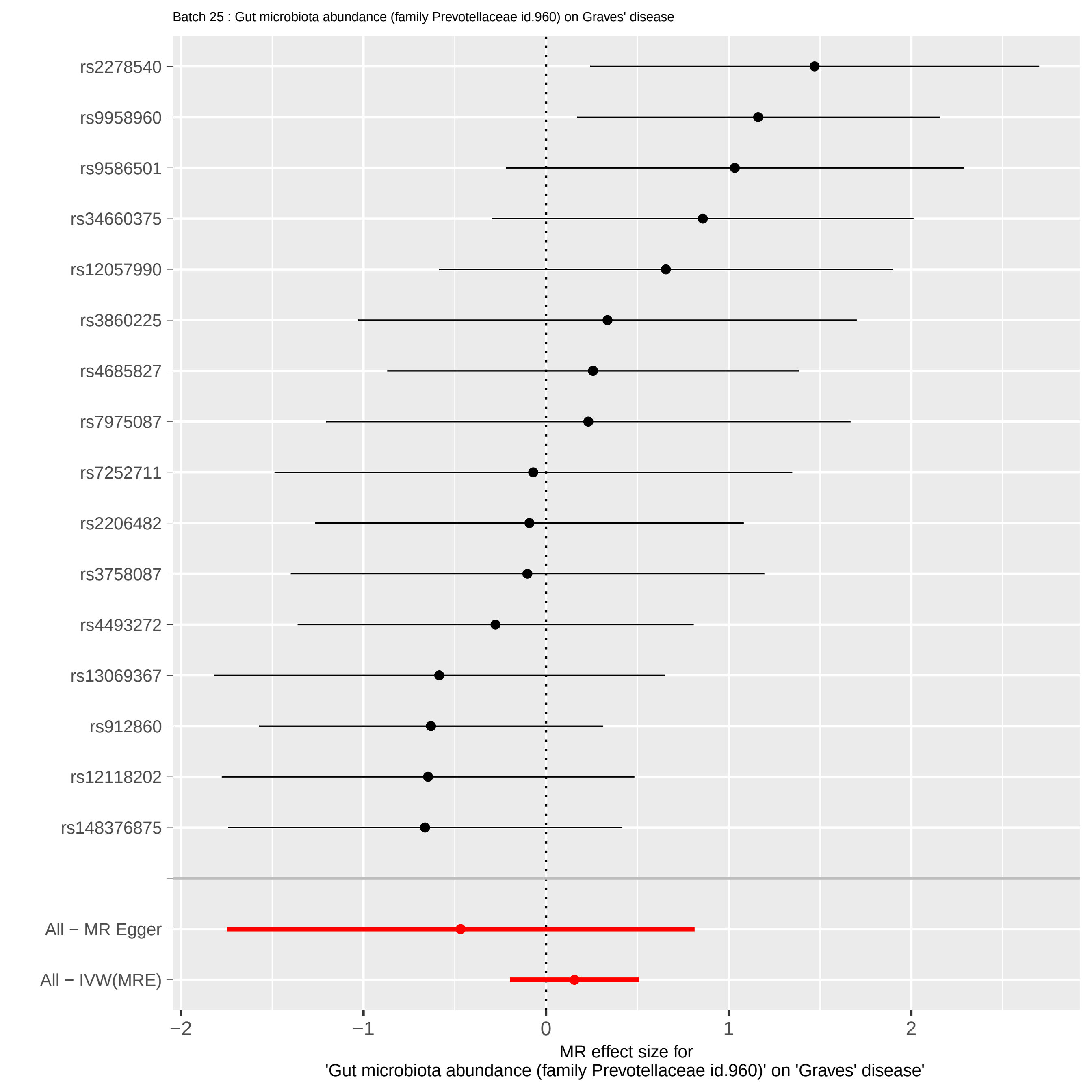
Batch 25 : Gut microbiota abundance (family Prevotellaceae id.960) on Graves' disease
rs2278540
rs9958960
rs9586501
rs34660375
rs12057990
rs3860225
rs4685827
rs7975087
rs7252711
rs2206482
rs3758087
rs4493272
rs13069367
rs912860
rs12118202
rs148376875
All − MR Egger
All − IVW(MRE)
−2
−1
0
1
2
MR effect size for
'Gut microbiota abundance (family Prevotellaceae id.960)' on 'Graves' disease'

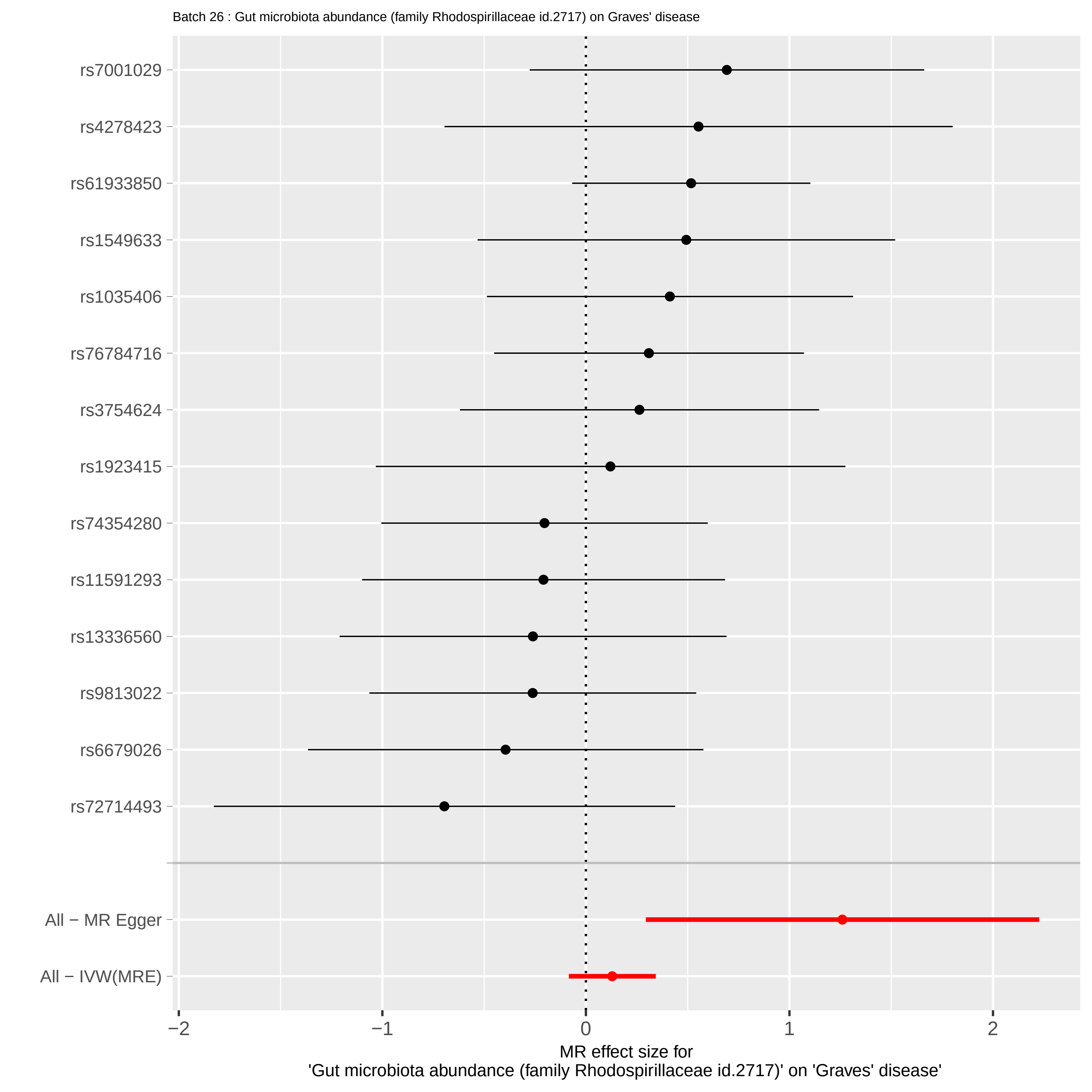

Batch 26 : Gut microbiota abundance (family Rhodospirillaceae id.2717) on Graves' disease
rs7001029
rs4278423
rs61933850
rs1549633
rs1035406
rs76784716
rs3754624
rs1923415
rs74354280
rs11591293
rs13336560
rs9813022
rs6679026
rs72714493
All − MR Egger
All − IVW(MRE)
−2
−1
0
1
2
MR effect size for
'Gut microbiota abundance (family Rhodospirillaceae id.2717)' on 'Graves' disease'

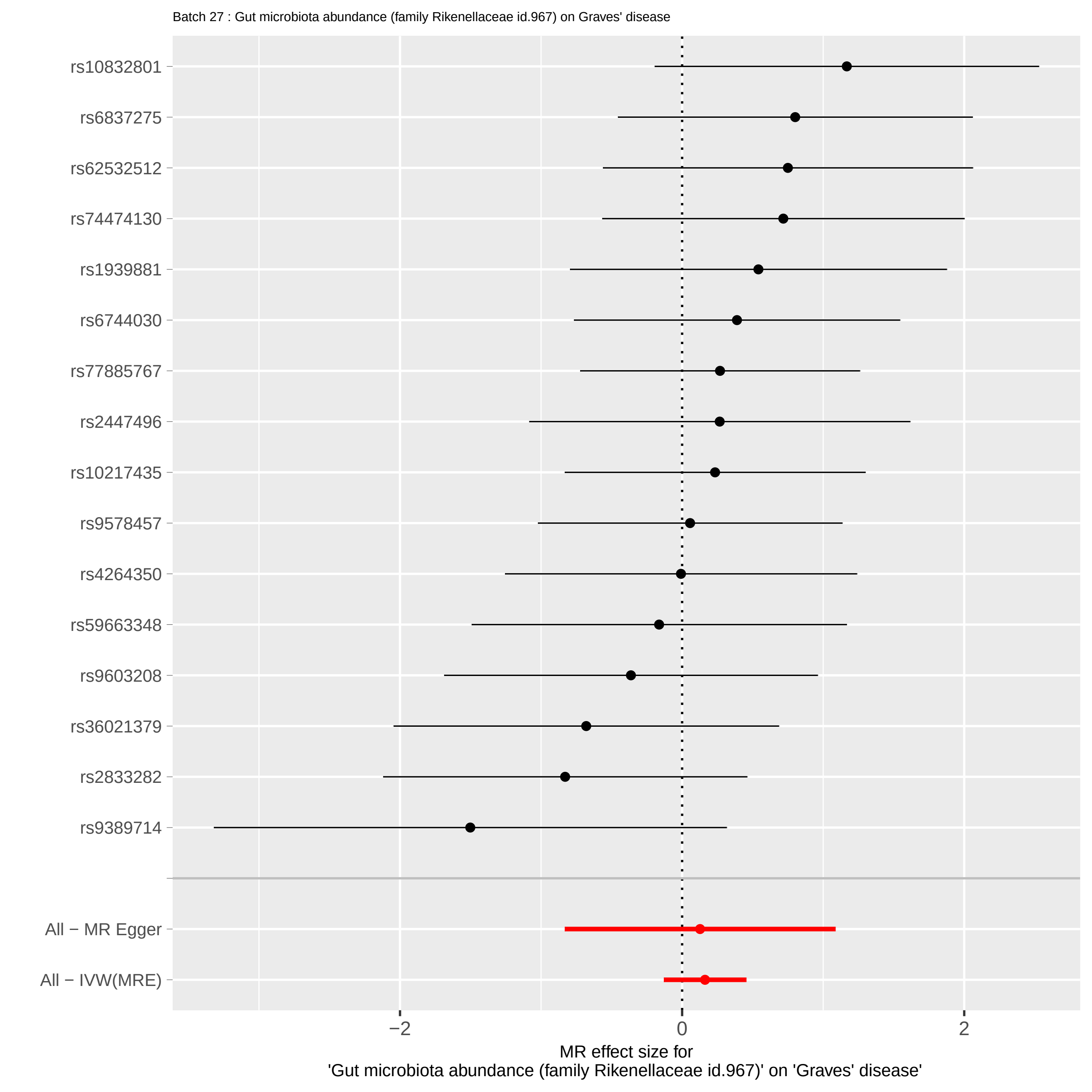

Batch 27 : Gut microbiota abundance (family Rikenellaceae id.967) on Graves' disease
rs10832801
rs6837275
rs62532512
rs74474130
rs1939881
rs6744030
rs77885767
rs2447496
rs10217435
rs9578457
rs4264350
rs59663348
rs9603208
rs36021379
rs2833282
rs9389714
All − MR Egger
All − IVW(MRE)
−2
0
2
MR effect size for
'Gut microbiota abundance (family Rikenellaceae id.967)' on 'Graves' disease'

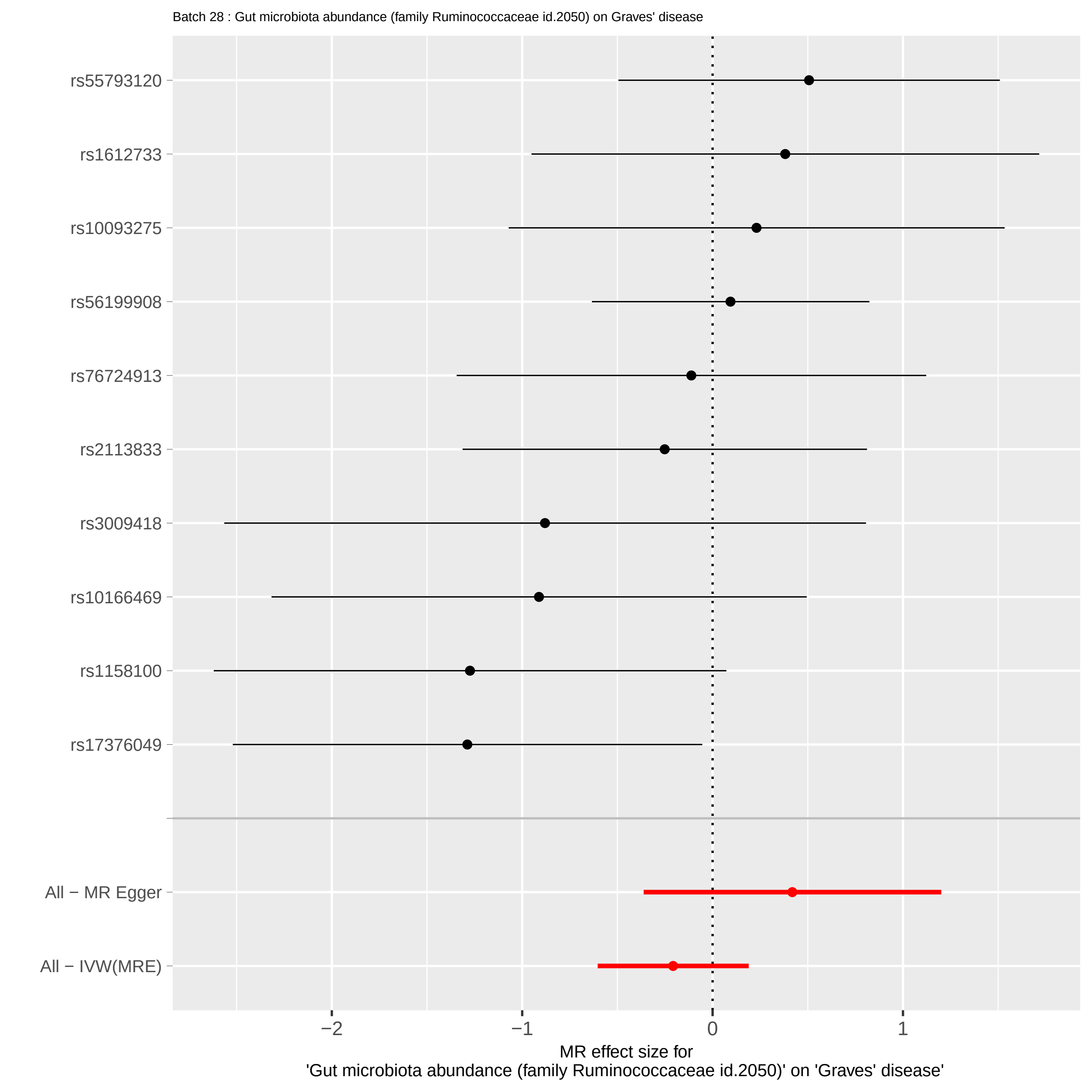

Batch 28 : Gut microbiota abundance (family Ruminococcaceae id.2050) on Graves' disease
rs55793120
rs1612733
rs10093275
rs56199908
rs76724913
rs2113833
rs3009418
rs10166469
rs1158100
rs17376049
All − MR Egger
All − IVW(MRE)
−2
−1
0
1
MR effect size for
'Gut microbiota abundance (family Ruminococcaceae id.2050)' on 'Graves' disease'

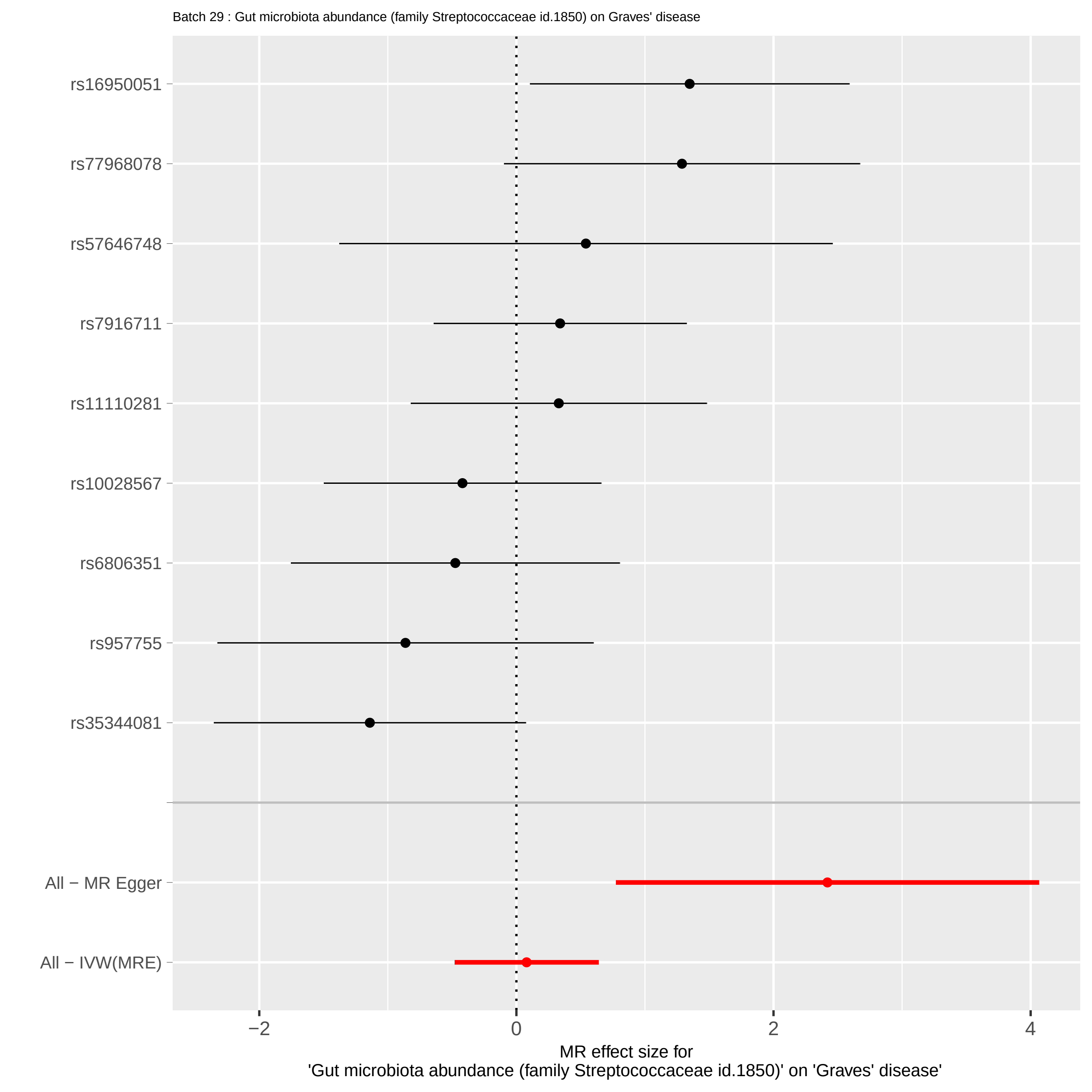
Batch 29 : Gut microbiota abundance (family Streptococcaceae id.1850) on Graves' disease
rs16950051
rs77968078
rs57646748
rs7916711
rs11110281
rs10028567
rs6806351
rs957755
rs35344081
All − MR Egger
All − IVW(MRE)
−2
0
2
4
MR effect size for
'Gut microbiota abundance (family Streptococcaceae id.1850)' on 'Graves' disease

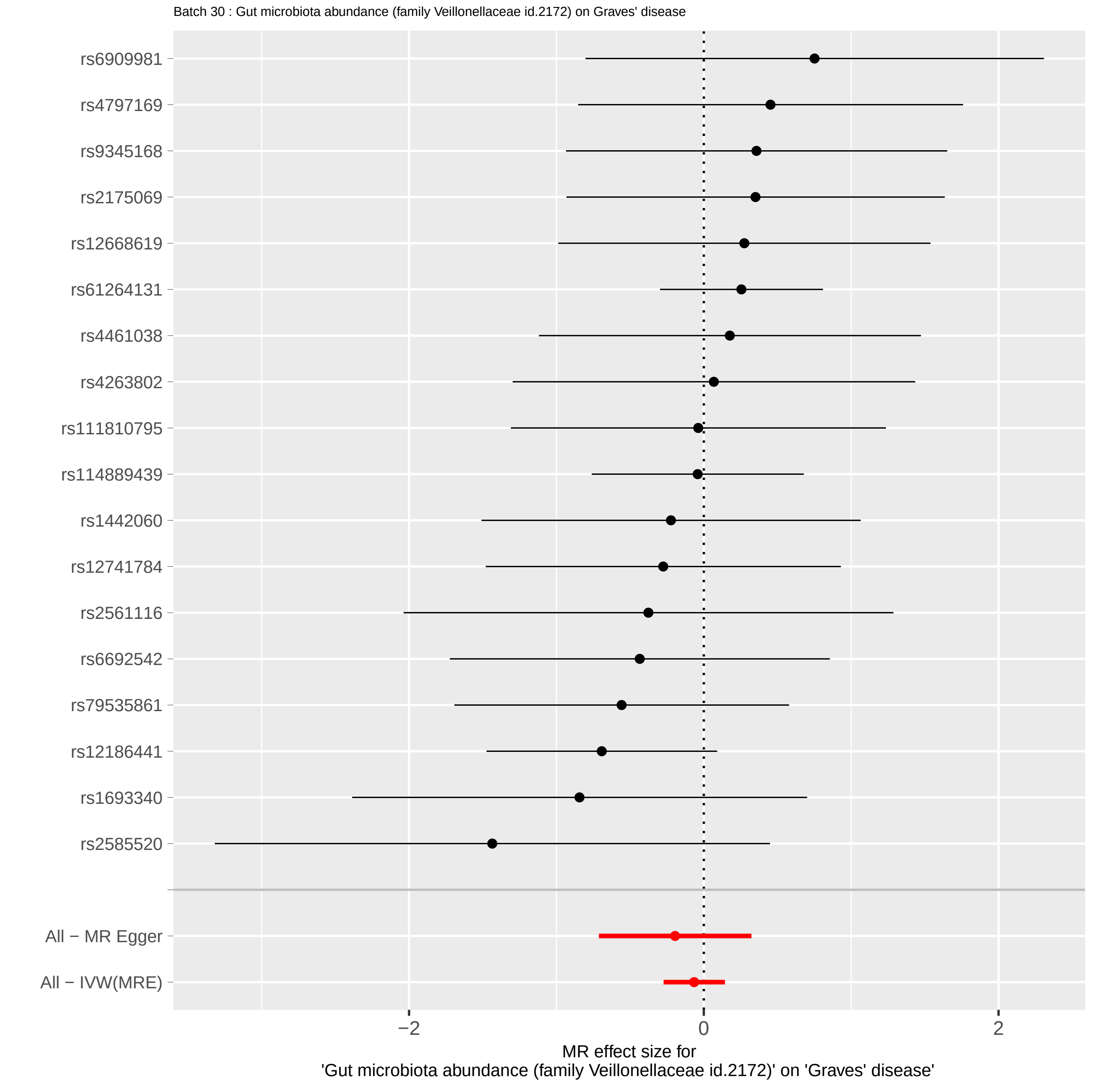

Batch 30 : Gut microbiota abundance (family Veillonellaceae id.2172) on Graves' disease
rs6909981
rs4797169
rs9345168
rs2175069
rs12668619
rs61264131
rs4461038
rs4263802
rs111810795
rs114889439
rs1442060
rs12741784
rs2561116
rs6692542
rs79535861
rs12186441
rs1693340
rs2585520
All − MR Egger
All − IVW(MRE)
−2
0
2
MR effect size for
'Gut microbiota abundance (family Veillonellaceae id.2172)' on 'Graves' disease'

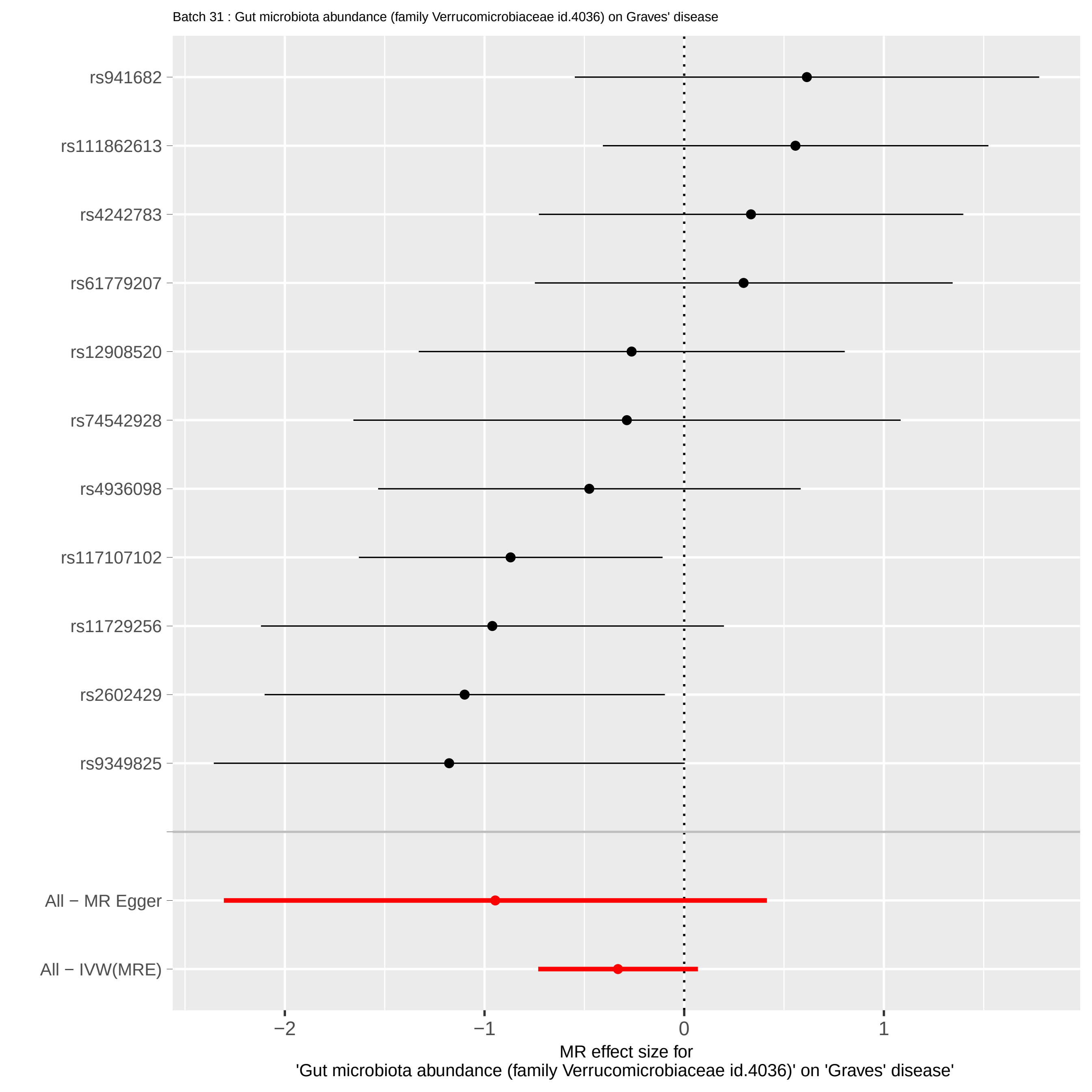
Batch 31 : Gut microbiota abundance (family Verrucomicrobiaceae id.4036) on Graves' disease
rs941682
rs111862613
rs4242783
rs61779207
rs12908520
rs74542928
rs4936098
rs117107102
rs11729256
rs2602429
rs9349825
All − MR Egger
All − IVW(MRE)
−2
−1
0
1
MR effect size for
'Gut microbiota abundance (family Verrucomicrobiaceae id.4036)' on 'Graves' disease'

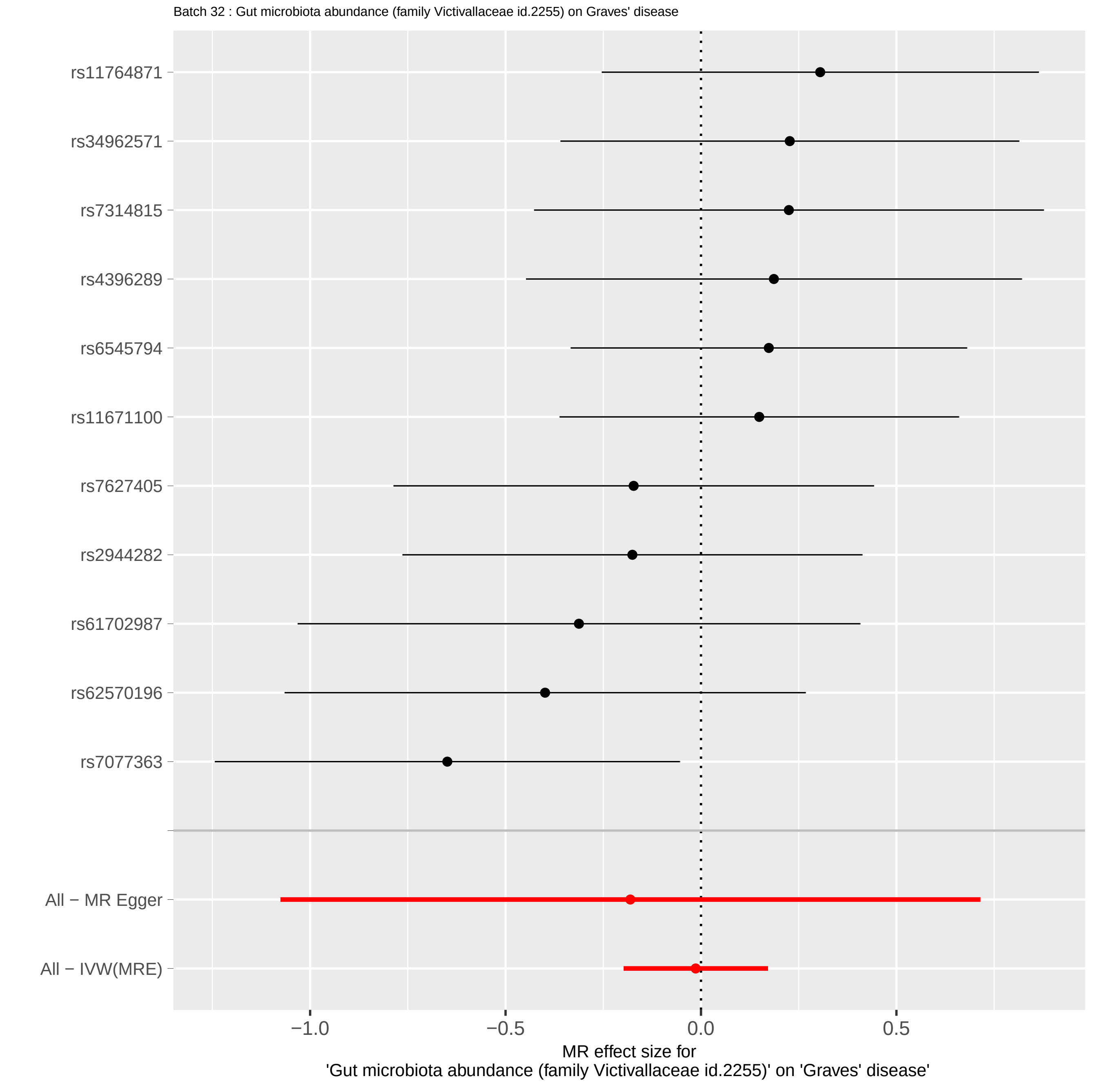

Batch 32 : Gut microbiota abundance (family Victivallaceae id.2255) on Graves' disease
rs11764871
rs34962571
rs7314815
rs4396289
rs6545794
rs11671100
rs7627405
rs2944282
rs61702987
rs62570196
rs7077363
All − MR Egger
All − IVW(MRE)
−1.0
−0.5
0.0
0.5
MR effect size for
'Gut microbiota abundance (family Victivallaceae id.2255)' on 'Graves' disease'

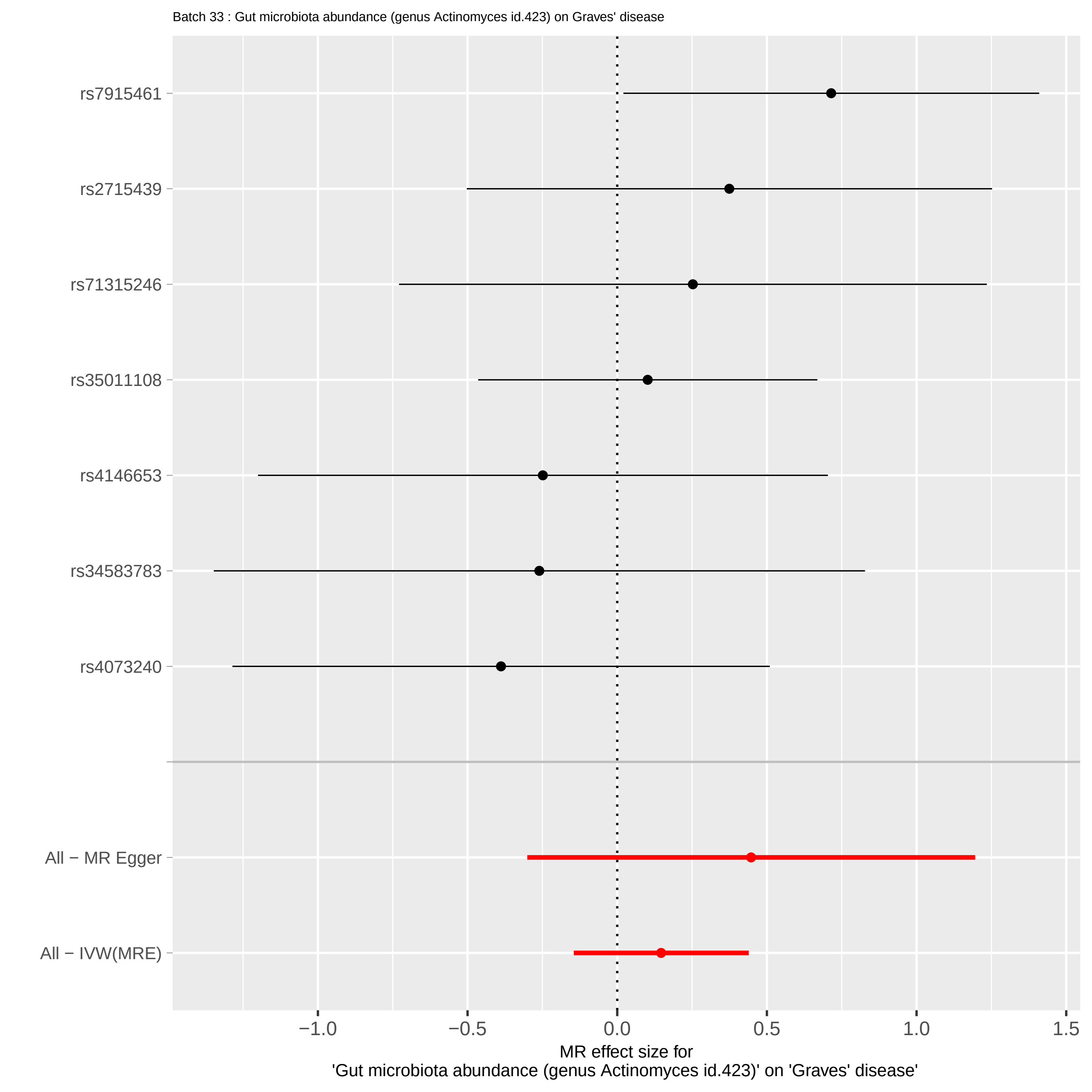

Batch 33 : Gut microbiota abundance (genus Actinomyces id.423) on Graves' disease
rs7915461
rs2715439
rs71315246
rs35011108
rs4146653
rs34583783
rs4073240
All − MR Egger
All − IVW(MRE)
−1.0
−0.5
0.0
0.5
1.0
1.5
MR effect size for
'Gut microbiota abundance (genus Actinomyces id.423)' on 'Graves' disease'

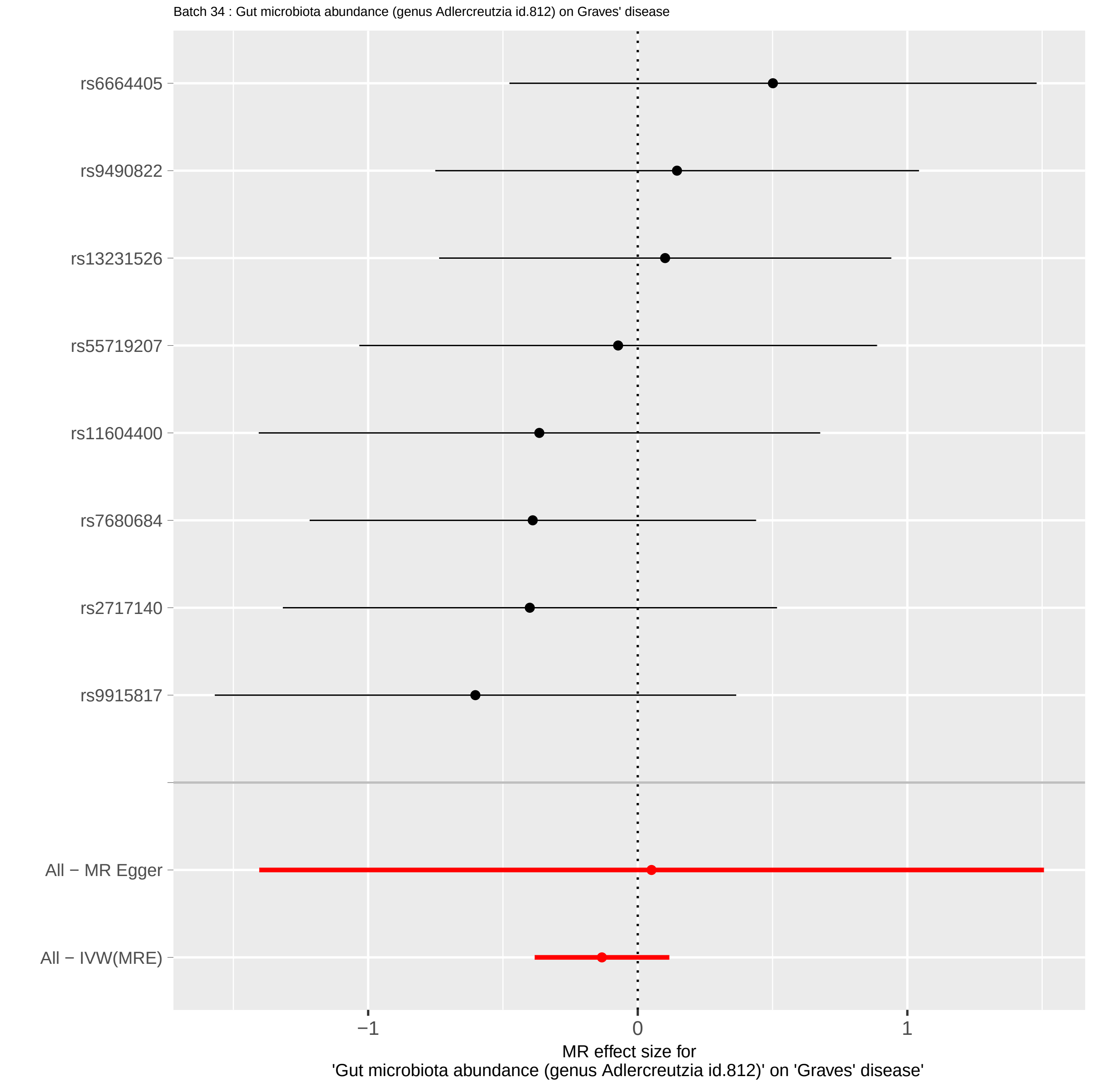
Batch 34 : Gut microbiota abundance (genus Adlercreutzia id.812) on Graves' disease
rs6664405
rs9490822
rs13231526
rs55719207
rs11604400
rs7680684
rs2717140
rs9915817
All − MR Egger
All − IVW(MRE)
−1
0
1
MR effect size for
'Gut microbiota abundance (genus Adlercreutzia id.812)' on 'Graves' disease'

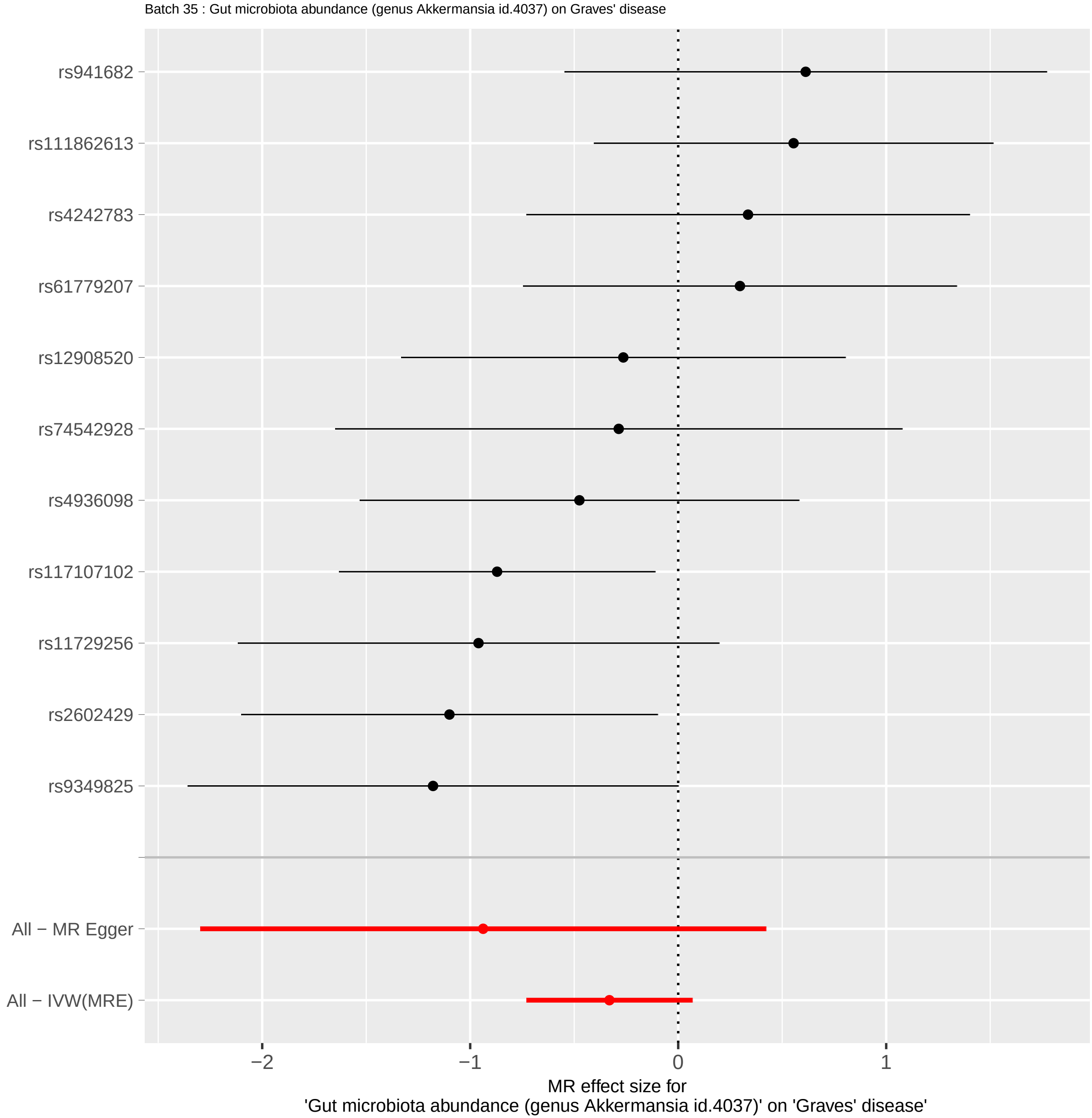
Batch 35 : Gut microbiota abundance (genus Akkermansia id.4037) on Graves' disease
rs941682
rs111862613
rs4242783
rs61779207
rs12908520
rs74542928
rs4936098
rs117107102
rs11729256
rs2602429
rs9349825
All − MR Egger
All − IVW(MRE)
−2
−1
0
1
MR effect size for
'Gut microbiota abundance (genus Akkermansia id.4037)' on 'Graves' disease'

Batch 36 : Gut microbiota abundance (genus Alistipes id.968) on Graves' disease

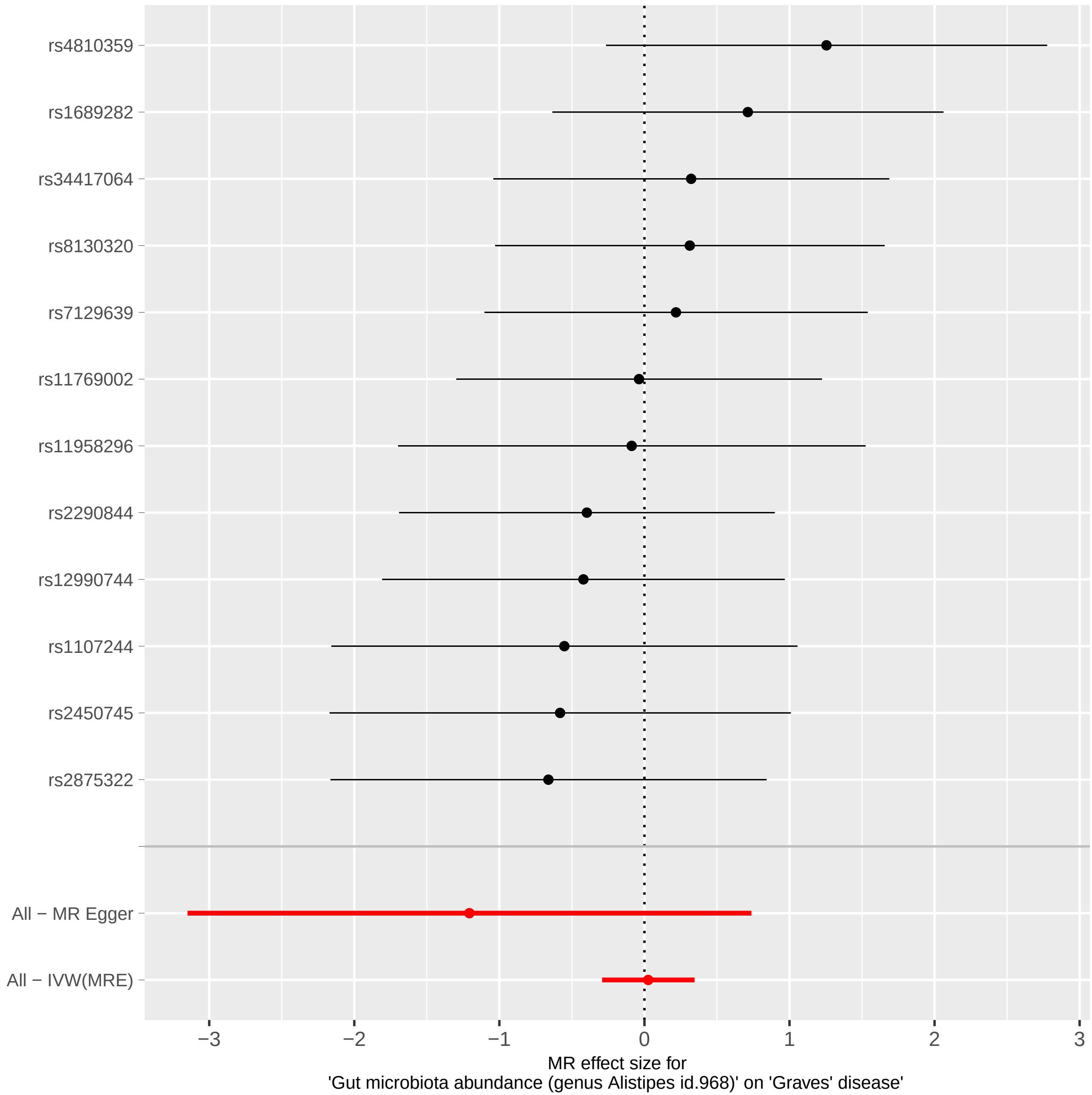

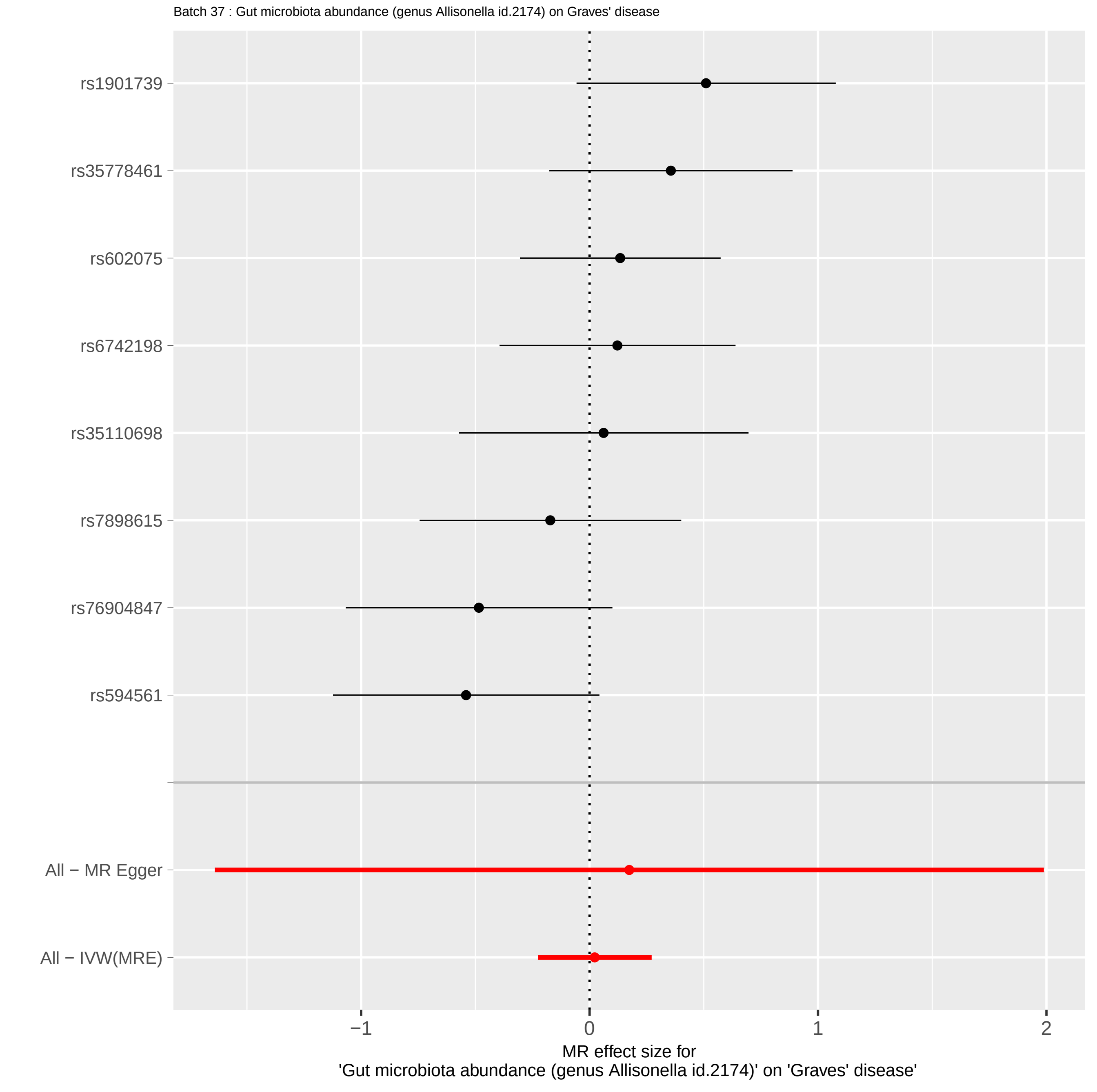
Batch 37 : Gut microbiota abundance (genus Allisonella id.2174) on Graves' disease
rs1901739
rs35778461
rs602075
rs6742198
rs35110698
rs7898615
rs76904847
rs594561
All − MR Egger
All − IVW(MRE)
−1
0
1
2
MR effect size for
'Gut microbiota abundance (genus Allisonella id.2174)' on 'Graves' disease'

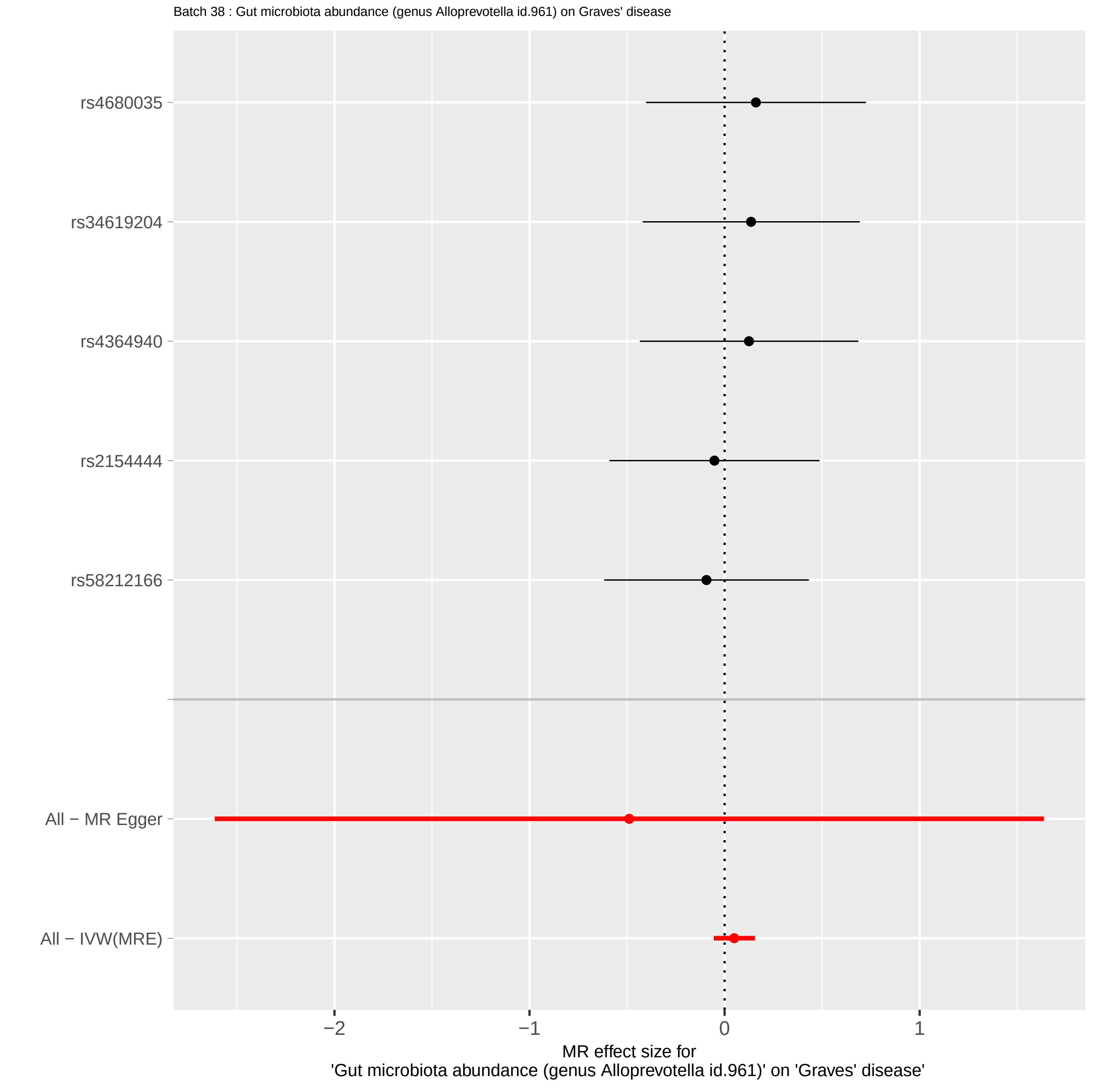

Batch 38 : Gut microbiota abundance (genus Alloprevotella id.961) on Graves' disease
rs4680035
rs34619204
rs4364940
rs2154444
rs58212166
All − MR Egger
All − IVW(MRE)
−2
−1
0
1
MR effect size for
'Gut microbiota abundance (genus Alloprevotella id.961)' on 'Graves' disease'

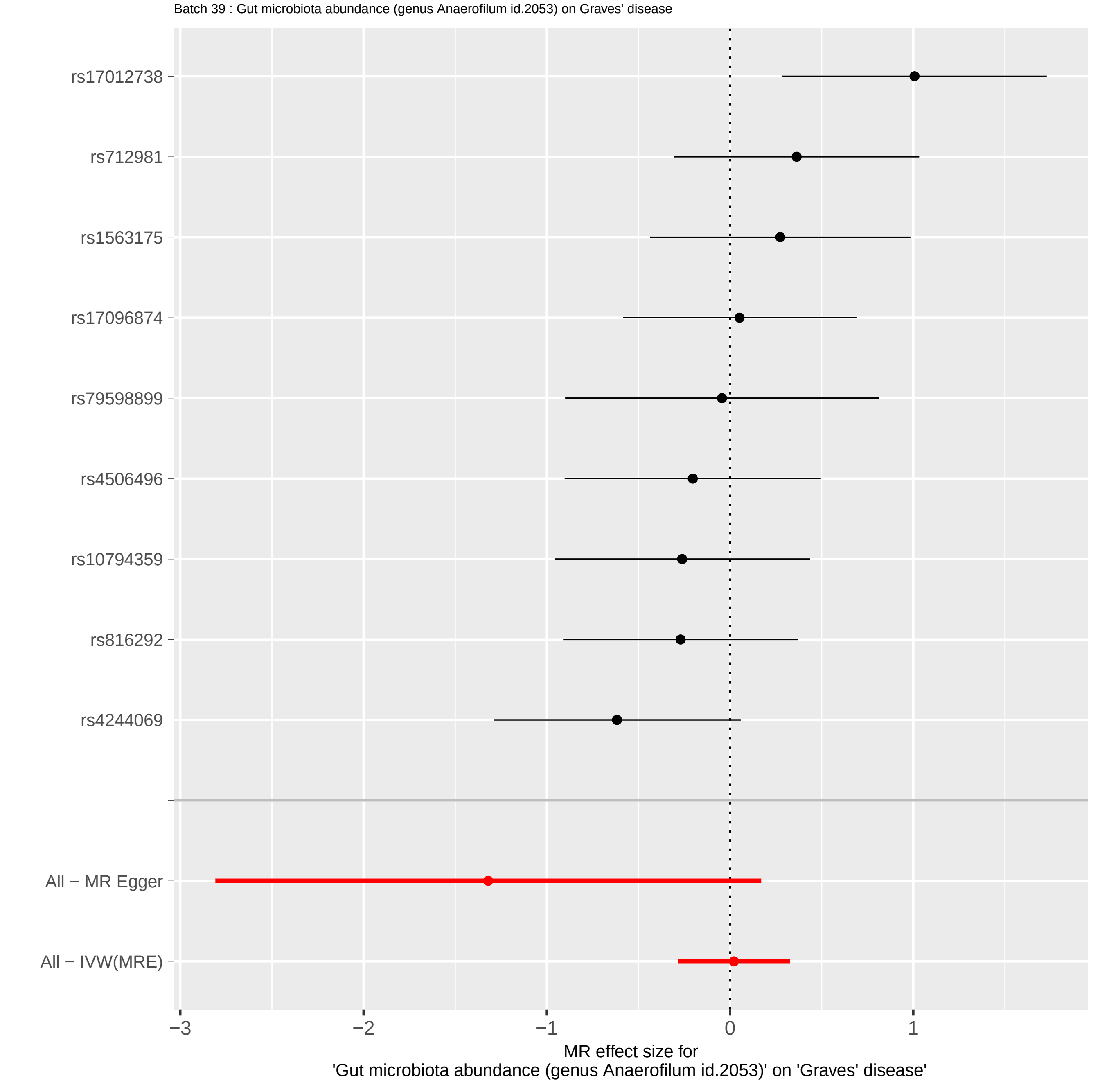
Batch 39 : Gut microbiota abundance (genus Anaerofilum id.2053) on Graves' disease
rs17012738
rs712981
rs1563175
rs17096874
rs79598899
rs4506496
rs10794359
rs816292
rs4244069
All − MR Egger
All − IVW(MRE)
−3
−2
−1
0
1
MR effect size for
'Gut microbiota abundance (genus Anaerofilum id.2053)' on 'Graves' disease

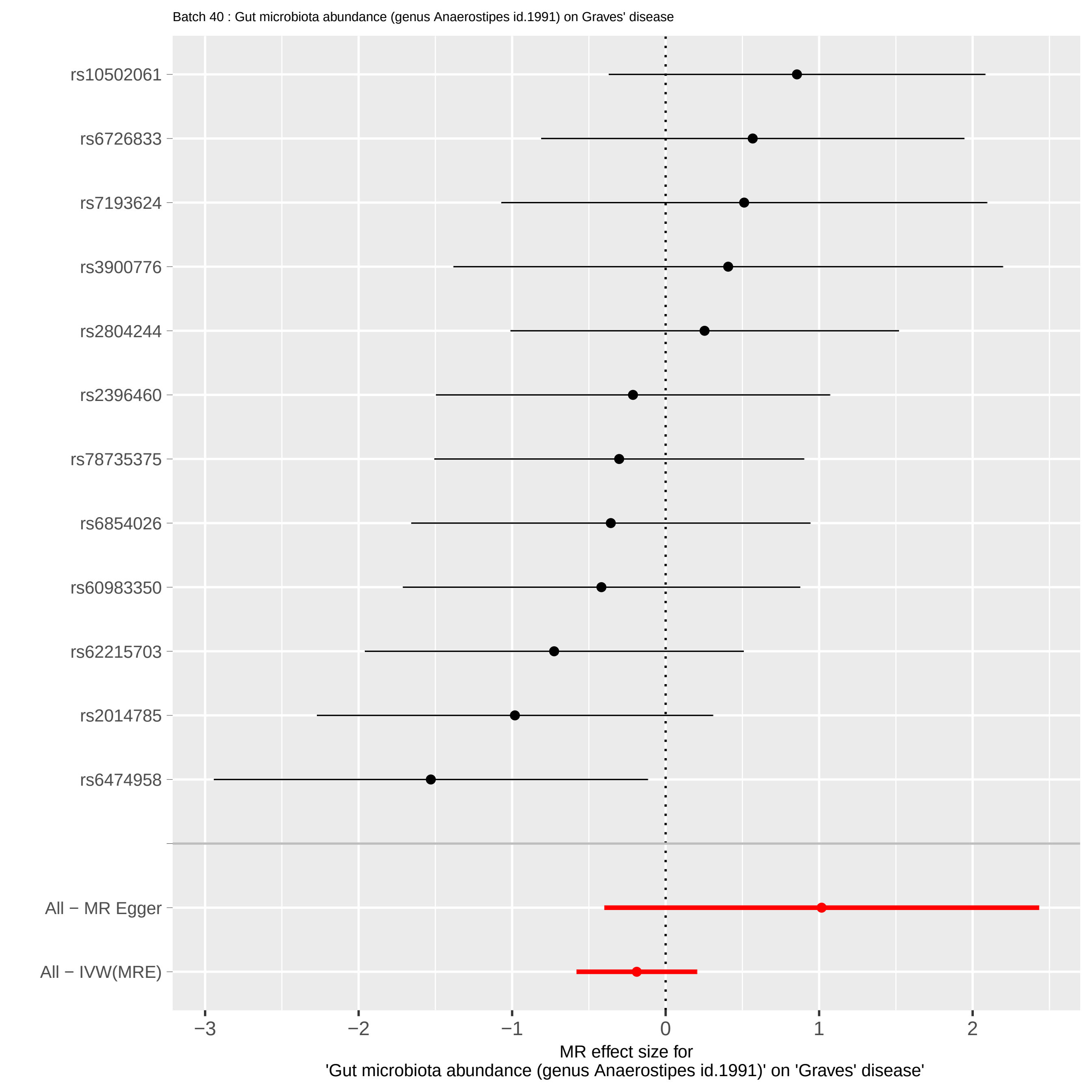

Batch 40 : Gut microbiota abundance (genus Anaerostipes id.1991) on Graves' disease
rs10502061
rs6726833
rs7193624
rs3900776
rs2804244
rs2396460
rs78735375
rs6854026
rs60983350
rs62215703
rs2014785
rs6474958
All − MR Egger
All − IVW(MRE)
−3
−2
−1
0
1
2
MR effect size for
'Gut microbiota abundance (genus Anaerostipes id.1991)' on 'Graves' disease'

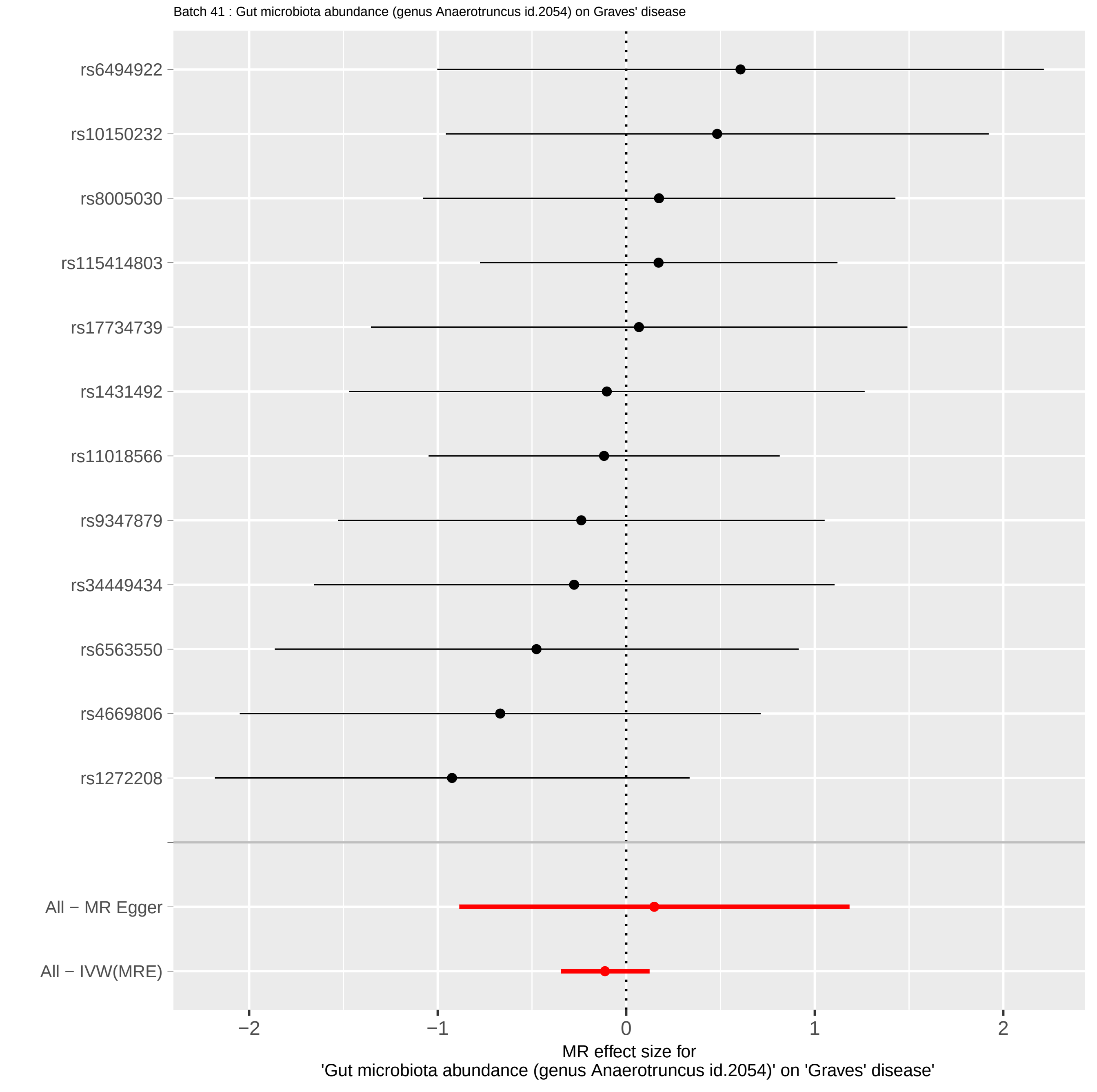
Batch 41 : Gut microbiota abundance (genus Anaerotruncus id.2054) on Graves' disease
rs6494922
rs10150232
rs8005030
rs115414803
rs17734739
rs1431492
rs11018566
rs9347879
rs34449434
rs6563550
rs4669806
rs1272208
All − MR Egger
All − IVW(MRE)
−2
−1
0
1
2
MR effect size for
'Gut microbiota abundance (genus Anaerotruncus id.2054)' on 'Graves' disease'

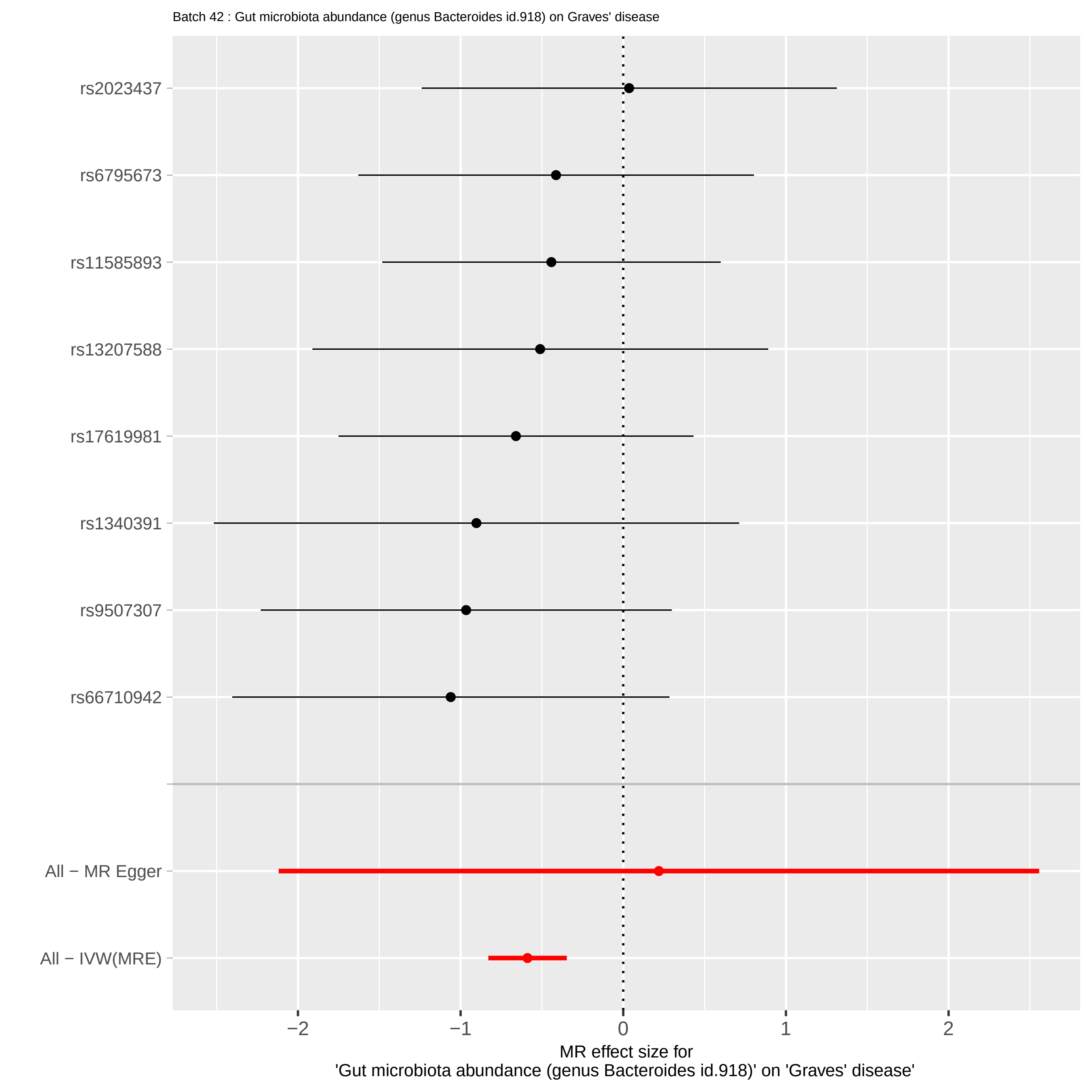
Batch 42 : Gut microbiota abundance (genus Bacteroides id.918) on Graves' disease
rs2023437
rs6795673
rs11585893
rs13207588
rs17619981
rs1340391
rs9507307
rs66710942
All − MR Egger
All − IVW(MRE)
−2
−1
0
1
2
MR effect size for
'Gut microbiota abundance (genus Bacteroides id.918)' on 'Graves' disease'

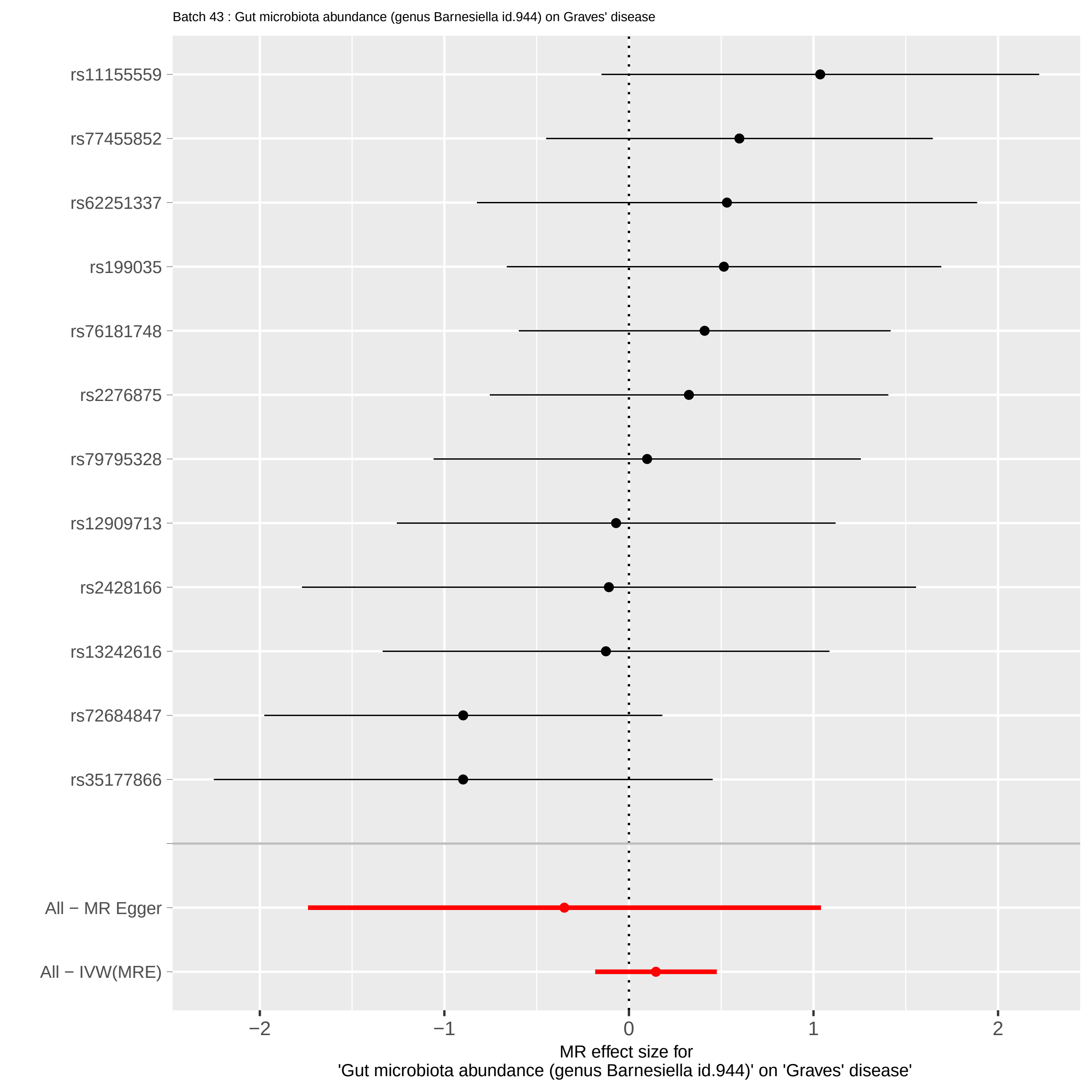

Batch 43 : Gut microbiota abundance (genus Barnesiella id.944) on Graves' disease
rs11155559
rs77455852
rs62251337
rs199035
rs76181748
rs2276875
rs79795328
rs12909713
rs2428166
rs13242616
rs72684847
rs35177866
All − MR Egger
All − IVW(MRE)
−2
−1
0
1
2
MR effect size for
'Gut microbiota abundance (genus Barnesiella id.944)' on 'Graves' disease'

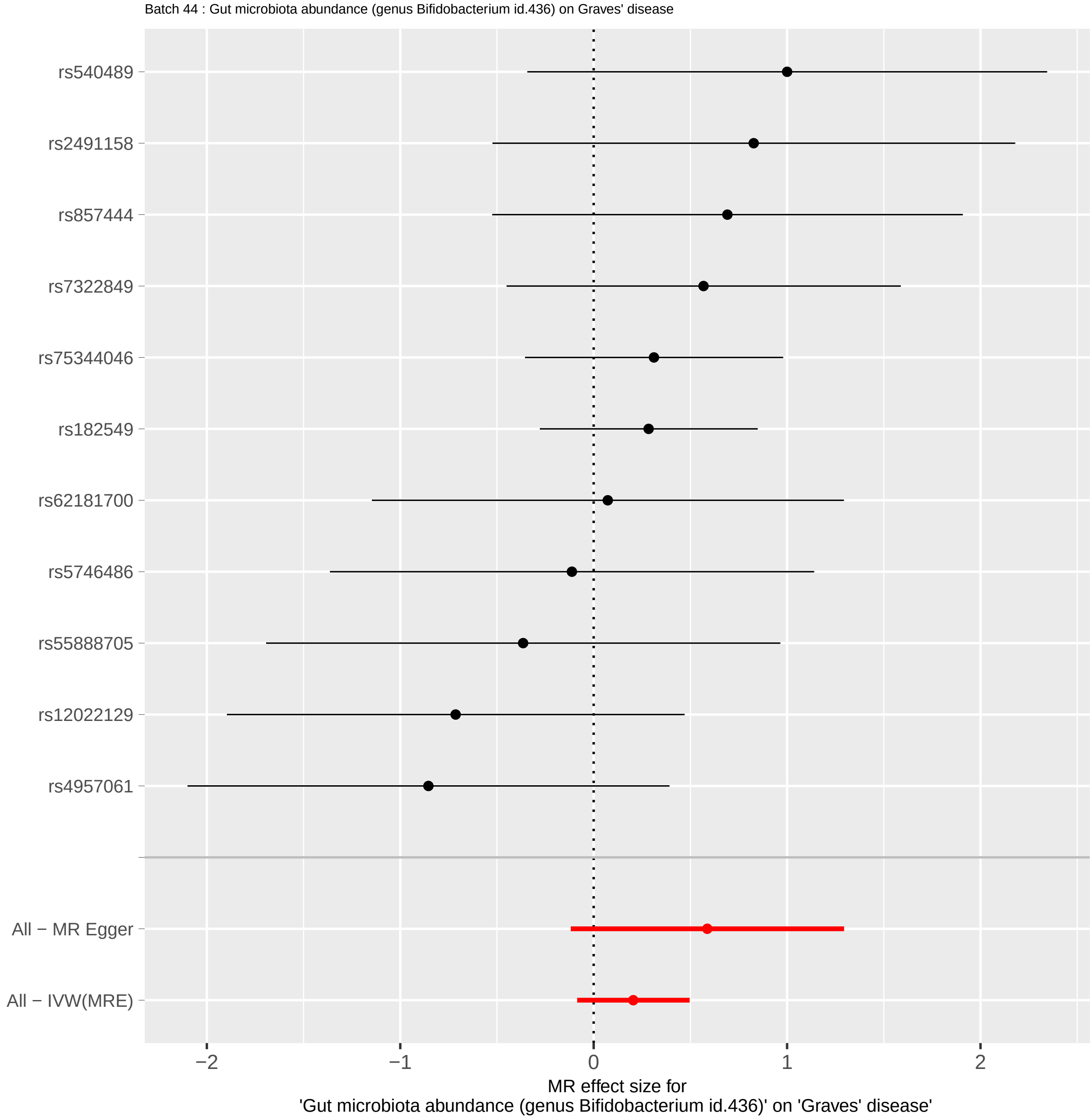

Batch 44 : Gut microbiota abundance (genus Bifidobacterium id.436) on Graves' disease
rs540489
rs2491158
rs857444
rs7322849
rs75344046
rs182549
rs62181700
rs5746486
rs55888705
rs12022129
rs4957061
All − MR Egger
All − IVW(MRE)
−2
−1
0
1
2
MR effect size for
'Gut microbiota abundance (genus Bifidobacterium id.436)' on 'Graves' disease'

Batch 45 : Gut microbiota abundance (genus Bilophila id.3170) on Graves' disease

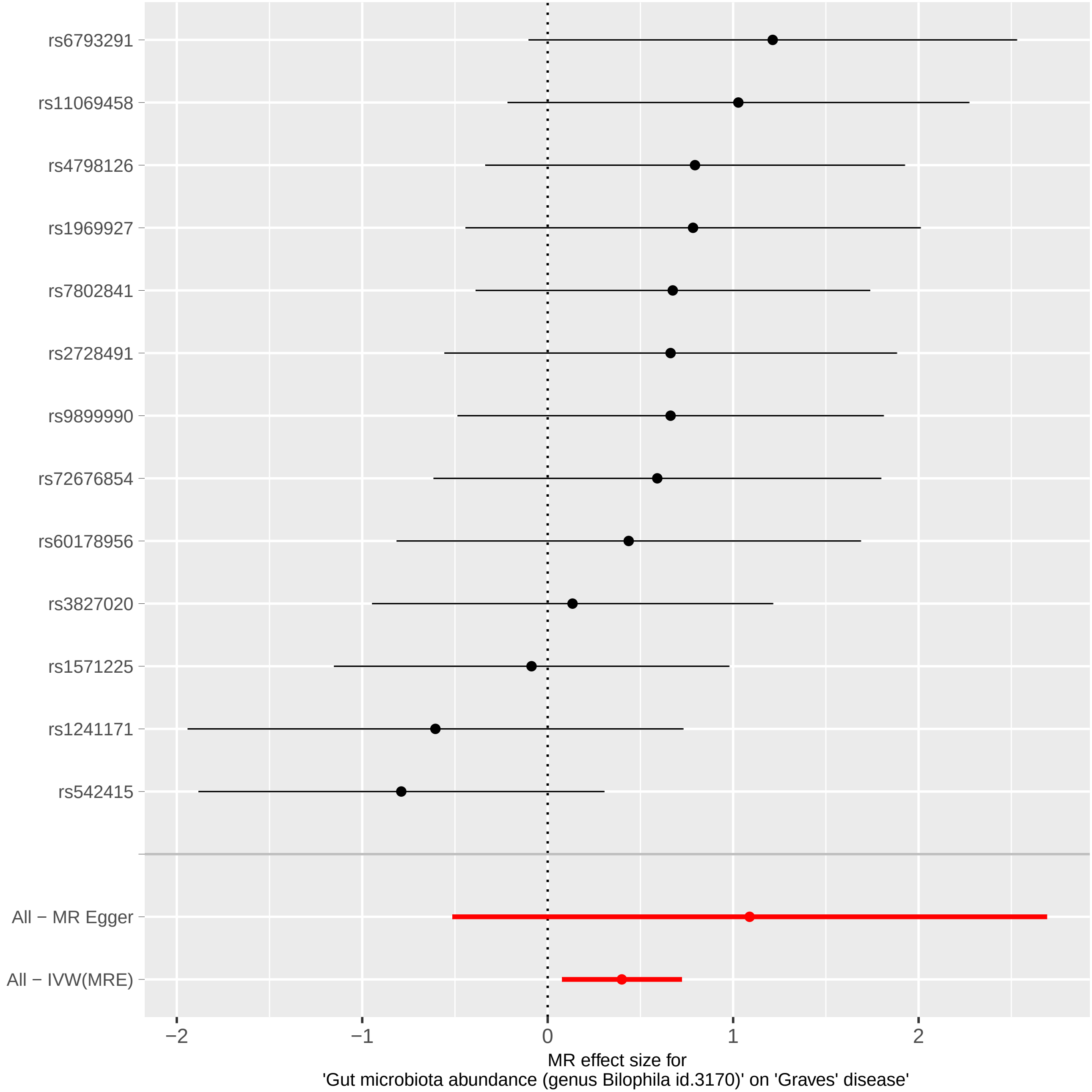

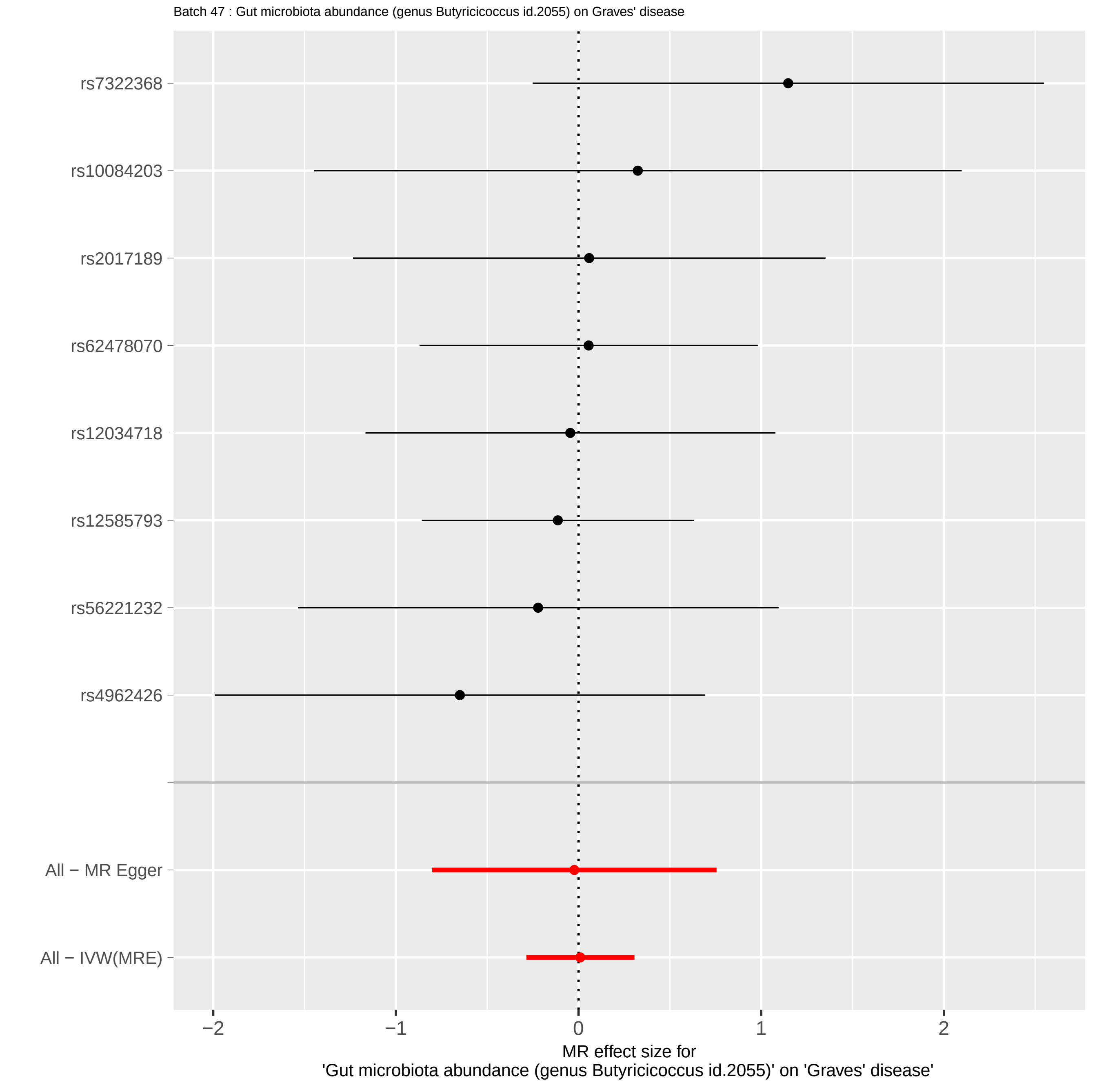

Batch 47 : Gut microbiota abundance (genus Butyricicoccus id.2055) on Graves' disease
rs7322368
rs10084203
rs2017189
rs62478070
rs12034718
rs12585793
rs56221232
rs4962426
All − MR Egger
All − IVW(MRE)
−2
−1
0
1
2
MR effect size for
'Gut microbiota abundance (genus Butyricicoccus id.2055)' on 'Graves' disease'

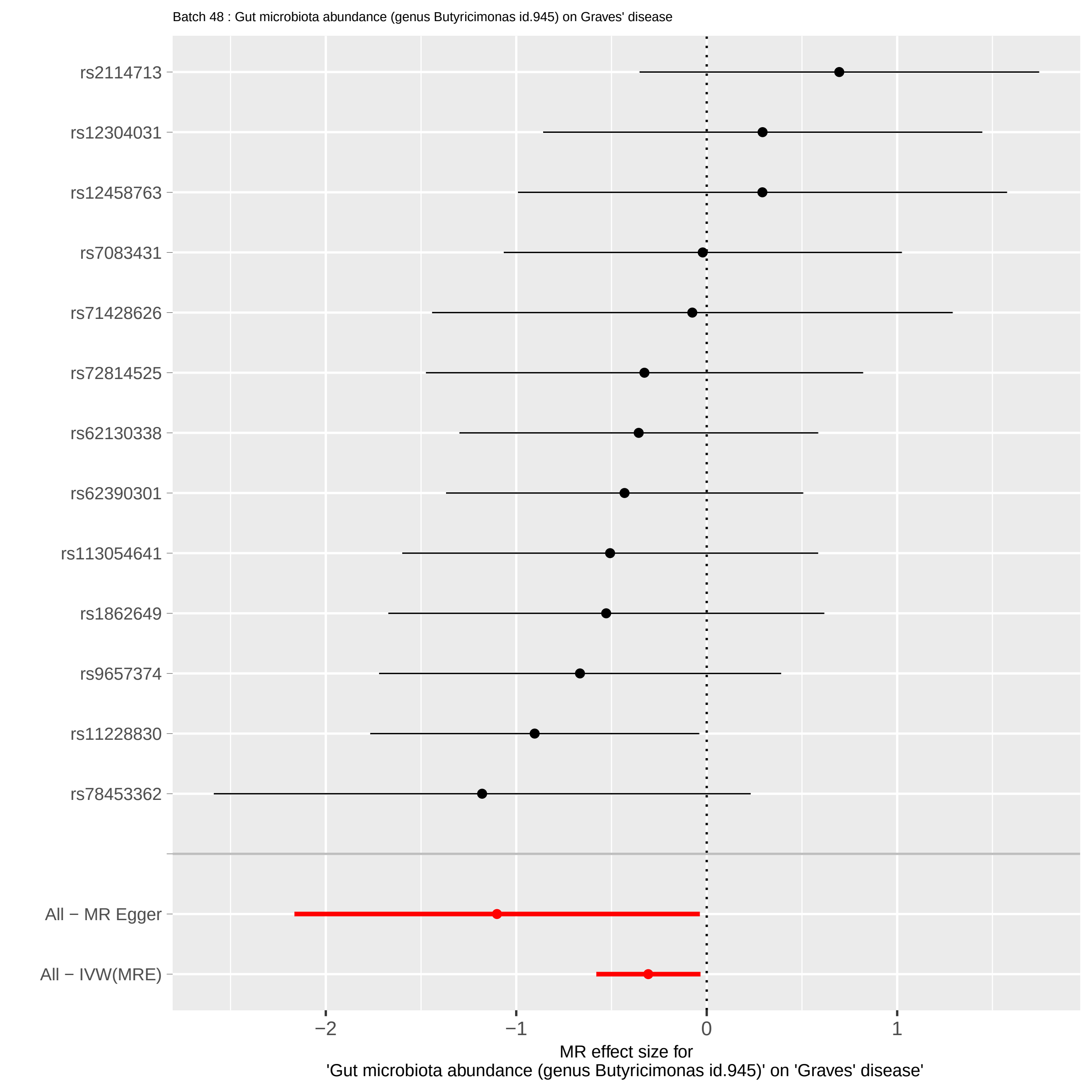

Batch 48 : Gut microbiota abundance (genus Butyricimonas id.945) on Graves' disease
rs2114713
rs12304031
rs12458763
rs7083431
rs71428626
rs72814525
rs62130338
rs62390301
rs113054641
rs1862649
rs9657374
rs11228830
rs78453362
All − MR Egger
All − IVW(MRE)
−2
−1
0
1
MR effect size for
'Gut microbiota abundance (genus Butyricimonas id.945)' on 'Graves' disease'

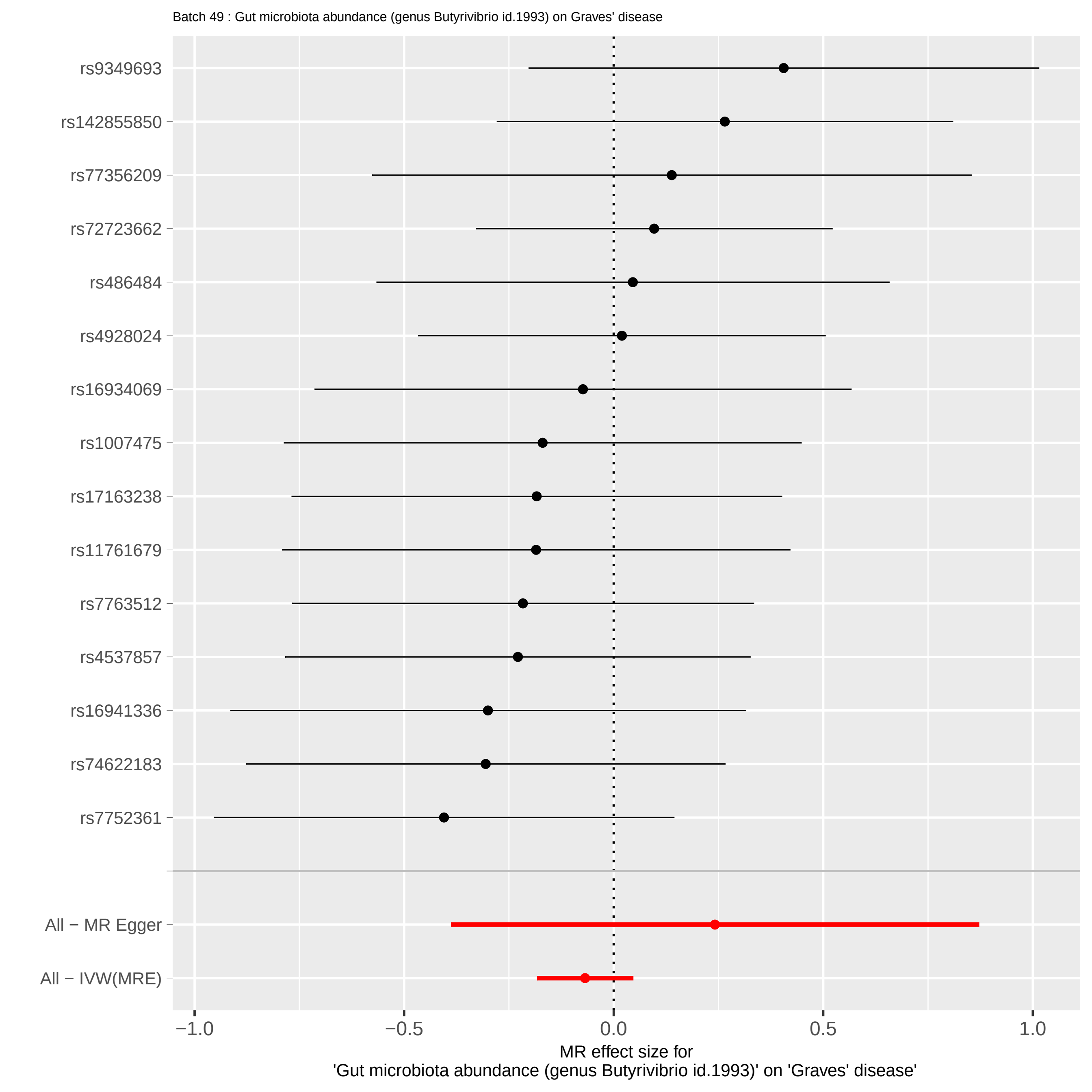
Batch 49 : Gut microbiota abundance (genus Butyrivibrio id.1993) on Graves' disease
rs9349693
rs142855850
rs77356209
rs72723662
rs486484
rs4928024
rs16934069
rs1007475
rs17163238
rs11761679
rs7763512
rs4537857
rs16941336
rs74622183
rs7752361
All − MR Egger
All − IVW(MRE)
−1.0
−0.5
0.0
0.5
1.0
MR effect size for
'Gut microbiota abundance (genus Butyrivibrio id.1993)' on 'Graves' disease'

Batch 50 : Gut microbiota abundance (genus Candidatus Soleaferrea id.11350) on Graves' disease

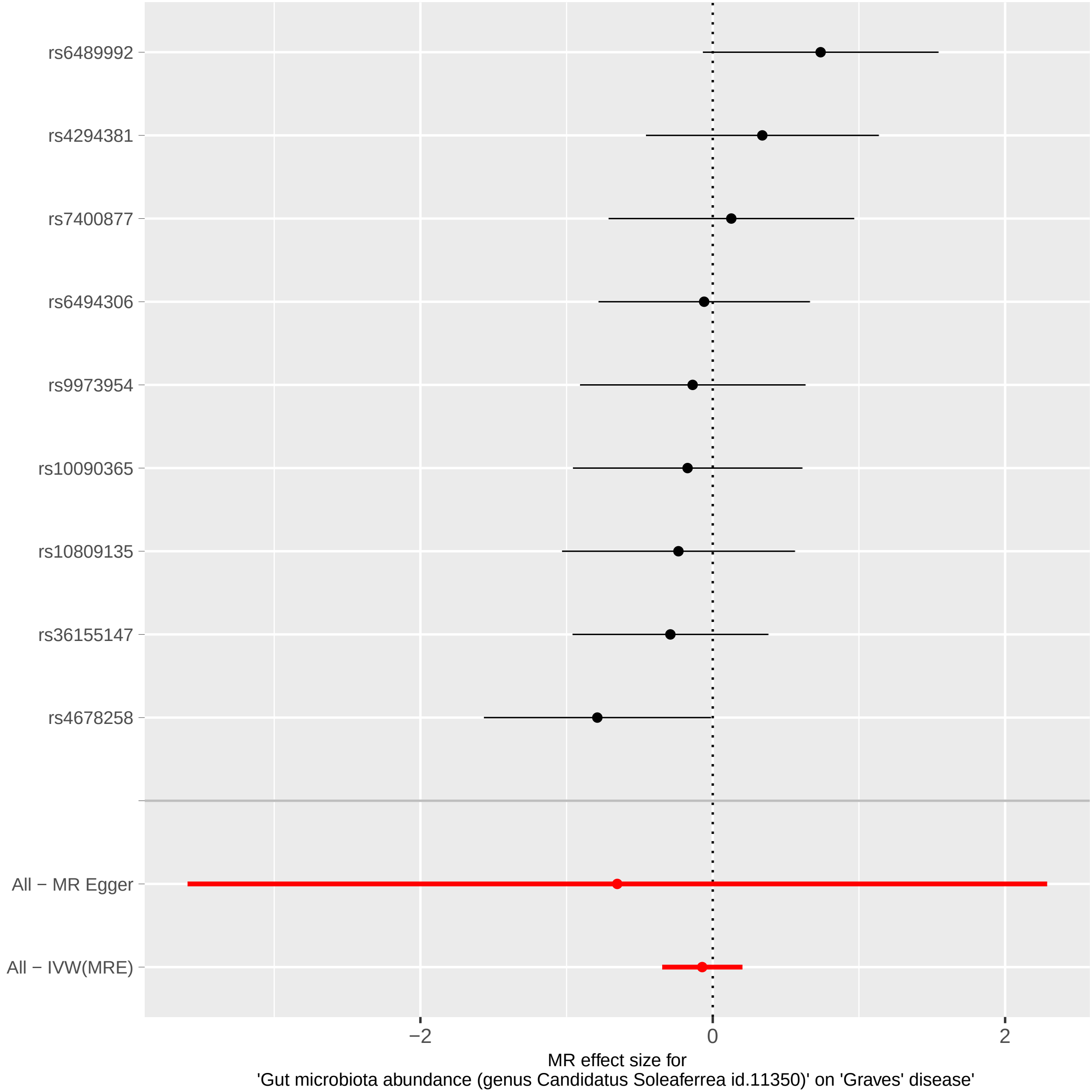

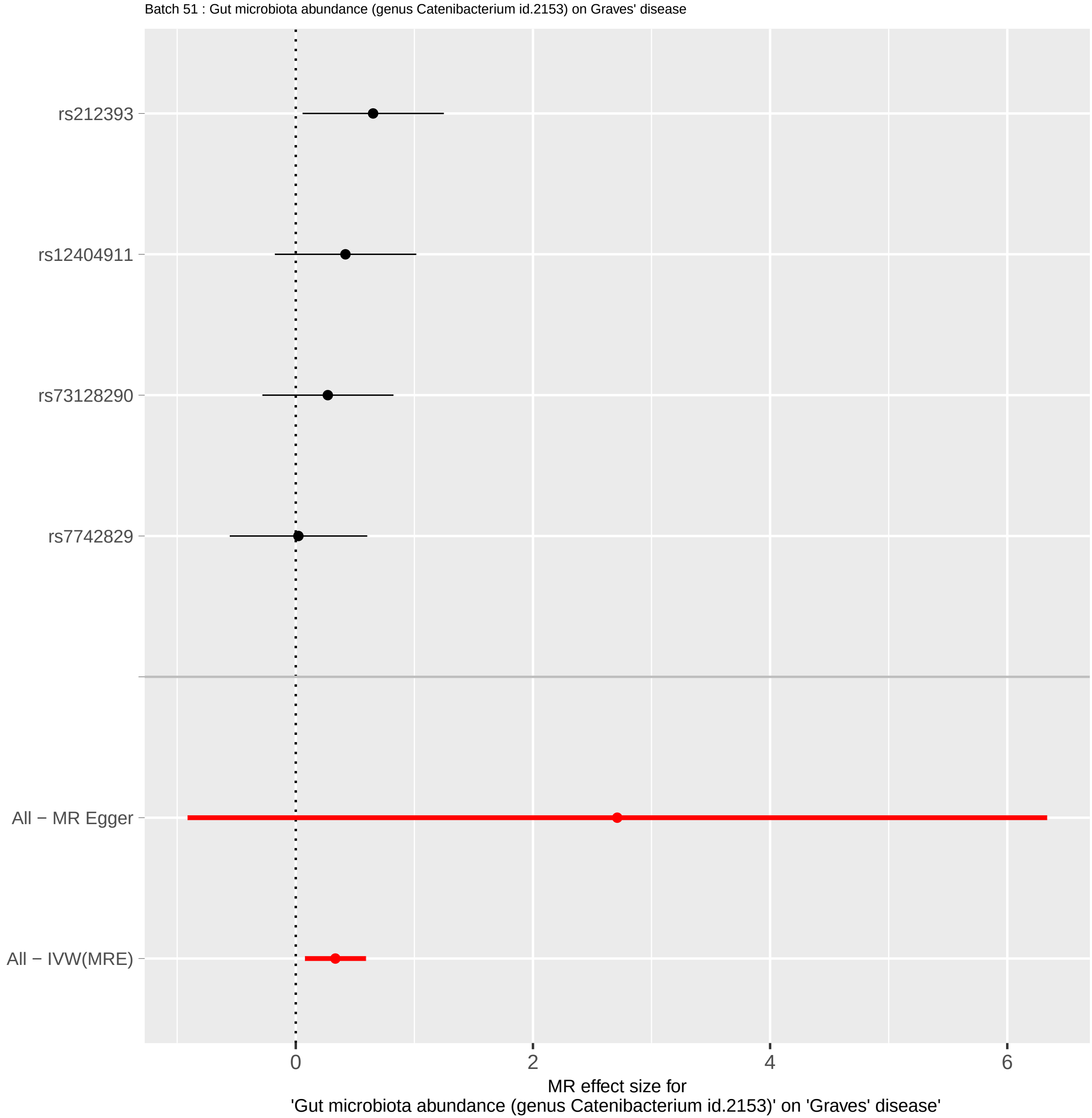

Batch 51 : Gut microbiota abundance (genus Catenibacterium id.2153) on Graves' disease
rs212393
rs12404911
rs73128290
rs7742829
All − MR Egger
All − IVW(MRE)
0
2
4
6
MR effect size for
'Gut microbiota abundance (genus Catenibacterium id.2153)' on 'Graves' disease'

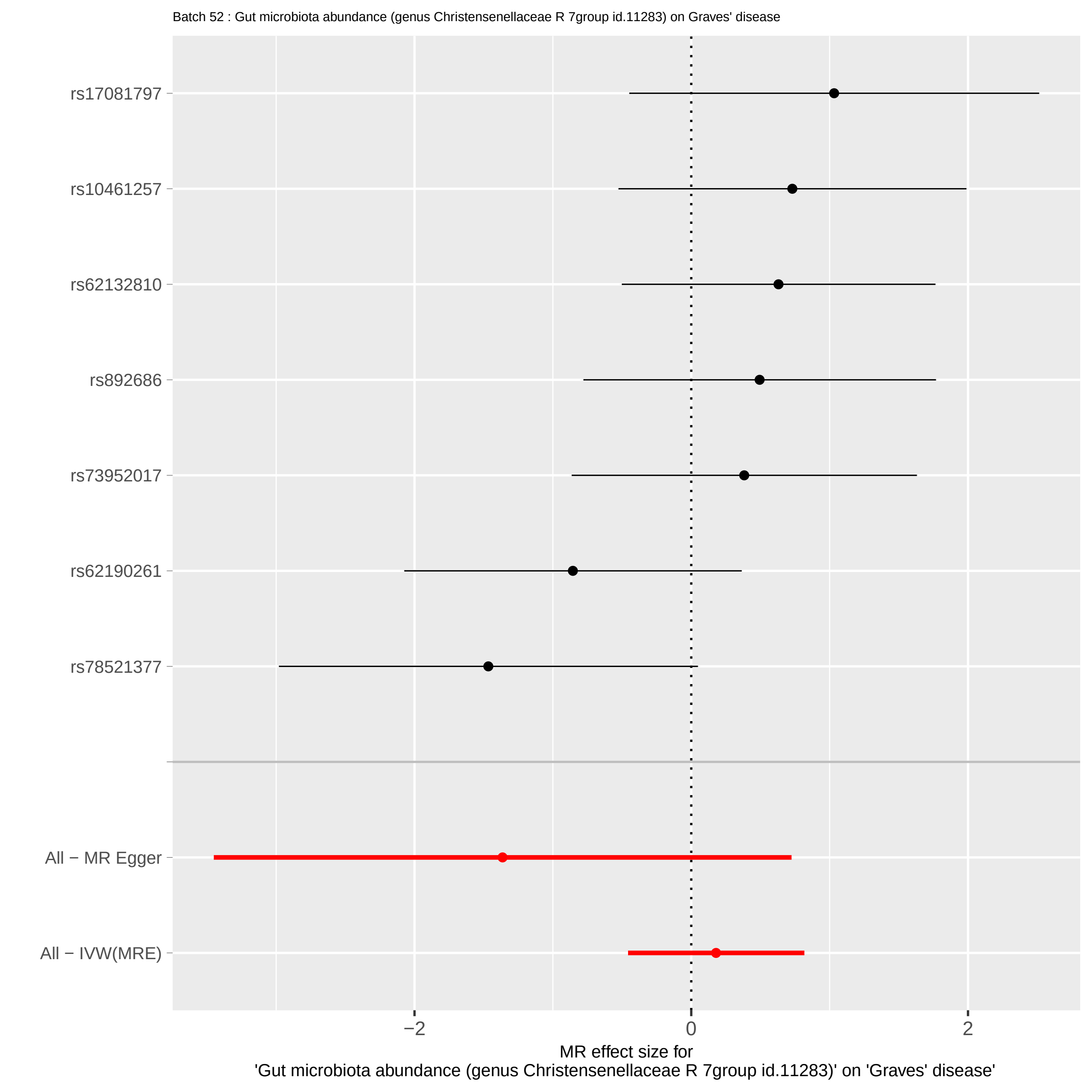

Batch 52 : Gut microbiota abundance (genus Christensenellaceae R 7group id.11283) on Graves' disease
rs17081797
rs10461257
rs62132810
rs892686
rs73952017
rs62190261
rs78521377
All − MR Egger
All − IVW(MRE)
−2
0
2
MR effect size for
'Gut microbiota abundance (genus Christensenellaceae R 7group id.11283)' on 'Graves' disease'

Batch 53 : Gut microbiota abundance (genus Clostridium innocuum group id.14397) on Graves' disease

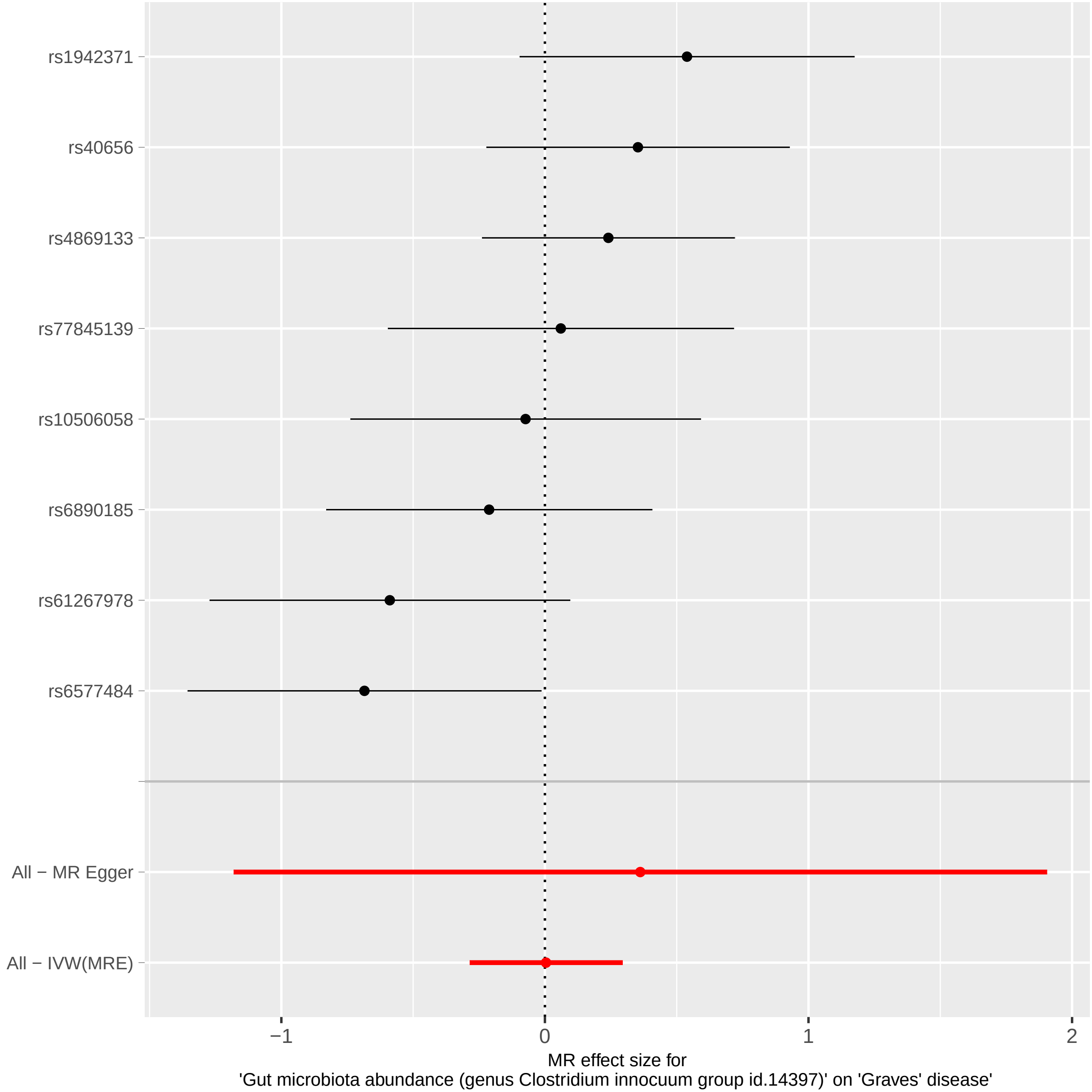

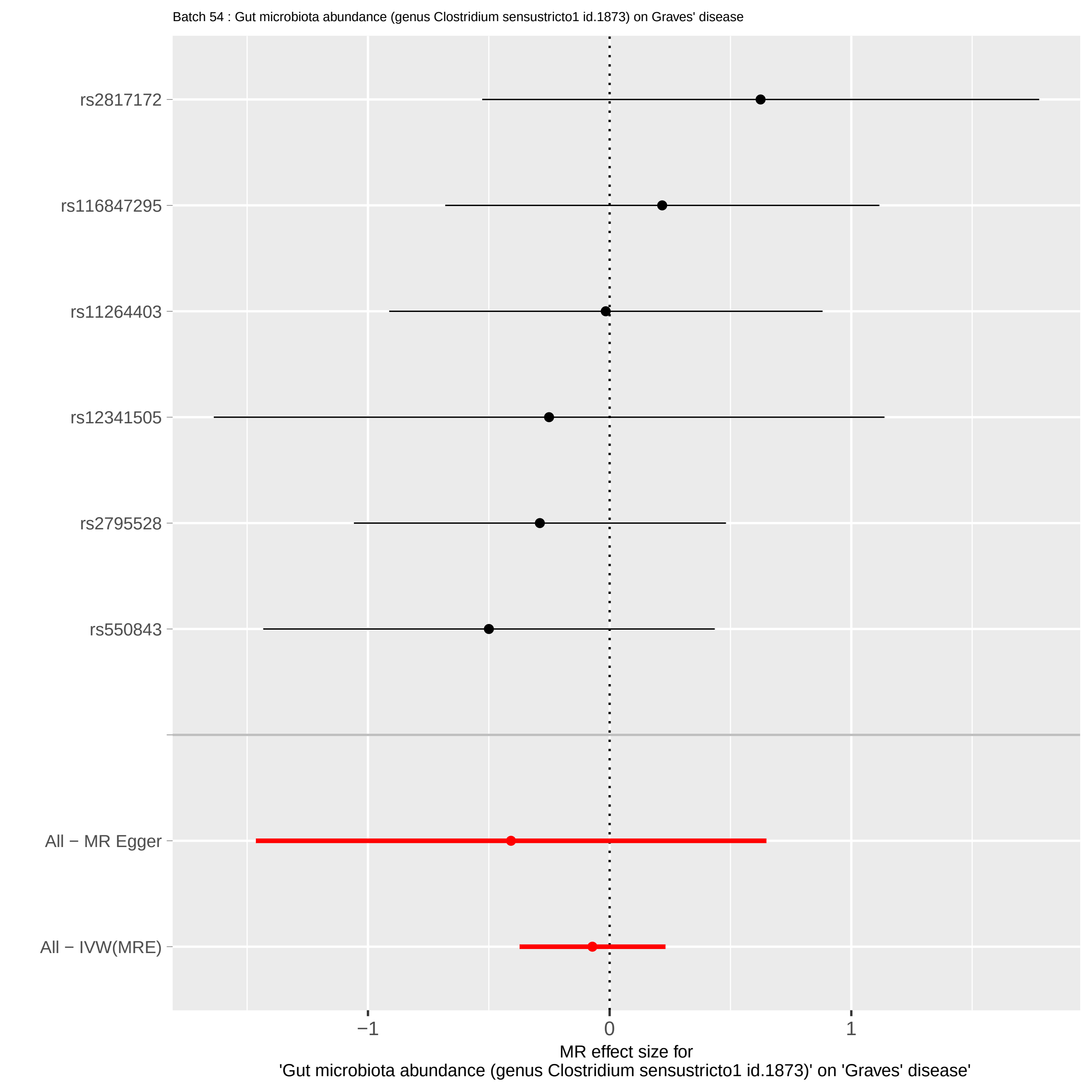

Batch 54 : Gut microbiota abundance (genus Clostridium sensustricto1 id.1873) on Graves' disease
rs2817172
rs116847295
rs11264403
rs12341505
rs2795528
rs550843
All − MR Egger
All − IVW(MRE)
−1
0
1
MR effect size for
'Gut microbiota abundance (genus Clostridium sensustricto1 id.1873)' on 'Graves' disease'

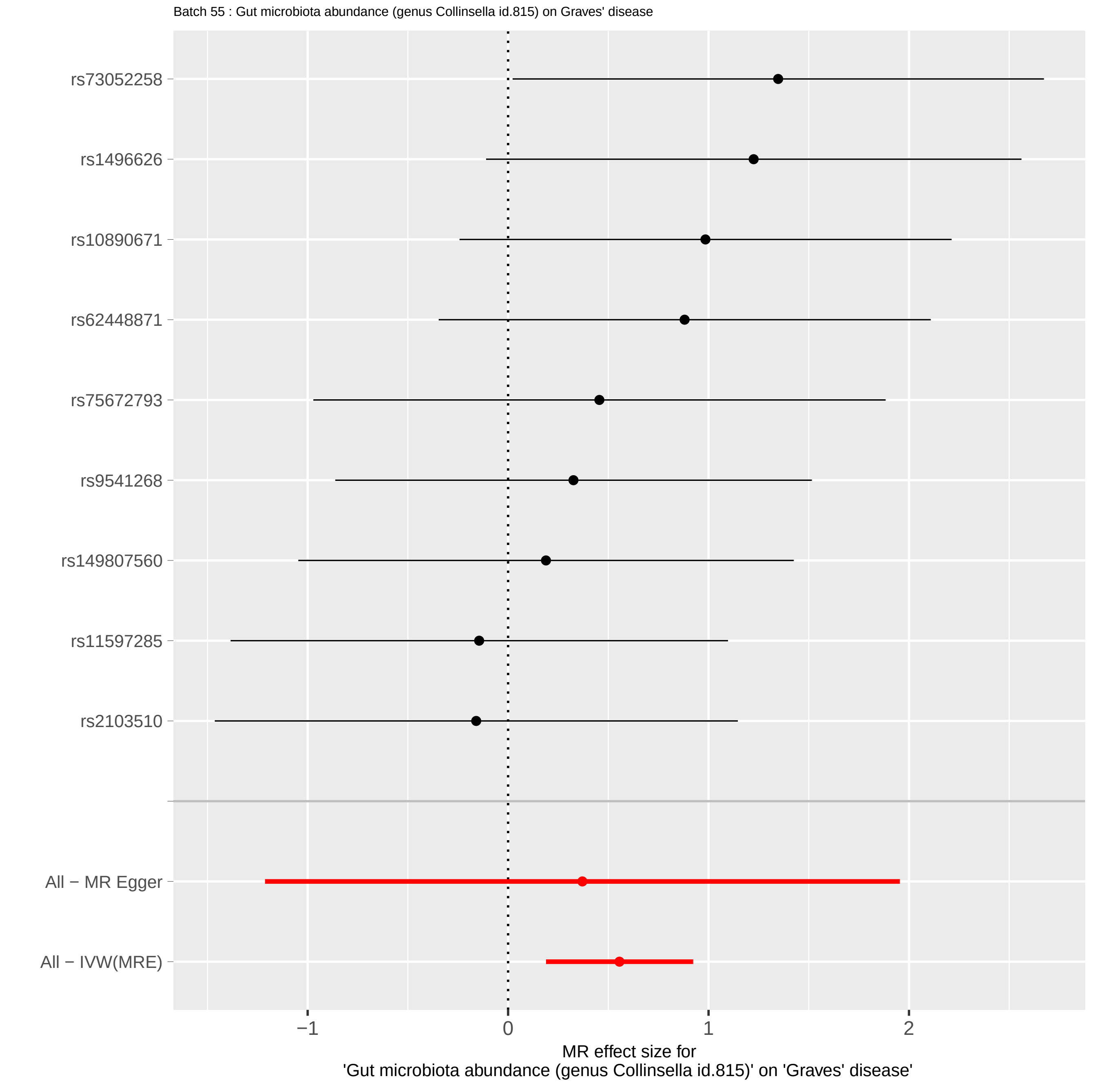

Batch 55 : Gut microbiota abundance (genus Collinsella id.815) on Graves' disease
rs73052258
rs1496626
rs10890671
rs62448871
rs75672793
rs9541268
rs149807560
rs11597285
rs2103510
All − MR Egger
All − IVW(MRE)
−1
0
1
2
MR effect size for
'Gut microbiota abundance (genus Collinsella id.815)' on 'Graves' disease'

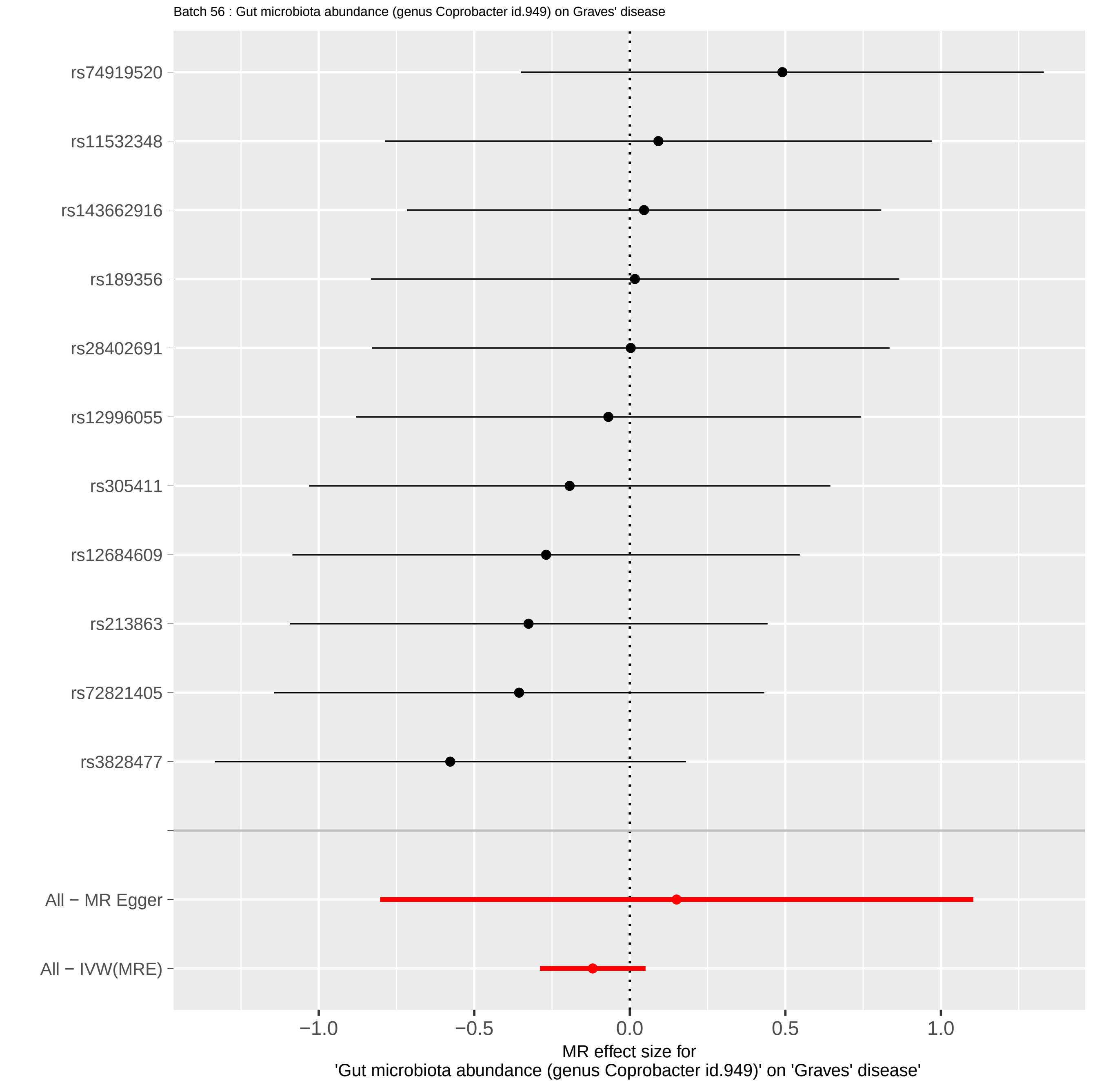
Batch 56 : Gut microbiota abundance (genus Coprobacter id.949) on Graves' disease
rs74919520
rs11532348
rs143662916
rs189356
rs28402691
rs12996055
rs305411
rs12684609
rs213863
rs72821405
rs3828477
All − MR Egger
All − IVW(MRE)
−1.0
−0.5
0.0
0.5
1.0
MR effect size for
'Gut microbiota abundance (genus Coprobacter id.949)' on 'Graves' disease'

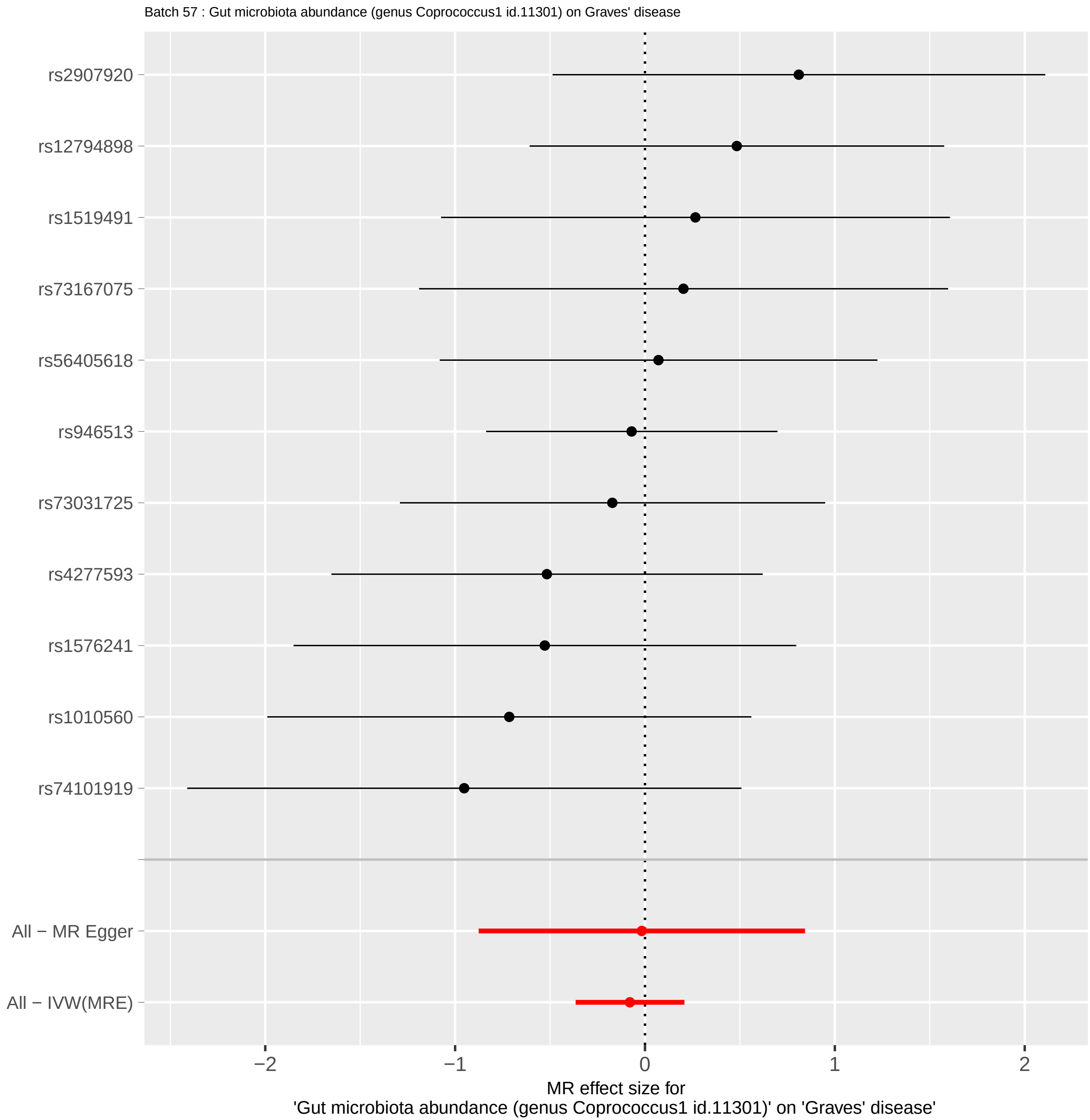
Batch 57 : Gut microbiota abundance (genus Coprococcus1 id.11301) on Graves' disease
rs2907920
rs12794898
rs1519491
rs73167075
rs56405618
rs946513
rs73031725
rs4277593
rs1576241
rs1010560
rs74101919
All − MR Egger
All − IVW(MRE)
−2
−1
0
1
2
MR effect size for
'Gut microbiota abundance (genus Coprococcus1 id.11301)' on 'Graves' disease'

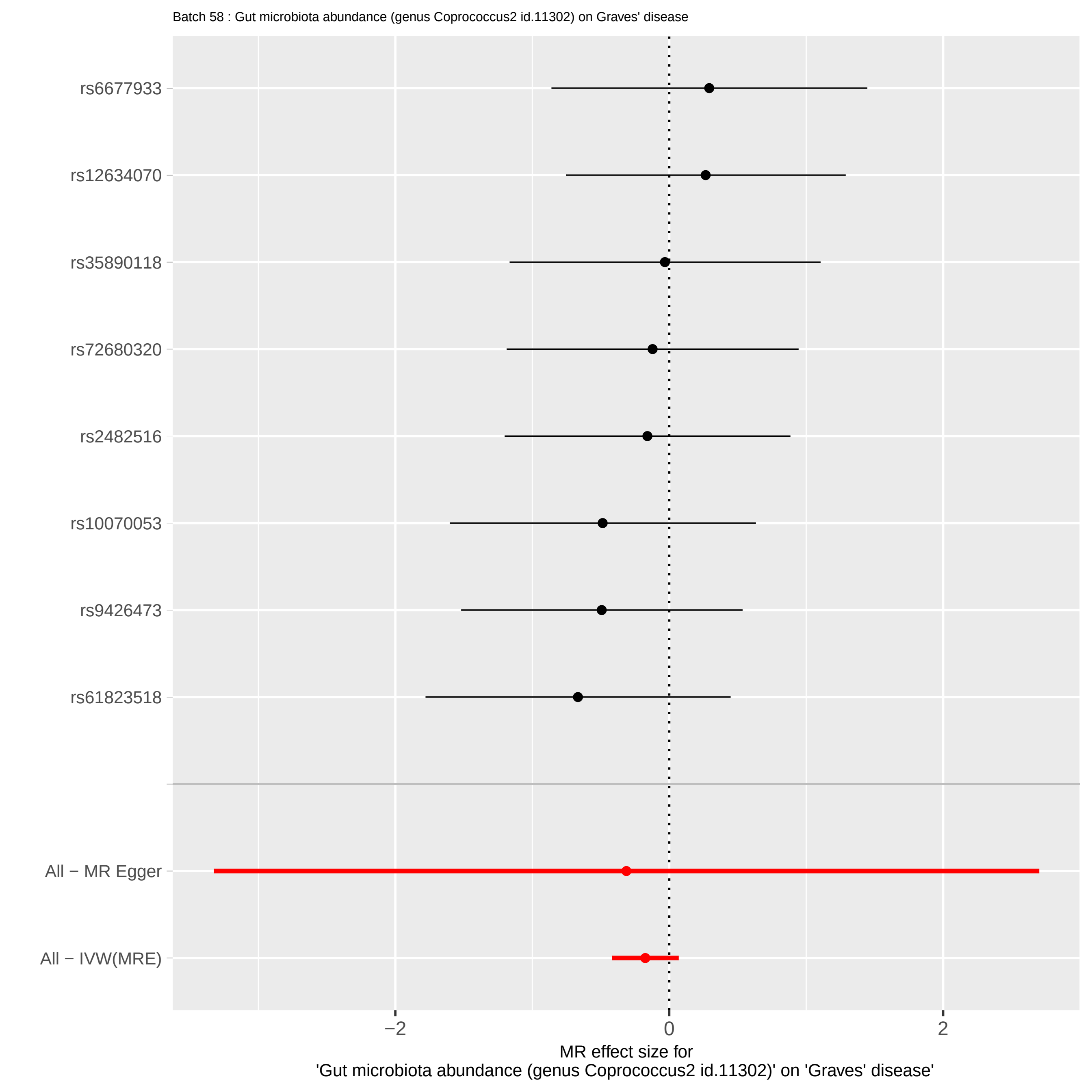

Batch 58 : Gut microbiota abundance (genus Coprococcus2 id.11302) on Graves' disease
rs6677933
rs12634070
rs35890118
rs72680320
rs2482516
rs10070053
rs9426473
rs61823518
All − MR Egger
All − IVW(MRE)
−2
0
2
MR effect size for
'Gut microbiota abundance (genus Coprococcus2 id.11302)' on 'Graves' disease'

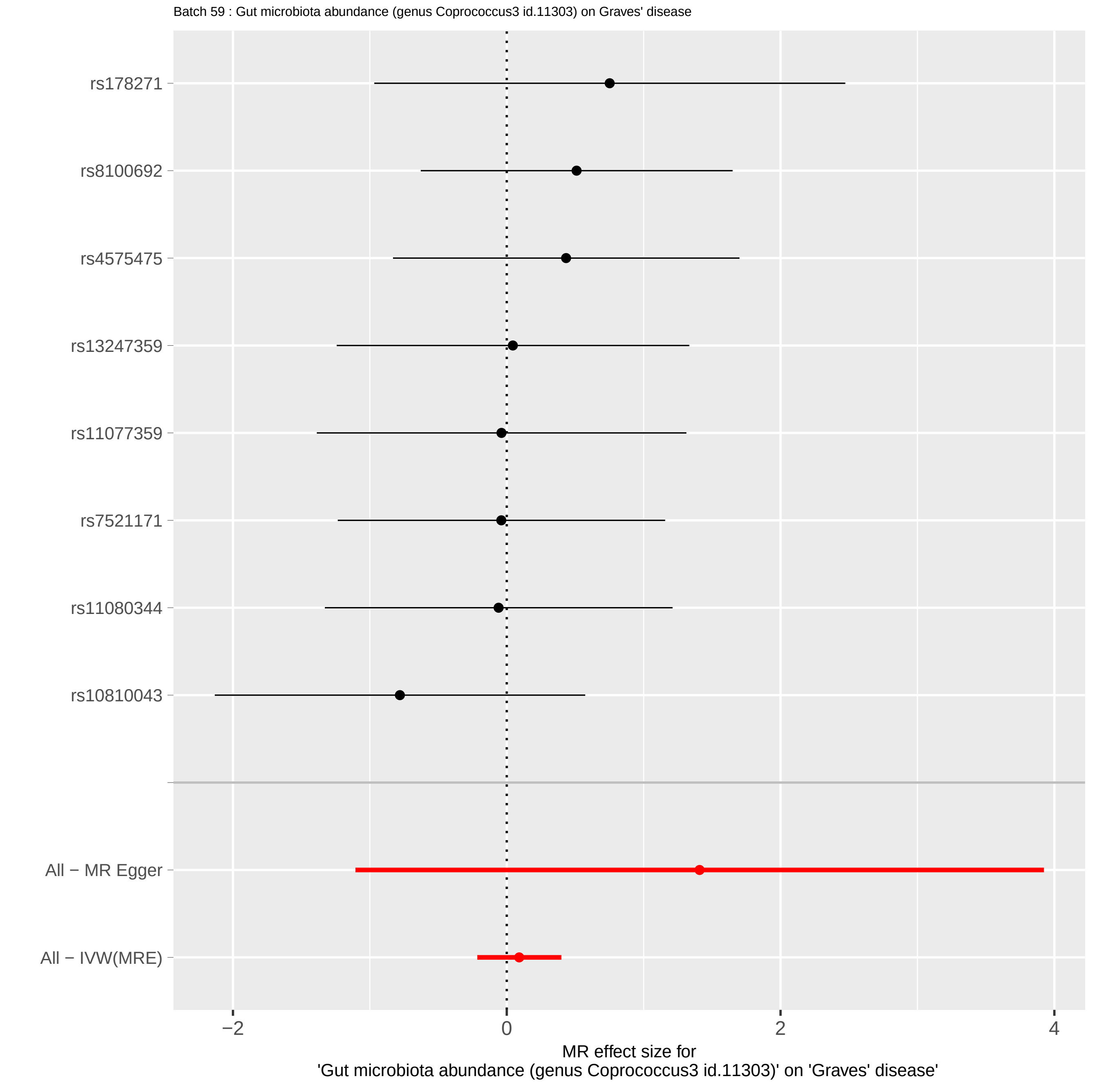
Batch 59 : Gut microbiota abundance (genus Coprococcus3 id.11303) on Graves' disease
rs178271
rs8100692
rs4575475
rs13247359
rs11077359
rs7521171
rs11080344
rs10810043
All − MR Egger
All − IVW(MRE)
−2
0
2
4
MR effect size for
'Gut microbiota abundance (genus Coprococcus3 id.11303)' on 'Graves' disease

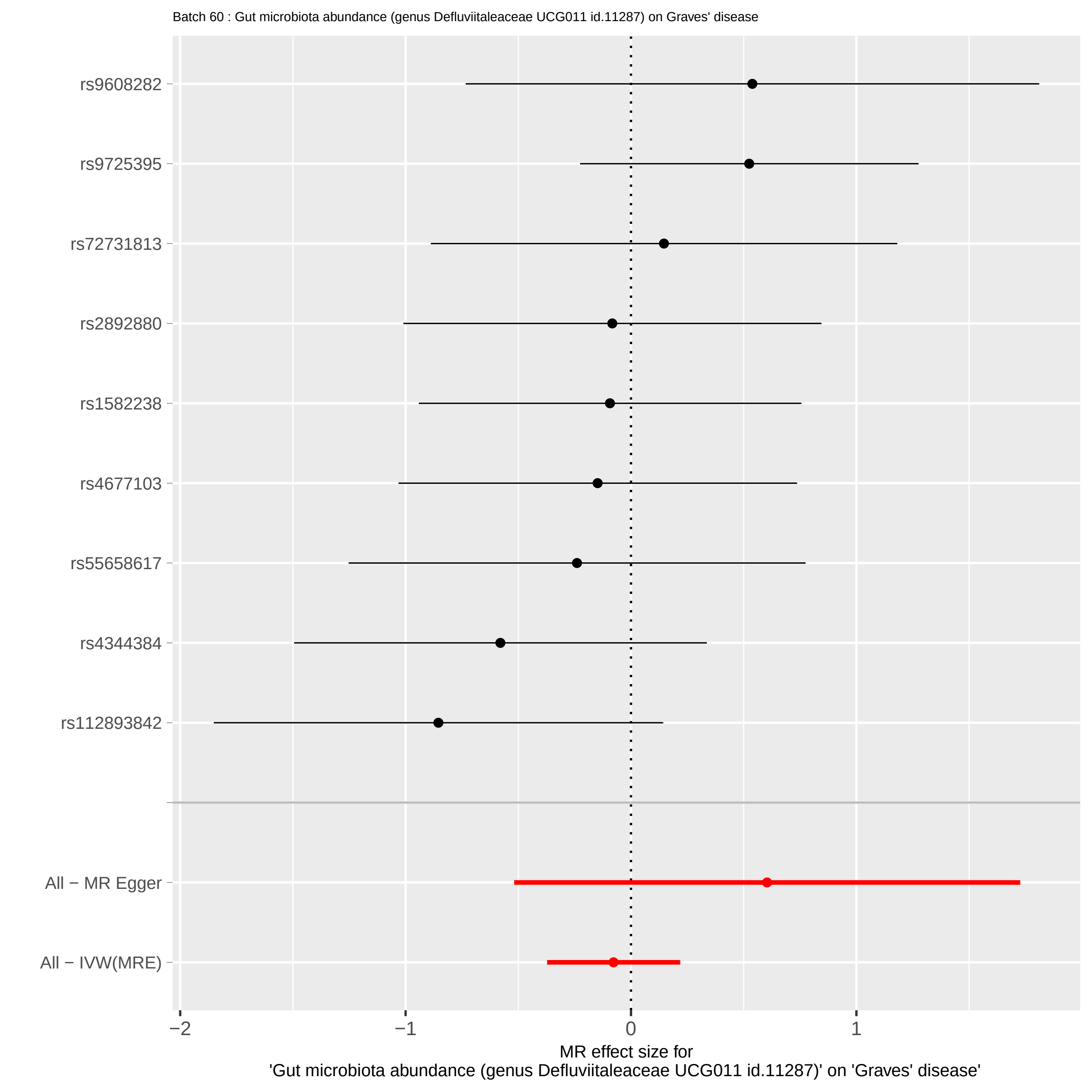

Batch 60 : Gut microbiota abundance (genus Defluviitaleaceae UCG011 id.11287) on Graves' disease
rs9608282
rs9725395
rs72731813
rs2892880
rs1582238
rs4677103
rs55658617
rs4344384
rs112893842
All − MR Egger
All − IVW(MRE)
−2
−1
0
1
MR effect size for
'Gut microbiota abundance (genus Defluviitaleaceae UCG011 id.11287)' on 'Graves' disease'

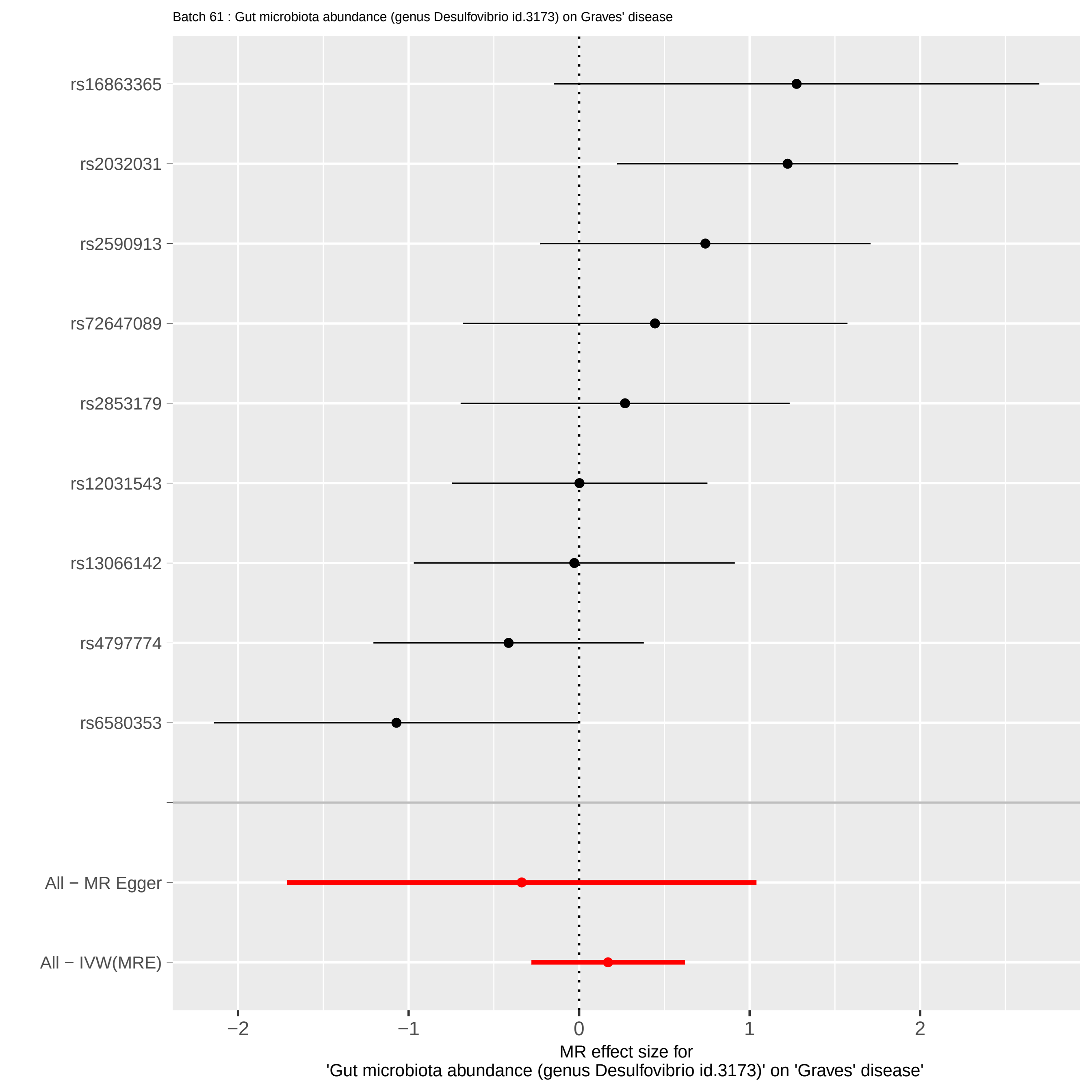
Batch 61 : Gut microbiota abundance (genus Desulfovibrio id.3173) on Graves' disease
rs16863365
rs2032031
rs2590913
rs72647089
rs2853179
rs12031543
rs13066142
rs4797774
rs6580353
All − MR Egger
All − IVW(MRE)
−2
−1
0
1
2
MR effect size for
'Gut microbiota abundance (genus Desulfovibrio id.3173)' on 'Graves' disease'

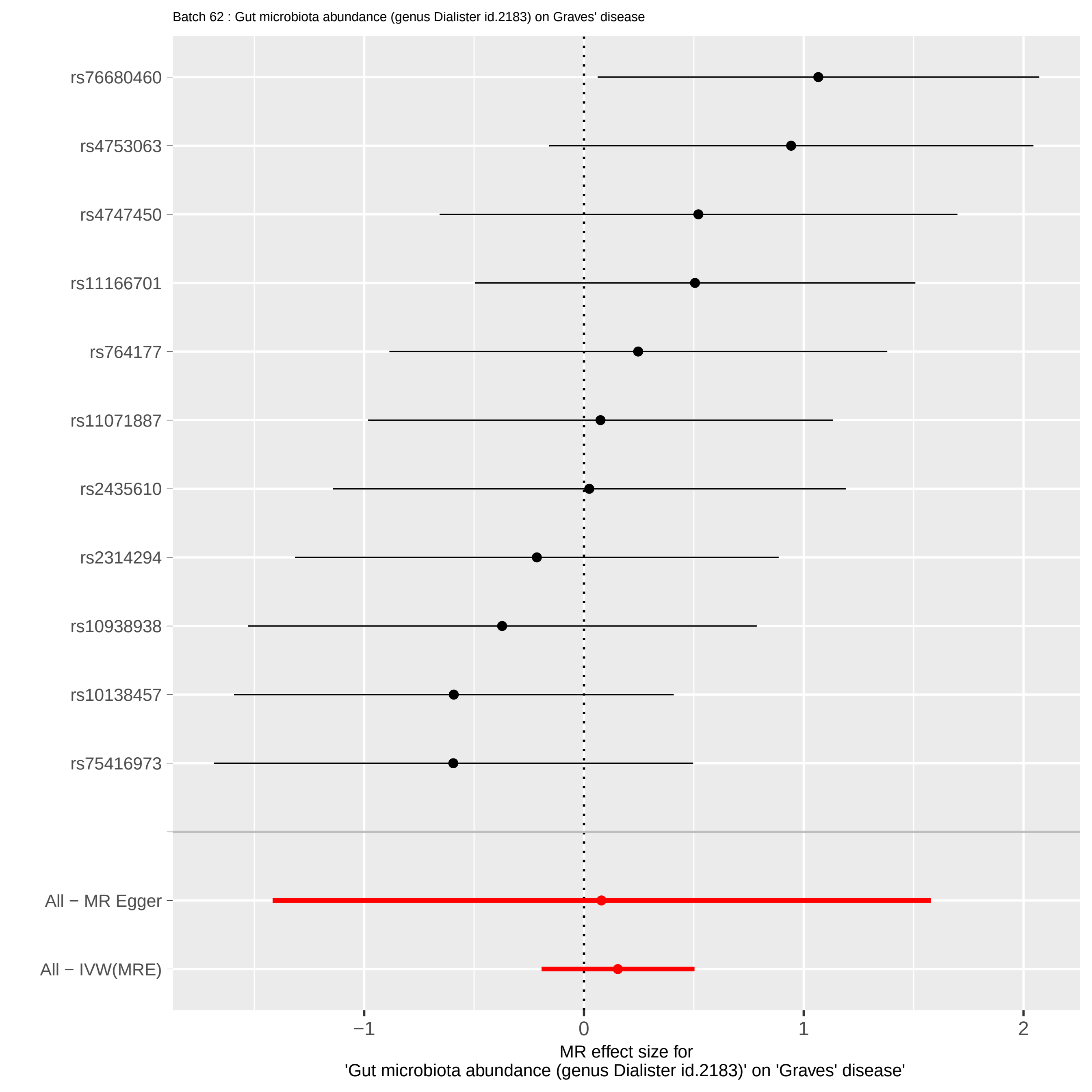
Batch 62 : Gut microbiota abundance (genus Dialister id.2183) on Graves' disease
rs76680460
rs4753063
rs4747450
rs11166701
rs764177
rs11071887
rs2435610
rs2314294
rs10938938
rs10138457
rs75416973
All − MR Egger
All − IVW(MRE)
−1
0
1
2
MR effect size for
'Gut microbiota abundance (genus Dialister id.2183)' on 'Graves' disease'

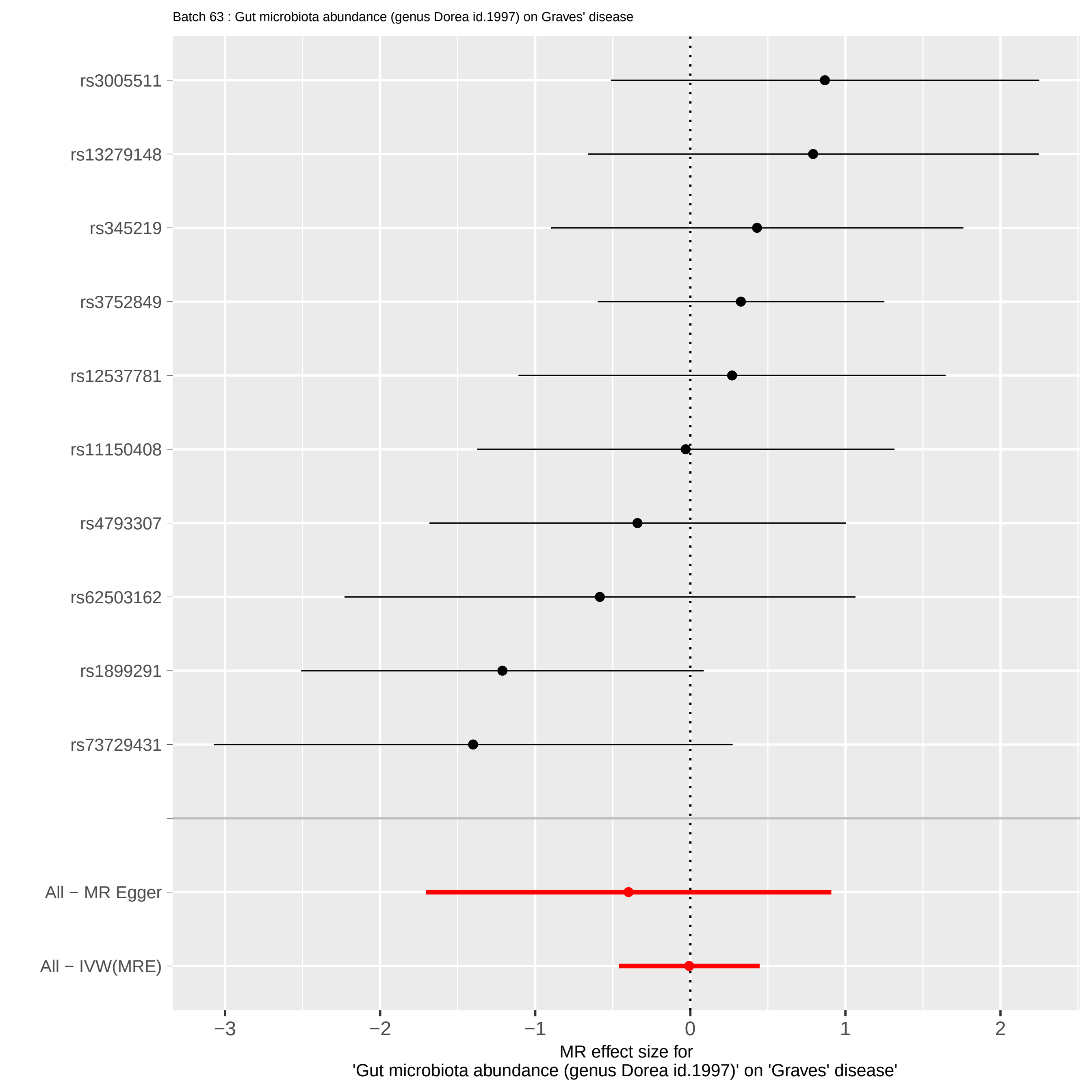

Batch 63 : Gut microbiota abundance (genus Dorea id.1997) on Graves' disease
rs3005511
rs13279148
rs345219
rs3752849
rs12537781
rs11150408
rs4793307
rs62503162
rs1899291
rs73729431
All − MR Egger
All − IVW(MRE)
−3
−2
−1
0
1
2
MR effect size for
'Gut microbiota abundance (genus Dorea id.1997)' on 'Graves' disease'

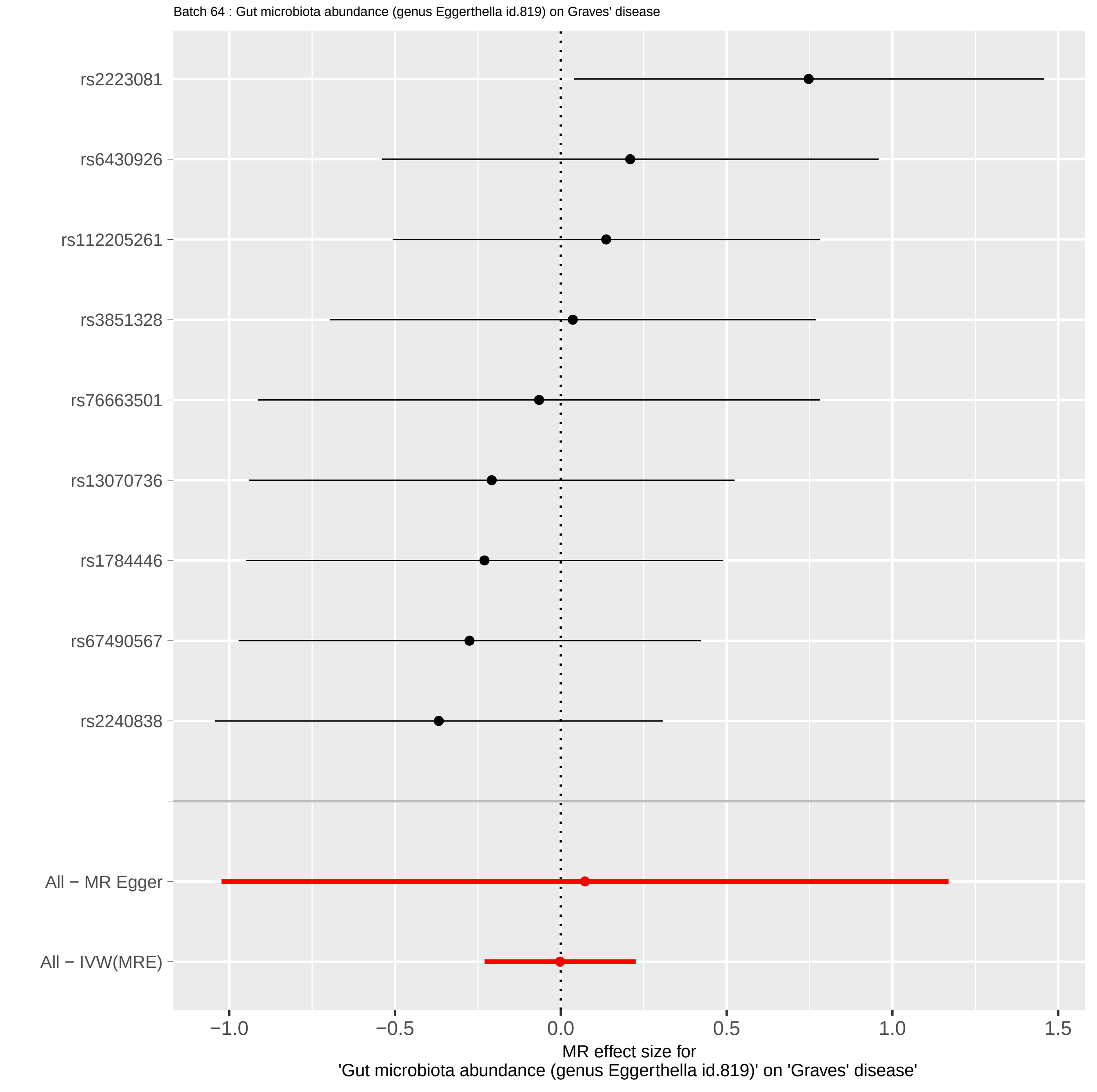

Batch 64 : Gut microbiota abundance (genus Eggerthella id.819) on Graves' disease
rs2223081
rs6430926
rs112205261
rs3851328
rs76663501
rs13070736
rs1784446
rs67490567
rs2240838
All − MR Egger
All − IVW(MRE)
−1.0
−0.5
0.0
0.5
1.0
1.5
MR effect size for
'Gut microbiota abundance (genus Eggerthella id.819)' on 'Graves' disease'

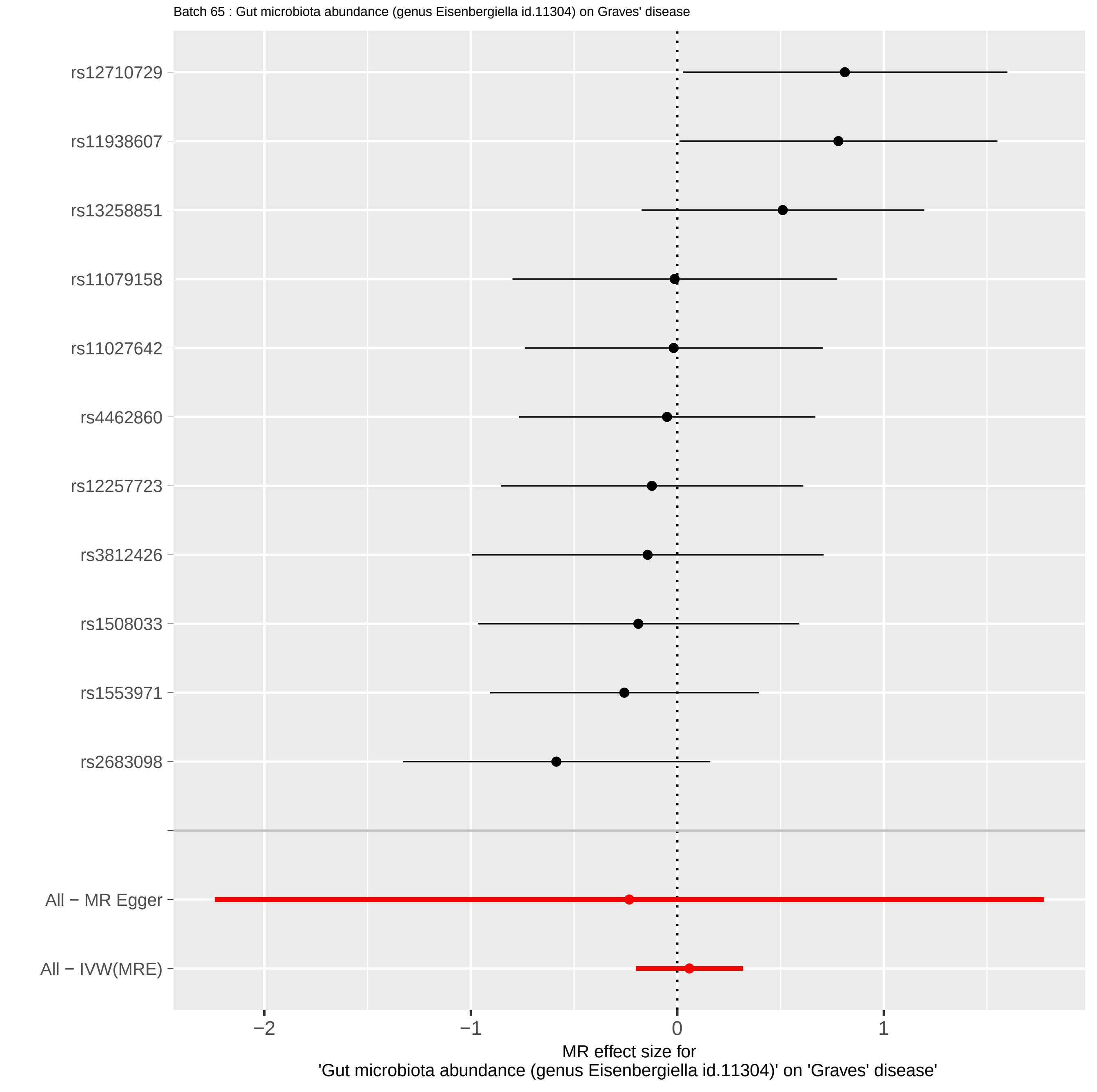

Batch 65 : Gut microbiota abundance (genus Eisenbergiella id.11304) on Graves' disease
rs12710729
rs11938607
rs13258851
rs11079158
rs11027642
rs4462860
rs12257723
rs3812426
rs1508033
rs1553971
rs2683098
All − MR Egger
All − IVW(MRE)
−2
−1
0
1
MR effect size for
'Gut microbiota abundance (genus Eisenbergiella id.11304)' on 'Graves' disease'

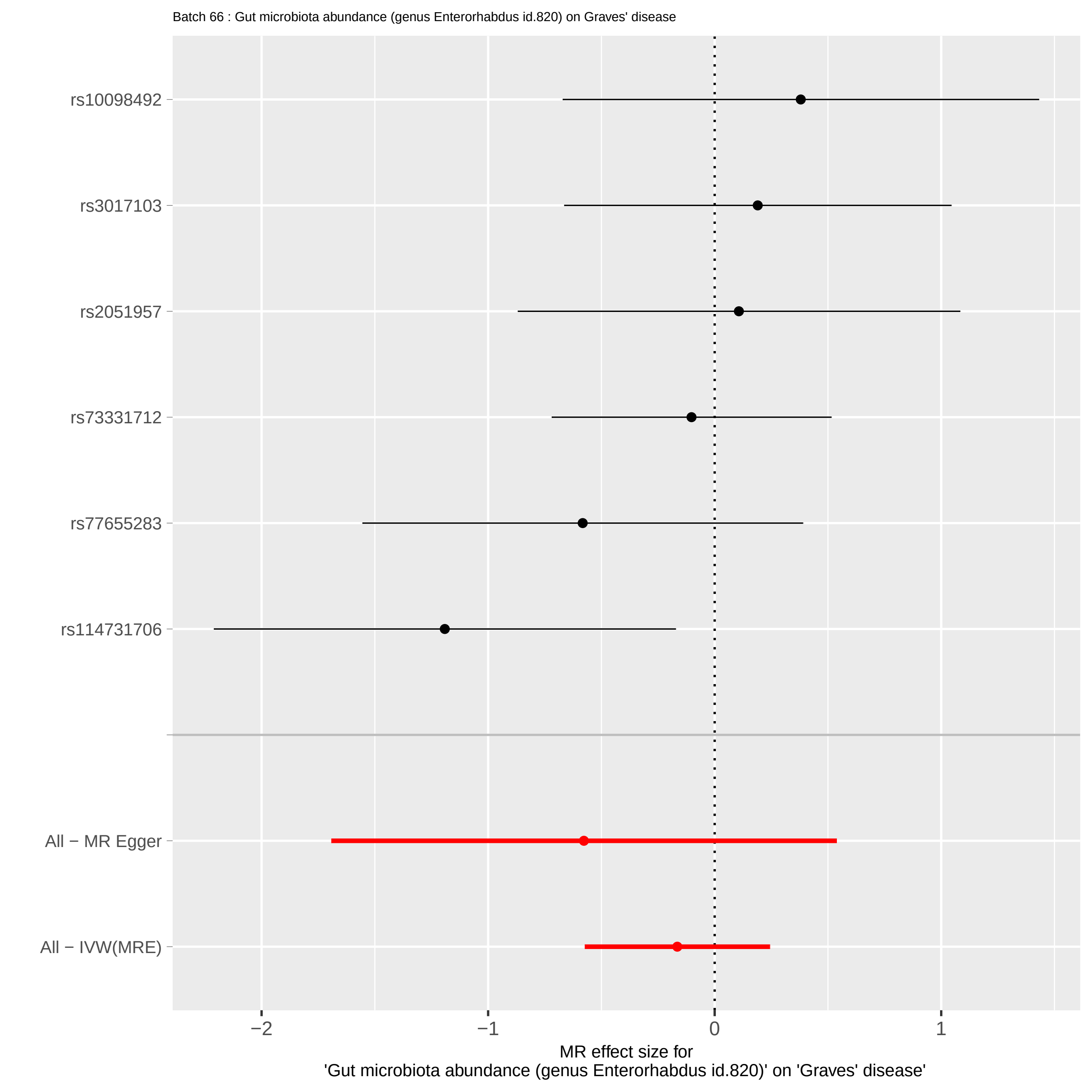
Batch 66 : Gut microbiota abundance (genus Enterorhabdus id.820) on Graves' disease
rs10098492
rs3017103
rs2051957
rs73331712
rs77655283
rs114731706
All − MR Egger
All − IVW(MRE)
−2
−1
0
1
MR effect size for
'Gut microbiota abundance (genus Enterorhabdus id.820)' on 'Graves' disease'

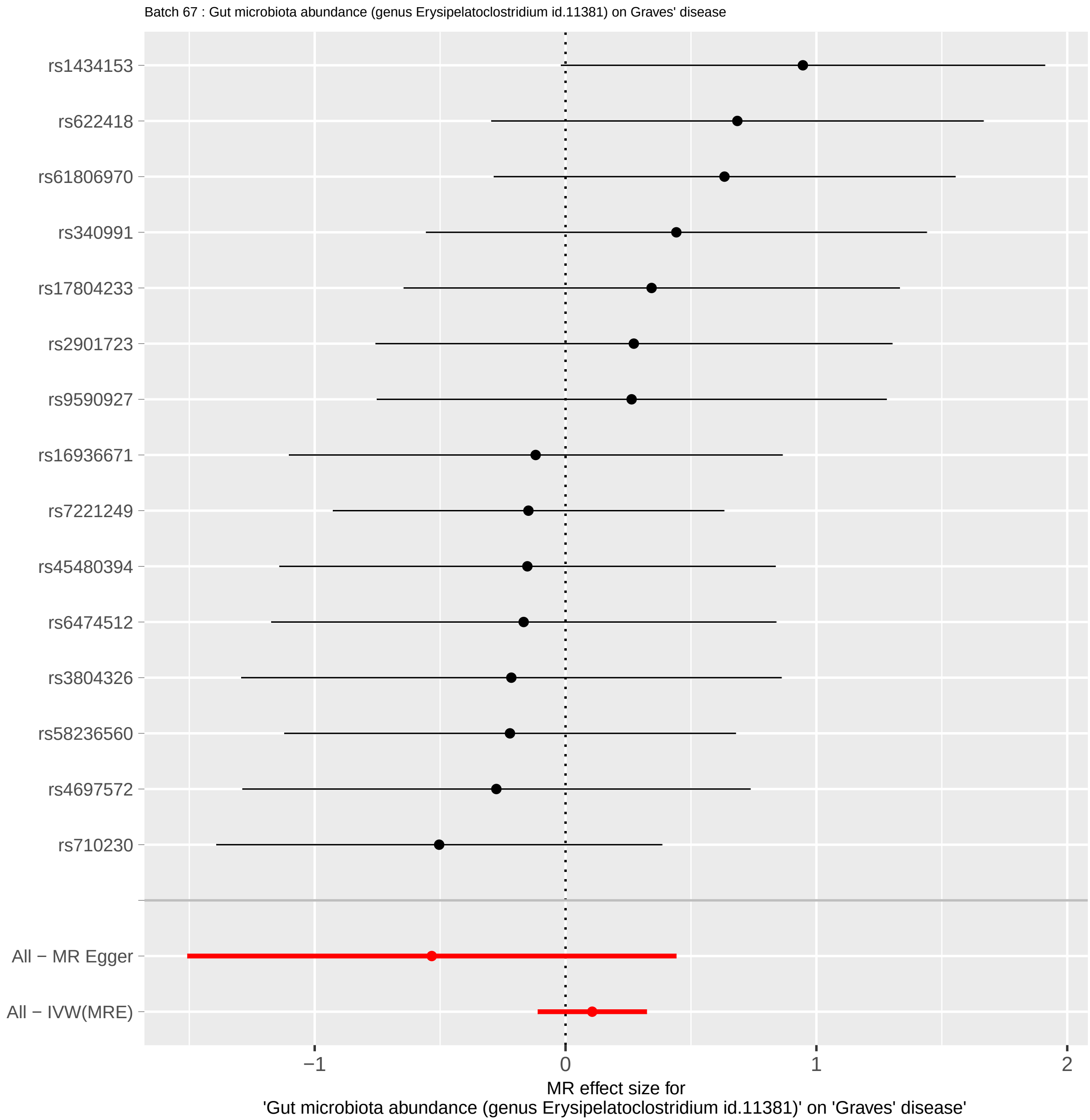

Batch 67 : Gut microbiota abundance (genus Erysipelatoclostridium id.11381) on Graves' disease
rs1434153
rs622418
rs61806970
rs340991
rs17804233
rs2901723
rs9590927
rs16936671
rs7221249
rs45480394
rs6474512
rs3804326
rs58236560
rs4697572
rs710230
All − MR Egger
All − IVW(MRE)
−1
0
1
2
MR effect size for
'Gut microbiota abundance (genus Erysipelatoclostridium id.11381)' on 'Graves' disease'

Batch 69 : Gut microbiota abundance (genus Escherichia Shigella id.3504) on Graves' disease

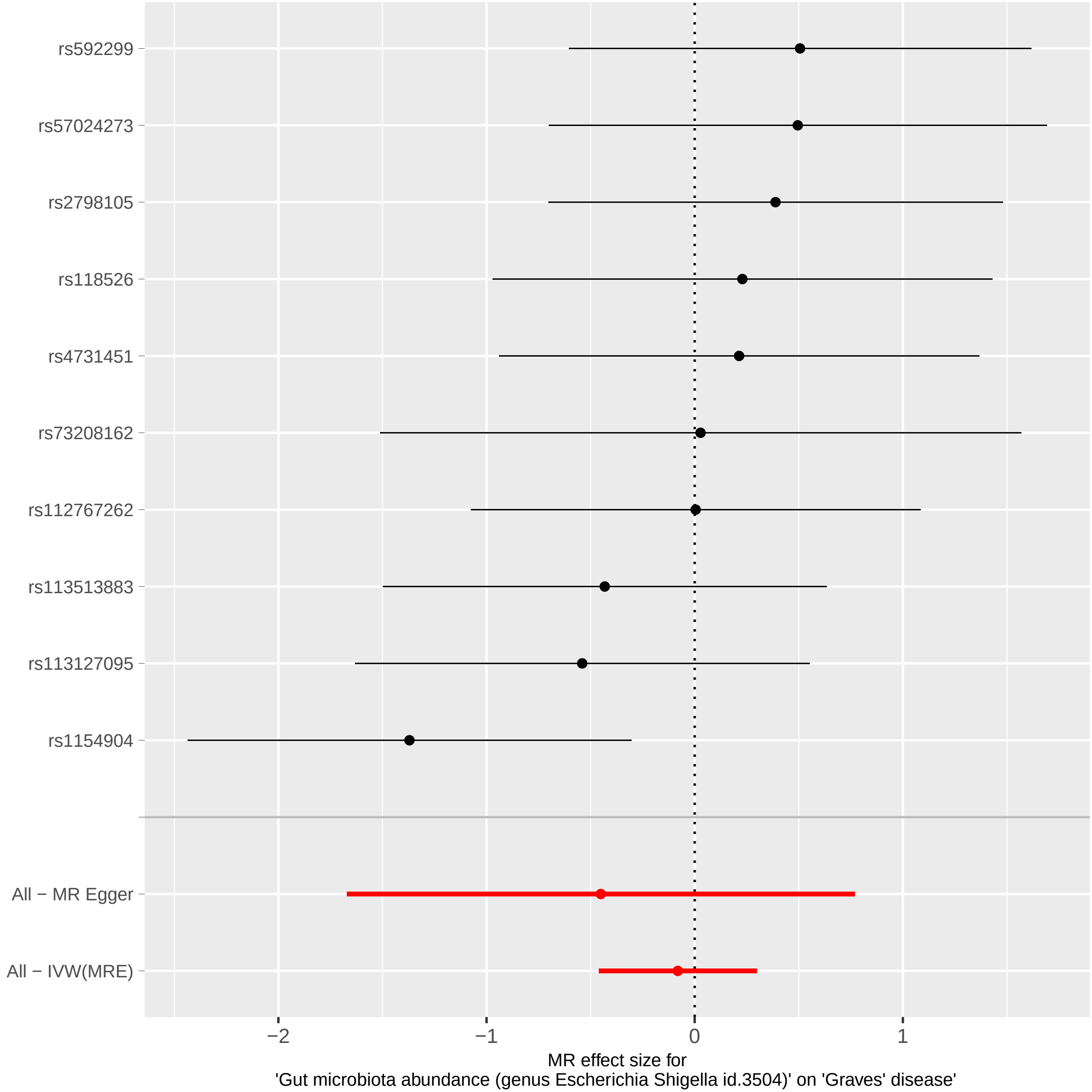

Batch 70 : Gut microbiota abundance (genus Eubacterium brachy group id.11296) on Graves' disease

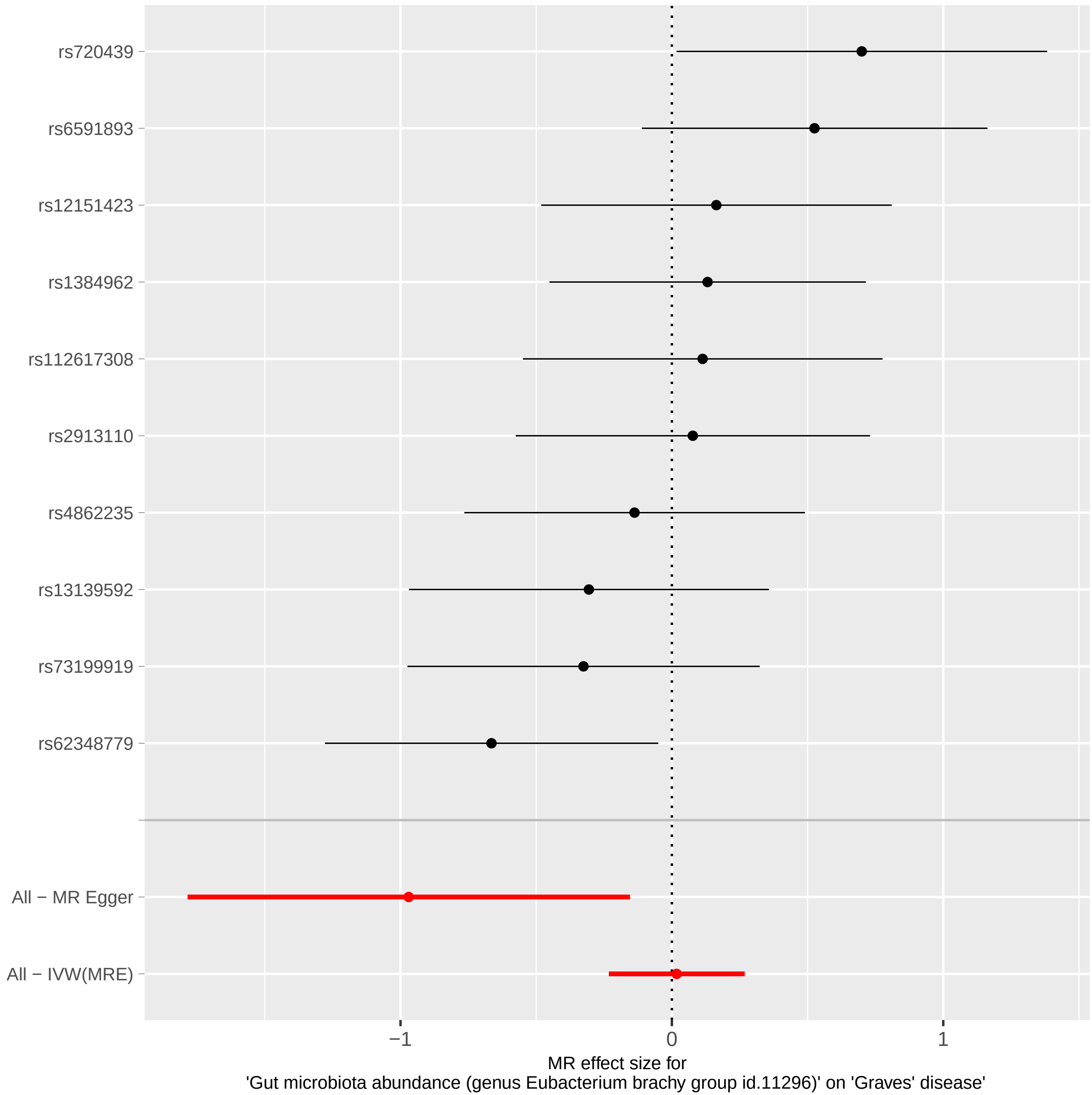

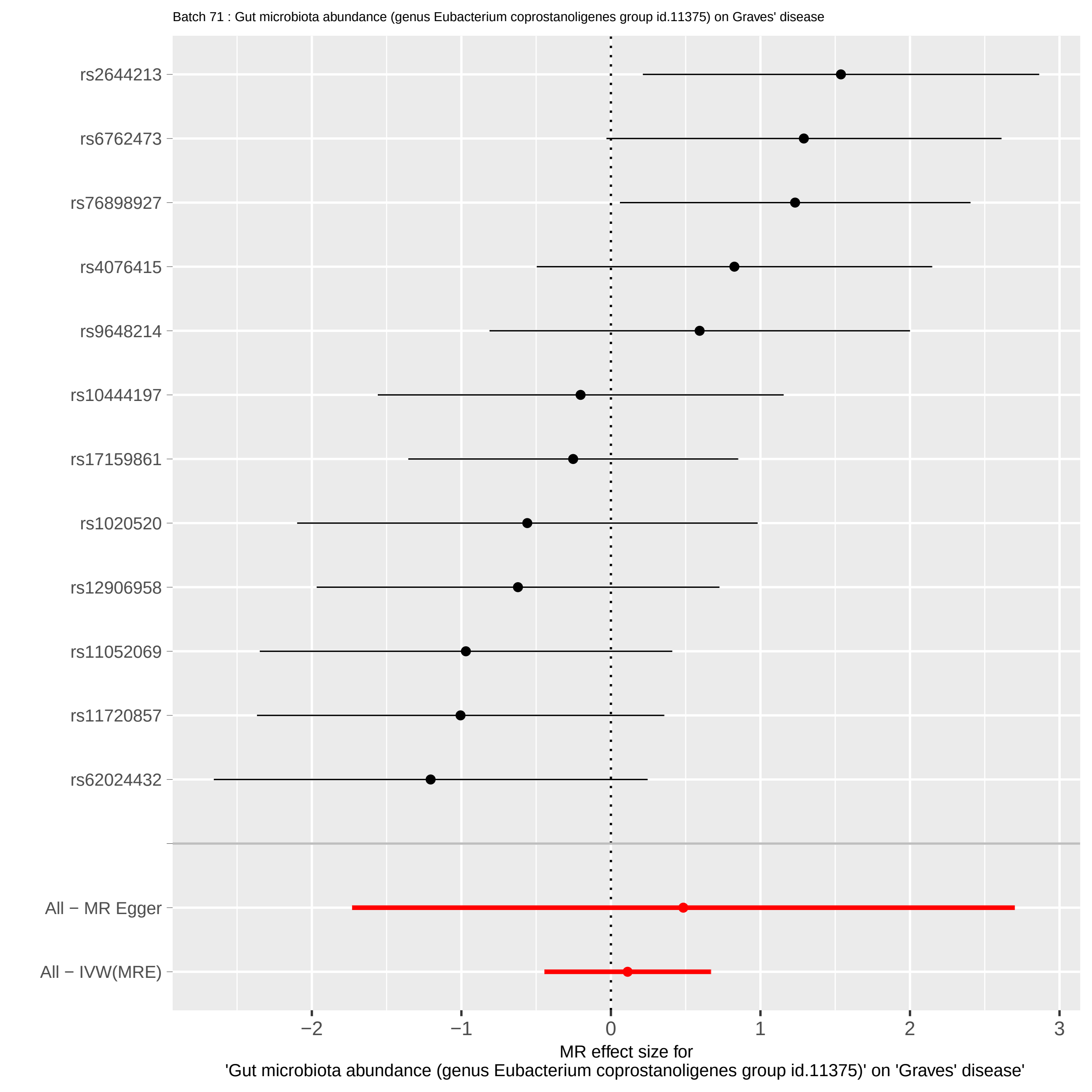
Batch 71 : Gut microbiota abundance (genus Eubacterium coprostanoligenes group id.11375) on Graves' disease
rs2644213
rs6762473
rs76898927
rs4076415
rs9648214
rs10444197
rs17159861
rs1020520
rs12906958
rs11052069
rs11720857
rs62024432
All − MR Egger
All − IVW(MRE)
−2
−1
0
1
2
3
MR effect size for
'Gut microbiota abundance (genus Eubacterium coprostanoligenes group id.11375)' on 'Graves' disease'

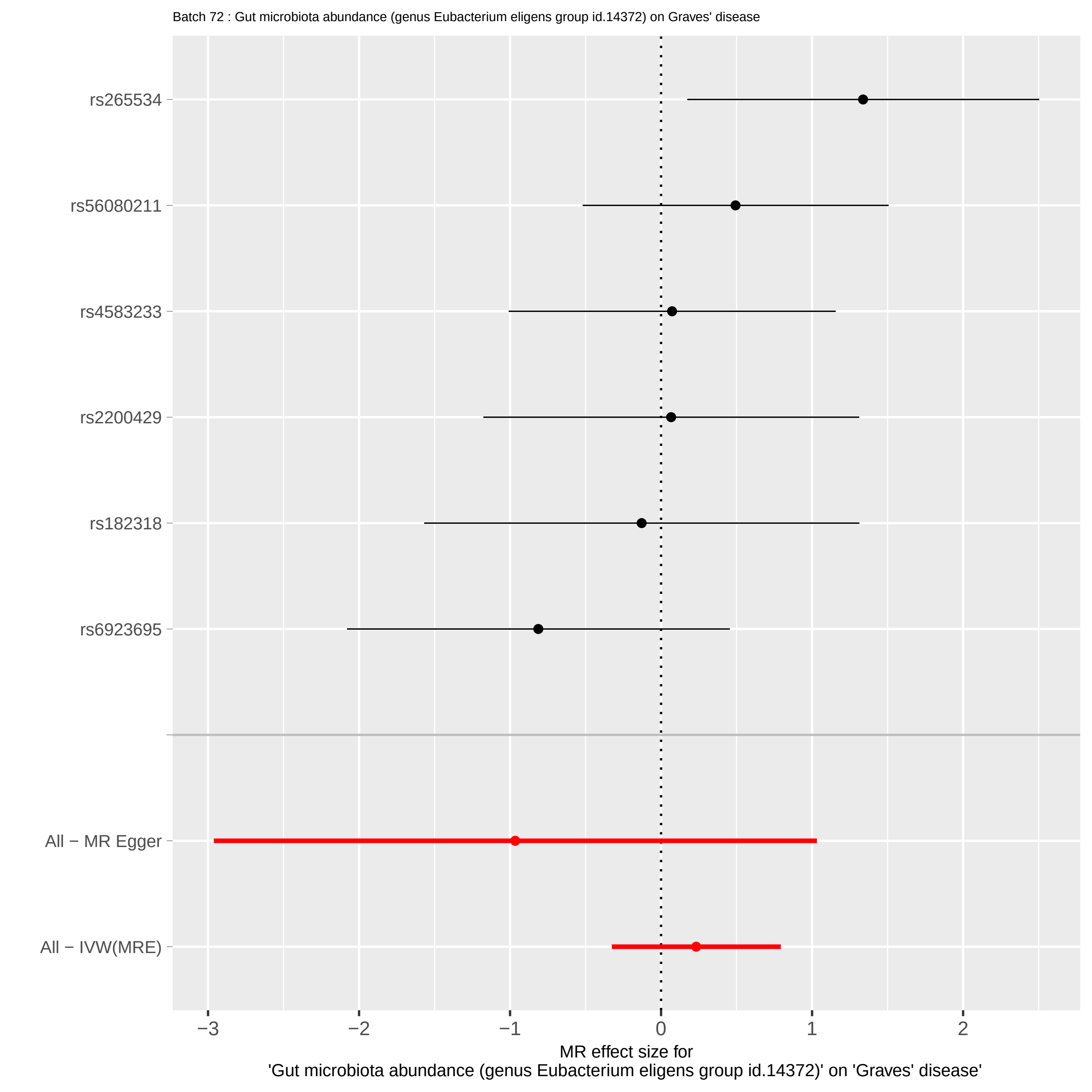
Batch 72 : Gut microbiota abundance (genus Eubacterium eligens group id.14372) on Graves' disease
rs265534
rs56080211
rs4583233
rs2200429
rs182318
rs6923695
All − MR Egger
All − IVW(MRE)
−3
−2
−1
0
1
2
MR effect size for
'Gut microbiota abundance (genus Eubacterium eligens group id.14372)' on 'Graves' disease'

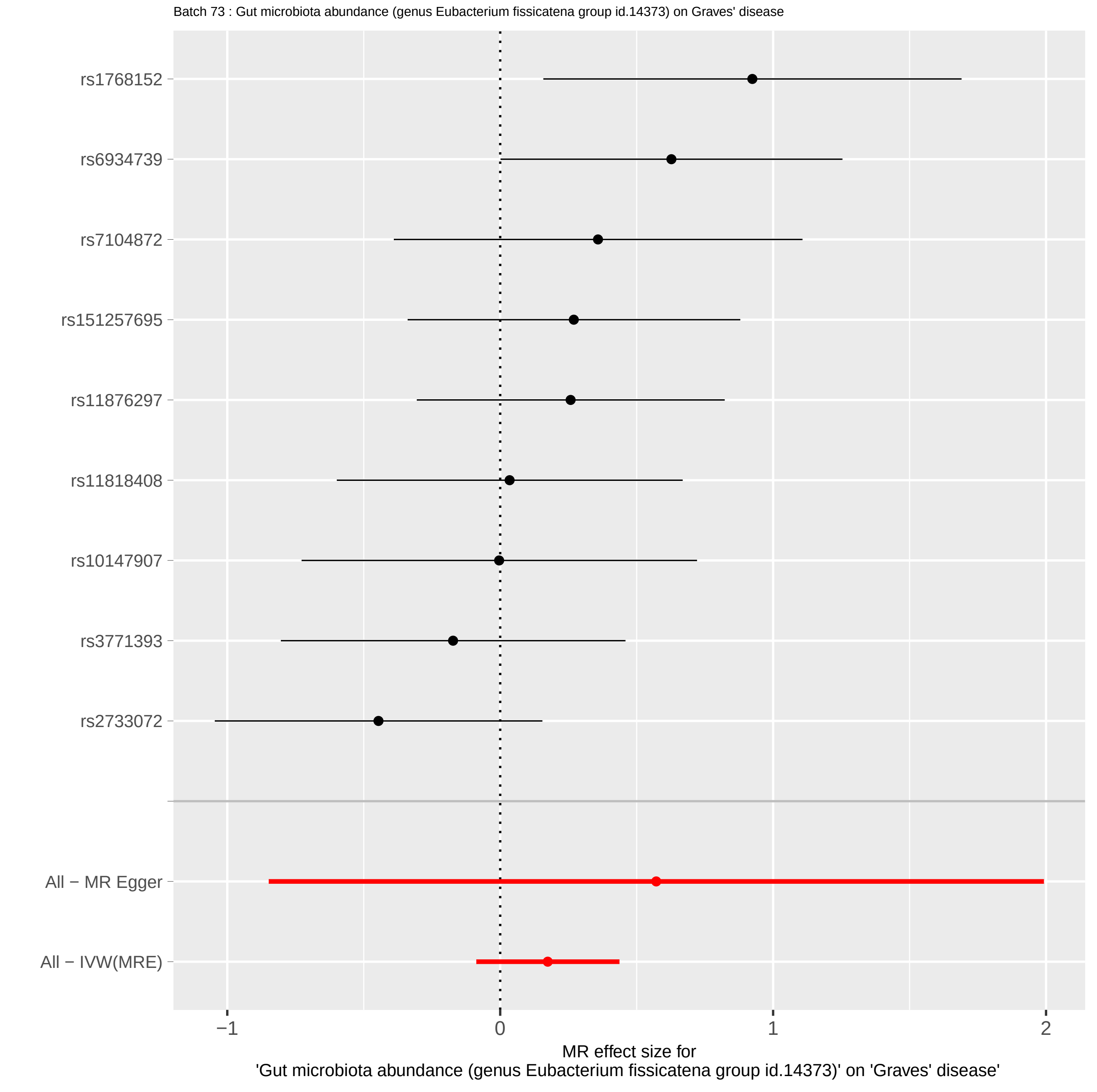
Batch 73 : Gut microbiota abundance (genus Eubacterium fissicatena group id.14373) on Graves' disease
rs1768152
rs6934739
rs7104872
rs151257695
rs11876297
rs11818408
rs10147907
rs3771393
rs2733072
All − MR Egger
All − IVW(MRE)
−1
0
1
2
MR effect size for
'Gut microbiota abundance (genus Eubacterium fissicatena group id.14373)' on 'Graves' disease'

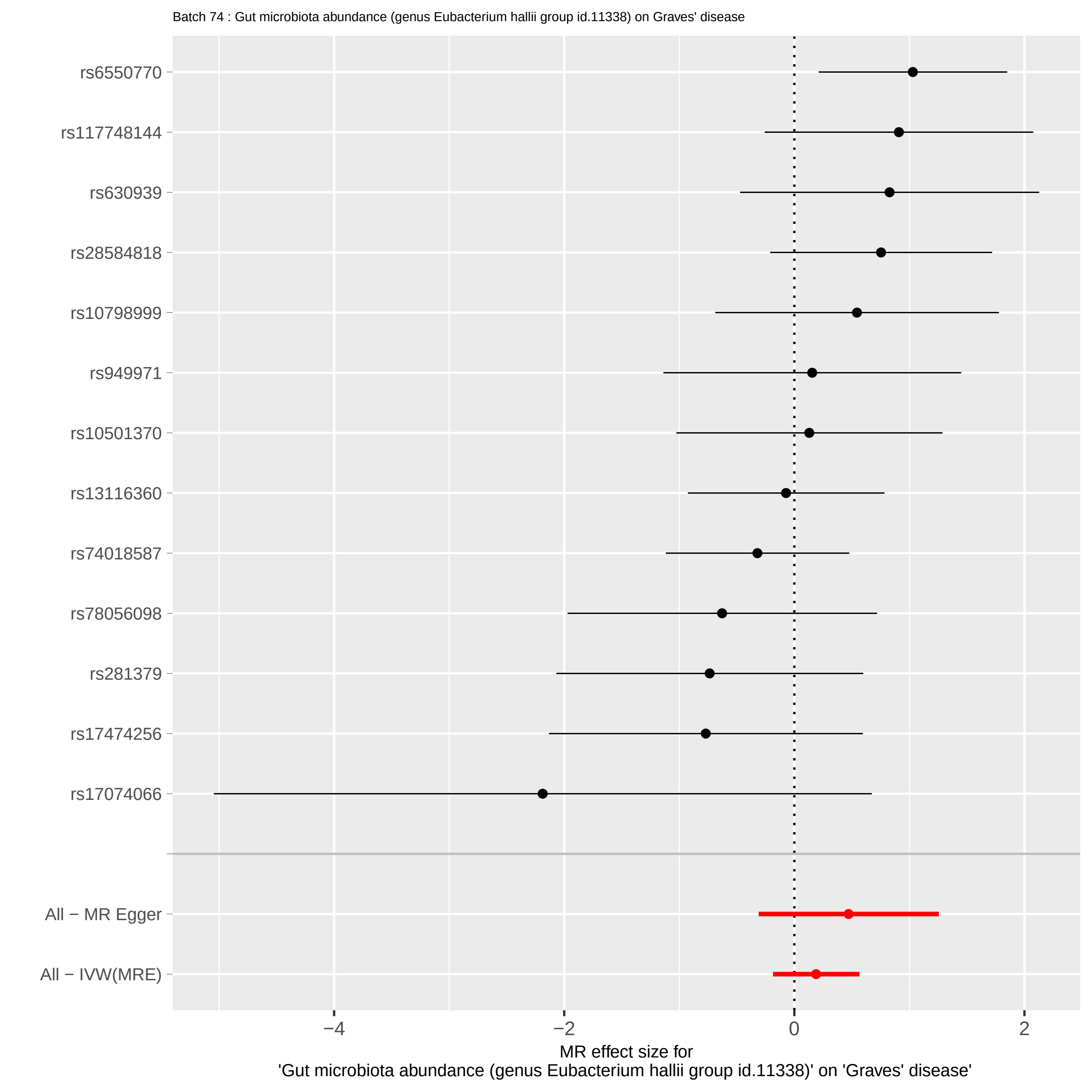

Batch 74 : Gut microbiota abundance (genus Eubacterium hallii group id.11338) on Graves' disease
rs6550770
rs117748144
rs630939
rs28584818
rs10798999
rs949971
rs10501370
rs13116360
rs74018587
rs78056098
rs281379
rs17474256
rs17074066
All − MR Egger
All − IVW(MRE)
−4
−2
0
2
MR effect size for
'Gut microbiota abundance (genus Eubacterium hallii group id.11338)' on 'Graves' disease'

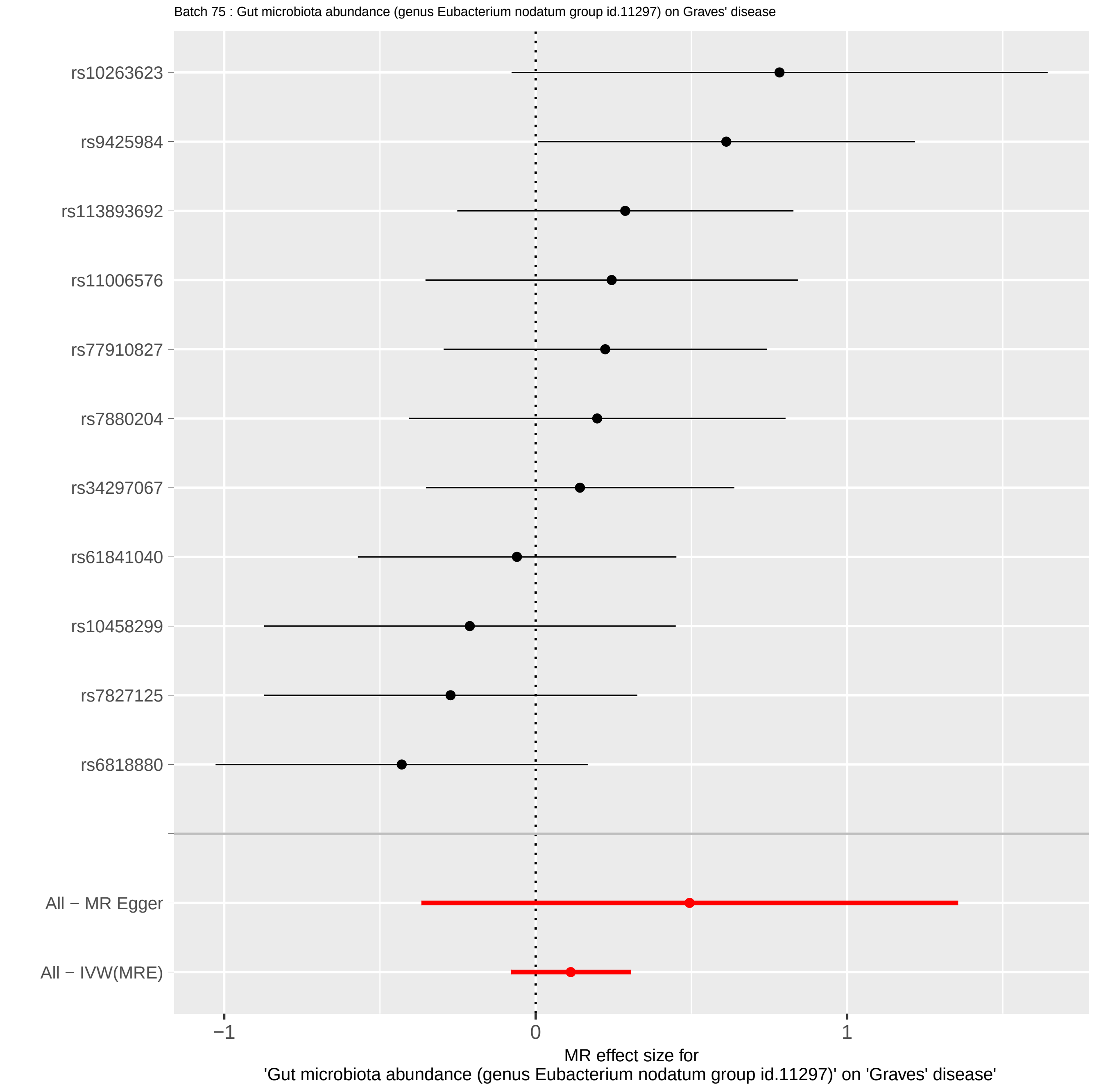

Batch 75 : Gut microbiota abundance (genus Eubacterium nodatum group id.11297) on Graves' disease
rs10263623
rs9425984
rs113893692
rs11006576
rs77910827
rs7880204
rs34297067
rs61841040
rs10458299
rs7827125
rs6818880
All − MR Egger
All − IVW(MRE)
−1
0
1
MR effect size for
'Gut microbiota abundance (genus Eubacterium nodatum group id.11297)' on 'Graves' disease'

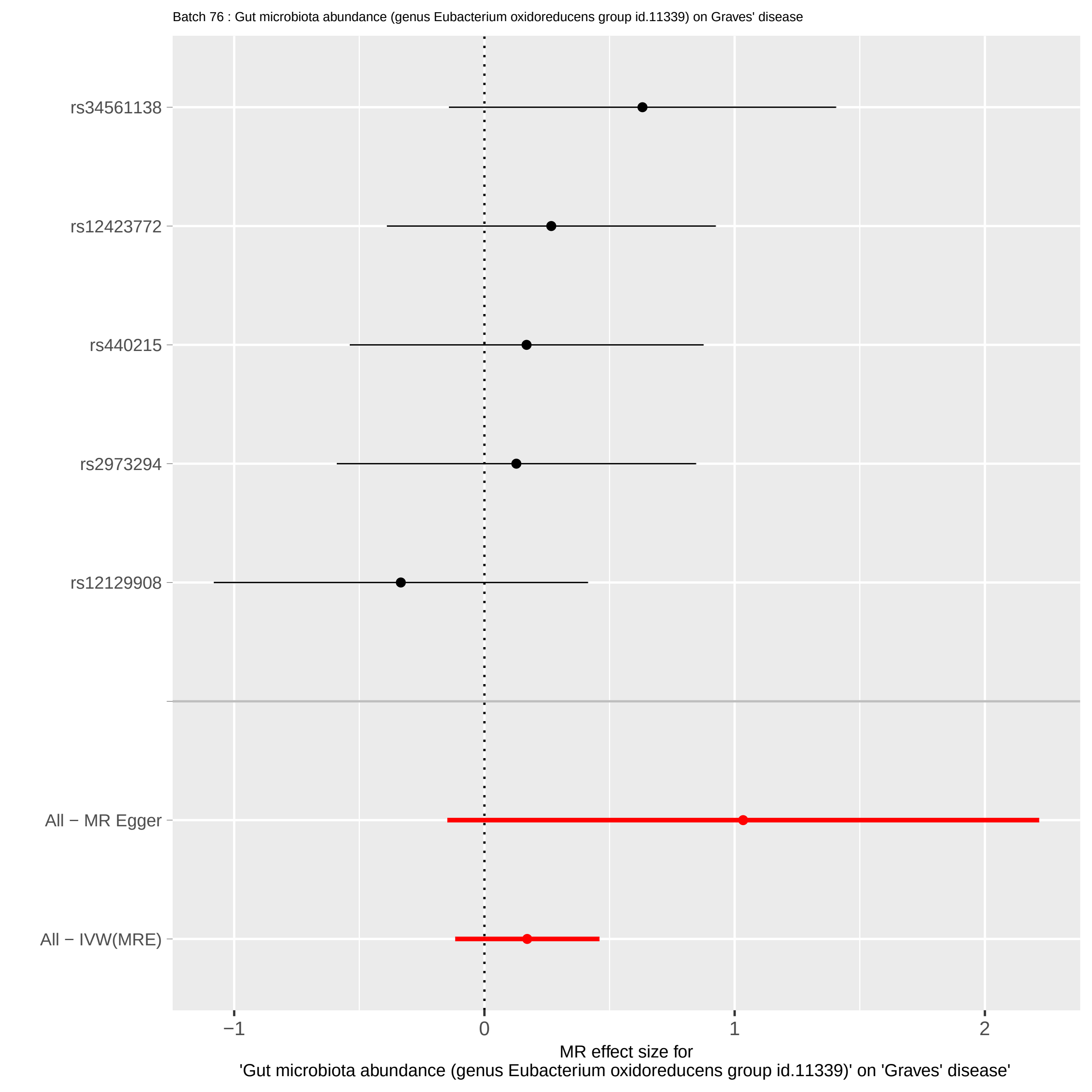

Batch 76 : Gut microbiota abundance (genus Eubacterium oxidoreducens group id.11339) on Graves' disease
rs34561138
rs12423772
rs440215
rs2973294
rs12129908
All − MR Egger
All − IVW(MRE)
−1
0
1
2
MR effect size for
'Gut microbiota abundance (genus Eubacterium oxidoreducens group id.11339)' on 'Graves' disease'

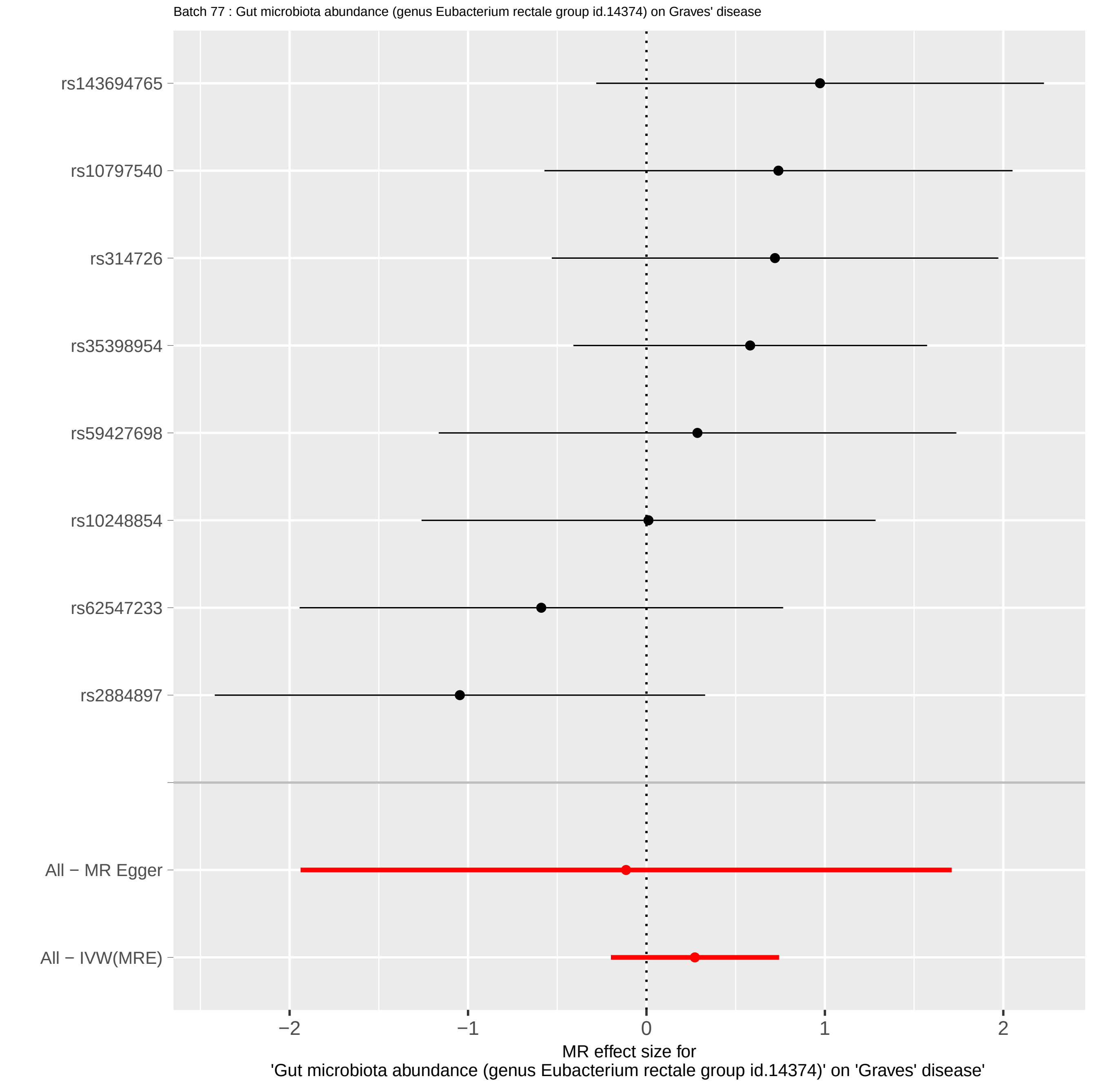
Batch 77 : Gut microbiota abundance (genus Eubacterium rectale group id.14374) on Graves' disease
rs143694765
rs10797540
rs314726
rs35398954
rs59427698
rs10248854
rs62547233
rs2884897
All − MR Egger
All − IVW(MRE)
−2
−1
0
1
2
MR effect size for
'Gut microbiota abundance (genus Eubacterium rectale group id.14374)' on 'Graves' disease'

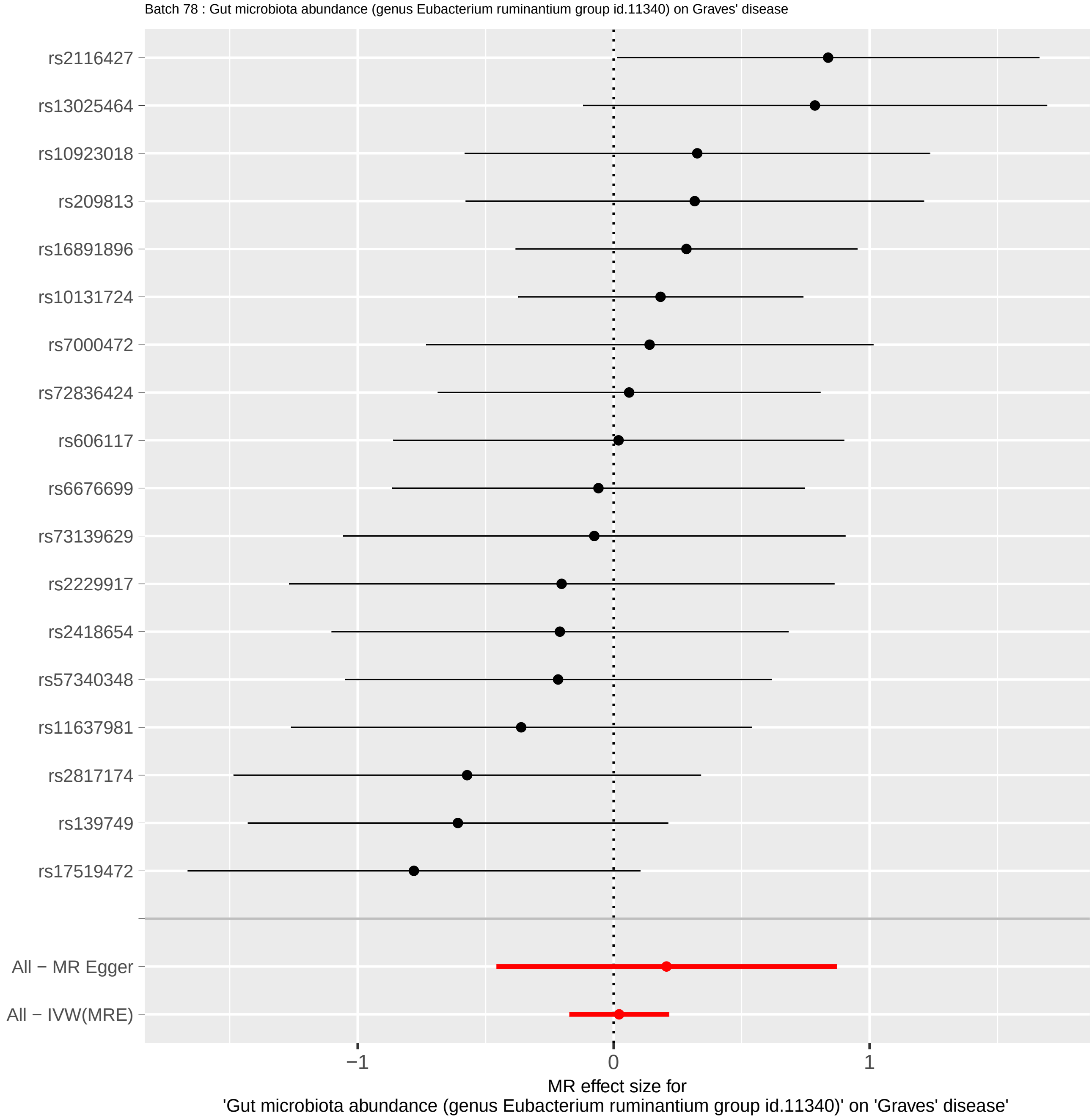
Batch 78 : Gut microbiota abundance (genus Eubacterium ruminantium group id.11340) on Graves' disease
rs2116427
rs13025464
rs10923018
rs209813
rs16891896
rs10131724
rs7000472
rs72836424
rs606117
rs6676699
rs73139629
rs2229917
rs2418654
rs57340348
rs11637981
rs2817174
rs139749
rs17519472
All − MR Egger
All − IVW(MRE)
−1
0
1
MR effect size for
'Gut microbiota abundance (genus Eubacterium ruminantium group id.11340)' on 'Graves' disease'

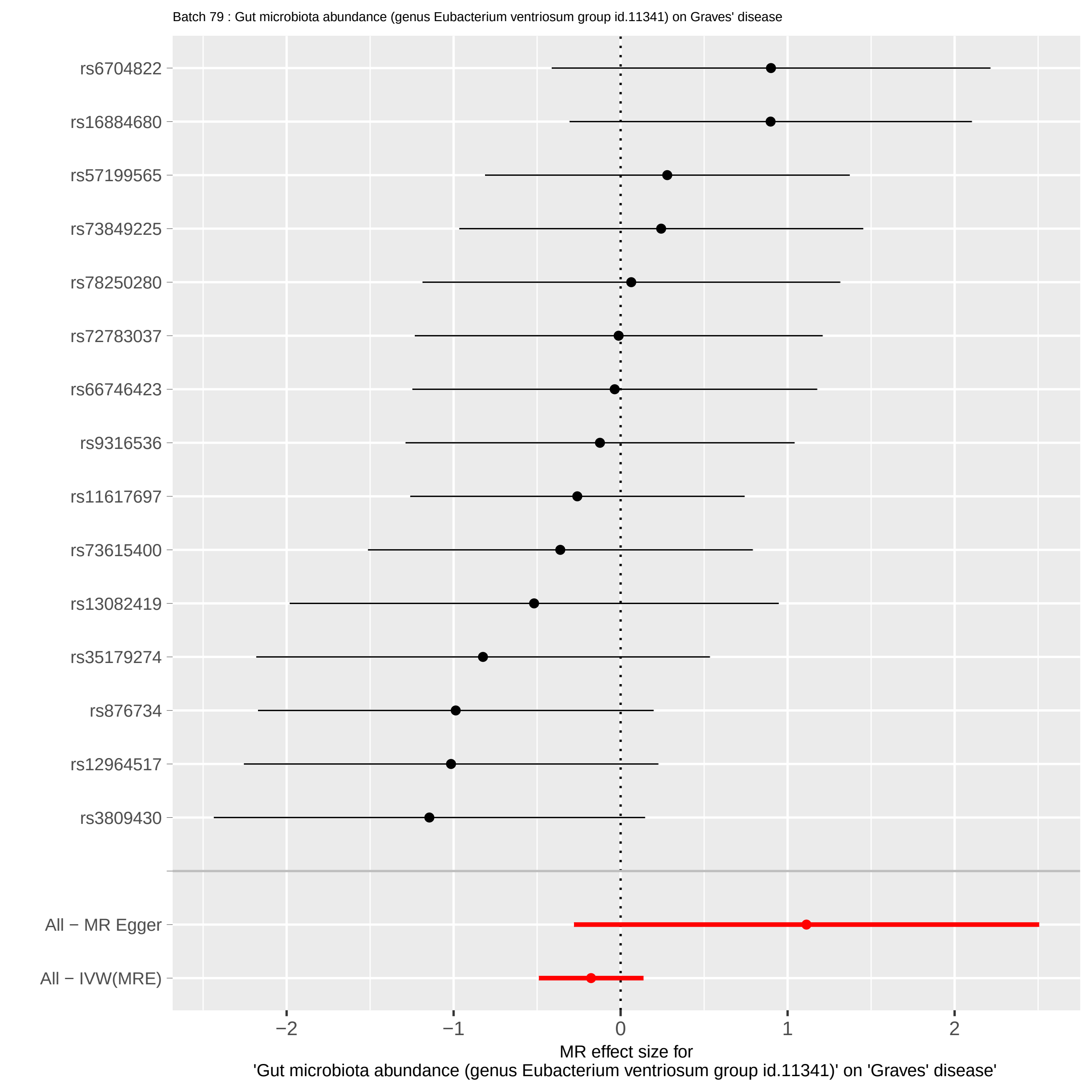

Batch 79 : Gut microbiota abundance (genus Eubacterium ventriosum group id.11341) on Graves' disease
rs6704822
rs16884680
rs57199565
rs73849225
rs78250280
rs72783037
rs66746423
rs9316536
rs11617697
rs73615400
rs13082419
rs35179274
rs876734
rs12964517
rs3809430
All − MR Egger
All − IVW(MRE)
−2
−1
0
1
2
MR effect size for
'Gut microbiota abundance (genus Eubacterium ventriosum group id.11341)' on 'Graves' disease'

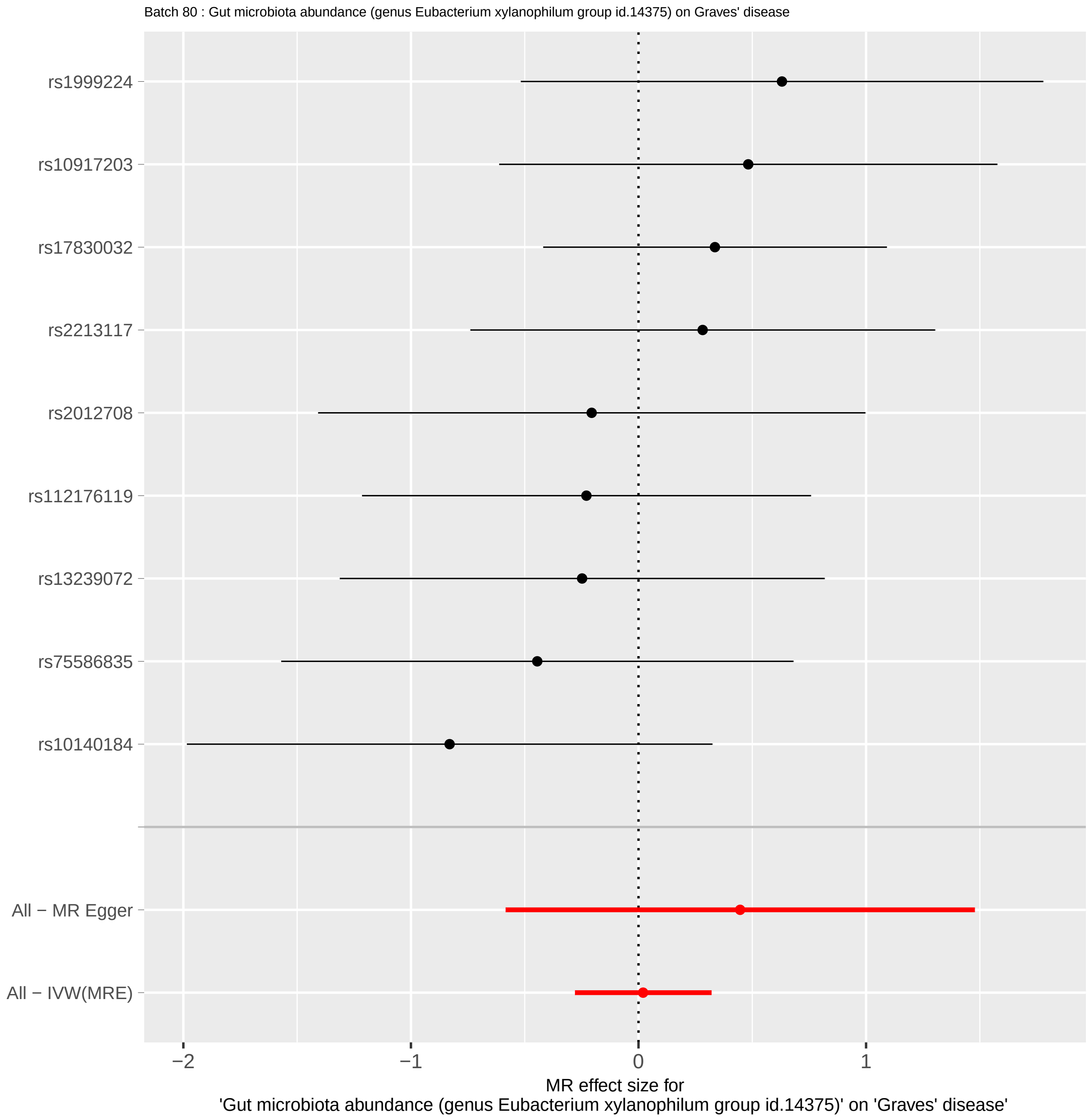

Batch 80 : Gut microbiota abundance (genus Eubacterium xylanophilum group id.14375) on Graves' disease
rs1999224
rs10917203
rs17830032
rs2213117
rs2012708
rs112176119
rs13239072
rs75586835
rs10140184
All − MR Egger
All − IVW(MRE)
−2
−1
0
1
MR effect size for
'Gut microbiota abundance (genus Eubacterium xylanophilum group id.14375)' on 'Graves' disease'

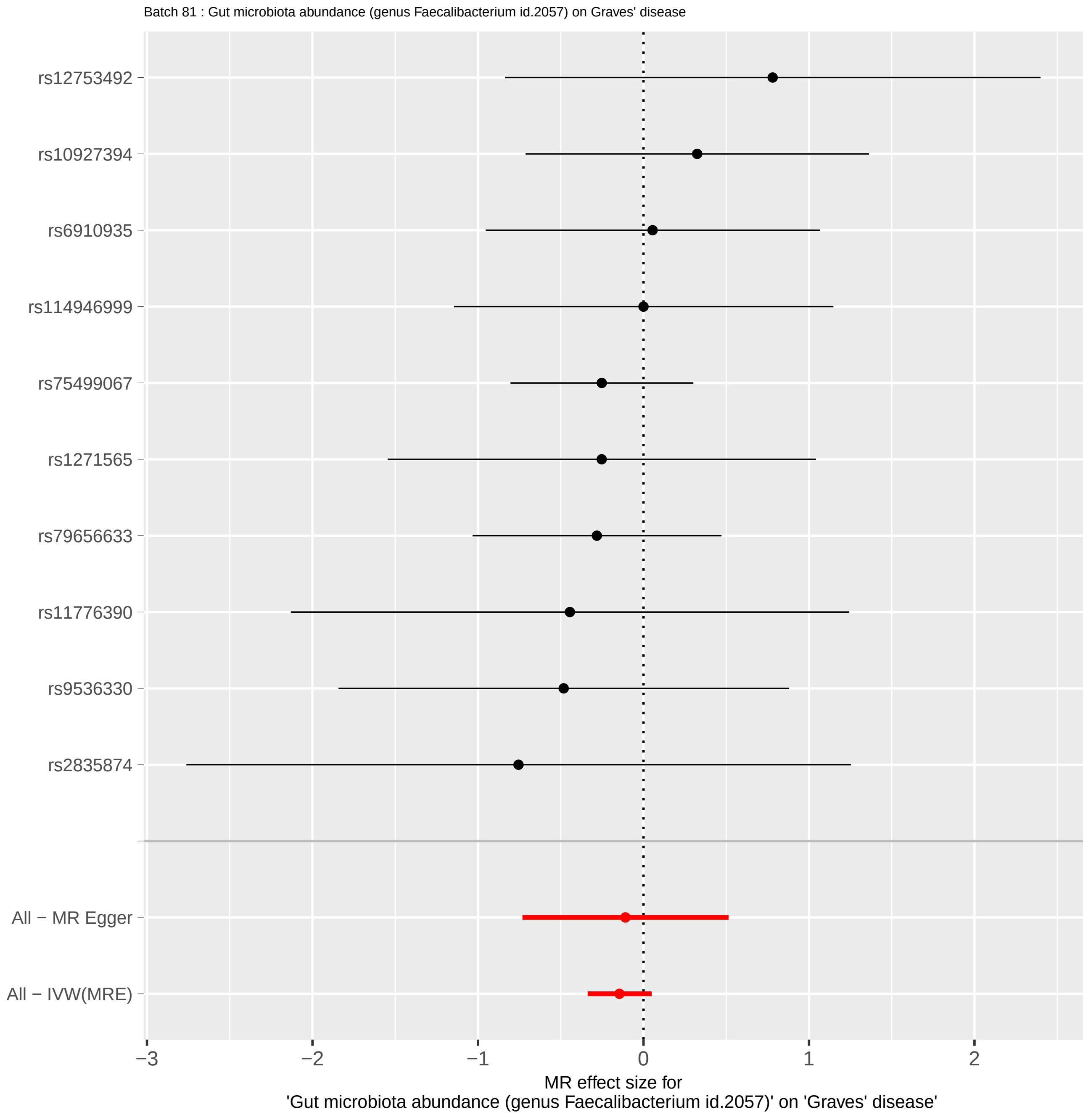

Batch 81 : Gut microbiota abundance (genus Faecalibacterium id.2057) on Graves' disease
rs12753492
rs10927394
rs6910935
rs114946999
rs75499067
rs1271565
rs79656633
rs11776390
rs9536330
rs2835874
All − MR Egger
All − IVW(MRE)
−3
−2
−1
0
1
2
MR effect size for
'Gut microbiota abundance (genus Faecalibacterium id.2057)' on 'Graves' disease'

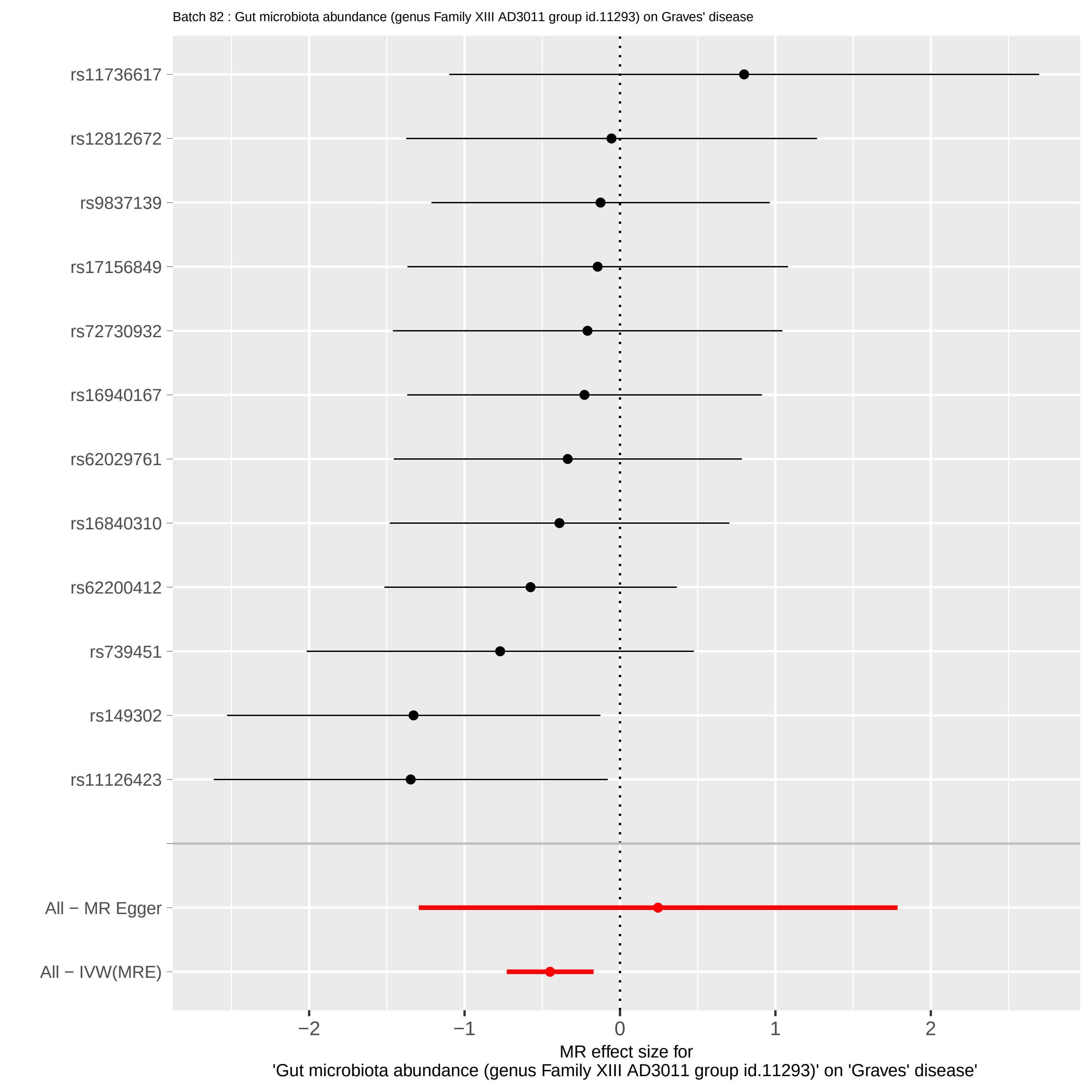

Batch 82 : Gut microbiota abundance (genus Family XIII AD3011 group id.11293) on Graves' disease
rs11736617
rs12812672
rs9837139
rs17156849
rs72730932
rs16940167
rs62029761
rs16840310
rs62200412
rs739451
rs149302
rs11126423
All − MR Egger
All − IVW(MRE)
−2
−1
0
1
2
MR effect size for
'Gut microbiota abundance (genus Family XIII AD3011 group id.11293)' on 'Graves' disease'

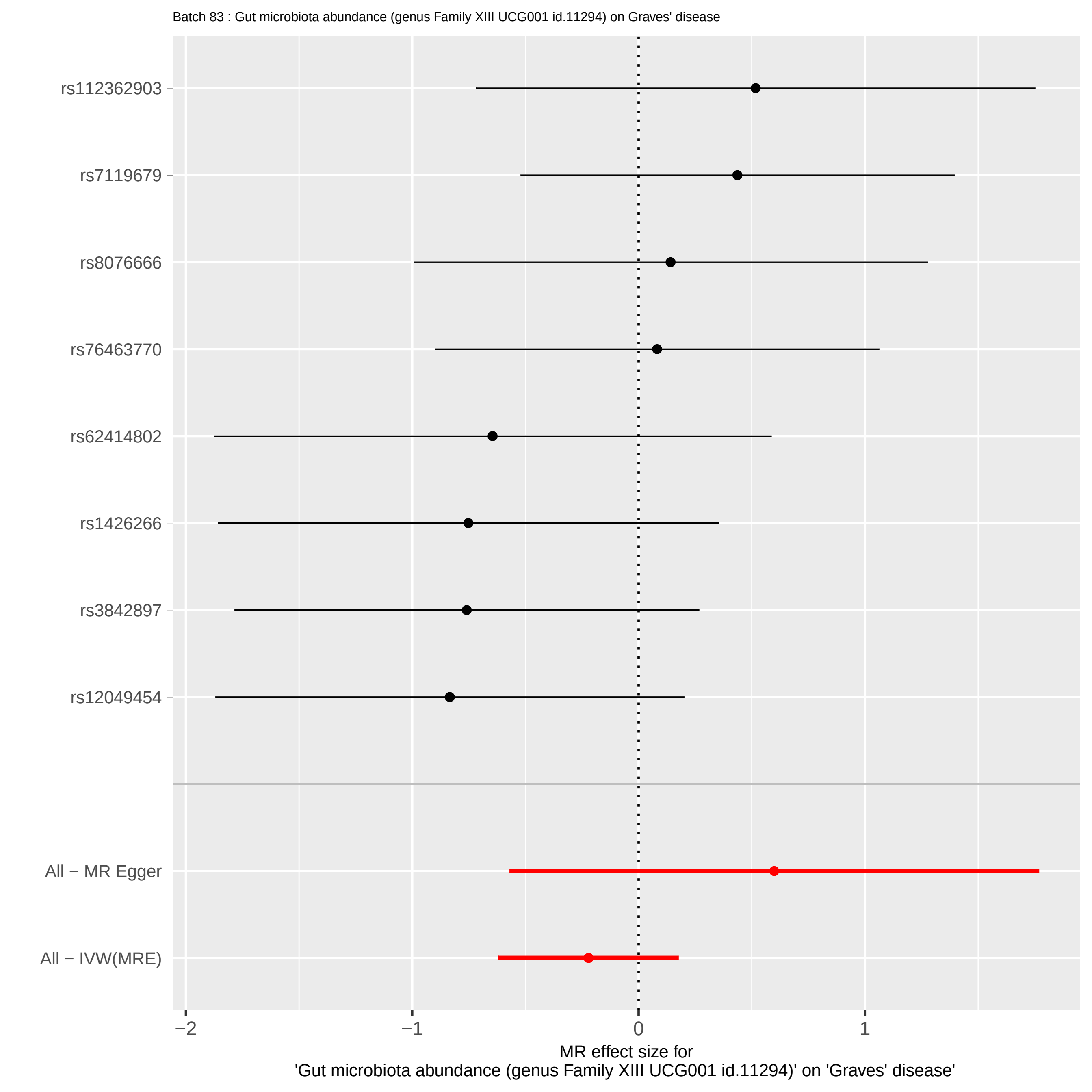
Batch 83 : Gut microbiota abundance (genus Family XIII UCG001 id.11294) on Graves' disease
rs112362903
rs7119679
rs8076666
rs76463770
rs62414802
rs1426266
rs3842897
rs12049454
All − MR Egger
All − IVW(MRE)
−2
−1
0
1
MR effect size for
'Gut microbiota abundance (genus Family XIII UCG001 id.11294)' on 'Graves' disease'

Batch 84 : Gut microbiota abundance (genus Flavonifractor id.2059) on Graves' disease

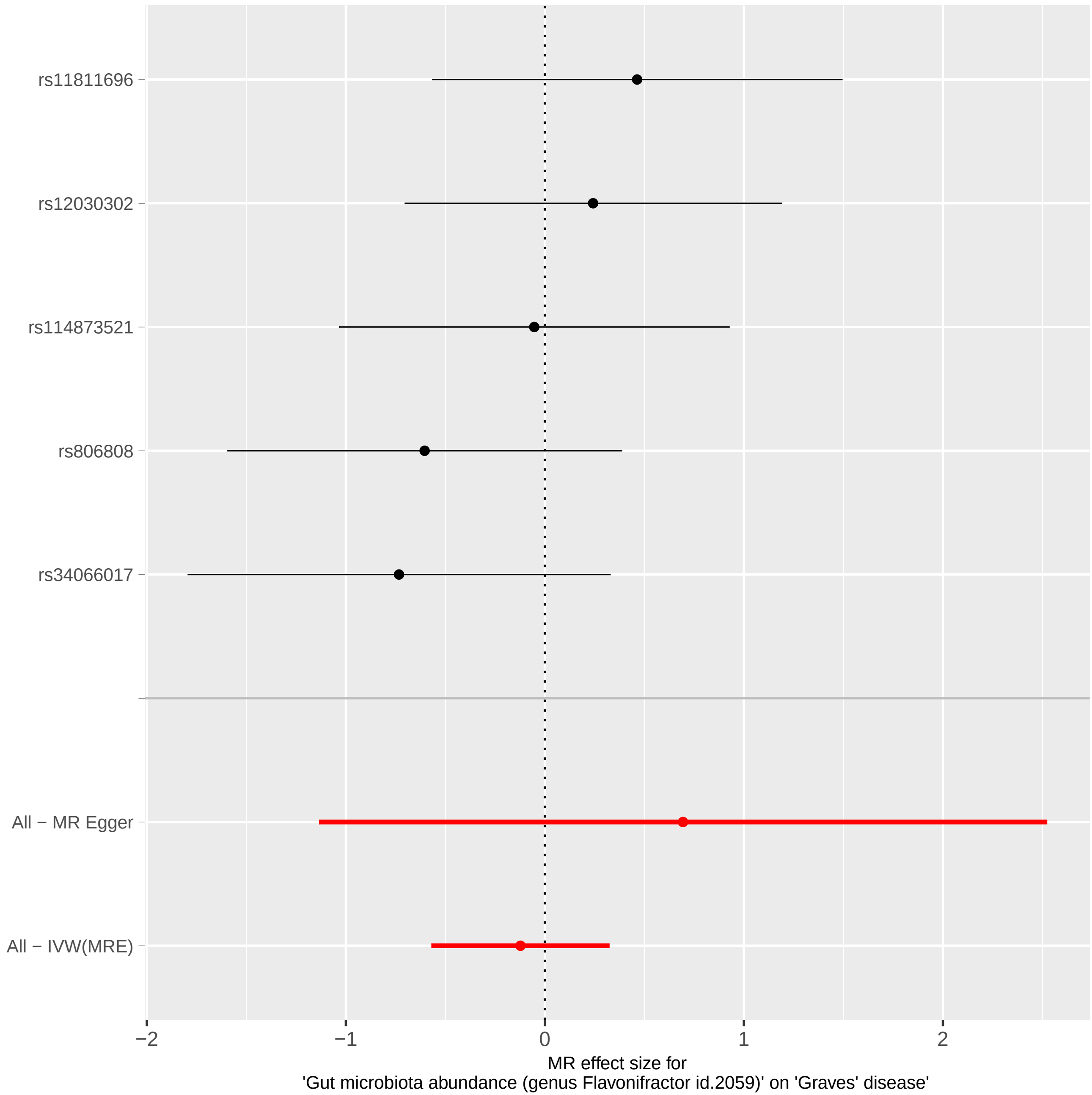

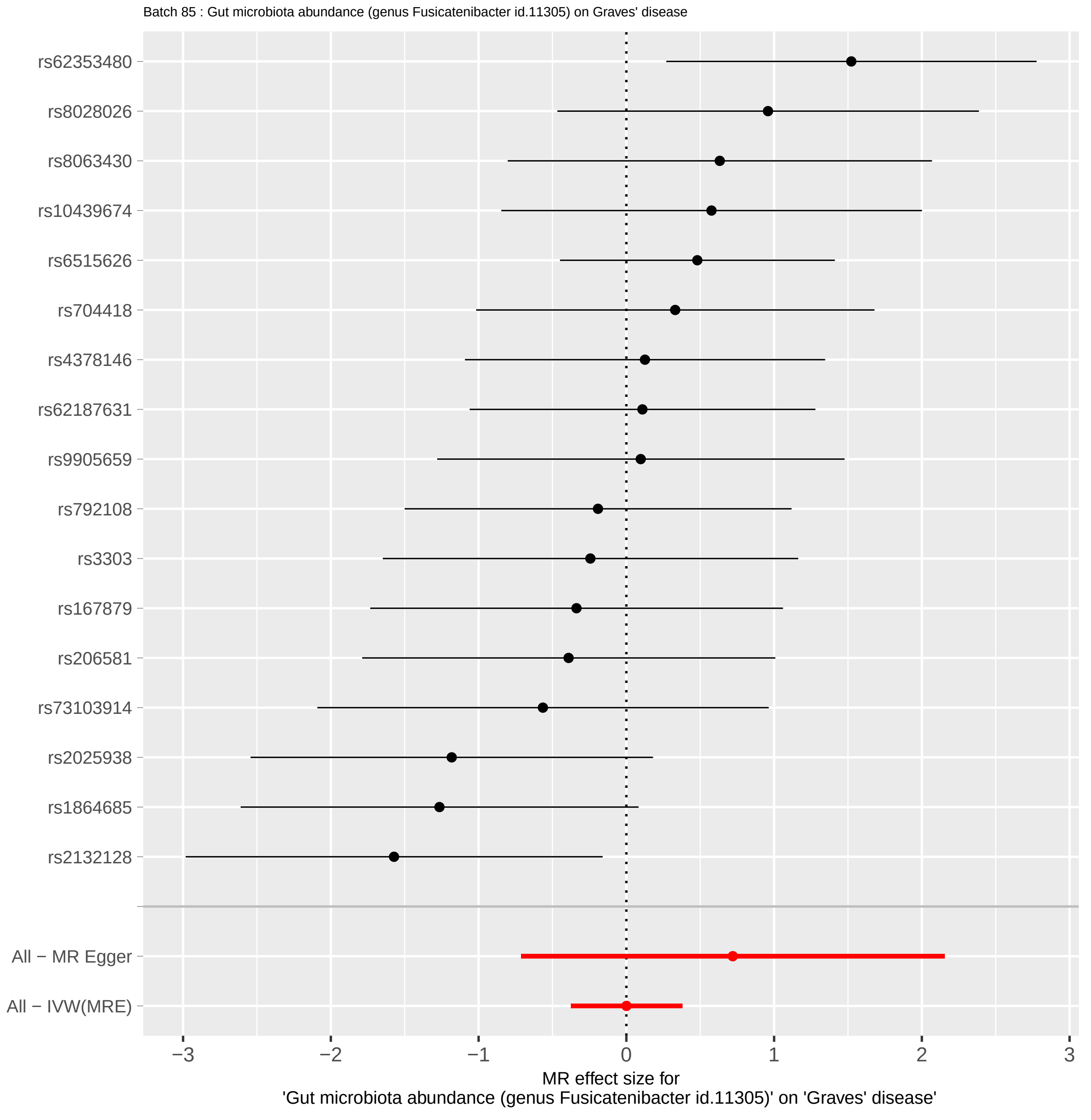
Batch 85 : Gut microbiota abundance (genus Fusicatenibacter id.11305) on Graves' disease
rs62353480
rs8028026
rs8063430
rs10439674
rs6515626
rs704418
rs4378146
rs62187631
rs9905659
rs792108
rs3303
rs167879
rs206581
rs73103914
rs2025938
rs1864685
rs2132128
All − MR Egger
All − IVW(MRE)
−3
−2
−1
0
1
2
3
MR effect size for
'Gut microbiota abundance (genus Fusicatenibacter id.11305)' on 'Graves' disease'

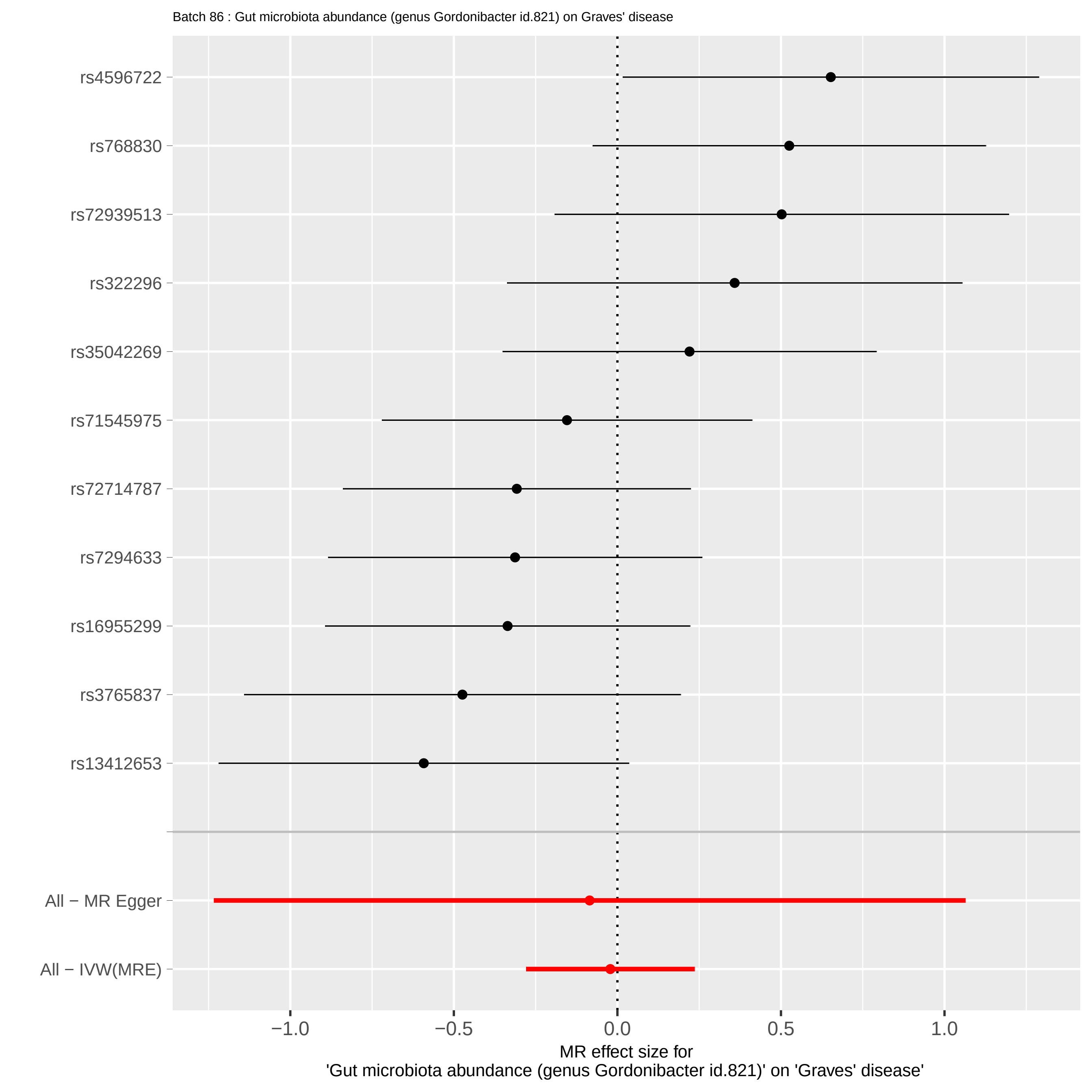
Batch 86 : Gut microbiota abundance (genus Gordonibacter id.821) on Graves' disease
rs4596722
rs768830
rs72939513
rs322296
rs35042269
rs71545975
rs72714787
rs7294633
rs16955299
rs3765837
rs13412653
All − MR Egger
All − IVW(MRE)
−1.0
−0.5
0.0
0.5
1.0
MR effect size for
'Gut microbiota abundance (genus Gordonibacter id.821)' on 'Graves' disease'

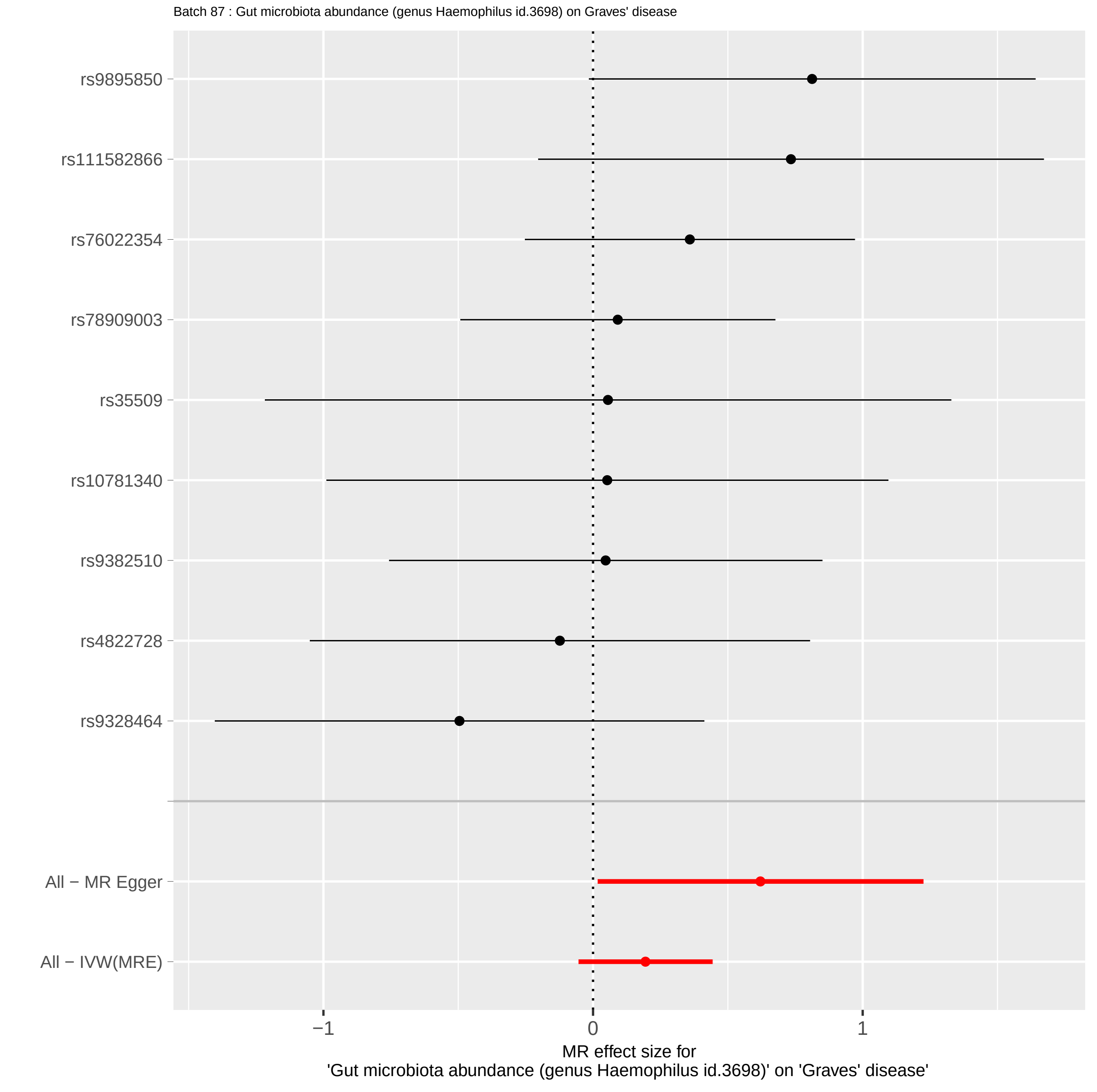
Batch 87 : Gut microbiota abundance (genus Haemophilus id.3698) on Graves' disease
rs9895850
rs111582866
rs76022354
rs78909003
rs35509
rs10781340
rs9382510
rs4822728
rs9328464
All − MR Egger
All − IVW(MRE)
−1
0
1
MR effect size for
'Gut microbiota abundance (genus Haemophilus id.3698)' on 'Graves' disease'

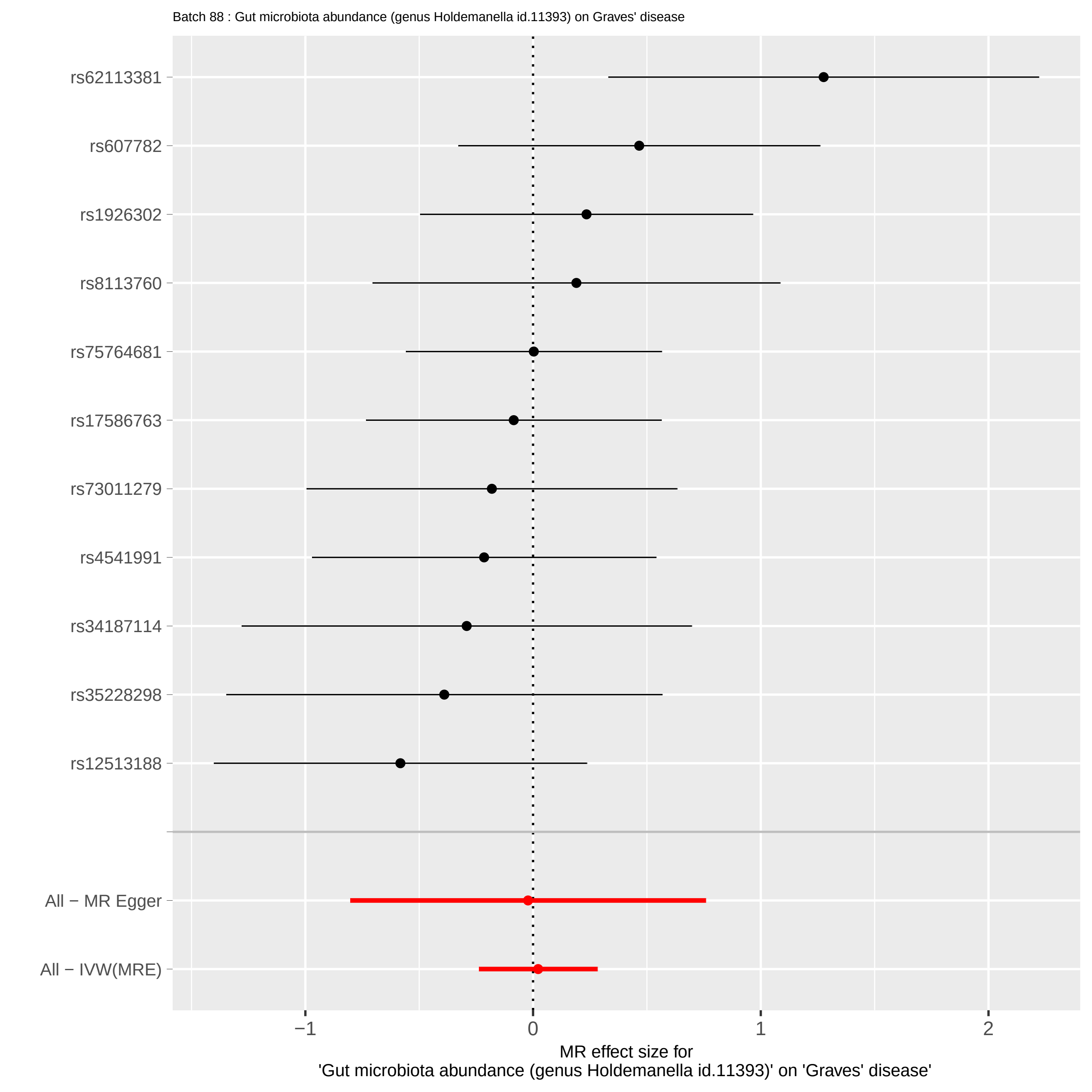
Batch 88 : Gut microbiota abundance (genus Holdemanella id.11393) on Graves' disease
rs62113381
rs607782
rs1926302
rs8113760
rs75764681
rs17586763
rs73011279
rs4541991
rs34187114
rs35228298
rs12513188
All − MR Egger
All − IVW(MRE)
−1
0
1
2
MR effect size for
'Gut microbiota abundance (genus Holdemanella id.11393)' on 'Graves' disease'

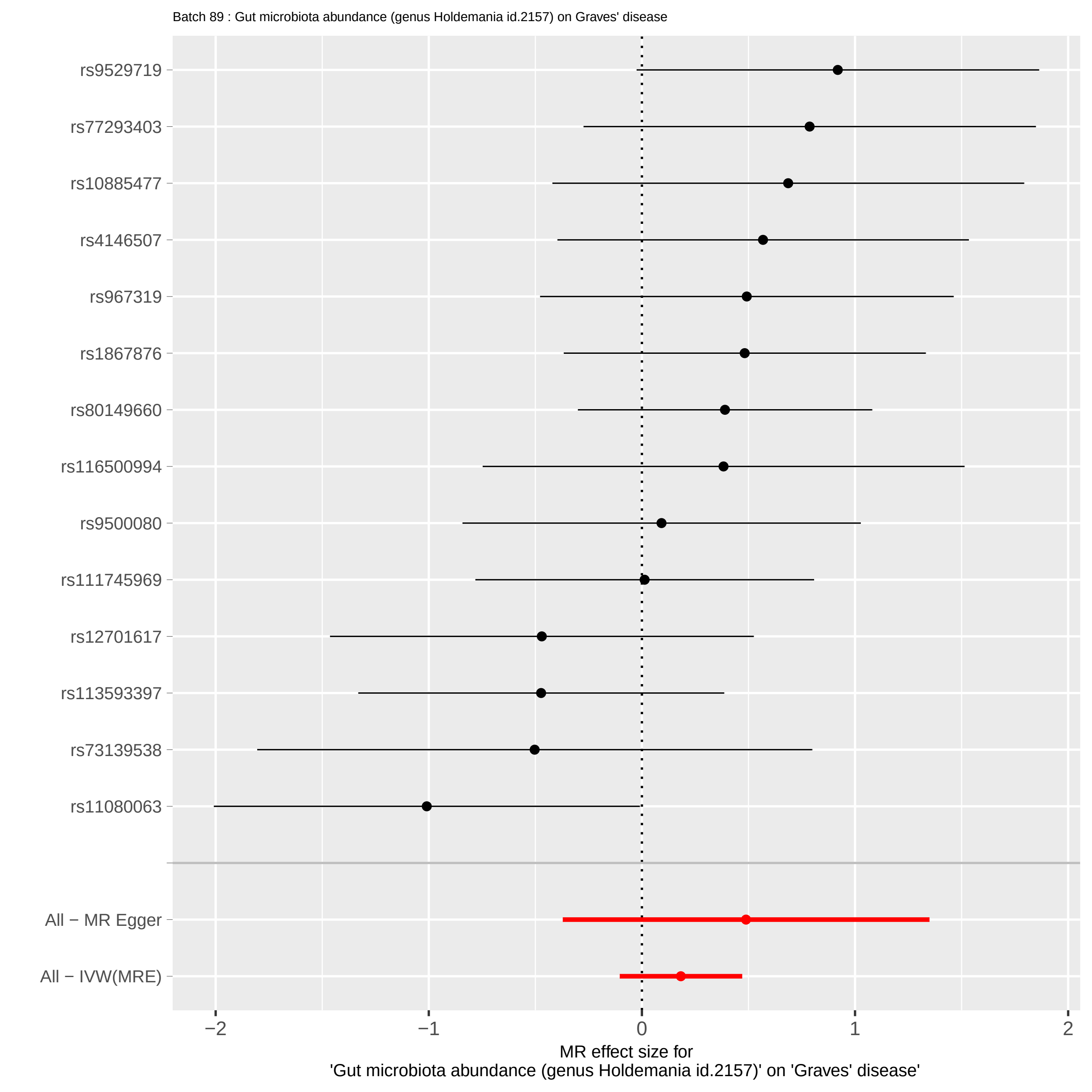

Batch 89 : Gut microbiota abundance (genus Holdemania id.2157) on Graves' disease
rs9529719
rs77293403
rs10885477
rs4146507
rs967319
rs1867876
rs80149660
rs116500994
rs9500080
rs111745969
rs12701617
rs113593397
rs73139538
rs11080063
All − MR Egger
All − IVW(MRE)
−2
−1
0
1
2
MR effect size for
'Gut microbiota abundance (genus Holdemania id.2157)' on 'Graves' disease

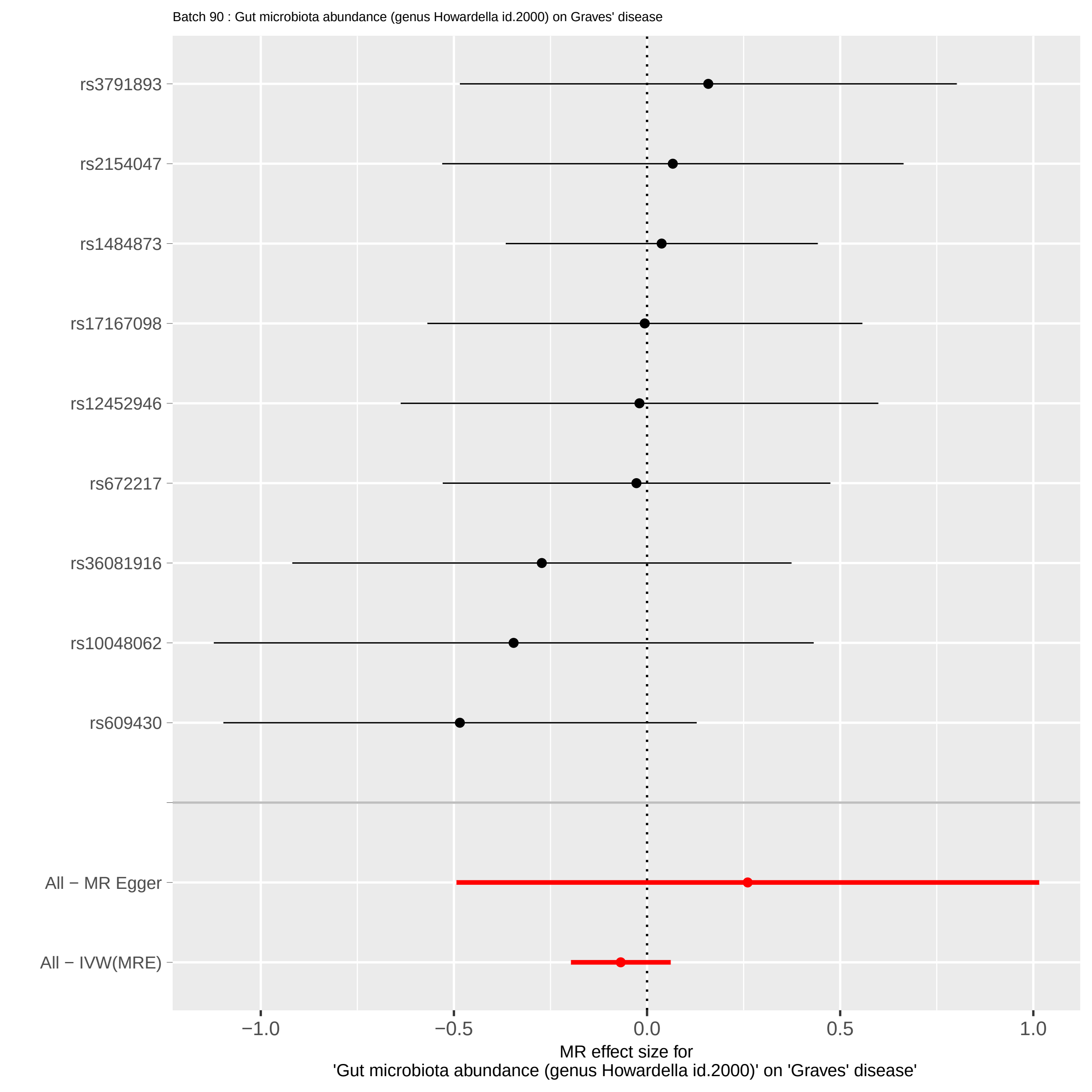
Batch 90 : Gut microbiota abundance (genus Howardella id.2000) on Graves' disease
rs3791893
rs2154047
rs1484873
rs17167098
rs12452946
rs672217
rs36081916
rs10048062
rs609430
All − MR Egger
All − IVW(MRE)
−1.0
−0.5
0.0
0.5
1.0
MR effect size for
'Gut microbiota abundance (genus Howardella id.2000)' on 'Graves' disease

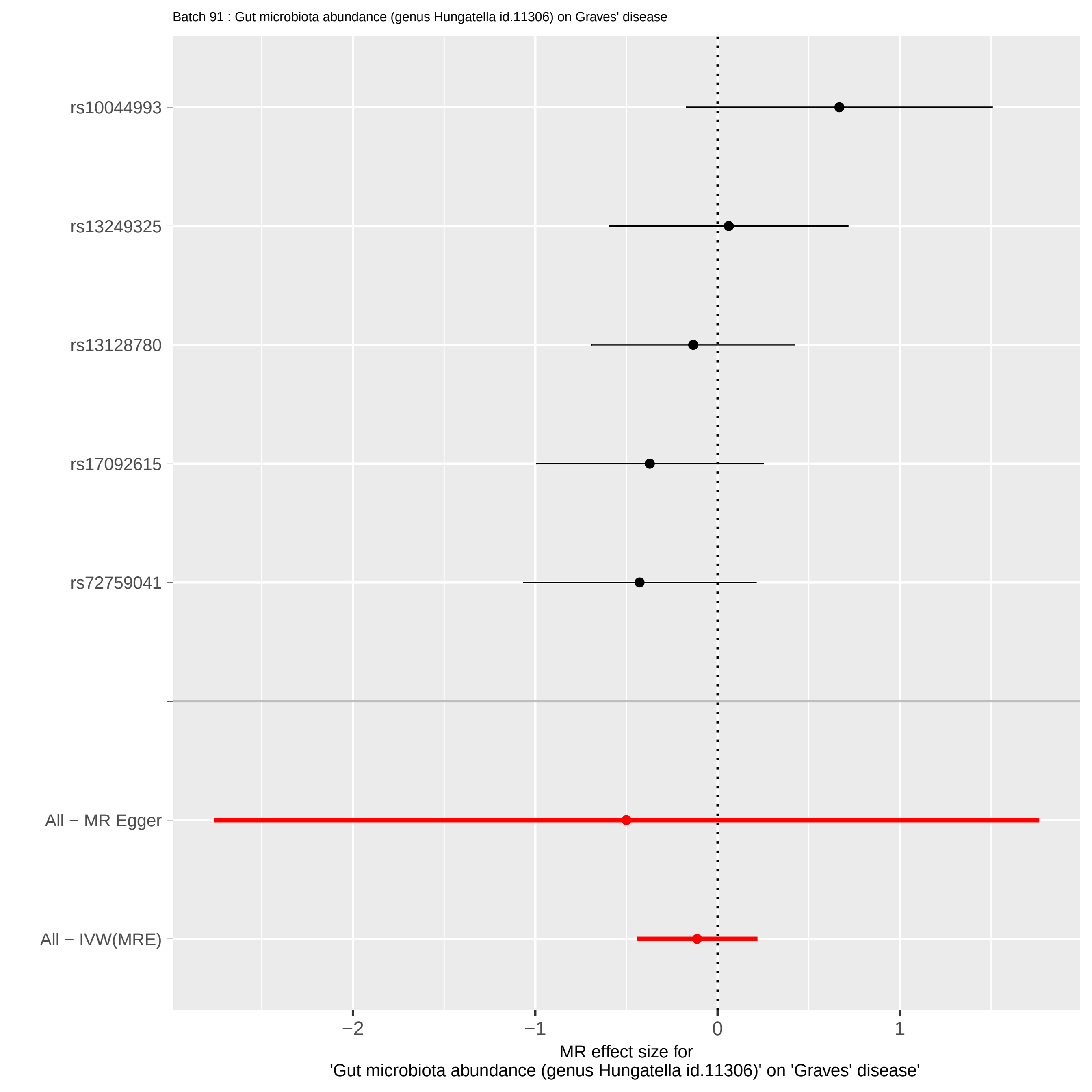

Batch 91 : Gut microbiota abundance (genus Hungatella id.11306) on Graves' disease
rs10044993
rs13249325
rs13128780
rs17092615
rs72759041
All − MR Egger
All − IVW(MRE)
−2
−1
0
1
MR effect size for
'Gut microbiota abundance (genus Hungatella id.11306)' on 'Graves' disease'

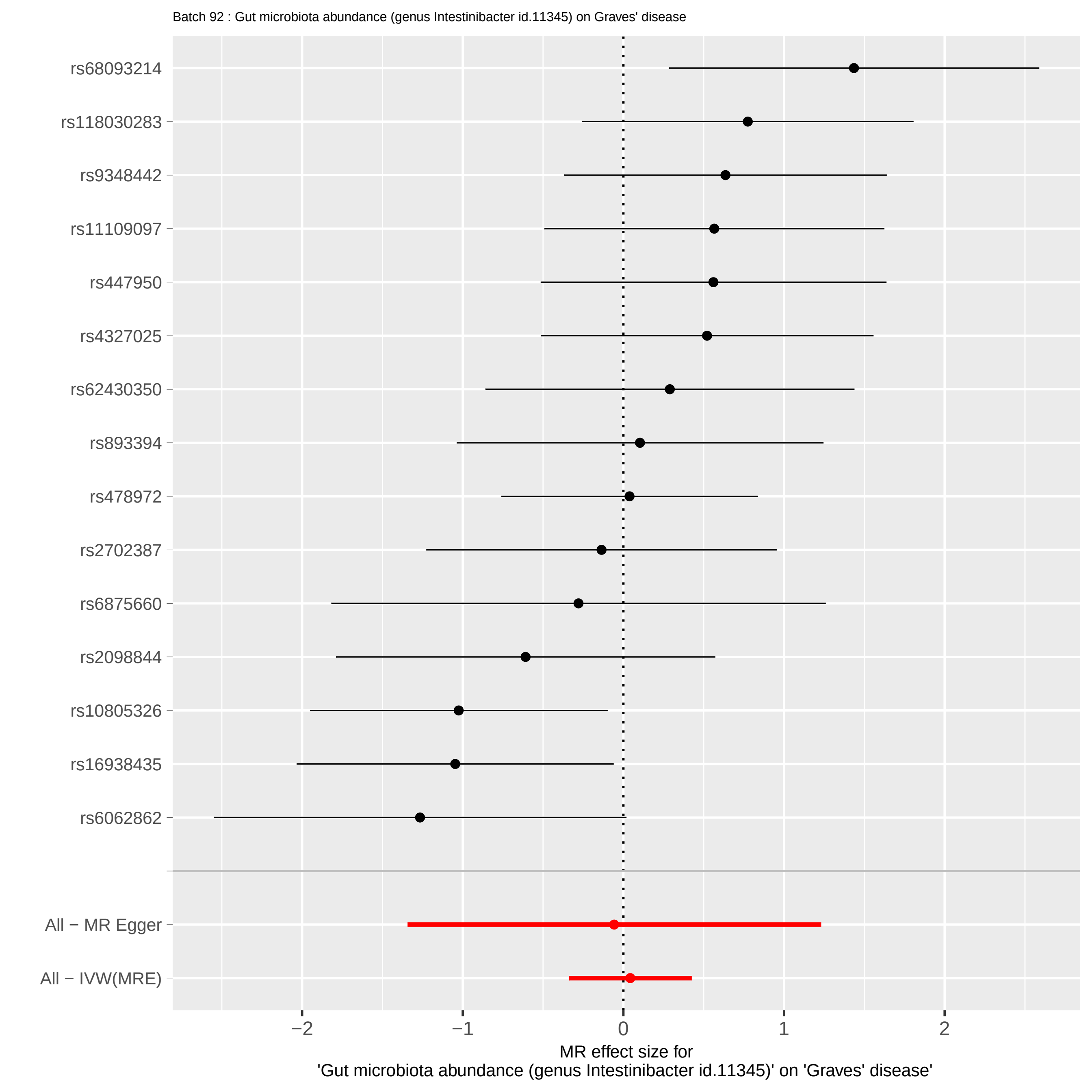
Batch 92 : Gut microbiota abundance (genus Intestinibacter id.11345) on Graves' disease
rs68093214
rs118030283
rs9348442
rs11109097
rs447950
rs4327025
rs62430350
rs893394
rs478972
rs2702387
rs6875660
rs2098844
rs10805326
rs16938435
rs6062862
All − MR Egger
All − IVW(MRE)
−2
−1
0
1
2
MR effect size for
'Gut microbiota abundance (genus Intestinibacter id.11345)' on 'Graves' disease

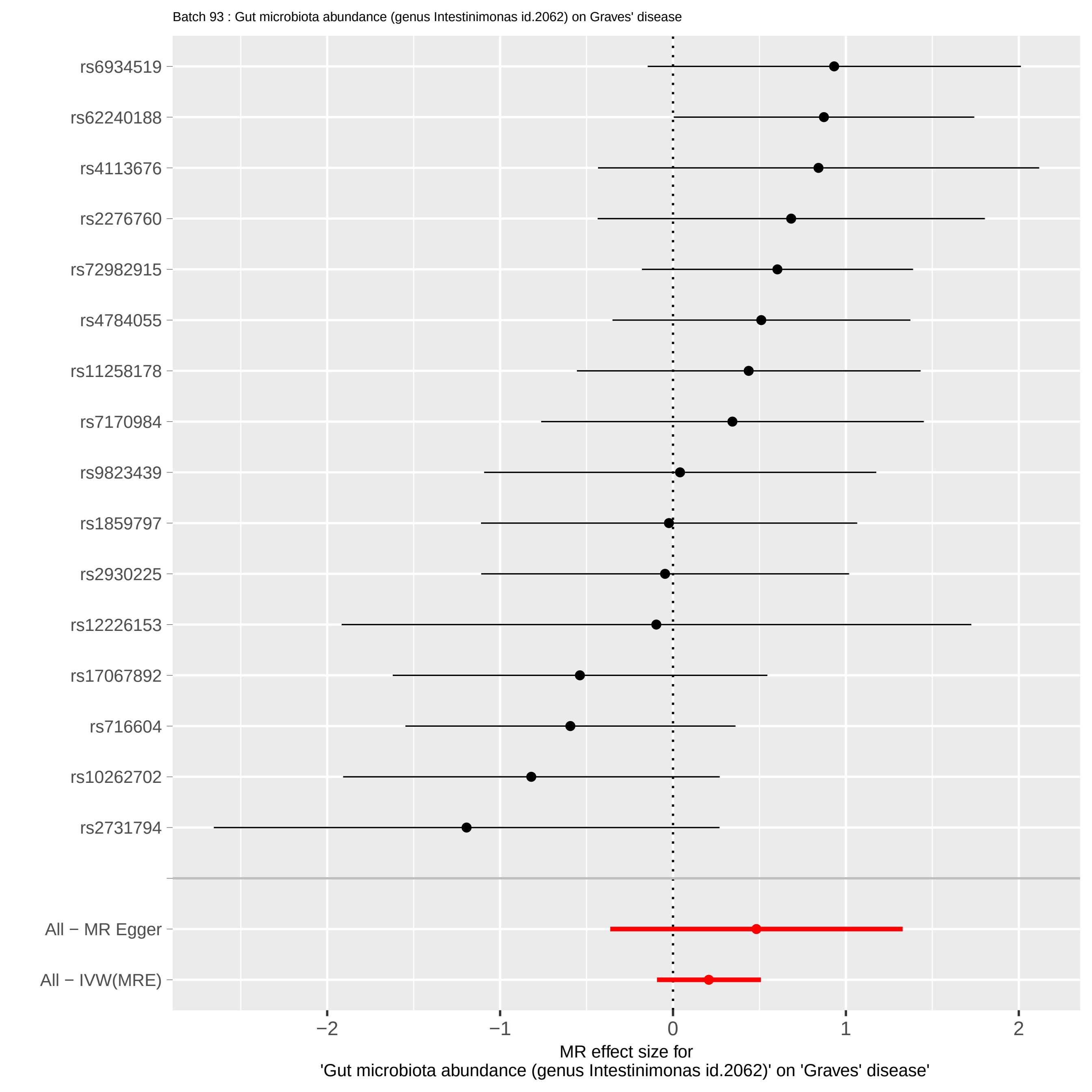

Batch 93 : Gut microbiota abundance (genus Intestinimonas id.2062) on Graves' disease
rs6934519
rs62240188
rs4113676
rs2276760
rs72982915
rs4784055
rs11258178
rs7170984
rs9823439
rs1859797
rs2930225
rs12226153
rs17067892
rs716604
rs10262702
rs2731794
All − MR Egger
All − IVW(MRE)
−2
−1
0
1
2
MR effect size for
'Gut microbiota abundance (genus Intestinimonas id.2062)' on 'Graves' disease'

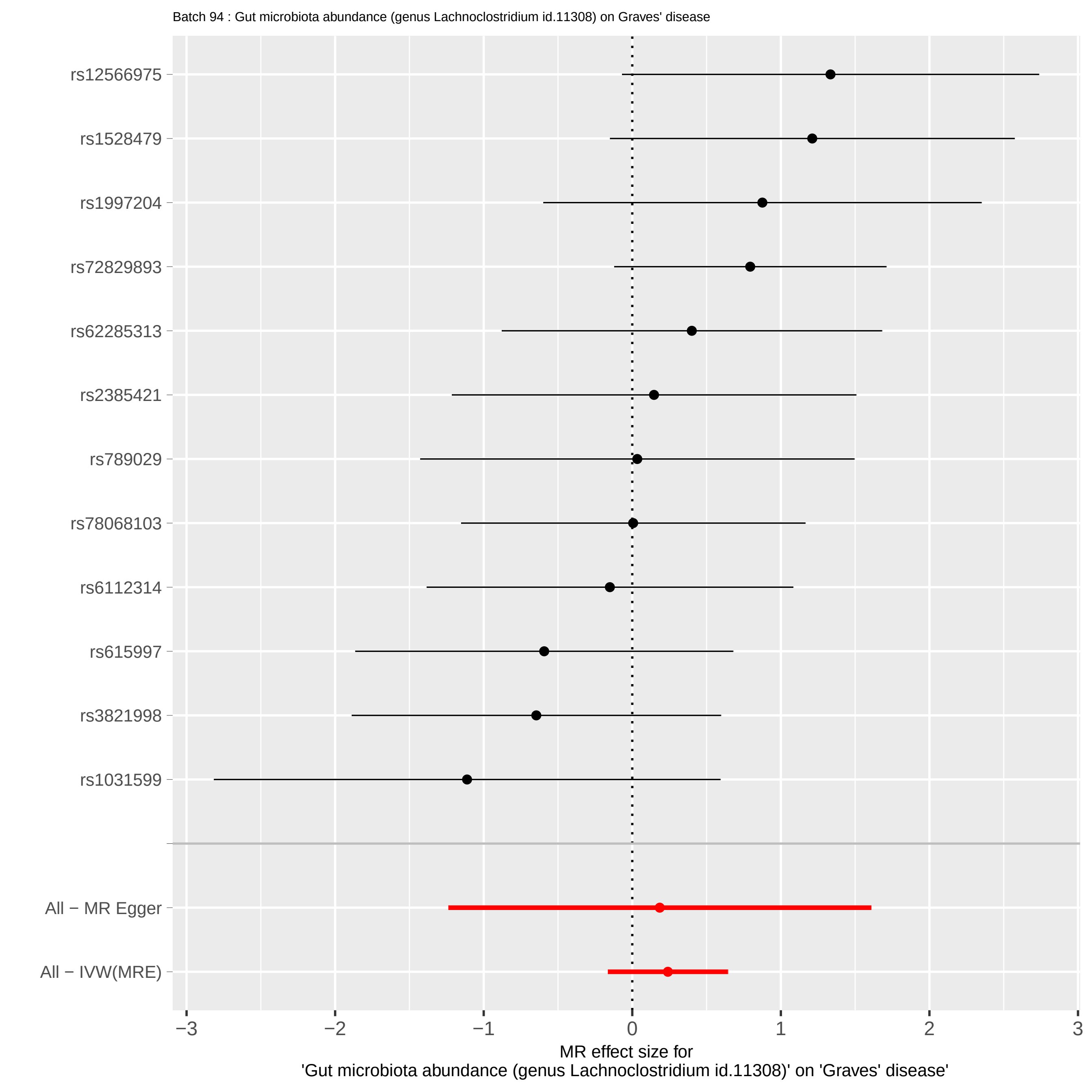

Batch 94 : Gut microbiota abundance (genus Lachnoclostridium id.11308) on Graves' disease
rs12566975
rs1528479
rs1997204
rs72829893
rs62285313
rs2385421
rs789029
rs78068103
rs6112314
rs615997
rs3821998
rs1031599
All − MR Egger
All − IVW(MRE)
−3
−2
−1
0
1
2
3
MR effect size for
'Gut microbiota abundance (genus Lachnoclostridium id.11308)' on 'Graves' disease'

Batch 95 : Gut microbiota abundance (genus Lachnospiraceae FCS020 group id.11314) on Graves' disease

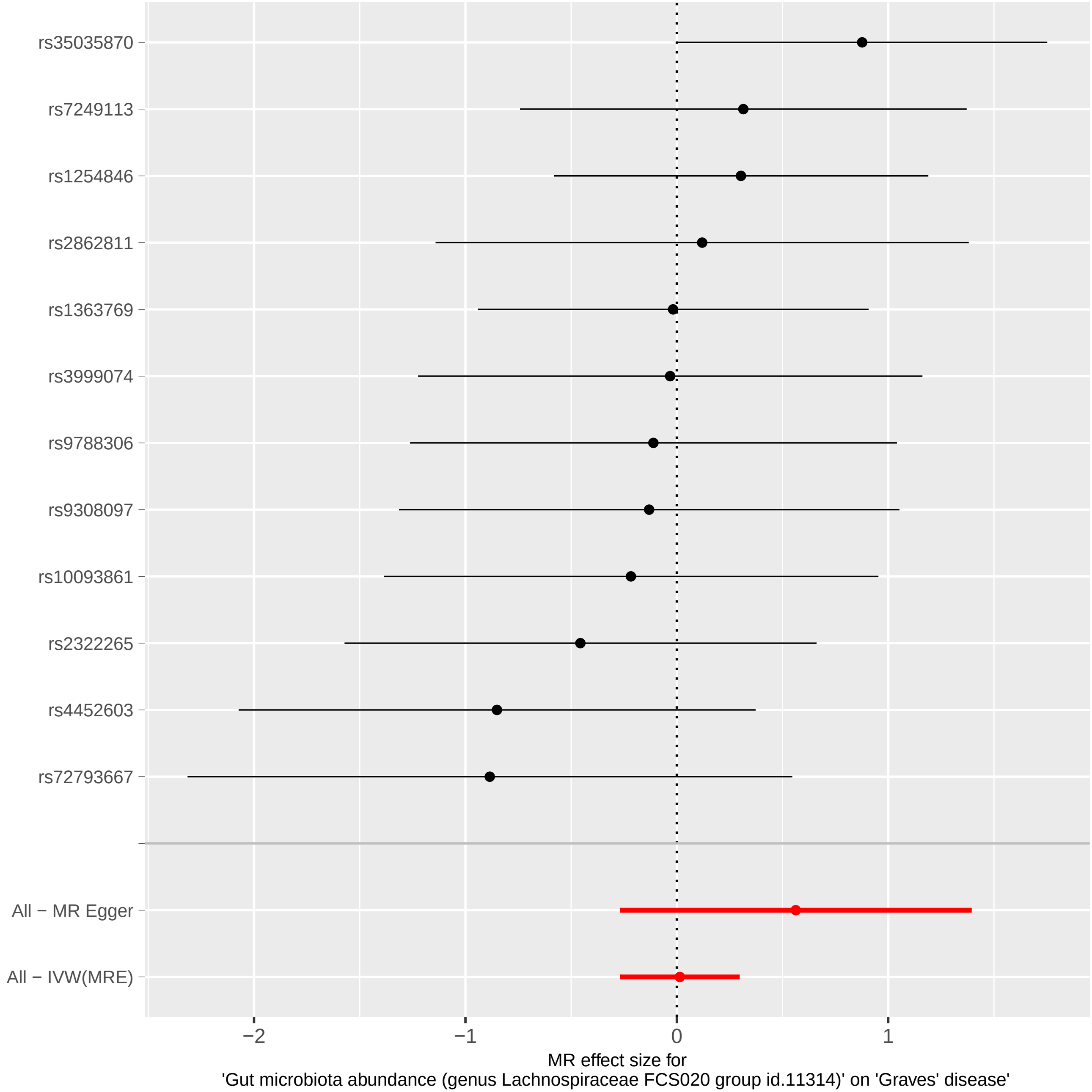

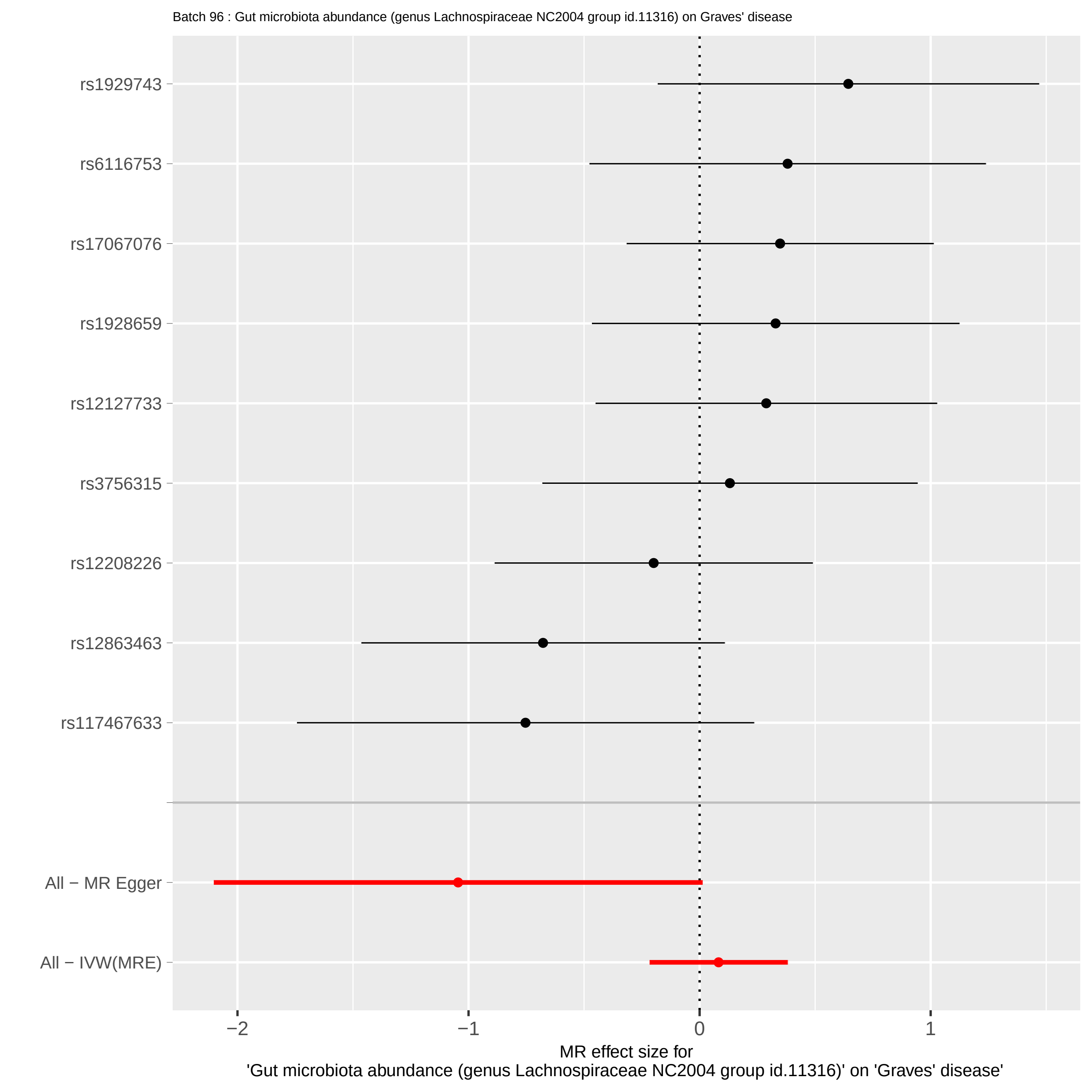

Batch 96 : Gut microbiota abundance (genus Lachnospiraceae NC2004 group id.11316) on Graves' disease
rs1929743
rs6116753
rs17067076
rs1928659
rs12127733
rs3756315
rs12208226
rs12863463
rs117467633
All − MR Egger
All − IVW(MRE)
−2
−1
0
1
MR effect size for
'Gut microbiota abundance (genus Lachnospiraceae NC2004 group id.11316)' on 'Graves' disease'

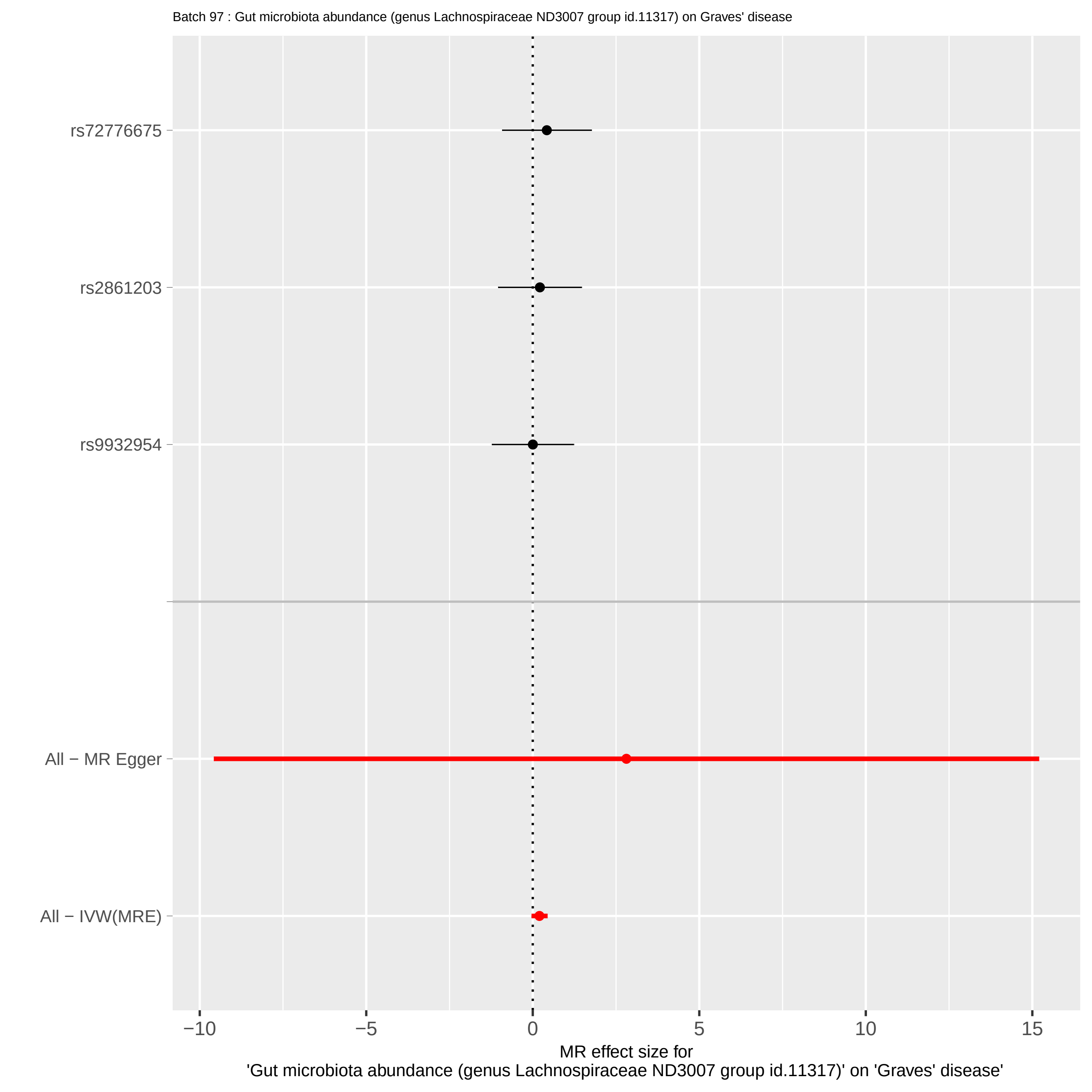

Batch 97 : Gut microbiota abundance (genus Lachnospiraceae ND3007 group id.11317) on Graves' disease
rs72776675
rs2861203
rs9932954
All − MR Egger
All − IVW(MRE)
−10
−5
0
5
10
15
MR effect size for
'Gut microbiota abundance (genus Lachnospiraceae ND3007 group id.11317)' on 'Graves' disease'

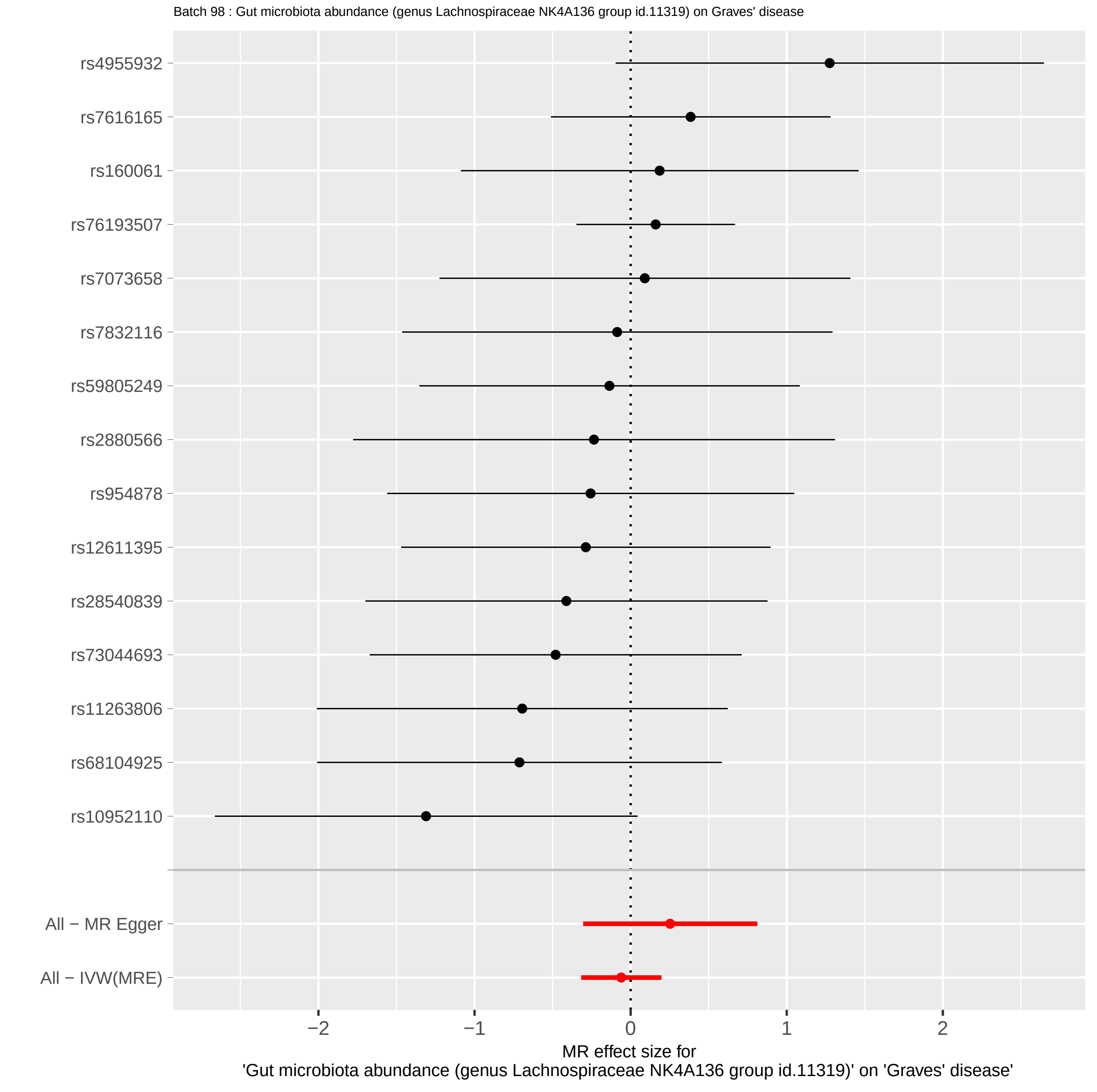

Batch 98 : Gut microbiota abundance (genus Lachnospiraceae NK4A136 group id.11319) on Graves' disease
rs4955932
rs7616165
rs160061
rs76193507
rs7073658
rs7832116
rs59805249
rs2880566
rs954878
rs12611395
rs28540839
rs73044693
rs11263806
rs68104925
rs10952110
All − MR Egger
All − IVW(MRE)
−2
−1
0
1
2
MR effect size for
'Gut microbiota abundance (genus Lachnospiraceae NK4A136 group id.11319)' on 'Graves' disease'

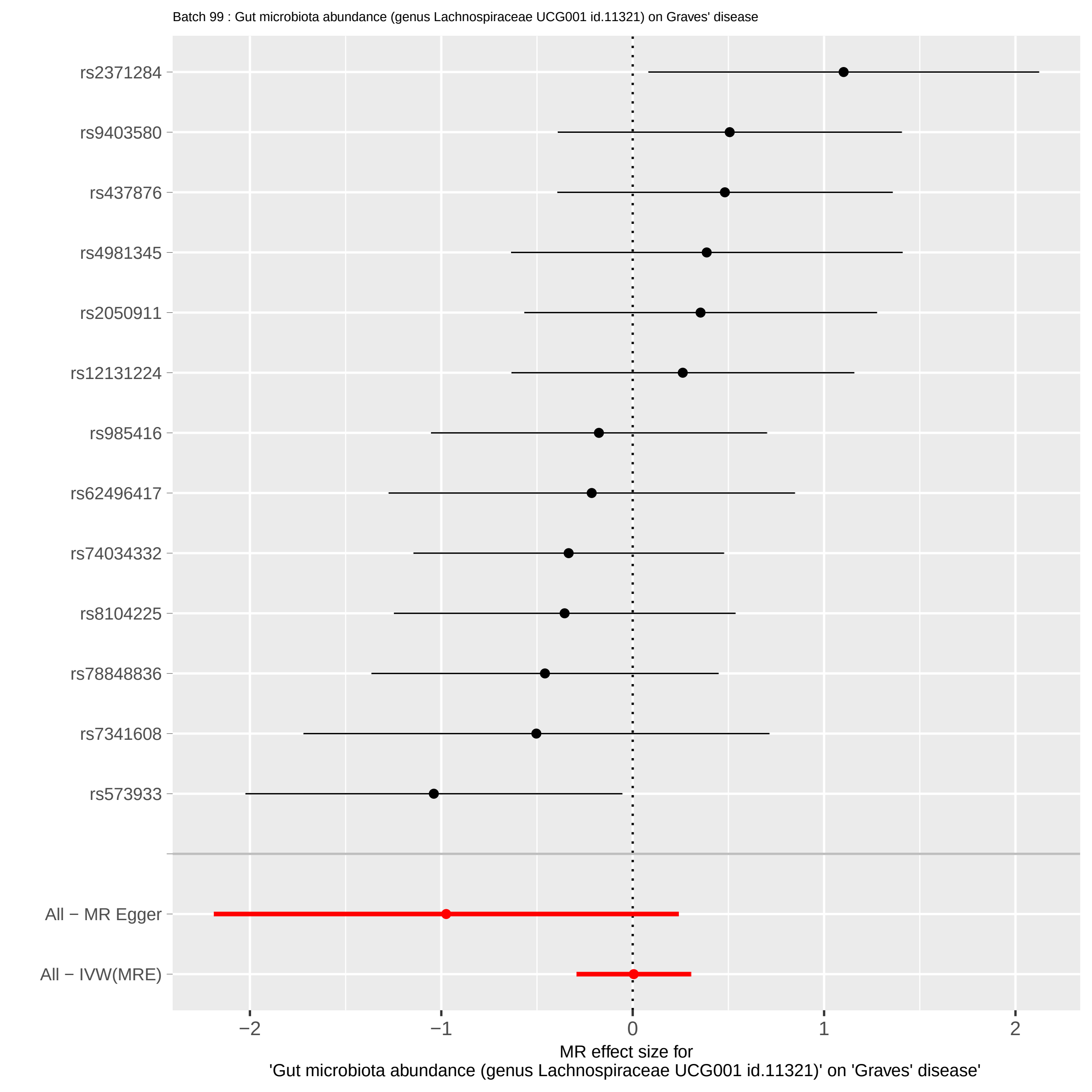

Batch 99 : Gut microbiota abundance (genus Lachnospiraceae UCG001 id.11321) on Graves' disease
rs2371284
rs9403580
rs437876
rs4981345
rs2050911
rs12131224
rs985416
rs62496417
rs74034332
rs8104225
rs78848836
rs7341608
rs573933
All − MR Egger
All − IVW(MRE)
−2
−1
0
1
2
MR effect size for
'Gut microbiota abundance (genus Lachnospiraceae UCG001 id.11321)' on 'Graves' disease'

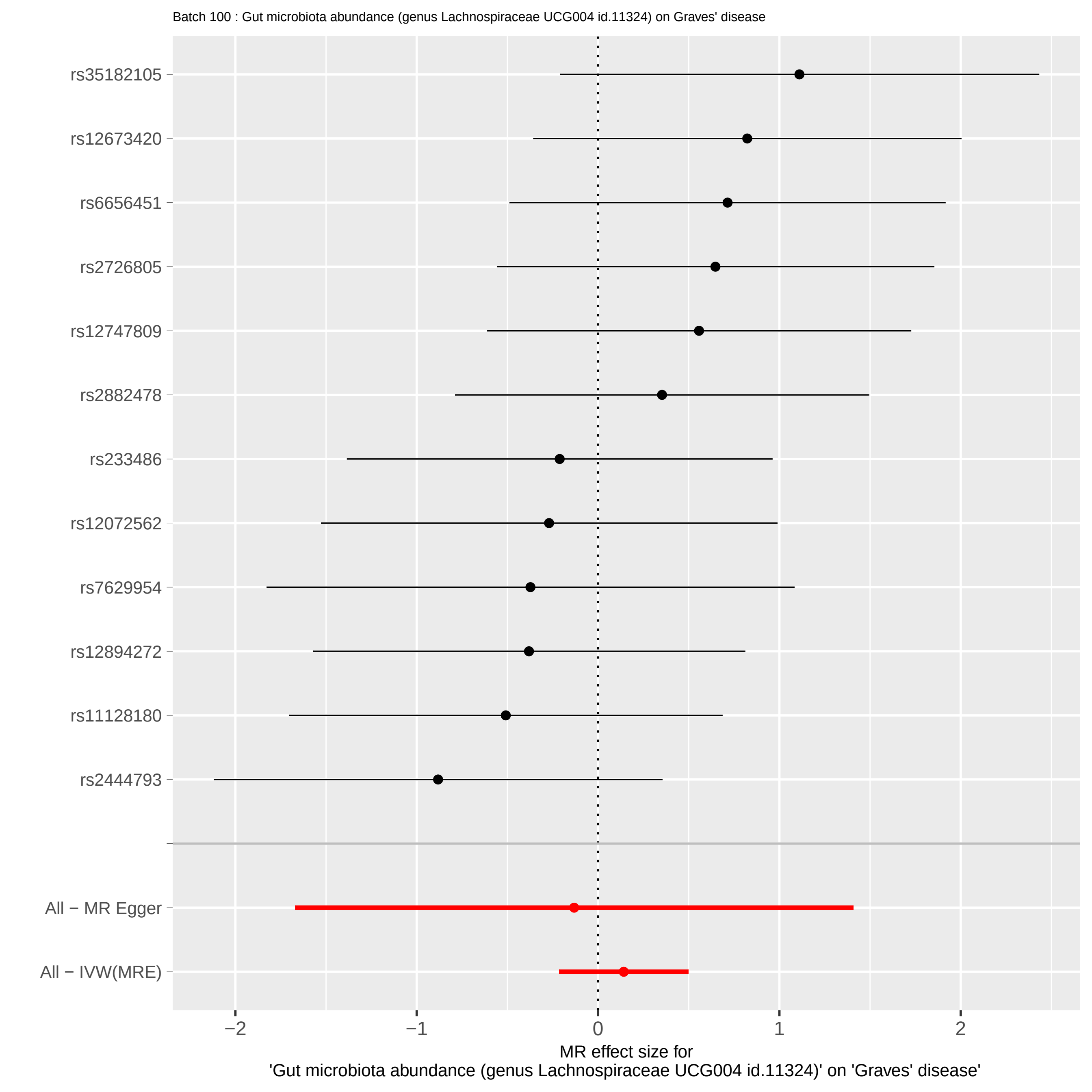

Batch 100 : Gut microbiota abundance (genus Lachnospiraceae UCG004 id.11324) on Graves' disease
rs35182105
rs12673420
rs6656451
rs2726805
rs12747809
rs2882478
rs233486
rs12072562
rs7629954
rs12894272
rs11128180
rs2444793
All − MR Egger
All − IVW(MRE)
−2
−1
0
1
2
MR effect size for
'Gut microbiota abundance (genus Lachnospiraceae UCG004 id.11324)' on 'Graves' disease

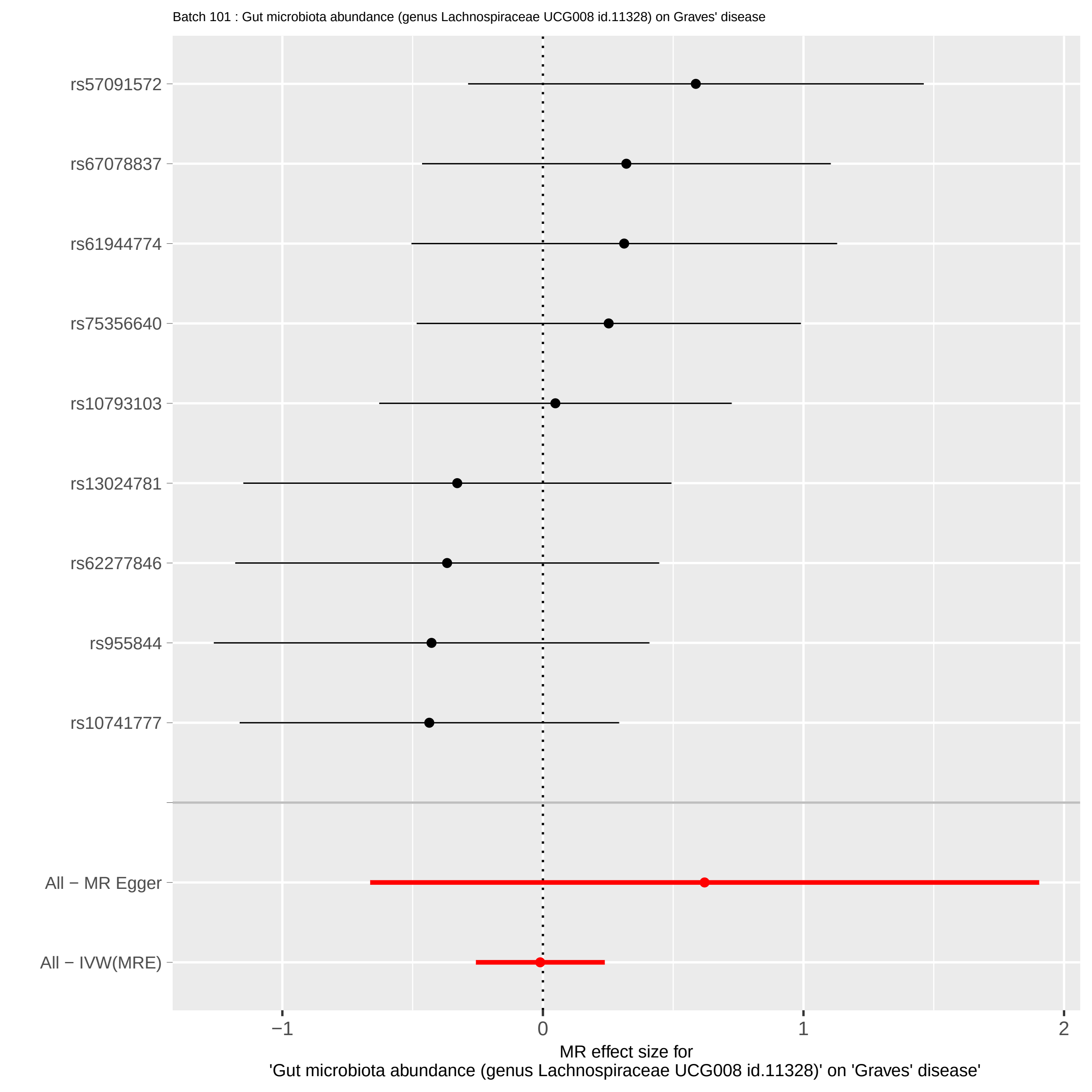
Batch 101 : Gut microbiota abundance (genus Lachnospiraceae UCG008 id.11328) on Graves' disease
rs57091572
rs67078837
rs61944774
rs75356640
rs10793103
rs13024781
rs62277846
rs955844
rs10741777
All − MR Egger
All − IVW(MRE)
−1
0
1
2
MR effect size for
'Gut microbiota abundance (genus Lachnospiraceae UCG008 id.11328)' on 'Graves' disease'

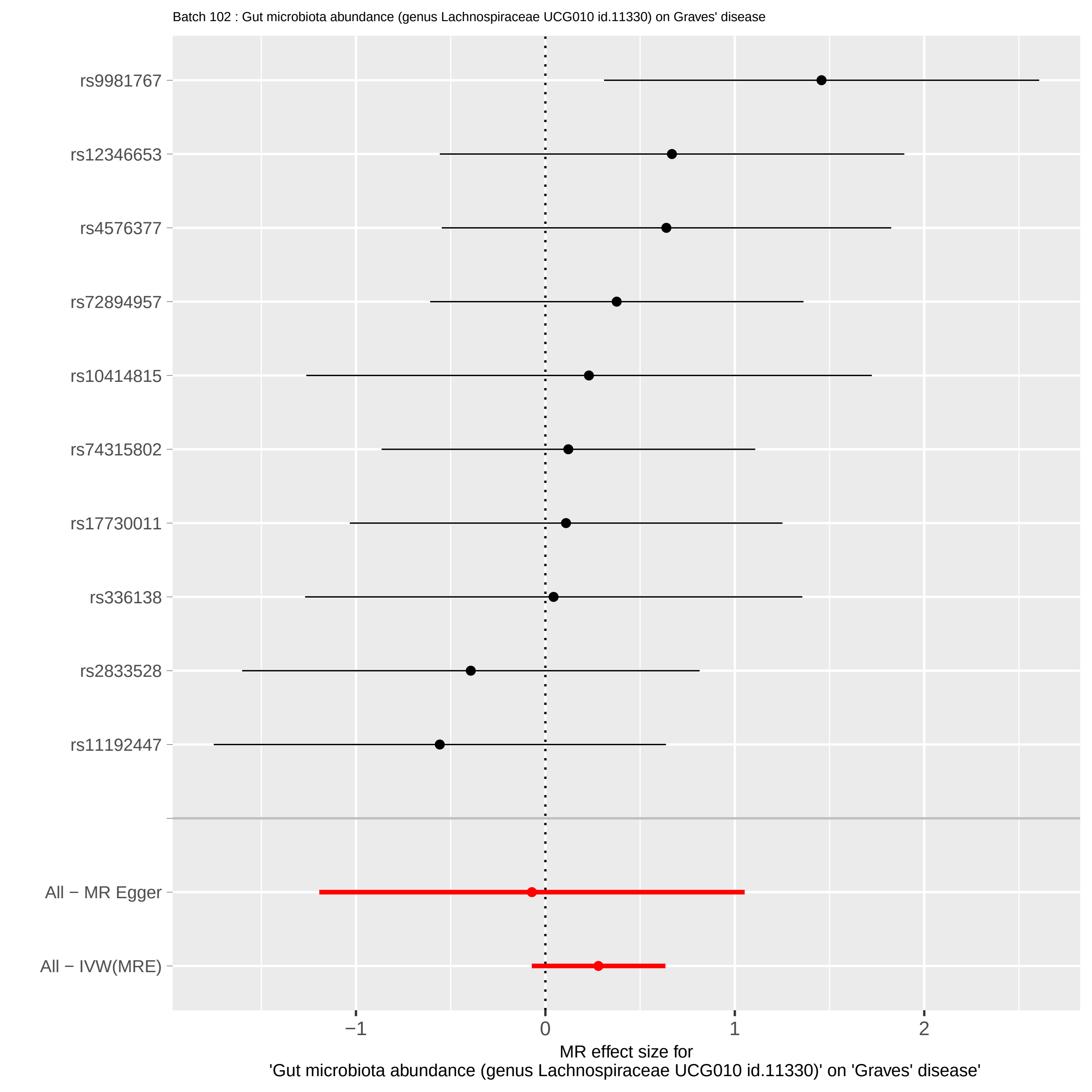

Batch 102 : Gut microbiota abundance (genus Lachnospiraceae UCG010 id.11330) on Graves' disease
rs9981767
rs12346653
rs4576377
rs72894957
rs10414815
rs74315802
rs17730011
rs336138
rs2833528
rs11192447
All − MR Egger
All − IVW(MRE)
−1
0
1
2
MR effect size for
'Gut microbiota abundance (genus Lachnospiraceae UCG010 id.11330)' on 'Graves' disease'

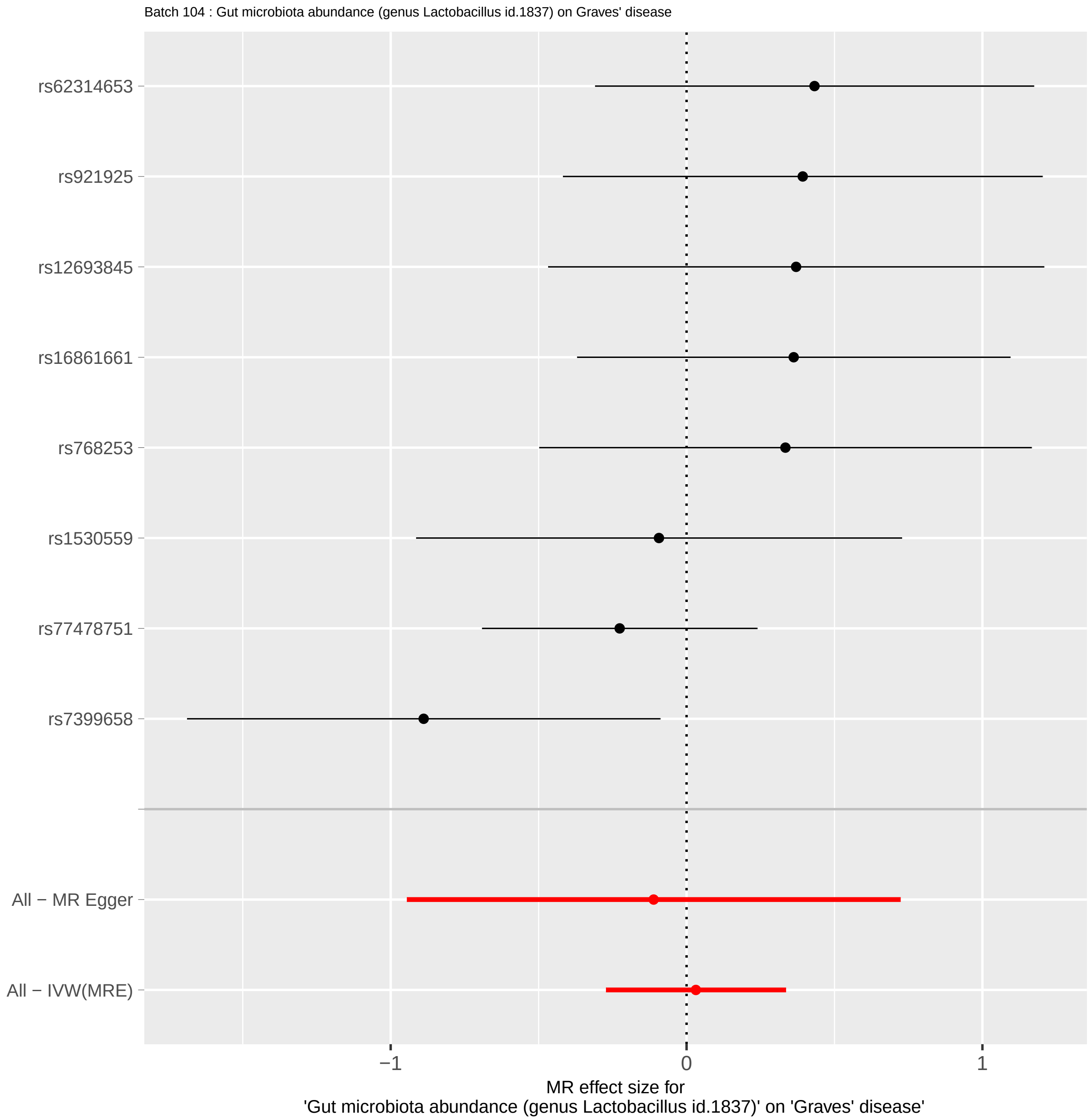
Batch 104 : Gut microbiota abundance (genus Lactobacillus id.1837) on Graves' disease
rs62314653
rs921925
rs12693845
rs16861661
rs768253
rs1530559
rs77478751
rs7399658
All − MR Egger
All − IVW(MRE)
−1
0
1
MR effect size for
'Gut microbiota abundance (genus Lactobacillus id.1837)' on 'Graves' disease'

Batch 105 : Gut microbiota abundance (genus Lactococcus id.1851) on Graves' disease

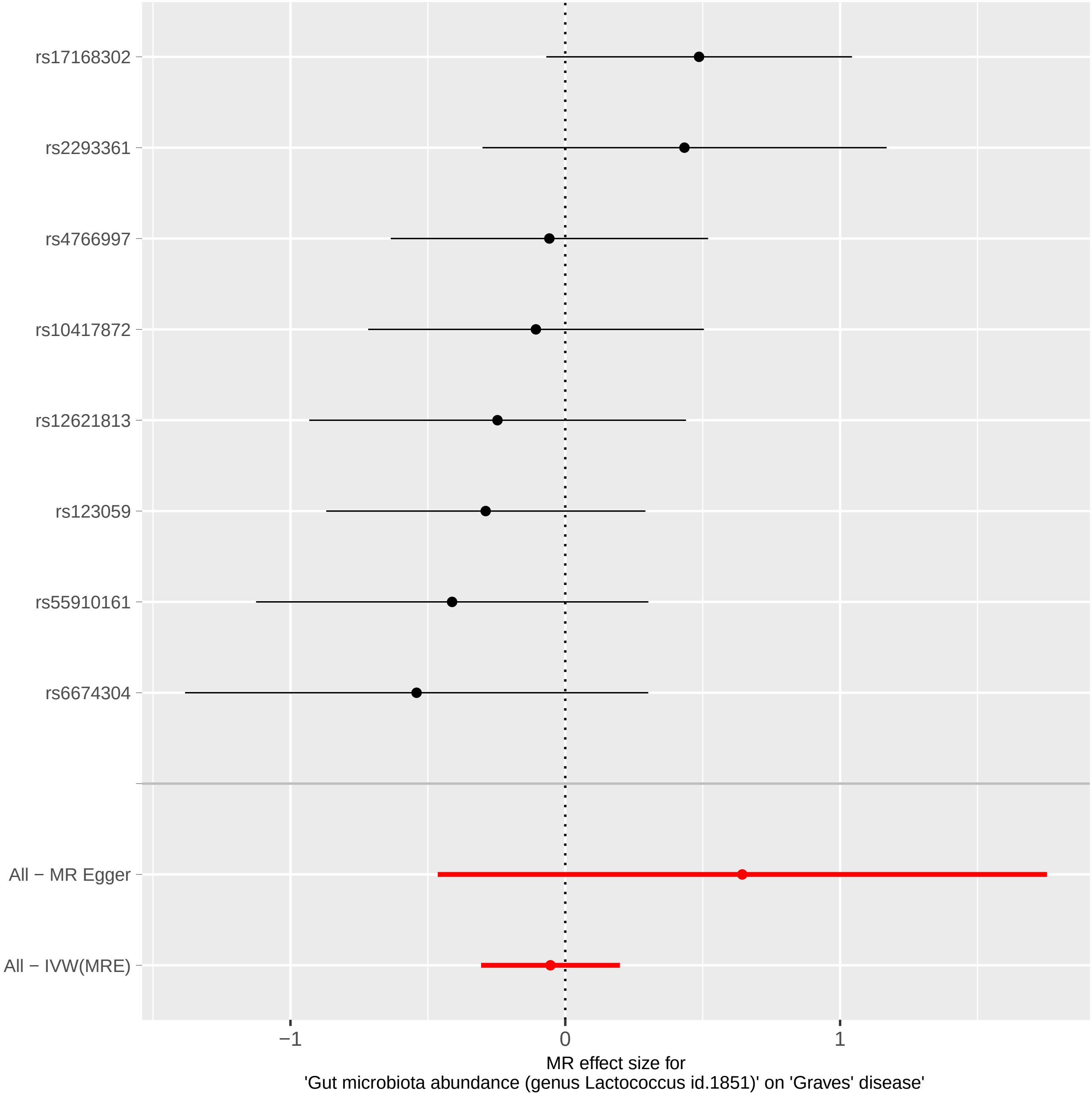

Batch 106 : Gut microbiota abundance (genus Marvinbryantia id.2005) on Graves' disease

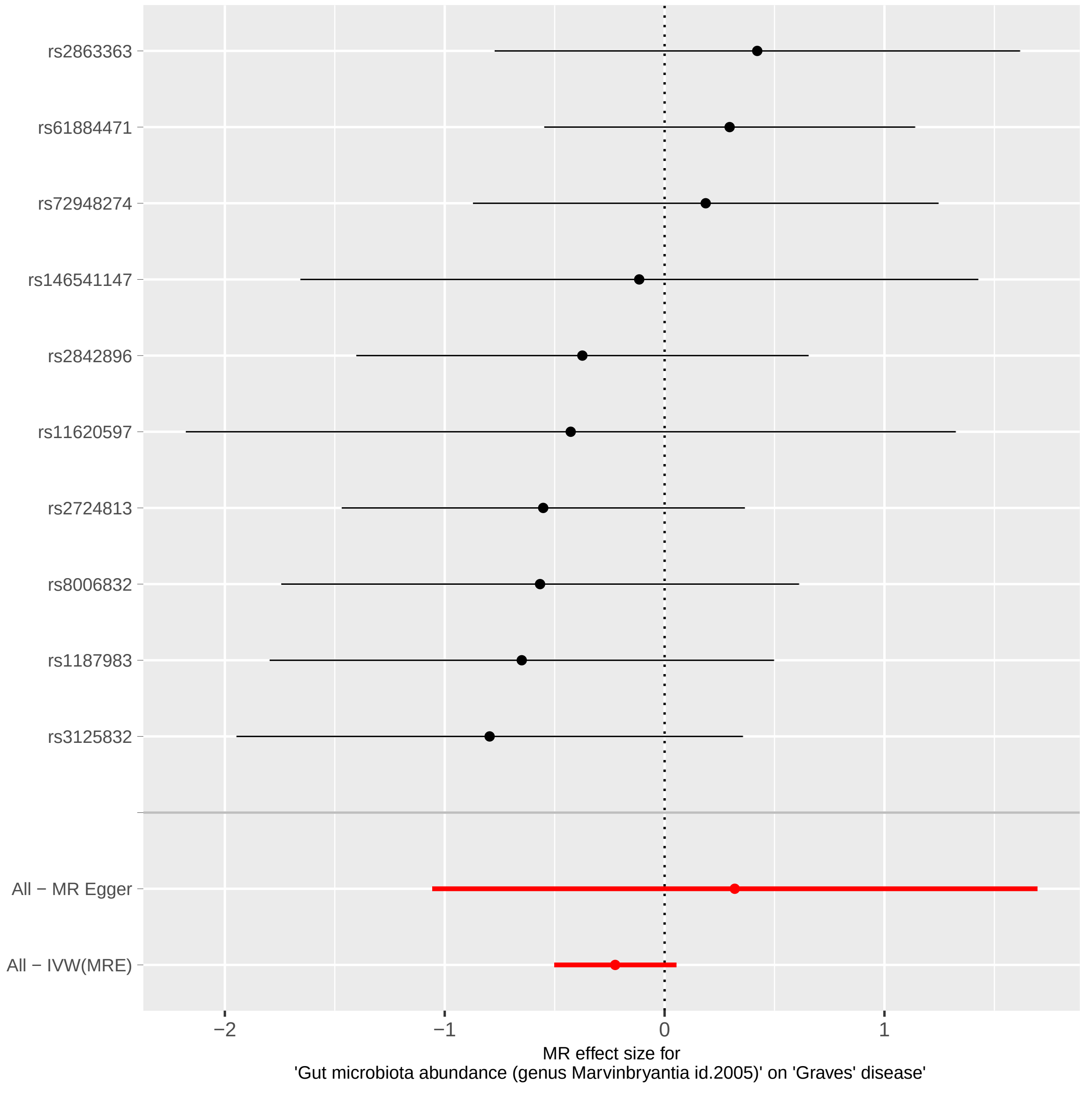

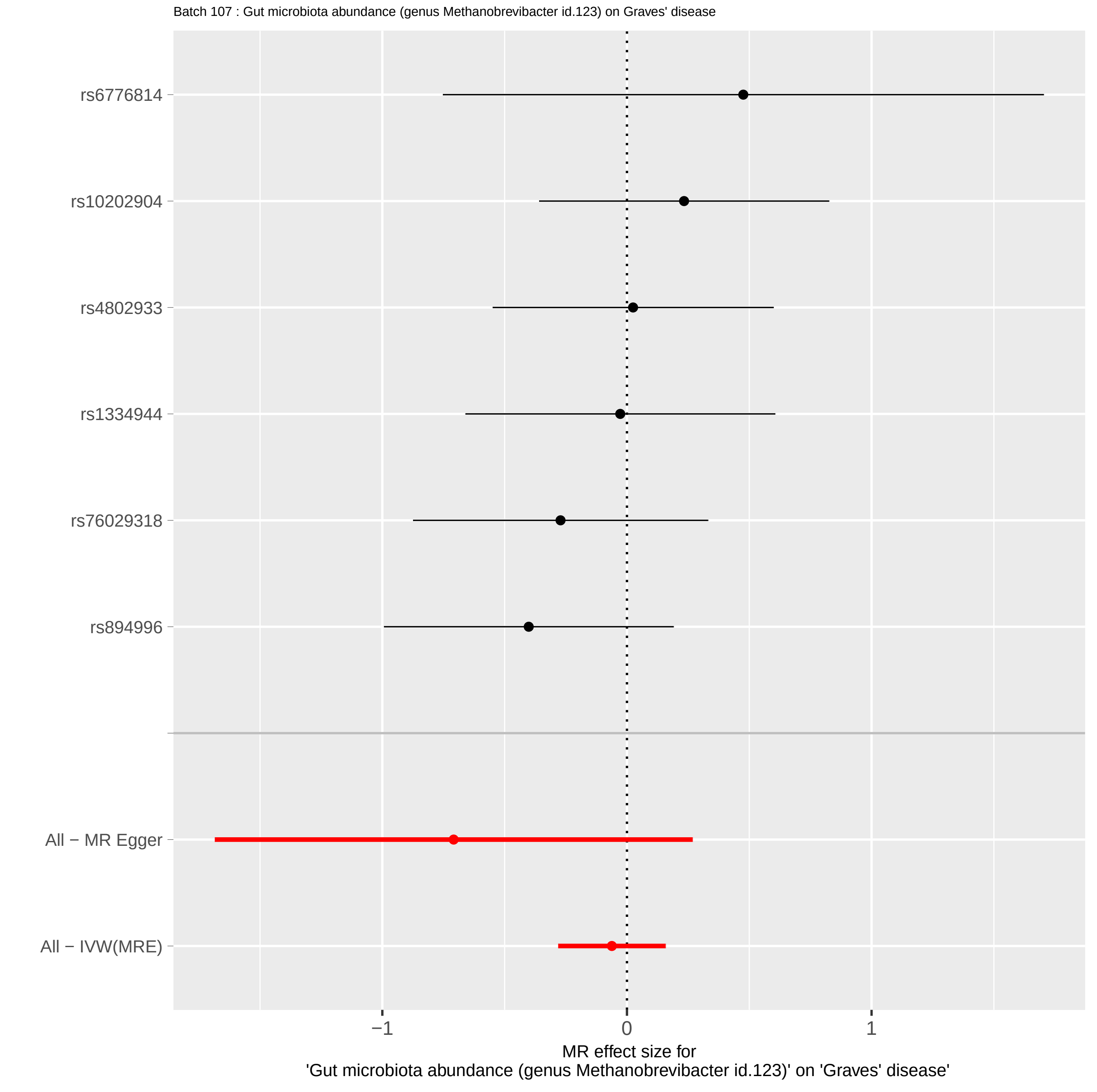
Batch 107 : Gut microbiota abundance (genus Methanobrevibacter id.123) on Graves' disease
rs6776814
rs10202904
rs4802933
rs1334944
rs76029318
rs894996
All − MR Egger
All − IVW(MRE)
−1
0
1
MR effect size for
'Gut microbiota abundance (genus Methanobrevibacter id.123)' on 'Graves' disease'

Batch 108 : Gut microbiota abundance (genus Odoribacter id.952) on Graves' disease

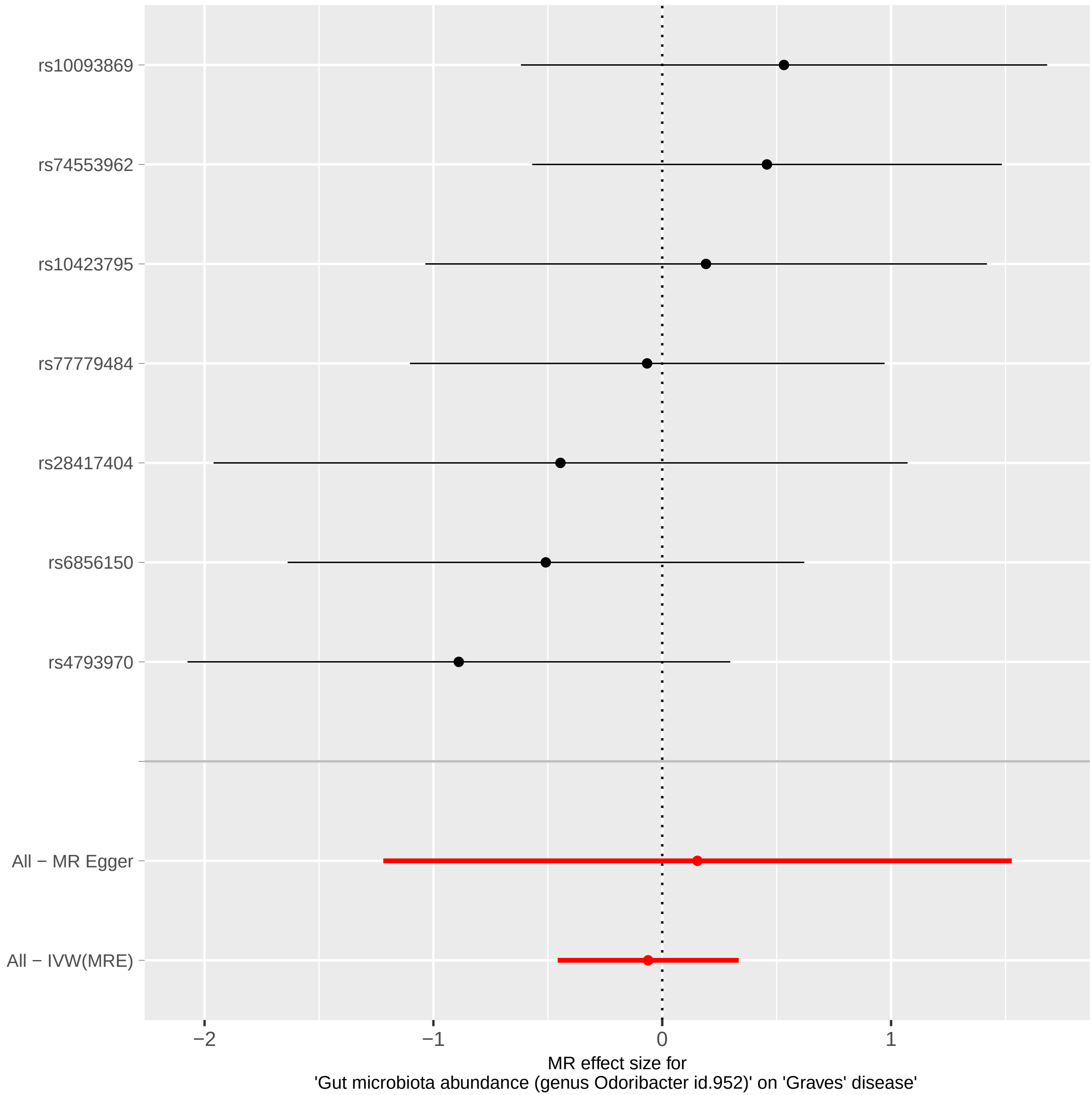

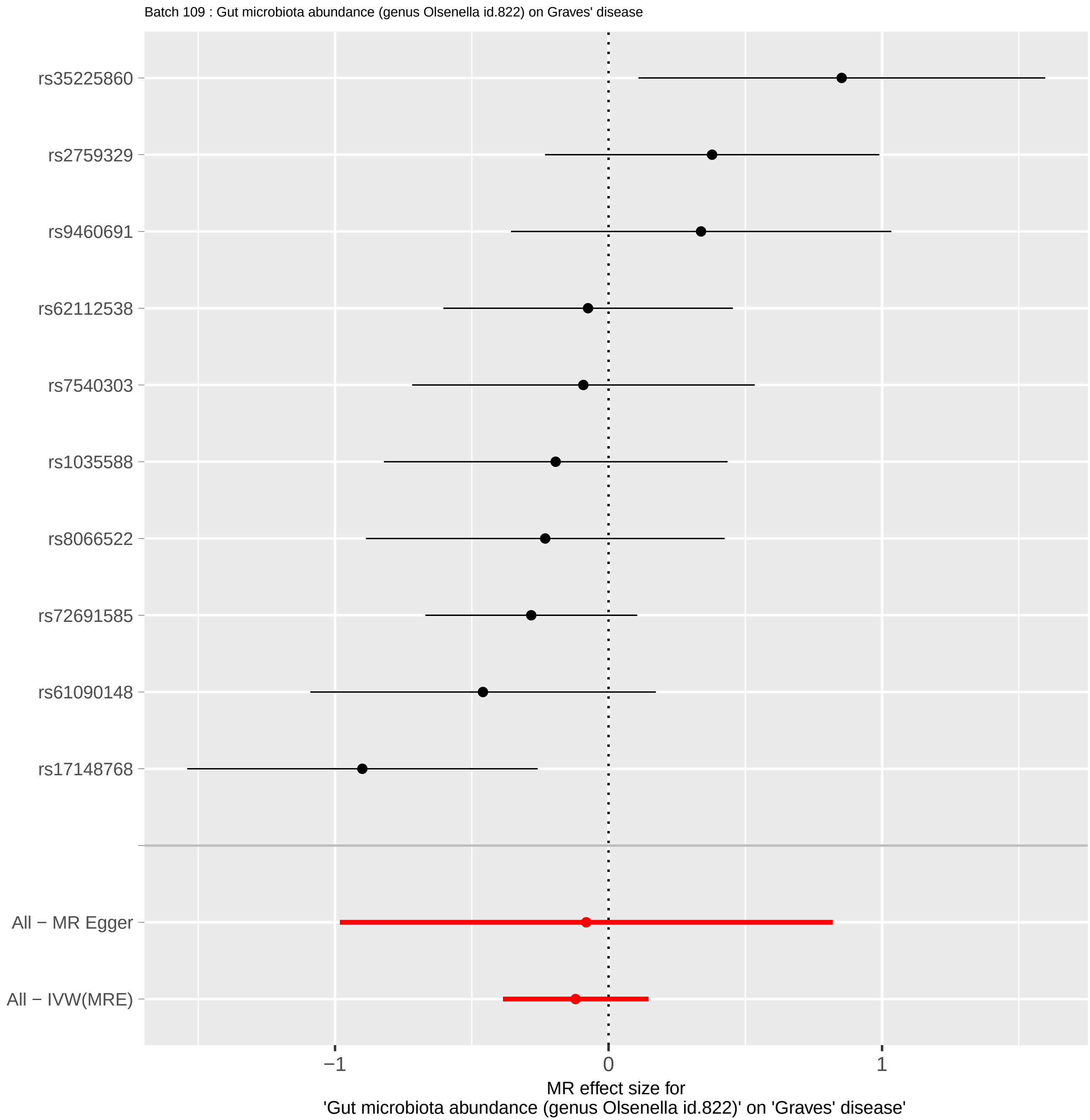

Batch 109 : Gut microbiota abundance (genus Olsenella id.822) on Graves' disease
rs35225860
rs2759329
rs9460691
rs62112538
rs7540303
rs1035588
rs8066522
rs72691585
rs61090148
rs17148768
All − MR Egger
All − IVW(MRE)
−1
0
1
MR effect size for
'Gut microbiota abundance (genus Olsenella id.822)' on 'Graves' disease'

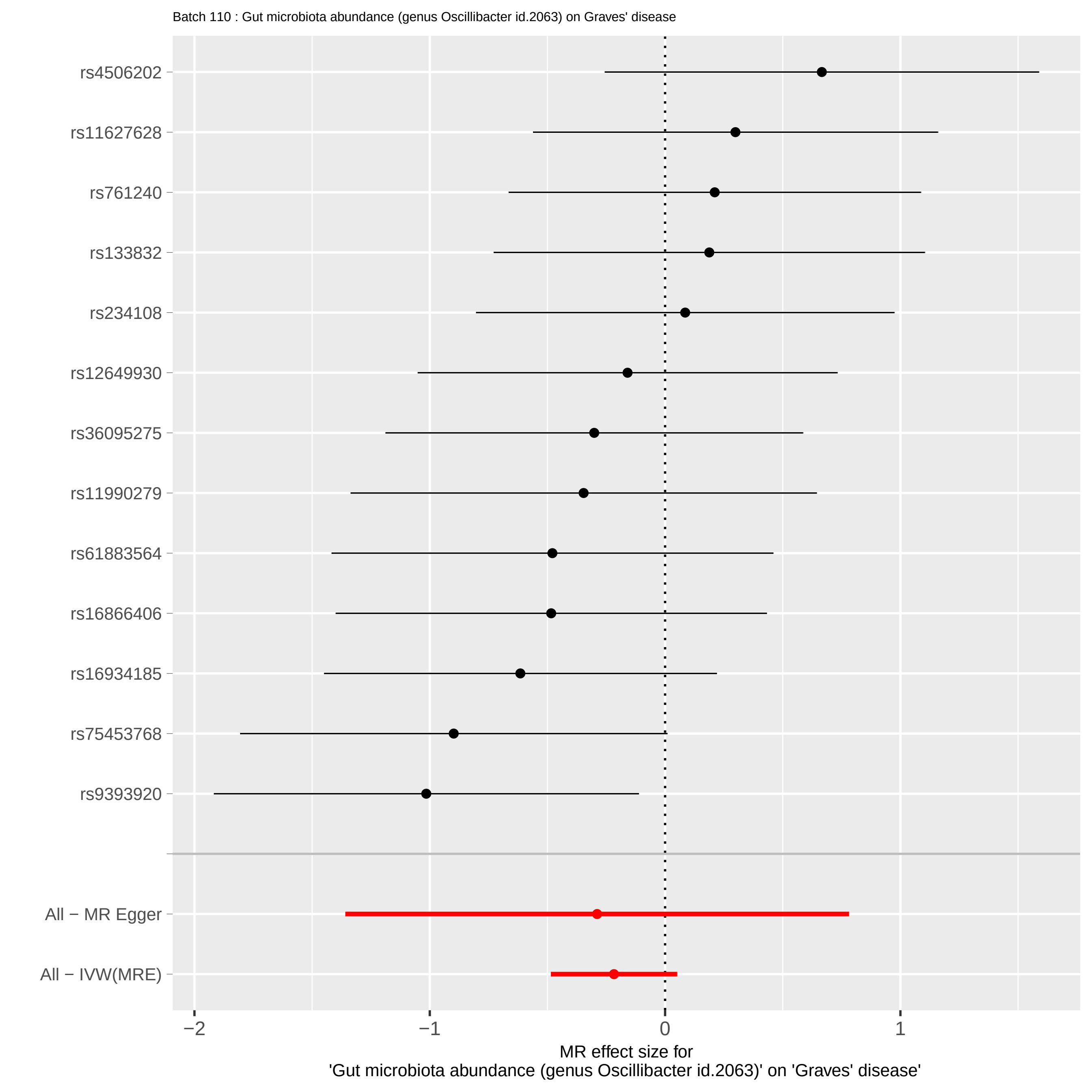
Batch 110 : Gut microbiota abundance (genus Oscillibacter id.2063) on Graves' disease
rs4506202
rs11627628
rs761240
rs133832
rs234108
rs12649930
rs36095275
rs11990279
rs61883564
rs16866406
rs16934185
rs75453768
rs9393920
All − MR Egger
All − IVW(MRE)
−2
−1
0
1
MR effect size for
'Gut microbiota abundance (genus Oscillibacter id.2063)' on 'Graves' disease'

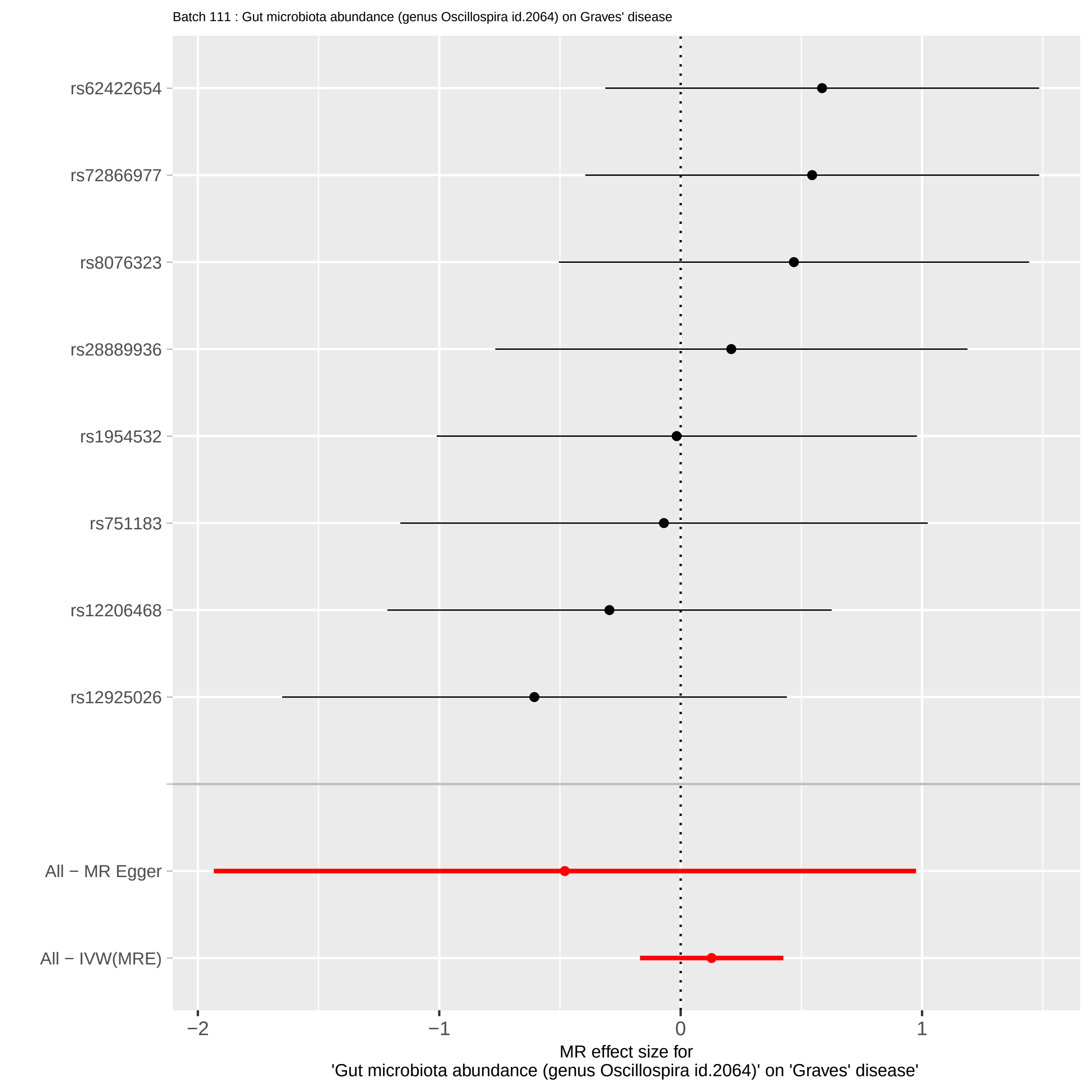
Batch 111 : Gut microbiota abundance (genus Oscillospira id.2064) on Graves' disease
rs62422654
rs72866977
rs8076323
rs28889936
rs1954532
rs751183
rs12206468
rs12925026
All − MR Egger
All − IVW(MRE)
−2
−1
0
1
MR effect size for
'Gut microbiota abundance (genus Oscillospira id.2064)' on 'Graves' disease'

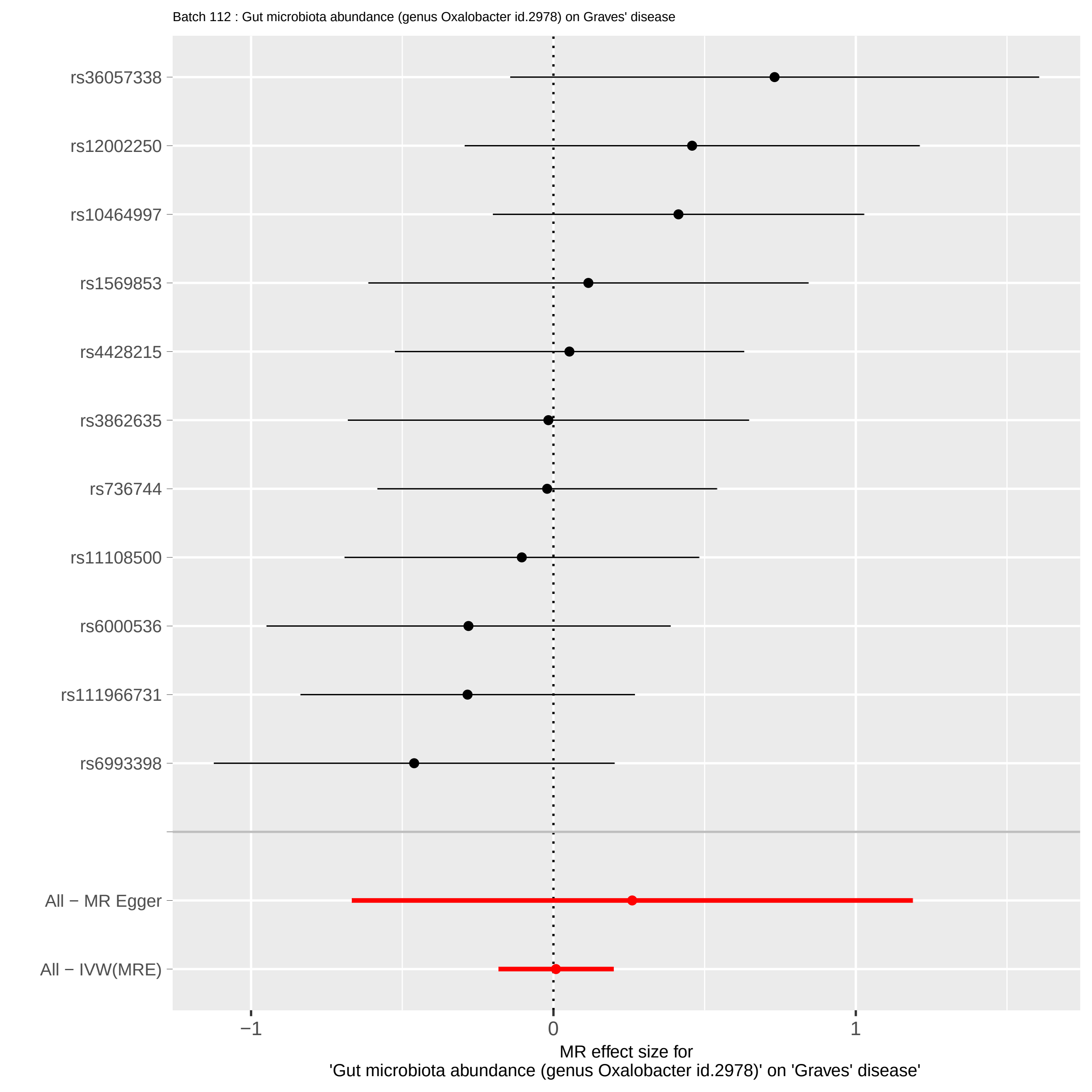

Batch 112 : Gut microbiota abundance (genus Oxalobacter id.2978) on Graves' disease
rs36057338
rs12002250
rs10464997
rs1569853
rs4428215
rs3862635
rs736744
rs11108500
rs6000536
rs111966731
rs6993398
All − MR Egger
All − IVW(MRE)
−1
0
1
MR effect size for
'Gut microbiota abundance (genus Oxalobacter id.2978)' on 'Graves' disease'

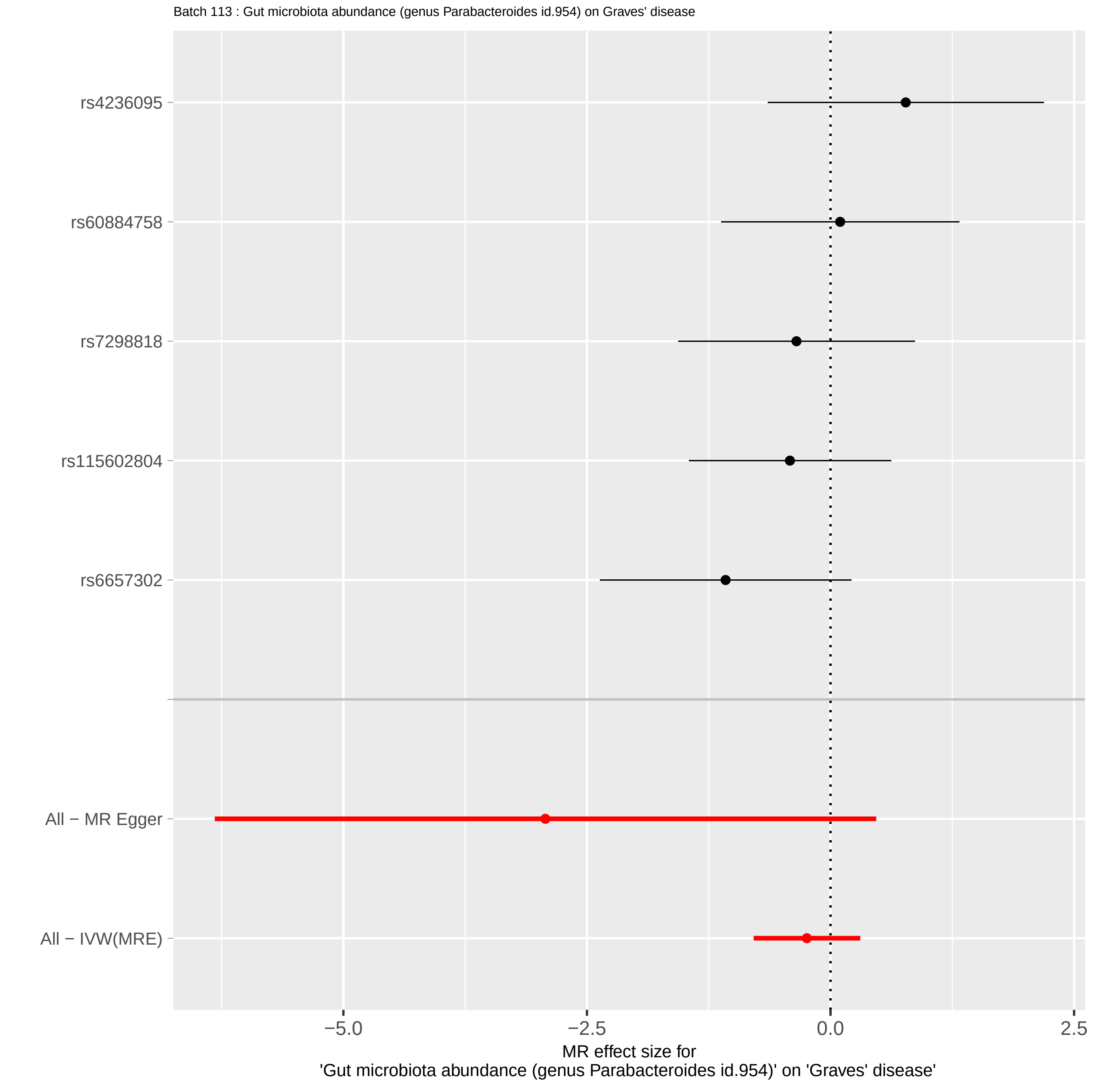

Batch 113 : Gut microbiota abundance (genus Parabacteroides id.954) on Graves' disease
rs4236095
rs60884758
rs7298818
rs115602804
rs6657302
All − MR Egger
All − IVW(MRE)
−5.0
−2.5
0.0
2.5
MR effect size for
'Gut microbiota abundance (genus Parabacteroides id.954)' on 'Graves' disease'

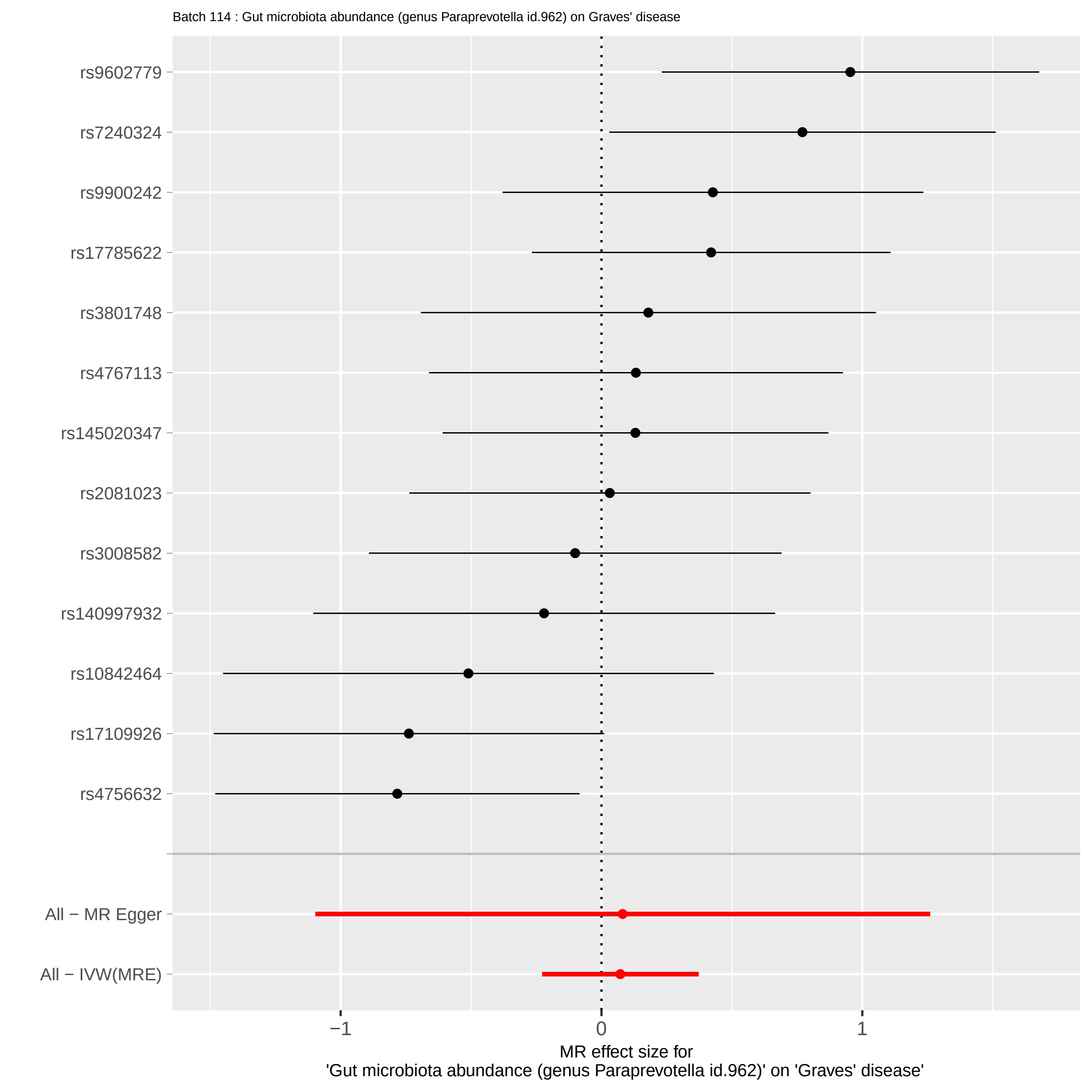

Batch 114 : Gut microbiota abundance (genus Paraprevotella id.962) on Graves' disease
rs9602779
rs7240324
rs9900242
rs17785622
rs3801748
rs4767113
rs145020347
rs2081023
rs3008582
rs140997932
rs10842464
rs17109926
rs4756632
All − MR Egger
All − IVW(MRE)
−1
0
1
MR effect size for
'Gut microbiota abundance (genus Paraprevotella id.962)' on 'Graves' disease'

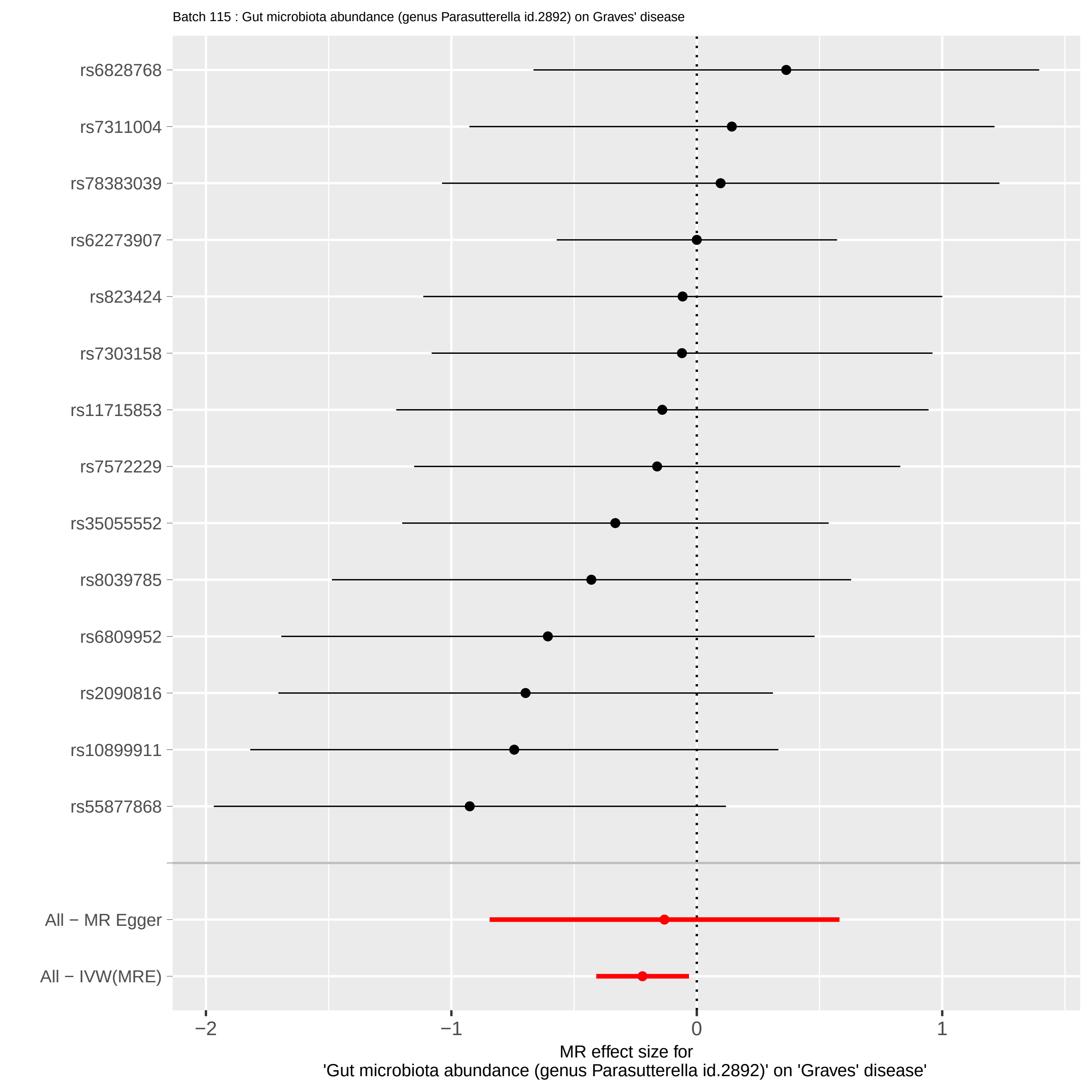
Batch 115 : Gut microbiota abundance (genus Parasutterella id.2892) on Graves' disease
rs6828768
rs7311004
rs78383039
rs62273907
rs823424
rs7303158
rs11715853
rs7572229
rs35055552
rs8039785
rs6809952
rs2090816
rs10899911
rs55877868
All − MR Egger
All − IVW(MRE)
−2
−1
0
1
MR effect size for
'Gut microbiota abundance (genus Parasutterella id.2892)' on 'Graves' disease'

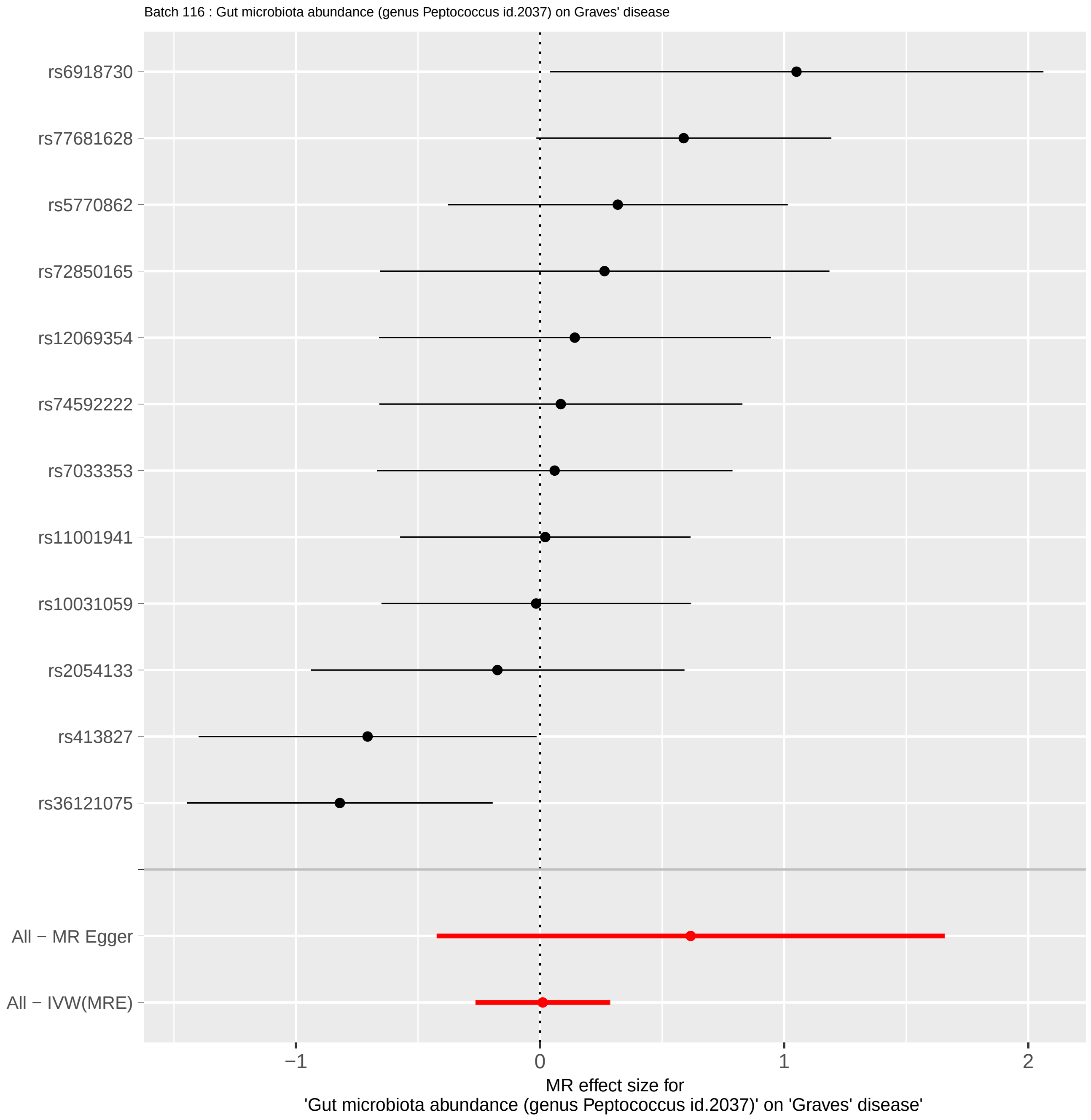
Batch 116 : Gut microbiota abundance (genus Peptococcus id.2037) on Graves' disease
rs6918730
rs77681628
rs5770862
rs72850165
rs12069354
rs74592222
rs7033353
rs11001941
rs10031059
rs2054133
rs413827
rs36121075
All − MR Egger
All − IVW(MRE)
−1
0
1
2
MR effect size for
'Gut microbiota abundance (genus Peptococcus id.2037)' on 'Graves' disease'

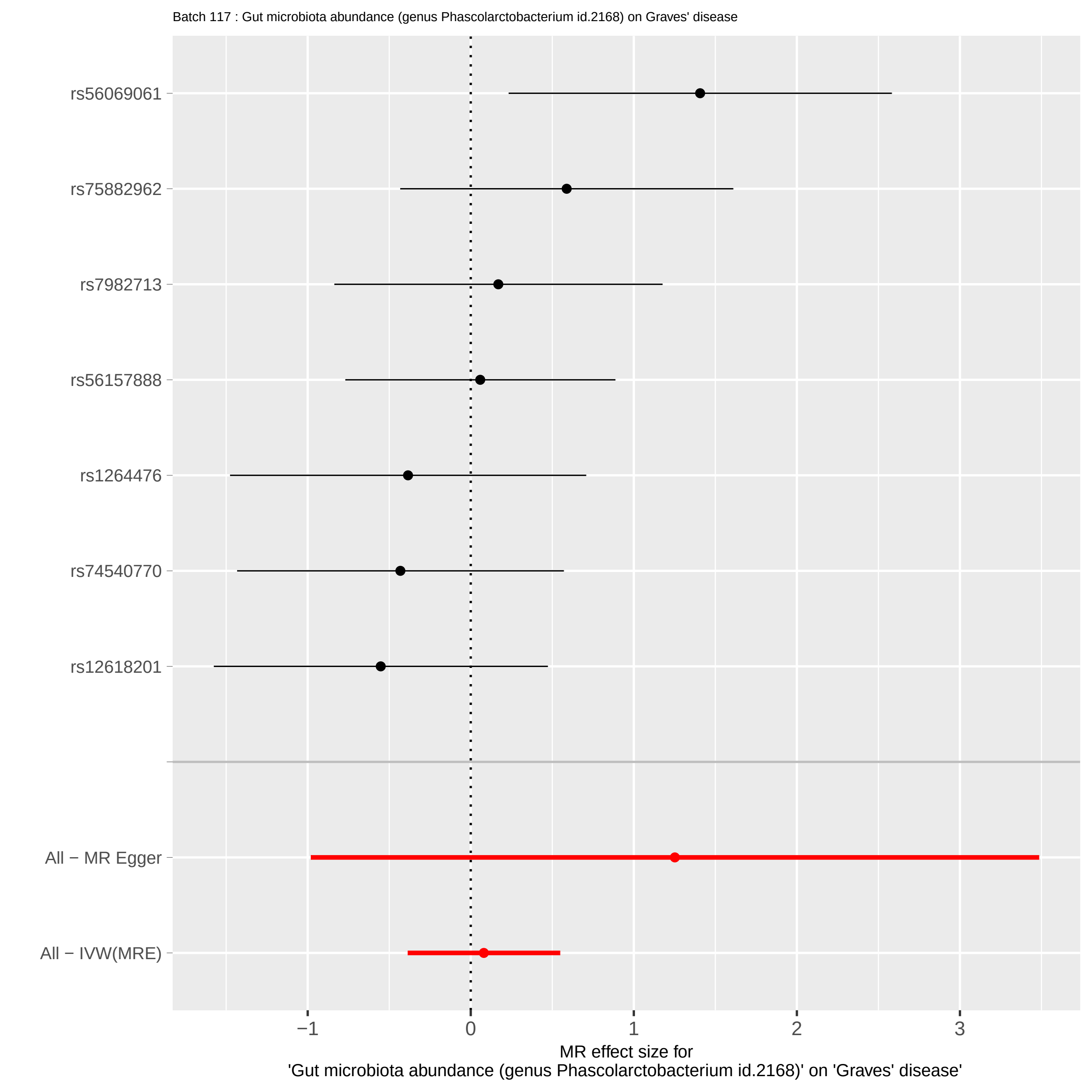
Batch 117 : Gut microbiota abundance (genus Phascolarctobacterium id.2168) on Graves' disease
rs56069061
rs75882962
rs7982713
rs56157888
rs1264476
rs74540770
rs12618201
All − MR Egger
All − IVW(MRE)
−1
0
1
2
3
MR effect size for
'Gut microbiota abundance (genus Phascolarctobacterium id.2168)' on 'Graves' disease'

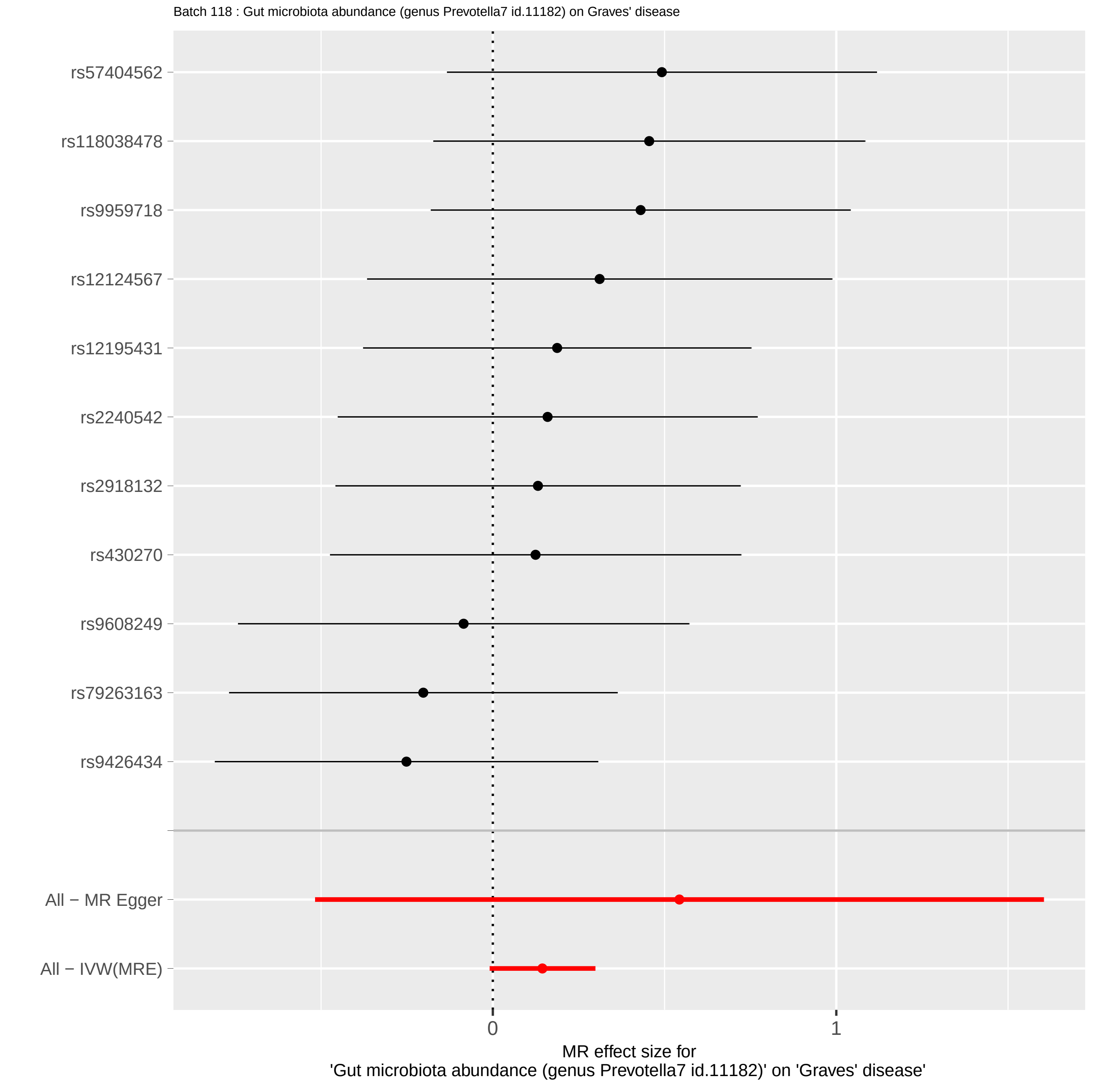

Batch 118 : Gut microbiota abundance (genus Prevotella7 id.11182) on Graves' disease
rs57404562
rs118038478
rs9959718
rs12124567
rs12195431
rs2240542
rs2918132
rs430270
rs9608249
rs79263163
rs9426434
All − MR Egger
All − IVW(MRE)
0
1
MR effect size for
'Gut microbiota abundance (genus Prevotella7 id.11182)' on 'Graves' disease'

Batch 119 : Gut microbiota abundance (genus Prevotella9 id.11183) on Graves' disease

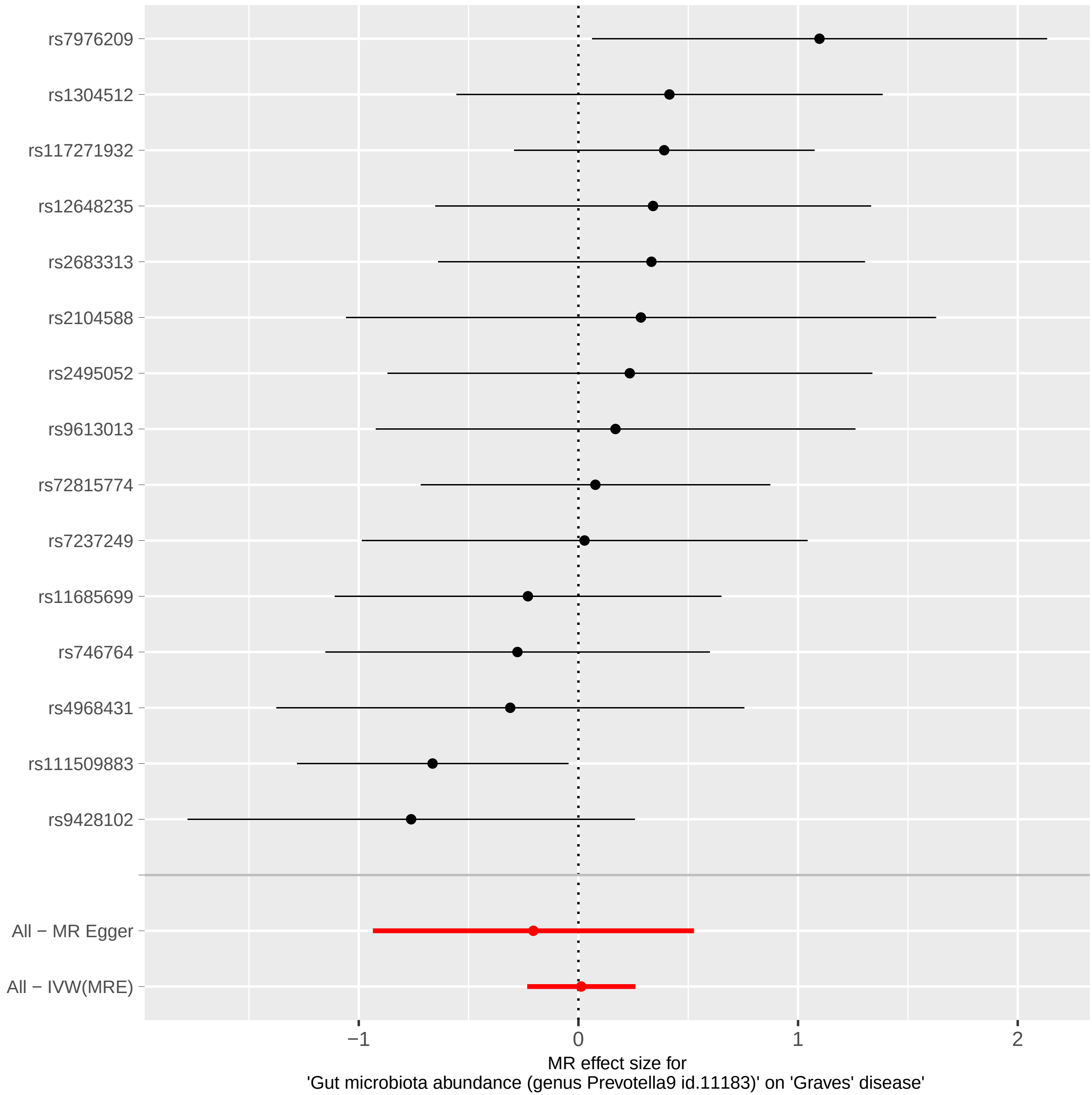

Batch 120 : Gut microbiota abundance (genus Rikenellaceae RC9 gut group id.11191) on Graves' disease

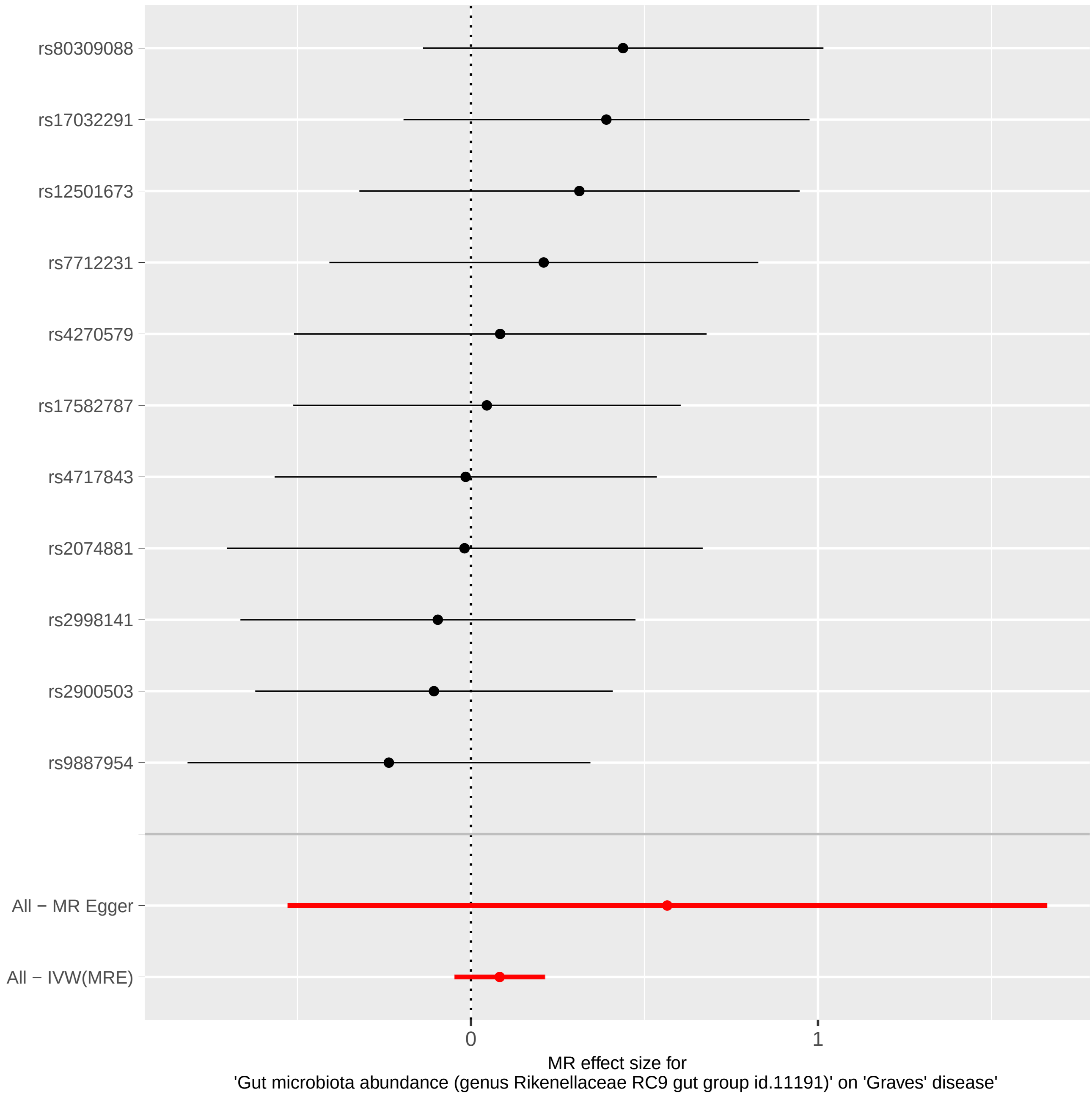

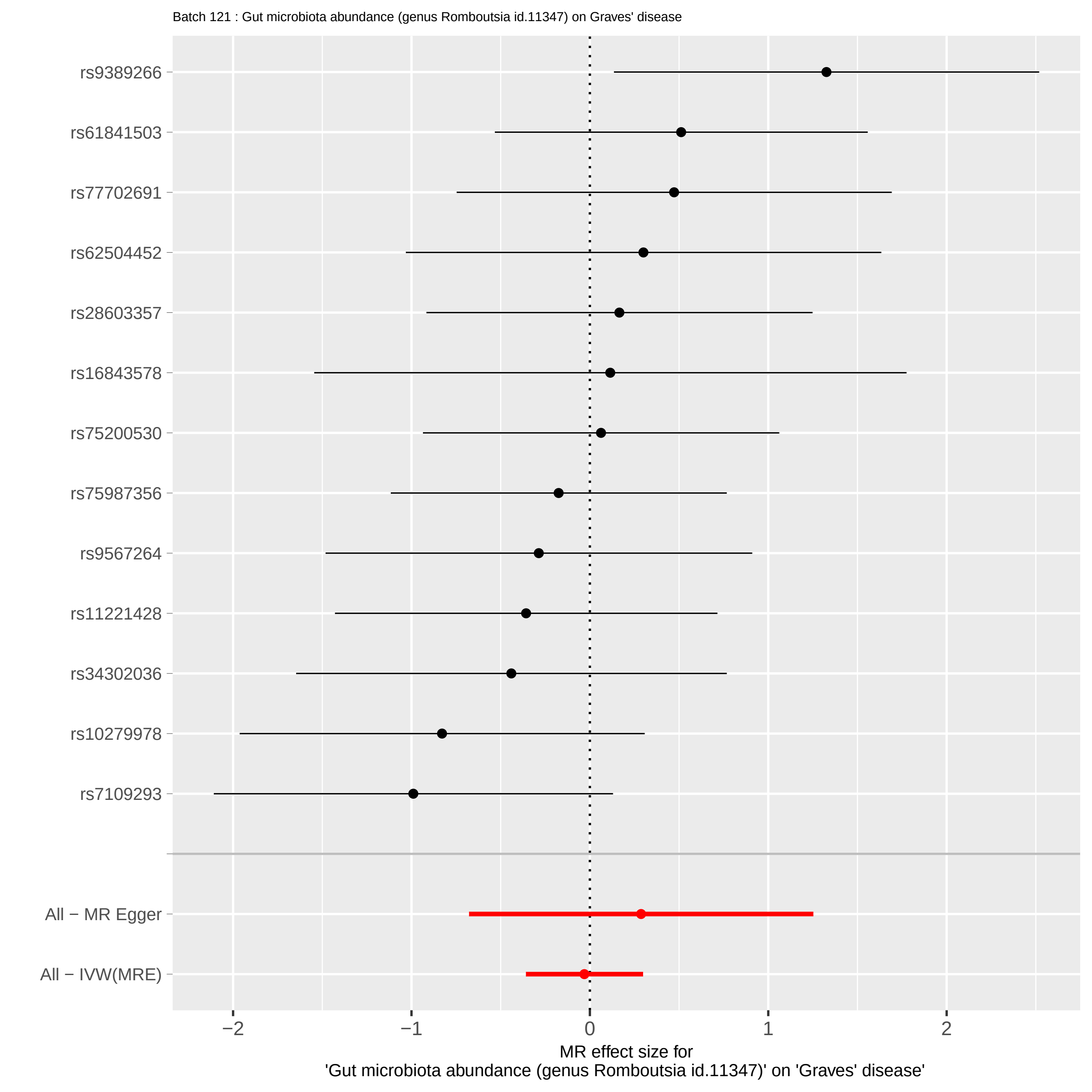
Batch 121 : Gut microbiota abundance (genus Romboutsia id.11347) on Graves' disease
rs9389266
rs61841503
rs77702691
rs62504452
rs28603357
rs16843578
rs75200530
rs75987356
rs9567264
rs11221428
rs34302036
rs10279978
rs7109293
All − MR Egger
All − IVW(MRE)
−2
−1
0
1
2
MR effect size for
'Gut microbiota abundance (genus Romboutsia id.11347)' on 'Graves' disease'

Batch 122 : Gut microbiota abundance (genus Roseburia id.2012) on Graves' disease

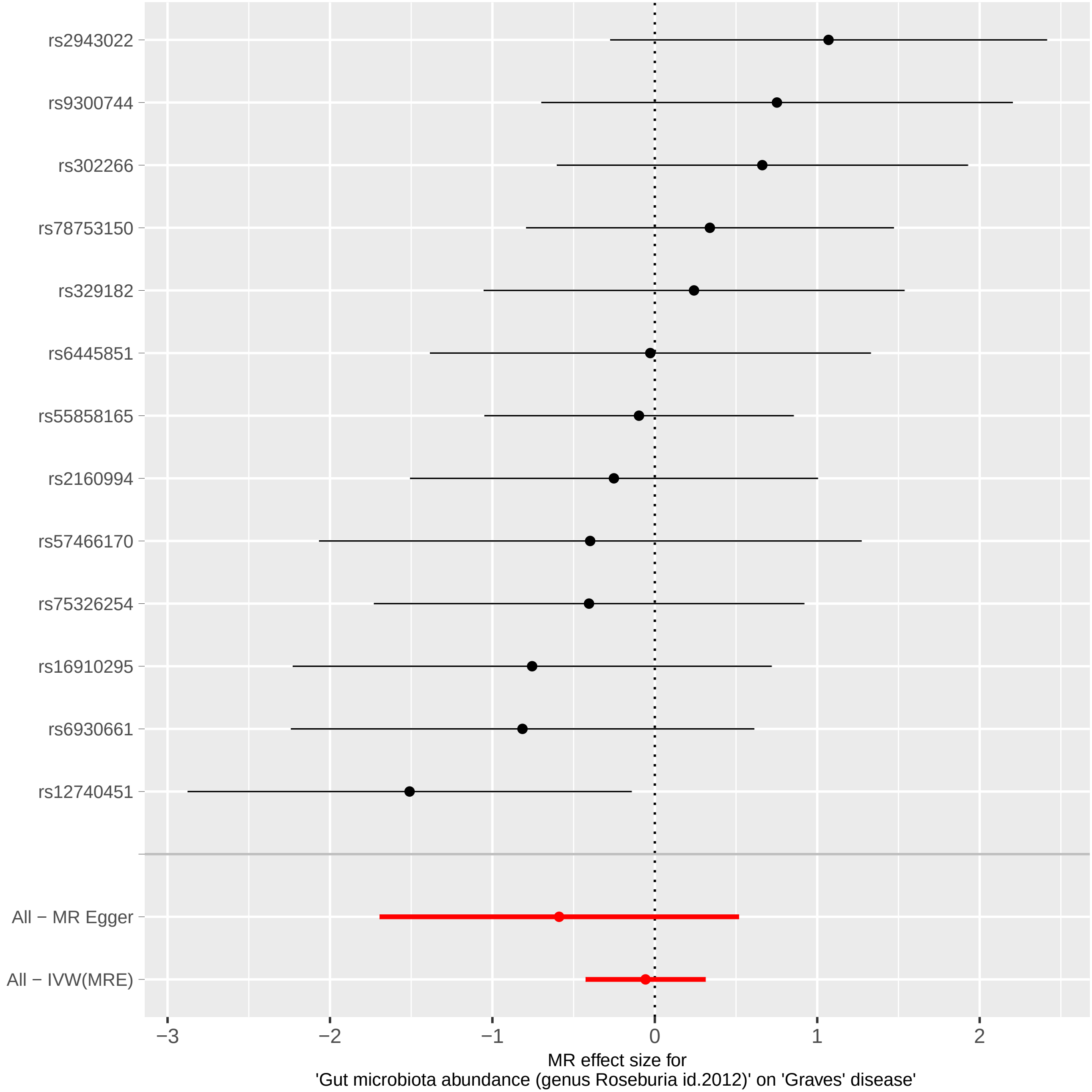

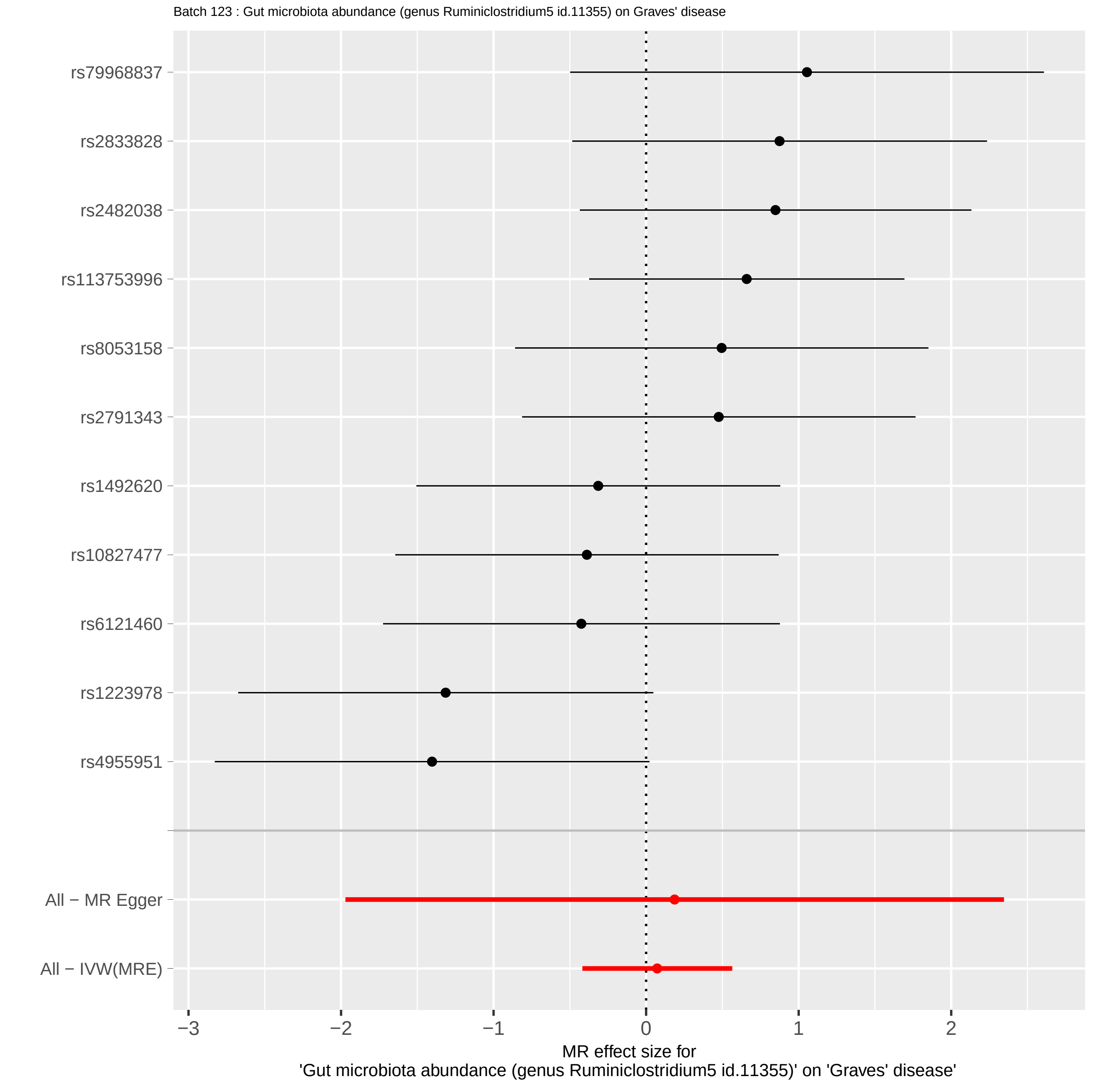
Batch 123 : Gut microbiota abundance (genus Ruminiclostridium5 id.11355) on Graves' disease
rs79968837
rs2833828
rs2482038
rs113753996
rs8053158
rs2791343
rs1492620
rs10827477
rs6121460
rs1223978
rs4955951
All − MR Egger
All − IVW(MRE)
−3
−2
−1
0
1
2
MR effect size for
'Gut microbiota abundance (genus Ruminiclostridium5 id.11355)' on 'Graves' disease'

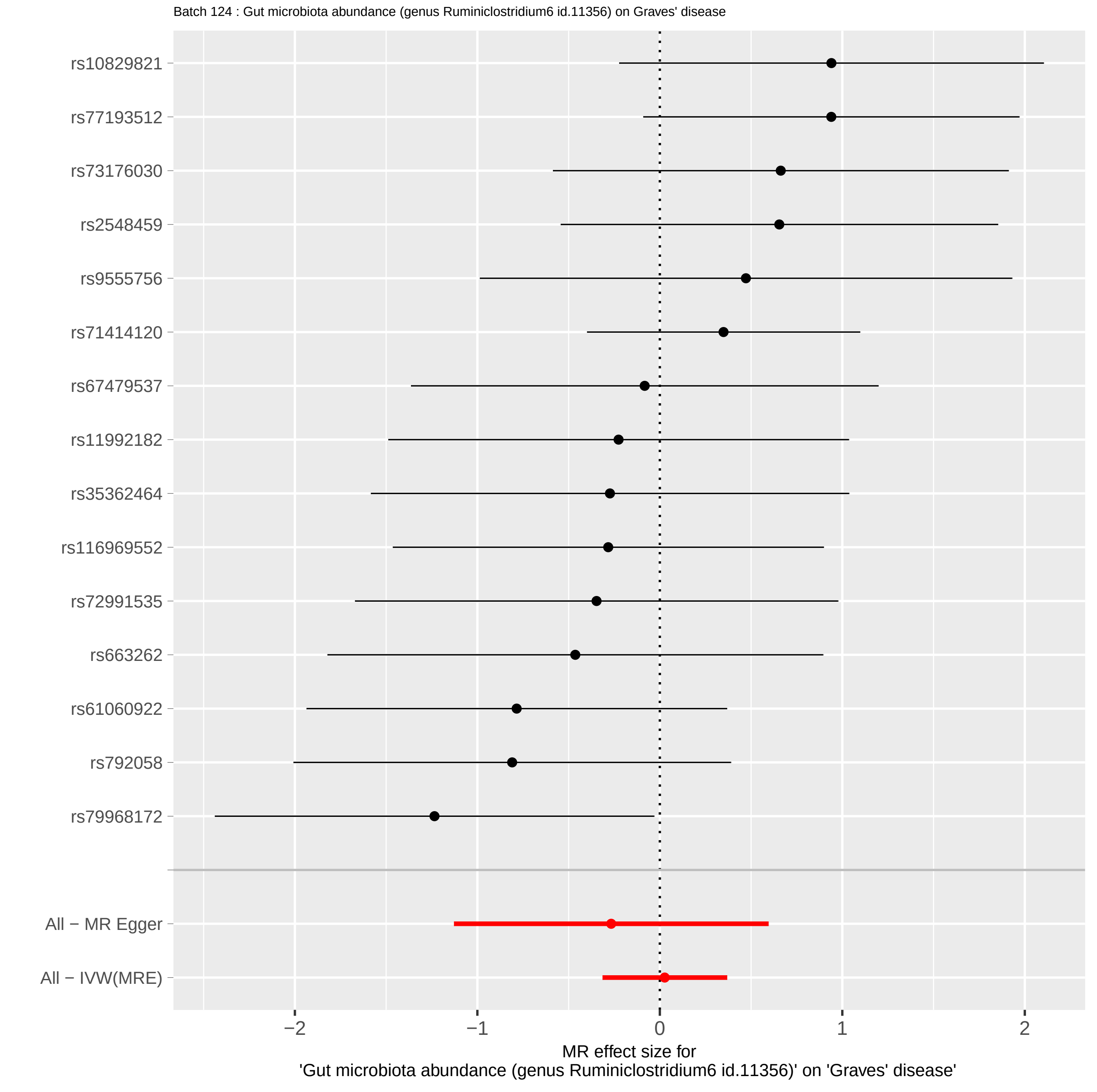
Batch 124 : Gut microbiota abundance (genus Ruminiclostridium6 id.11356) on Graves' disease
rs10829821
rs77193512
rs73176030
rs2548459
rs9555756
rs71414120
rs67479537
rs11992182
rs35362464
rs116969552
rs72991535
rs663262
rs61060922
rs792058
rs79968172
All − MR Egger
All − IVW(MRE)
−2
−1
0
1
2
MR effect size for
'Gut microbiota abundance (genus Ruminiclostridium6 id.11356)' on 'Graves' disease'

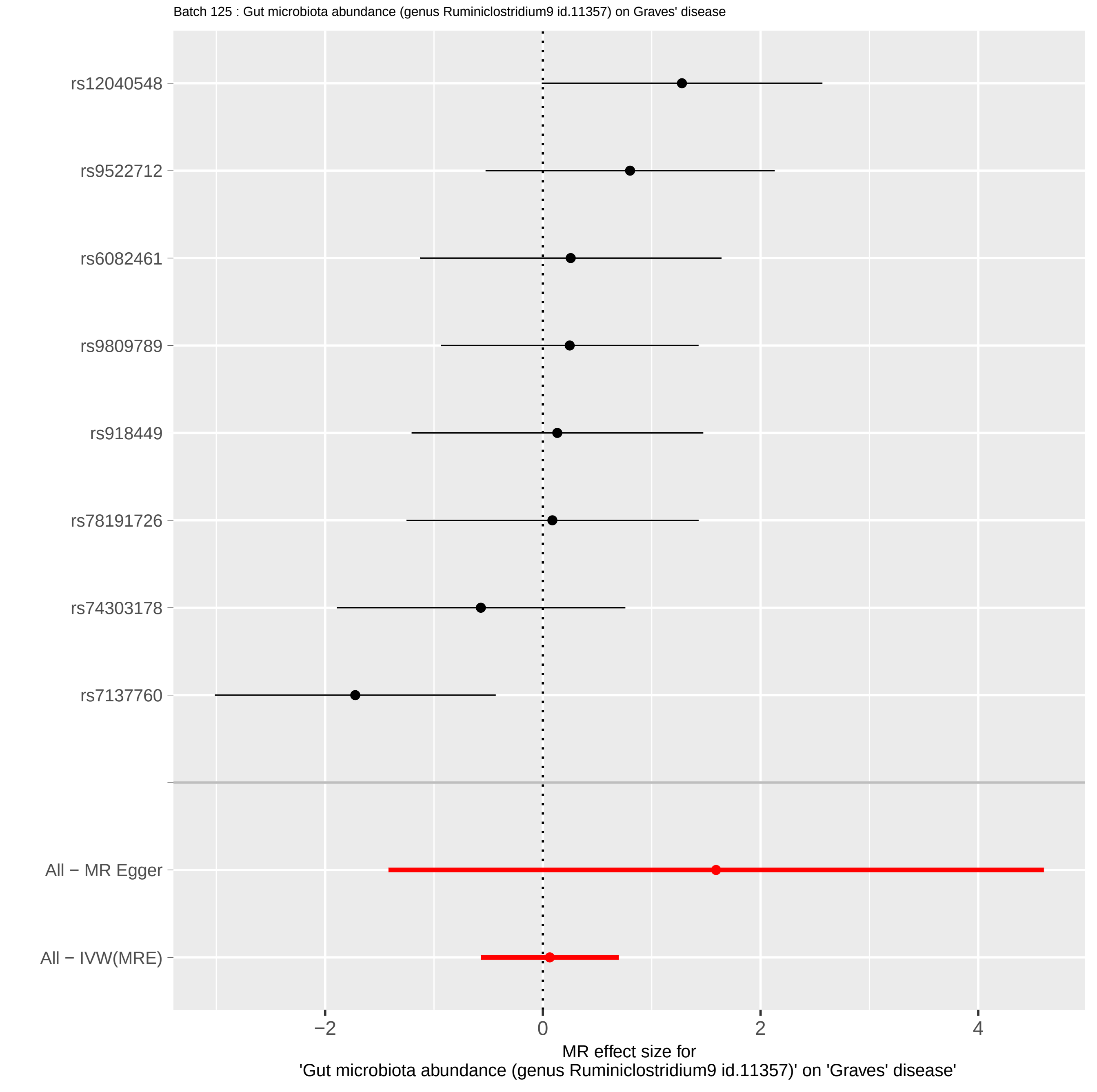

Batch 125 : Gut microbiota abundance (genus Ruminiclostridium9 id.11357) on Graves' disease
rs12040548
rs9522712
rs6082461
rs9809789
rs918449
rs78191726
rs74303178
rs7137760
All − MR Egger
All − IVW(MRE)
−2
0
2
4
MR effect size for
'Gut microbiota abundance (genus Ruminiclostridium9 id.11357)' on 'Graves' disease'

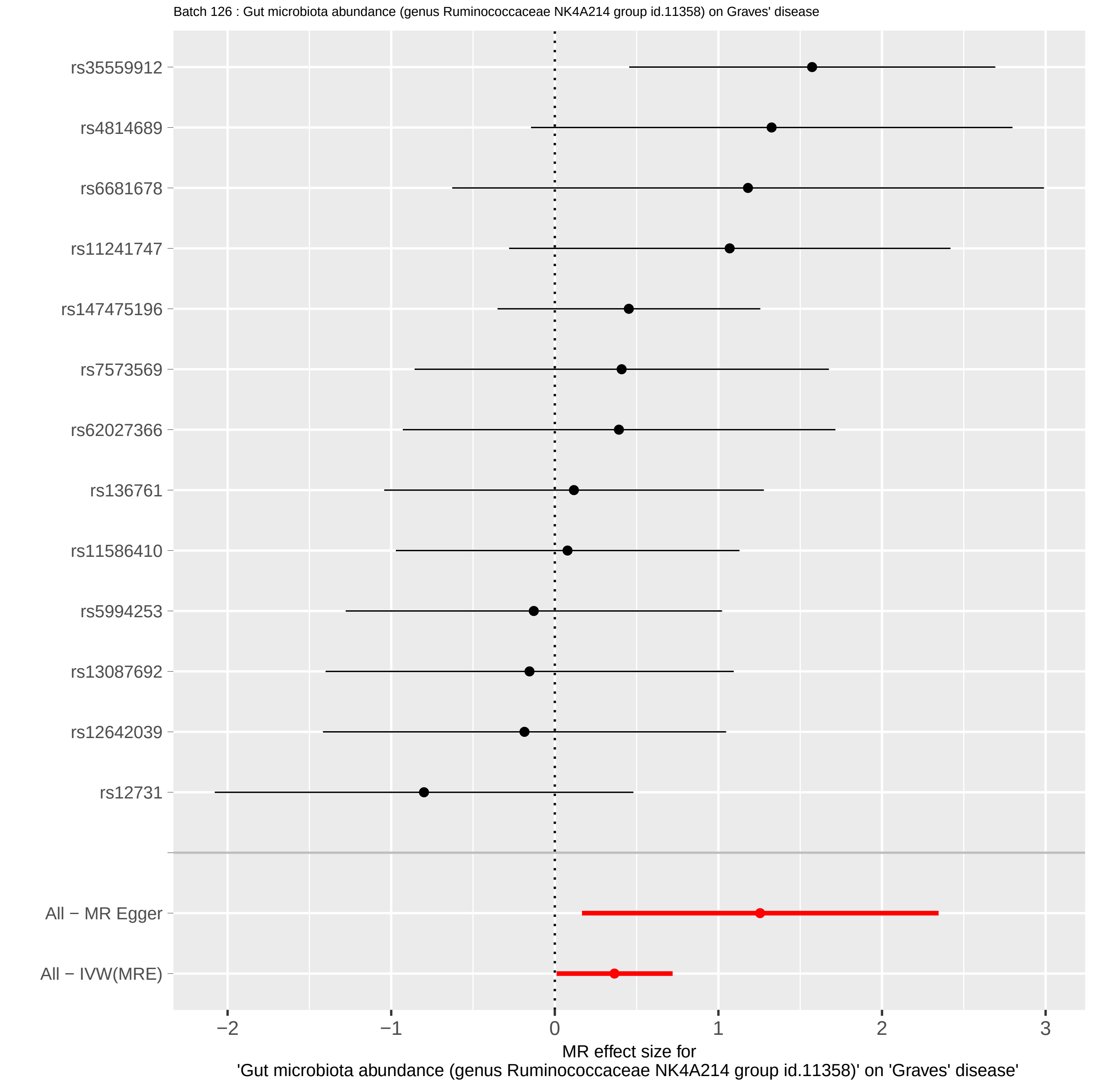

Batch 126 : Gut microbiota abundance (genus Ruminococcaceae NK4A214 group id.11358) on Graves' disease
rs35559912
rs4814689
rs6681678
rs11241747
rs147475196
rs7573569
rs62027366
rs136761
rs11586410
rs5994253
rs13087692
rs12642039
rs12731
All − MR Egger
All − IVW(MRE)
−2
−1
0
1
2
3
MR effect size for
'Gut microbiota abundance (genus Ruminococcaceae NK4A214 group id.11358)' on 'Graves' disease'

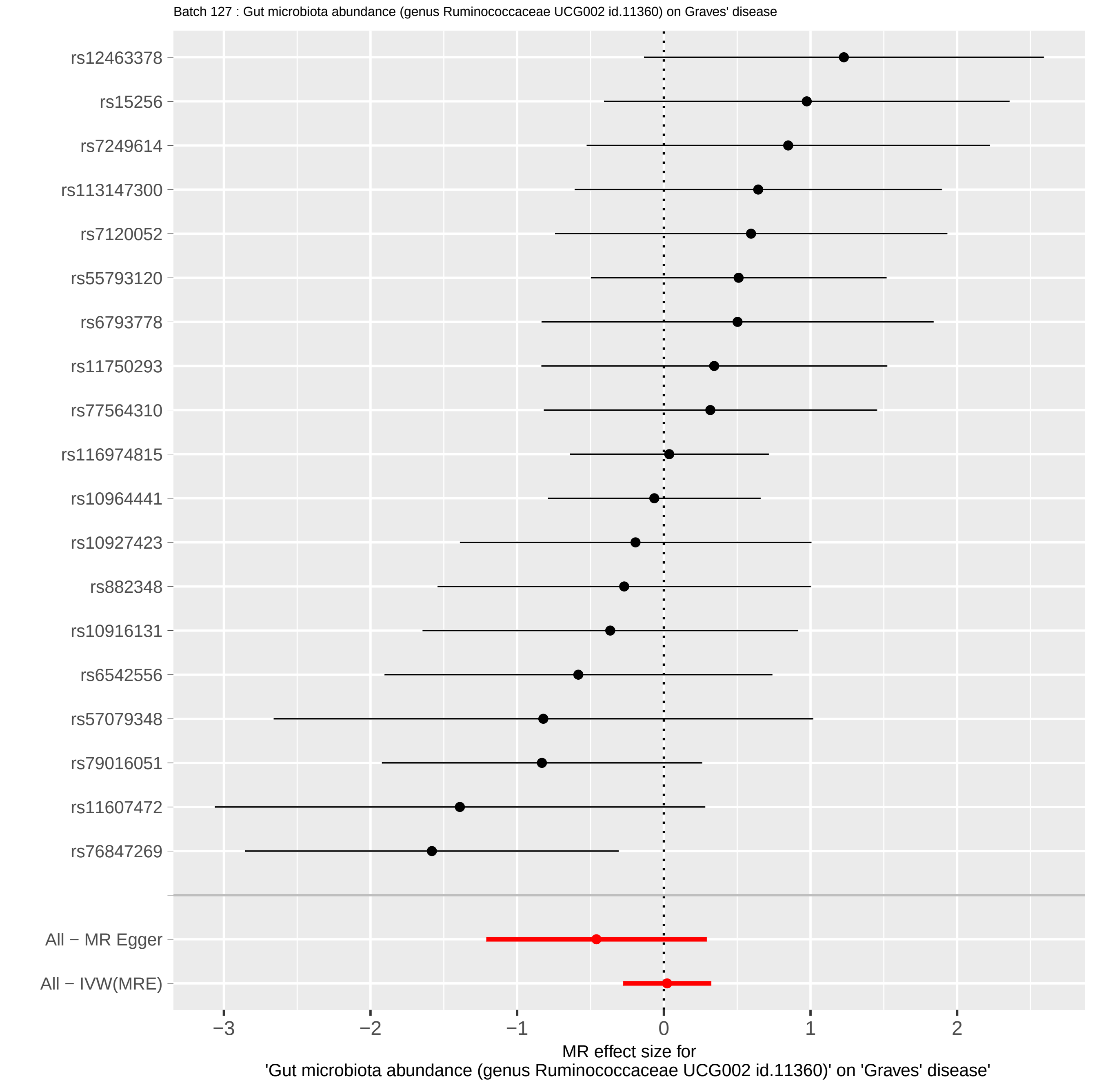

Batch 127 : Gut microbiota abundance (genus Ruminococcaceae UCG002 id.11360) on Graves' disease
rs12463378
rs15256
rs7249614
rs113147300
rs7120052
rs55793120
rs6793778
rs11750293
rs77564310
rs116974815
rs10964441
rs10927423
rs882348
rs10916131
rs6542556
rs57079348
rs79016051
rs11607472
rs76847269
All − MR Egger
All − IVW(MRE)
−3
−2
−1
0
1
2
MR effect size for
'Gut microbiota abundance (genus Ruminococcaceae UCG002 id.11360)' on 'Graves' disease'

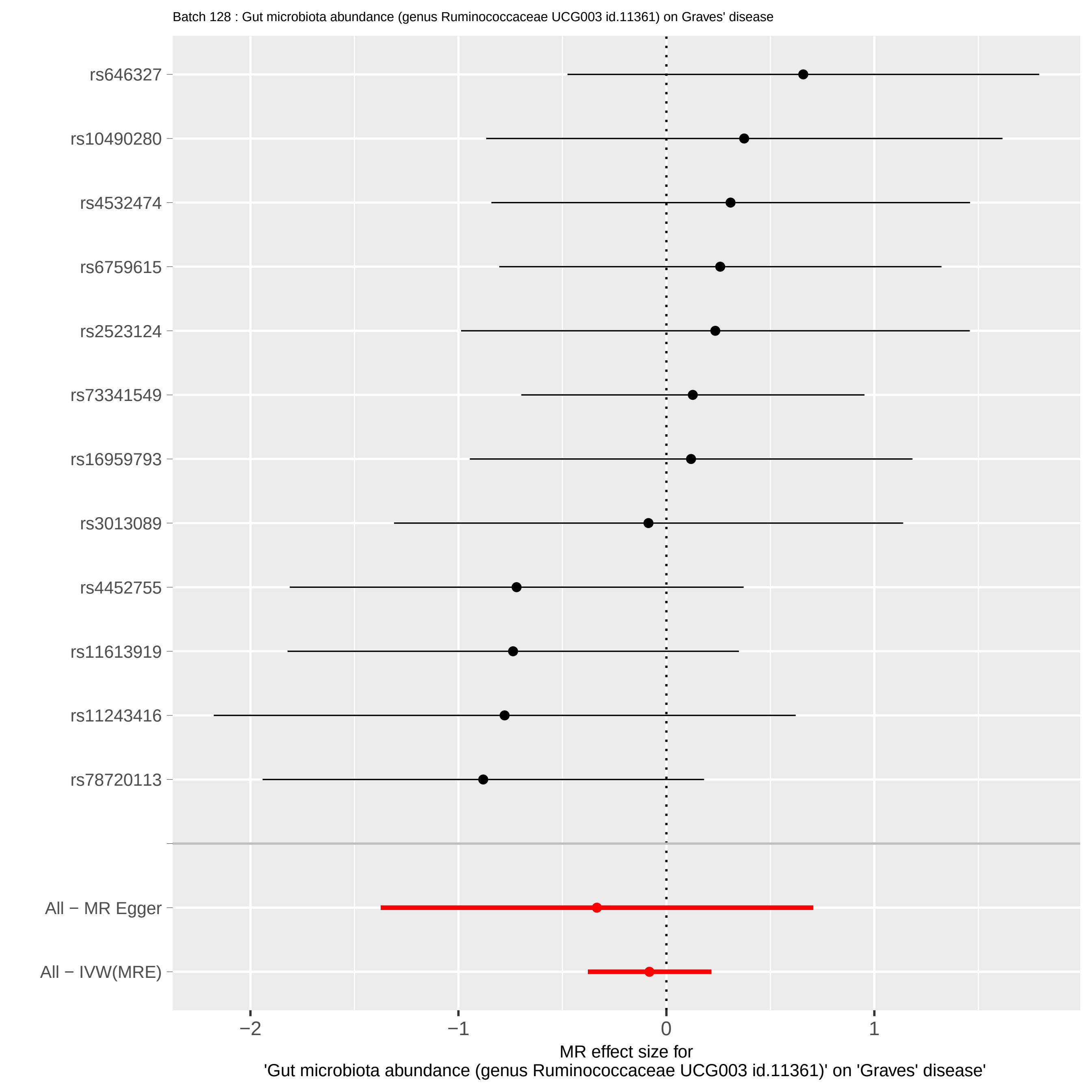
Batch 128 : Gut microbiota abundance (genus Ruminococcaceae UCG003 id.11361) on Graves' disease
rs646327
rs10490280
rs4532474
rs6759615
rs2523124
rs73341549
rs16959793
rs3013089
rs4452755
rs11613919
rs11243416
rs78720113
All − MR Egger
All − IVW(MRE)
−2
−1
0
1
MR effect size for
'Gut microbiota abundance (genus Ruminococcaceae UCG003 id.11361)' on 'Graves' disease'

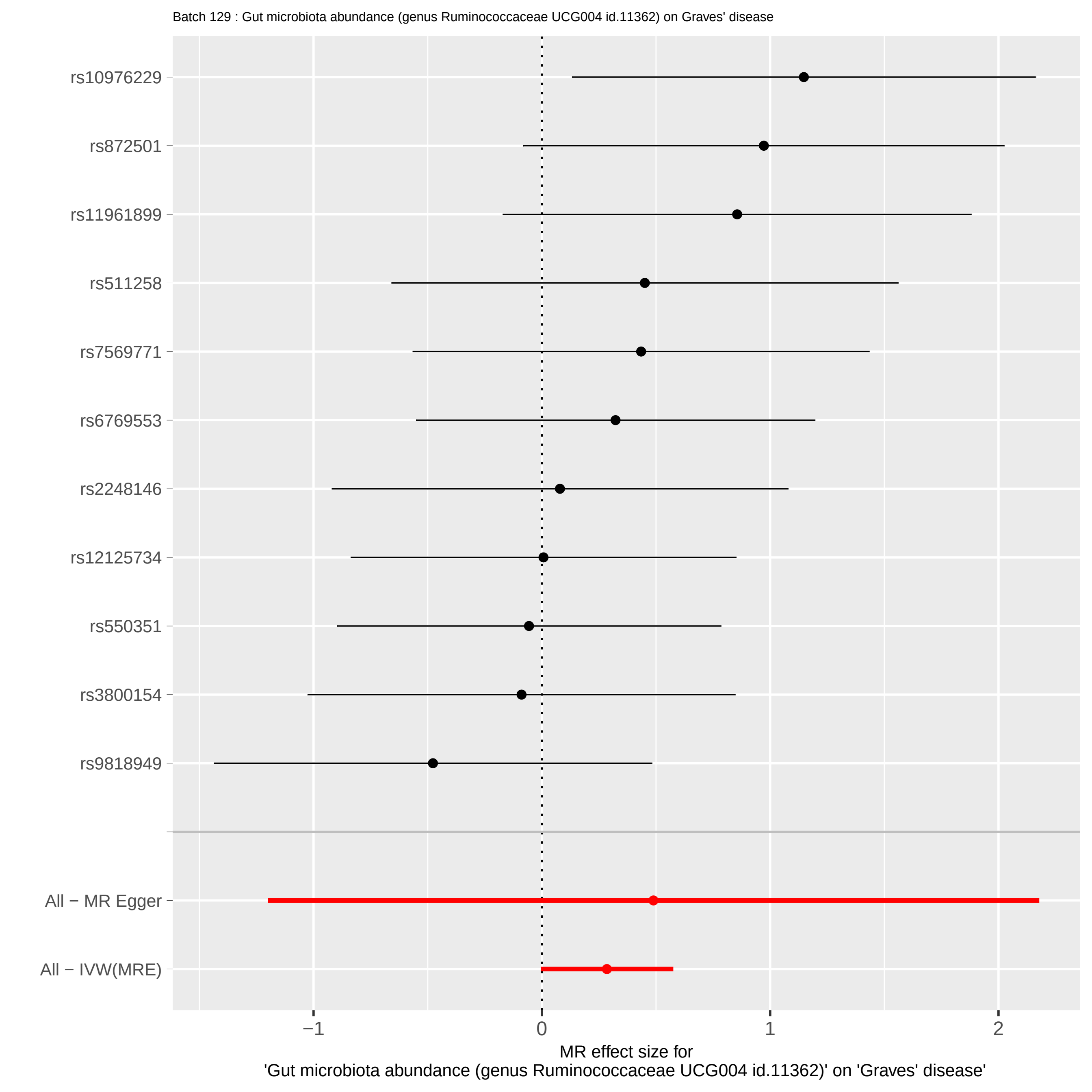
Batch 129 : Gut microbiota abundance (genus Ruminococcaceae UCG004 id.11362) on Graves' disease
rs10976229
rs872501
rs11961899
rs511258
rs7569771
rs6769553
rs2248146
rs12125734
rs550351
rs3800154
rs9818949
All − MR Egger
All − IVW(MRE)
−1
0
1
2
MR effect size for
'Gut microbiota abundance (genus Ruminococcaceae UCG004 id.11362)' on 'Graves' disease'

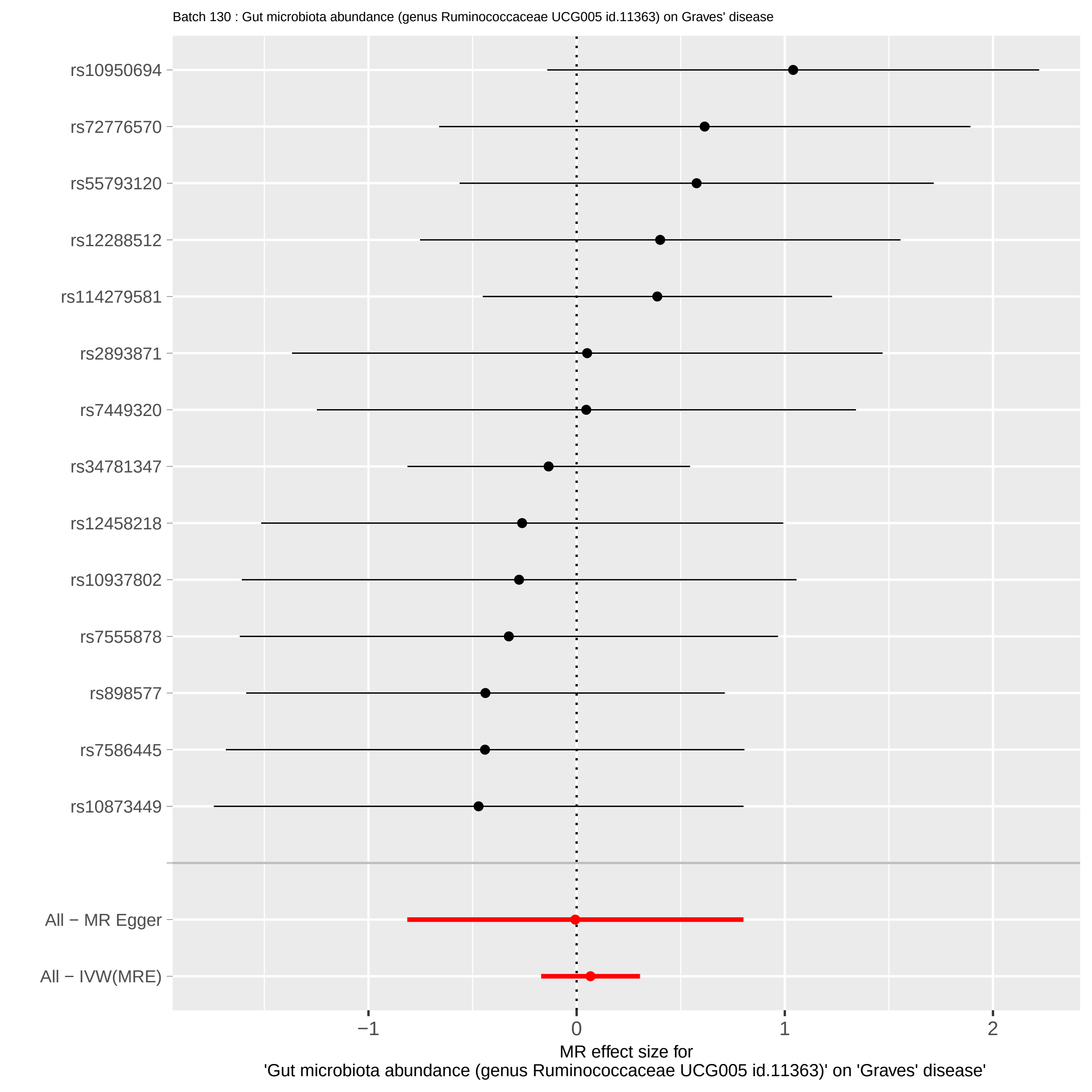

Batch 130 : Gut microbiota abundance (genus Ruminococcaceae UCG005 id.11363) on Graves' disease
rs10950694
rs72776570
rs55793120
rs12288512
rs114279581
rs2893871
rs7449320
rs34781347
rs12458218
rs10937802
rs7555878
rs898577
rs7586445
rs10873449
All − MR Egger
All − IVW(MRE)
−1
0
1
2
MR effect size for
'Gut microbiota abundance (genus Ruminococcaceae UCG005 id.11363)' on 'Graves' disease'

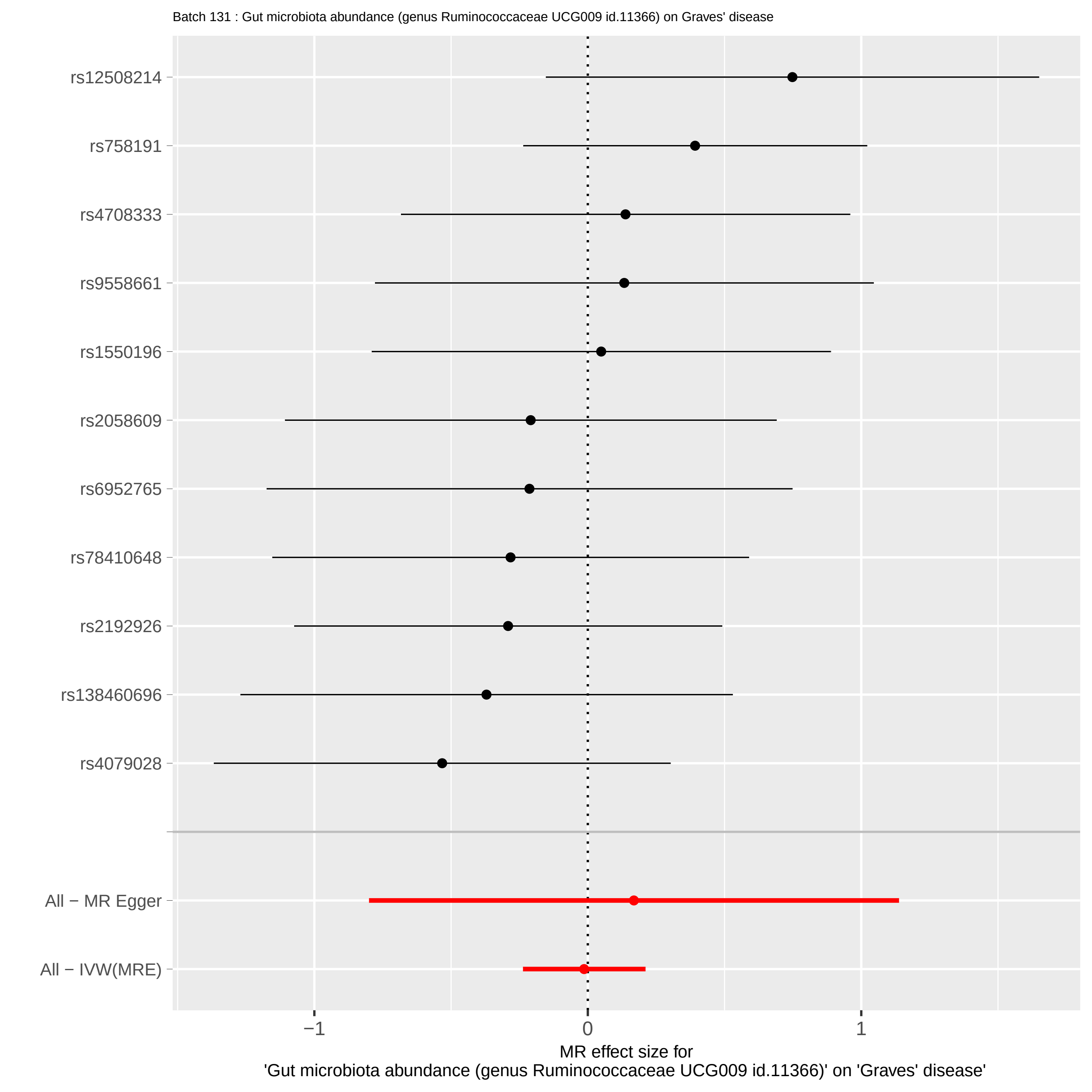

Batch 131 : Gut microbiota abundance (genus Ruminococcaceae UCG009 id.11366) on Graves' disease
rs12508214
rs758191
rs4708333
rs9558661
rs1550196
rs2058609
rs6952765
rs78410648
rs2192926
rs138460696
rs4079028
All − MR Egger
All − IVW(MRE)
−1
0
1
MR effect size for
'Gut microbiota abundance (genus Ruminococcaceae UCG009 id.11366)' on 'Graves' disease'

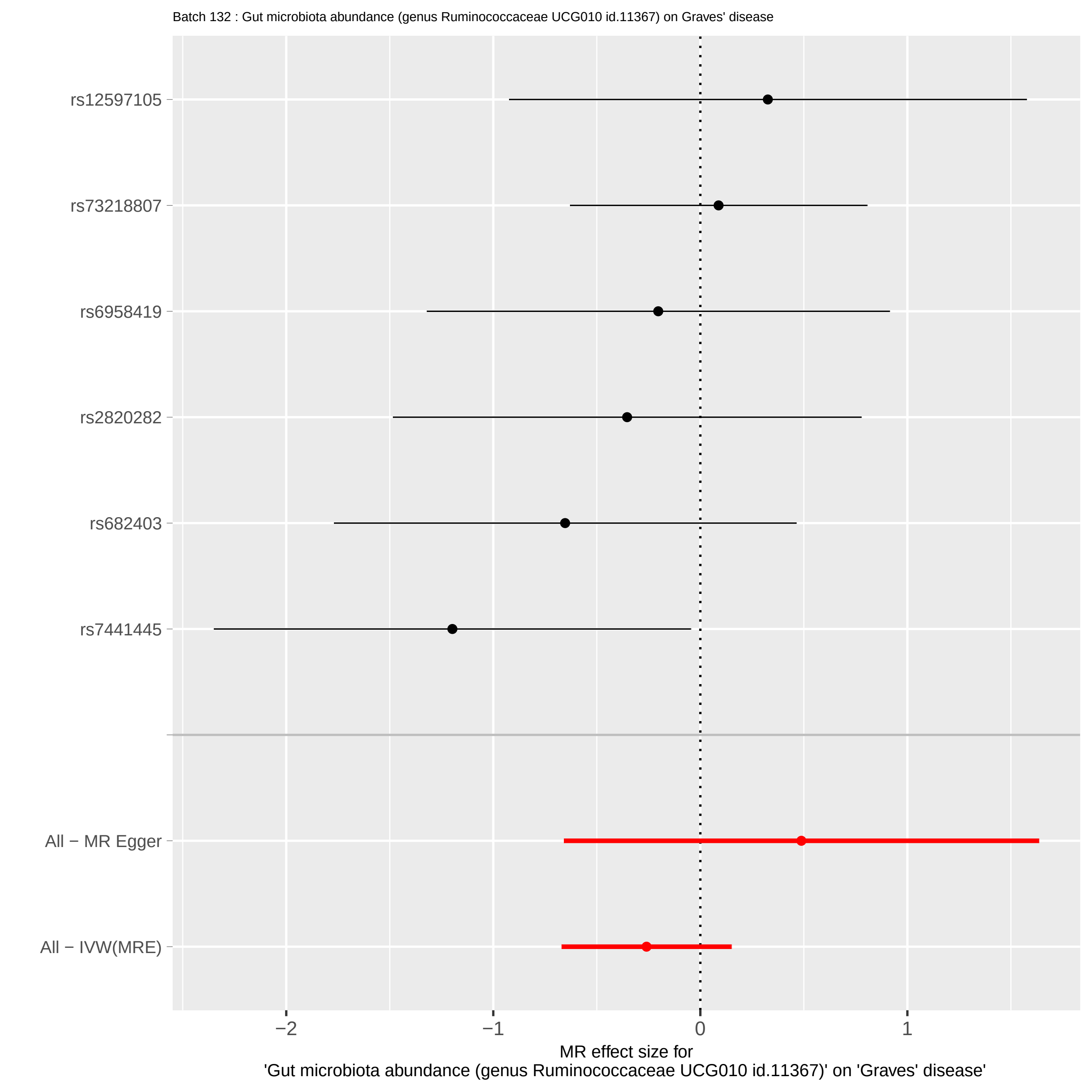
Batch 132 : Gut microbiota abundance (genus Ruminococcaceae UCG010 id.11367) on Graves' disease
rs12597105
rs73218807
rs6958419
rs2820282
rs682403
rs7441445
All − MR Egger
All − IVW(MRE)
−2
−1
0
1
MR effect size for
'Gut microbiota abundance (genus Ruminococcaceae UCG010 id.11367)' on 'Graves' disease'

Batch 133 : Gut microbiota abundance (genus Ruminococcaceae UCG011 id.11368) on Graves' disease

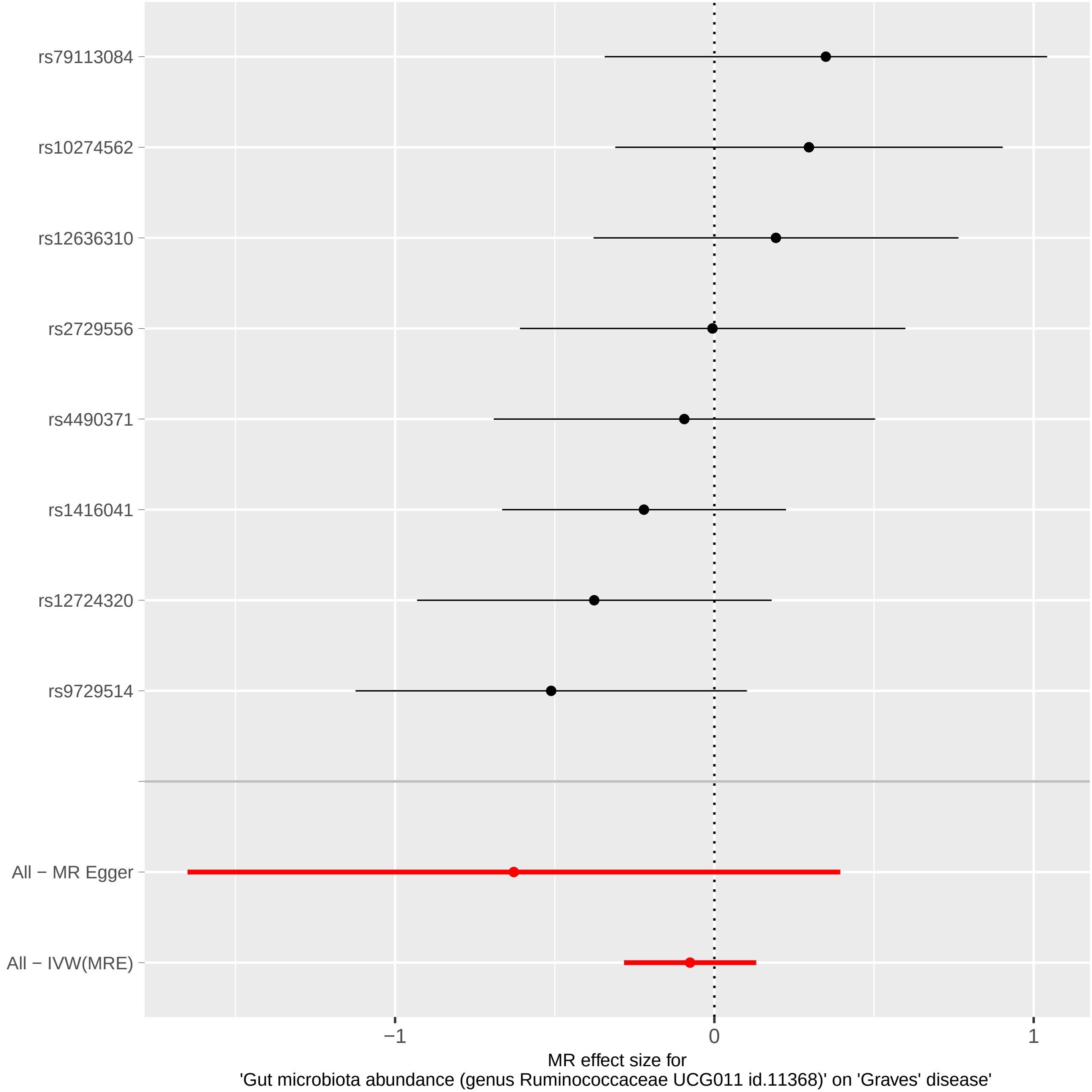

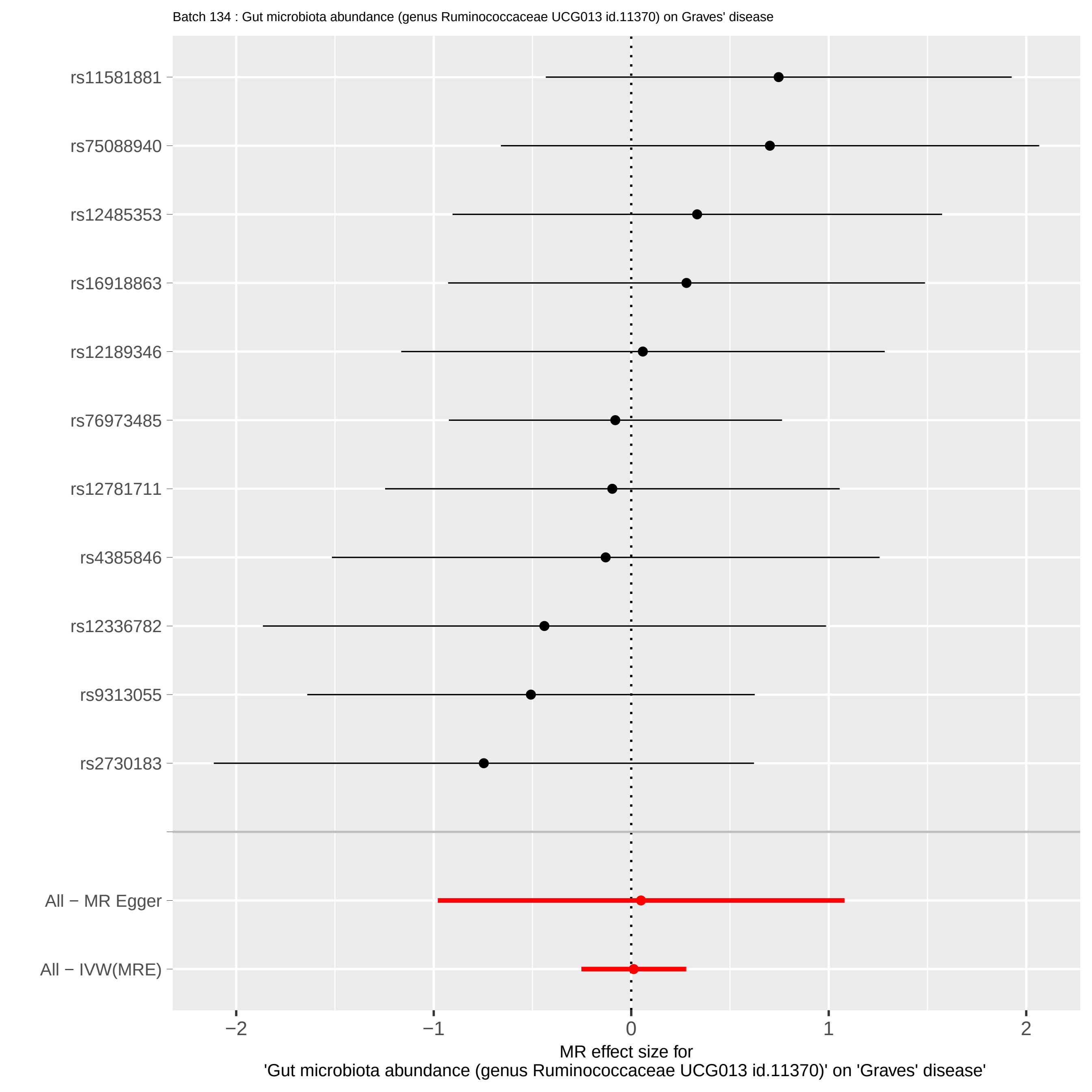
Batch 134 : Gut microbiota abundance (genus Ruminococcaceae UCG013 id.11370) on Graves' disease
rs11581881
rs75088940
rs12485353
rs16918863
rs12189346
rs76973485
rs12781711
rs4385846
rs12336782
rs9313055
rs2730183
All − MR Egger
All − IVW(MRE)
−2
−1
0
1
2
MR effect size for
'Gut microbiota abundance (genus Ruminococcaceae UCG013 id.11370)' on 'Graves' disease'

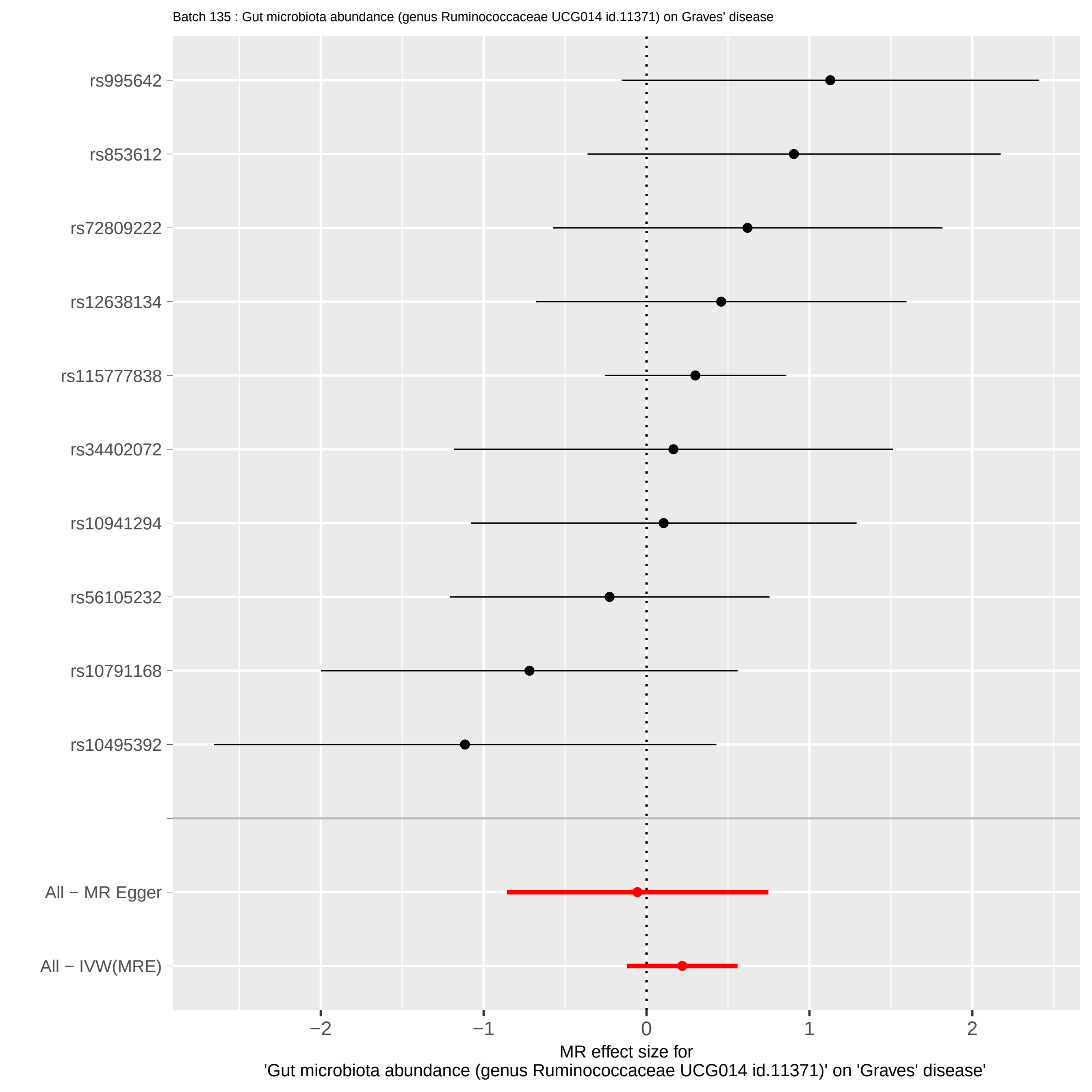
Batch 135 : Gut microbiota abundance (genus Ruminococcaceae UCG014 id.11371) on Graves' disease
rs995642
rs853612
rs72809222
rs12638134
rs115777838
rs34402072
rs10941294
rs56105232
rs10791168
rs10495392
All − MR Egger
All − IVW(MRE)
−2
−1
0
1
2
MR effect size for
'Gut microbiota abundance (genus Ruminococcaceae UCG014 id.11371)' on 'Graves' disease'

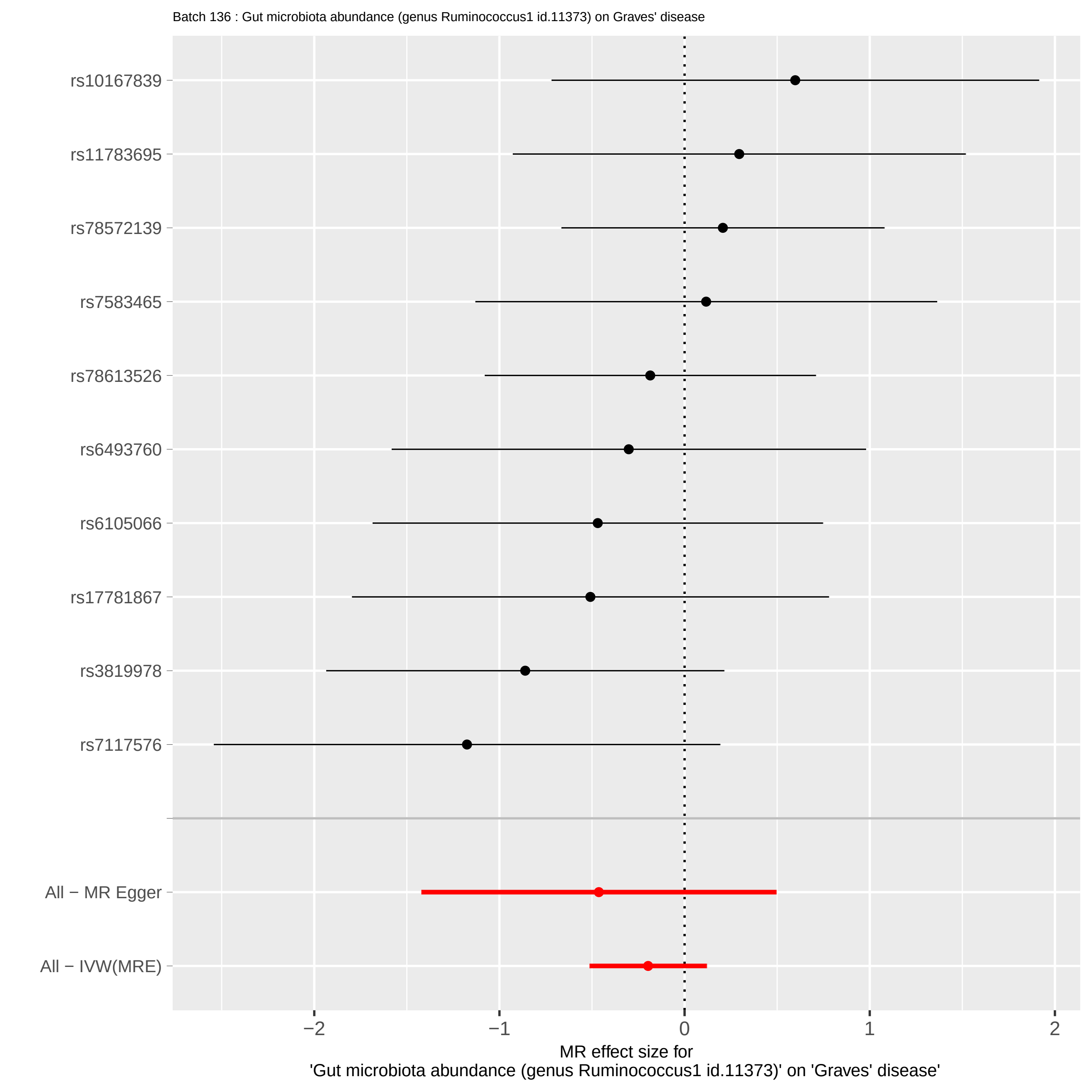

Batch 136 : Gut microbiota abundance (genus Ruminococcus1 id.11373) on Graves' disease
rs10167839
rs11783695
rs78572139
rs7583465
rs78613526
rs6493760
rs6105066
rs17781867
rs3819978
rs7117576
All − MR Egger
All − IVW(MRE)
−2
−1
0
1
2
MR effect size for
'Gut microbiota abundance (genus Ruminococcus1 id.11373)' on 'Graves' disease'

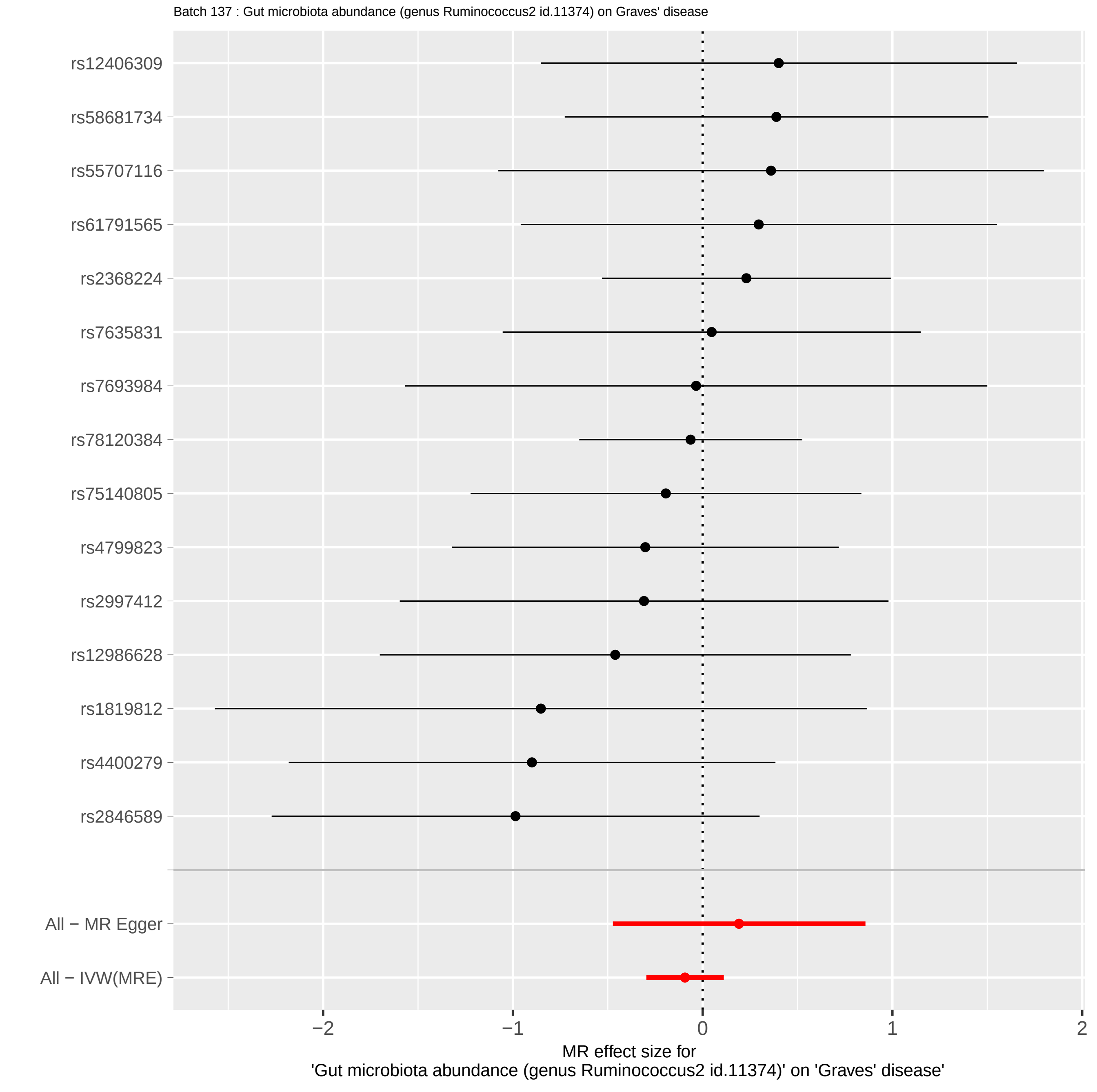

Batch 137 : Gut microbiota abundance (genus Ruminococcus2 id.11374) on Graves' disease
rs12406309
rs58681734
rs55707116
rs61791565
rs2368224
rs7635831
rs7693984
rs78120384
rs75140805
rs4799823
rs2997412
rs12986628
rs1819812
rs4400279
rs2846589
All − MR Egger
All − IVW(MRE)
−2
−1
0
1
2
MR effect size for
'Gut microbiota abundance (genus Ruminococcus2 id.11374)' on 'Graves' disease'

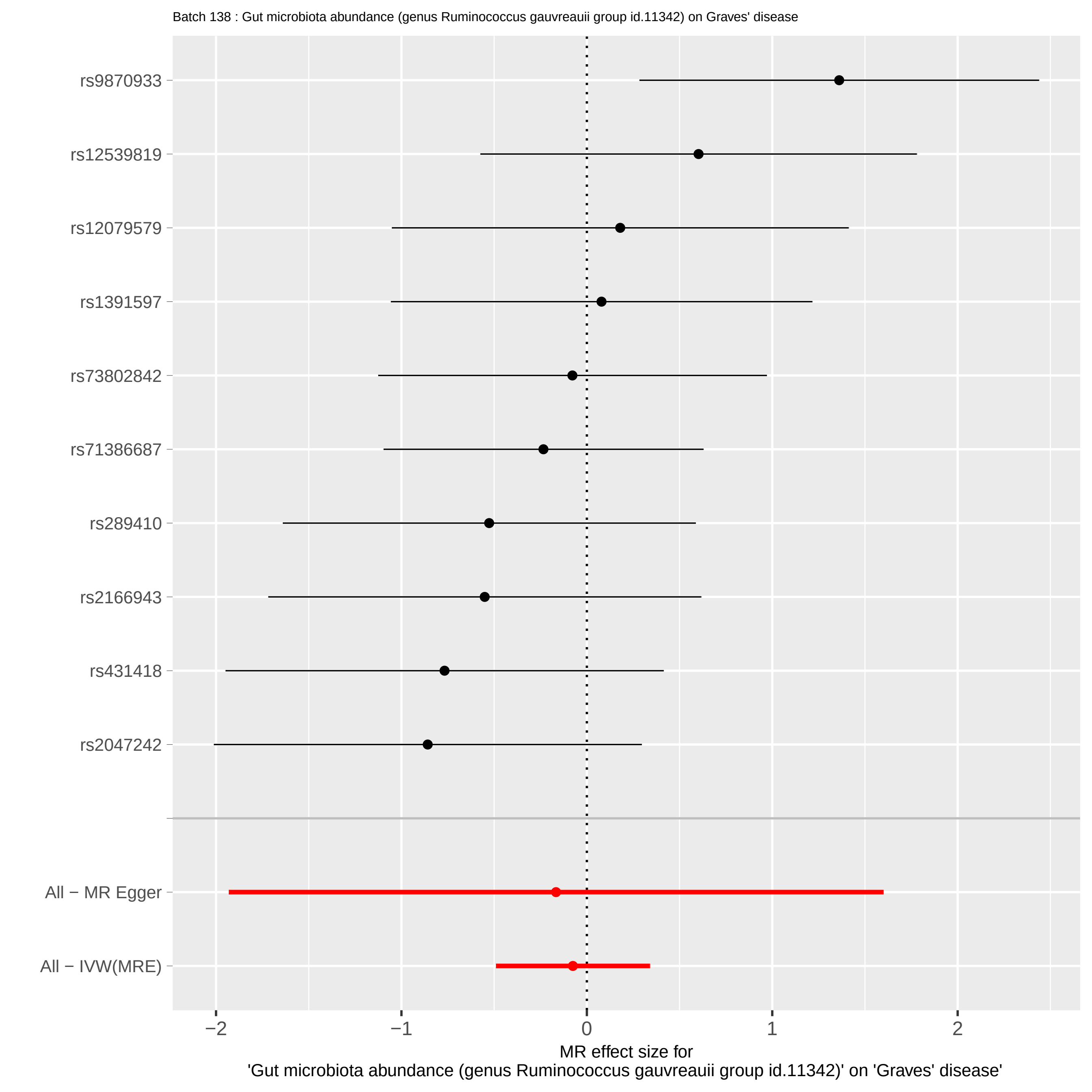

Batch 138 : Gut microbiota abundance (genus Ruminococcus gauvreauii group id.11342) on Graves' disease
rs9870933
rs12539819
rs12079579
rs1391597
rs73802842
rs71386687
rs289410
rs2166943
rs431418
rs2047242
All − MR Egger
All − IVW(MRE)
−2
−1
0
1
2
MR effect size for
'Gut microbiota abundance (genus Ruminococcus gauvreauii group id.11342)' on 'Graves' disease'

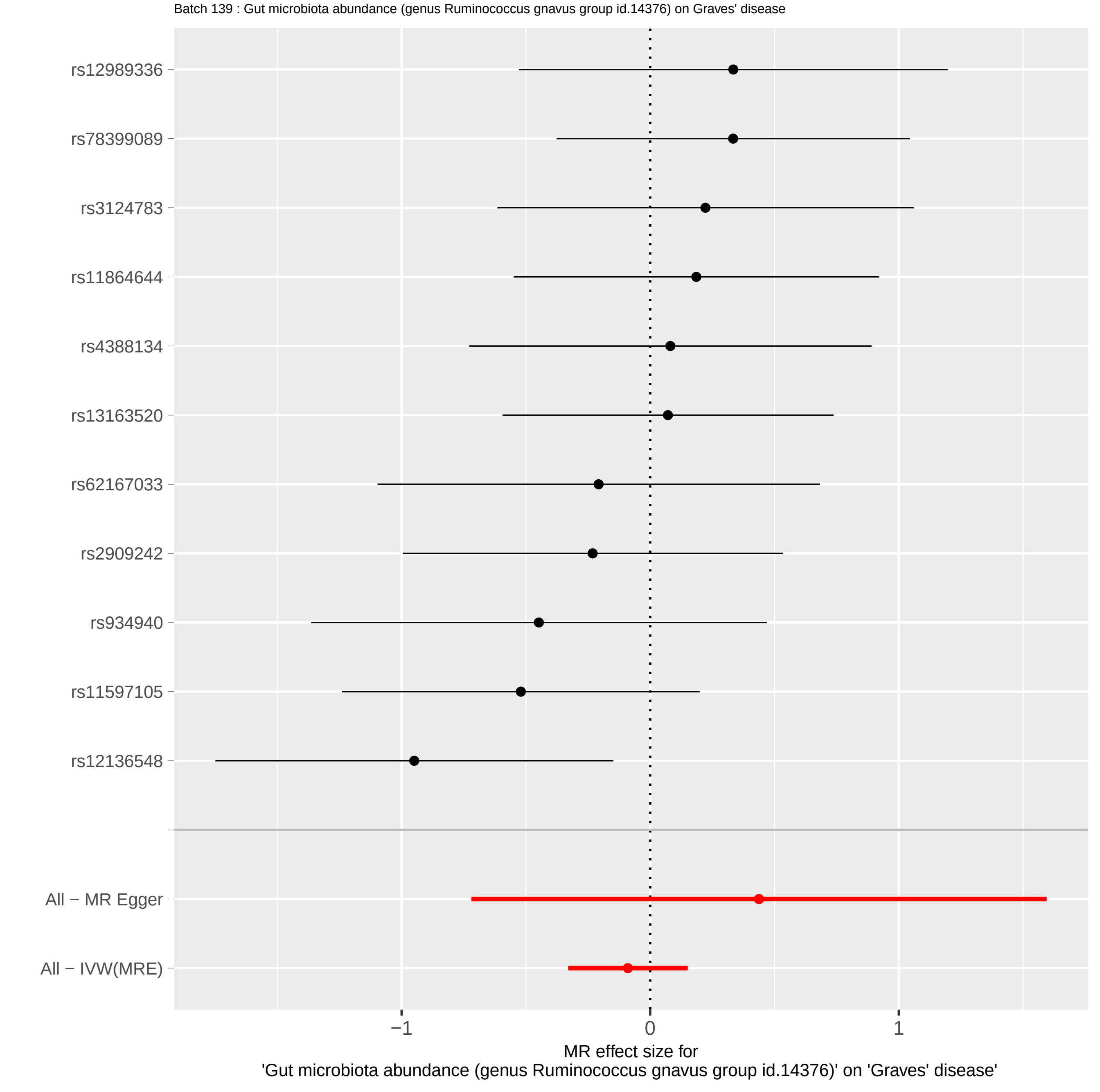
Batch 139 : Gut microbiota abundance (genus Ruminococcus gnavus group id.14376) on Graves' disease
rs12989336
rs78399089
rs3124783
rs11864644
rs4388134
rs13163520
rs62167033
rs2909242
rs934940
rs11597105
rs12136548
All − MR Egger
All − IVW(MRE)
−1
0
1
MR effect size for
'Gut microbiota abundance (genus Ruminococcus gnavus group id.14376)' on 'Graves' disease'

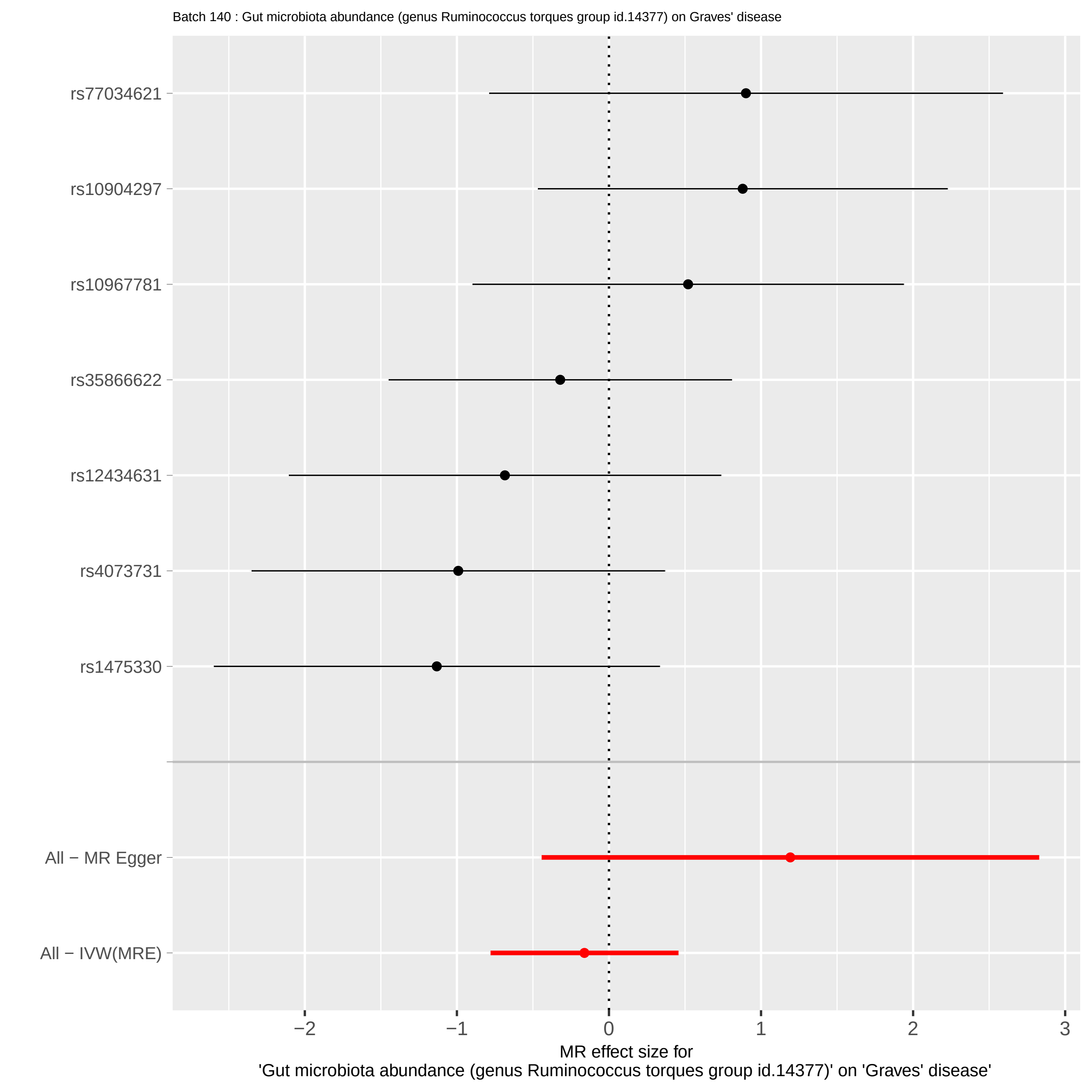

Batch 140 : Gut microbiota abundance (genus Ruminococcus torques group id.14377) on Graves' disease
rs77034621
rs10904297
rs10967781
rs35866622
rs12434631
rs4073731
rs1475330
All − MR Egger
All − IVW(MRE)
−2
−1
0
1
2
3
MR effect size for
'Gut microbiota abundance (genus Ruminococcus torques group id.14377)' on 'Graves' disease'

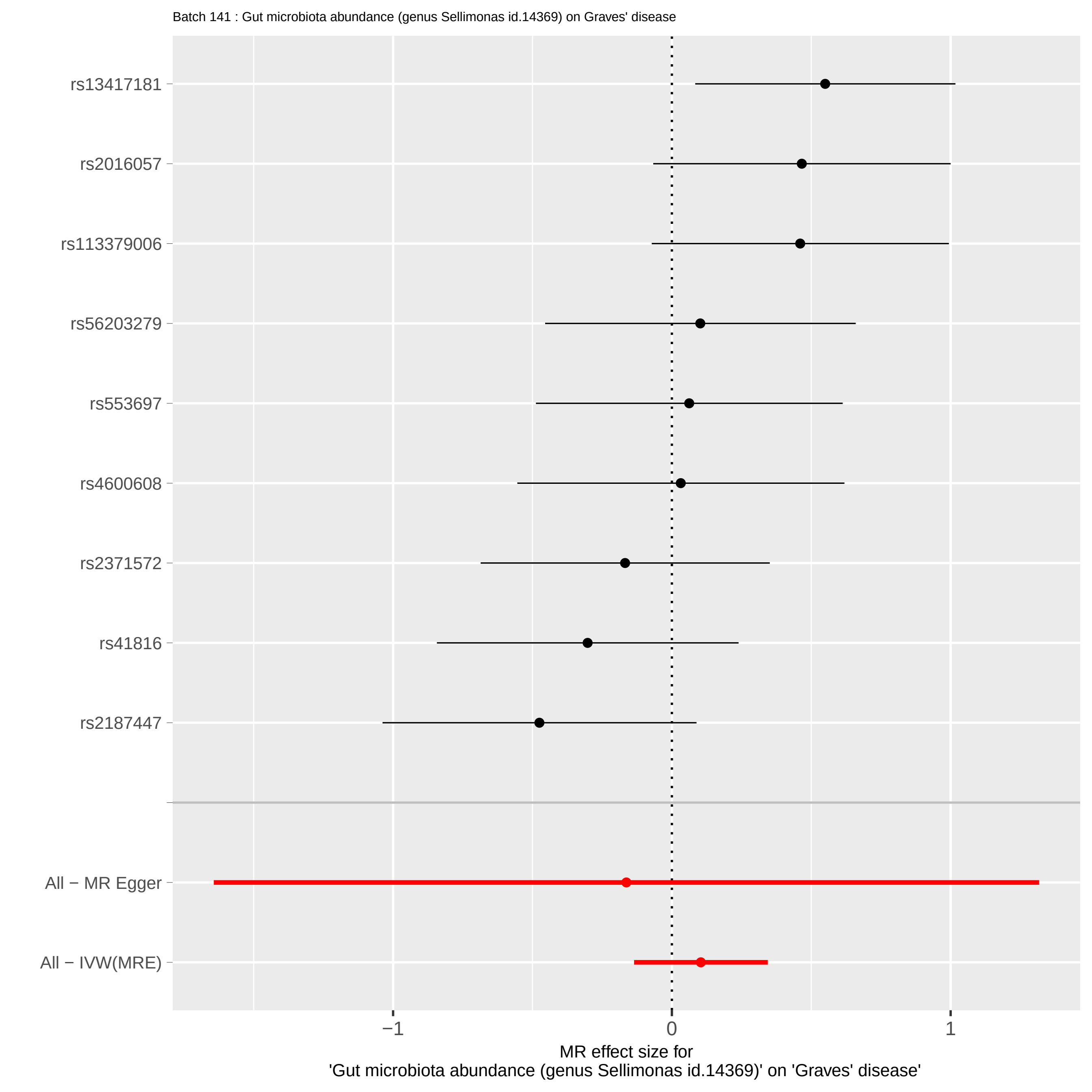

Batch 141 : Gut microbiota abundance (genus Sellimonas id.14369) on Graves' disease
rs13417181
rs2016057
rs113379006
rs56203279
rs553697
rs4600608
rs2371572
rs41816
rs2187447
All − MR Egger
All − IVW(MRE)
−1
0
1
MR effect size for
'Gut microbiota abundance (genus Sellimonas id.14369)' on 'Graves' disease

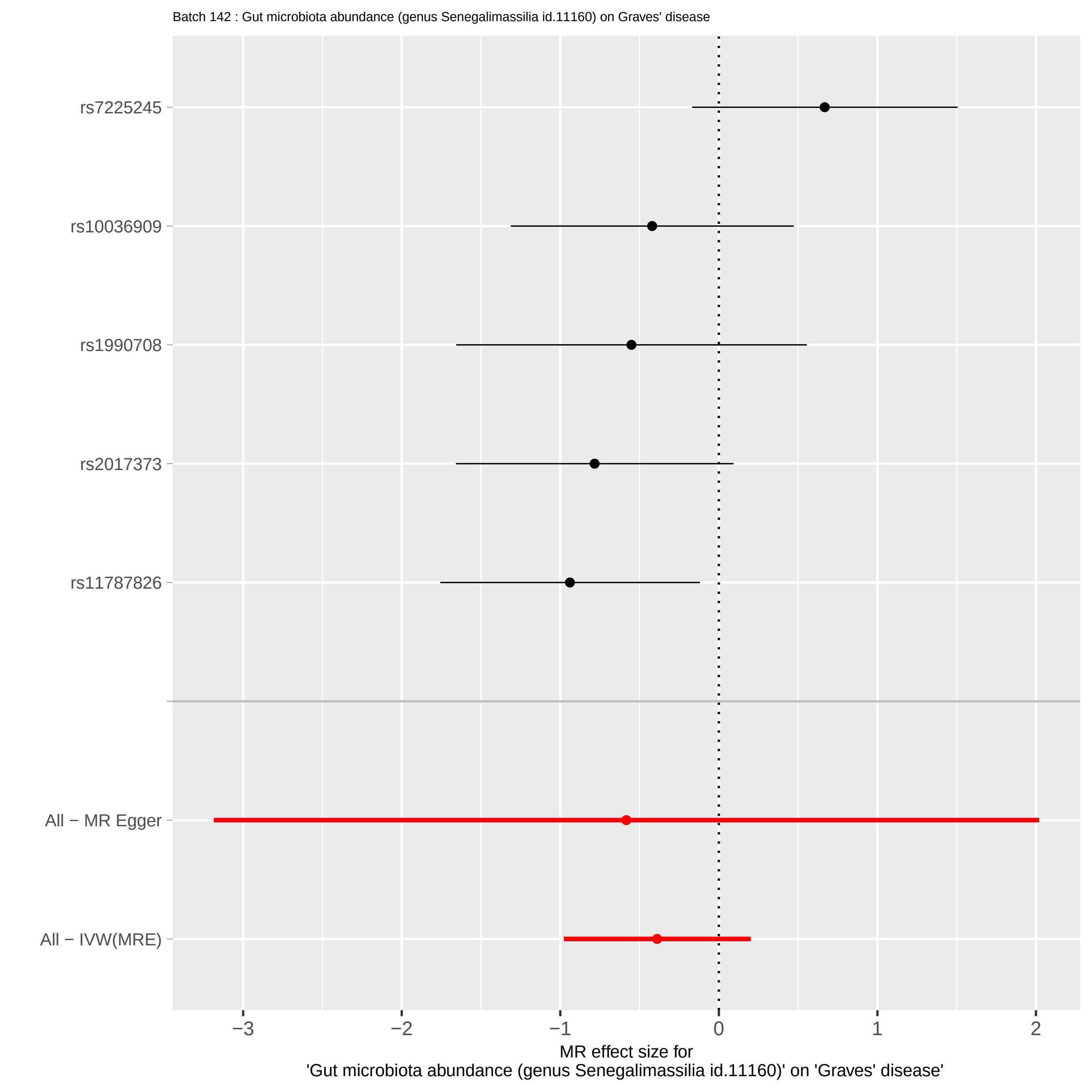

Batch 142 : Gut microbiota abundance (genus Senegalimassilia id.11160) on Graves' disease
rs7225245
rs10036909
rs1990708
rs2017373
rs11787826
All − MR Egger
All − IVW(MRE)
−3
−2
−1
0
1
2
MR effect size for
'Gut microbiota abundance (genus Senegalimassilia id.11160)' on 'Graves' disease'

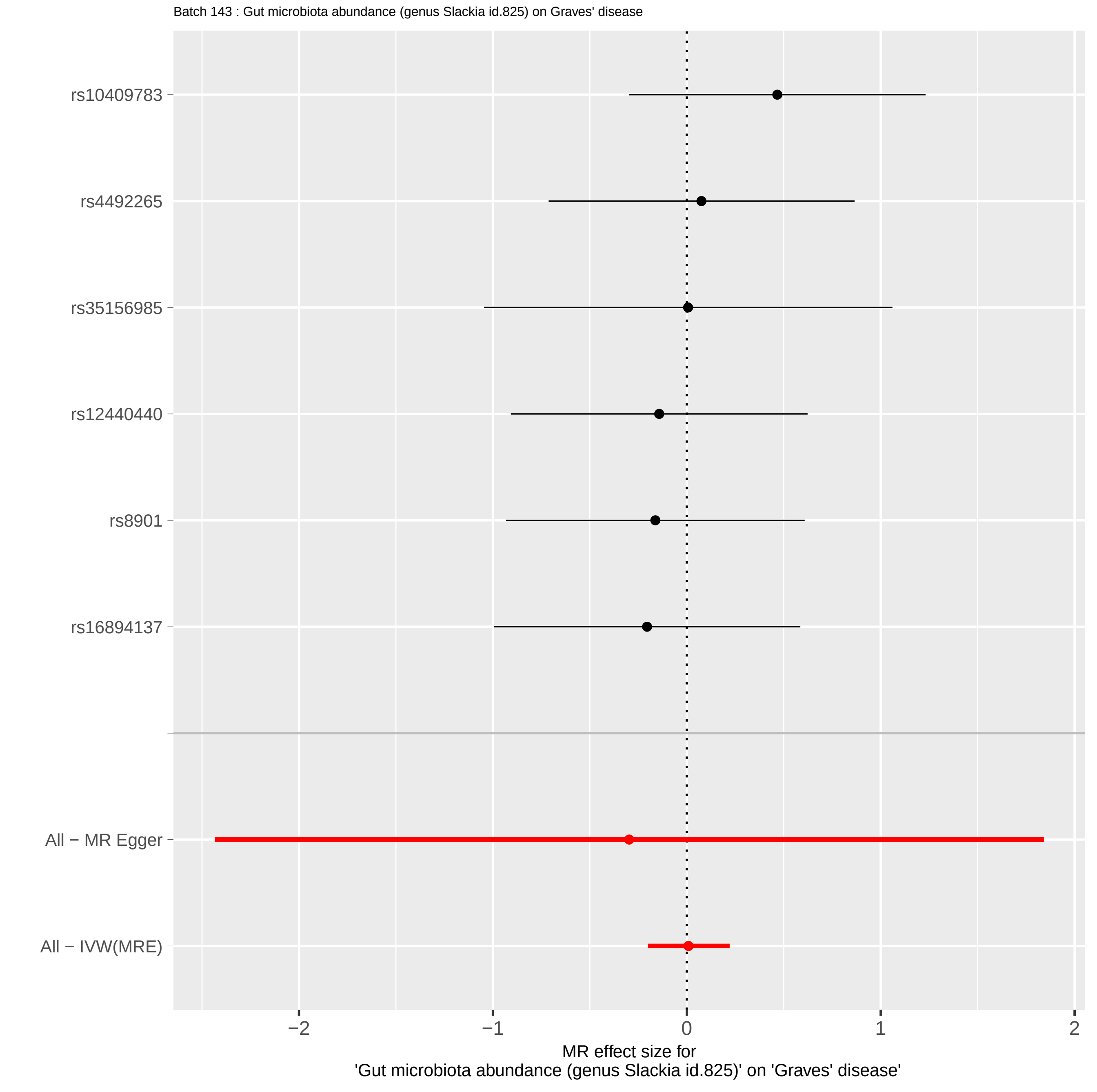

Batch 143 : Gut microbiota abundance (genus Slackia id.825) on Graves' disease
rs10409783
rs4492265
rs35156985
rs12440440
rs8901
rs16894137
All − MR Egger
All − IVW(MRE)
−2
−1
0
1
2
MR effect size for
'Gut microbiota abundance (genus Slackia id.825)' on 'Graves' disease'

Batch 144 : Gut microbiota abundance (genus Streptococcus id.1853) on Graves' disease

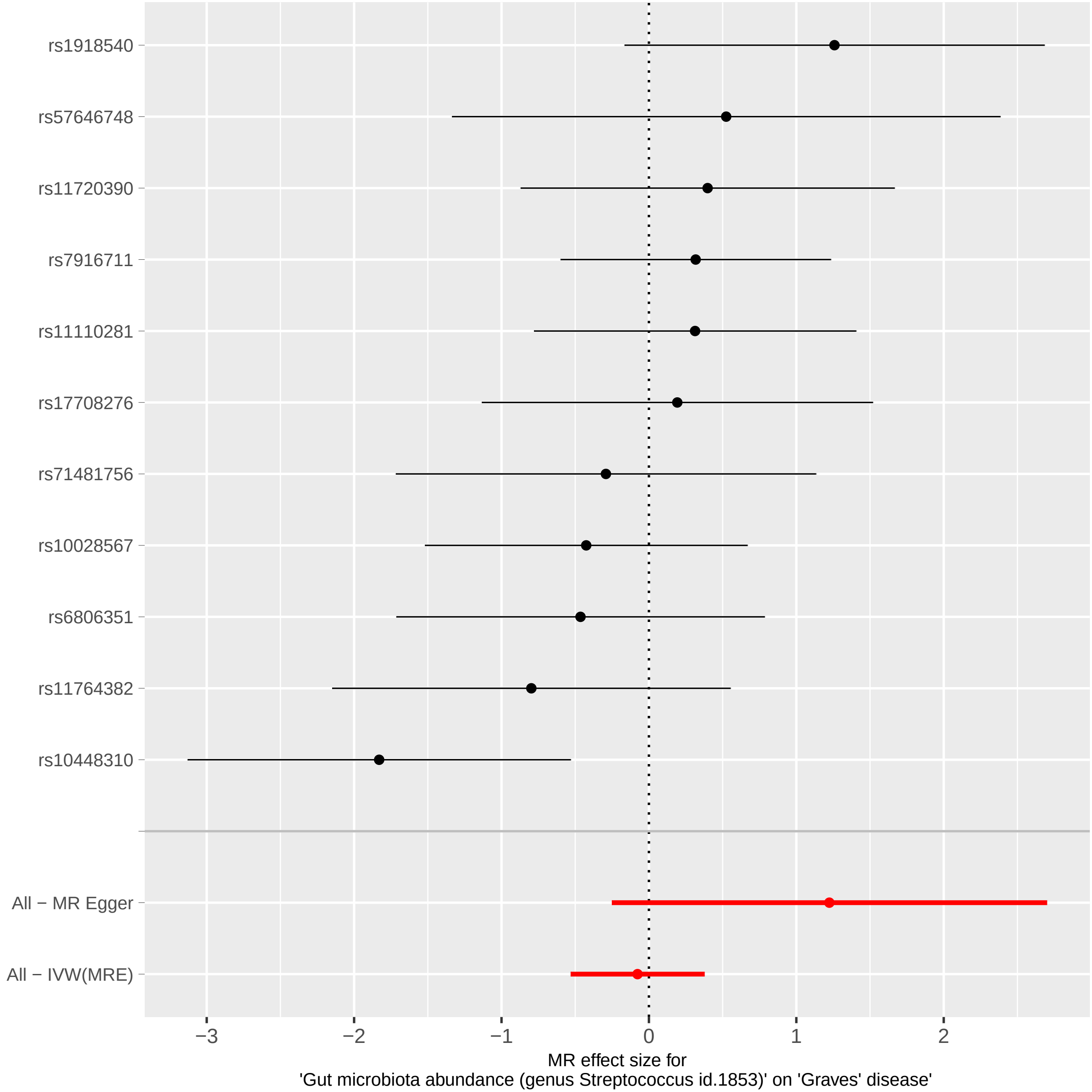

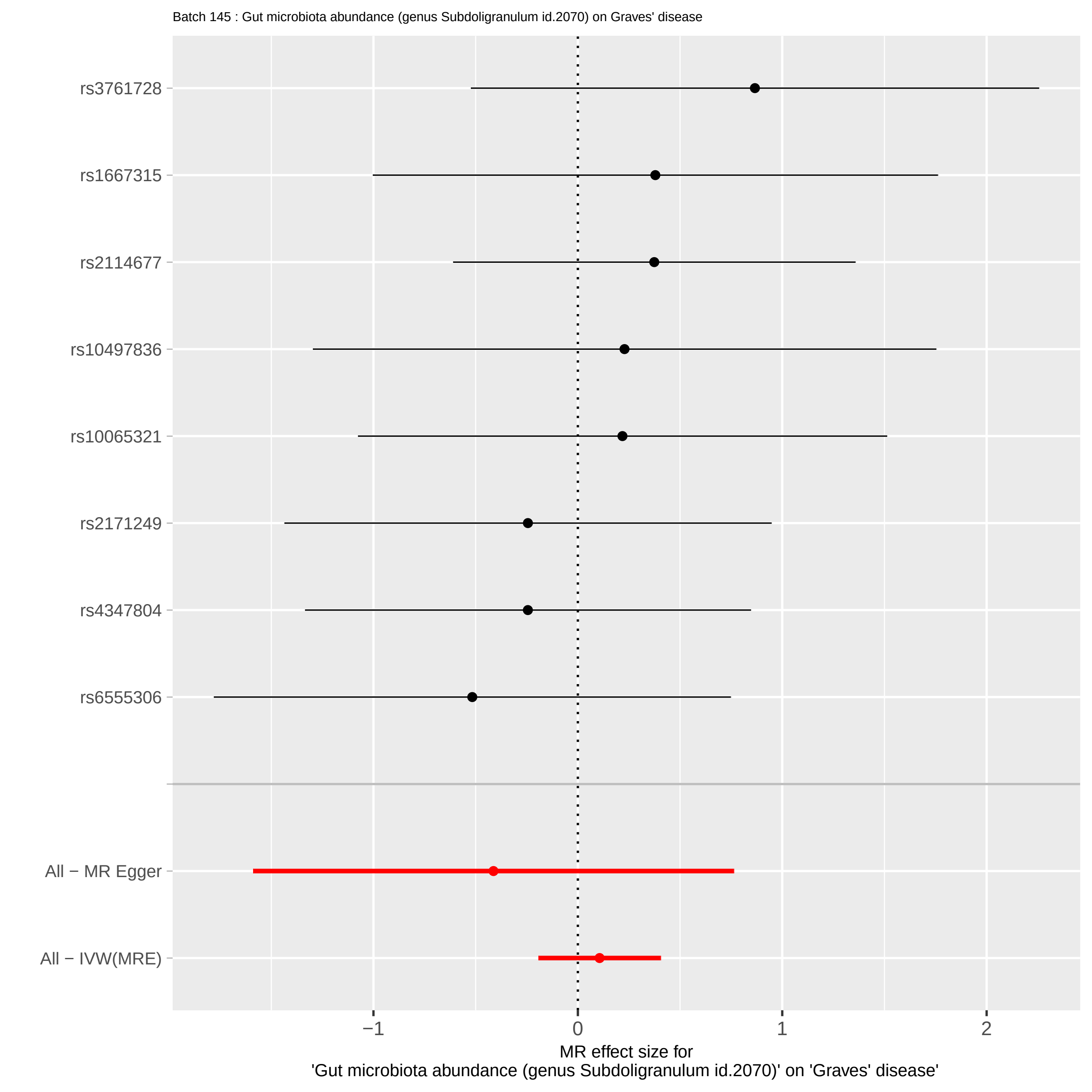

Batch 145 : Gut microbiota abundance (genus Subdoligranulum id.2070) on Graves' disease
rs3761728
rs1667315
rs2114677
rs10497836
rs10065321
rs2171249
rs4347804
rs6555306
All − MR Egger
All − IVW(MRE)
−1
0
1
2
MR effect size for
'Gut microbiota abundance (genus Subdoligranulum id.2070)' on 'Graves' disease'

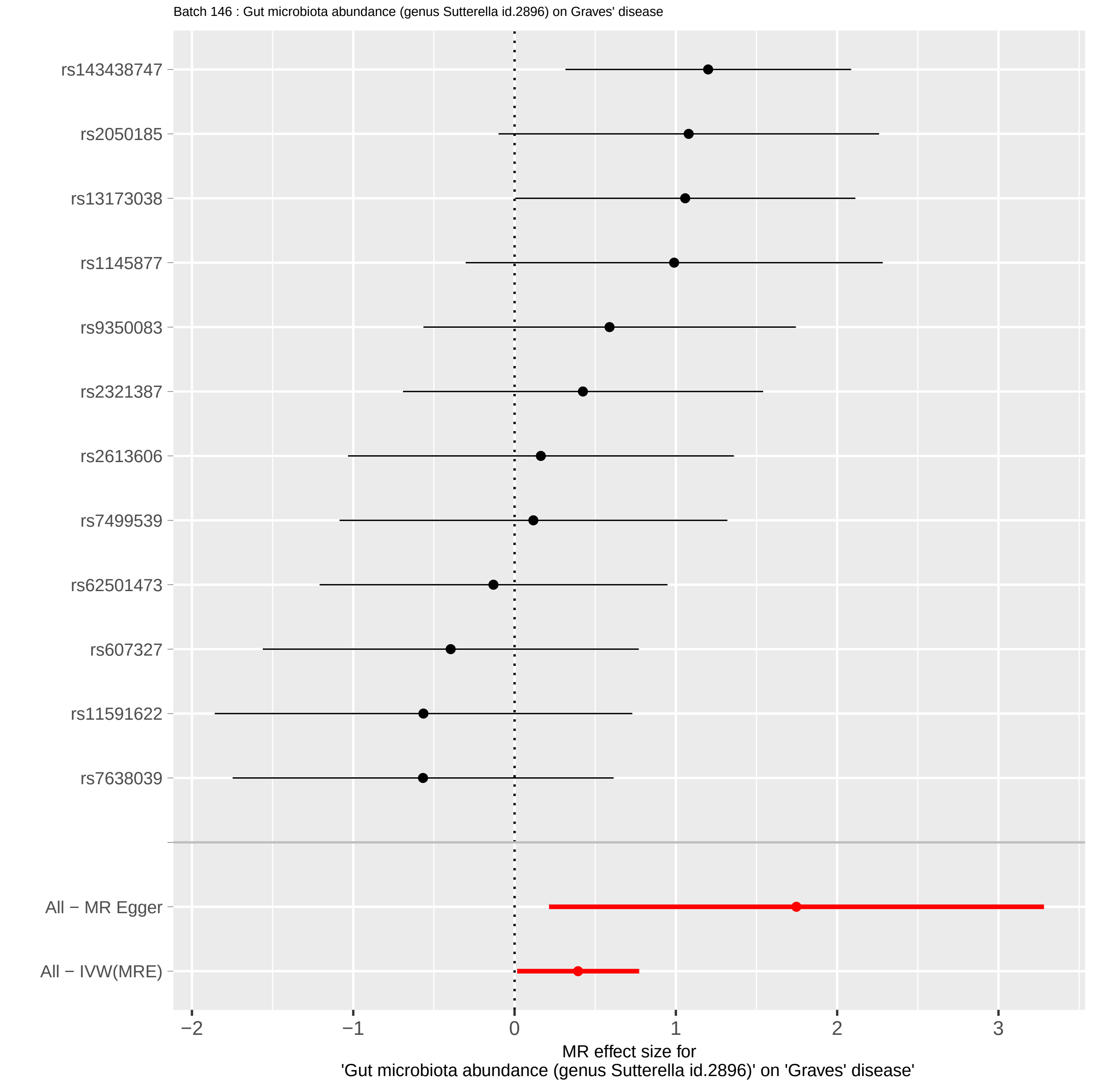
Batch 146 : Gut microbiota abundance (genus Sutterella id.2896) on Graves' disease
rs143438747
rs2050185
rs13173038
rs1145877
rs9350083
rs2321387
rs2613606
rs7499539
rs62501473
rs607327
rs11591622
rs7638039
All − MR Egger
All − IVW(MRE)
−2
−1
0
1
2
3
MR effect size for
'Gut microbiota abundance (genus Sutterella id.2896)' on 'Graves' disease'

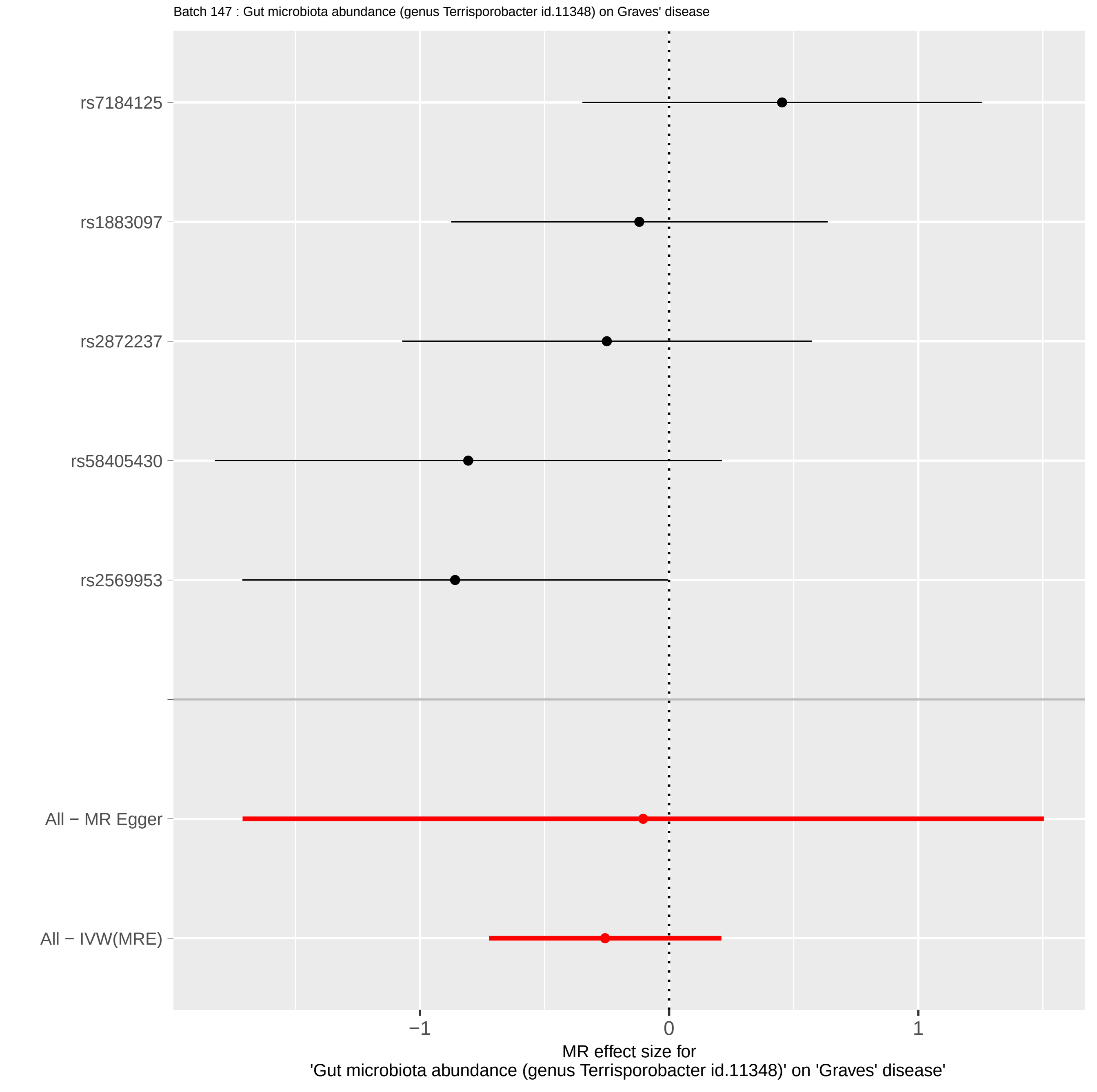
Batch 147 : Gut microbiota abundance (genus Terrisporobacter id.11348) on Graves' disease
rs7184125
rs1883097
rs2872237
rs58405430
rs2569953
All − MR Egger
All − IVW(MRE)
−1
0
1
MR effect size for
'Gut microbiota abundance (genus Terrisporobacter id.11348)' on 'Graves' disease'

Batch 148 : Gut microbiota abundance (genus Turicibacter id.2162) on Graves' disease

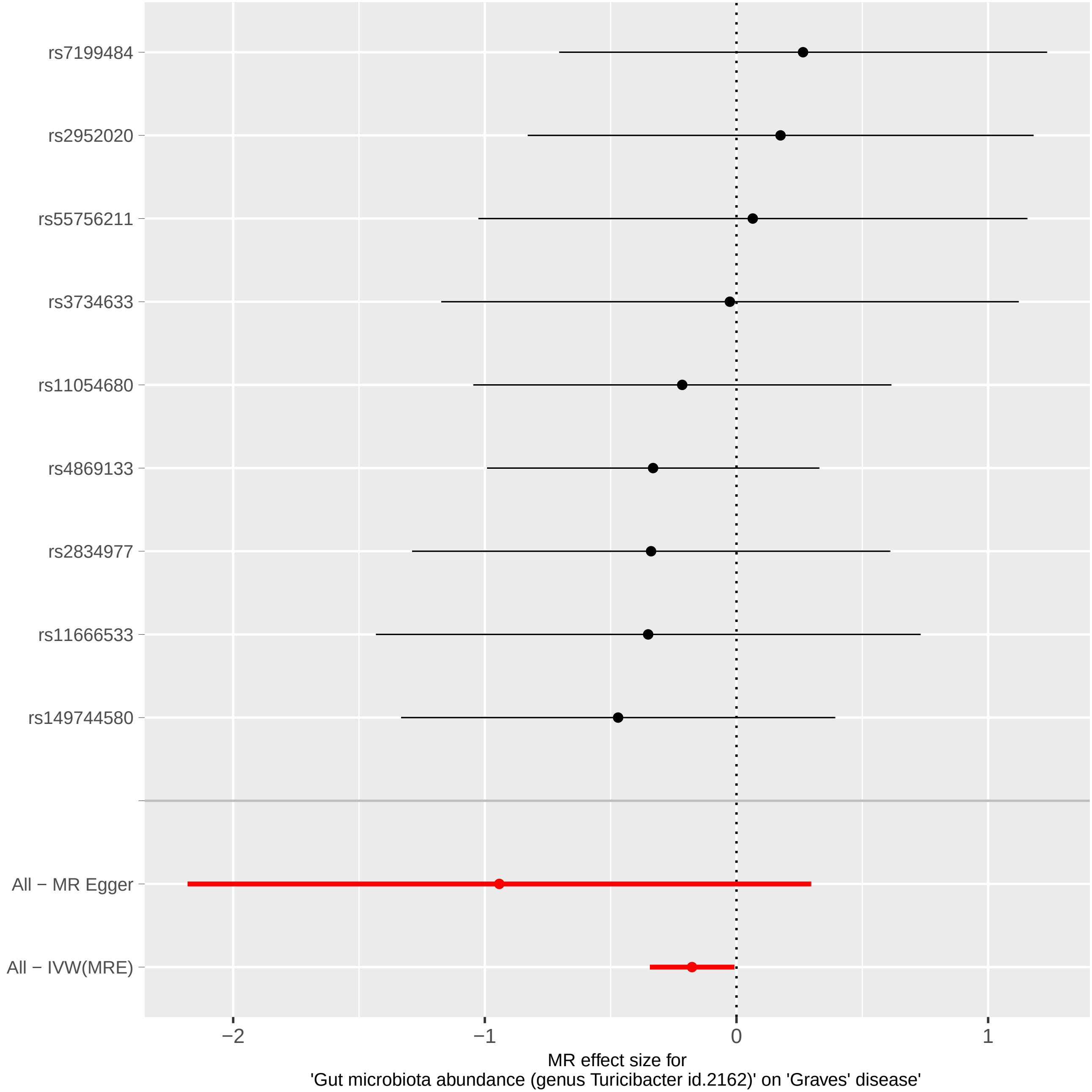

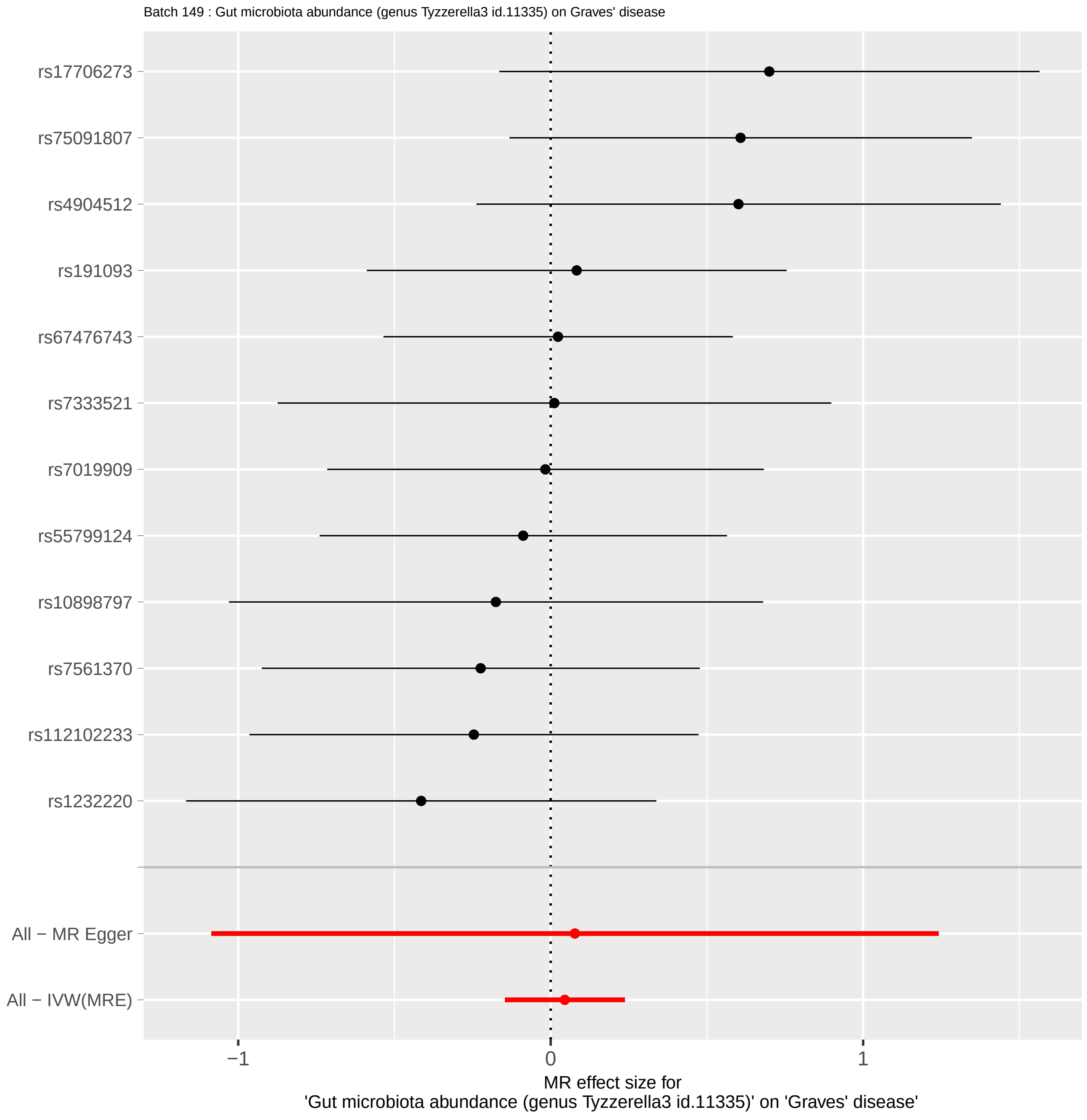
Batch 149 : Gut microbiota abundance (genus Tyzzerella3 id.11335) on Graves' disease
rs17706273
rs75091807
rs4904512
rs191093
rs67476743
rs7333521
rs7019909
rs55799124
rs10898797
rs7561370
rs112102233
rs1232220
All − MR Egger
All − IVW(MRE)
−1
0
1
MR effect size for
'Gut microbiota abundance (genus Tyzzerella3 id.11335)' on 'Graves' disease'

Batch 150 : Gut microbiota abundance (genus Veillonella id.2198) on Graves' disease

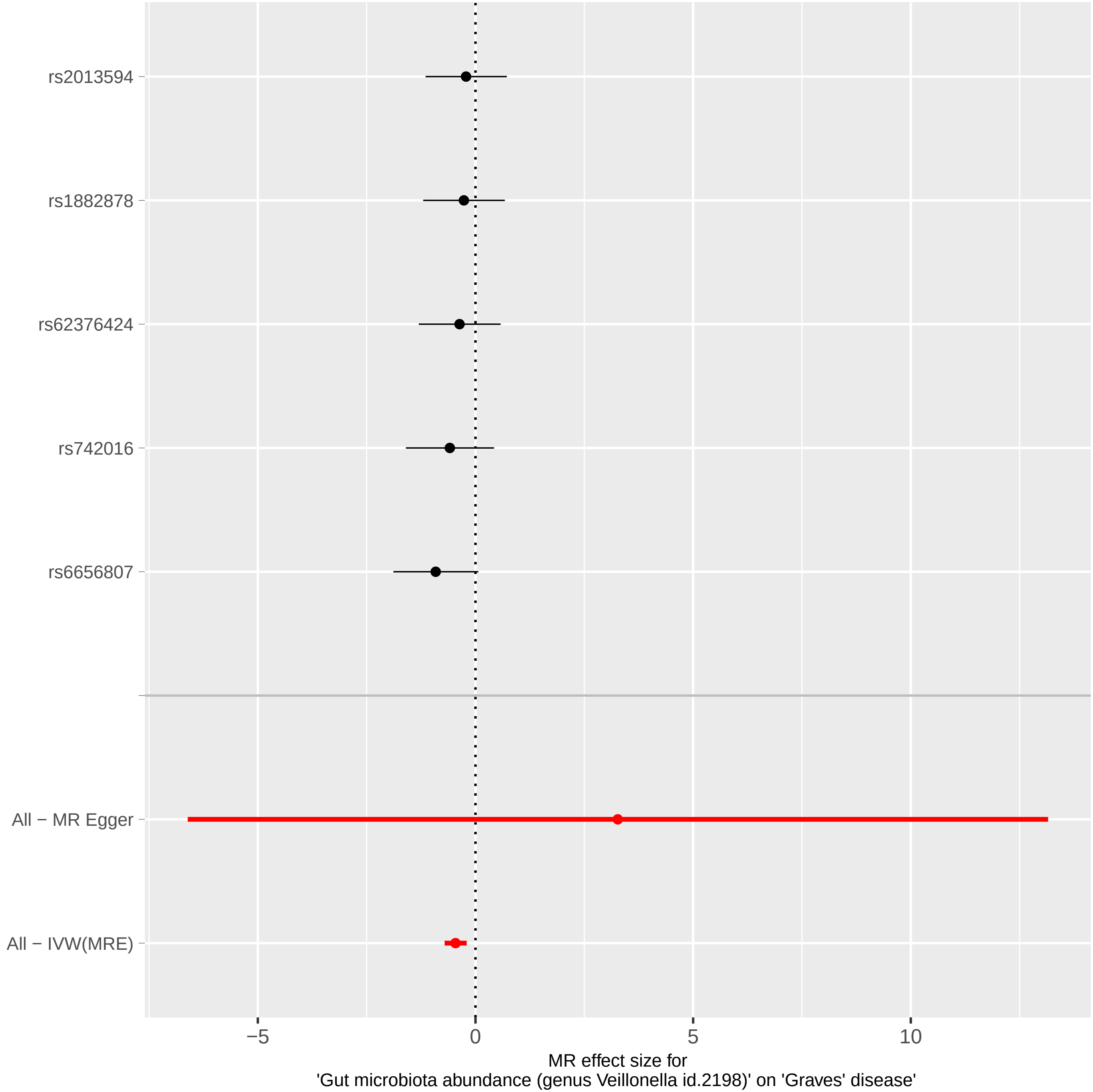

Supplement: Supplementary file 1 [file DataSheet_1.pdf]
