# Supplementary material for: Bacteroidaceae, Bacteroides, and Veillonella: emerging protectors against Graves’ disease
Source: Front Cell Infect Microbiol. 2024 Feb 9;14:1288222. doi: 10.3389/fcimb.2024.1288222 (PMC10884117; doi:10.3389/fcimb.2024.1288222)
Supplement: Supplementary file 2 [file DataSheet_2.pdf]

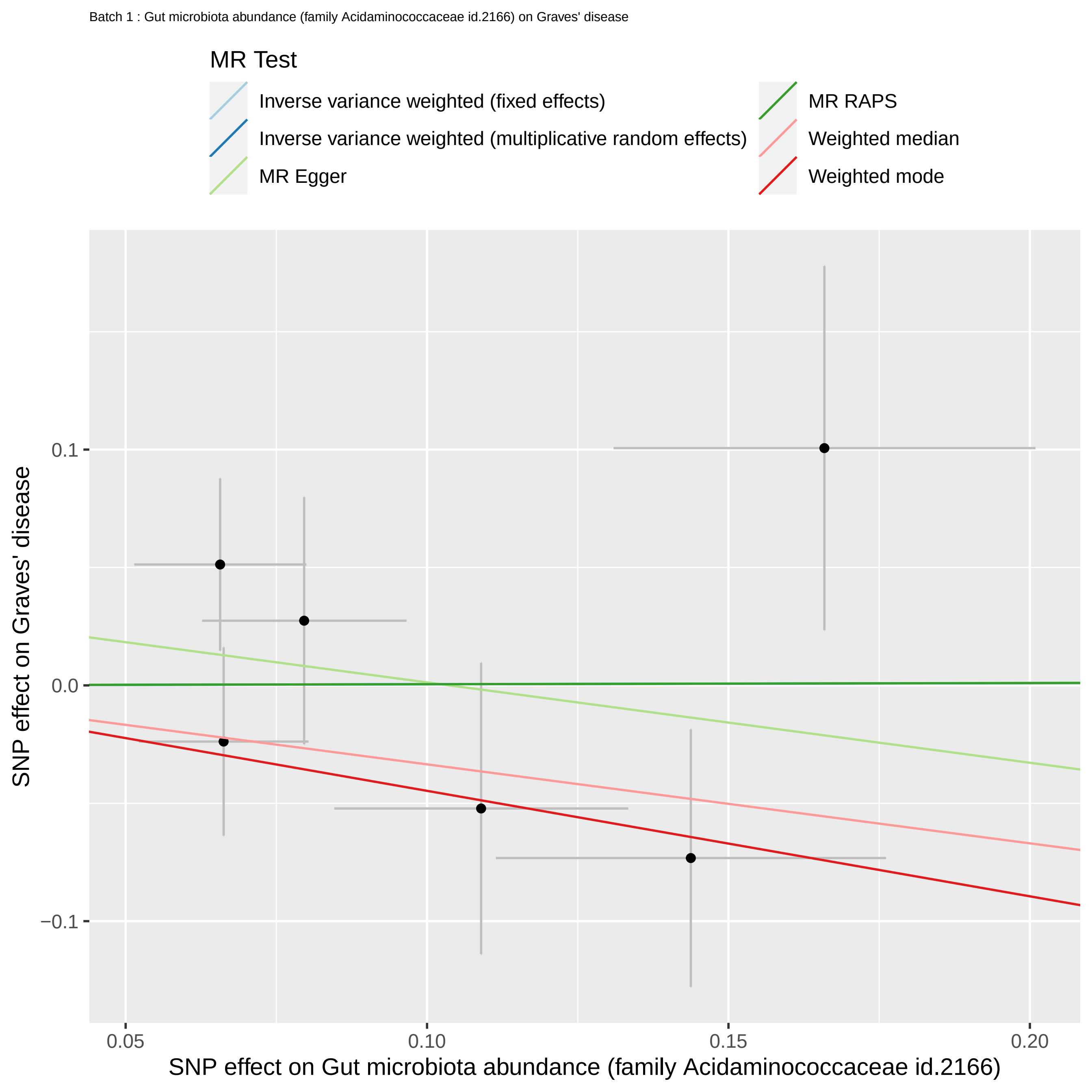

Batch 1 : Gut microbiota abundance (family Acidaminococcaceae id.2166) on Graves' disease
MR Test
Inverse variance weighted (fixed effects)
Inverse variance weighted (multiplicative random effects)
MR Egger
MR RAPS
Weighted median
Weighted mode
0.1
0.0
−0.1
SNP effect on Graves' disease
0.05
0.10
0.15
0.20
SNP effect on Gut microbiota abundance (family Acidaminococcaceae id.2166)

Batch 2 : Gut microbiota abundance (family Actinomycetaceae id.421) on Graves' disease

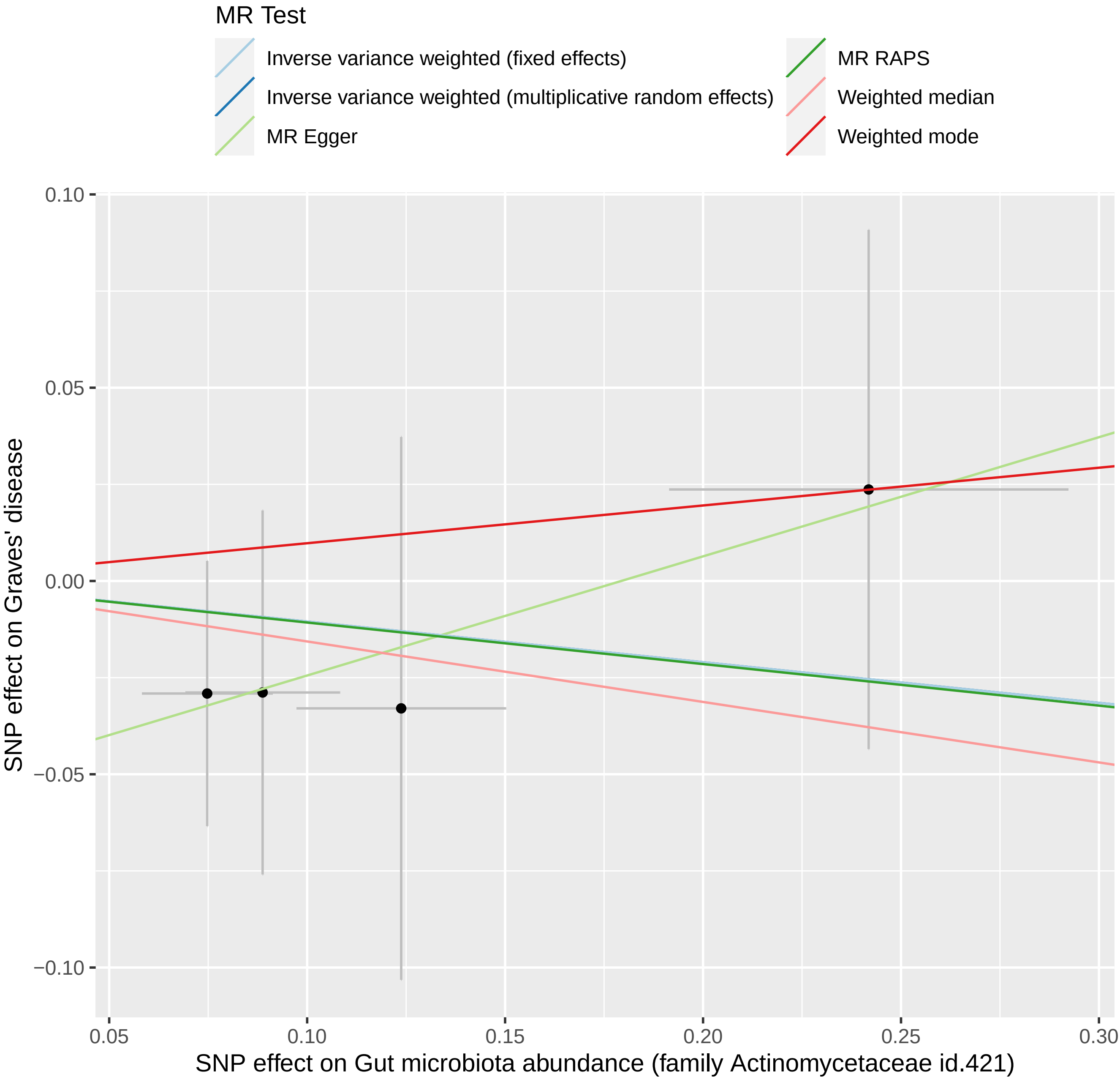

Batch 3 : Gut microbiota abundance (family Alcaligenaceae id.2875) on Graves' disease

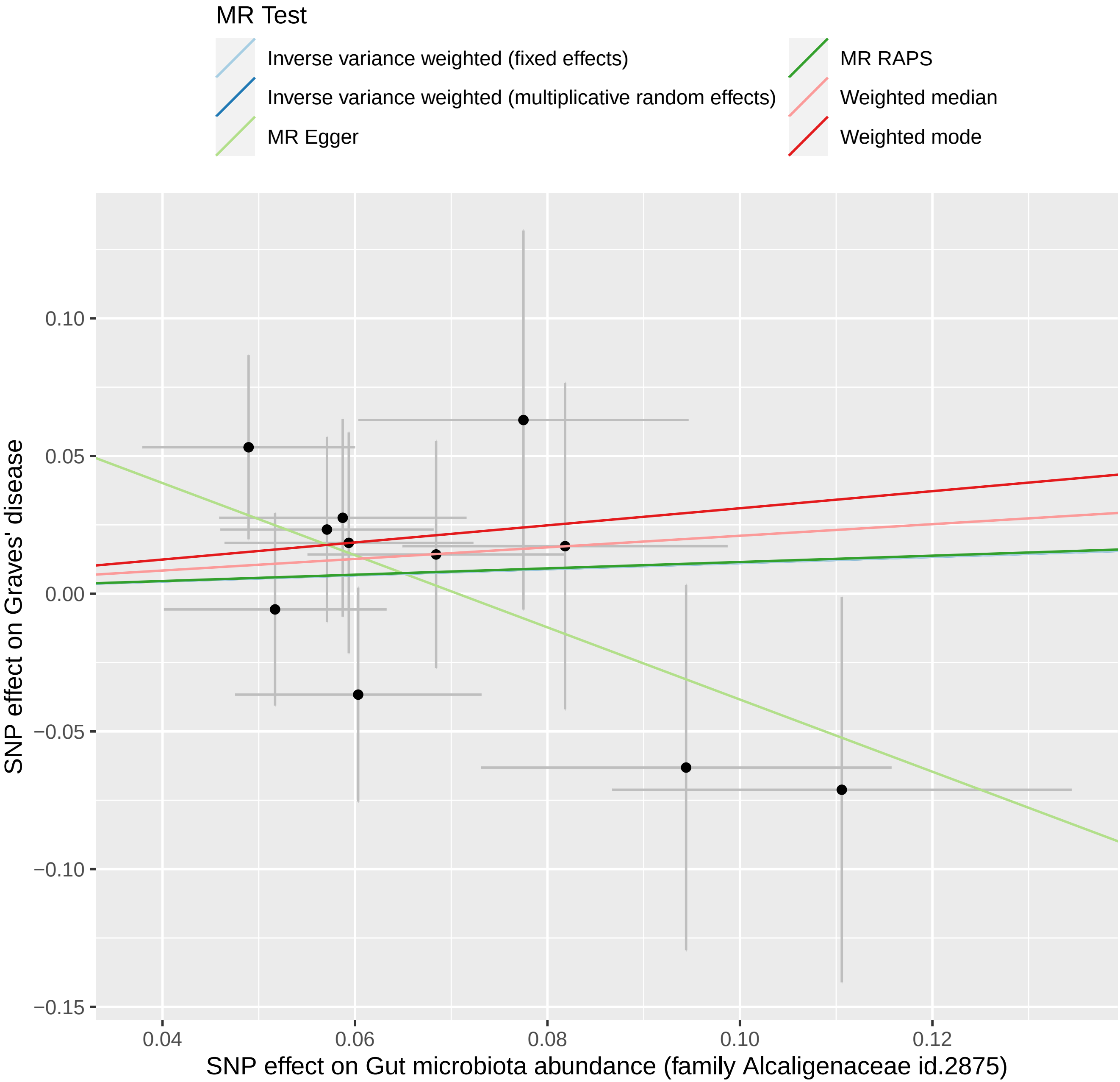

Batch 4 : Gut microbiota abundance (family Bacteroidaceae id.917) on Graves' disease

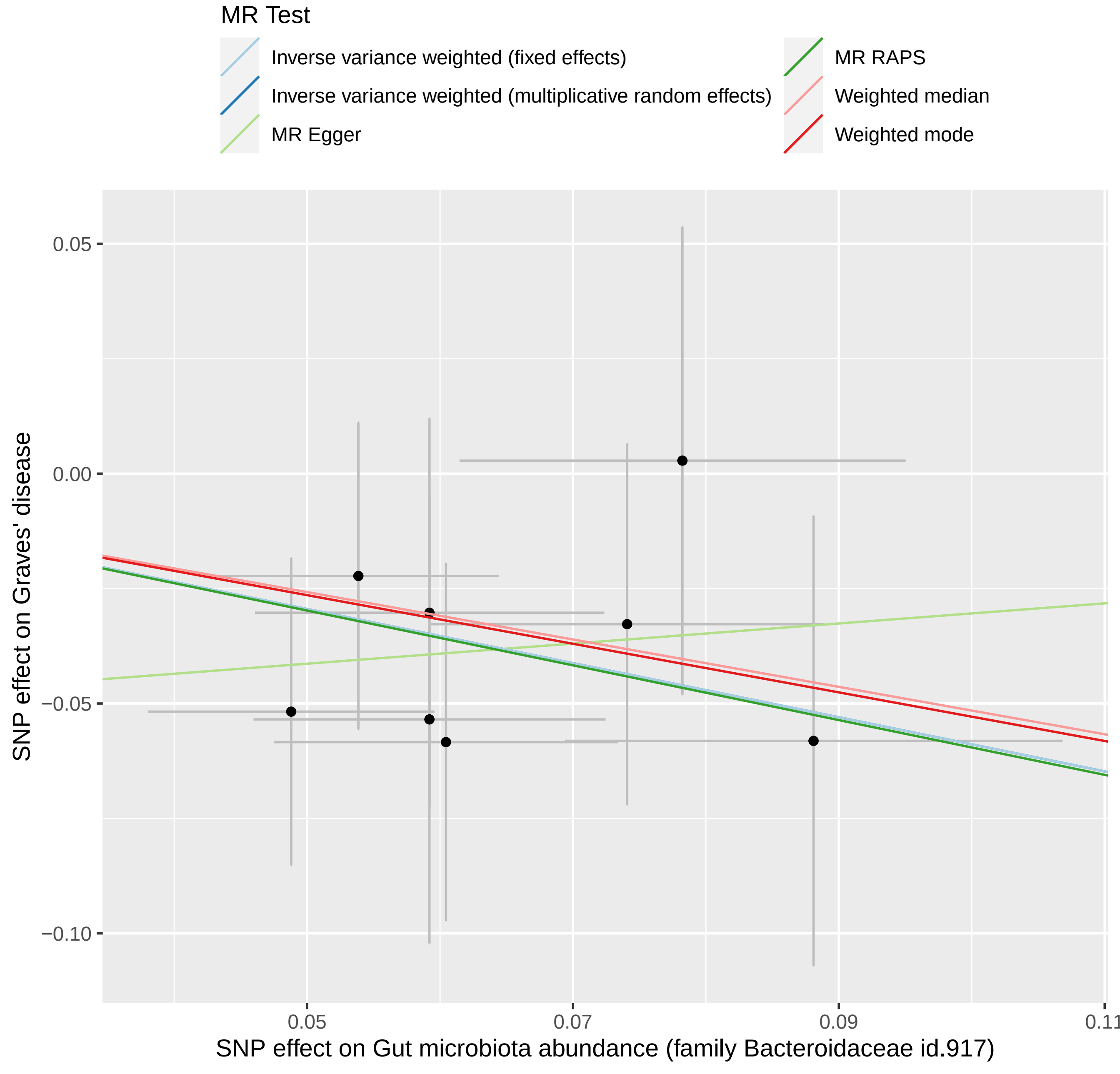

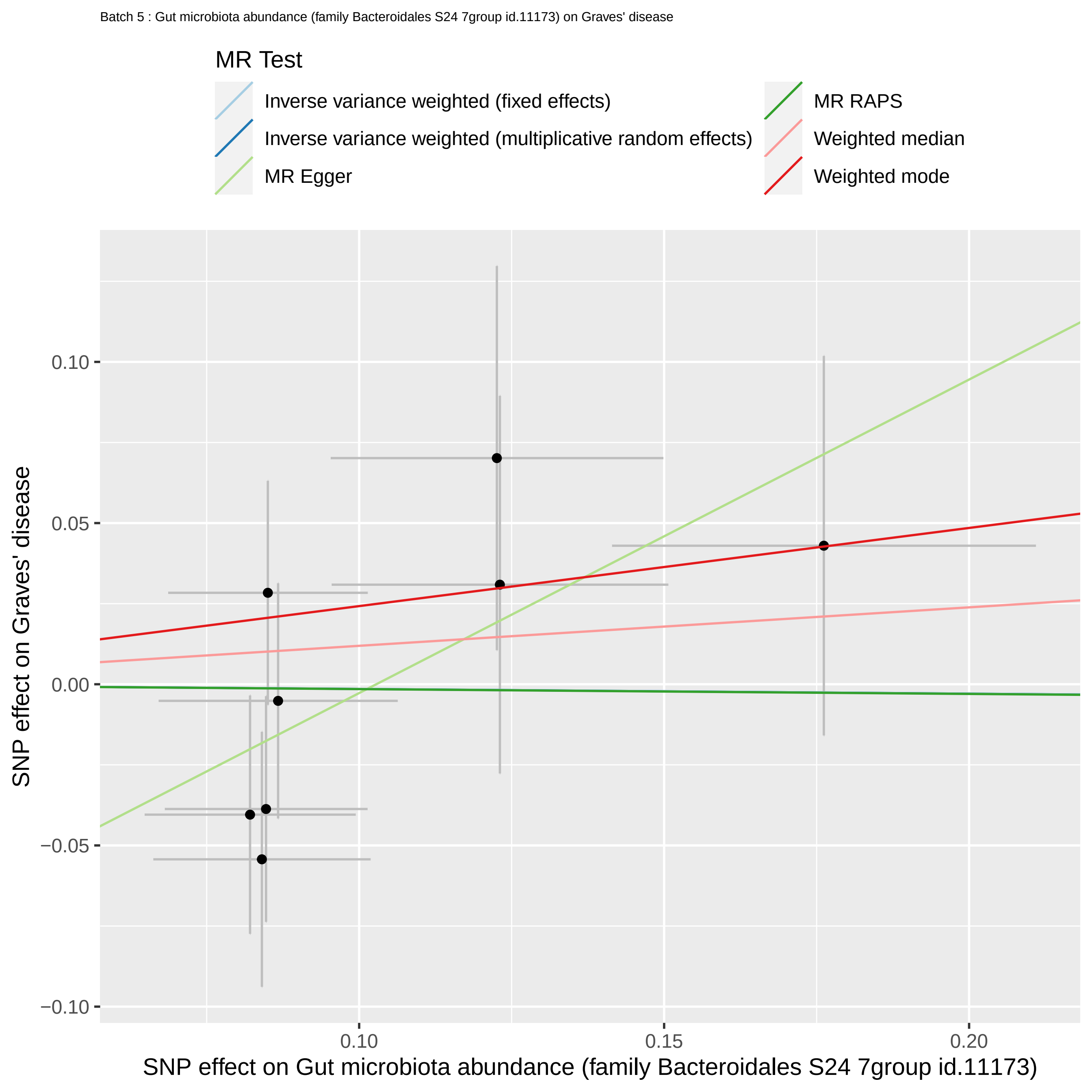

Batch 5 : Gut microbiota abundance (family Bacteroidales S24 7group id.11173) on Graves' disease
MR Test
Inverse variance weighted (fixed effects)
Inverse variance weighted (multiplicative random effects)
MR Egger
MR RAPS
Weighted median
Weighted mode
0.10
0.05
0.00
−0.05
−0.10
SNP effect on Graves' disease
0.10
0.15
0.20
SNP effect on Gut microbiota abundance (family Bacteroidales S24 7group id.11173)

Batch 6 : Gut microbiota abundance (family Bifidobacteriaceae id.433) on Graves' disease

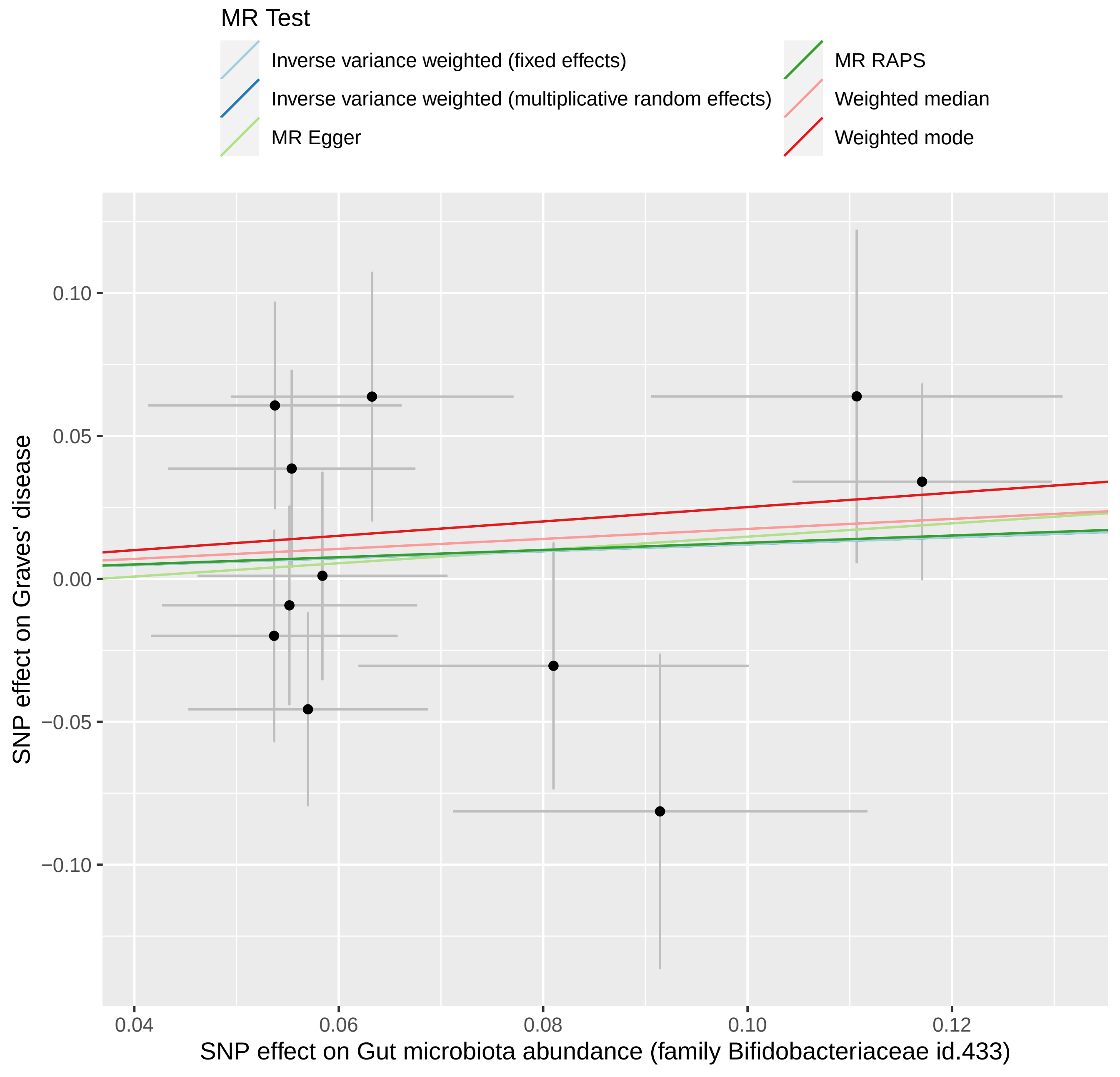

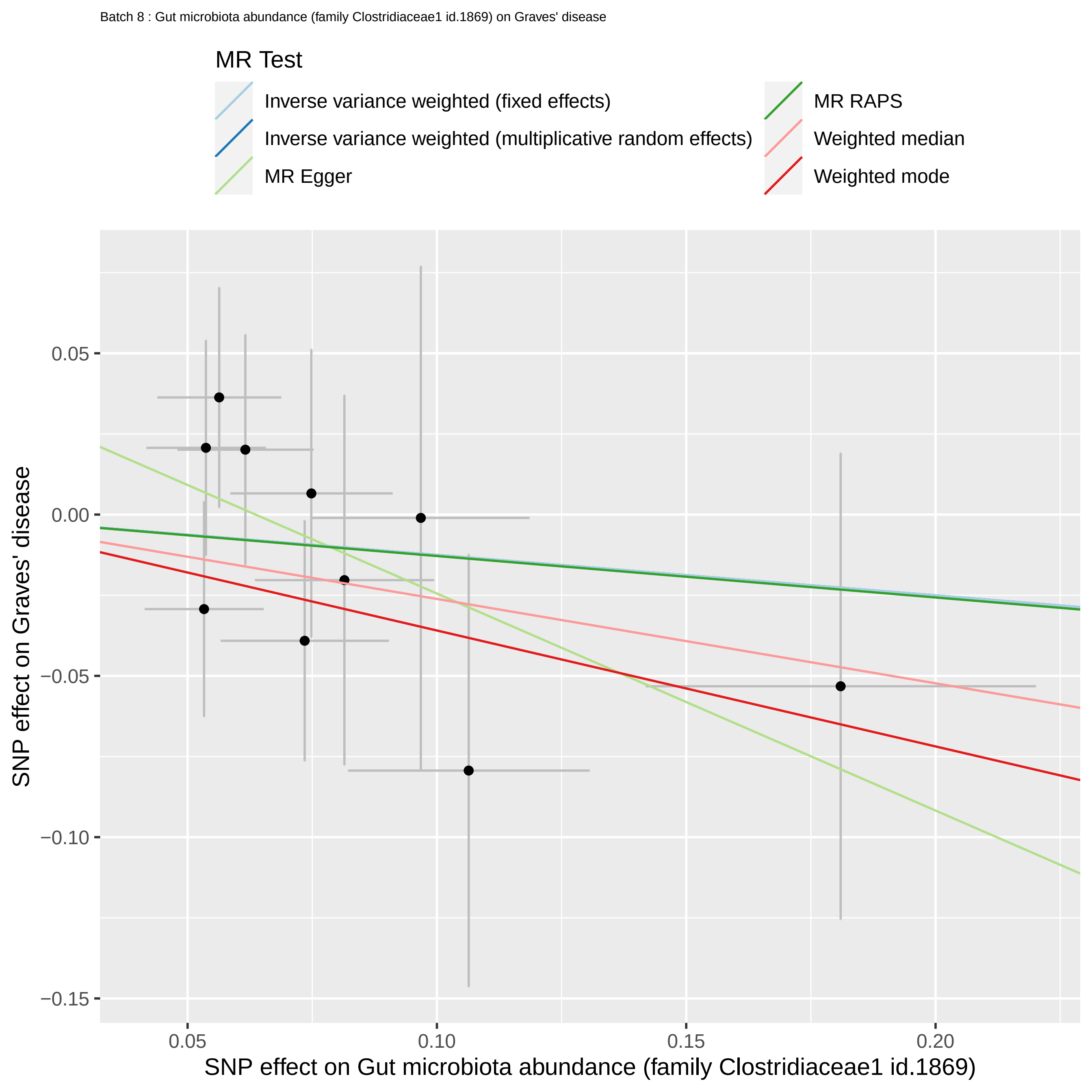
Batch 8 : Gut microbiota abundance (family Clostridiaceae1 id.1869) on Graves' disease
MR Test
Inverse variance weighted (fixed effects)
Inverse variance weighted (multiplicative random effects)
MR Egger
MR RAPS
Weighted median
Weighted mode
0.05
0.00
−0.05
−0.10
−0.15
0.05
0.10
0.15
0.20
SNP effect on Graves' disease
SNP effect on Gut microbiota abundance (family Clostridiaceae1 id.1869)

Batch 9 : Gut microbiota abundance (family Clostridiales vadin BB60 group id.11286) on Graves' disease

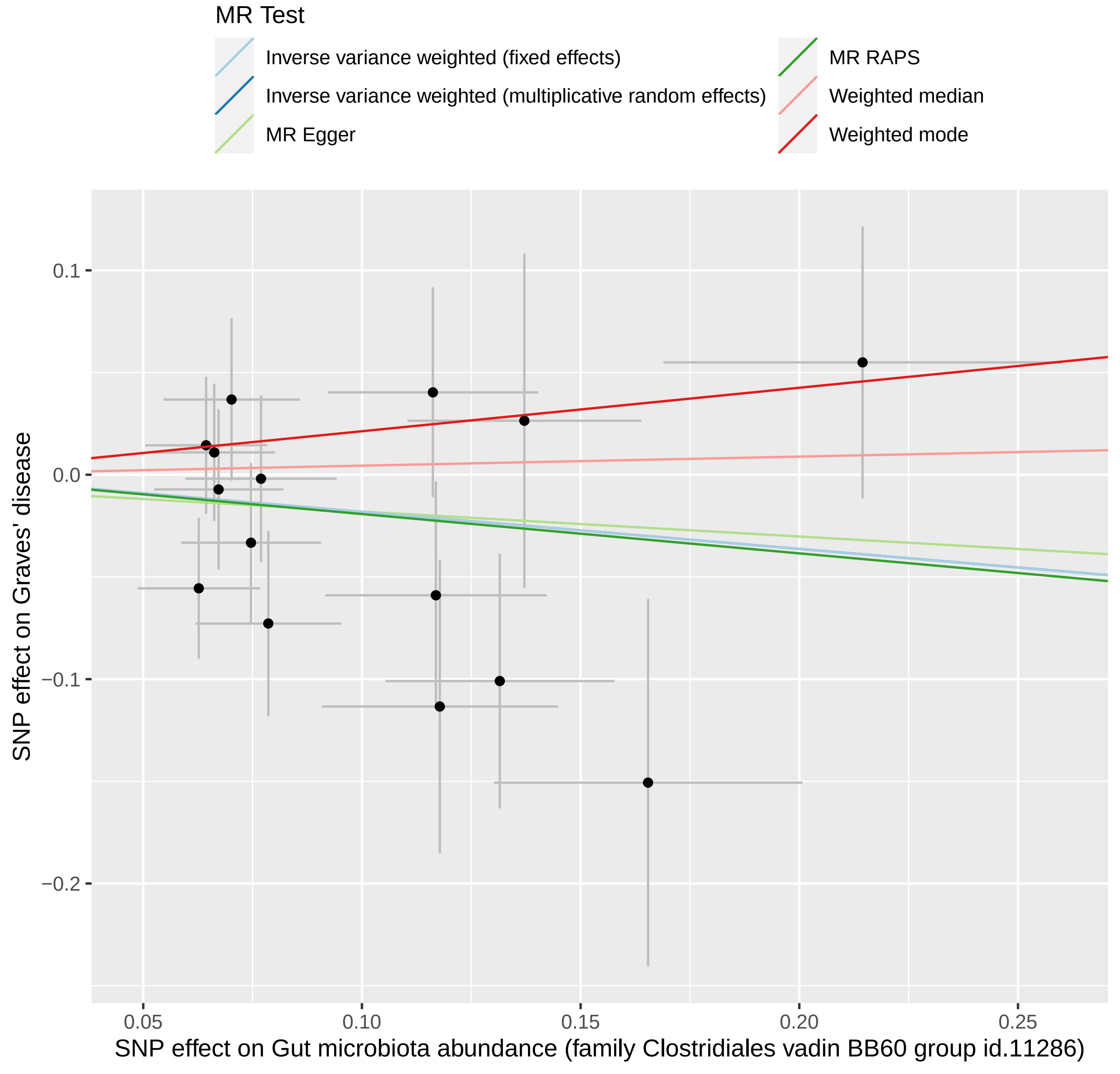

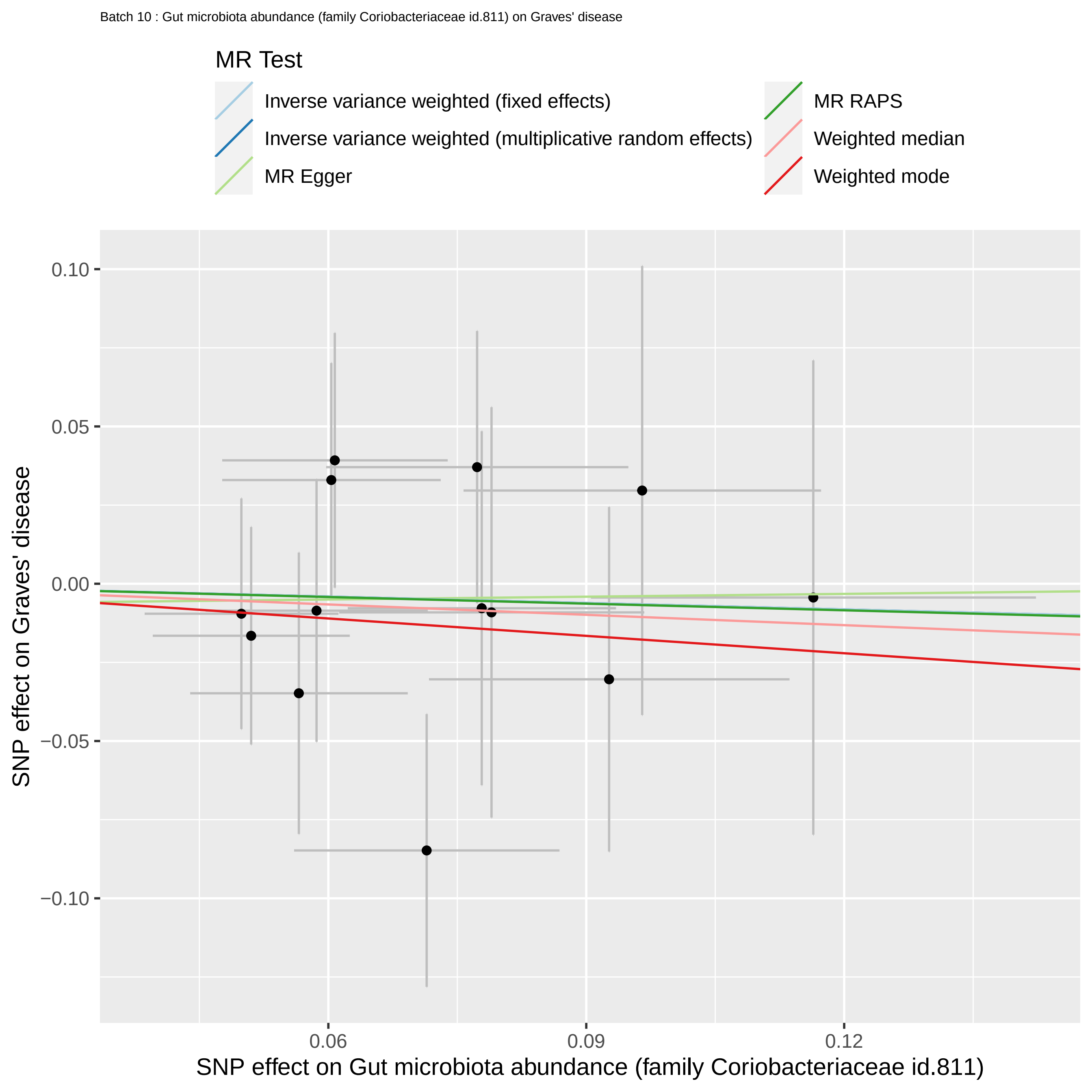

Batch 10 : Gut microbiota abundance (family Coriobacteriaceae id.811) on Graves' disease
MR Test
Inverse variance weighted (fixed effects)
Inverse variance weighted (multiplicative random effects)
MR Egger
MR RAPS
Weighted median
Weighted mode
0.10
0.05
0.00
−0.05
−0.10
SNP effect on Graves' disease
0.06
0.09
0.12
SNP effect on Gut microbiota abundance (family Coriobacteriaceae id.811)

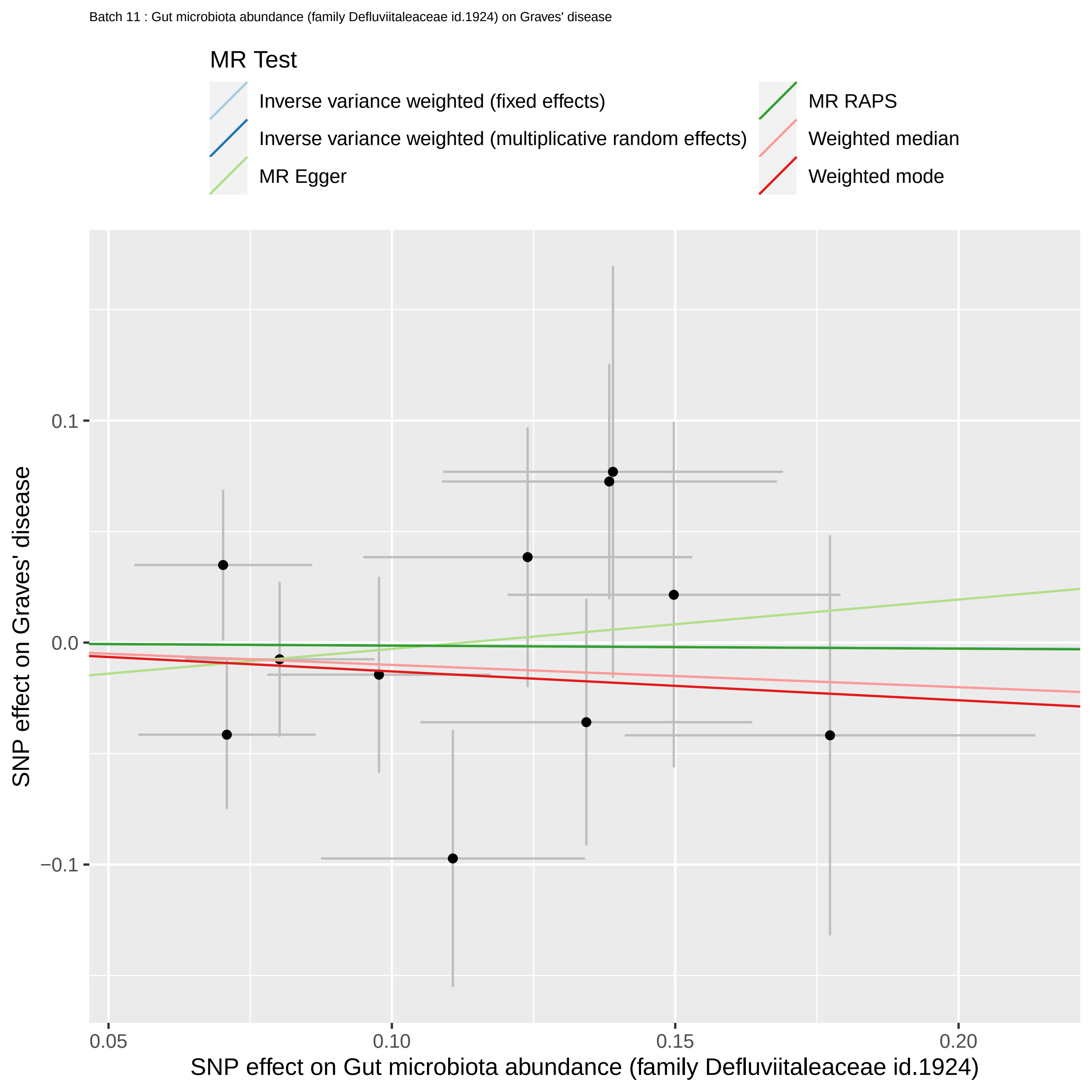
Batch 11 : Gut microbiota abundance (family Defluviitaleaceae id.1924) on Graves' disease
MR Test
Inverse variance weighted (fixed effects)
Inverse variance weighted (multiplicative random effects)
MR Egger
MR RAPS
Weighted median
Weighted mode
0.1
0.0
−0.1
SNP effect on Graves' disease
0.05
0.10
0.15
0.20
SNP effect on Gut microbiota abundance (family Defluviitaleaceae id.1924)

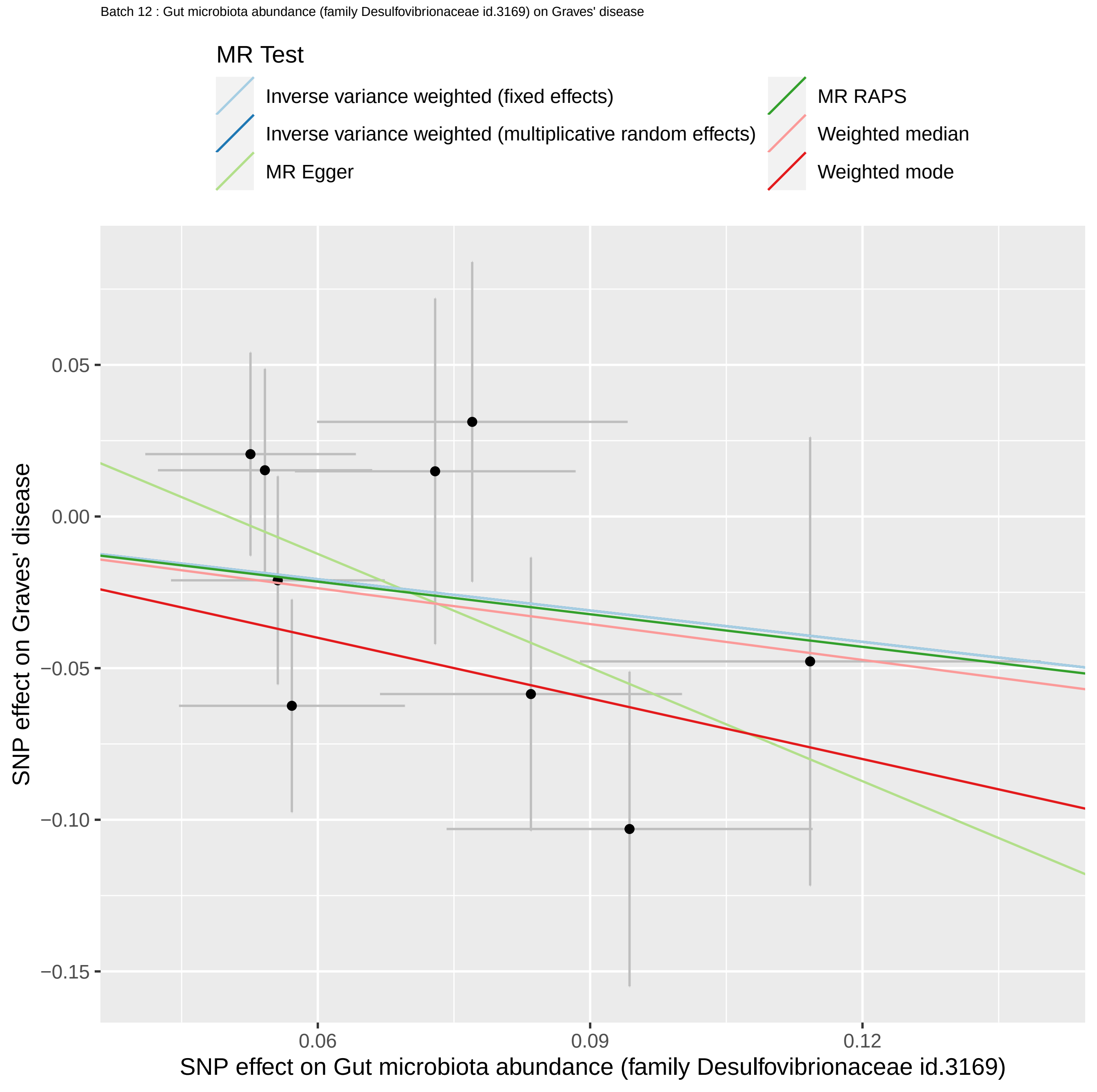
Batch 12 : Gut microbiota abundance (family Desulfovibrionaceae id.3169) on Graves' disease
MR Test
Inverse variance weighted (fixed effects)
Inverse variance weighted (multiplicative random effects)
MR Egger
MR RAPS
Weighted median
Weighted mode
SNP effect on Graves' disease
0.05
0.00
−0.05
−0.10
−0.15
0.06
0.09
0.12
SNP effect on Gut microbiota abundance (family Desulfovibrionaceae id.3169)

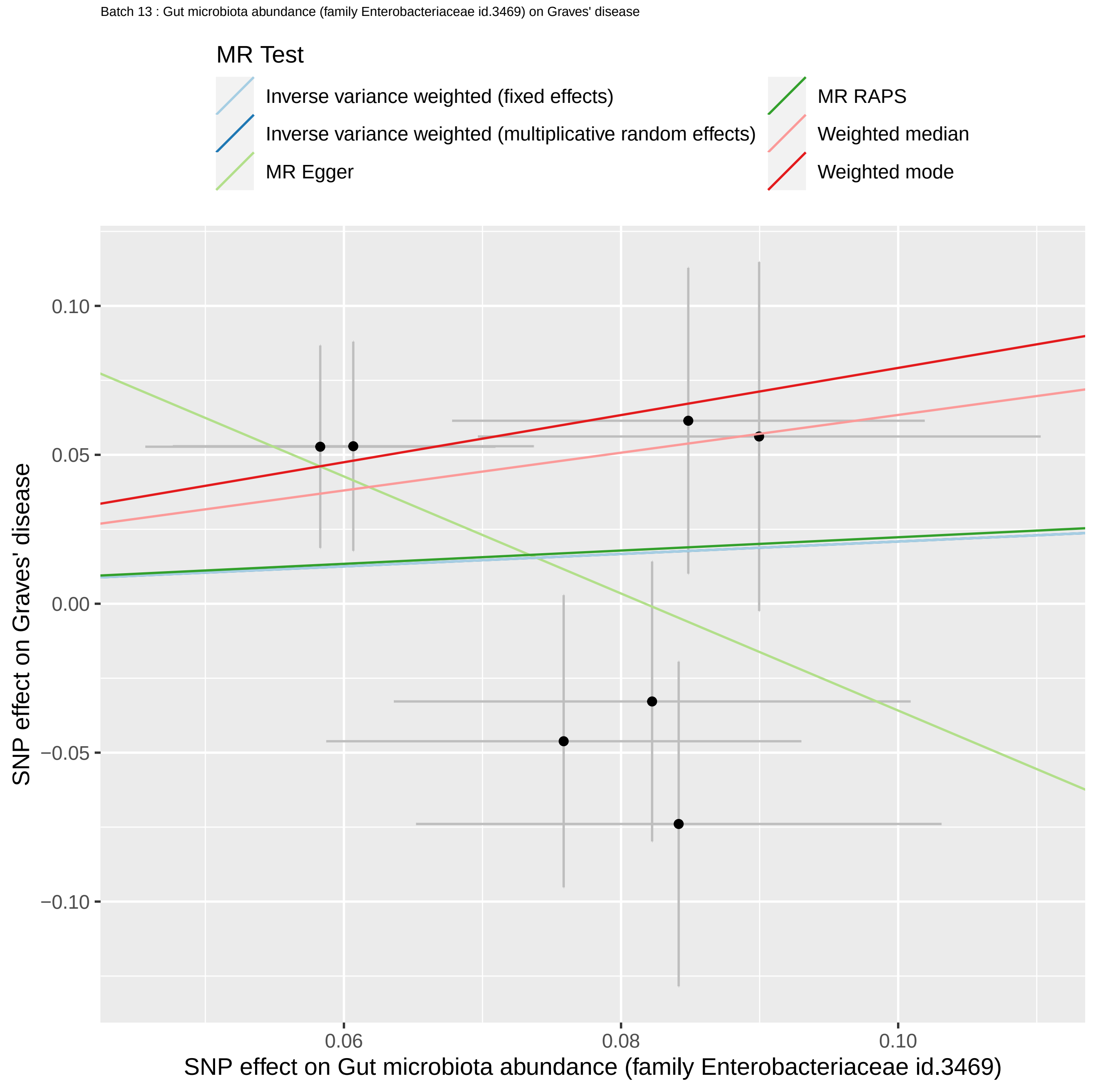

Batch 13 : Gut microbiota abundance (family Enterobacteriaceae id.3469) on Graves' disease
MR Test
Inverse variance weighted (fixed effects)
Inverse variance weighted (multiplicative random effects)
MR Egger
MR RAPS
Weighted median
Weighted mode
SNP effect on Graves' disease
0.10
0.05
0.00
−0.05
−0.10
0.06
0.08
0.10
SNP effect on Gut microbiota abundance (family Enterobacteriaceae id.3469)

Batch 14 : Gut microbiota abundance (family Erysipelotrichaceae id.2149) on Graves' disease

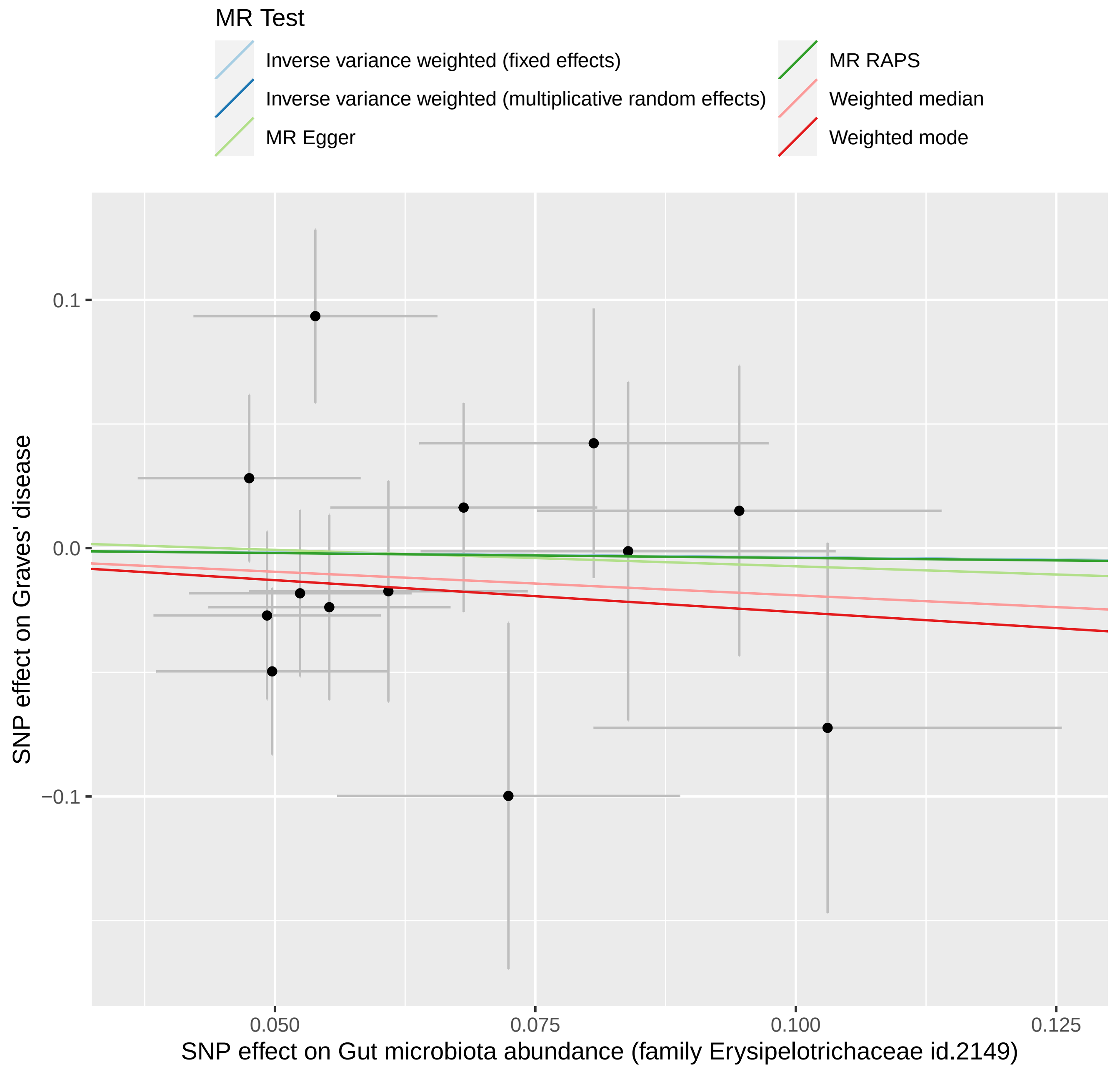

Batch 15 : Gut microbiota abundance (family Family XI id.1936) on Graves' disease

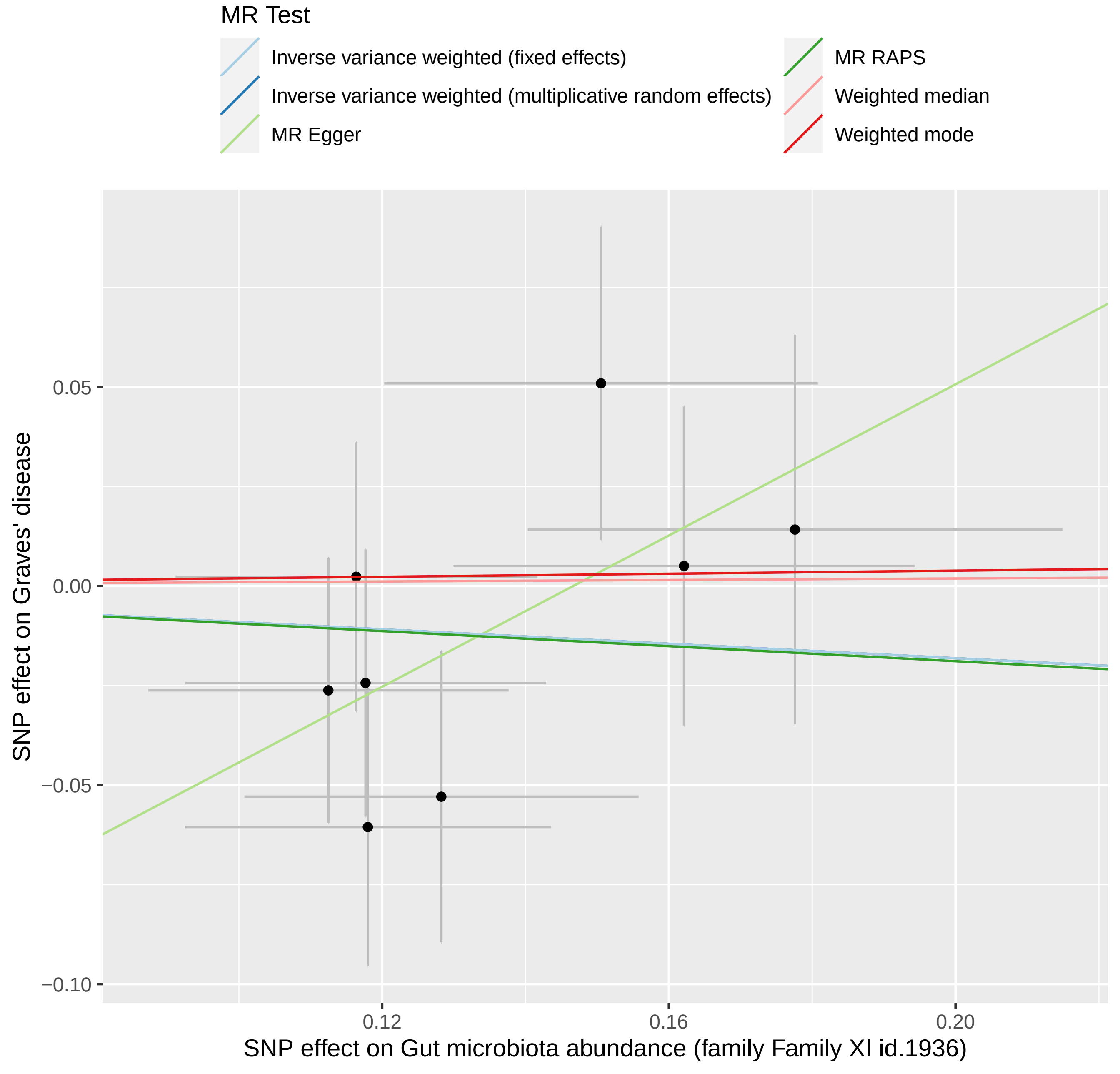

Batch 16 : Gut microbiota abundance (family Family XIII id.1957) on Graves' disease

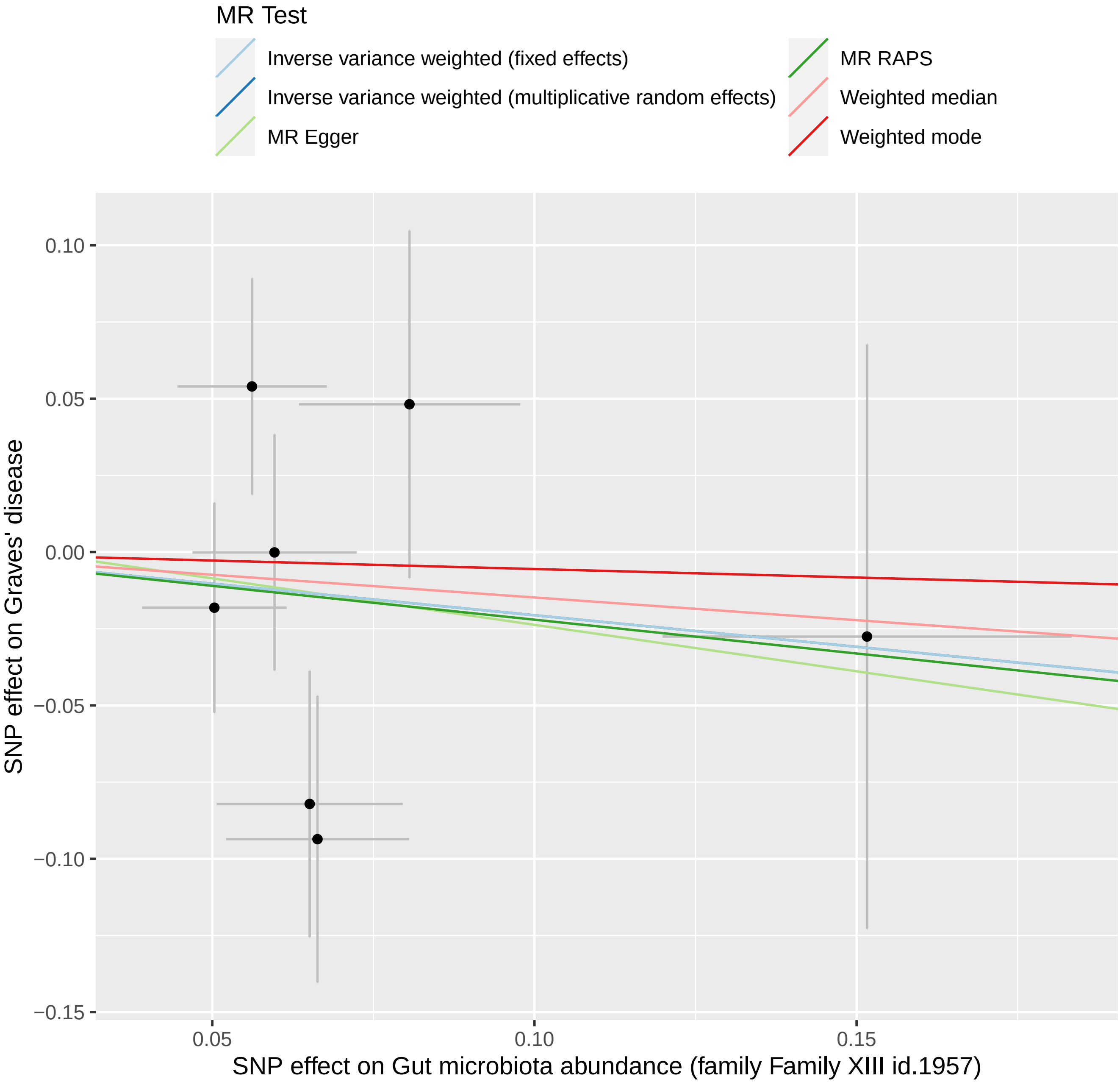

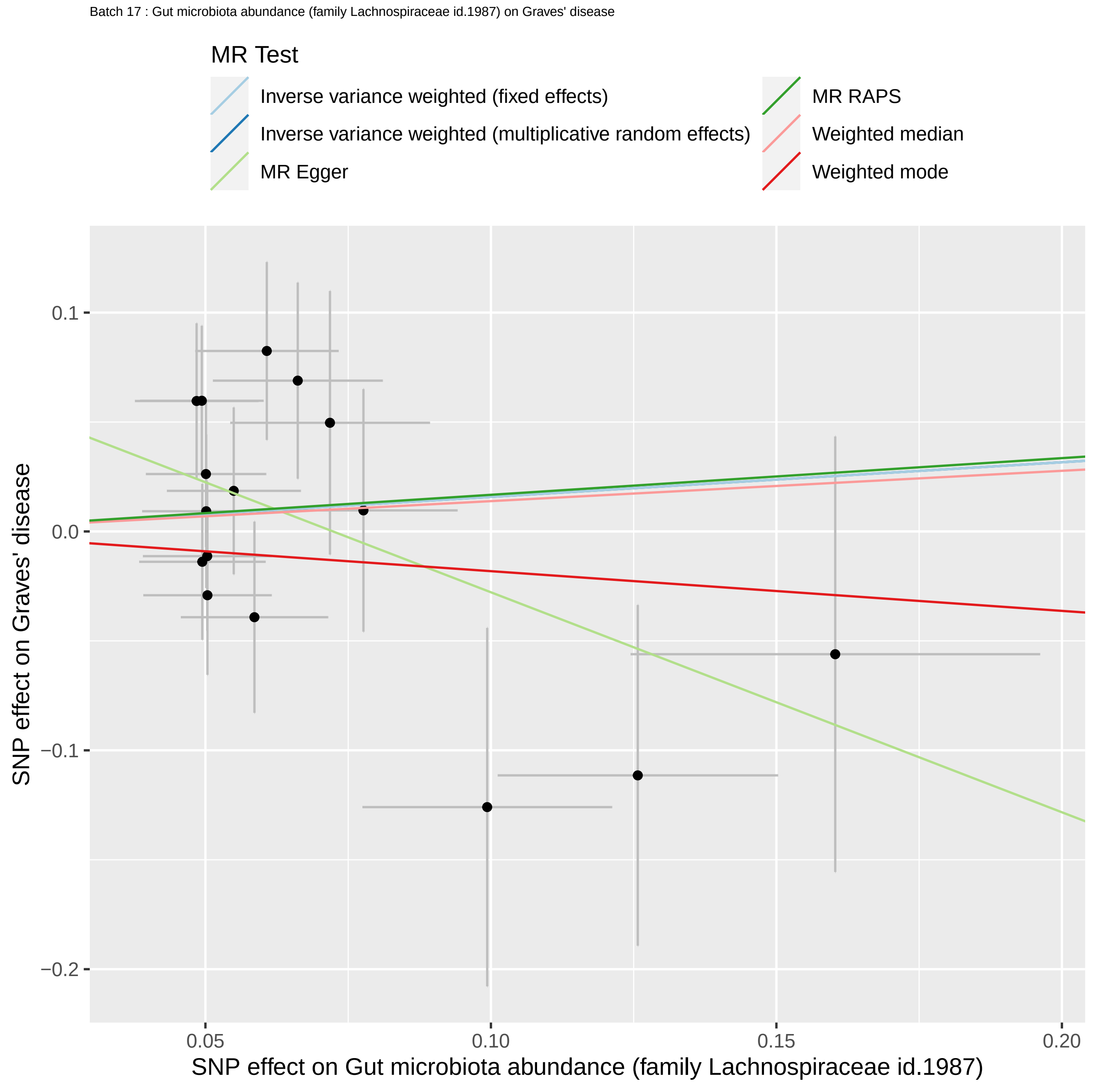
Batch 17 : Gut microbiota abundance (family Lachnospiraceae id.1987) on Graves' disease
MR Test
Inverse variance weighted (fixed effects)
Inverse variance weighted (multiplicative random effects)
MR Egger
MR RAPS
Weighted median
Weighted mode
SNP effect on Graves' disease
0.1
0.0
−0.1
−0.2
0.05
0.10
0.15
0.20
SNP effect on Gut microbiota abundance (family Lachnospiraceae id.1987)

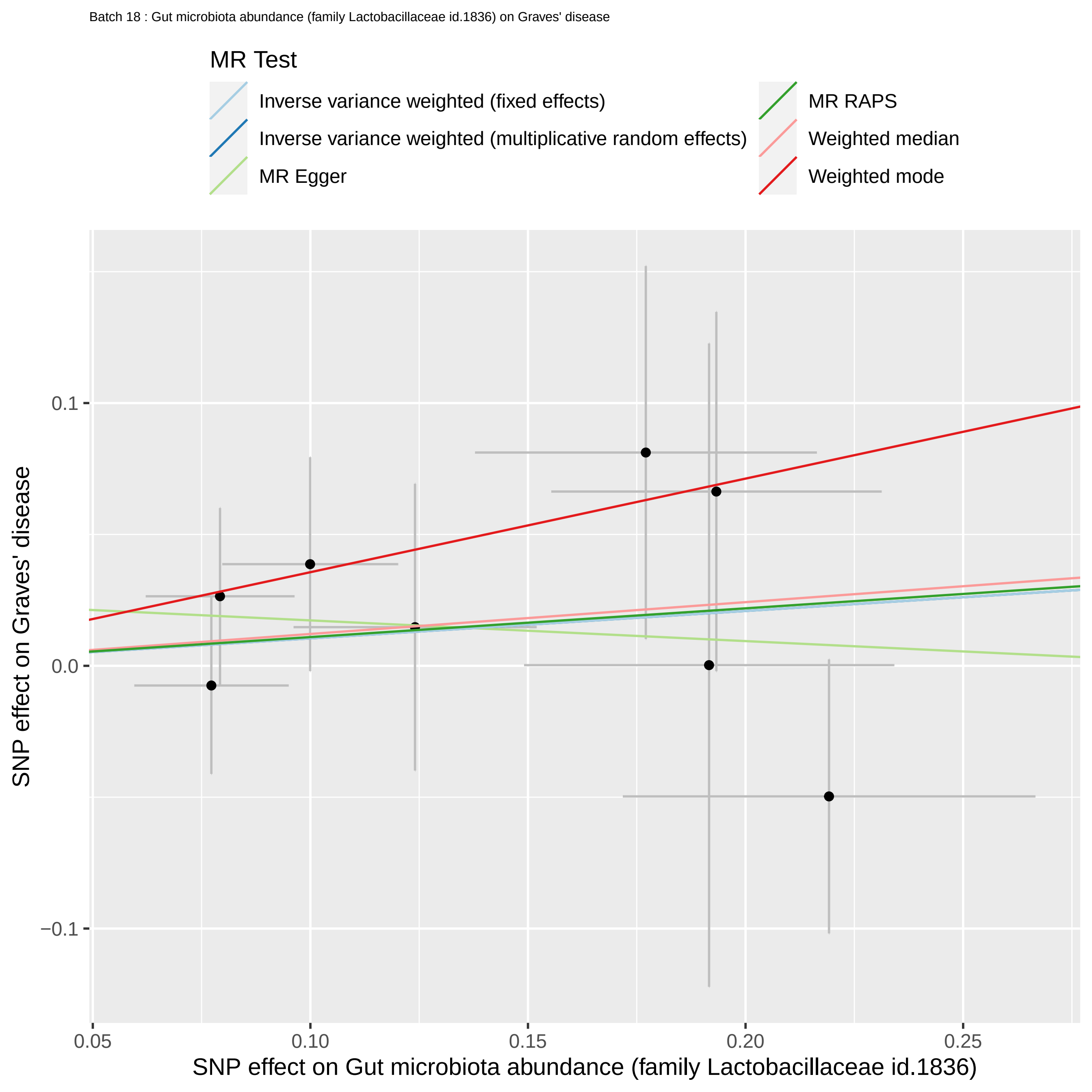
Batch 18 : Gut microbiota abundance (family Lactobacillaceae id.1836) on Graves' disease
MR Test
Inverse variance weighted (fixed effects)
Inverse variance weighted (multiplicative random effects)
MR Egger
MR RAPS
Weighted median
Weighted mode
0.1
0.0
−0.1
0.05
0.10
0.15
0.20
0.25
SNP effect on Graves' disease
SNP effect on Gut microbiota abundance (family Lactobacillaceae id.1836)

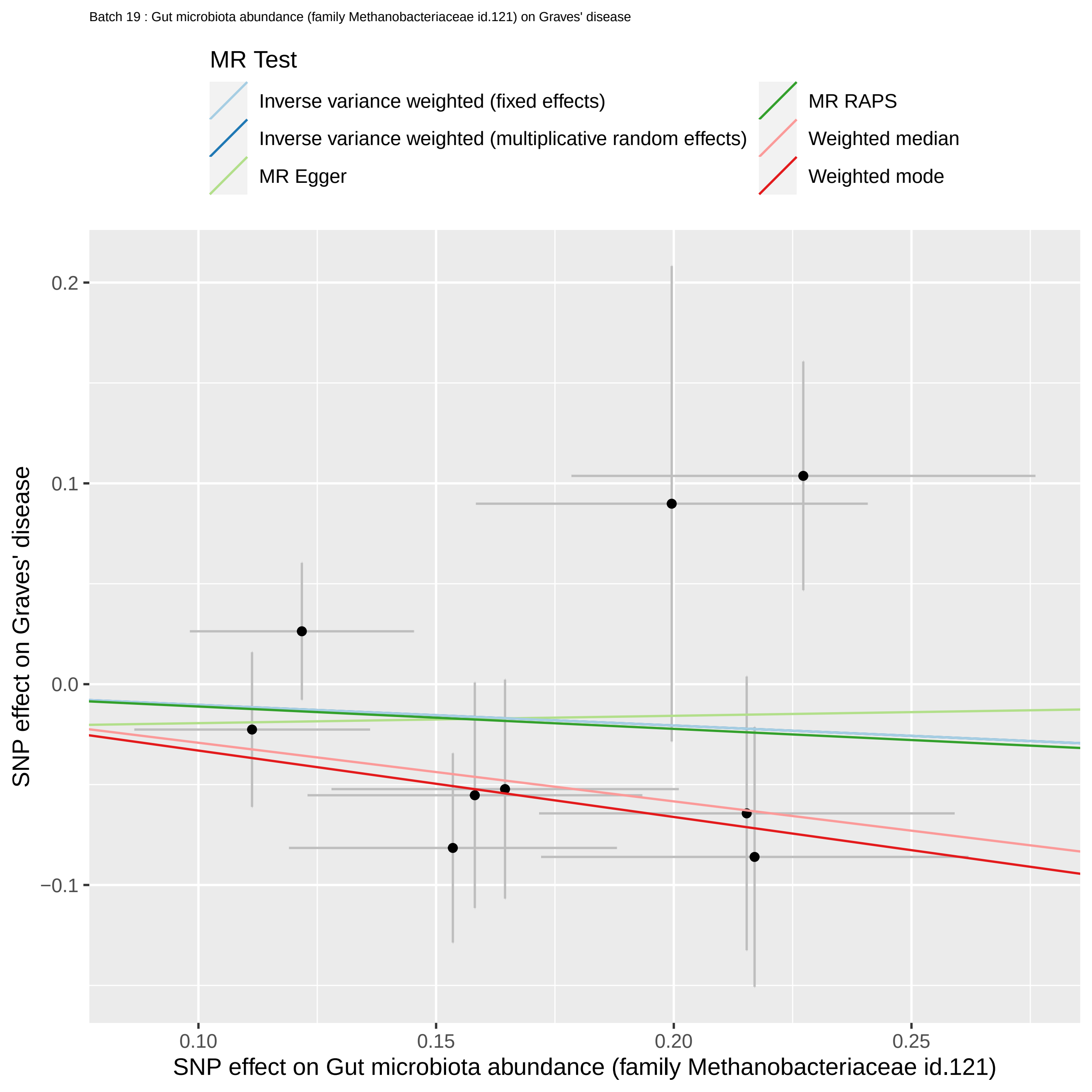

Batch 19 : Gut microbiota abundance (family Methanobacteriaceae id.121) on Graves' disease
MR Test
Inverse variance weighted (fixed effects)
Inverse variance weighted (multiplicative random effects)
MR Egger
MR RAPS
Weighted median
Weighted mode
0.2
0.1
0.0
−0.1
0.10
0.15
0.20
0.25
SNP effect on Graves' disease
SNP effect on Gut microbiota abundance (family Methanobacteriaceae id.121)

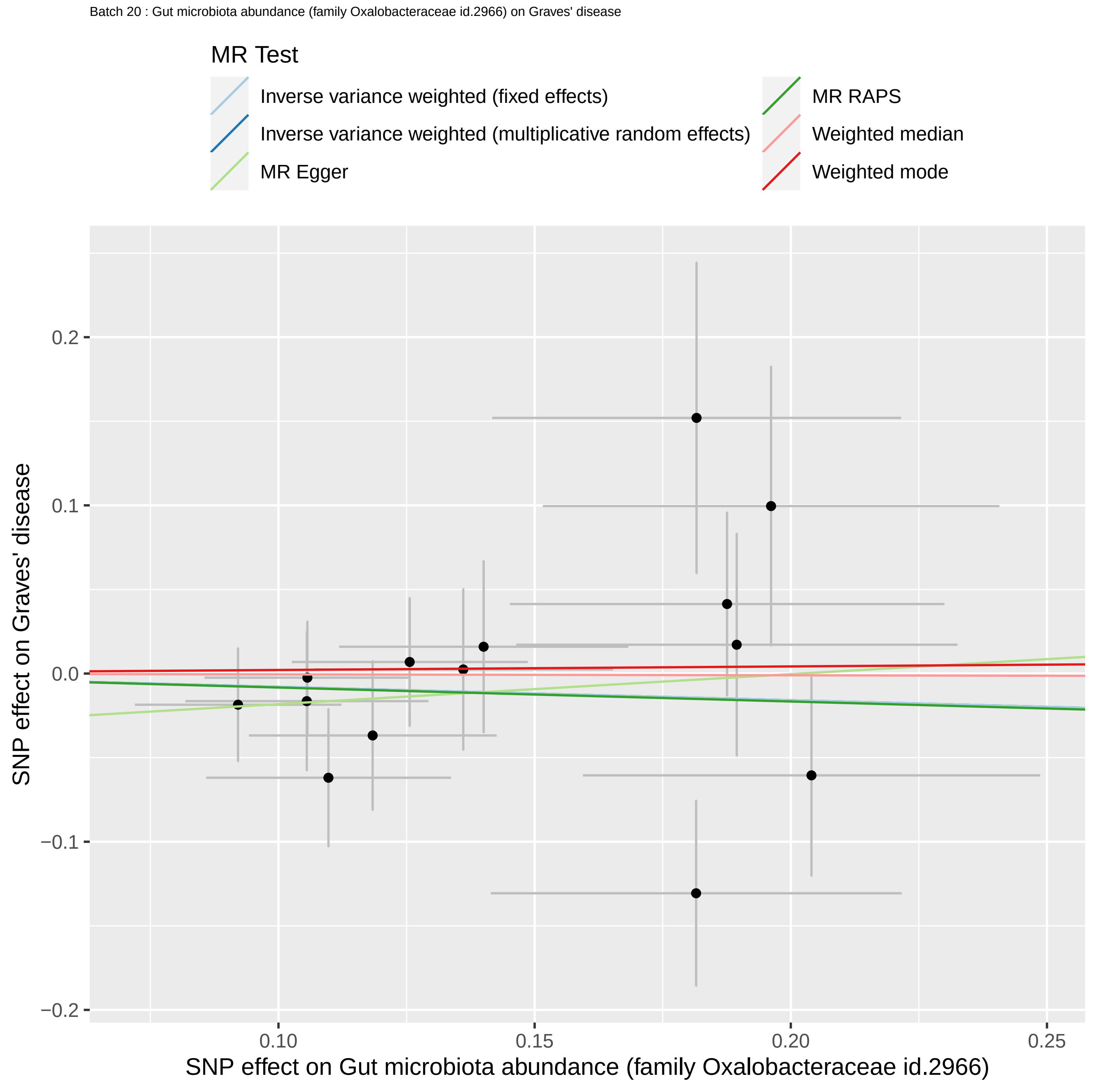

Batch 20 : Gut microbiota abundance (family Oxalobacteraceae id.2966) on Graves' disease
MR Test
Inverse variance weighted (fixed effects)
Inverse variance weighted (multiplicative random effects)
MR Egger
MR RAPS
Weighted median
Weighted mode
0.2
0.1
0.0
−0.1
−0.2
0.10
0.15
0.20
0.25
SNP effect on Graves' disease
SNP effect on Gut microbiota abundance (family Oxalobacteraceae id.2966)

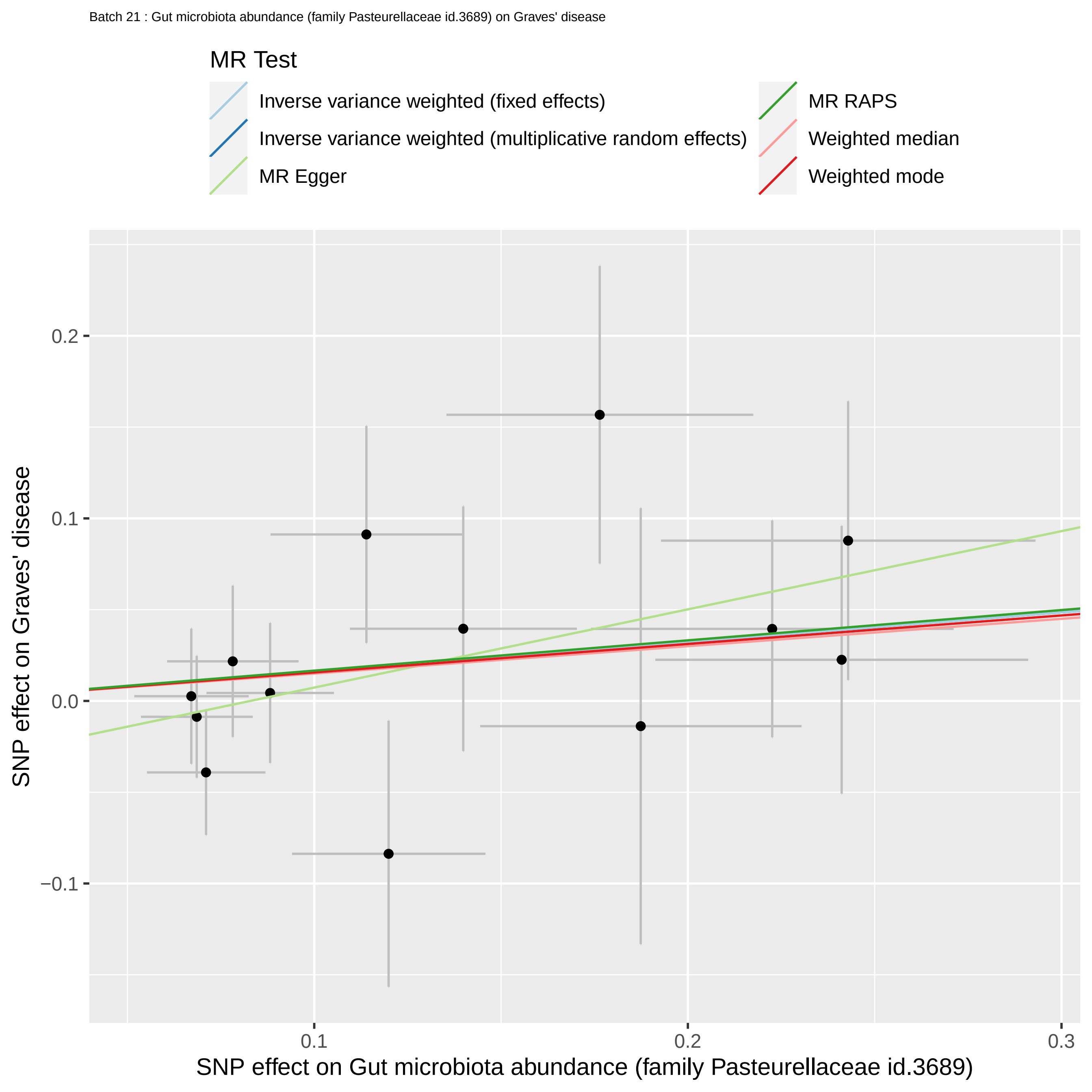
Batch 21 : Gut microbiota abundance (family Pasteurellaceae id.3689) on Graves' disease
MR Test
Inverse variance weighted (fixed effects)
Inverse variance weighted (multiplicative random effects)
MR Egger
MR RAPS
Weighted median
Weighted mode
SNP effect on Graves' disease
0.2
0.1
0.0
−0.1
0.1
0.2
0.3
SNP effect on Gut microbiota abundance (family Pasteurellaceae id.3689)

Batch 22 : Gut microbiota abundance (family Peptococcaceae id.2024) on Graves' disease

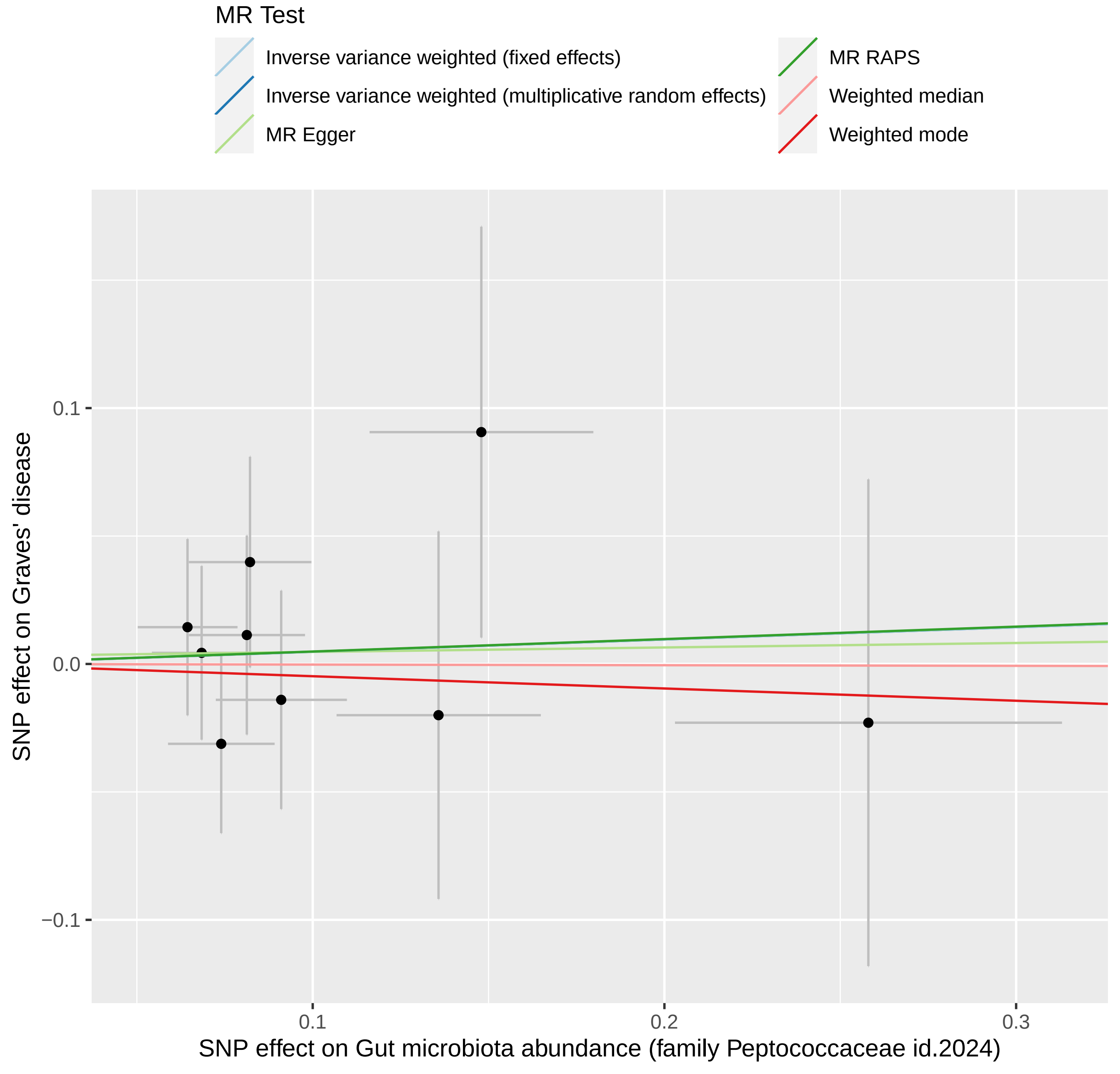

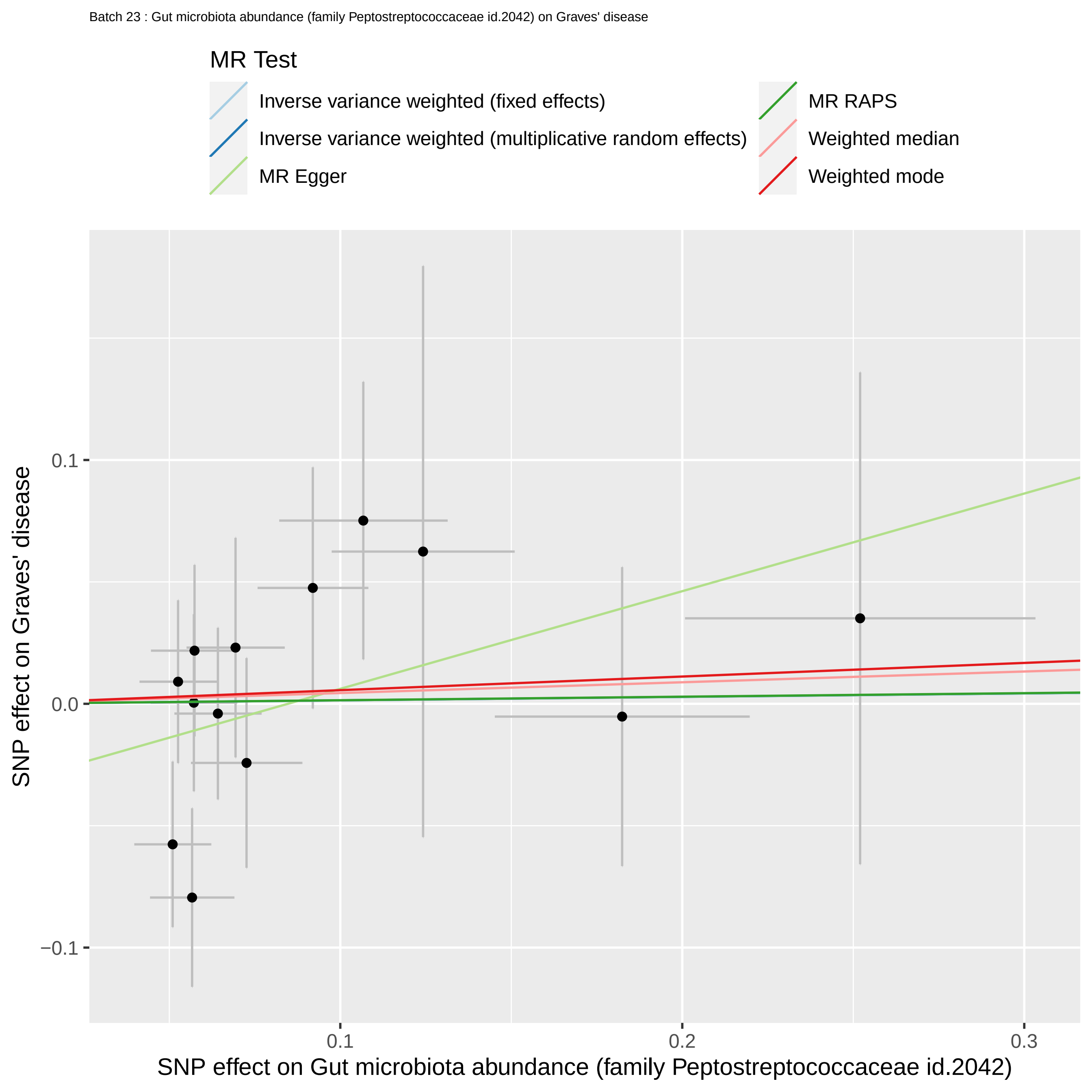

Batch 23 : Gut microbiota abundance (family Peptostreptococcaceae id.2042) on Graves' disease
MR Test
Inverse variance weighted (fixed effects)
Inverse variance weighted (multiplicative random effects)
MR Egger
MR RAPS
Weighted median
Weighted mode
SNP effect on Graves' disease
0.1
0.0
−0.1
0.1
0.2
0.3
SNP effect on Gut microbiota abundance (family Peptostreptococcaceae id.2042)

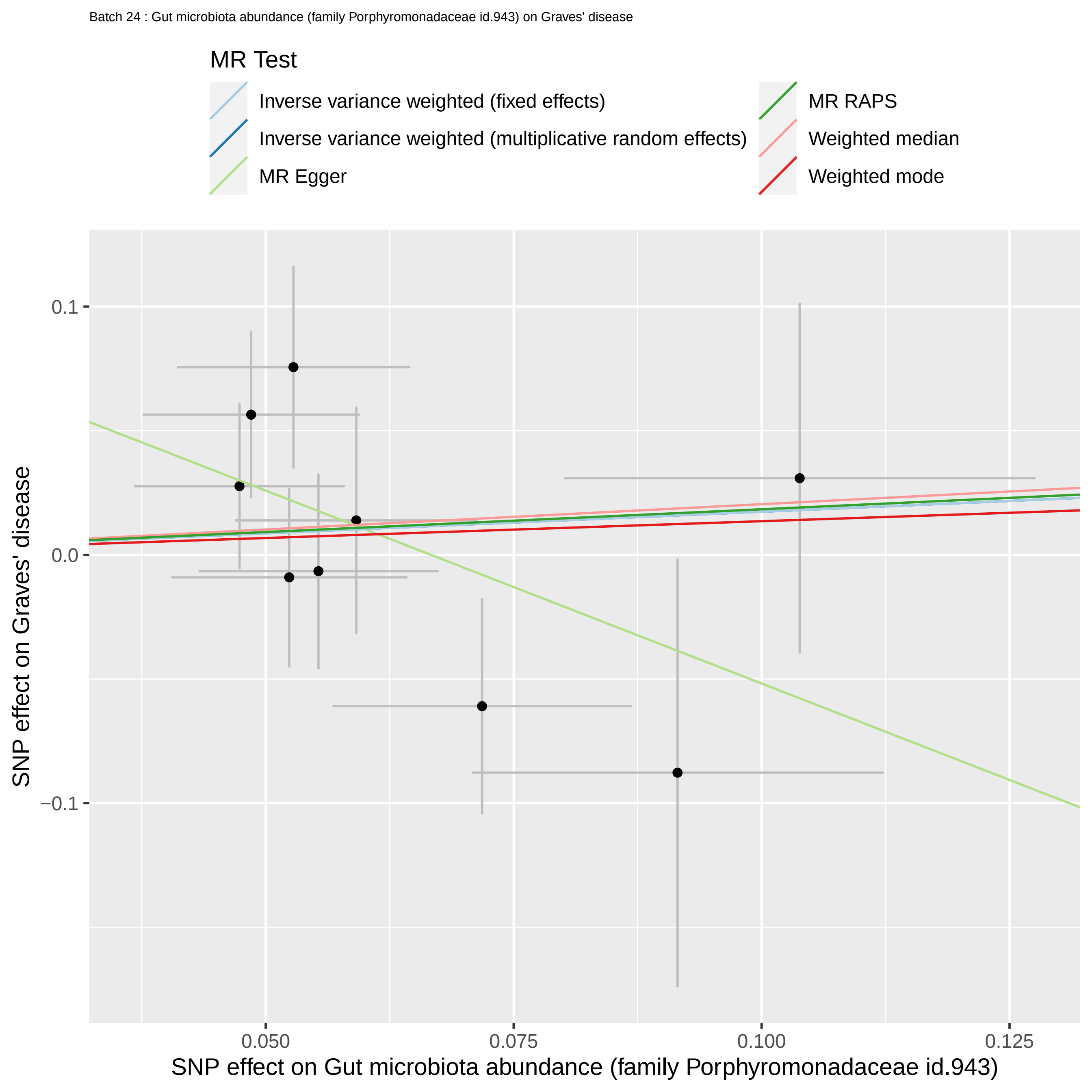
Batch 24 : Gut microbiota abundance (family Porphyromonadaceae id.943) on Graves' disease
MR Test
Inverse variance weighted (fixed effects)
Inverse variance weighted (multiplicative random effects)
MR Egger
MR RAPS
Weighted median
Weighted mode
0.1
0.0
−0.1
0.050
0.075
0.100
0.125
SNP effect on Graves' disease
SNP effect on Gut microbiota abundance (family Porphyromonadaceae id.943)

Batch 25 : Gut microbiota abundance (family Prevotellaceae id.960) on Graves' disease

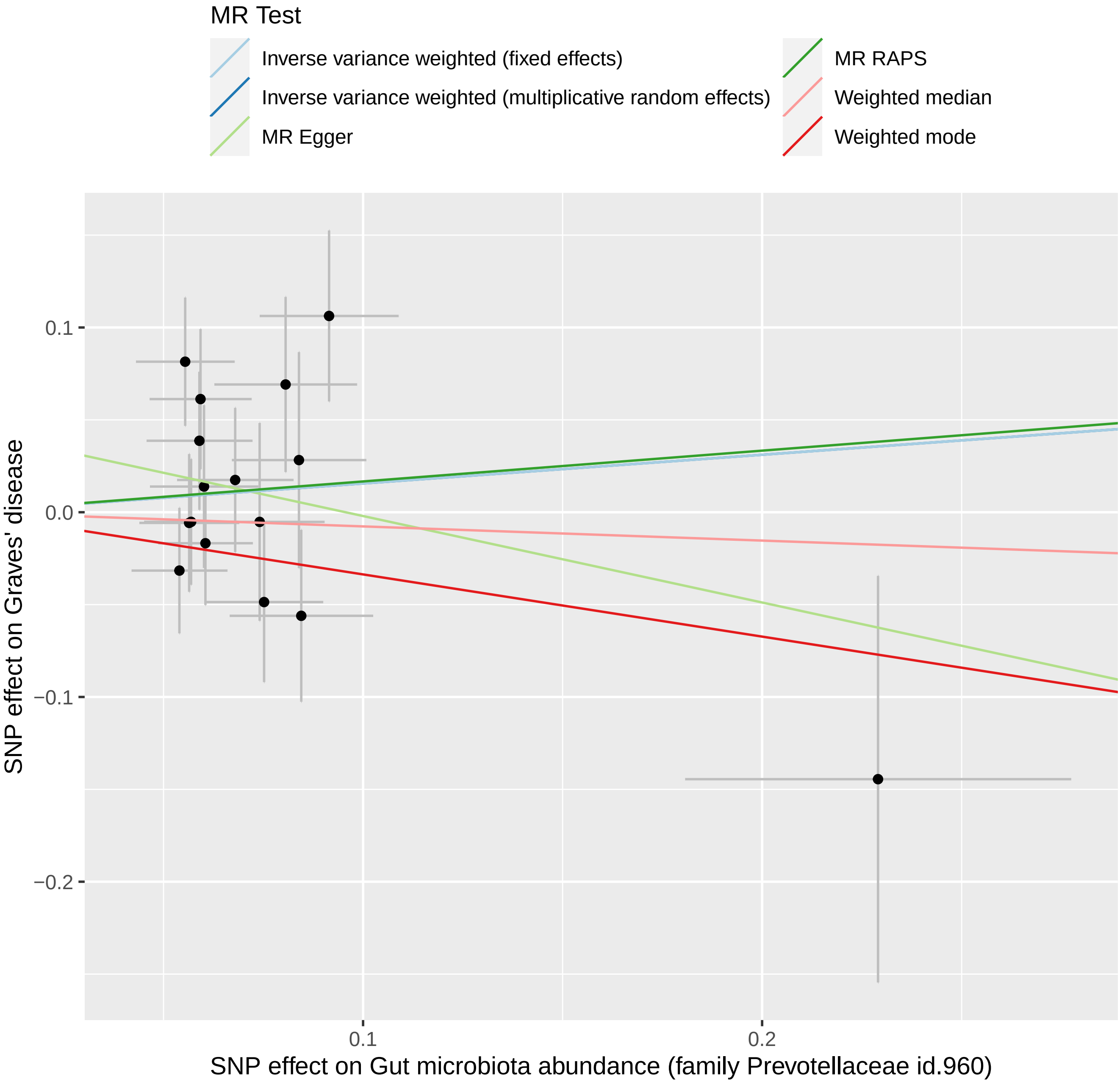

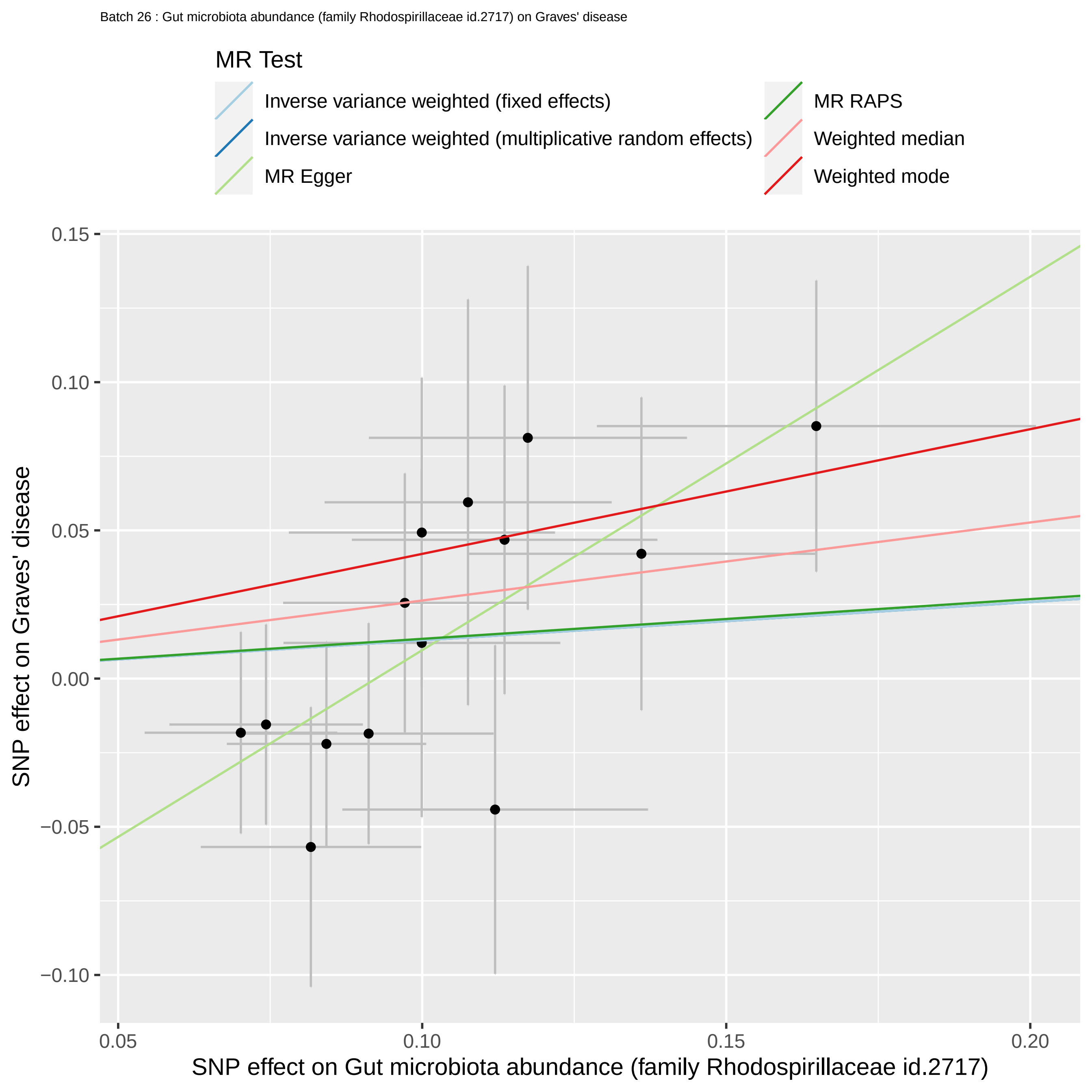
Batch 26 : Gut microbiota abundance (family Rhodospirillaceae id.2717) on Graves' disease
MR Test
Inverse variance weighted (fixed effects)
Inverse variance weighted (multiplicative random effects)
MR Egger
MR RAPS
Weighted median
Weighted mode
0.15
0.10
0.05
0.00
−0.05
−0.10
SNP effect on Graves' disease
0.05
0.10
0.15
0.20
SNP effect on Gut microbiota abundance (family Rhodospirillaceae id.2717)

Batch 27 : Gut microbiota abundance (family Rikenellaceae id.967) on Graves' disease

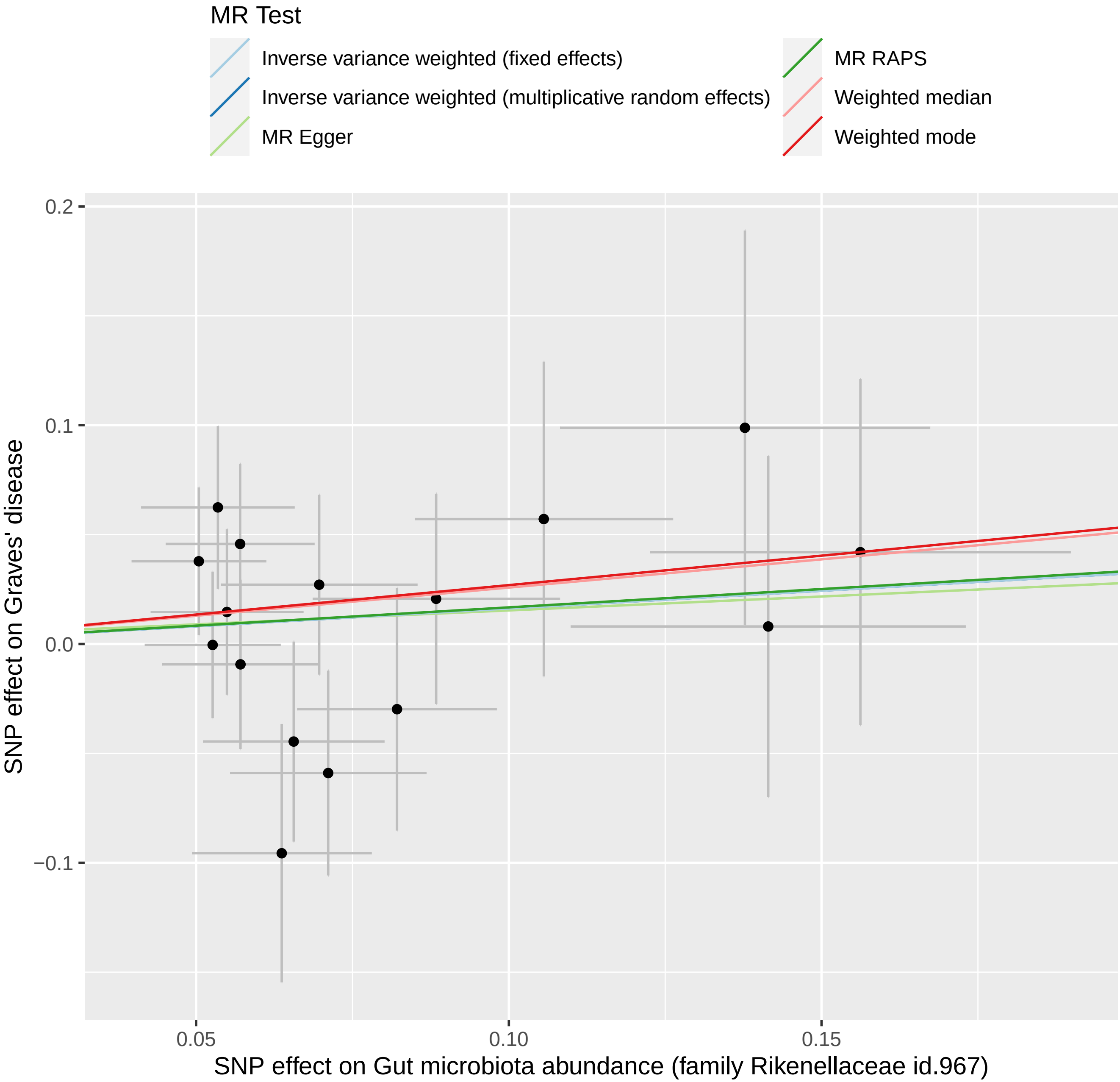

Batch 28 : Gut microbiota abundance (family Ruminococcaceae id.2050) on Graves' disease

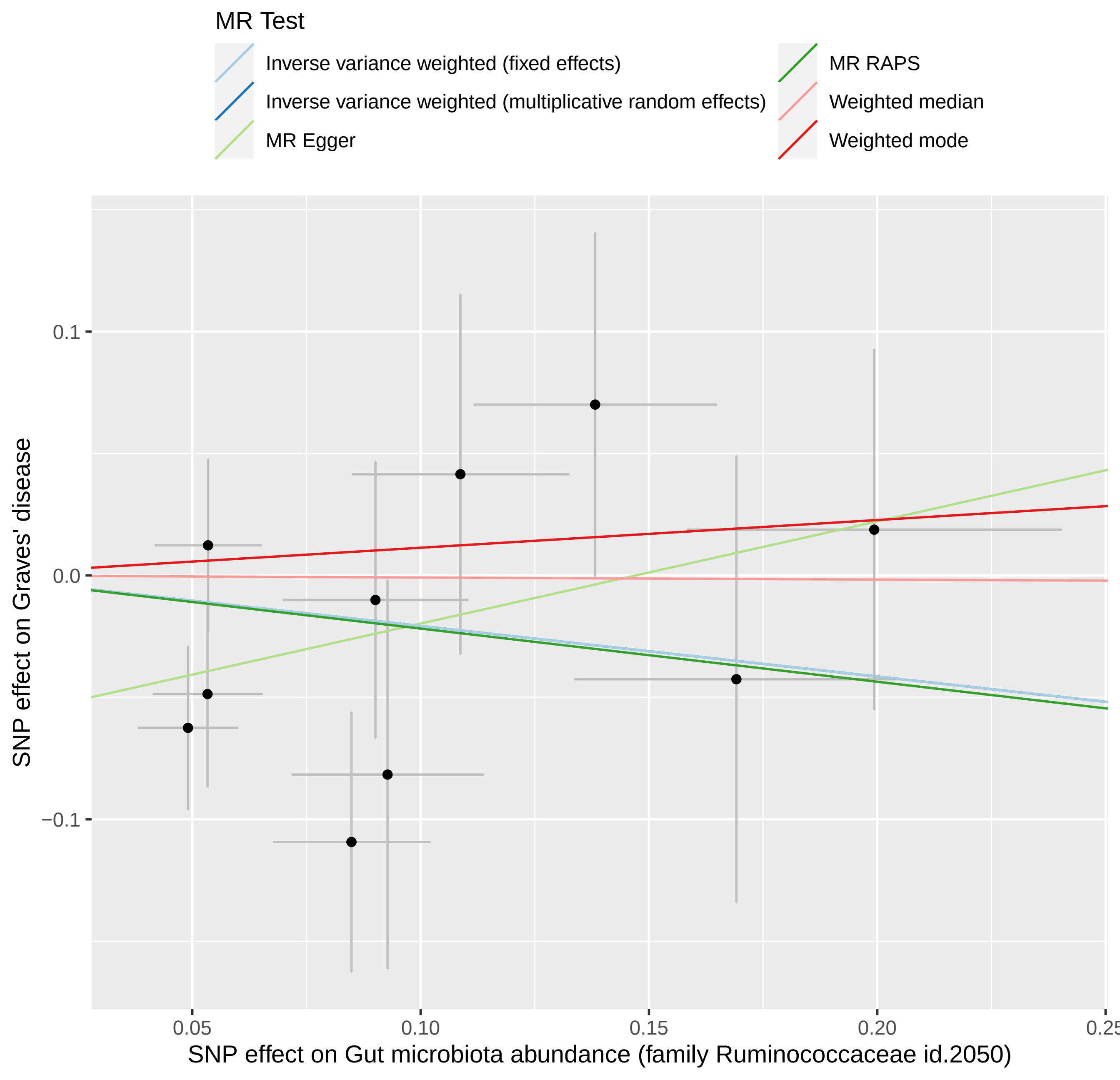

Batch 29 : Gut microbiota abundance (family Streptococcaceae id.1850) on Graves' disease

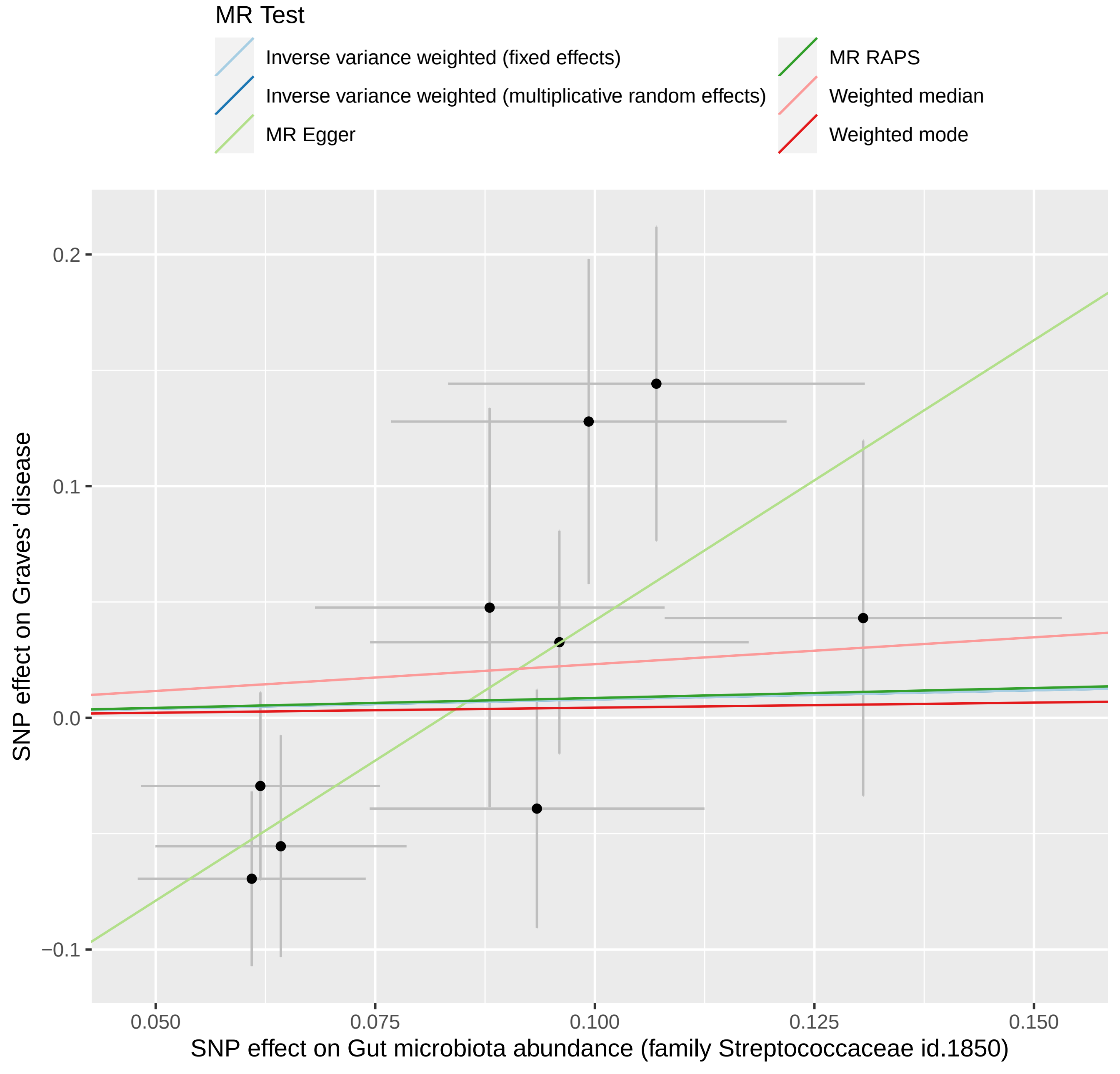

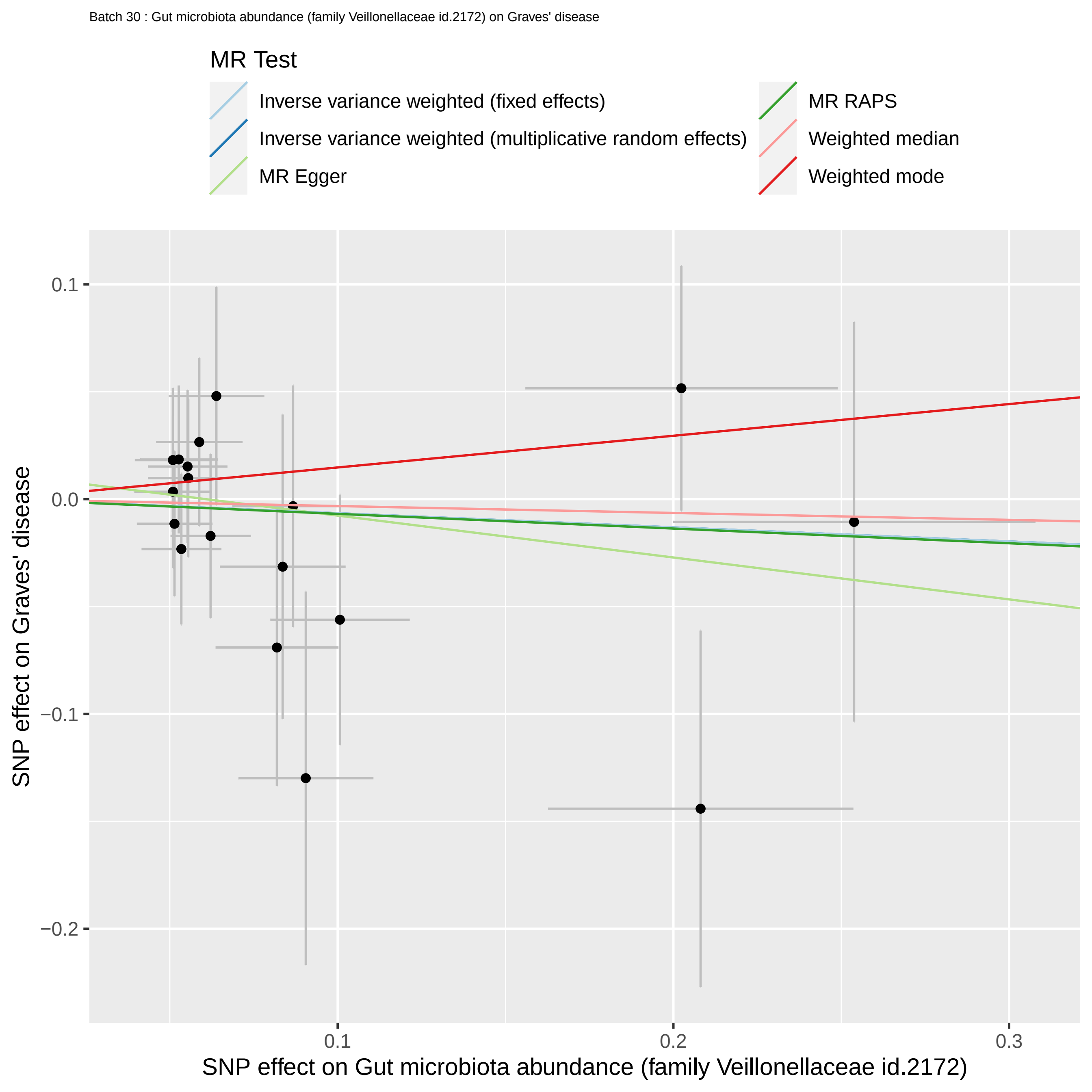
Batch 30 : Gut microbiota abundance (family Veillonellaceae id.2172) on Graves' disease
MR Test
Inverse variance weighted (fixed effects)
Inverse variance weighted (multiplicative random effects)
MR Egger
MR RAPS
Weighted median
Weighted mode
0.1
0.0
−0.1
−0.2
0.1
0.2
0.3
SNP effect on Graves' disease
SNP effect on Gut microbiota abundance (family Veillonellaceae id.2172)

Batch 31 : Gut microbiota abundance (family Verrucomicrobiaceae id.4036) on Graves' disease

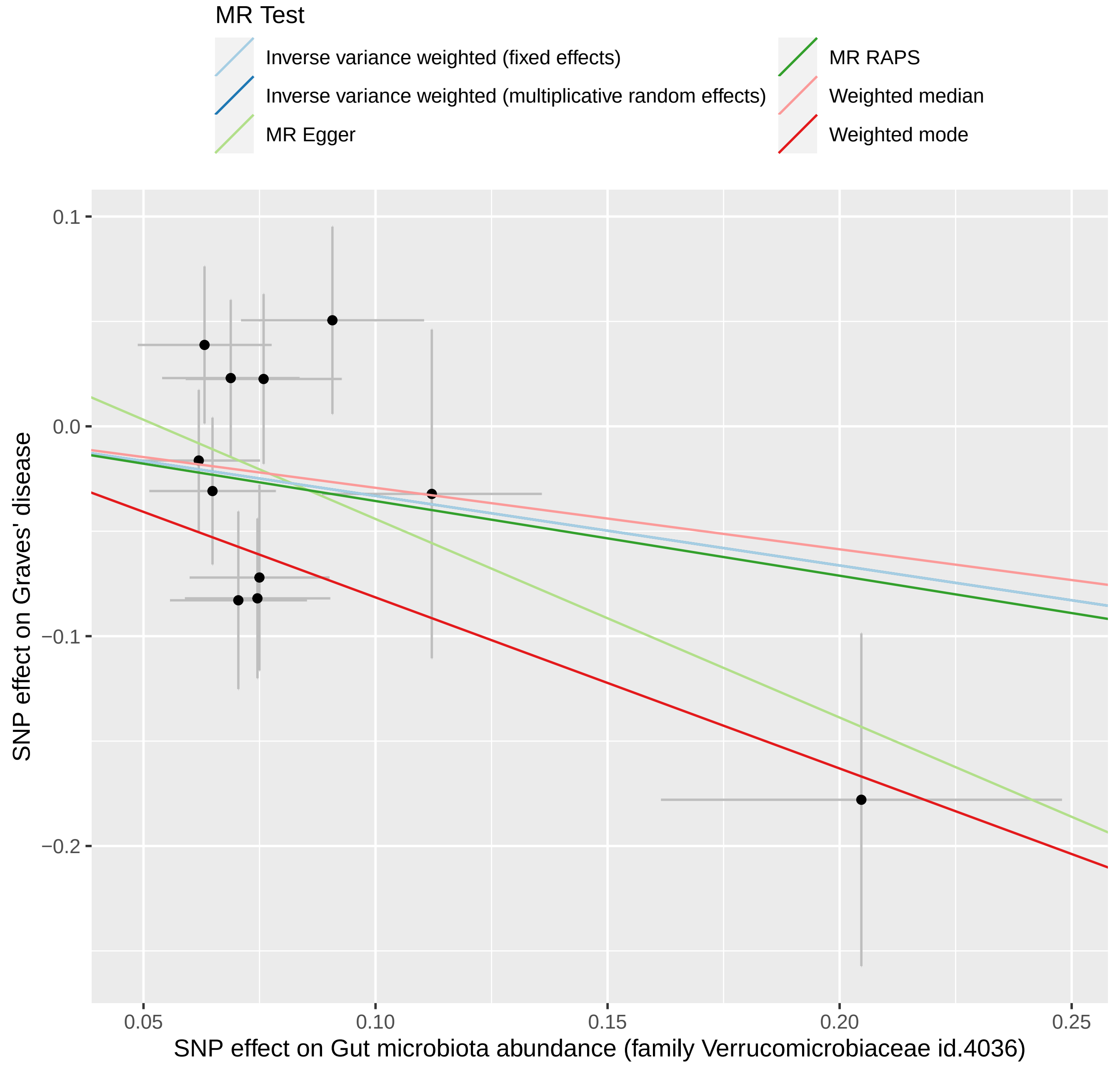

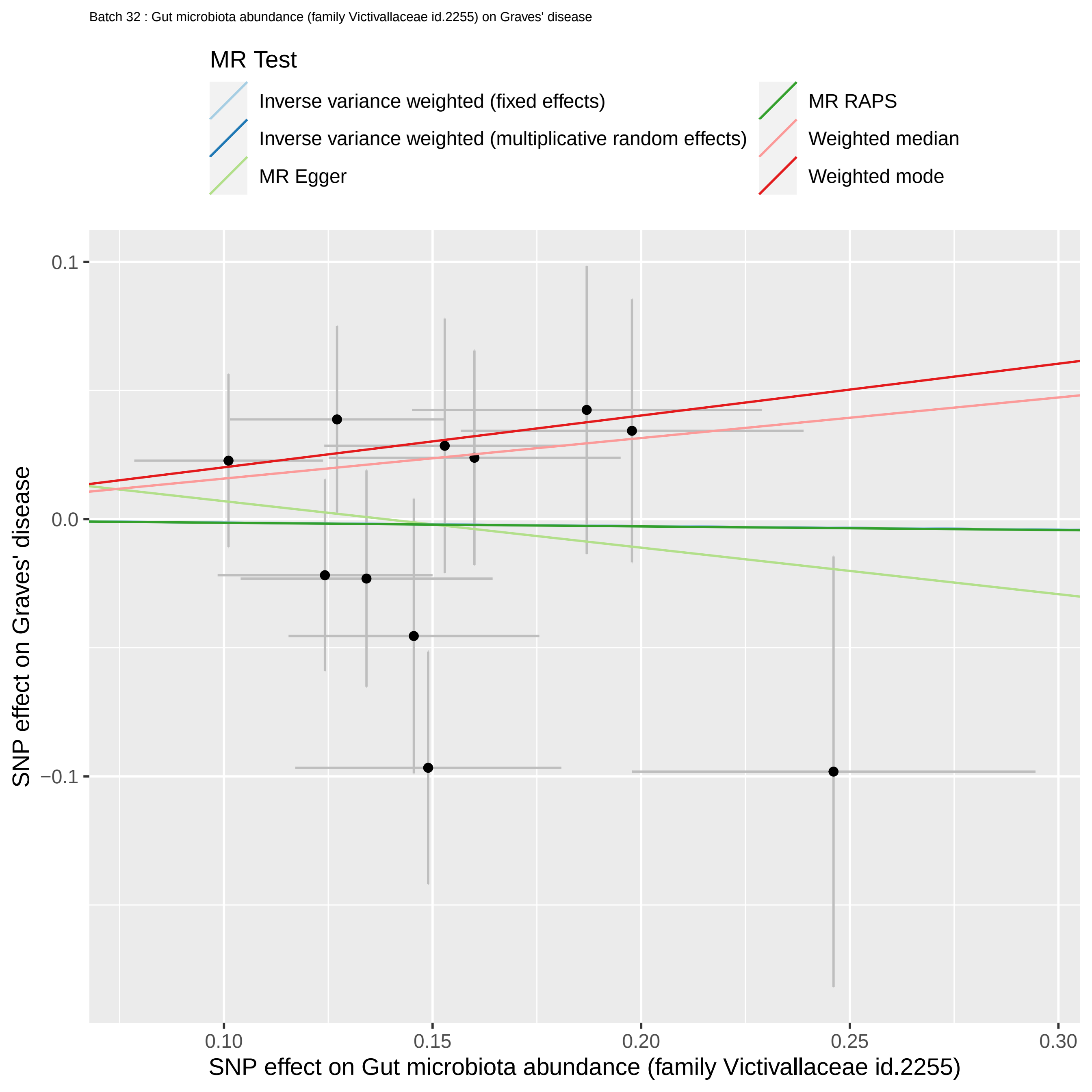
Batch 32 : Gut microbiota abundance (family Victivallaceae id.2255) on Graves' disease
MR Test
Inverse variance weighted (fixed effects)
Inverse variance weighted (multiplicative random effects)
MR Egger
MR RAPS
Weighted median
Weighted mode
0.1
0.0
−0.1
0.10
0.15
0.20
0.25
0.30
SNP effect on Graves' disease
SNP effect on Gut microbiota abundance (family Victivallaceae id.2255)

Batch 33 : Gut microbiota abundance (genus Actinomyces id.423) on Graves' disease

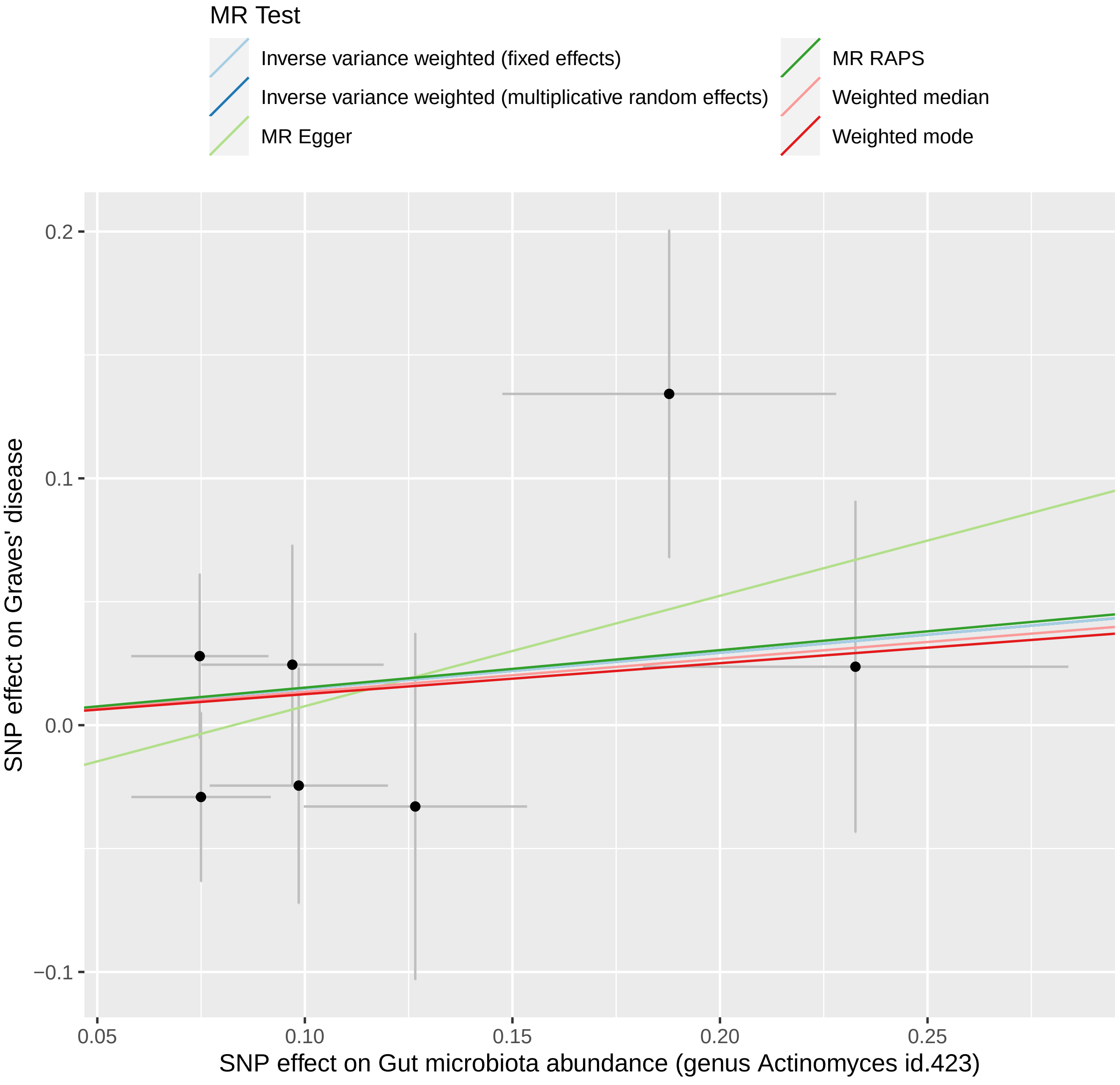

Batch 34 : Gut microbiota abundance (genus Adlercreutzia id.812) on Graves' disease

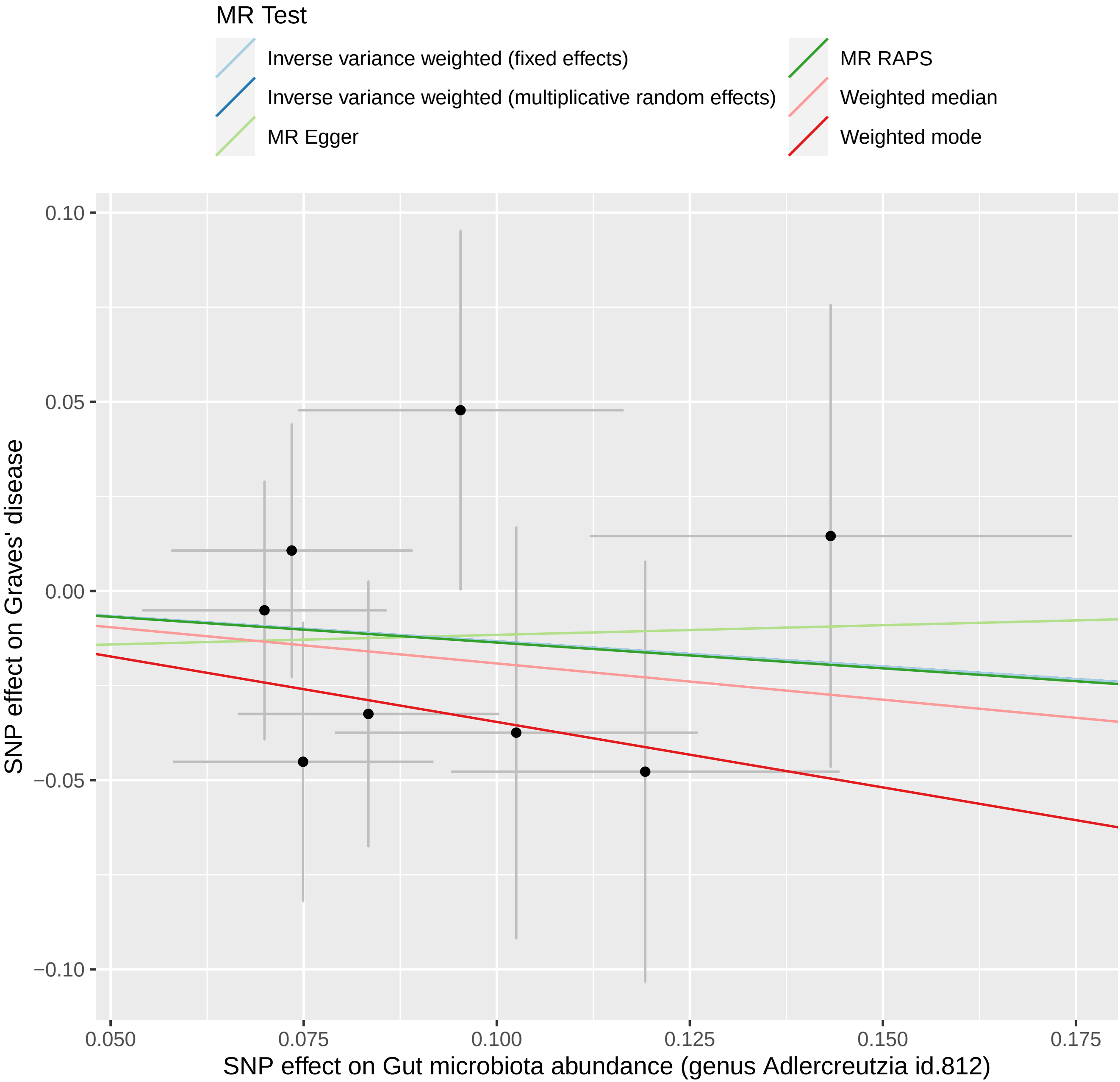

Batch 35 : Gut microbiota abundance (genus Akkermansia id.4037) on Graves' disease

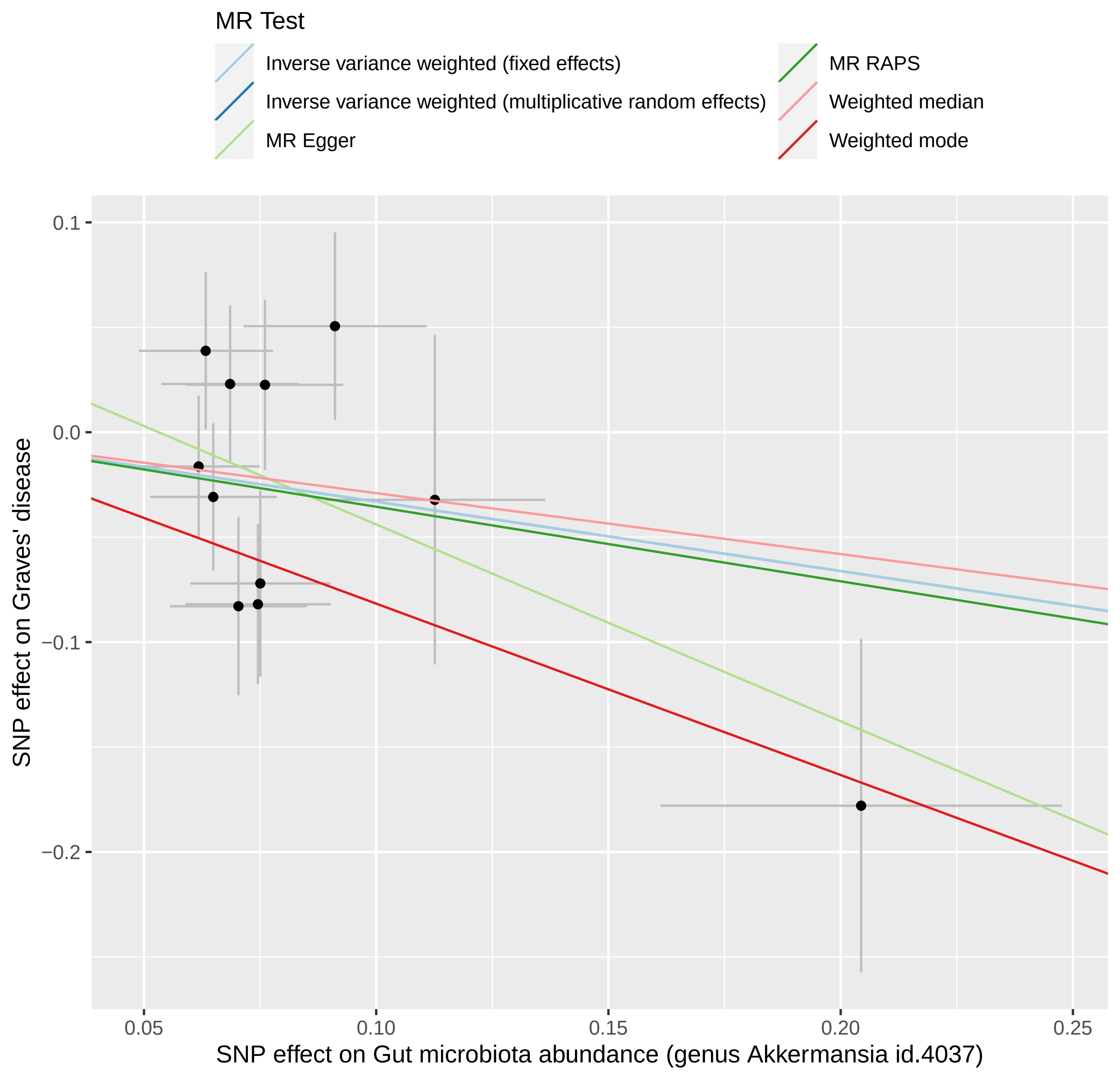

Batch 36 : Gut microbiota abundance (genus Alistipes id.968) on Graves' disease

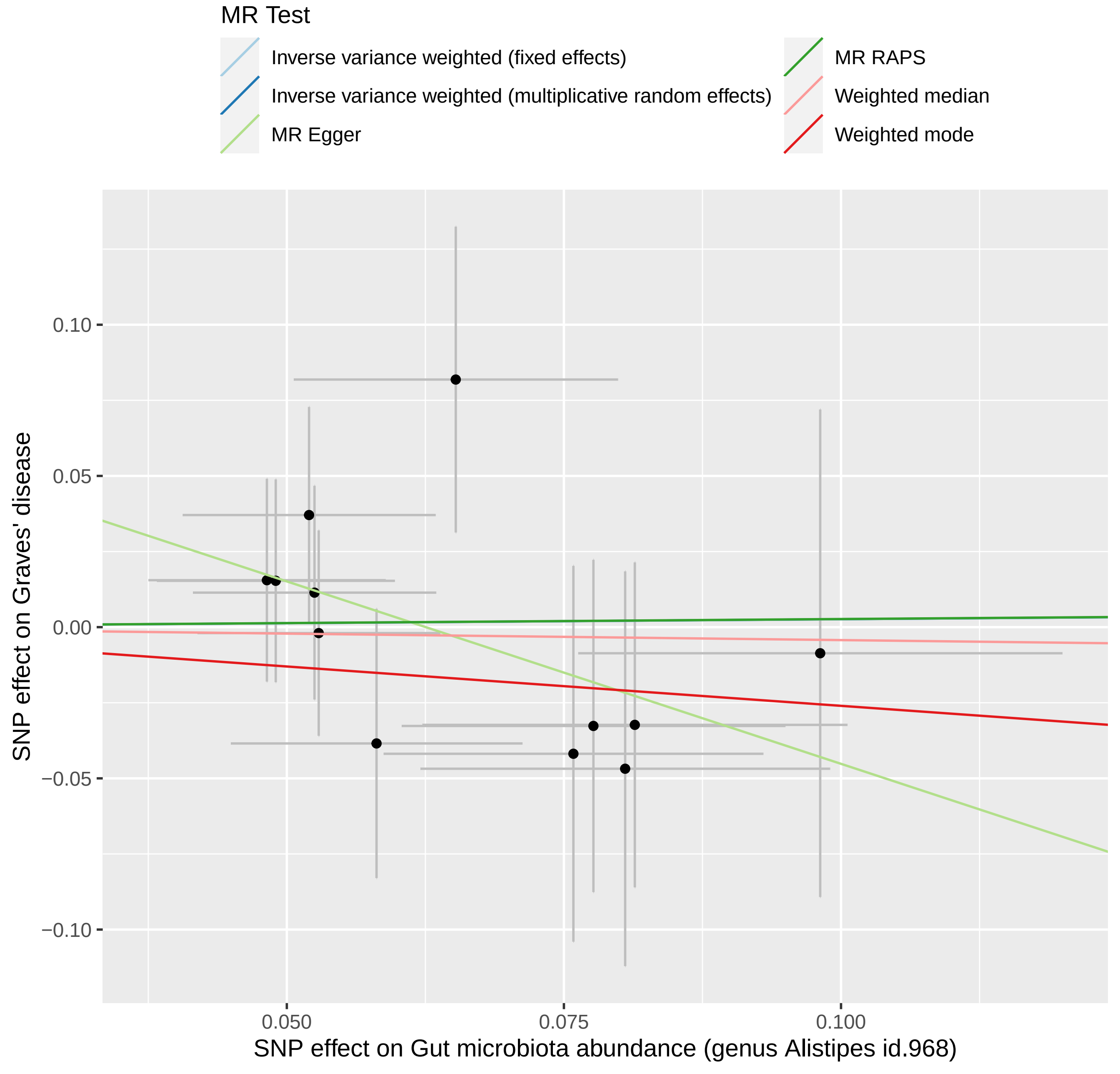

Batch 37 : Gut microbiota abundance (genus Allisonella id.2174) on Graves' disease

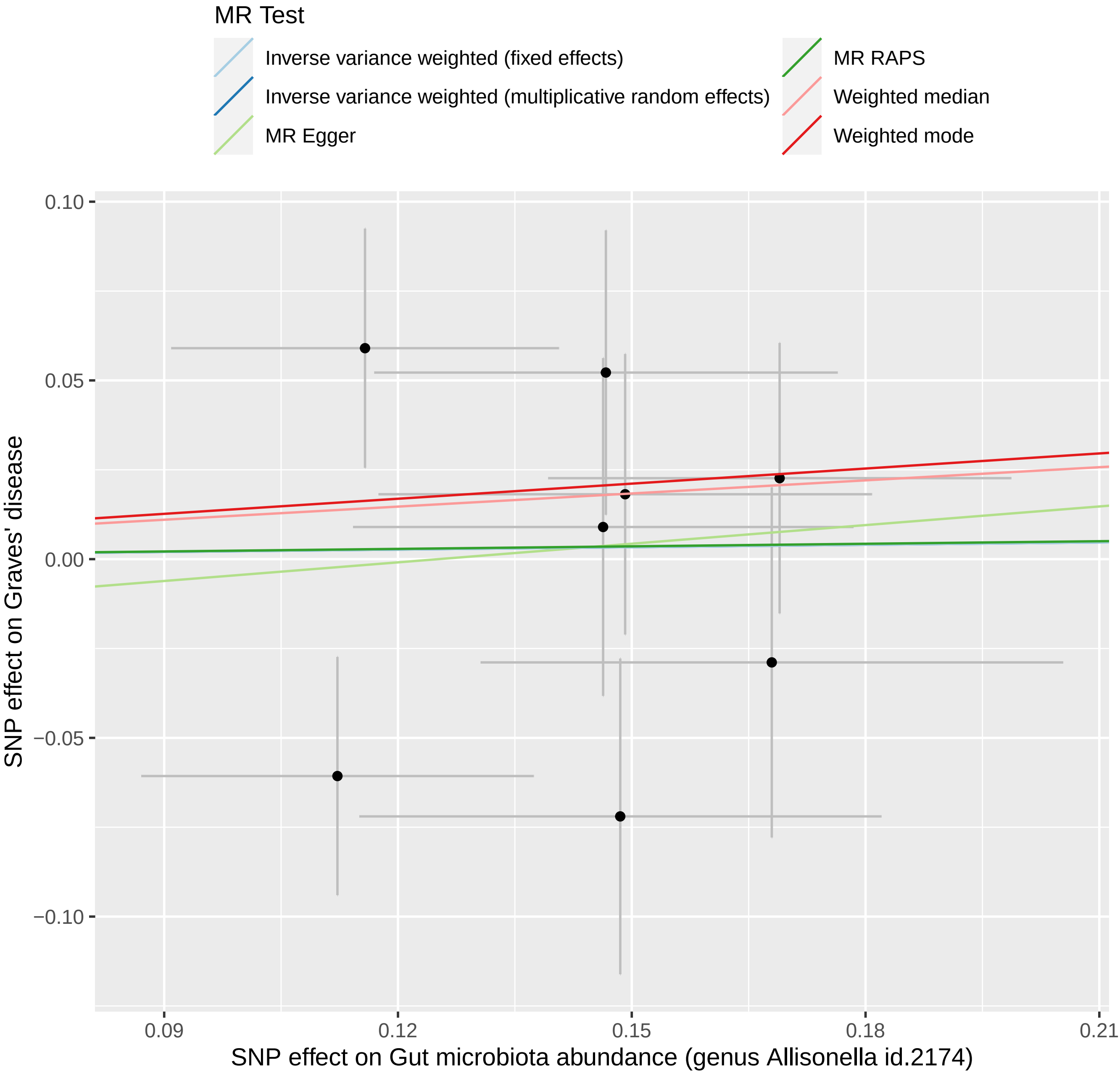

Batch 38 : Gut microbiota abundance (genus Alloprevotella id.961) on Graves' disease

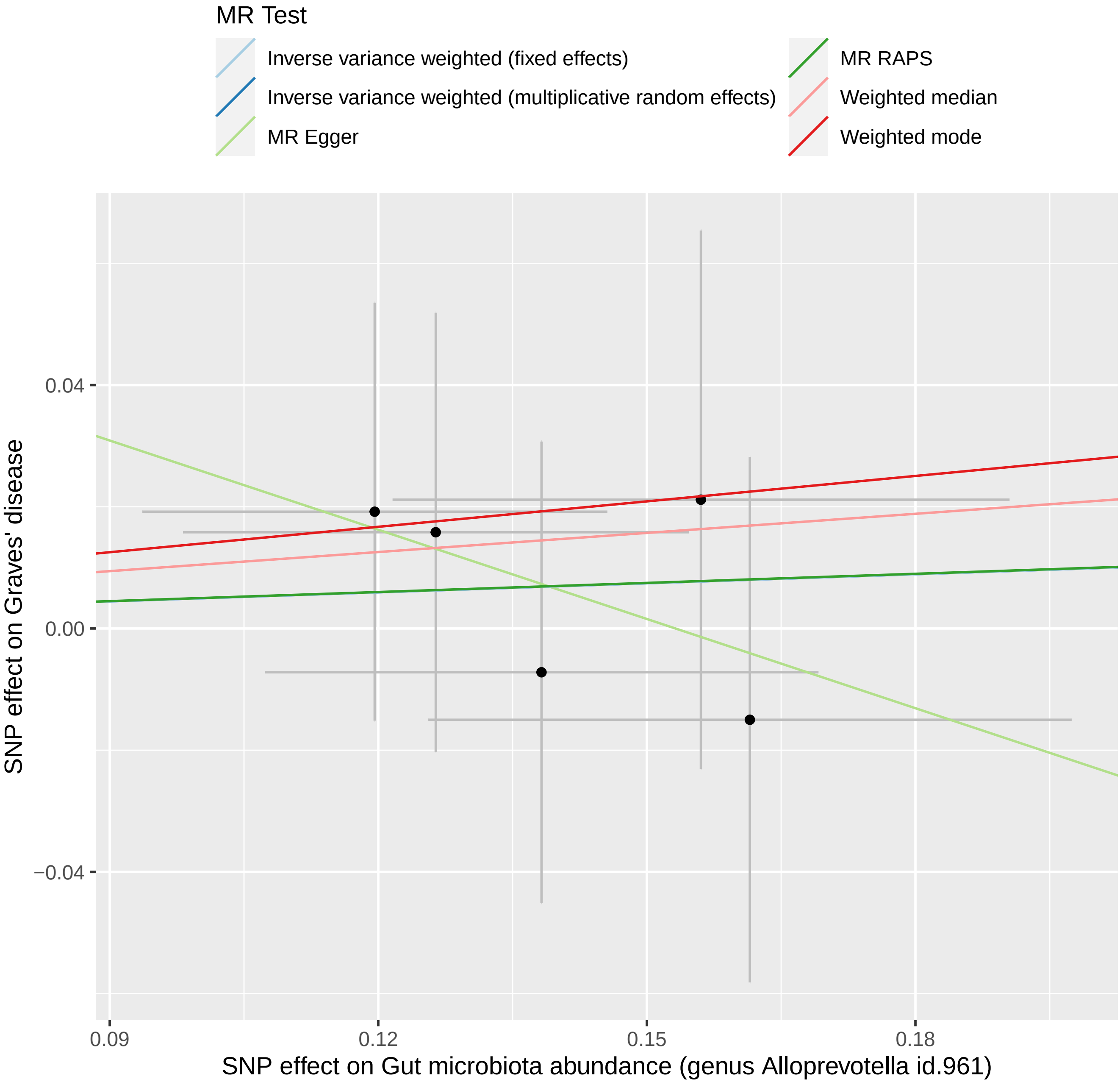

Batch 39 : Gut microbiota abundance (genus Anaerofilum id.2053) on Graves' disease

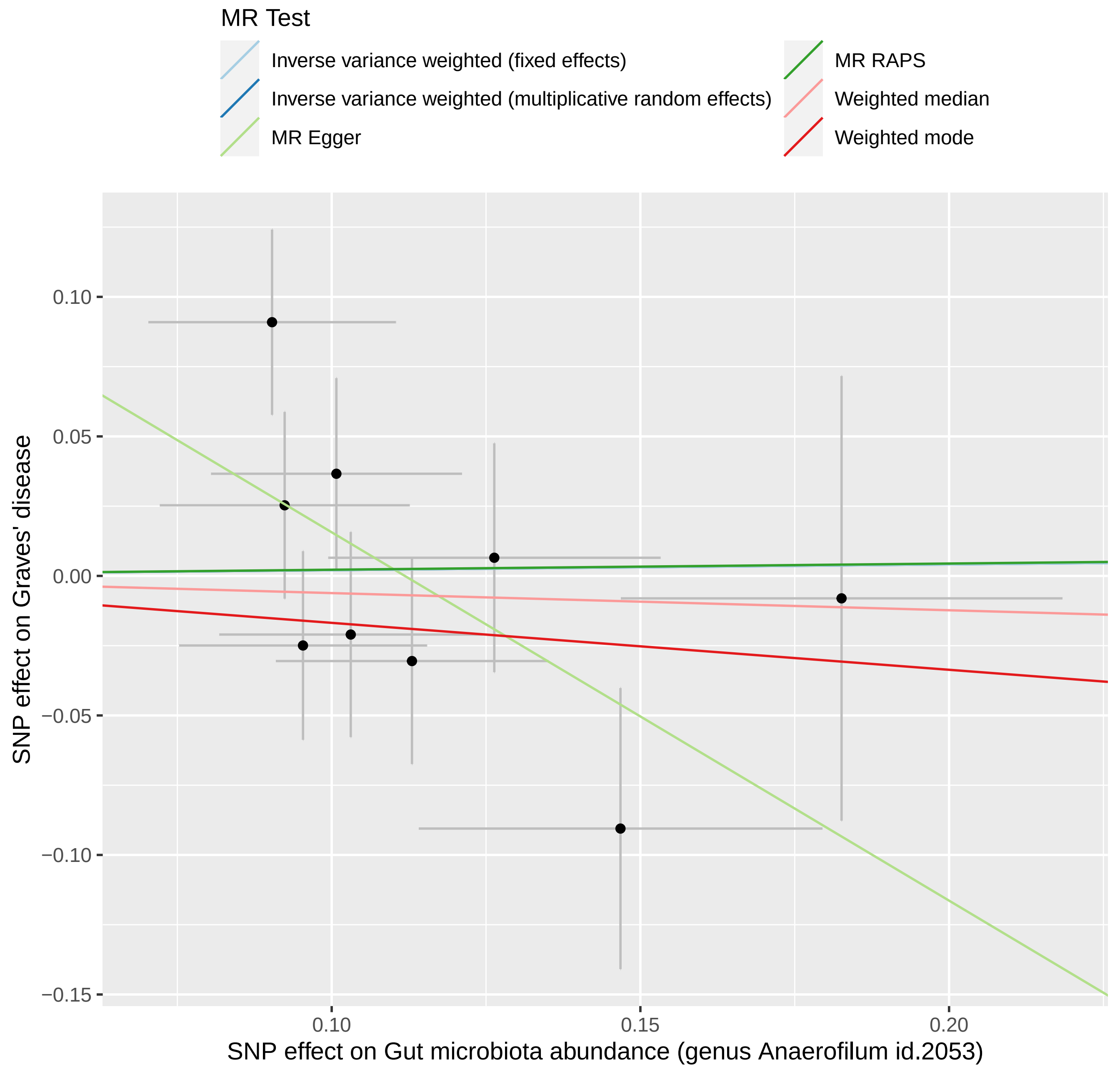

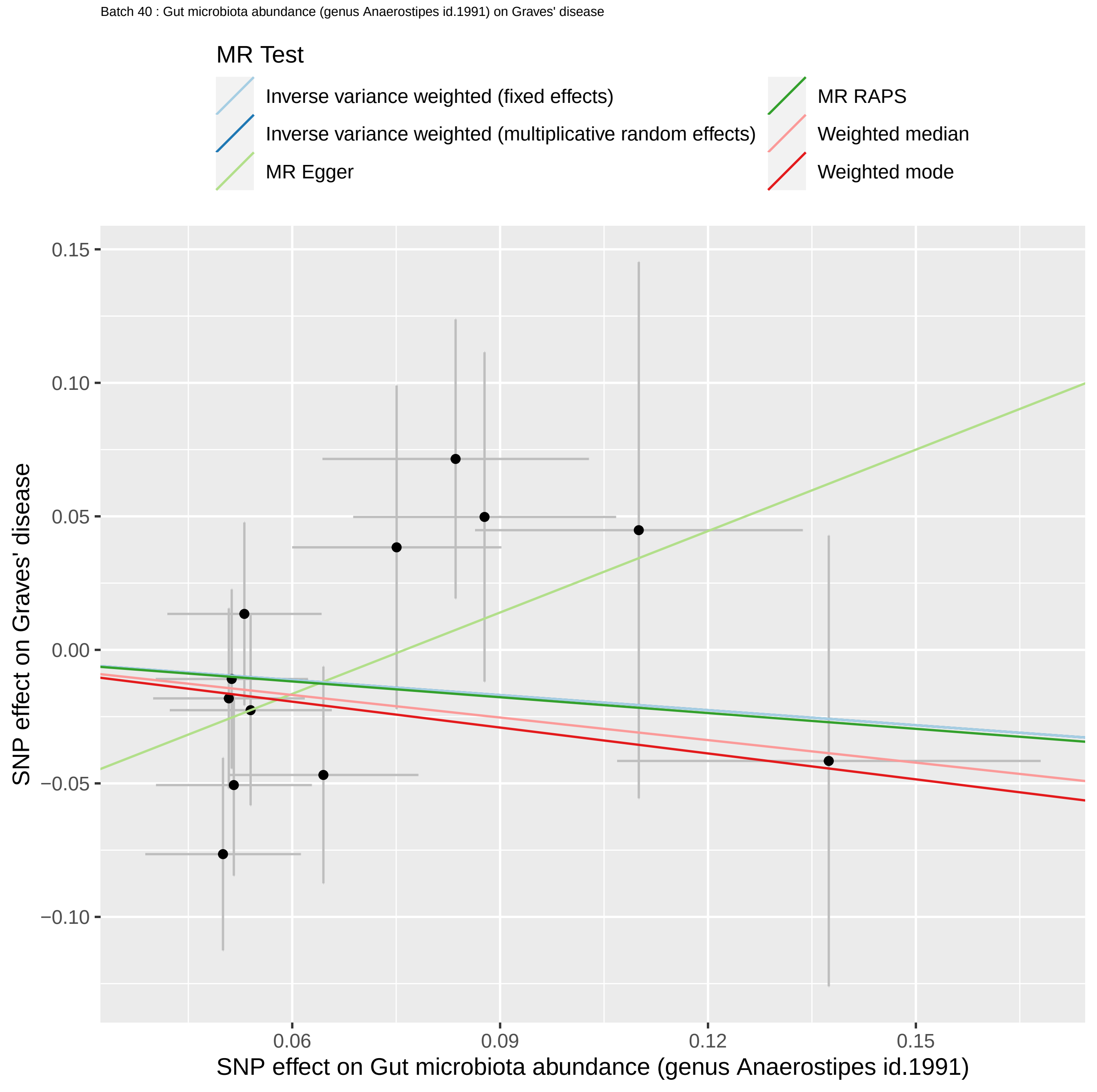

Batch 40 : Gut microbiota abundance (genus Anaerostipes id.1991) on Graves' disease
MR Test
Inverse variance weighted (fixed effects)
Inverse variance weighted (multiplicative random effects)
MR Egger
MR RAPS
Weighted median
Weighted mode
0.15
0.10
0.05
0.00
−0.05
−0.10
0.06
0.09
0.12
0.15
SNP effect on Graves' disease
SNP effect on Gut microbiota abundance (genus Anaerostipes id.1991)

Batch 41 : Gut microbiota abundance (genus Anaerotruncus id.2054) on Graves' disease

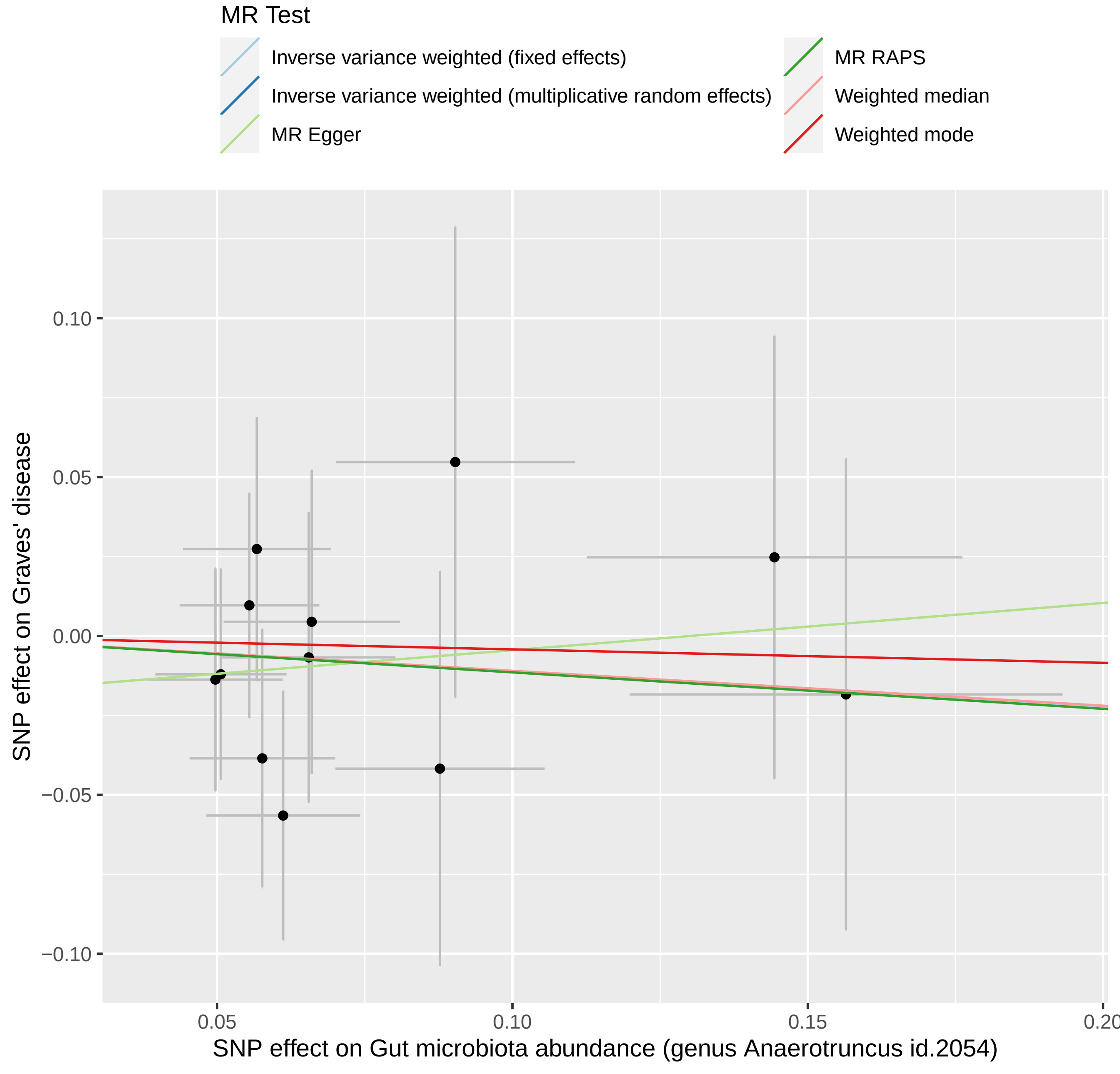

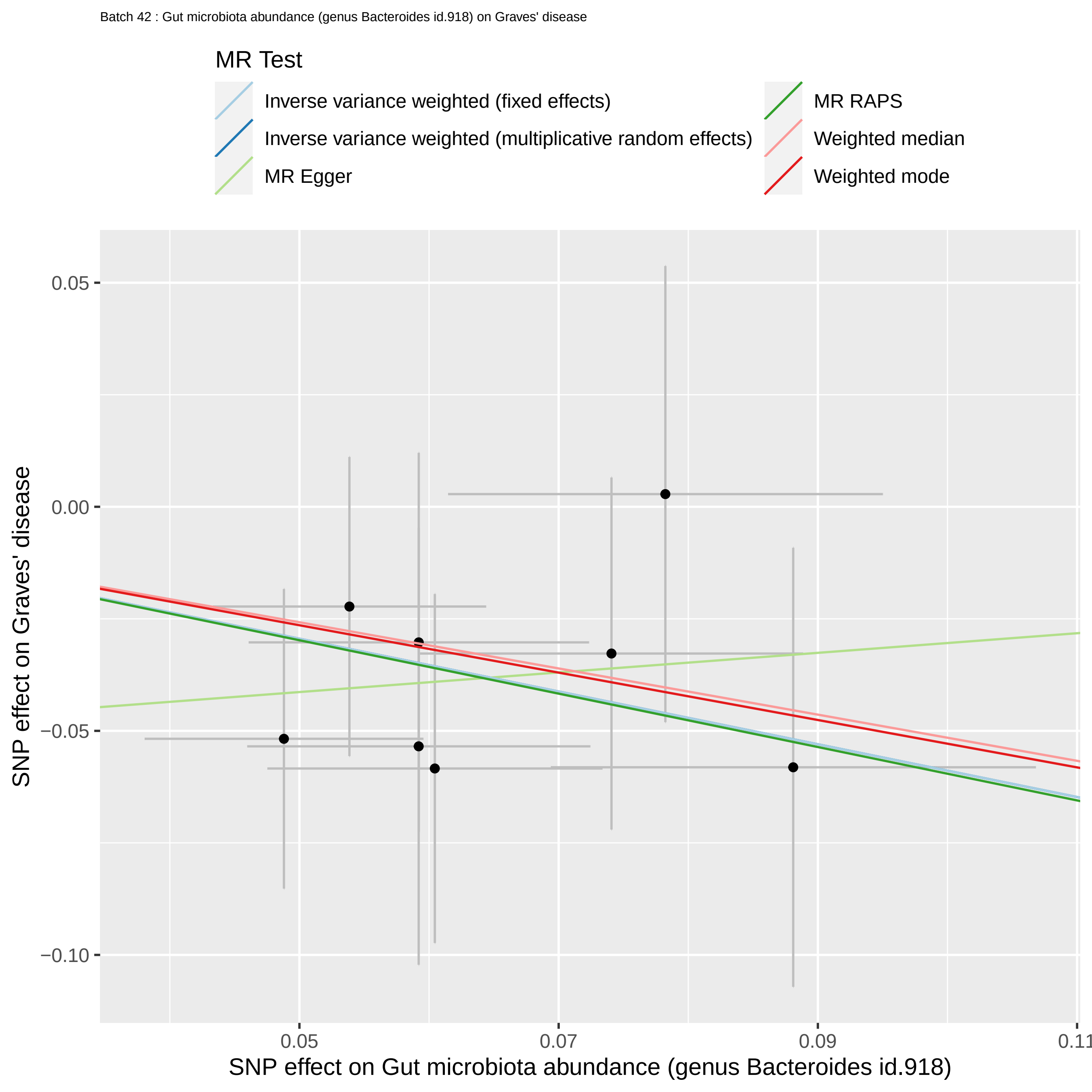
Batch 42 : Gut microbiota abundance (genus Bacteroides id.918) on Graves' disease
MR Test
Inverse variance weighted (fixed effects)
Inverse variance weighted (multiplicative random effects)
MR Egger
MR RAPS
Weighted median
Weighted mode
0.05
0.00
−0.05
−0.10
0.05
0.07
0.09
0.11
SNP effect on Graves' disease
SNP effect on Gut microbiota abundance (genus Bacteroides id.918)

Batch 43 : Gut microbiota abundance (genus Barnesiella id.944) on Graves' disease

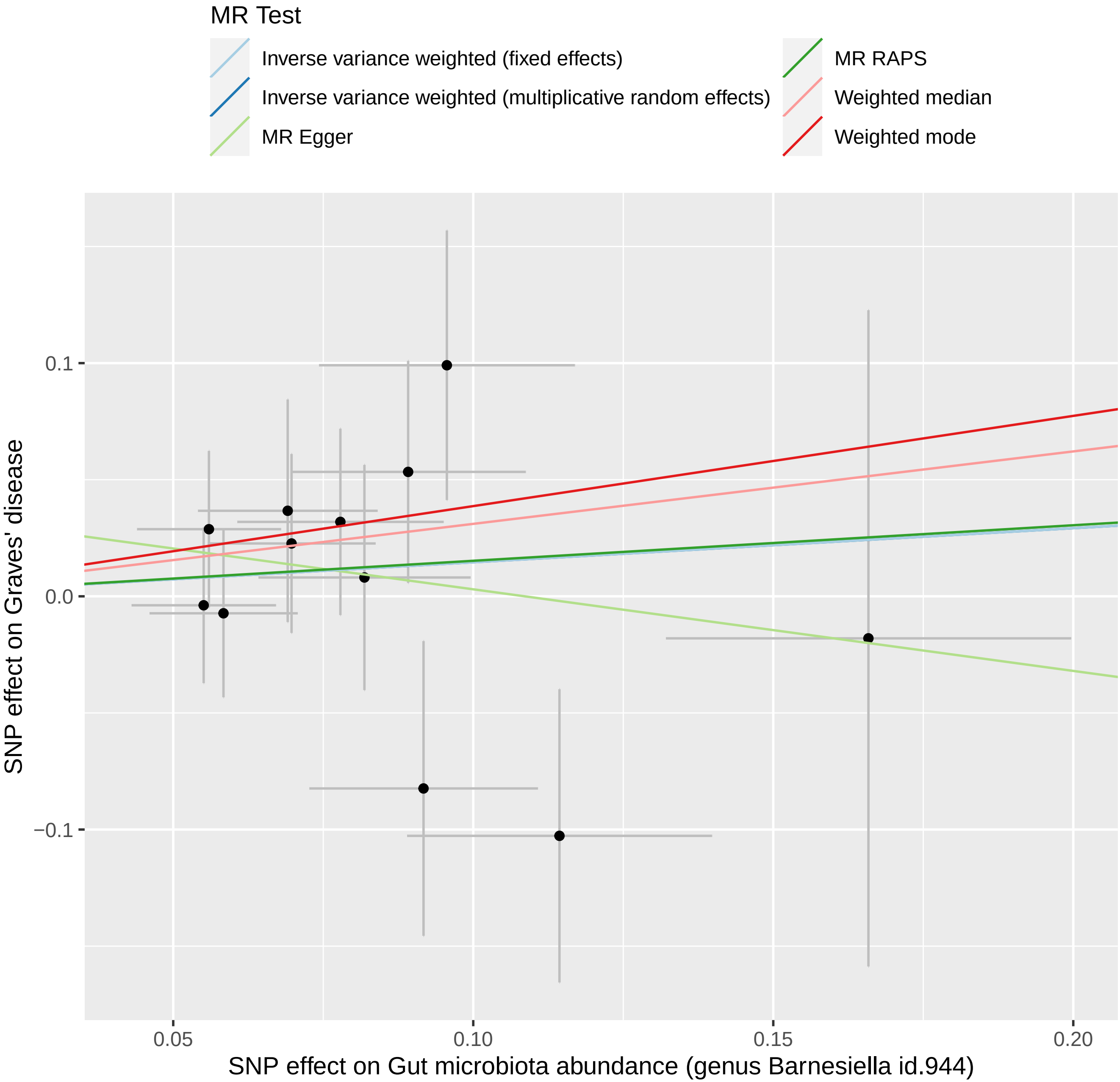

Batch 44 : Gut microbiota abundance (genus Bifidobacterium id.436) on Graves' disease

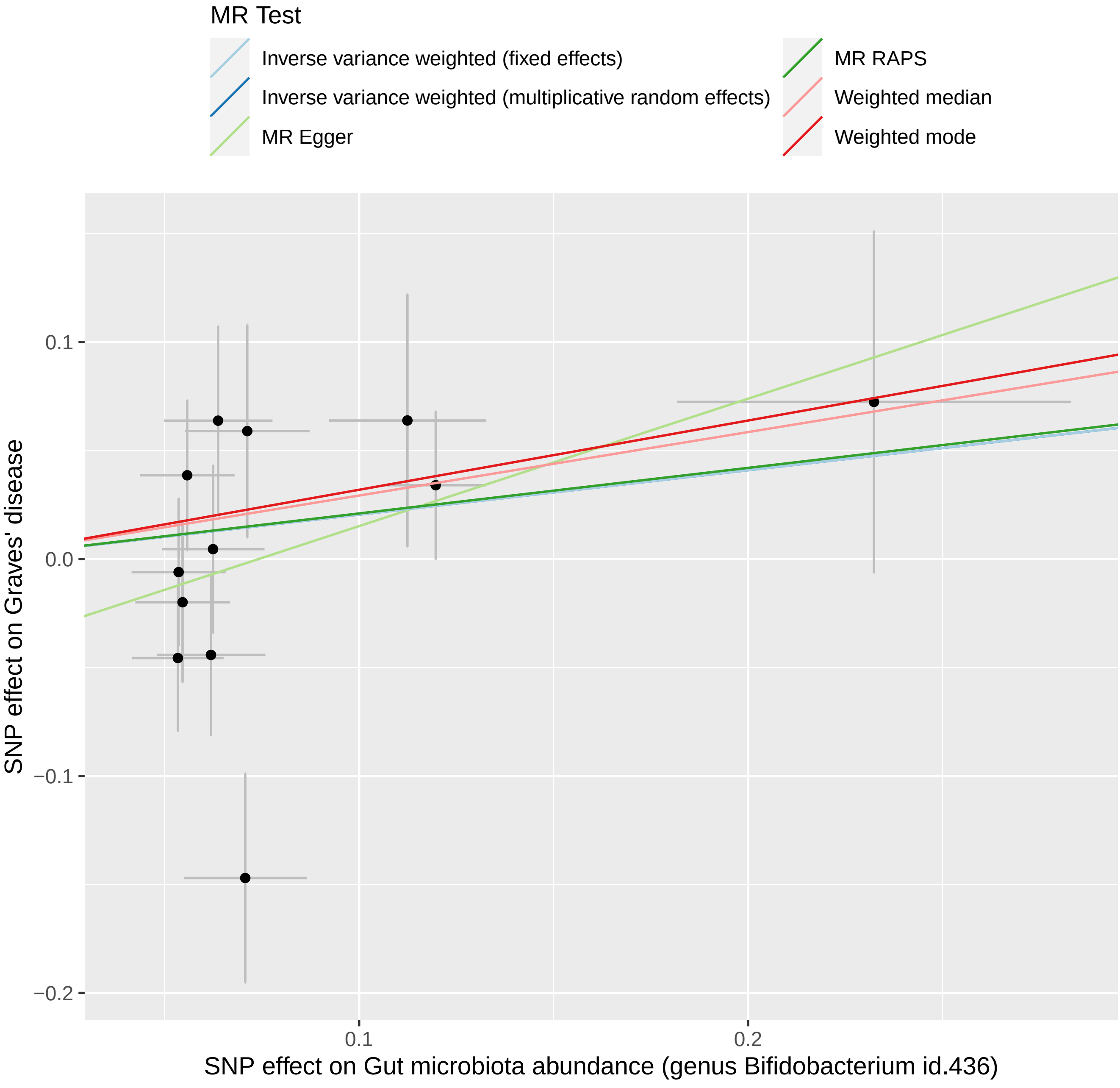

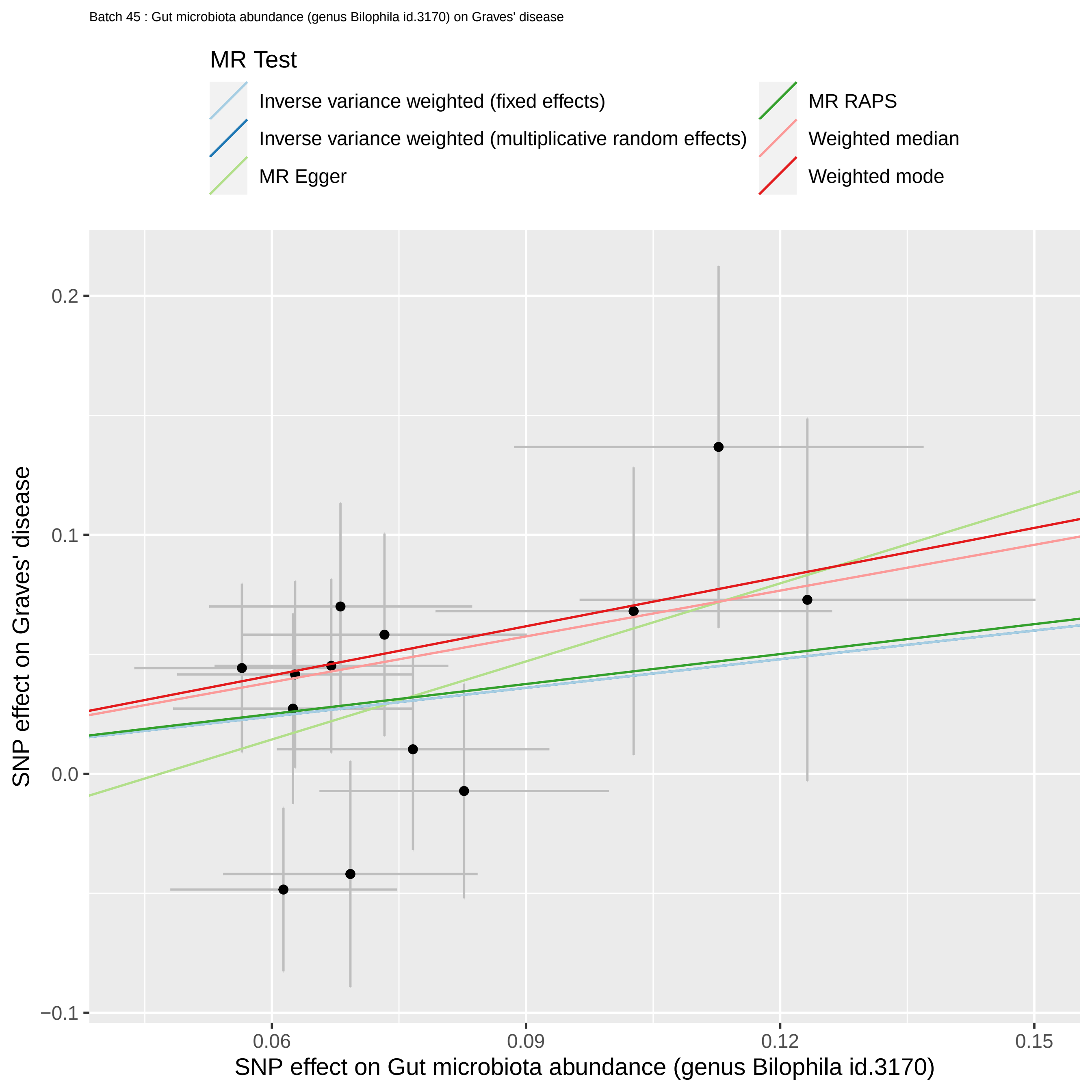

Batch 45 : Gut microbiota abundance (genus Bilophila id.3170) on Graves' disease
MR Test
Inverse variance weighted (fixed effects)
Inverse variance weighted (multiplicative random effects)
MR Egger
MR RAPS
Weighted median
Weighted mode
0.2
0.1
0.0
−0.1
0.06
0.09
0.12
0.15
SNP effect on Graves' disease
SNP effect on Gut microbiota abundance (genus Bilophila id.3170)

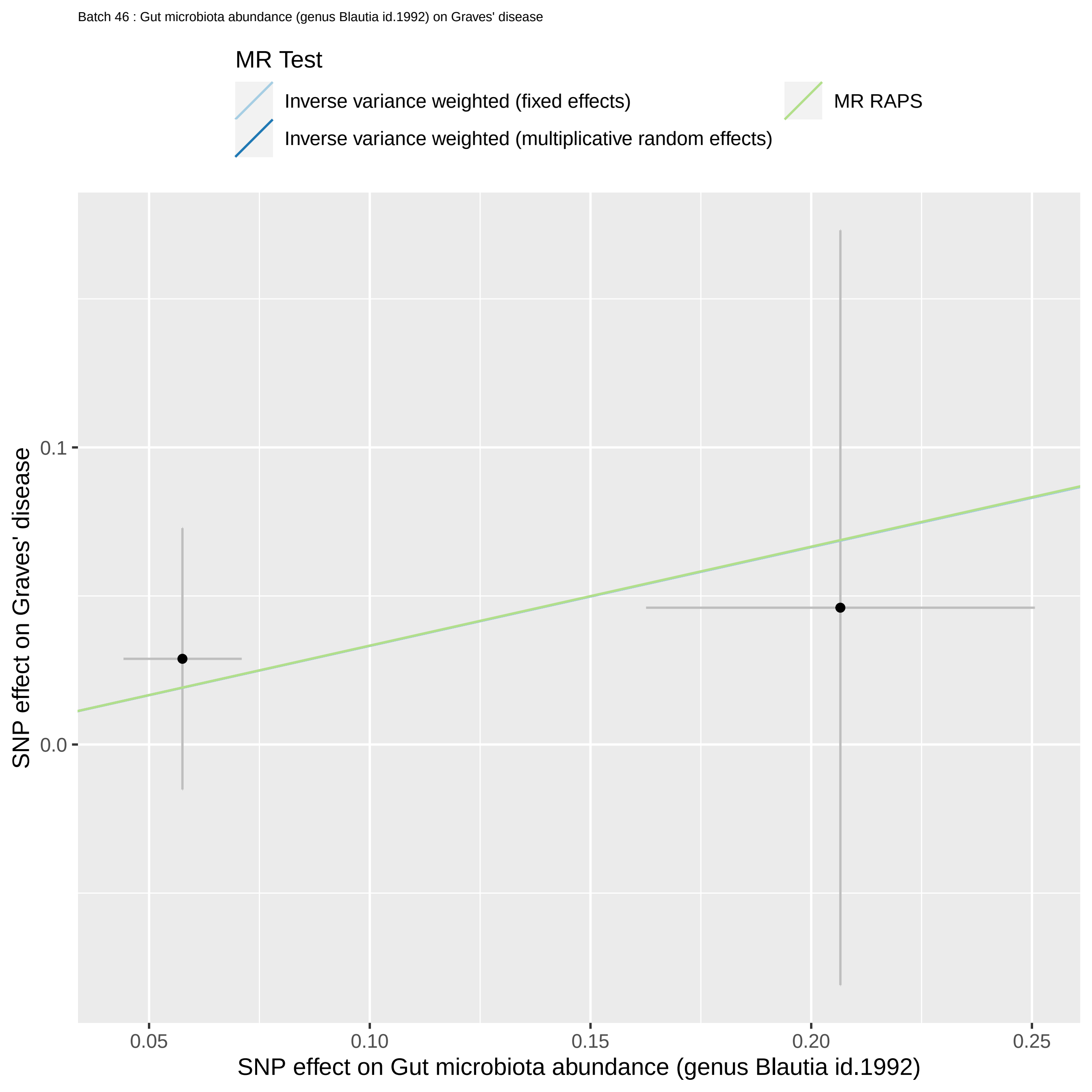
Batch 46 : Gut microbiota abundance (genus Blautia id.1992) on Graves' disease
MR Test
Inverse variance weighted (fixed effects)
Inverse variance weighted (multiplicative random effects)
MR RAPS
SNP effect on Graves' disease
0.1
0.0
0.05
0.10
0.15
0.20
0.25
SNP effect on Gut microbiota abundance (genus Blautia id.1992)

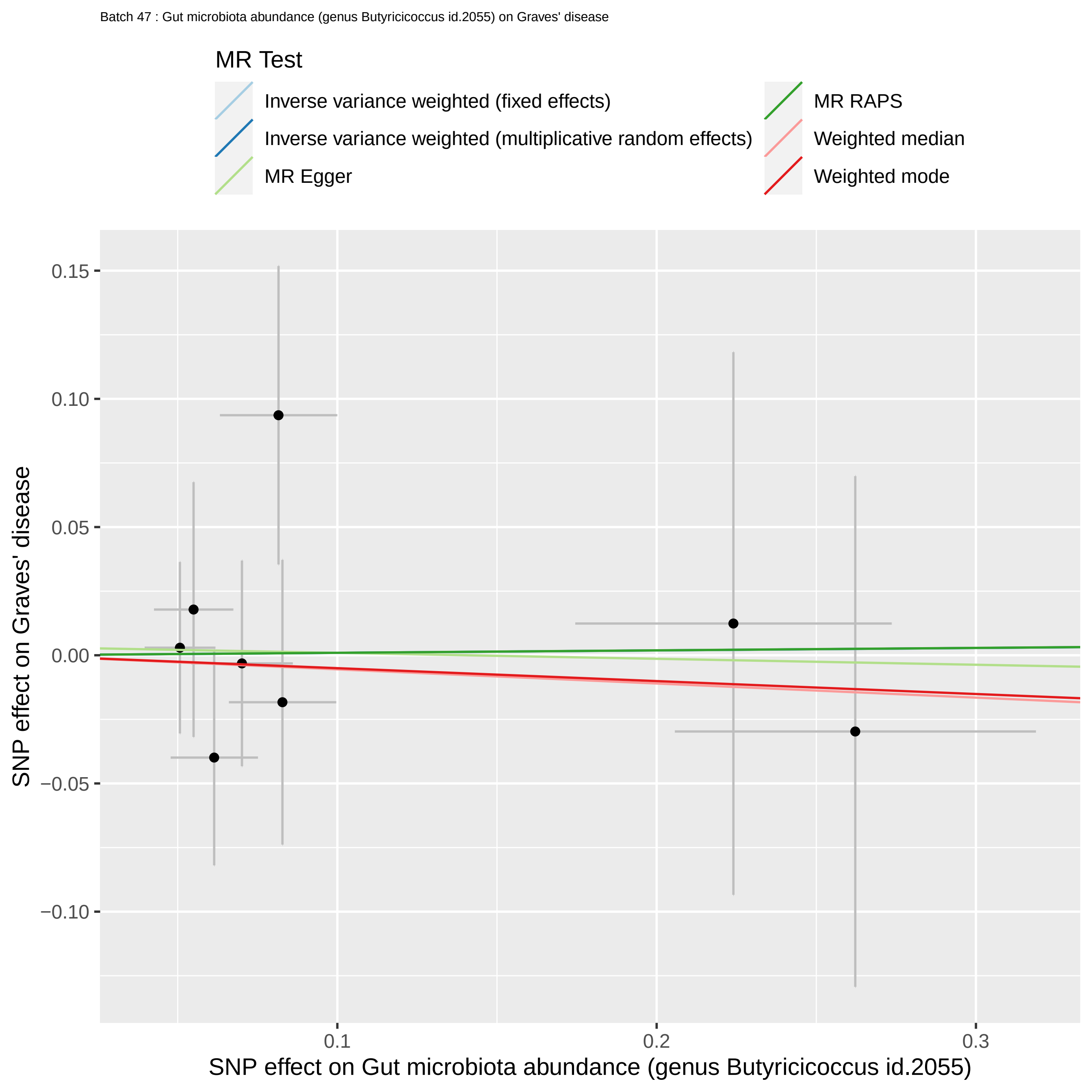
Batch 47 : Gut microbiota abundance (genus Butyricicoccus id.2055) on Graves' disease
MR Test
Inverse variance weighted (fixed effects)
Inverse variance weighted (multiplicative random effects)
MR Egger
MR RAPS
Weighted median
Weighted mode
0.15
0.10
0.05
0.00
−0.05
−0.10
0.1
0.2
0.3
SNP effect on Graves' disease
SNP effect on Gut microbiota abundance (genus Butyricicoccus id.2055)

Batch 48 : Gut microbiota abundance (genus Butyricimonas id.945) on Graves' disease

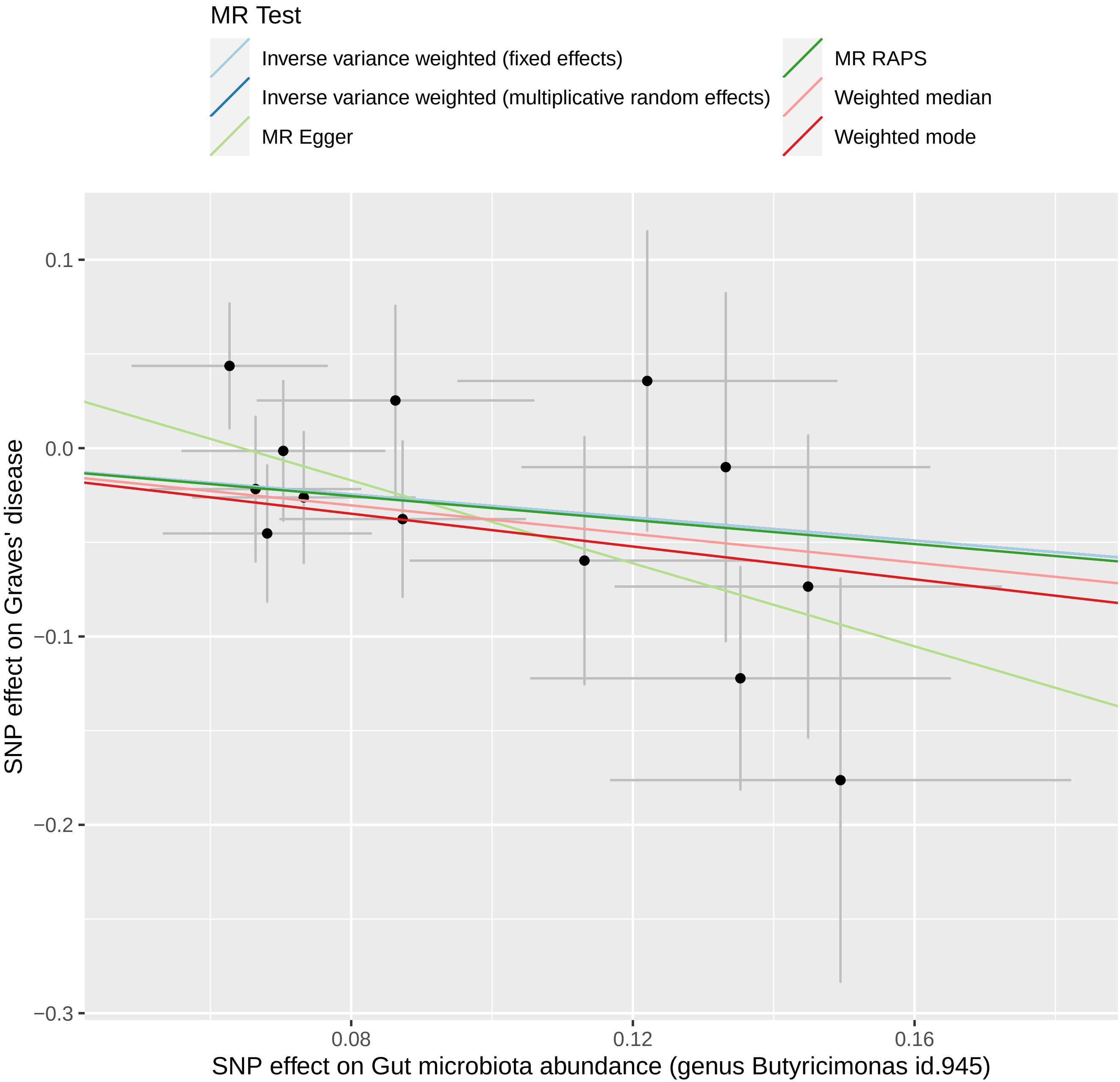

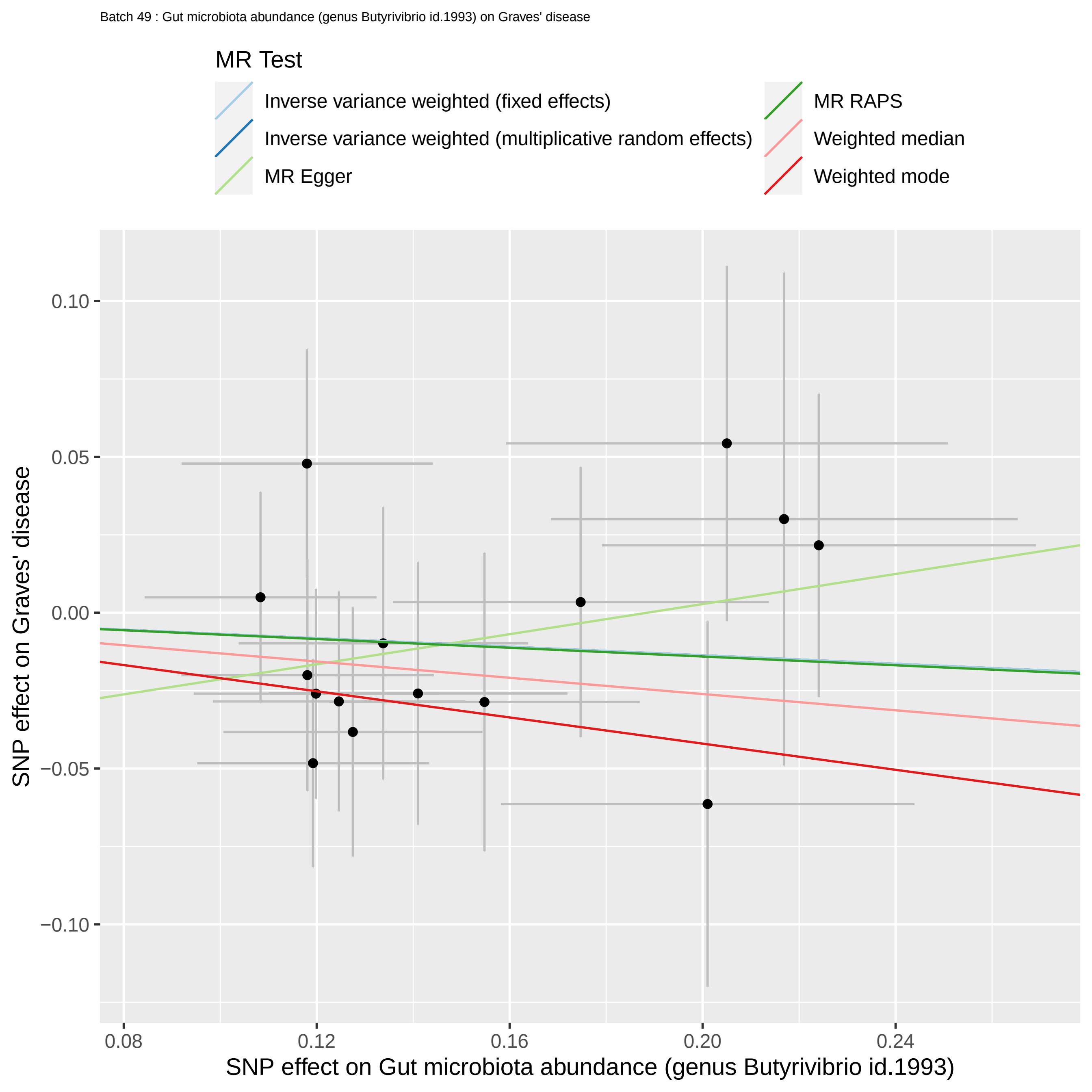

Batch 49 : Gut microbiota abundance (genus Butyrivibrio id.1993) on Graves' disease
MR Test
Inverse variance weighted (fixed effects)
Inverse variance weighted (multiplicative random effects)
MR Egger
MR RAPS
Weighted median
Weighted mode
0.10
0.05
0.00
−0.05
−0.10
SNP effect on Graves' disease
0.08
0.12
0.16
0.20
0.24
SNP effect on Gut microbiota abundance (genus Butyrivibrio id.1993)

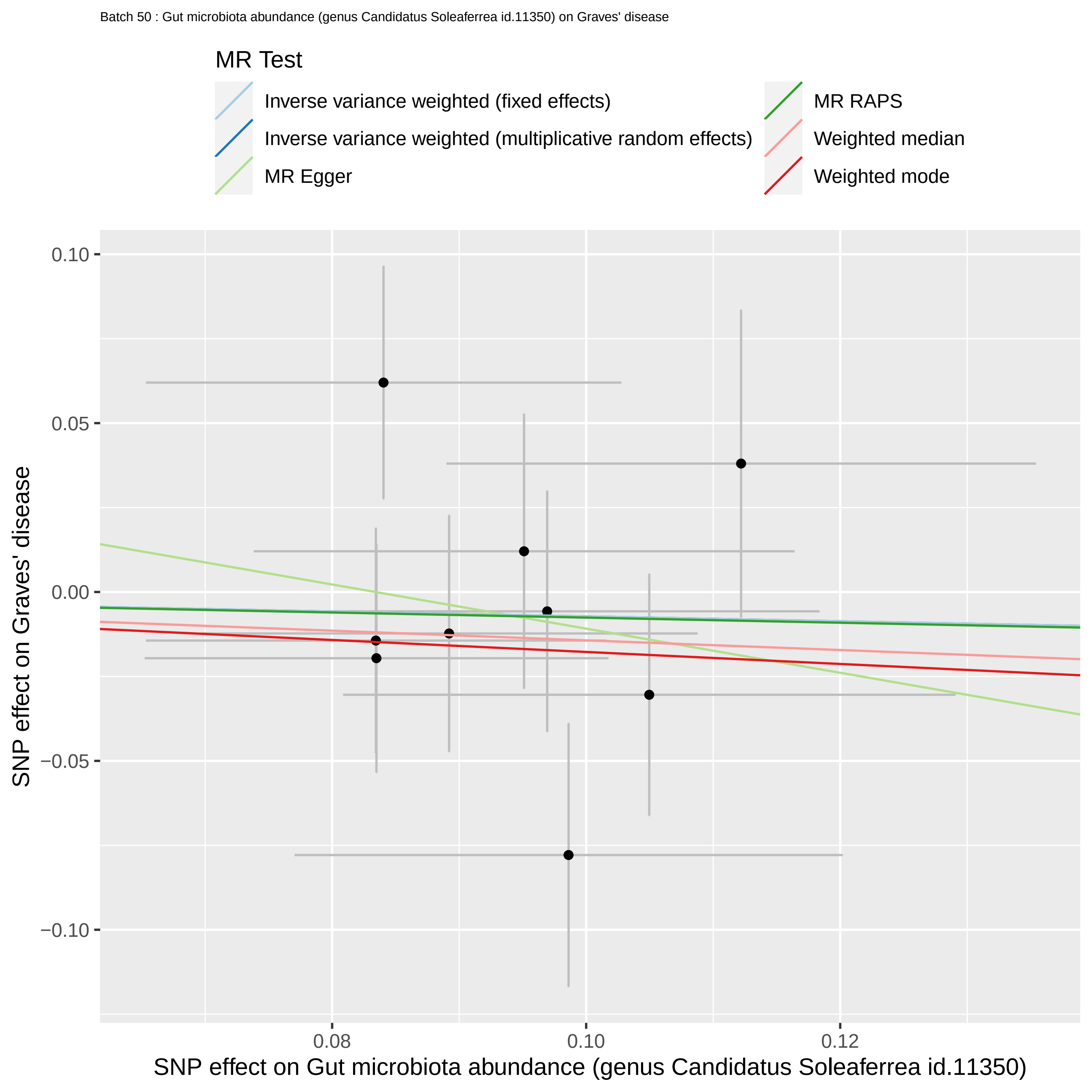

Batch 50 : Gut microbiota abundance (genus Candidatus Soleaferrea id.11350) on Graves' disease
MR Test
Inverse variance weighted (fixed effects)
Inverse variance weighted (multiplicative random effects)
MR Egger
MR RAPS
Weighted median
Weighted mode
0.10
0.05
0.00
−0.05
−0.10
SNP effect on Graves' disease
0.08
0.10
0.12
SNP effect on Gut microbiota abundance (genus Candidatus Soleaferrea id.11350)

Batch 51 : Gut microbiota abundance (genus Catenibacterium id.2153) on Graves' disease

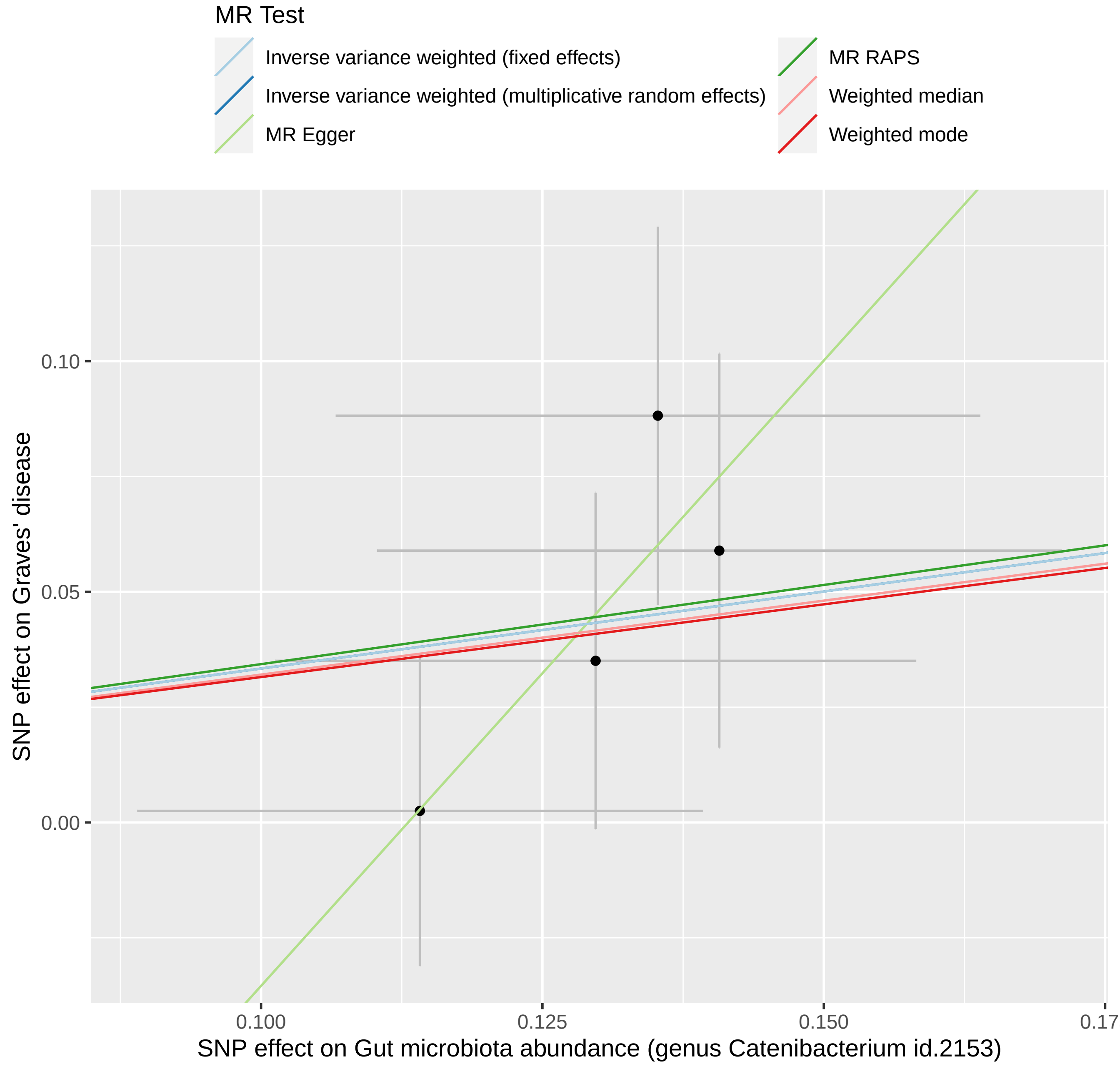

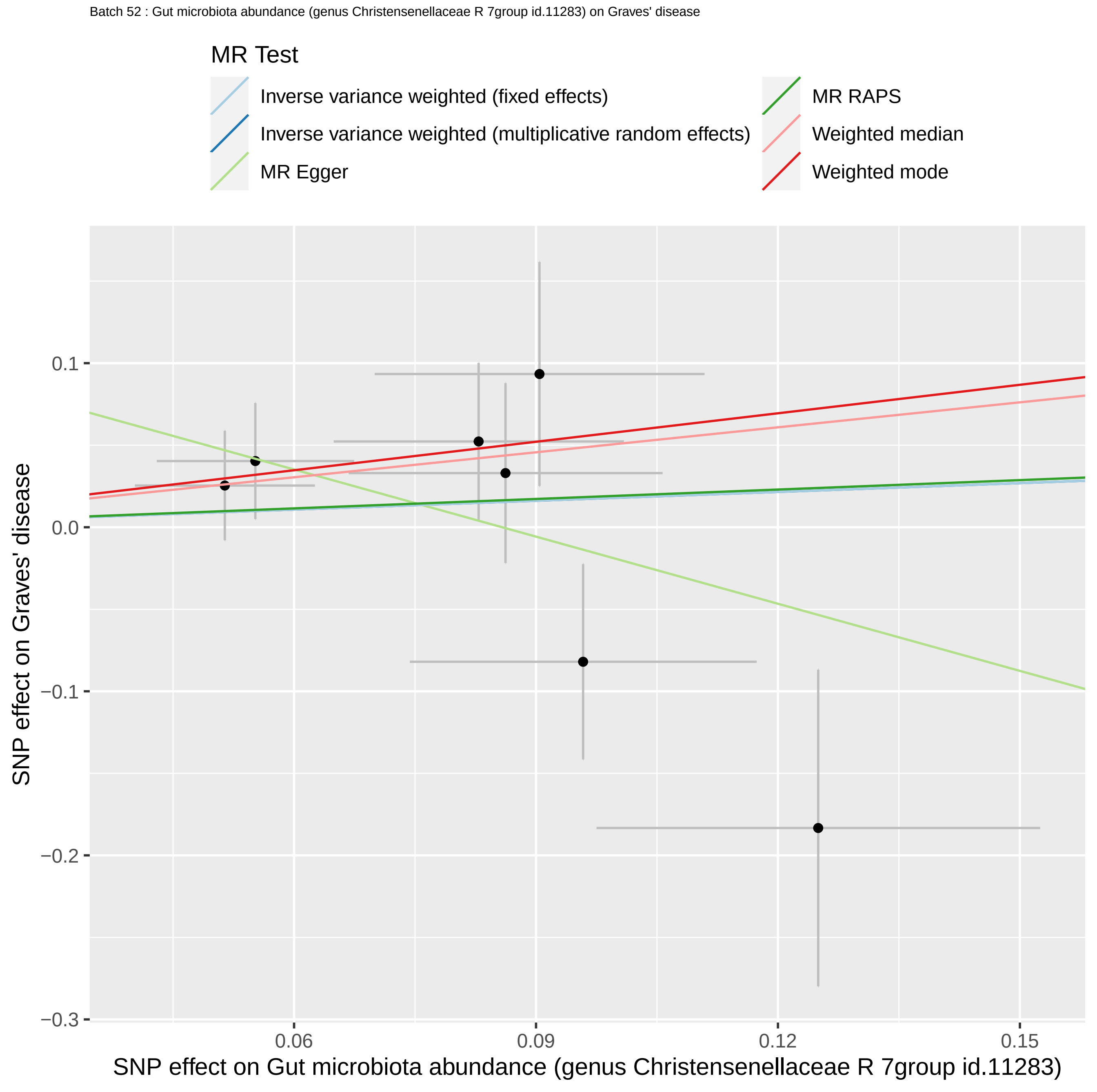
Batch 52 : Gut microbiota abundance (genus Christensenellaceae R 7group id.11283) on Graves' disease
MR Test
Inverse variance weighted (fixed effects)
Inverse variance weighted (multiplicative random effects)
MR Egger
MR RAPS
Weighted median
Weighted mode
0.1
0.0
−0.1
−0.2
−0.3
0.06
0.09
0.12
0.15
SNP effect on Graves' disease
SNP effect on Gut microbiota abundance (genus Christensenellaceae R 7group id.11283)

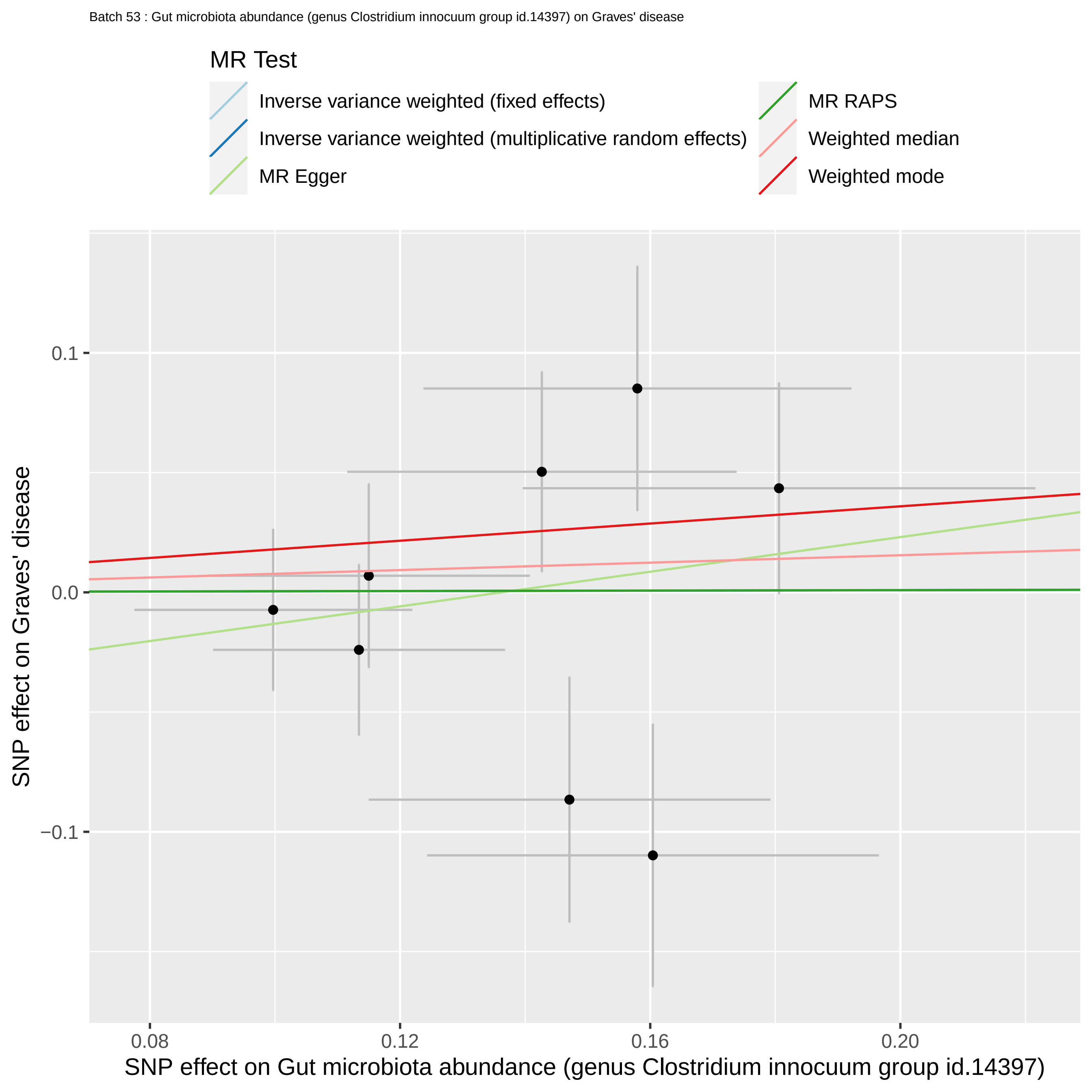

Batch 53 : Gut microbiota abundance (genus Clostridium innocuum group id.14397) on Graves' disease
MR Test
Inverse variance weighted (fixed effects)
Inverse variance weighted (multiplicative random effects)
MR Egger
MR RAPS
Weighted median
Weighted mode
0.1
0.0
−0.1
0.08
0.12
0.16
0.20
SNP effect on Graves' disease
SNP effect on Gut microbiota abundance (genus Clostridium innocuum group id.14397)

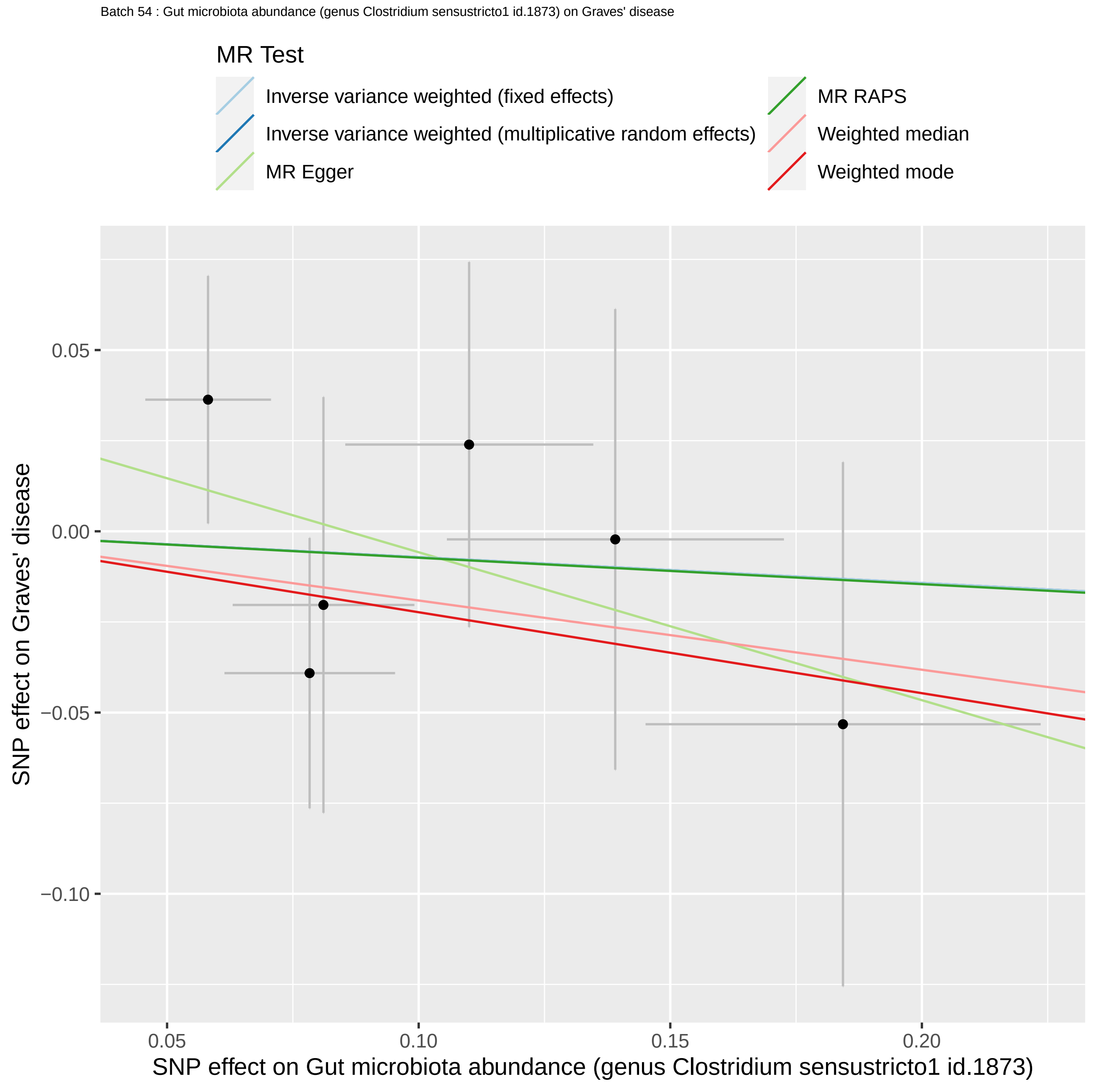
Batch 54 : Gut microbiota abundance (genus Clostridium sensustricto1 id.1873) on Graves' disease
MR Test
Inverse variance weighted (fixed effects)
Inverse variance weighted (multiplicative random effects)
MR Egger
MR RAPS
Weighted median
Weighted mode
0.05
0.00
−0.05
−0.10
0.05
0.10
0.15
0.20
SNP effect on Graves' disease
SNP effect on Gut microbiota abundance (genus Clostridium sensustricto1 id.1873)

Batch 55 : Gut microbiota abundance (genus Collinsella id.815) on Graves' disease

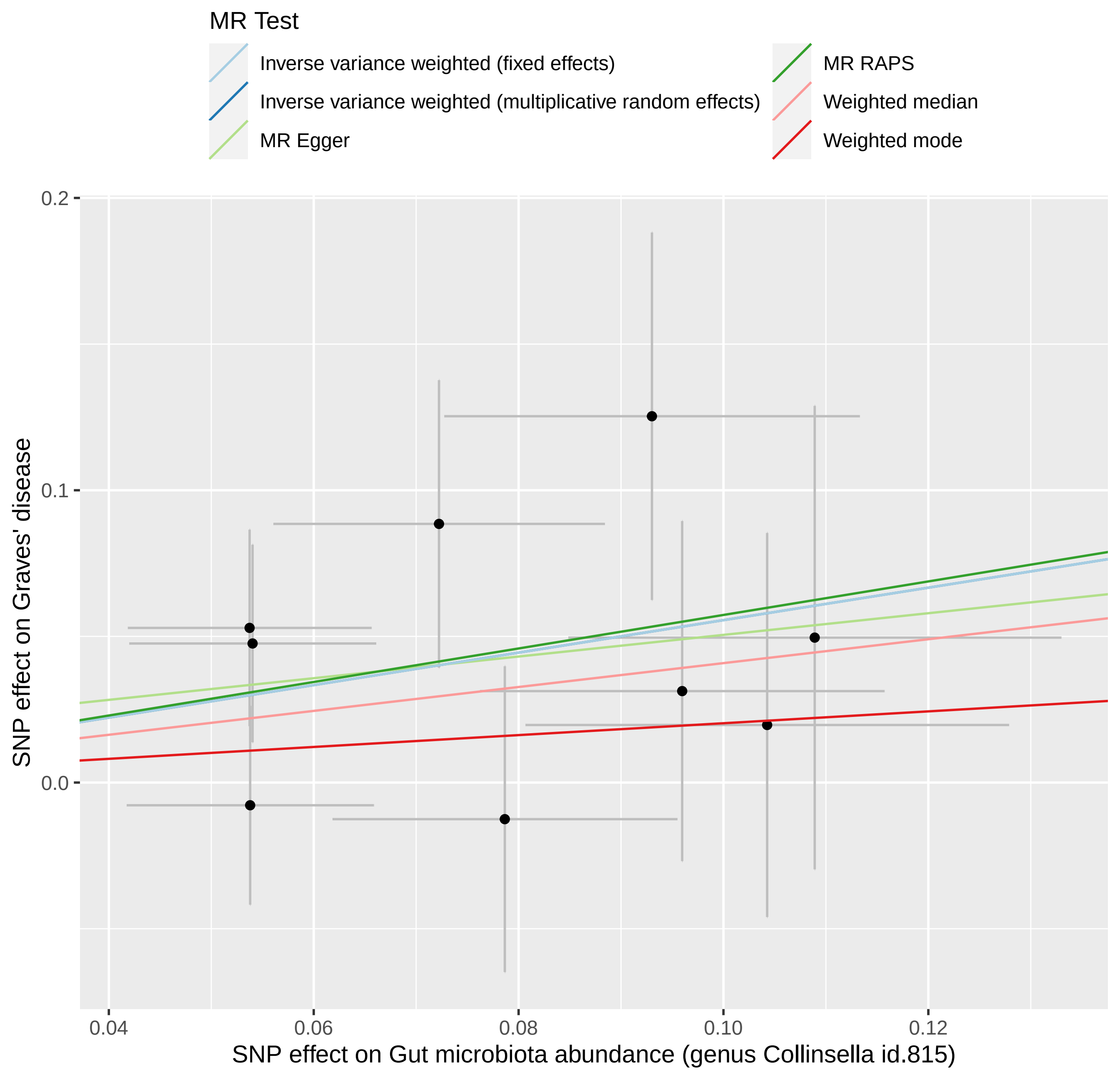

Batch 56 : Gut microbiota abundance (genus Coprobacter id.949) on Graves' disease

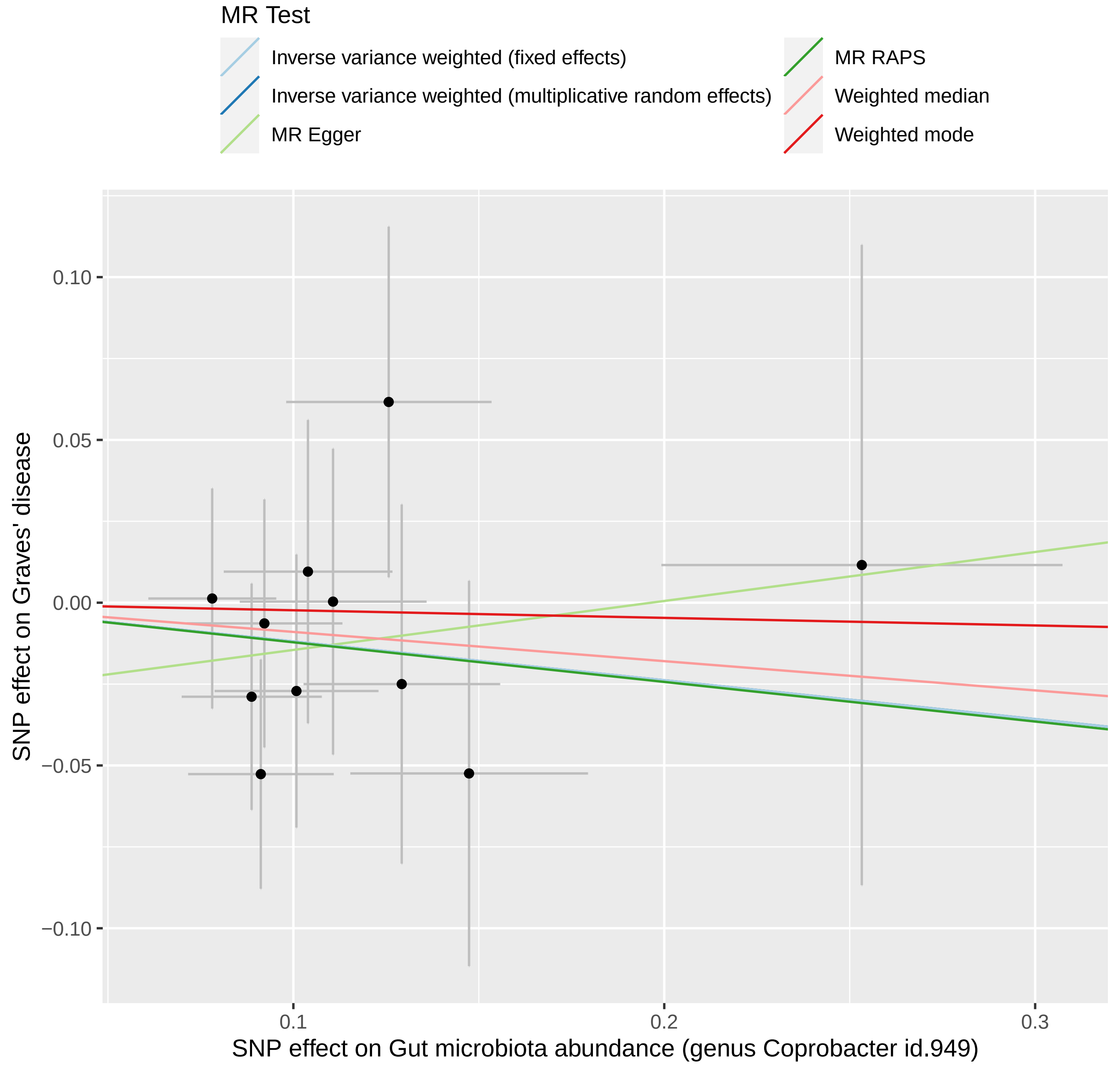

Batch 57 : Gut microbiota abundance (genus Coprococcus1 id.11301) on Graves' disease

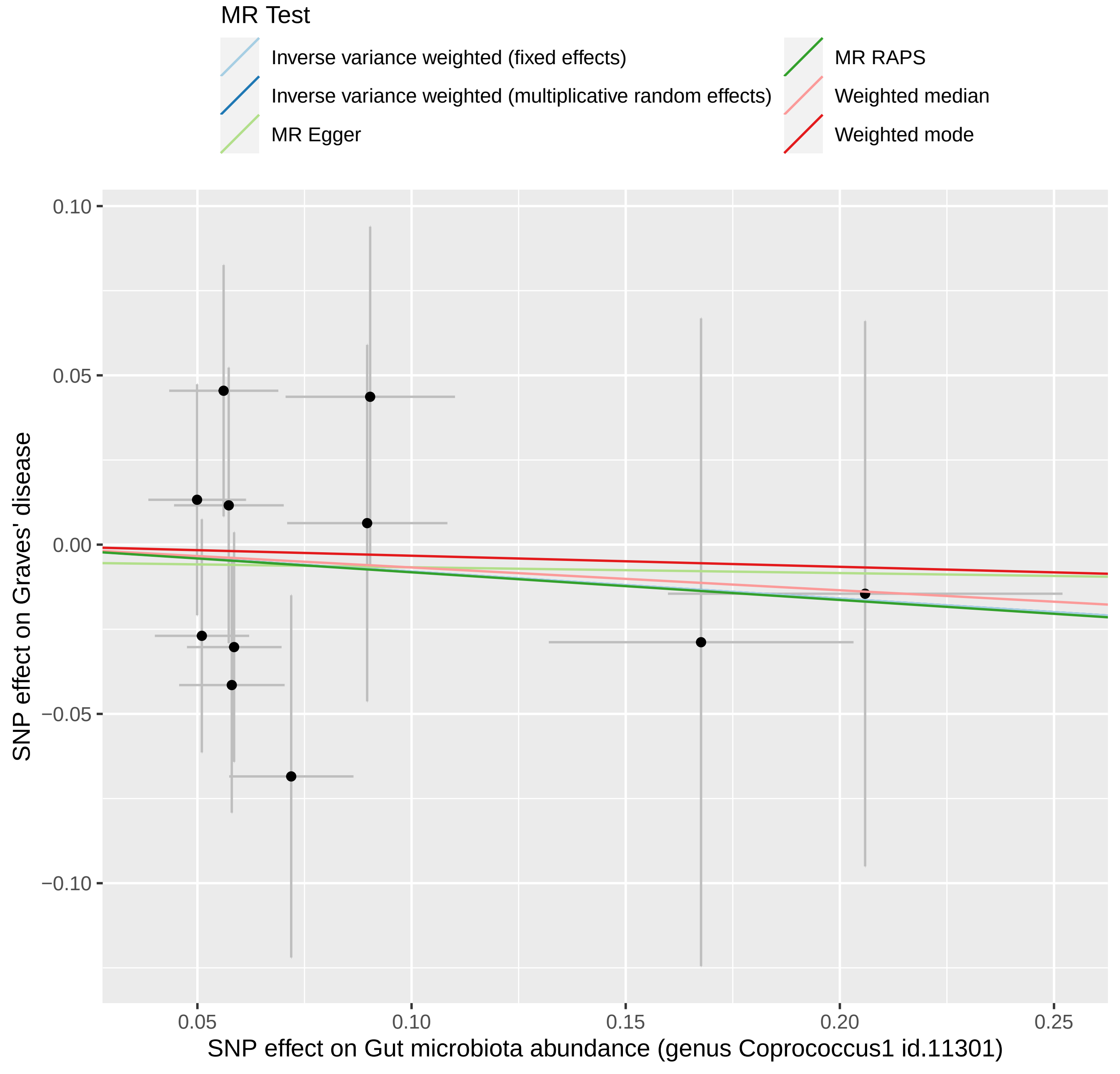

Batch 58 : Gut microbiota abundance (genus Coprococcus2 id.11302) on Graves' disease

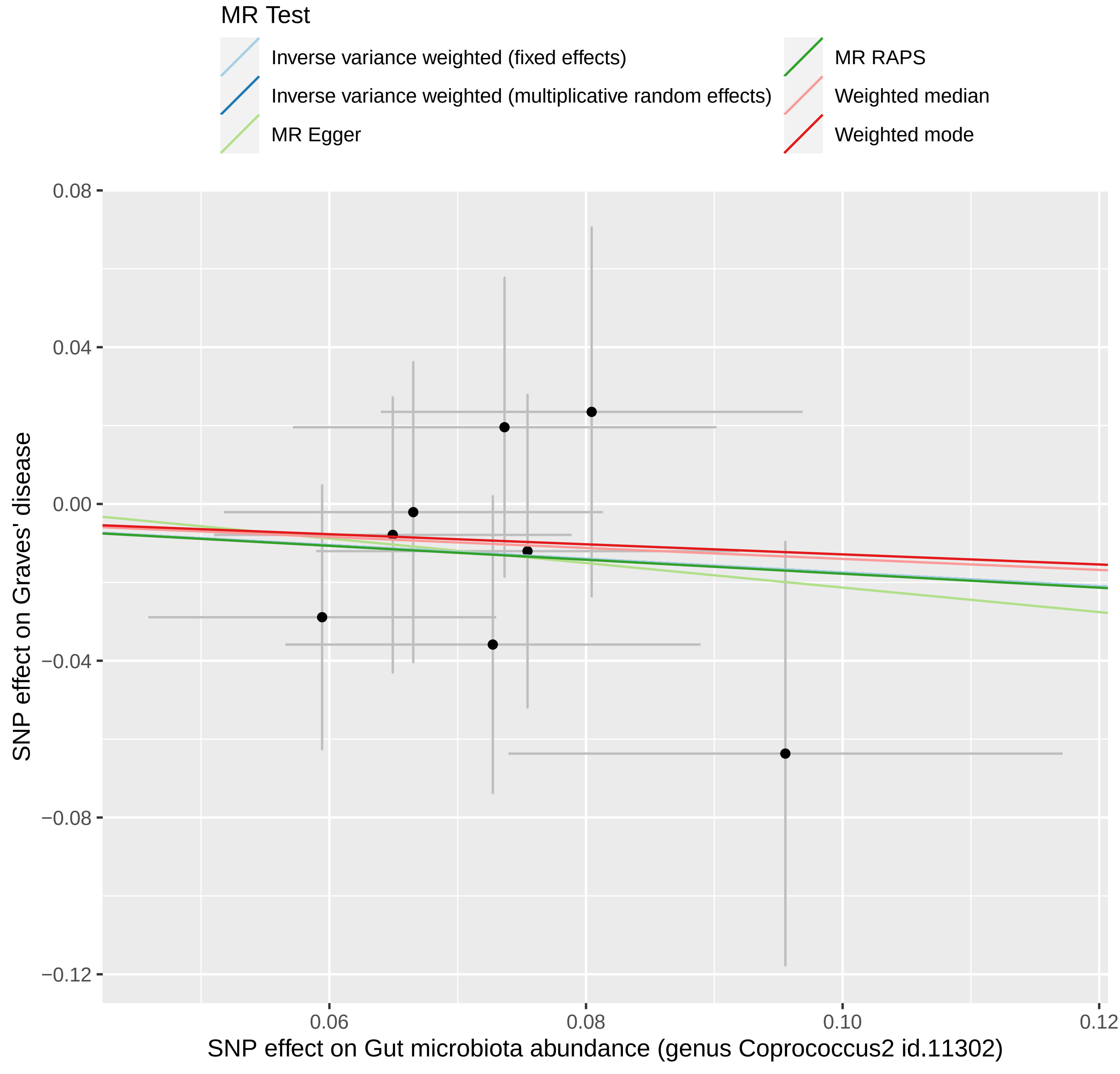

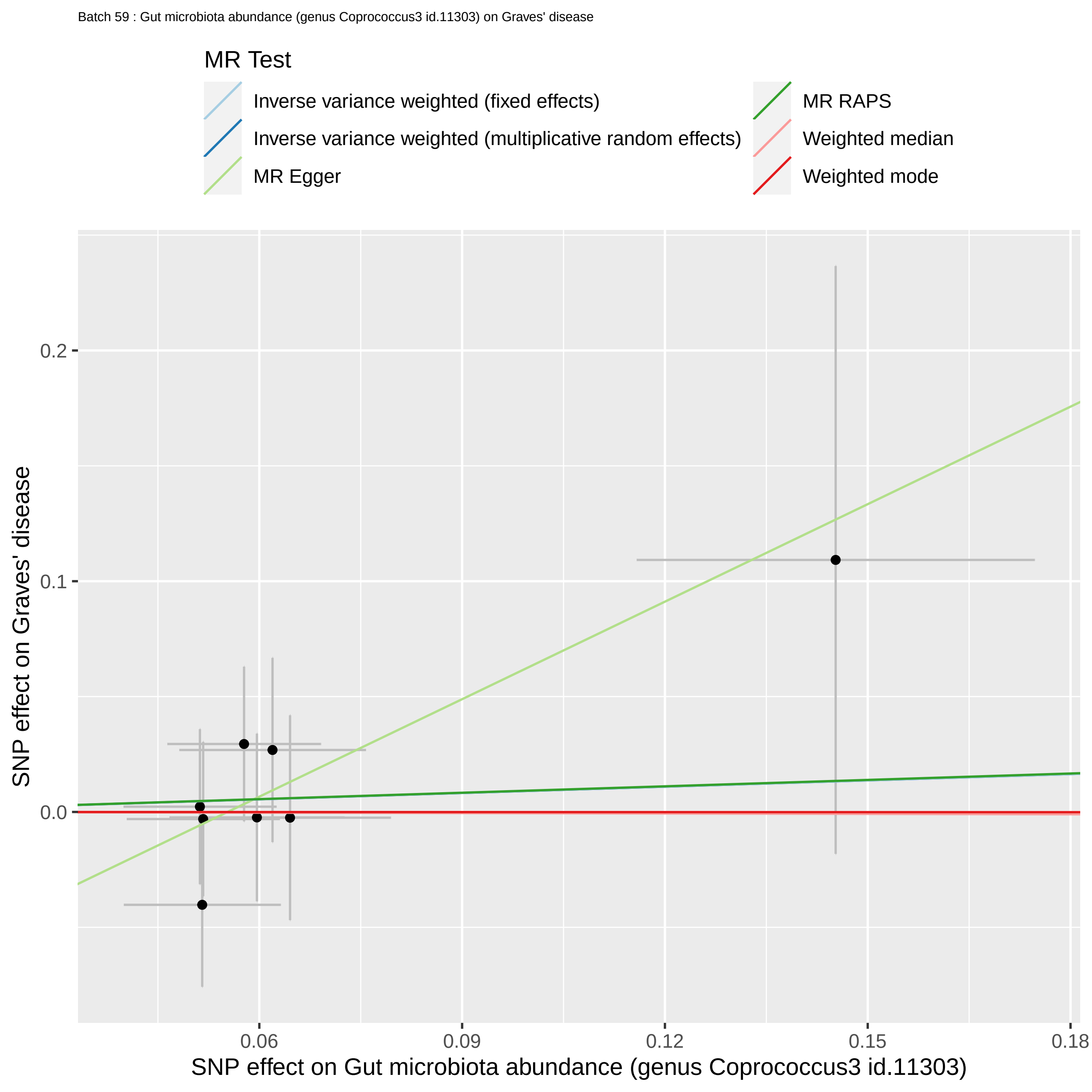
Batch 59 : Gut microbiota abundance (genus Coprococcus3 id.11303) on Graves' disease
MR Test
Inverse variance weighted (fixed effects)
Inverse variance weighted (multiplicative random effects)
MR Egger
MR RAPS
Weighted median
Weighted mode
SNP effect on Graves' disease
0.2
0.1
0.0
0.06
0.09
0.12
0.15
0.18
SNP effect on Gut microbiota abundance (genus Coprococcus3 id.11303)

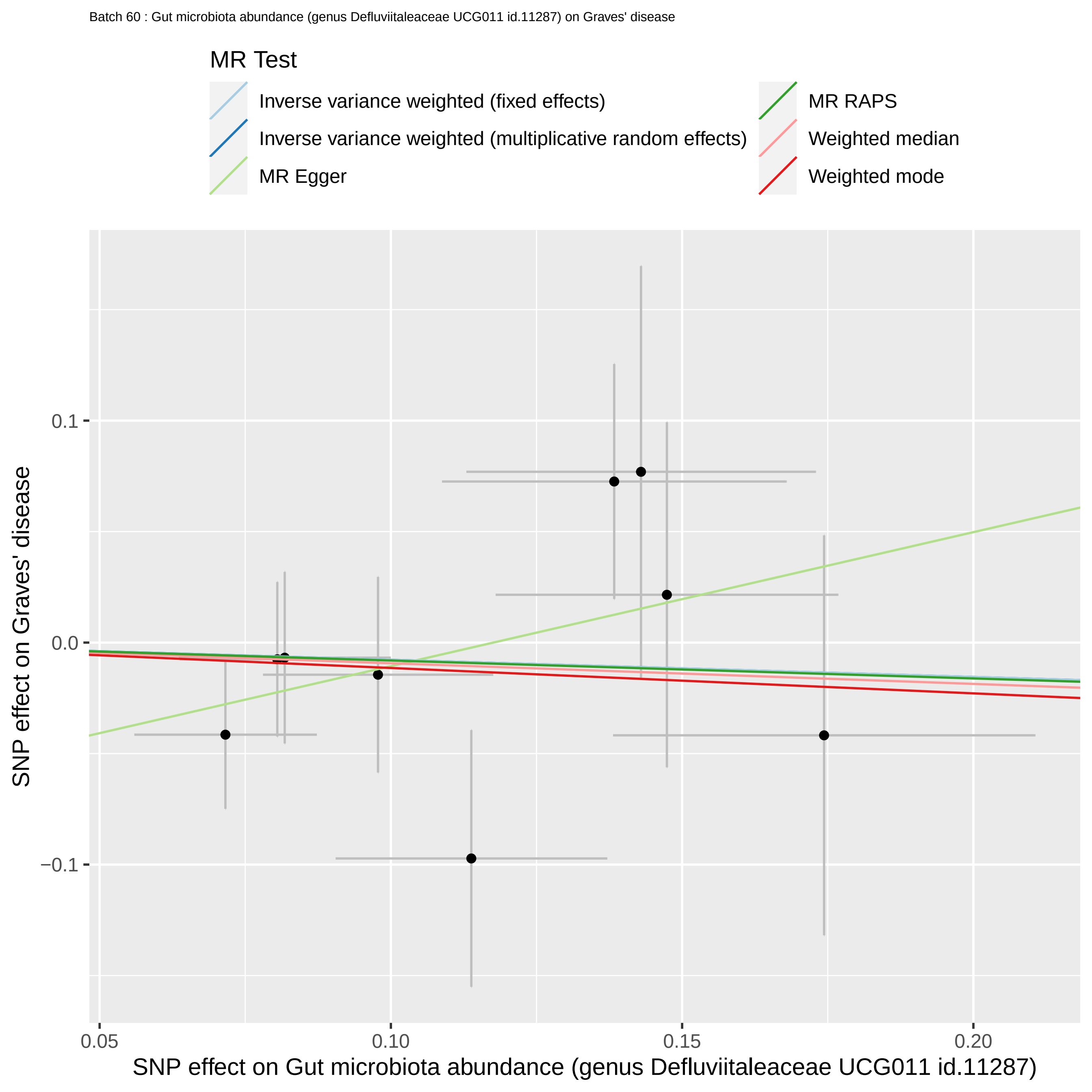
Batch 60 : Gut microbiota abundance (genus Defluviitaleaceae UCG011 id.11287) on Graves' disease
MR Test
Inverse variance weighted (fixed effects)
Inverse variance weighted (multiplicative random effects)
MR Egger
MR RAPS
Weighted median
Weighted mode
0.1
0.0
−0.1
0.05
0.10
0.15
0.20
SNP effect on Graves' disease
SNP effect on Gut microbiota abundance (genus Defluviitaleaceae UCG011 id.11287)

Batch 61 : Gut microbiota abundance (genus Desulfovibrio id.3173) on Graves' disease

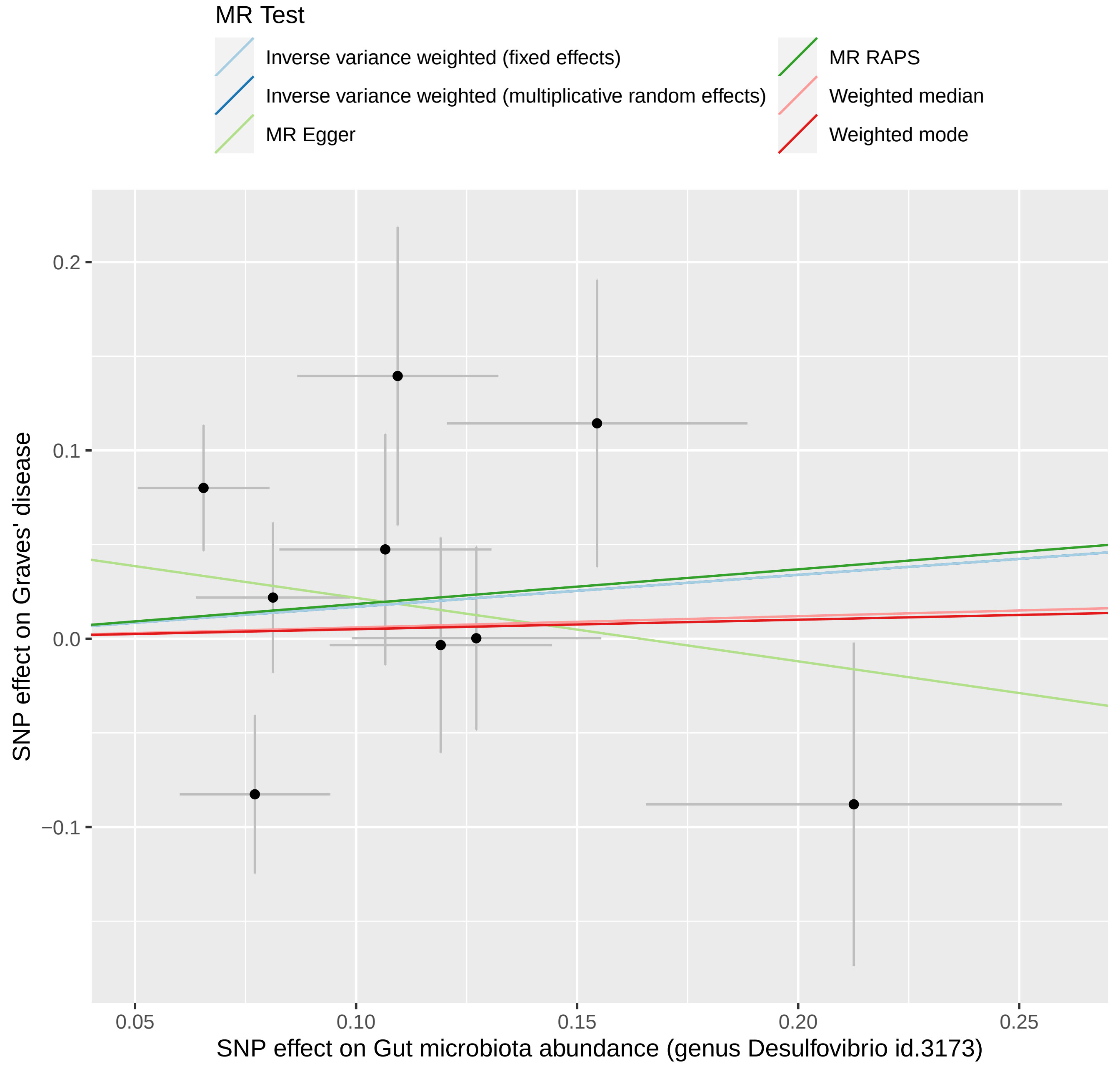

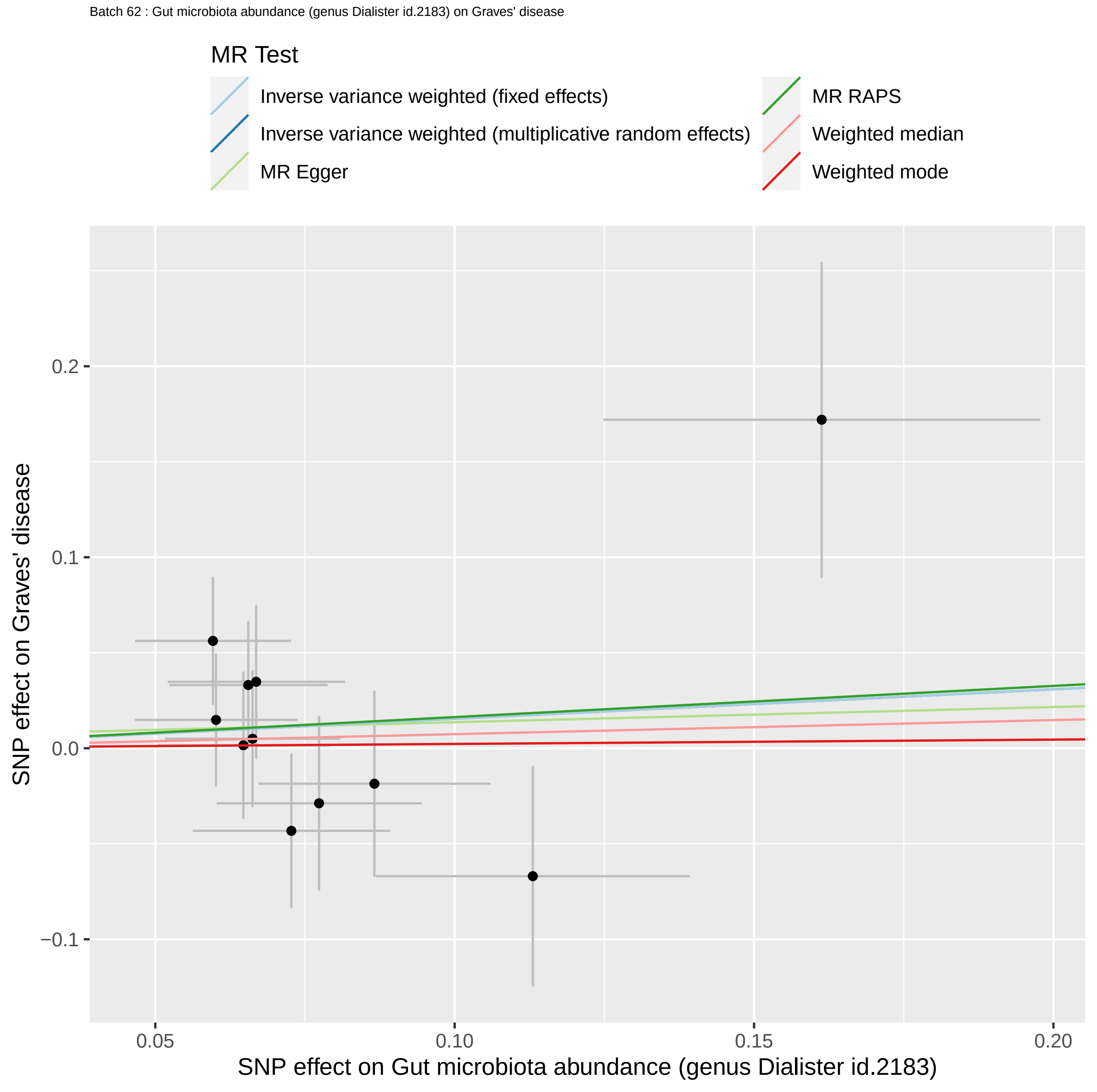

Batch 62 : Gut microbiota abundance (genus Dialister id.2183) on Graves' disease
MR Test
Inverse variance weighted (fixed effects)
Inverse variance weighted (multiplicative random effects)
MR Egger
MR RAPS
Weighted median
Weighted mode
SNP effect on Graves' disease
0.2
0.1
0.0
−0.1
0.05
0.10
0.15
0.20
SNP effect on Gut microbiota abundance (genus Dialister id.2183)

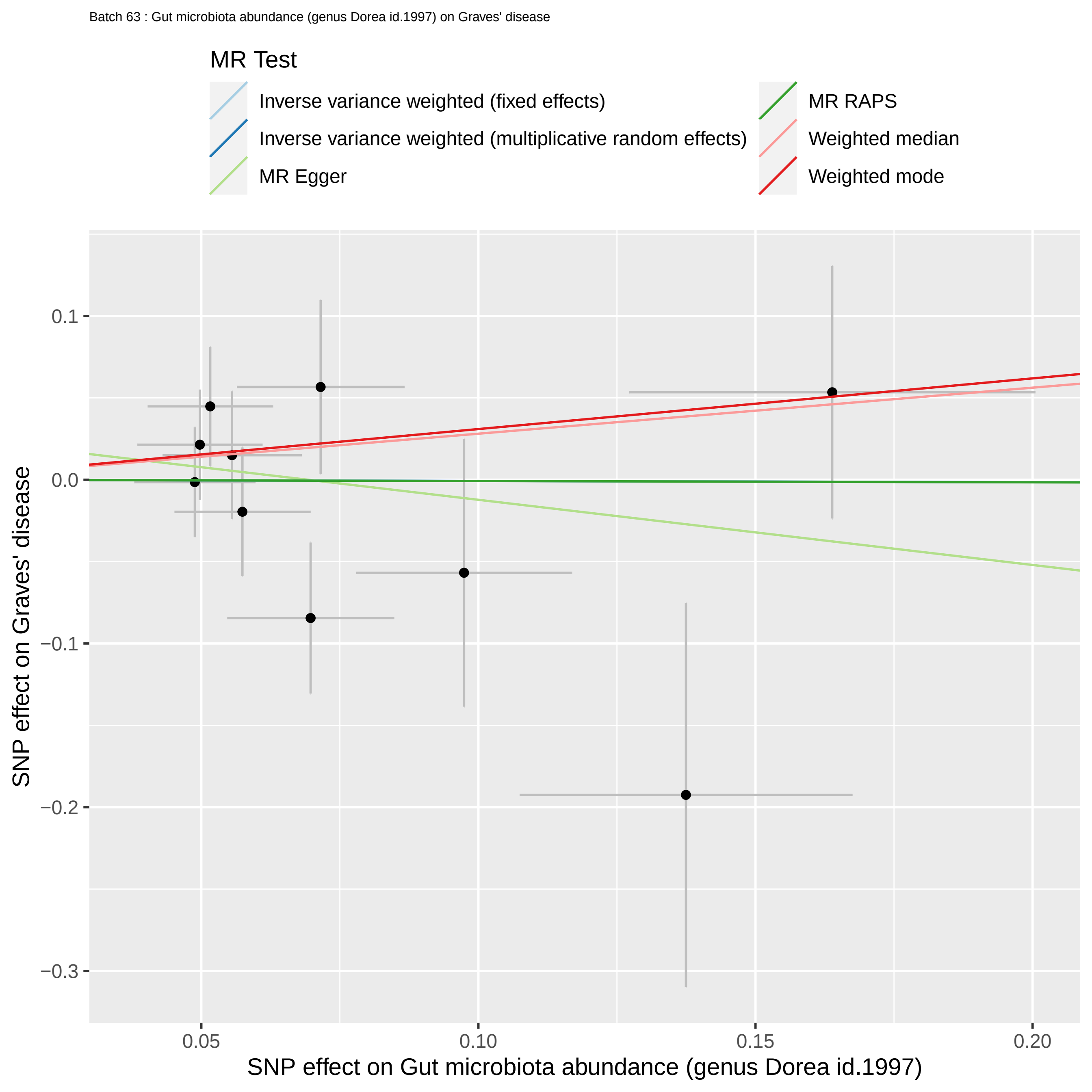

Batch 63 : Gut microbiota abundance (genus Dorea id.1997) on Graves' disease
MR Test
Inverse variance weighted (fixed effects)
Inverse variance weighted (multiplicative random effects)
MR Egger
MR RAPS
Weighted median
Weighted mode
0.1
0.0
−0.1
−0.2
−0.3
0.05
0.10
0.15
0.20
SNP effect on Graves' disease
SNP effect on Gut microbiota abundance (genus Dorea id.1997)

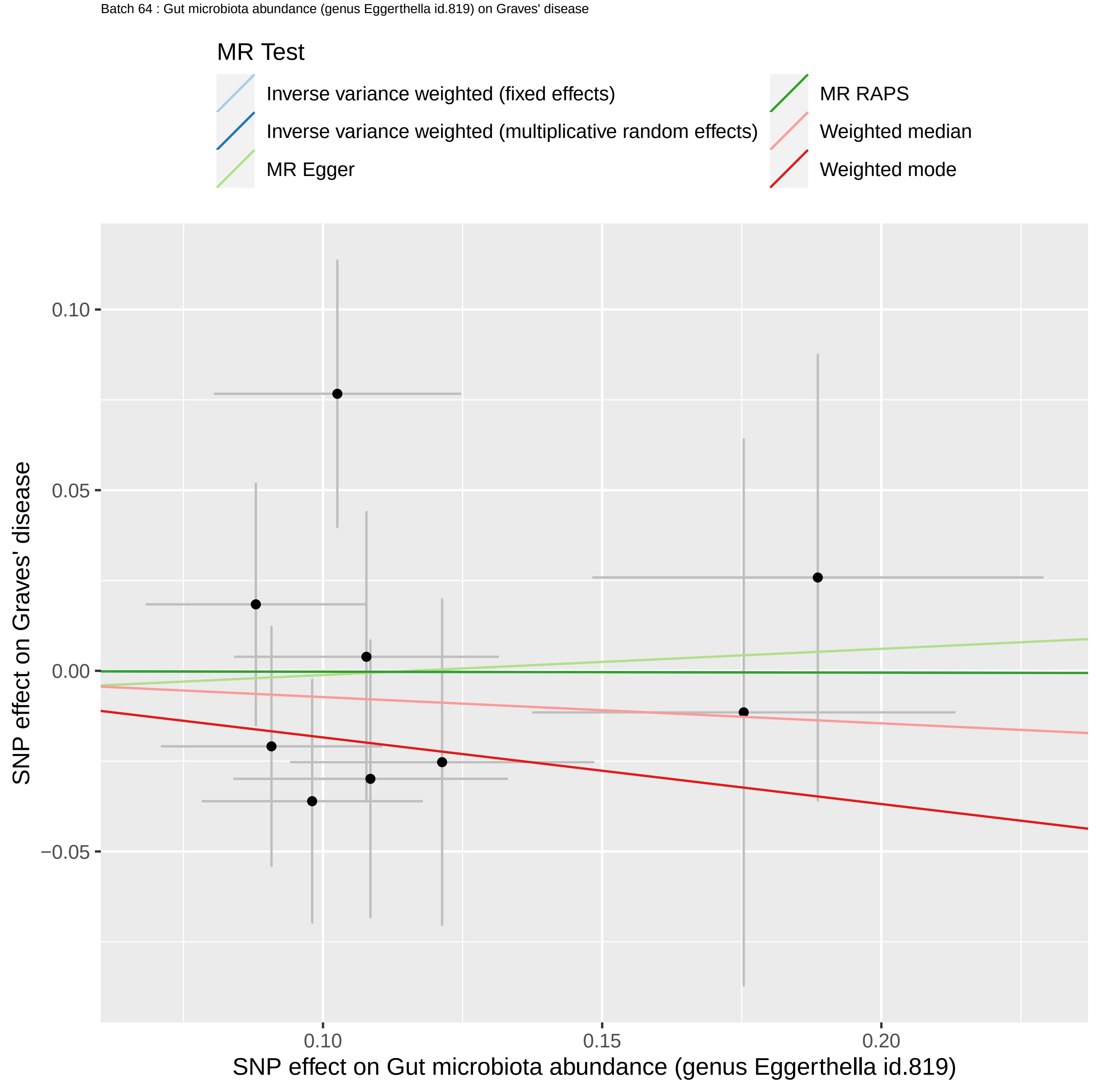
Batch 64 : Gut microbiota abundance (genus Eggerthella id.819) on Graves' disease
MR Test
Inverse variance weighted (fixed effects)
Inverse variance weighted (multiplicative random effects)
MR Egger
MR RAPS
Weighted median
Weighted mode
0.10
0.05
0.00
−0.05
0.10
0.15
0.20
SNP effect on Graves' disease
SNP effect on Gut microbiota abundance (genus Eggerthella id.819)

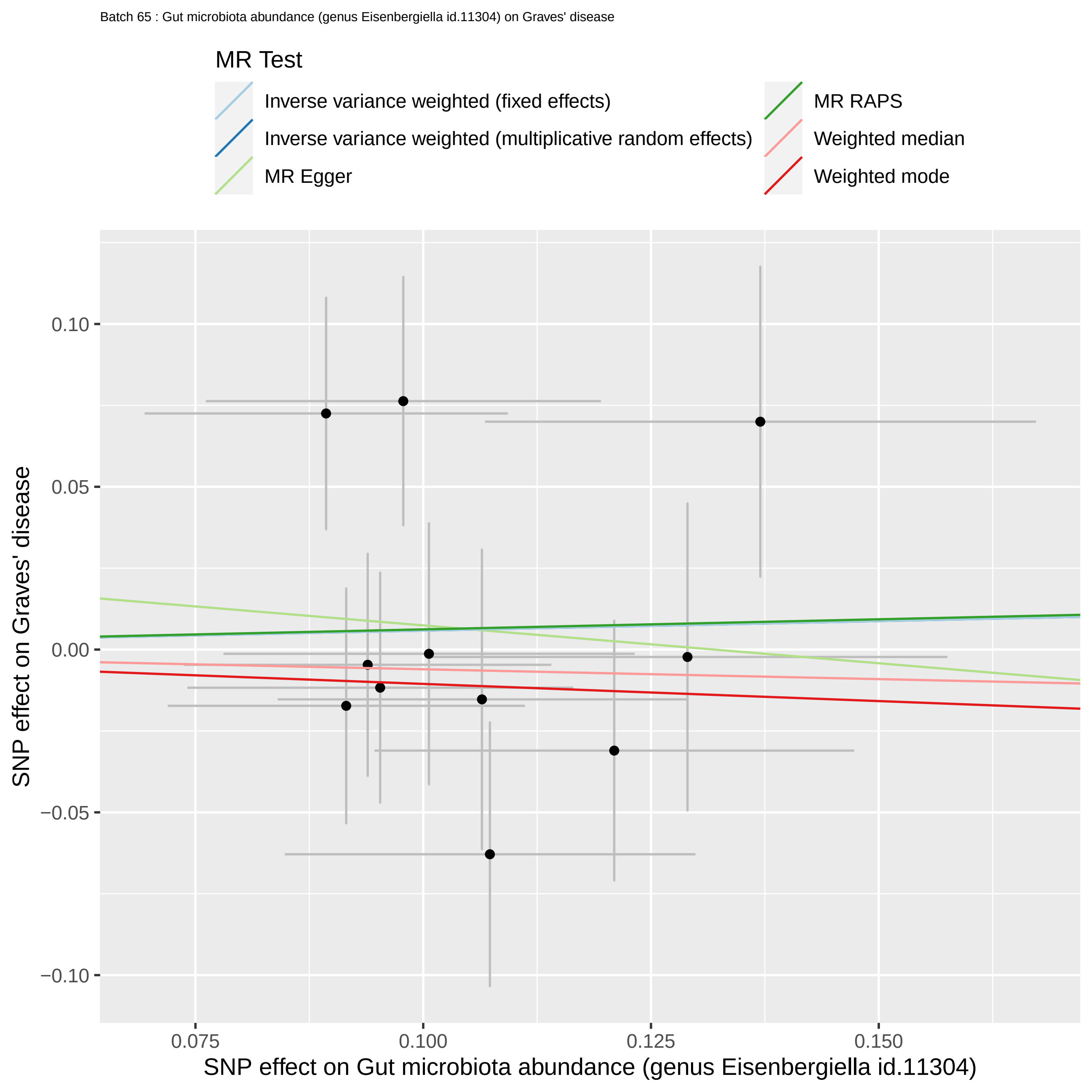

Batch 65 : Gut microbiota abundance (genus Eisenbergiella id.11304) on Graves' disease
MR Test
Inverse variance weighted (fixed effects)
Inverse variance weighted (multiplicative random effects)
MR Egger
MR RAPS
Weighted median
Weighted mode
0.10
0.05
0.00
−0.05
−0.10
SNP effect on Graves' disease
0.075
0.100
0.125
0.150
SNP effect on Gut microbiota abundance (genus Eisenbergiella id.11304)

Batch 66 : Gut microbiota abundance (genus Enterorhabdus id.820) on Graves' disease

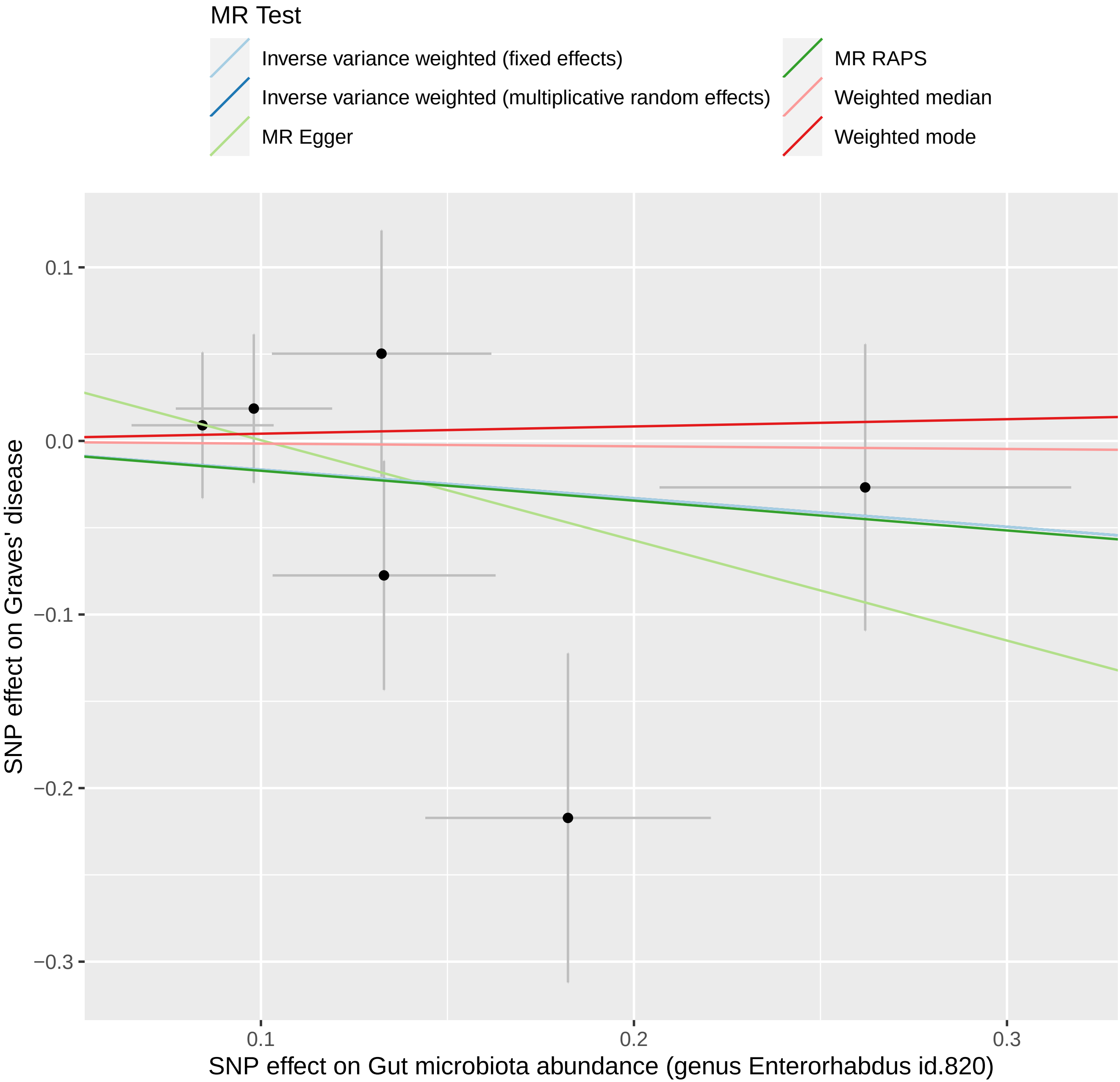

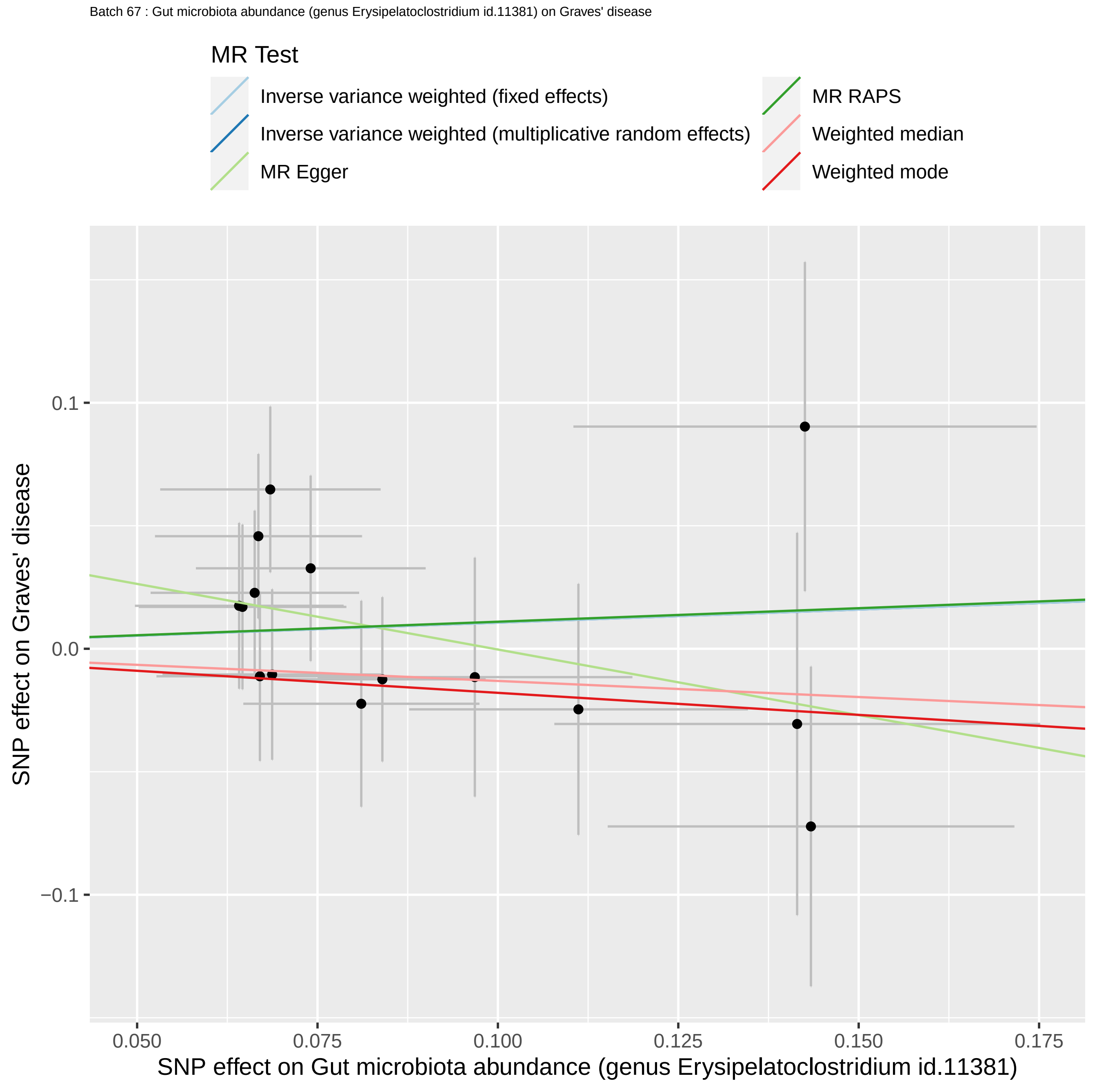

Batch 67 : Gut microbiota abundance (genus Erysipelatoclostridium id.11381) on Graves' disease
MR Test
Inverse variance weighted (fixed effects)
Inverse variance weighted (multiplicative random effects)
MR Egger
MR RAPS
Weighted median
Weighted mode
0.1
0.0
−0.1
0.050
0.075
0.100
0.125
0.150
0.175
SNP effect on Graves' disease
SNP effect on Gut microbiota abundance (genus Erysipelatoclostridium id.11381)

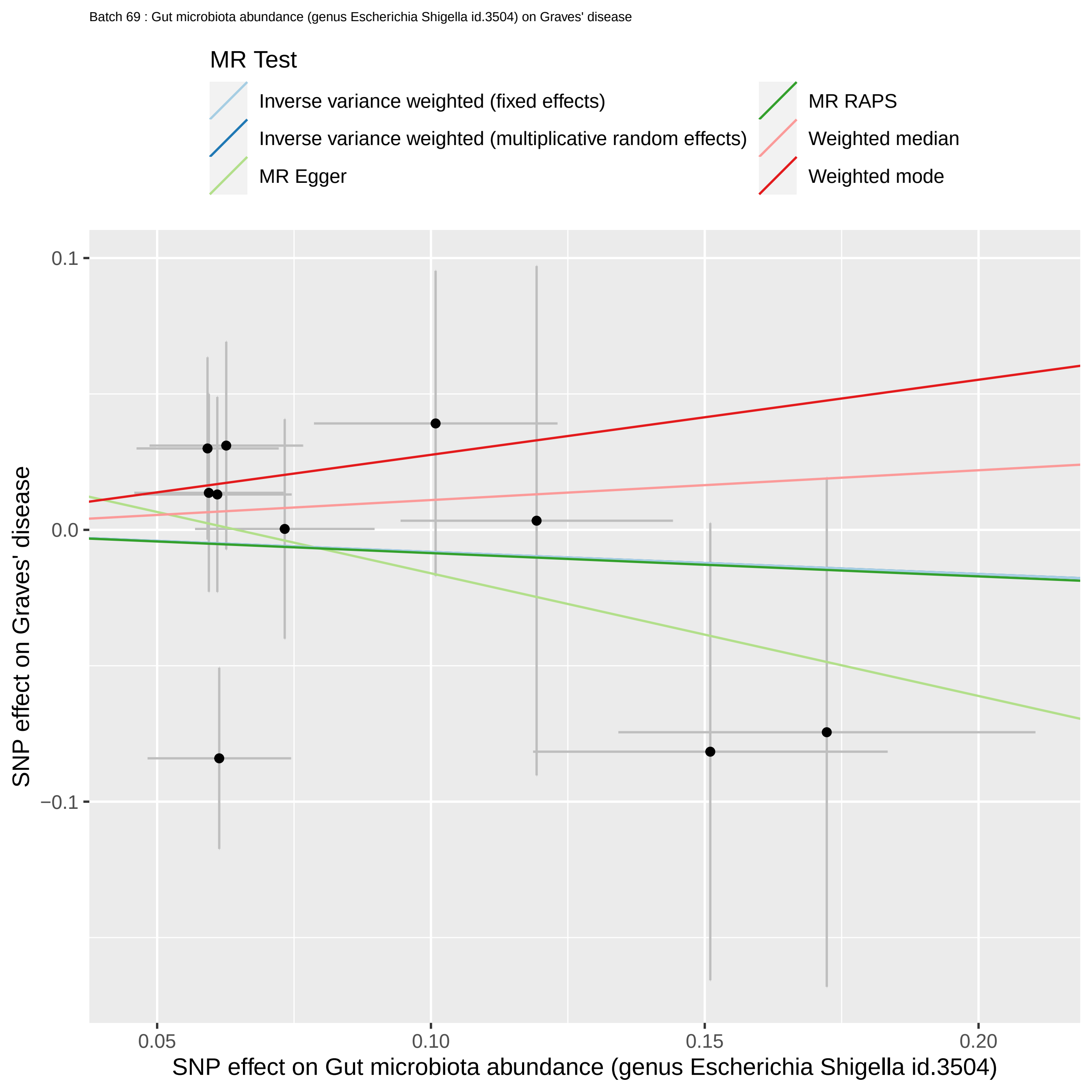
Batch 69 : Gut microbiota abundance (genus Escherichia Shigella id.3504) on Graves' disease
MR Test
Inverse variance weighted (fixed effects)
Inverse variance weighted (multiplicative random effects)
MR Egger
MR RAPS
Weighted median
Weighted mode
0.1
0.0
−0.1
0.05
0.10
0.15
0.20
SNP effect on Graves' disease
SNP effect on Gut microbiota abundance (genus Escherichia Shigella id.3504)

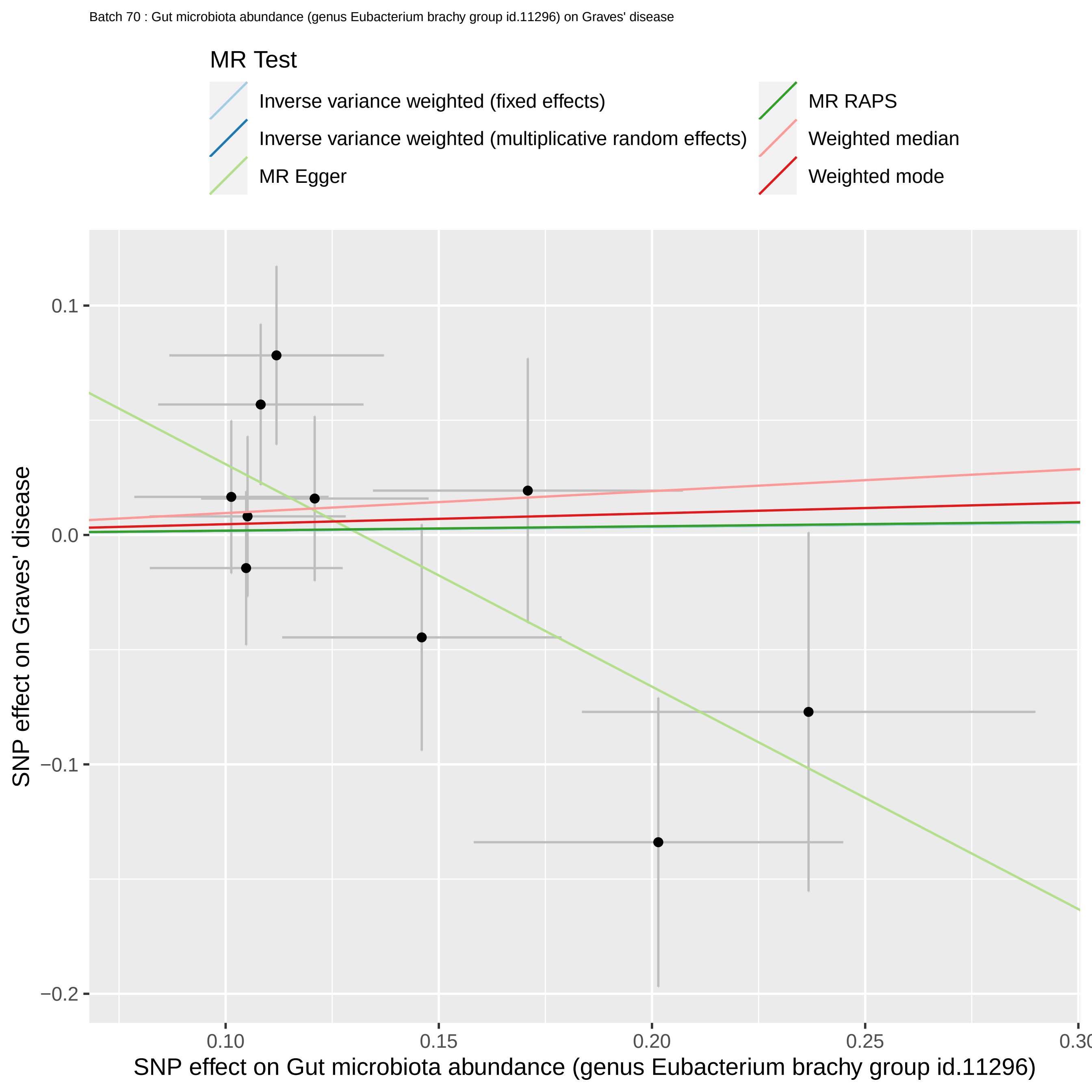
Batch 70 : Gut microbiota abundance (genus Eubacterium brachy group id.11296) on Graves' disease
MR Test
Inverse variance weighted (fixed effects)
Inverse variance weighted (multiplicative random effects)
MR Egger
MR RAPS
Weighted median
Weighted mode
0.1
0.0
−0.1
−0.2
SNP effect on Graves' disease
0.10
0.15
0.20
0.25
0.30
SNP effect on Gut microbiota abundance (genus Eubacterium brachy group id.11296)

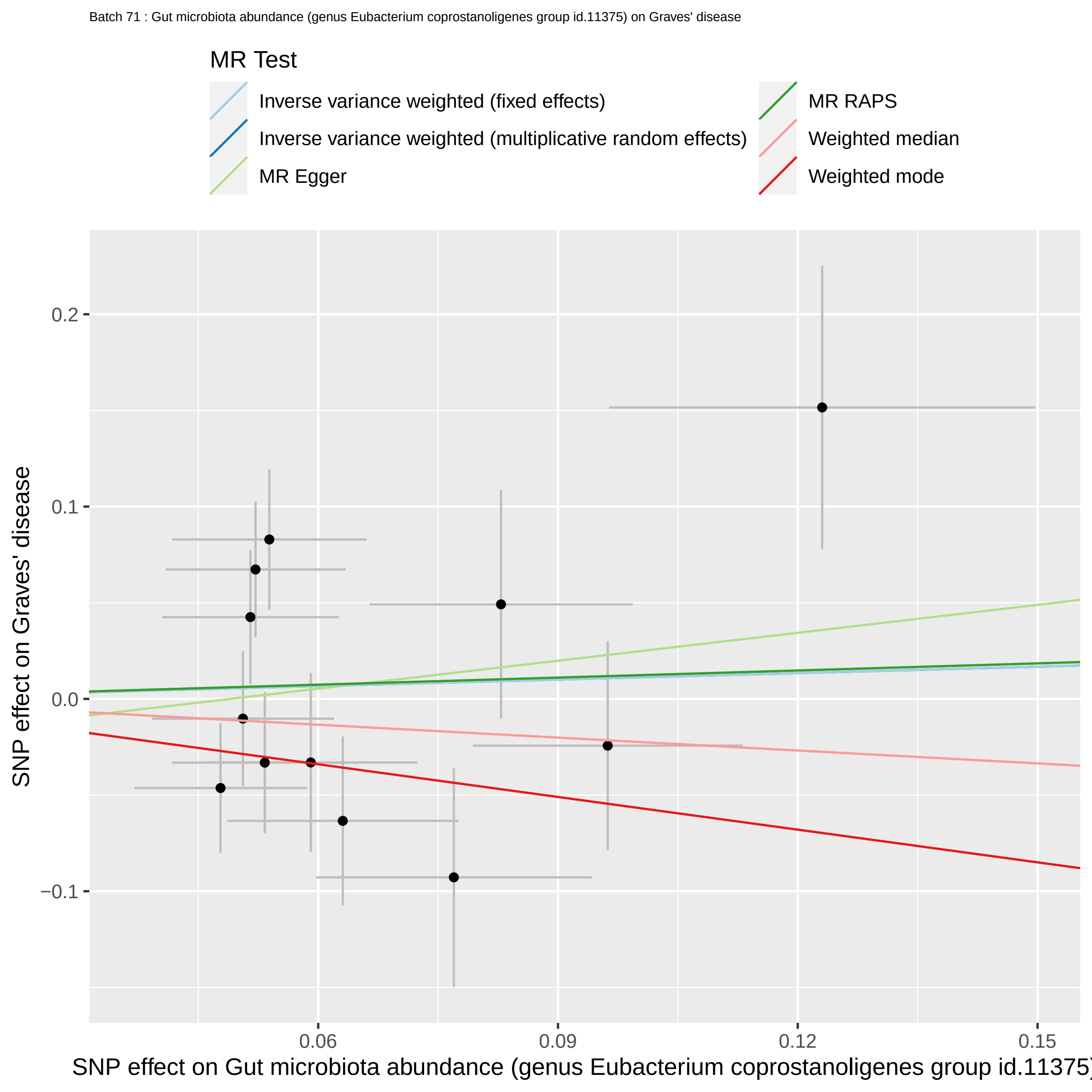
Batch 71 : Gut microbiota abundance (genus Eubacterium coprostanoligenes group id.11375) on Graves' disease
MR Test
Inverse variance weighted (fixed effects)
Inverse variance weighted (multiplicative random effects)
MR Egger
MR RAPS
Weighted median
Weighted mode
0.2
0.1
0.0
−0.1
0.06
0.09
0.12
0.15
SNP effect on Graves' disease
SNP effect on Gut microbiota abundance (genus Eubacterium coprostanoligenes group id.11375)

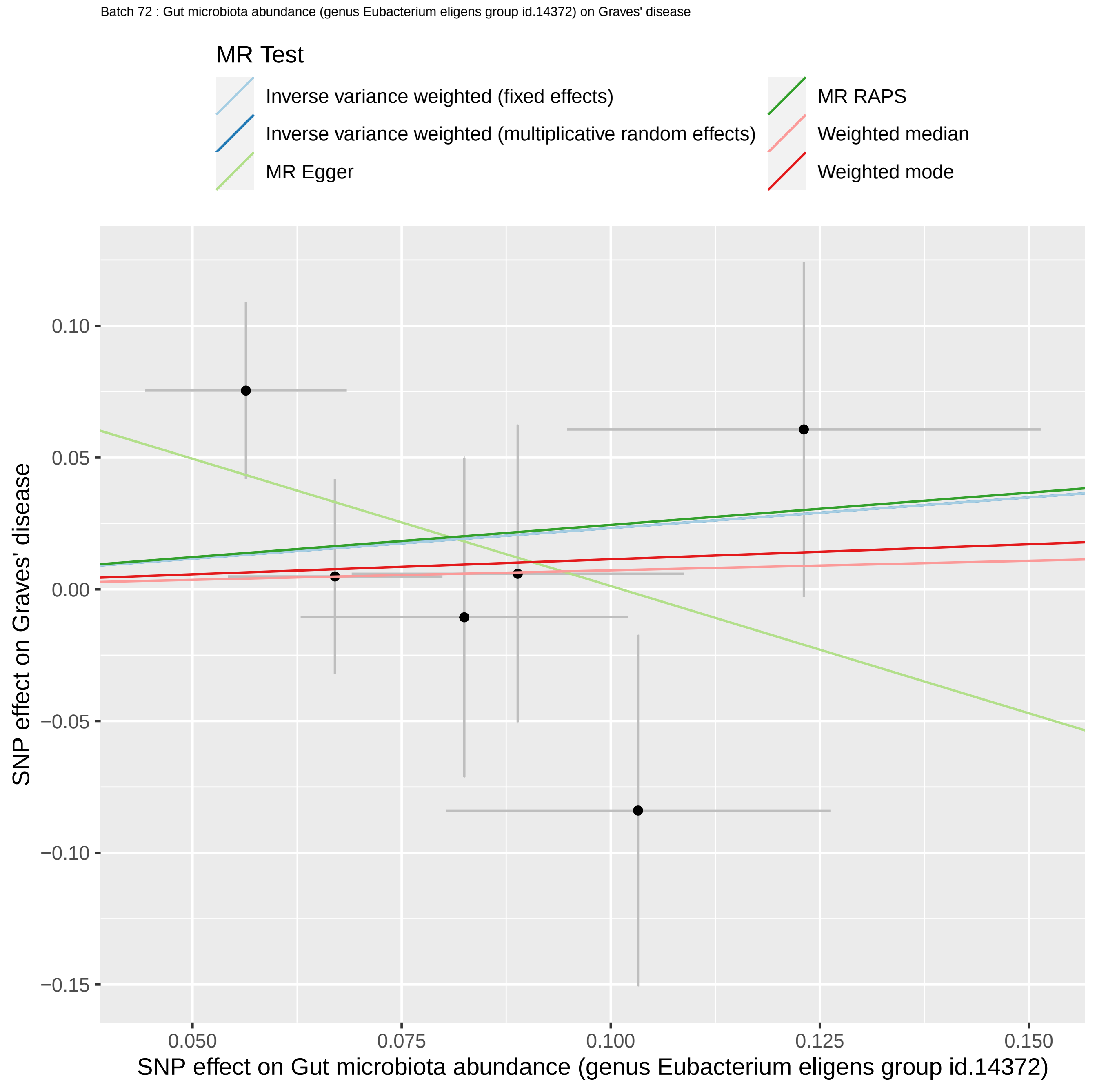

Batch 72 : Gut microbiota abundance (genus Eubacterium eligens group id.14372) on Graves' disease
MR Test
Inverse variance weighted (fixed effects)
Inverse variance weighted (multiplicative random effects)
MR Egger
MR RAPS
Weighted median
Weighted mode
SNP effect on Graves' disease
0.10
0.05
0.00
−0.05
−0.10
−0.15
0.050
0.075
0.100
0.125
0.150
SNP effect on Gut microbiota abundance (genus Eubacterium eligens group id.14372)

Batch 73 : Gut microbiota abundance (genus Eubacterium fissicatena group id.14373) on Graves' disease

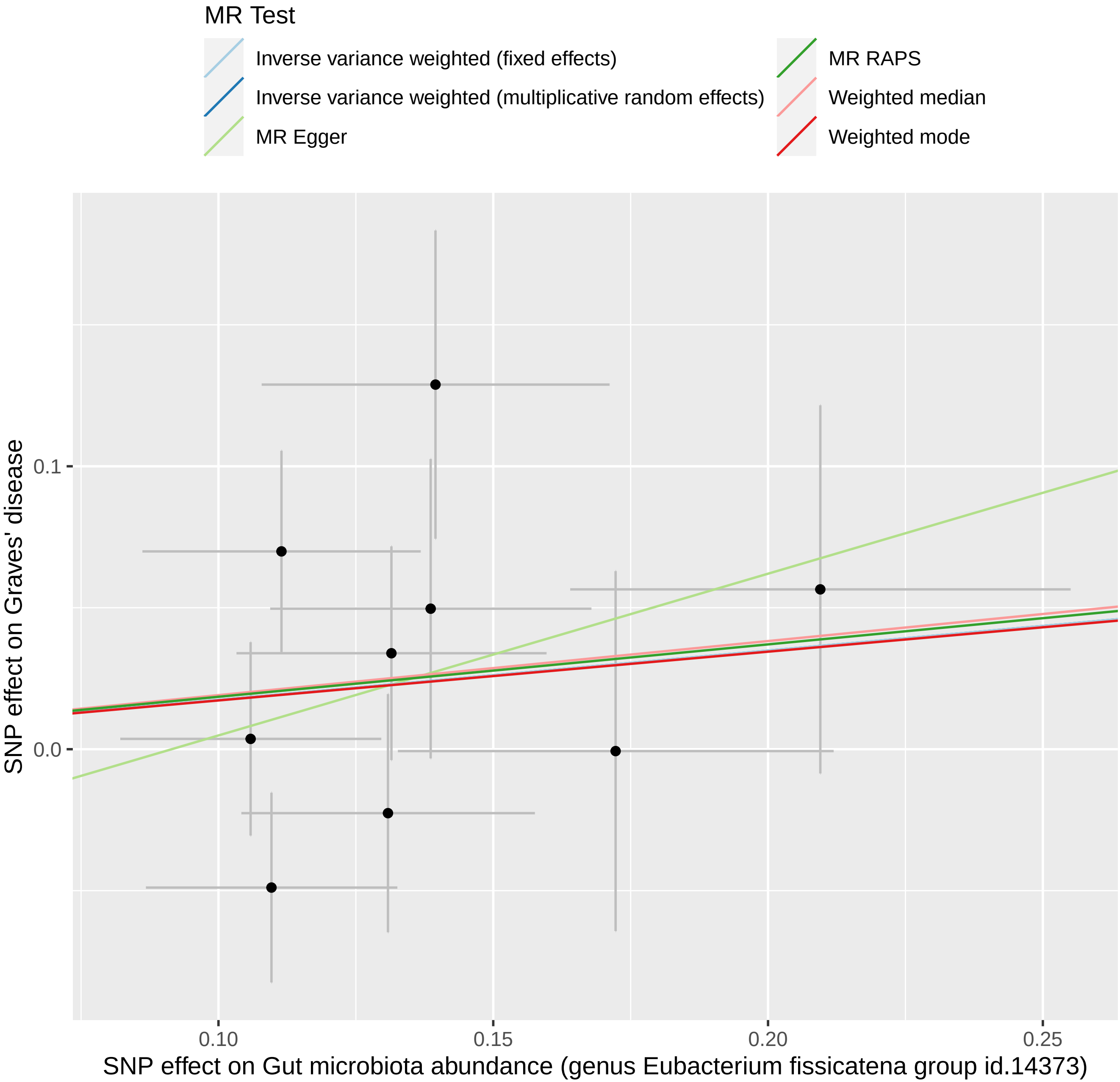

Batch 74 : Gut microbiota abundance (genus Eubacterium hallii group id.11338) on Graves' disease

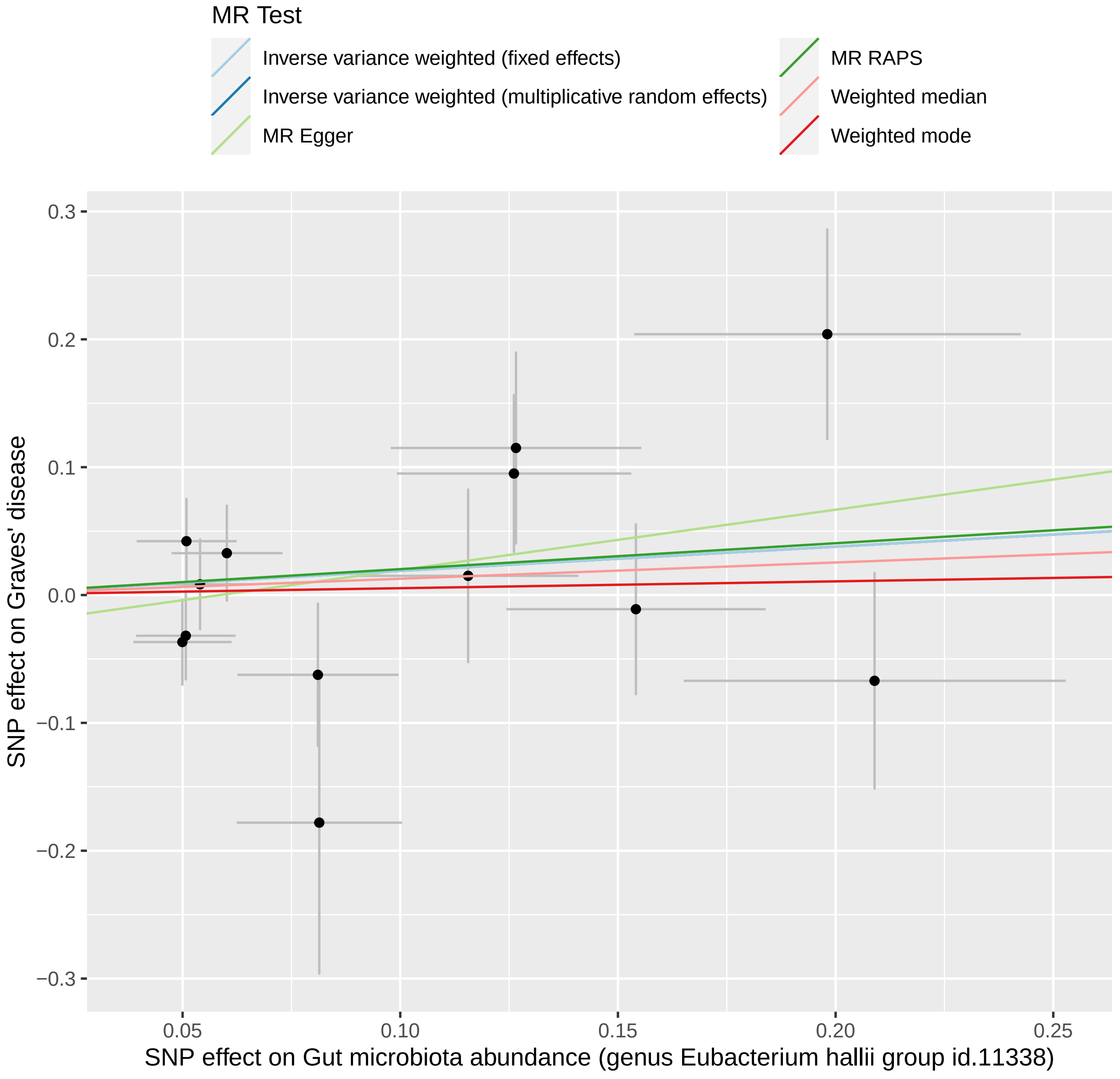

Batch 75 : Gut microbiota abundance (genus Eubacterium nodatum group id.11297) on Graves' disease

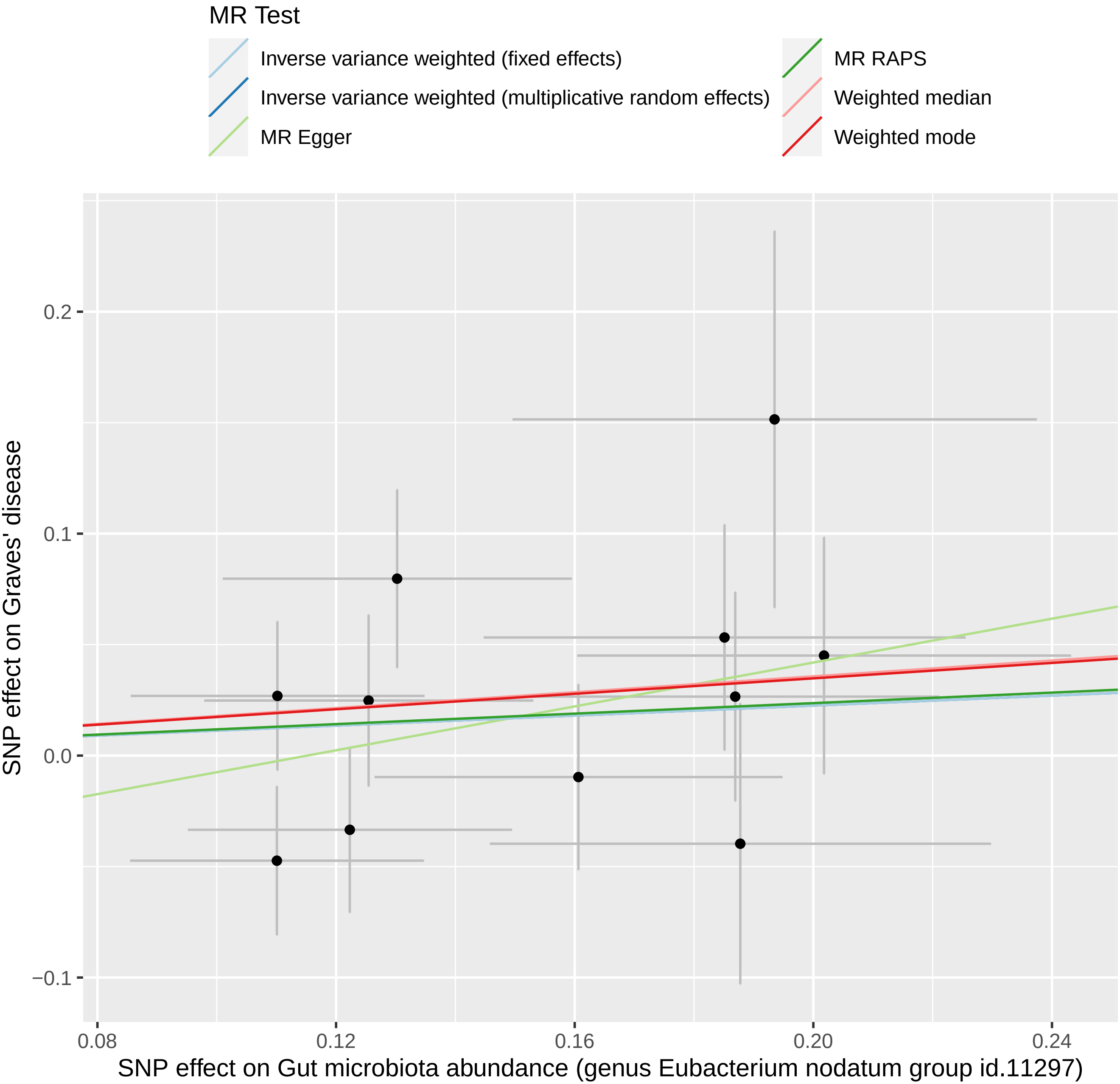

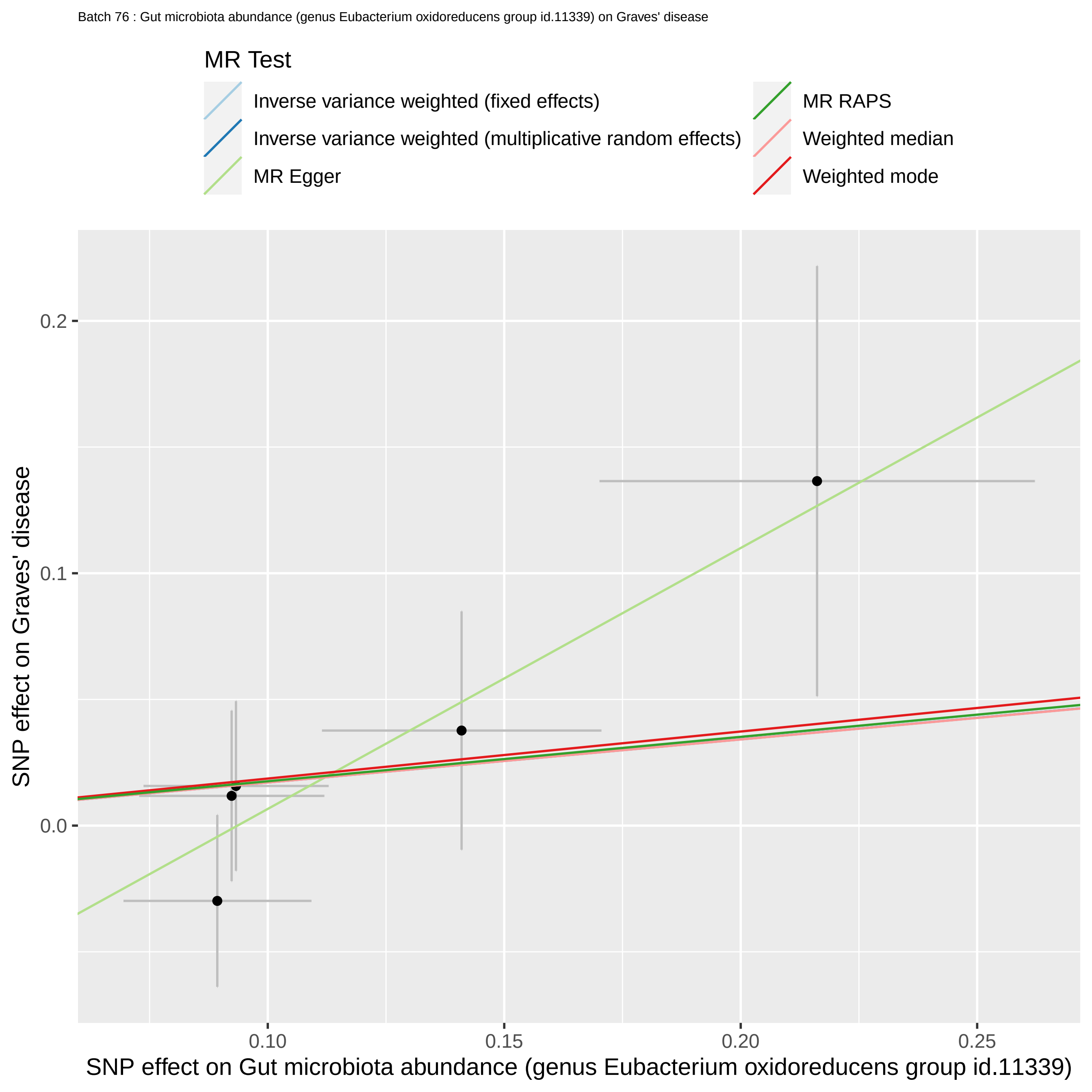

Batch 76 : Gut microbiota abundance (genus Eubacterium oxidoreducens group id.11339) on Graves' disease
MR Test
Inverse variance weighted (fixed effects)
Inverse variance weighted (multiplicative random effects)
MR Egger
MR RAPS
Weighted median
Weighted mode
SNP effect on Graves' disease
0.2
0.1
0.0
0.10
0.15
0.20
0.25
SNP effect on Gut microbiota abundance (genus Eubacterium oxidoreducens group id.11339)

Batch 77 : Gut microbiota abundance (genus Eubacterium rectale group id.14374) on Graves' disease

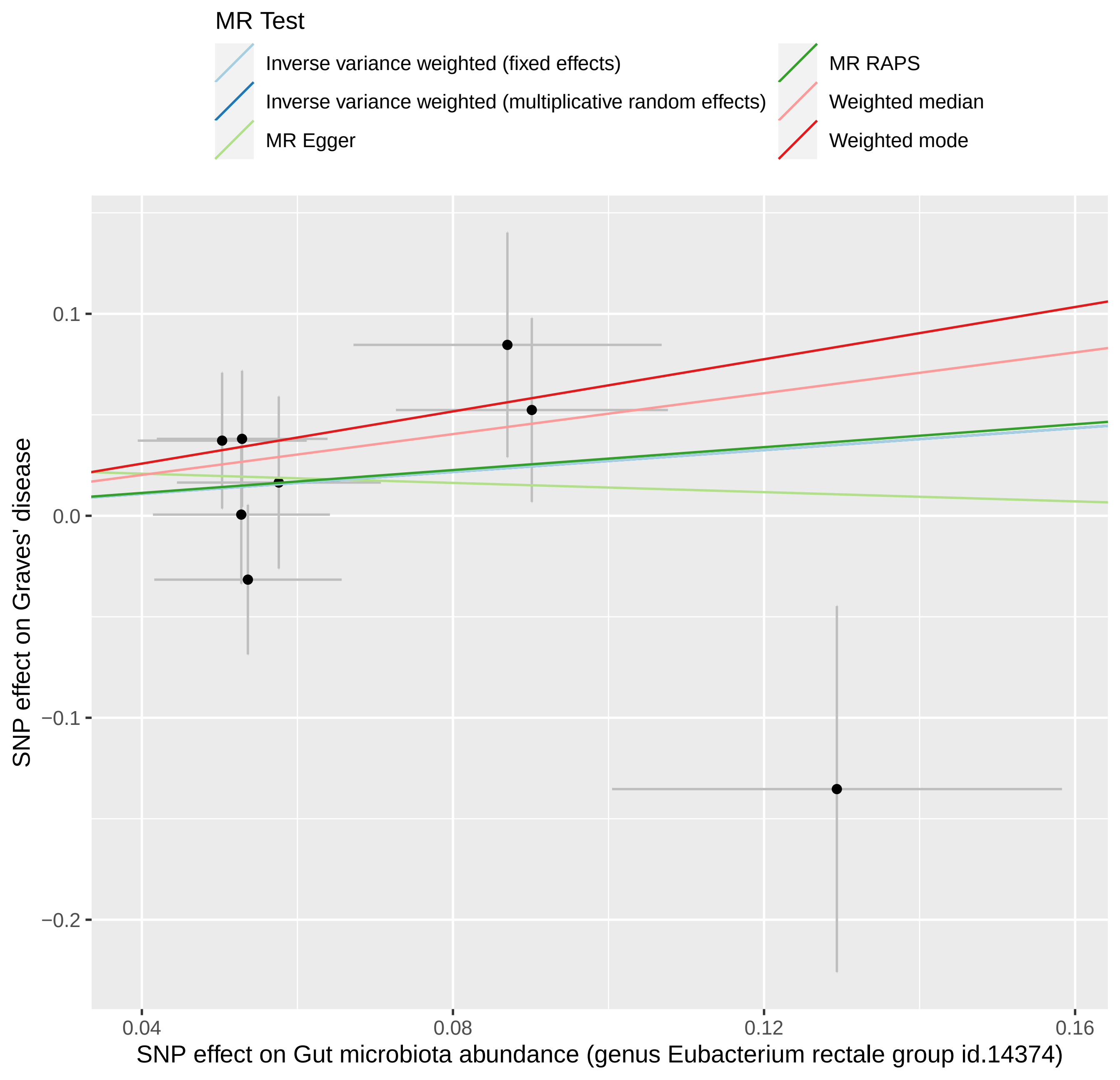

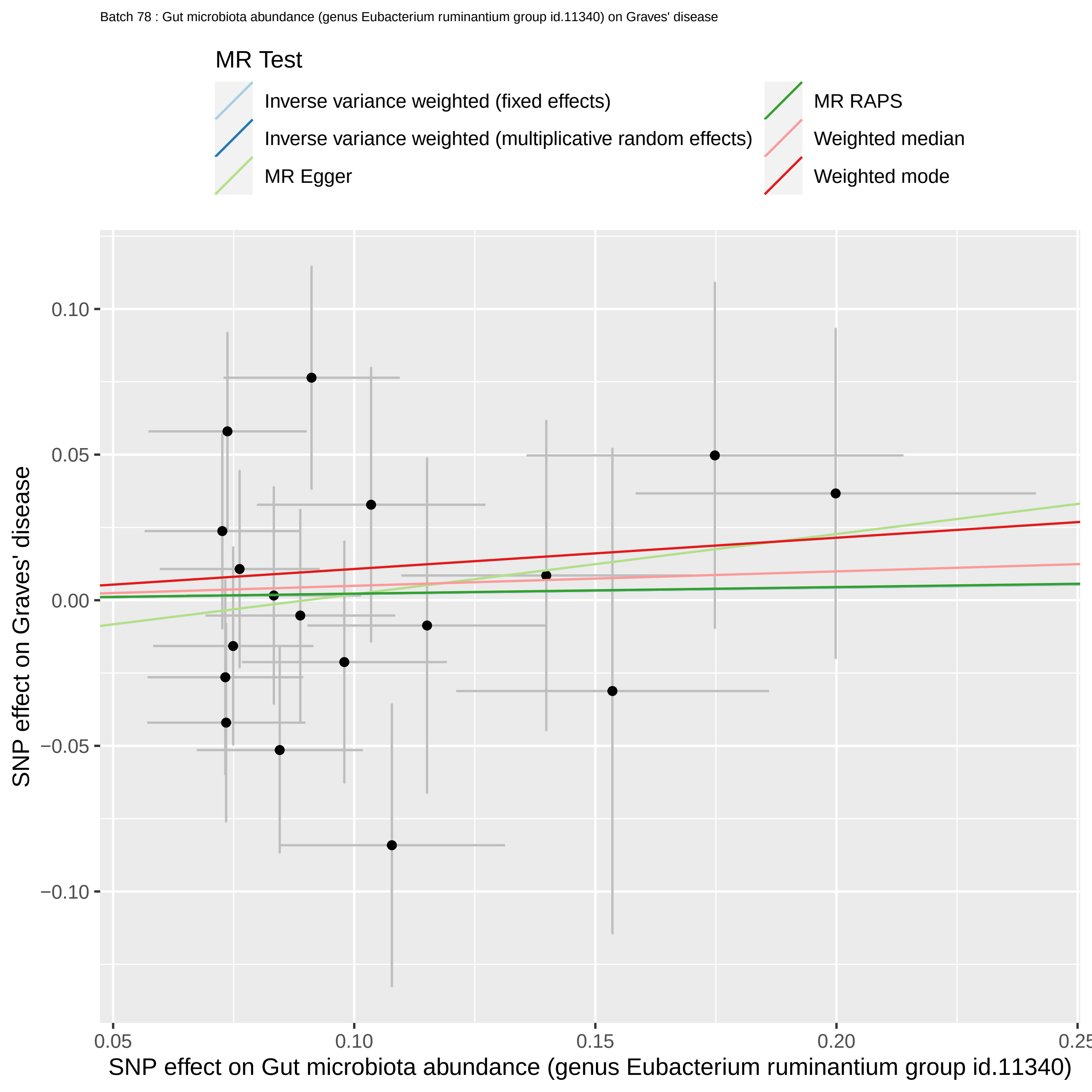
Batch 78 : Gut microbiota abundance (genus Eubacterium ruminantium group id.11340) on Graves' disease
MR Test
Inverse variance weighted (fixed effects)
Inverse variance weighted (multiplicative random effects)
MR Egger
MR RAPS
Weighted median
Weighted mode
0.10
0.05
0.00
−0.05
−0.10
SNP effect on Graves' disease
0.05
0.10
0.15
0.20
0.25
SNP effect on Gut microbiota abundance (genus Eubacterium ruminantium group id.11340)

Batch 79 : Gut microbiota abundance (genus Eubacterium ventriosum group id.11341) on Graves' disease

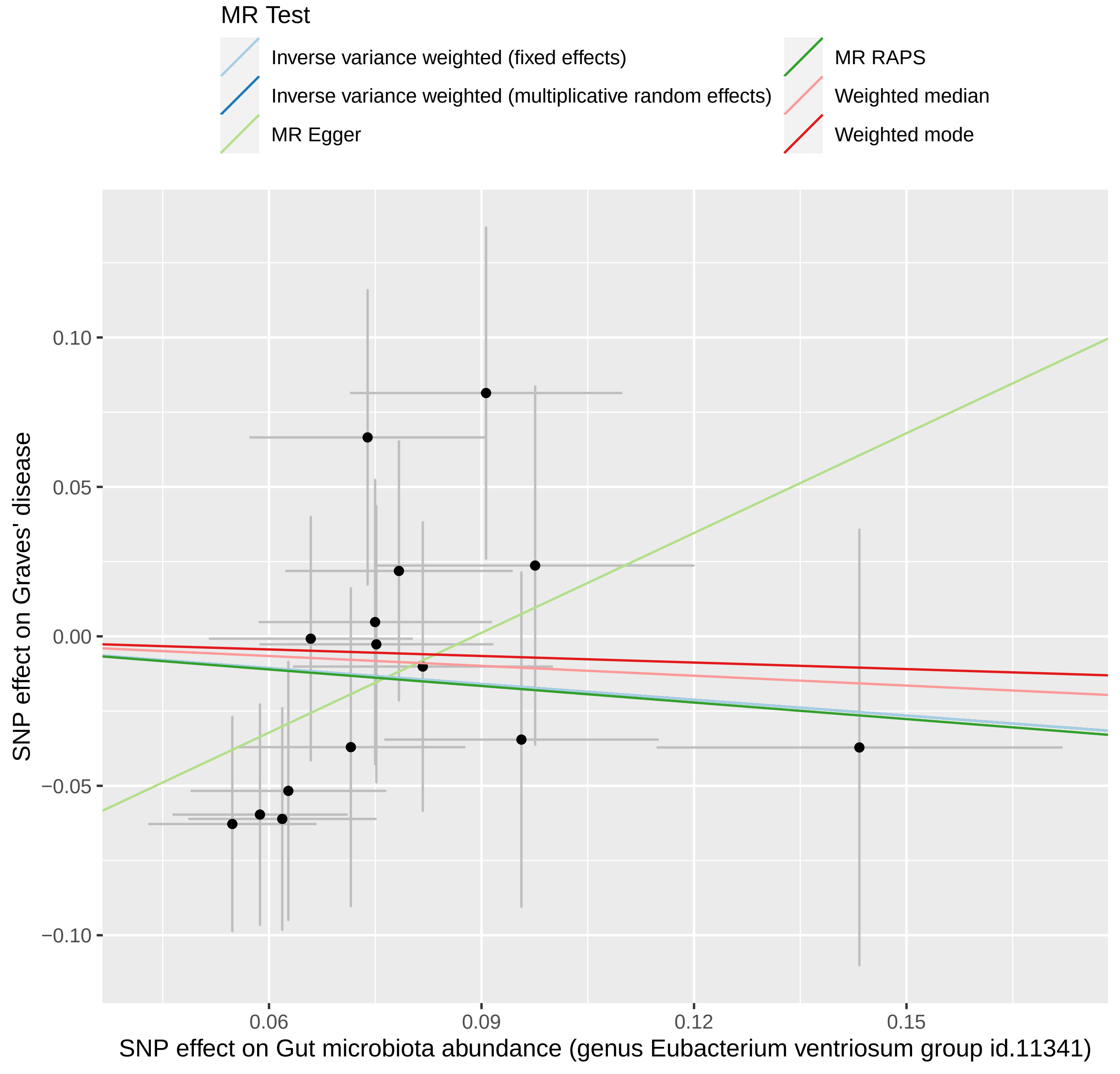

Batch 80 : Gut microbiota abundance (genus Eubacterium xylanophilum group id.14375) on Graves' disease

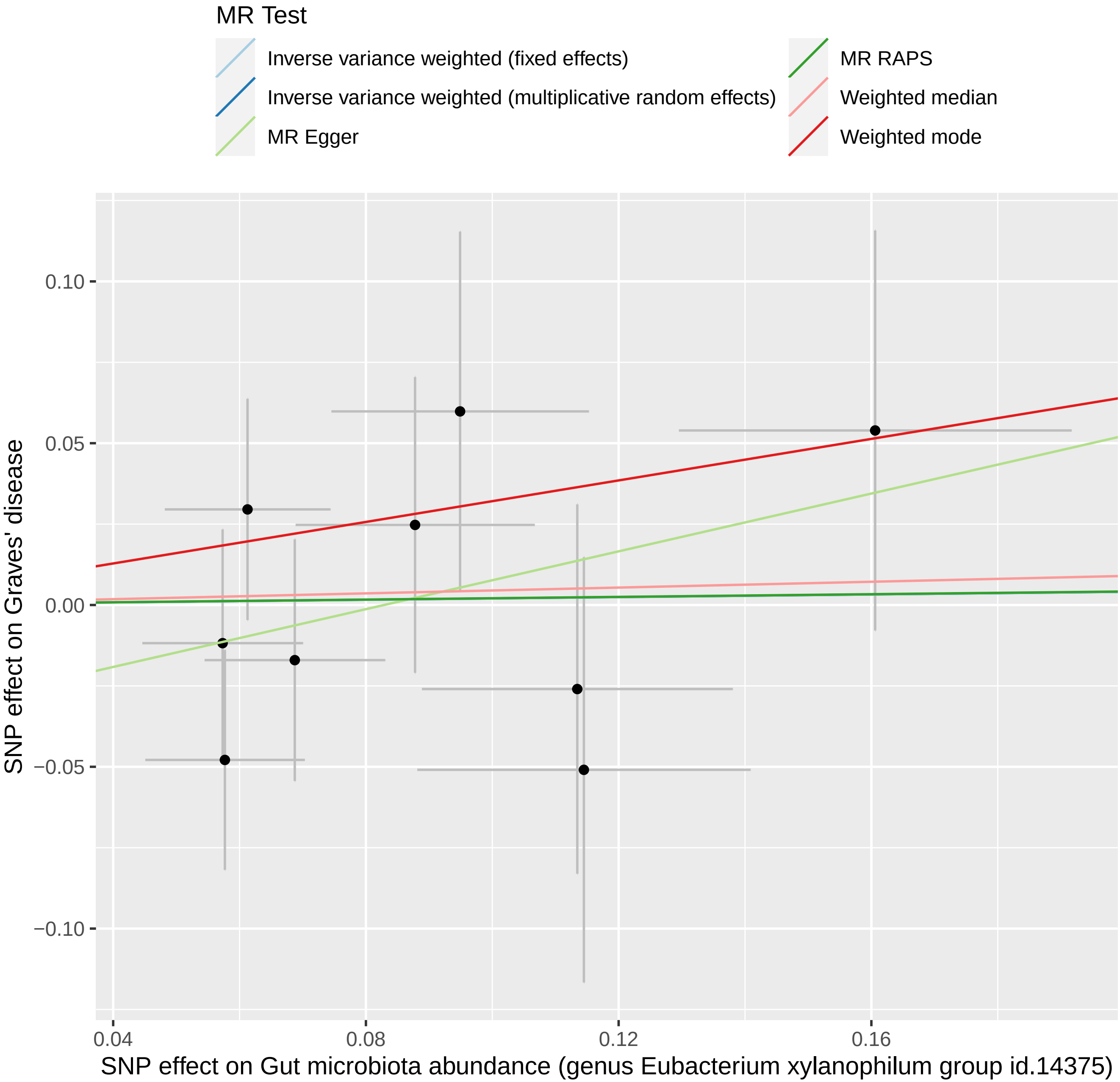

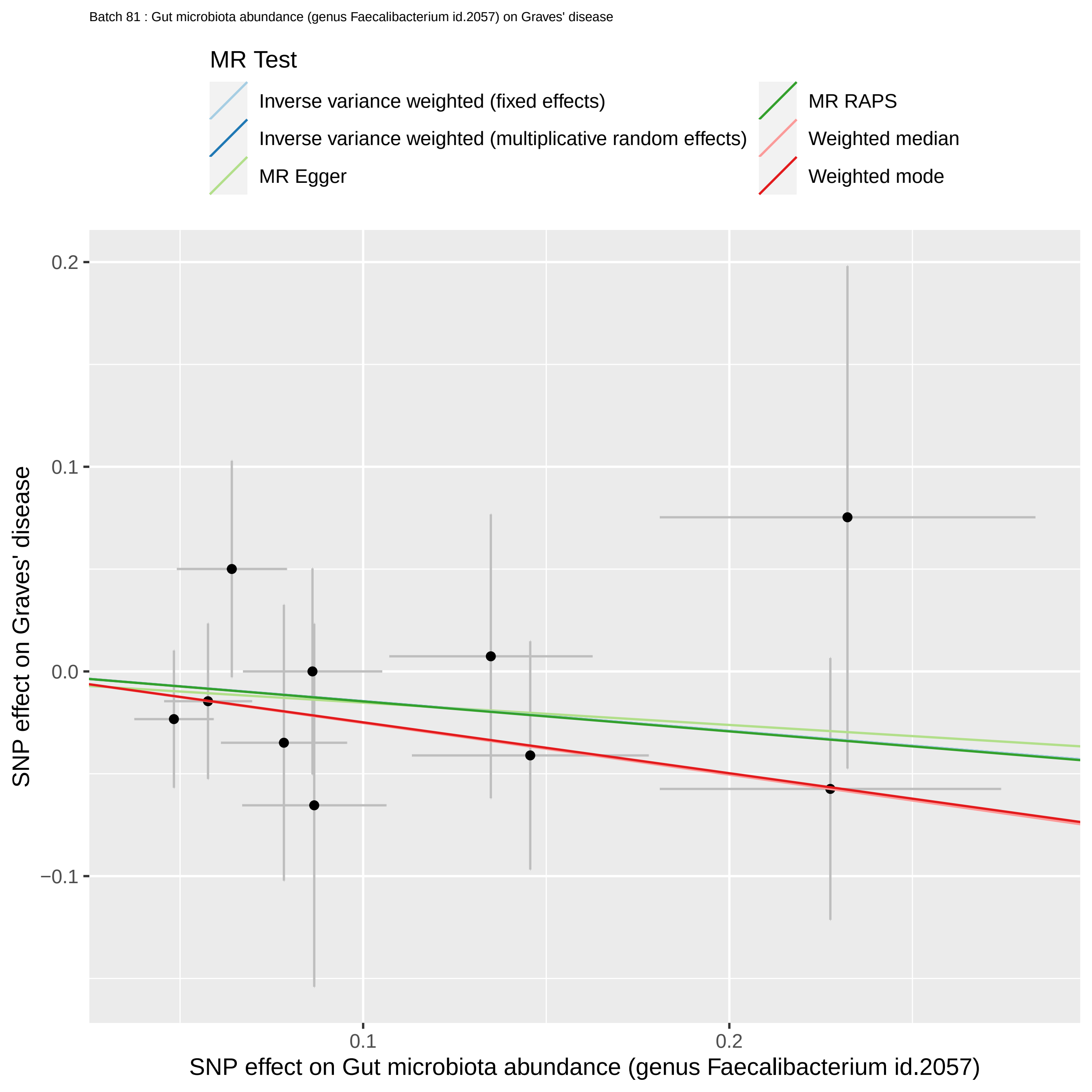

Batch 81 : Gut microbiota abundance (genus Faecalibacterium id.2057) on Graves' disease
MR Test
Inverse variance weighted (fixed effects)
Inverse variance weighted (multiplicative random effects)
MR Egger
MR RAPS
Weighted median
Weighted mode
0.2
0.1
0.0
−0.1
SNP effect on Graves' disease
0.1
0.2
SNP effect on Gut microbiota abundance (genus Faecalibacterium id.2057)

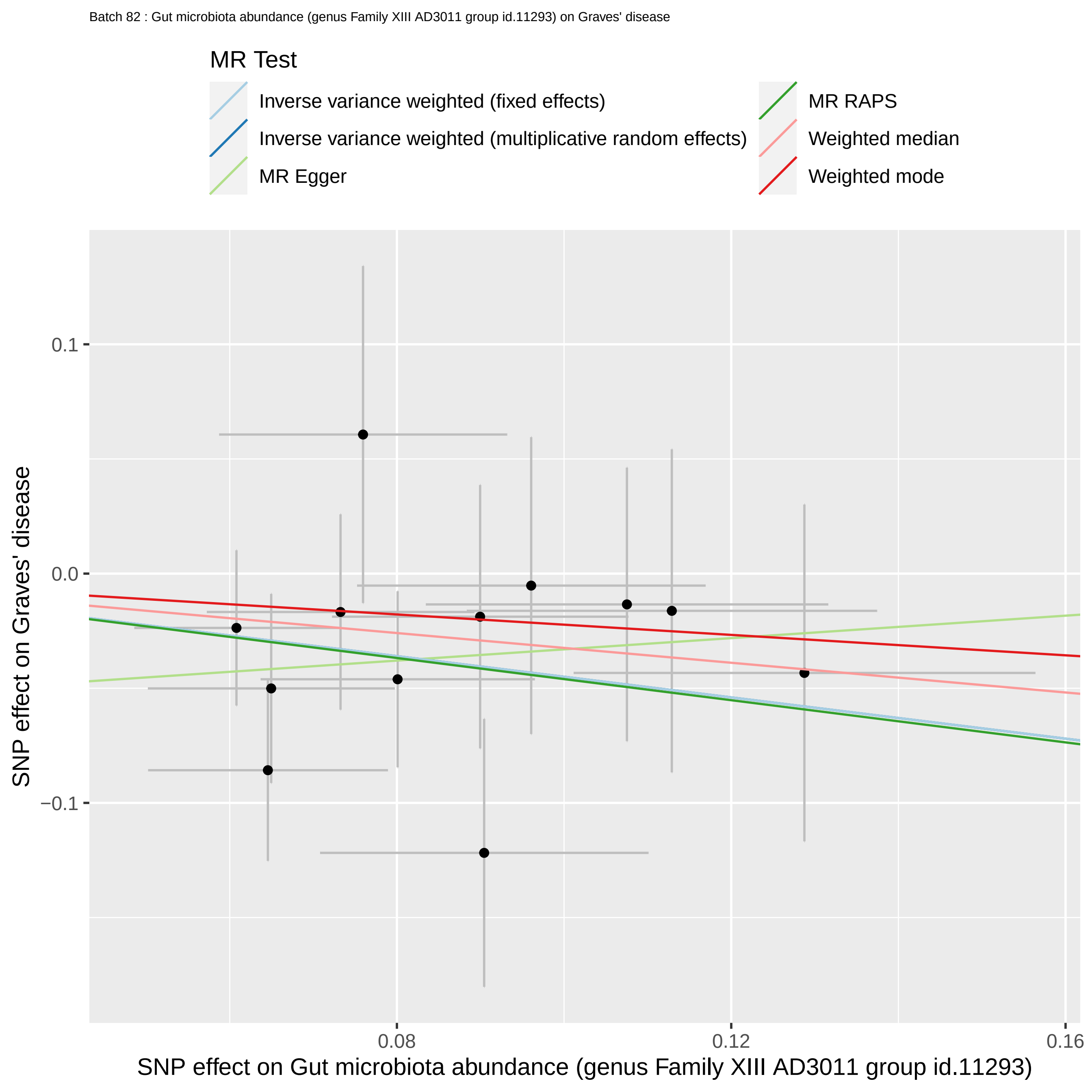

Batch 82 : Gut microbiota abundance (genus Family XIII AD3011 group id.11293) on Graves' disease
MR Test
Inverse variance weighted (fixed effects)
Inverse variance weighted (multiplicative random effects)
MR Egger
MR RAPS
Weighted median
Weighted mode
0.1
0.0
−0.1
0.08
0.12
0.16
SNP effect on Graves' disease
SNP effect on Gut microbiota abundance (genus Family XIII AD3011 group id.11293)

Batch 83 : Gut microbiota abundance (genus Family XIII UCG001 id.11294) on Graves' disease

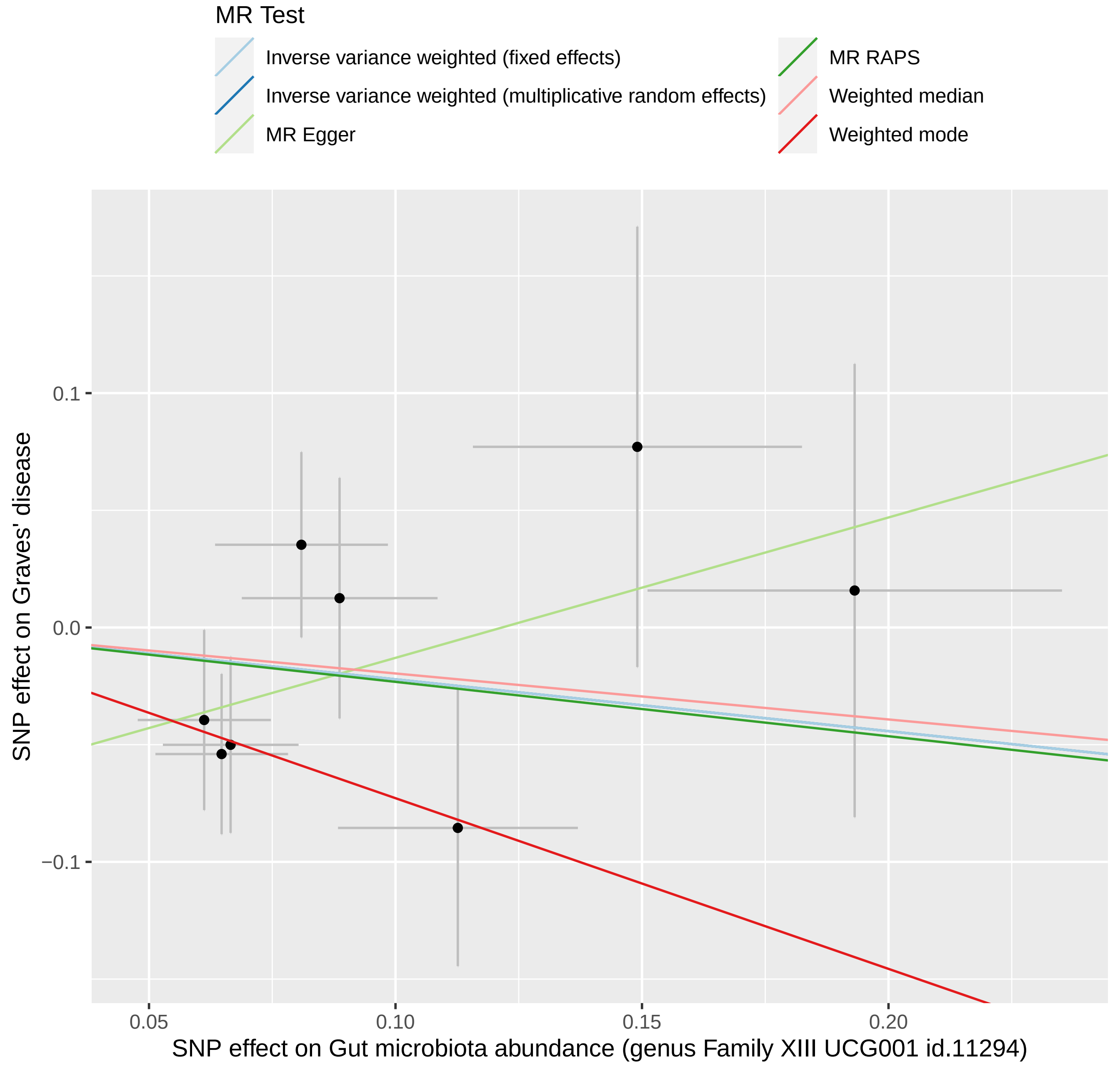

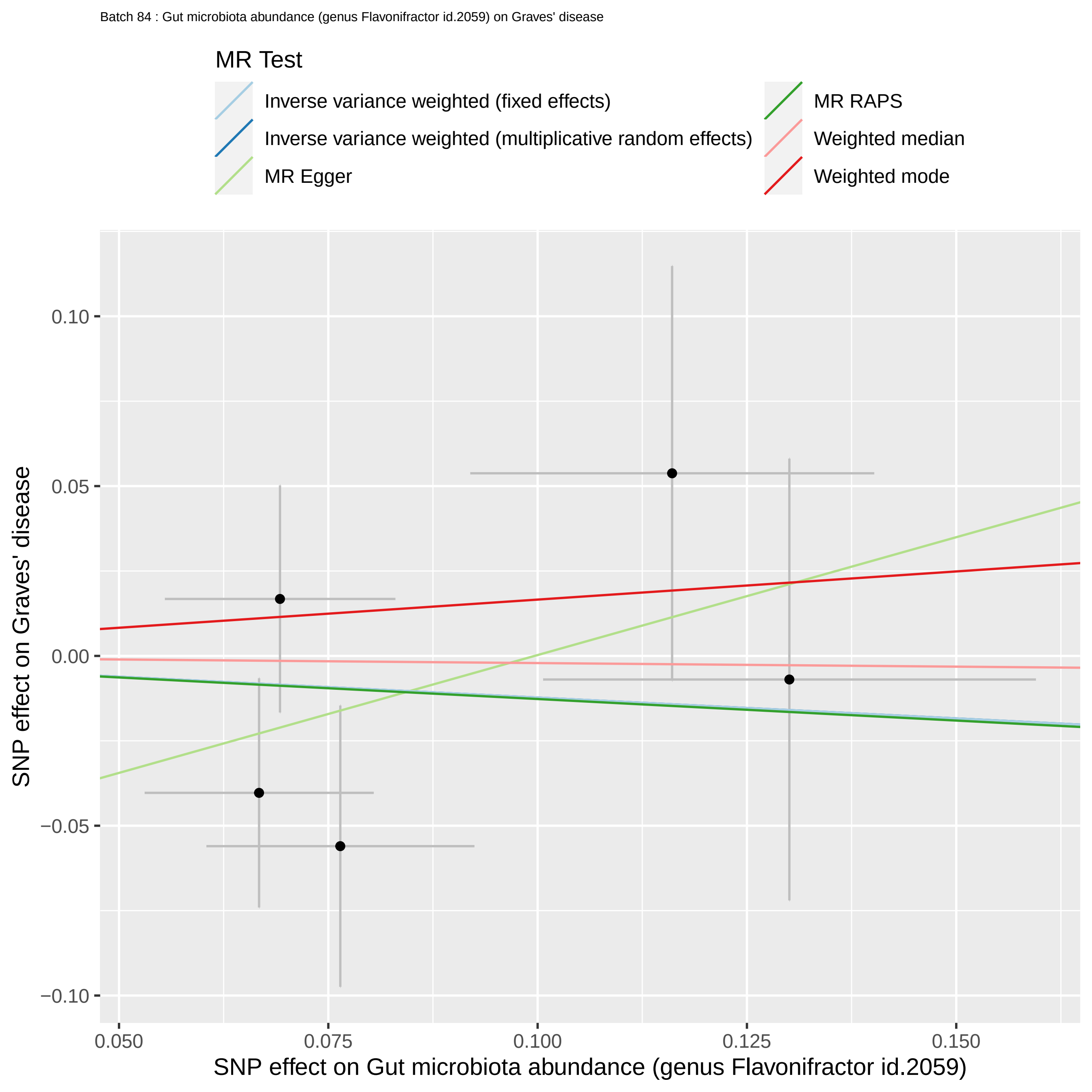

Batch 84 : Gut microbiota abundance (genus Flavonifractor id.2059) on Graves' disease
MR Test
Inverse variance weighted (fixed effects)
Inverse variance weighted (multiplicative random effects)
MR Egger
MR RAPS
Weighted median
Weighted mode
0.10
0.05
0.00
−0.05
−0.10
0.050
0.075
0.100
0.125
0.150
SNP effect on Graves' disease
SNP effect on Gut microbiota abundance (genus Flavonifractor id.2059)

Batch 85 : Gut microbiota abundance (genus Fusicatenibacter id.11305) on Graves' disease

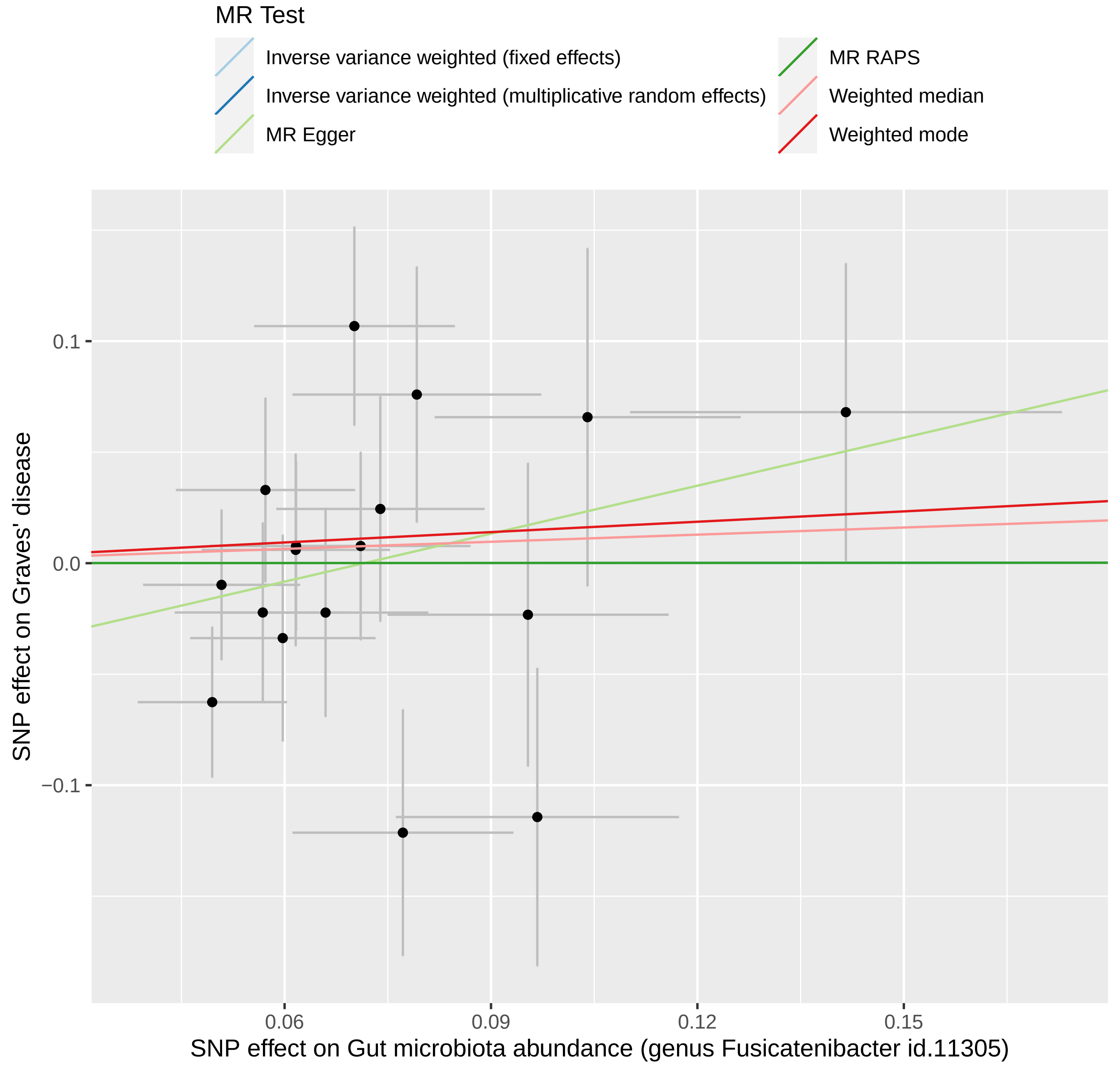

Batch 86 : Gut microbiota abundance (genus Gordonibacter id.821) on Graves' disease

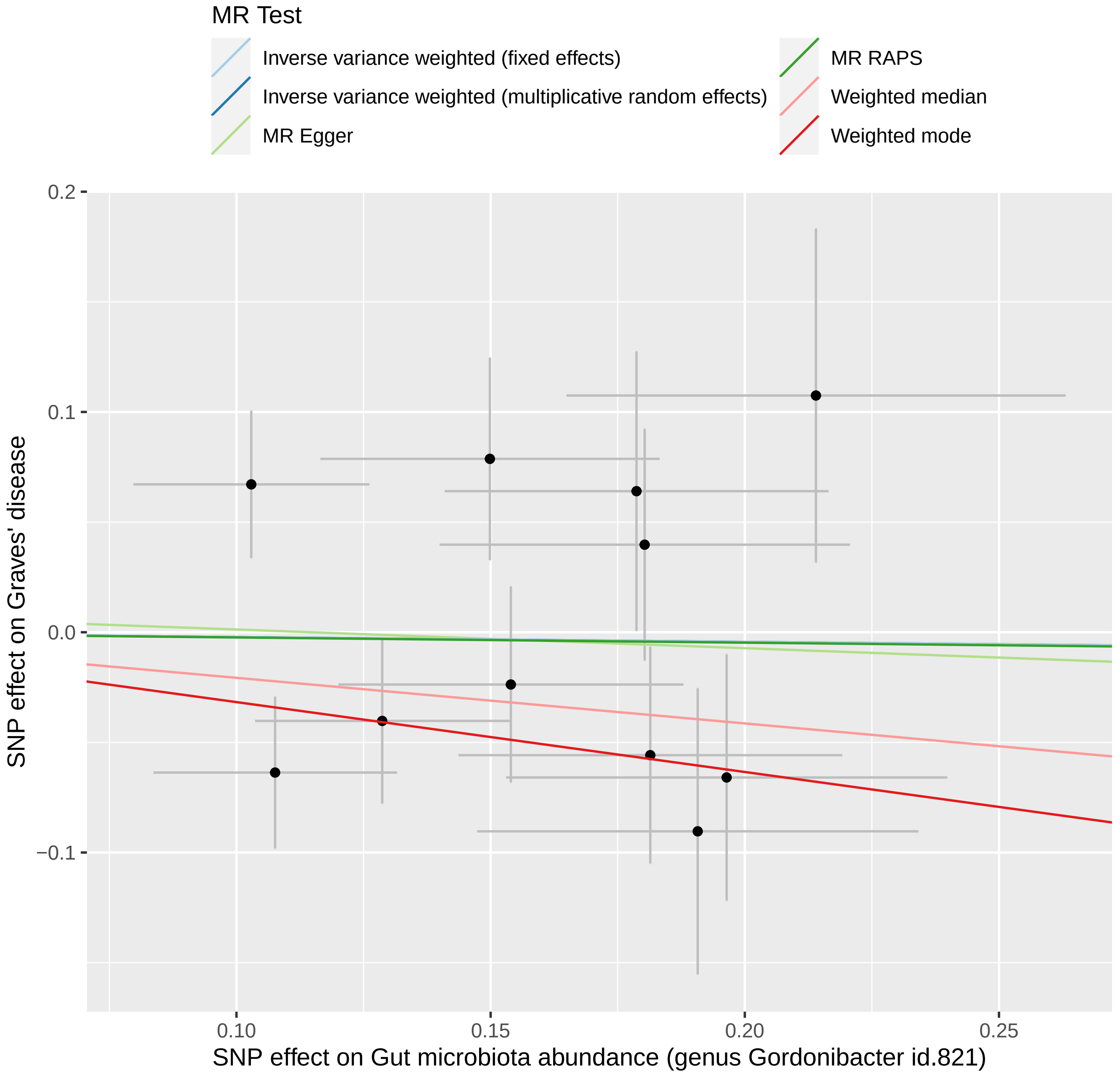

Batch 87 : Gut microbiota abundance (genus Haemophilus id.3698) on Graves' disease

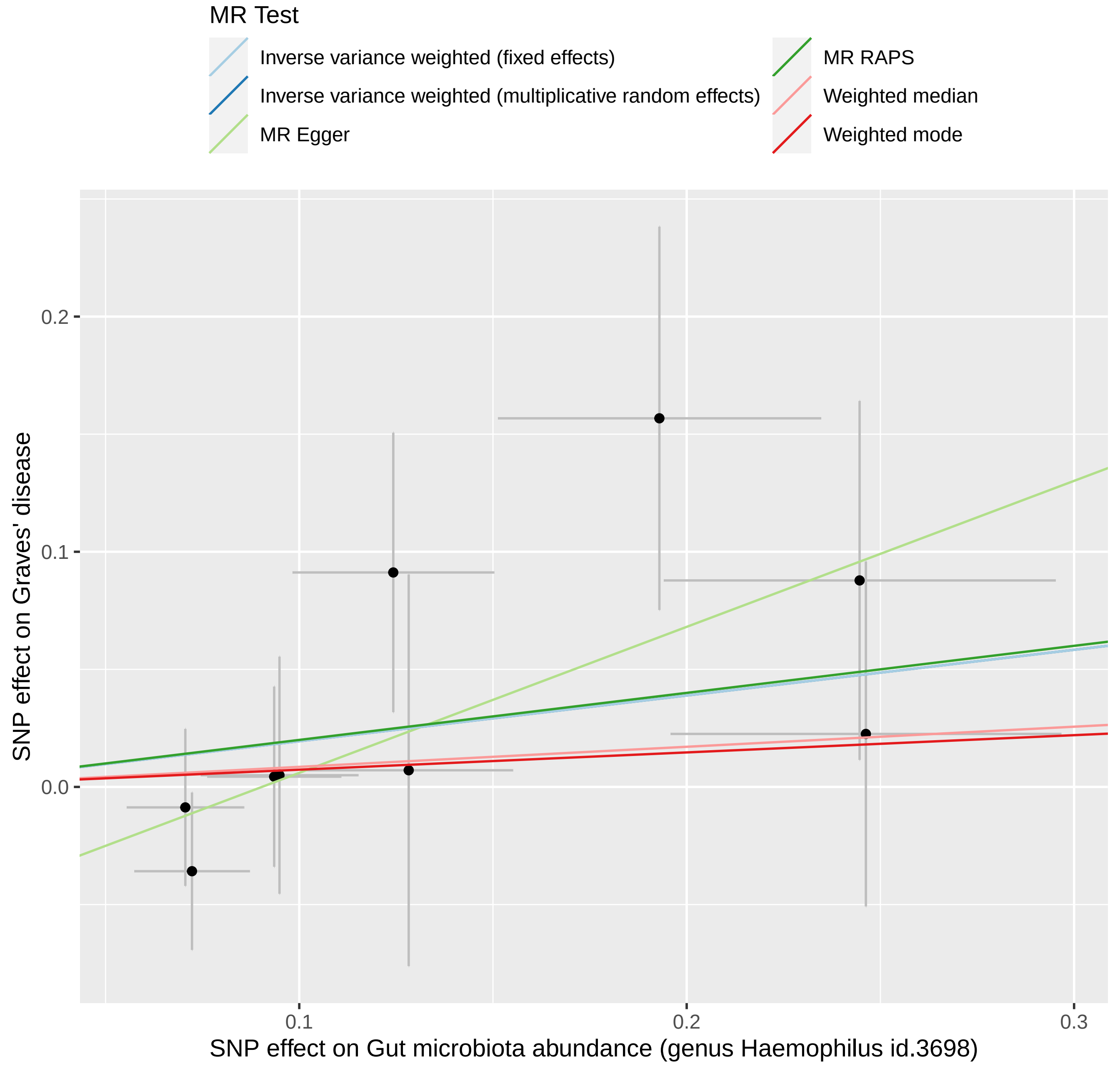

Batch 88 : Gut microbiota abundance (genus Holdemanella id.11393) on Graves' disease

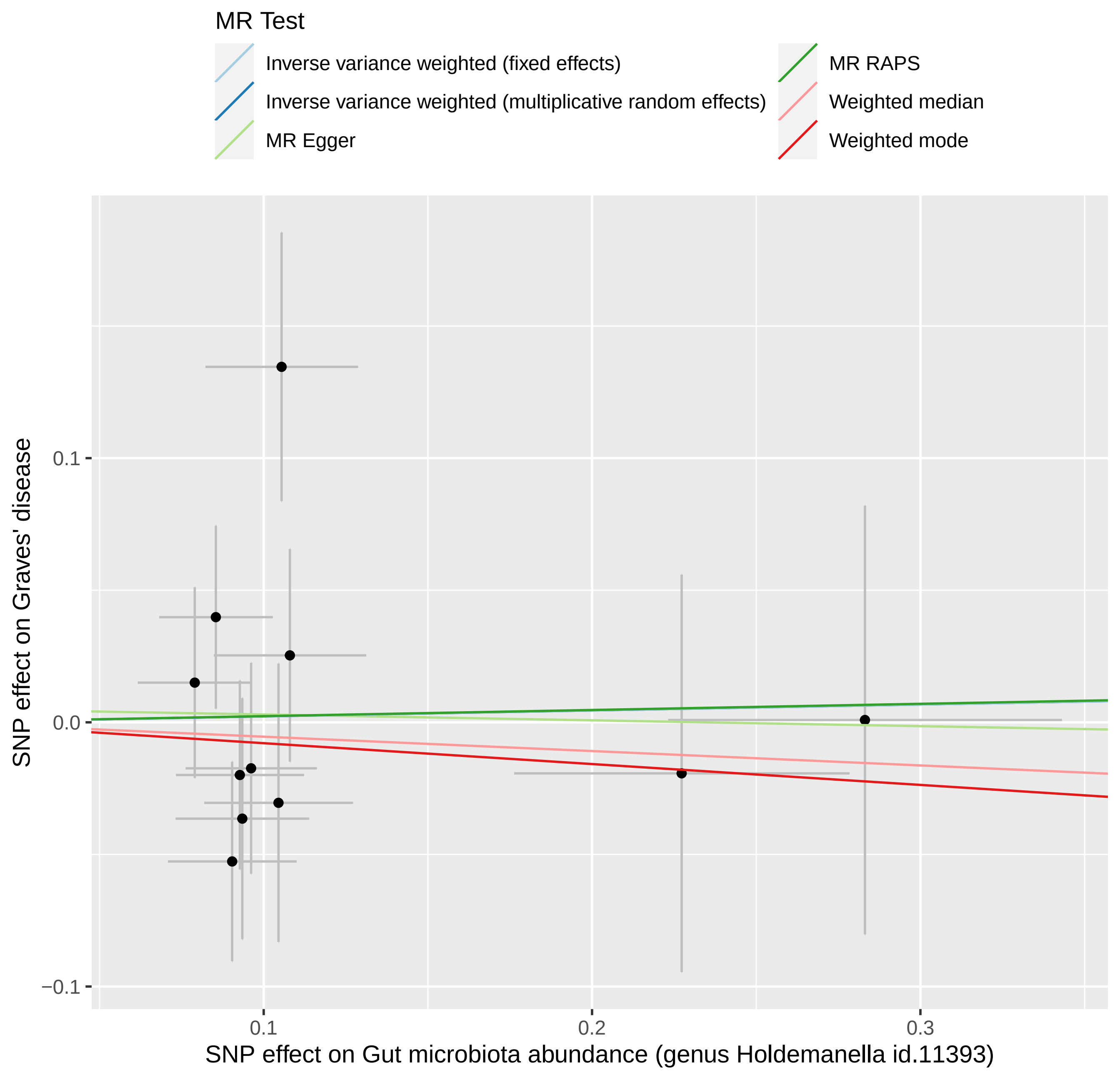

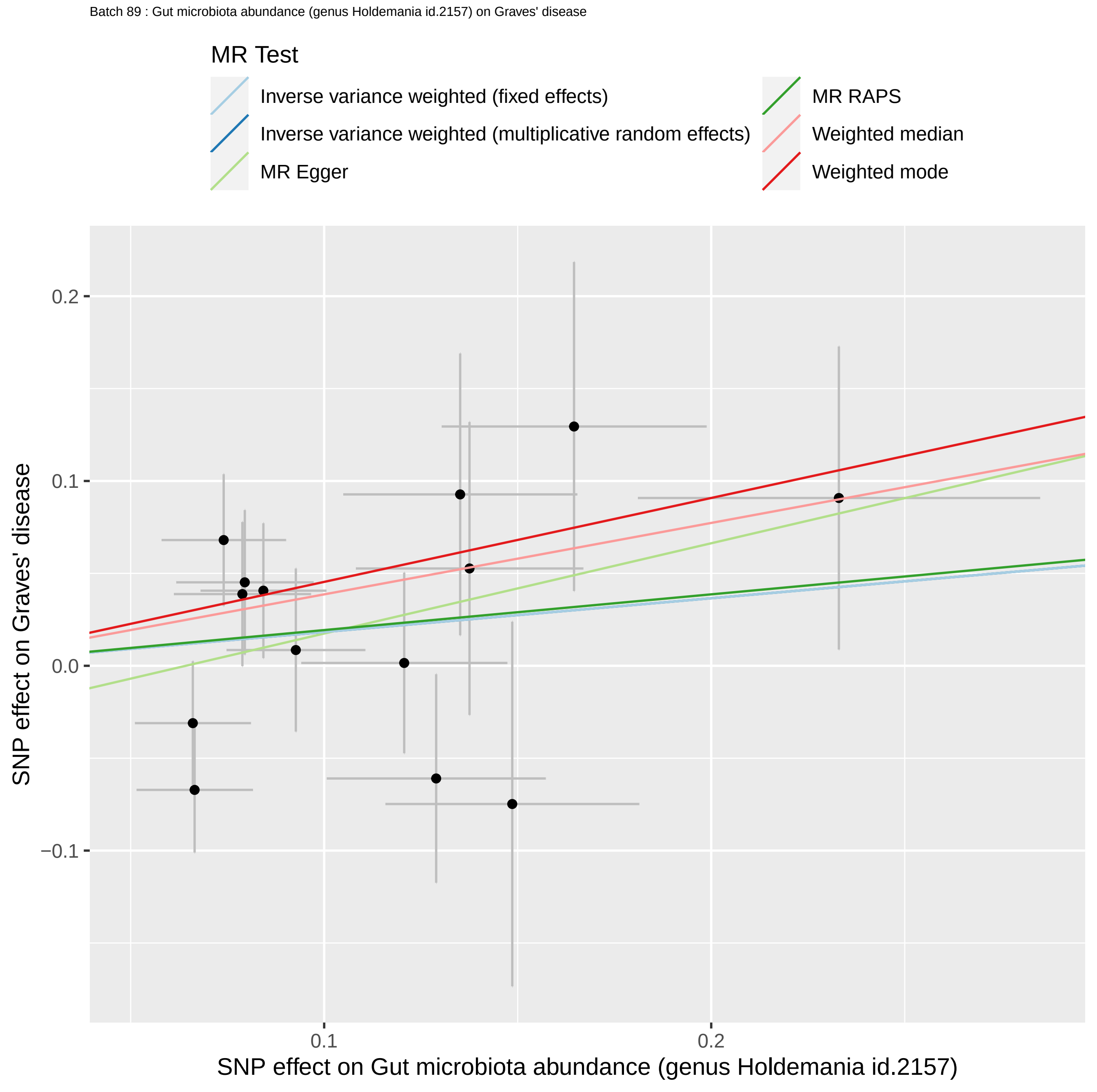
Batch 89 : Gut microbiota abundance (genus Holdemania id.2157) on Graves' disease
MR Test
Inverse variance weighted (fixed effects)
Inverse variance weighted (multiplicative random effects)
MR Egger
MR RAPS
Weighted median
Weighted mode
SNP effect on Graves' disease
SNP effect on Gut microbiota abundance (genus Holdemania id.2157)
0.2
0.1
0.0
−0.1
0.1
0.2

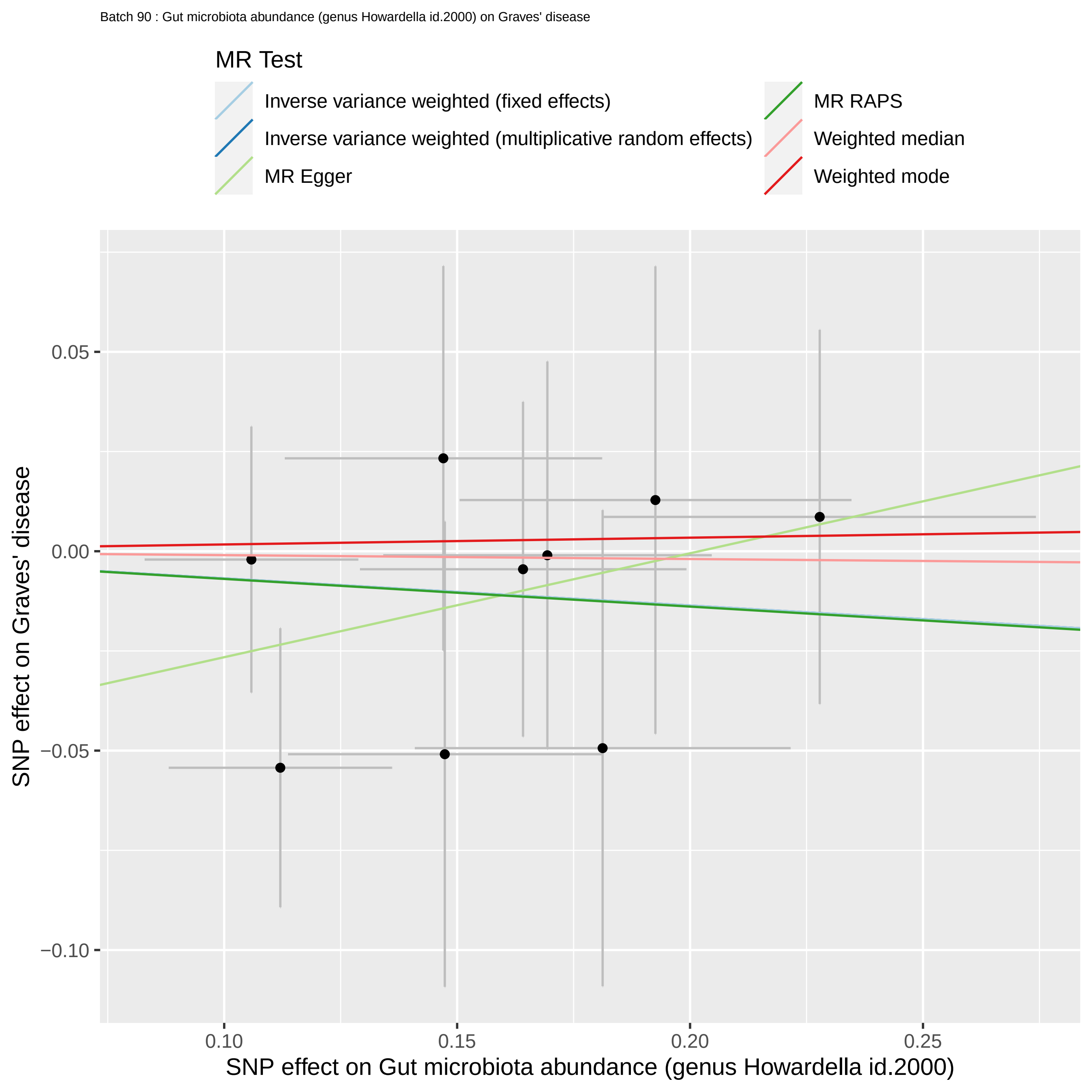
Batch 90 : Gut microbiota abundance (genus Howardella id.2000) on Graves' disease
MR Test
Inverse variance weighted (fixed effects)
Inverse variance weighted (multiplicative random effects)
MR Egger
MR RAPS
Weighted median
Weighted mode
0.05
0.00
−0.05
−0.10
0.10
0.15
0.20
0.25
SNP effect on Graves' disease
SNP effect on Gut microbiota abundance (genus Howardella id.2000)

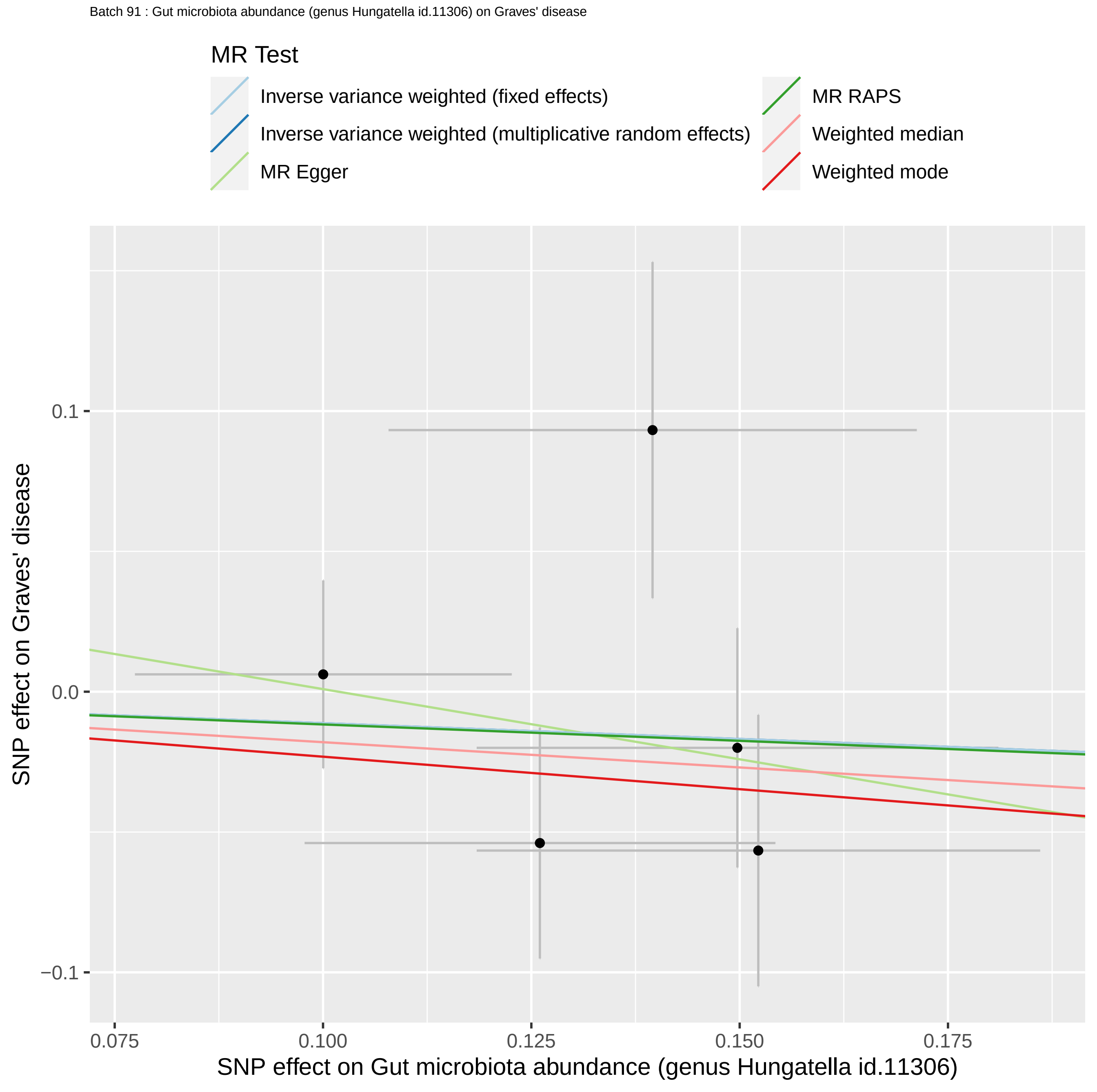

Batch 91 : Gut microbiota abundance (genus Hungatella id.11306) on Graves' disease
MR Test
Inverse variance weighted (fixed effects)
Inverse variance weighted (multiplicative random effects)
MR Egger
MR RAPS
Weighted median
Weighted mode
0.1
0.0
−0.1
0.075
0.100
0.125
0.150
0.175
SNP effect on Graves' disease
SNP effect on Gut microbiota abundance (genus Hungatella id.11306)

Batch 92 : Gut microbiota abundance (genus Intestinibacter id.11345) on Graves' disease

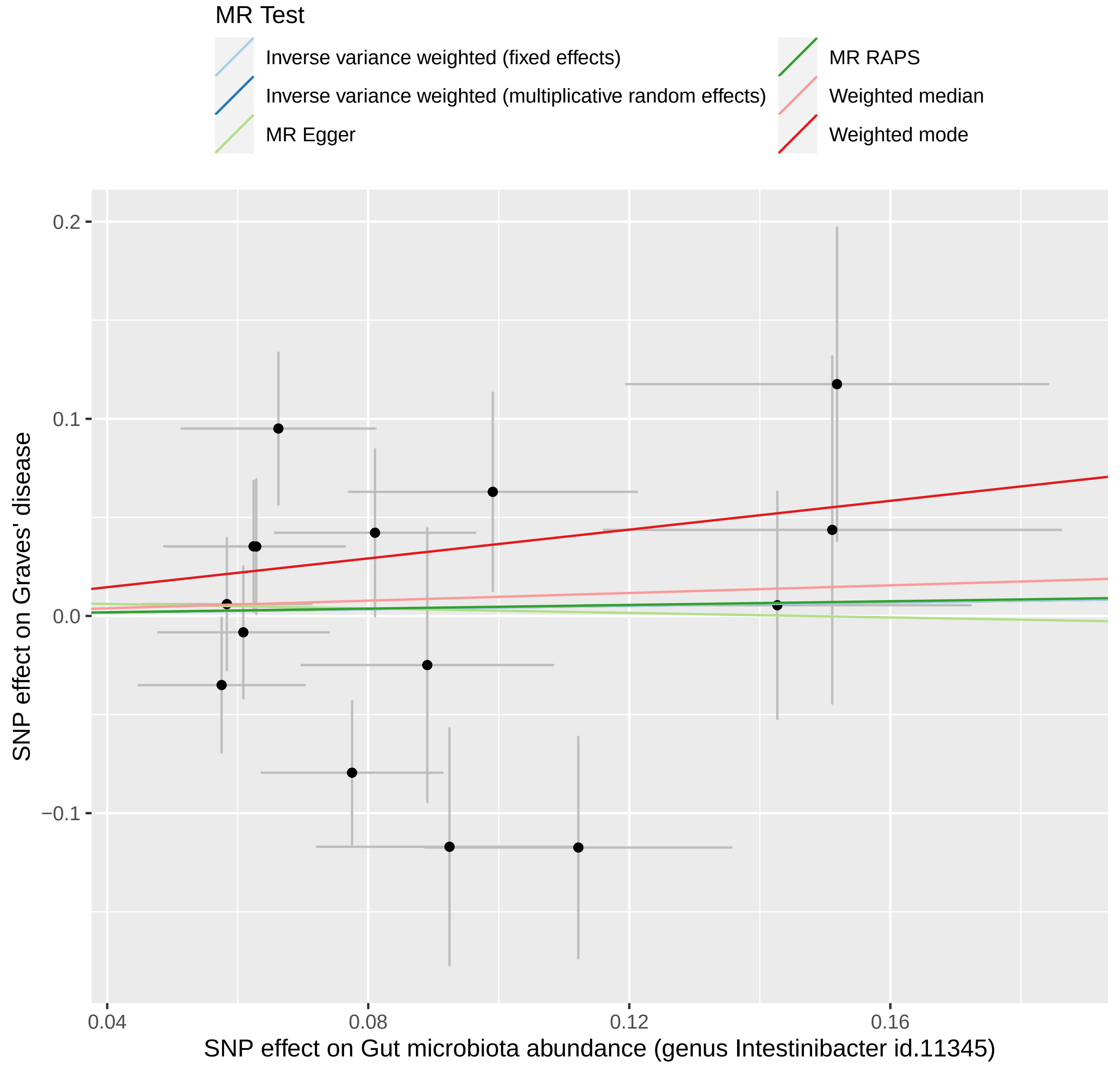

Batch 93 : Gut microbiota abundance (genus Intestinimonas id.2062) on Graves' disease

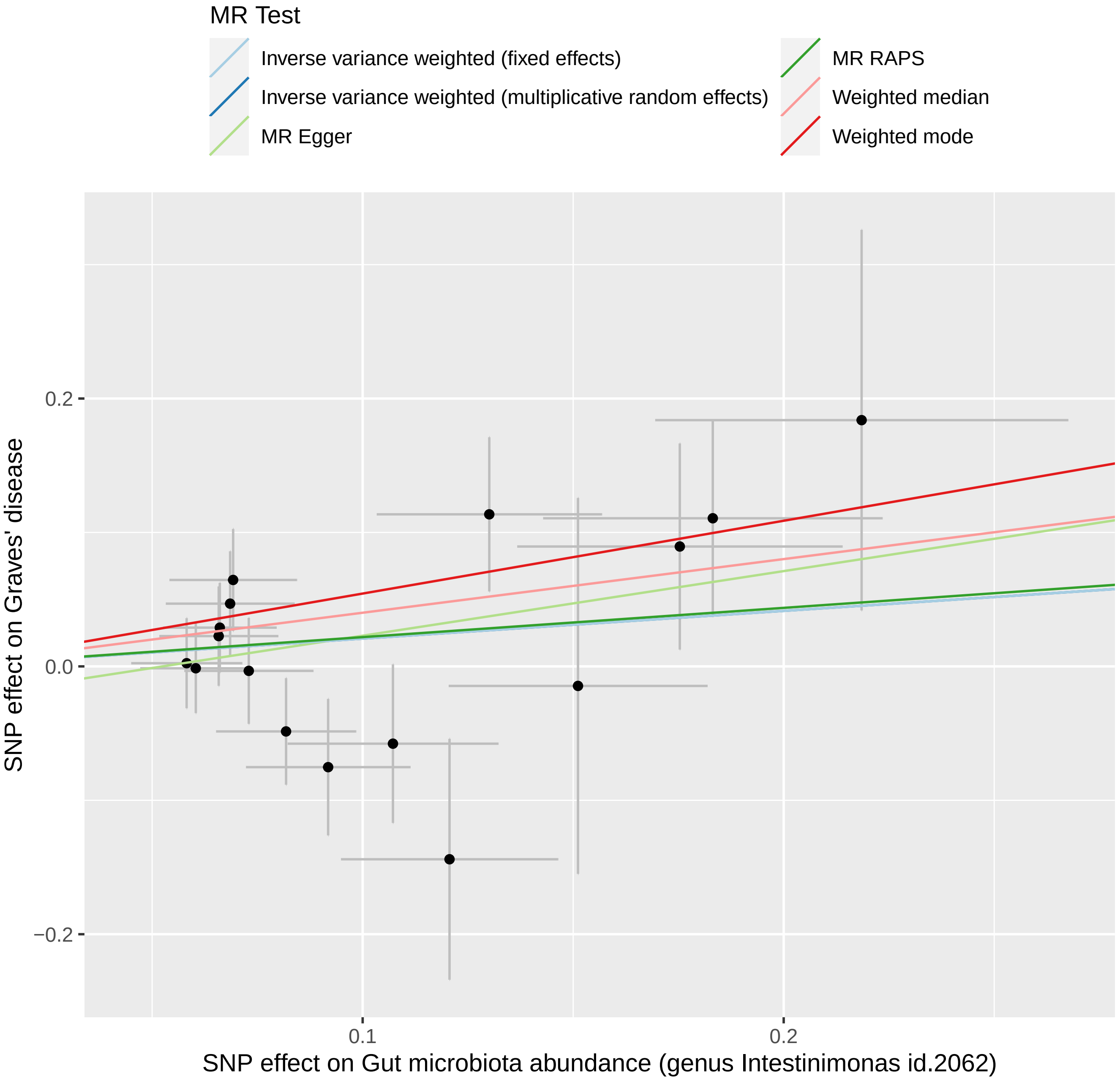

Batch 94 : Gut microbiota abundance (genus Lachnoclostridium id.11308) on Graves' disease

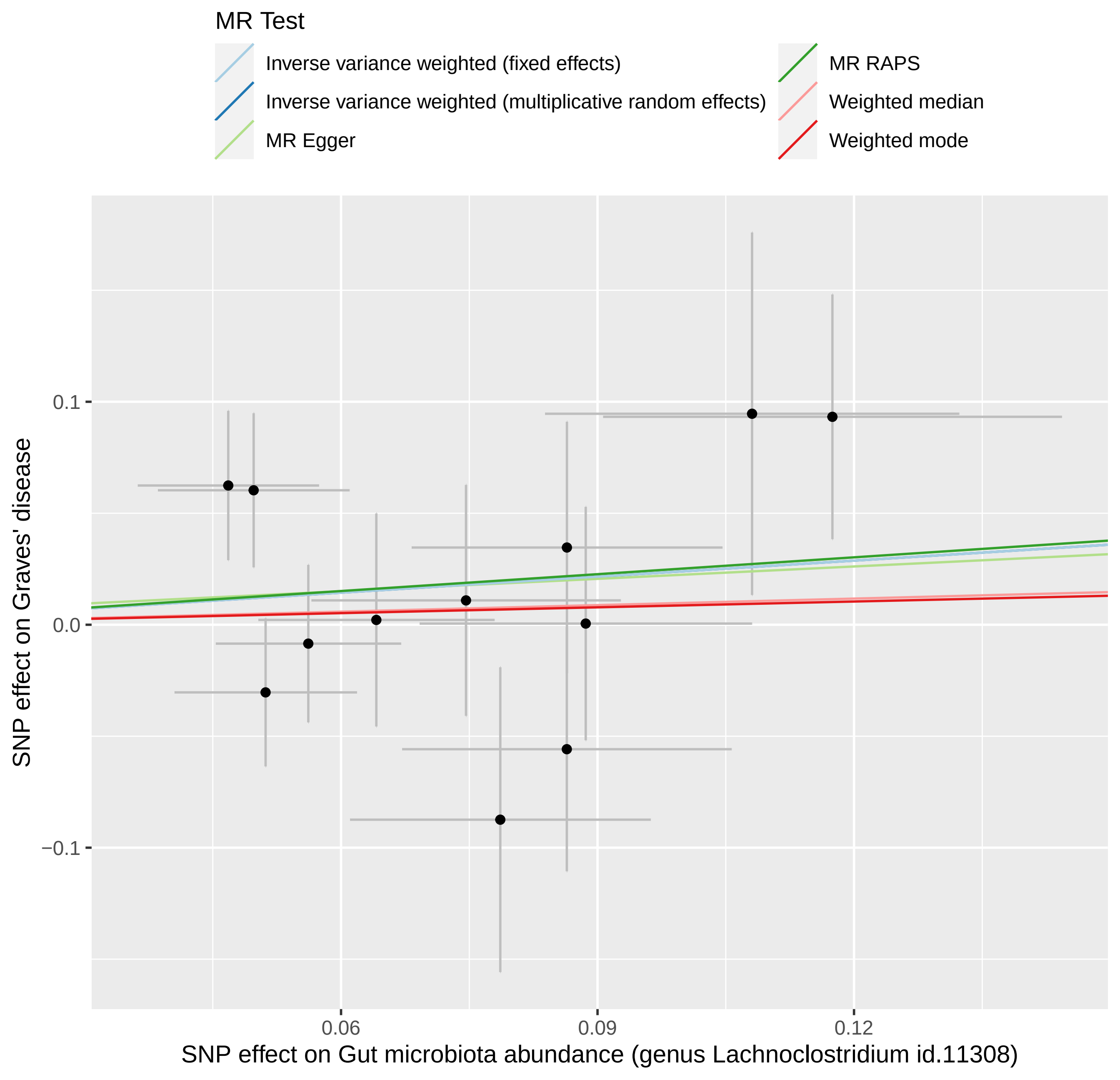

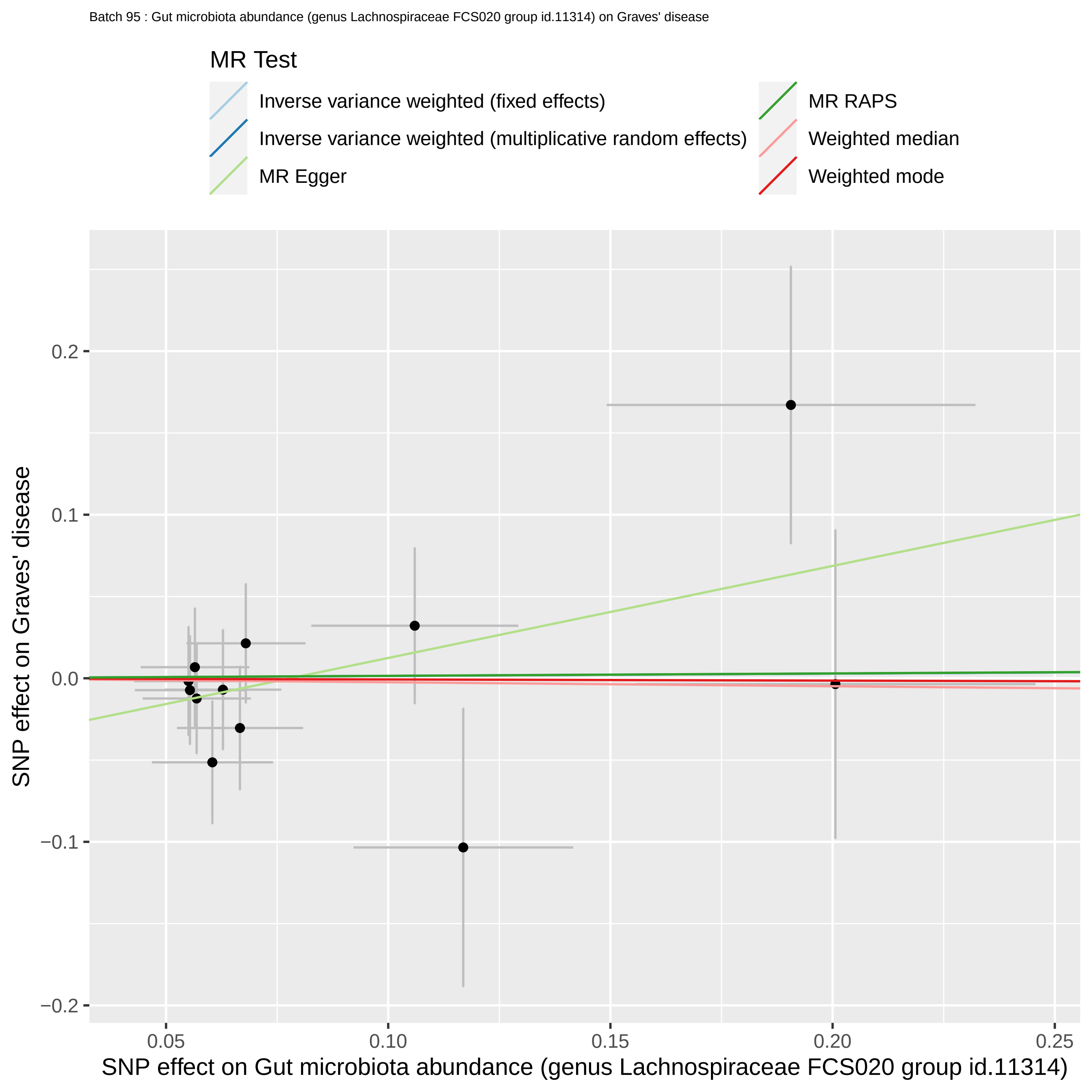

Batch 95 : Gut microbiota abundance (genus Lachnospiraceae FCS020 group id.11314) on Graves' disease
MR Test
Inverse variance weighted (fixed effects)
Inverse variance weighted (multiplicative random effects)
MR Egger
MR RAPS
Weighted median
Weighted mode
SNP effect on Graves' disease
0.2
0.1
0.0
−0.1
−0.2
0.05
0.10
0.15
0.20
0.25
SNP effect on Gut microbiota abundance (genus Lachnospiraceae FCS020 group id.11314)

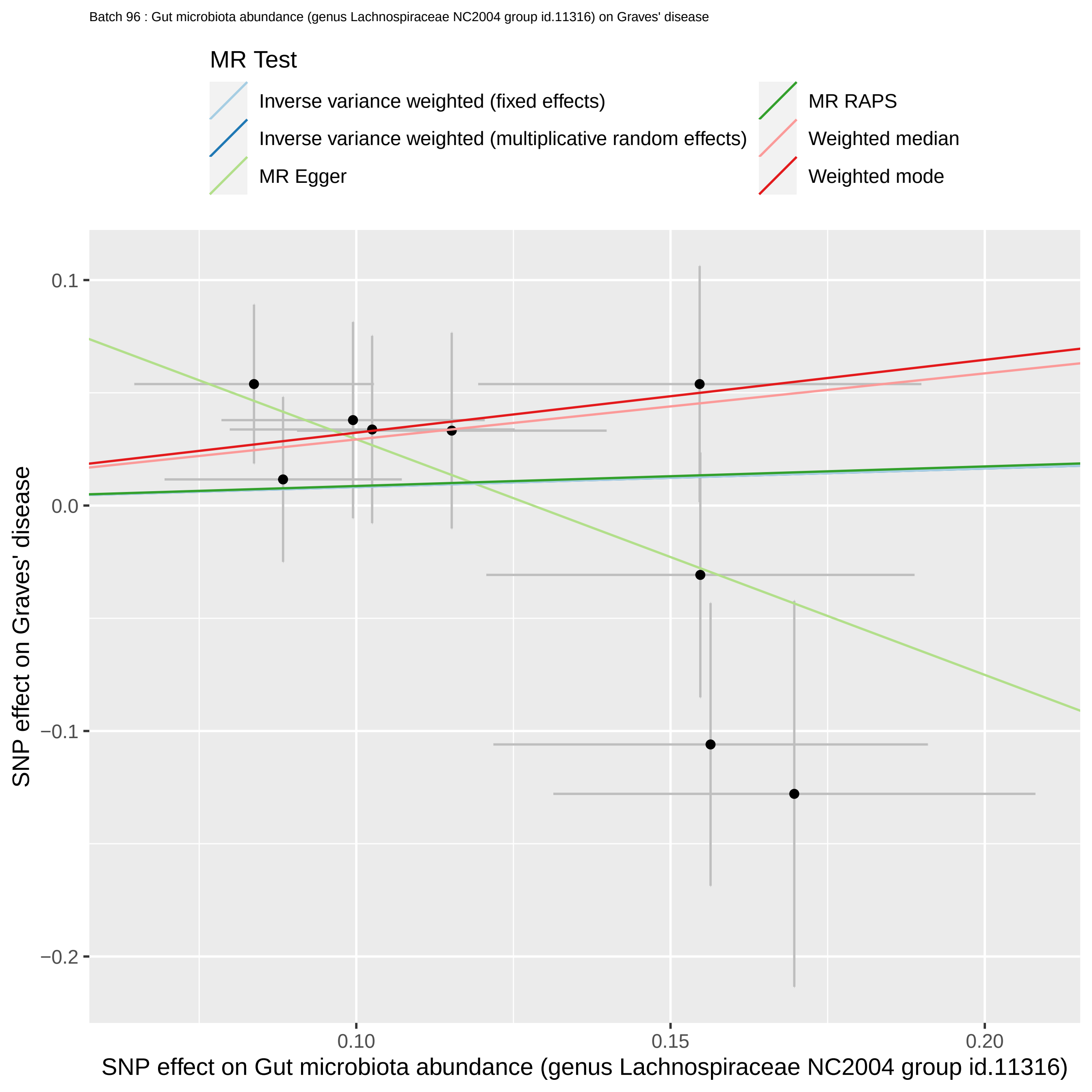

Batch 96 : Gut microbiota abundance (genus Lachnospiraceae NC2004 group id.11316) on Graves' disease
MR Test
Inverse variance weighted (fixed effects)
Inverse variance weighted (multiplicative random effects)
MR Egger
MR RAPS
Weighted median
Weighted mode
0.1
0.0
−0.1
−0.2
0.10
0.15
0.20
SNP effect on Graves' disease
SNP effect on Gut microbiota abundance (genus Lachnospiraceae NC2004 group id.11316)

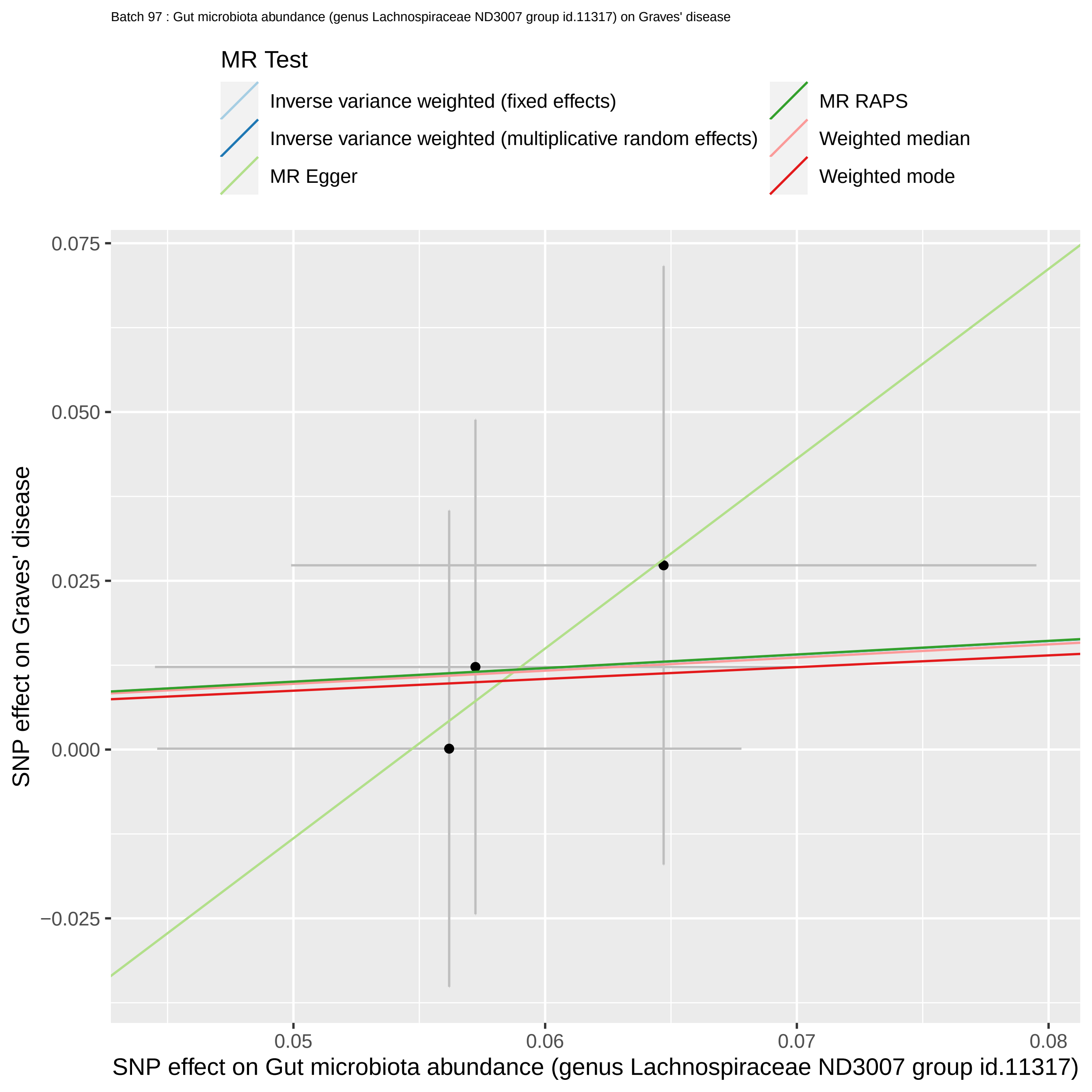

Batch 97 : Gut microbiota abundance (genus Lachnospiraceae ND3007 group id.11317) on Graves' disease
MR Test
Inverse variance weighted (fixed effects)
Inverse variance weighted (multiplicative random effects)
MR Egger
MR RAPS
Weighted median
Weighted mode
0.075
0.050
0.025
0.000
−0.025
0.05
0.06
0.07
0.08
SNP effect on Graves' disease
SNP effect on Gut microbiota abundance (genus Lachnospiraceae ND3007 group id.11317)

Batch 98 : Gut microbiota abundance (genus Lachnospiraceae NK4A136 group id.11319) on Graves' disease

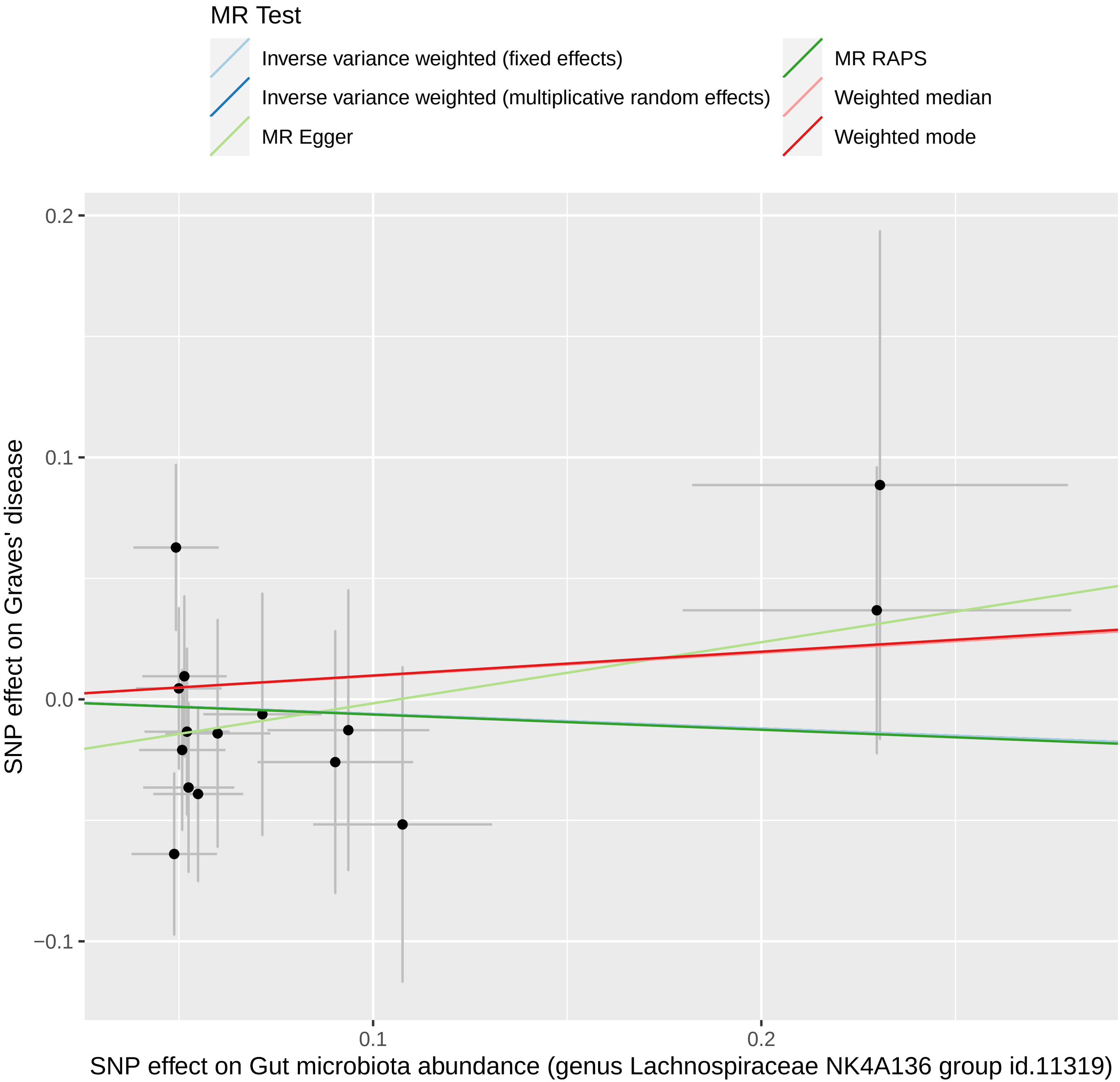

Batch 99 : Gut microbiota abundance (genus Lachnospiraceae UCG001 id.11321) on Graves' disease

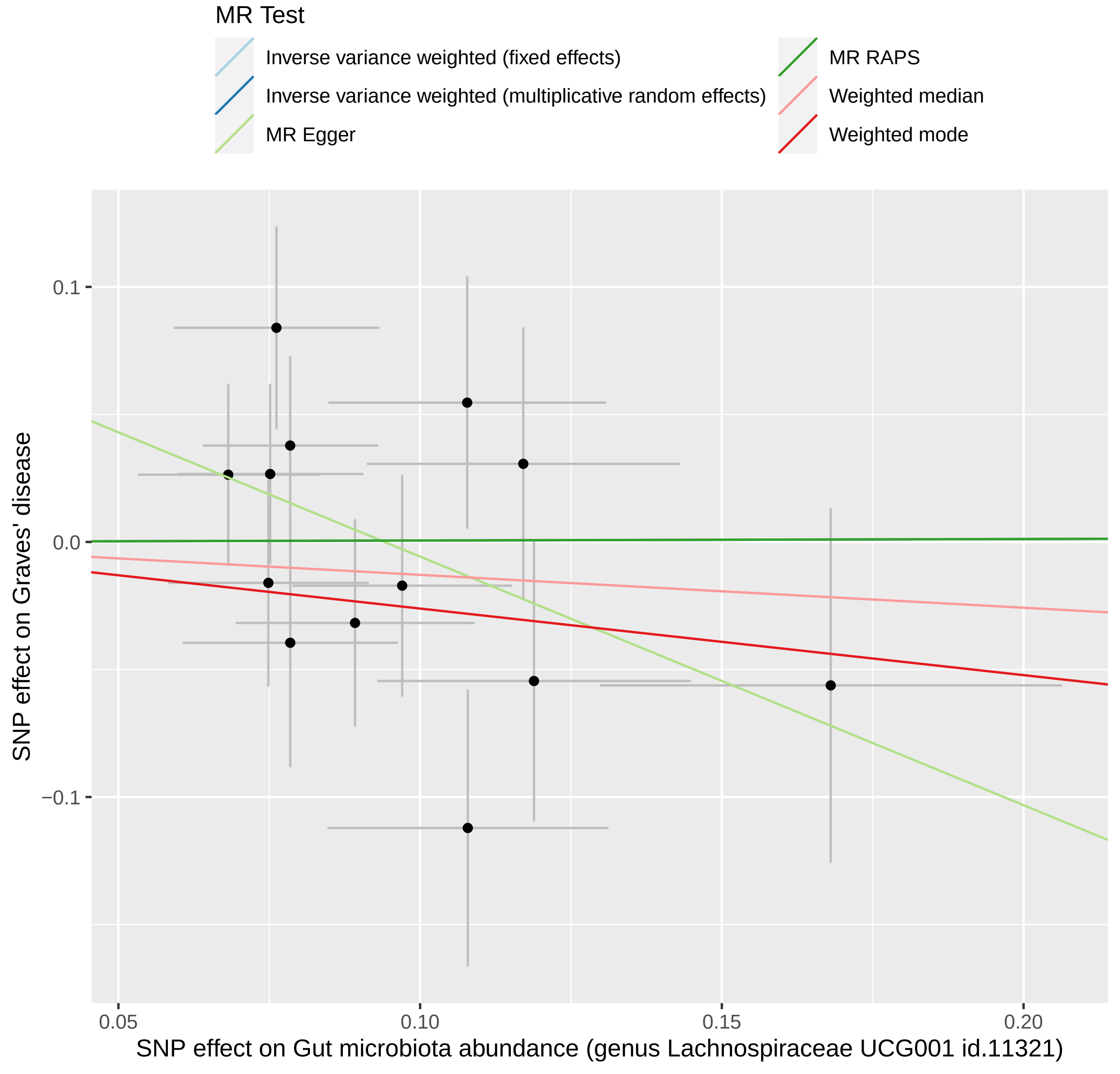

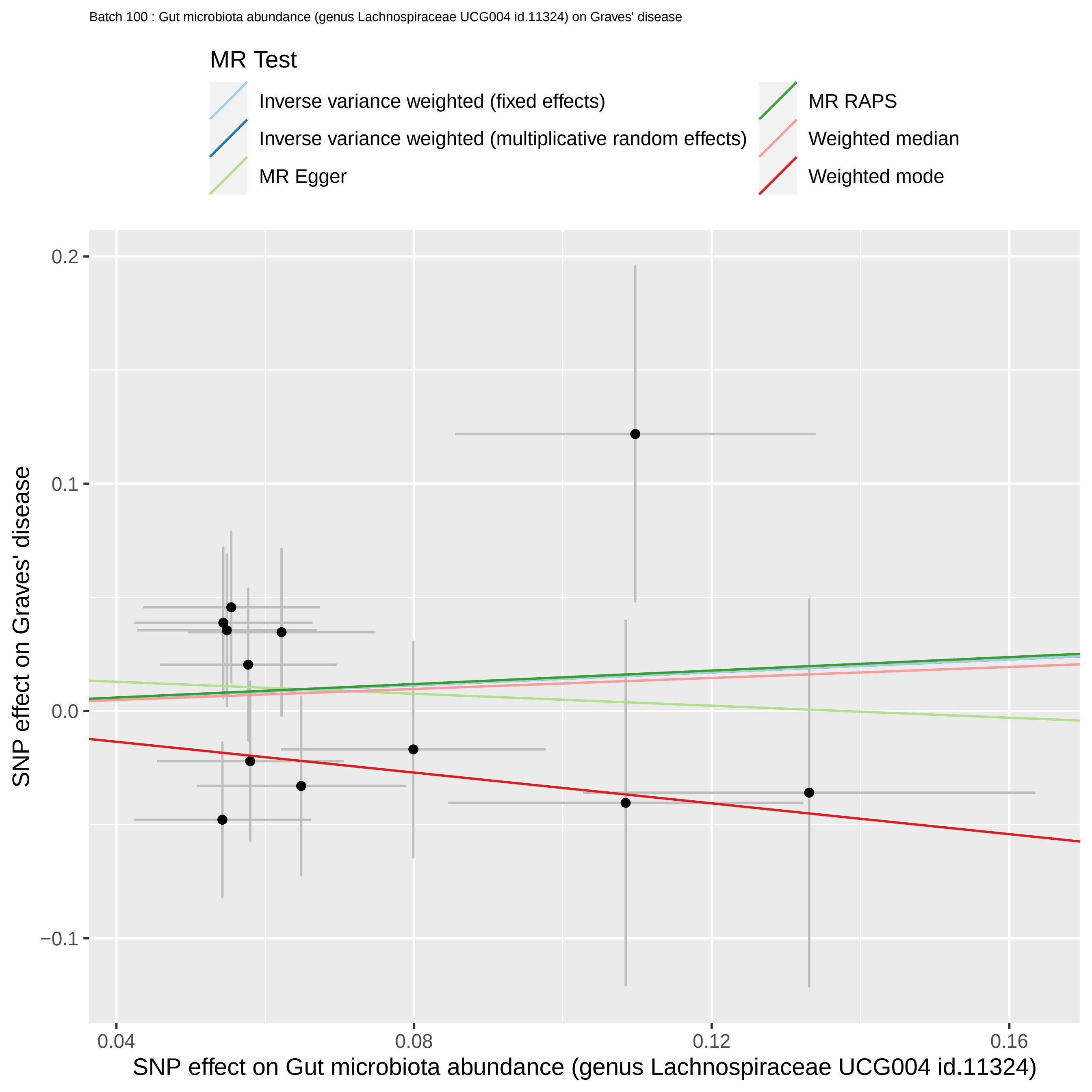

Batch 100 : Gut microbiota abundance (genus Lachnospiraceae UCG004 id.11324) on Graves' disease
MR Test
Inverse variance weighted (fixed effects)
Inverse variance weighted (multiplicative random effects)
MR Egger
MR RAPS
Weighted median
Weighted mode
SNP effect on Graves' disease
0.2
0.1
0.0
−0.1
0.04
0.08
0.12
0.16
SNP effect on Gut microbiota abundance (genus Lachnospiraceae UCG004 id.11324)

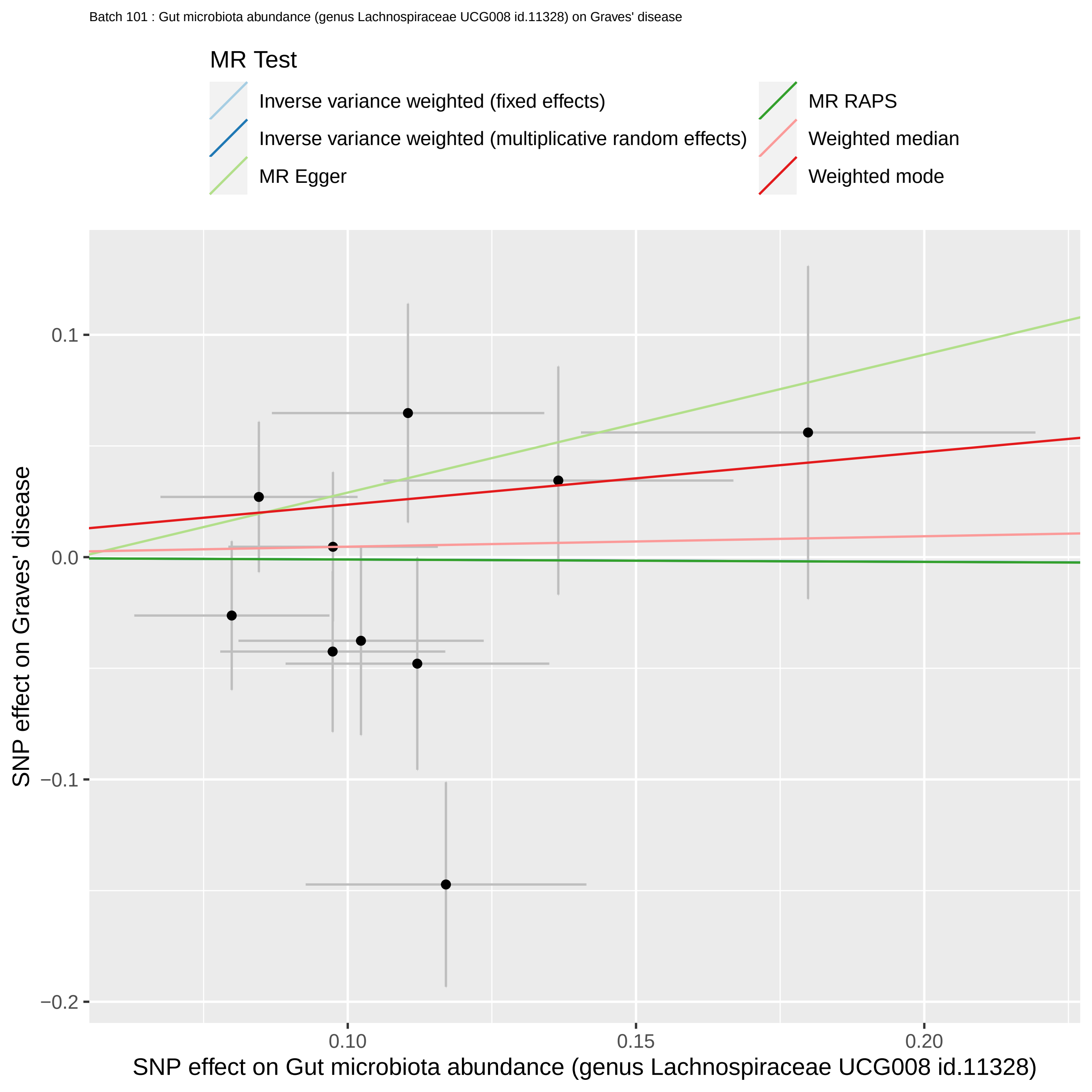

Batch 101 : Gut microbiota abundance (genus Lachnospiraceae UCG008 id.11328) on Graves' disease
MR Test
Inverse variance weighted (fixed effects)
Inverse variance weighted (multiplicative random effects)
MR Egger
MR RAPS
Weighted median
Weighted mode
0.1
0.0
−0.1
−0.2
0.10
0.15
0.20
SNP effect on Graves' disease
SNP effect on Gut microbiota abundance (genus Lachnospiraceae UCG008 id.11328)

Batch 102 : Gut microbiota abundance (genus Lachnospiraceae UCG010 id.11330) on Graves' disease

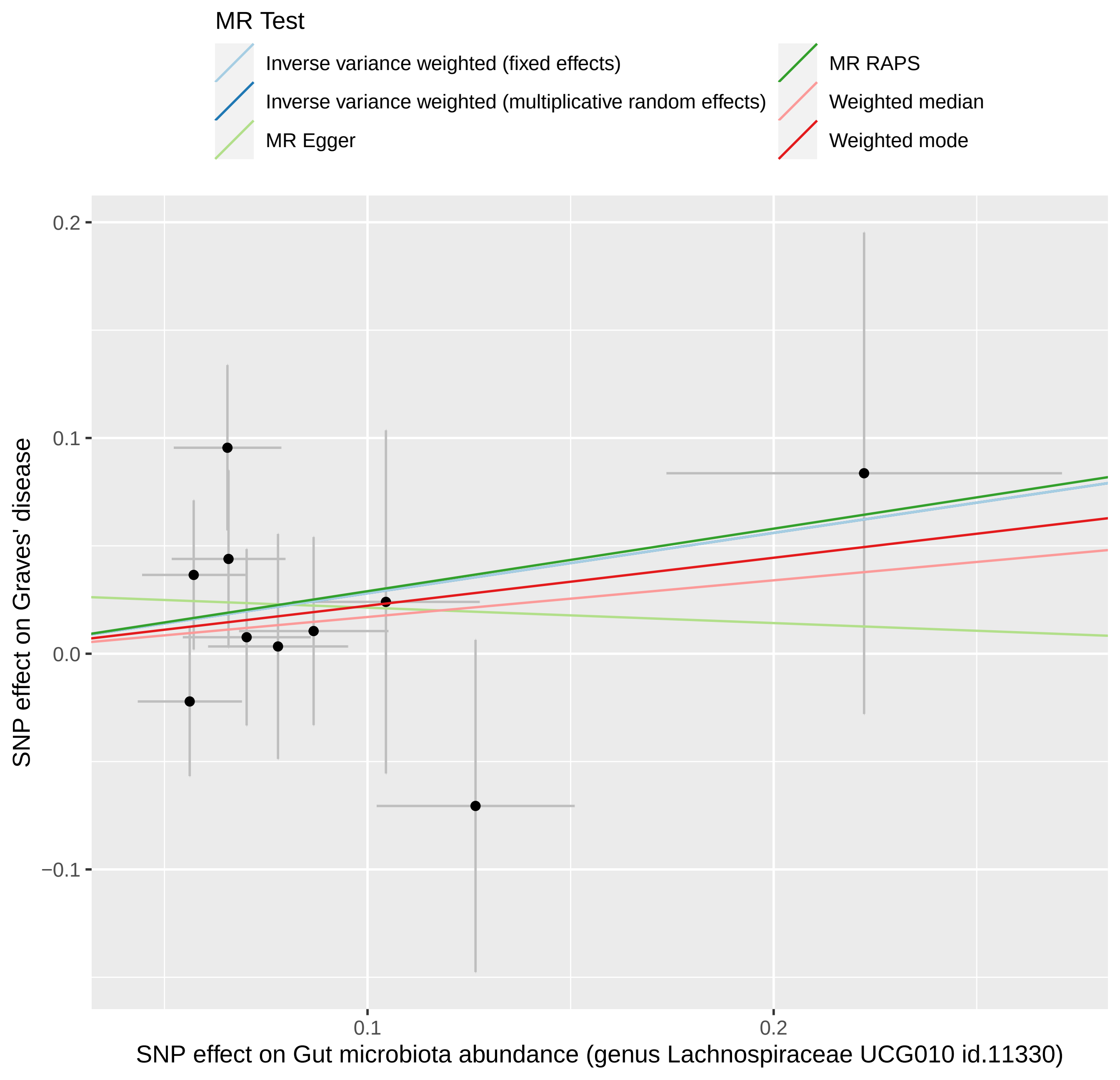

Batch 104 : Gut microbiota abundance (genus Lactobacillus id.1837) on Graves' disease

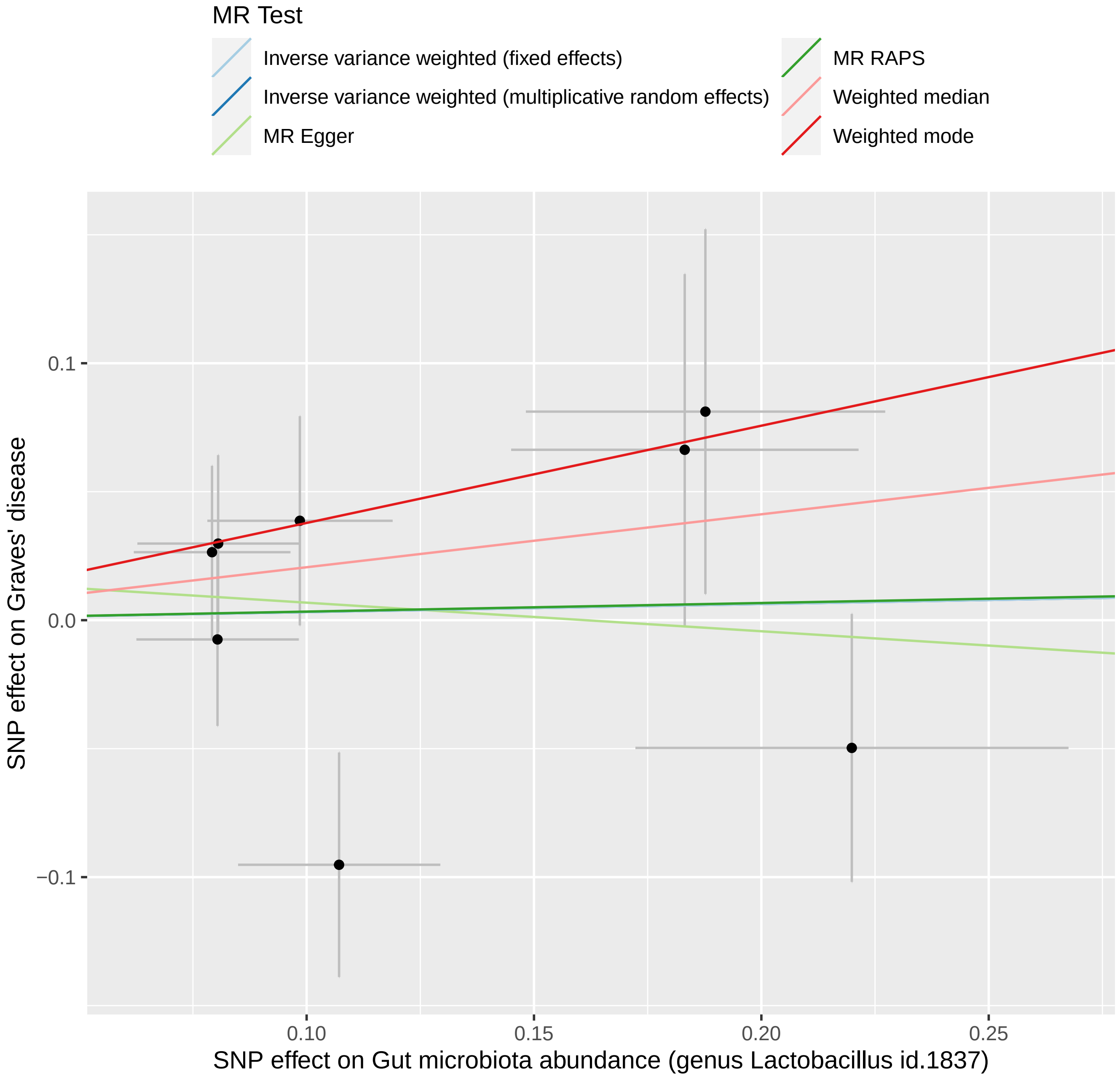

Batch 105 : Gut microbiota abundance (genus Lactococcus id.1851) on Graves' disease

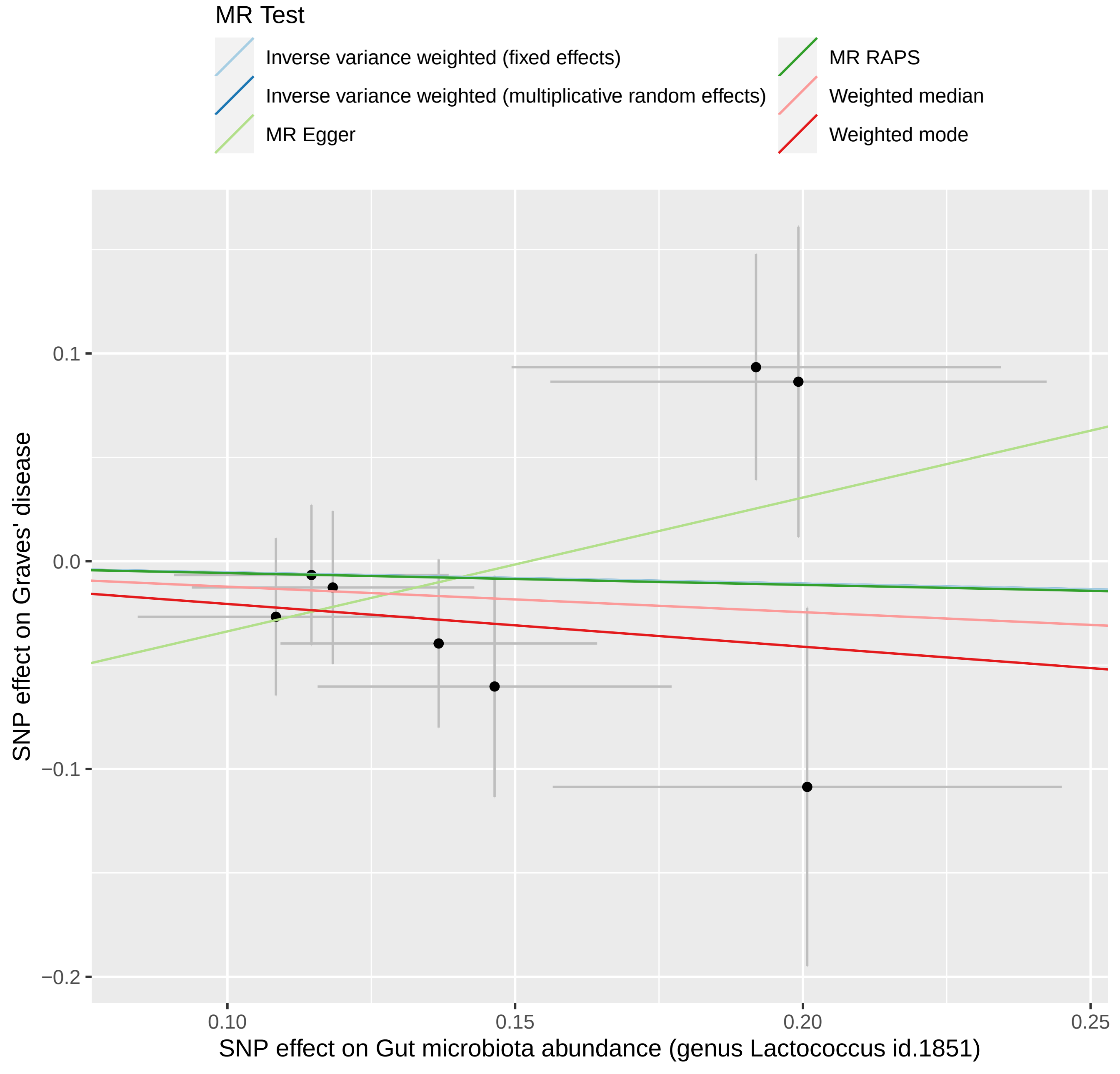

Batch 106 : Gut microbiota abundance (genus Marvinbryantia id.2005) on Graves' disease

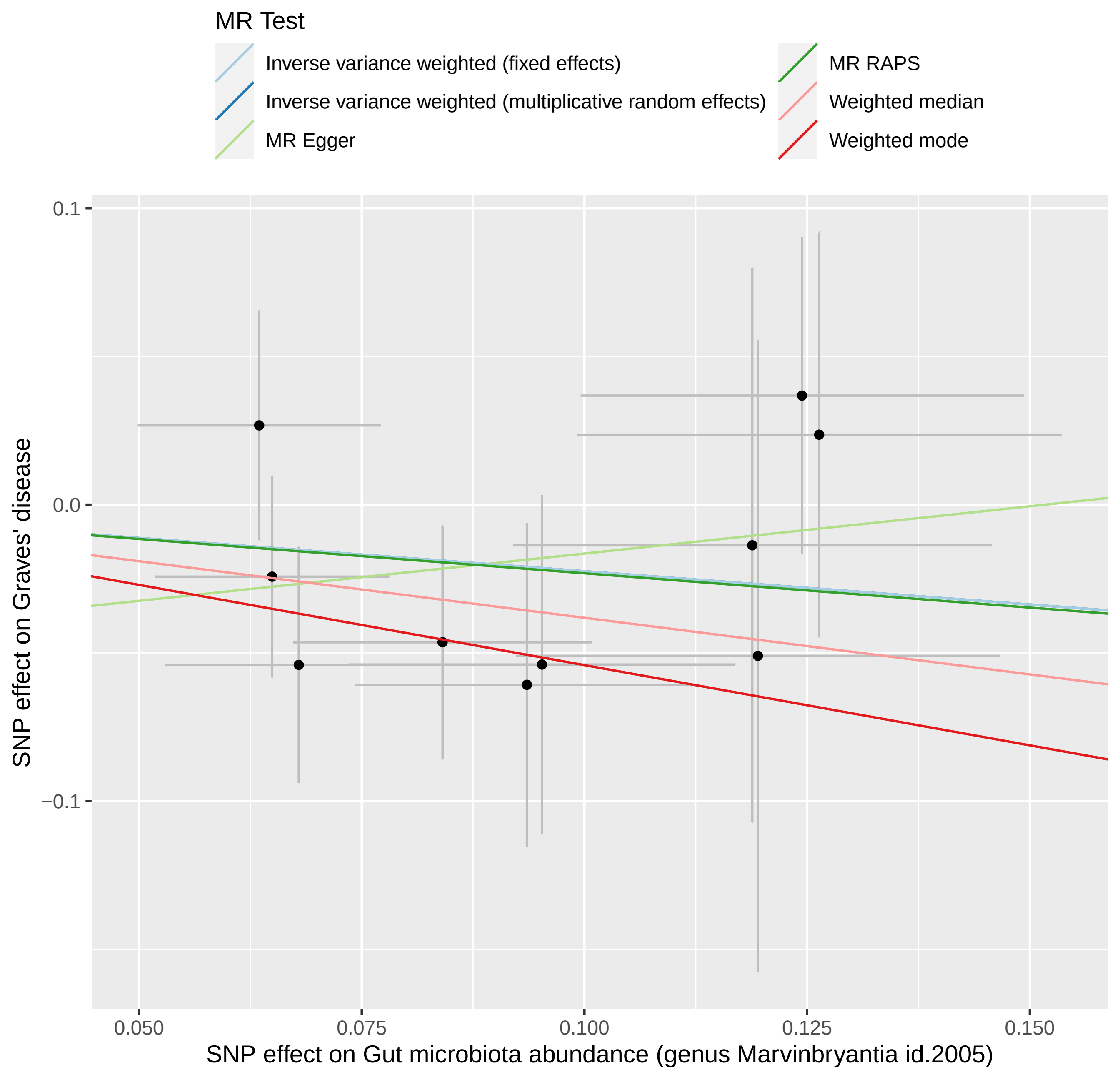

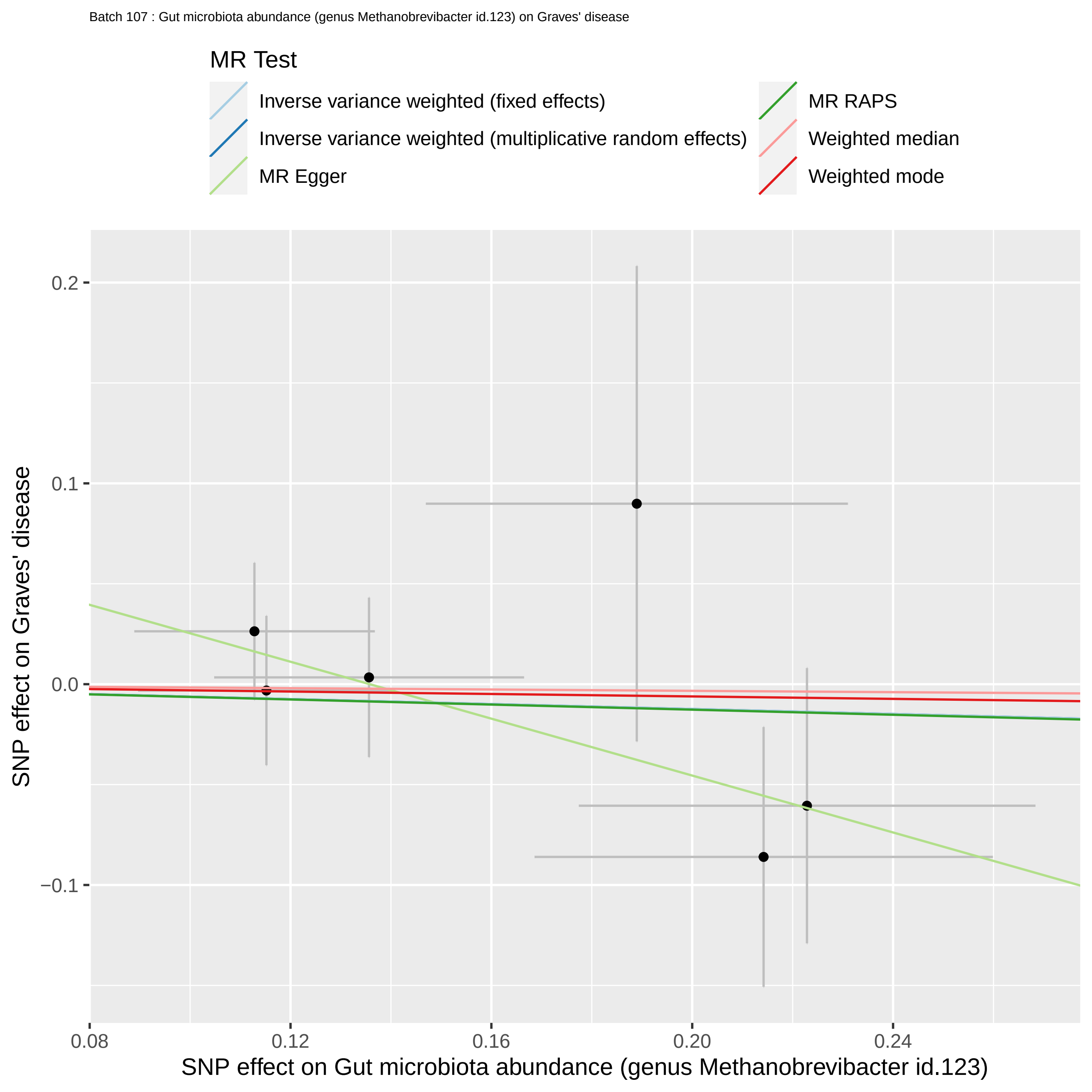
Batch 107 : Gut microbiota abundance (genus Methanobrevibacter id.123) on Graves' disease
MR Test
Inverse variance weighted (fixed effects)
Inverse variance weighted (multiplicative random effects)
MR Egger
MR RAPS
Weighted median
Weighted mode
0.2
0.1
0.0
−0.1
SNP effect on Graves' disease
0.08
0.12
0.16
0.20
0.24
SNP effect on Gut microbiota abundance (genus Methanobrevibacter id.123)

Batch 108 : Gut microbiota abundance (genus Odoribacter id.952) on Graves' disease

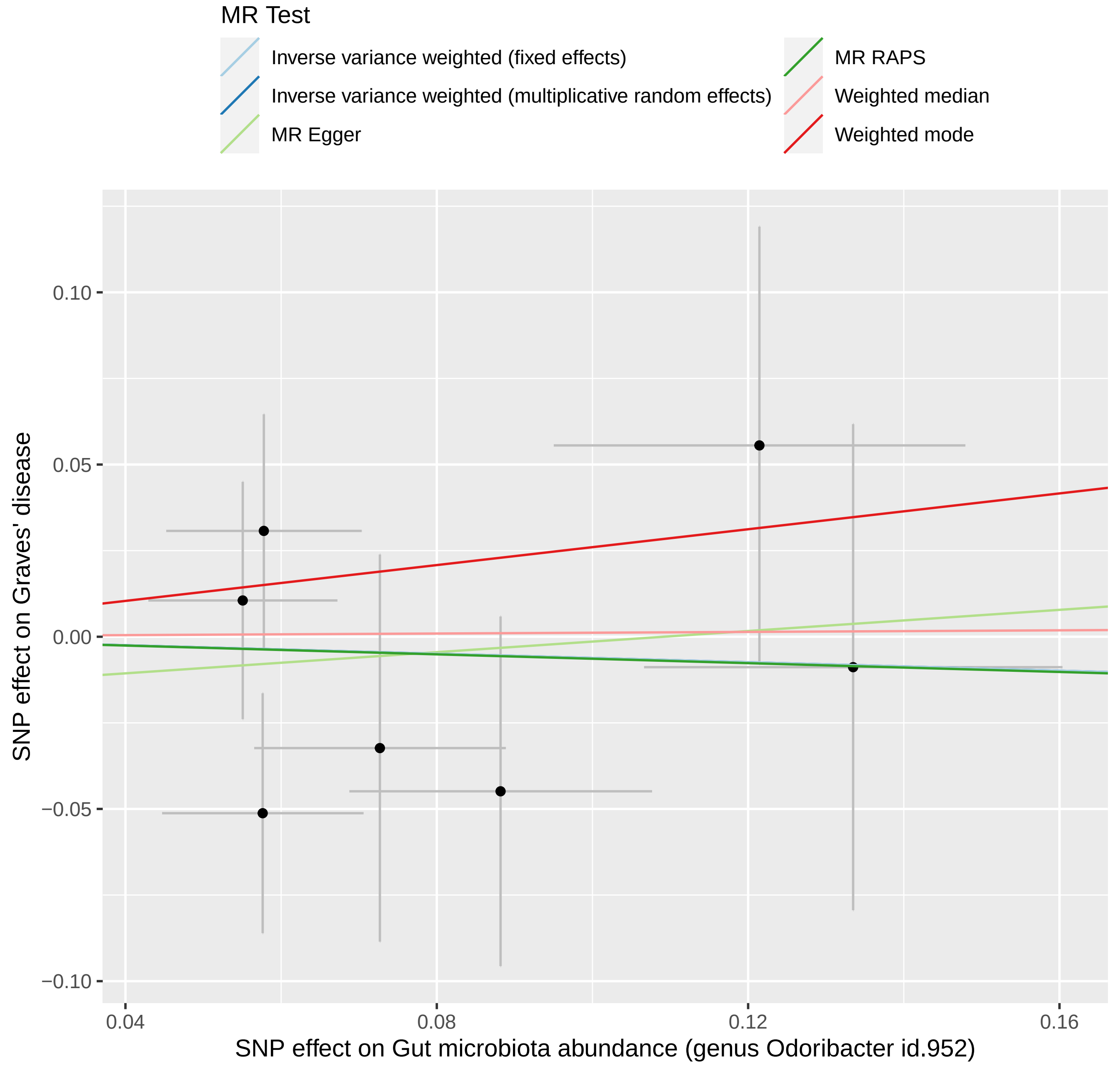

Batch 109 : Gut microbiota abundance (genus Olsenella id.822) on Graves' disease

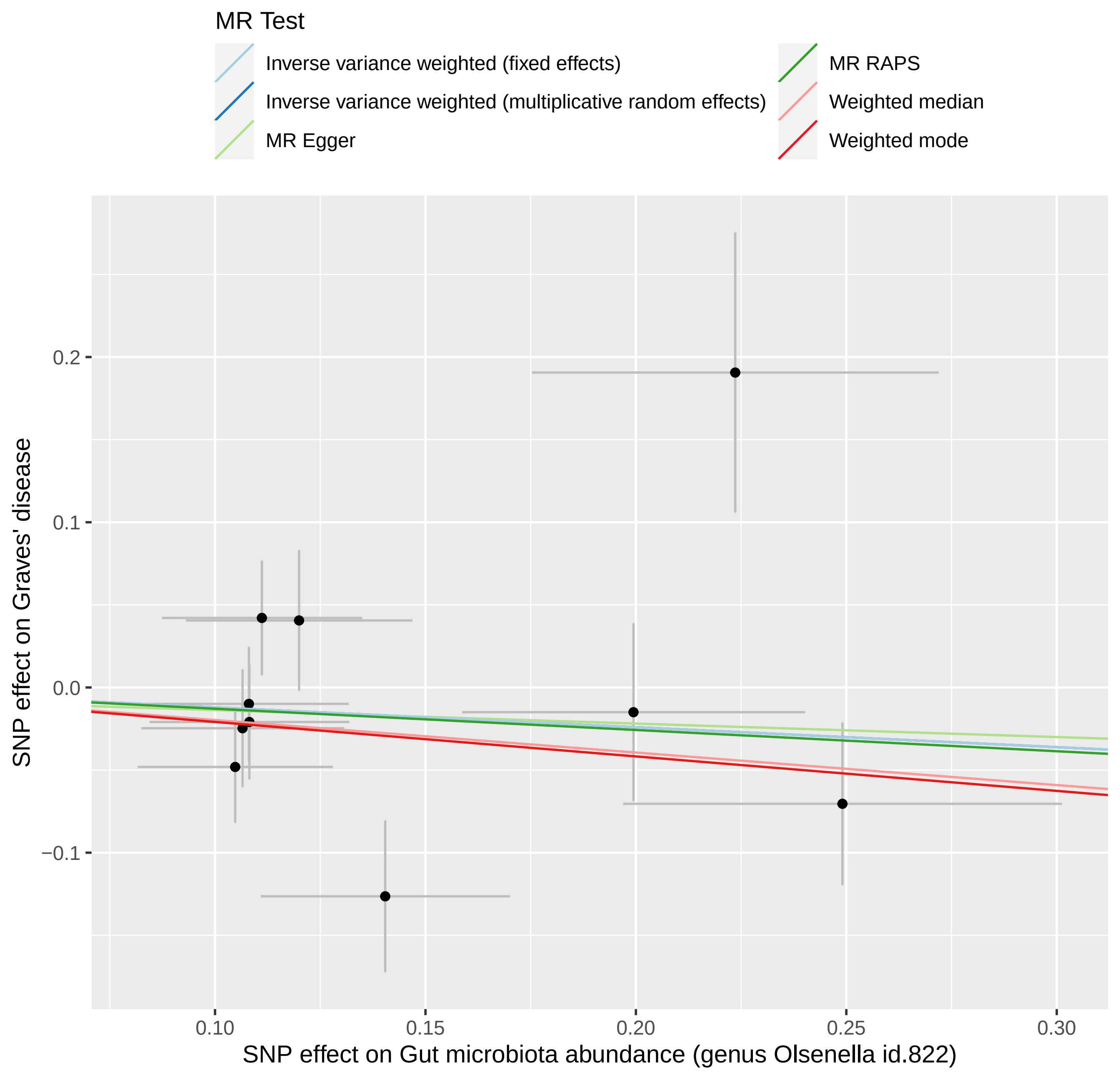

Batch 110 : Gut microbiota abundance (genus Oscillibacter id.2063) on Graves' disease

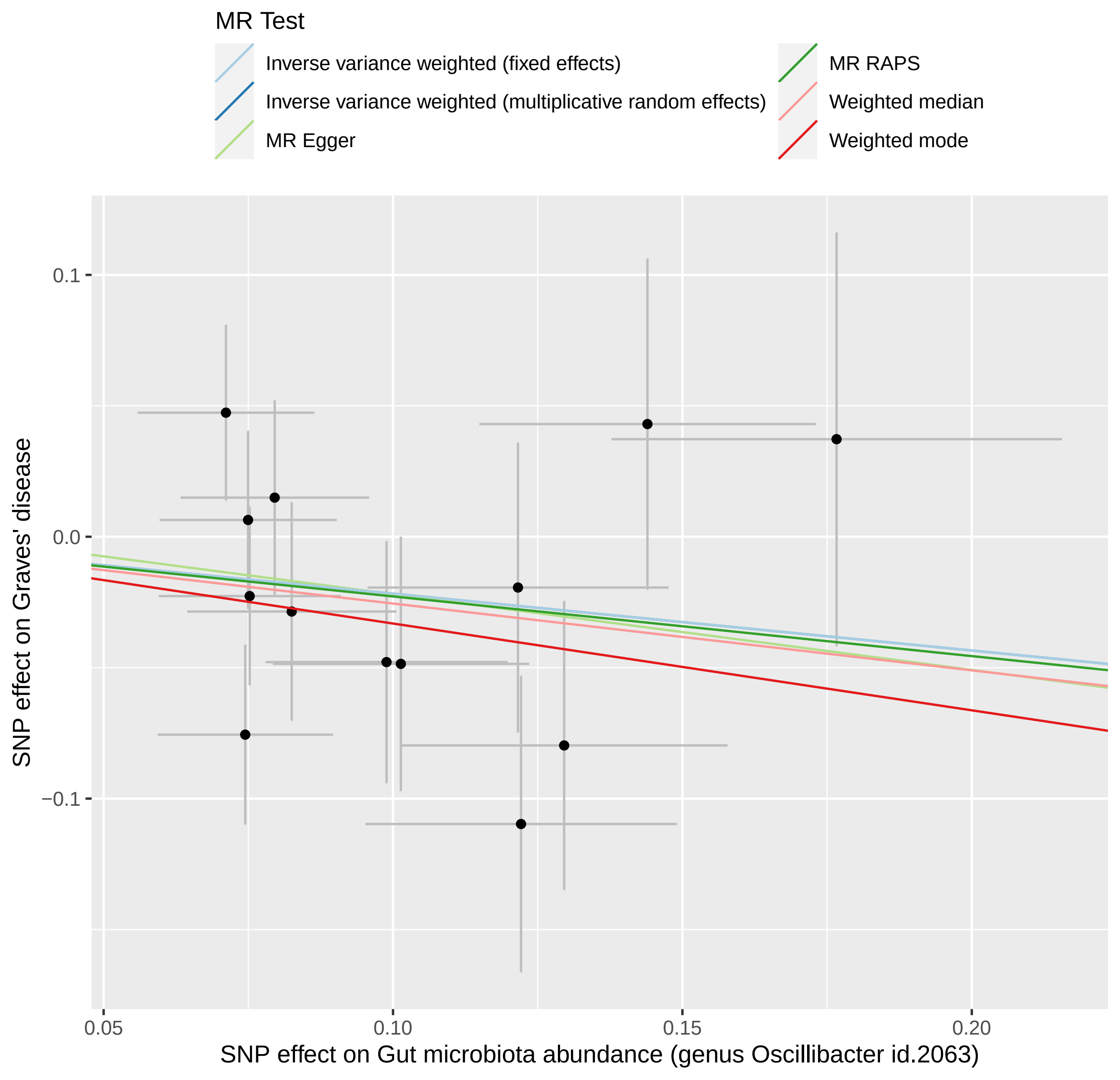

Batch 111 : Gut microbiota abundance (genus Oscillospira id.2064) on Graves' disease

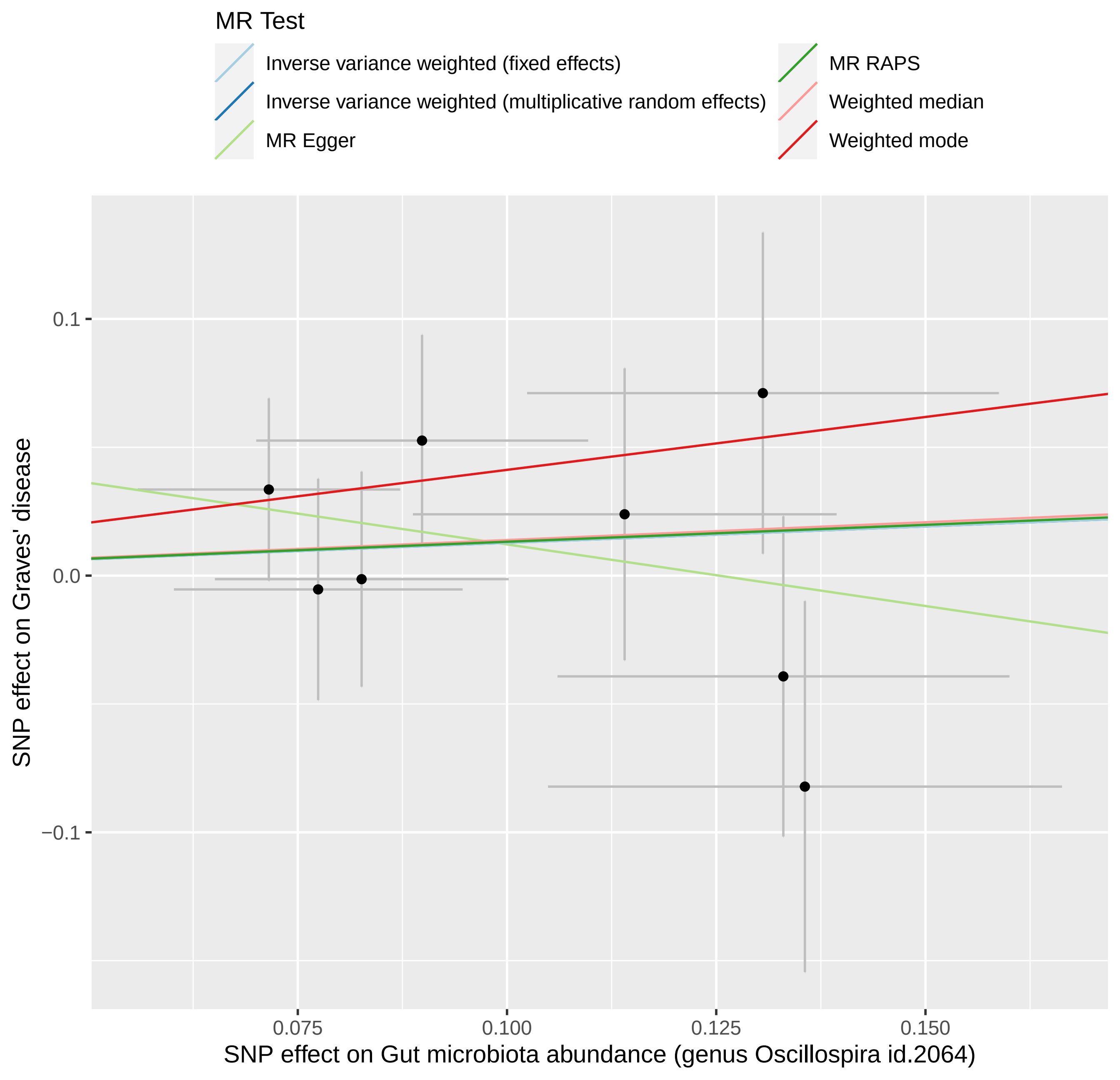

Batch 112 : Gut microbiota abundance (genus Oxalobacter id.2978) on Graves' disease

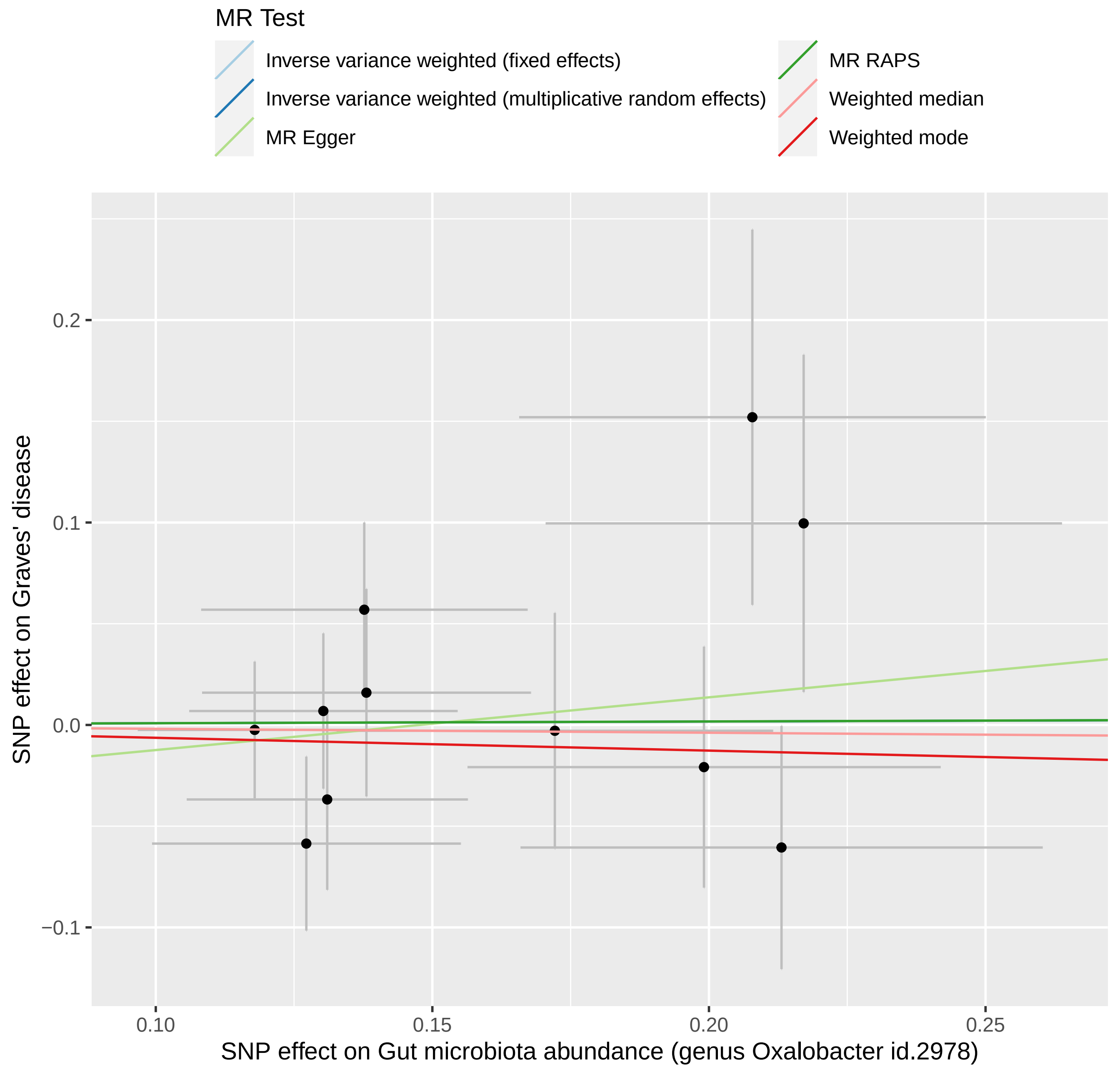

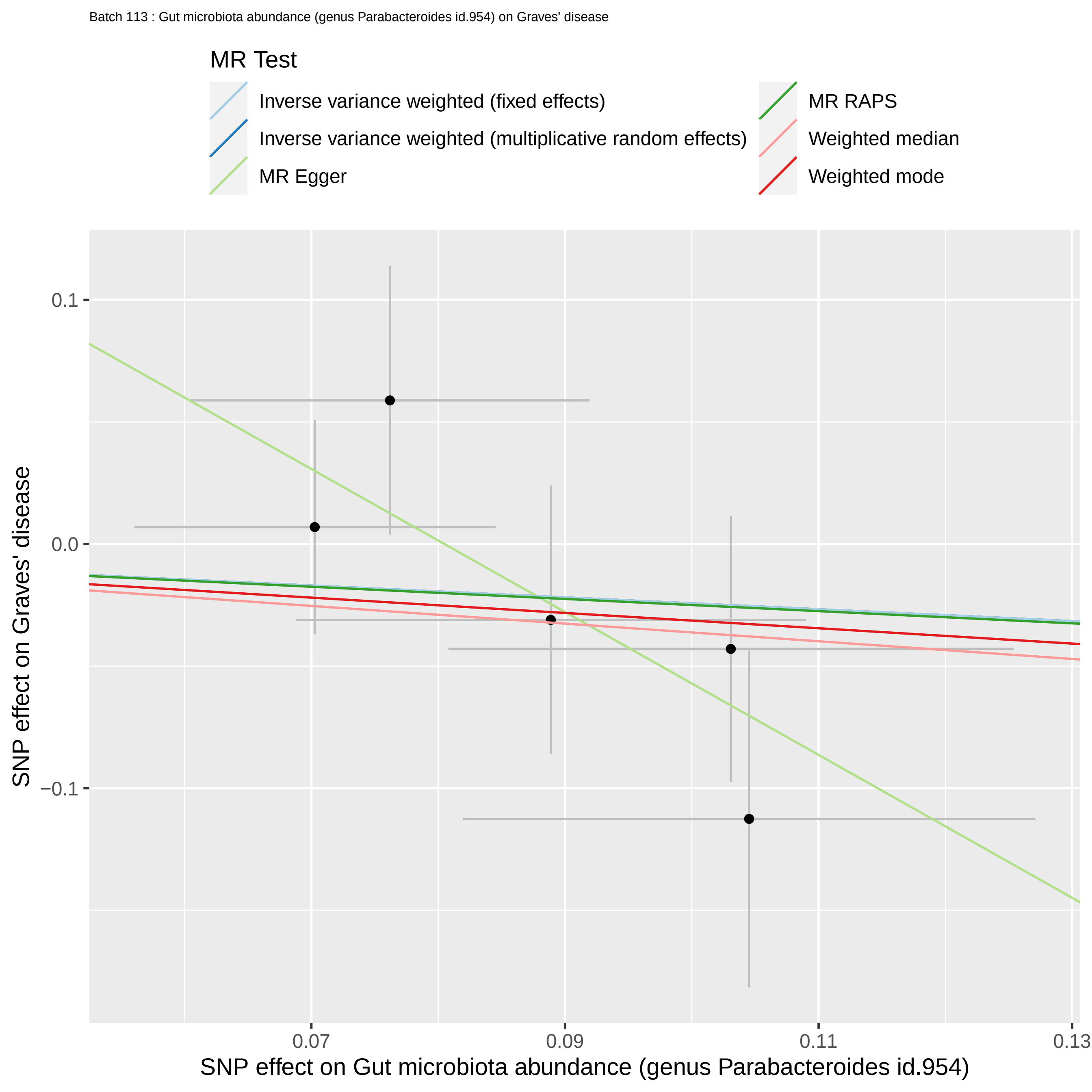
Batch 113 : Gut microbiota abundance (genus Parabacteroides id.954) on Graves' disease
MR Test
Inverse variance weighted (fixed effects)
Inverse variance weighted (multiplicative random effects)
MR Egger
MR RAPS
Weighted median
Weighted mode
0.1
0.0
−0.1
0.07
0.09
0.11
0.13
SNP effect on Graves' disease
SNP effect on Gut microbiota abundance (genus Parabacteroides id.954)

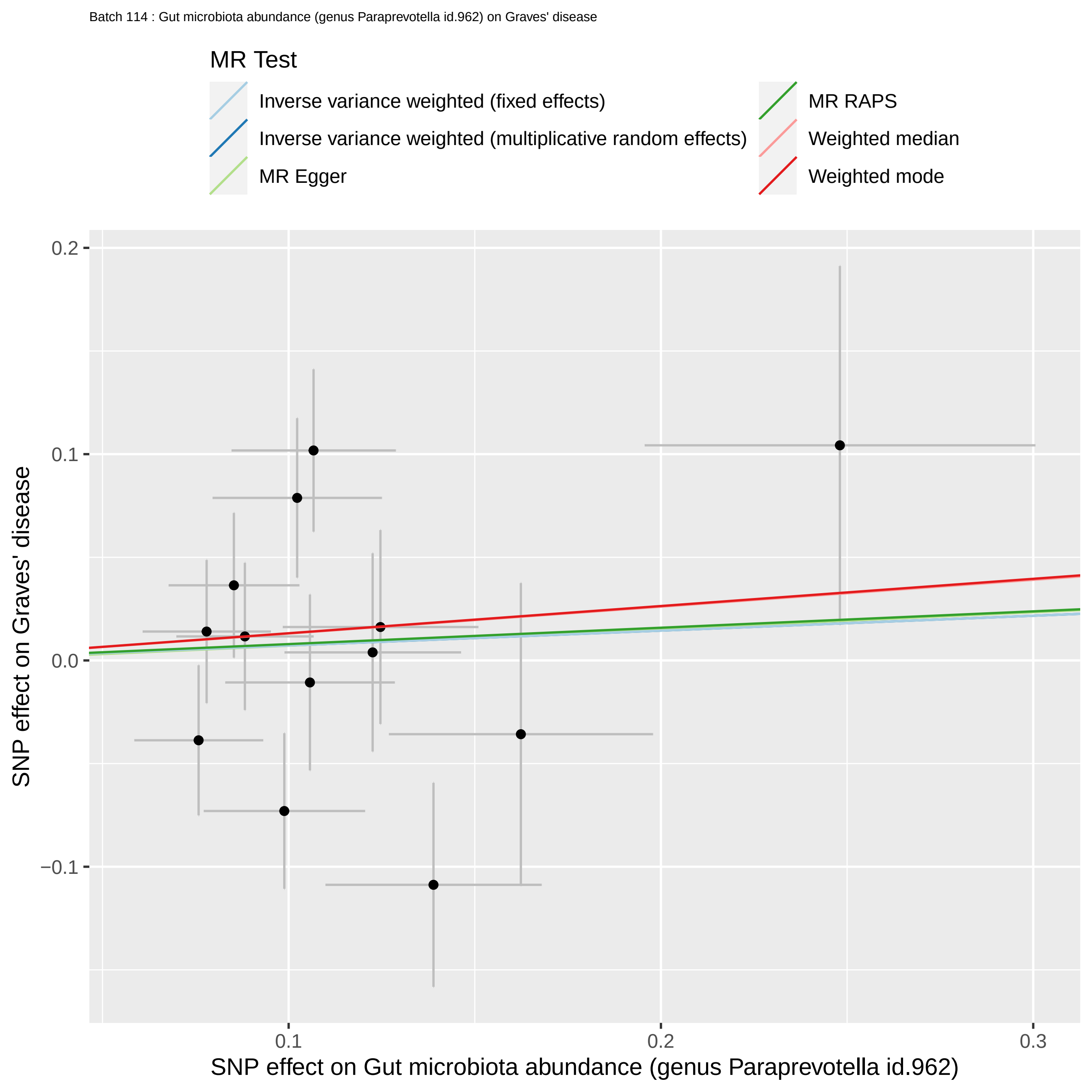

Batch 114 : Gut microbiota abundance (genus Paraprevotella id.962) on Graves' disease
MR Test
Inverse variance weighted (fixed effects)
Inverse variance weighted (multiplicative random effects)
MR Egger
MR RAPS
Weighted median
Weighted mode
0.2
0.1
0.0
−0.1
SNP effect on Graves' disease
0.1
0.2
0.3
SNP effect on Gut microbiota abundance (genus Paraprevotella id.962)

Batch 115 : Gut microbiota abundance (genus Parasutterella id.2892) on Graves' disease

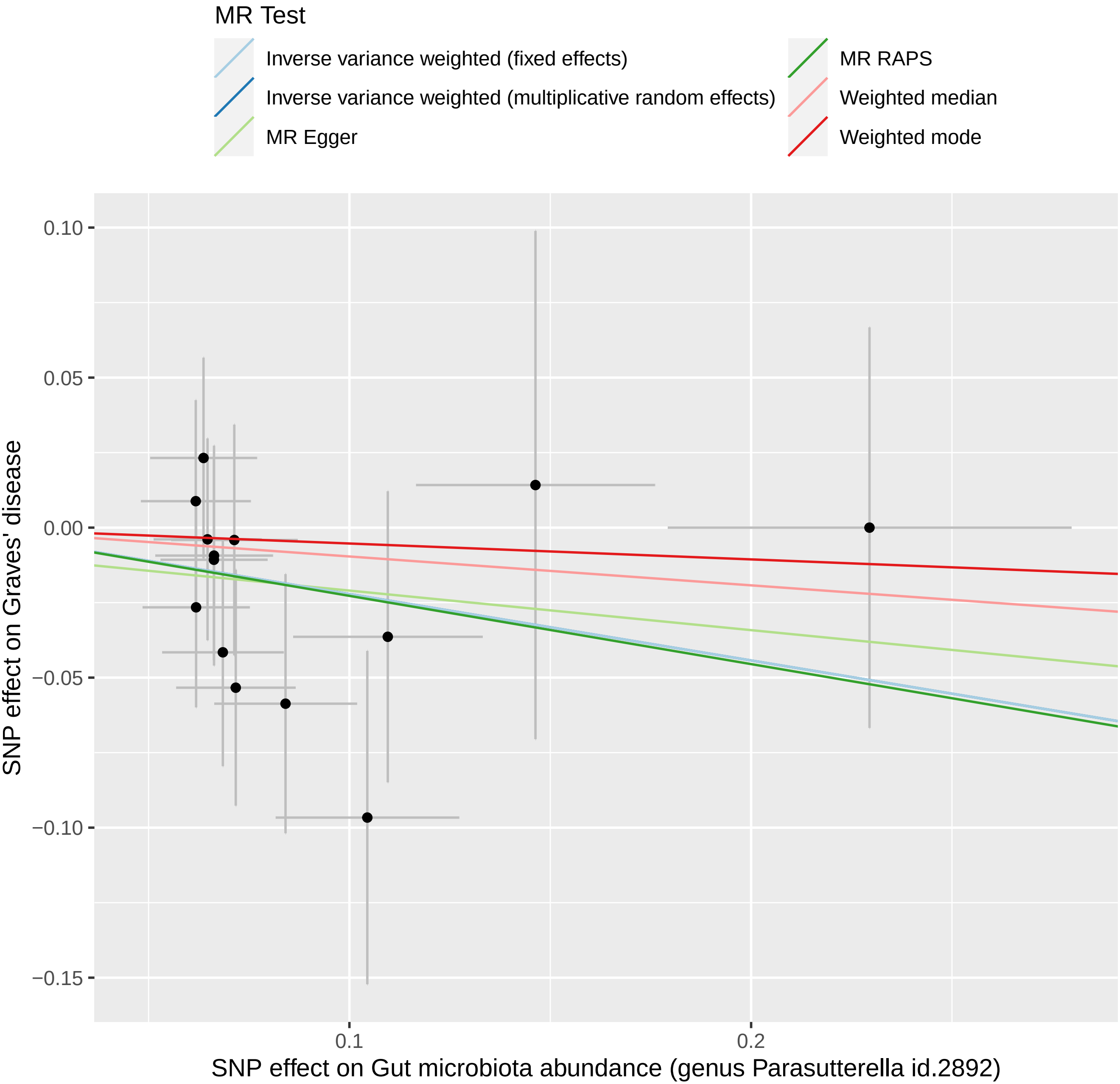

Batch 116 : Gut microbiota abundance (genus Peptococcus id.2037) on Graves' disease

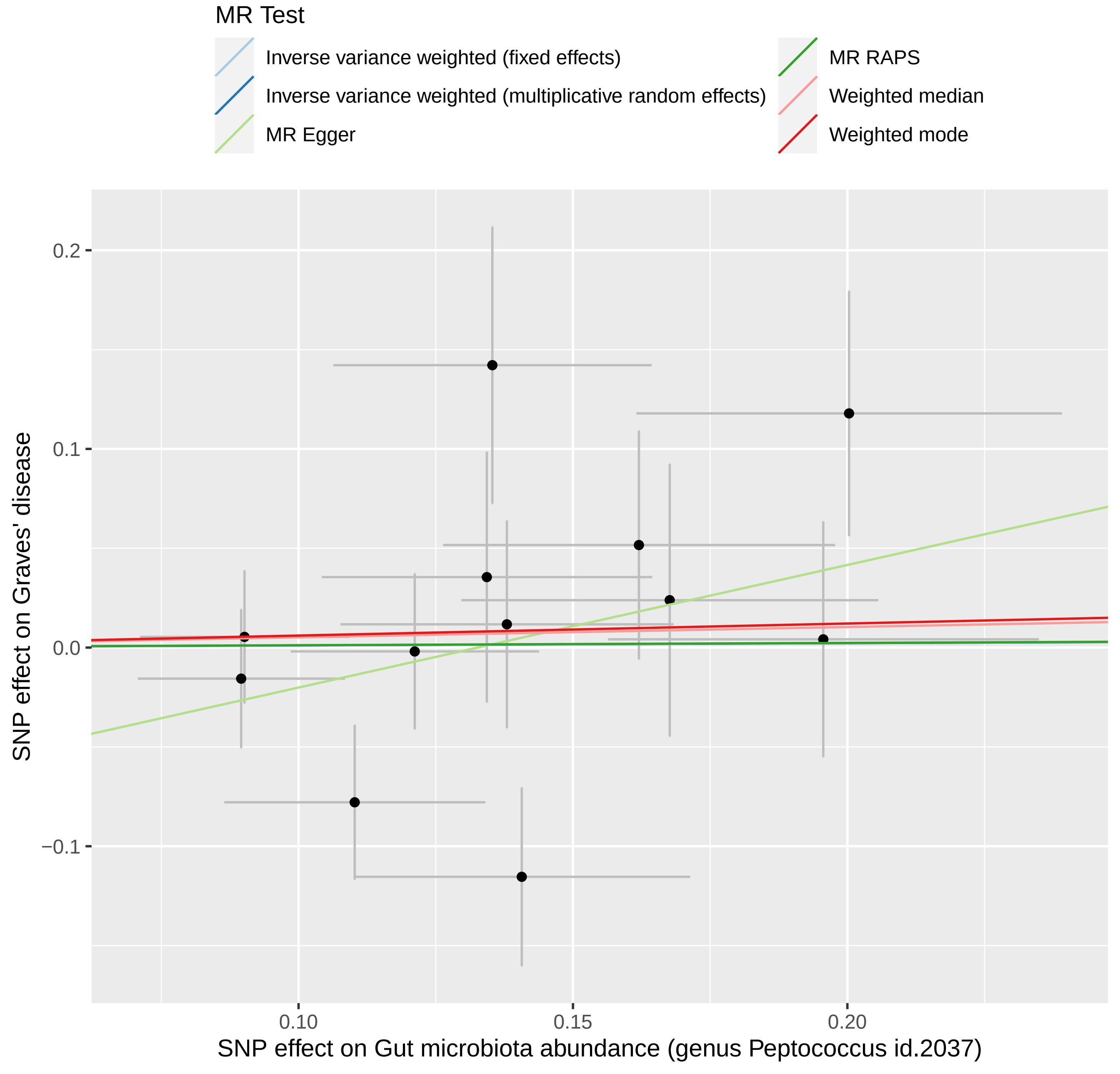

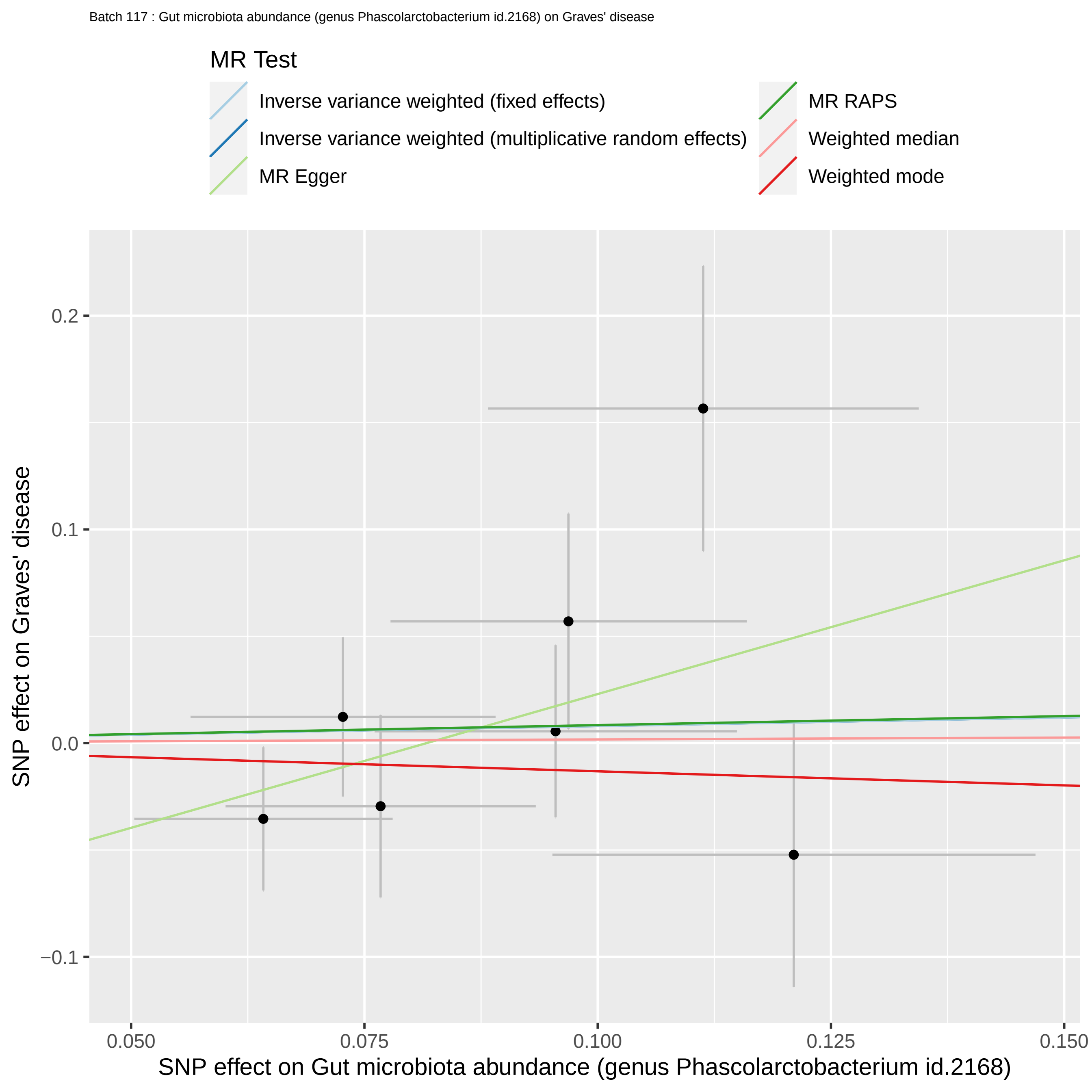

Batch 117 : Gut microbiota abundance (genus Phascolarctobacterium id.2168) on Graves' disease
MR Test
Inverse variance weighted (fixed effects)
Inverse variance weighted (multiplicative random effects)
MR Egger
MR RAPS
Weighted median
Weighted mode
0.2
0.1
0.0
−0.1
0.050
0.075
0.100
0.125
0.150
SNP effect on Graves' disease
SNP effect on Gut microbiota abundance (genus Phascolarctobacterium id.2168)

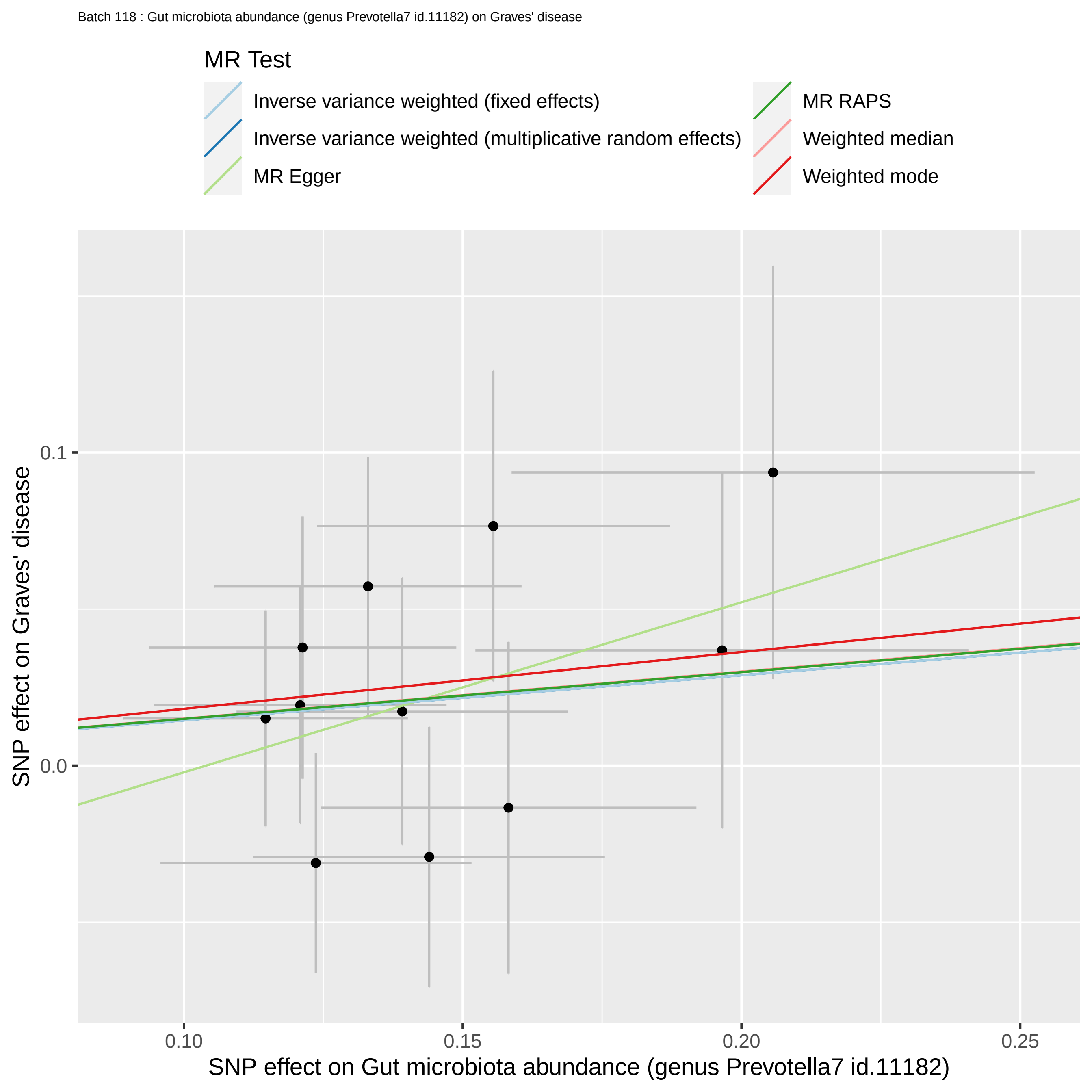

Batch 118 : Gut microbiota abundance (genus Prevotella7 id.11182) on Graves' disease
MR Test
Inverse variance weighted (fixed effects)
Inverse variance weighted (multiplicative random effects)
MR Egger
MR RAPS
Weighted median
Weighted mode
SNP effect on Graves' disease
0.1
0.0
0.10
0.15
0.20
0.25
SNP effect on Gut microbiota abundance (genus Prevotella7 id.11182)

Batch 119 : Gut microbiota abundance (genus Prevotella9 id.11183) on Graves' disease

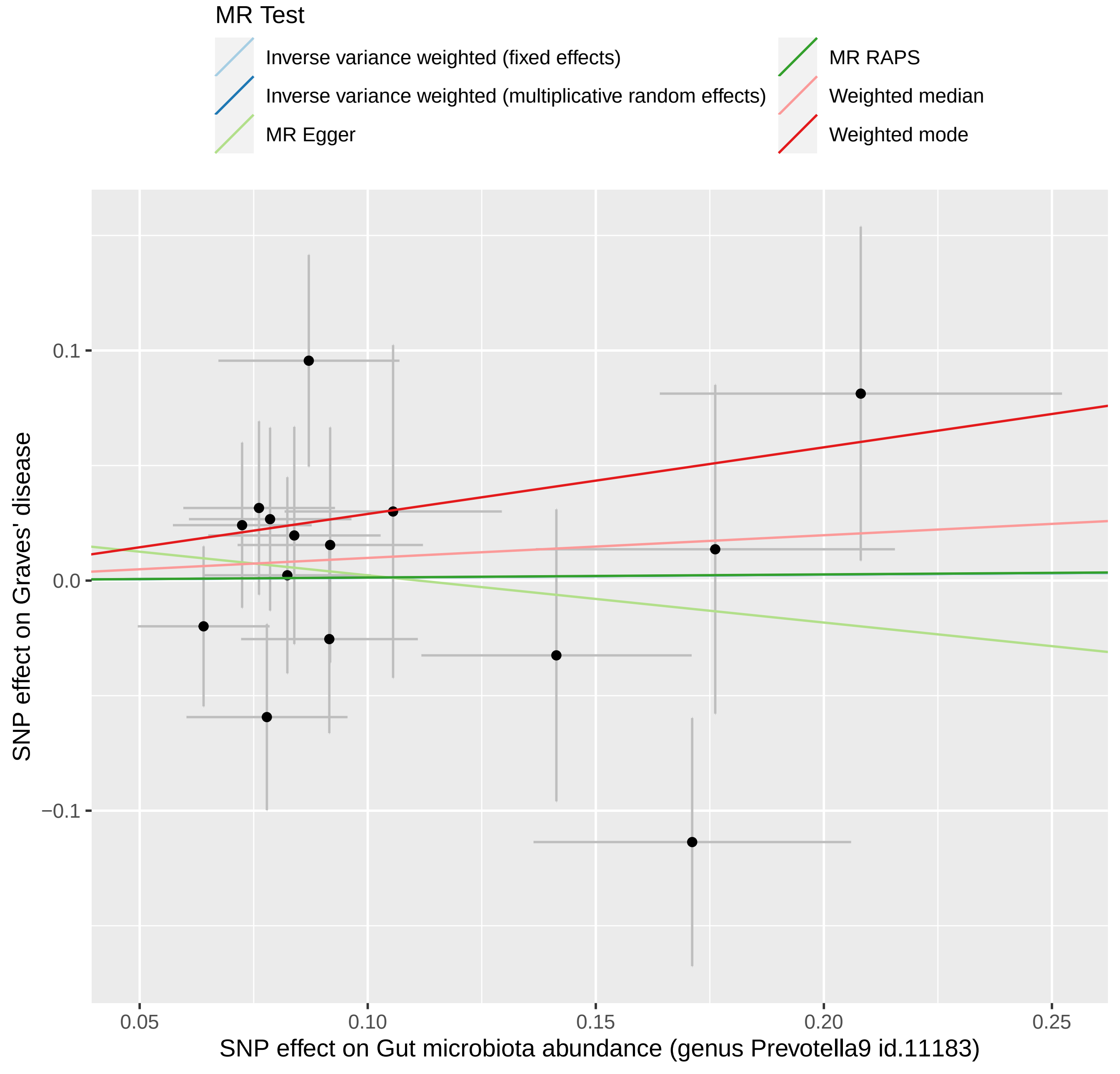

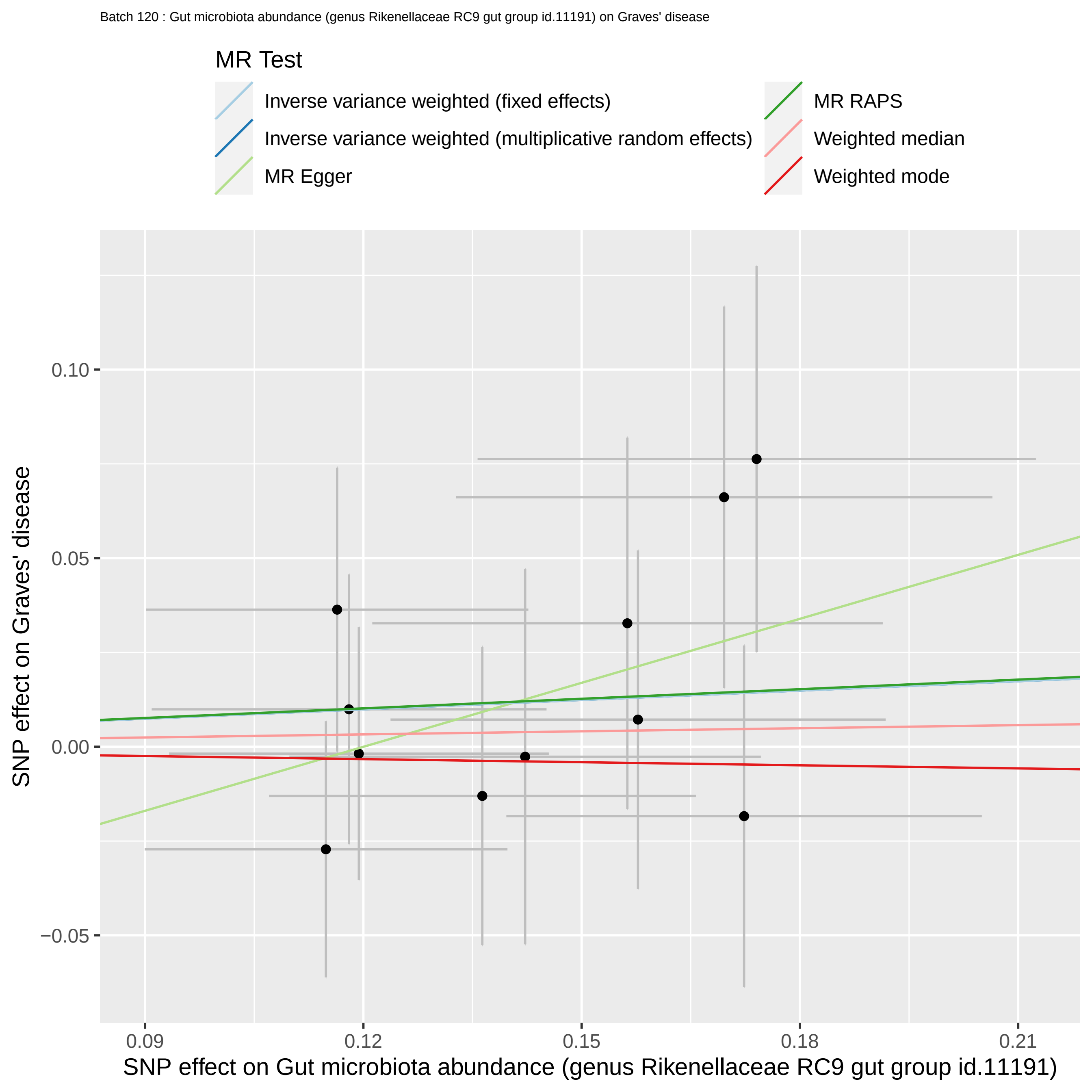
Batch 120 : Gut microbiota abundance (genus Rikenellaceae RC9 gut group id.11191) on Graves' disease
MR Test
Inverse variance weighted (fixed effects)
Inverse variance weighted (multiplicative random effects)
MR Egger
MR RAPS
Weighted median
Weighted mode
0.10
0.05
0.00
−0.05
0.09
0.12
0.15
0.18
0.21
SNP effect on Graves' disease
SNP effect on Gut microbiota abundance (genus Rikenellaceae RC9 gut group id.11191)

Batch 121 : Gut microbiota abundance (genus Romboutsia id.11347) on Graves' disease

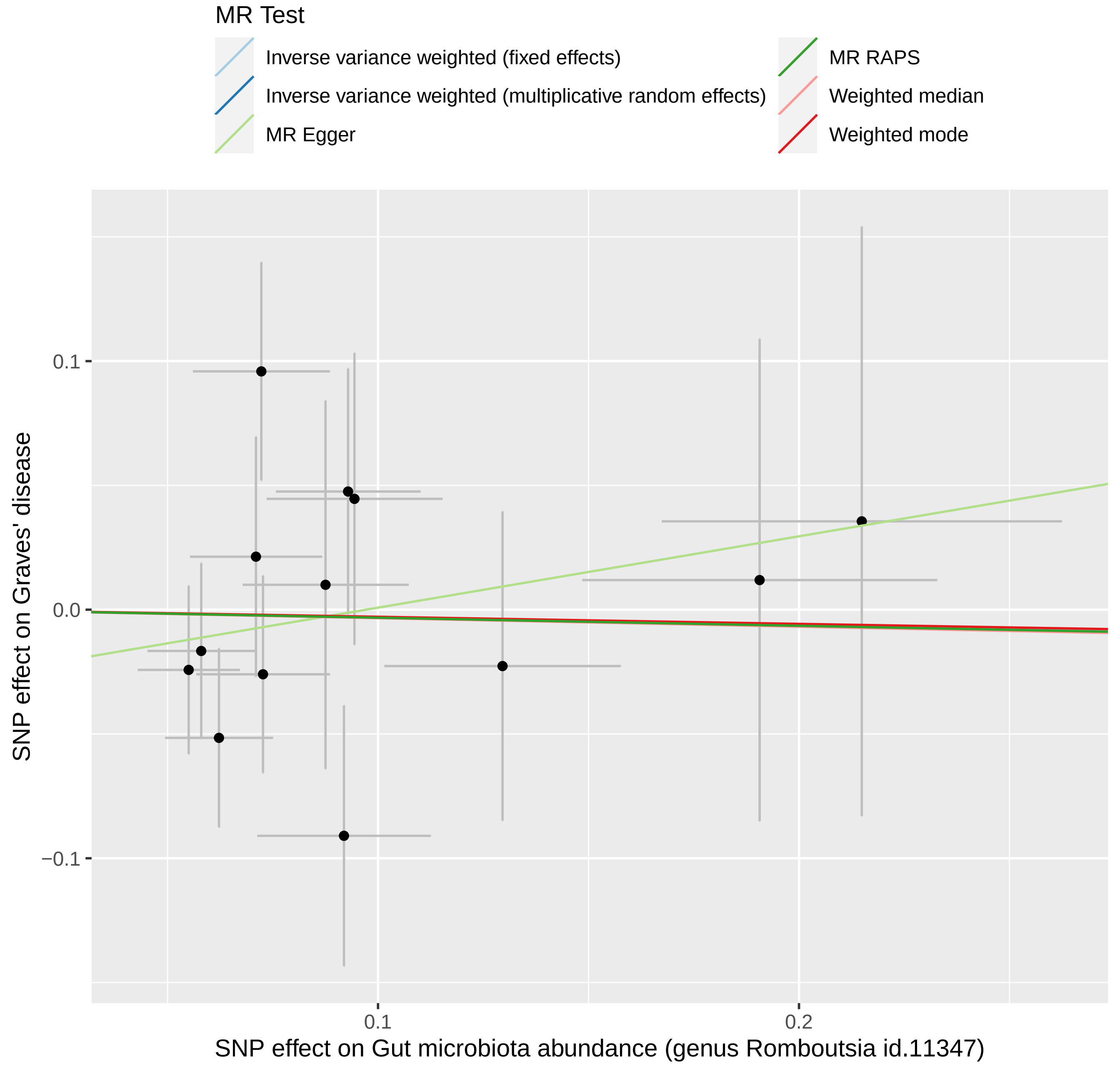

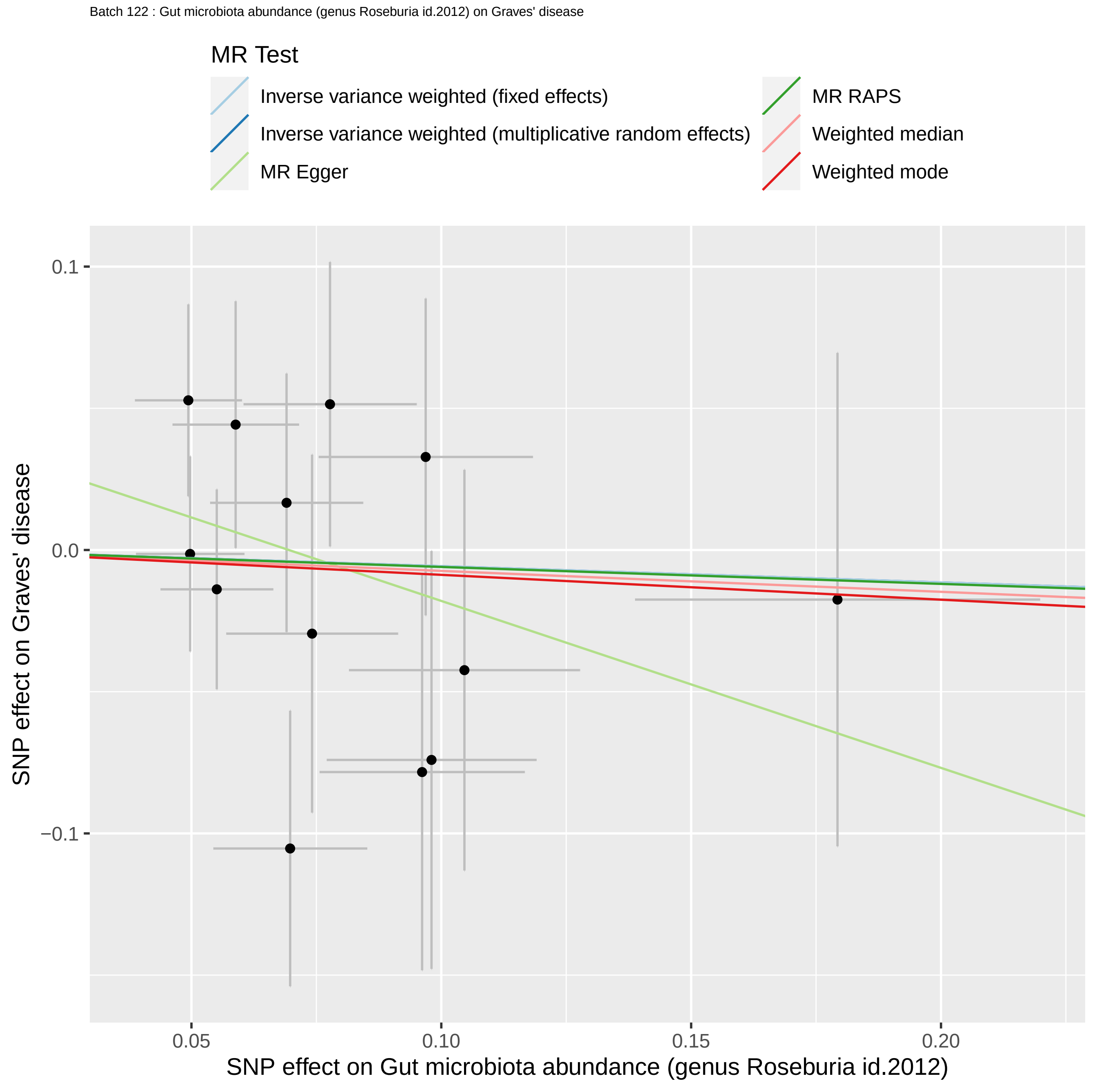

Batch 122 : Gut microbiota abundance (genus Roseburia id.2012) on Graves' disease
MR Test
Inverse variance weighted (fixed effects)
Inverse variance weighted (multiplicative random effects)
MR Egger
MR RAPS
Weighted median
Weighted mode
0.1
0.0
−0.1
0.05
0.10
0.15
0.20
SNP effect on Graves' disease
SNP effect on Gut microbiota abundance (genus Roseburia id.2012)

Batch 123 : Gut microbiota abundance (genus Ruminiclostridium5 id.11355) on Graves' disease

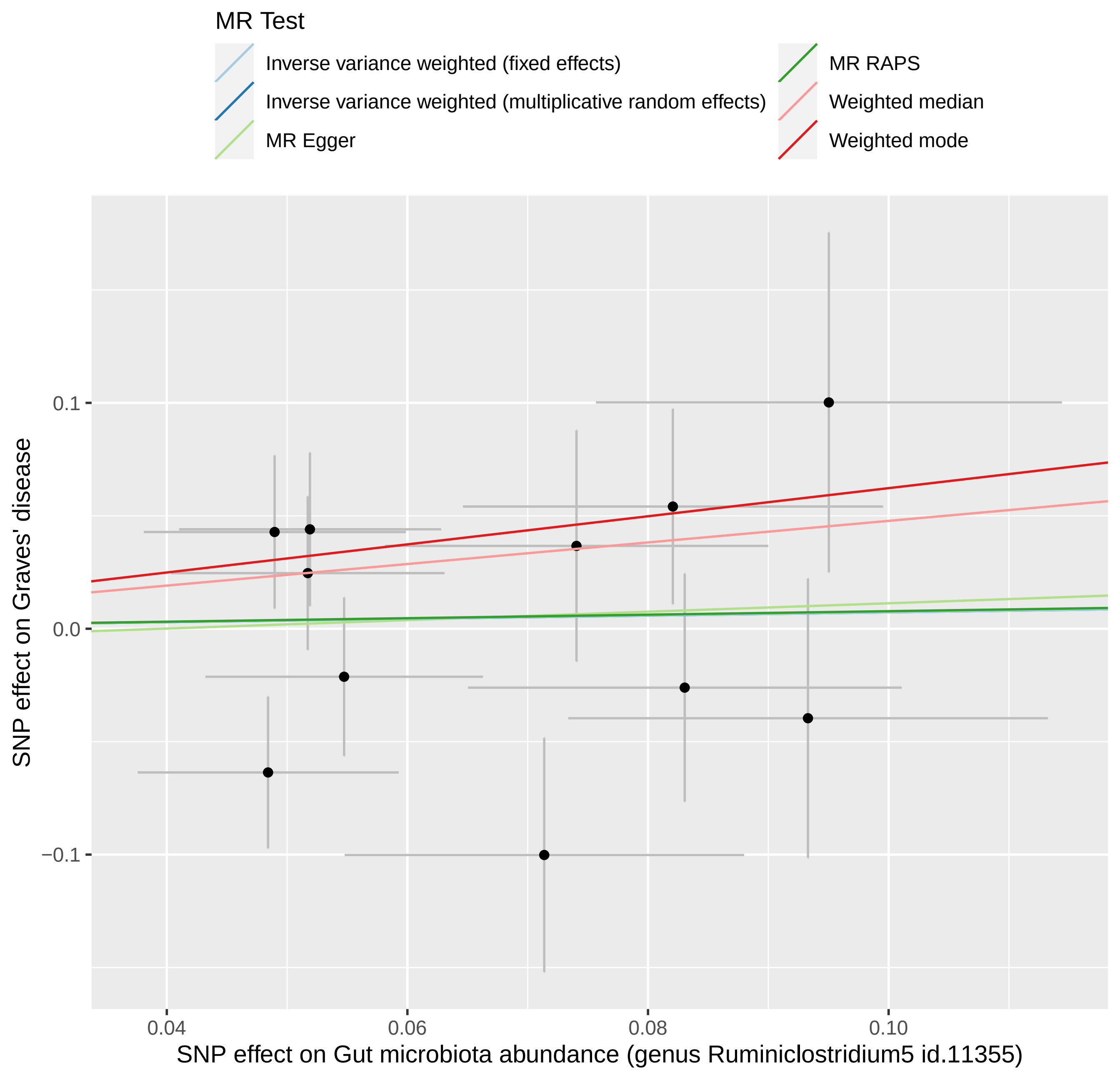

Batch 124 : Gut microbiota abundance (genus Ruminiclostridium6 id.11356) on Graves' disease

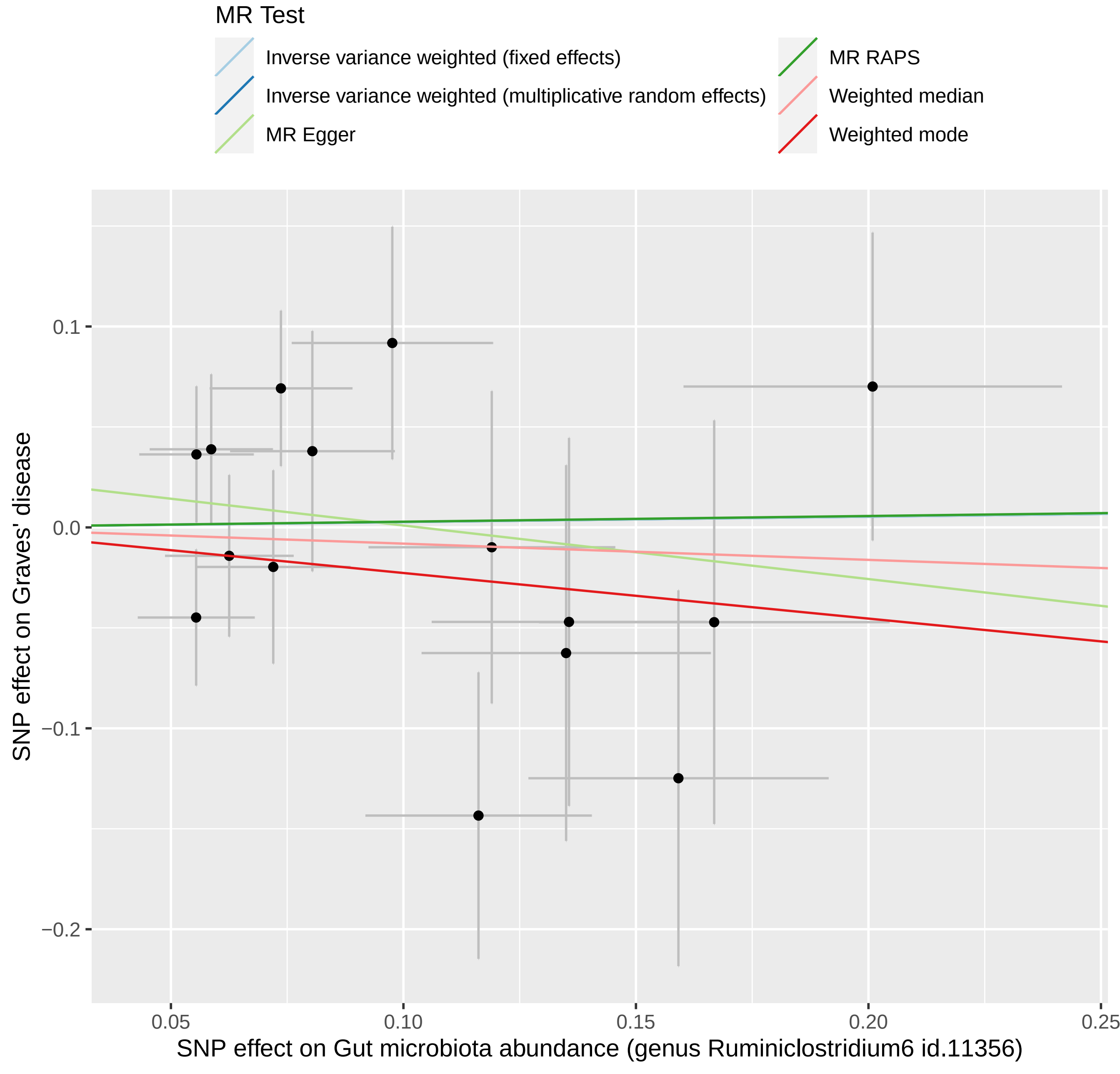

Batch 125 : Gut microbiota abundance (genus Ruminiclostridium9 id.11357) on Graves' disease

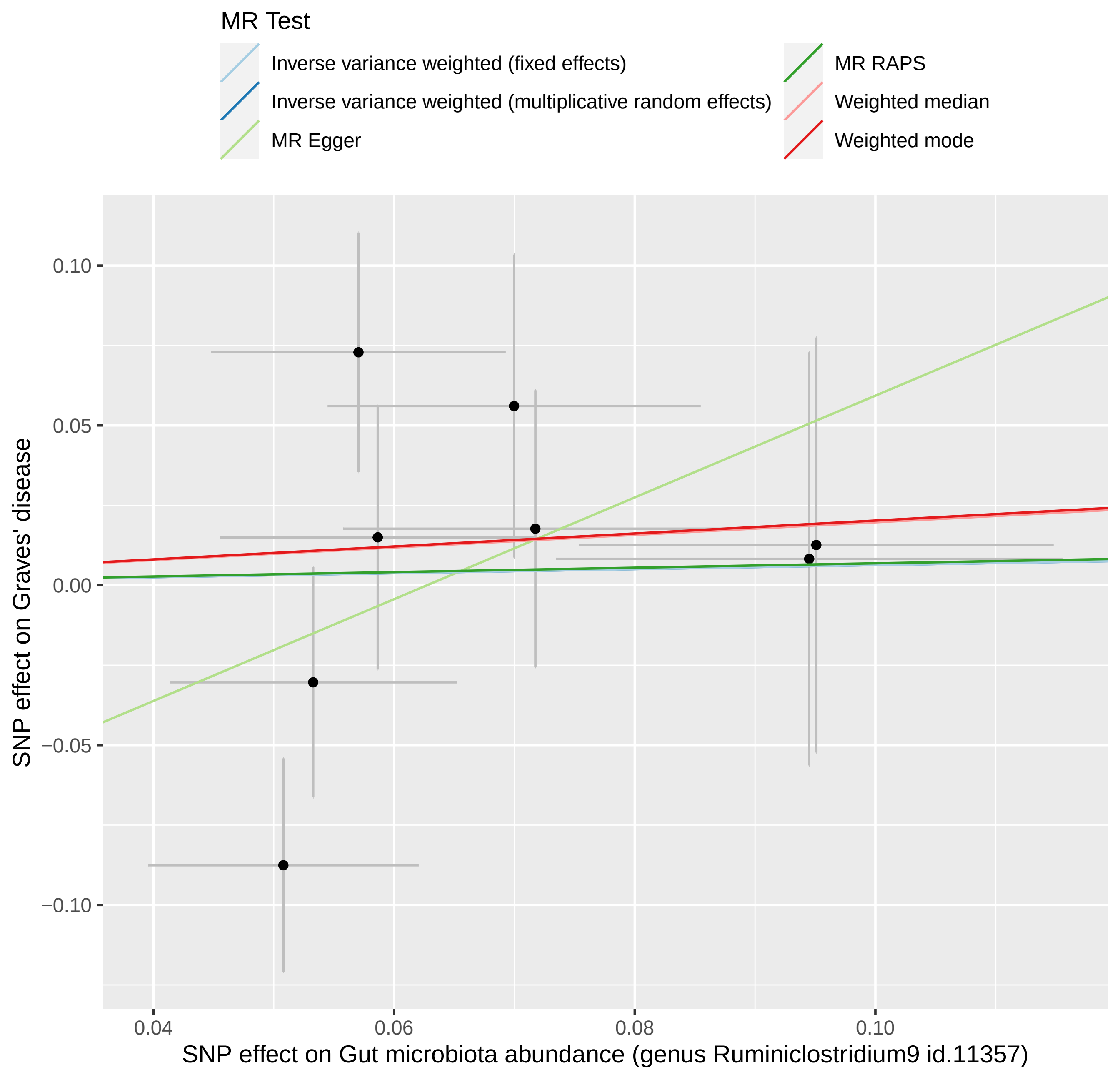

Batch 126 : Gut microbiota abundance (genus Ruminococcaceae NK4A214 group id.11358) on Graves' disease

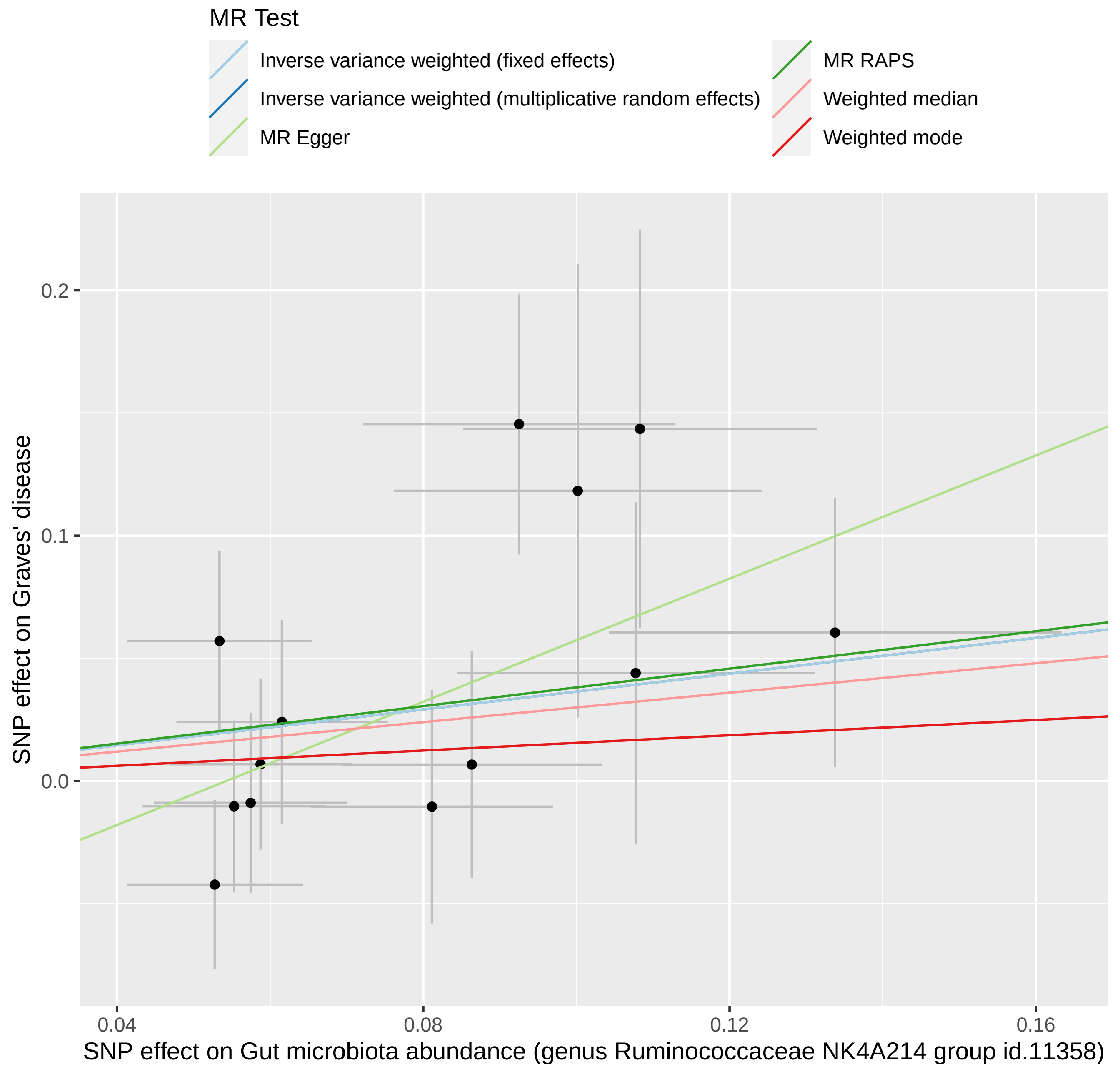

Batch 127 : Gut microbiota abundance (genus Ruminococcaceae UCG002 id.11360) on Graves' disease

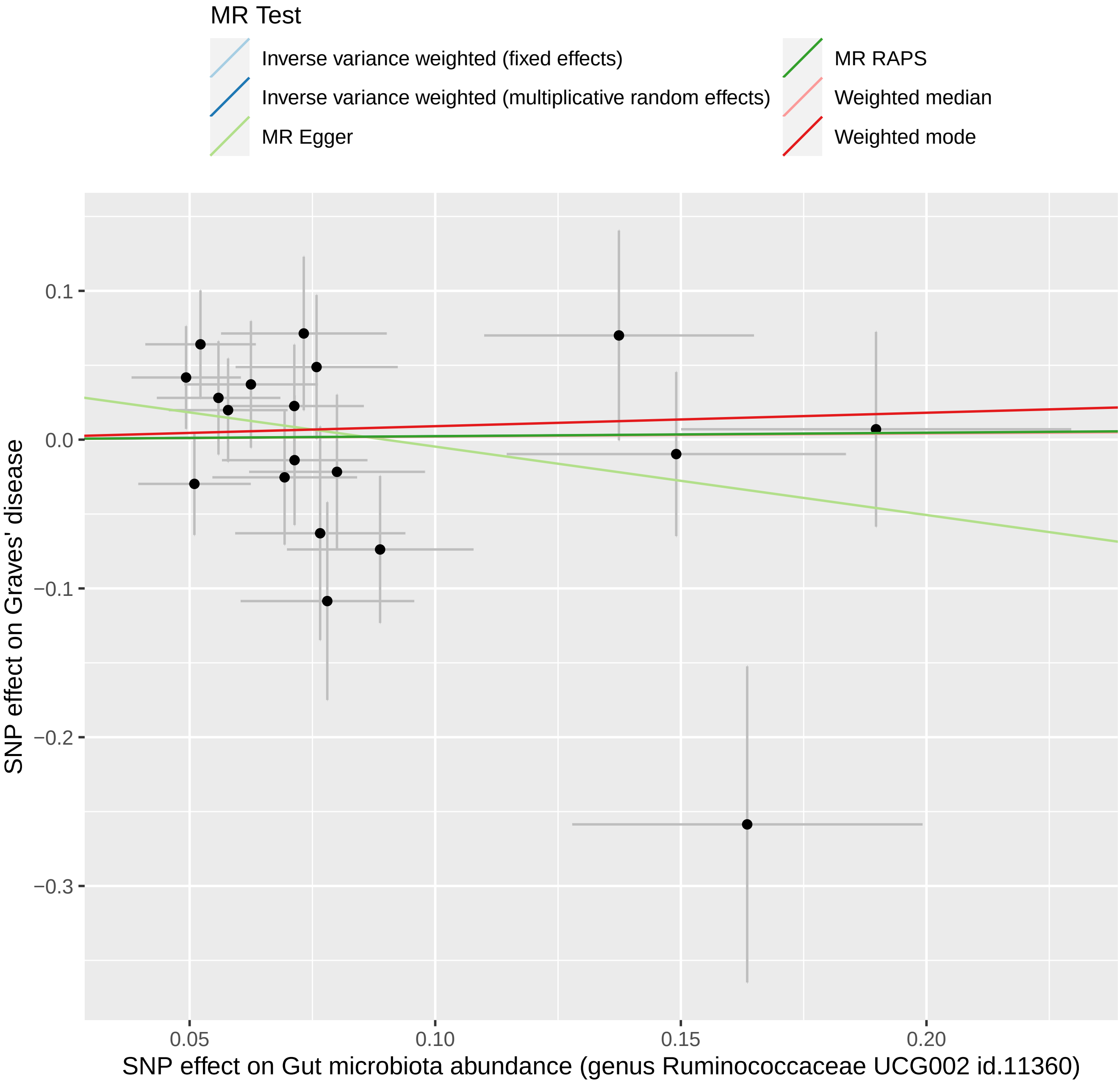

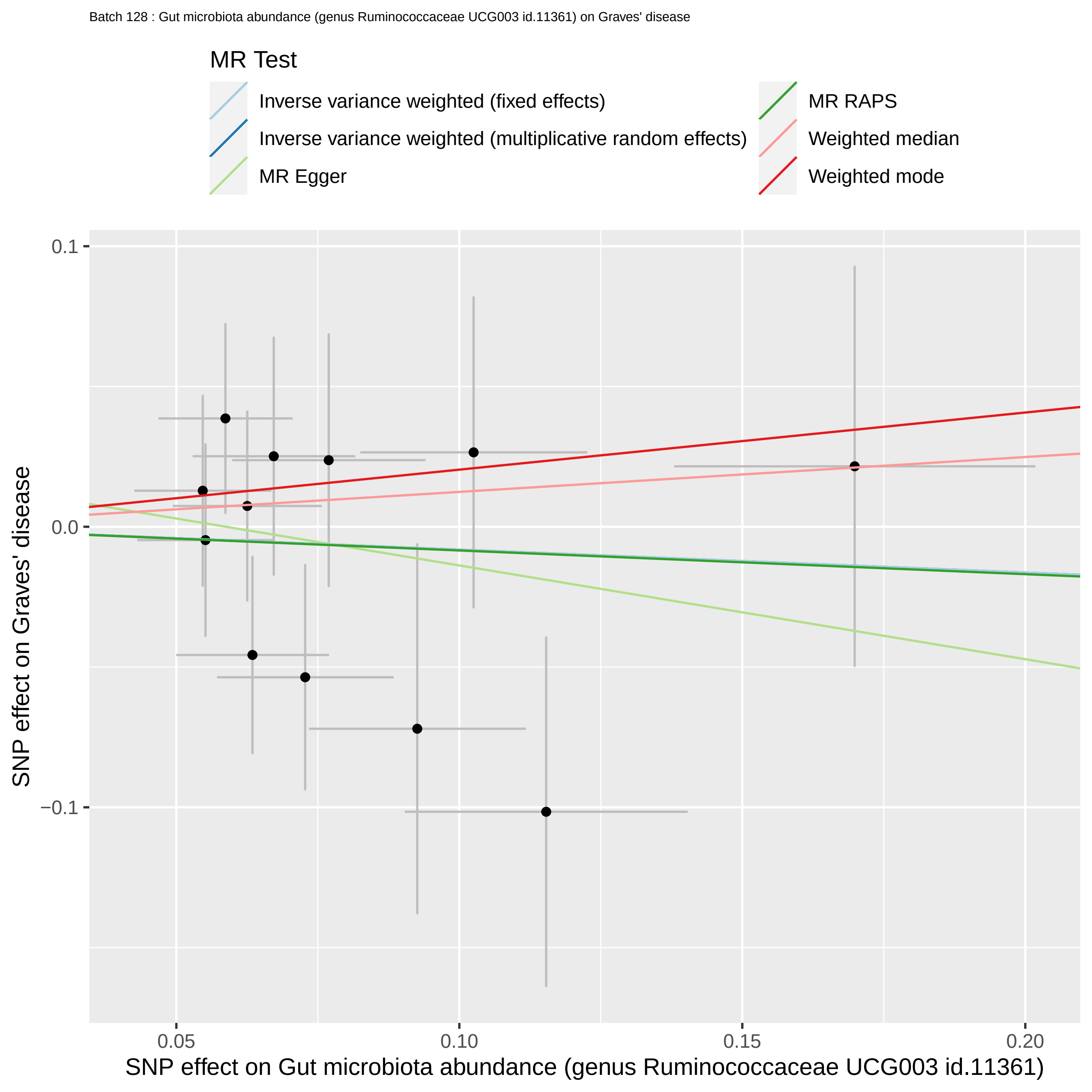
Batch 128 : Gut microbiota abundance (genus Ruminococcaceae UCG003 id.11361) on Graves' disease
MR Test
Inverse variance weighted (fixed effects)
Inverse variance weighted (multiplicative random effects)
MR Egger
MR RAPS
Weighted median
Weighted mode
0.1
0.0
−0.1
0.05
0.10
0.15
0.20
SNP effect on Graves' disease
SNP effect on Gut microbiota abundance (genus Ruminococcaceae UCG003 id.11361)

Batch 129 : Gut microbiota abundance (genus Ruminococcaceae UCG004 id.11362) on Graves' disease

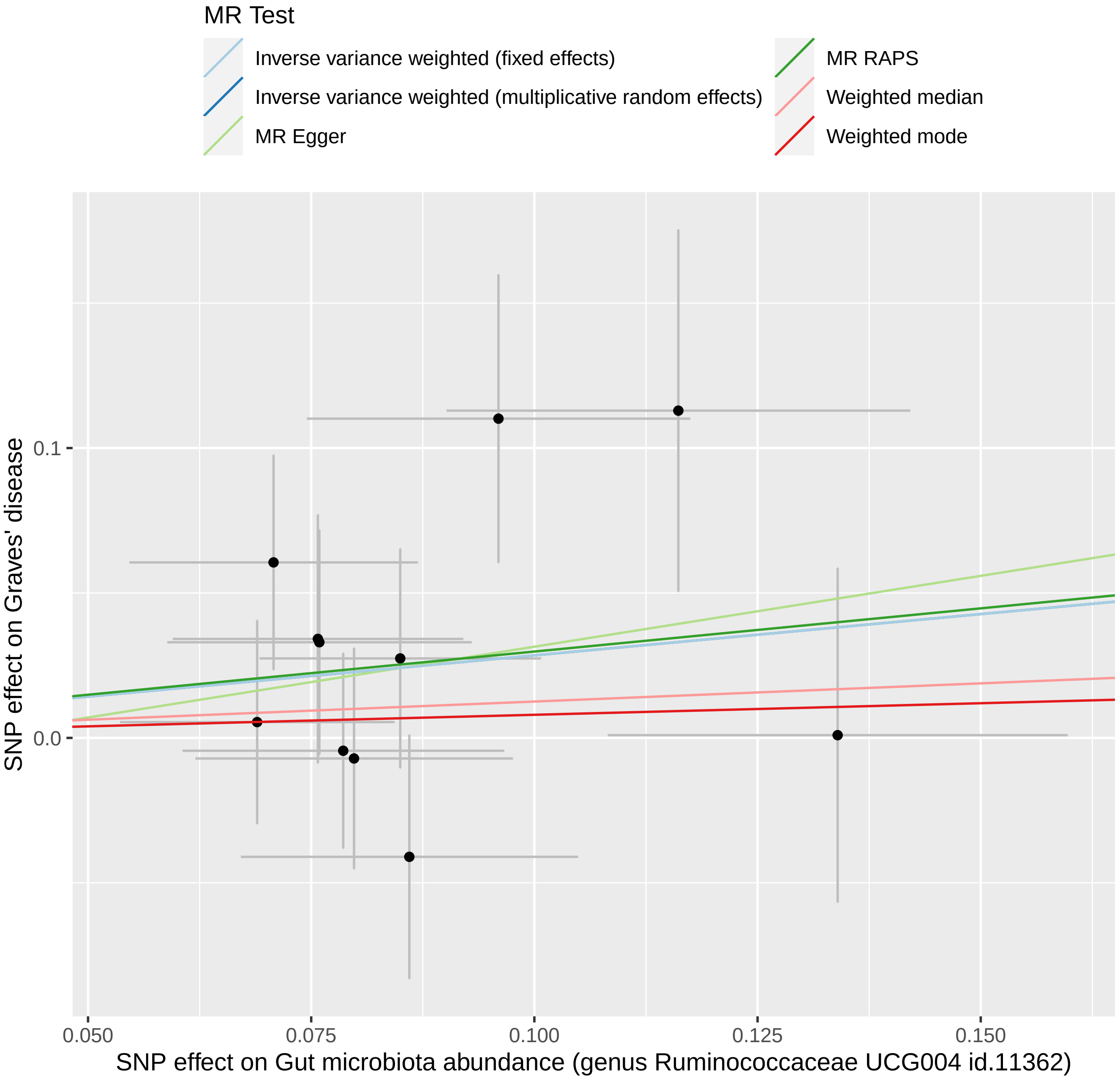

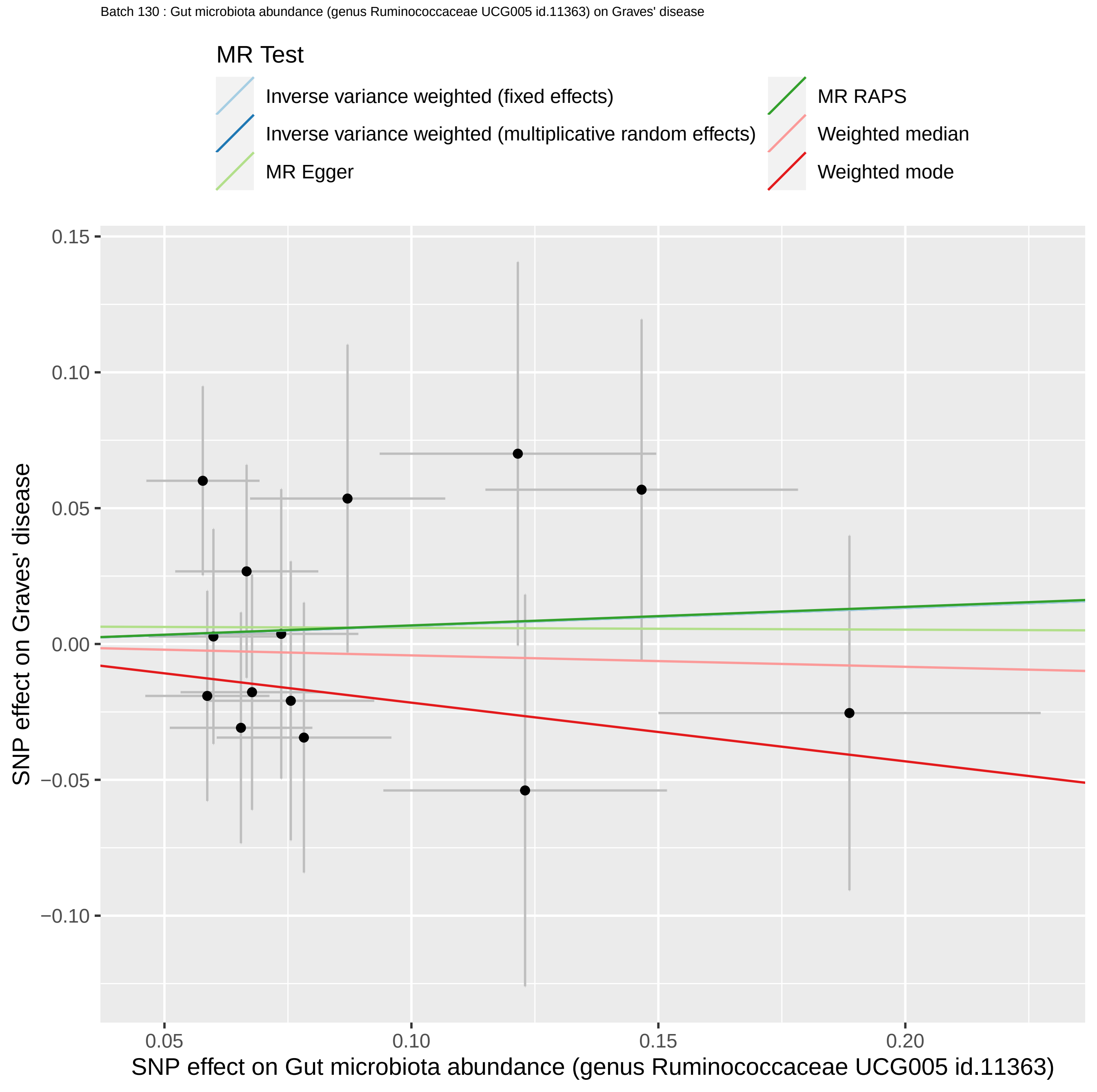

Batch 130 : Gut microbiota abundance (genus Ruminococcaceae UCG005 id.11363) on Graves' disease
MR Test
Inverse variance weighted (fixed effects)
Inverse variance weighted (multiplicative random effects)
MR Egger
MR RAPS
Weighted median
Weighted mode
0.15
0.10
0.05
0.00
−0.05
−0.10
0.05
0.10
0.15
0.20
SNP effect on Graves' disease
SNP effect on Gut microbiota abundance (genus Ruminococcaceae UCG005 id.11363)

Batch 131 : Gut microbiota abundance (genus Ruminococcaceae UCG009 id.11366) on Graves' disease

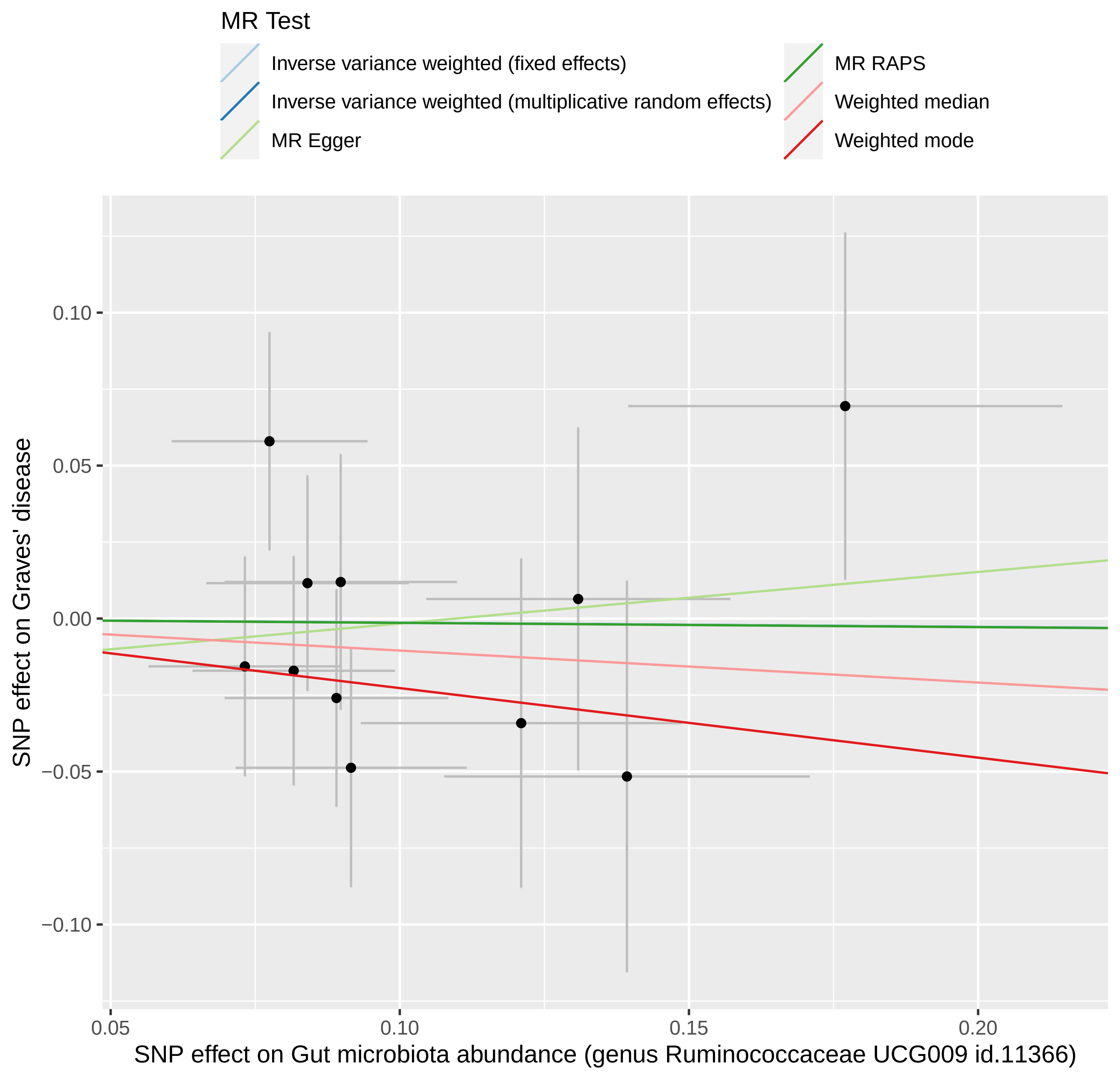

Batch 132 : Gut microbiota abundance (genus Ruminococcaceae UCG010 id.11367) on Graves' disease

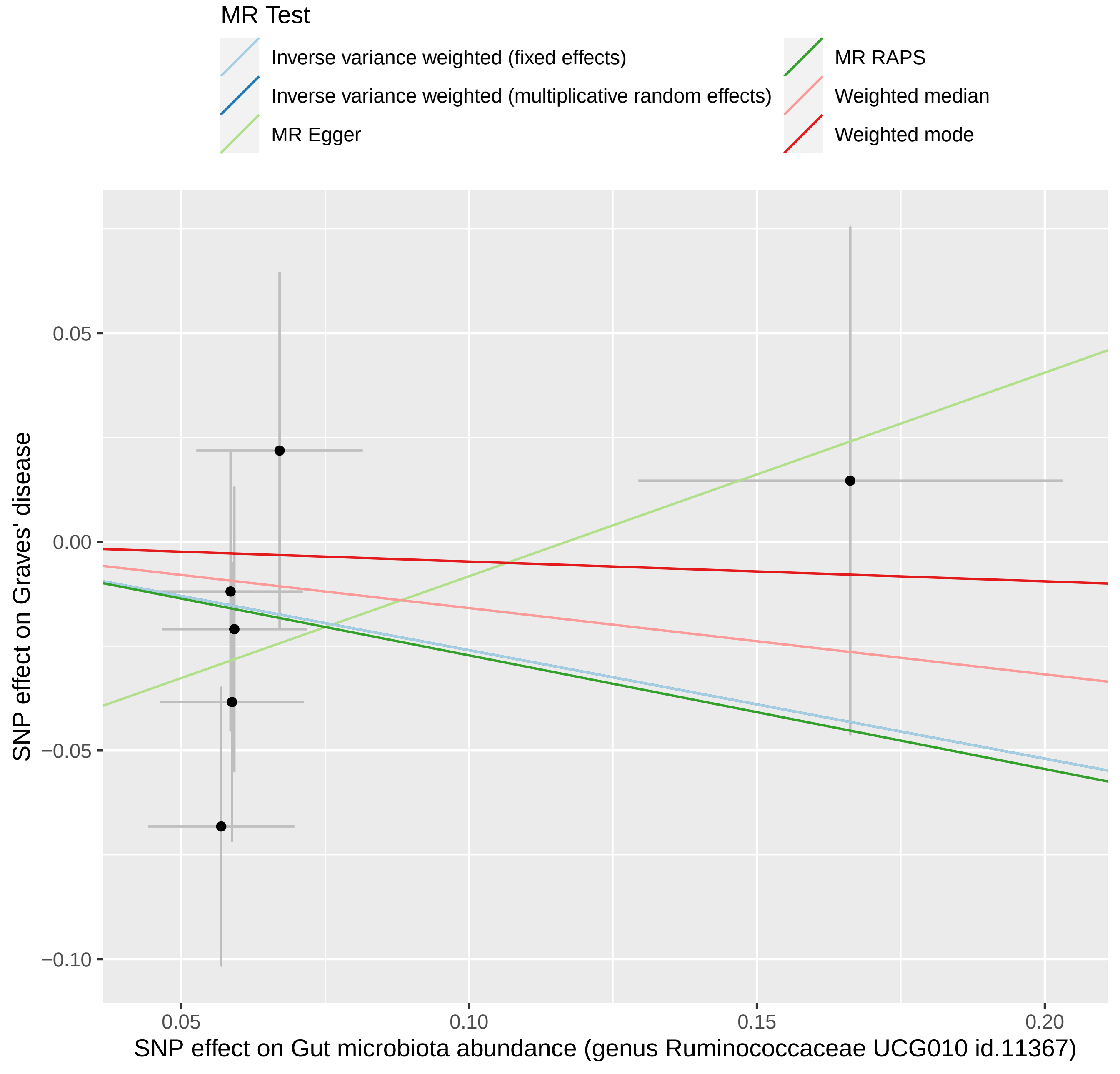

Batch 133 : Gut microbiota abundance (genus Ruminococcaceae UCG011 id.11368) on Graves' disease

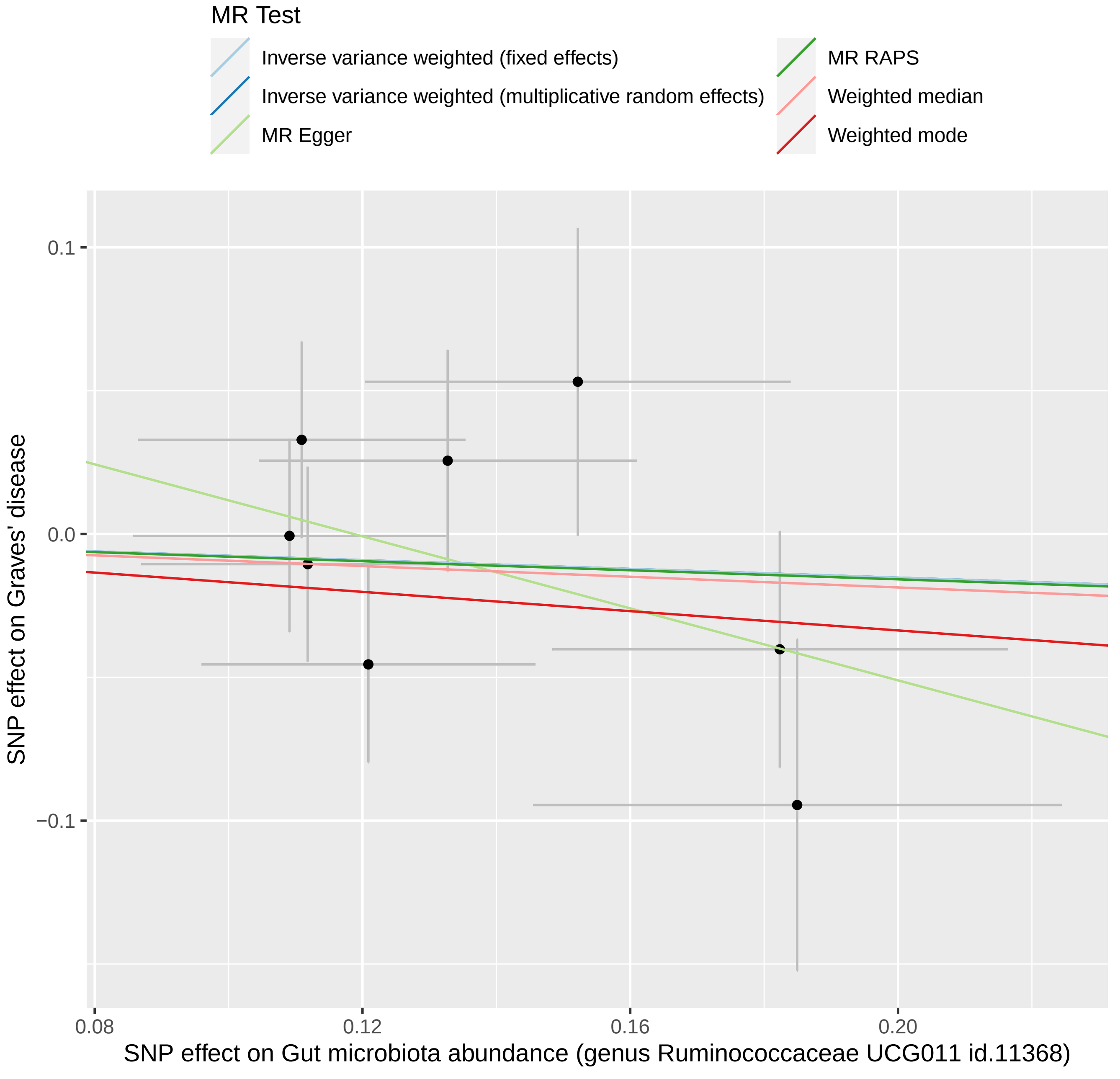

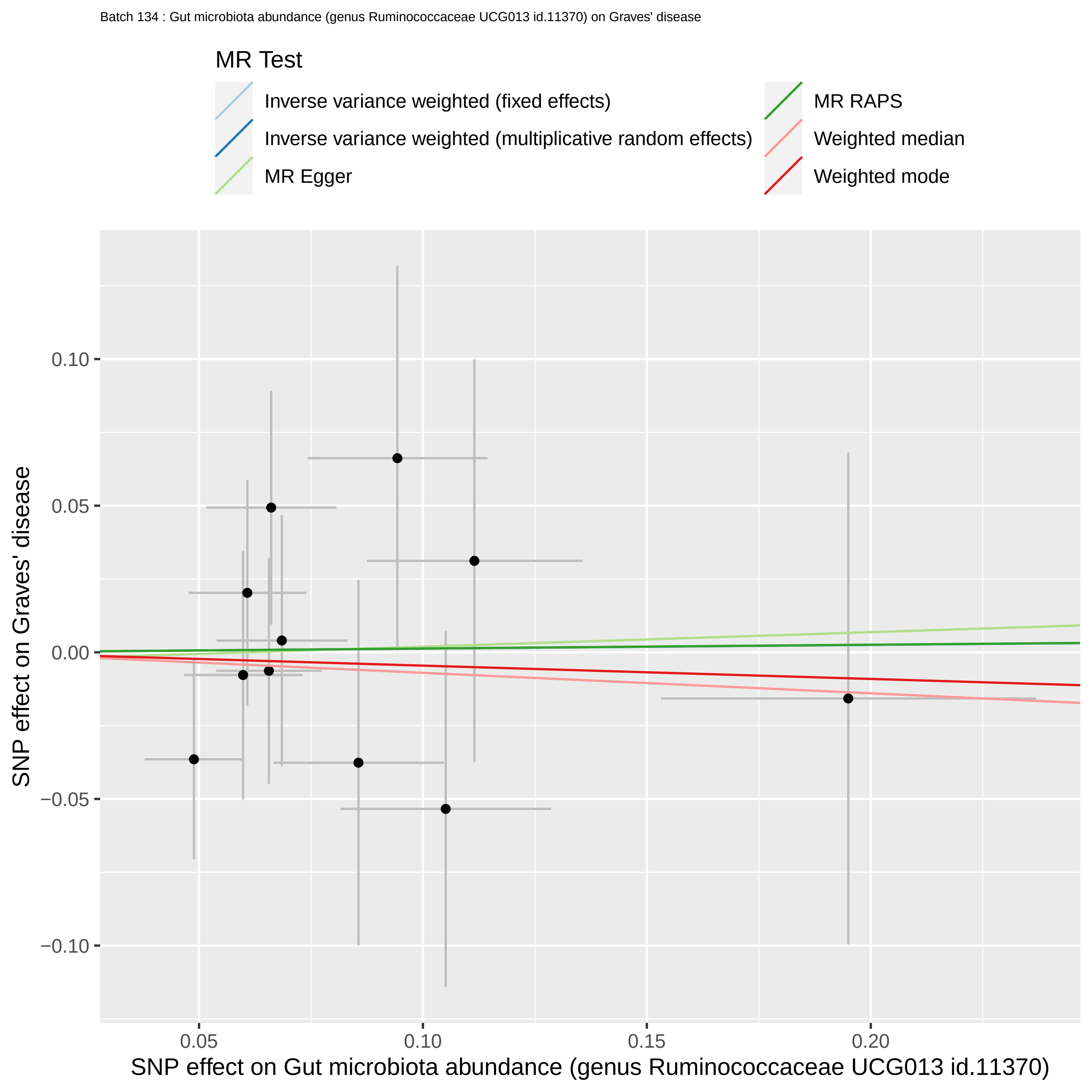

Batch 134 : Gut microbiota abundance (genus Ruminococcaceae UCG013 id.11370) on Graves' disease
MR Test
Inverse variance weighted (fixed effects)
Inverse variance weighted (multiplicative random effects)
MR Egger
MR RAPS
Weighted median
Weighted mode
SNP effect on Graves' disease
0.10
0.05
0.00
−0.05
−0.10
0.05
0.10
0.15
0.20
SNP effect on Gut microbiota abundance (genus Ruminococcaceae UCG013 id.11370)

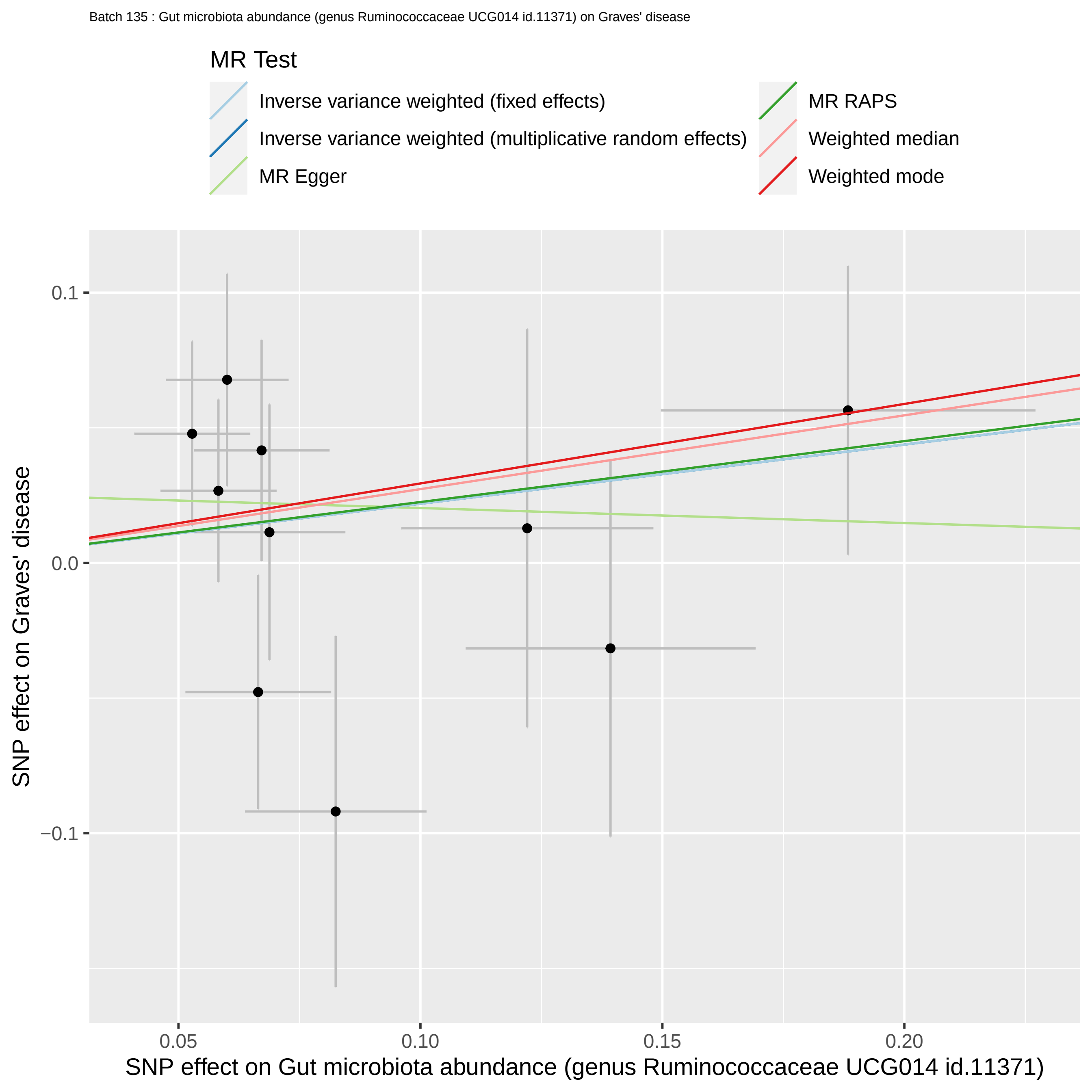
Batch 135 : Gut microbiota abundance (genus Ruminococcaceae UCG014 id.11371) on Graves' disease
MR Test
Inverse variance weighted (fixed effects)
Inverse variance weighted (multiplicative random effects)
MR Egger
MR RAPS
Weighted median
Weighted mode
0.1
0.0
−0.1
0.05
0.10
0.15
0.20
SNP effect on Graves' disease
SNP effect on Gut microbiota abundance (genus Ruminococcaceae UCG014 id.11371)

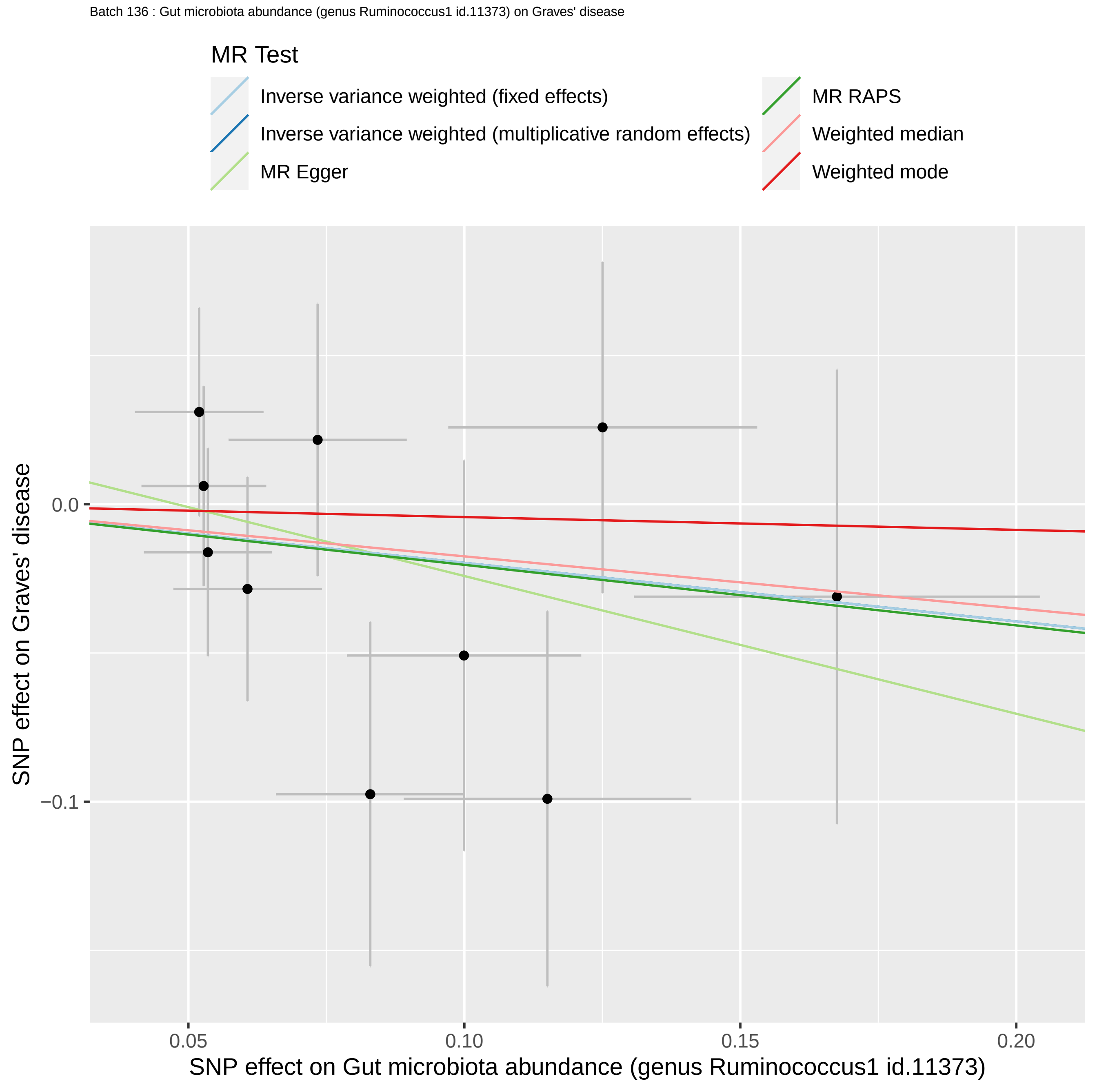
Batch 136 : Gut microbiota abundance (genus Ruminococcus1 id.11373) on Graves' disease
MR Test
Inverse variance weighted (fixed effects)
Inverse variance weighted (multiplicative random effects)
MR Egger
MR RAPS
Weighted median
Weighted mode
0.0
−0.1
0.05
0.10
0.15
0.20
SNP effect on Graves' disease
SNP effect on Gut microbiota abundance (genus Ruminococcus1 id.11373)

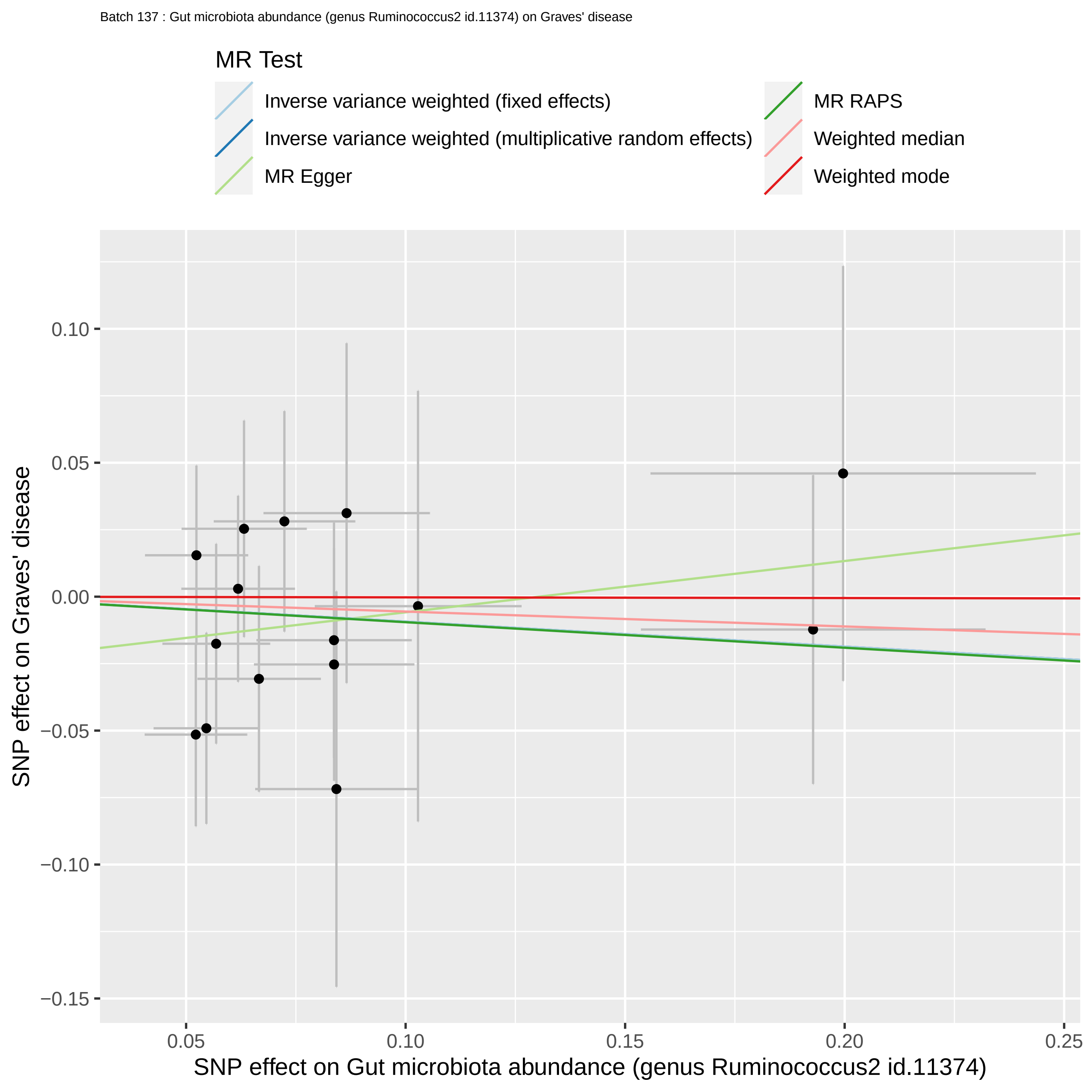

Batch 137 : Gut microbiota abundance (genus Ruminococcus2 id.11374) on Graves' disease
MR Test
Inverse variance weighted (fixed effects)
Inverse variance weighted (multiplicative random effects)
MR Egger
MR RAPS
Weighted median
Weighted mode
SNP effect on Graves' disease
0.10
0.05
0.00
−0.05
−0.10
−0.15
0.05
0.10
0.15
0.20
0.25
SNP effect on Gut microbiota abundance (genus Ruminococcus2 id.11374)

Batch 138 : Gut microbiota abundance (genus Ruminococcus gauvreauii group id.11342) on Graves' disease

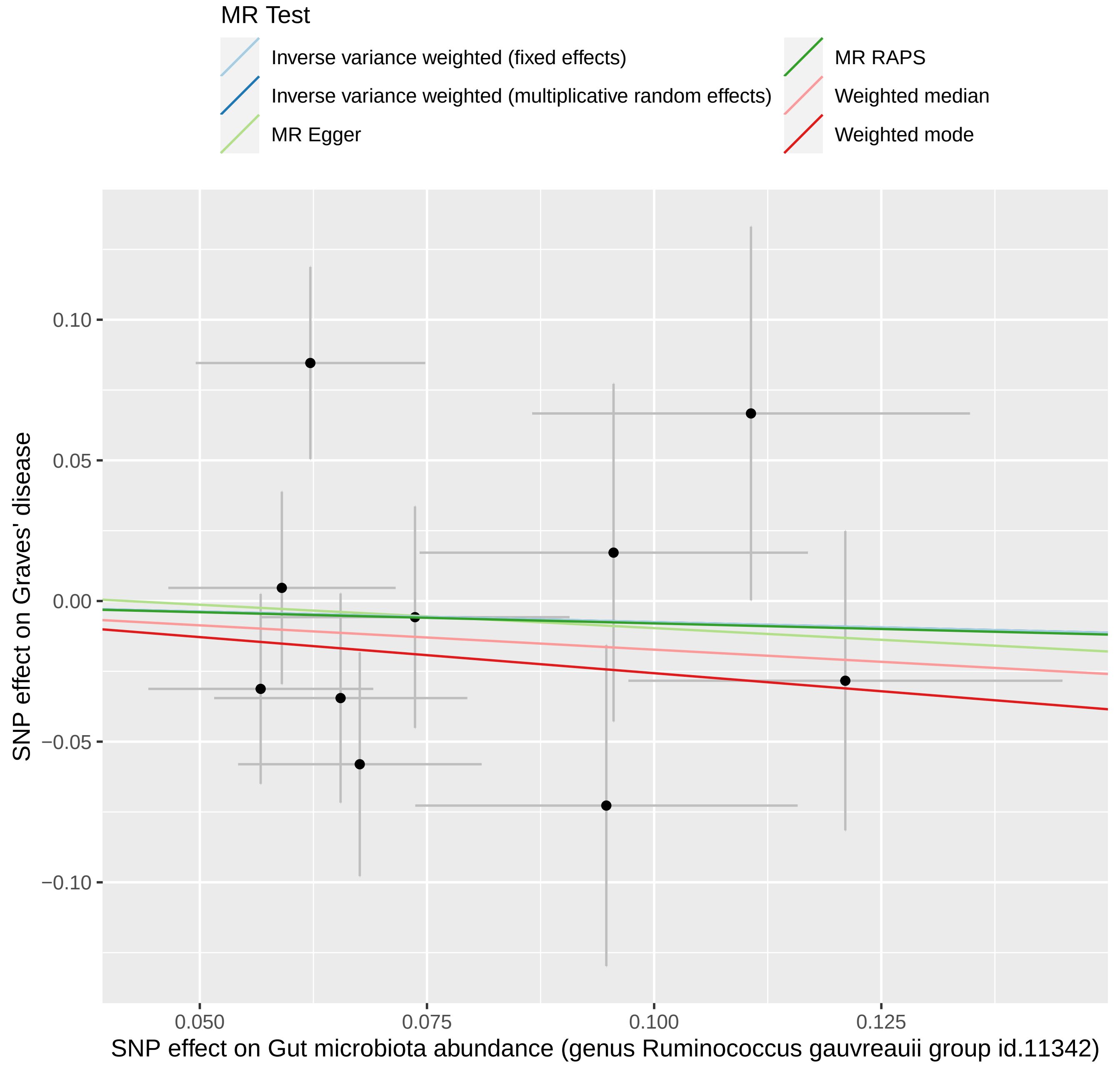

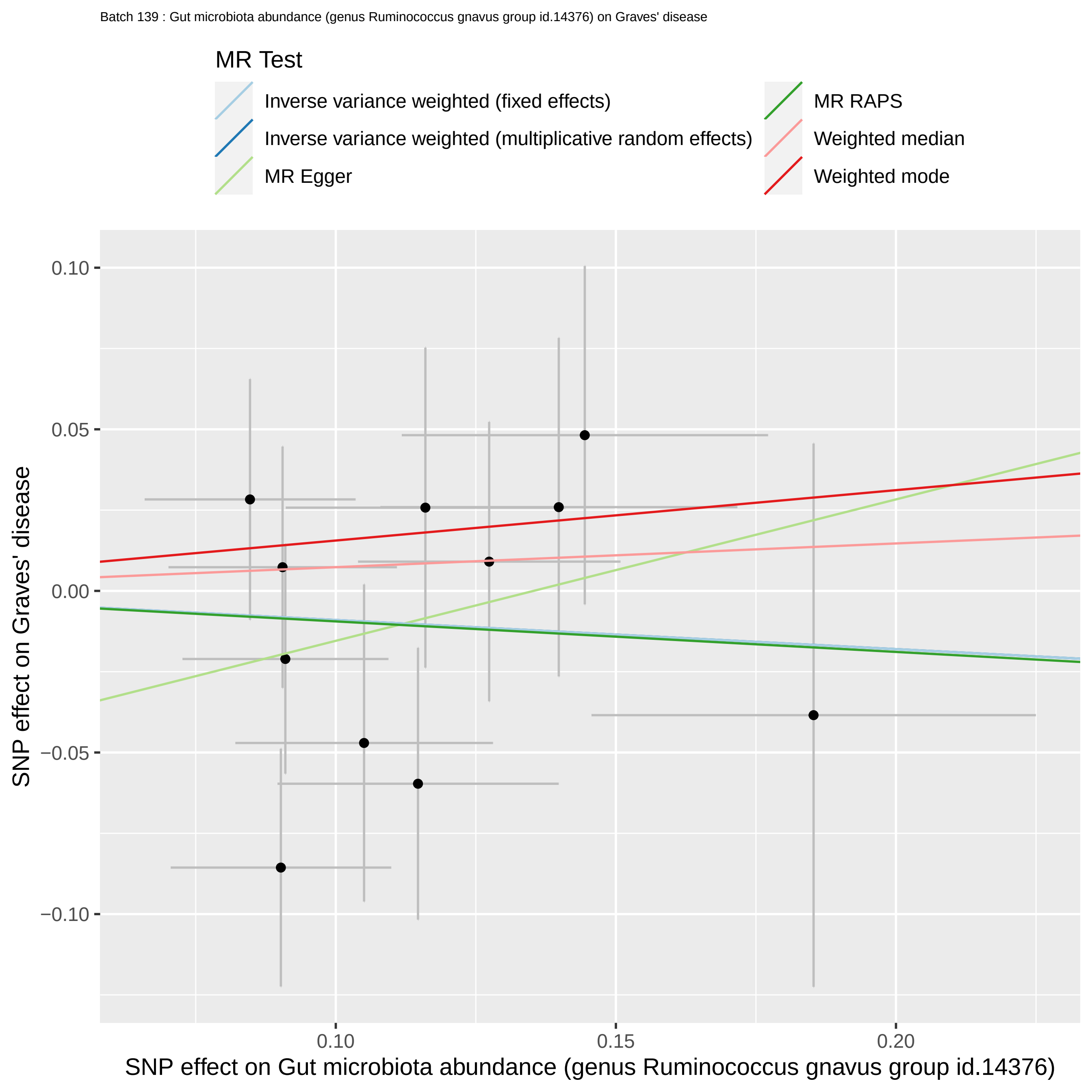

Batch 139 : Gut microbiota abundance (genus Ruminococcus gnavus group id.14376) on Graves' disease
MR Test
Inverse variance weighted (fixed effects)
Inverse variance weighted (multiplicative random effects)
MR Egger
MR RAPS
Weighted median
Weighted mode
0.10
0.05
0.00
−0.05
−0.10
SNP effect on Graves' disease
0.10
0.15
0.20
SNP effect on Gut microbiota abundance (genus Ruminococcus gnavus group id.14376)

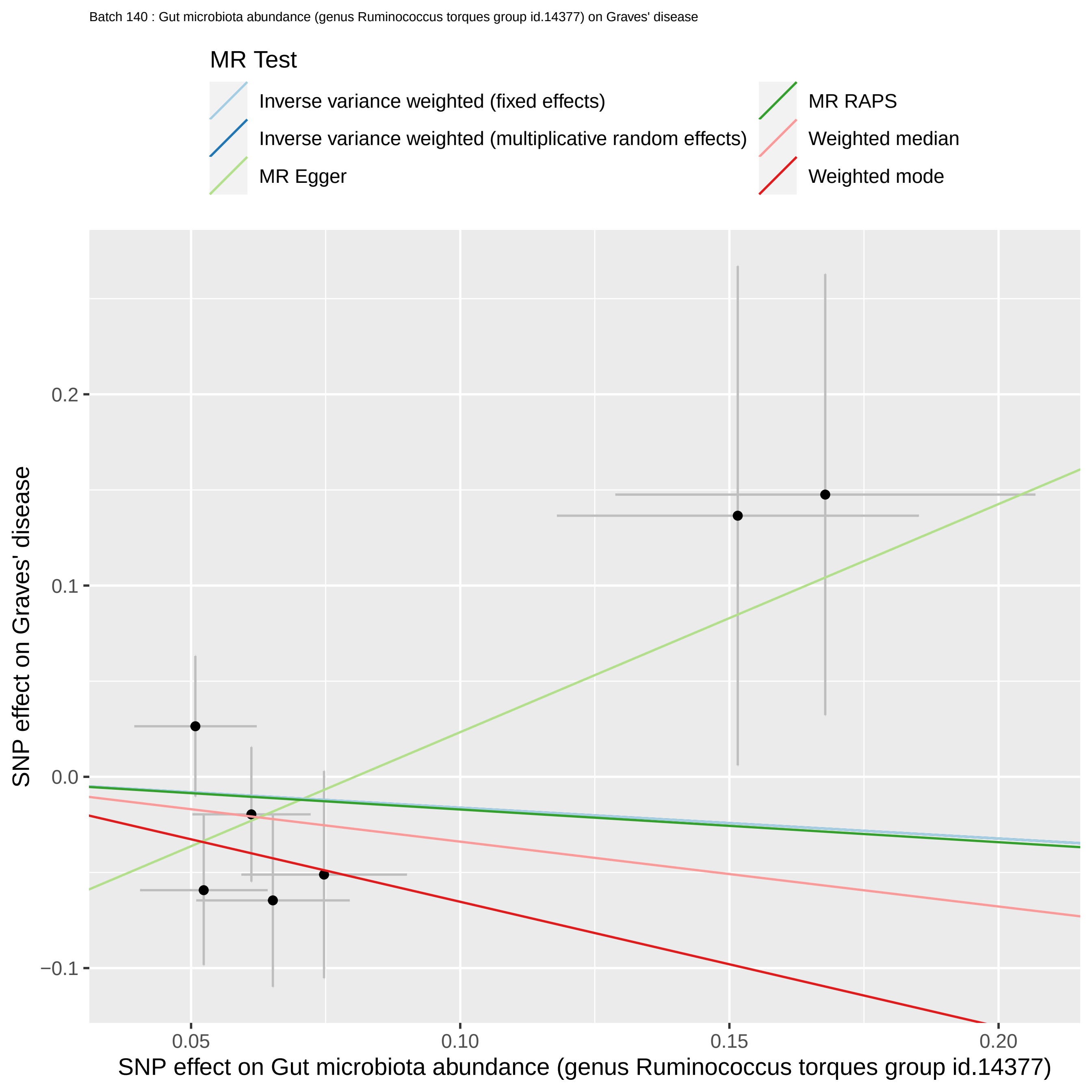

Batch 140 : Gut microbiota abundance (genus Ruminococcus torques group id.14377) on Graves' disease
MR Test
Inverse variance weighted (fixed effects)
Inverse variance weighted (multiplicative random effects)
MR Egger
MR RAPS
Weighted median
Weighted mode
0.2
0.1
0.0
−0.1
0.05
0.10
0.15
0.20
SNP effect on Graves' disease
SNP effect on Gut microbiota abundance (genus Ruminococcus torques group id.14377)

Batch 141 : Gut microbiota abundance (genus Sellimonas id.14369) on Graves' disease

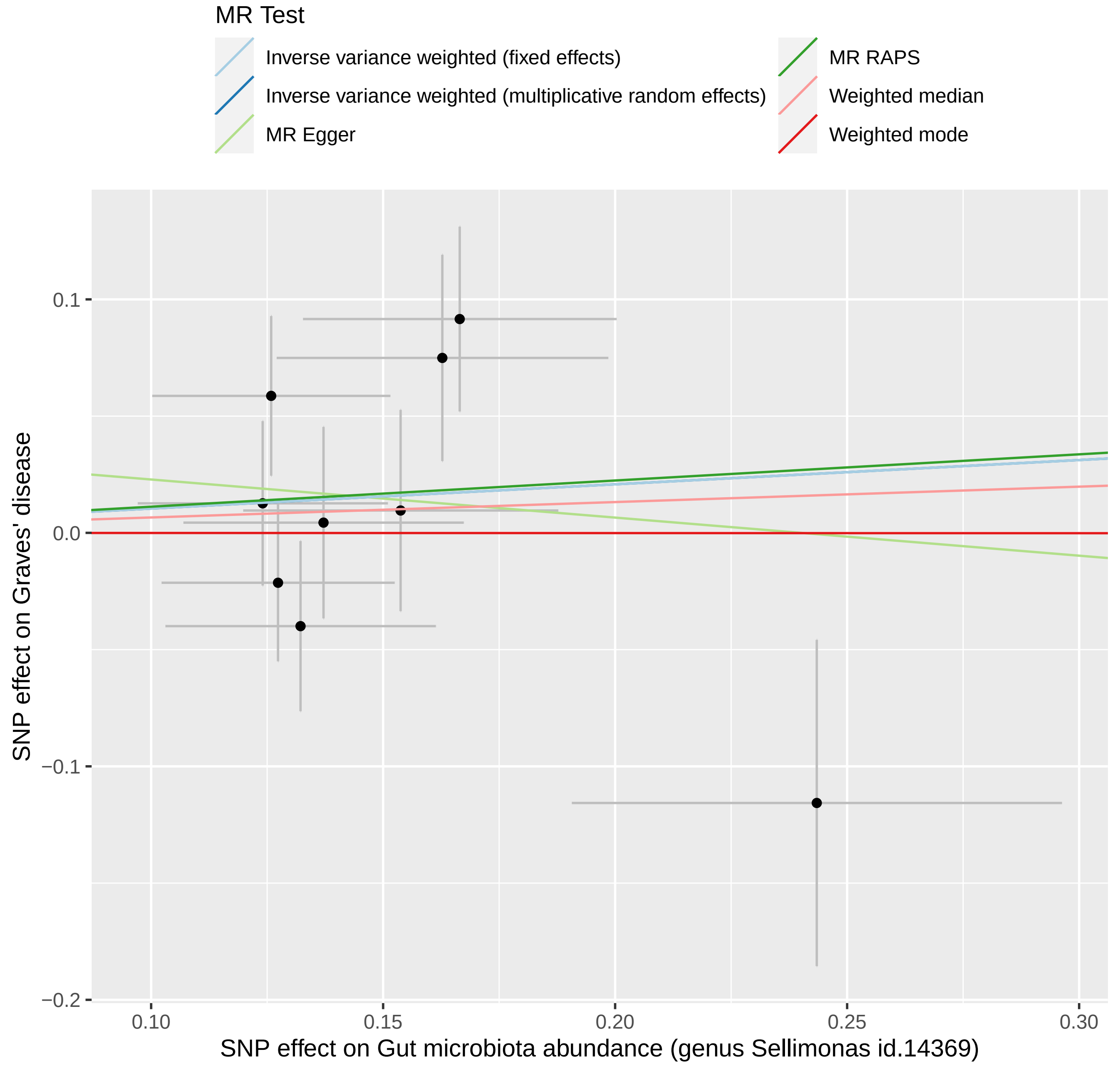

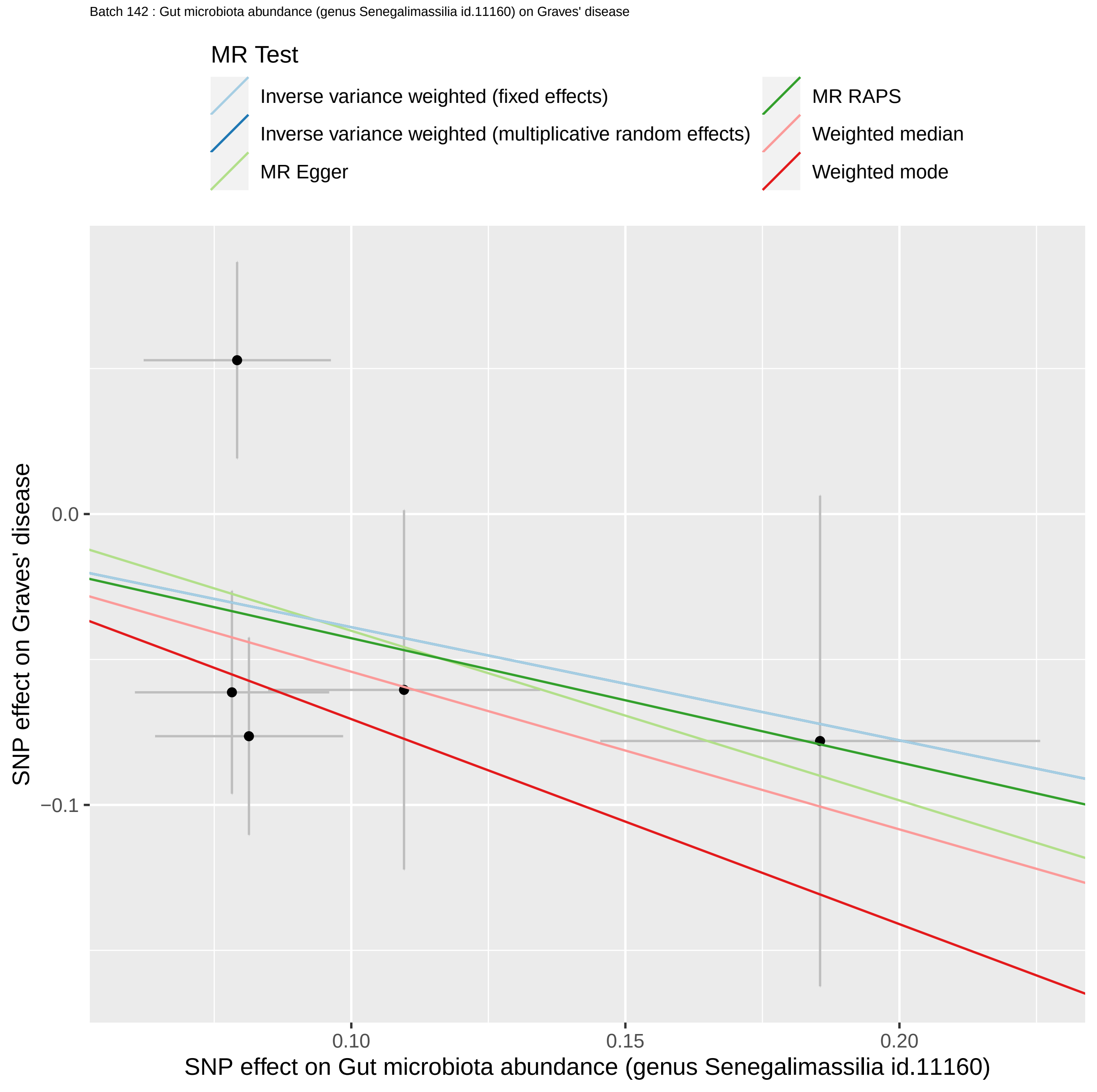

Batch 142 : Gut microbiota abundance (genus Senegalimassilia id.11160) on Graves' disease
MR Test
Inverse variance weighted (fixed effects)
Inverse variance weighted (multiplicative random effects)
MR Egger
MR RAPS
Weighted median
Weighted mode
0.0
−0.1
0.10
0.15
0.20
SNP effect on Graves' disease
SNP effect on Gut microbiota abundance (genus Senegalimassilia id.11160)

Batch 143 : Gut microbiota abundance (genus Slackia id.825) on Graves' disease

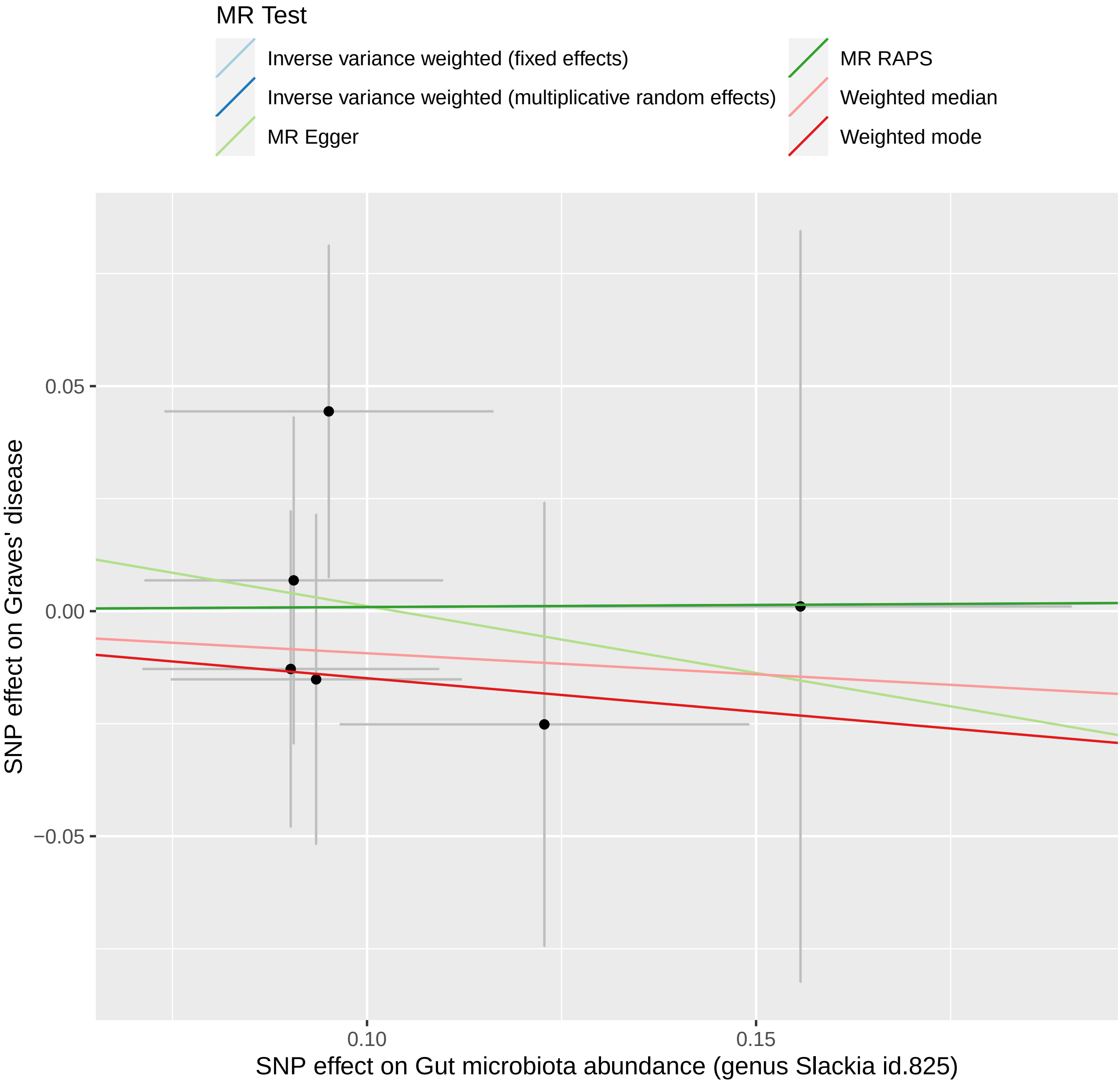

Batch 144 : Gut microbiota abundance (genus Streptococcus id.1853) on Graves' disease

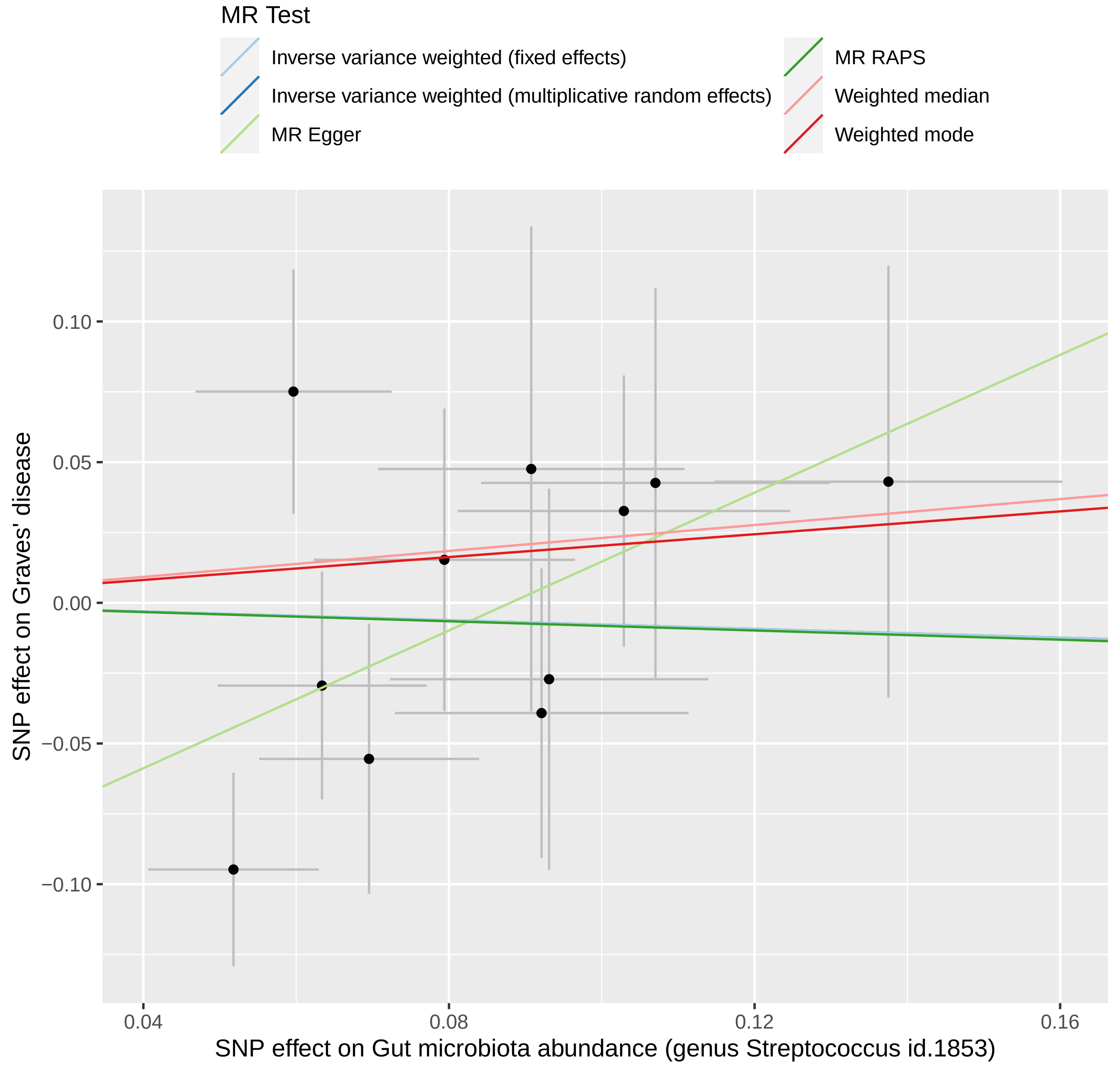

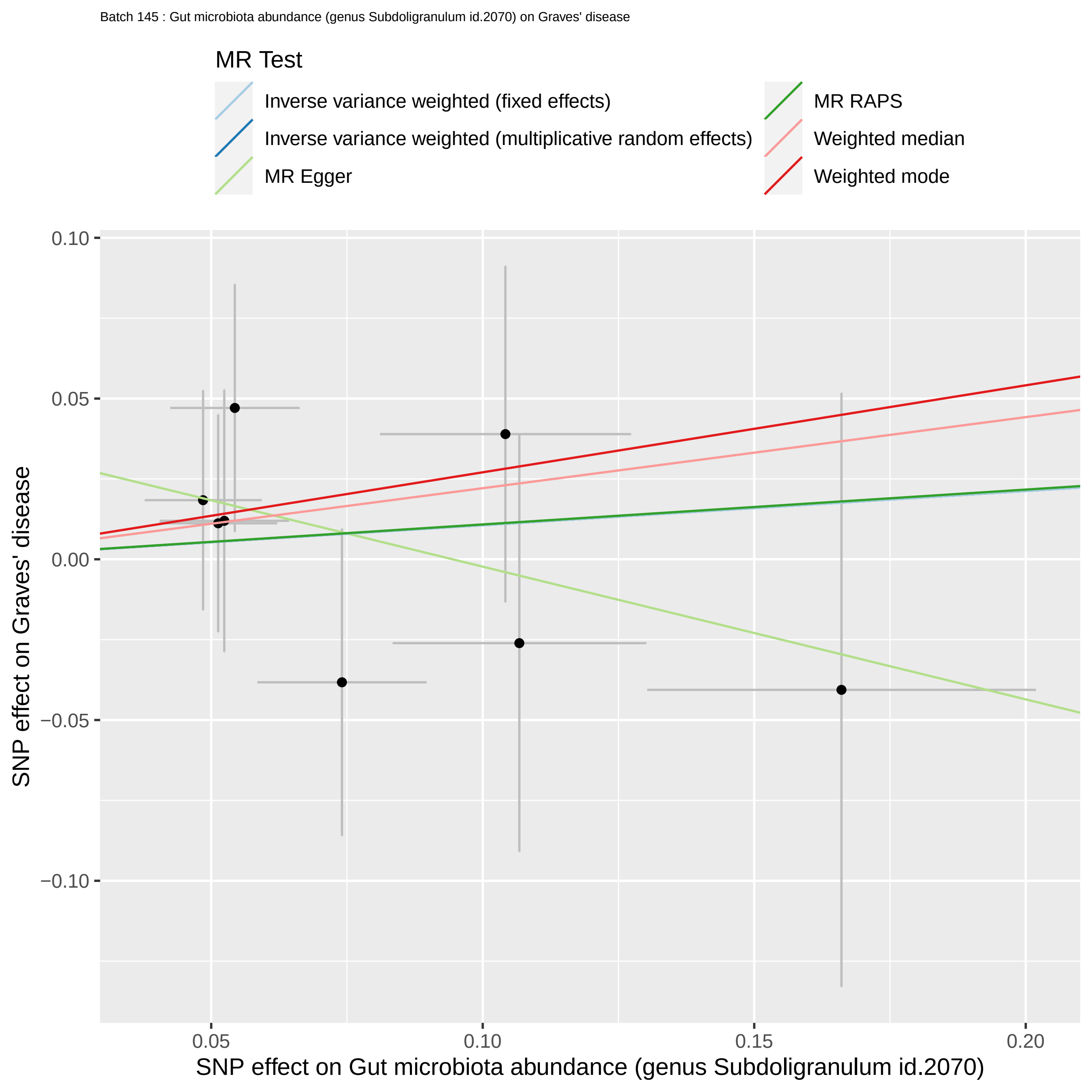

Batch 145 : Gut microbiota abundance (genus Subdoligranulum id.2070) on Graves' disease
MR Test
Inverse variance weighted (fixed effects)
Inverse variance weighted (multiplicative random effects)
MR Egger
MR RAPS
Weighted median
Weighted mode
0.10
0.05
0.00
−0.05
−0.10
SNP effect on Graves' disease
0.05
0.10
0.15
0.20
SNP effect on Gut microbiota abundance (genus Subdoligranulum id.2070)

Batch 146 : Gut microbiota abundance (genus Sutterella id.2896) on Graves' disease

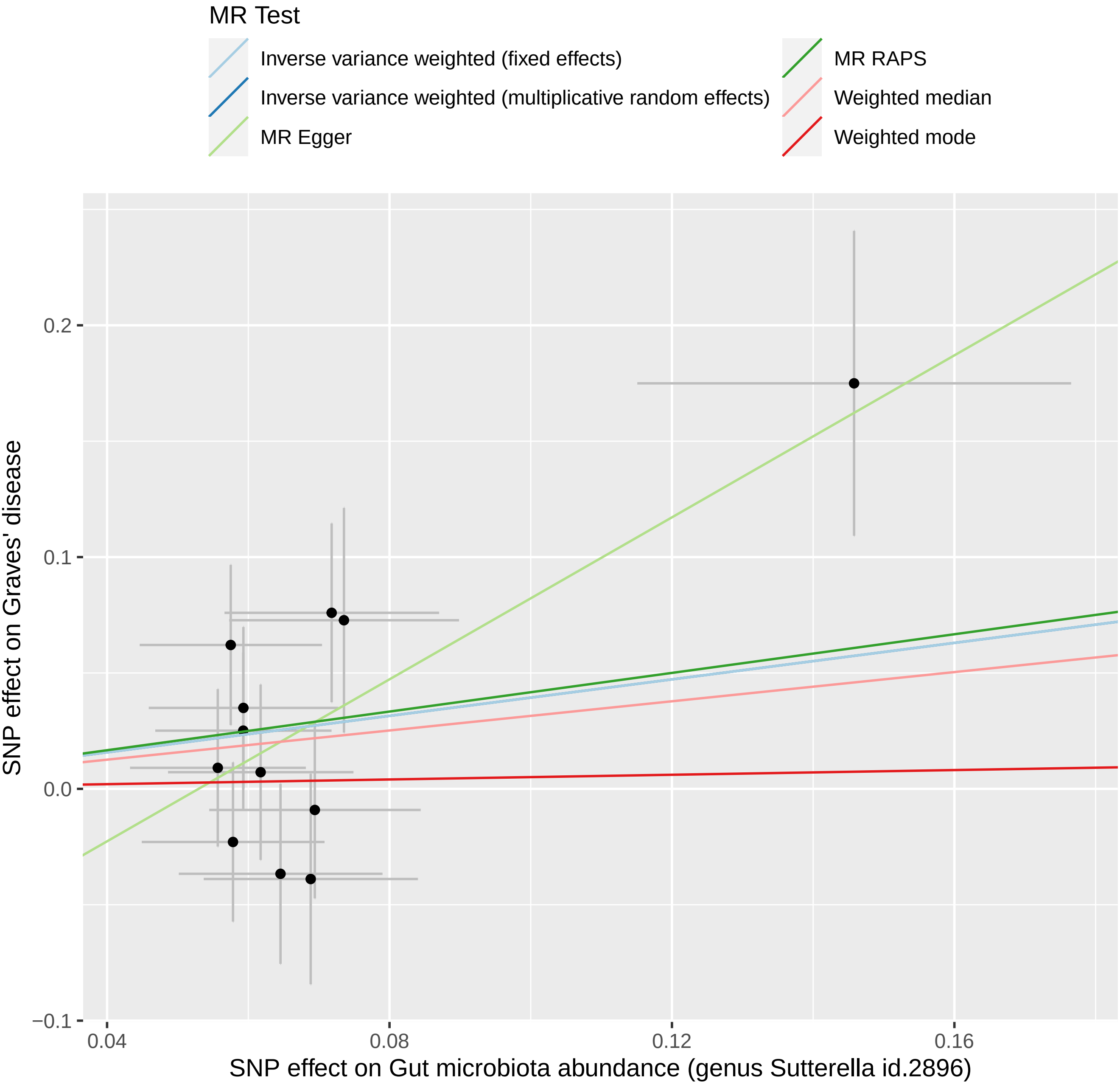

Batch 147 : Gut microbiota abundance (genus Terrisporobacter id.11348) on Graves' disease

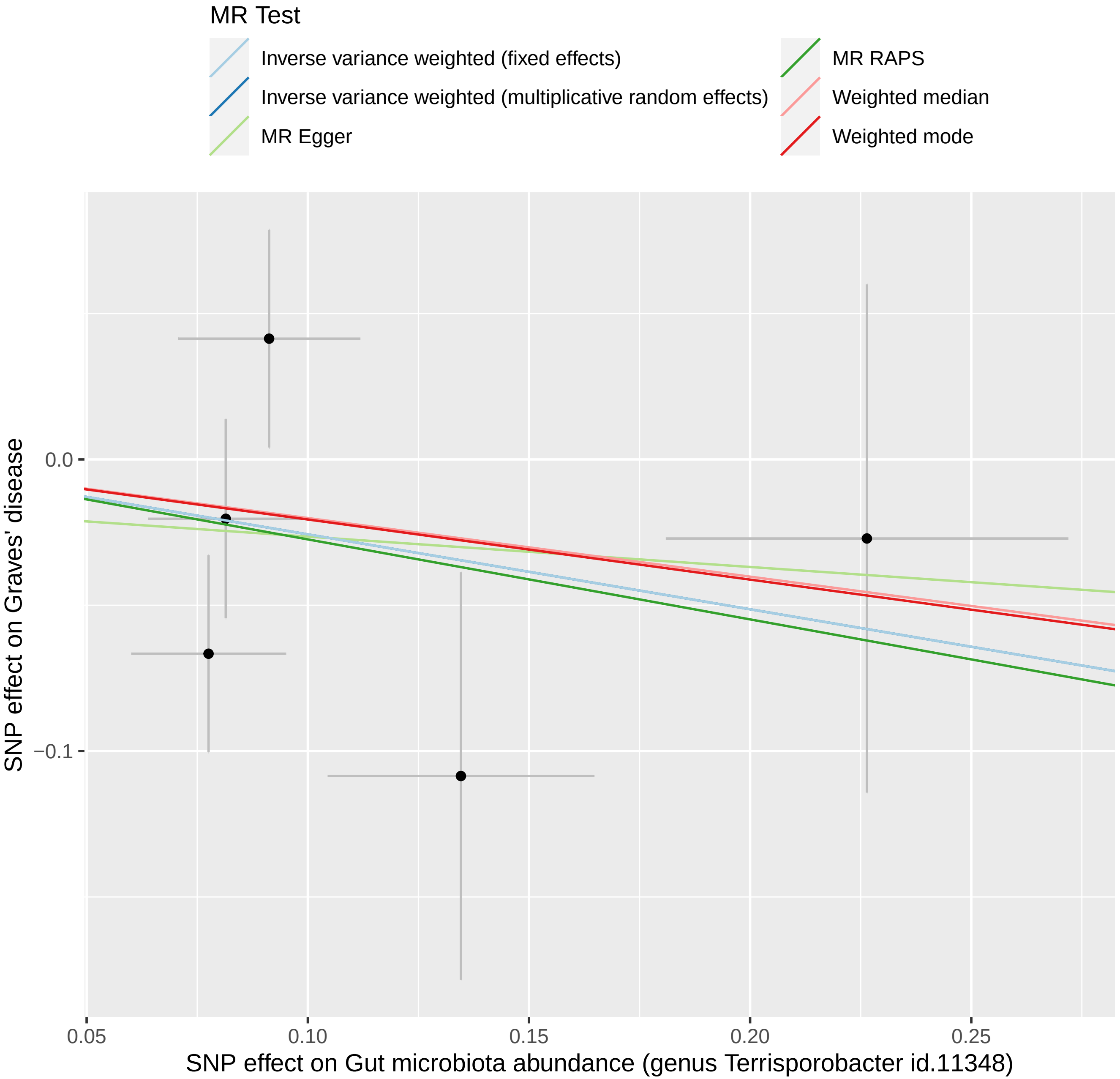

Batch 148 : Gut microbiota abundance (genus Turicibacter id.2162) on Graves' disease

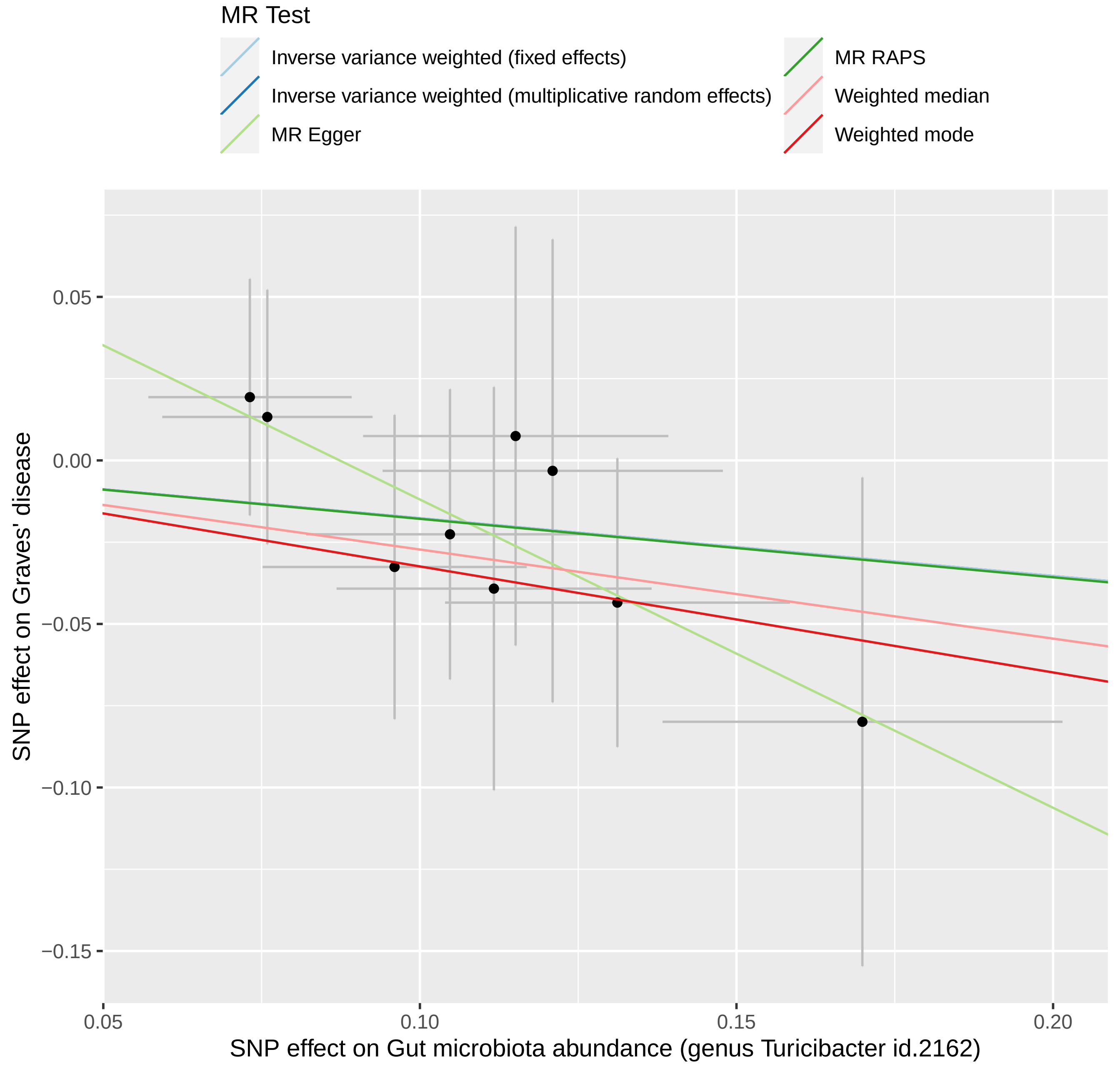

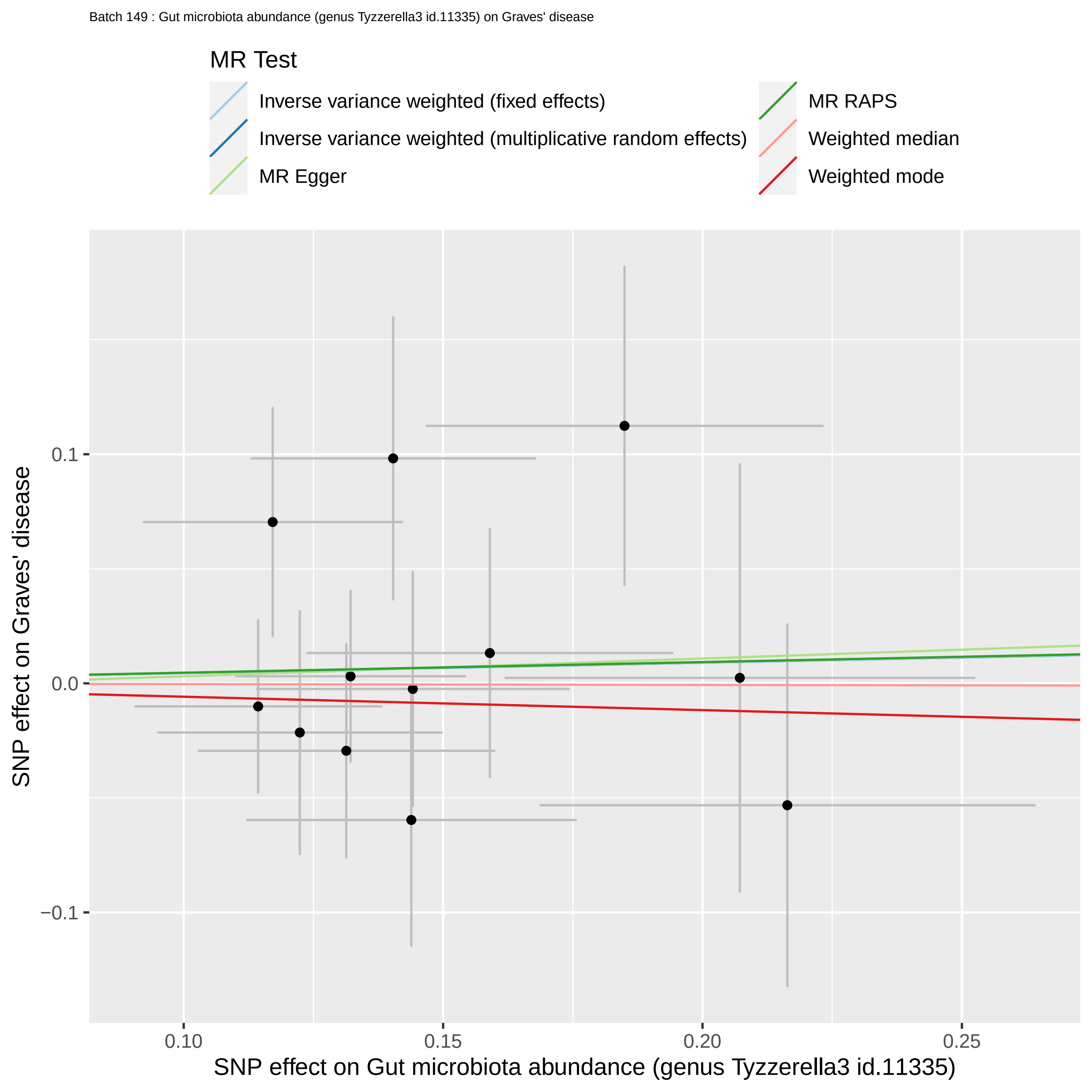
Batch 149 : Gut microbiota abundance (genus Tyzzerella3 id.11335) on Graves' disease
MR Test
Inverse variance weighted (fixed effects)
Inverse variance weighted (multiplicative random effects)
MR Egger
MR RAPS
Weighted median
Weighted mode
0.1
0.0
−0.1
0.10
0.15
0.20
0.25
SNP effect on Graves' disease
SNP effect on Gut microbiota abundance (genus Tyzzerella3 id.11335)

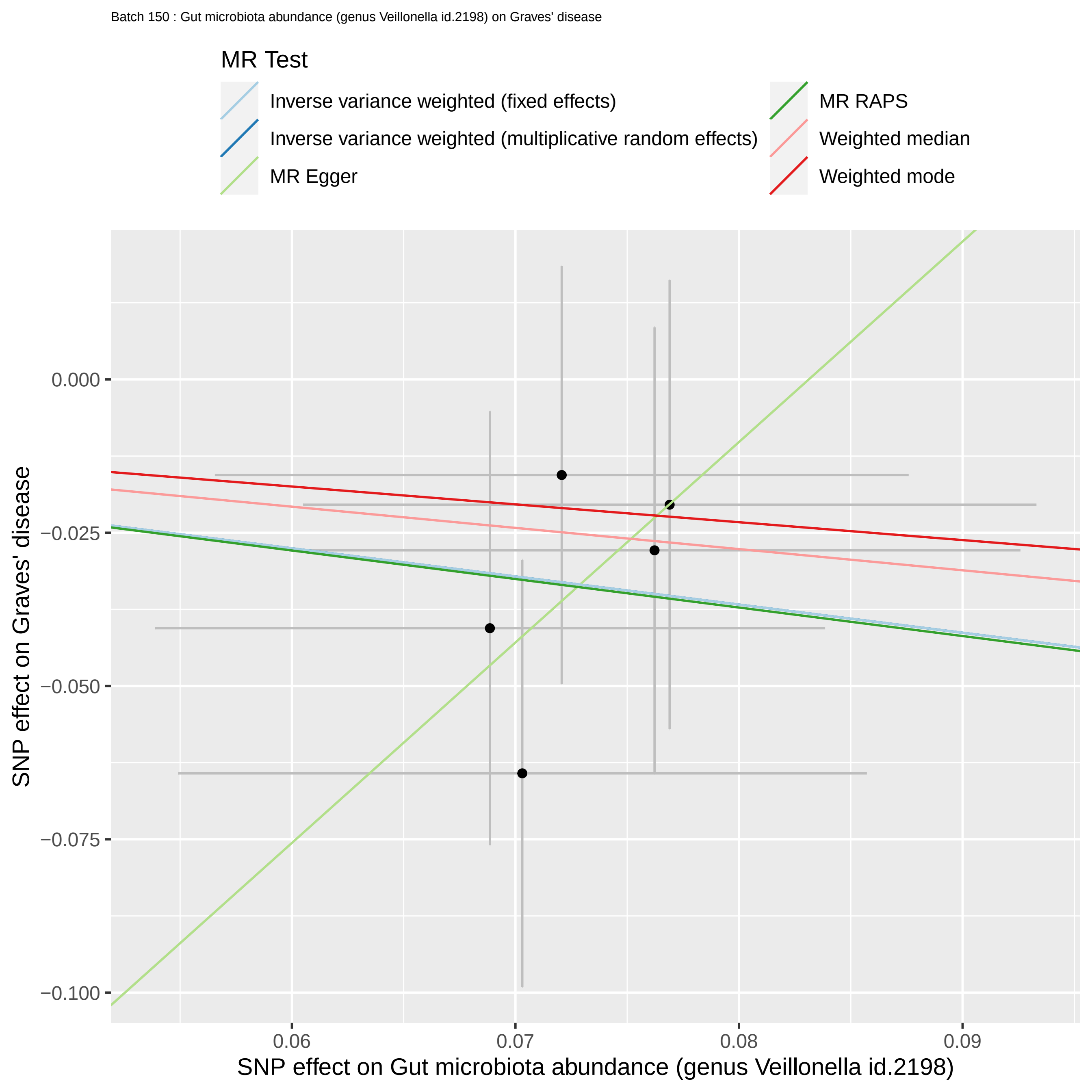

Batch 150 : Gut microbiota abundance (genus Veillonella id.2198) on Graves' disease
MR Test
Inverse variance weighted (fixed effects)
Inverse variance weighted (multiplicative random effects)
MR Egger
MR RAPS
Weighted median
Weighted mode
0.000
−0.025
−0.050
−0.075
−0.100
0.06
0.07
0.08
0.09
SNP effect on Graves' disease
SNP effect on Gut microbiota abundance (genus Veillonella id.2198)
