# Supplementary material for: Bacteroidaceae, Bacteroides, and Veillonella: emerging protectors against Graves’ disease
Source: Front Cell Infect Microbiol. 2024 Feb 9;14:1288222. doi: 10.3389/fcimb.2024.1288222 (PMC10884117; doi:10.3389/fcimb.2024.1288222)
Supplement: Supplementary file 3 [file DataSheet_3.pdf]

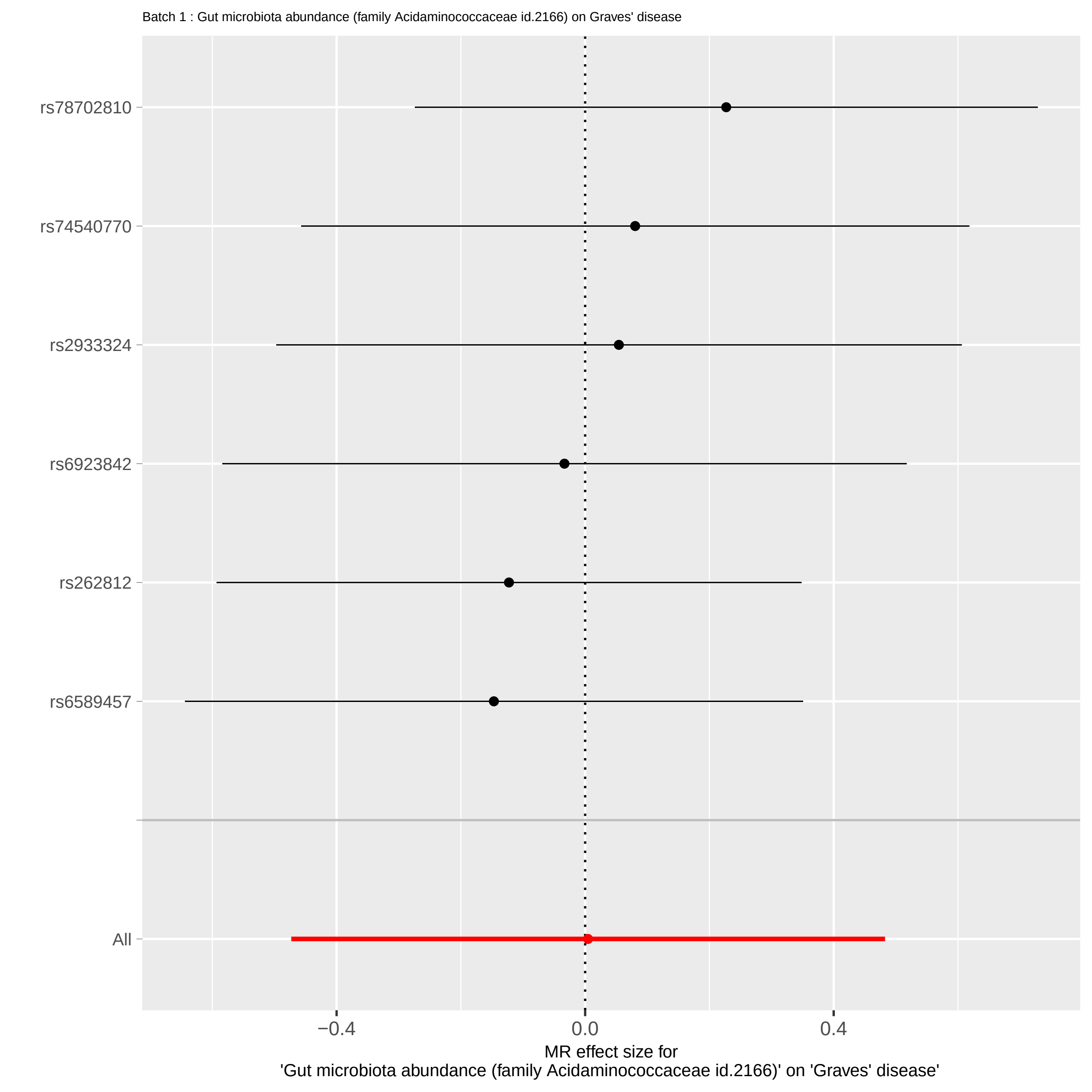
Batch 1 : Gut microbiota abundance (family Acidaminococcaceae id.2166) on Graves' disease
rs78702810
rs74540770
rs2933324
rs6923842
rs262812
rs6589457
All
−0.4
0.0
0.4
MR effect size for
'Gut microbiota abundance (family Acidaminococcaceae id.2166)' on 'Graves' disease'

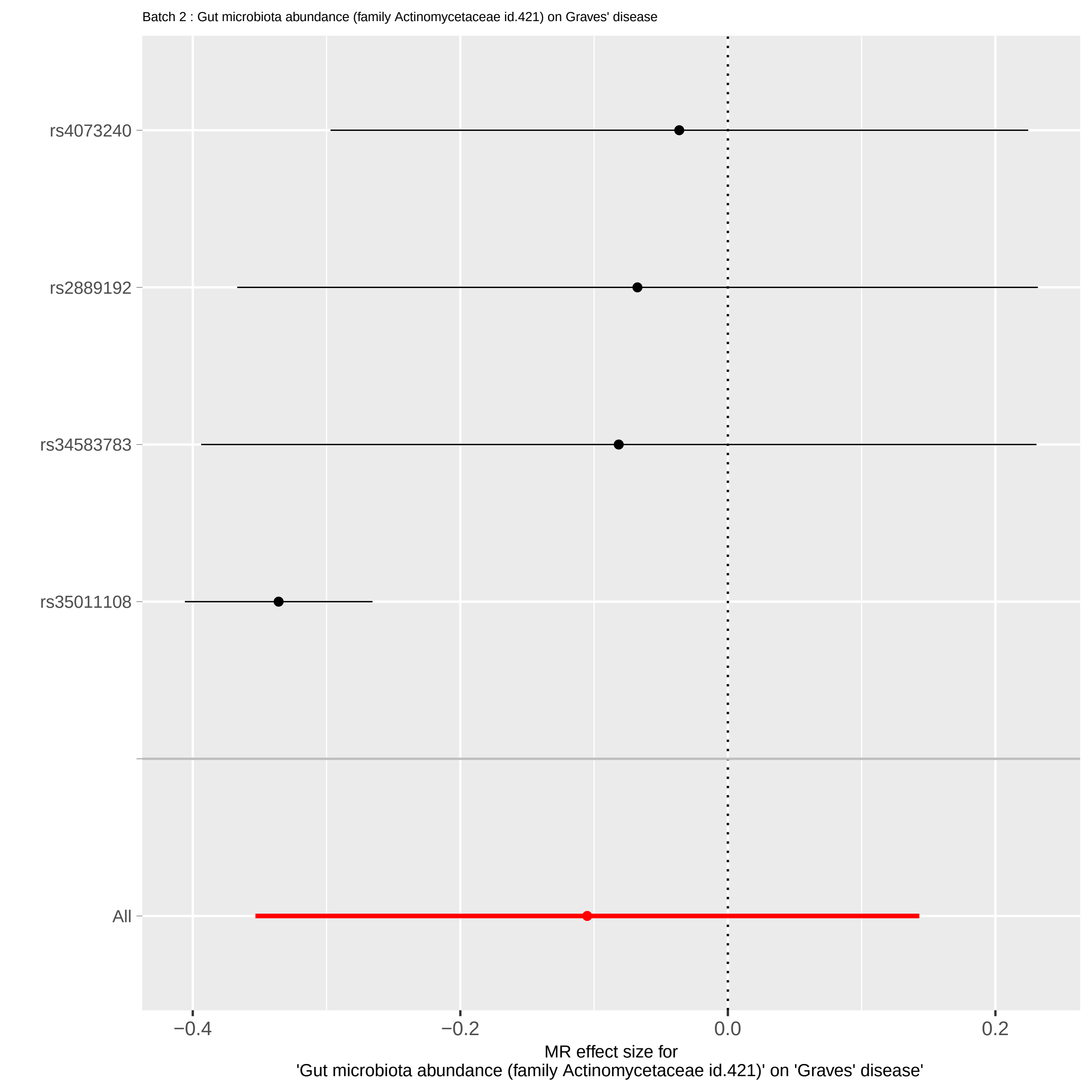

Batch 2 : Gut microbiota abundance (family Actinomycetaceae id.421) on Graves' disease
rs4073240
rs2889192
rs34583783
rs35011108
All
−0.4
−0.2
0.0
0.2
MR effect size for
'Gut microbiota abundance (family Actinomycetaceae id.421)' on 'Graves' disease'

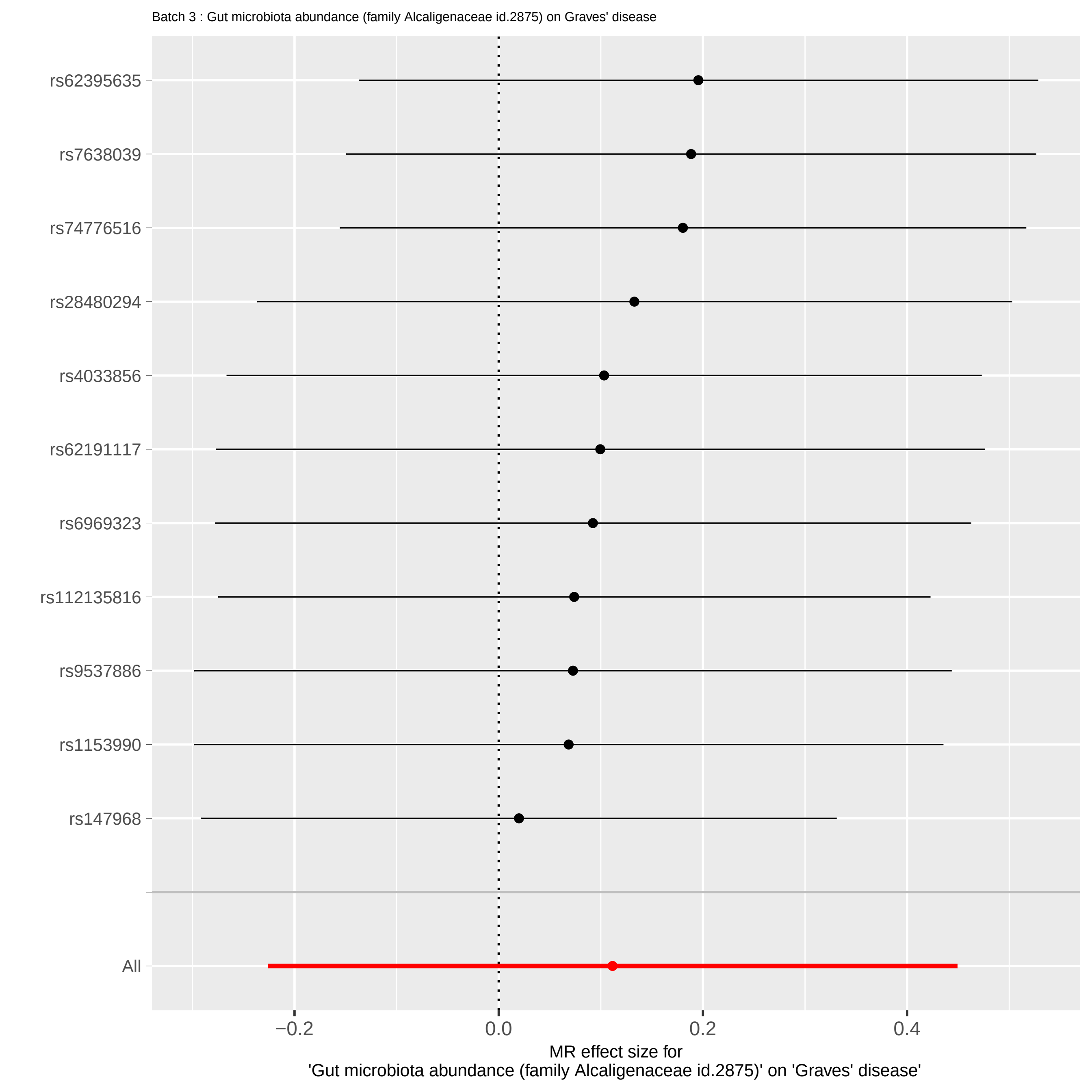
Batch 3 : Gut microbiota abundance (family Alcaligenaceae id.2875) on Graves' disease
rs62395635
rs7638039
rs74776516
rs28480294
rs4033856
rs62191117
rs6969323
rs112135816
rs9537886
rs1153990
rs147968
All
−0.2
0.0
0.2
0.4
MR effect size for
'Gut microbiota abundance (family Alcaligenaceae id.2875)' on 'Graves' disease'

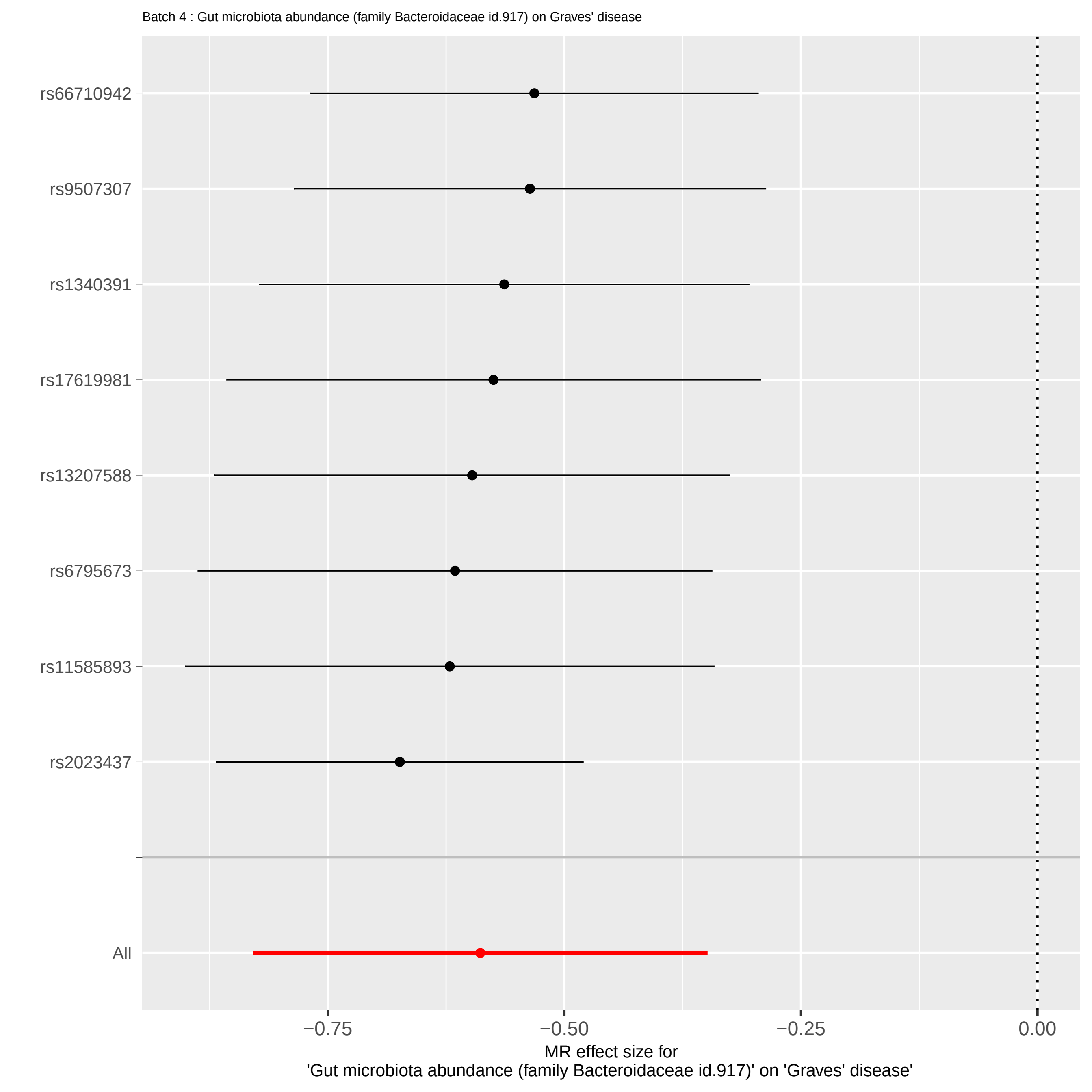

Batch 4 : Gut microbiota abundance (family Bacteroidaceae id.917) on Graves' disease
rs66710942
rs9507307
rs1340391
rs17619981
rs13207588
rs6795673
rs11585893
rs2023437
All
−0.75
−0.50
−0.25
0.00
MR effect size for
'Gut microbiota abundance (family Bacteroidaceae id.917)' on 'Graves' disease'

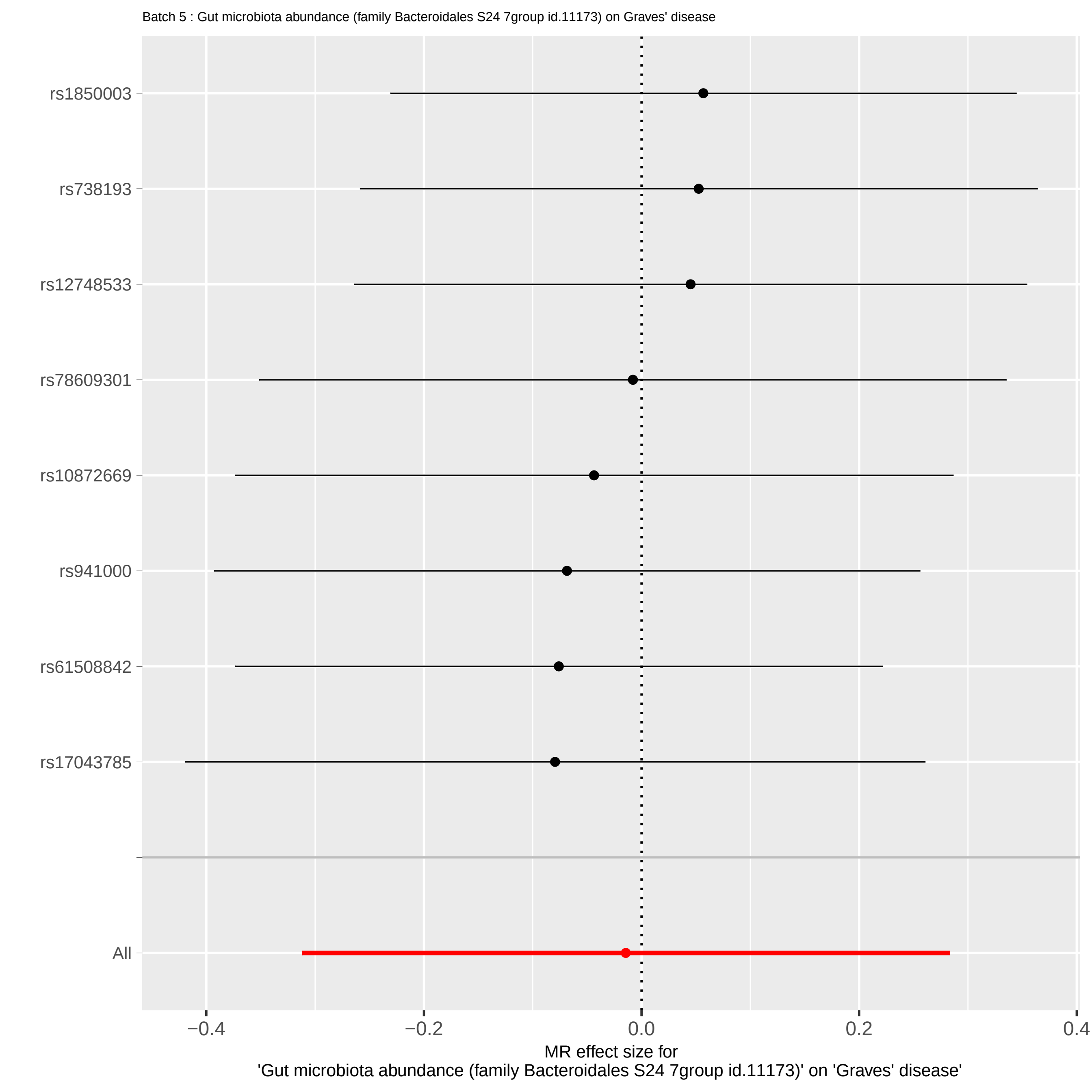
Batch 5 : Gut microbiota abundance (family Bacteroidales S24 7group id.11173) on Graves' disease
rs1850003
rs738193
rs12748533
rs78609301
rs10872669
rs941000
rs61508842
rs17043785
All
−0.4
−0.2
0.0
0.2
0.4
MR effect size for
'Gut microbiota abundance (family Bacteroidales S24 7group id.11173)' on 'Graves' disease'

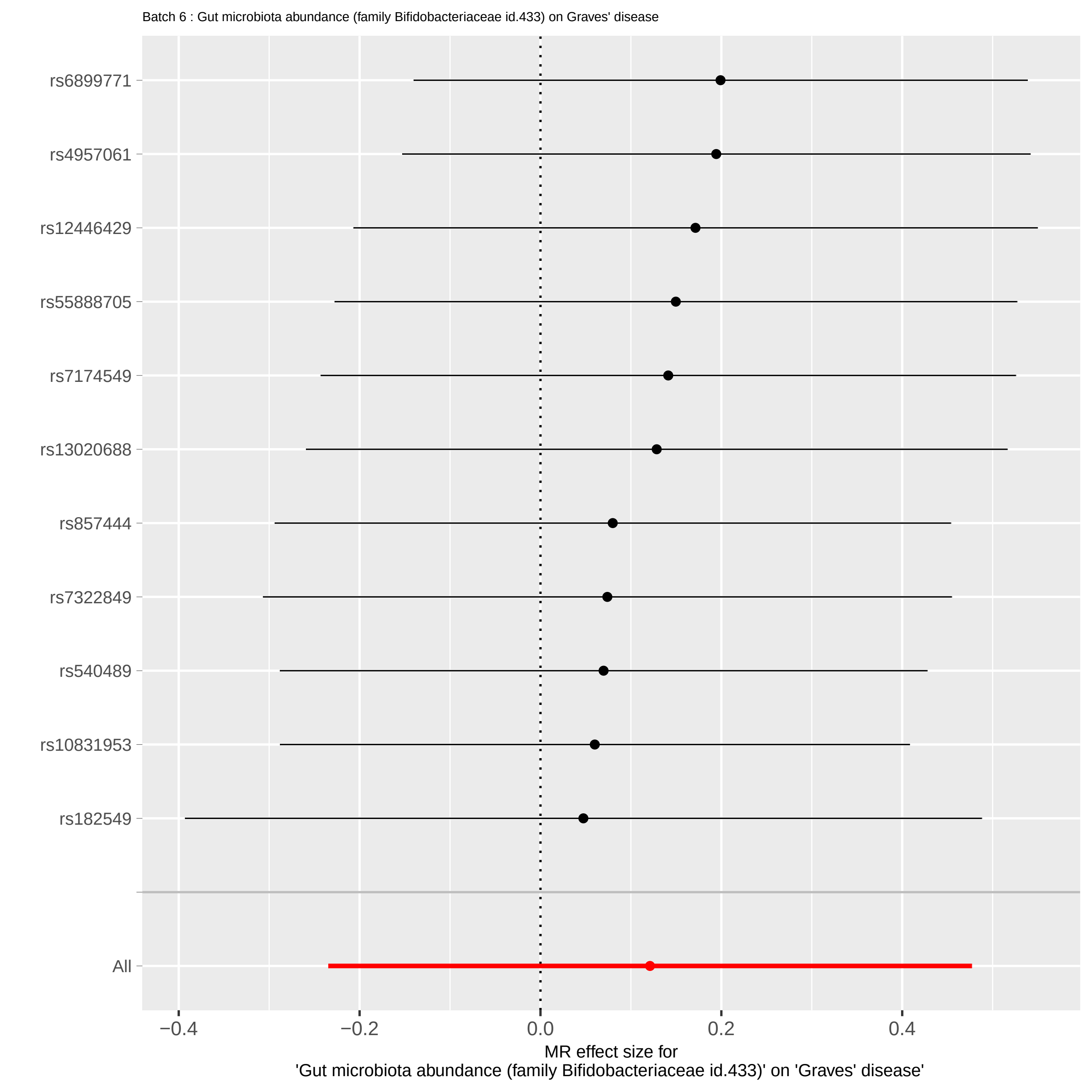
Batch 6 : Gut microbiota abundance (family Bifidobacteriaceae id.433) on Graves' disease
rs6899771
rs4957061
rs12446429
rs55888705
rs7174549
rs13020688
rs857444
rs7322849
rs540489
rs10831953
rs182549
All
−0.4
−0.2
0.0
0.2
0.4
MR effect size for
'Gut microbiota abundance (family Bifidobacteriaceae id.433)' on 'Graves' disease'

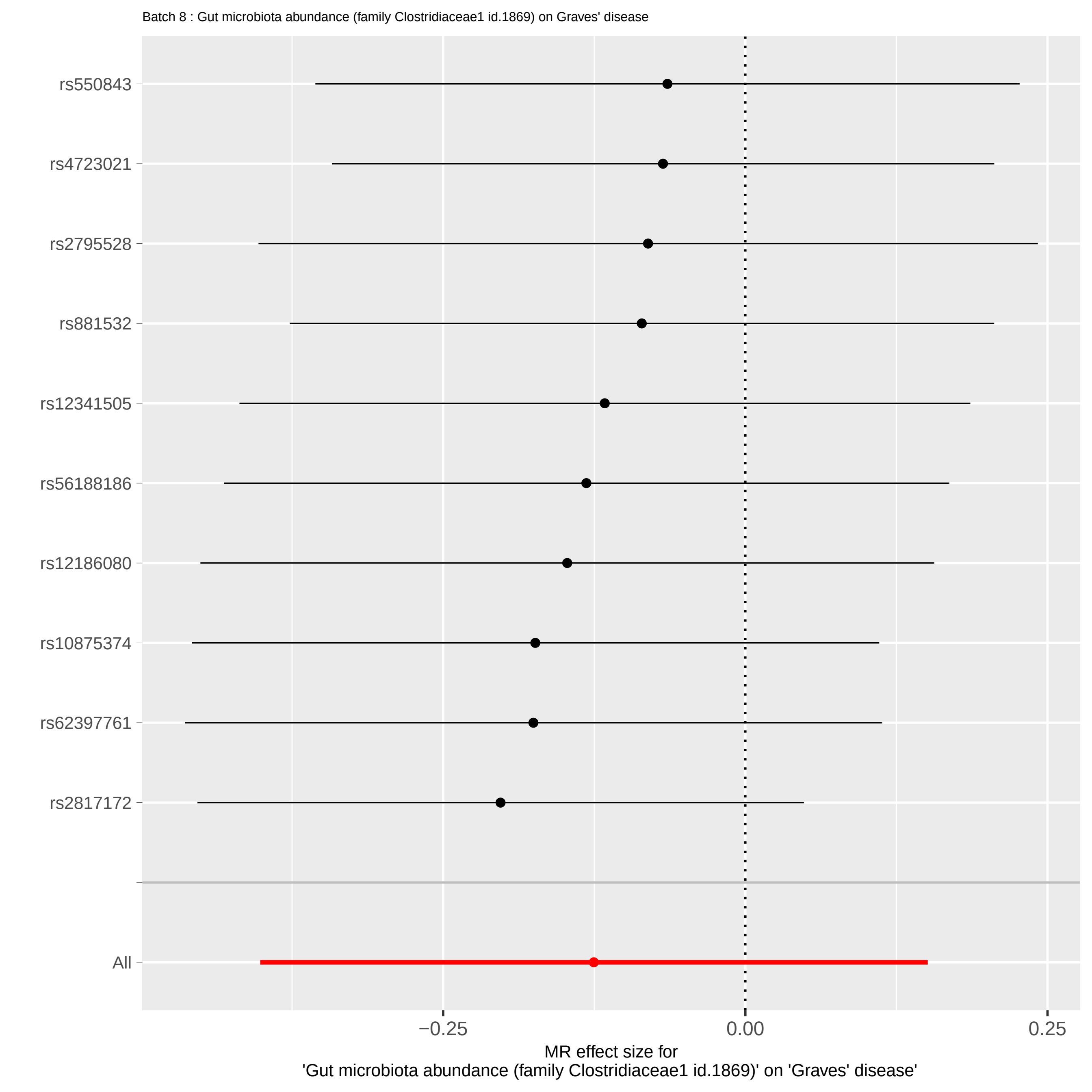

Batch 8 : Gut microbiota abundance (family Clostridiaceae1 id.1869) on Graves' disease
rs550843
rs4723021
rs2795528
rs881532
rs12341505
rs56188186
rs12186080
rs10875374
rs62397761
rs2817172
All
−0.25
0.00
0.25
MR effect size for
'Gut microbiota abundance (family Clostridiaceae1 id.1869)' on 'Graves' disease'

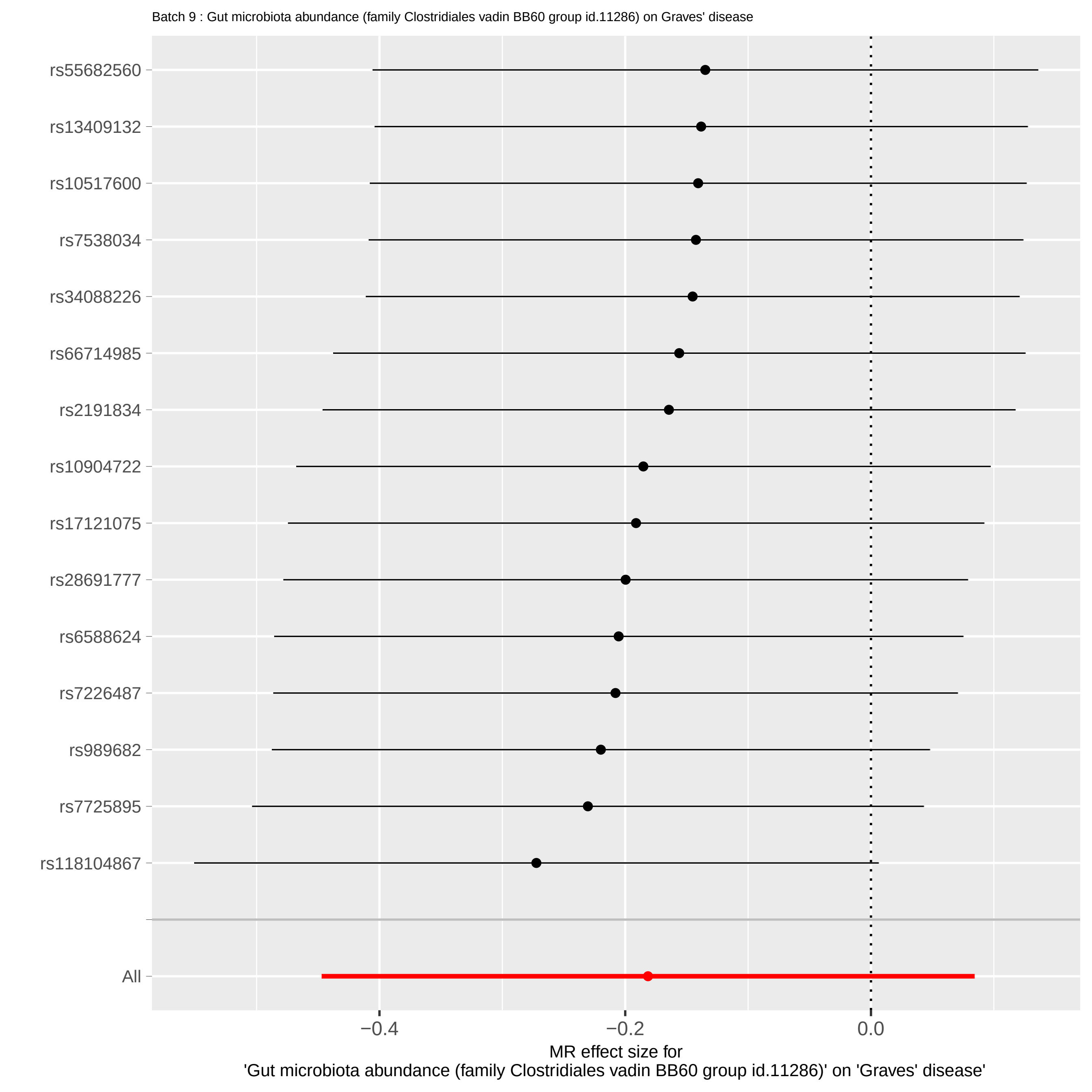

Batch 9 : Gut microbiota abundance (family Clostridiales vadin BB60 group id.11286) on Graves' disease
rs55682560
rs13409132
rs10517600
rs7538034
rs34088226
rs66714985
rs2191834
rs10904722
rs17121075
rs28691777
rs6588624
rs7226487
rs989682
rs7725895
rs118104867
All
−0.4
−0.2
0.0
MR effect size for
'Gut microbiota abundance (family Clostridiales vadin BB60 group id.11286)' on 'Graves' disease'

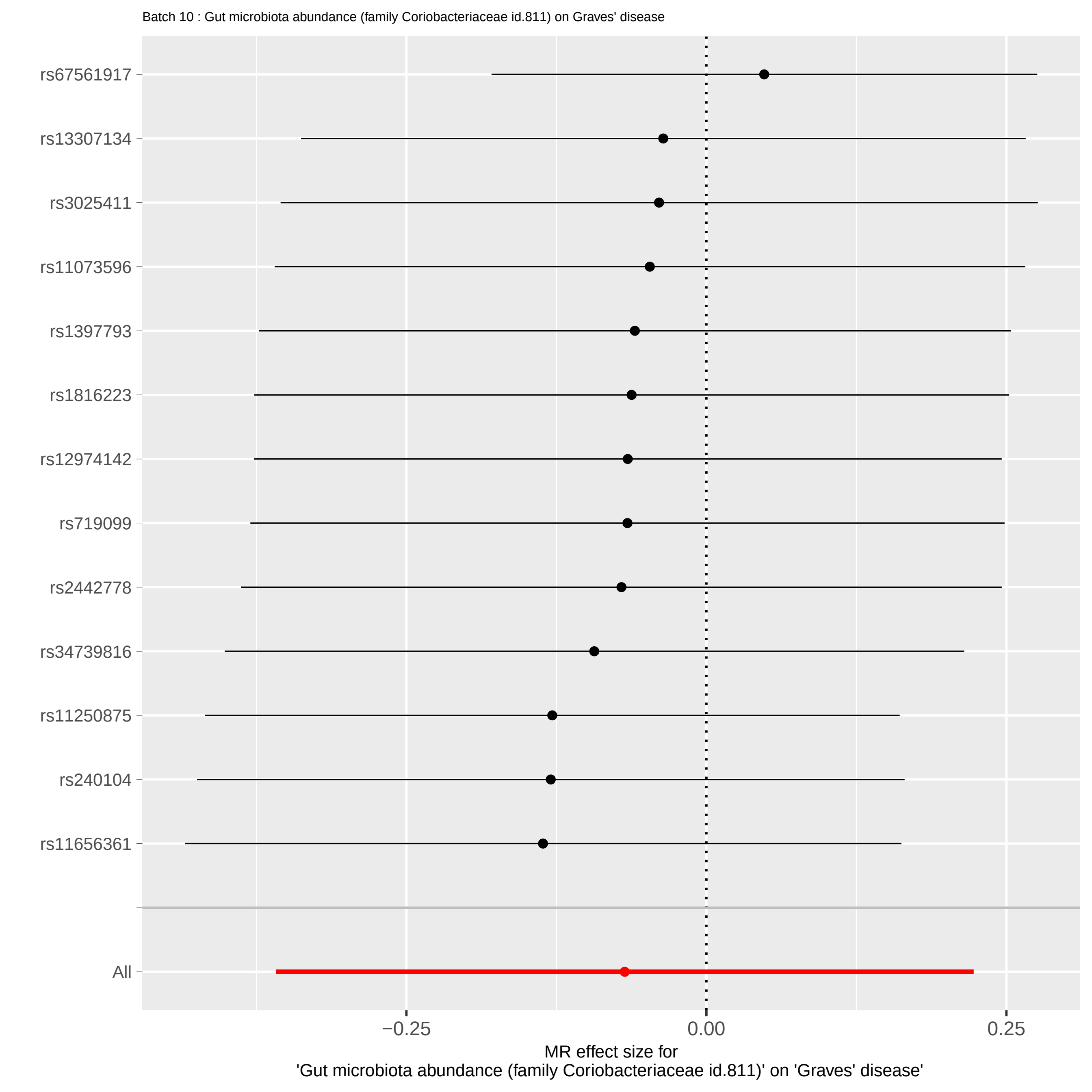
Batch 10 : Gut microbiota abundance (family Coriobacteriaceae id.811) on Graves' disease
rs67561917
rs13307134
rs3025411
rs11073596
rs1397793
rs1816223
rs12974142
rs719099
rs2442778
rs34739816
rs11250875
rs240104
rs11656361
All
−0.25
0.00
0.25
MR effect size for
'Gut microbiota abundance (family Coriobacteriaceae id.811)' on 'Graves' disease'

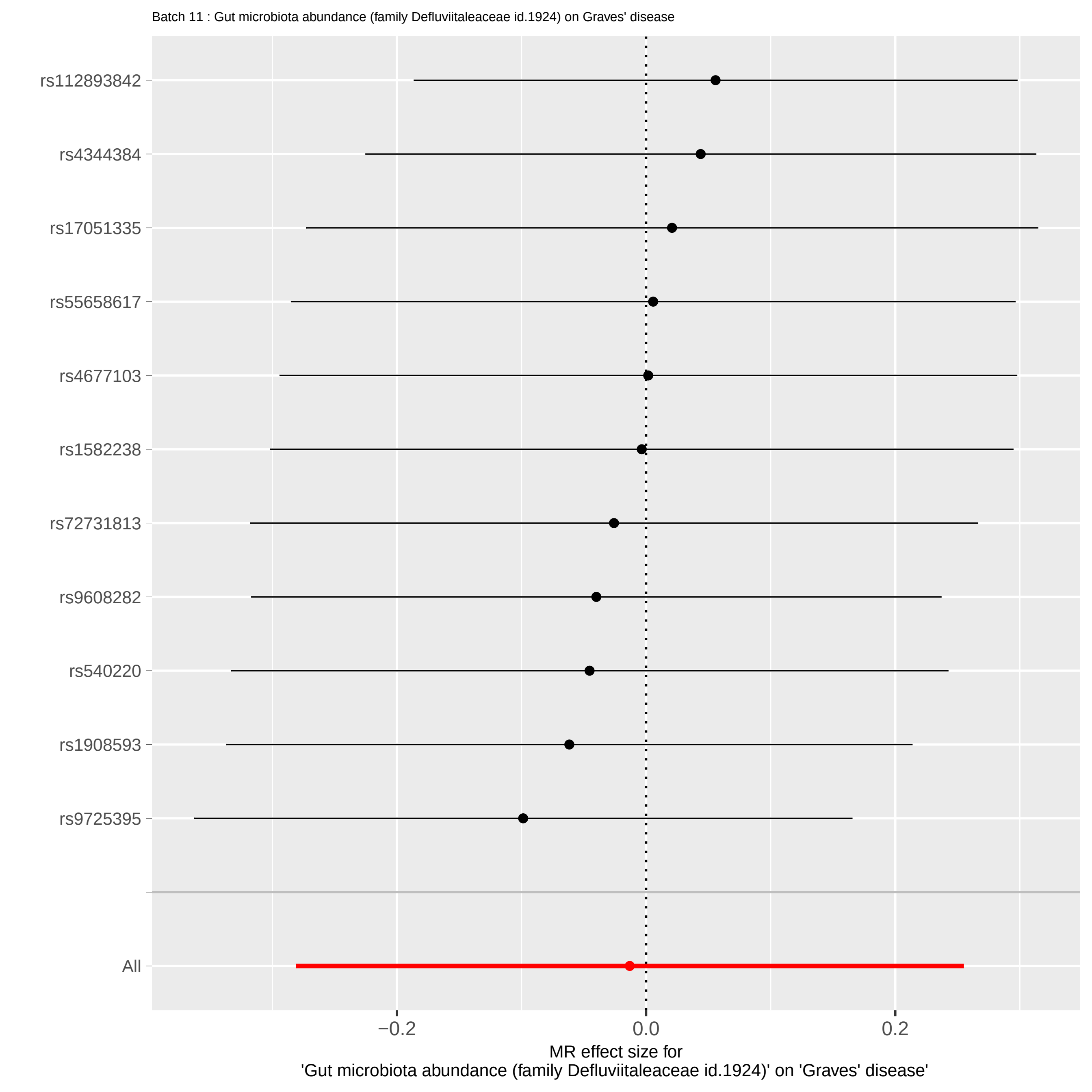

Batch 11 : Gut microbiota abundance (family Defluviitaleaceae id.1924) on Graves' disease
rs112893842
rs4344384
rs17051335
rs55658617
rs4677103
rs1582238
rs72731813
rs9608282
rs540220
rs1908593
rs9725395
All
−0.2
0.0
0.2
MR effect size for
'Gut microbiota abundance (family Defluviitaleaceae id.1924)' on 'Graves' disease'

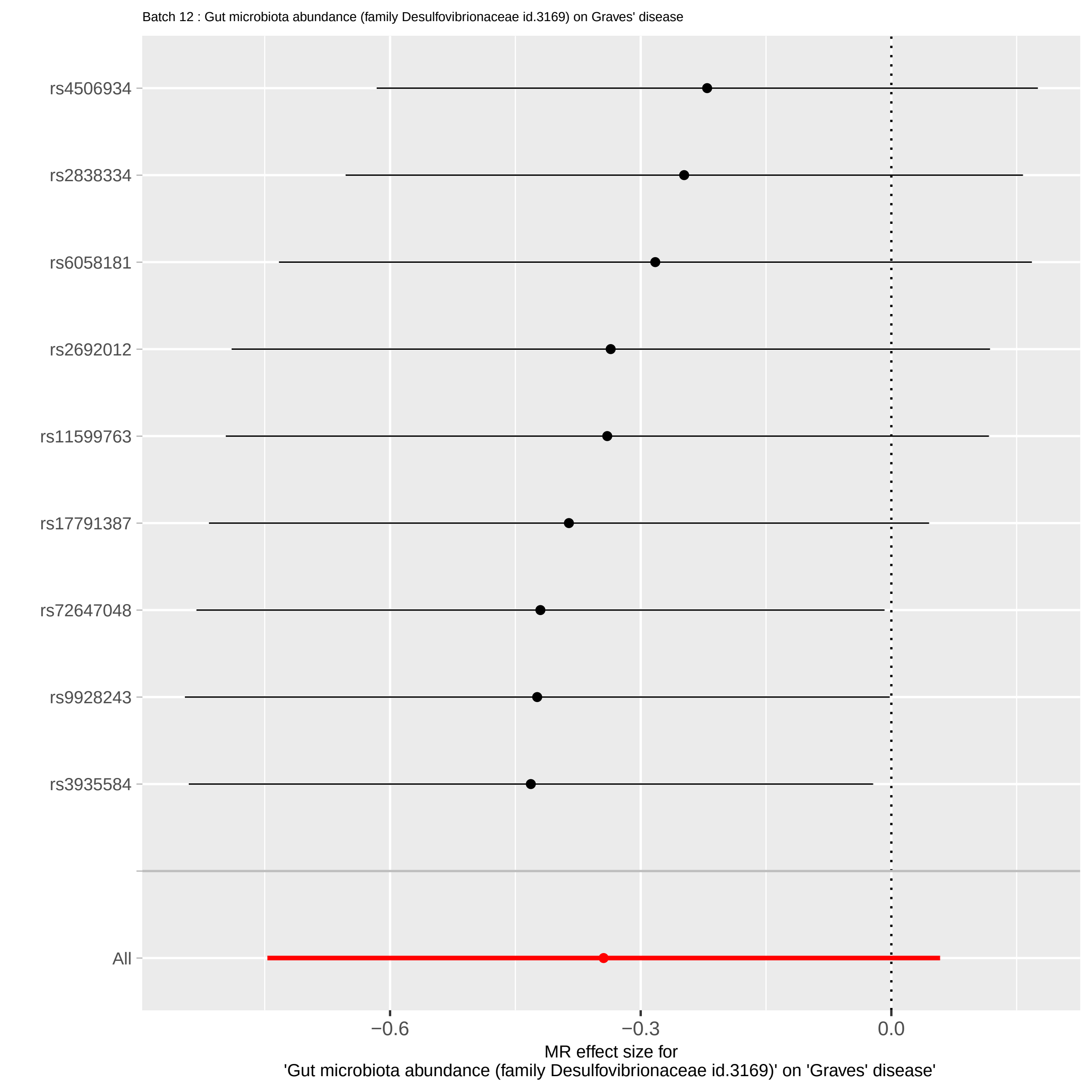
Batch 12 : Gut microbiota abundance (family Desulfovibrionaceae id.3169) on Graves' disease
rs4506934
rs2838334
rs6058181
rs2692012
rs11599763
rs17791387
rs72647048
rs9928243
rs3935584
All
−0.6
−0.3
0.0
MR effect size for
'Gut microbiota abundance (family Desulfovibrionaceae id.3169)' on 'Graves' disease'

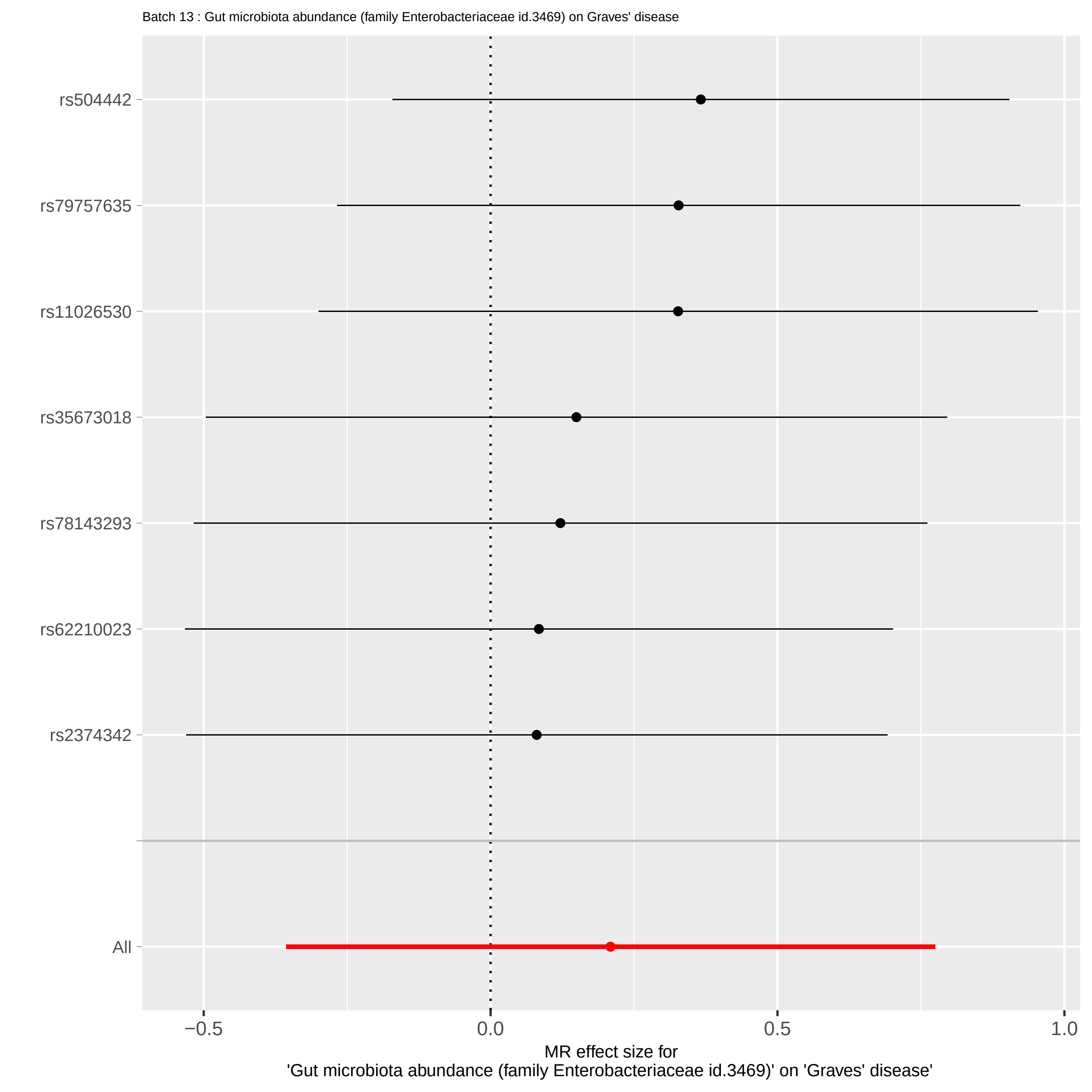
Batch 13 : Gut microbiota abundance (family Enterobacteriaceae id.3469) on Graves' disease
rs504442
rs79757635
rs11026530
rs35673018
rs78143293
rs62210023
rs2374342
All
−0.5
0.0
0.5
1.0
MR effect size for
'Gut microbiota abundance (family Enterobacteriaceae id.3469)' on 'Graves' disease'

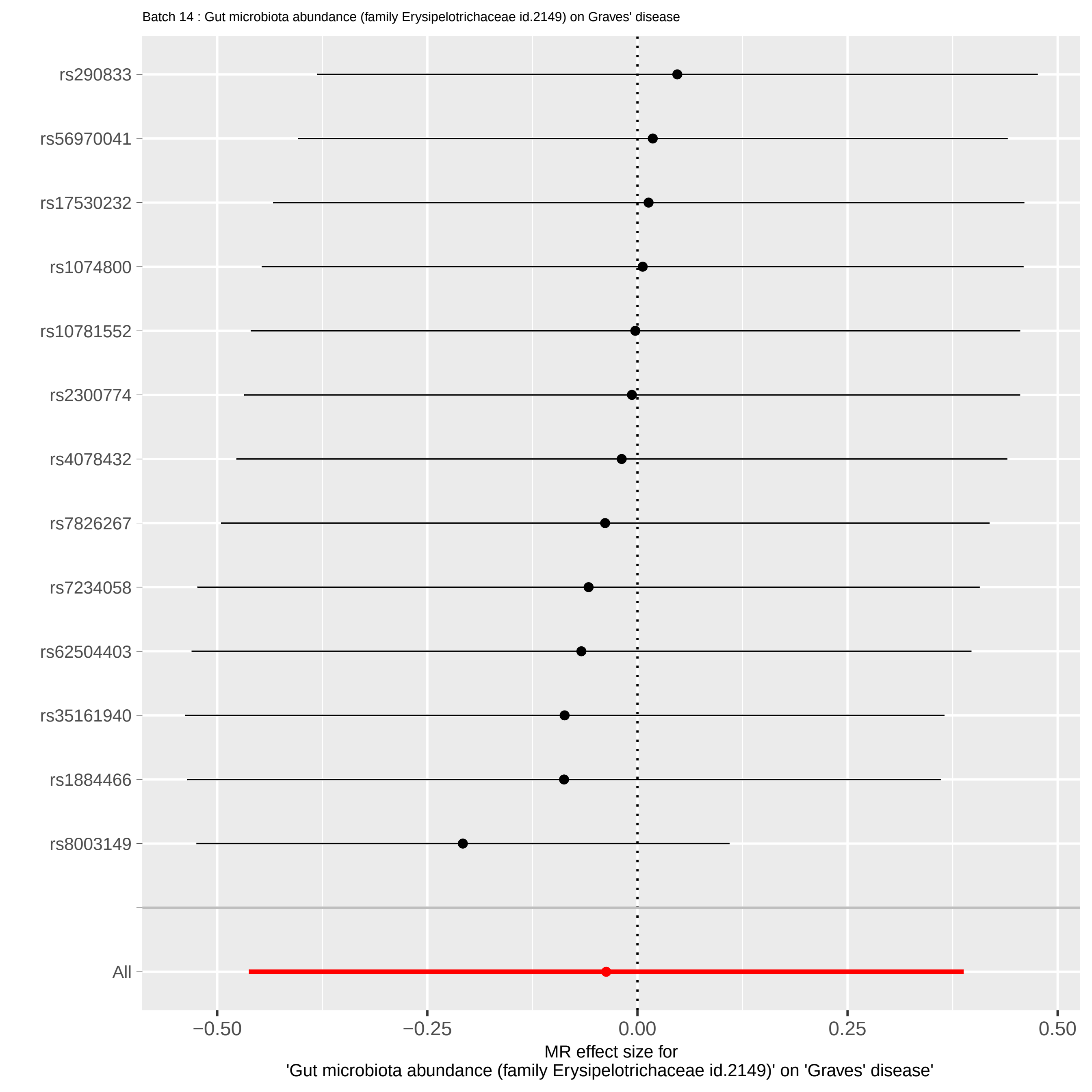

Batch 14 : Gut microbiota abundance (family Erysipelotrichaceae id.2149) on Graves' disease
rs290833
rs56970041
rs17530232
rs1074800
rs10781552
rs2300774
rs4078432
rs7826267
rs7234058
rs62504403
rs35161940
rs1884466
rs8003149
All
−0.50
−0.25
0.00
0.25
0.50
MR effect size for
'Gut microbiota abundance (family Erysipelotrichaceae id.2149)' on 'Graves' disease'

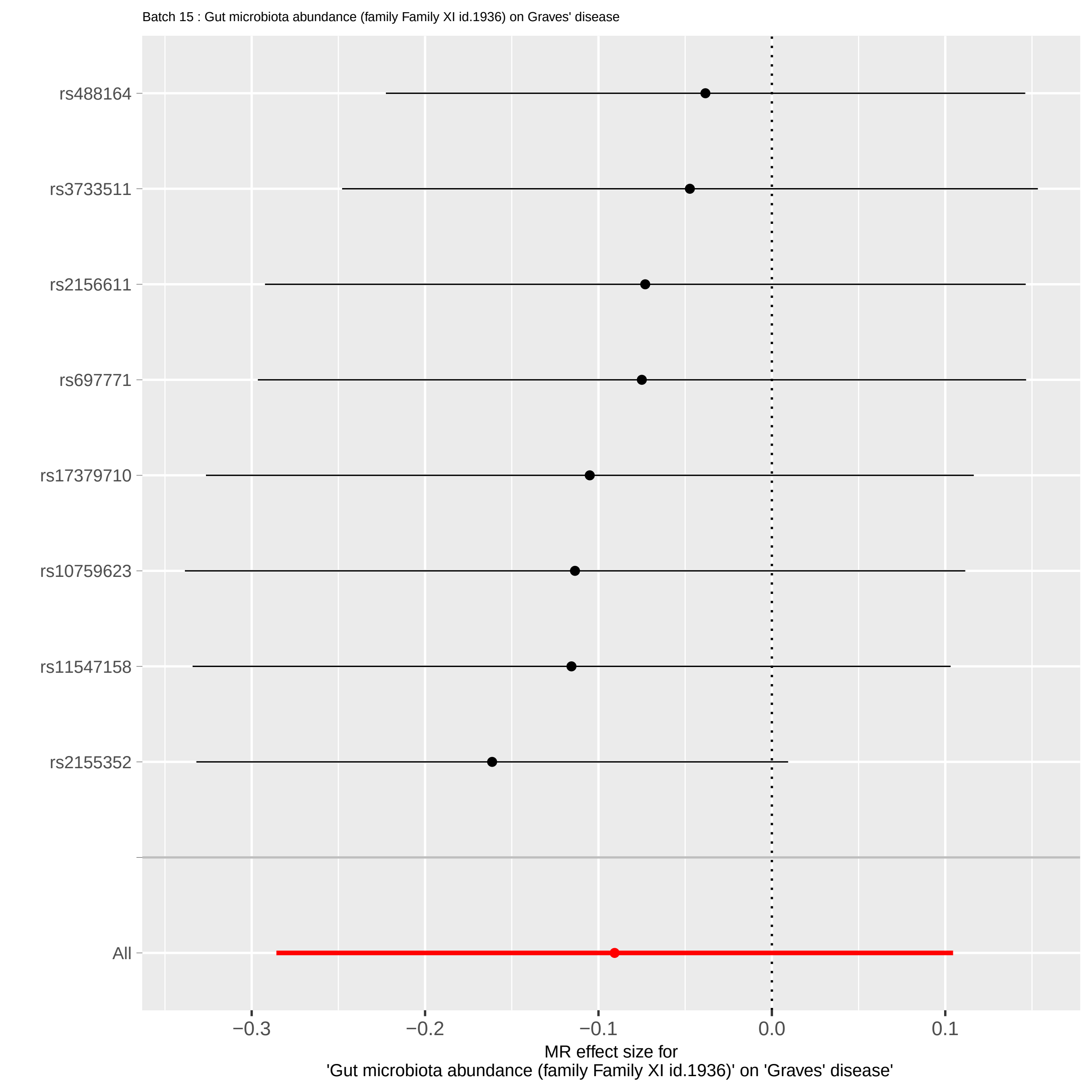
Batch 15 : Gut microbiota abundance (family Family XI id.1936) on Graves' disease
rs488164
rs3733511
rs2156611
rs697771
rs17379710
rs10759623
rs11547158
rs2155352
All
−0.3
−0.2
−0.1
0.0
0.1
MR effect size for
'Gut microbiota abundance (family Family XI id.1936)' on 'Graves' disease'

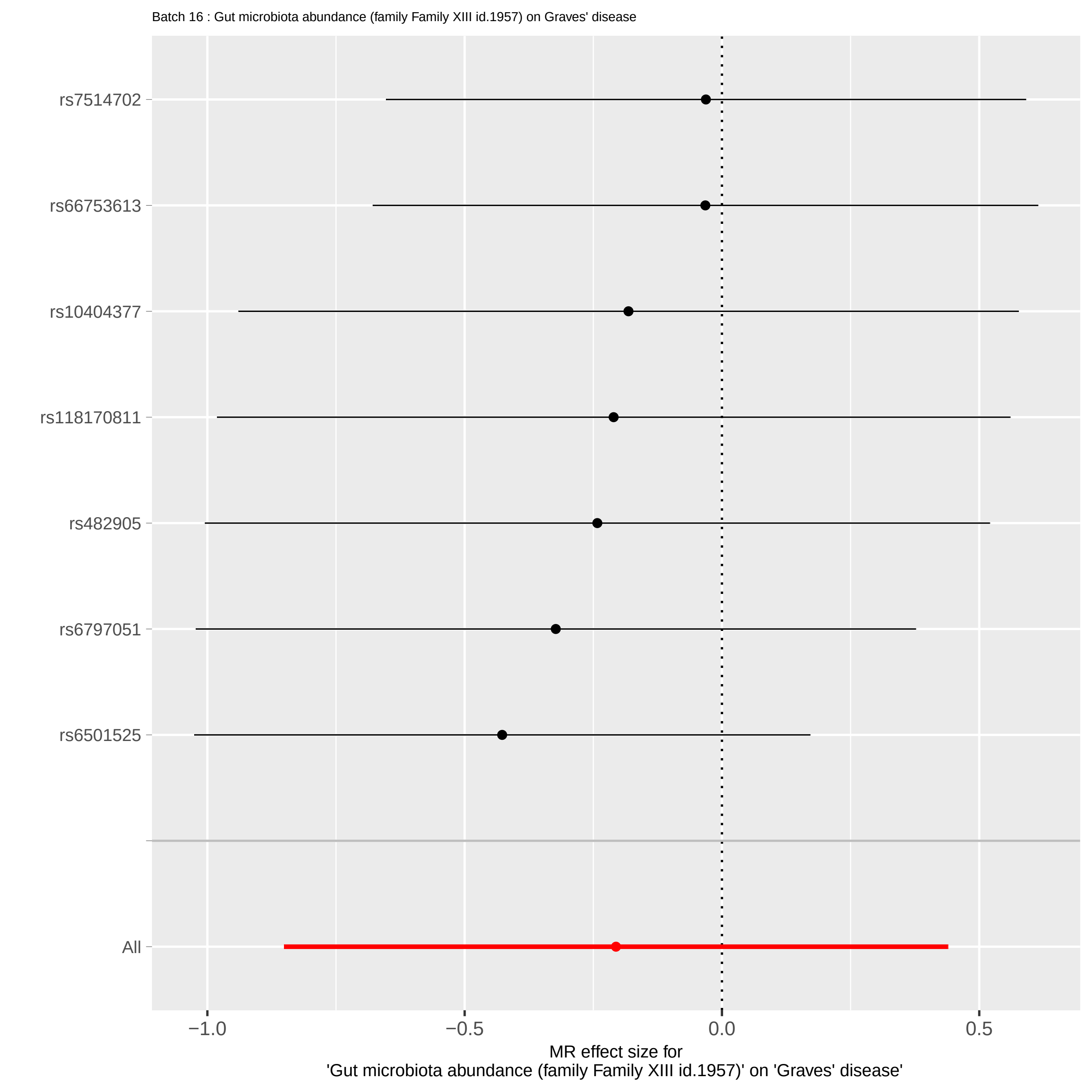
Batch 16 : Gut microbiota abundance (family Family XIII id.1957) on Graves' disease
rs7514702
rs66753613
rs10404377
rs118170811
rs482905
rs6797051
rs6501525
All
−1.0
−0.5
0.0
0.5
MR effect size for
'Gut microbiota abundance (family Family XIII id.1957)' on 'Graves' disease

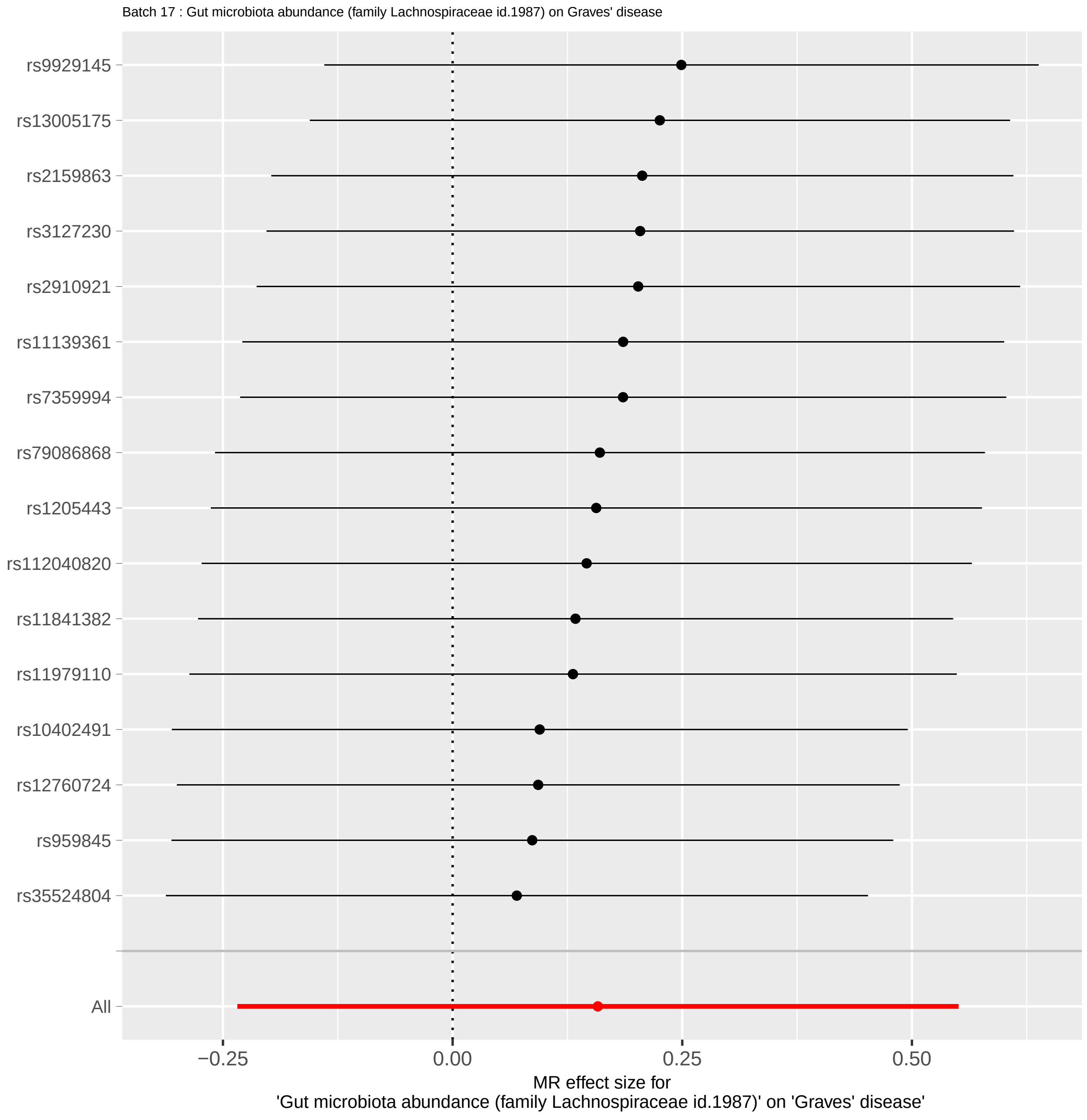

Batch 17 : Gut microbiota abundance (family Lachnospiraceae id.1987) on Graves' disease
rs9929145
rs13005175
rs2159863
rs3127230
rs2910921
rs11139361
rs7359994
rs79086868
rs1205443
rs112040820
rs11841382
rs11979110
rs10402491
rs12760724
rs959845
rs35524804
All
−0.25
0.00
0.25
0.50
MR effect size for
'Gut microbiota abundance (family Lachnospiraceae id.1987)' on 'Graves' disease'

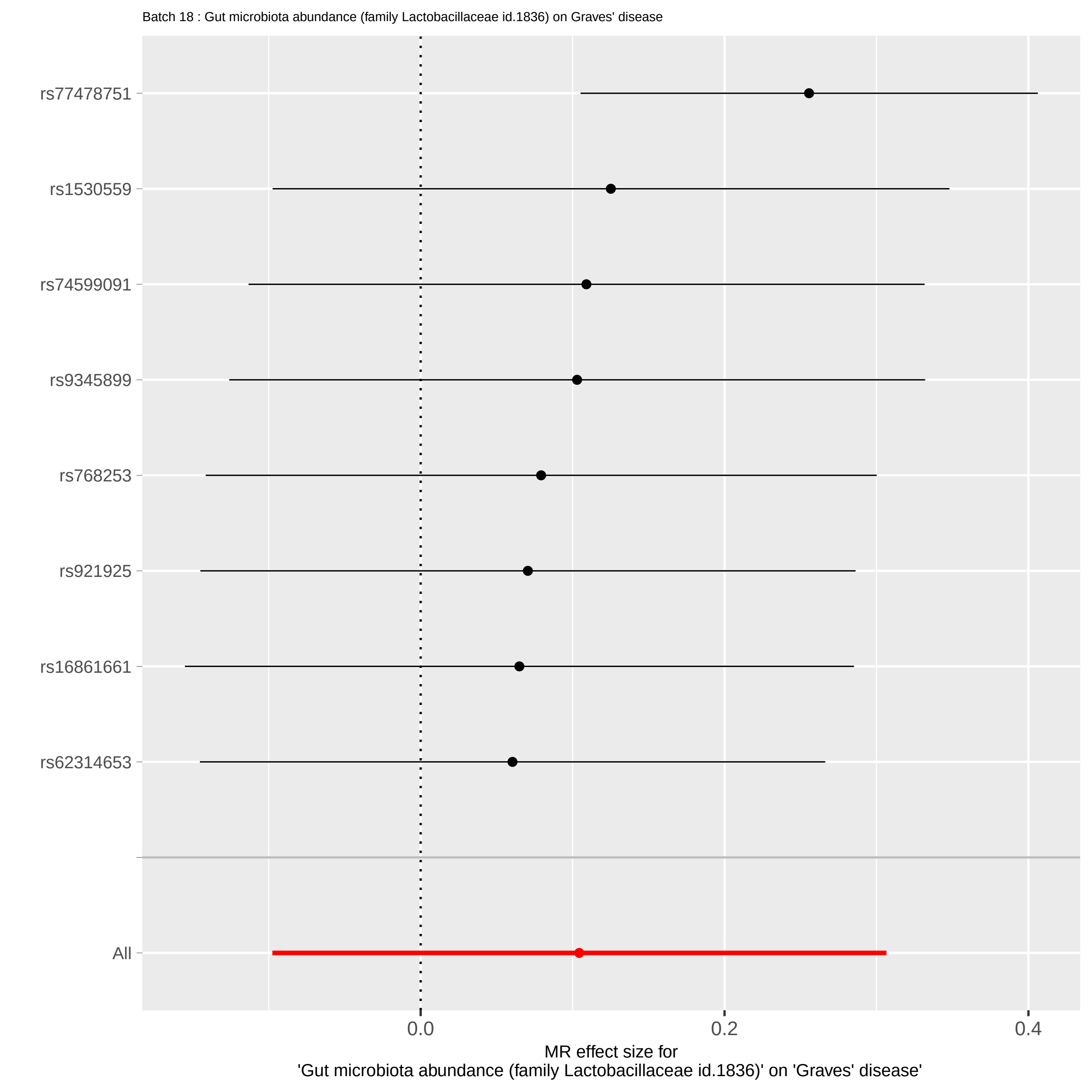

Batch 18 : Gut microbiota abundance (family Lactobacillaceae id.1836) on Graves' disease
rs77478751
rs1530559
rs74599091
rs9345899
rs768253
rs921925
rs16861661
rs62314653
All
0.0
0.2
0.4
MR effect size for
'Gut microbiota abundance (family Lactobacillaceae id.1836)' on 'Graves' disease'

Batch 19 : Gut microbiota abundance (family Methanobacteriaceae id.121) on Graves' disease

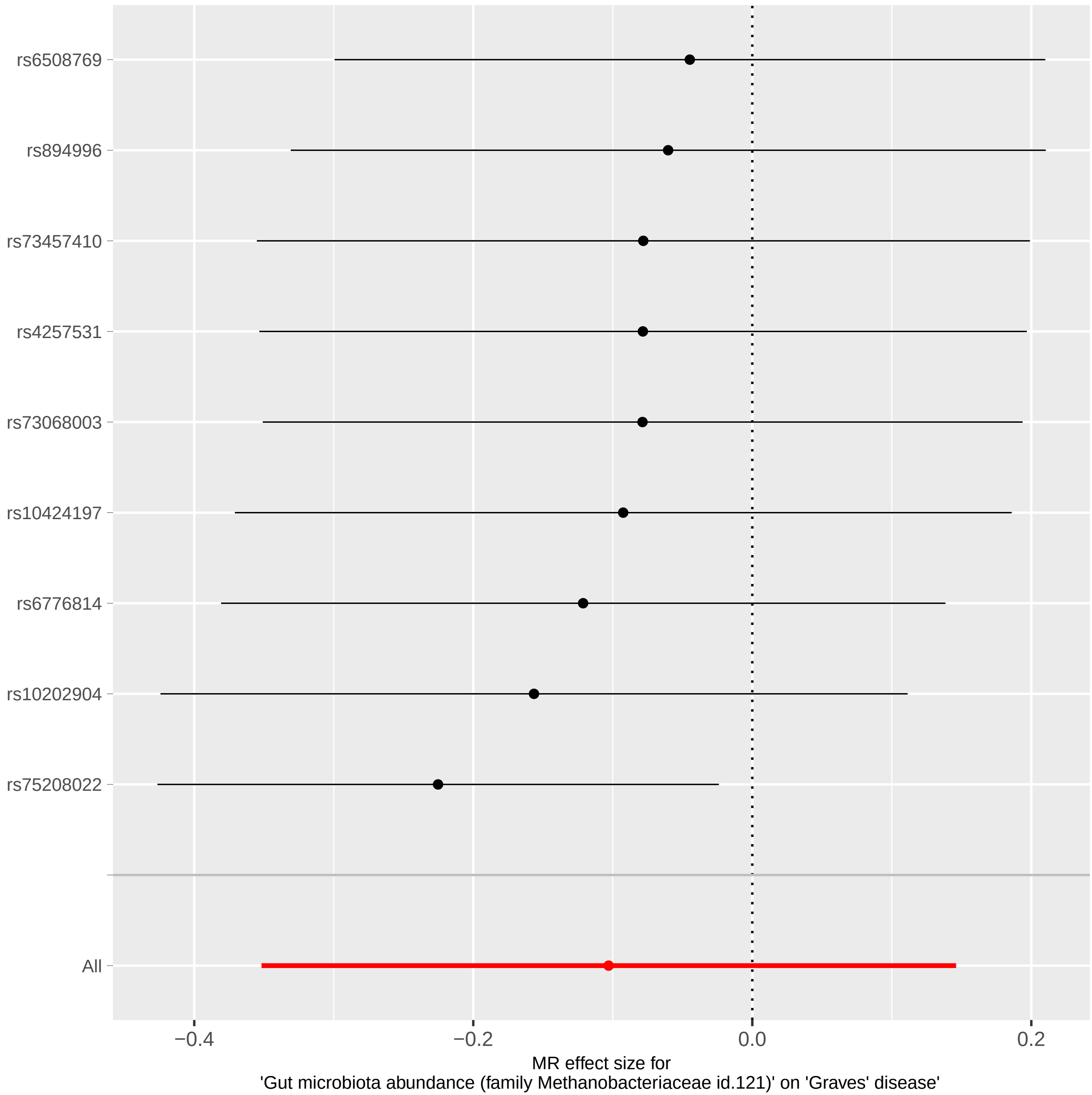

Batch 20 : Gut microbiota abundance (family Oxalobacteraceae id.2966) on Graves' disease

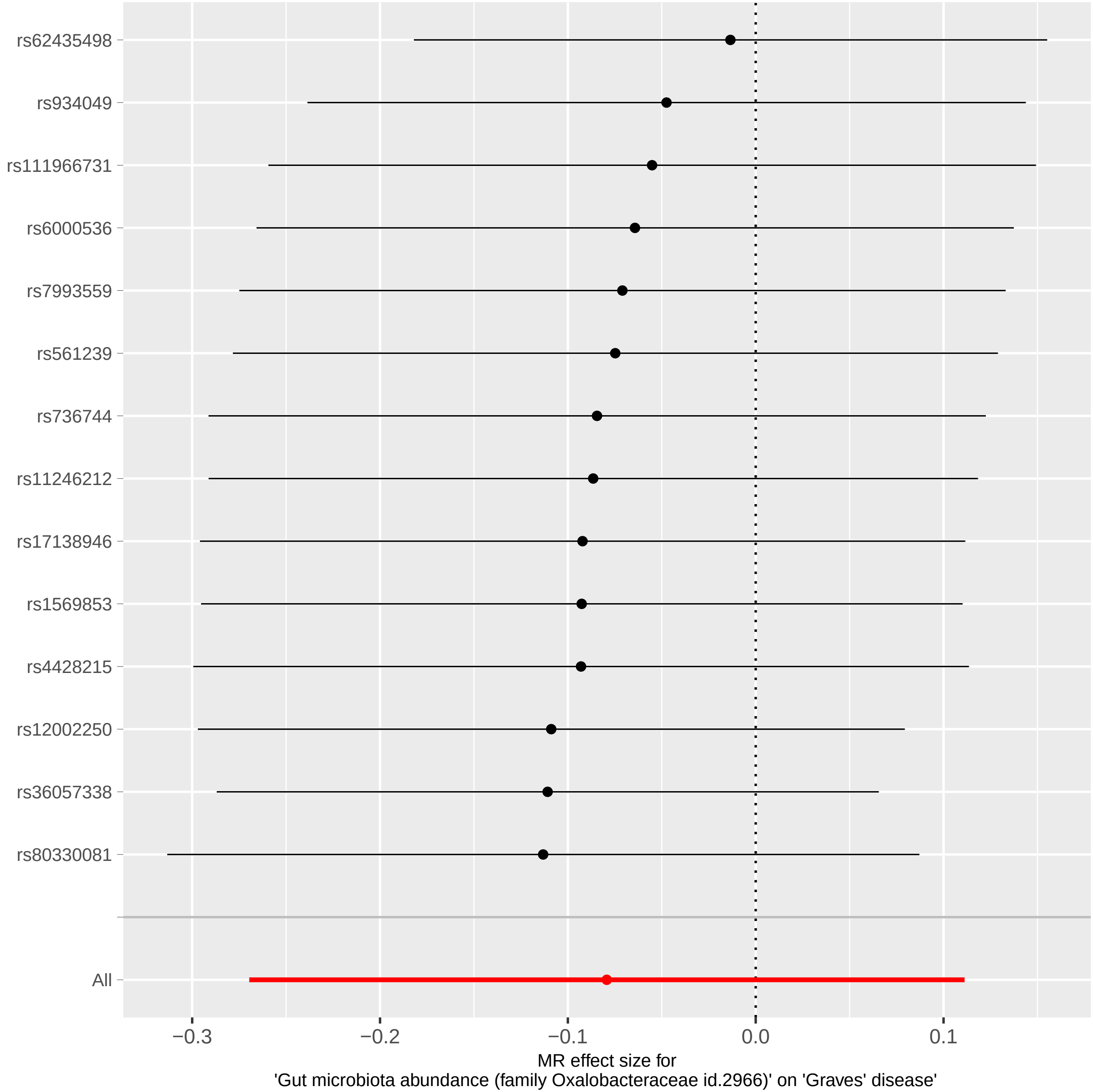

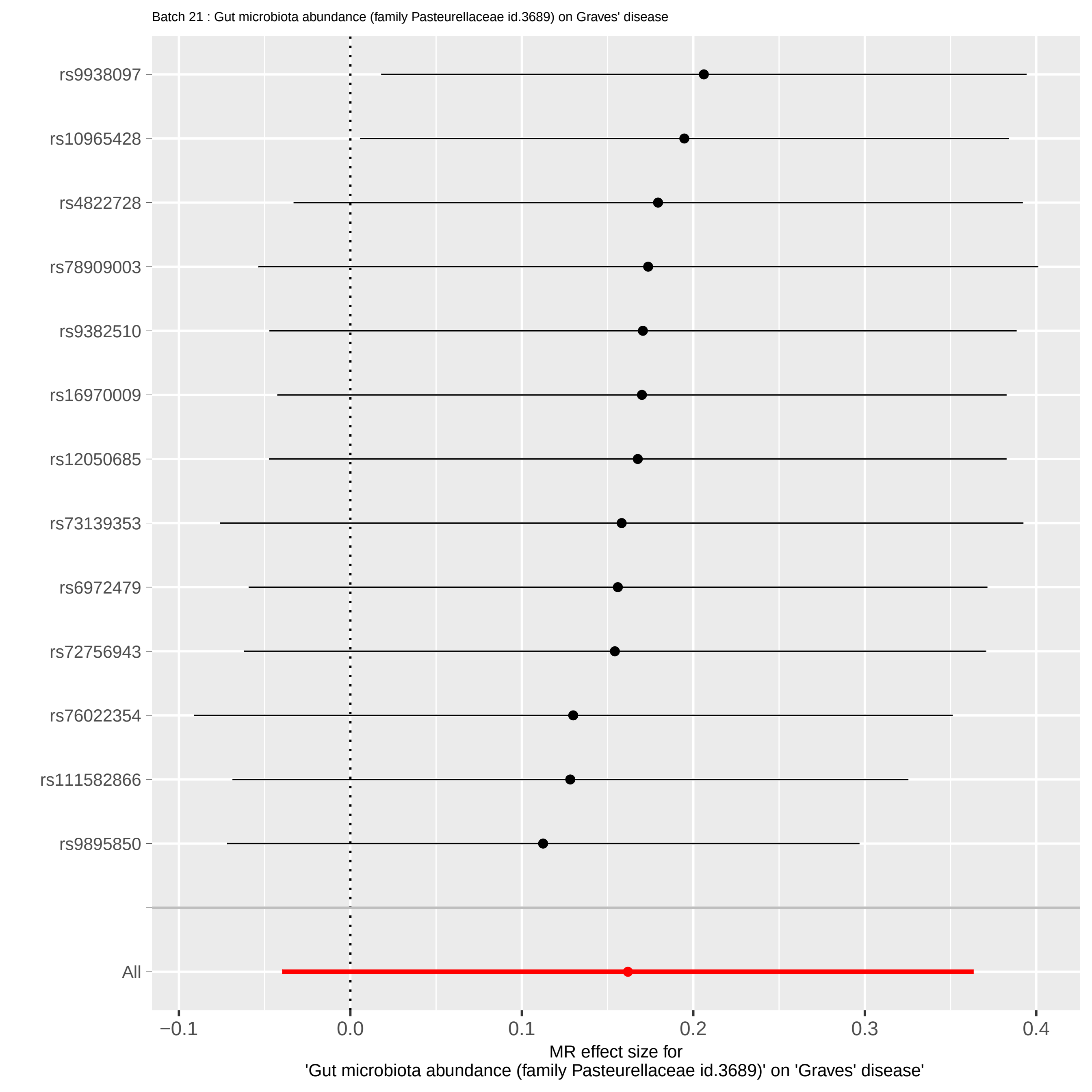

Batch 21 : Gut microbiota abundance (family Pasteurellaceae id.3689) on Graves' disease
rs9938097
rs10965428
rs4822728
rs78909003
rs9382510
rs16970009
rs12050685
rs73139353
rs6972479
rs72756943
rs76022354
rs111582866
rs9895850
All
−0.1
0.0
0.1
0.2
0.3
0.4
MR effect size for
'Gut microbiota abundance (family Pasteurellaceae id.3689)' on 'Graves' disease'

Batch 22 : Gut microbiota abundance (family Peptococcaceae id.2024) on Graves' disease

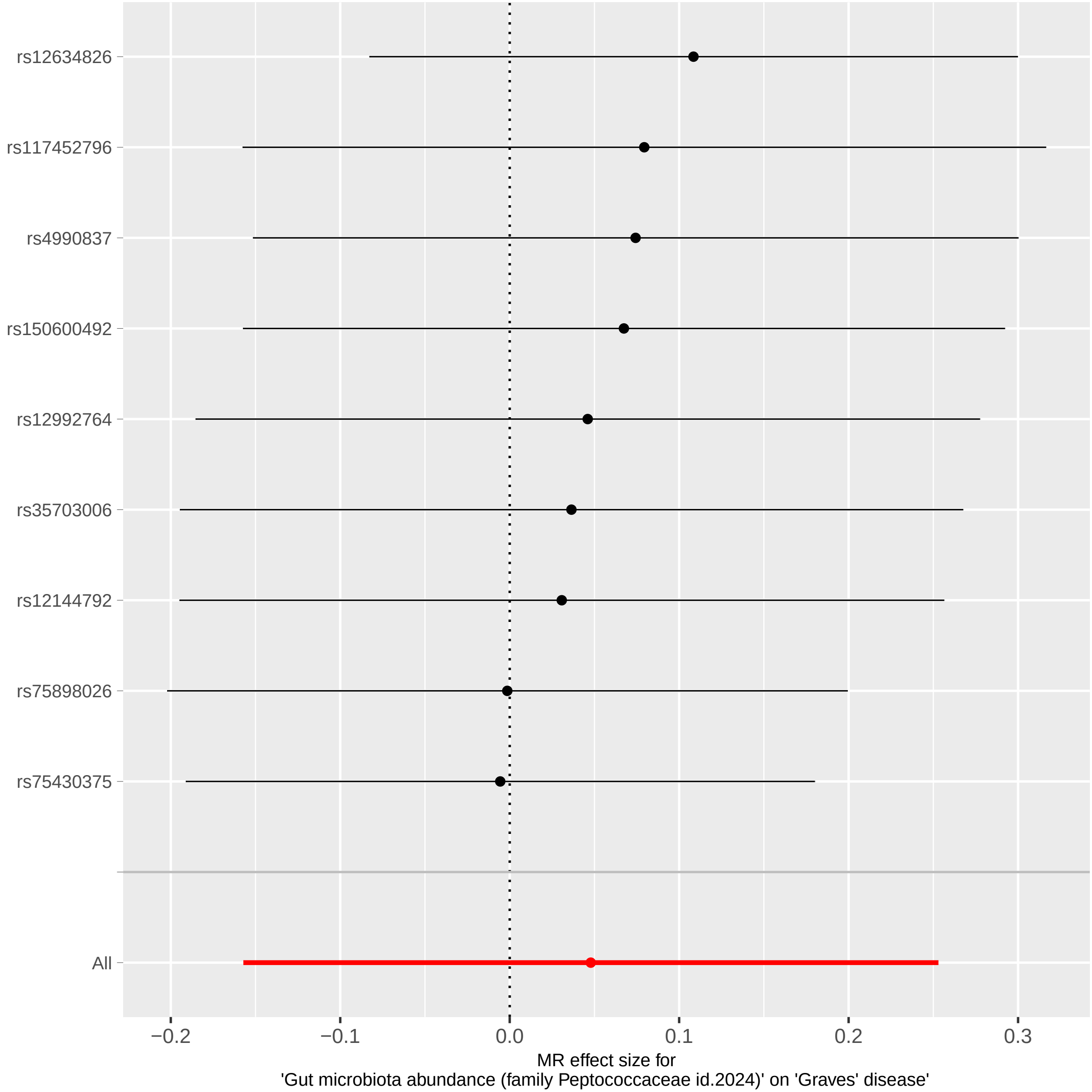

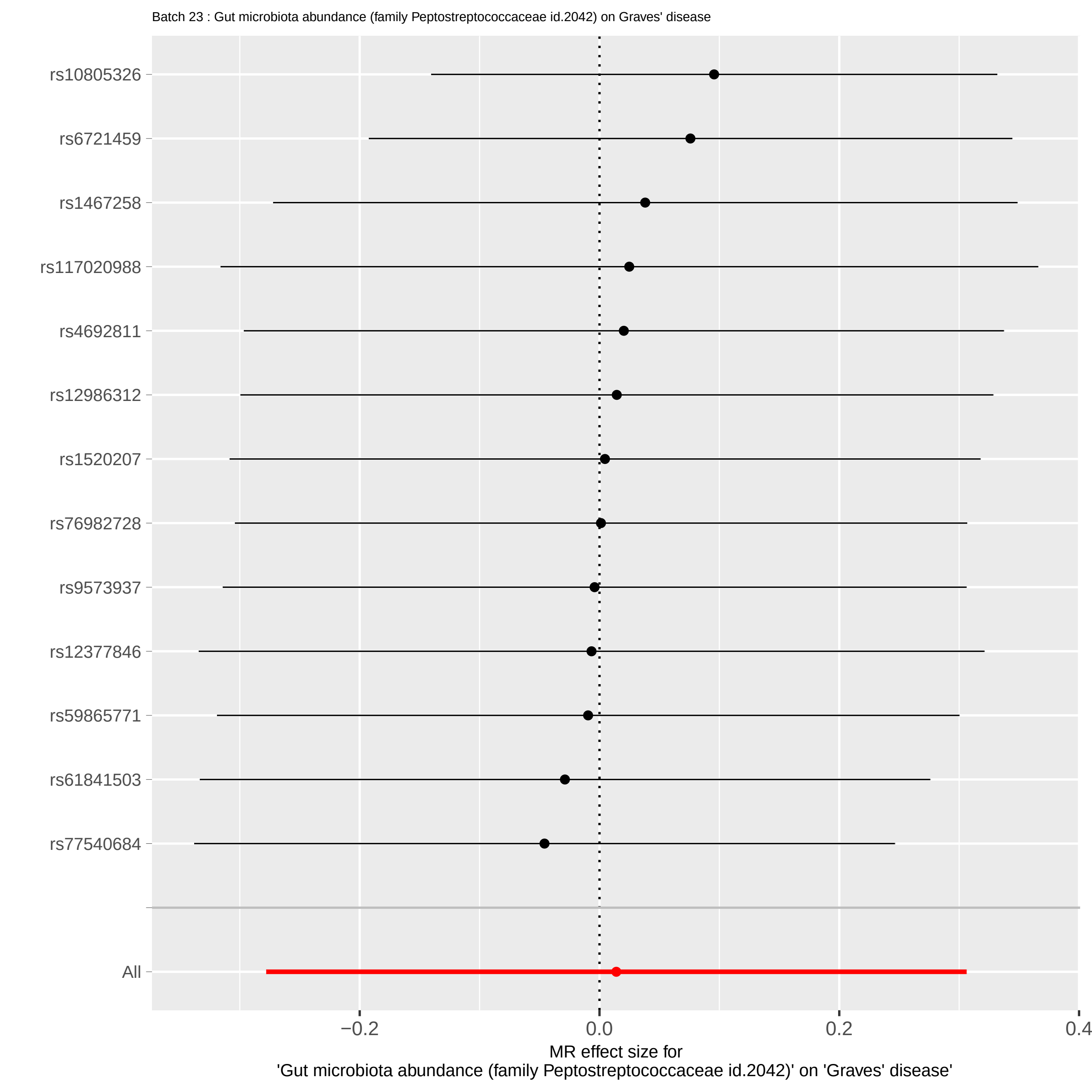

Batch 23 : Gut microbiota abundance (family Peptostreptococcaceae id.2042) on Graves' disease
rs10805326
rs6721459
rs1467258
rs117020988
rs4692811
rs12986312
rs1520207
rs76982728
rs9573937
rs12377846
rs59865771
rs61841503
rs77540684
All
−0.2
0.0
0.2
0.4
MR effect size for
'Gut microbiota abundance (family Peptostreptococcaceae id.2042)' on 'Graves' disease'

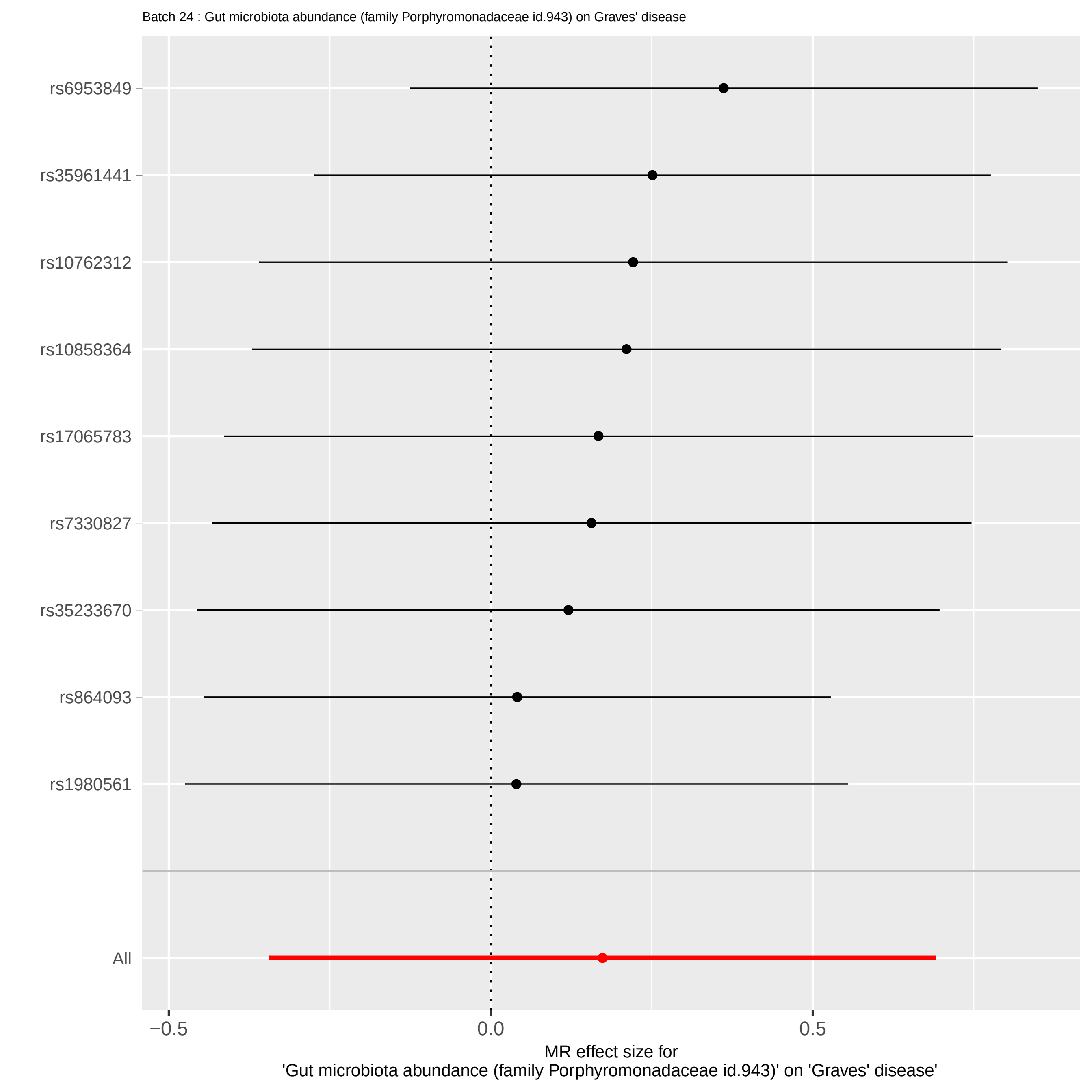

Batch 24 : Gut microbiota abundance (family Porphyromonadaceae id.943) on Graves' disease
rs6953849
rs35961441
rs10762312
rs10858364
rs17065783
rs7330827
rs35233670
rs864093
rs1980561
All
−0.5
0.0
0.5
MR effect size for
'Gut microbiota abundance (family Porphyromonadaceae id.943)' on 'Graves' disease'

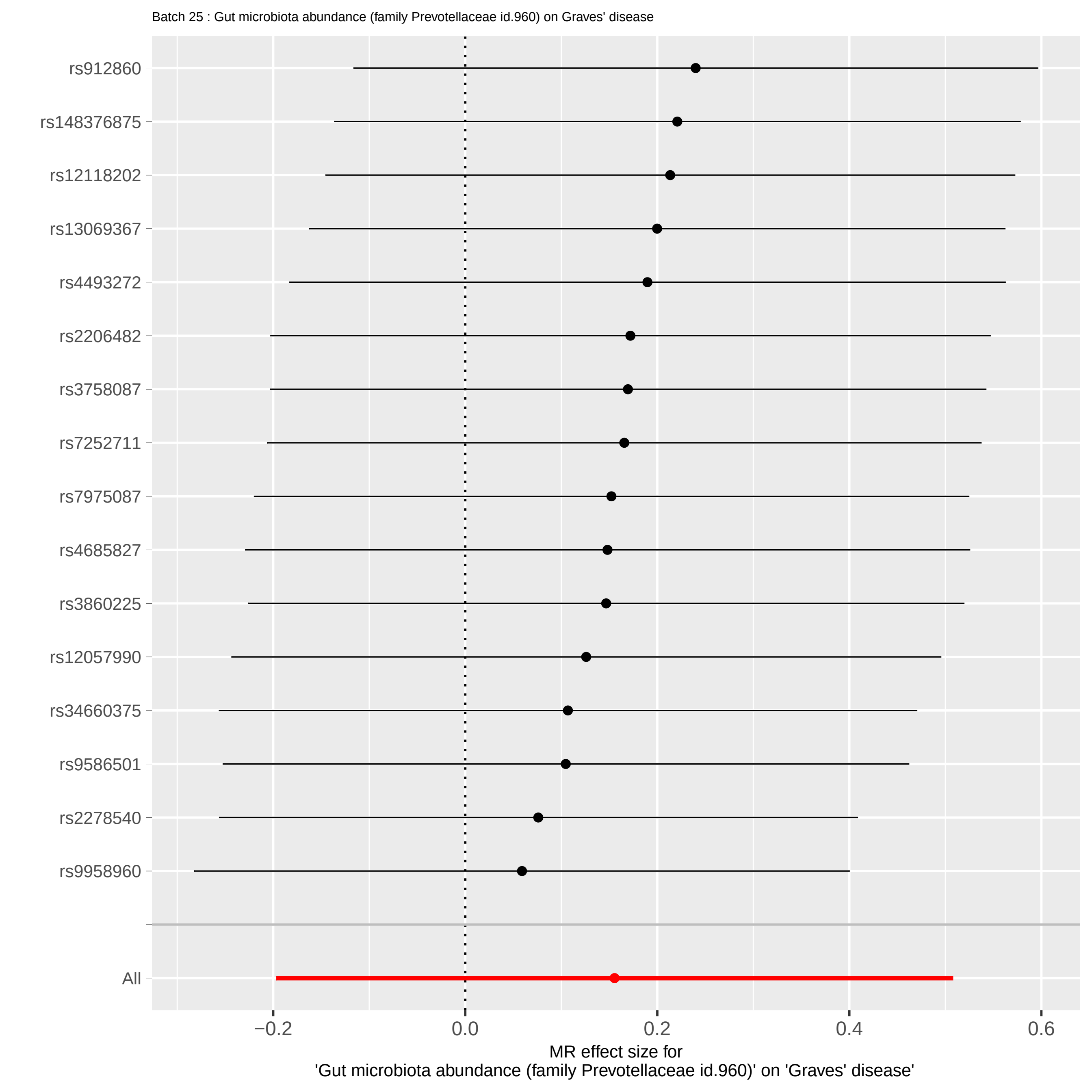

Batch 25 : Gut microbiota abundance (family Prevotellaceae id.960) on Graves' disease
rs912860
rs148376875
rs12118202
rs13069367
rs4493272
rs2206482
rs3758087
rs7252711
rs7975087
rs4685827
rs3860225
rs12057990
rs34660375
rs9586501
rs2278540
rs9958960
All
−0.2
0.0
0.2
0.4
0.6
MR effect size for
'Gut microbiota abundance (family Prevotellaceae id.960)' on 'Graves' disease'

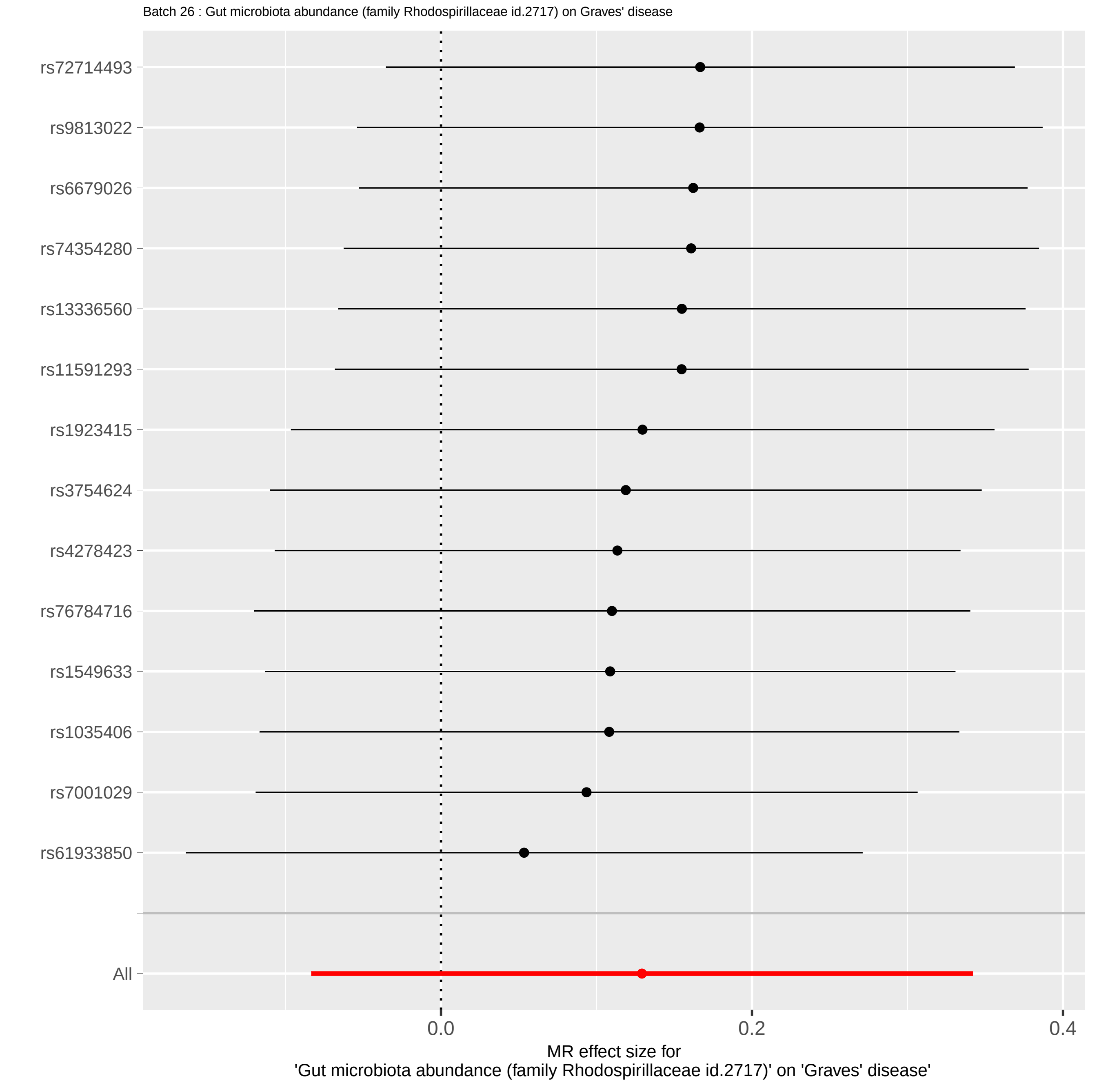

Batch 26 : Gut microbiota abundance (family Rhodospirillaceae id.2717) on Graves' disease
rs72714493
rs9813022
rs6679026
rs74354280
rs13336560
rs11591293
rs1923415
rs3754624
rs4278423
rs76784716
rs1549633
rs1035406
rs7001029
rs61933850
All
0.0
0.2
0.4
MR effect size for
'Gut microbiota abundance (family Rhodospirillaceae id.2717)' on 'Graves' disease'

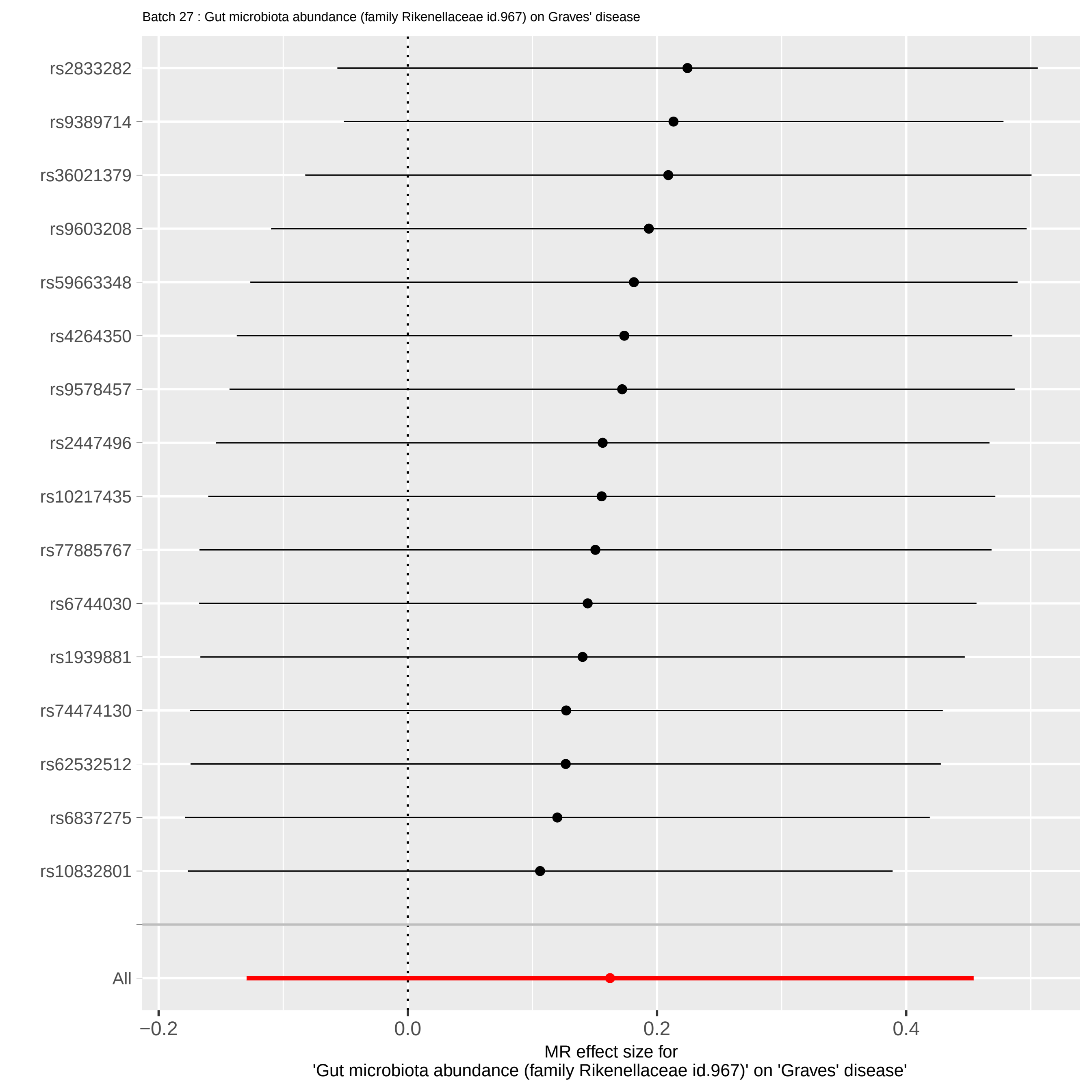

Batch 27 : Gut microbiota abundance (family Rikenellaceae id.967) on Graves' disease
rs2833282
rs9389714
rs36021379
rs9603208
rs59663348
rs4264350
rs9578457
rs2447496
rs10217435
rs77885767
rs6744030
rs1939881
rs74474130
rs62532512
rs6837275
rs10832801
All
−0.2
0.0
0.2
0.4
MR effect size for
'Gut microbiota abundance (family Rikenellaceae id.967)' on 'Graves' disease'

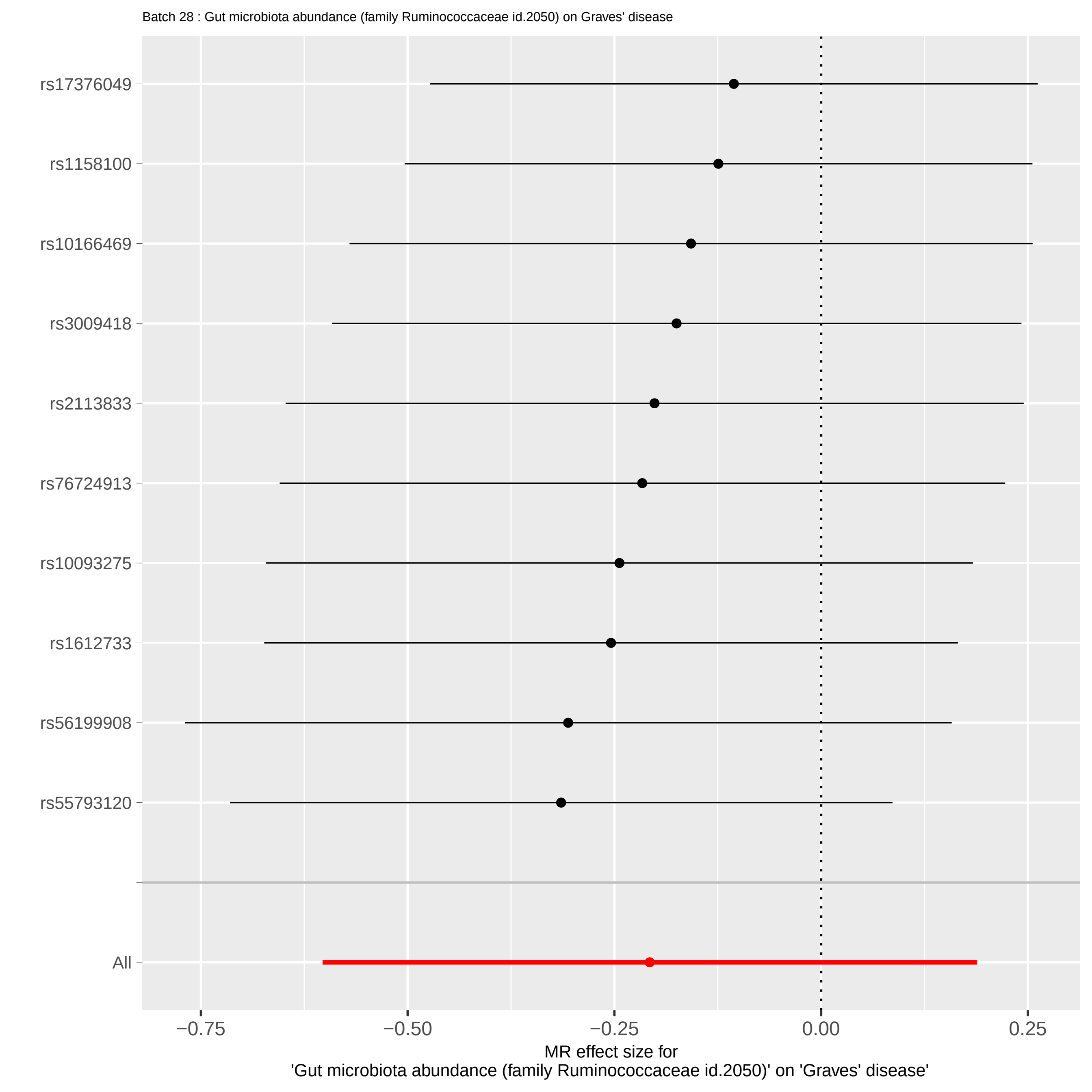

Batch 28 : Gut microbiota abundance (family Ruminococcaceae id.2050) on Graves' disease
rs17376049
rs1158100
rs10166469
rs3009418
rs2113833
rs76724913
rs10093275
rs1612733
rs56199908
rs55793120
All
−0.75
−0.50
−0.25
0.00
0.25
MR effect size for
'Gut microbiota abundance (family Ruminococcaceae id.2050)' on 'Graves' disease'

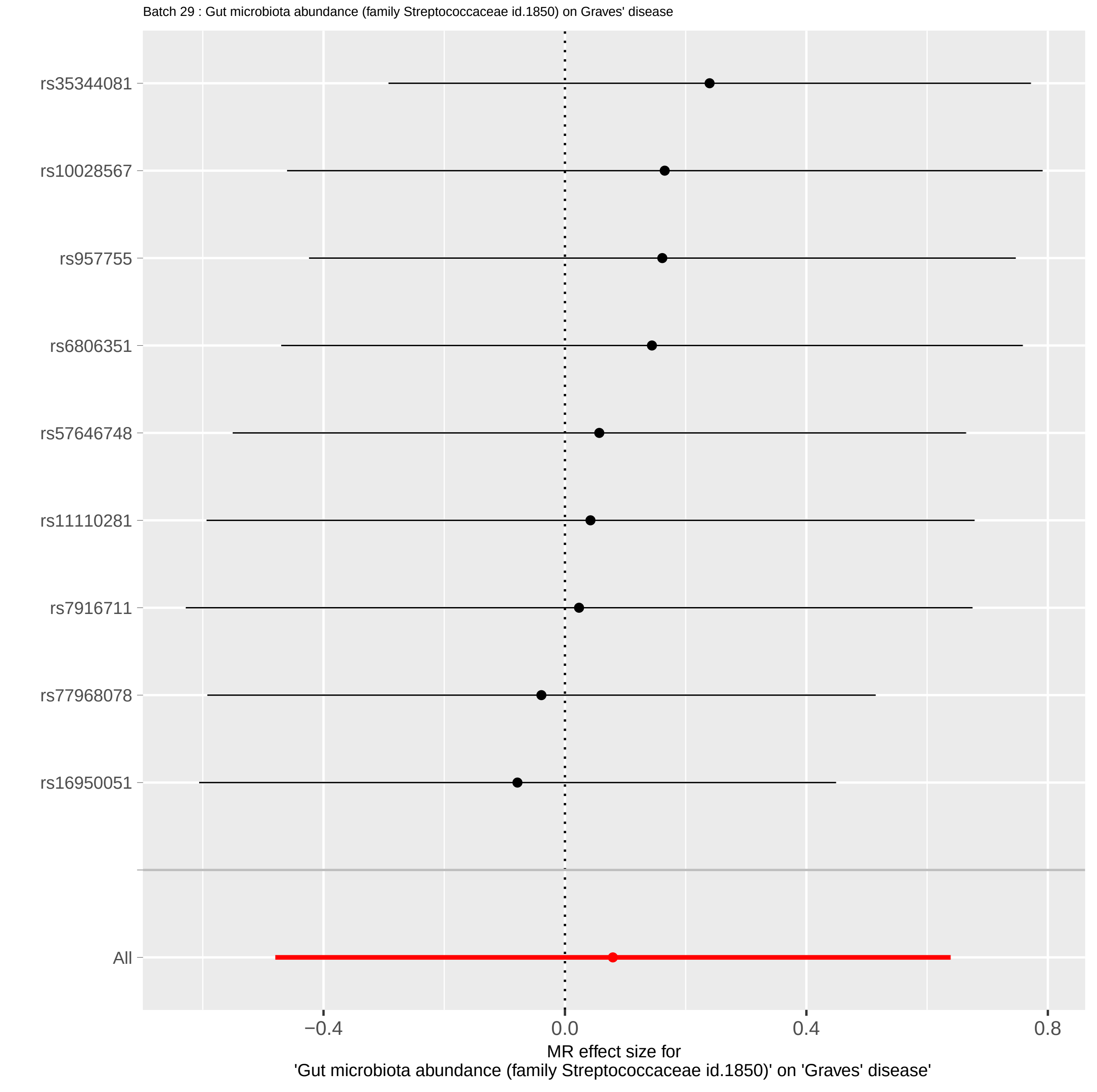

Batch 29 : Gut microbiota abundance (family Streptococcaceae id.1850) on Graves' disease
rs35344081
rs10028567
rs957755
rs6806351
rs57646748
rs11110281
rs7916711
rs77968078
rs16950051
All
−0.4
0.0
0.4
0.8
MR effect size for
'Gut microbiota abundance (family Streptococcaceae id.1850)' on 'Graves' disease'

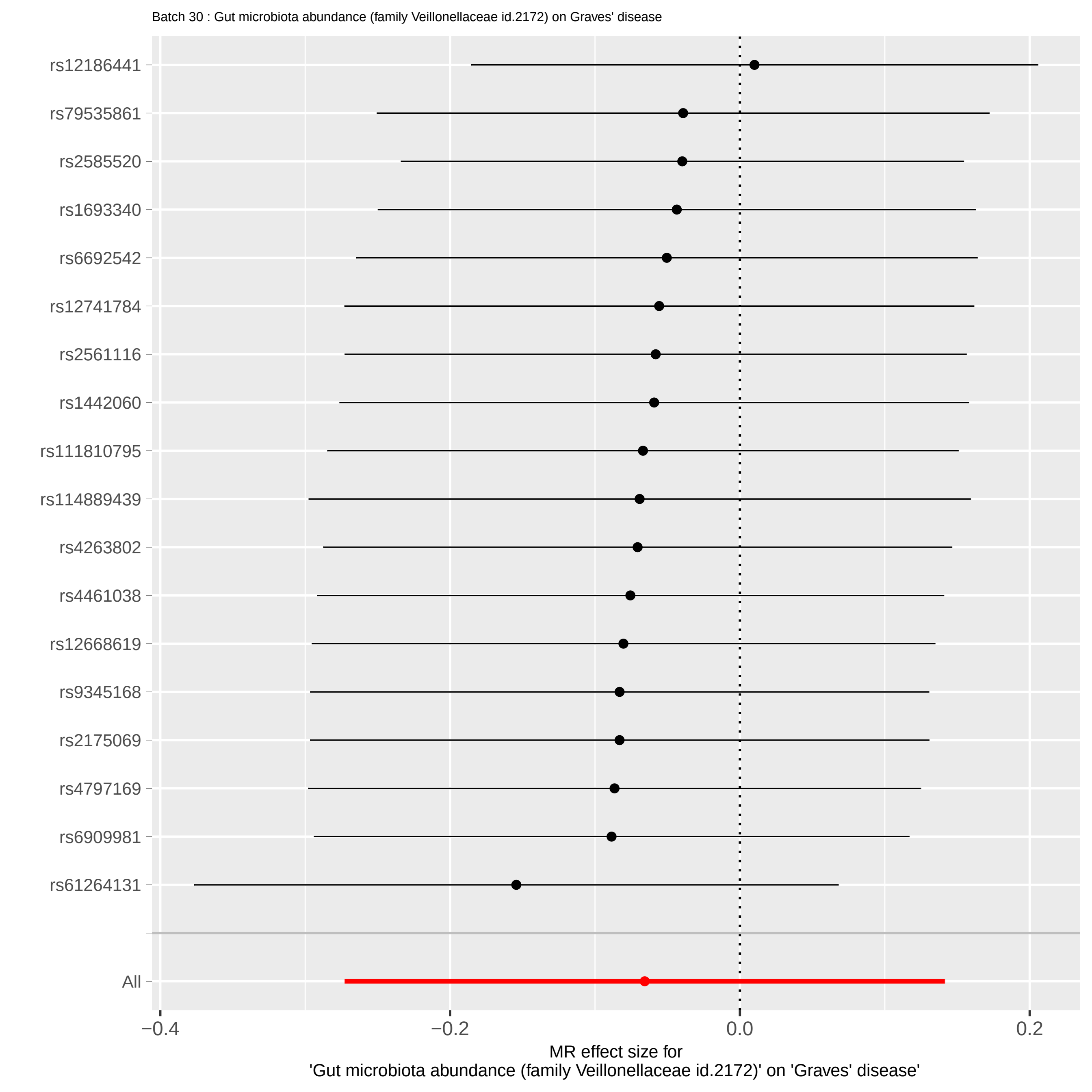

Batch 30 : Gut microbiota abundance (family Veillonellaceae id.2172) on Graves' disease
rs12186441
rs79535861
rs2585520
rs1693340
rs6692542
rs12741784
rs2561116
rs1442060
rs111810795
rs114889439
rs4263802
rs4461038
rs12668619
rs9345168
rs2175069
rs4797169
rs6909981
rs61264131
All
−0.4
−0.2
0.0
0.2
MR effect size for
'Gut microbiota abundance (family Veillonellaceae id.2172)' on 'Graves' disease'

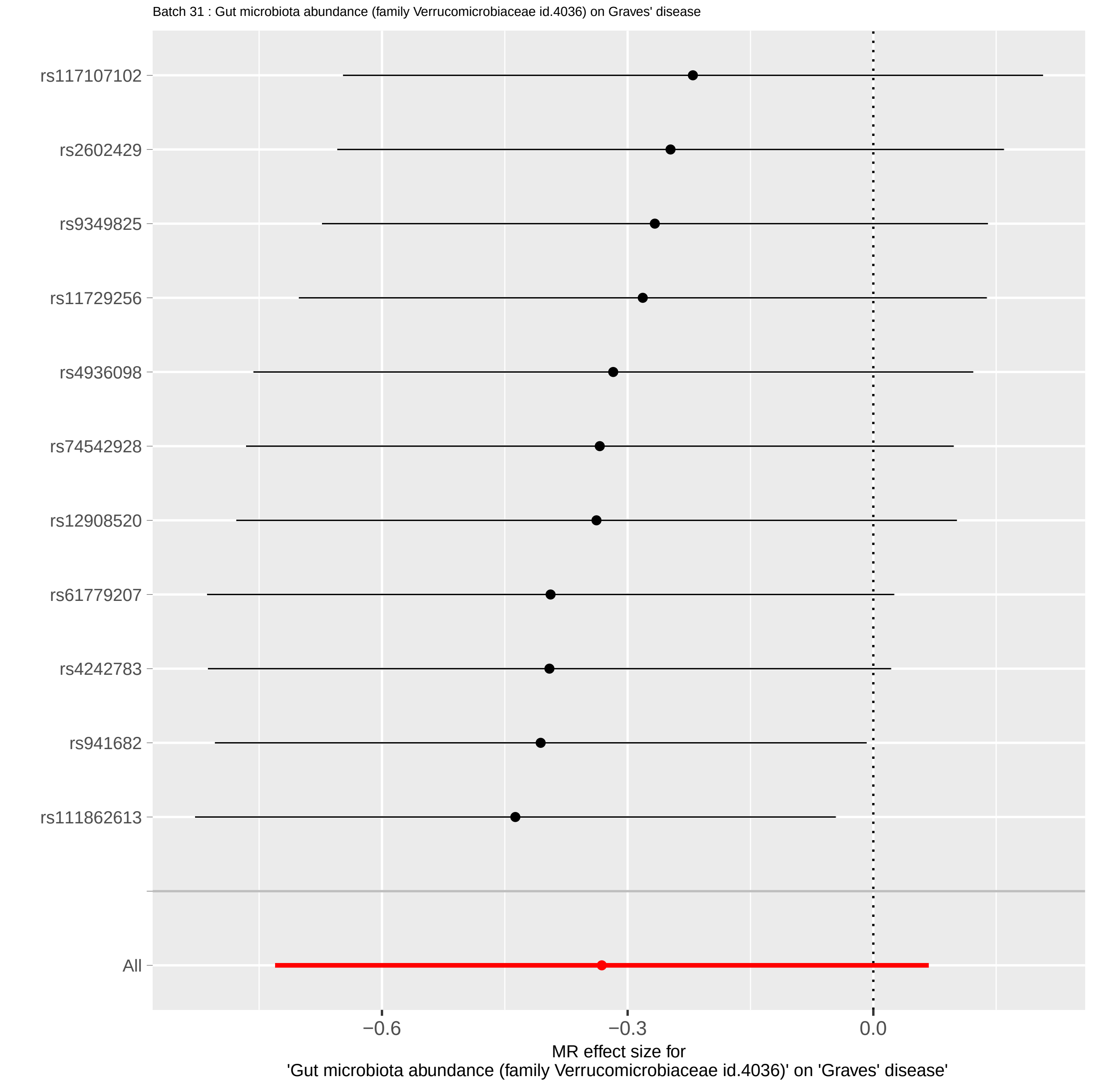
Batch 31 : Gut microbiota abundance (family Verrucomicrobiaceae id.4036) on Graves' disease
rs117107102
rs2602429
rs9349825
rs11729256
rs4936098
rs74542928
rs12908520
rs61779207
rs4242783
rs941682
rs111862613
All
−0.6
−0.3
0.0
MR effect size for
'Gut microbiota abundance (family Verrucomicrobiaceae id.4036)' on 'Graves' disease'

Batch 32 : Gut microbiota abundance (family Victivallaceae id.2255) on Graves' disease

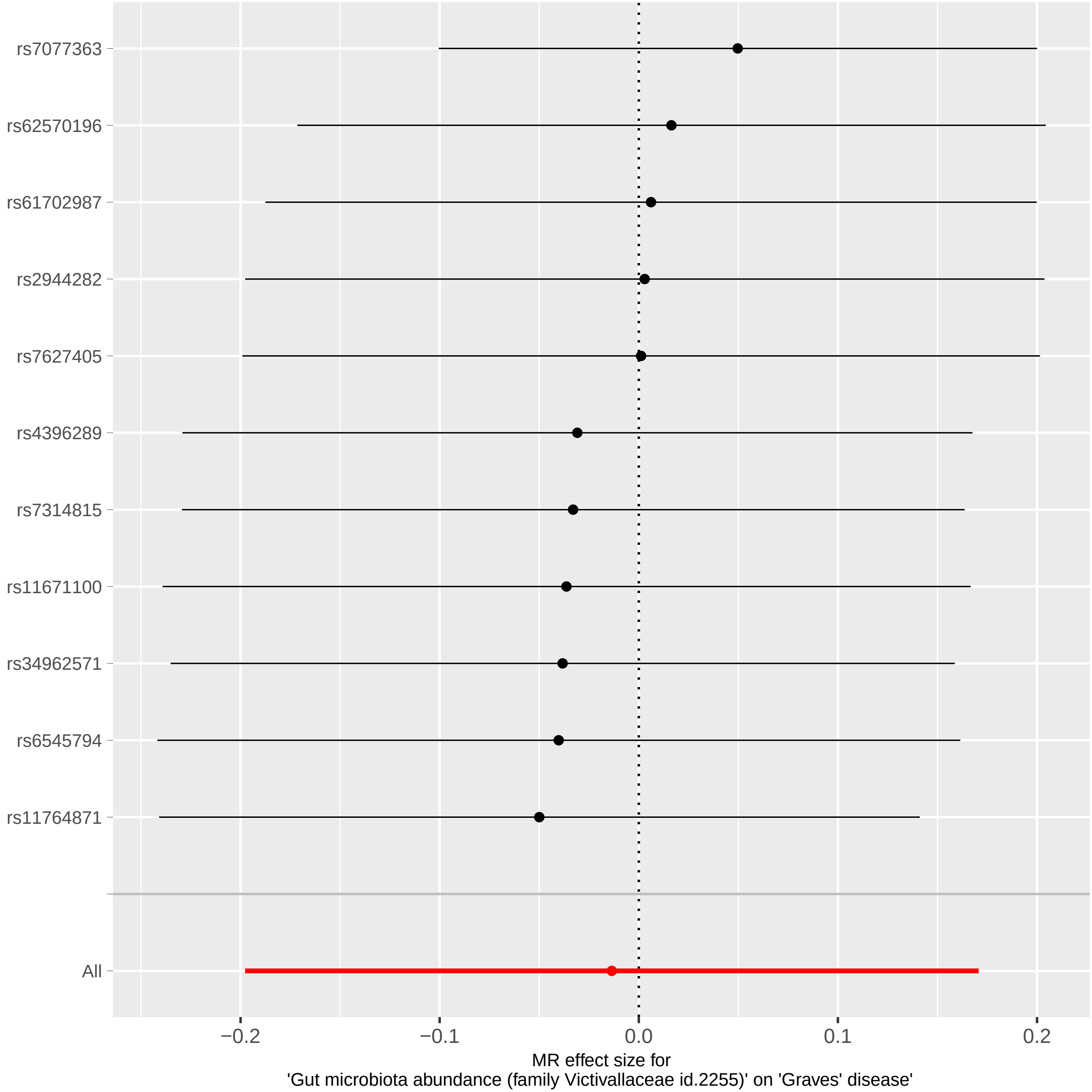

Batch 33 : Gut microbiota abundance (genus Actinomyces id.423) on Graves' disease

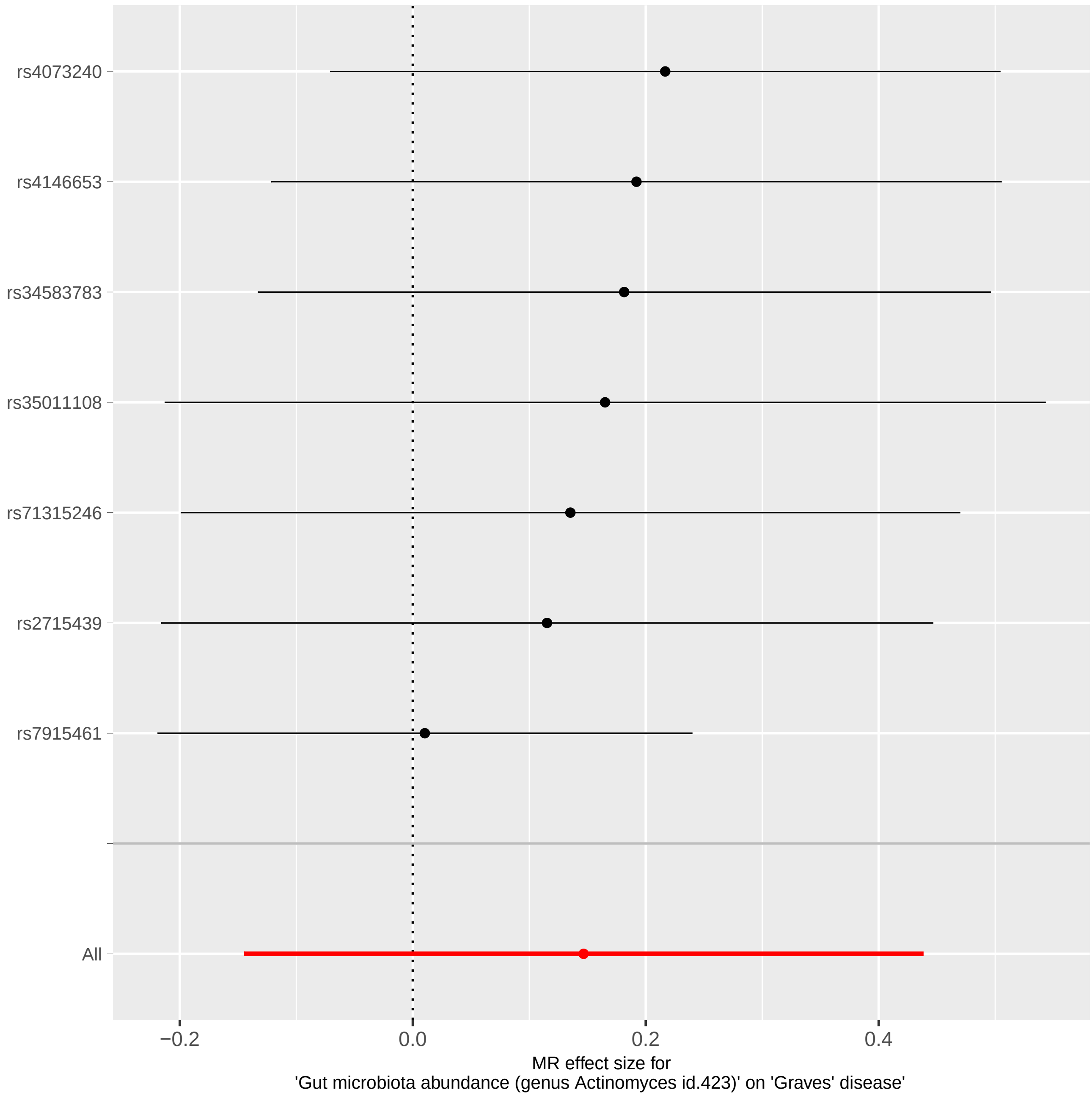

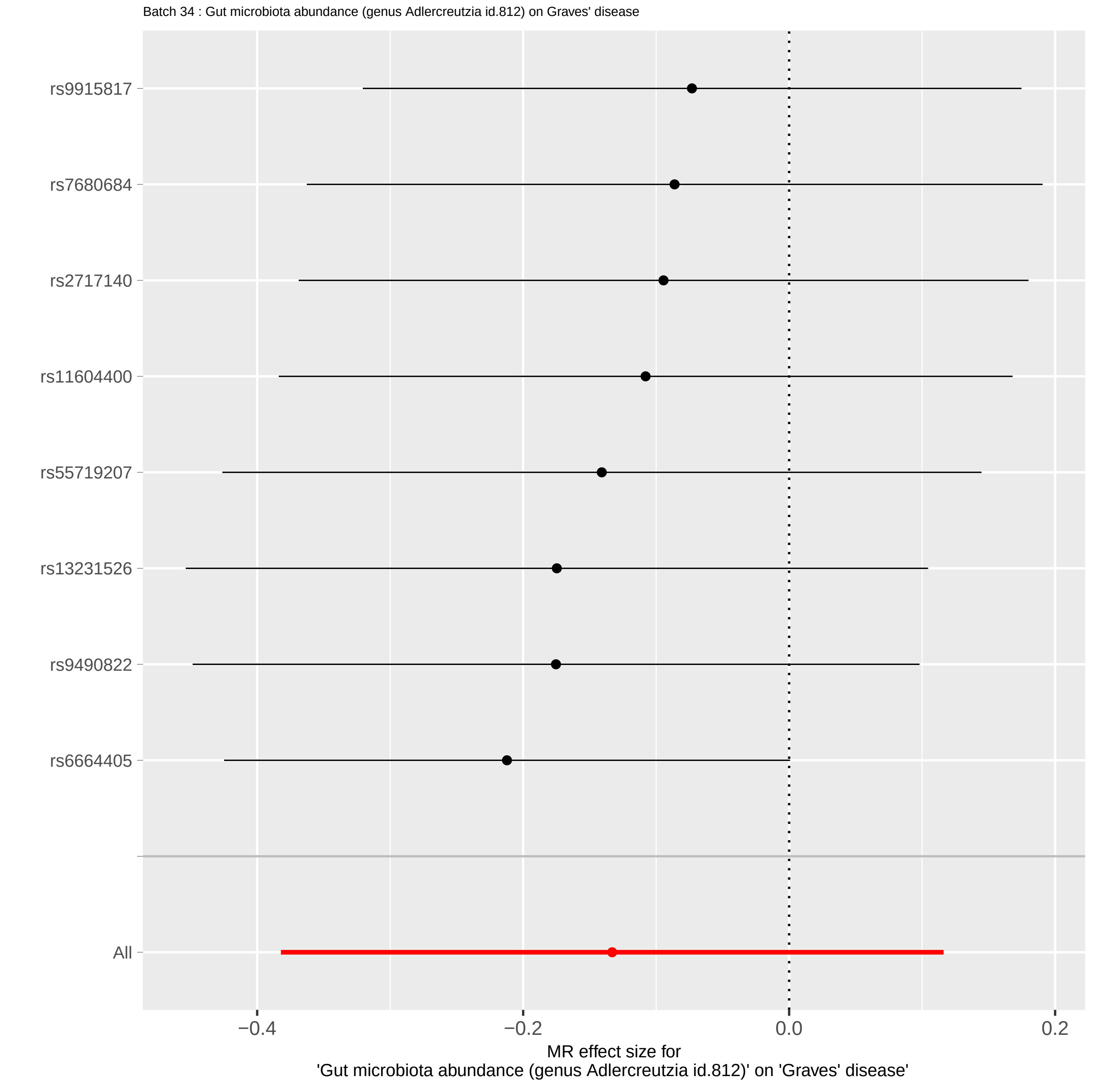

Batch 34 : Gut microbiota abundance (genus Adlercreutzia id.812) on Graves' disease
rs9915817
rs7680684
rs2717140
rs11604400
rs55719207
rs13231526
rs9490822
rs6664405
All
−0.4
−0.2
0.0
0.2
MR effect size for
'Gut microbiota abundance (genus Adlercreutzia id.812)' on 'Graves' disease'

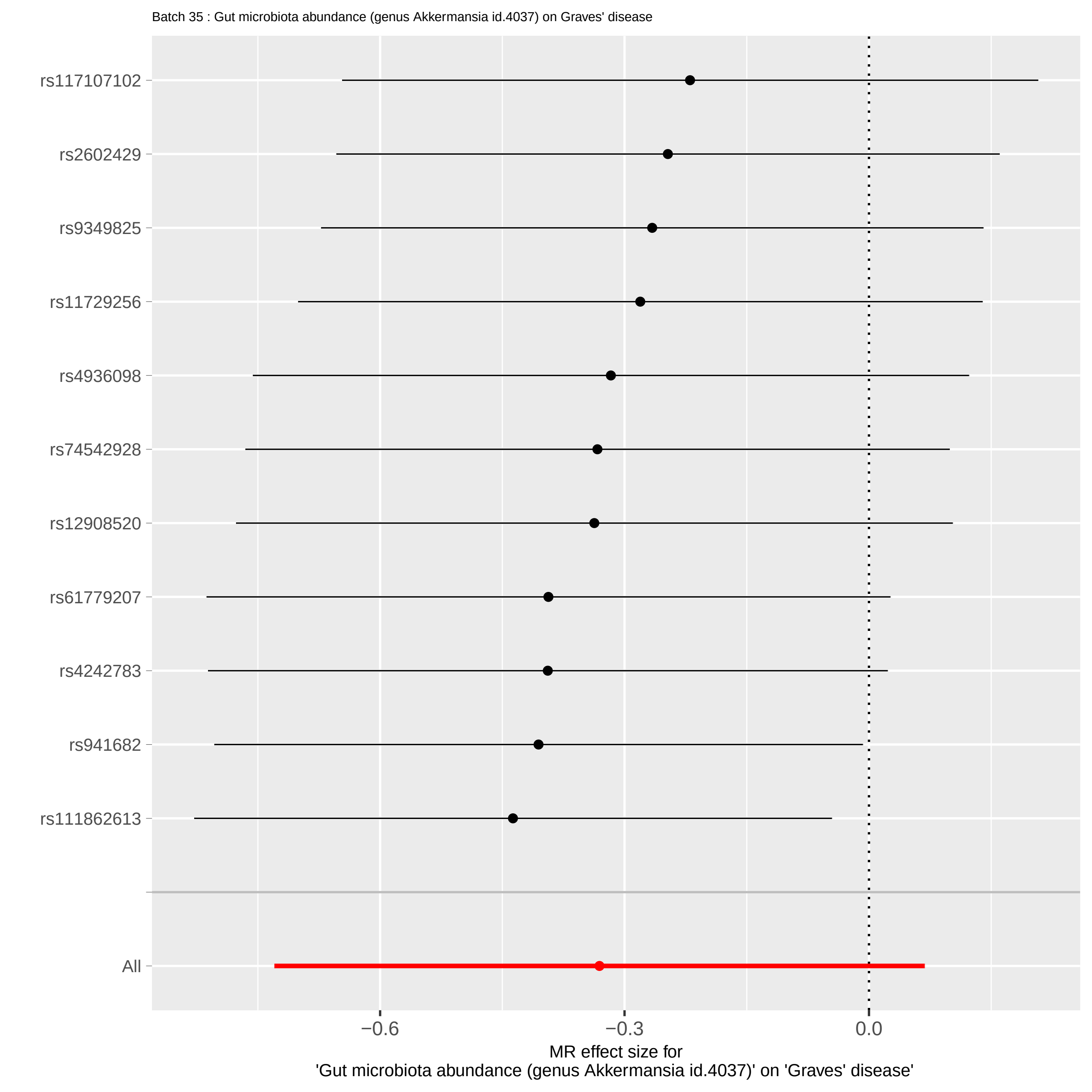
Batch 35 : Gut microbiota abundance (genus Akkermansia id.4037) on Graves' disease
rs117107102
rs2602429
rs9349825
rs11729256
rs4936098
rs74542928
rs12908520
rs61779207
rs4242783
rs941682
rs111862613
All
−0.6
−0.3
0.0
MR effect size for
'Gut microbiota abundance (genus Akkermansia id.4037)' on 'Graves' disease'

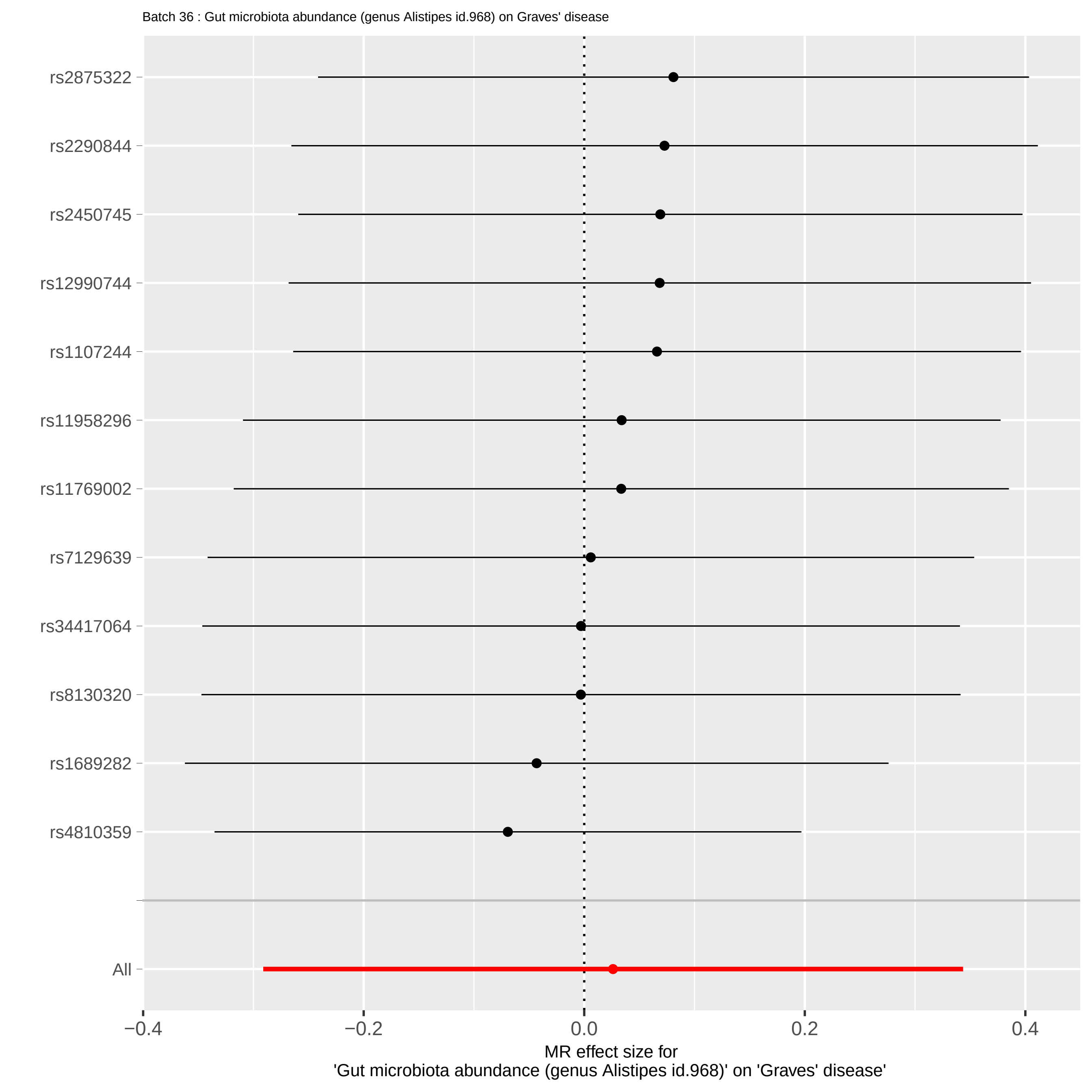
Batch 36 : Gut microbiota abundance (genus Alistipes id.968) on Graves' disease
rs2875322
rs2290844
rs2450745
rs12990744
rs1107244
rs11958296
rs11769002
rs7129639
rs34417064
rs8130320
rs1689282
rs4810359
All
−0.4
−0.2
0.0
0.2
0.4
MR effect size for
'Gut microbiota abundance (genus Alistipes id.968)' on 'Graves' disease'

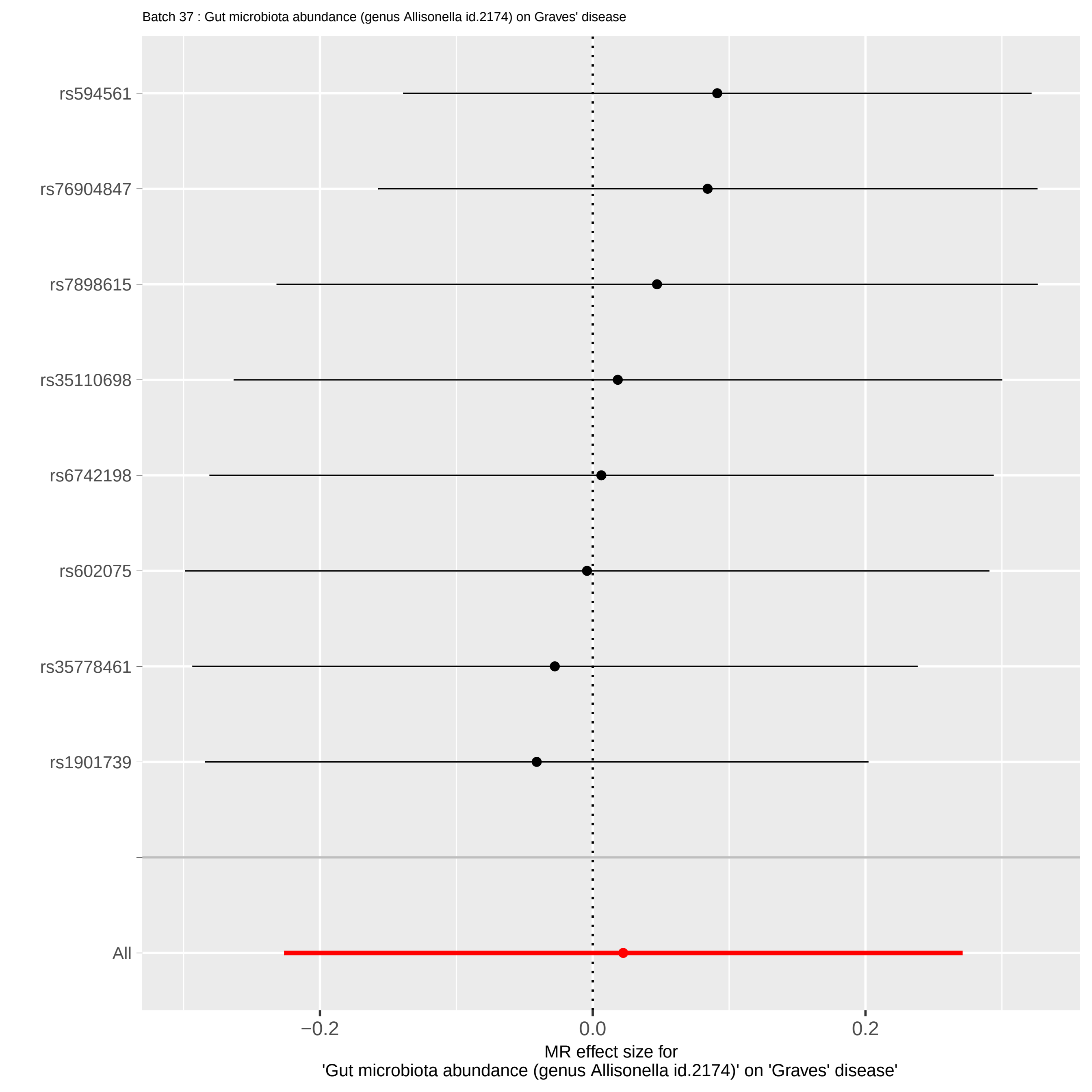

Batch 37 : Gut microbiota abundance (genus Allisonella id.2174) on Graves' disease
rs594561
rs76904847
rs7898615
rs35110698
rs6742198
rs602075
rs35778461
rs1901739
All
−0.2
0.0
0.2
MR effect size for
'Gut microbiota abundance (genus Allisonella id.2174)' on 'Graves' disease'

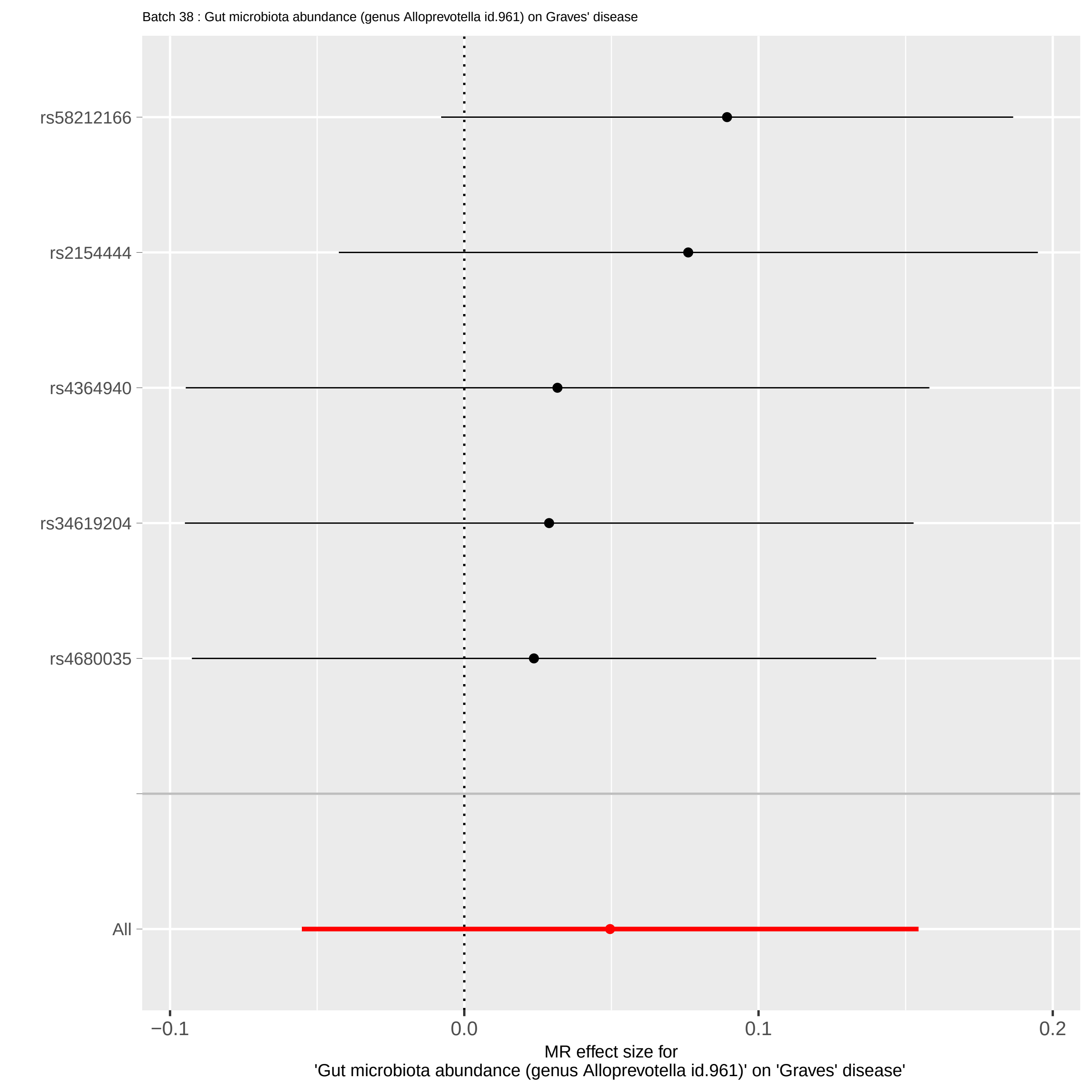
Batch 38 : Gut microbiota abundance (genus Alloprevotella id.961) on Graves' disease
rs58212166
rs2154444
rs4364940
rs34619204
rs4680035
All
−0.1
0.0
0.1
0.2
MR effect size for
'Gut microbiota abundance (genus Alloprevotella id.961)' on 'Graves' disease'

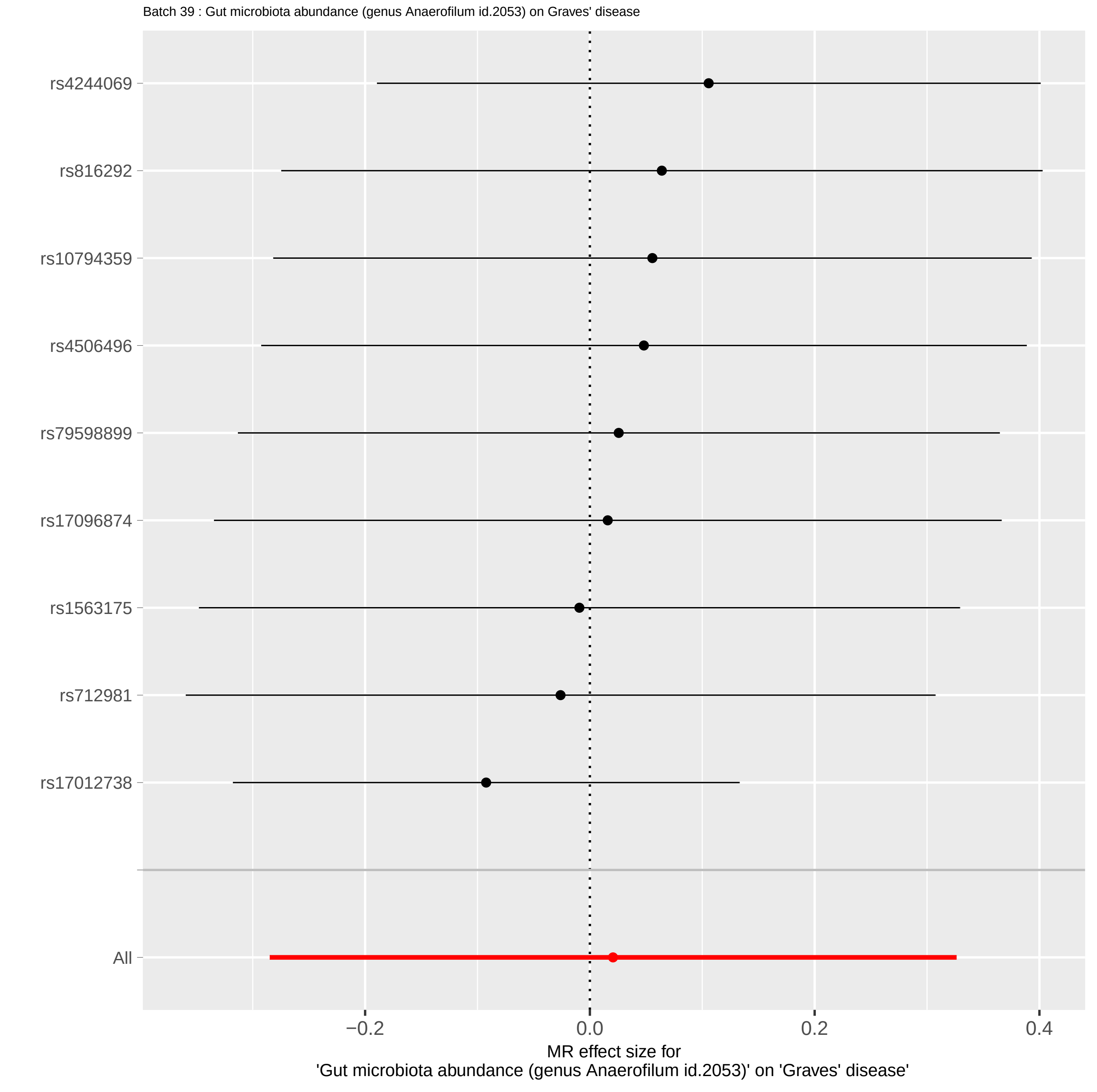

Batch 39 : Gut microbiota abundance (genus Anaerofilum id.2053) on Graves' disease
rs4244069
rs816292
rs10794359
rs4506496
rs79598899
rs17096874
rs1563175
rs712981
rs17012738
All
−0.2
0.0
0.2
0.4
MR effect size for
'Gut microbiota abundance (genus Anaerofilum id.2053)' on 'Graves' disease'

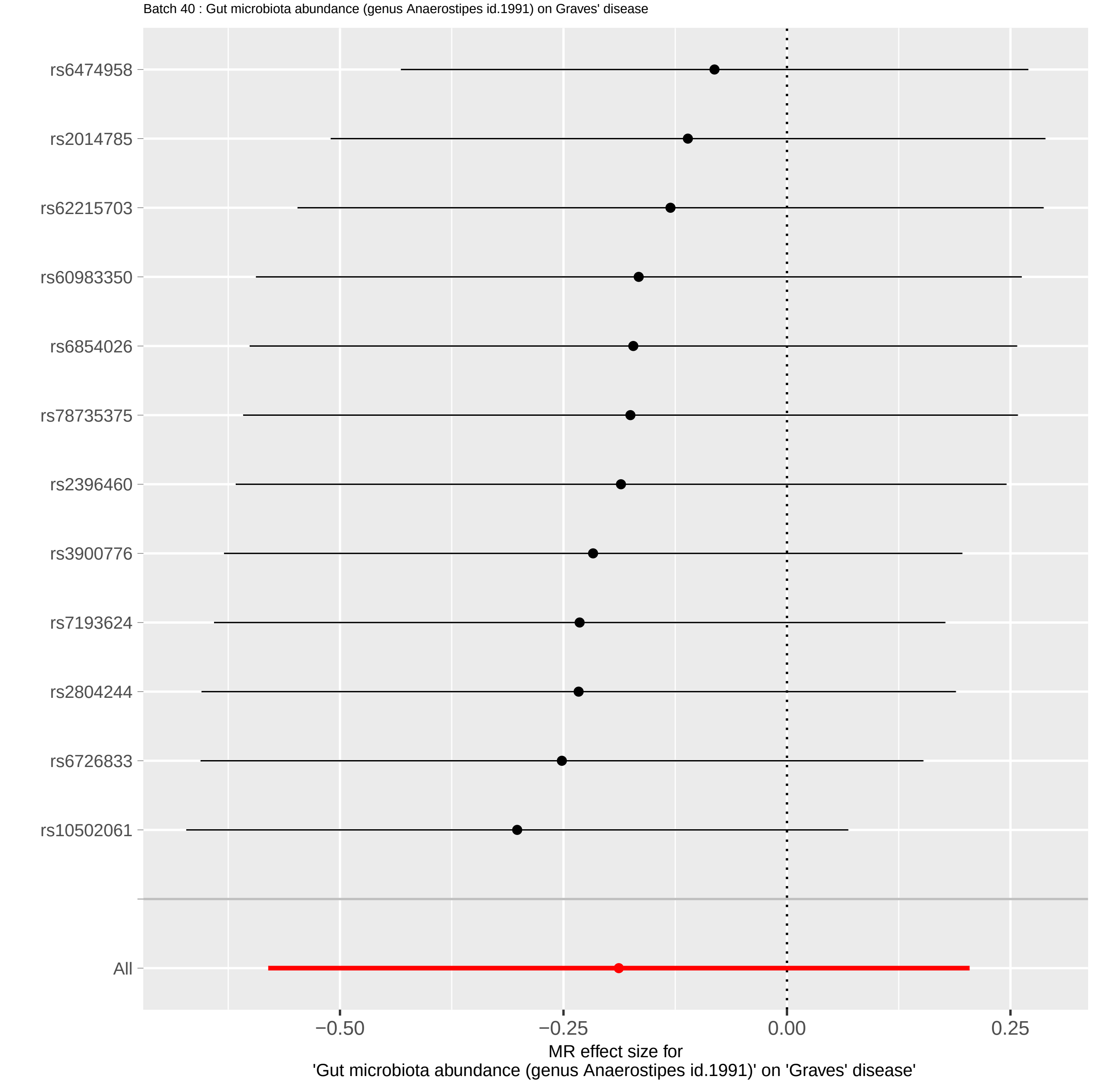
Batch 40 : Gut microbiota abundance (genus Anaerostipes id.1991) on Graves' disease
rs6474958
rs2014785
rs62215703
rs60983350
rs6854026
rs78735375
rs2396460
rs3900776
rs7193624
rs2804244
rs6726833
rs10502061
All
−0.50
−0.25
0.00
0.25
MR effect size for
'Gut microbiota abundance (genus Anaerostipes id.1991)' on 'Graves' disease'

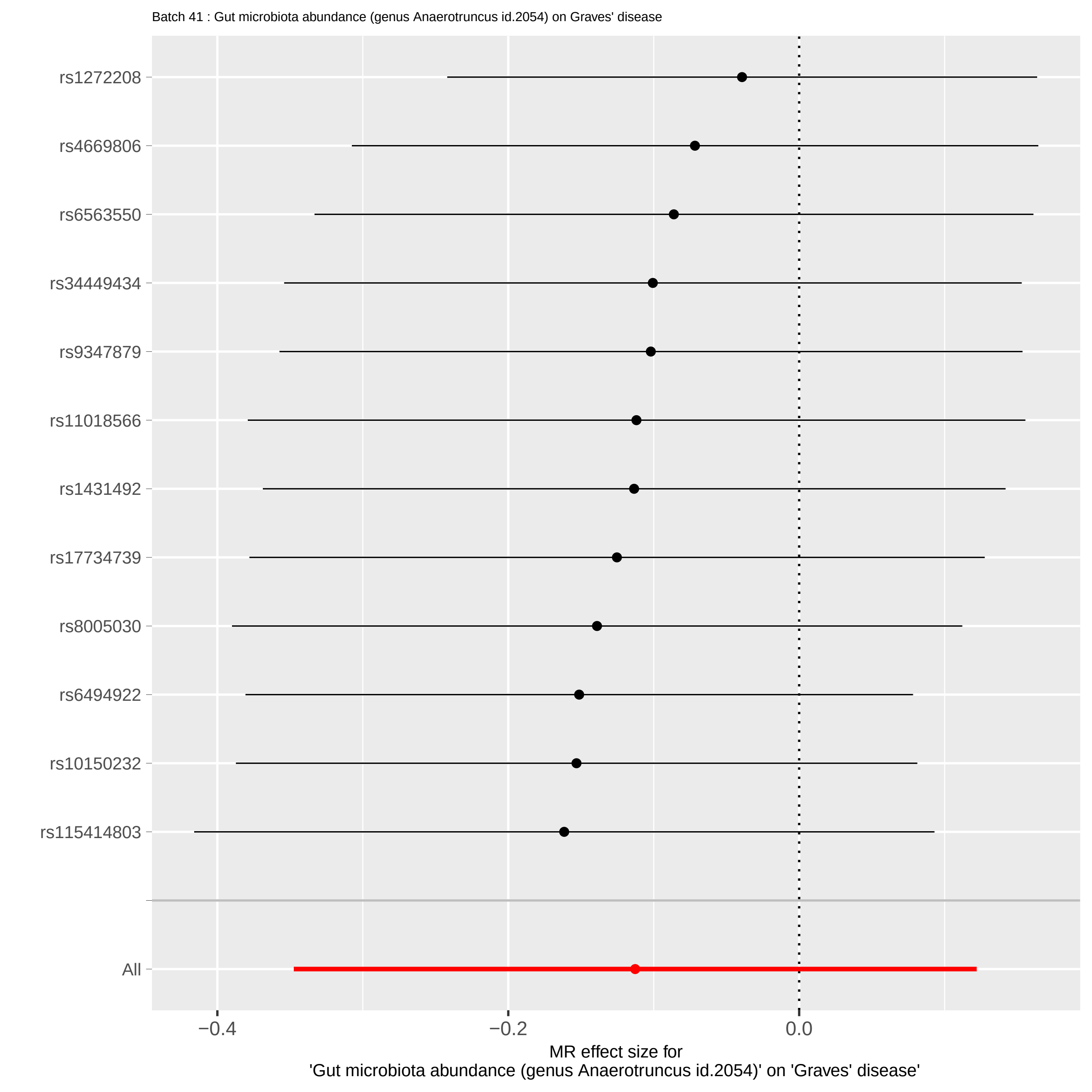

Batch 41 : Gut microbiota abundance (genus Anaerotruncus id.2054) on Graves' disease
rs1272208
rs4669806
rs6563550
rs34449434
rs9347879
rs11018566
rs1431492
rs17734739
rs8005030
rs6494922
rs10150232
rs115414803
All
−0.4
−0.2
0.0
MR effect size for
'Gut microbiota abundance (genus Anaerotruncus id.2054)' on 'Graves' disease'

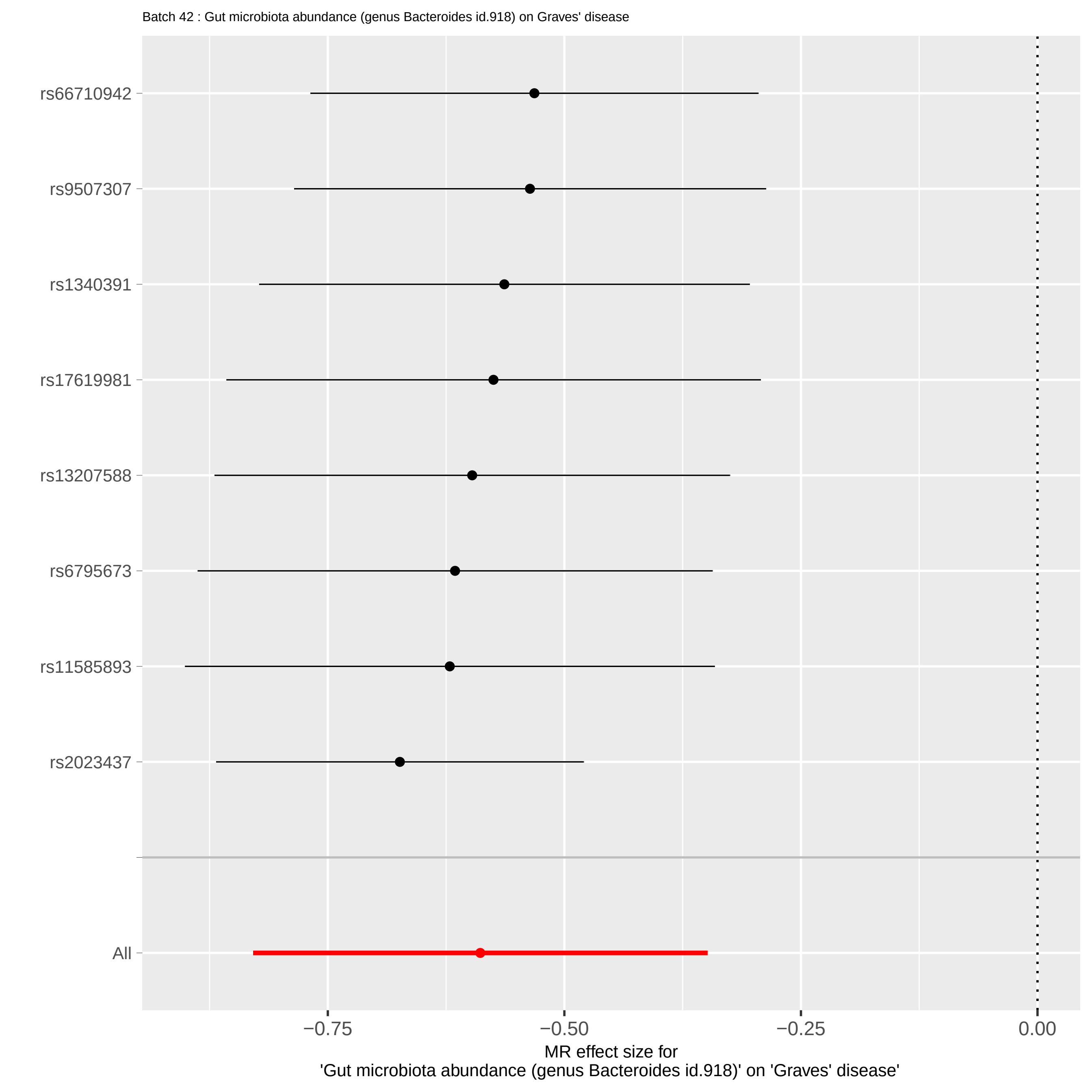

Batch 42 : Gut microbiota abundance (genus Bacteroides id.918) on Graves' disease
rs66710942
rs9507307
rs1340391
rs17619981
rs13207588
rs6795673
rs11585893
rs2023437
All
−0.75
−0.50
−0.25
0.00
MR effect size for
'Gut microbiota abundance (genus Bacteroides id.918)' on 'Graves' disease'

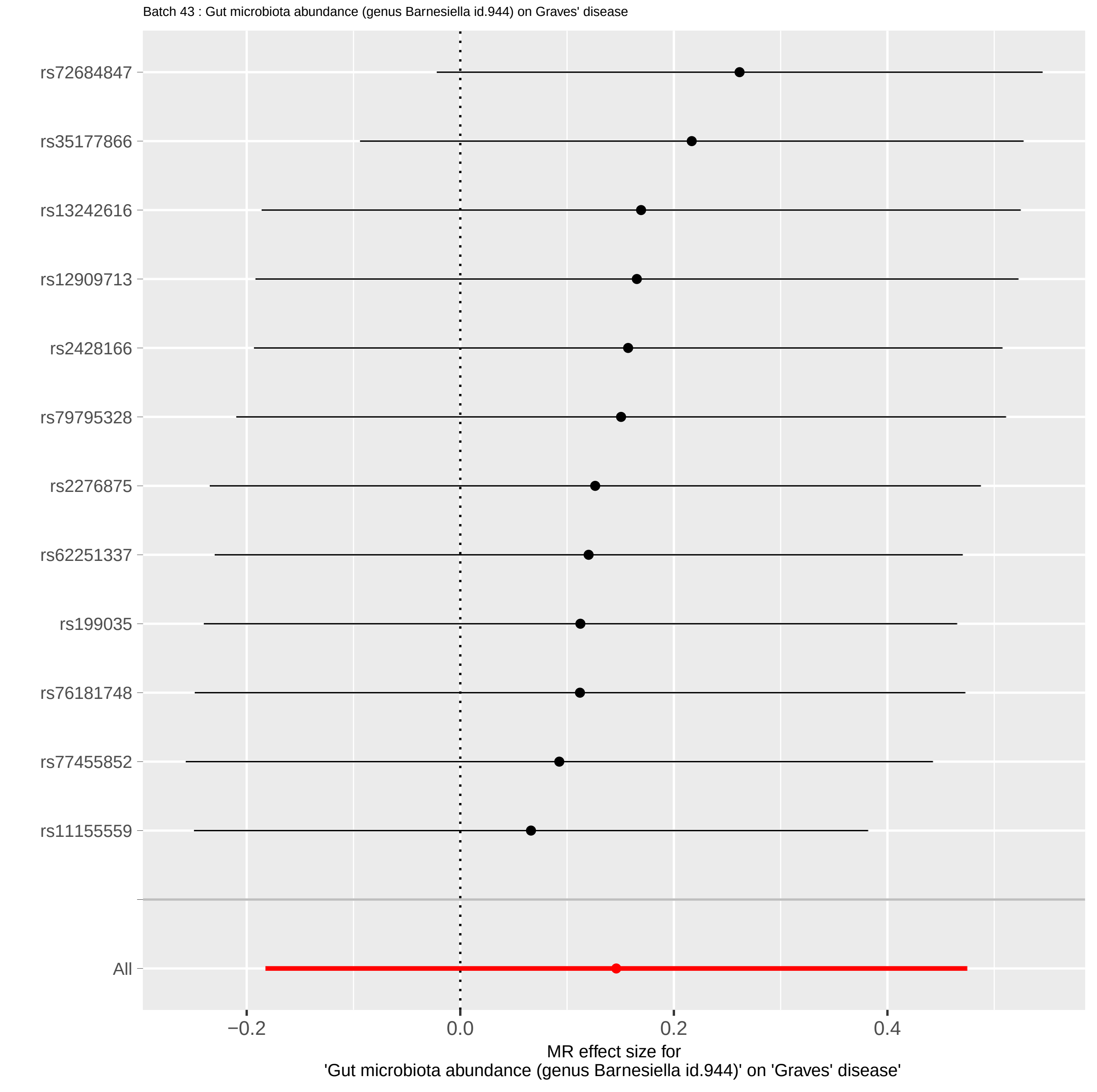
Batch 43 : Gut microbiota abundance (genus Barnesiella id.944) on Graves' disease
rs72684847
rs35177866
rs13242616
rs12909713
rs2428166
rs79795328
rs2276875
rs62251337
rs199035
rs76181748
rs77455852
rs11155559
All
−0.2
0.0
0.2
0.4
MR effect size for
'Gut microbiota abundance (genus Barnesiella id.944)' on 'Graves' disease'

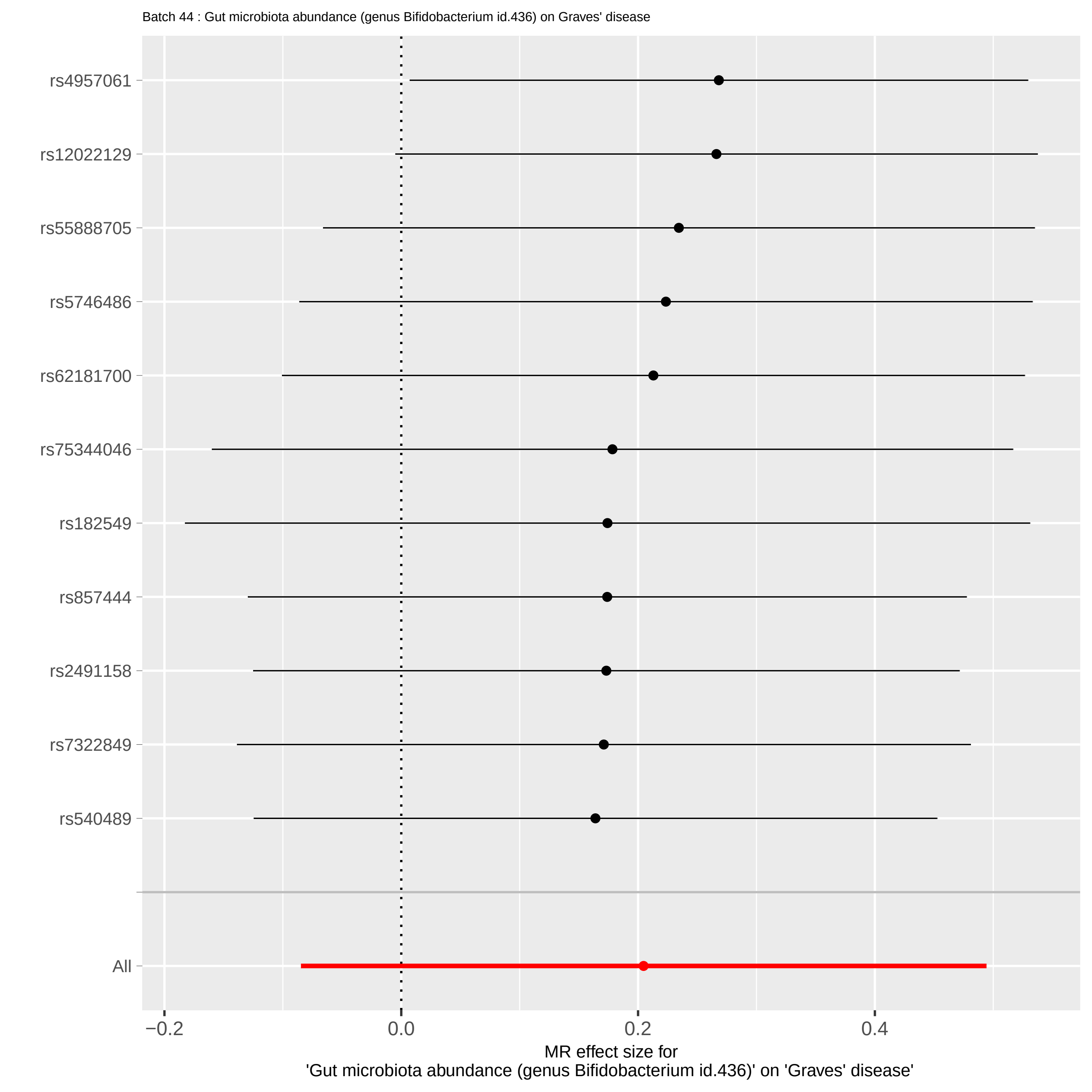
Batch 44 : Gut microbiota abundance (genus Bifidobacterium id.436) on Graves' disease
rs4957061
rs12022129
rs55888705
rs5746486
rs62181700
rs75344046
rs182549
rs857444
rs2491158
rs7322849
rs540489
All
−0.2
0.0
0.2
0.4
MR effect size for
'Gut microbiota abundance (genus Bifidobacterium id.436)' on 'Graves' disease'

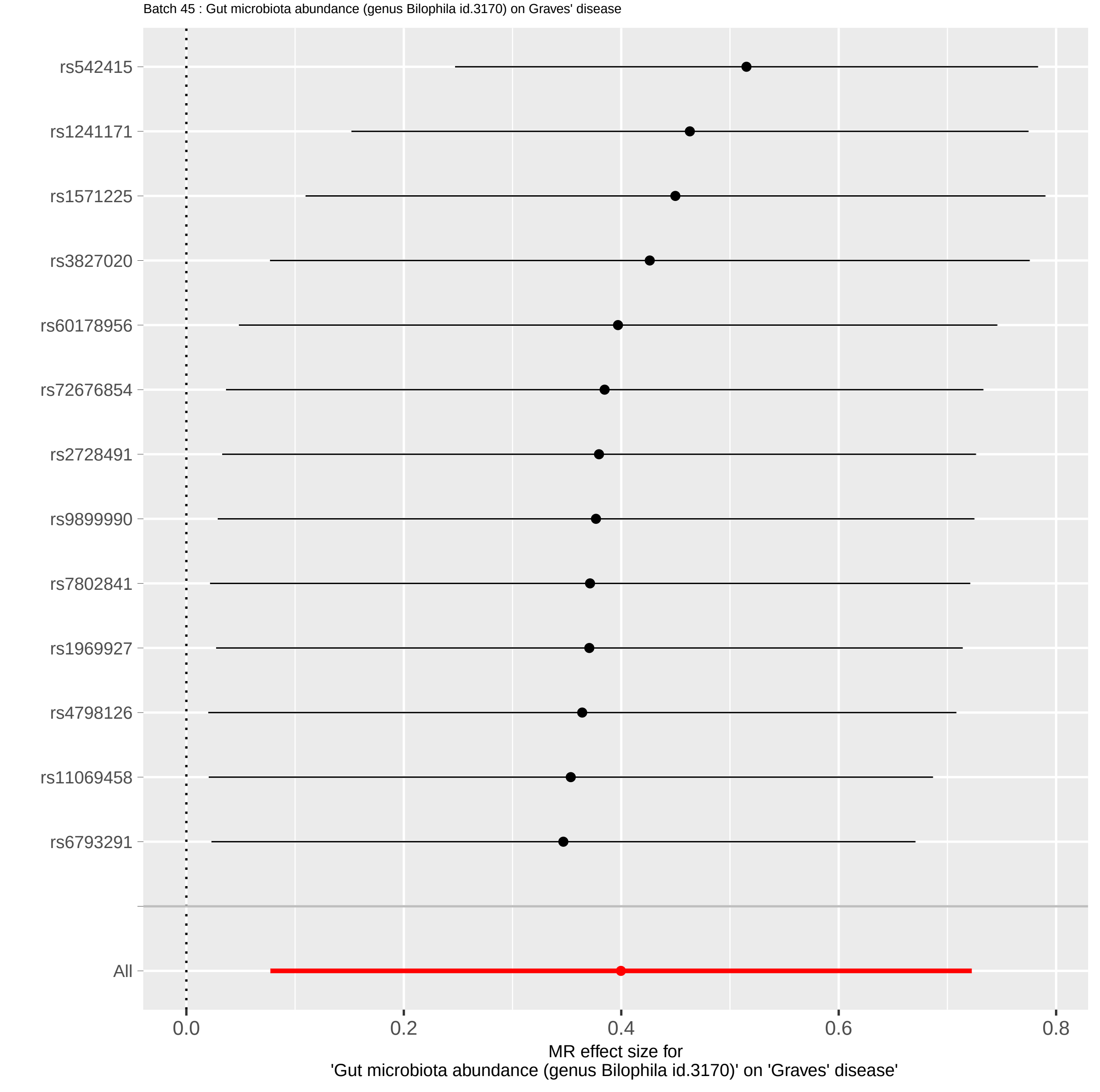
Batch 45 : Gut microbiota abundance (genus Bilophila id.3170) on Graves' disease
rs542415
rs1241171
rs1571225
rs3827020
rs60178956
rs72676854
rs2728491
rs9899990
rs7802841
rs1969927
rs4798126
rs11069458
rs6793291
All
0.0
0.2
0.4
0.6
0.8
MR effect size for
'Gut microbiota abundance (genus Bilophila id.3170)' on 'Graves' disease'

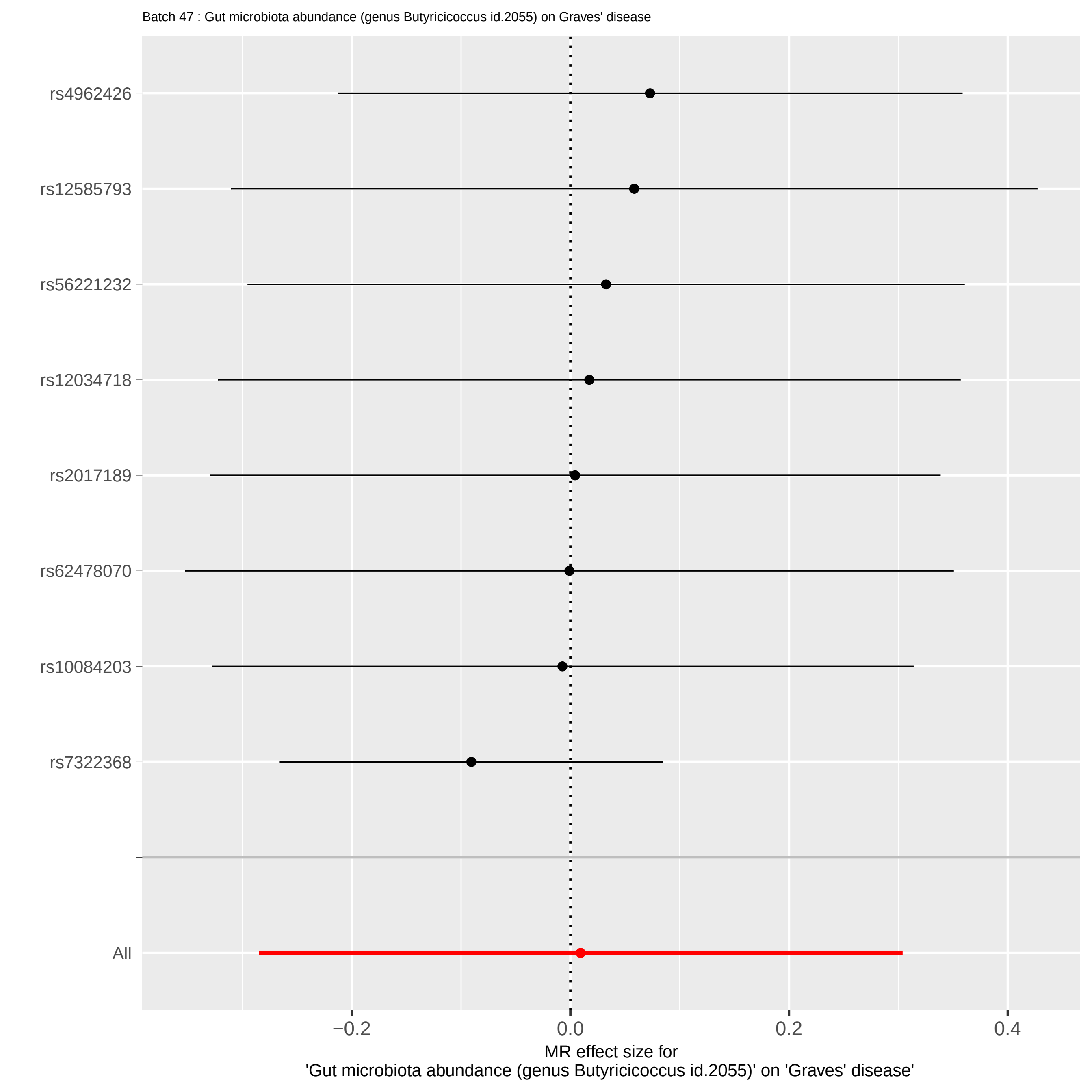

Batch 47 : Gut microbiota abundance (genus Butyricicoccus id.2055) on Graves' disease
rs4962426
rs12585793
rs56221232
rs12034718
rs2017189
rs62478070
rs10084203
rs7322368
All
−0.2
0.0
0.2
0.4
MR effect size for
'Gut microbiota abundance (genus Butyricicoccus id.2055)' on 'Graves' disease'

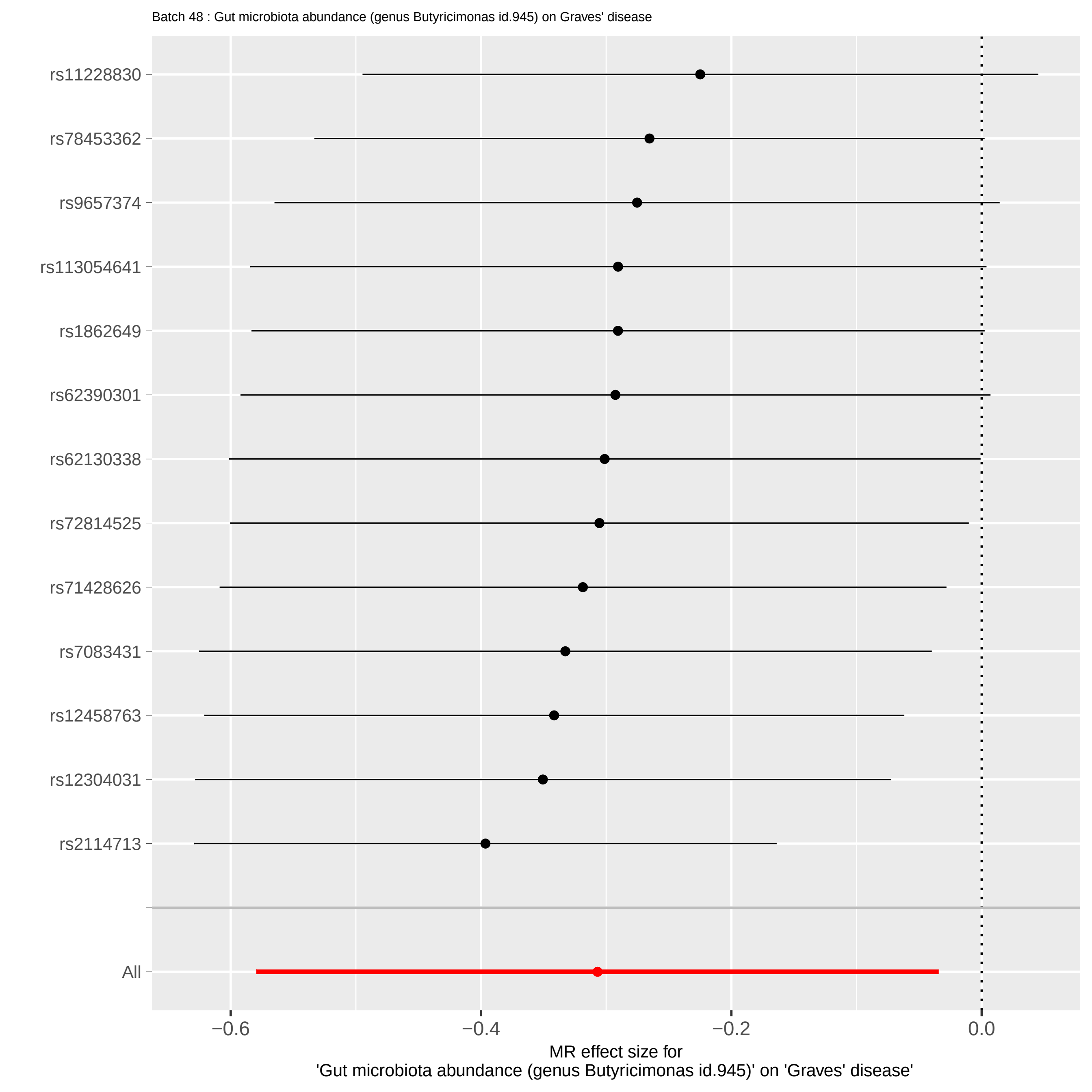

Batch 48 : Gut microbiota abundance (genus Butyricimonas id.945) on Graves' disease
rs11228830
rs78453362
rs9657374
rs113054641
rs1862649
rs62390301
rs62130338
rs72814525
rs71428626
rs7083431
rs12458763
rs12304031
rs2114713
All
−0.6
−0.4
−0.2
0.0
MR effect size for
'Gut microbiota abundance (genus Butyricimonas id.945)' on 'Graves' disease'

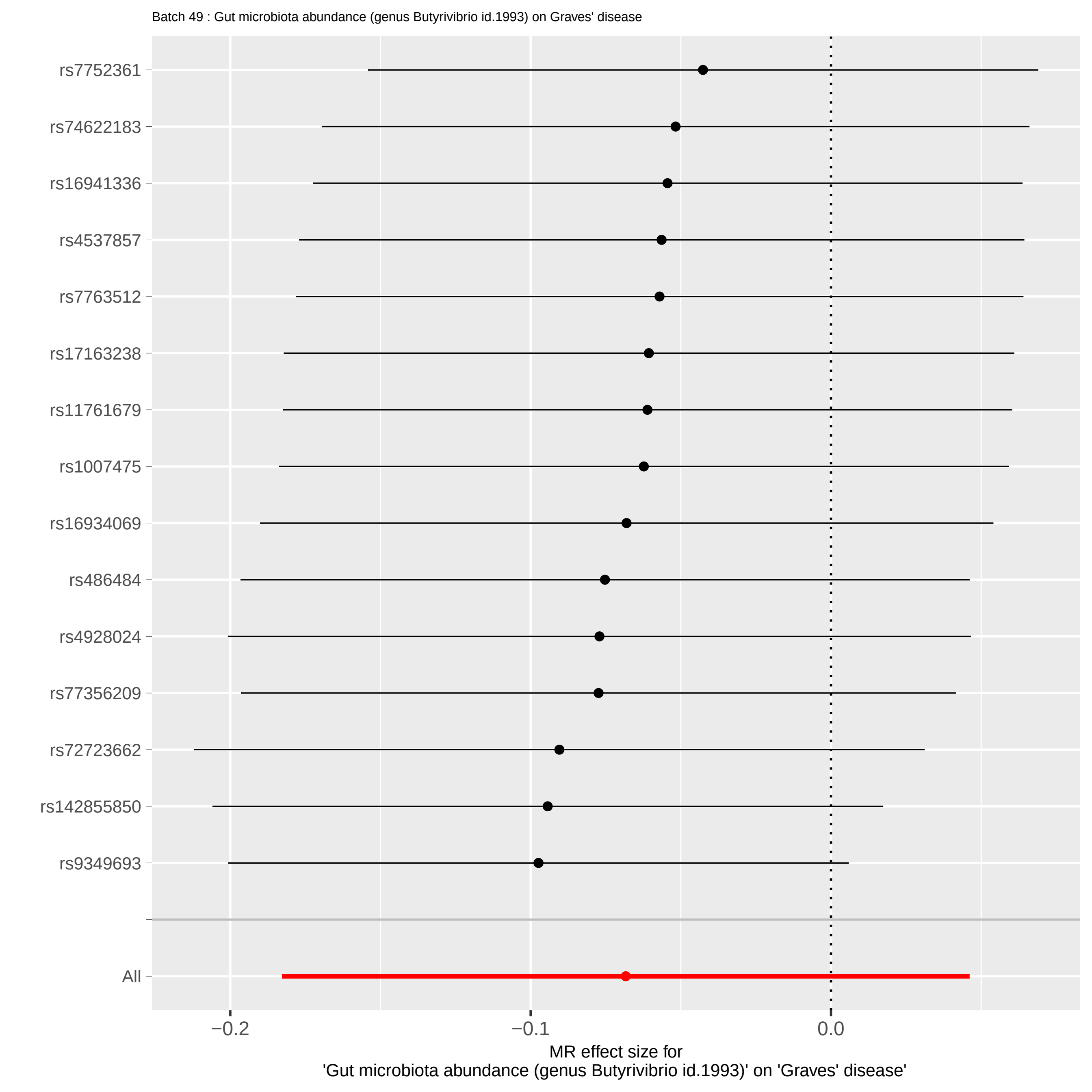
Batch 49 : Gut microbiota abundance (genus Butyrivibrio id.1993) on Graves' disease
rs7752361
rs74622183
rs16941336
rs4537857
rs7763512
rs17163238
rs11761679
rs1007475
rs16934069
rs486484
rs4928024
rs77356209
rs72723662
rs142855850
rs9349693
All
−0.2
−0.1
0.0
MR effect size for
'Gut microbiota abundance (genus Butyrivibrio id.1993)' on 'Graves' disease'

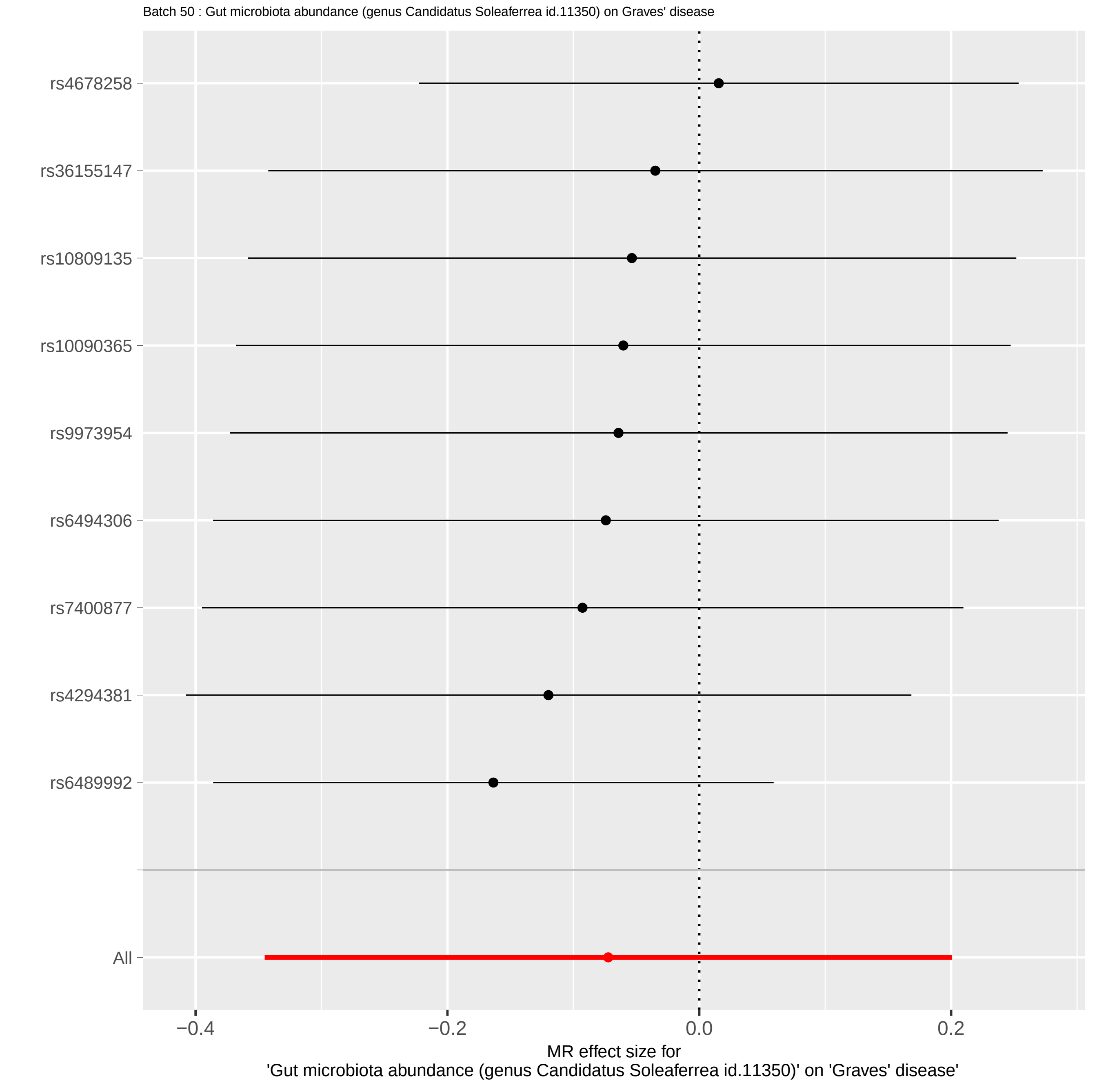

Batch 50 : Gut microbiota abundance (genus Candidatus Soleaferrea id.11350) on Graves' disease
rs4678258
rs36155147
rs10809135
rs10090365
rs9973954
rs6494306
rs7400877
rs4294381
rs6489992
All
−0.4
−0.2
0.0
0.2
MR effect size for
'Gut microbiota abundance (genus Candidatus Soleaferrea id.11350)' on 'Graves' disease'

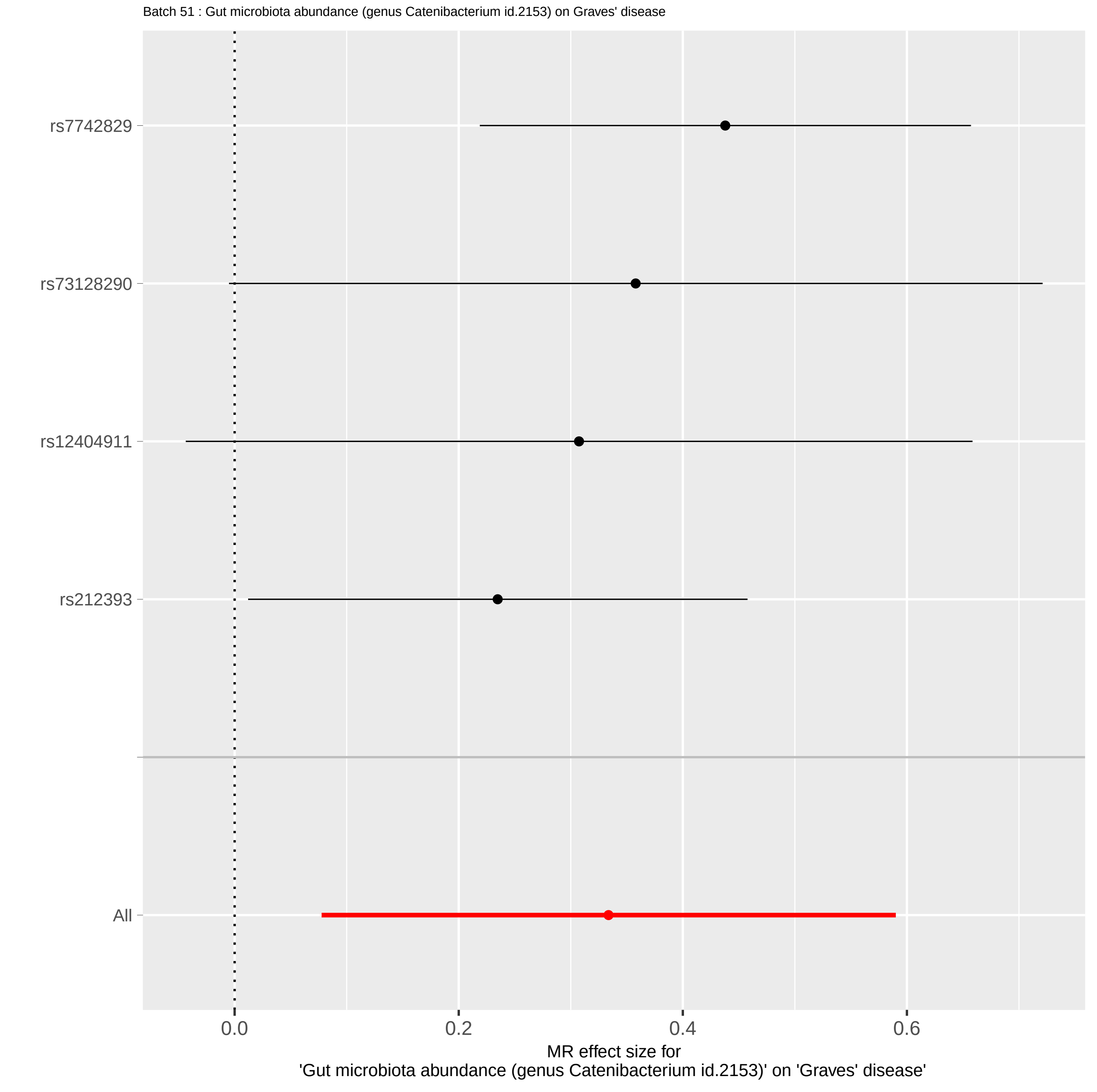

Batch 51 : Gut microbiota abundance (genus Catenibacterium id.2153) on Graves' disease
rs7742829
rs73128290
rs12404911
rs212393
All
0.0
0.2
0.4
0.6
MR effect size for
'Gut microbiota abundance (genus Catenibacterium id.2153)' on 'Graves' disease'

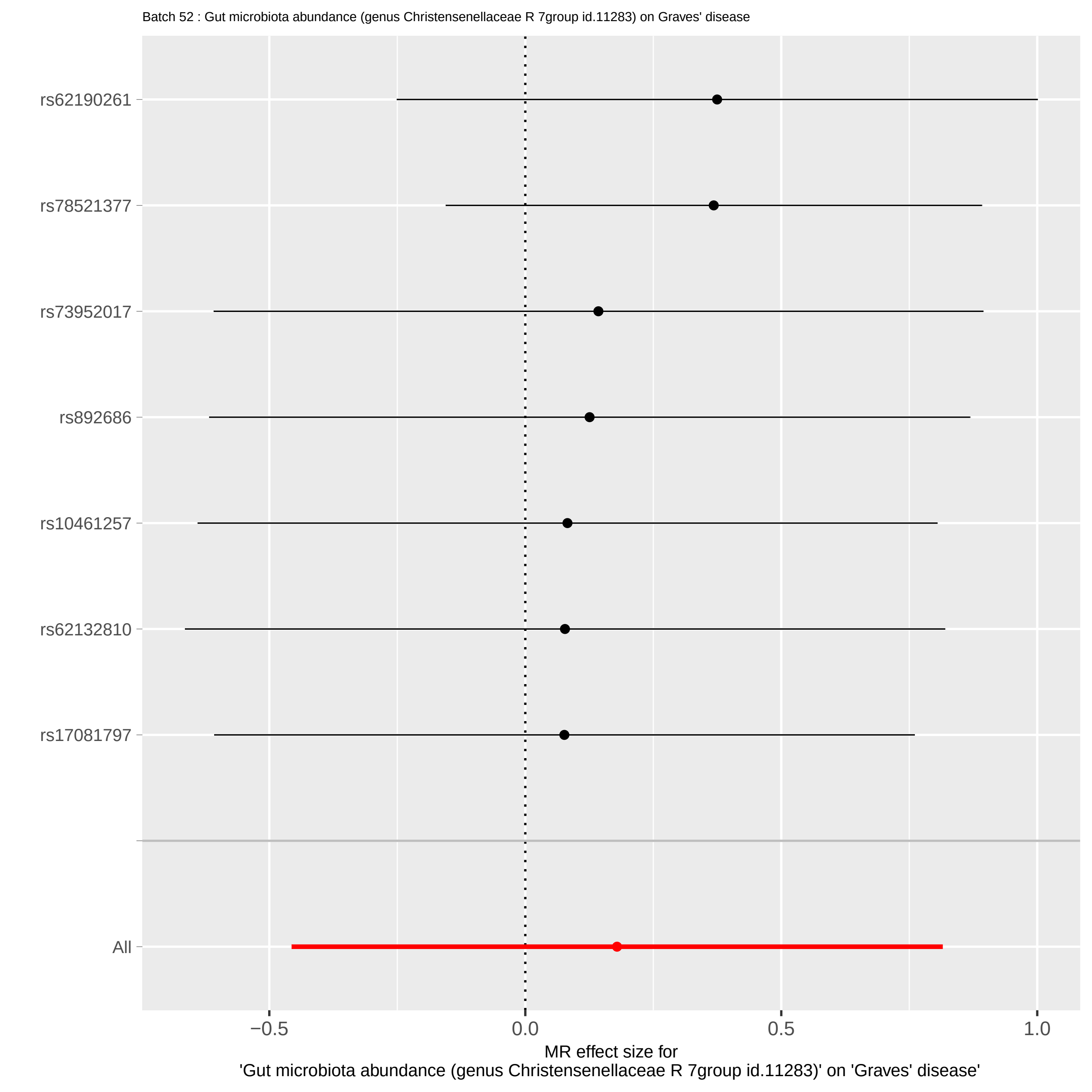
Batch 52 : Gut microbiota abundance (genus Christensenellaceae R 7group id.11283) on Graves' disease
rs62190261
rs78521377
rs73952017
rs892686
rs10461257
rs62132810
rs17081797
All
−0.5
0.0
0.5
1.0
MR effect size for
'Gut microbiota abundance (genus Christensenellaceae R 7group id.11283)' on 'Graves' disease'

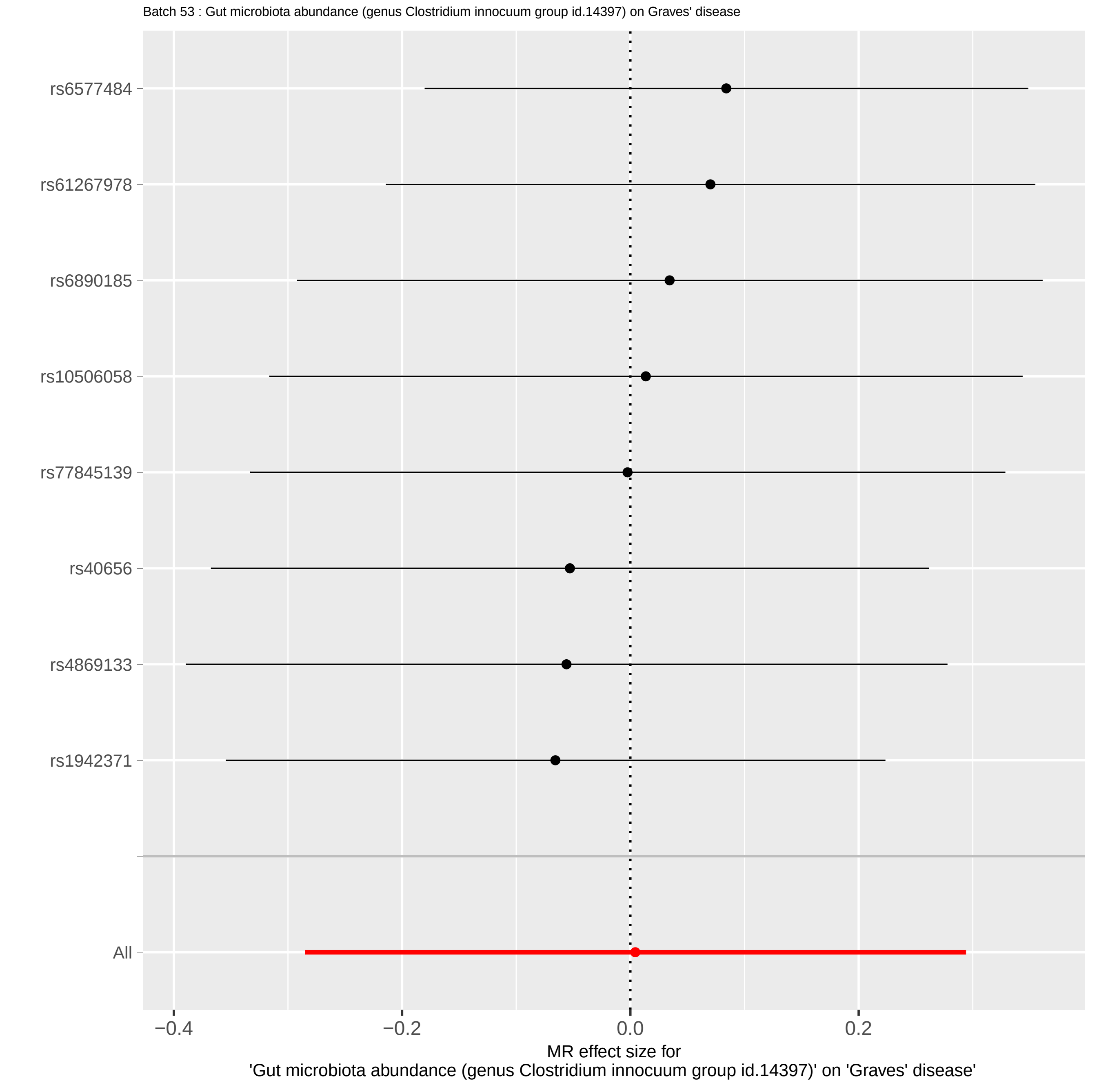
Batch 53 : Gut microbiota abundance (genus Clostridium innocuum group id.14397) on Graves' disease
rs6577484
rs61267978
rs6890185
rs10506058
rs77845139
rs40656
rs4869133
rs1942371
All
−0.4
−0.2
0.0
0.2
MR effect size for
'Gut microbiota abundance (genus Clostridium innocuum group id.14397)' on 'Graves' disease'

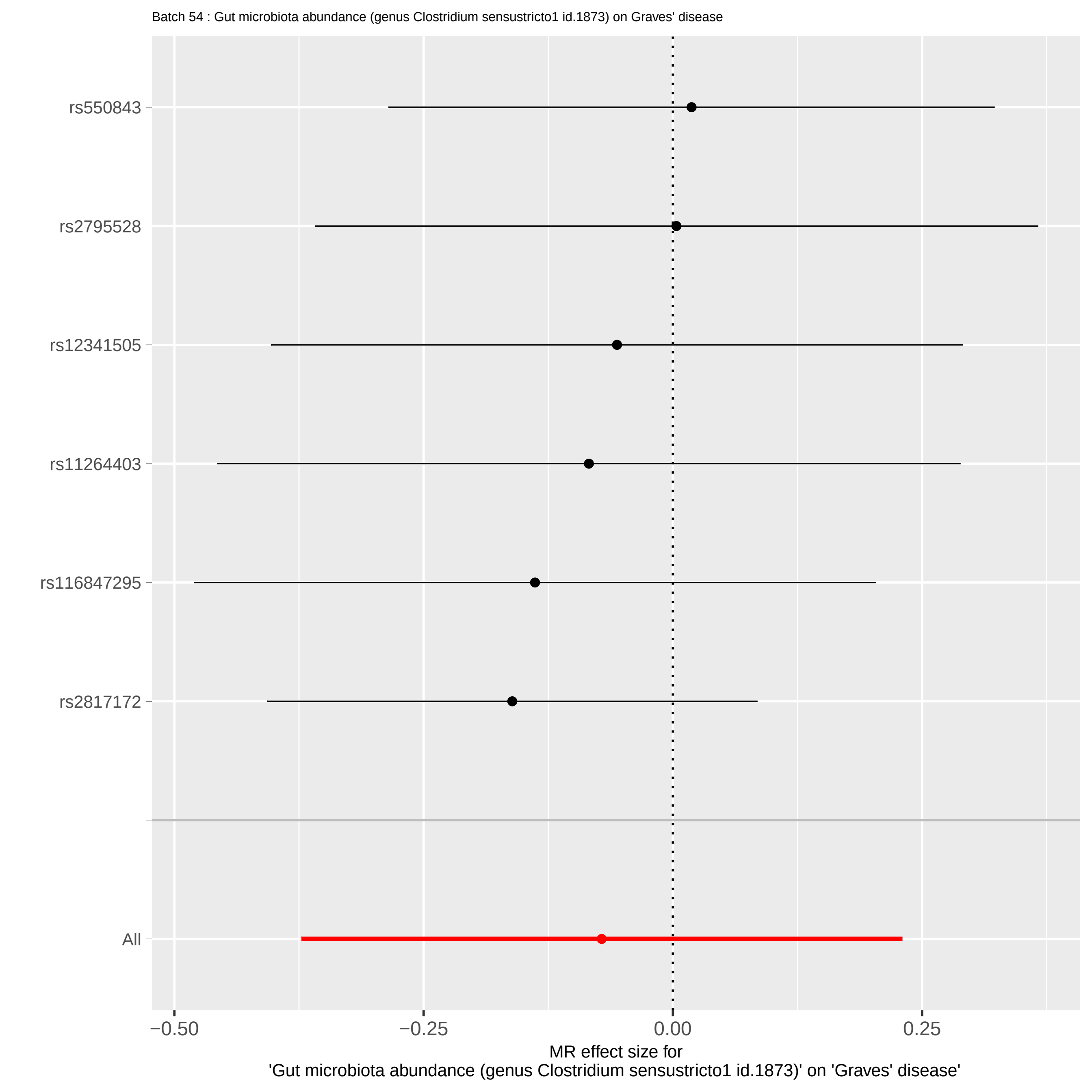

Batch 54 : Gut microbiota abundance (genus Clostridium sensustricto1 id.1873) on Graves' disease
rs550843
rs2795528
rs12341505
rs11264403
rs116847295
rs2817172
All
−0.50
−0.25
0.00
0.25
MR effect size for
'Gut microbiota abundance (genus Clostridium sensustricto1 id.1873)' on 'Graves' disease'

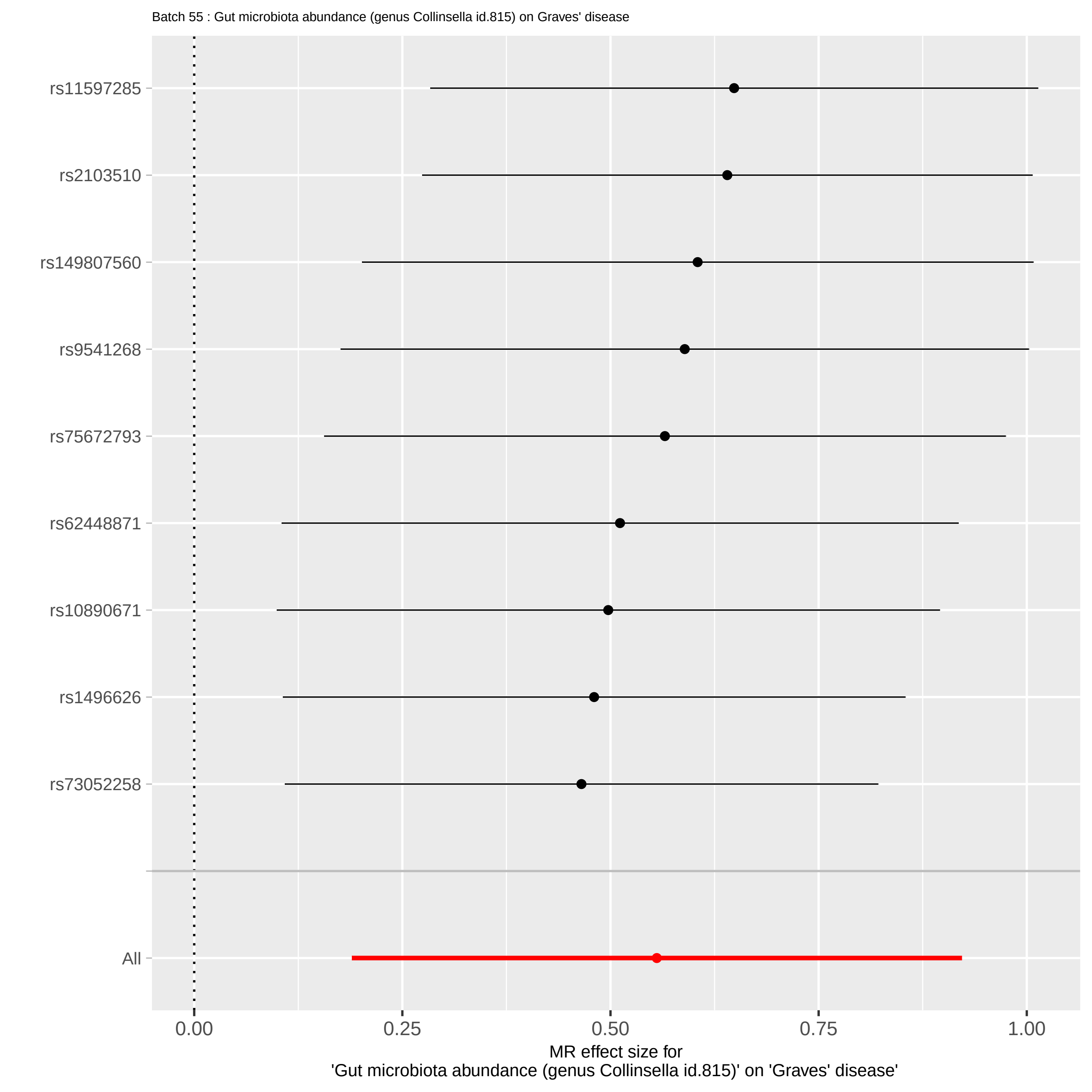

Batch 55 : Gut microbiota abundance (genus Collinsella id.815) on Graves' disease
rs11597285
rs2103510
rs149807560
rs9541268
rs75672793
rs62448871
rs10890671
rs1496626
rs73052258
All
0.00
0.25
0.50
0.75
1.00
MR effect size for
'Gut microbiota abundance (genus Collinsella id.815)' on 'Graves' disease'

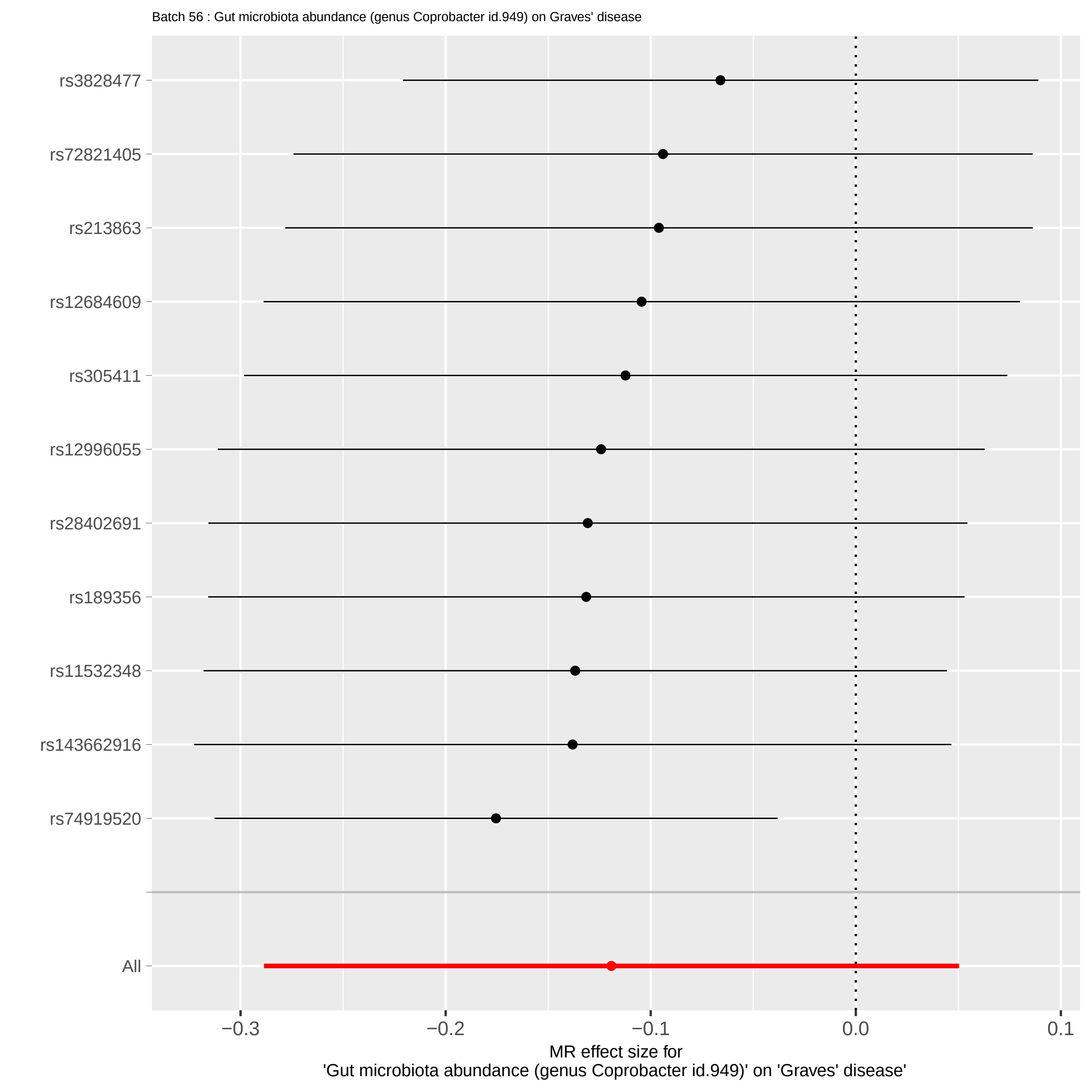

Batch 56 : Gut microbiota abundance (genus Coprobacter id.949) on Graves' disease
rs3828477
rs72821405
rs213863
rs12684609
rs305411
rs12996055
rs28402691
rs189356
rs11532348
rs143662916
rs74919520
All
−0.3
−0.2
−0.1
0.0
0.1
MR effect size for
'Gut microbiota abundance (genus Coprobacter id.949)' on 'Graves' disease'

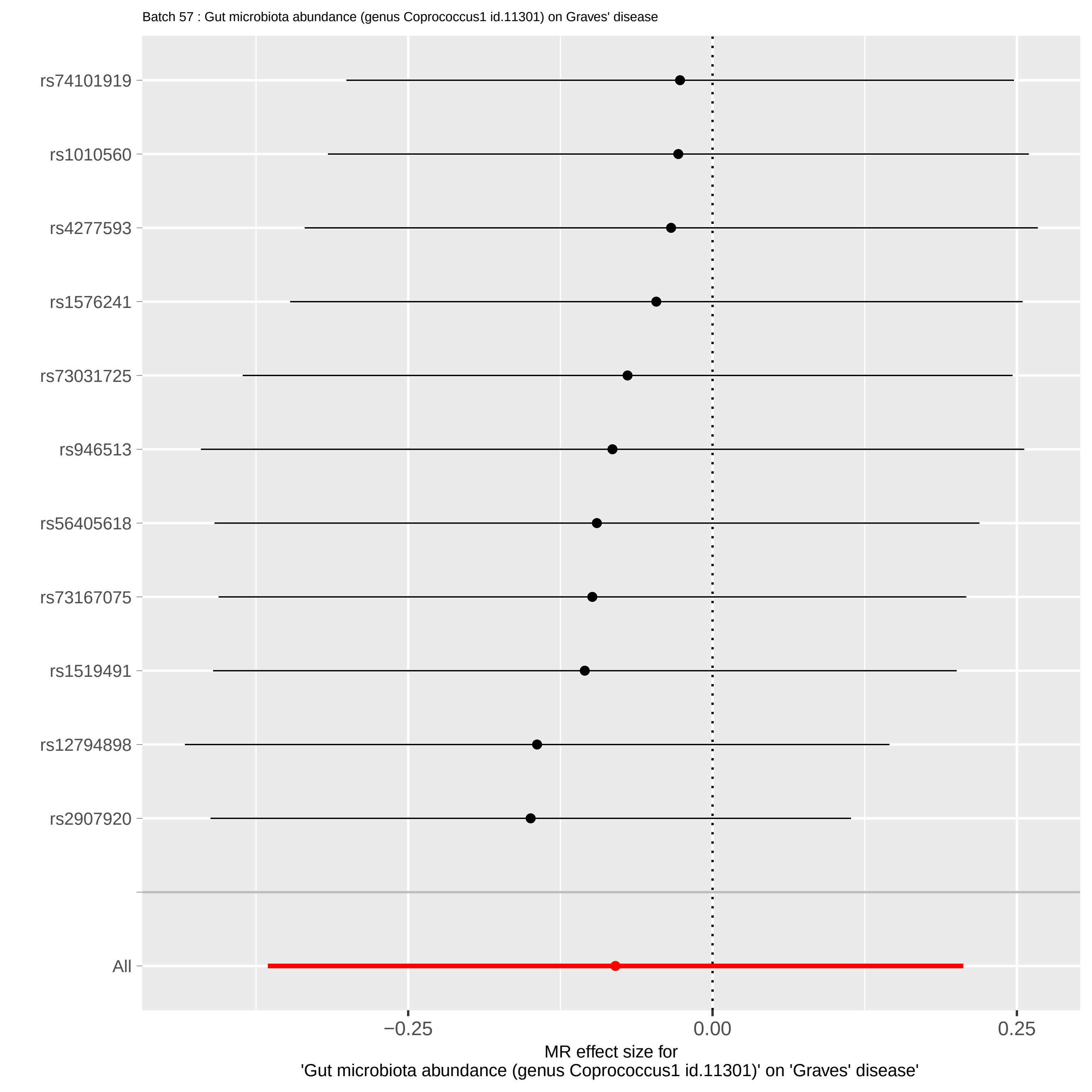
Batch 57 : Gut microbiota abundance (genus Coprococcus1 id.11301) on Graves' disease
rs74101919
rs1010560
rs4277593
rs1576241
rs73031725
rs946513
rs56405618
rs73167075
rs1519491
rs12794898
rs2907920
All
−0.25
0.00
0.25
MR effect size for
'Gut microbiota abundance (genus Coprococcus1 id.11301)' on 'Graves' disease'

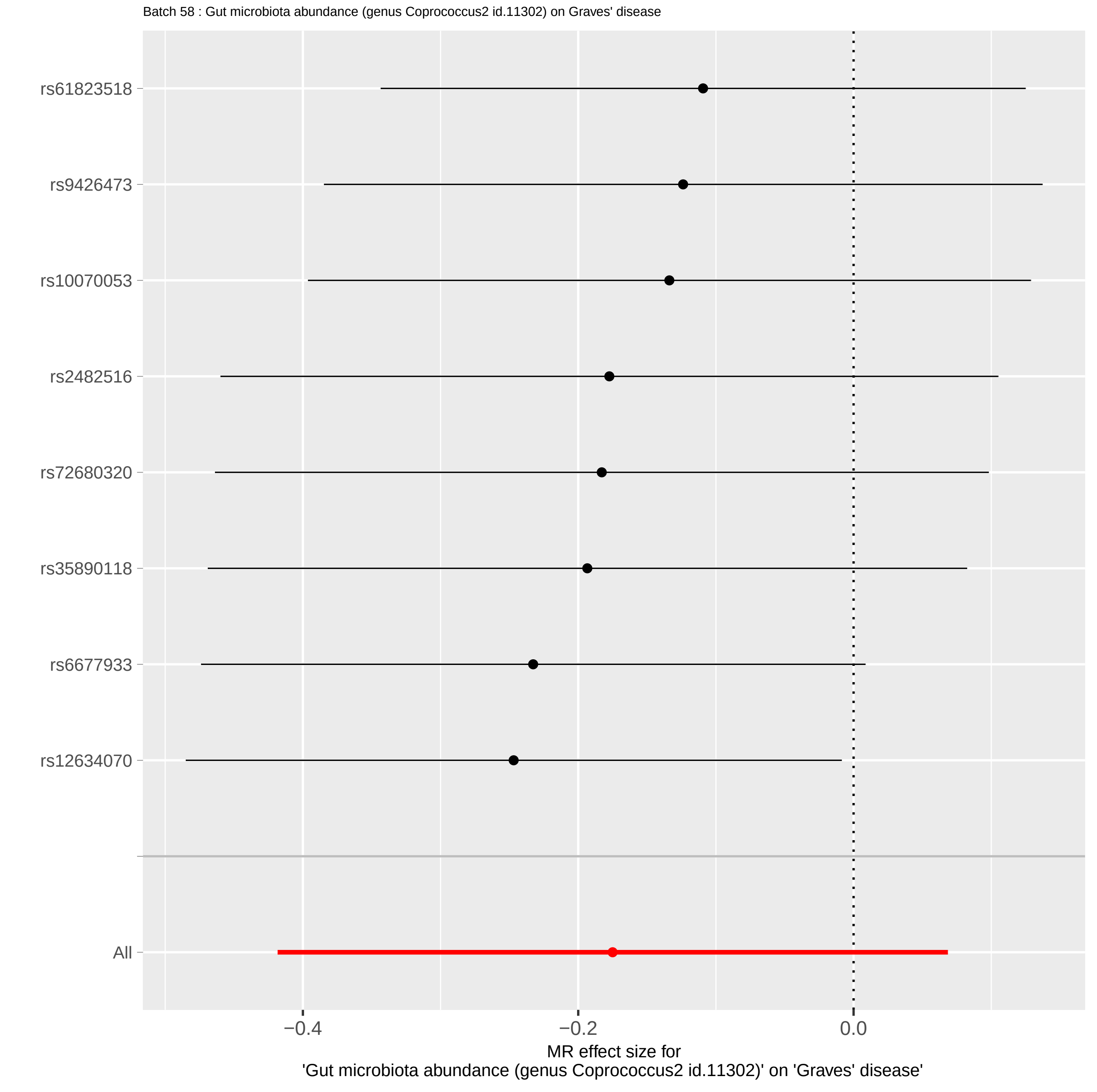

Batch 58 : Gut microbiota abundance (genus Coprococcus2 id.11302) on Graves' disease
rs61823518
rs9426473
rs10070053
rs2482516
rs72680320
rs35890118
rs6677933
rs12634070
All
−0.4
−0.2
0.0
MR effect size for
'Gut microbiota abundance (genus Coprococcus2 id.11302)' on 'Graves' disease'

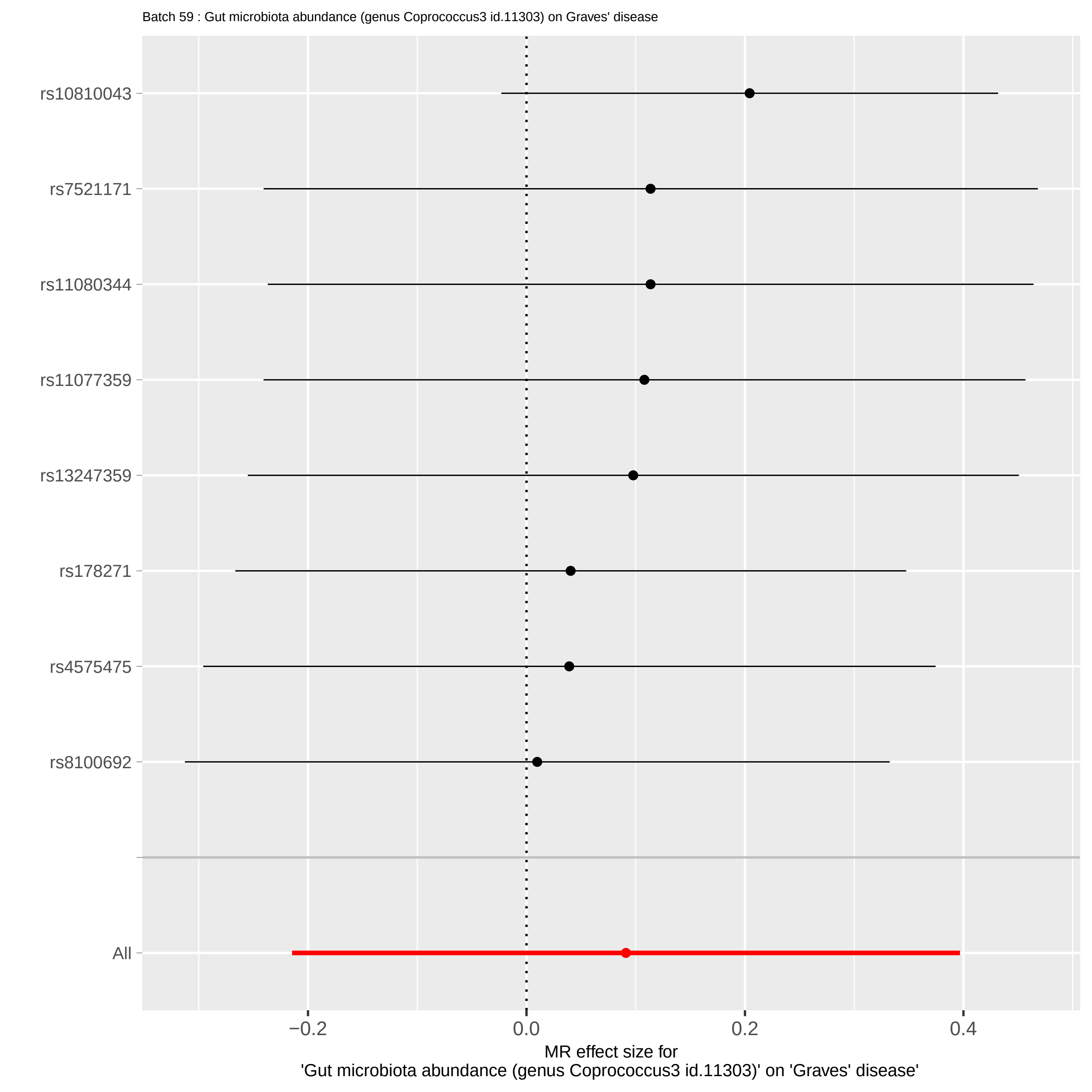
Batch 59 : Gut microbiota abundance (genus Coprococcus3 id.11303) on Graves' disease
rs10810043
rs7521171
rs11080344
rs11077359
rs13247359
rs178271
rs4575475
rs8100692
All
−0.2
0.0
0.2
0.4
MR effect size for
'Gut microbiota abundance (genus Coprococcus3 id.11303)' on 'Graves' disease'

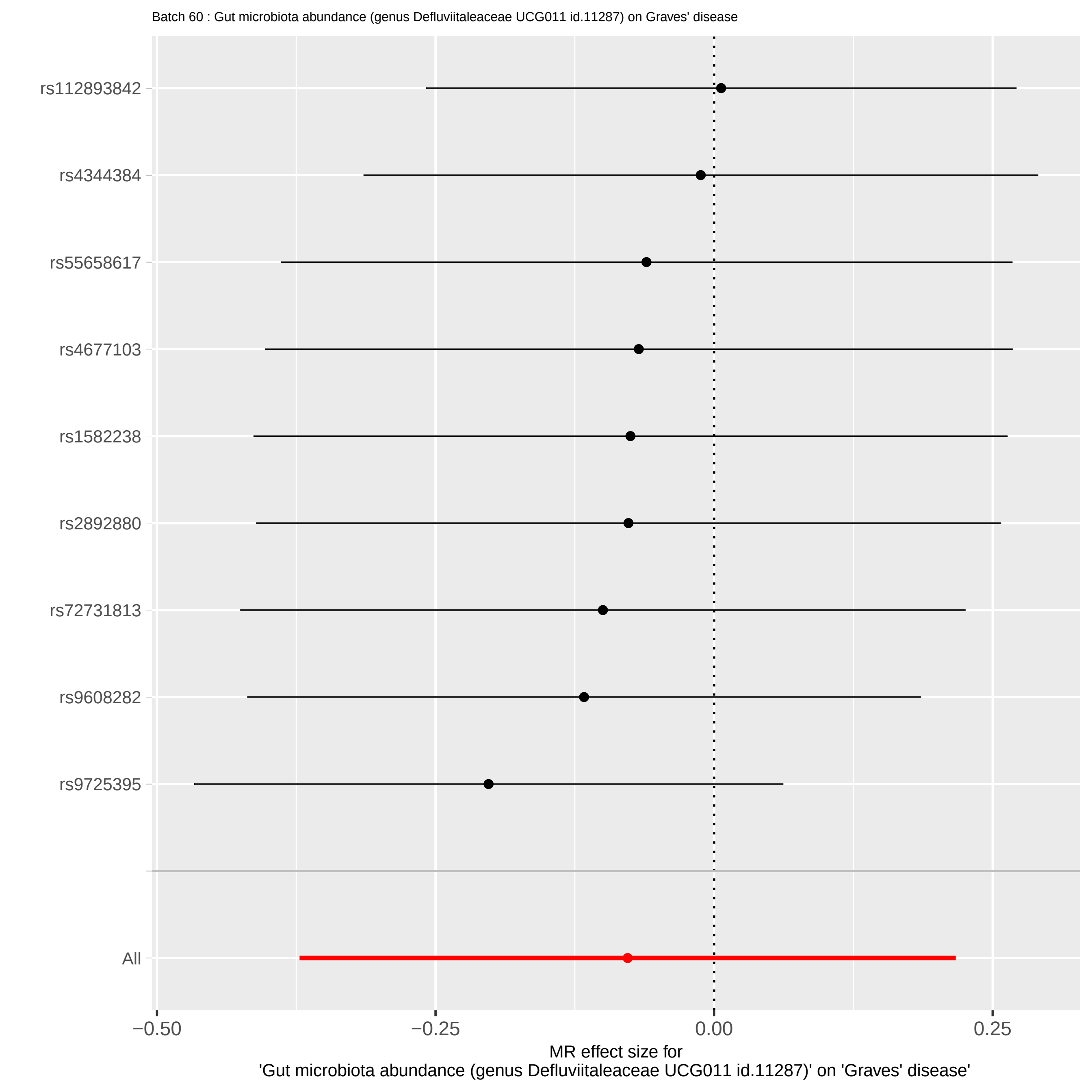

Batch 60 : Gut microbiota abundance (genus Defluviitaleaceae UCG011 id.11287) on Graves' disease
rs112893842
rs4344384
rs55658617
rs4677103
rs1582238
rs2892880
rs72731813
rs9608282
rs9725395
All
−0.50
−0.25
0.00
0.25
MR effect size for
'Gut microbiota abundance (genus Defluviitaleaceae UCG011 id.11287)' on 'Graves' disease'

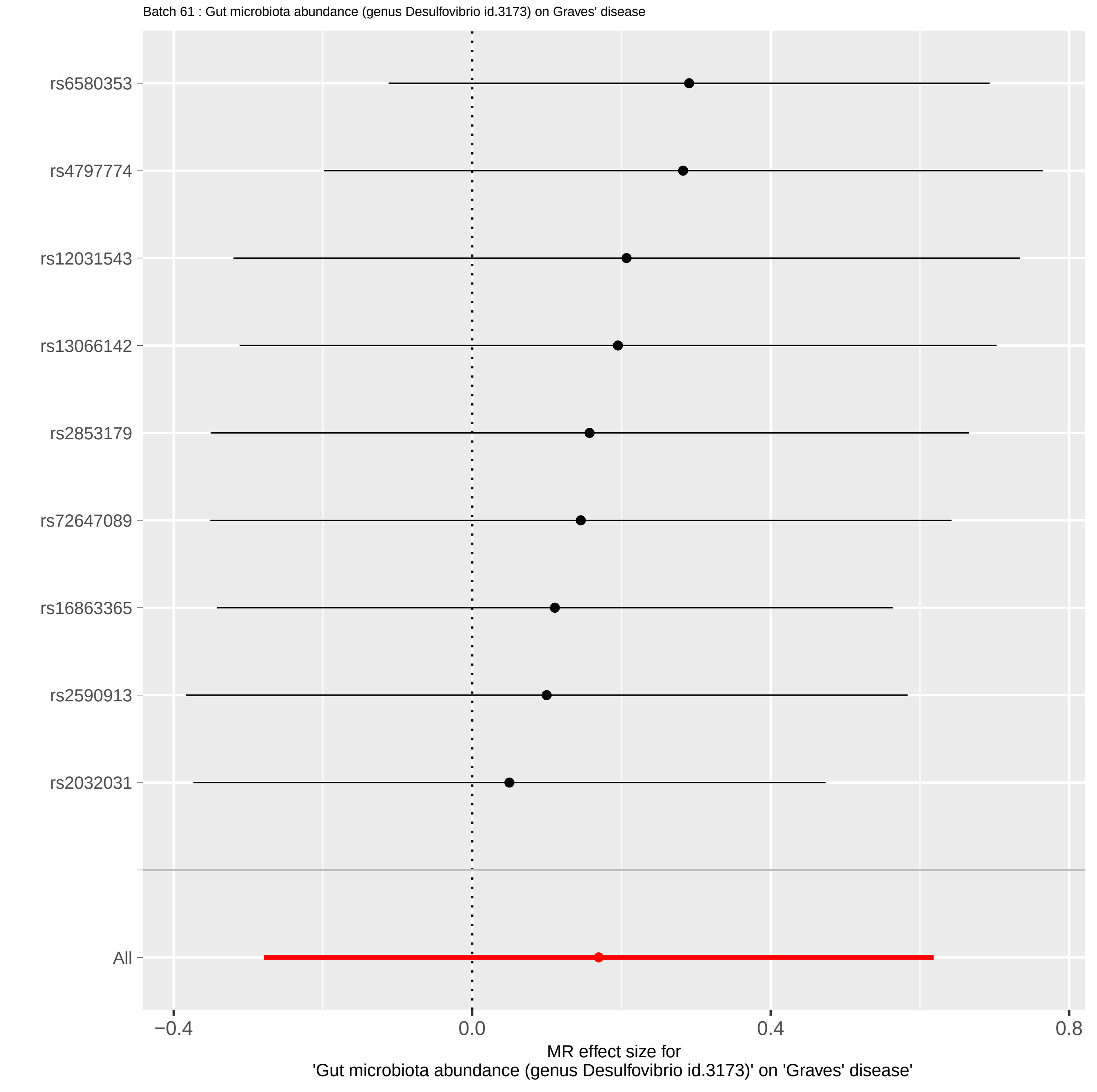

Batch 61 : Gut microbiota abundance (genus Desulfovibrio id.3173) on Graves' disease
rs6580353
rs4797774
rs12031543
rs13066142
rs2853179
rs72647089
rs16863365
rs2590913
rs2032031
All
−0.4
0.0
0.4
0.8
MR effect size for
'Gut microbiota abundance (genus Desulfovibrio id.3173)' on 'Graves' disease'

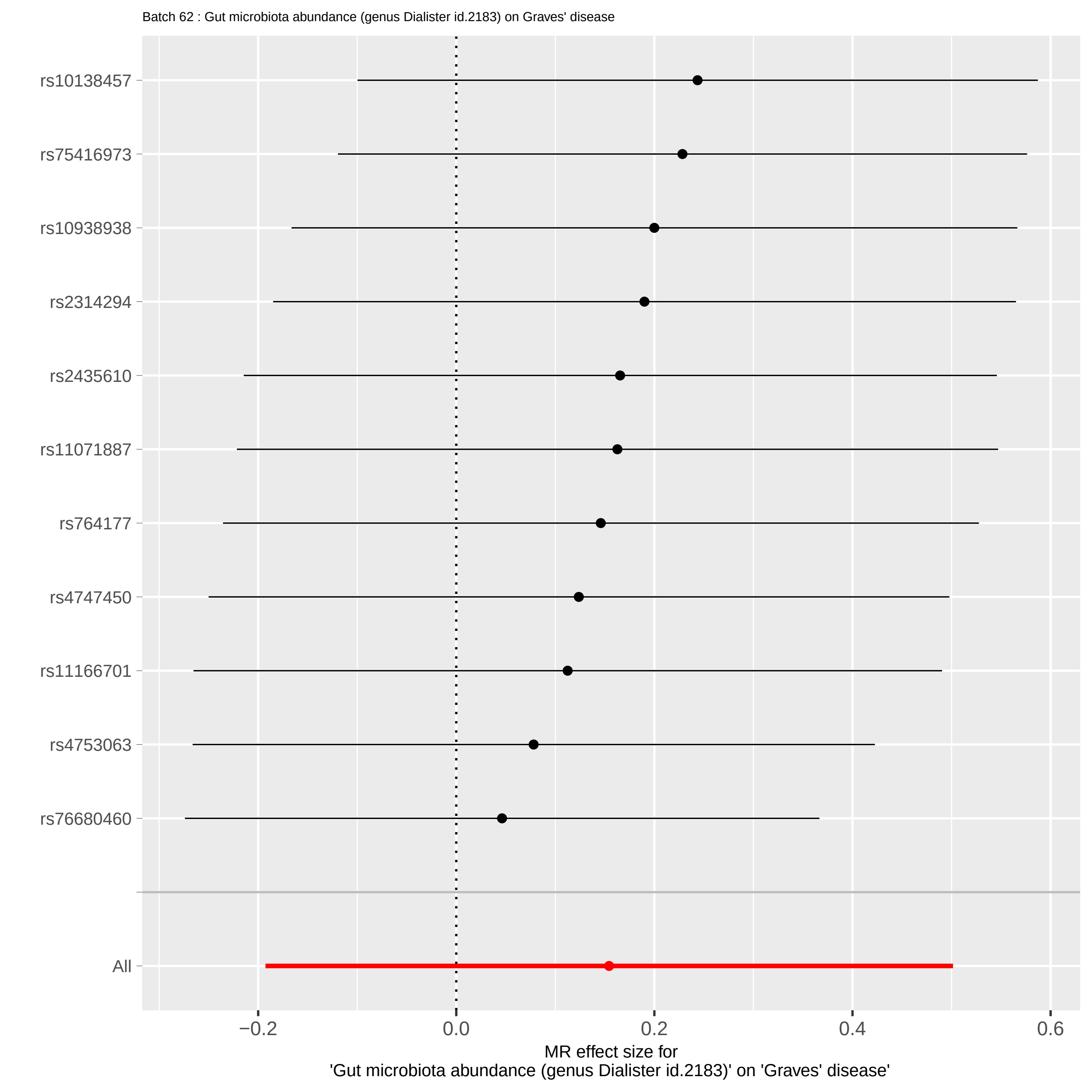
Batch 62 : Gut microbiota abundance (genus Dialister id.2183) on Graves' disease
rs10138457
rs75416973
rs10938938
rs2314294
rs2435610
rs11071887
rs764177
rs4747450
rs11166701
rs4753063
rs76680460
All
−0.2
0.0
0.2
0.4
0.6
MR effect size for
'Gut microbiota abundance (genus Dialister id.2183)' on 'Graves' disease'

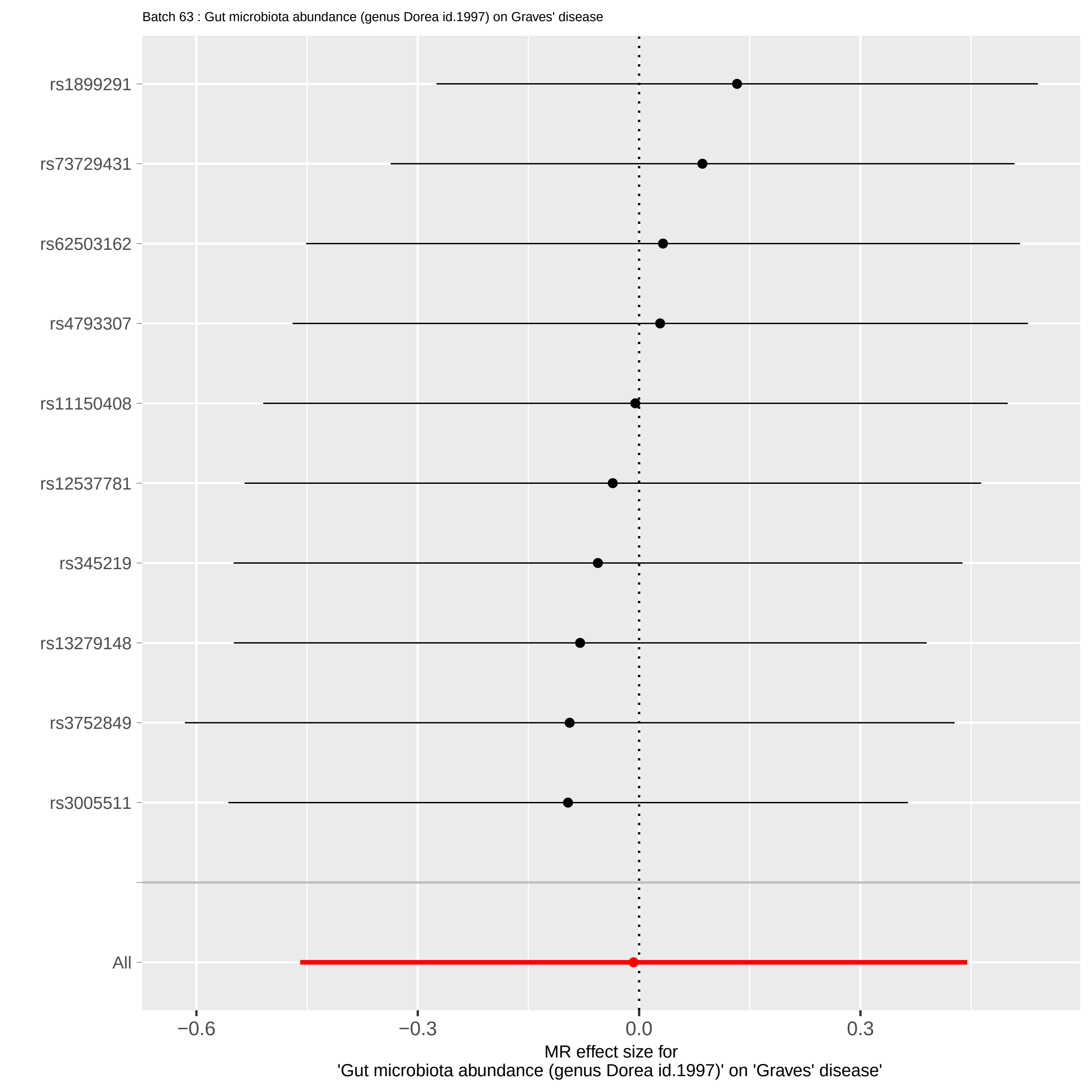

Batch 63 : Gut microbiota abundance (genus Dorea id.1997) on Graves' disease
rs1899291
rs73729431
rs62503162
rs4793307
rs11150408
rs12537781
rs345219
rs13279148
rs3752849
rs3005511
All
−0.6
−0.3
0.0
0.3
MR effect size for
'Gut microbiota abundance (genus Dorea id.1997)' on 'Graves' disease'

Batch 64 : Gut microbiota abundance (genus Eggerthella id.819) on Graves' disease

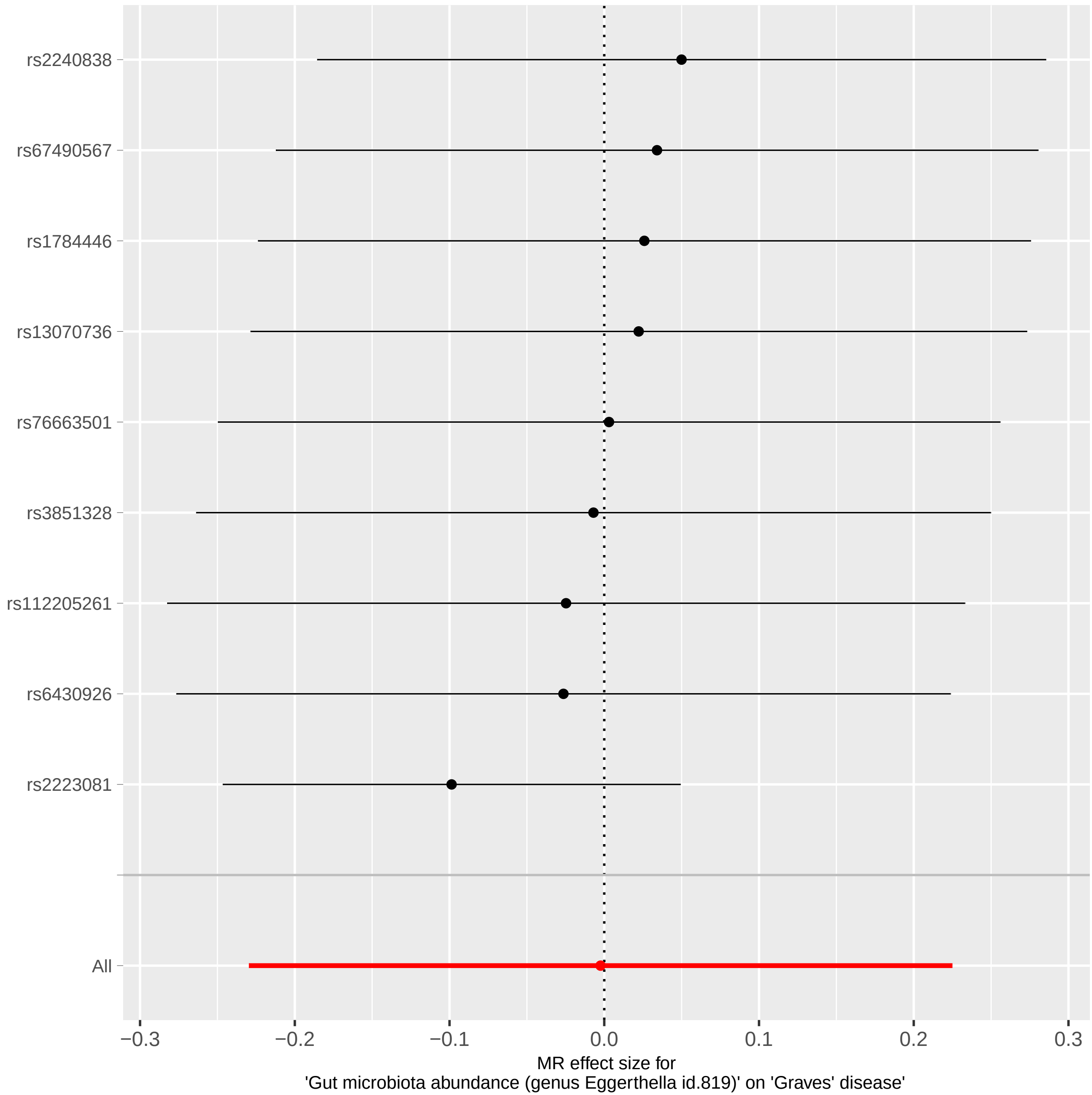

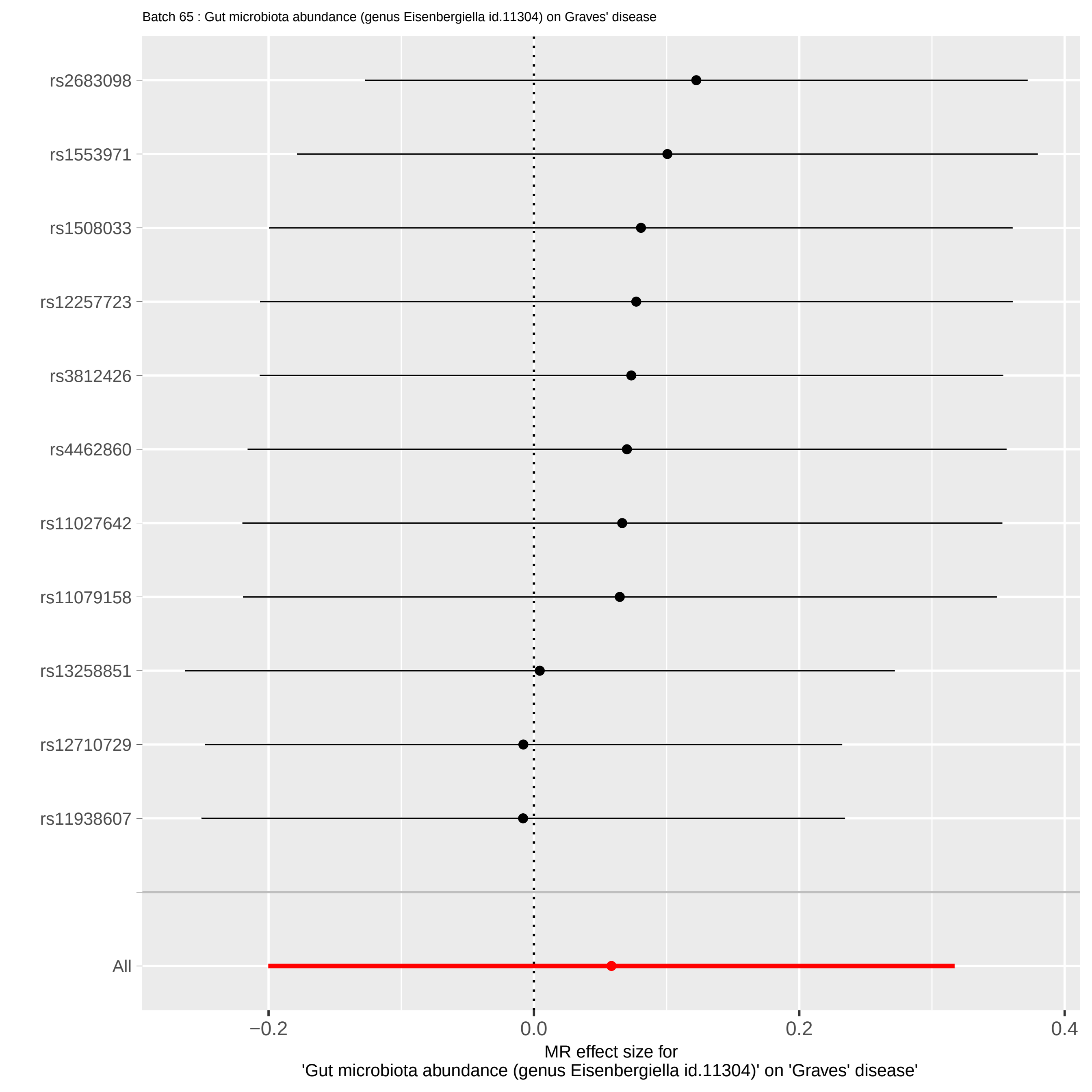

Batch 65 : Gut microbiota abundance (genus Eisenbergiella id.11304) on Graves' disease
rs2683098
rs1553971
rs1508033
rs12257723
rs3812426
rs4462860
rs11027642
rs11079158
rs13258851
rs12710729
rs11938607
All
−0.2
0.0
0.2
0.4
MR effect size for
'Gut microbiota abundance (genus Eisenbergiella id.11304)' on 'Graves' disease'

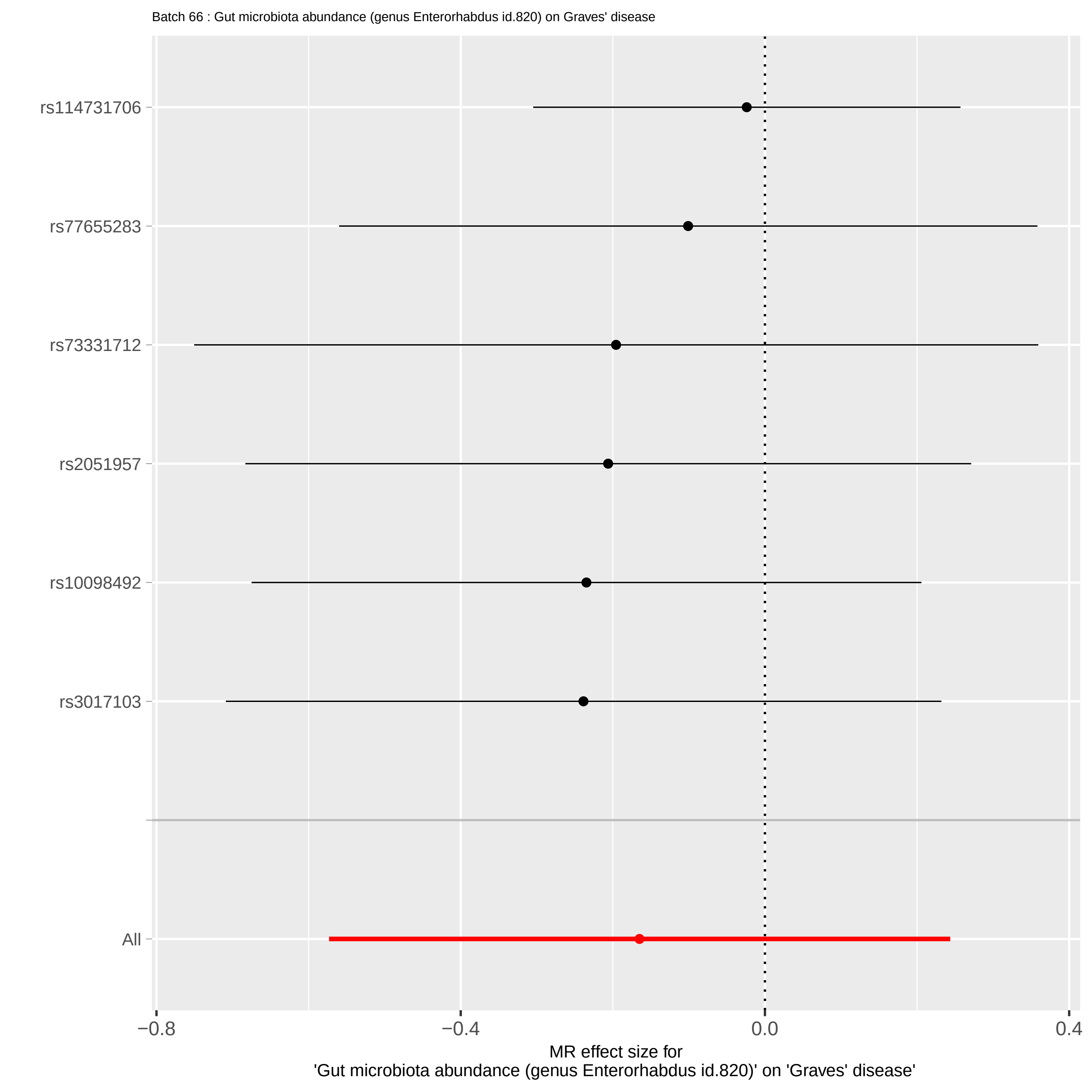

Batch 66 : Gut microbiota abundance (genus Enterorhabdus id.820) on Graves' disease
rs114731706
rs77655283
rs73331712
rs2051957
rs10098492
rs3017103
All
−0.8
−0.4
0.0
0.4
MR effect size for
'Gut microbiota abundance (genus Enterorhabdus id.820)' on 'Graves' disease'

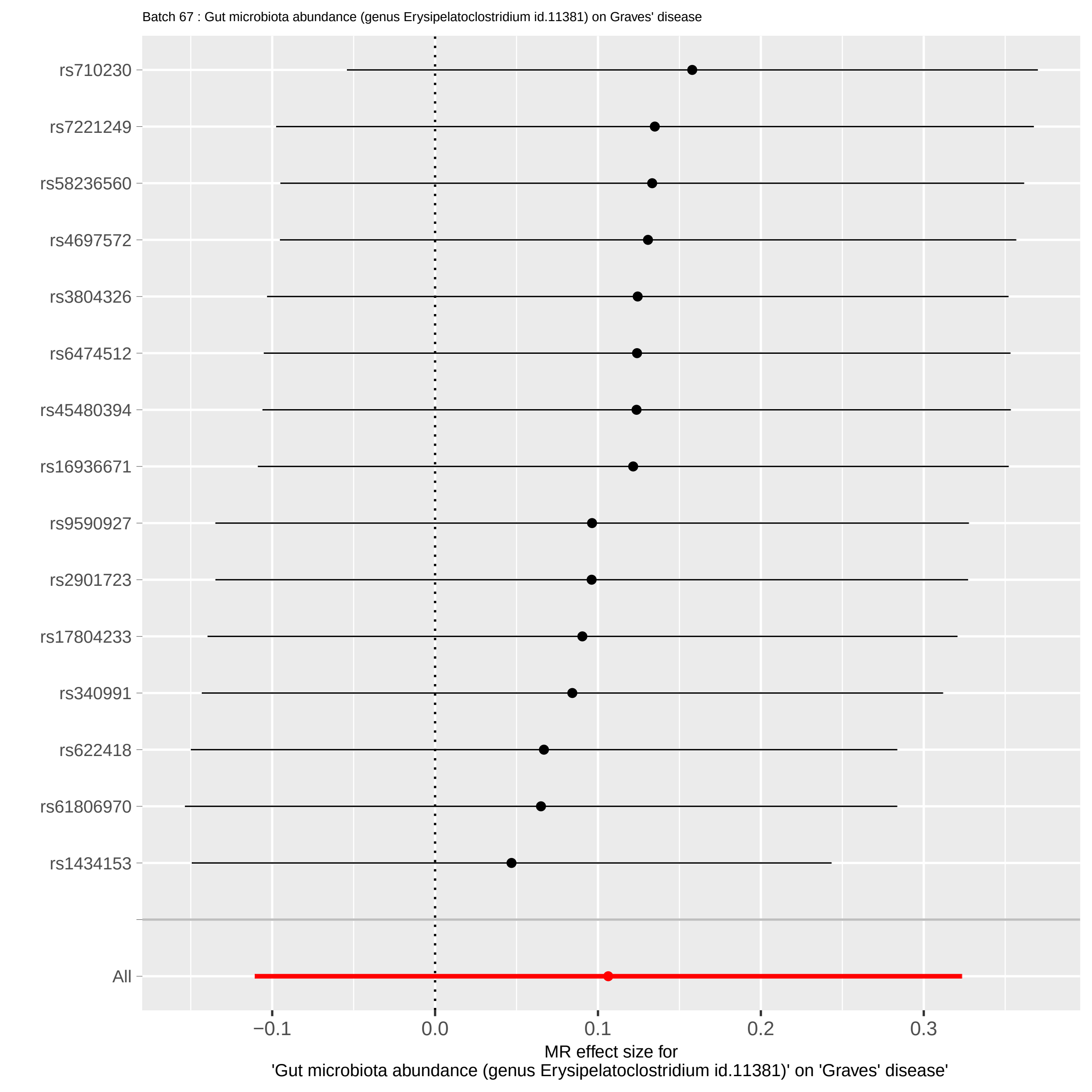
Batch 67 : Gut microbiota abundance (genus Erysipelatoclostridium id.11381) on Graves' disease
rs710230
rs7221249
rs58236560
rs4697572
rs3804326
rs6474512
rs45480394
rs16936671
rs9590927
rs2901723
rs17804233
rs340991
rs622418
rs61806970
rs1434153
All
−0.1
0.0
0.1
0.2
0.3
MR effect size for
'Gut microbiota abundance (genus Erysipelatoclostridium id.11381)' on 'Graves' disease'

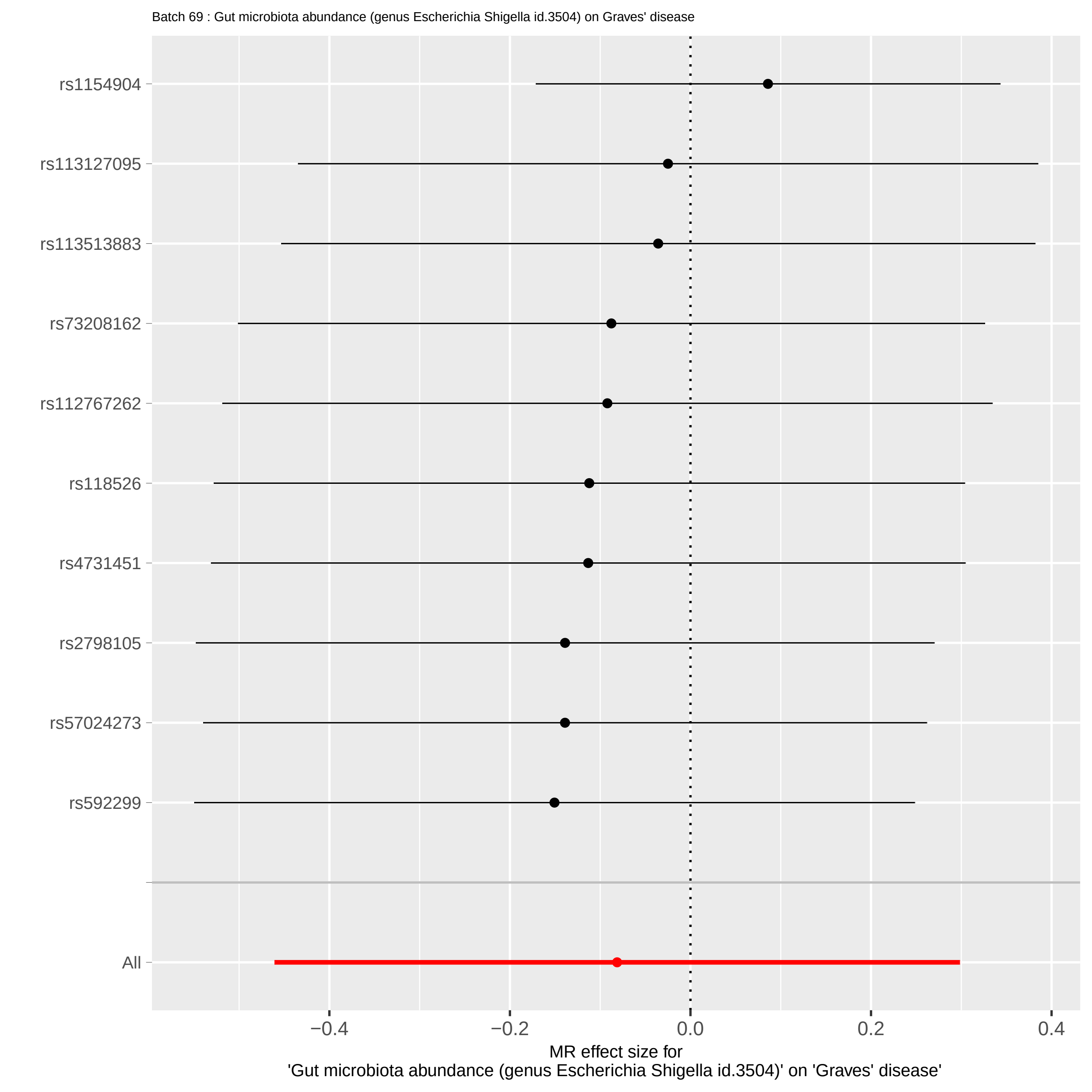
Batch 69 : Gut microbiota abundance (genus Escherichia Shigella id.3504) on Graves' disease
rs1154904
rs113127095
rs113513883
rs73208162
rs112767262
rs118526
rs4731451
rs2798105
rs57024273
rs592299
All
−0.4
−0.2
0.0
0.2
0.4
MR effect size for
'Gut microbiota abundance (genus Escherichia Shigella id.3504)' on 'Graves' disease'

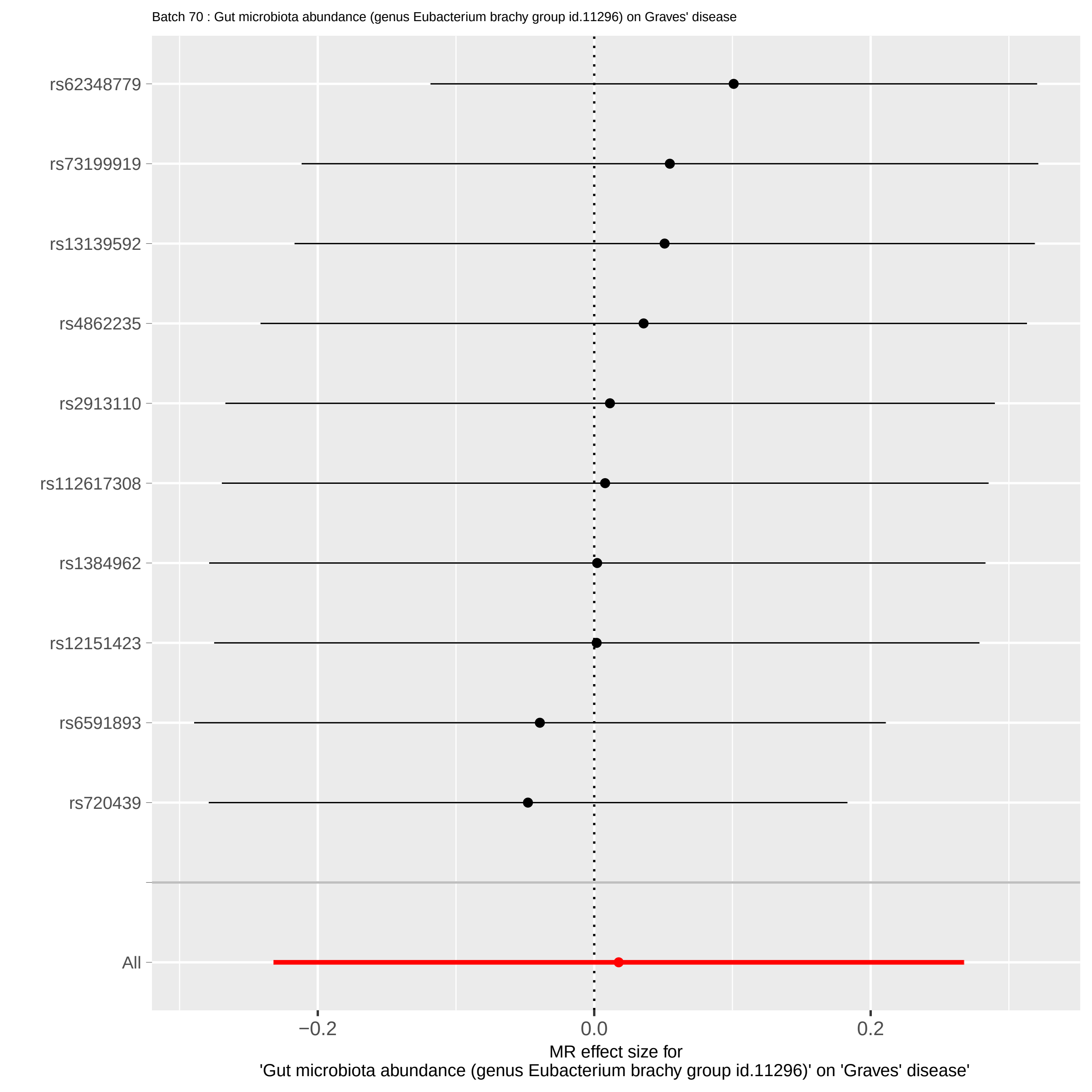

Batch 70 : Gut microbiota abundance (genus Eubacterium brachy group id.11296) on Graves' disease
rs62348779
rs73199919
rs13139592
rs4862235
rs2913110
rs112617308
rs1384962
rs12151423
rs6591893
rs720439
All
−0.2
0.0
0.2
MR effect size for
'Gut microbiota abundance (genus Eubacterium brachy group id.11296)' on 'Graves' disease'

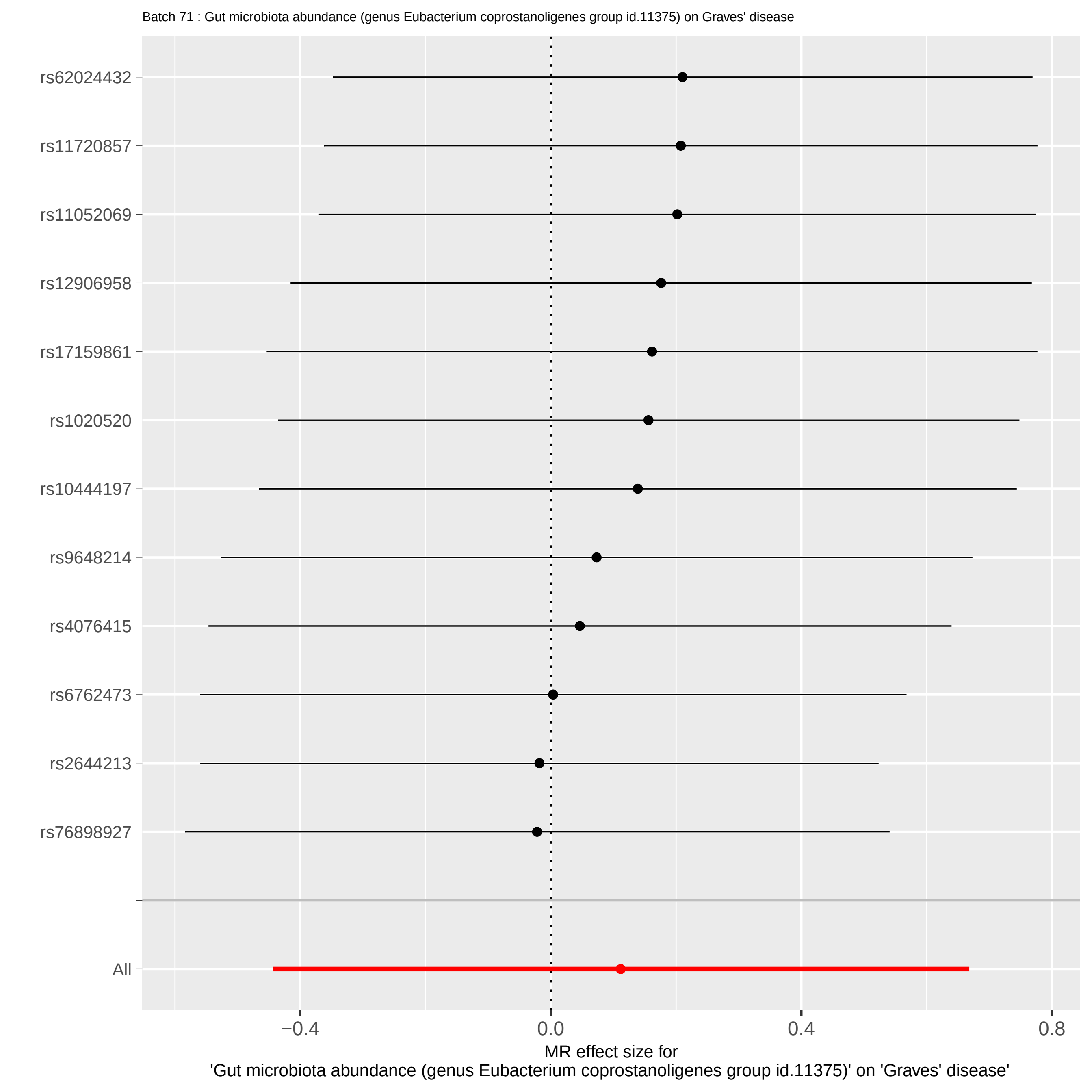

Batch 71 : Gut microbiota abundance (genus Eubacterium coprostanoligenes group id.11375) on Graves' disease
rs62024432
rs11720857
rs11052069
rs12906958
rs17159861
rs1020520
rs10444197
rs9648214
rs4076415
rs6762473
rs2644213
rs76898927
All
−0.4
0.0
0.4
0.8
MR effect size for
'Gut microbiota abundance (genus Eubacterium coprostanoligenes group id.11375)' on 'Graves' disease'

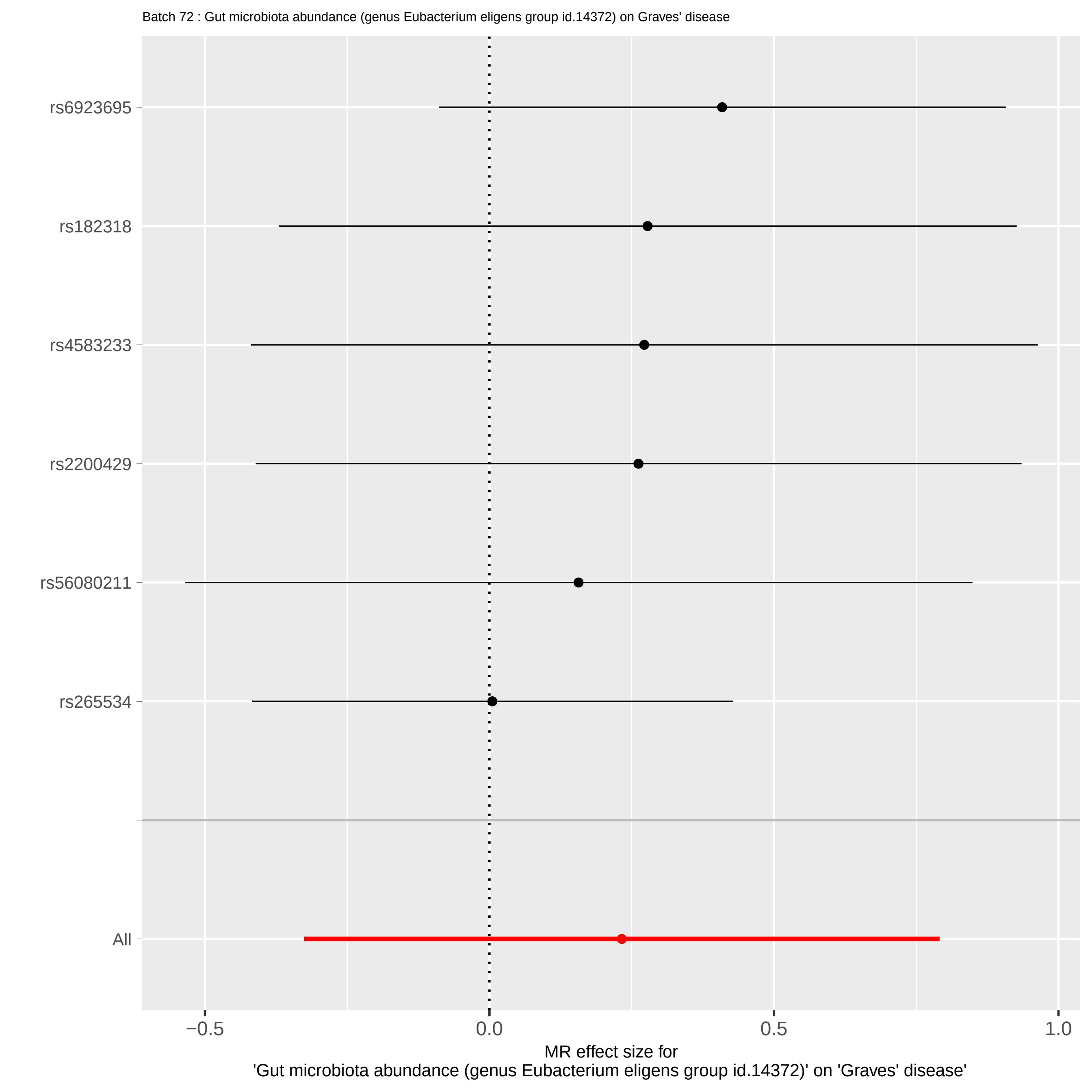
Batch 72 : Gut microbiota abundance (genus Eubacterium eligens group id.14372) on Graves' disease
rs6923695
rs182318
rs4583233
rs2200429
rs56080211
rs265534
All
−0.5
0.0
0.5
1.0
MR effect size for
'Gut microbiota abundance (genus Eubacterium eligens group id.14372)' on 'Graves' disease'

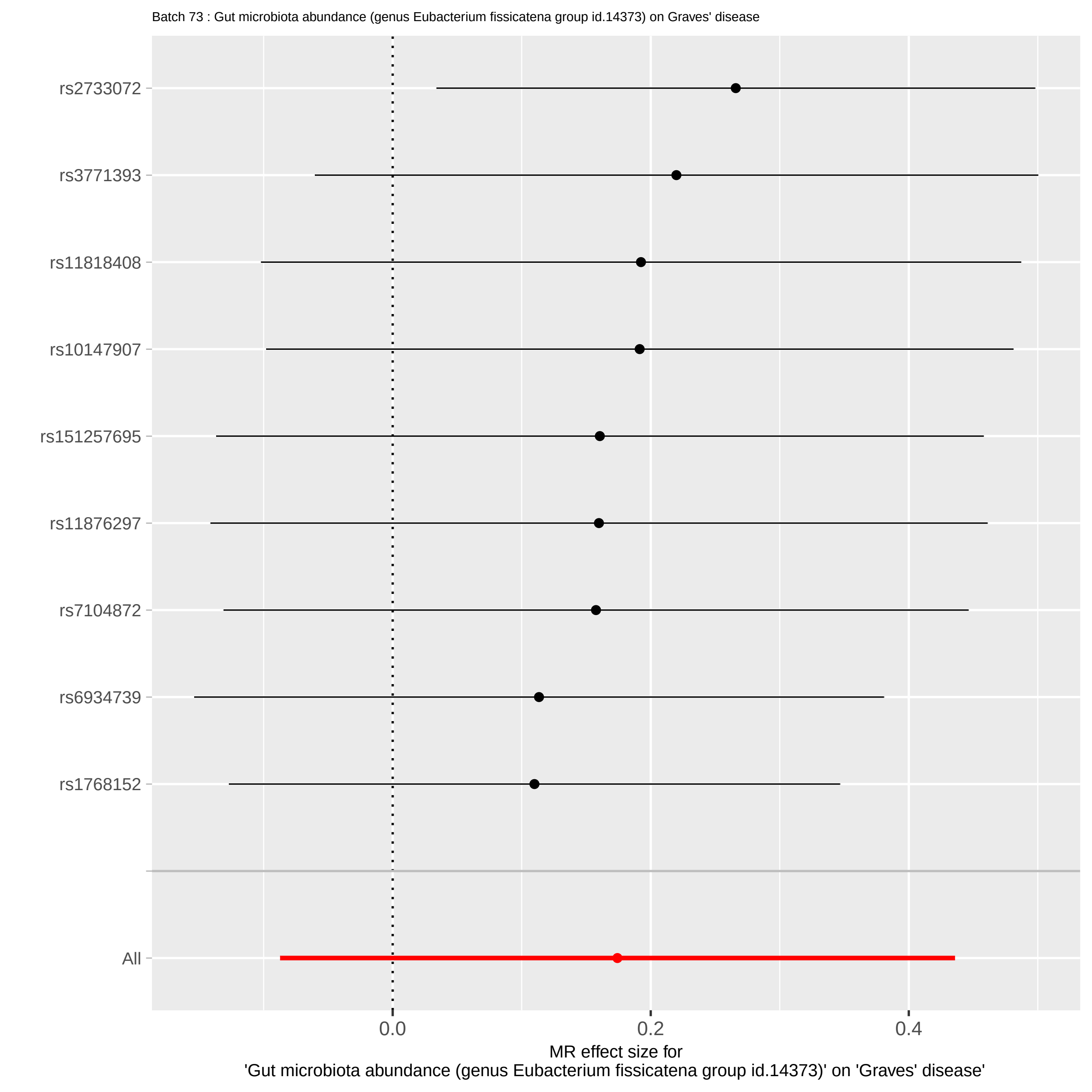
Batch 73 : Gut microbiota abundance (genus Eubacterium fissicatena group id.14373) on Graves' disease
rs2733072
rs3771393
rs11818408
rs10147907
rs151257695
rs11876297
rs7104872
rs6934739
rs1768152
All
0.0
0.2
0.4
MR effect size for
'Gut microbiota abundance (genus Eubacterium fissicatena group id.14373)' on 'Graves' disease'

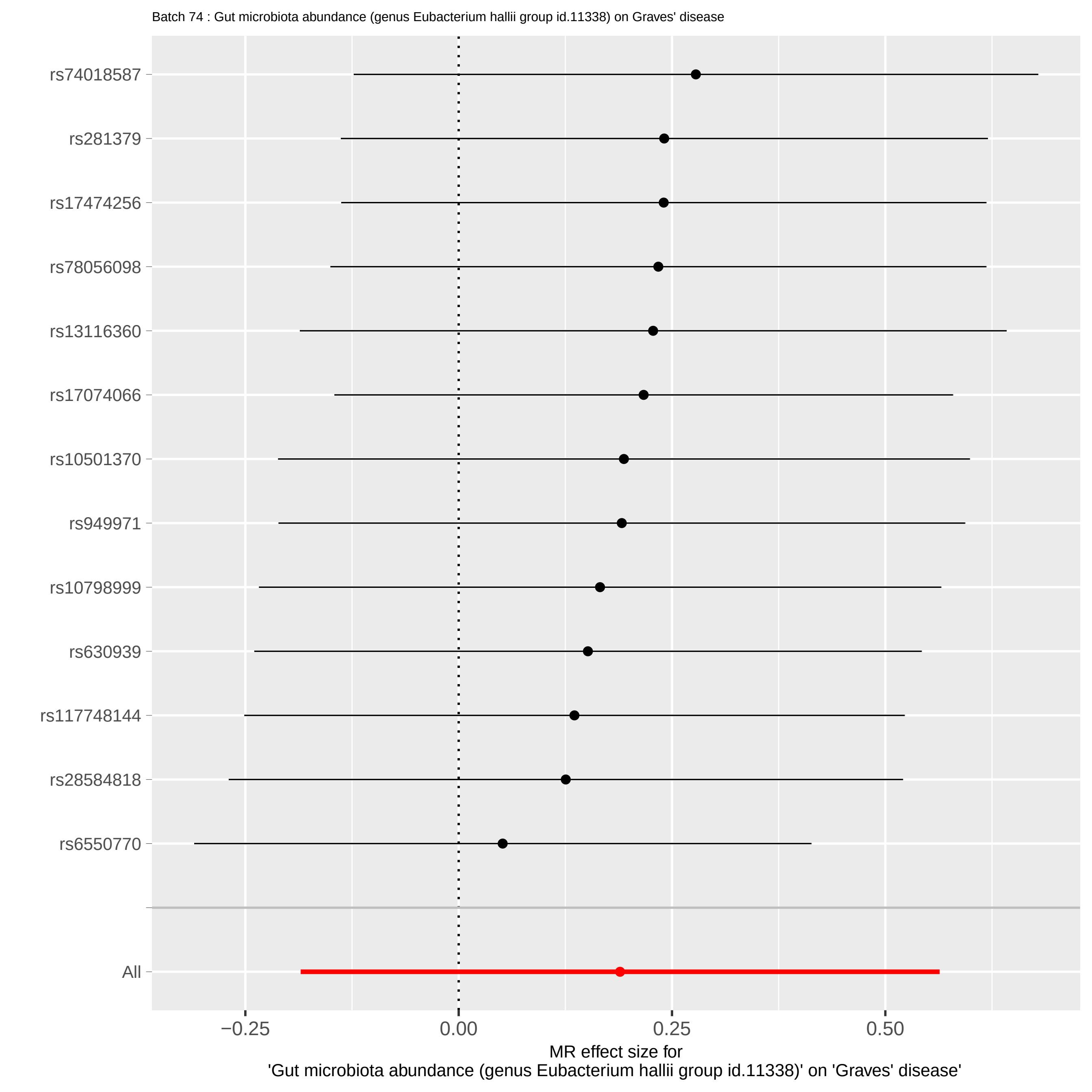

Batch 74 : Gut microbiota abundance (genus Eubacterium hallii group id.11338) on Graves' disease
rs74018587
rs281379
rs17474256
rs78056098
rs13116360
rs17074066
rs10501370
rs949971
rs10798999
rs630939
rs117748144
rs28584818
rs6550770
All
−0.25
0.00
0.25
0.50
MR effect size for
'Gut microbiota abundance (genus Eubacterium hallii group id.11338)' on 'Graves' disease'

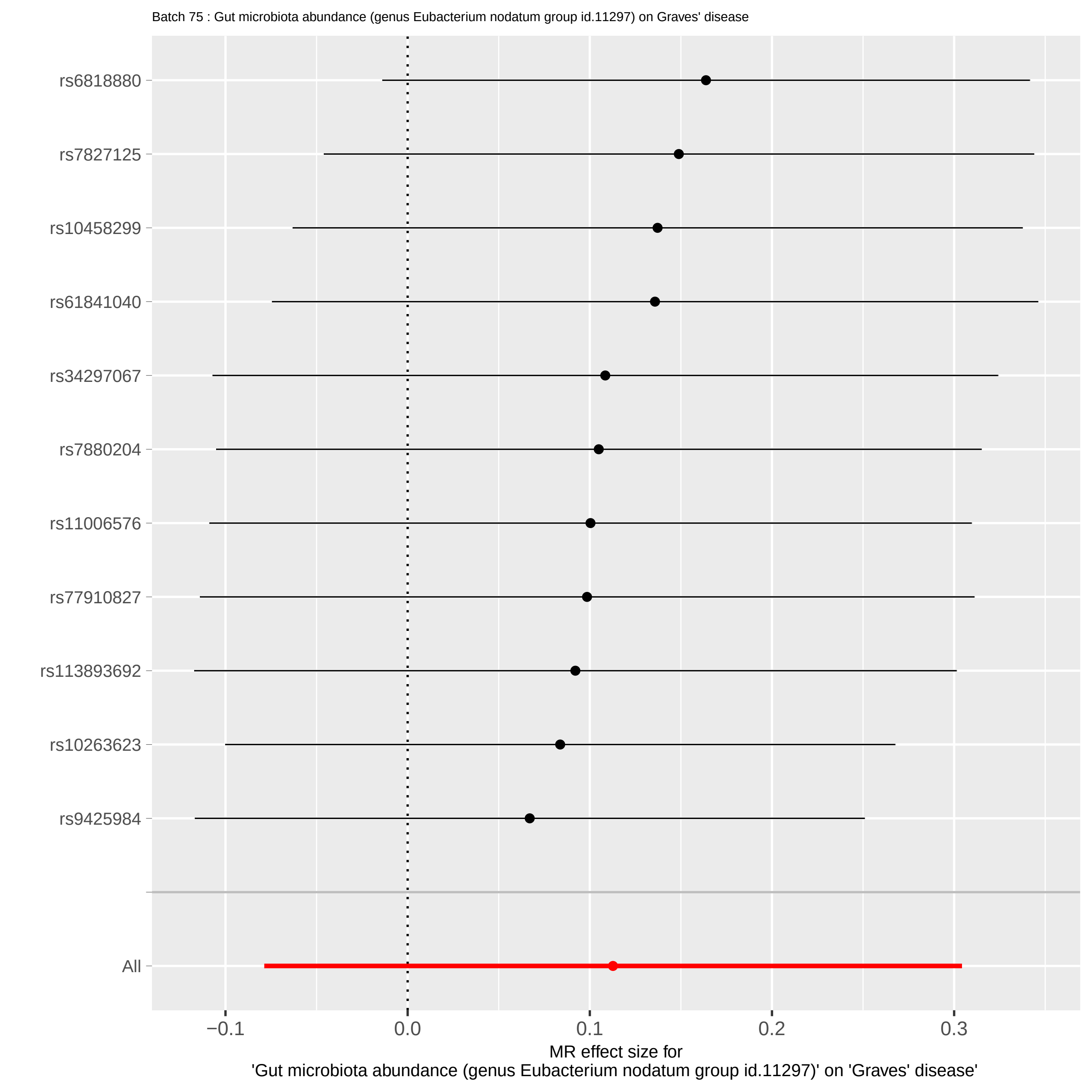
Batch 75 : Gut microbiota abundance (genus Eubacterium nodatum group id.11297) on Graves' disease
rs6818880
rs7827125
rs10458299
rs61841040
rs34297067
rs7880204
rs11006576
rs77910827
rs113893692
rs10263623
rs9425984
All
−0.1
0.0
0.1
0.2
0.3
MR effect size for
'Gut microbiota abundance (genus Eubacterium nodatum group id.11297)' on 'Graves' disease'

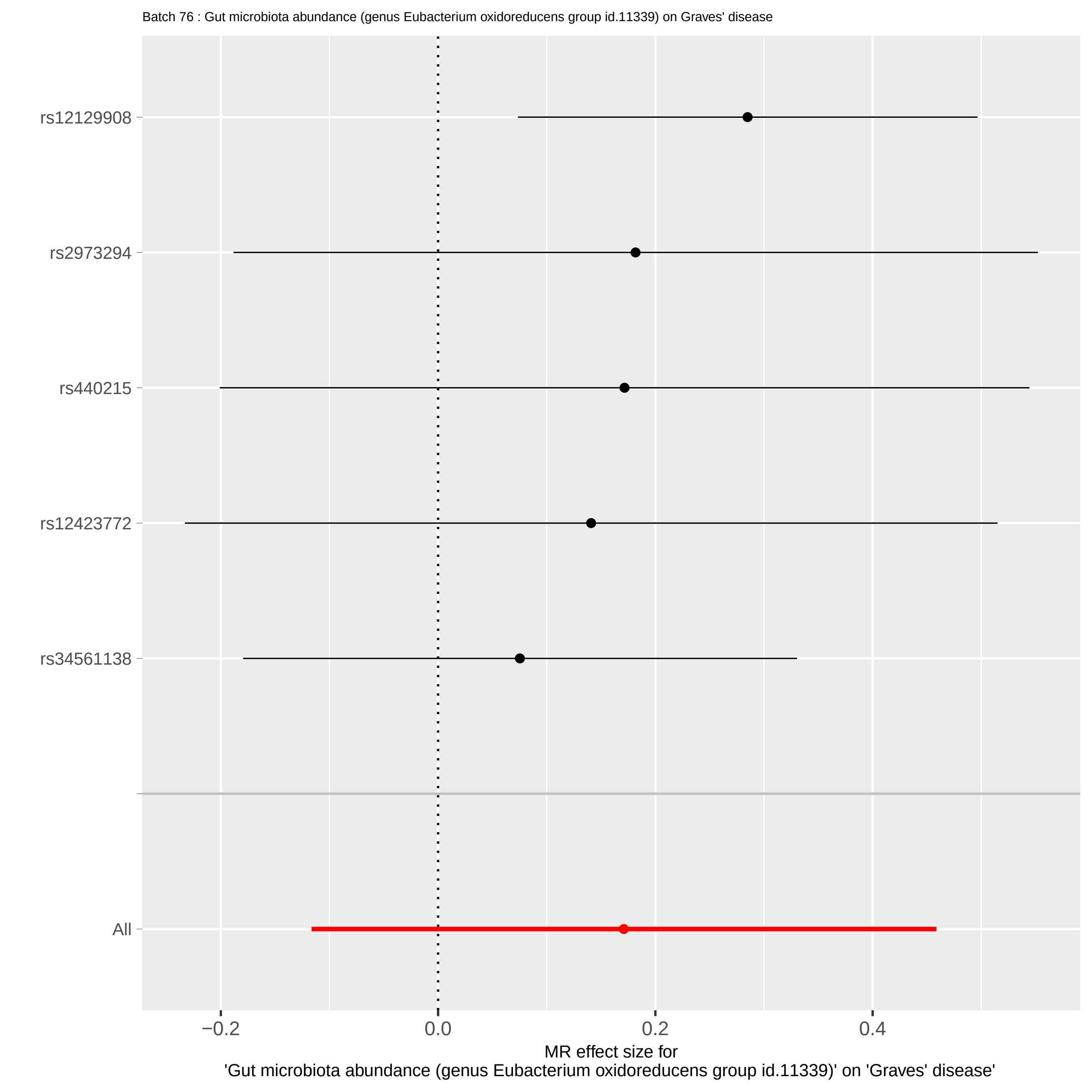

Batch 76 : Gut microbiota abundance (genus Eubacterium oxidoreducens group id.11339) on Graves' disease
rs12129908
rs2973294
rs440215
rs12423772
rs34561138
All
−0.2
0.0
0.2
0.4
MR effect size for
'Gut microbiota abundance (genus Eubacterium oxidoreducens group id.11339)' on 'Graves' disease'

Batch 77 : Gut microbiota abundance (genus Eubacterium rectale group id.14374) on Graves' disease

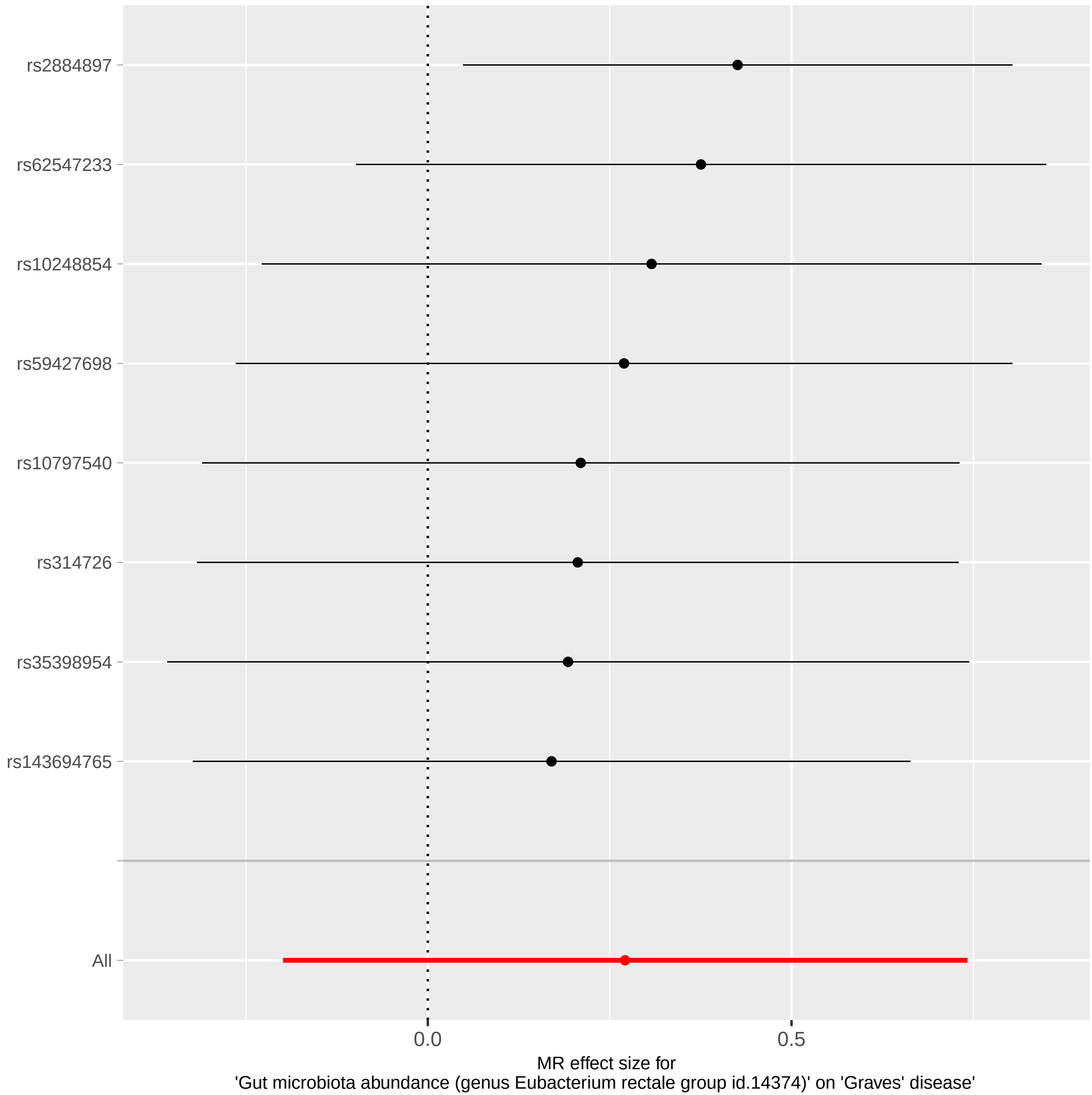

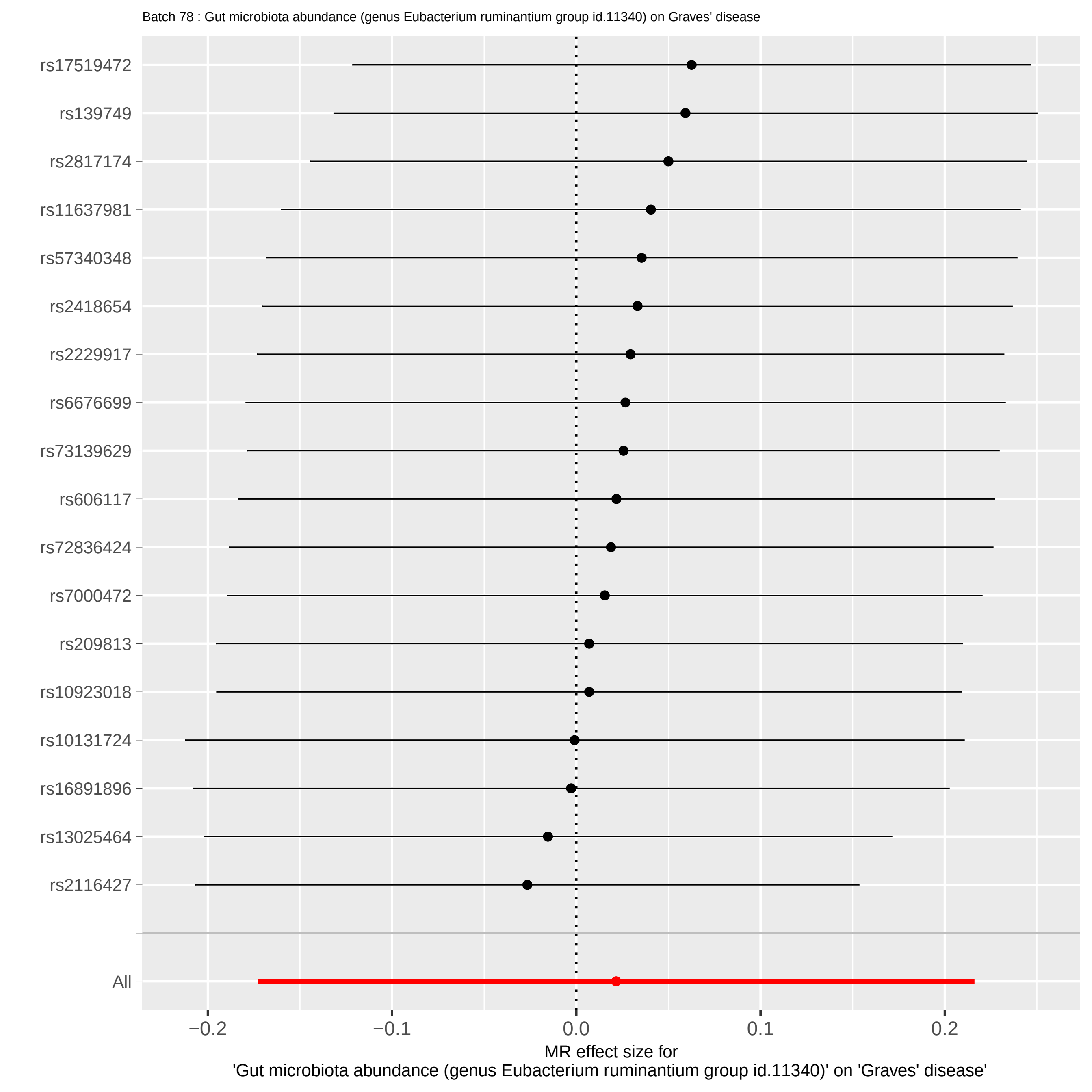

Batch 78 : Gut microbiota abundance (genus Eubacterium ruminantium group id.11340) on Graves' disease
rs17519472
rs139749
rs2817174
rs11637981
rs57340348
rs2418654
rs2229917
rs6676699
rs73139629
rs606117
rs72836424
rs7000472
rs209813
rs10923018
rs10131724
rs16891896
rs13025464
rs2116427
All
−0.2
−0.1
0.0
0.1
0.2
MR effect size for
'Gut microbiota abundance (genus Eubacterium ruminantium group id.11340)' on 'Graves' disease'

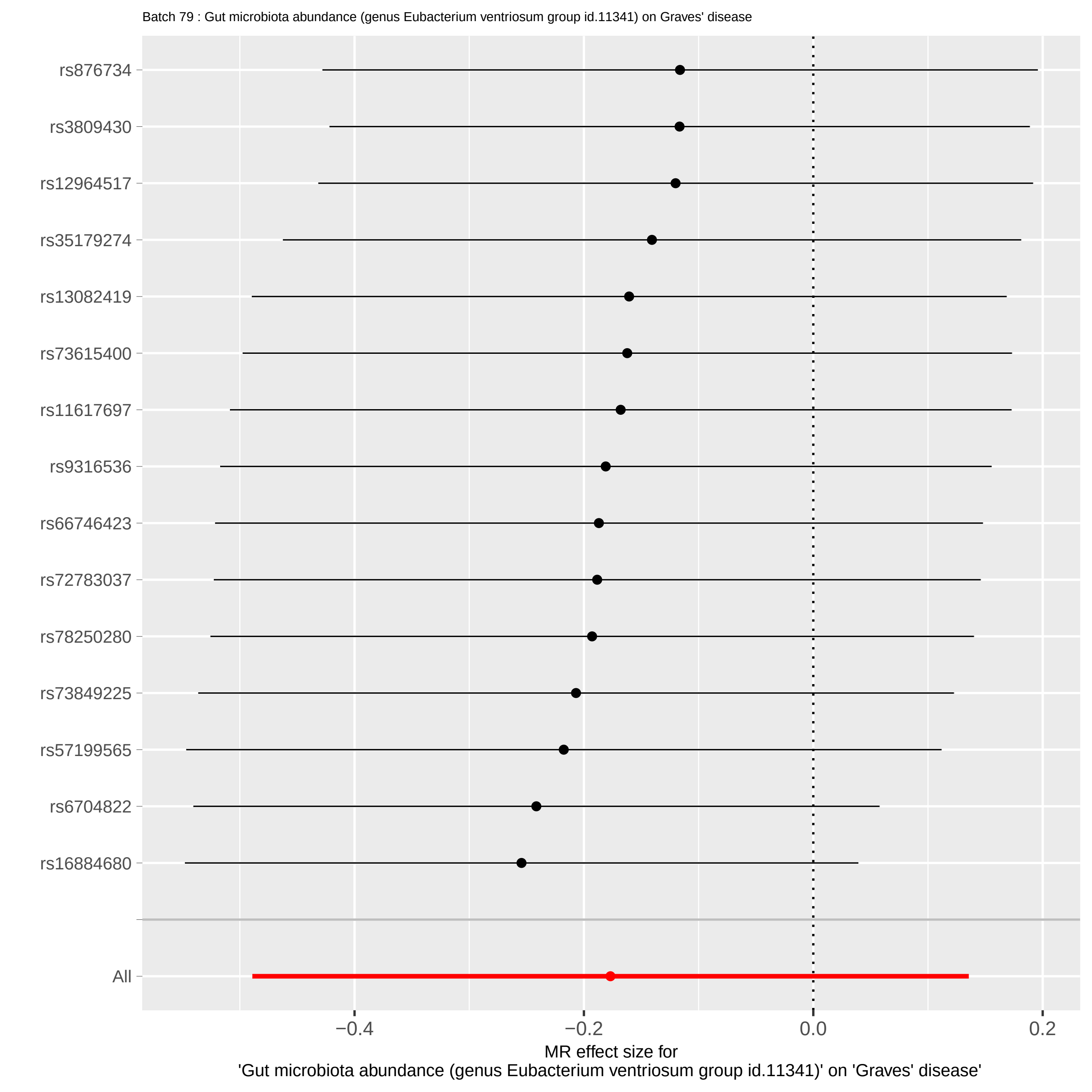
Batch 79 : Gut microbiota abundance (genus Eubacterium ventriosum group id.11341) on Graves' disease
rs876734
rs3809430
rs12964517
rs35179274
rs13082419
rs73615400
rs11617697
rs9316536
rs66746423
rs72783037
rs78250280
rs73849225
rs57199565
rs6704822
rs16884680
All
−0.4
−0.2
0.0
0.2
MR effect size for
'Gut microbiota abundance (genus Eubacterium ventriosum group id.11341)' on 'Graves' disease'

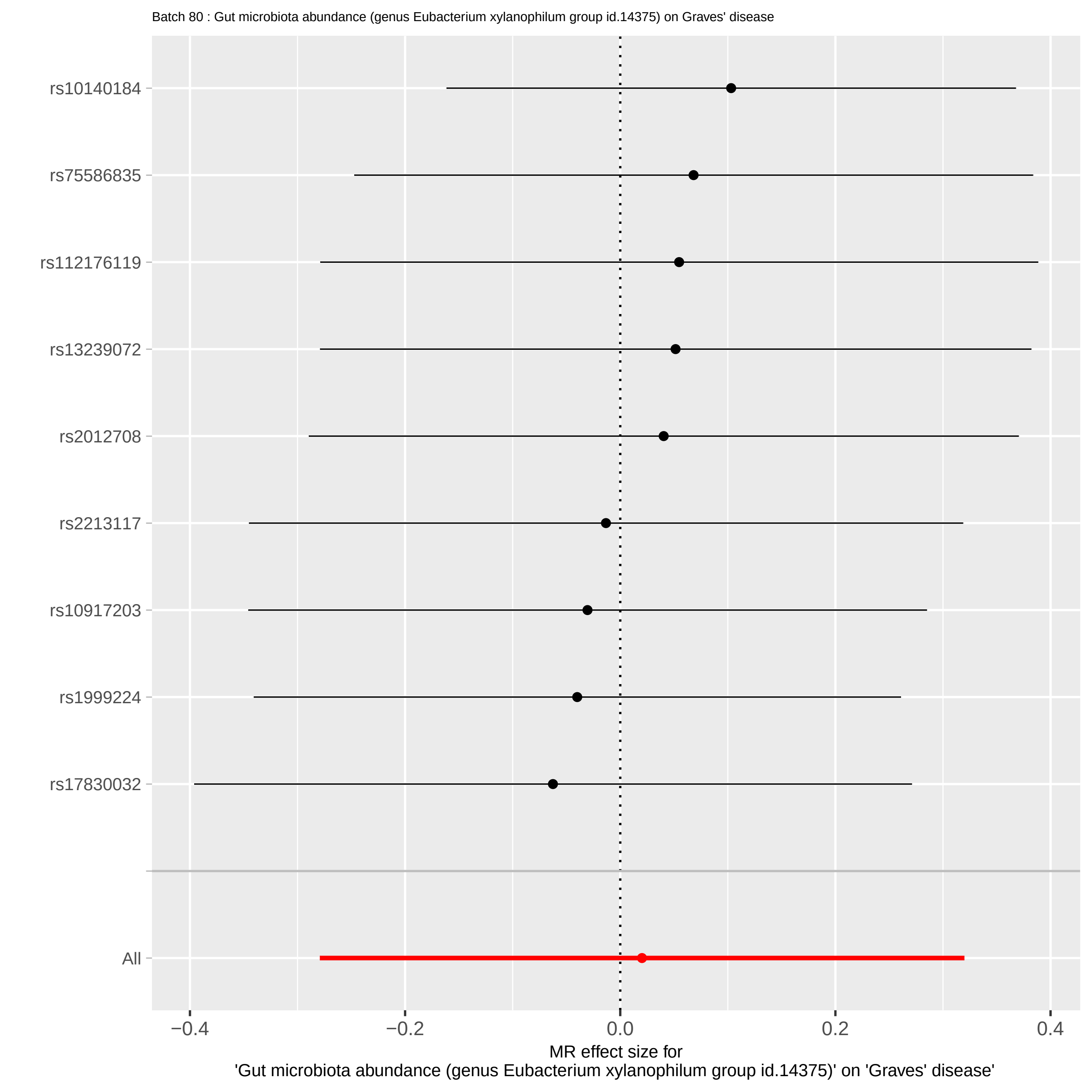

Batch 80 : Gut microbiota abundance (genus Eubacterium xylanophilum group id.14375) on Graves' disease
rs10140184
rs75586835
rs112176119
rs13239072
rs2012708
rs2213117
rs10917203
rs1999224
rs17830032
All
−0.4
−0.2
0.0
0.2
0.4
MR effect size for
'Gut microbiota abundance (genus Eubacterium xylanophilum group id.14375)' on 'Graves' disease'

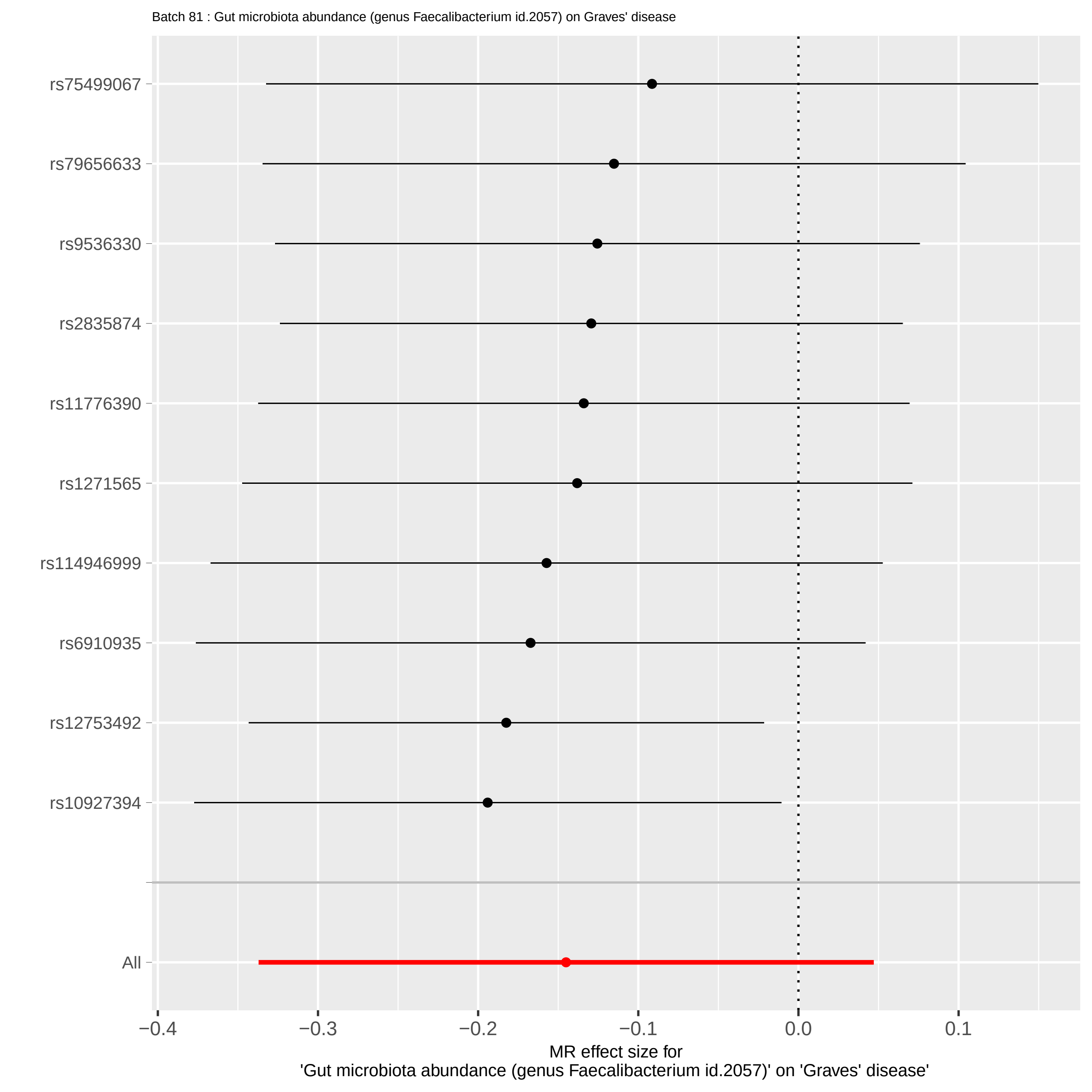
Batch 81 : Gut microbiota abundance (genus Faecalibacterium id.2057) on Graves' disease
rs75499067
rs79656633
rs9536330
rs2835874
rs11776390
rs1271565
rs114946999
rs6910935
rs12753492
rs10927394
All
−0.4
−0.3
−0.2
−0.1
0.0
0.1
MR effect size for
'Gut microbiota abundance (genus Faecalibacterium id.2057)' on 'Graves' disease'

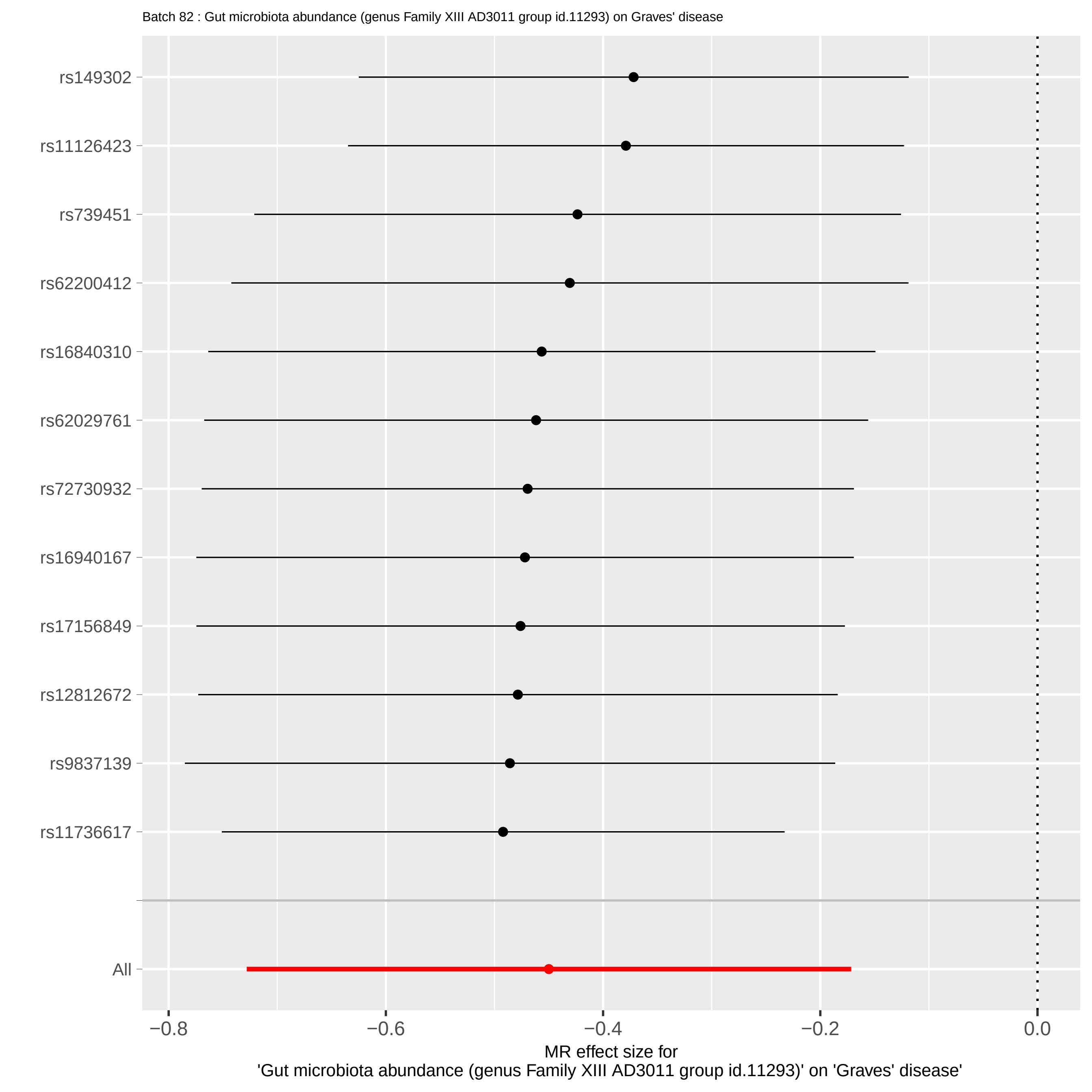

Batch 82 : Gut microbiota abundance (genus Family XIII AD3011 group id.11293) on Graves' disease
rs149302
rs11126423
rs739451
rs62200412
rs16840310
rs62029761
rs72730932
rs16940167
rs17156849
rs12812672
rs9837139
rs11736617
All
−0.8
−0.6
−0.4
−0.2
0.0
MR effect size for
'Gut microbiota abundance (genus Family XIII AD3011 group id.11293)' on 'Graves' disease'

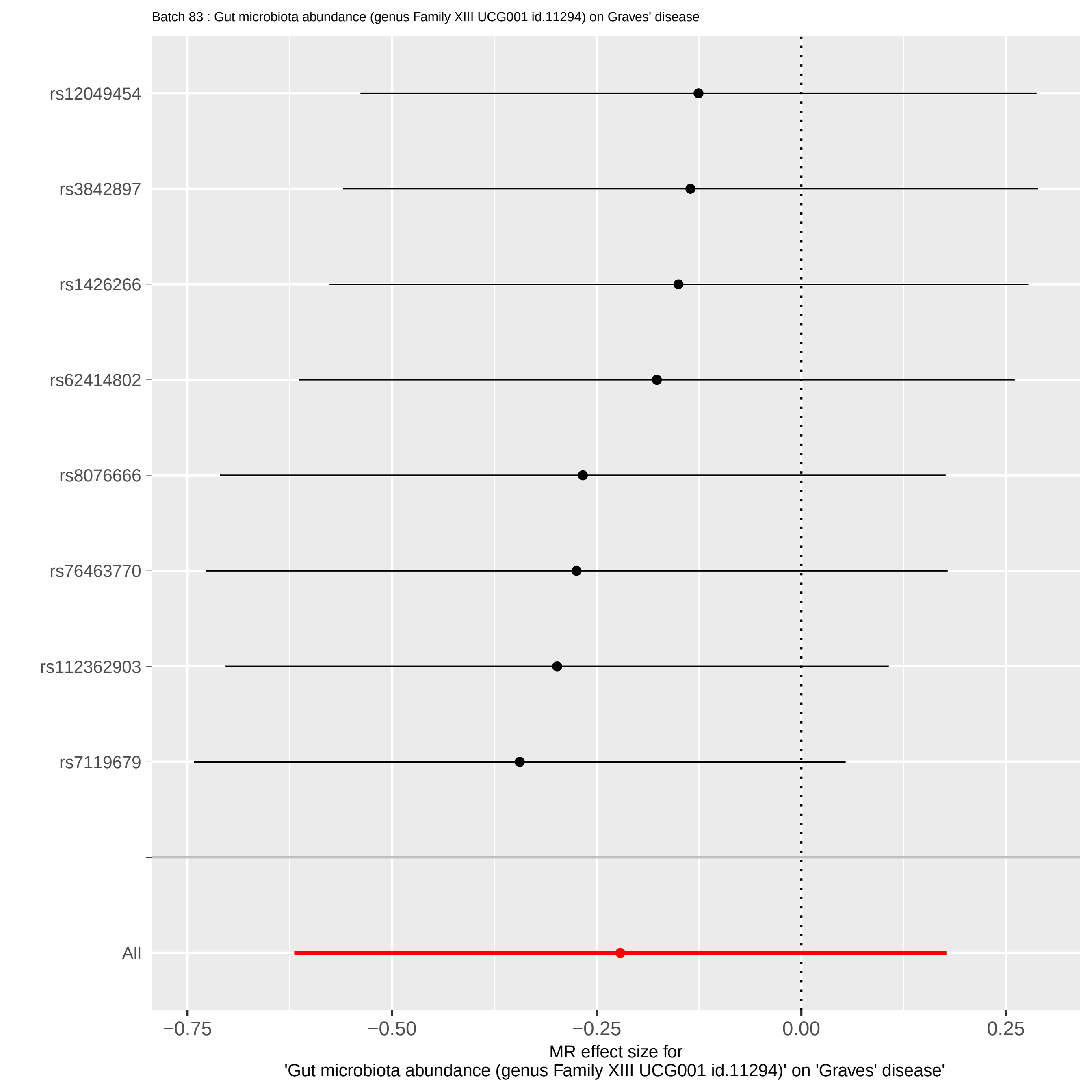

Batch 83 : Gut microbiota abundance (genus Family XIII UCG001 id.11294) on Graves' disease
rs12049454
rs3842897
rs1426266
rs62414802
rs8076666
rs76463770
rs112362903
rs7119679
All
−0.75
−0.50
−0.25
0.00
0.25
MR effect size for
'Gut microbiota abundance (genus Family XIII UCG001 id.11294)' on 'Graves' disease'

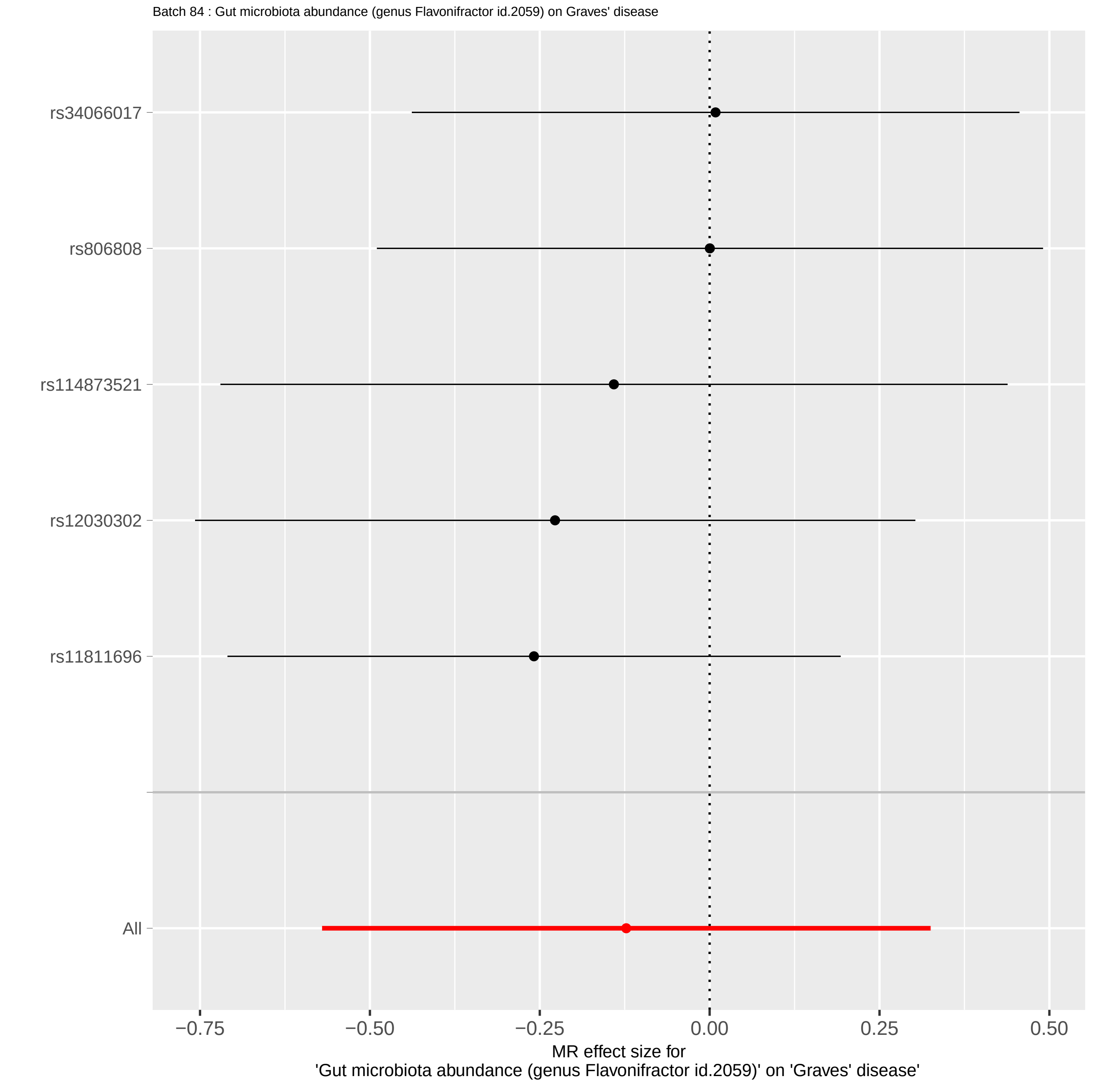

Batch 84 : Gut microbiota abundance (genus Flavonifractor id.2059) on Graves' disease
rs34066017
rs806808
rs114873521
rs12030302
rs11811696
All
−0.75
−0.50
−0.25
0.00
0.25
0.50
MR effect size for
'Gut microbiota abundance (genus Flavonifractor id.2059)' on 'Graves' disease'

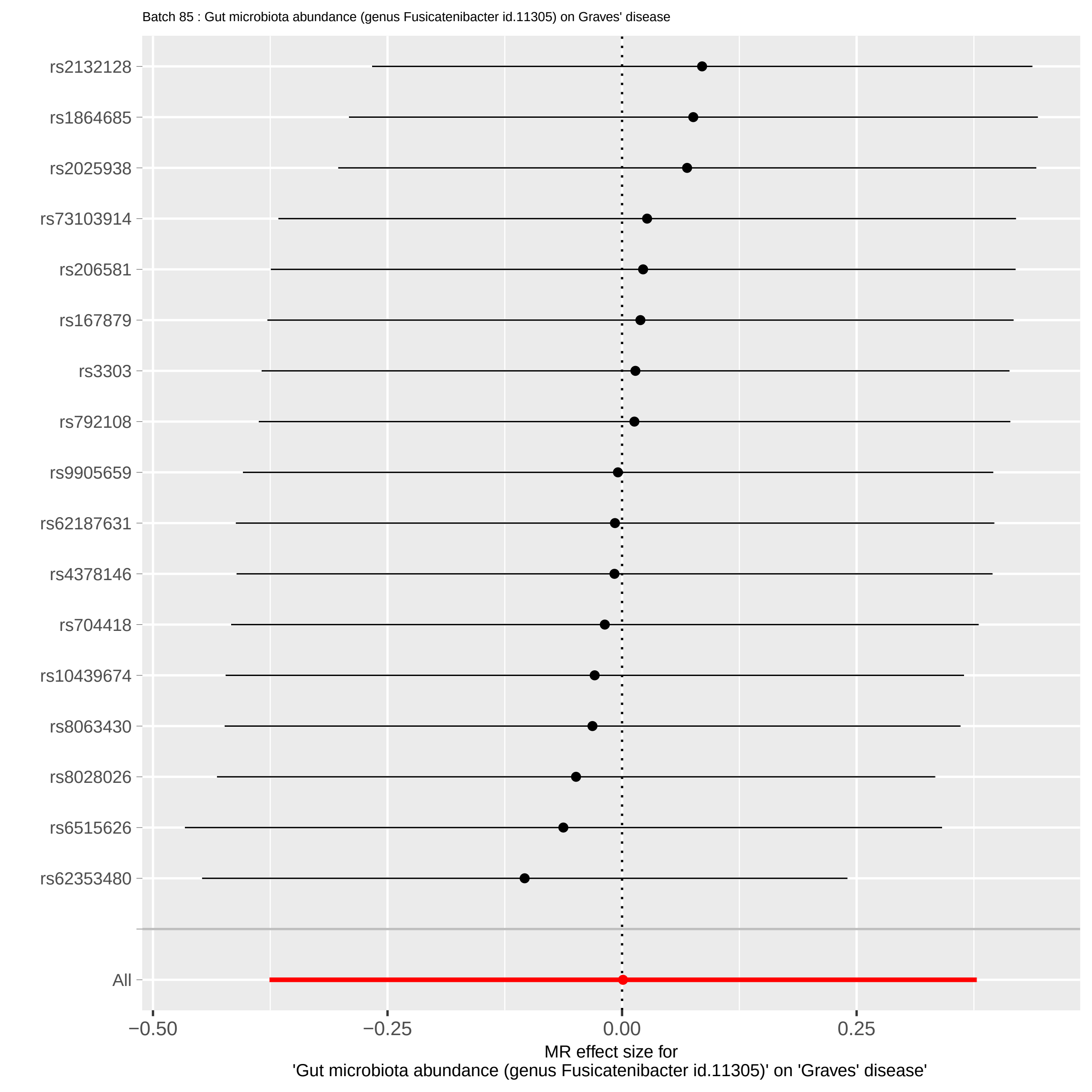

Batch 85 : Gut microbiota abundance (genus Fusicatenibacter id.11305) on Graves' disease
rs2132128
rs1864685
rs2025938
rs73103914
rs206581
rs167879
rs3303
rs792108
rs9905659
rs62187631
rs4378146
rs704418
rs10439674
rs8063430
rs8028026
rs6515626
rs62353480
All
−0.50
−0.25
0.00
0.25
MR effect size for
'Gut microbiota abundance (genus Fusicatenibacter id.11305)' on 'Graves' disease'

Batch 86 : Gut microbiota abundance (genus Gordonibacter id.821) on Graves' disease

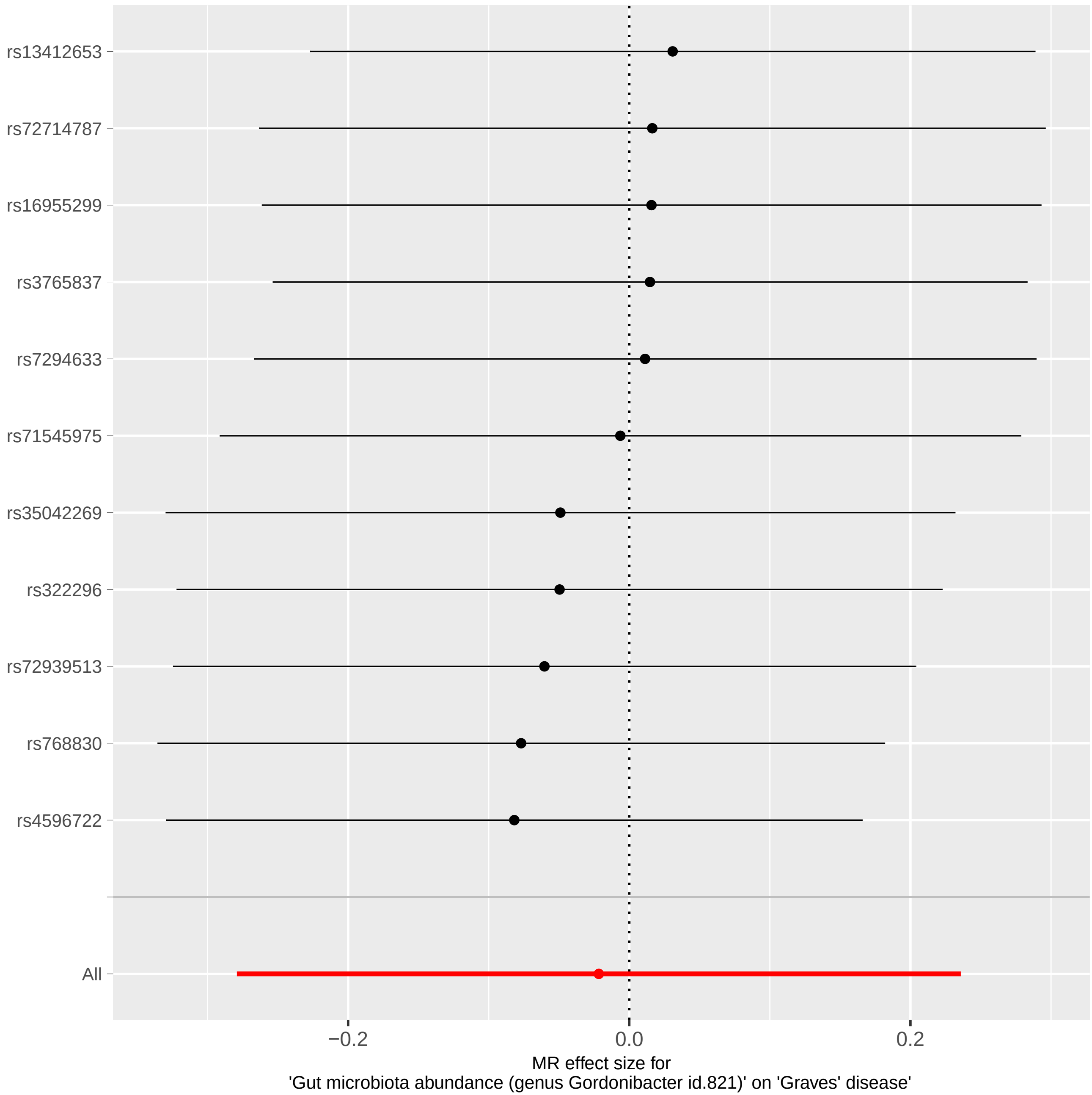

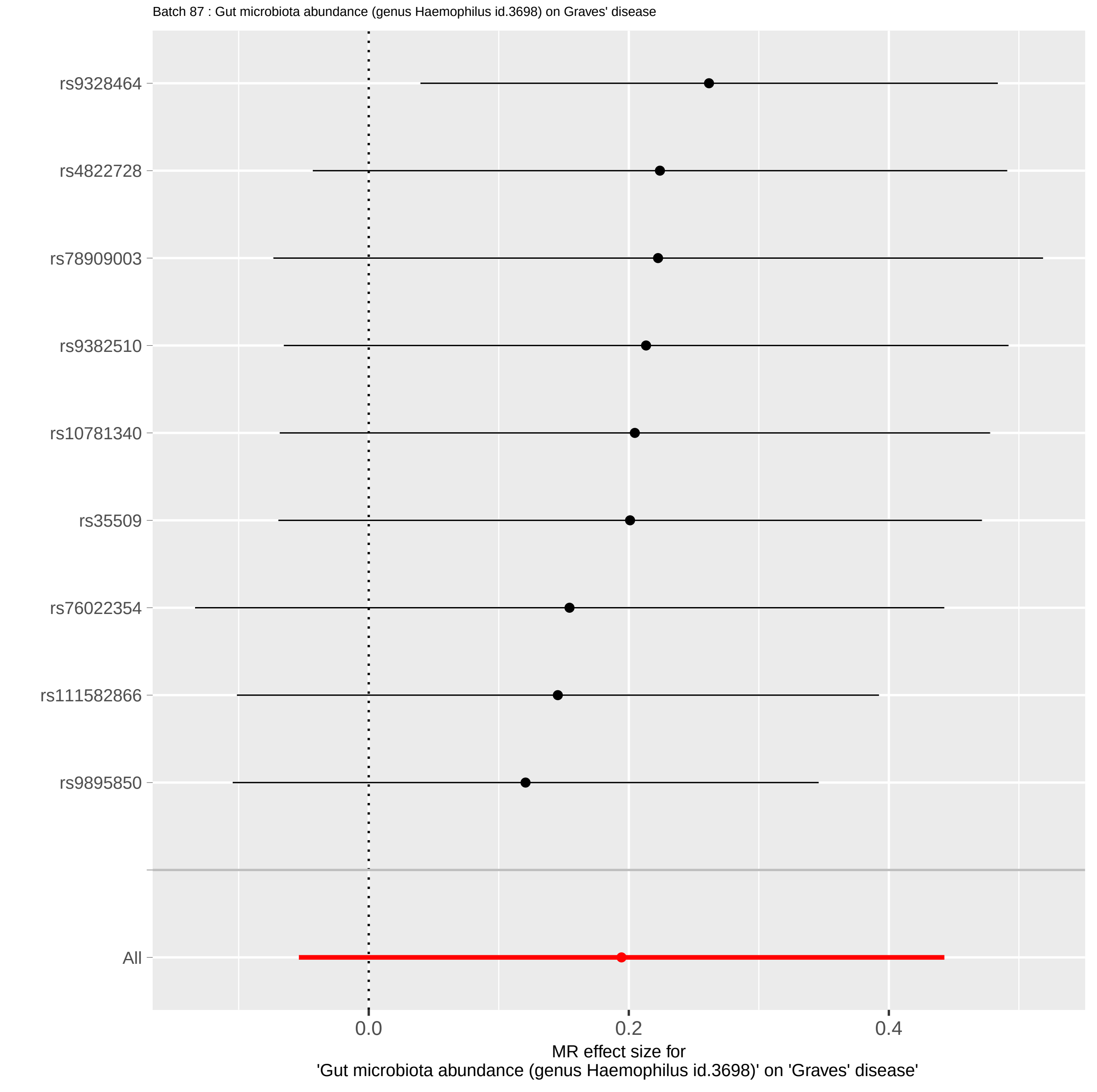
Batch 87 : Gut microbiota abundance (genus Haemophilus id.3698) on Graves' disease
rs9328464
rs4822728
rs78909003
rs9382510
rs10781340
rs35509
rs76022354
rs111582866
rs9895850
All
0.0
0.2
0.4
MR effect size for
'Gut microbiota abundance (genus Haemophilus id.3698)' on 'Graves' disease'

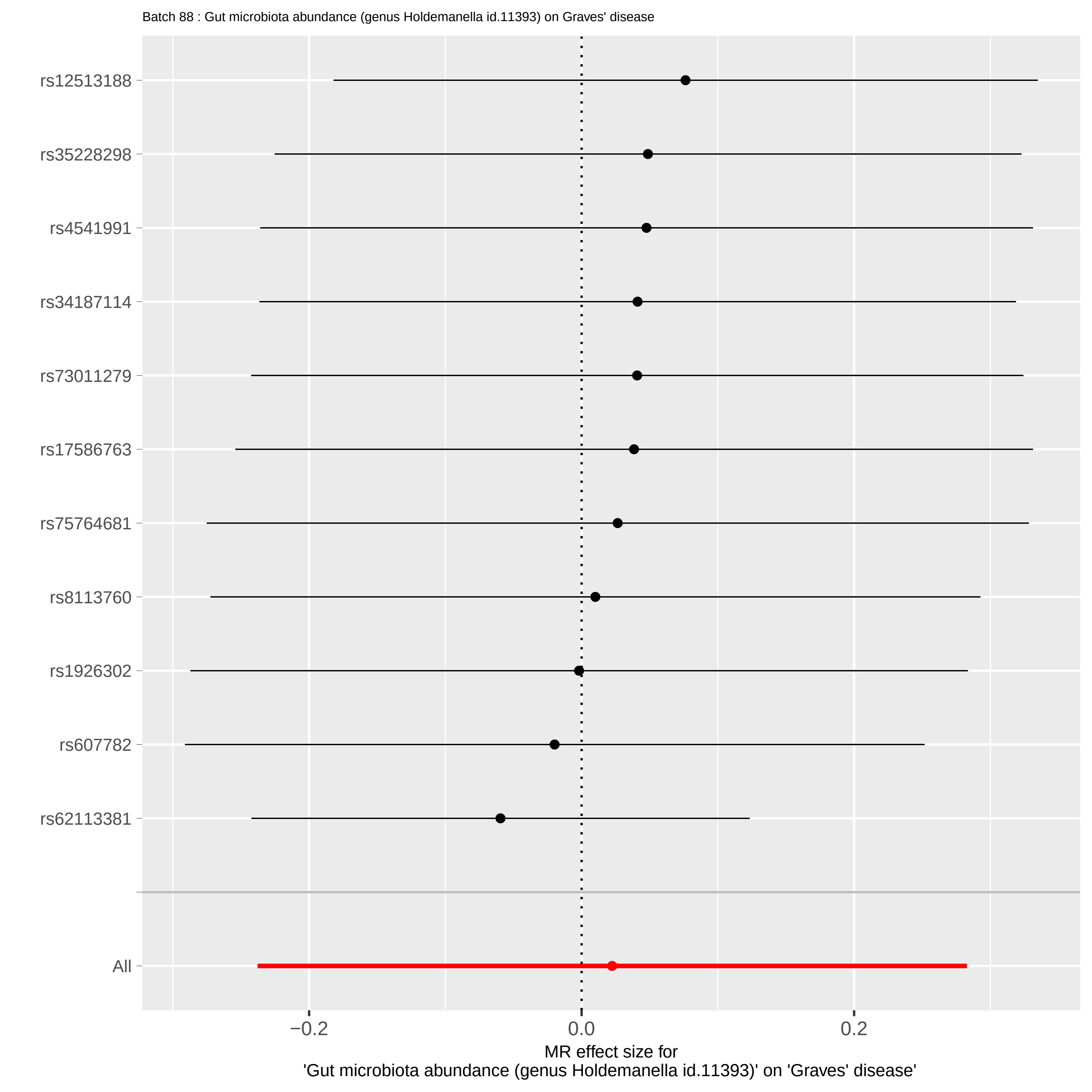

Batch 88 : Gut microbiota abundance (genus Holdemanella id.11393) on Graves' disease
rs12513188
rs35228298
rs4541991
rs34187114
rs73011279
rs17586763
rs75764681
rs8113760
rs1926302
rs607782
rs62113381
All
−0.2
0.0
0.2
MR effect size for
'Gut microbiota abundance (genus Holdemanella id.11393)' on 'Graves' disease'

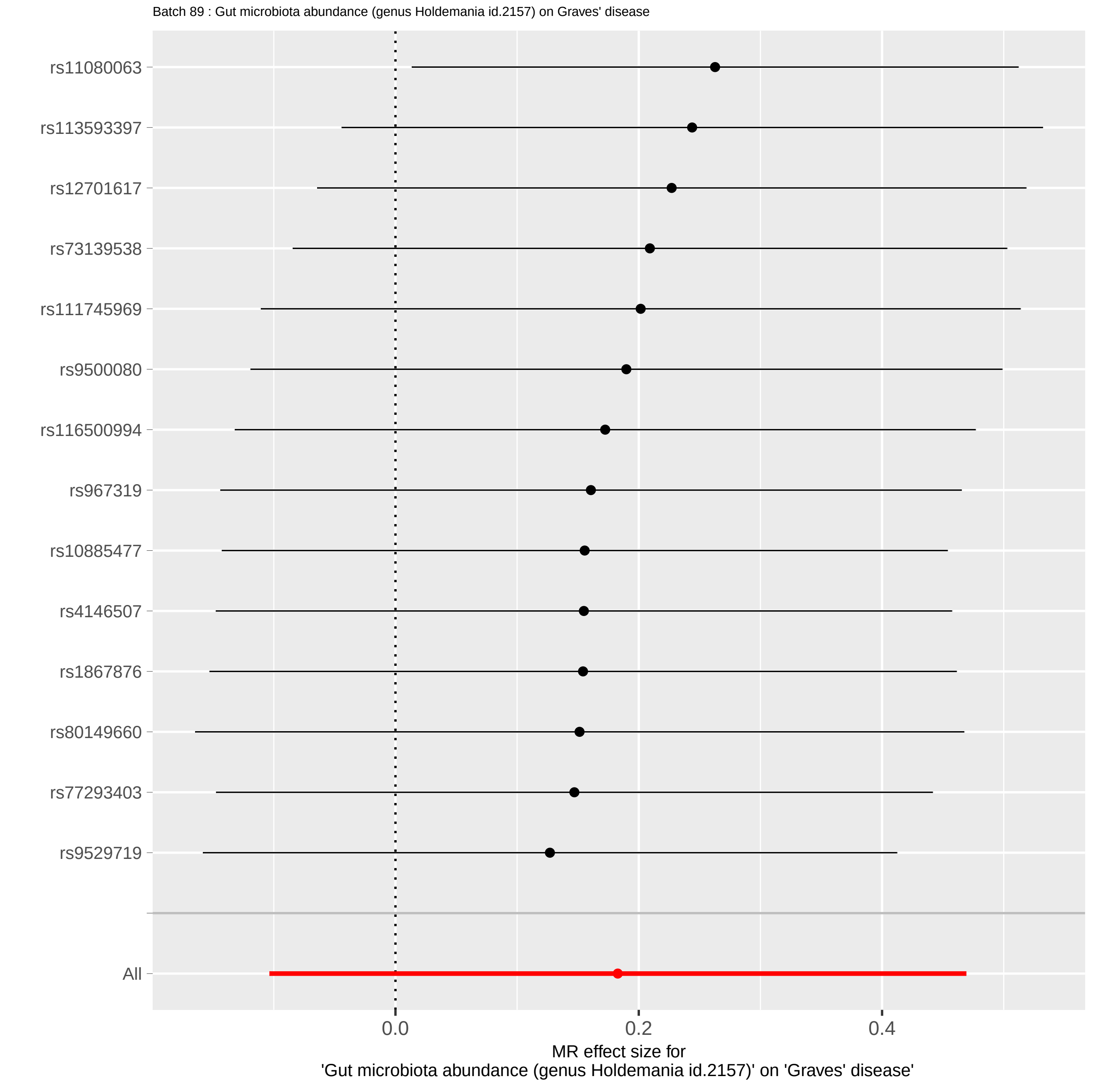

Batch 89 : Gut microbiota abundance (genus Holdemania id.2157) on Graves' disease
rs11080063
rs113593397
rs12701617
rs73139538
rs111745969
rs9500080
rs116500994
rs967319
rs10885477
rs4146507
rs1867876
rs80149660
rs77293403
rs9529719
All
0.0
0.2
0.4
MR effect size for
'Gut microbiota abundance (genus Holdemania id.2157)' on 'Graves' disease'

Batch 90 : Gut microbiota abundance (genus Howardella id.2000) on Graves' disease

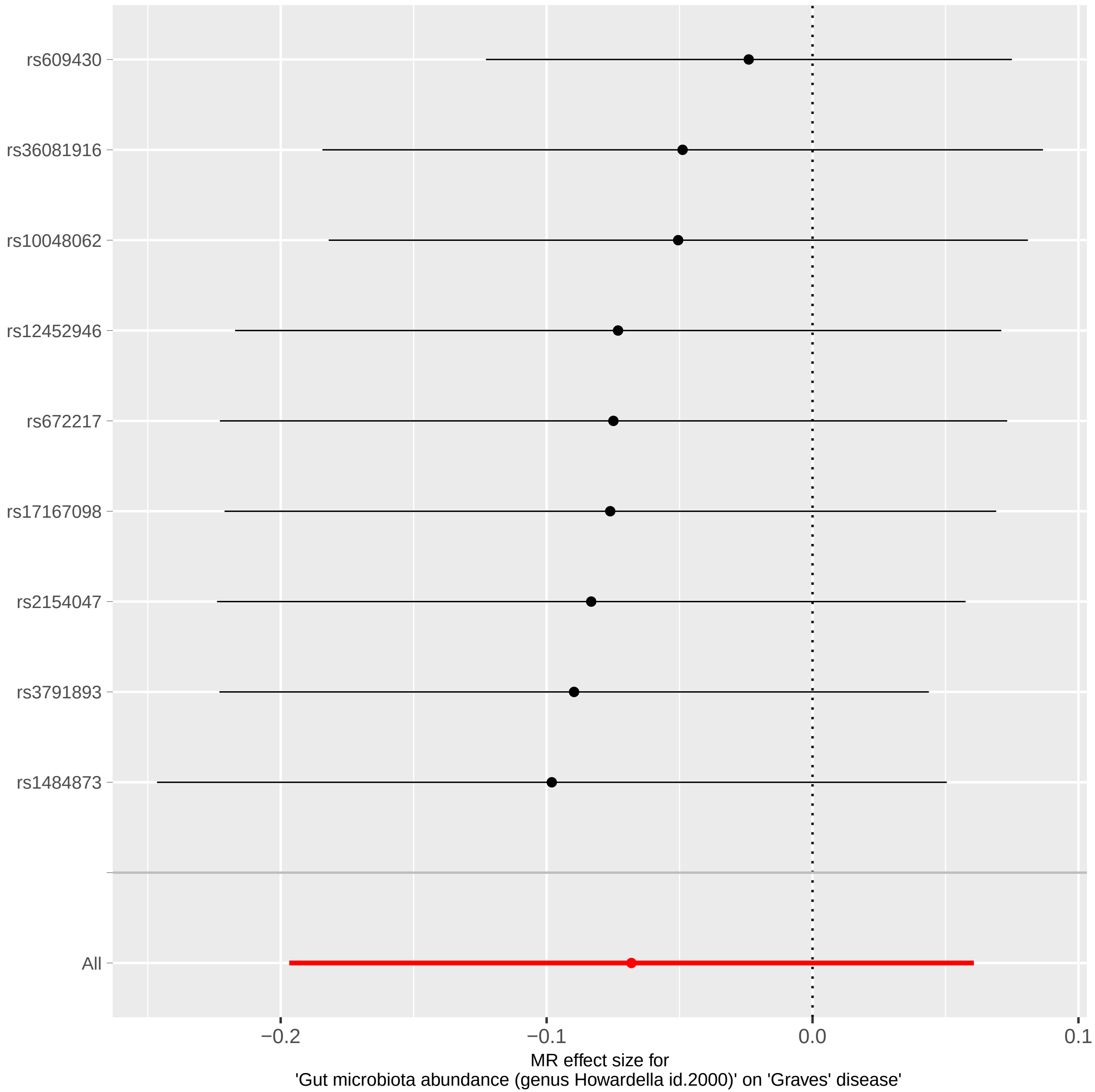

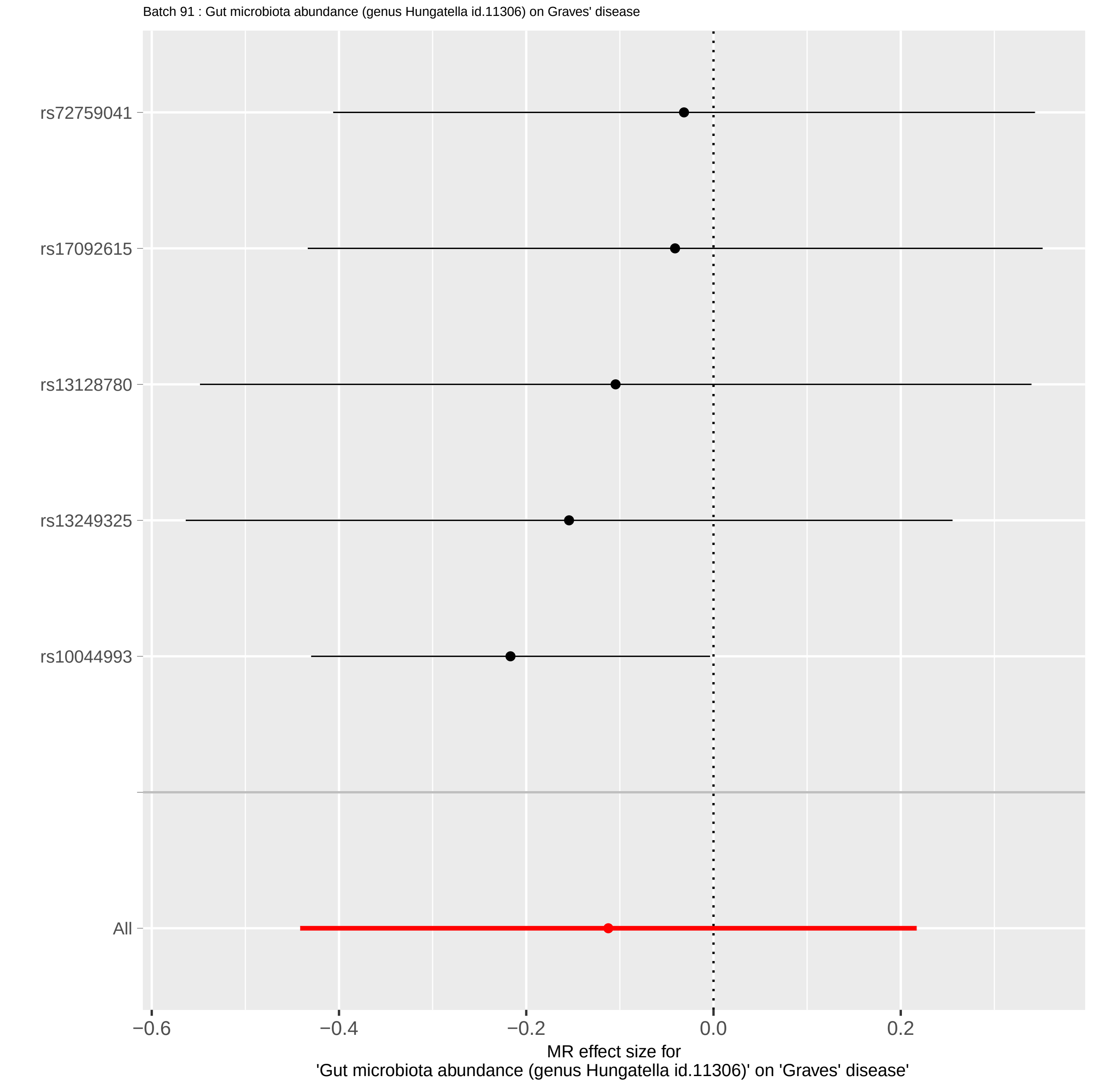
Batch 91 : Gut microbiota abundance (genus Hungatella id.11306) on Graves' disease
rs72759041
rs17092615
rs13128780
rs13249325
rs10044993
All
−0.6
−0.4
−0.2
0.0
0.2
MR effect size for
'Gut microbiota abundance (genus Hungatella id.11306)' on 'Graves' disease'

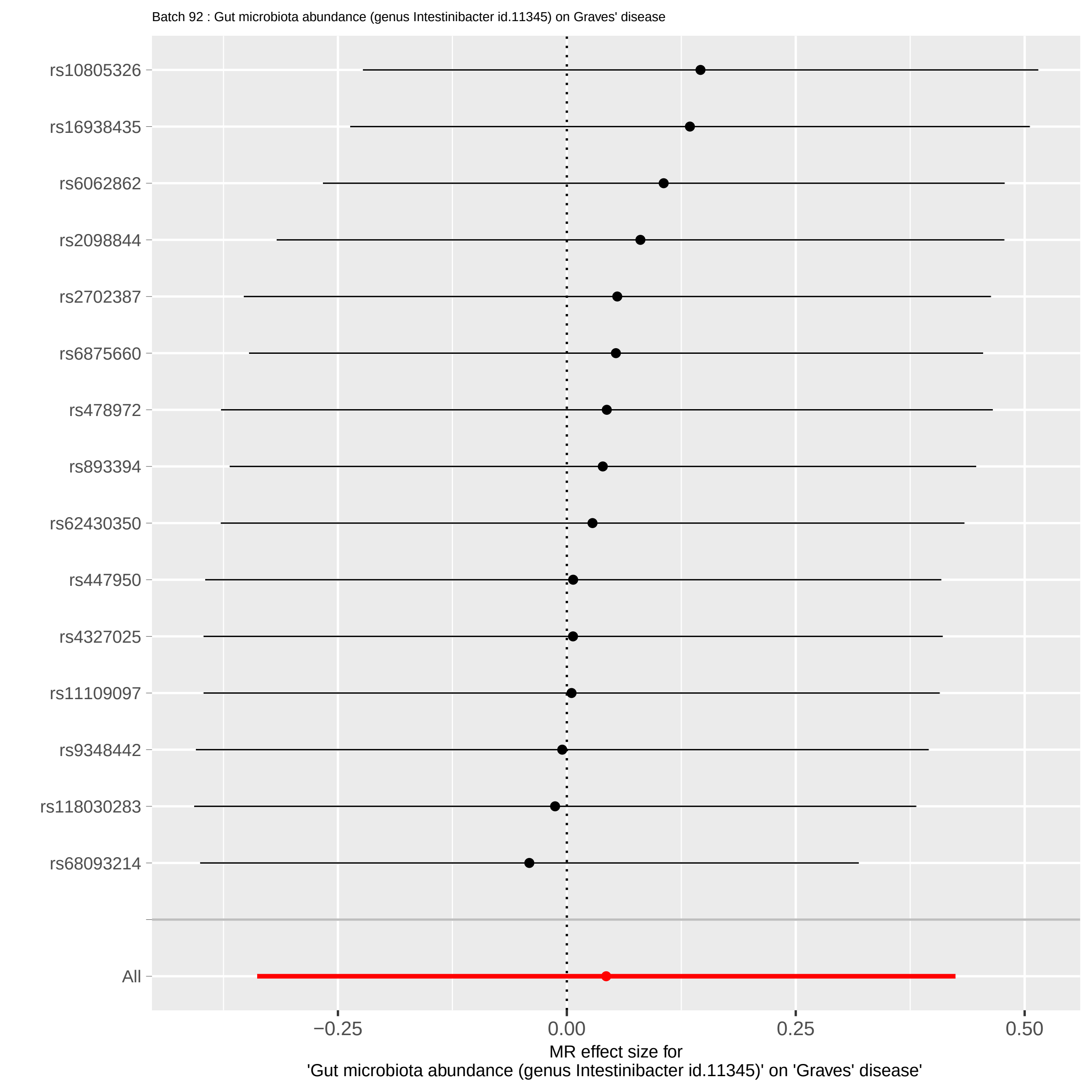
Batch 92 : Gut microbiota abundance (genus Intestinibacter id.11345) on Graves' disease
rs10805326
rs16938435
rs6062862
rs2098844
rs2702387
rs6875660
rs478972
rs893394
rs62430350
rs447950
rs4327025
rs11109097
rs9348442
rs118030283
rs68093214
All
−0.25
0.00
0.25
0.50
MR effect size for
'Gut microbiota abundance (genus Intestinibacter id.11345)' on 'Graves' disease'

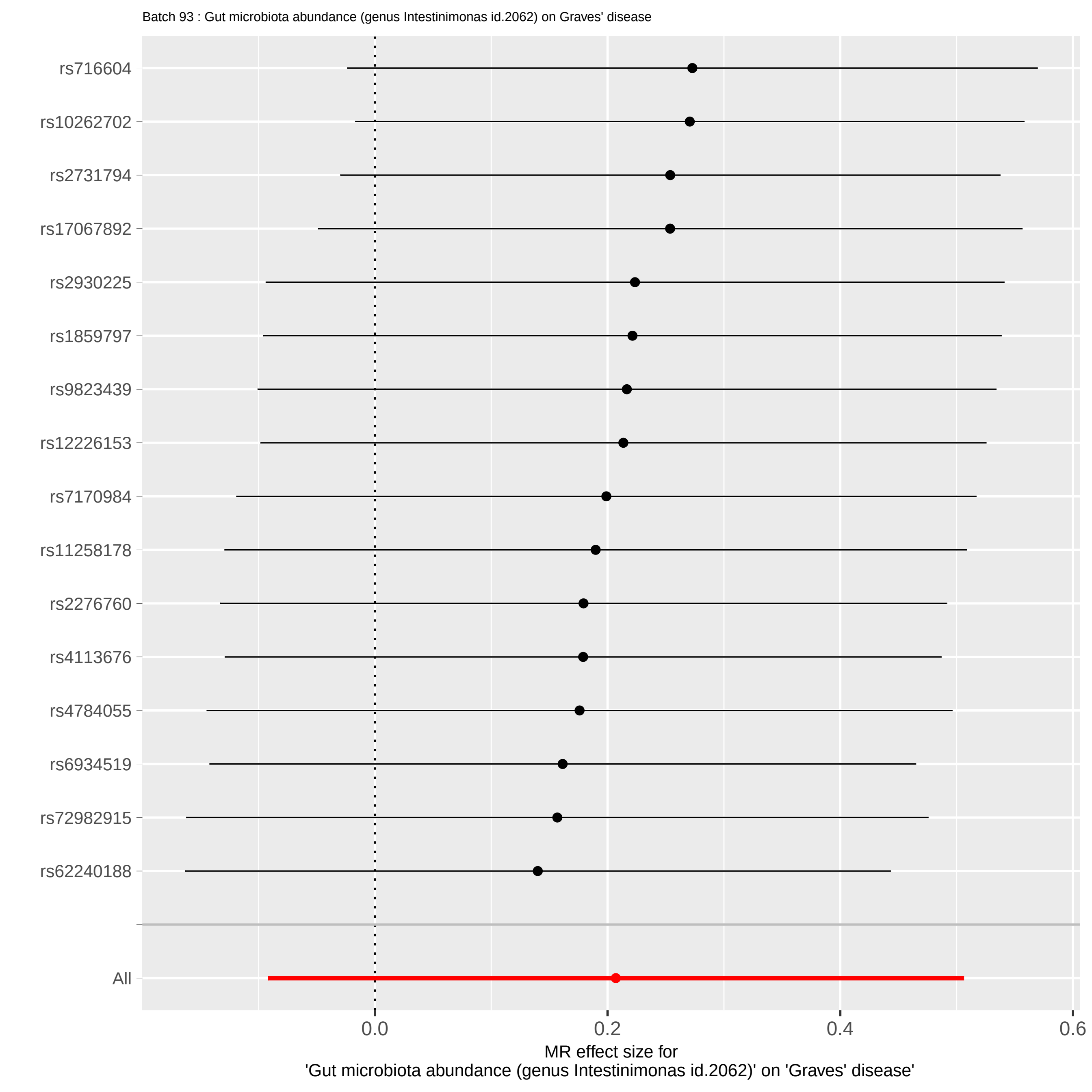
Batch 93 : Gut microbiota abundance (genus Intestinimonas id.2062) on Graves' disease
rs716604
rs10262702
rs2731794
rs17067892
rs2930225
rs1859797
rs9823439
rs12226153
rs7170984
rs11258178
rs2276760
rs4113676
rs4784055
rs6934519
rs72982915
rs62240188
All
0.0
0.2
0.4
0.6
MR effect size for
'Gut microbiota abundance (genus Intestinimonas id.2062)' on 'Graves' disease'

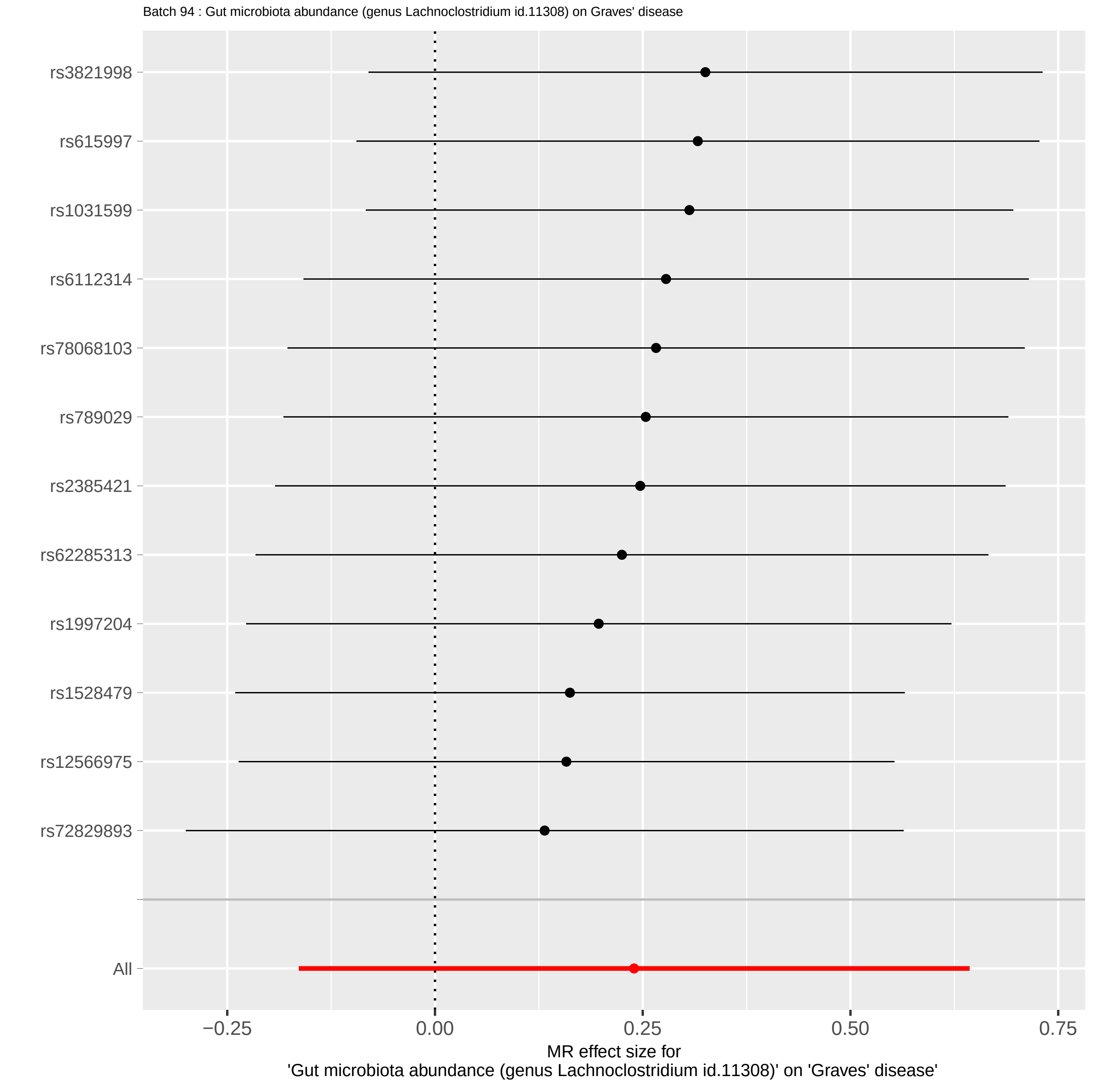

Batch 94 : Gut microbiota abundance (genus Lachnoclostridium id.11308) on Graves' disease
rs3821998
rs615997
rs1031599
rs6112314
rs78068103
rs789029
rs2385421
rs62285313
rs1997204
rs1528479
rs12566975
rs72829893
All
−0.25
0.00
0.25
0.50
0.75
MR effect size for
'Gut microbiota abundance (genus Lachnoclostridium id.11308)' on 'Graves' disease'

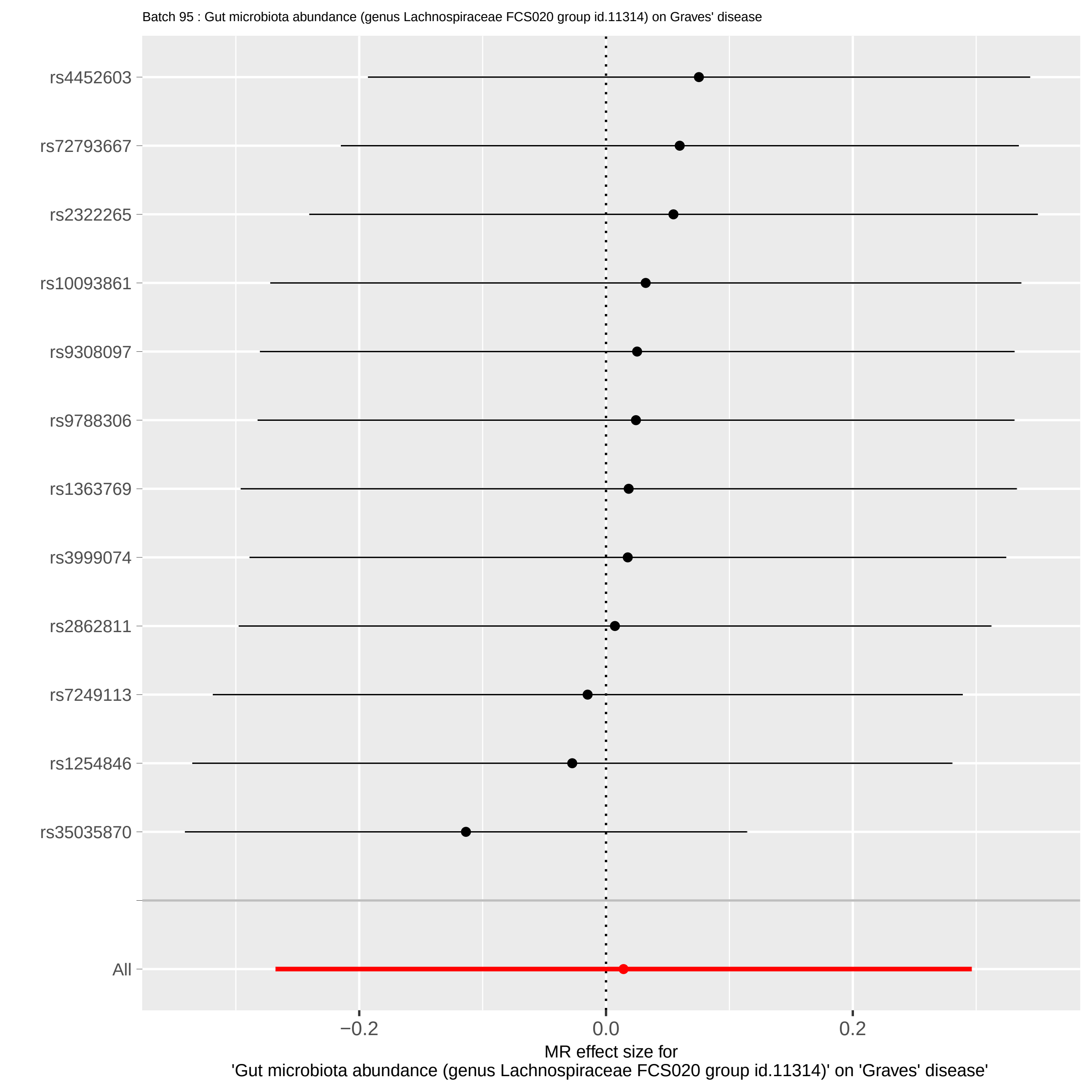

Batch 95 : Gut microbiota abundance (genus Lachnospiraceae FCS020 group id.11314) on Graves' disease
rs4452603
rs72793667
rs2322265
rs10093861
rs9308097
rs9788306
rs1363769
rs3999074
rs2862811
rs7249113
rs1254846
rs35035870
All
−0.2
0.0
0.2
MR effect size for
'Gut microbiota abundance (genus Lachnospiraceae FCS020 group id.11314)' on 'Graves' disease'

Batch 96 : Gut microbiota abundance (genus Lachnospiraceae NC2004 group id.11316) on Graves' disease

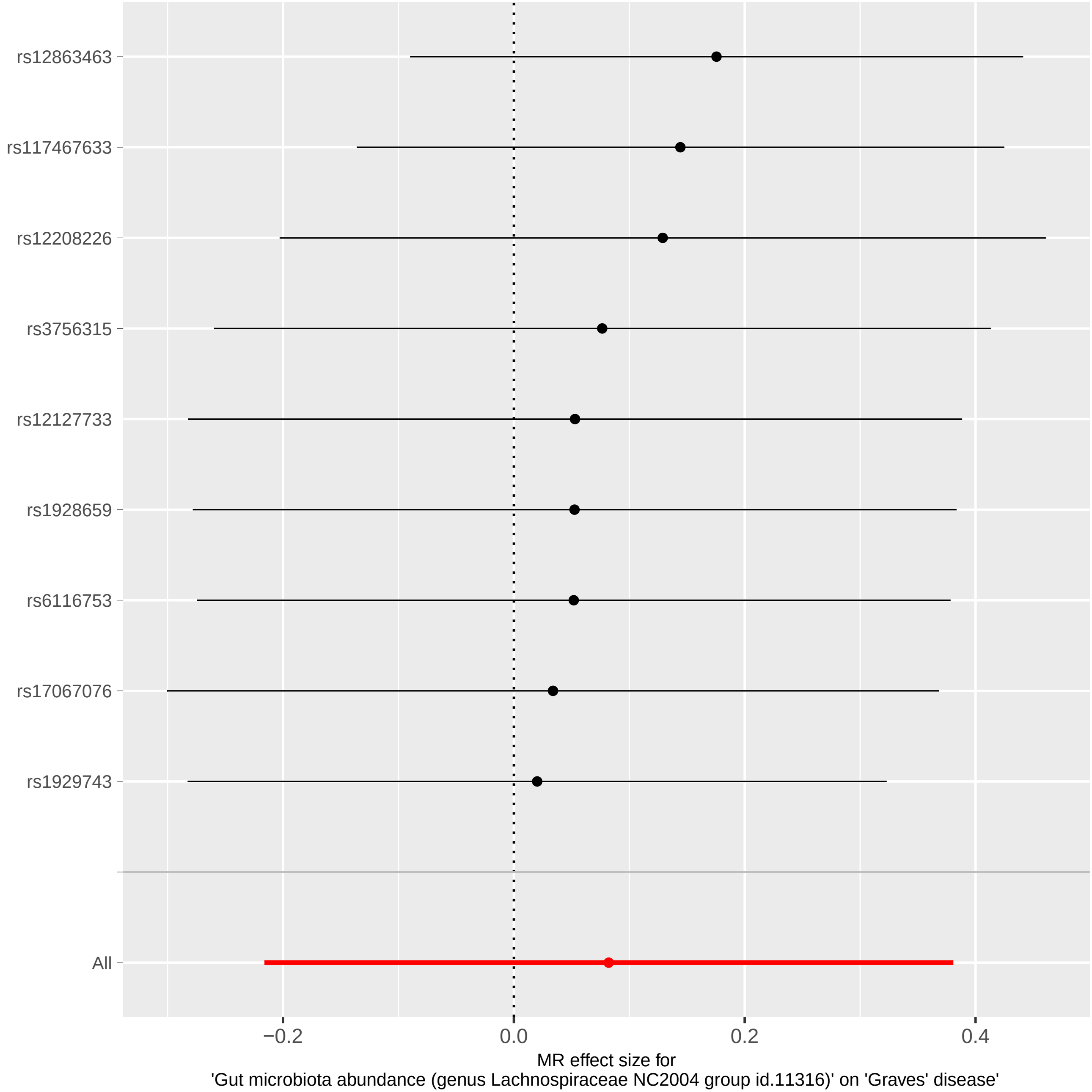

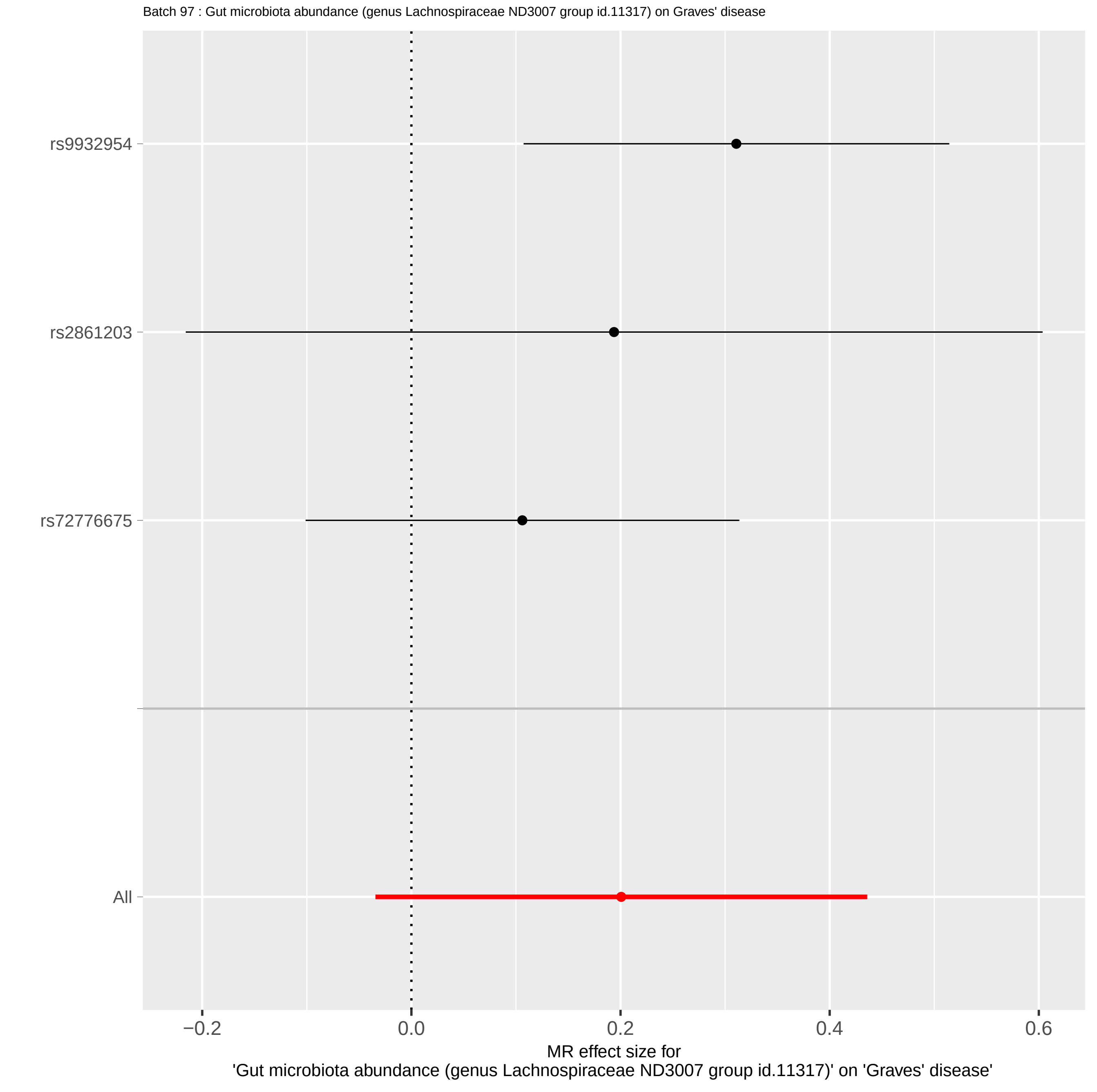

Batch 97 : Gut microbiota abundance (genus Lachnospiraceae ND3007 group id.11317) on Graves' disease
rs9932954
rs2861203
rs72776675
All
−0.2
0.0
0.2
0.4
0.6
MR effect size for
'Gut microbiota abundance (genus Lachnospiraceae ND3007 group id.11317)' on 'Graves' disease'

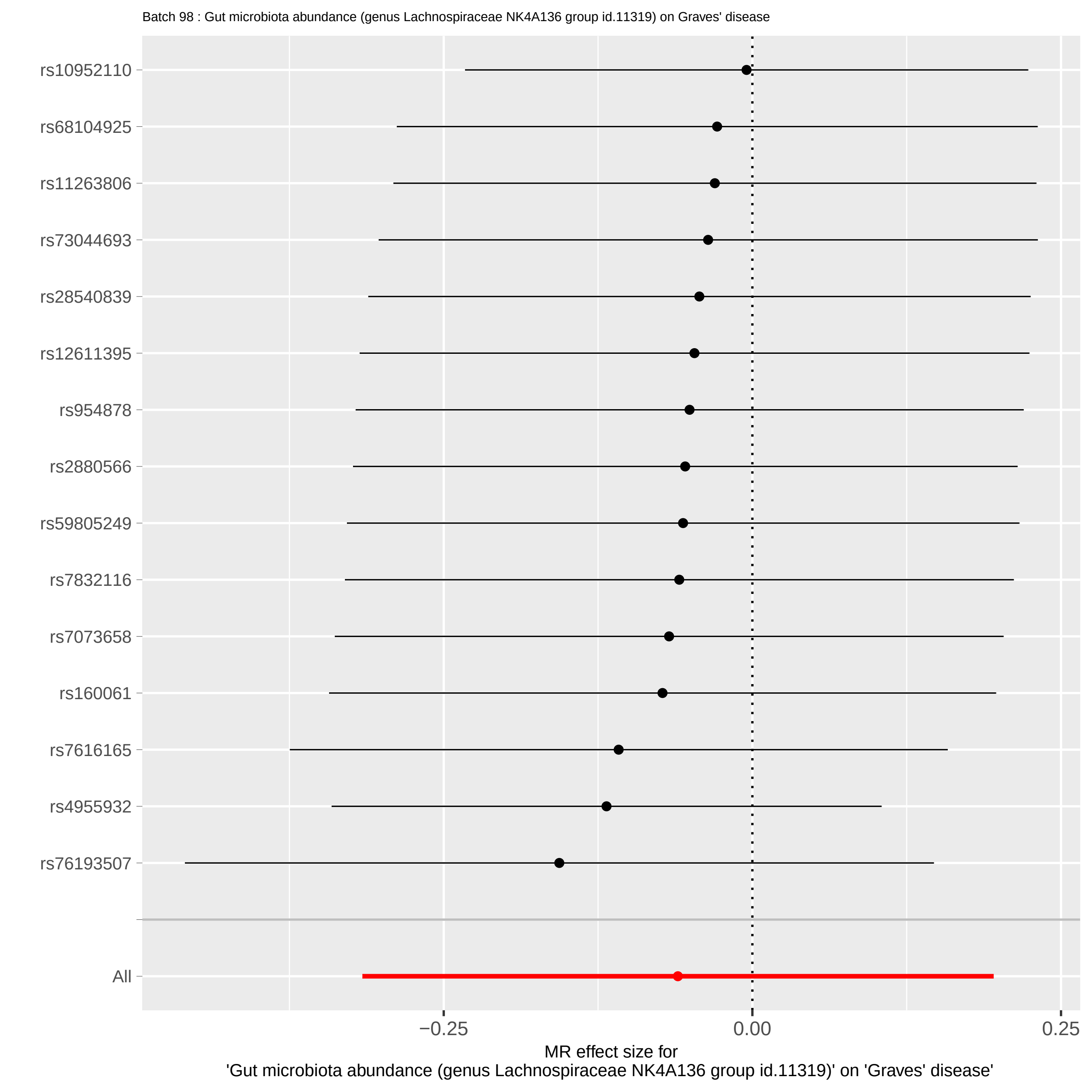

Batch 98 : Gut microbiota abundance (genus Lachnospiraceae NK4A136 group id.11319) on Graves' disease
rs10952110
rs68104925
rs11263806
rs73044693
rs28540839
rs12611395
rs954878
rs2880566
rs59805249
rs7832116
rs7073658
rs160061
rs7616165
rs4955932
rs76193507
All
−0.25
0.00
0.25
MR effect size for
'Gut microbiota abundance (genus Lachnospiraceae NK4A136 group id.11319)' on 'Graves' disease'

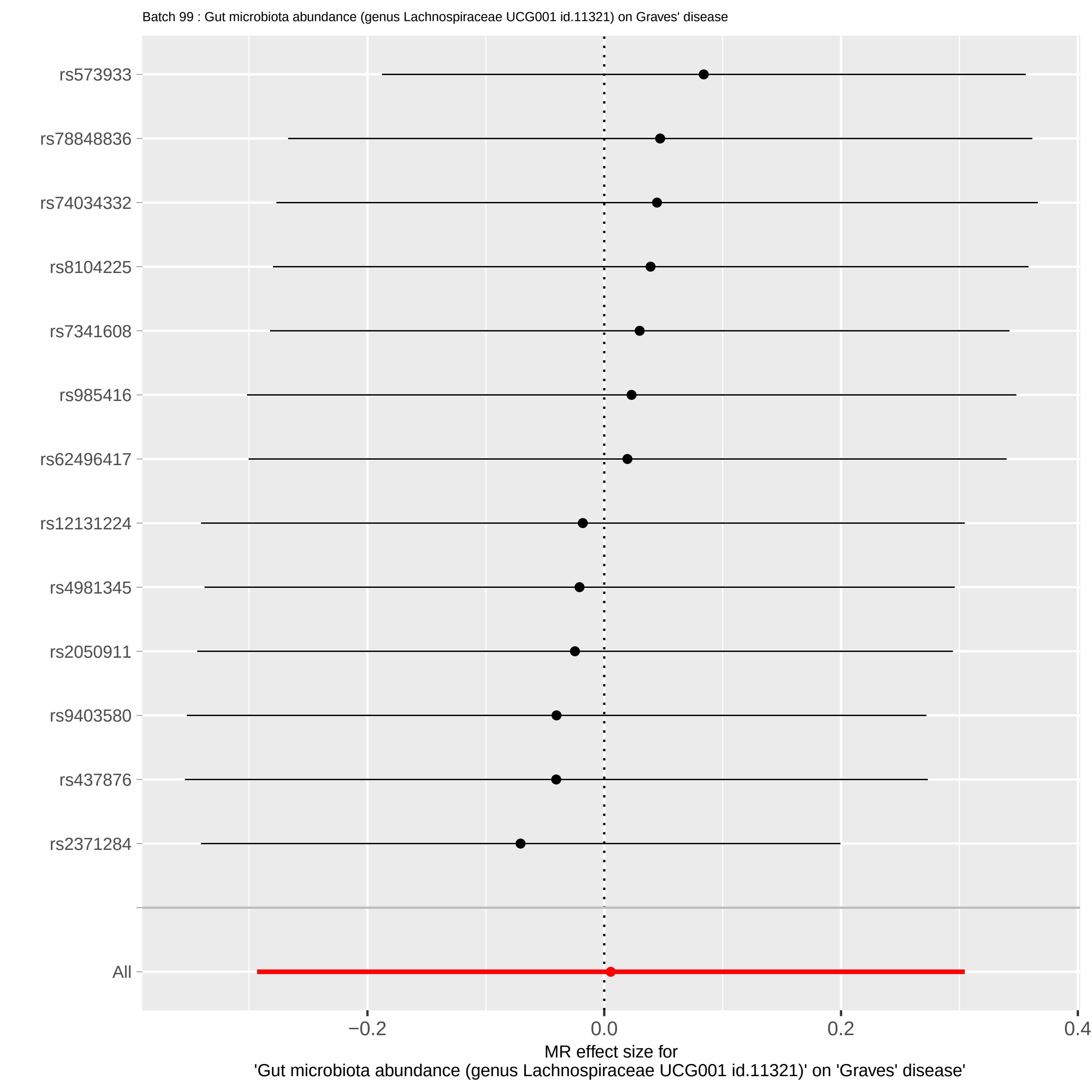

Batch 99 : Gut microbiota abundance (genus Lachnospiraceae UCG001 id.11321) on Graves' disease
rs573933
rs78848836
rs74034332
rs8104225
rs7341608
rs985416
rs62496417
rs12131224
rs4981345
rs2050911
rs9403580
rs437876
rs2371284
All
−0.2
0.0
0.2
0.4
MR effect size for
'Gut microbiota abundance (genus Lachnospiraceae UCG001 id.11321)' on 'Graves' disease'

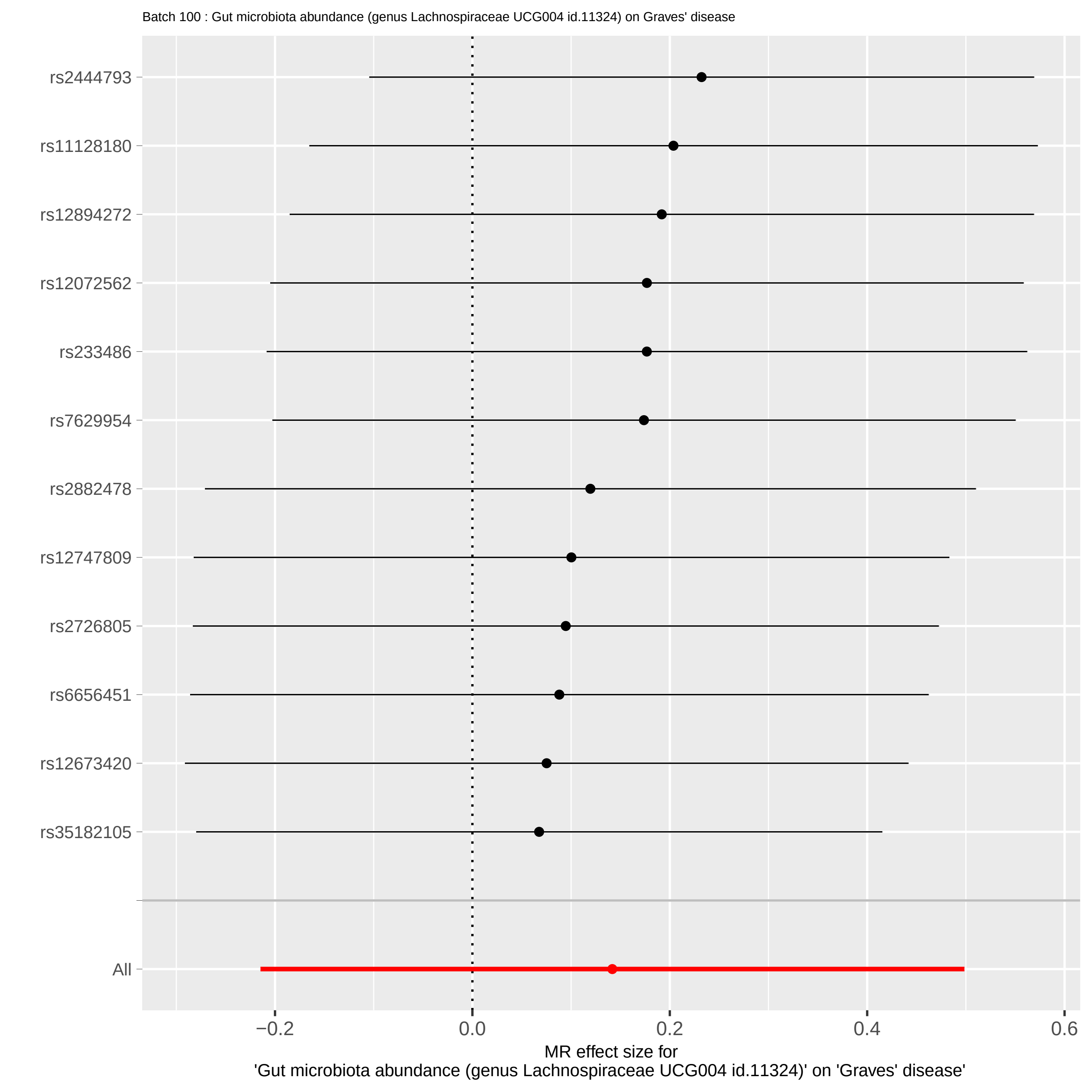

Batch 100 : Gut microbiota abundance (genus Lachnospiraceae UCG004 id.11324) on Graves' disease
rs2444793
rs11128180
rs12894272
rs12072562
rs233486
rs7629954
rs2882478
rs12747809
rs2726805
rs6656451
rs12673420
rs35182105
All
−0.2
0.0
0.2
0.4
0.6
MR effect size for
'Gut microbiota abundance (genus Lachnospiraceae UCG004 id.11324)' on 'Graves' disease'

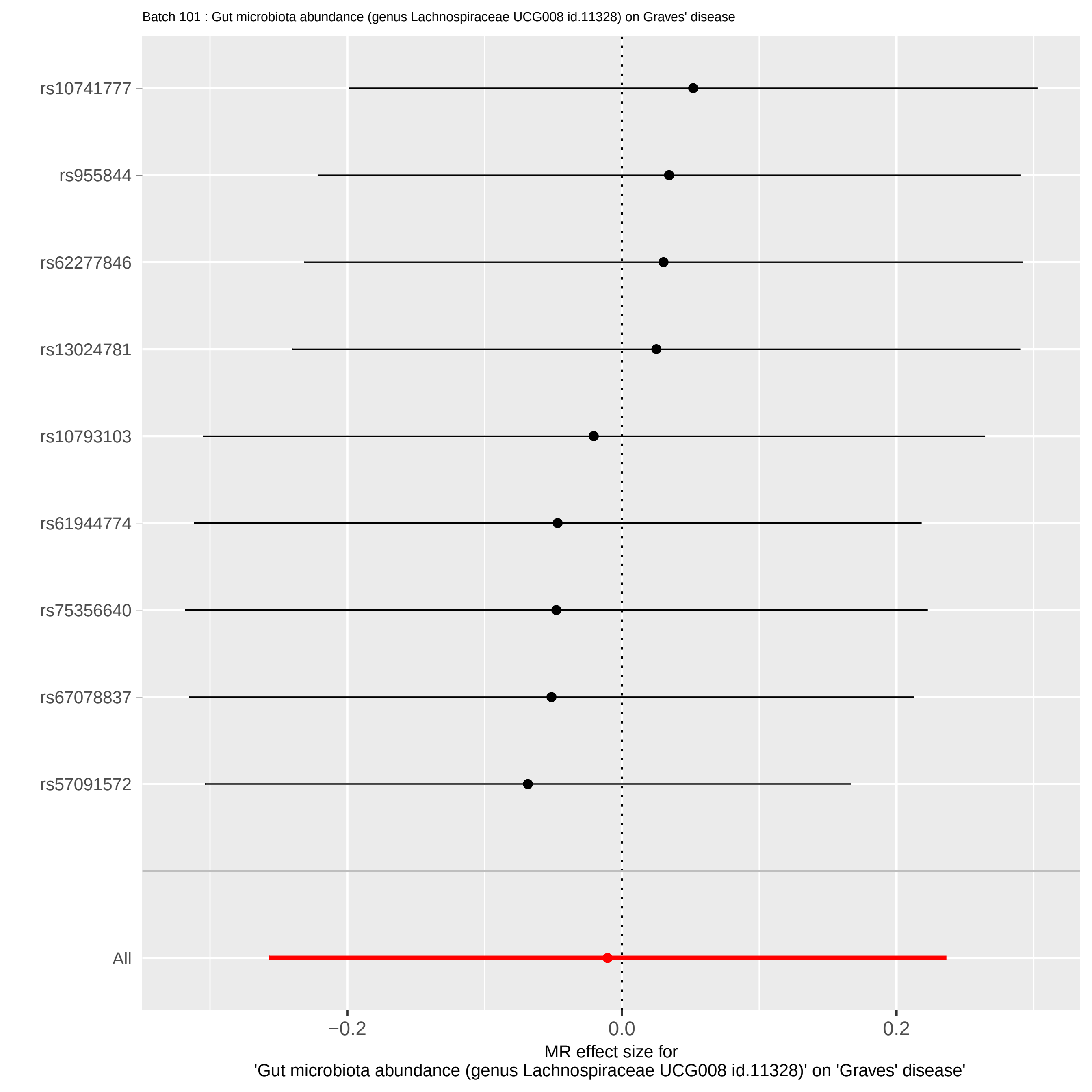

Batch 101 : Gut microbiota abundance (genus Lachnospiraceae UCG008 id.11328) on Graves' disease
rs10741777
rs955844
rs62277846
rs13024781
rs10793103
rs61944774
rs75356640
rs67078837
rs57091572
All
−0.2
0.0
0.2
MR effect size for
'Gut microbiota abundance (genus Lachnospiraceae UCG008 id.11328)' on 'Graves' disease'

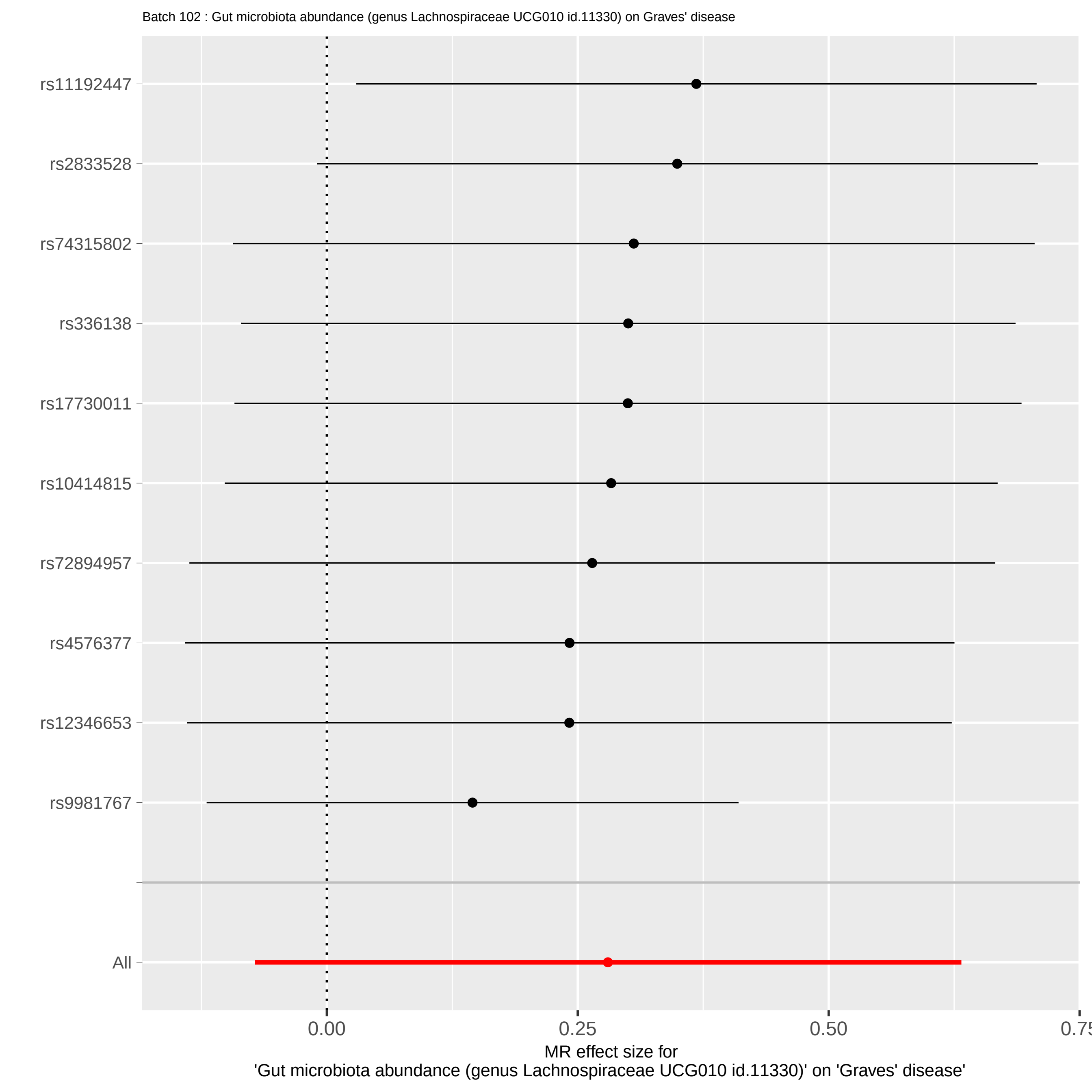

Batch 102 : Gut microbiota abundance (genus Lachnospiraceae UCG010 id.11330) on Graves' disease
rs11192447
rs2833528
rs74315802
rs336138
rs17730011
rs10414815
rs72894957
rs4576377
rs12346653
rs9981767
All
0.00
0.25
0.50
0.75
MR effect size for
'Gut microbiota abundance (genus Lachnospiraceae UCG010 id.11330)' on 'Graves' disease'

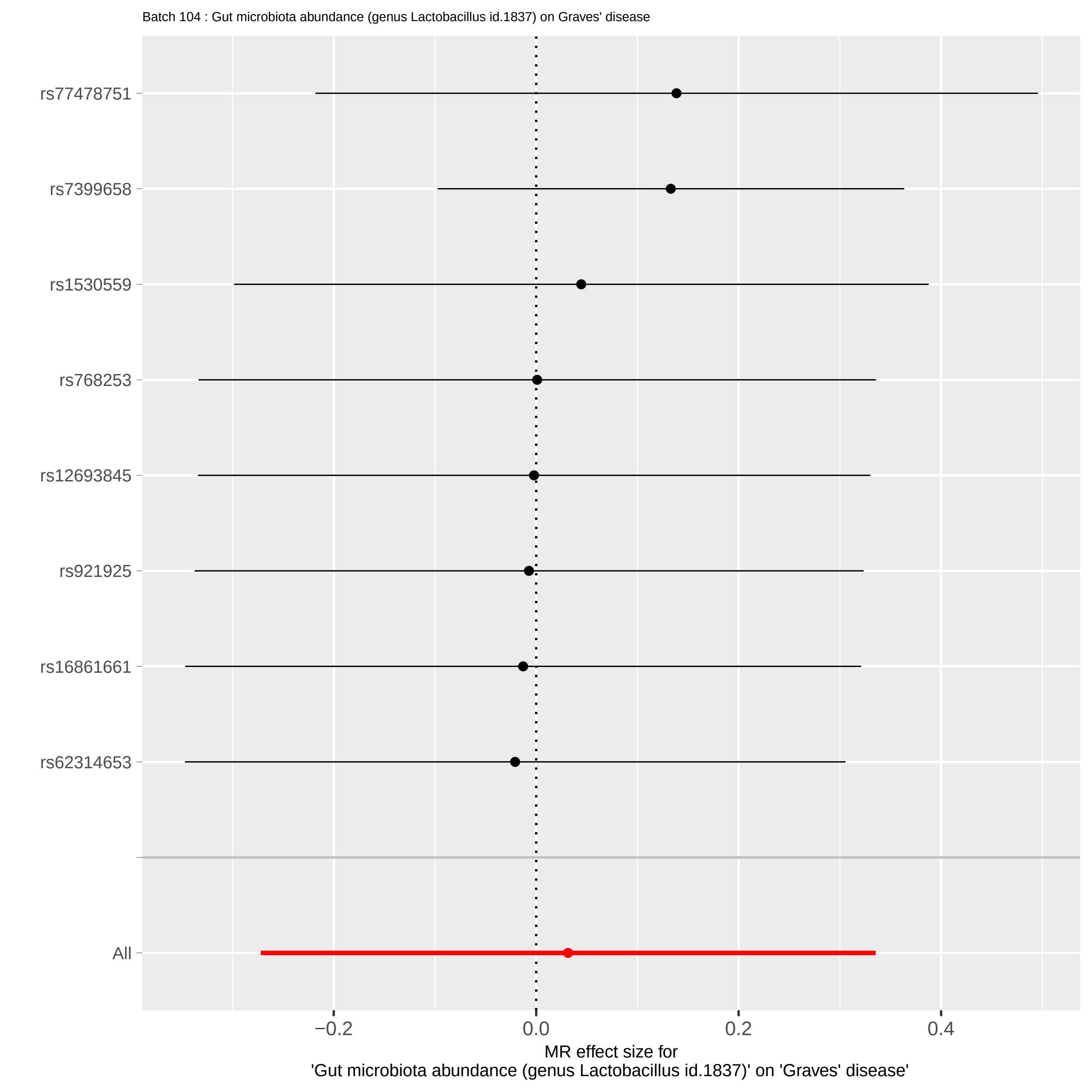
Batch 104 : Gut microbiota abundance (genus Lactobacillus id.1837) on Graves' disease
rs77478751
rs7399658
rs1530559
rs768253
rs12693845
rs921925
rs16861661
rs62314653
All
−0.2
0.0
0.2
0.4
MR effect size for
'Gut microbiota abundance (genus Lactobacillus id.1837)' on 'Graves' disease'

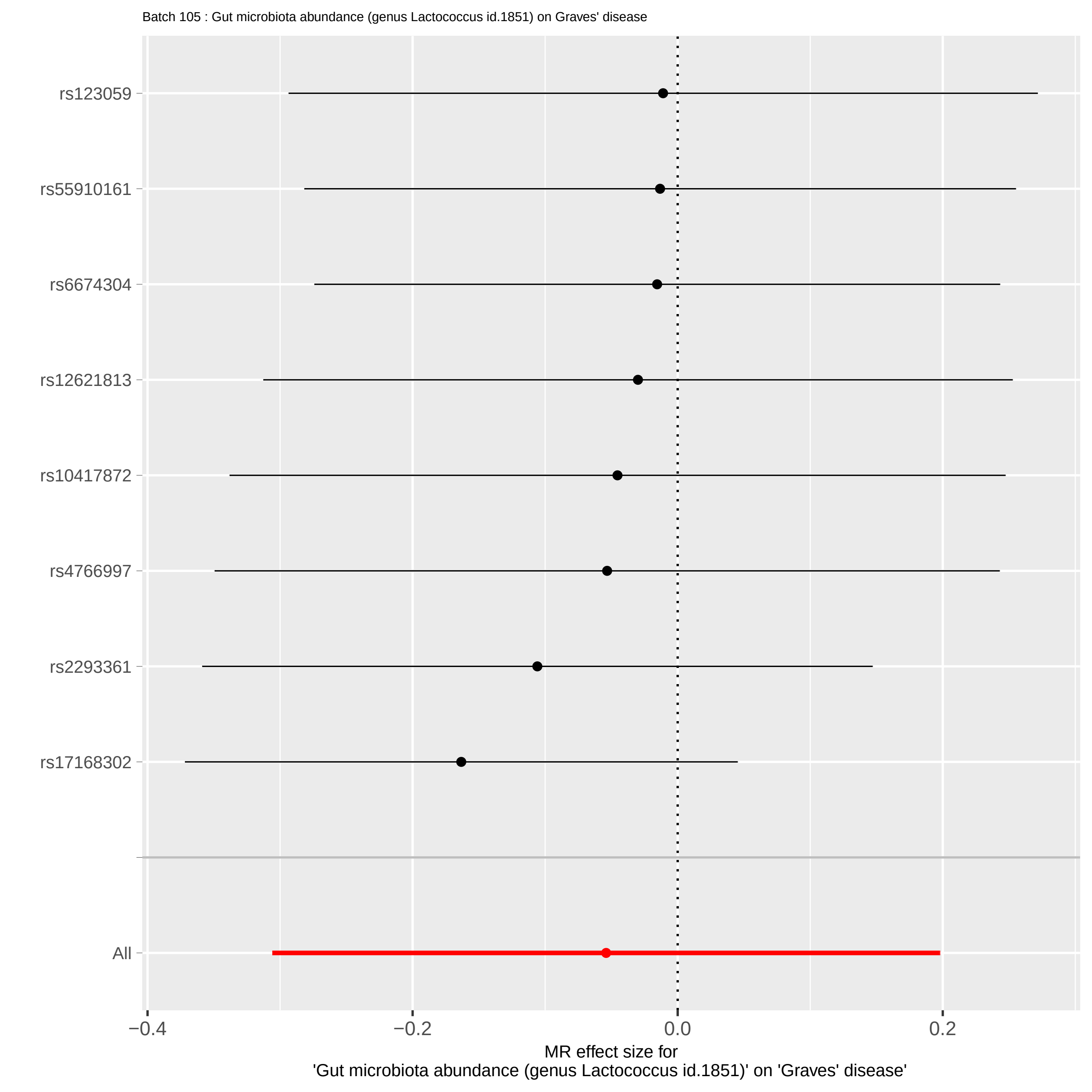

Batch 105 : Gut microbiota abundance (genus Lactococcus id.1851) on Graves' disease
rs123059
rs55910161
rs6674304
rs12621813
rs10417872
rs4766997
rs2293361
rs17168302
All
−0.4
−0.2
0.0
0.2
MR effect size for
'Gut microbiota abundance (genus Lactococcus id.1851)' on 'Graves' disease'

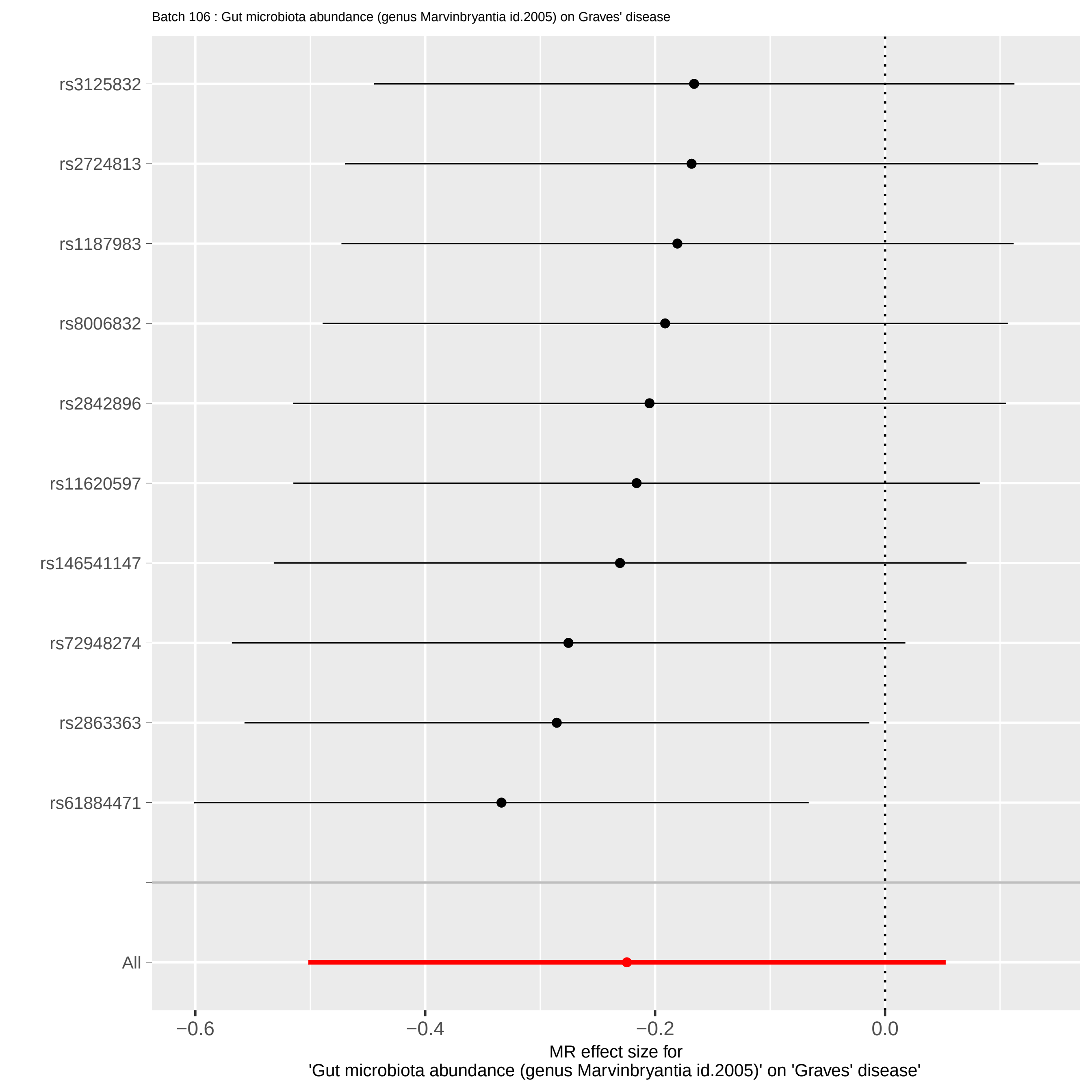

Batch 106 : Gut microbiota abundance (genus Marvinbryantia id.2005) on Graves' disease
rs3125832
rs2724813
rs1187983
rs8006832
rs2842896
rs11620597
rs146541147
rs72948274
rs2863363
rs61884471
All
−0.6
−0.4
−0.2
0.0
MR effect size for
'Gut microbiota abundance (genus Marvinbryantia id.2005)' on 'Graves' disease'

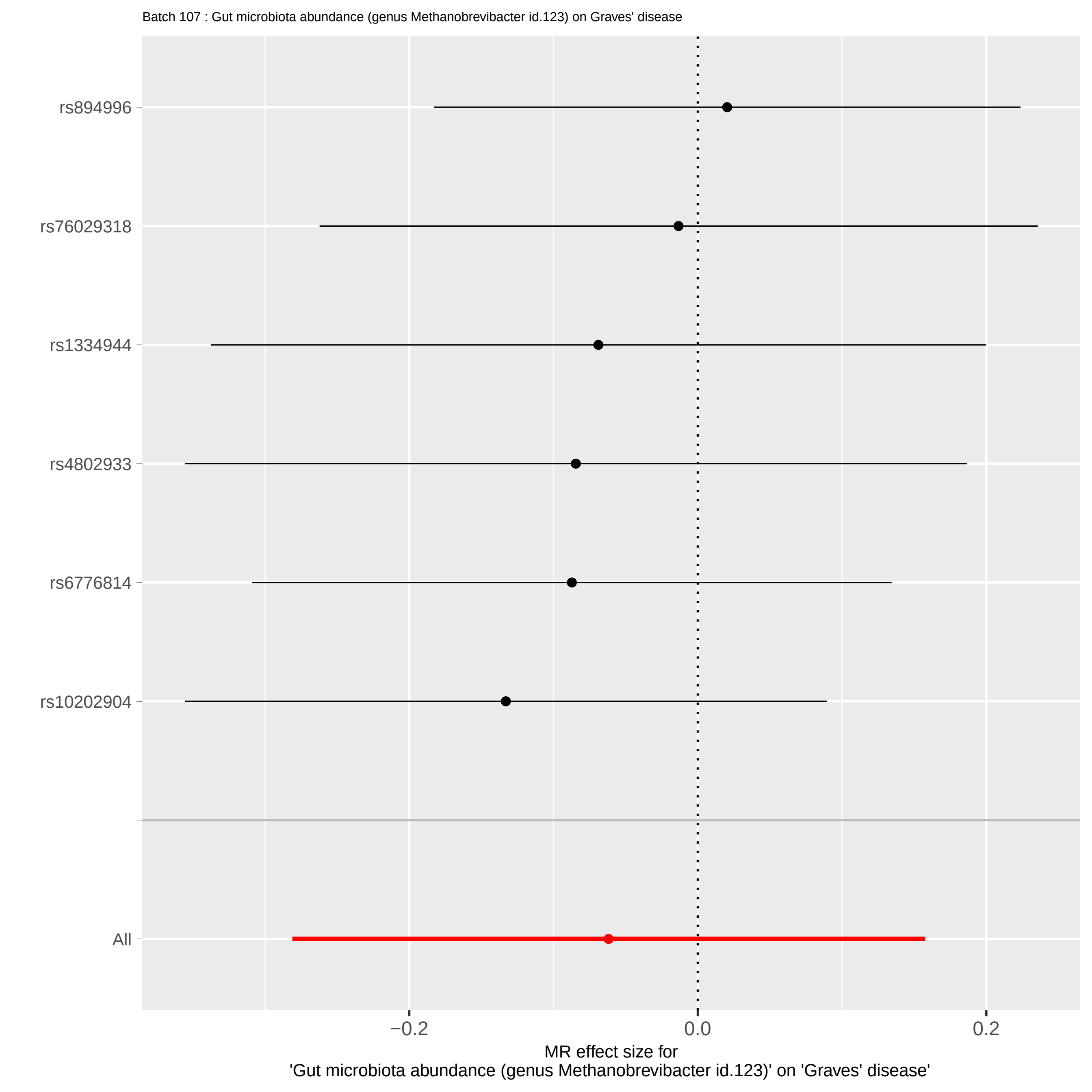
Batch 107 : Gut microbiota abundance (genus Methanobrevibacter id.123) on Graves' disease
rs894996
rs76029318
rs1334944
rs4802933
rs6776814
rs10202904
All
−0.2
0.0
0.2
MR effect size for
'Gut microbiota abundance (genus Methanobrevibacter id.123)' on 'Graves' disease'

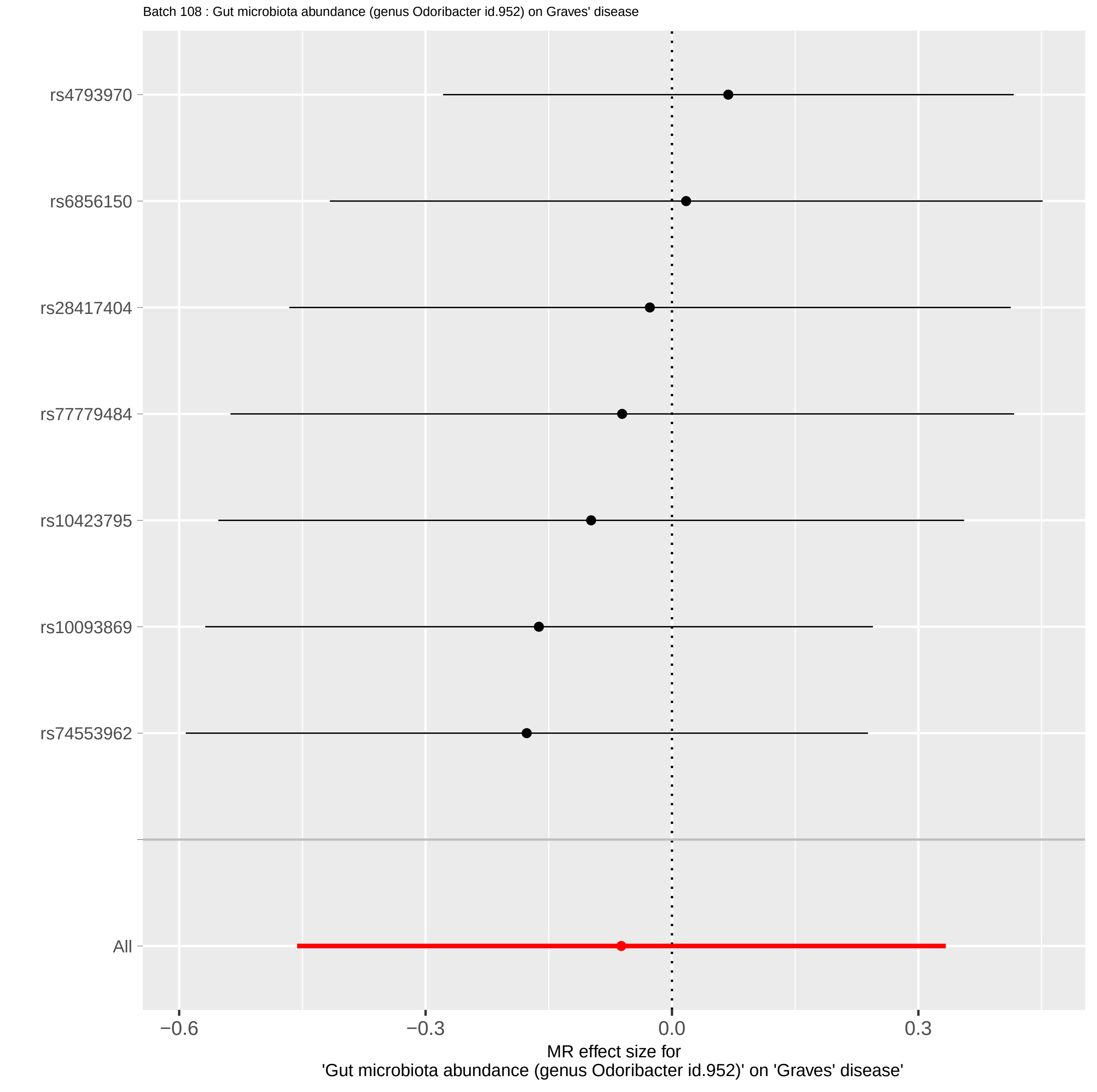

Batch 108 : Gut microbiota abundance (genus Odoribacter id.952) on Graves' disease
rs4793970
rs6856150
rs28417404
rs77779484
rs10423795
rs10093869
rs74553962
All
−0.6
−0.3
0.0
0.3
MR effect size for
'Gut microbiota abundance (genus Odoribacter id.952)' on 'Graves' disease'

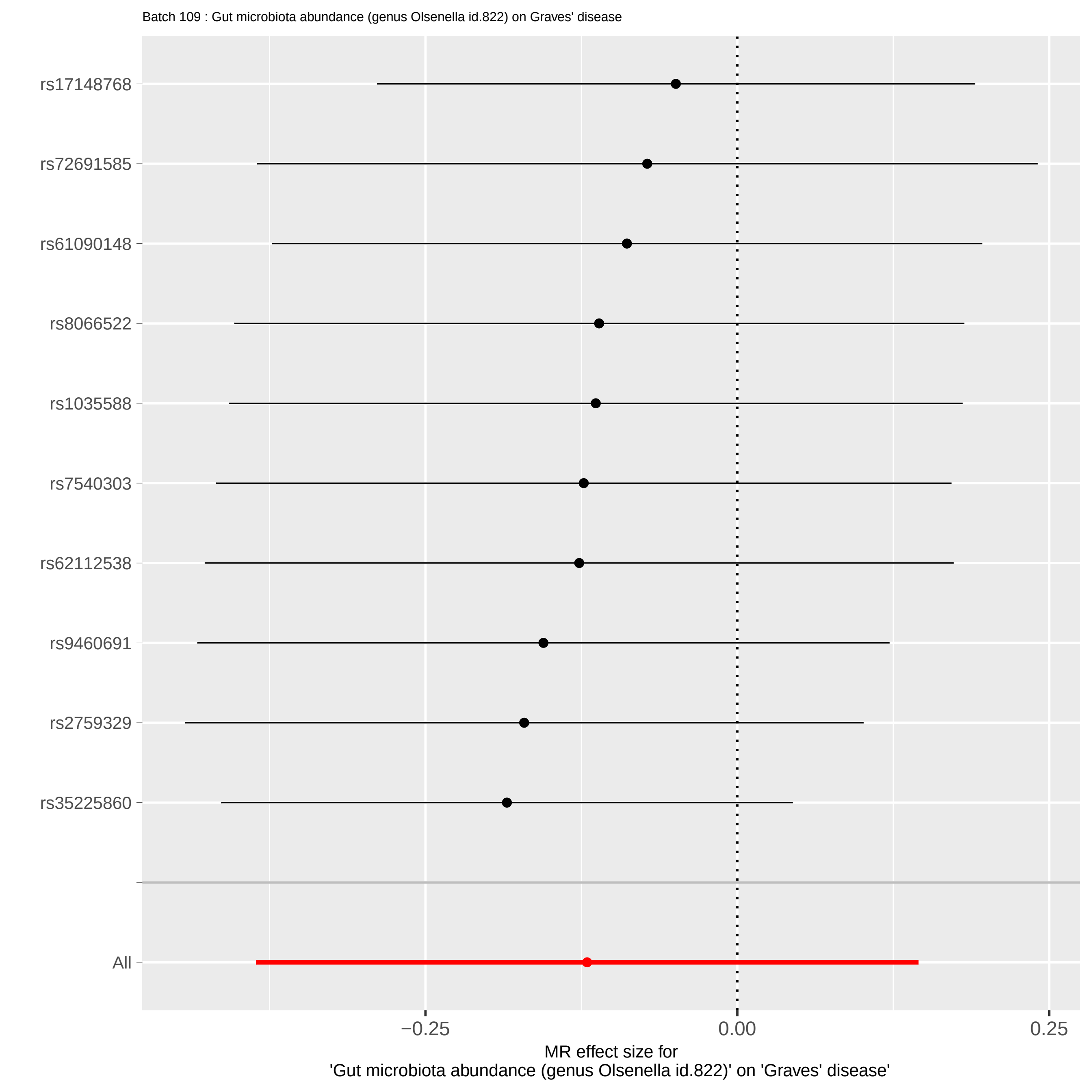

Batch 109 : Gut microbiota abundance (genus Olsenella id.822) on Graves' disease
rs17148768
rs72691585
rs61090148
rs8066522
rs1035588
rs7540303
rs62112538
rs9460691
rs2759329
rs35225860
All
−0.25
0.00
0.25
MR effect size for
'Gut microbiota abundance (genus Olsenella id.822)' on 'Graves' disease'

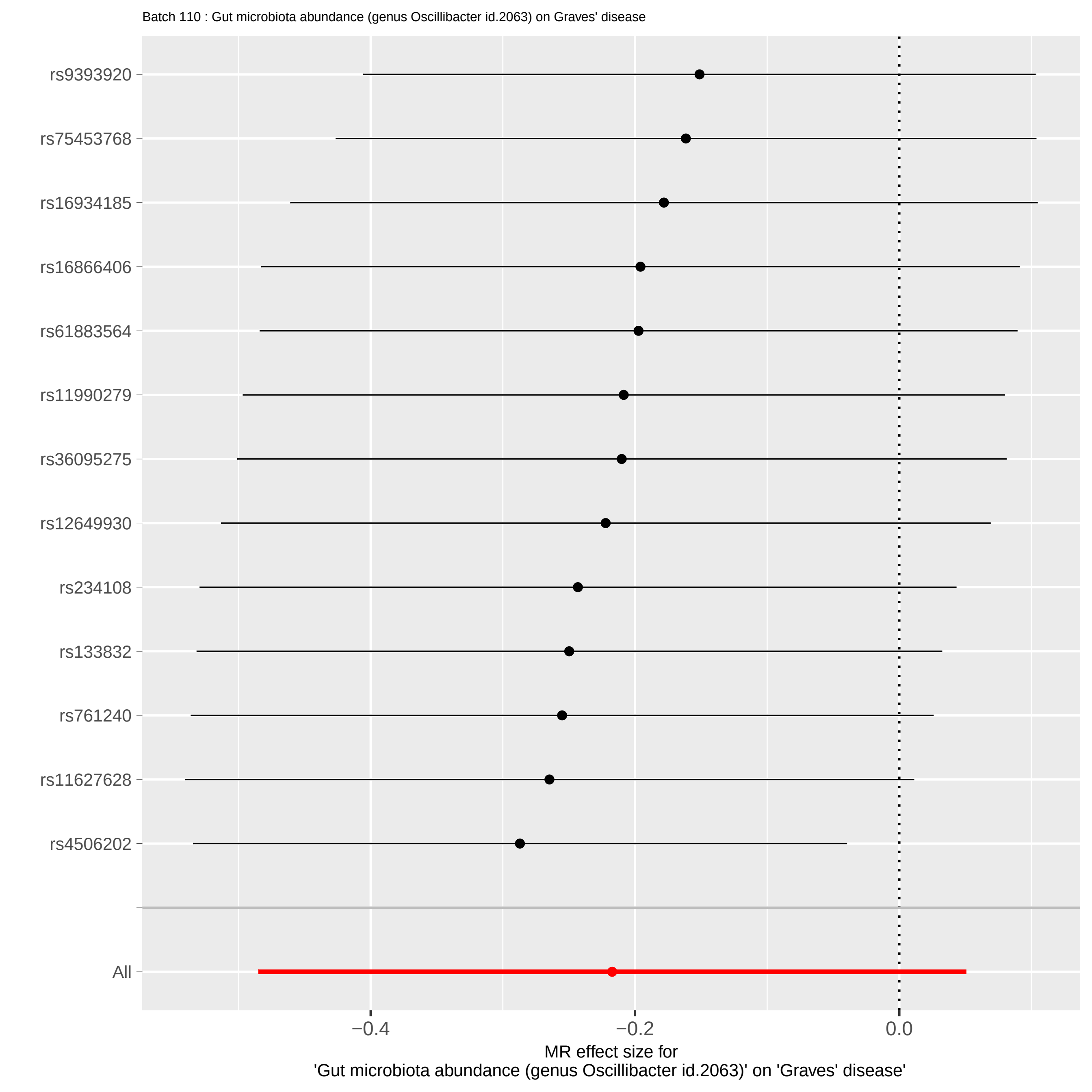

Batch 110 : Gut microbiota abundance (genus Oscillibacter id.2063) on Graves' disease
rs9393920
rs75453768
rs16934185
rs16866406
rs61883564
rs11990279
rs36095275
rs12649930
rs234108
rs133832
rs761240
rs11627628
rs4506202
All
−0.4
−0.2
0.0
MR effect size for
'Gut microbiota abundance (genus Oscillibacter id.2063)' on 'Graves' disease'

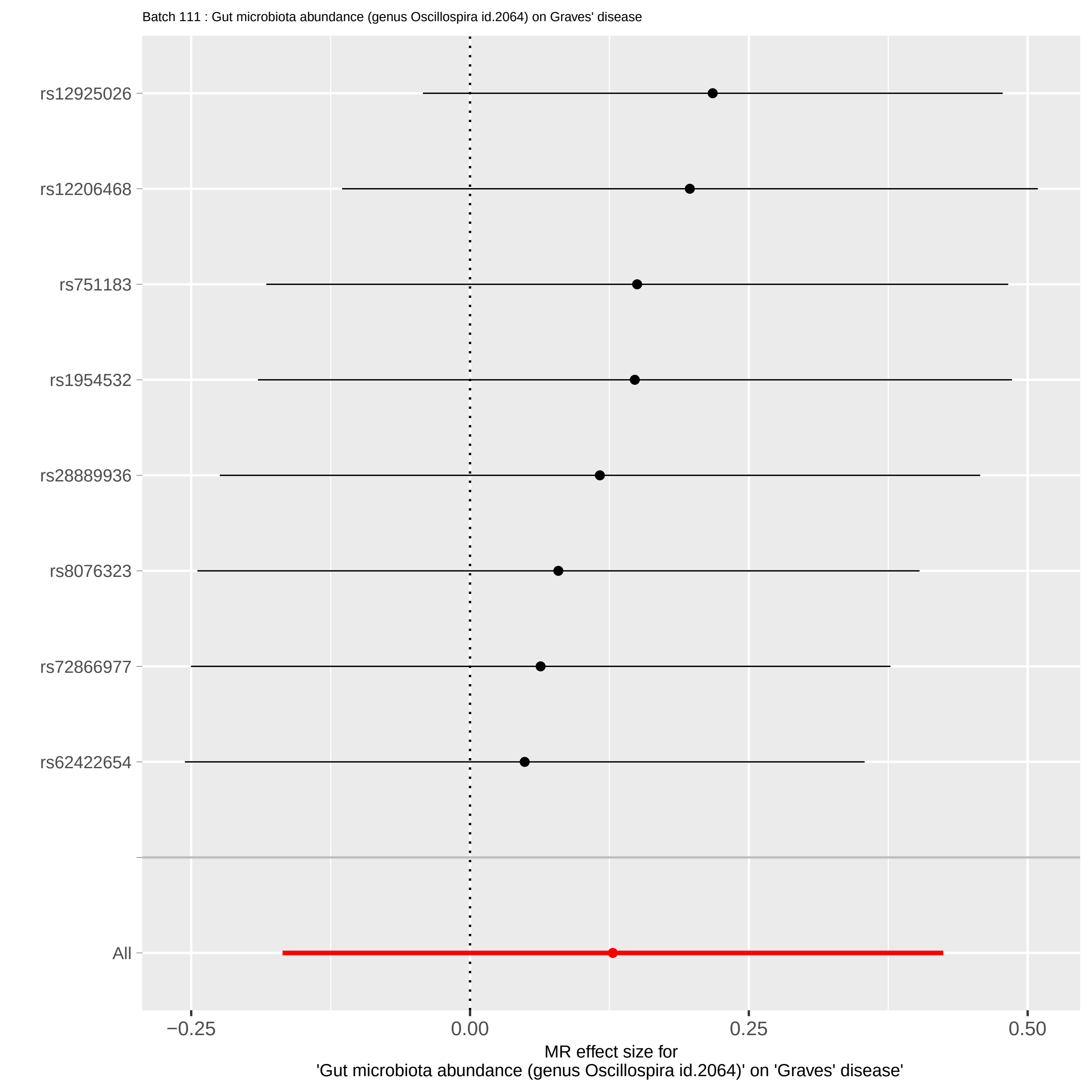

Batch 111 : Gut microbiota abundance (genus Oscillospira id.2064) on Graves' disease
rs12925026
rs12206468
rs751183
rs1954532
rs28889936
rs8076323
rs72866977
rs62422654
All
−0.25
0.00
0.25
0.50
MR effect size for
'Gut microbiota abundance (genus Oscillospira id.2064)' on 'Graves' disease'

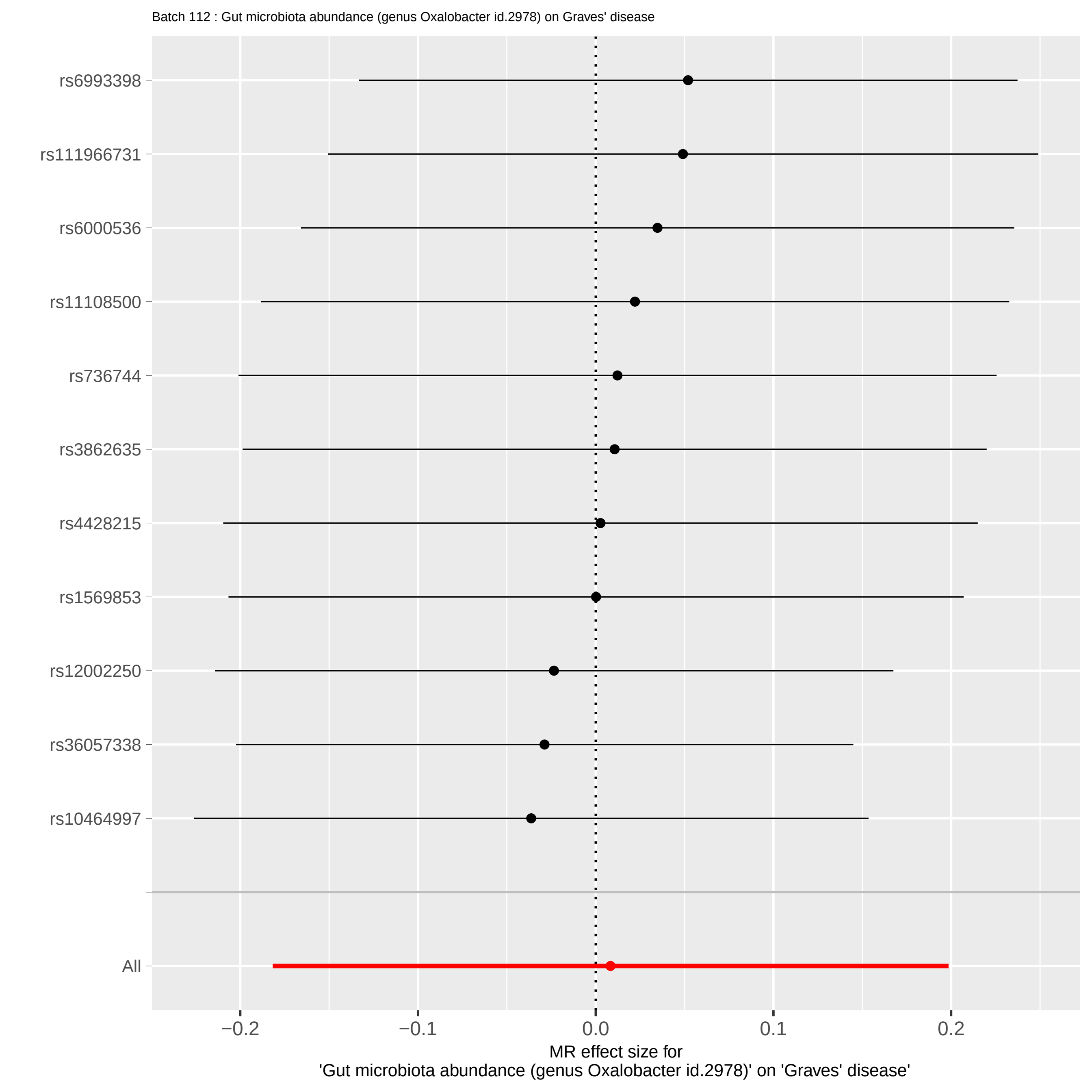

Batch 112 : Gut microbiota abundance (genus Oxalobacter id.2978) on Graves' disease
rs6993398
rs111966731
rs6000536
rs11108500
rs736744
rs3862635
rs4428215
rs1569853
rs12002250
rs36057338
rs10464997
All
−0.2
−0.1
0.0
0.1
0.2
MR effect size for
'Gut microbiota abundance (genus Oxalobacter id.2978)' on 'Graves' disease'

Batch 113 : Gut microbiota abundance (genus Parabacteroides id.954) on Graves' disease

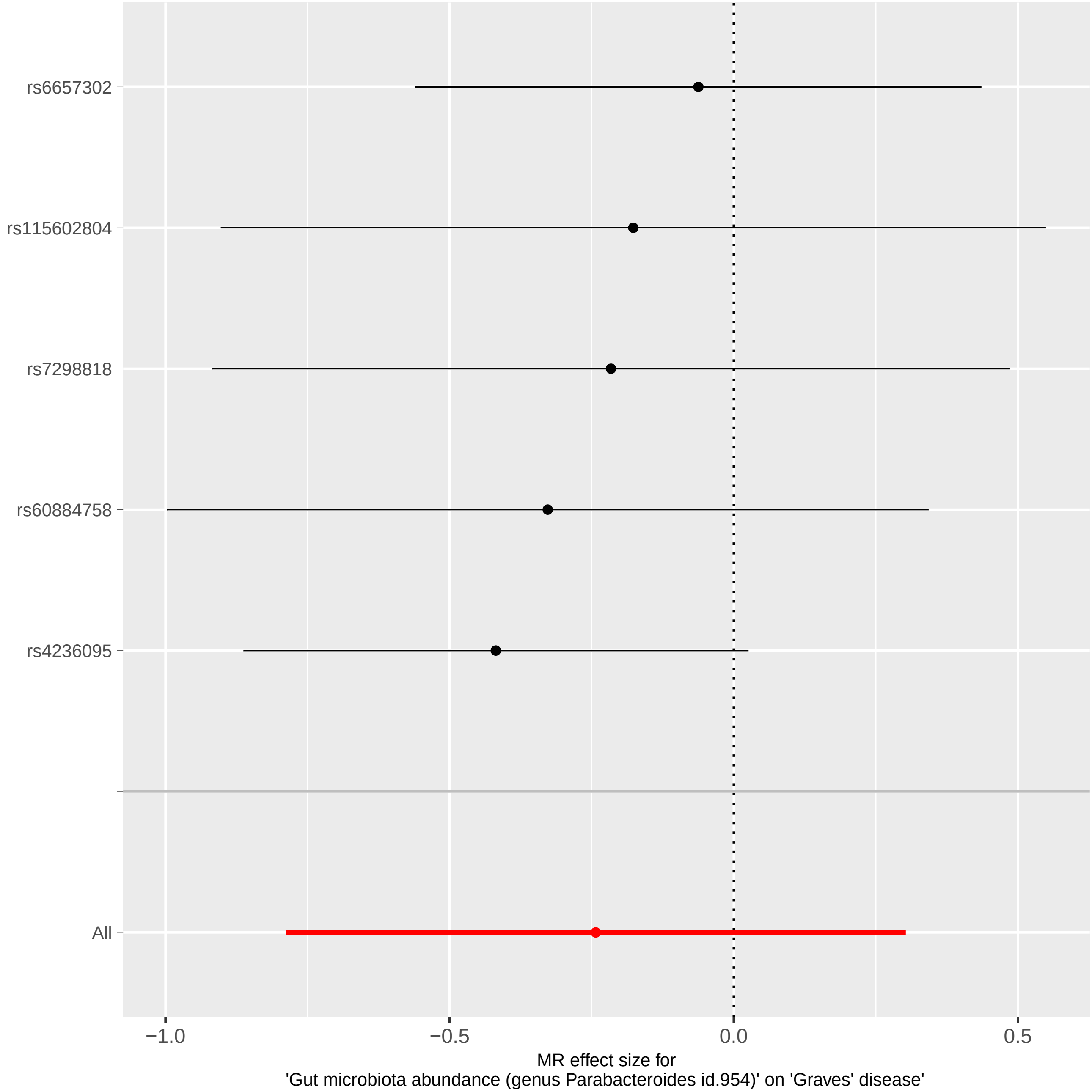

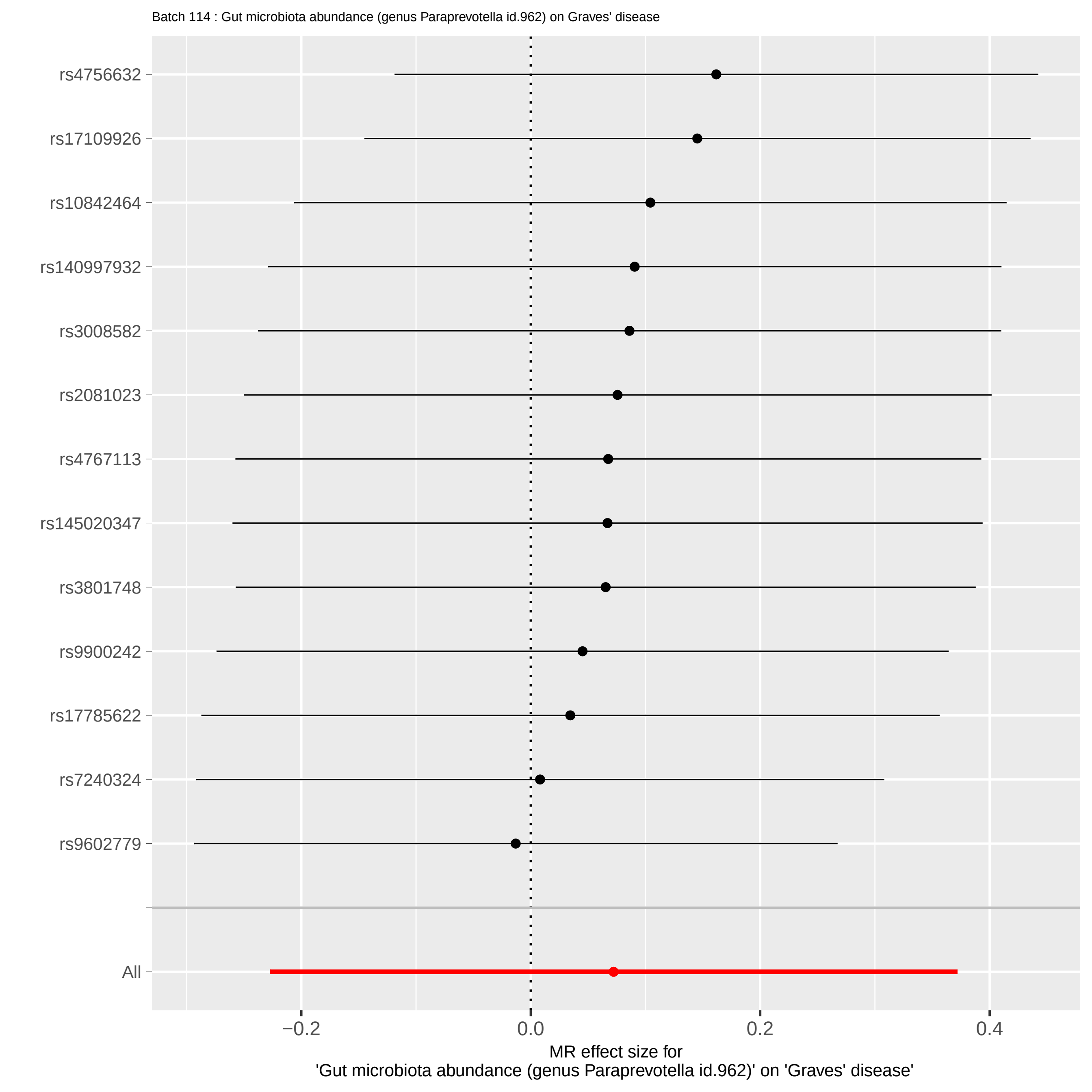

Batch 114 : Gut microbiota abundance (genus Paraprevotella id.962) on Graves' disease
rs4756632
rs17109926
rs10842464
rs140997932
rs3008582
rs2081023
rs4767113
rs145020347
rs3801748
rs9900242
rs17785622
rs7240324
rs9602779
All
−0.2
0.0
0.2
0.4
MR effect size for
'Gut microbiota abundance (genus Paraprevotella id.962)' on 'Graves' disease'

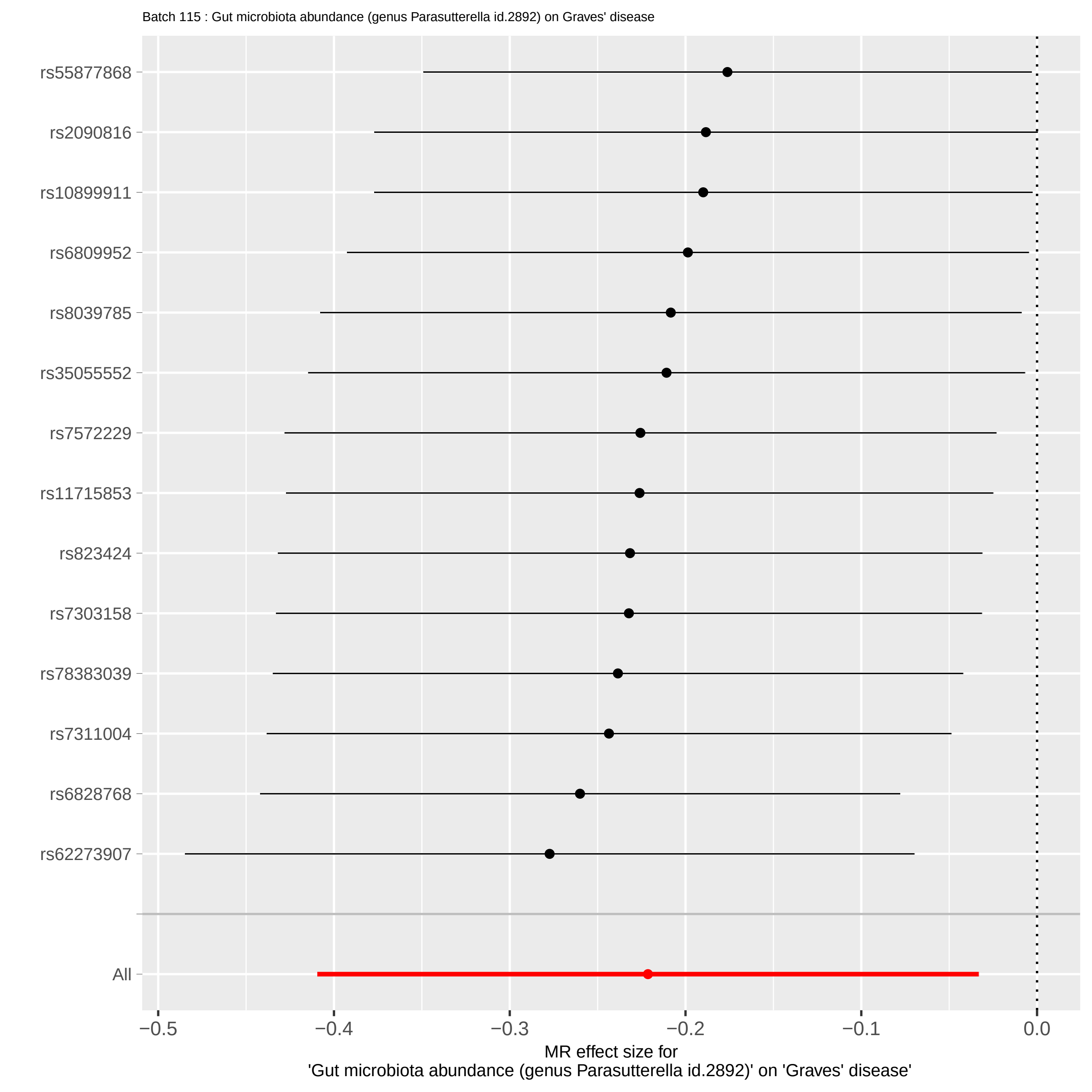

Batch 115 : Gut microbiota abundance (genus Parasutterella id.2892) on Graves' disease
rs55877868
rs2090816
rs10899911
rs6809952
rs8039785
rs35055552
rs7572229
rs11715853
rs823424
rs7303158
rs78383039
rs7311004
rs6828768
rs62273907
All
−0.5
−0.4
−0.3
−0.2
−0.1
0.0
MR effect size for
'Gut microbiota abundance (genus Parasutterella id.2892)' on 'Graves' disease'

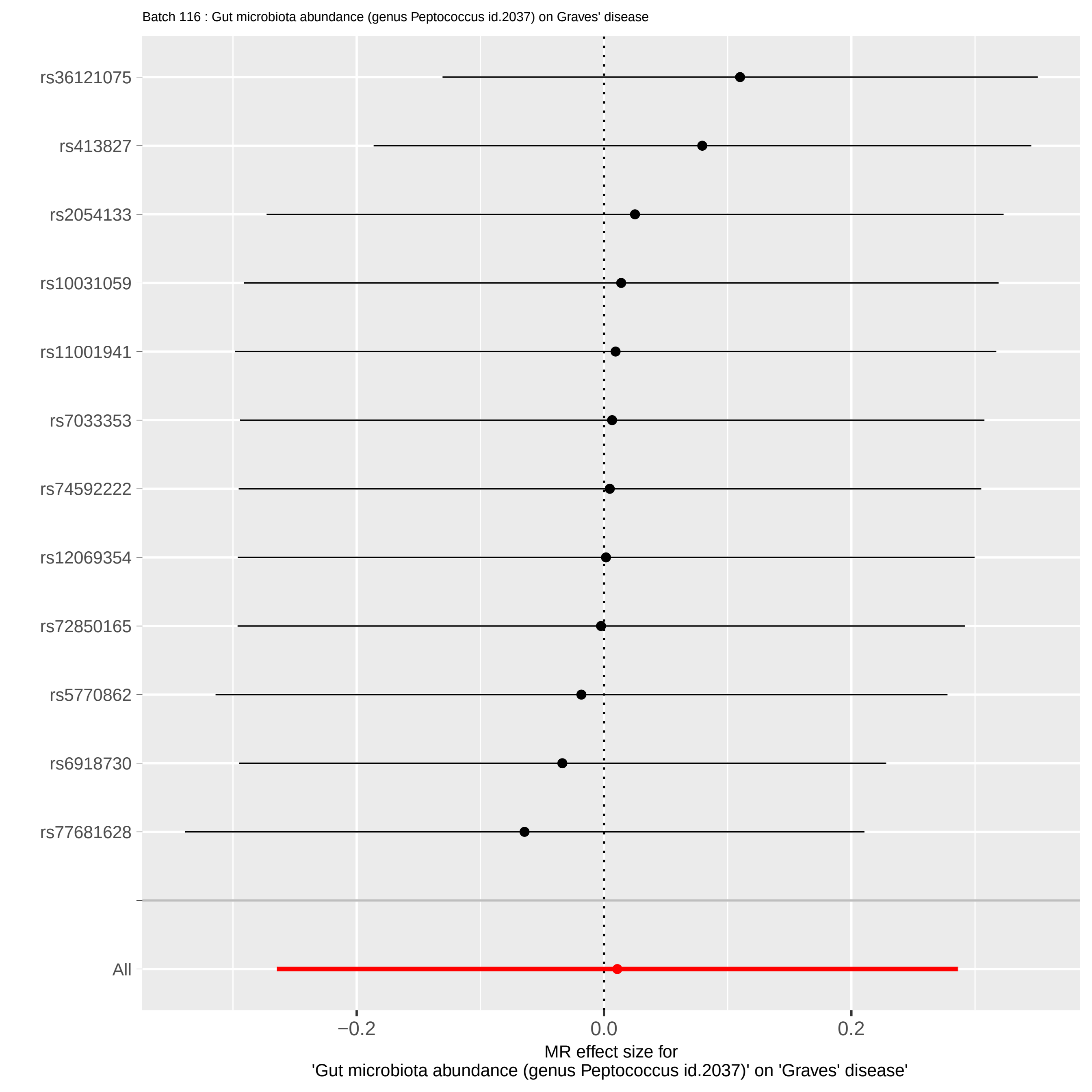

Batch 116 : Gut microbiota abundance (genus Peptococcus id.2037) on Graves' disease
rs36121075
rs413827
rs2054133
rs10031059
rs11001941
rs7033353
rs74592222
rs12069354
rs72850165
rs5770862
rs6918730
rs77681628
All
−0.2
0.0
0.2
MR effect size for
'Gut microbiota abundance (genus Peptococcus id.2037)' on 'Graves' disease'

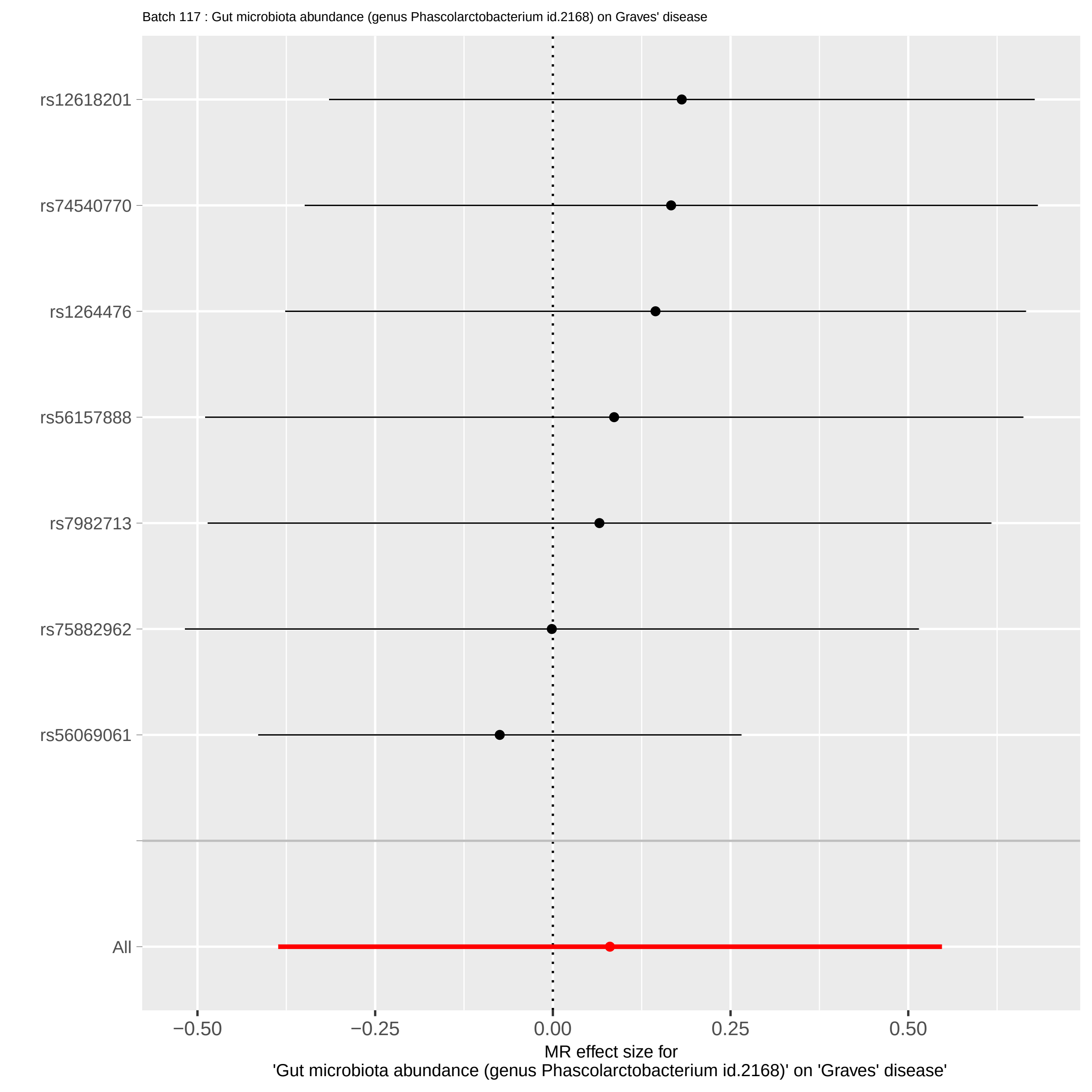

Batch 117 : Gut microbiota abundance (genus Phascolarctobacterium id.2168) on Graves' disease
rs12618201
rs74540770
rs1264476
rs56157888
rs7982713
rs75882962
rs56069061
All
−0.50
−0.25
0.00
0.25
0.50
MR effect size for
'Gut microbiota abundance (genus Phascolarctobacterium id.2168)' on 'Graves' disease'

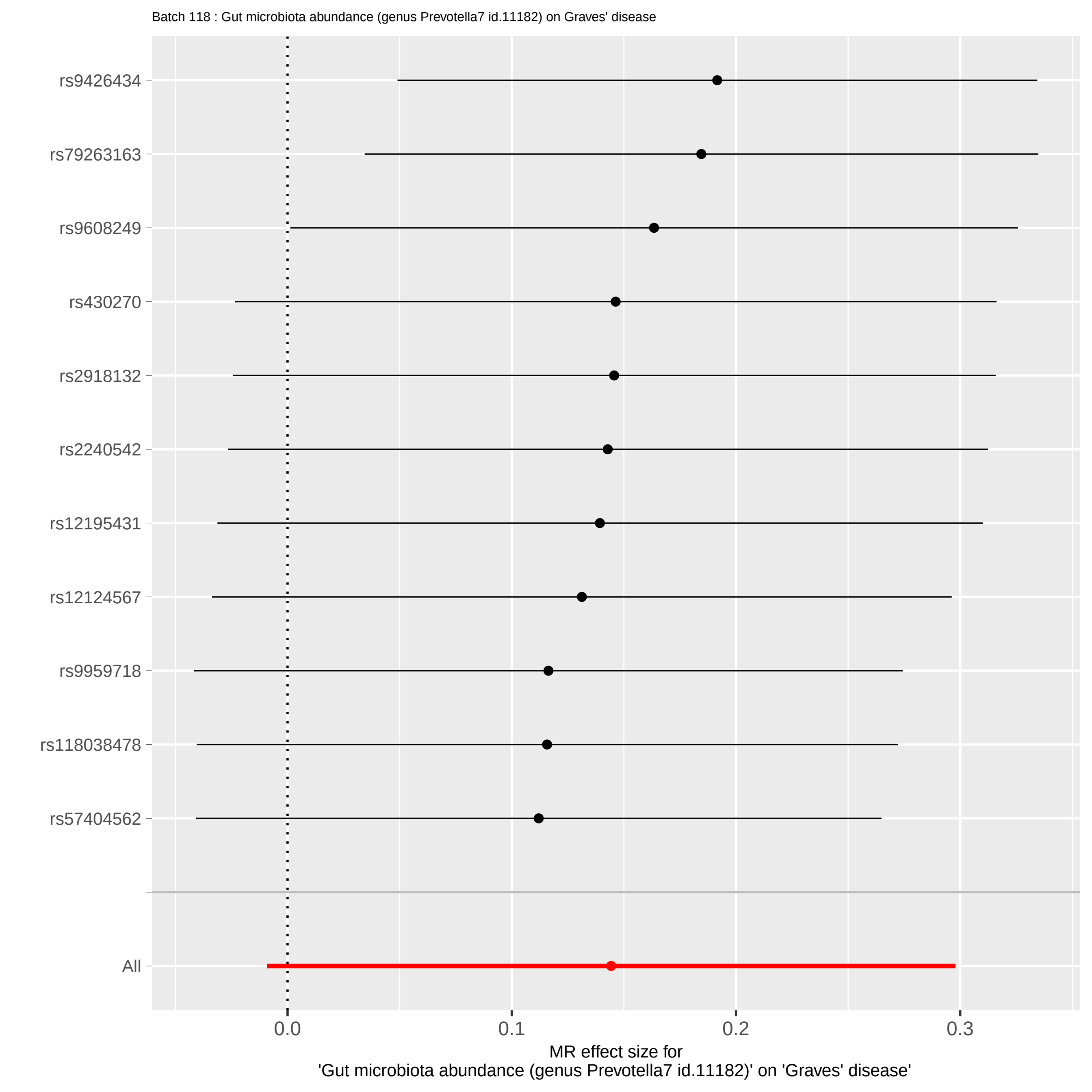

Batch 118 : Gut microbiota abundance (genus Prevotella7 id.11182) on Graves' disease
rs9426434
rs79263163
rs9608249
rs430270
rs2918132
rs2240542
rs12195431
rs12124567
rs9959718
rs118038478
rs57404562
All
0.0
0.1
0.2
0.3
MR effect size for
'Gut microbiota abundance (genus Prevotella7 id.11182)' on 'Graves' disease'

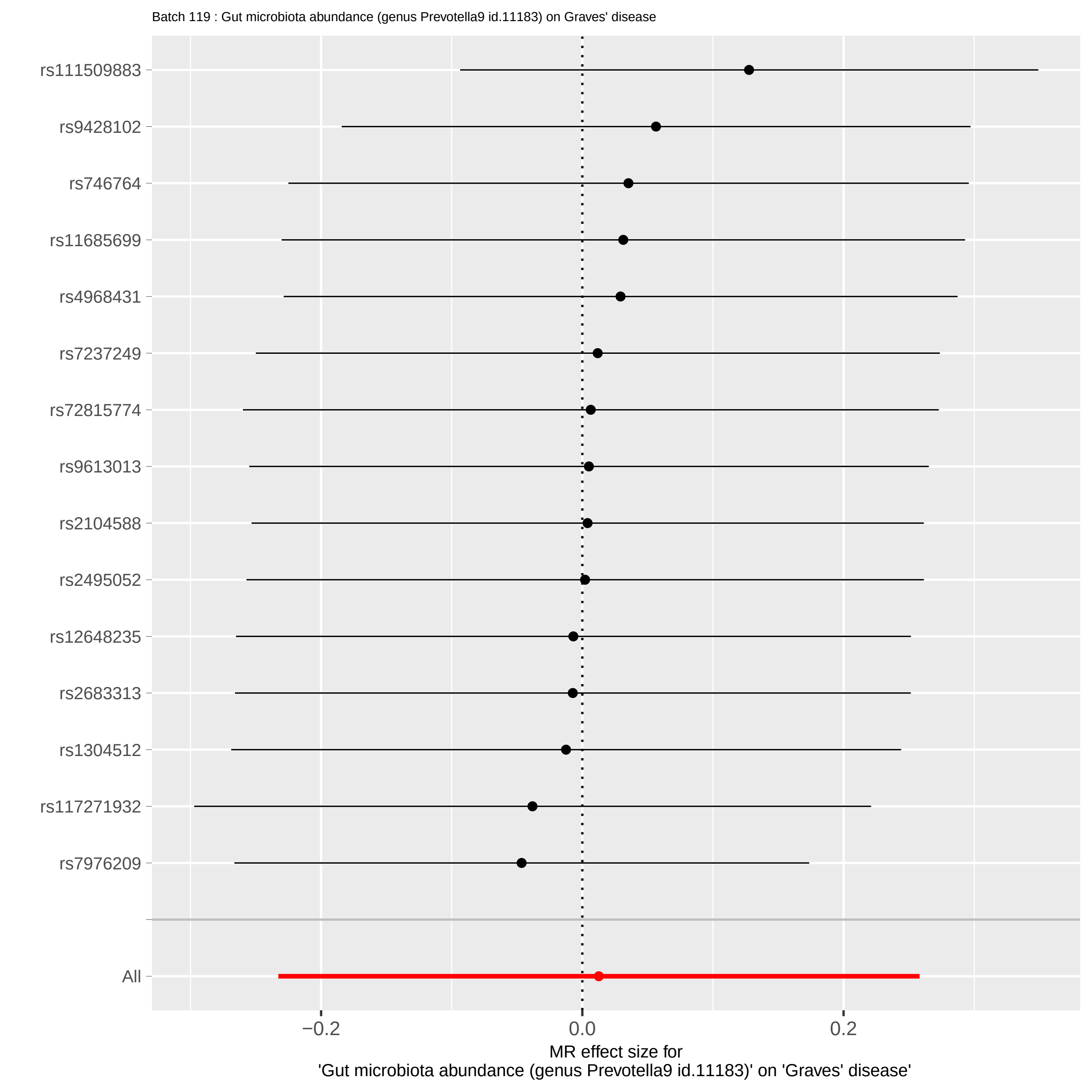

Batch 119 : Gut microbiota abundance (genus Prevotella9 id.11183) on Graves' disease
rs111509883
rs9428102
rs746764
rs11685699
rs4968431
rs7237249
rs72815774
rs9613013
rs2104588
rs2495052
rs12648235
rs2683313
rs1304512
rs117271932
rs7976209
All
−0.2
0.0
0.2
MR effect size for
'Gut microbiota abundance (genus Prevotella9 id.11183)' on 'Graves' disease'

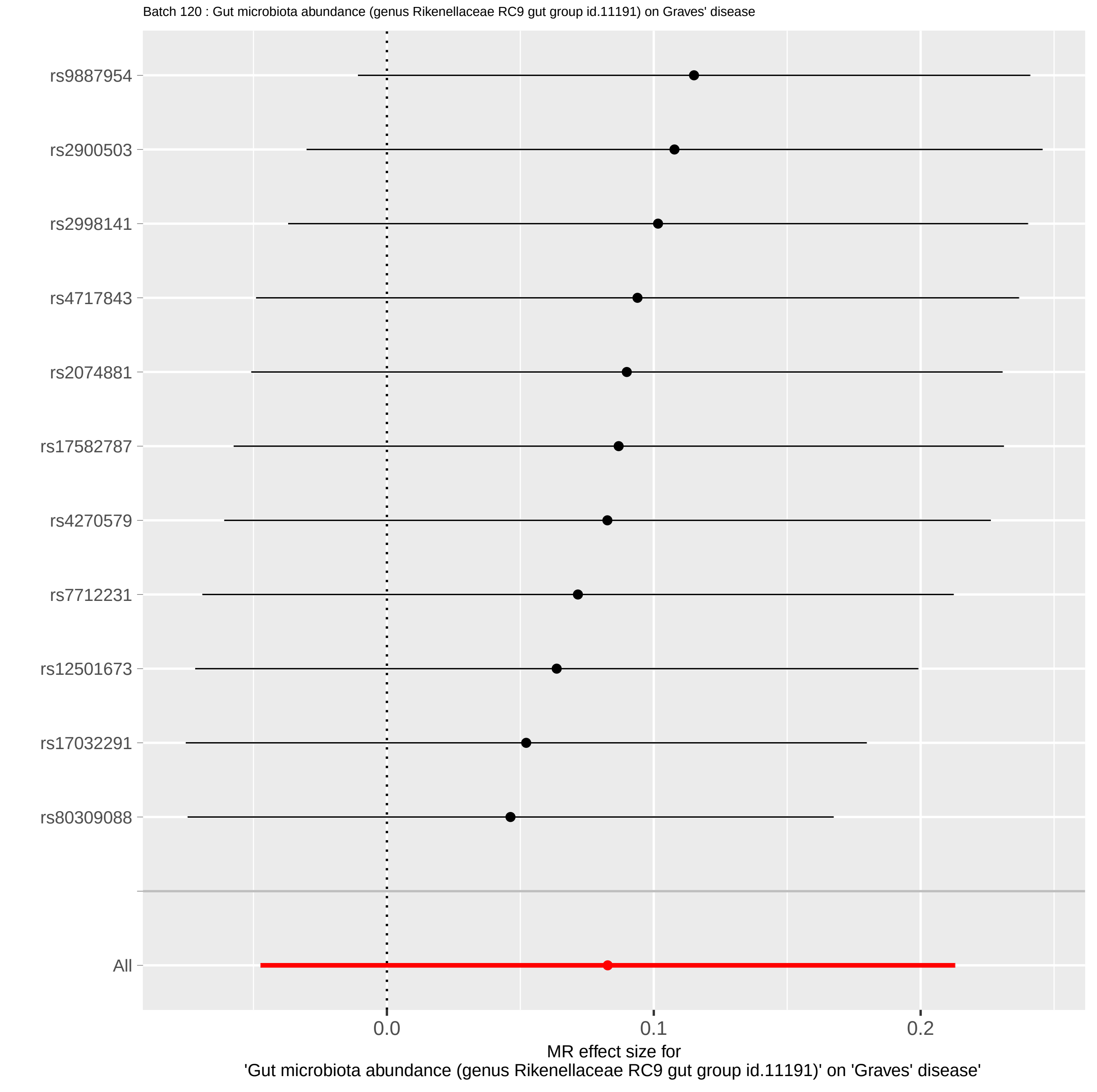

Batch 120 : Gut microbiota abundance (genus Rikenellaceae RC9 gut group id.11191) on Graves' disease
rs9887954
rs2900503
rs2998141
rs4717843
rs2074881
rs17582787
rs4270579
rs7712231
rs12501673
rs17032291
rs80309088
All
0.0
0.1
0.2
MR effect size for
'Gut microbiota abundance (genus Rikenellaceae RC9 gut group id.11191)' on 'Graves' disease'

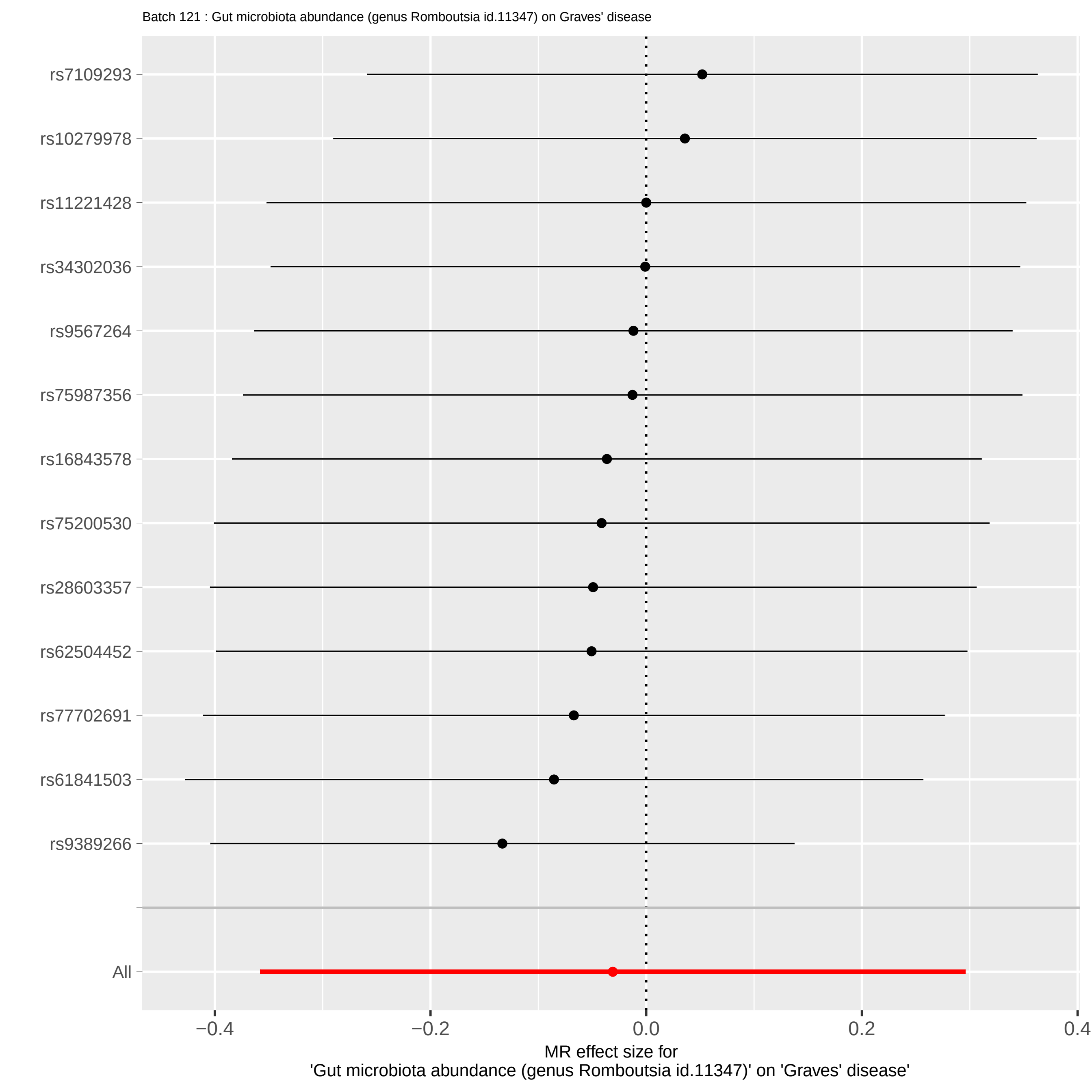

Batch 121 : Gut microbiota abundance (genus Romboutsia id.11347) on Graves' disease
rs7109293
rs10279978
rs11221428
rs34302036
rs9567264
rs75987356
rs16843578
rs75200530
rs28603357
rs62504452
rs77702691
rs61841503
rs9389266
All
−0.4
−0.2
0.0
0.2
0.4
MR effect size for
'Gut microbiota abundance (genus Romboutsia id.11347)' on 'Graves' disease'

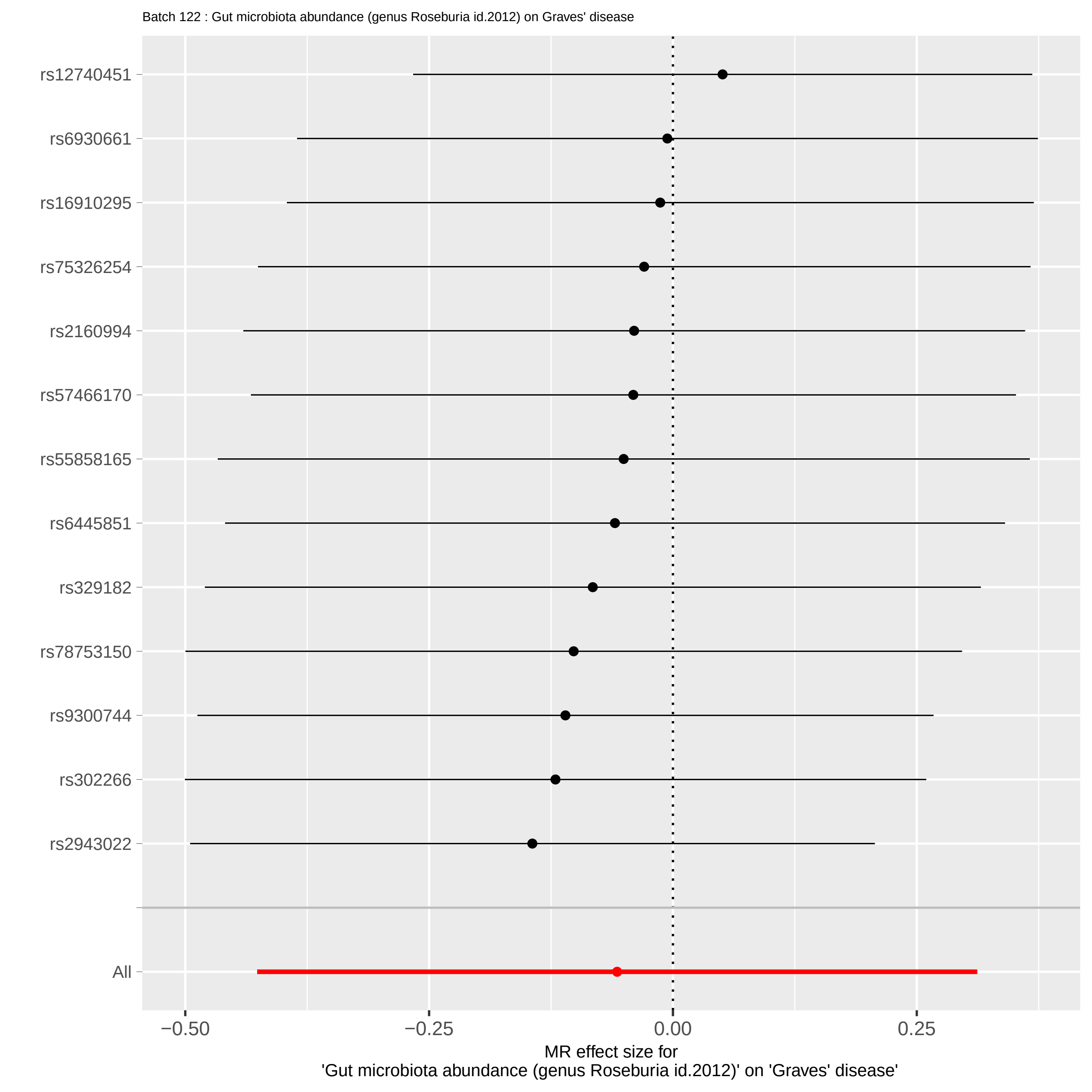

Batch 122 : Gut microbiota abundance (genus Roseburia id.2012) on Graves' disease
rs12740451
rs6930661
rs16910295
rs75326254
rs2160994
rs57466170
rs55858165
rs6445851
rs329182
rs78753150
rs9300744
rs302266
rs2943022
All
−0.50
−0.25
0.00
0.25
MR effect size for
'Gut microbiota abundance (genus Roseburia id.2012)' on 'Graves' disease'

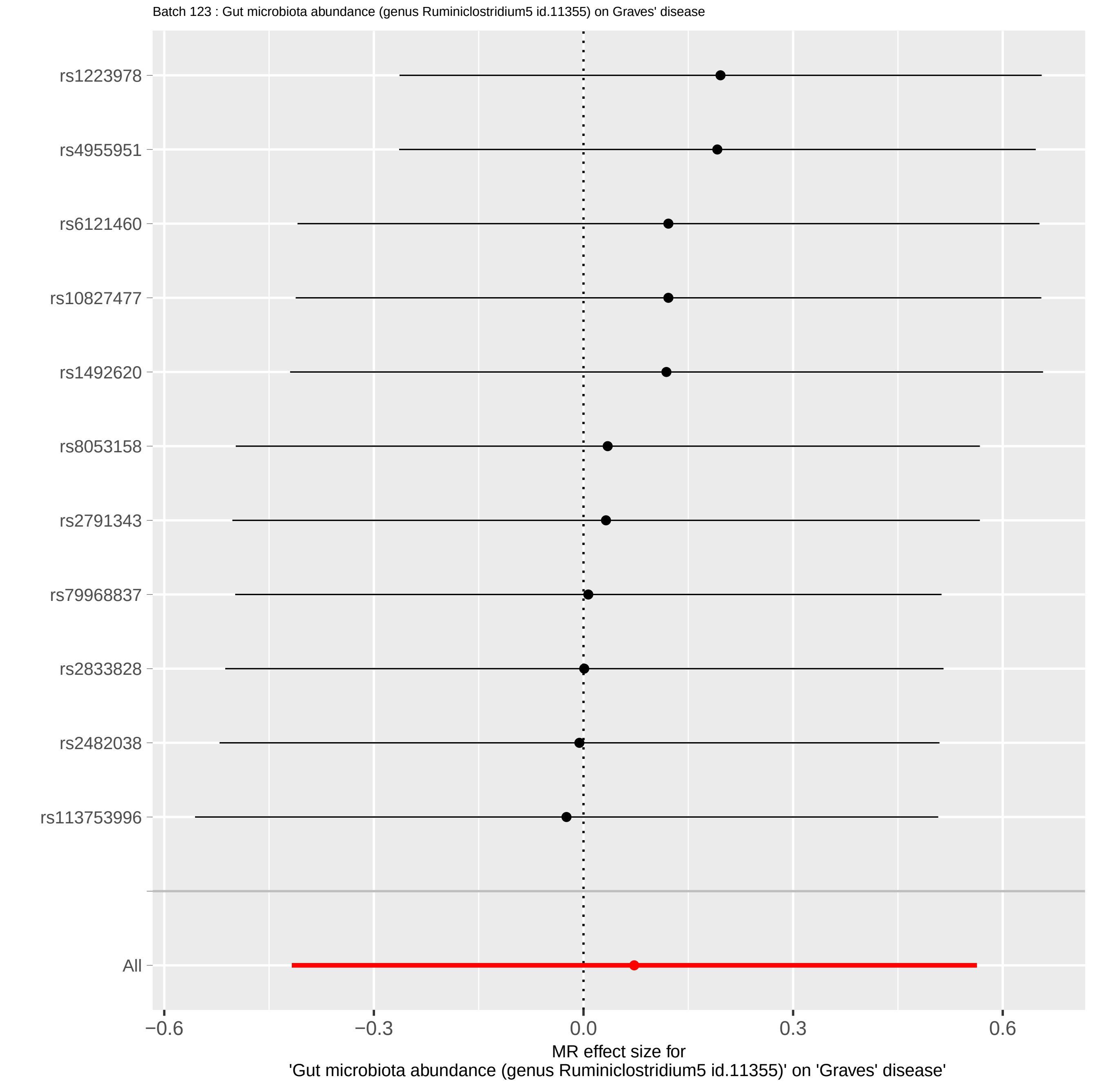

Batch 123 : Gut microbiota abundance (genus Ruminiclostridium5 id.11355) on Graves' disease
rs1223978
rs4955951
rs6121460
rs10827477
rs1492620
rs8053158
rs2791343
rs79968837
rs2833828
rs2482038
rs113753996
All
−0.6
−0.3
0.0
0.3
0.6
MR effect size for
'Gut microbiota abundance (genus Ruminiclostridium5 id.11355)' on 'Graves' disease'

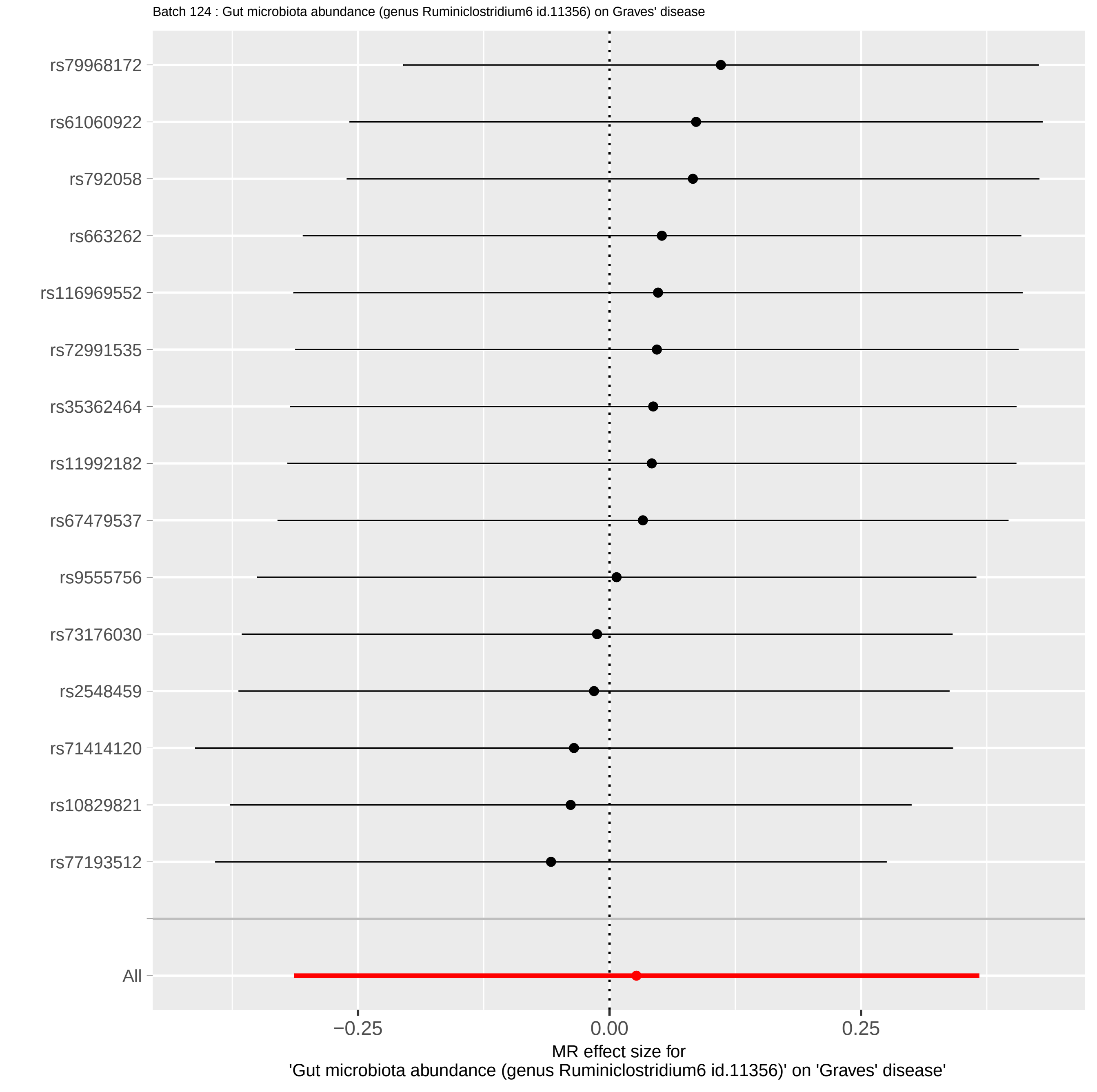

Batch 124 : Gut microbiota abundance (genus Ruminiclostridium6 id.11356) on Graves' disease
rs79968172
rs61060922
rs792058
rs663262
rs116969552
rs72991535
rs35362464
rs11992182
rs67479537
rs9555756
rs73176030
rs2548459
rs71414120
rs10829821
rs77193512
All
−0.25
0.00
0.25
MR effect size for
'Gut microbiota abundance (genus Ruminiclostridium6 id.11356)' on 'Graves' disease'

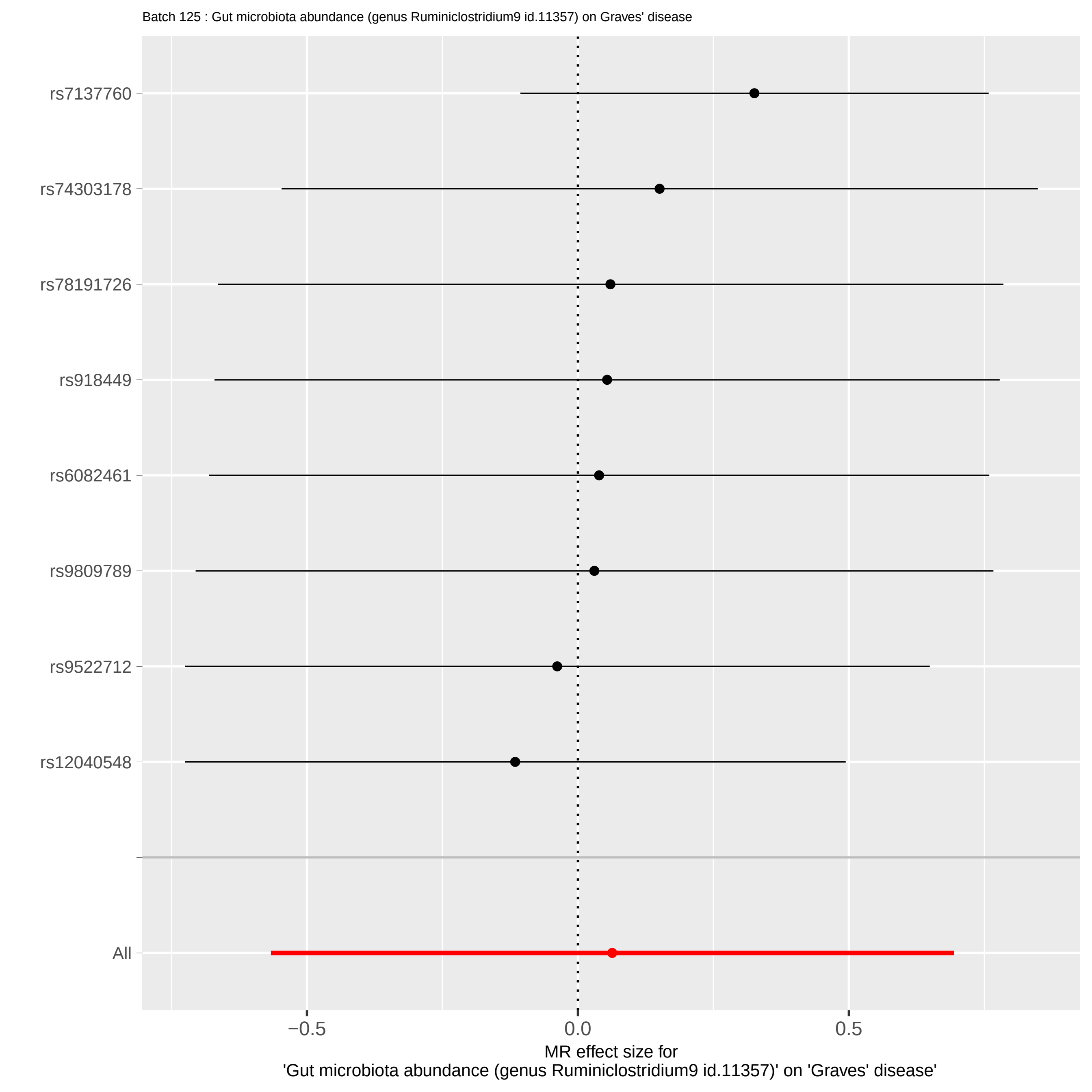

Batch 125 : Gut microbiota abundance (genus Ruminiclostridium9 id.11357) on Graves' disease
rs7137760
rs74303178
rs78191726
rs918449
rs6082461
rs9809789
rs9522712
rs12040548
All
−0.5
0.0
0.5
MR effect size for
'Gut microbiota abundance (genus Ruminiclostridium9 id.11357)' on 'Graves' disease'

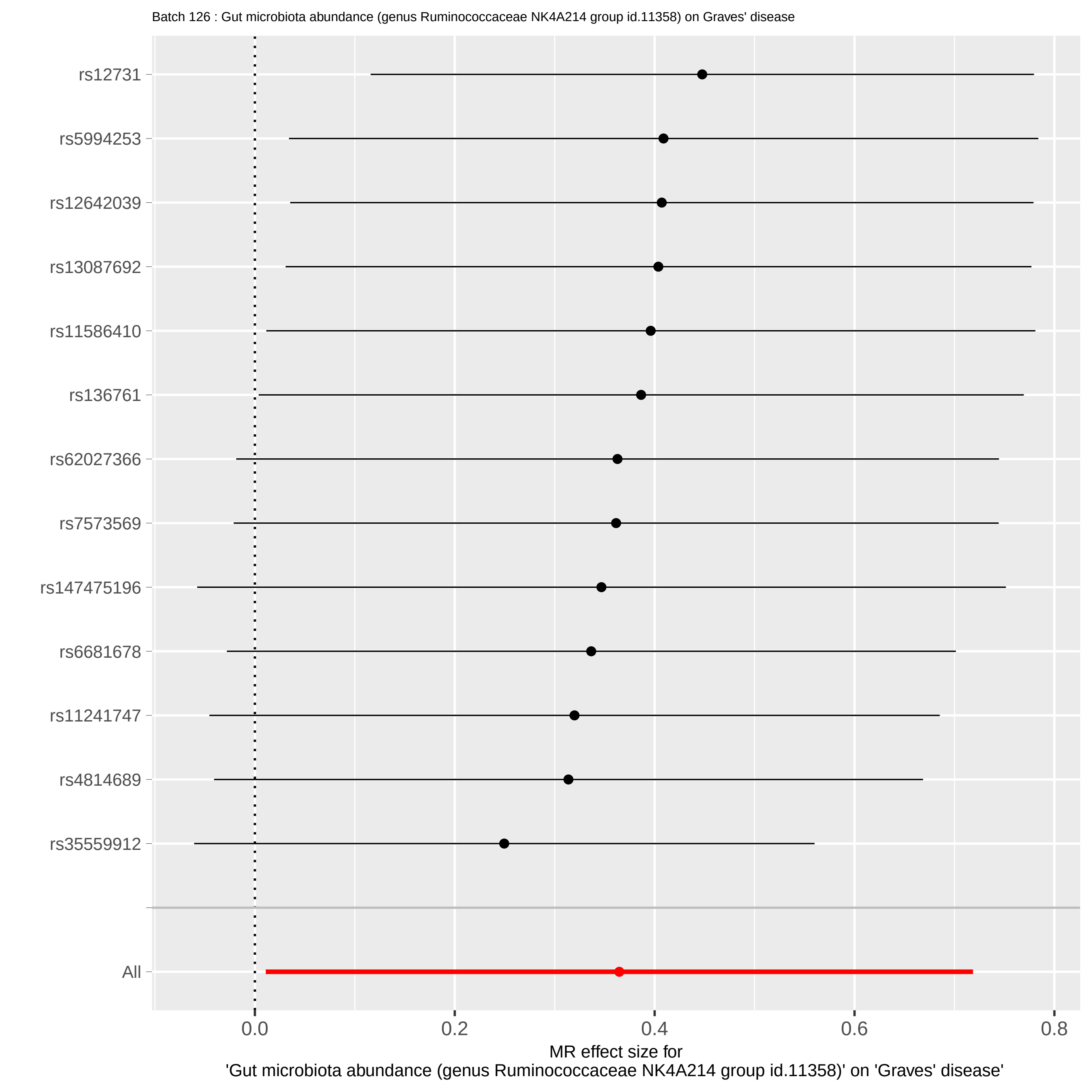
Batch 126 : Gut microbiota abundance (genus Ruminococcaceae NK4A214 group id.11358) on Graves' disease
rs12731
rs5994253
rs12642039
rs13087692
rs11586410
rs136761
rs62027366
rs7573569
rs147475196
rs6681678
rs11241747
rs4814689
rs35559912
All
0.0
0.2
0.4
0.6
0.8
MR effect size for
'Gut microbiota abundance (genus Ruminococcaceae NK4A214 group id.11358)' on 'Graves' disease'

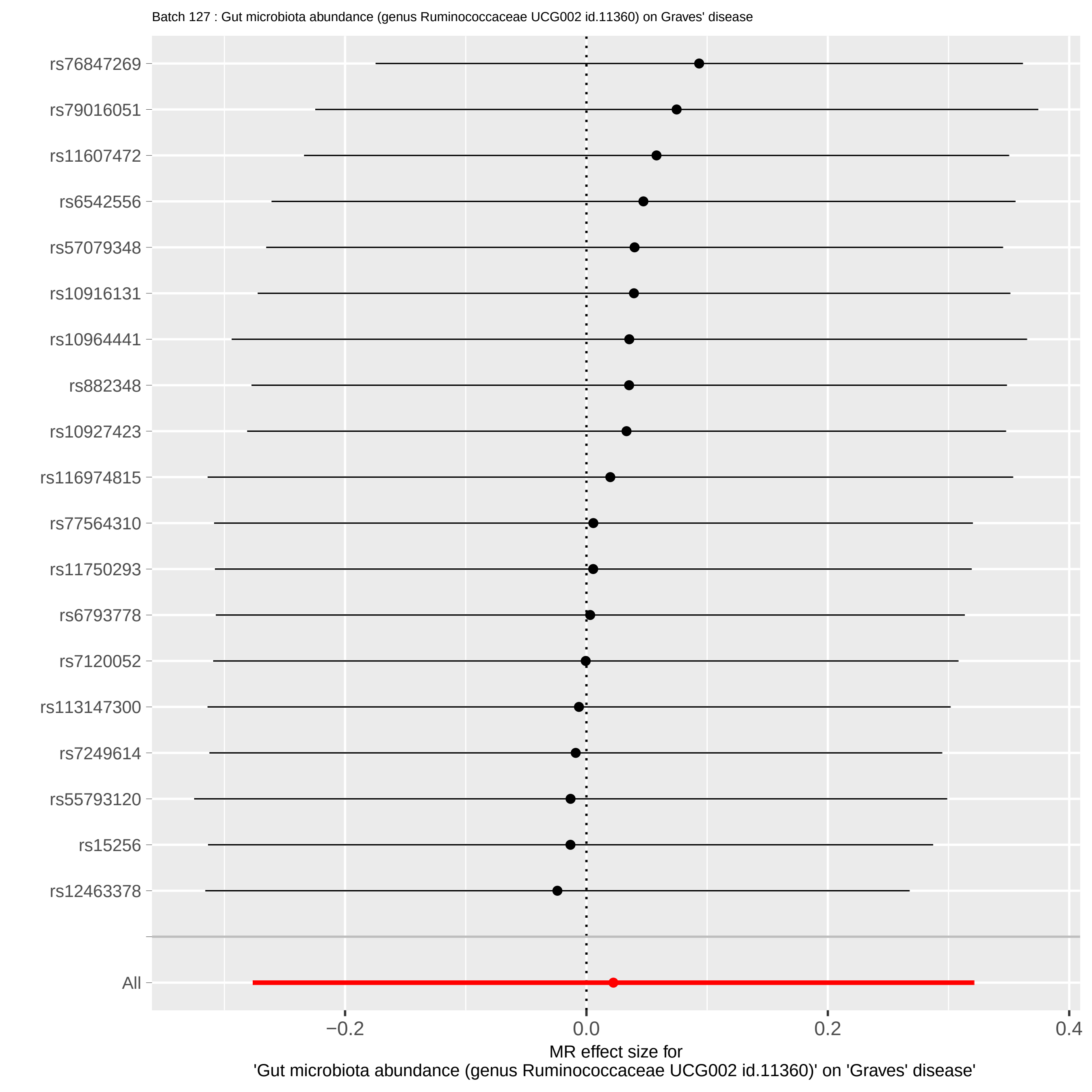

Batch 127 : Gut microbiota abundance (genus Ruminococcaceae UCG002 id.11360) on Graves' disease
rs76847269
rs79016051
rs11607472
rs6542556
rs57079348
rs10916131
rs10964441
rs882348
rs10927423
rs116974815
rs77564310
rs11750293
rs6793778
rs7120052
rs113147300
rs7249614
rs55793120
rs15256
rs12463378
All
−0.2
0.0
0.2
0.4
MR effect size for
'Gut microbiota abundance (genus Ruminococcaceae UCG002 id.11360)' on 'Graves' disease'

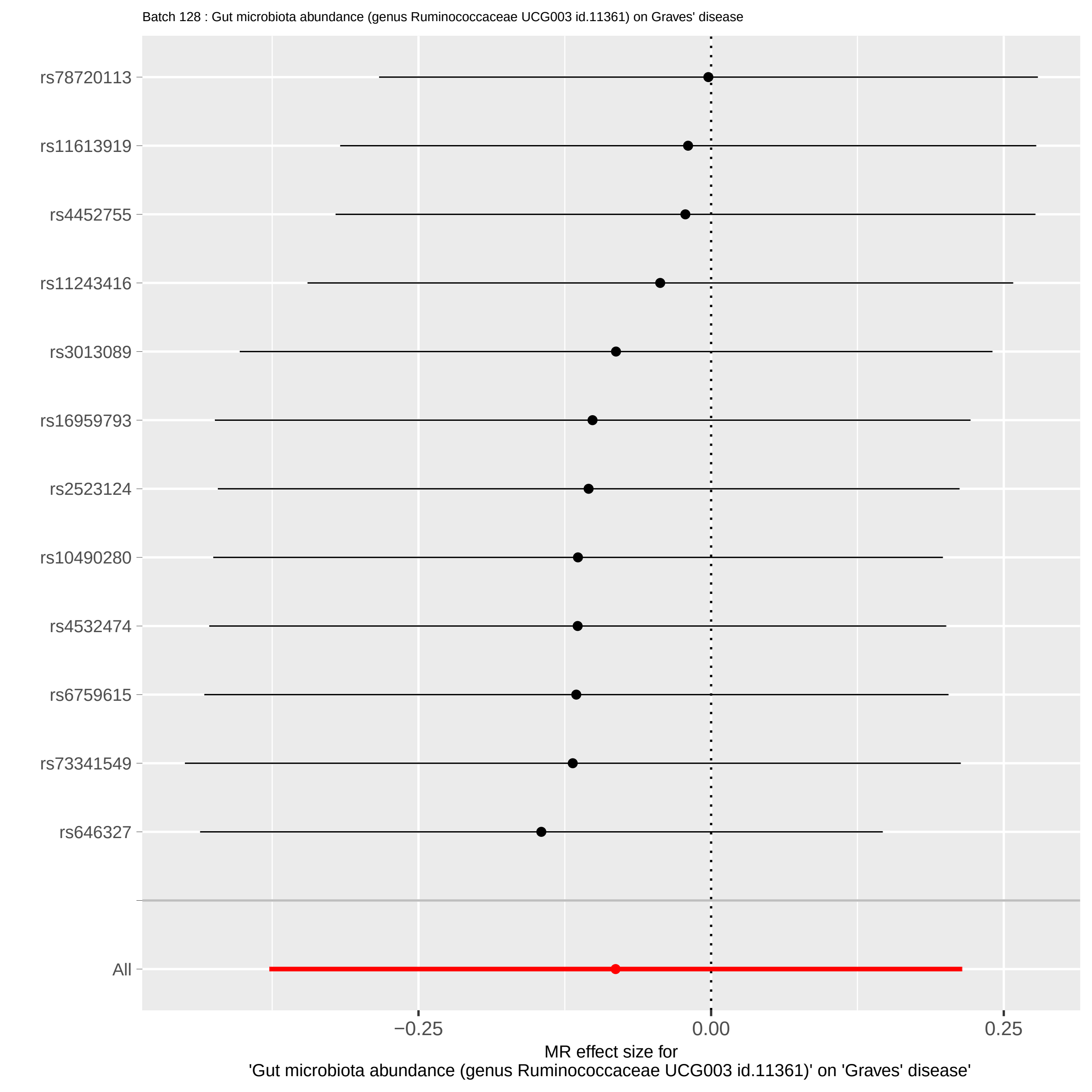

Batch 128 : Gut microbiota abundance (genus Ruminococcaceae UCG003 id.11361) on Graves' disease
rs78720113
rs11613919
rs4452755
rs11243416
rs3013089
rs16959793
rs2523124
rs10490280
rs4532474
rs6759615
rs73341549
rs646327
All
−0.25
0.00
0.25
MR effect size for
'Gut microbiota abundance (genus Ruminococcaceae UCG003 id.11361)' on 'Graves' disease'

Batch 129 : Gut microbiota abundance (genus Ruminococcaceae UCG004 id.11362) on Graves' disease

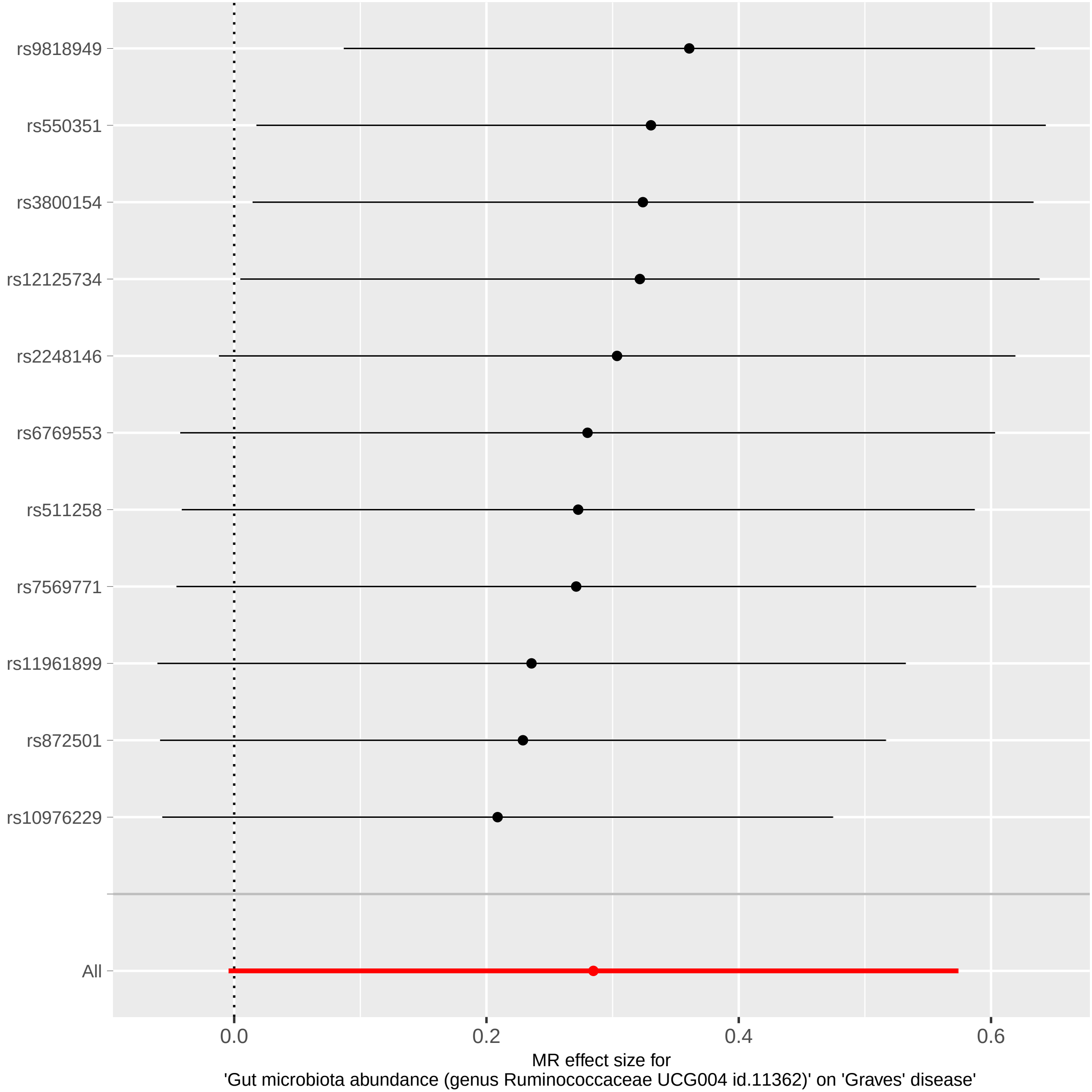

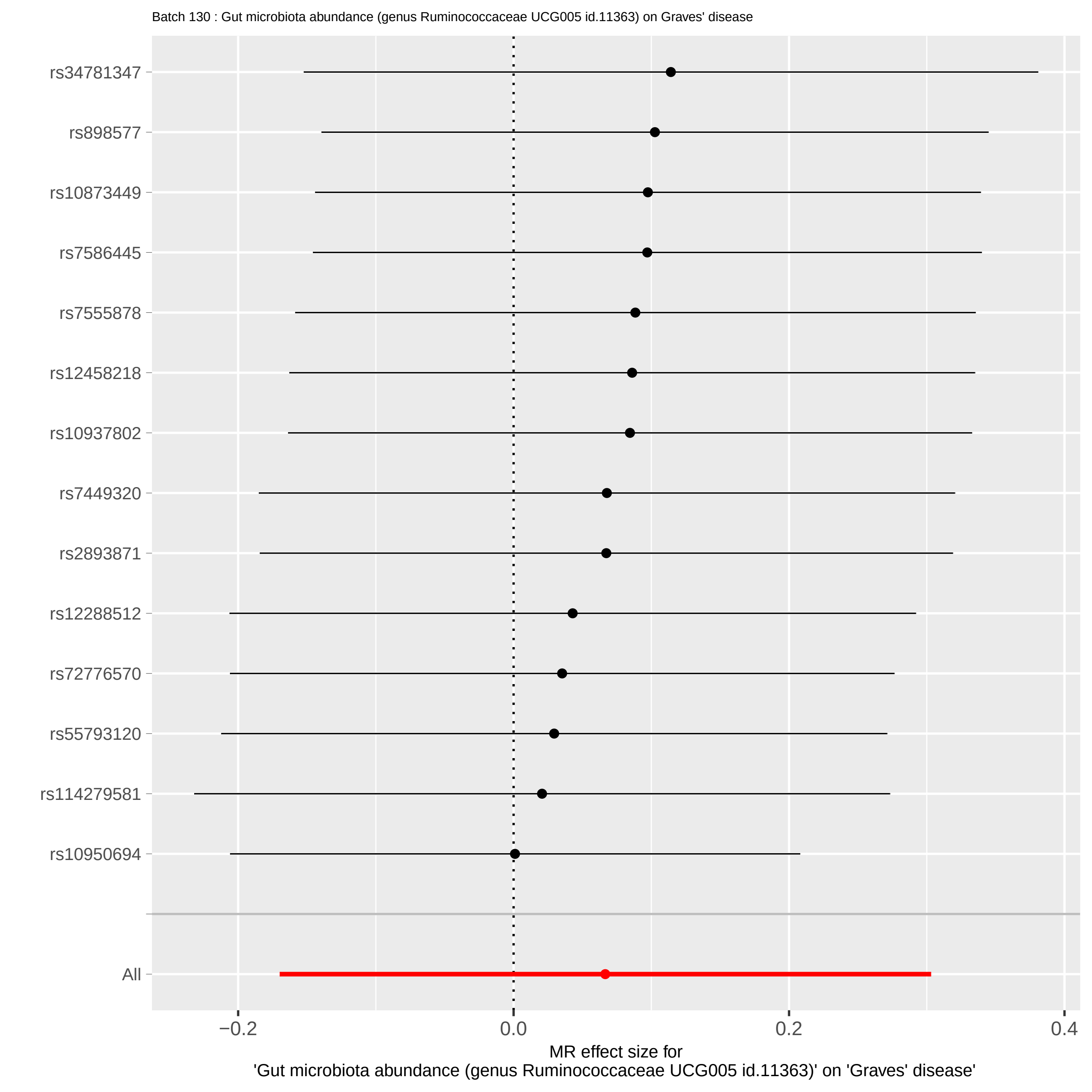

Batch 130 : Gut microbiota abundance (genus Ruminococcaceae UCG005 id.11363) on Graves' disease
rs34781347
rs898577
rs10873449
rs7586445
rs7555878
rs12458218
rs10937802
rs7449320
rs2893871
rs12288512
rs72776570
rs55793120
rs114279581
rs10950694
All
−0.2
0.0
0.2
0.4
MR effect size for
'Gut microbiota abundance (genus Ruminococcaceae UCG005 id.11363)' on 'Graves' disease'

Batch 131 : Gut microbiota abundance (genus Ruminococcaceae UCG009 id.11366) on Graves' disease

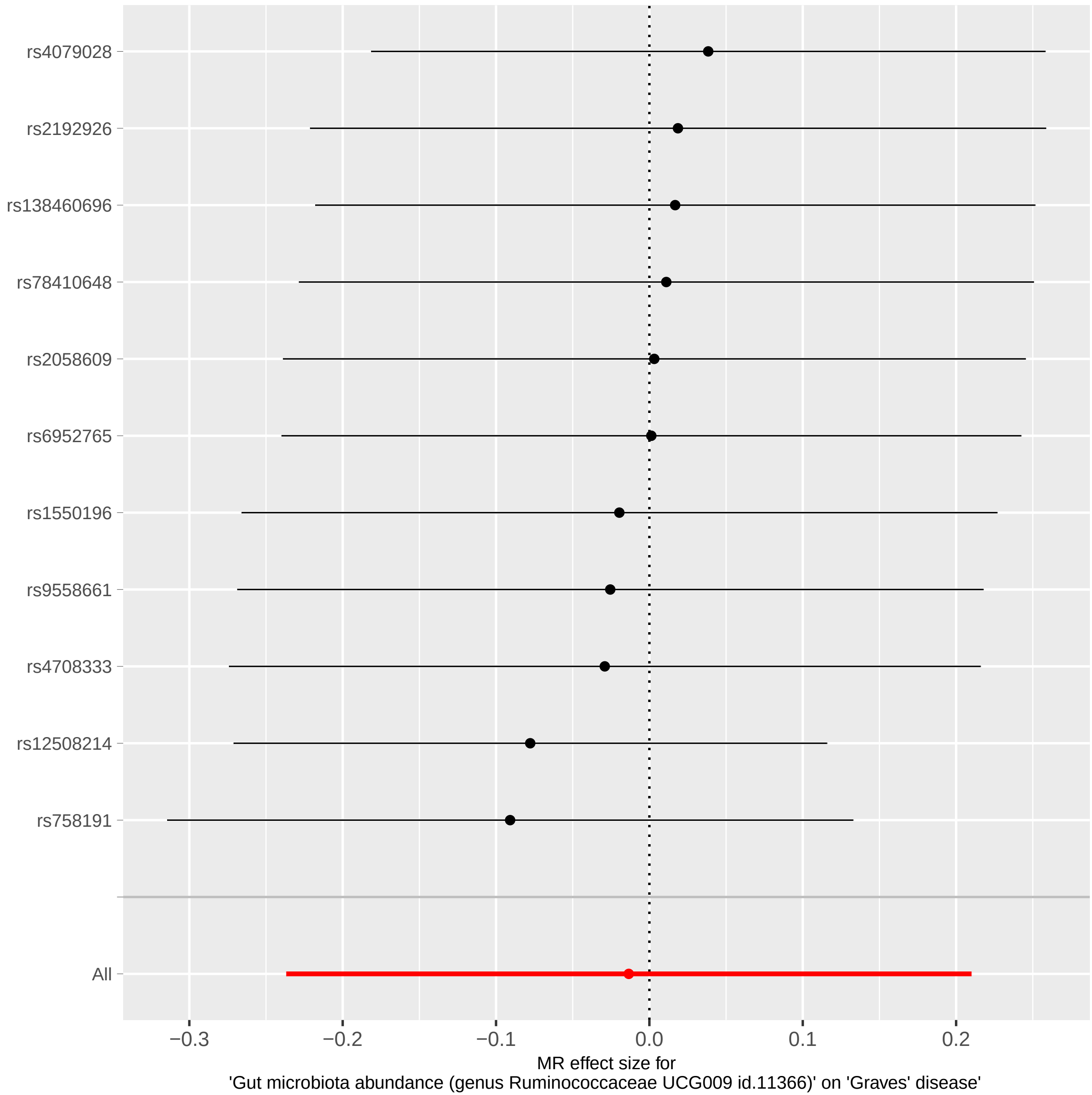

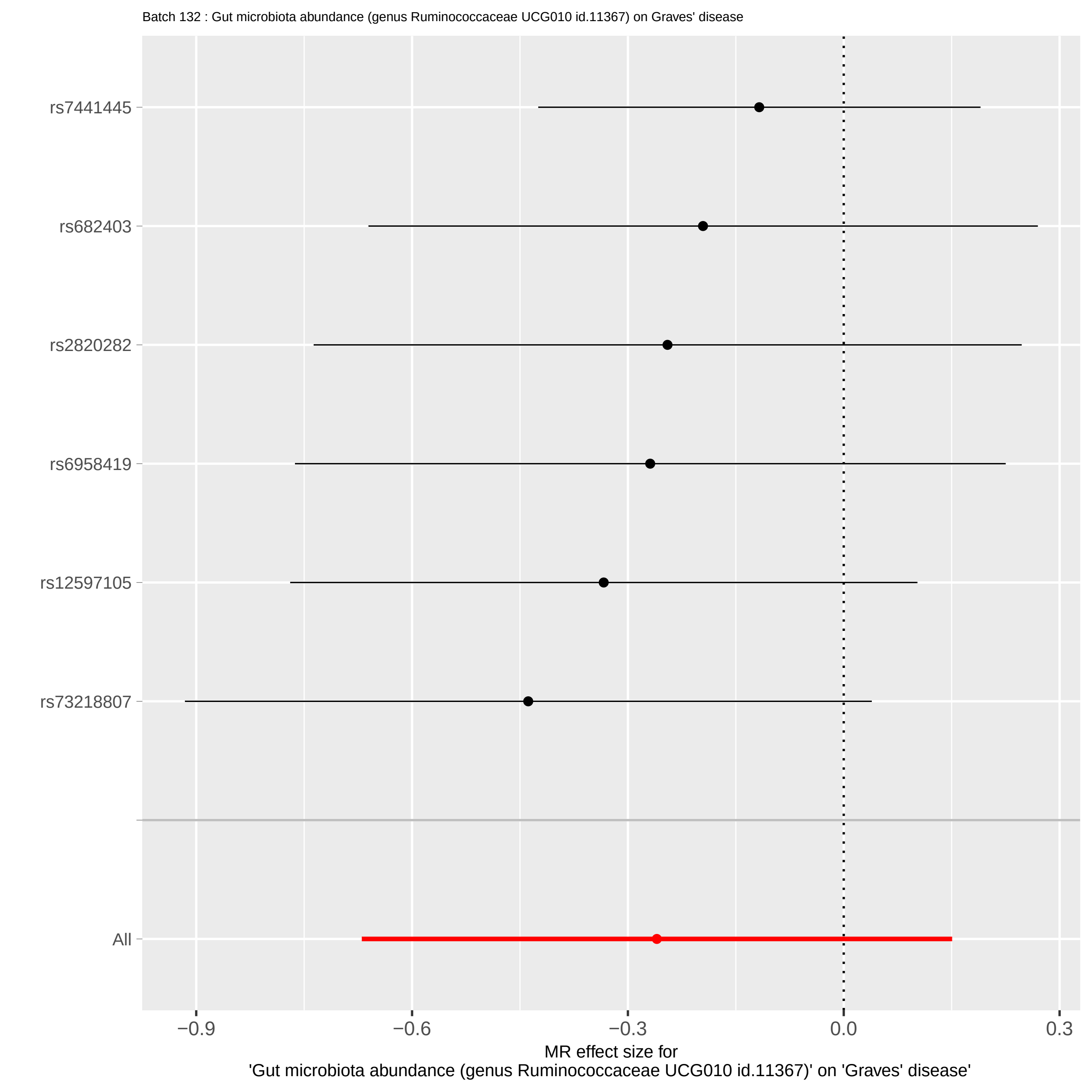
Batch 132 : Gut microbiota abundance (genus Ruminococcaceae UCG010 id.11367) on Graves' disease
rs7441445
rs682403
rs2820282
rs6958419
rs12597105
rs73218807
All
−0.9
−0.6
−0.3
0.0
0.3
MR effect size for
'Gut microbiota abundance (genus Ruminococcaceae UCG010 id.11367)' on 'Graves' disease'

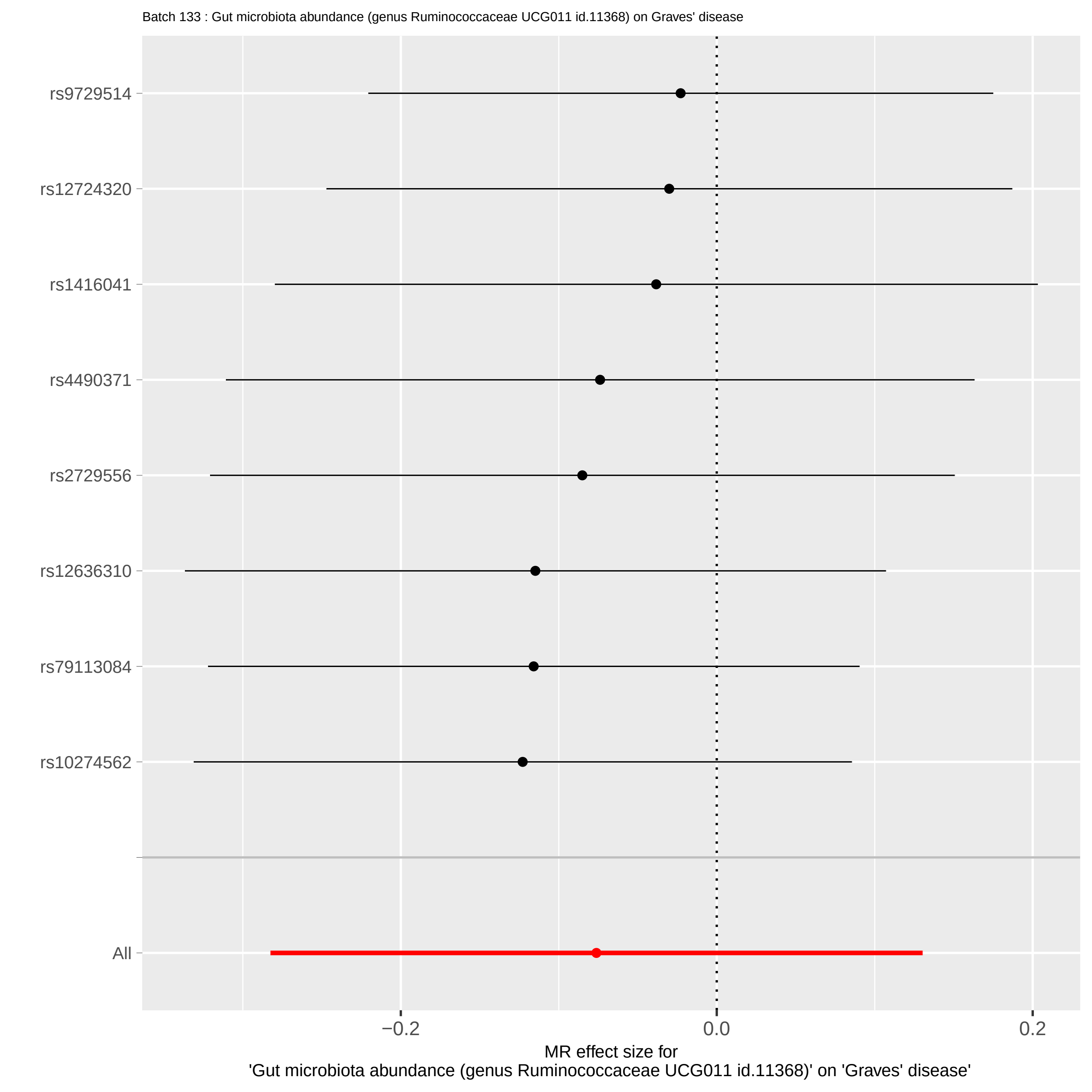
Batch 133 : Gut microbiota abundance (genus Ruminococcaceae UCG011 id.11368) on Graves' disease
rs9729514
rs12724320
rs1416041
rs4490371
rs2729556
rs12636310
rs79113084
rs10274562
All
−0.2
0.0
0.2
MR effect size for
'Gut microbiota abundance (genus Ruminococcaceae UCG011 id.11368)' on 'Graves' disease'

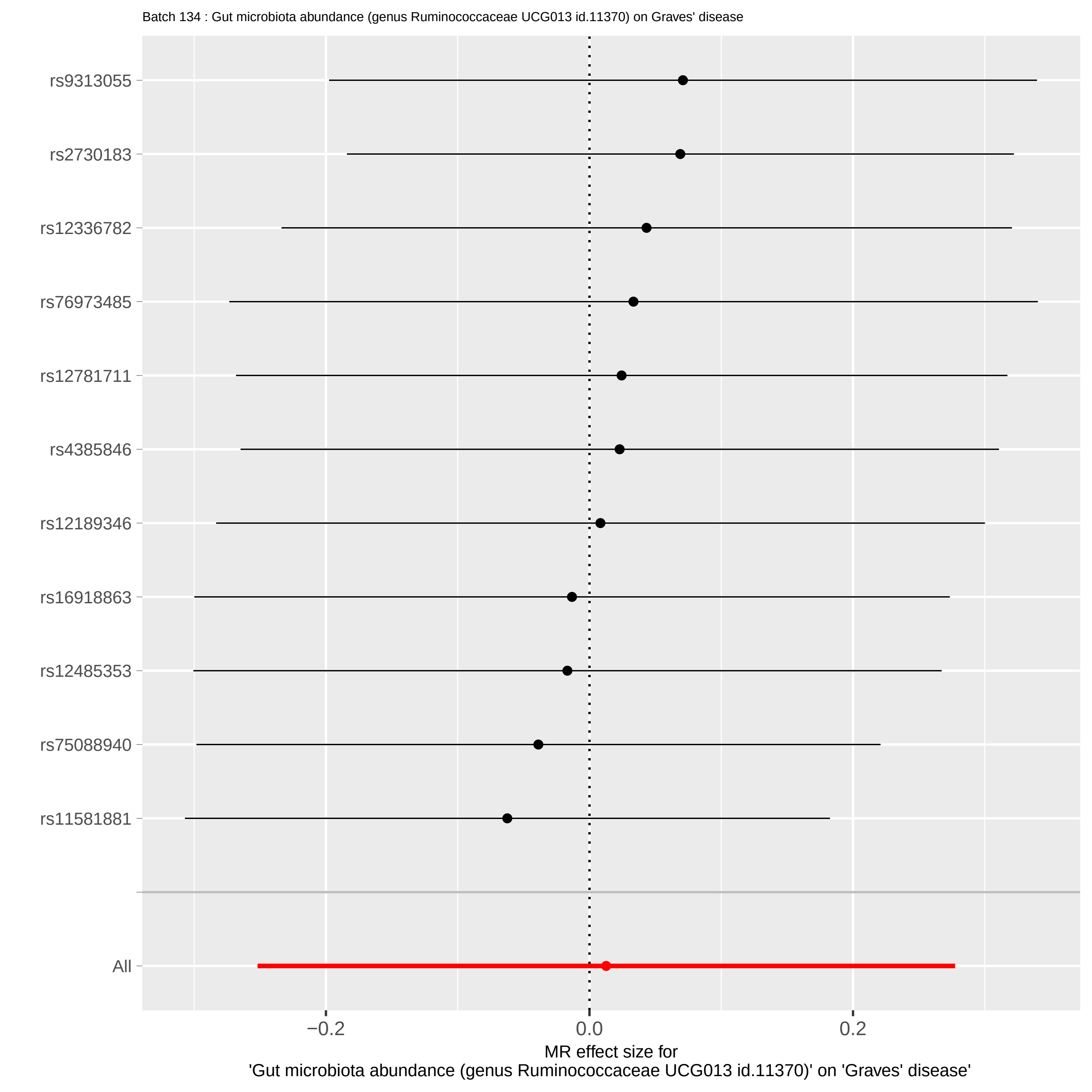
Batch 134 : Gut microbiota abundance (genus Ruminococcaceae UCG013 id.11370) on Graves' disease
rs9313055
rs2730183
rs12336782
rs76973485
rs12781711
rs4385846
rs12189346
rs16918863
rs12485353
rs75088940
rs11581881
All
−0.2
0.0
0.2
MR effect size for
'Gut microbiota abundance (genus Ruminococcaceae UCG013 id.11370)' on 'Graves' disease'

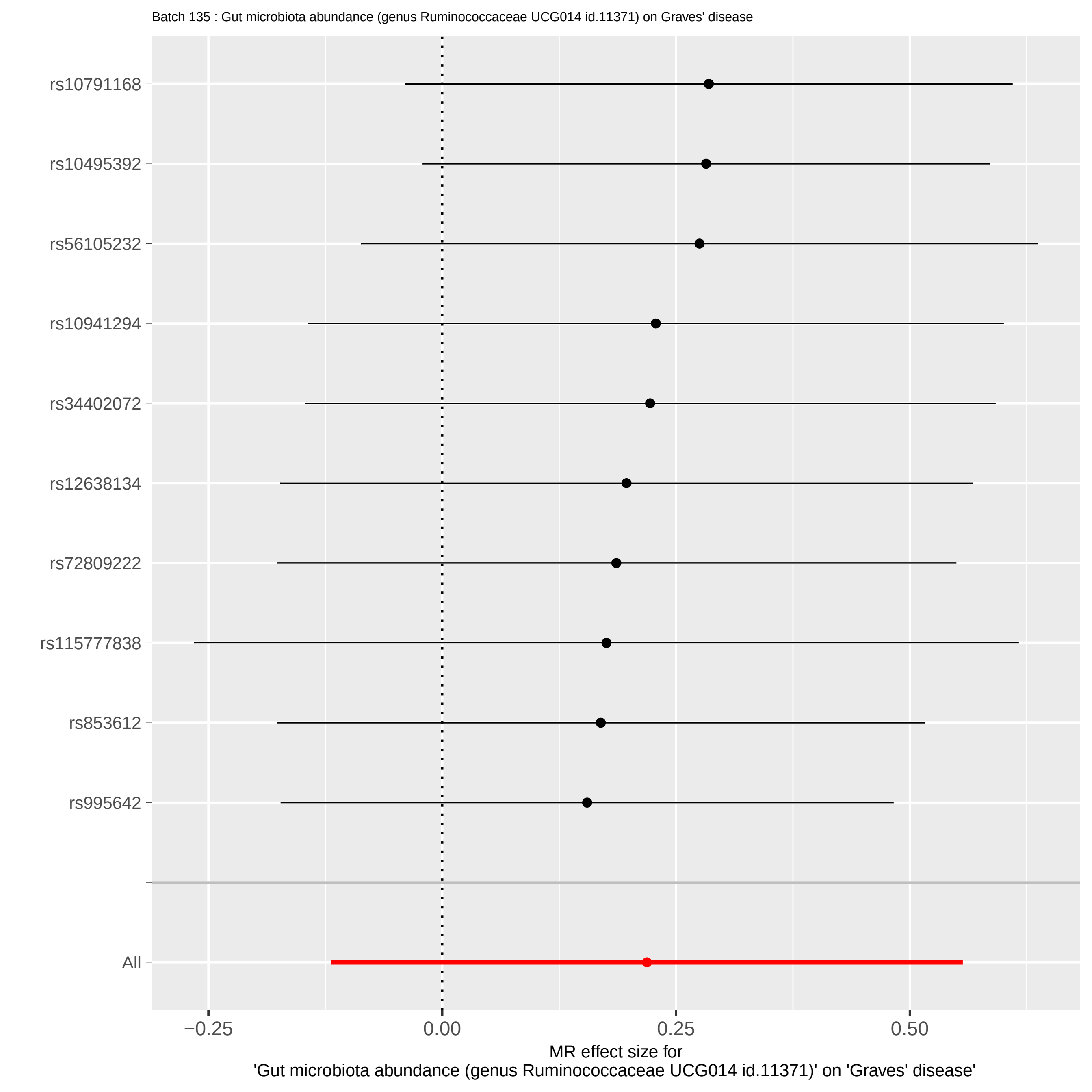
Batch 135 : Gut microbiota abundance (genus Ruminococcaceae UCG014 id.11371) on Graves' disease
rs10791168
rs10495392
rs56105232
rs10941294
rs34402072
rs12638134
rs72809222
rs115777838
rs853612
rs995642
All
−0.25
0.00
0.25
0.50
MR effect size for
'Gut microbiota abundance (genus Ruminococcaceae UCG014 id.11371)' on 'Graves' disease'

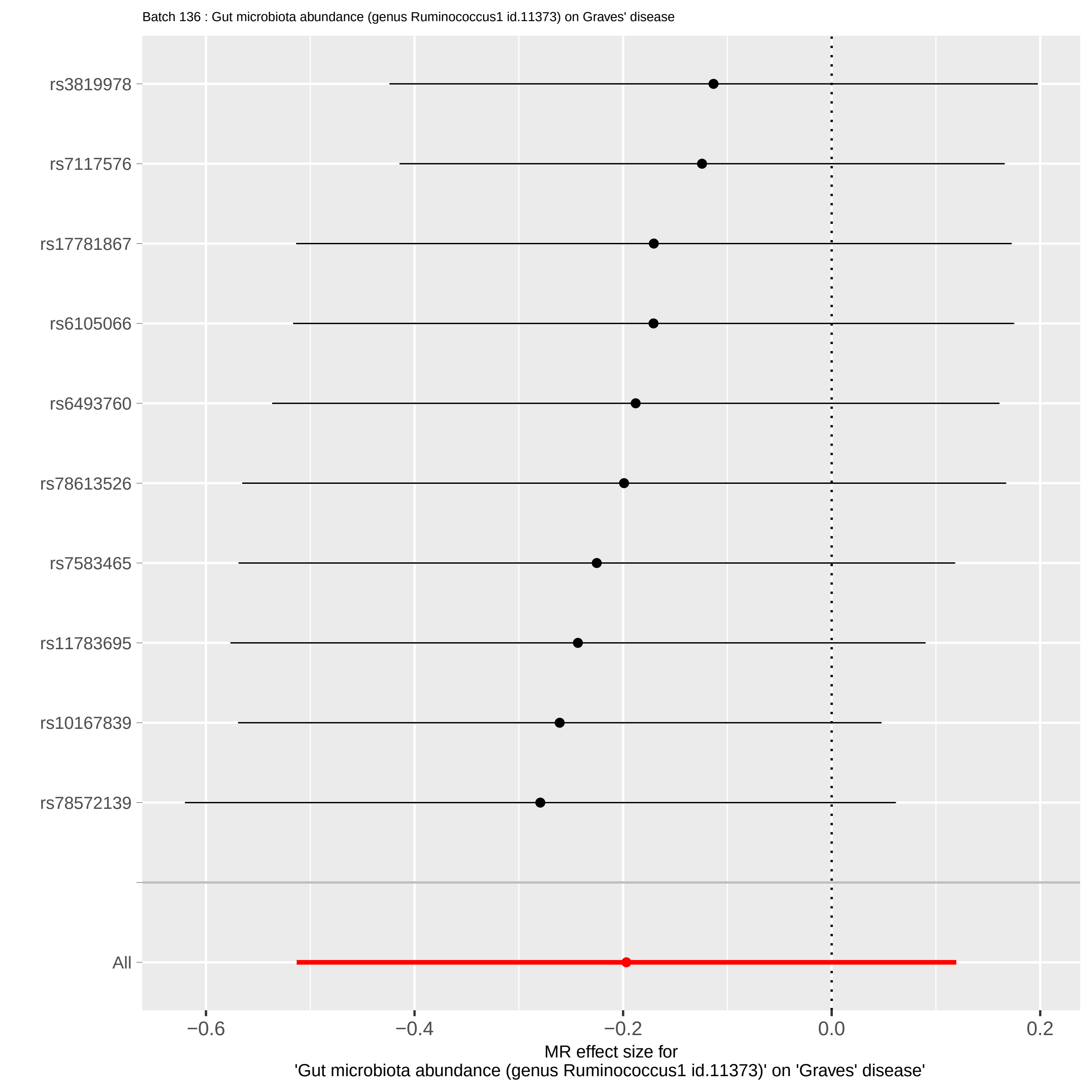

Batch 136 : Gut microbiota abundance (genus Ruminococcus1 id.11373) on Graves' disease
rs3819978
rs7117576
rs17781867
rs6105066
rs6493760
rs78613526
rs7583465
rs11783695
rs10167839
rs78572139
All
−0.6
−0.4
−0.2
0.0
0.2
MR effect size for
'Gut microbiota abundance (genus Ruminococcus1 id.11373)' on 'Graves' disease'

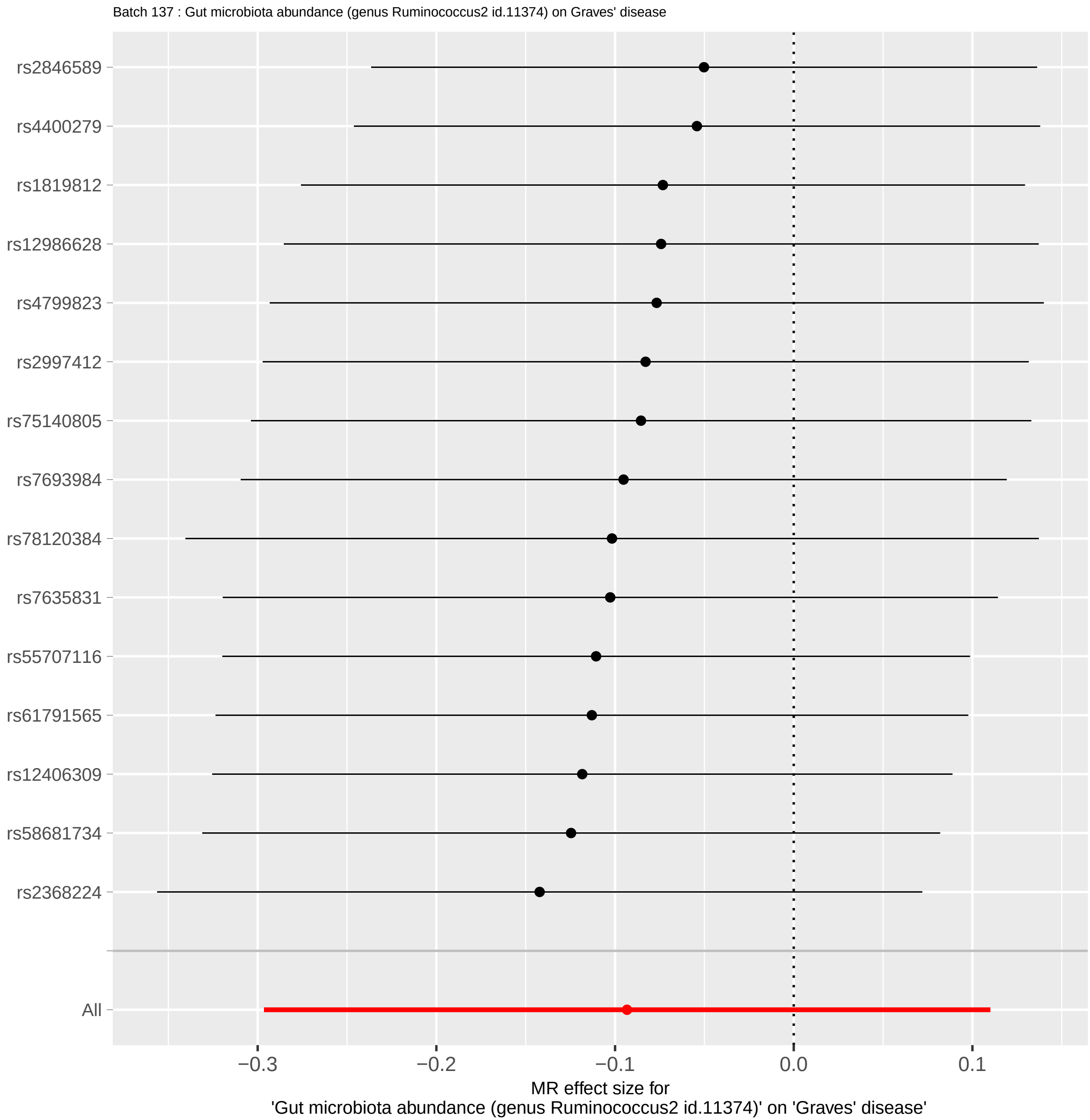

Batch 137 : Gut microbiota abundance (genus Ruminococcus2 id.11374) on Graves' disease
rs2846589
rs4400279
rs1819812
rs12986628
rs4799823
rs2997412
rs75140805
rs7693984
rs78120384
rs7635831
rs55707116
rs61791565
rs12406309
rs58681734
rs2368224
All
−0.3
−0.2
−0.1
0.0
0.1
MR effect size for
'Gut microbiota abundance (genus Ruminococcus2 id.11374)' on 'Graves' disease'

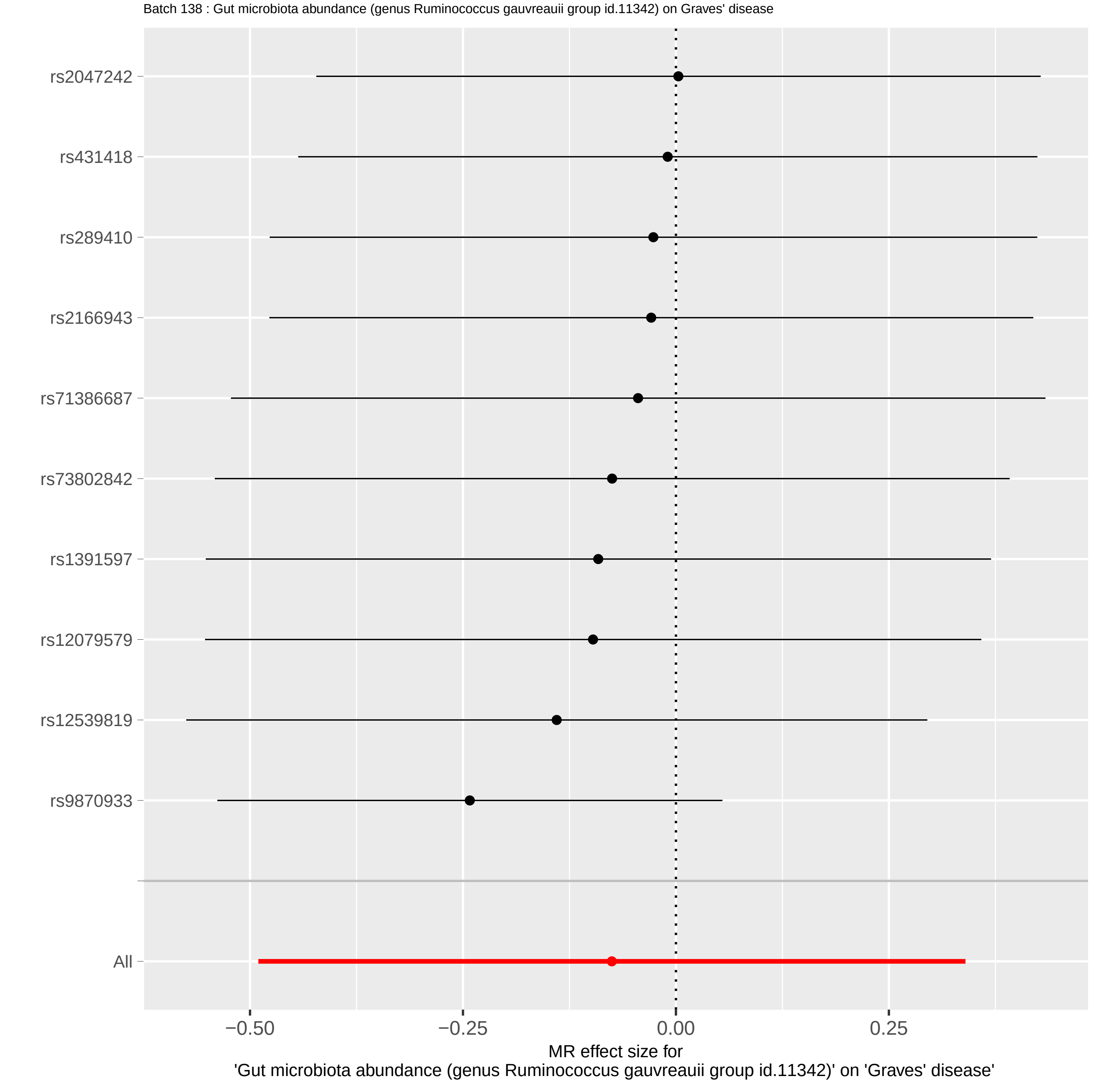

Batch 138 : Gut microbiota abundance (genus Ruminococcus gauvreauii group id.11342) on Graves' disease
rs2047242
rs431418
rs289410
rs2166943
rs71386687
rs73802842
rs1391597
rs12079579
rs12539819
rs9870933
All
−0.50
−0.25
0.00
0.25
MR effect size for
'Gut microbiota abundance (genus Ruminococcus gauvreauii group id.11342)' on 'Graves' disease'

Batch 139 : Gut microbiota abundance (genus Ruminococcus gnavus group id.14376) on Graves' disease

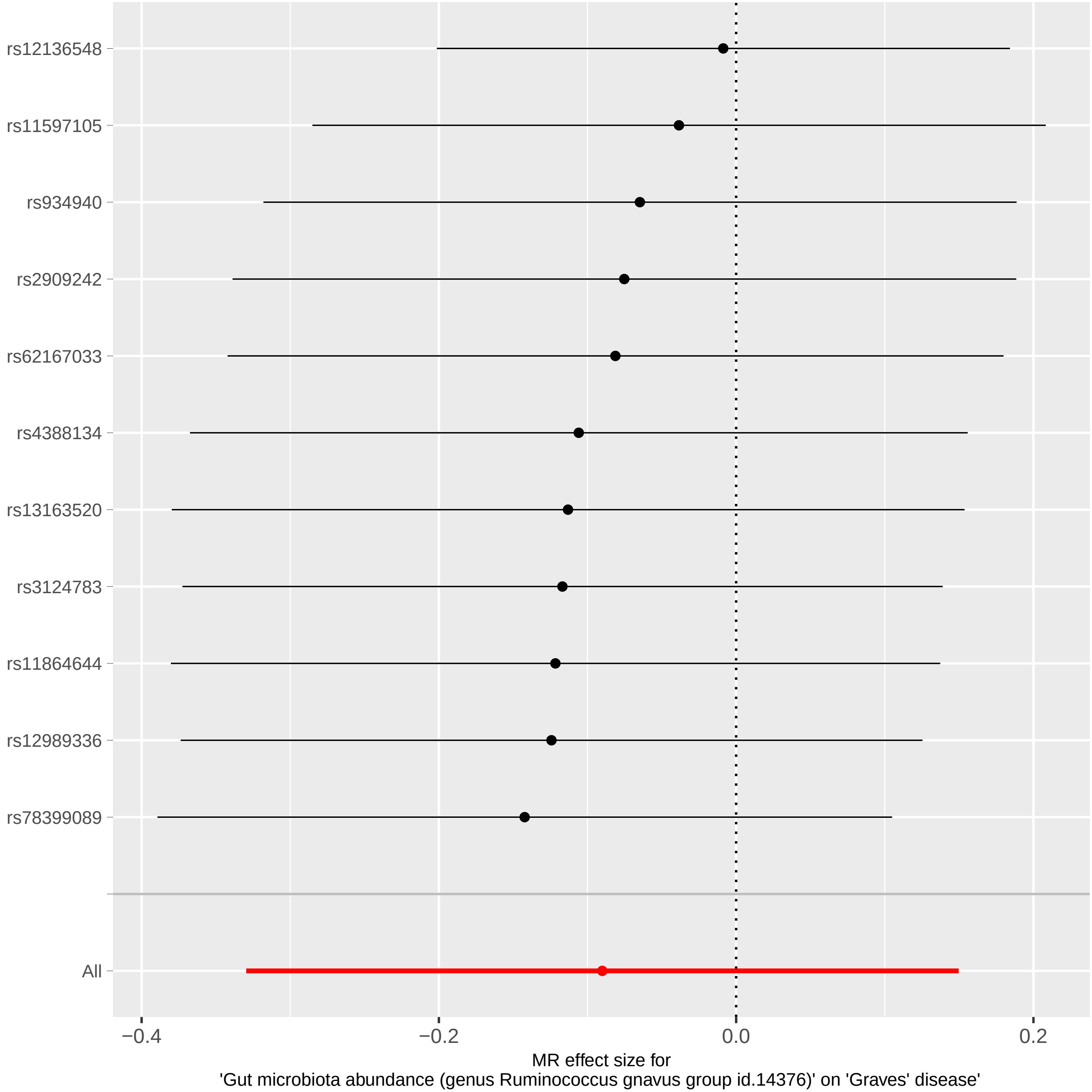

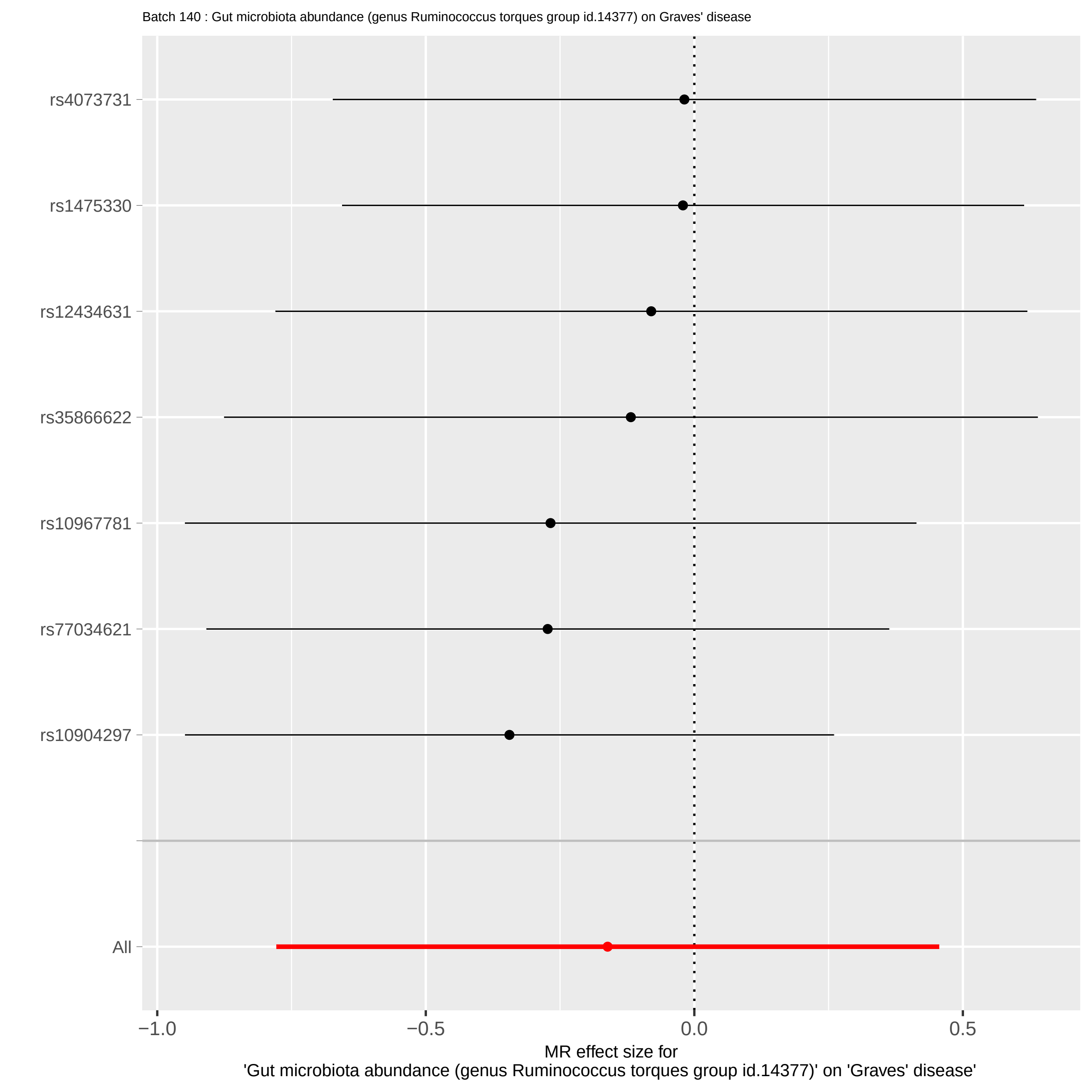

Batch 140 : Gut microbiota abundance (genus Ruminococcus torques group id.14377) on Graves' disease
rs4073731
rs1475330
rs12434631
rs35866622
rs10967781
rs77034621
rs10904297
All
−1.0
−0.5
0.0
0.5
MR effect size for
'Gut microbiota abundance (genus Ruminococcus torques group id.14377)' on 'Graves' disease'

Batch 141 : Gut microbiota abundance (genus Sellimonas id.14369) on Graves' disease

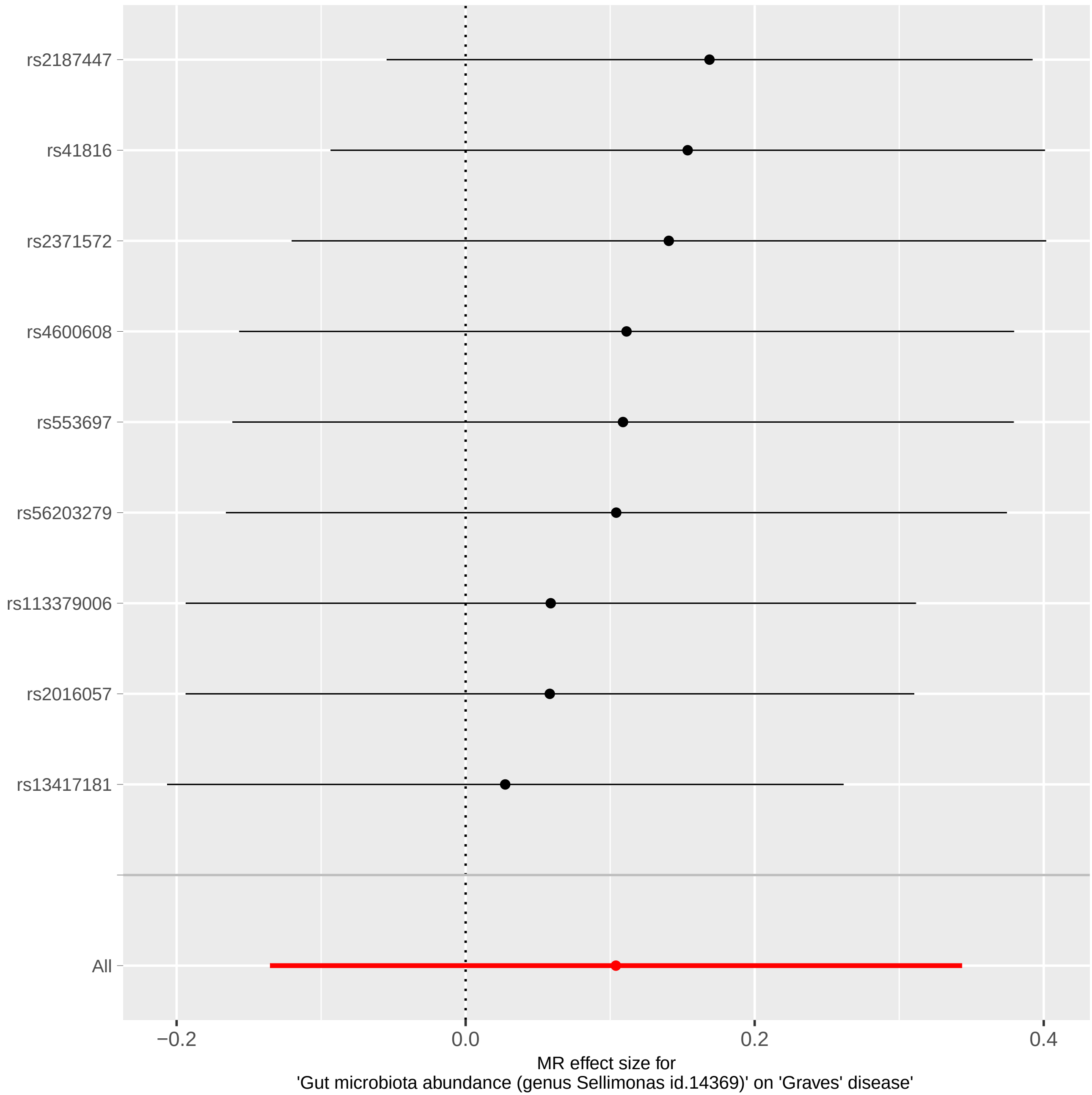

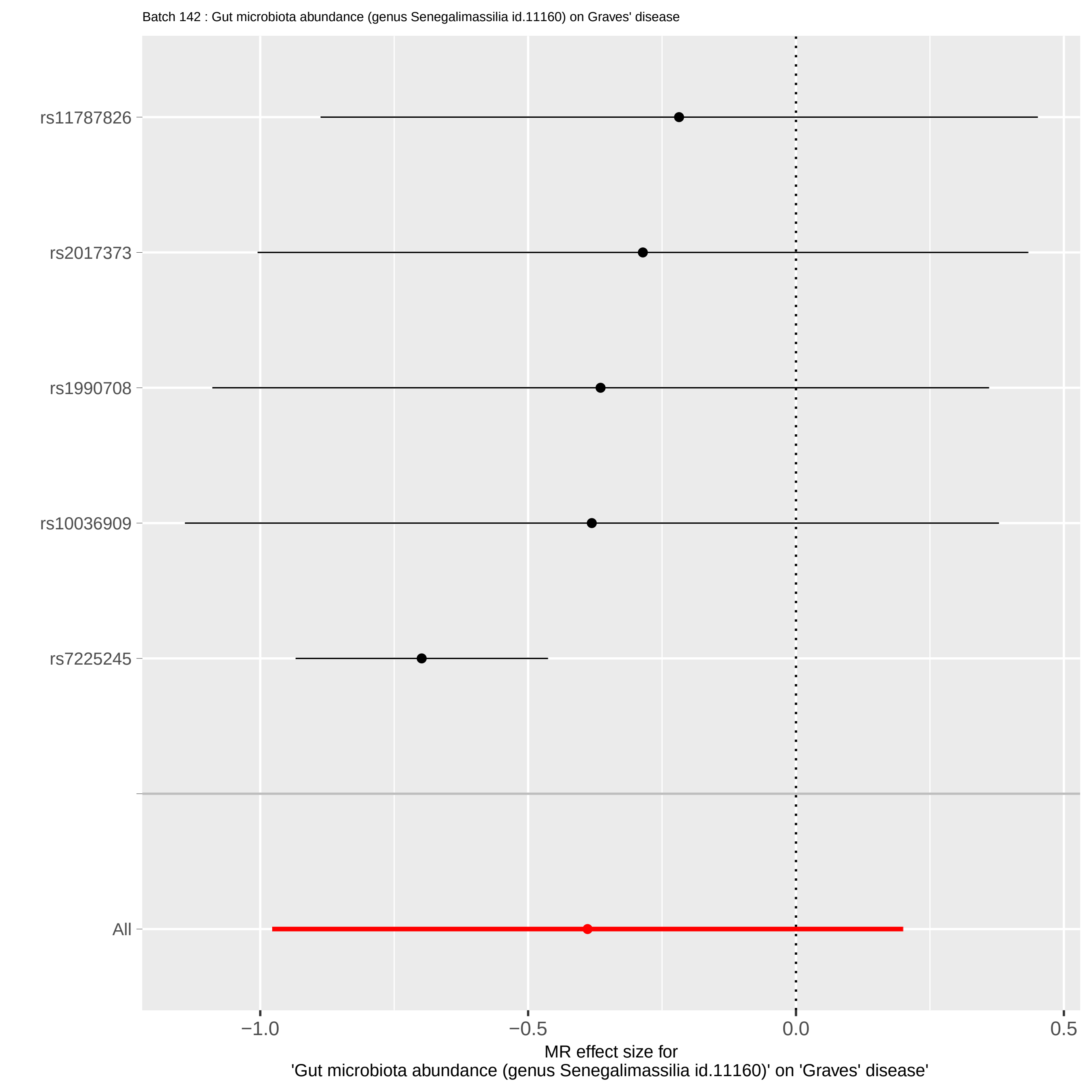

Batch 142 : Gut microbiota abundance (genus Senegalimassilia id.11160) on Graves' disease
rs11787826
rs2017373
rs1990708
rs10036909
rs7225245
All
−1.0
−0.5
0.0
0.5
MR effect size for
'Gut microbiota abundance (genus Senegalimassilia id.11160)' on 'Graves' disease

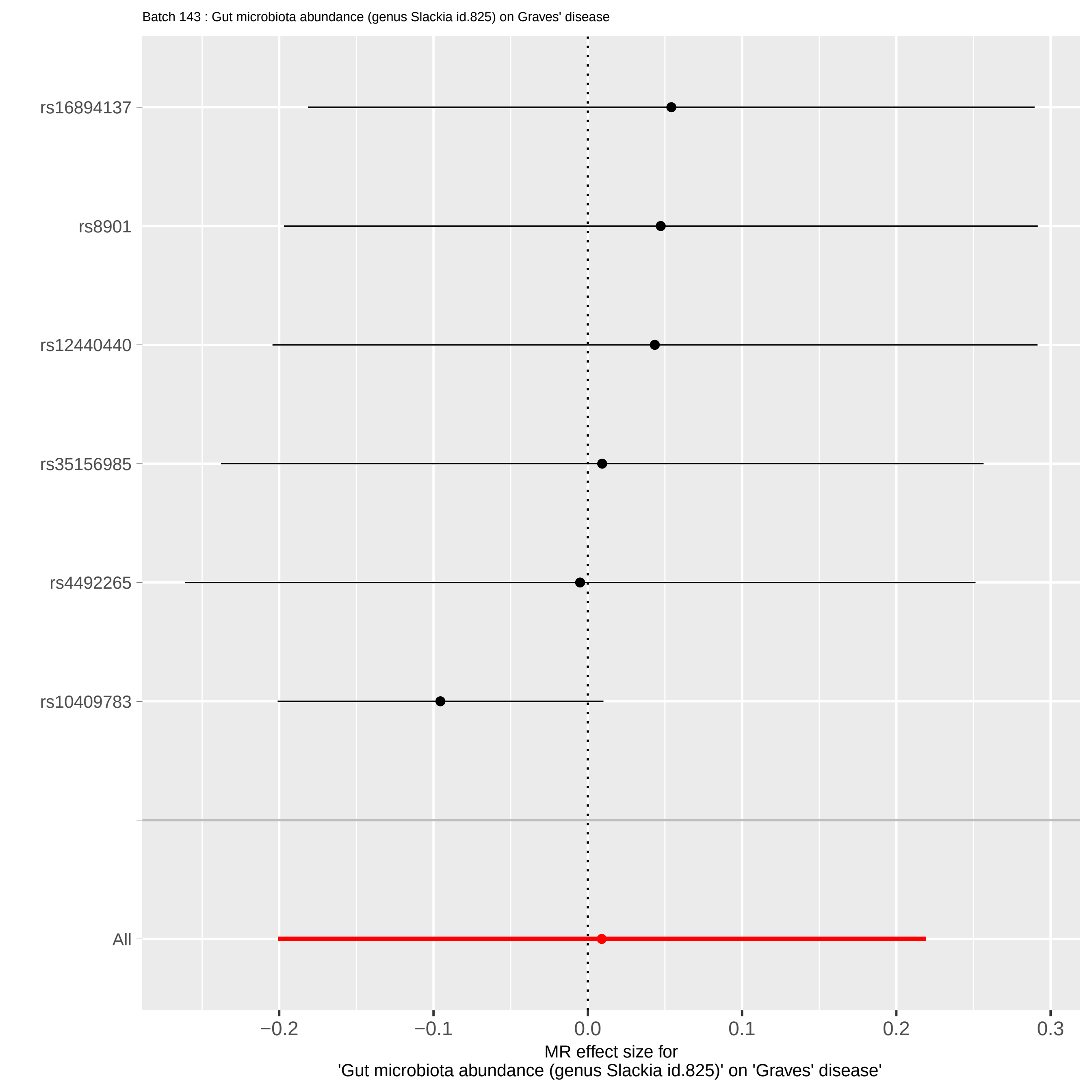

Batch 143 : Gut microbiota abundance (genus Slackia id.825) on Graves' disease
rs16894137
rs8901
rs12440440
rs35156985
rs4492265
rs10409783
All
−0.2
−0.1
0.0
0.1
0.2
0.3
MR effect size for
'Gut microbiota abundance (genus Slackia id.825)' on 'Graves' disease'

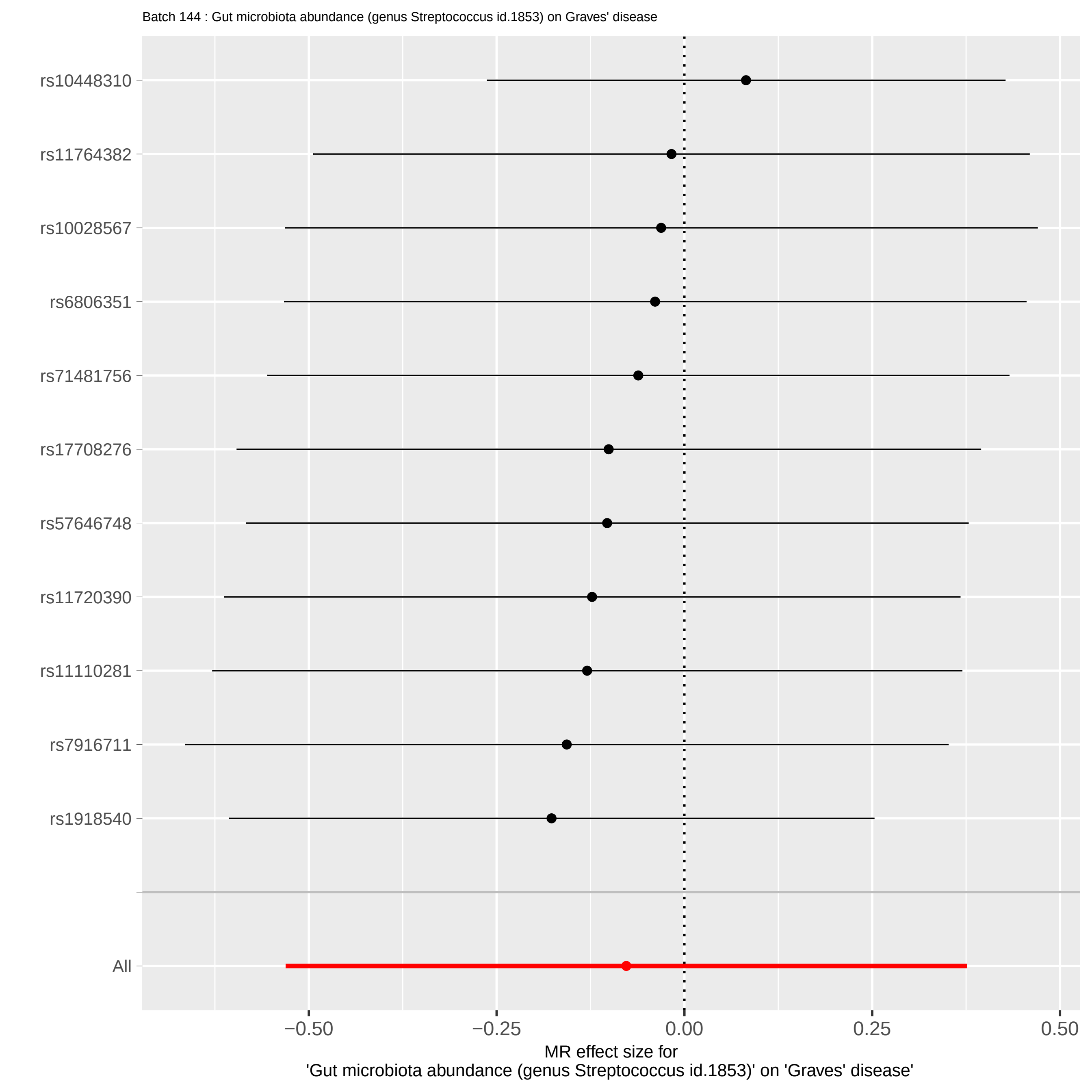

Batch 144 : Gut microbiota abundance (genus Streptococcus id.1853) on Graves' disease
rs10448310
rs11764382
rs10028567
rs6806351
rs71481756
rs17708276
rs57646748
rs11720390
rs11110281
rs7916711
rs1918540
All
−0.50
−0.25
0.00
0.25
0.50
MR effect size for
'Gut microbiota abundance (genus Streptococcus id.1853)' on 'Graves' disease'

Batch 145 : Gut microbiota abundance (genus Subdoligranulum id.2070) on Graves' disease

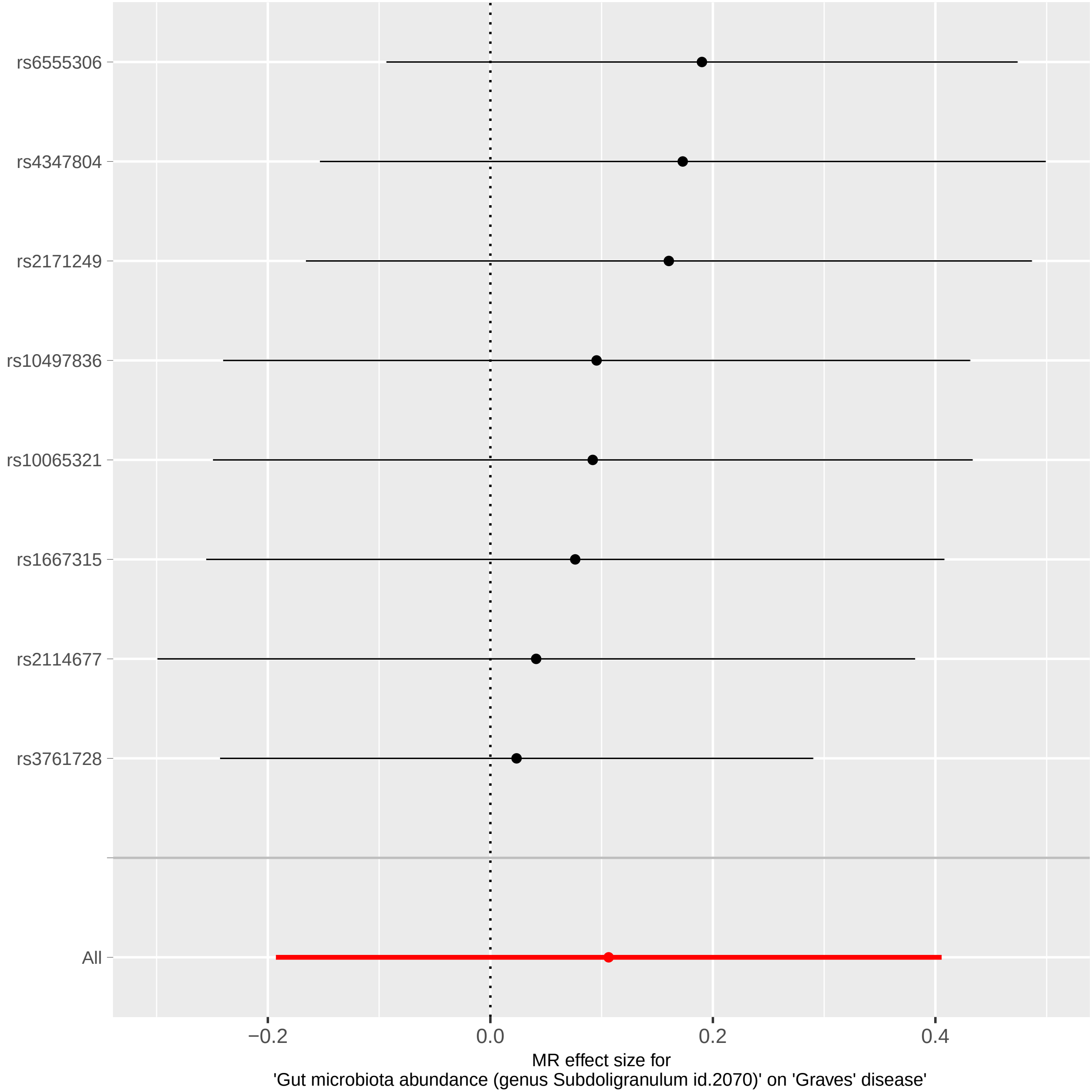

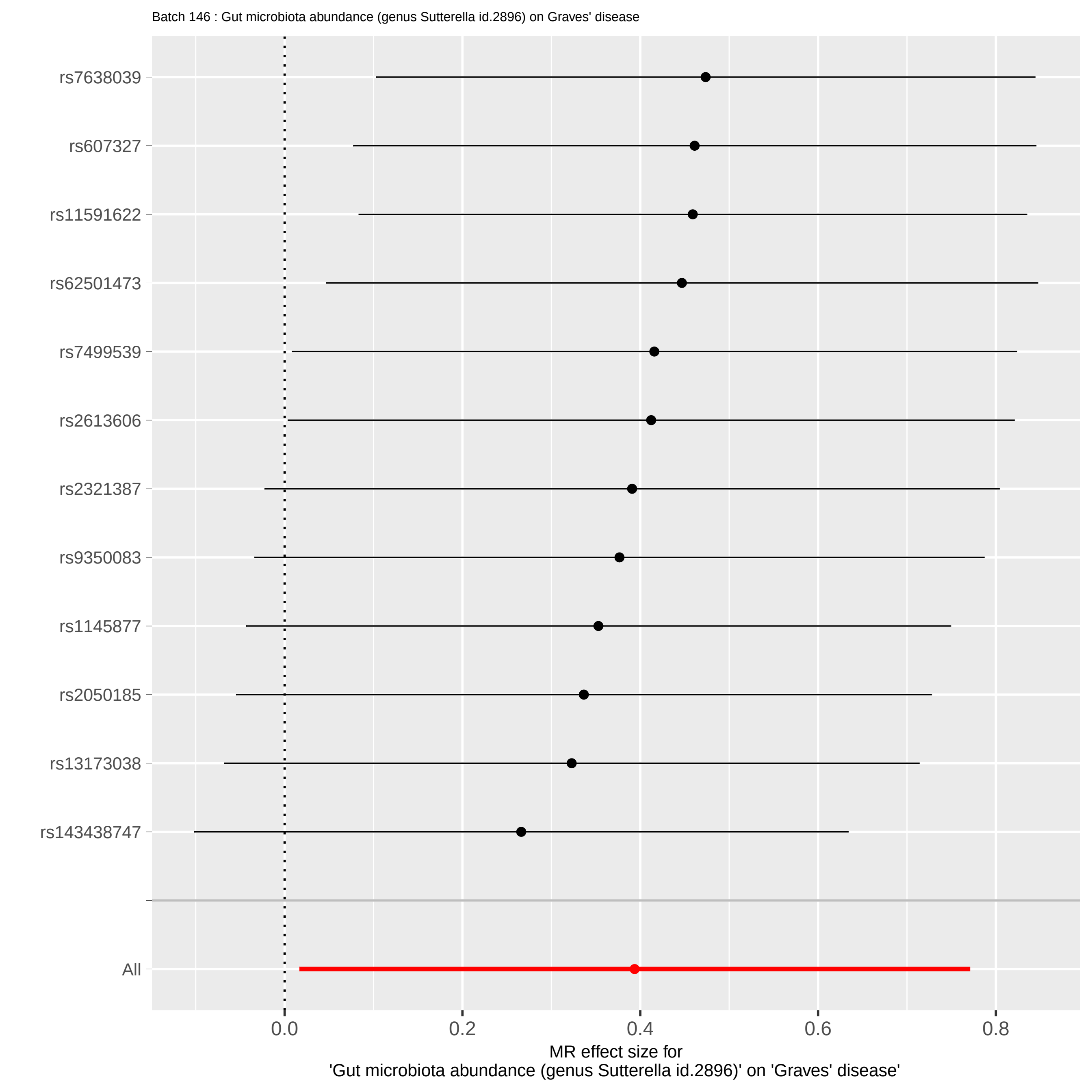
Batch 146 : Gut microbiota abundance (genus Sutterella id.2896) on Graves' disease
rs7638039
rs607327
rs11591622
rs62501473
rs7499539
rs2613606
rs2321387
rs9350083
rs1145877
rs2050185
rs13173038
rs143438747
All
0.0
0.2
0.4
0.6
0.8
MR effect size for
'Gut microbiota abundance (genus Sutterella id.2896)' on 'Graves' disease'

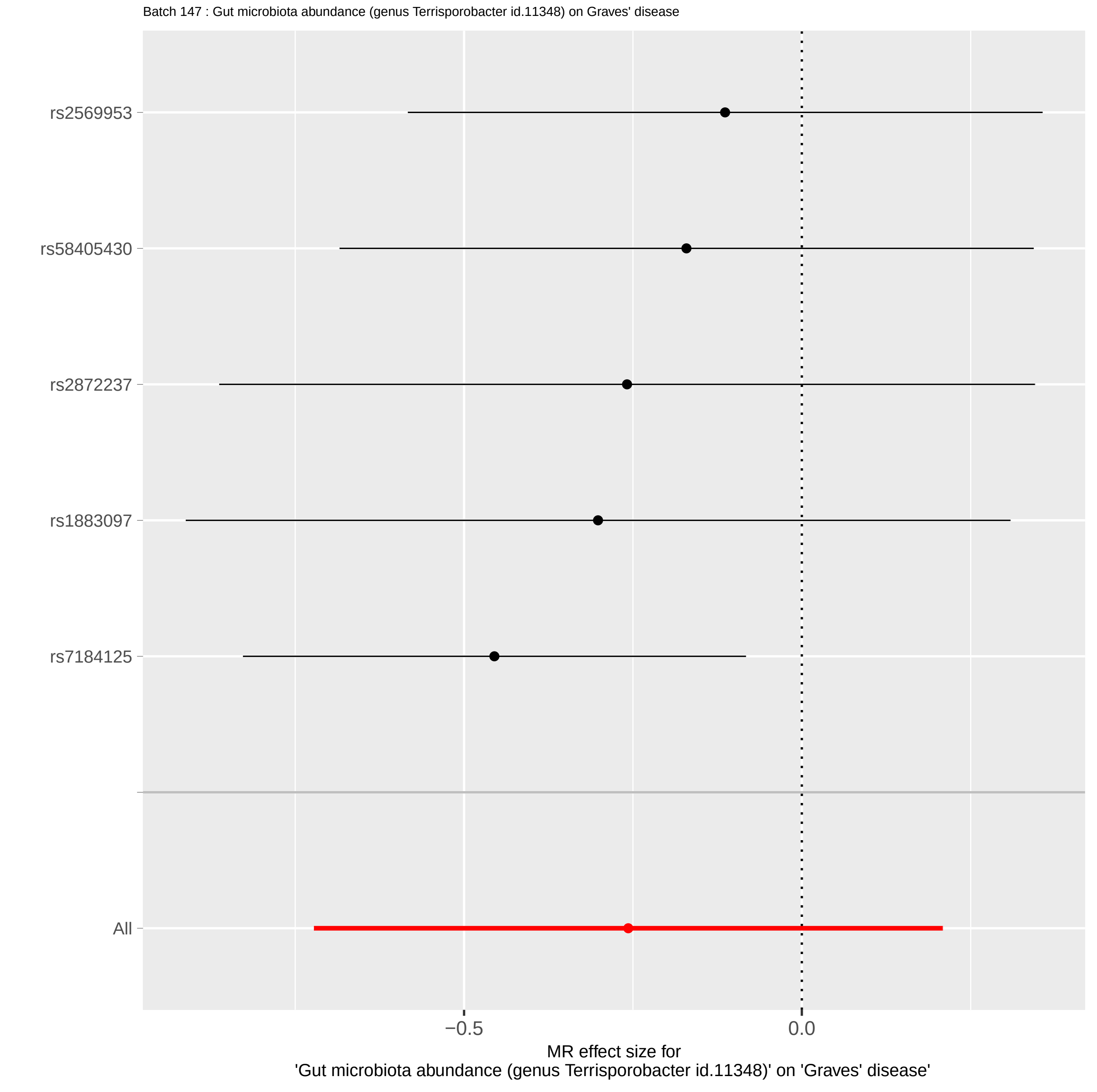
Batch 147 : Gut microbiota abundance (genus Terrisporobacter id.11348) on Graves' disease
rs2569953
rs58405430
rs2872237
rs1883097
rs7184125
All
−0.5
0.0
MR effect size for
'Gut microbiota abundance (genus Terrisporobacter id.11348)' on 'Graves' disease'

Batch 148 : Gut microbiota abundance (genus Turicibacter id.2162) on Graves' disease

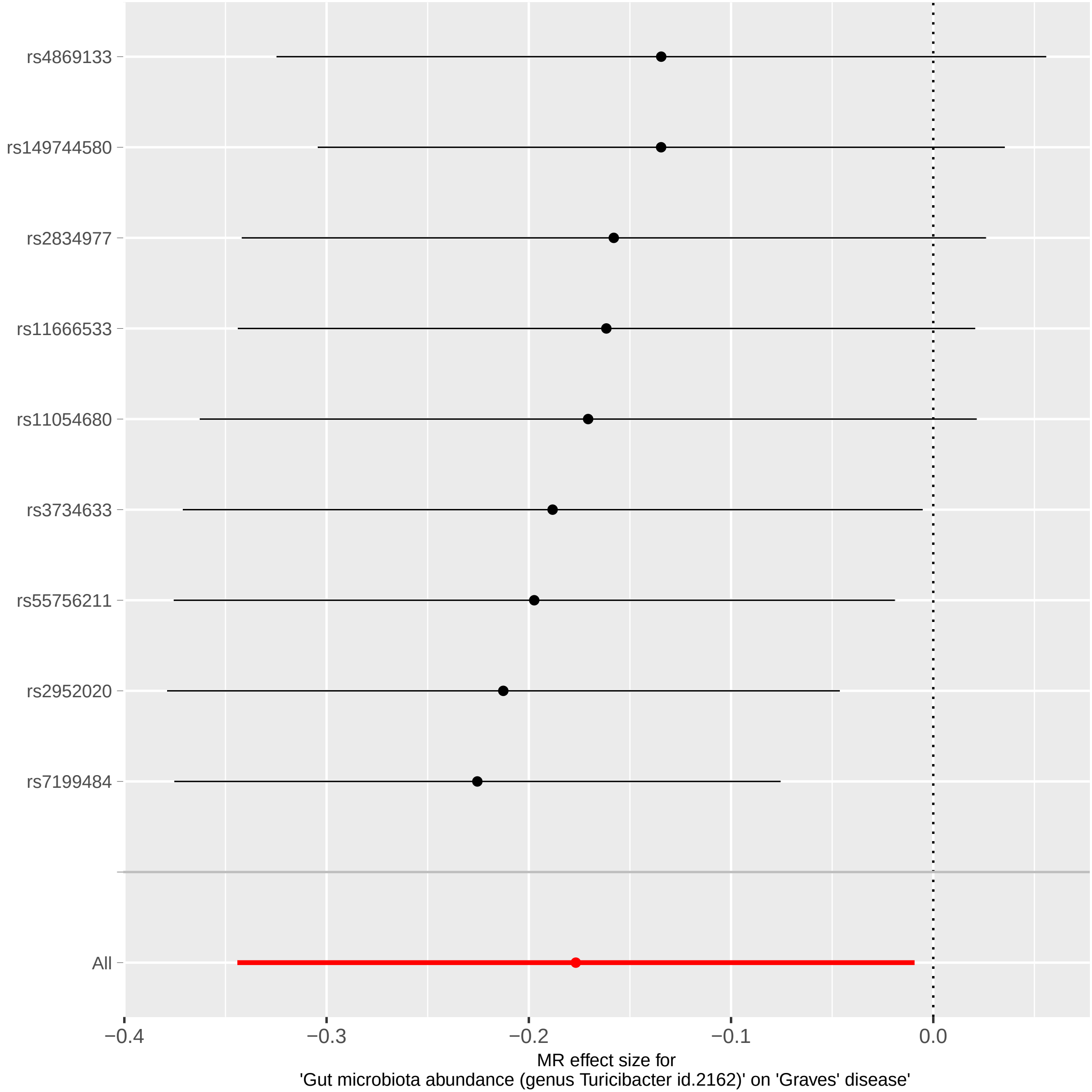

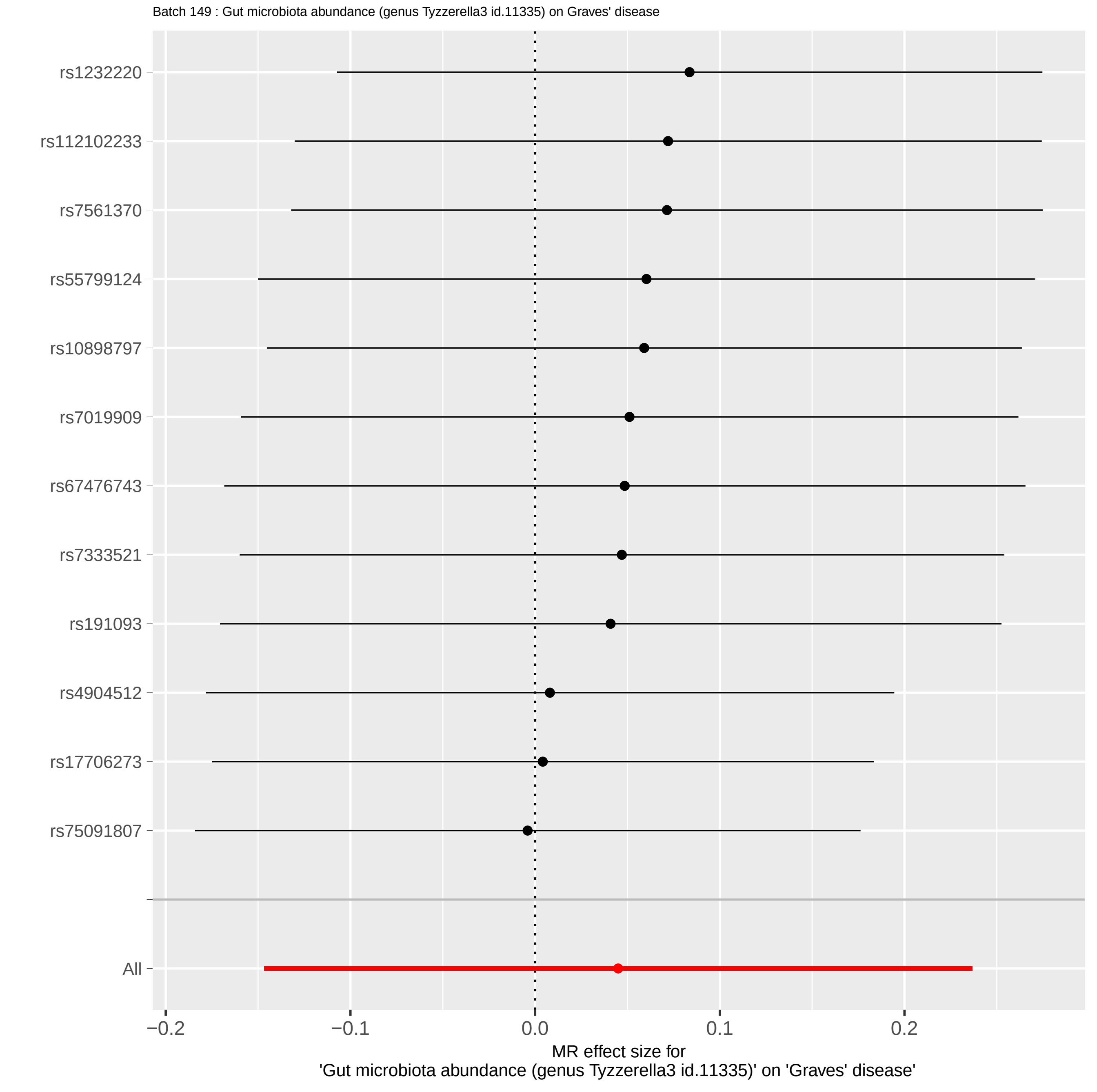
Batch 149 : Gut microbiota abundance (genus Tyzzerella3 id.11335) on Graves' disease
rs1232220
rs112102233
rs7561370
rs55799124
rs10898797
rs7019909
rs67476743
rs7333521
rs191093
rs4904512
rs17706273
rs75091807
All
−0.2
−0.1
0.0
0.1
0.2
MR effect size for
'Gut microbiota abundance (genus Tyzzerella3 id.11335)' on 'Graves' disease'

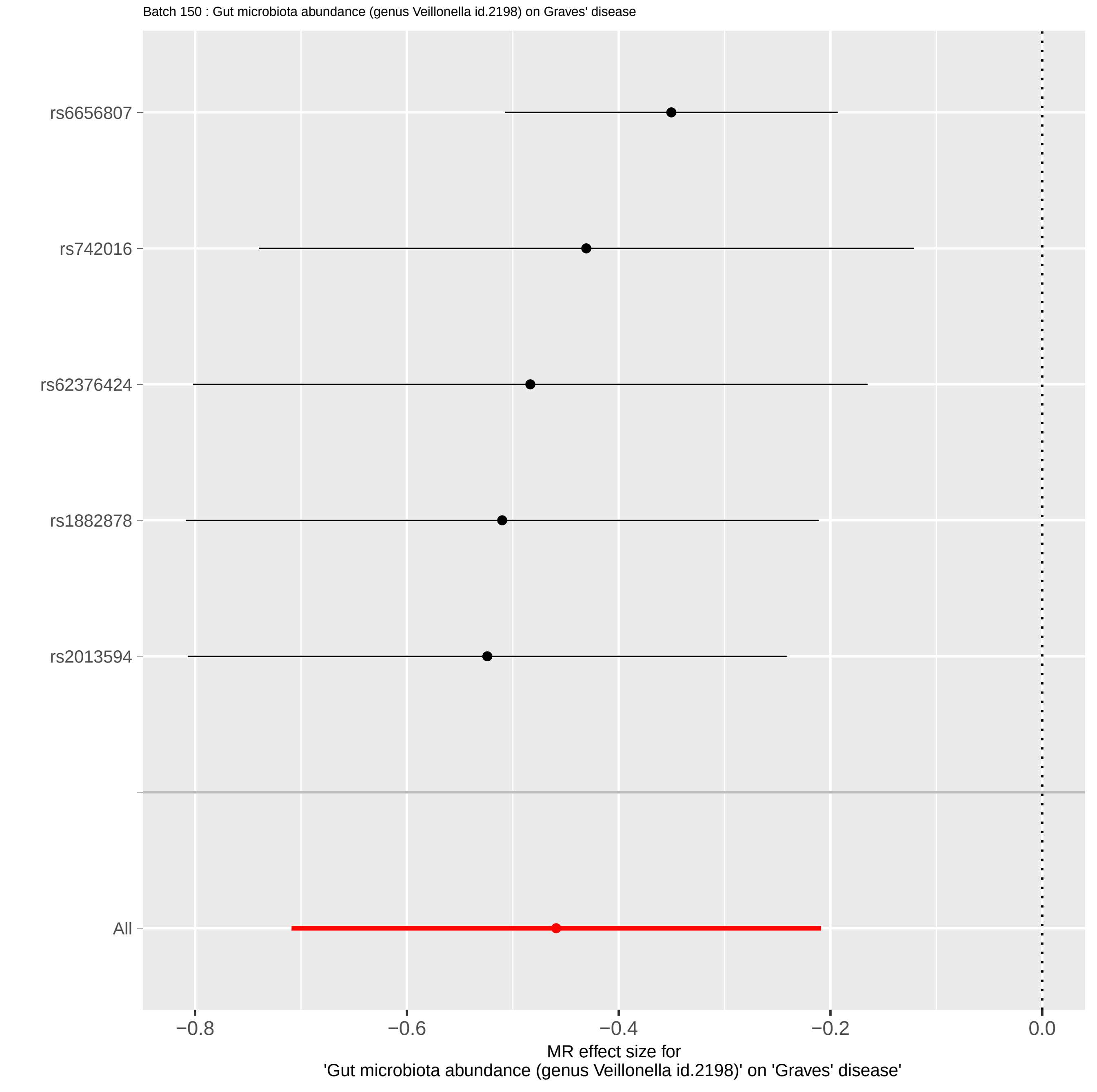
Batch 150 : Gut microbiota abundance (genus Veillonella id.2198) on Graves' disease
rs6656807
rs742016
rs62376424
rs1882878
rs2013594
All
−0.8
−0.6
−0.4
−0.2
0.0
MR effect size for
'Gut microbiota abundance (genus Veillonella id.2198)' on 'Graves' disease'
