# Supplementary material for: Bacteroidaceae, Bacteroides, and Veillonella: emerging protectors against Graves’ disease
Source: Front Cell Infect Microbiol. 2024 Feb 9;14:1288222. doi: 10.3389/fcimb.2024.1288222 (PMC10884117; doi:10.3389/fcimb.2024.1288222)

Batch 1 : Gut microbiota abundance (family Acidaminococcaceae id.2166) on Graves' disease

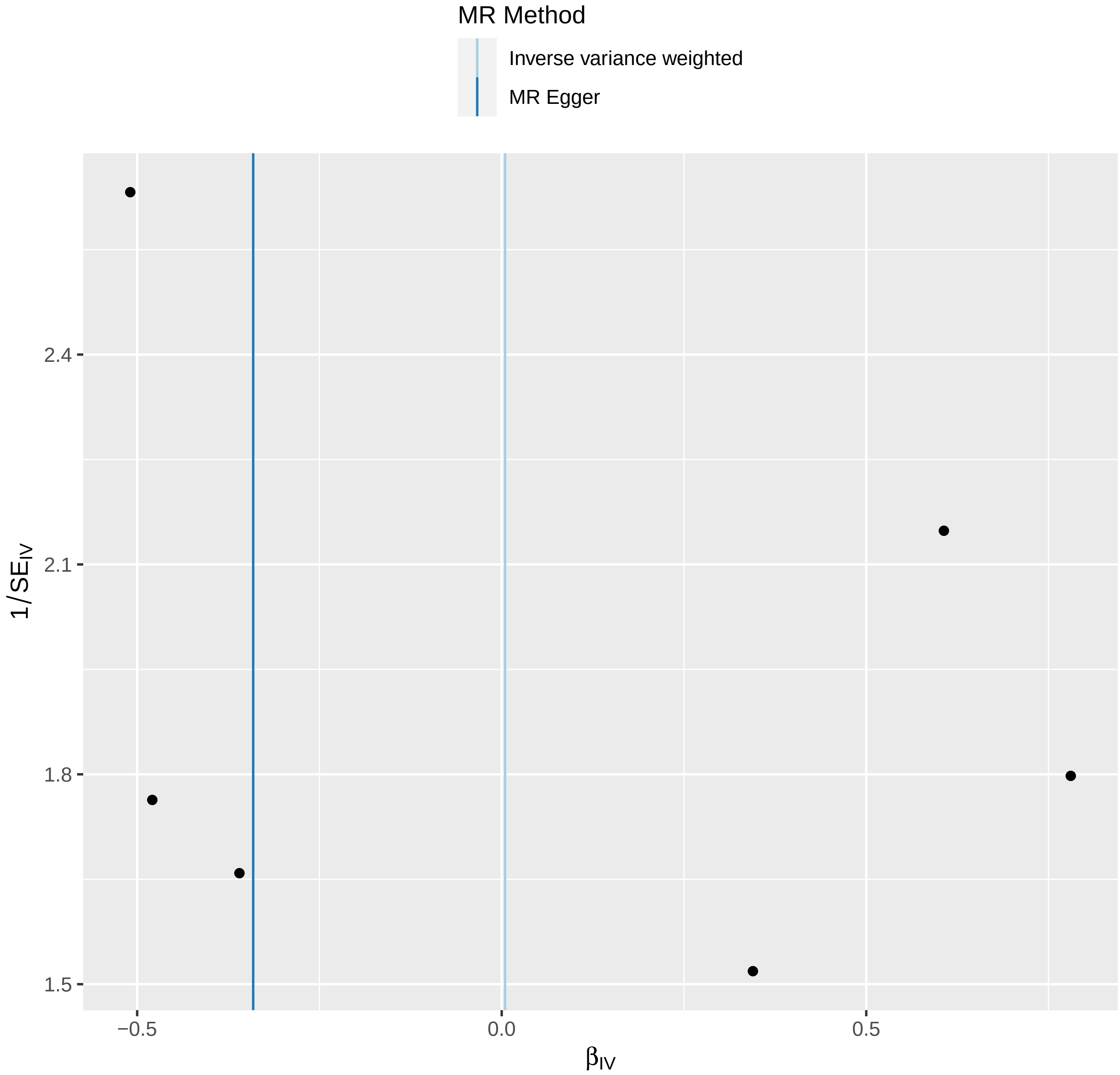

Batch 2 : Gut microbiota abundance (family Actinomycetaceae id.421) on Graves' disease

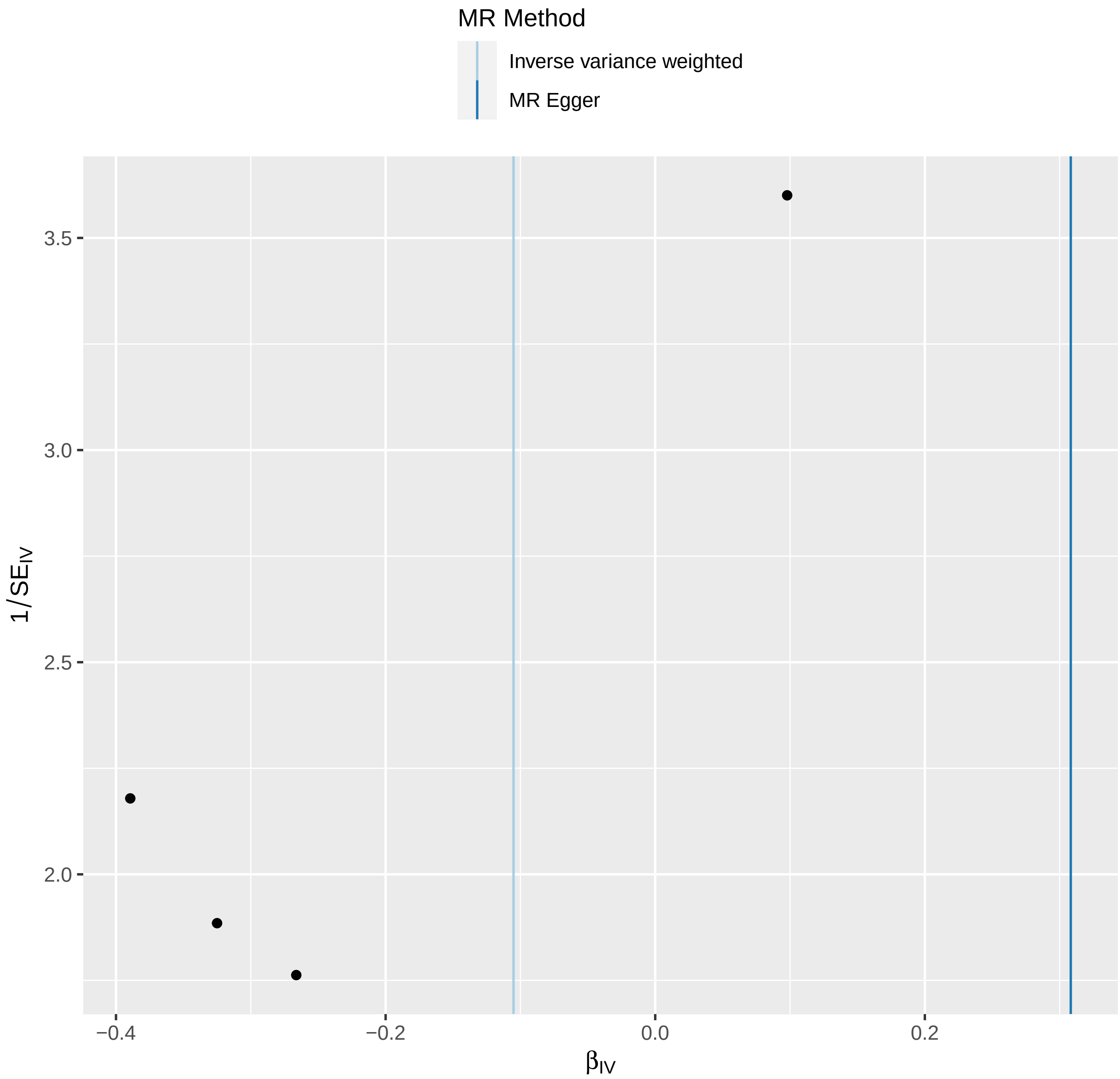

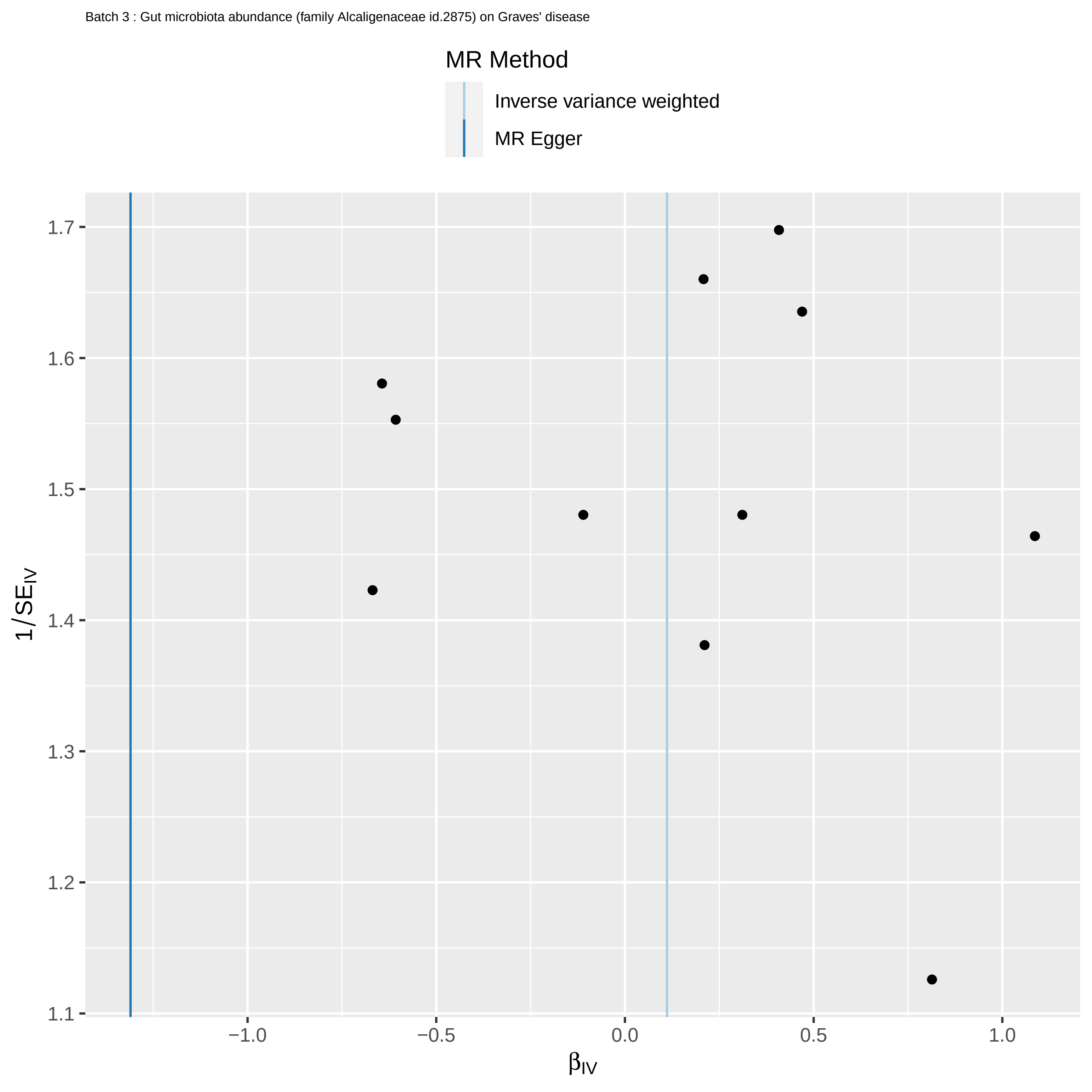
Batch 3 : Gut microbiota abundance (family Alcaligenaceae id.2875) on Graves' disease
MR Method
Inverse variance weighted
MR Egger
1/SE_IV
β_IV
1.7
1.6
1.5
1.4
1.3
1.2
1.1
−1.0
−0.5
0.0
0.5
1.0

Batch 4 : Gut microbiota abundance (family Bacteroidaceae id.917) on Graves' disease

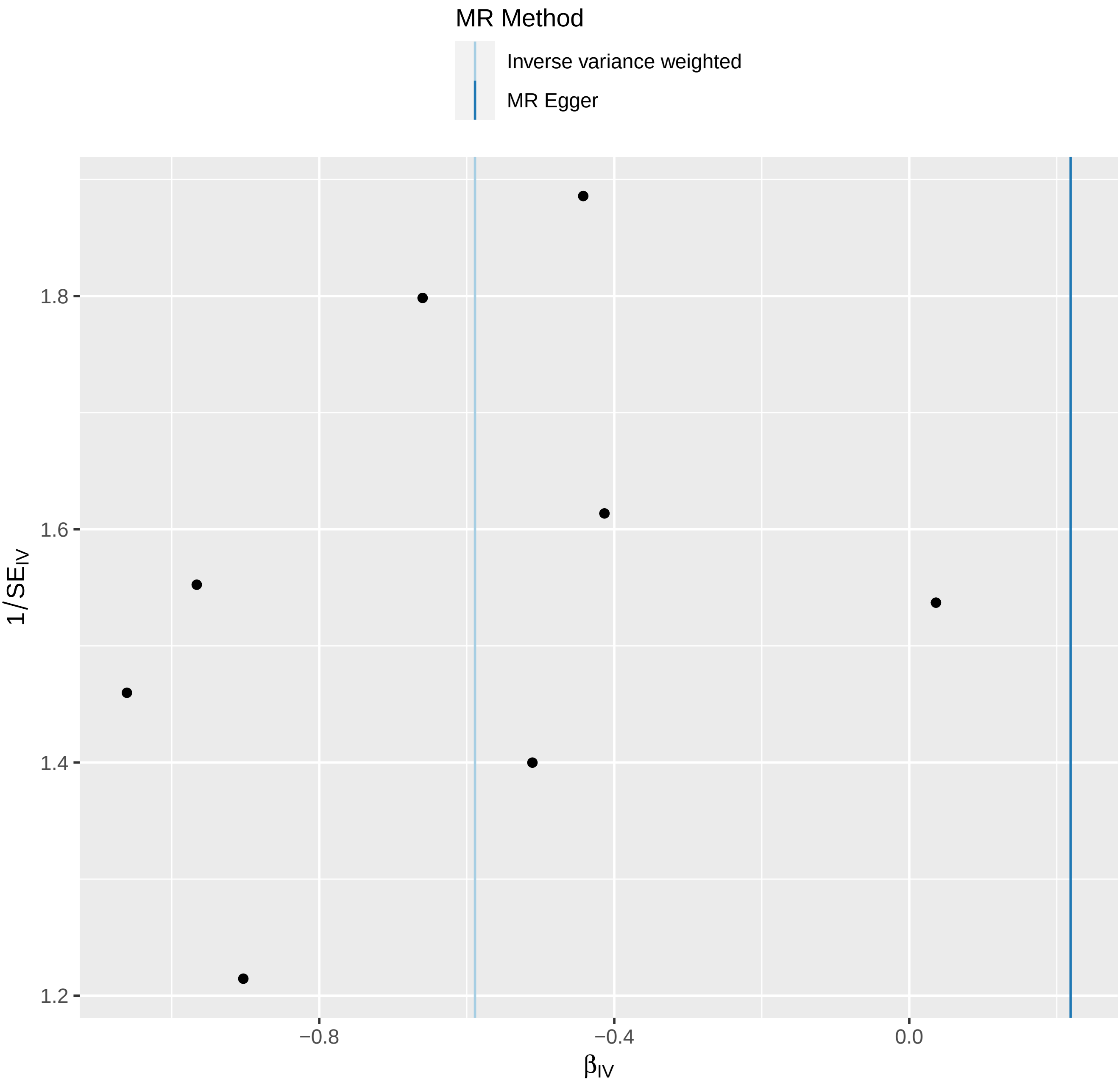

Batch 5 : Gut microbiota abundance (family Bacteroidales S24 7group id.11173) on Graves' disease

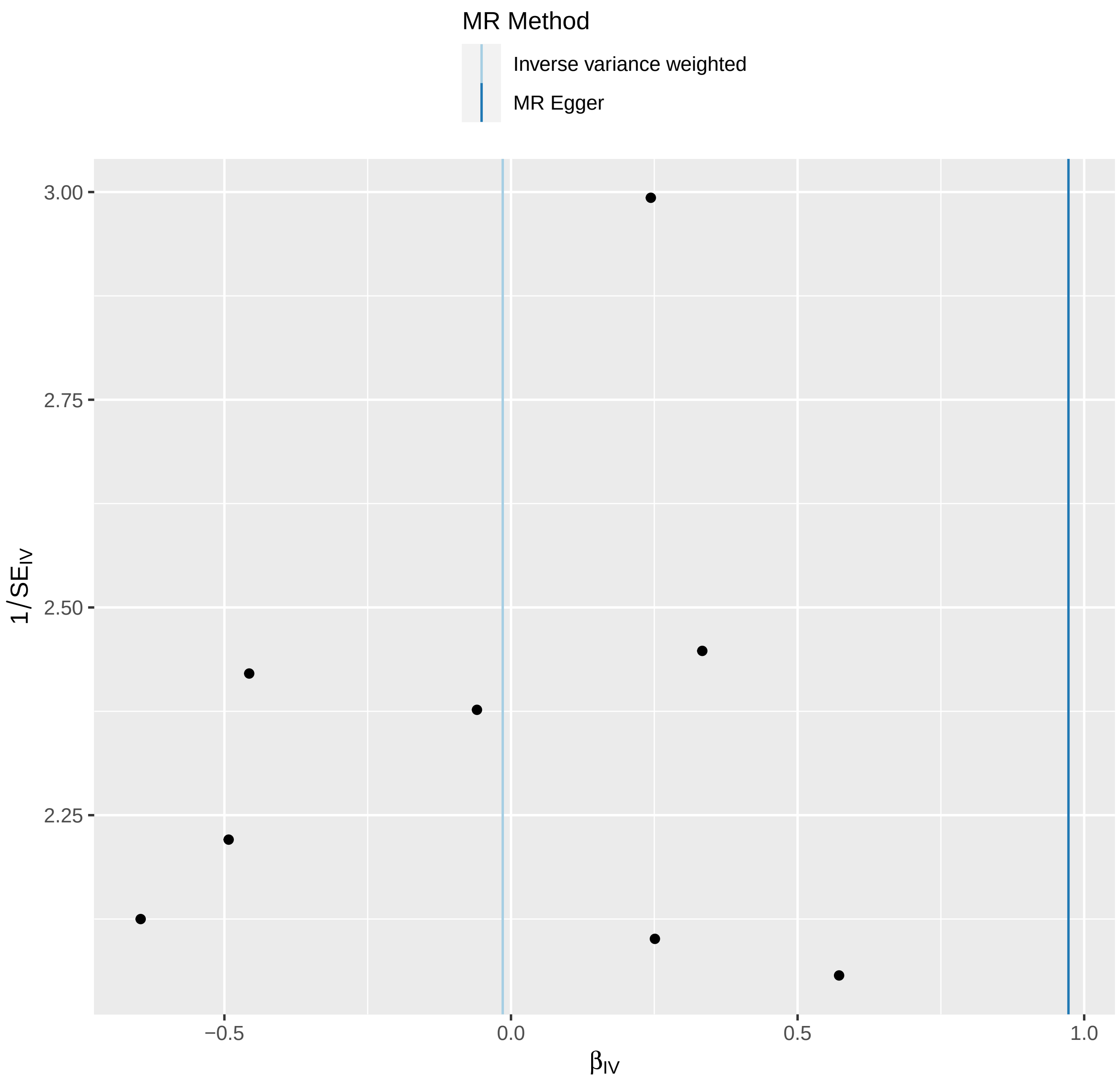

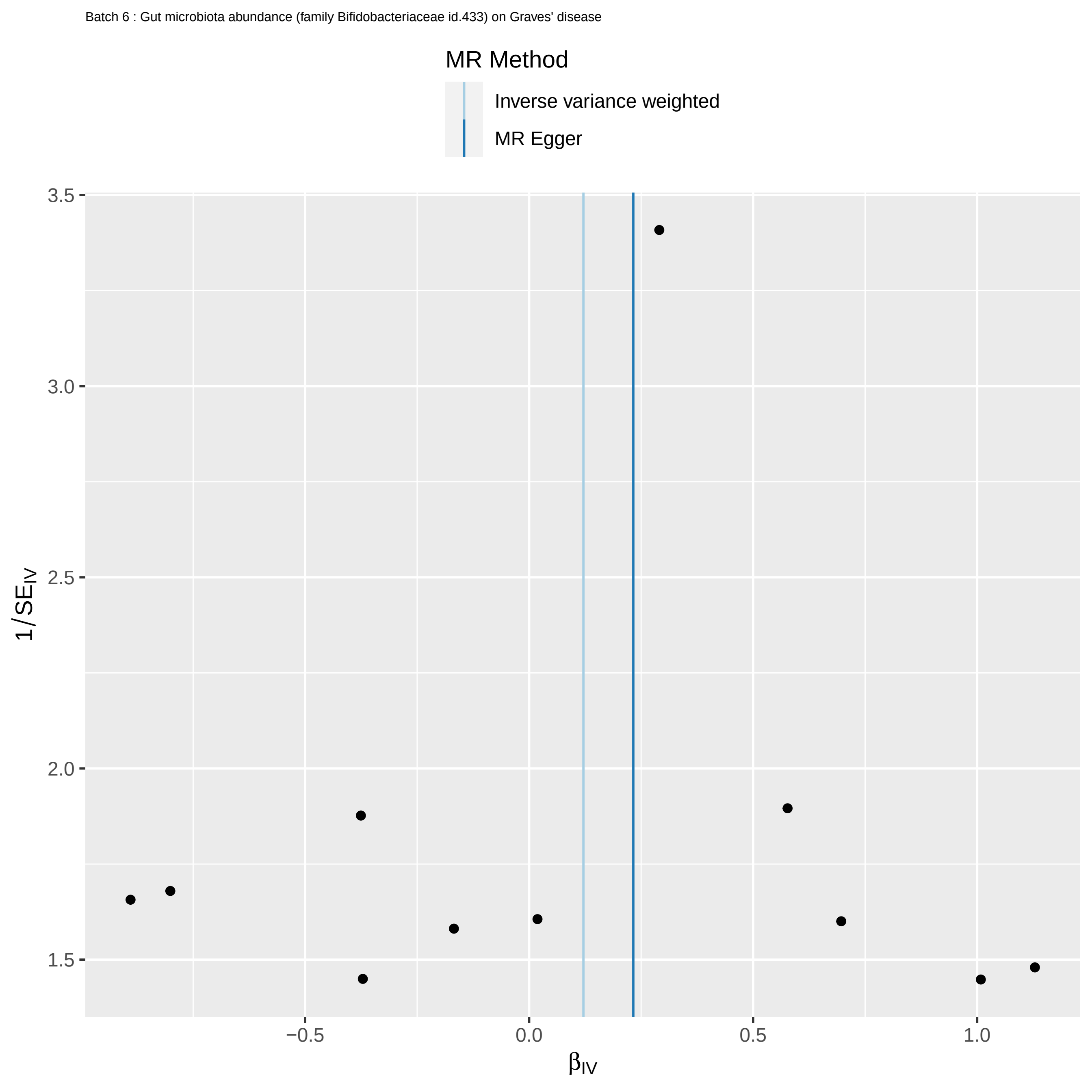
Batch 6 : Gut microbiota abundance (family Bifidobacteriaceae id.433) on Graves' disease
MR Method
Inverse variance weighted
MR Egger
3.5
3.0
2.5
2.0
1.5
1/SE$_{IV}$
−0.5
0.0
0.5
1.0
β_{IV}

Batch 8 : Gut microbiota abundance (family Clostridiaceae1 id.1869) on Graves' disease

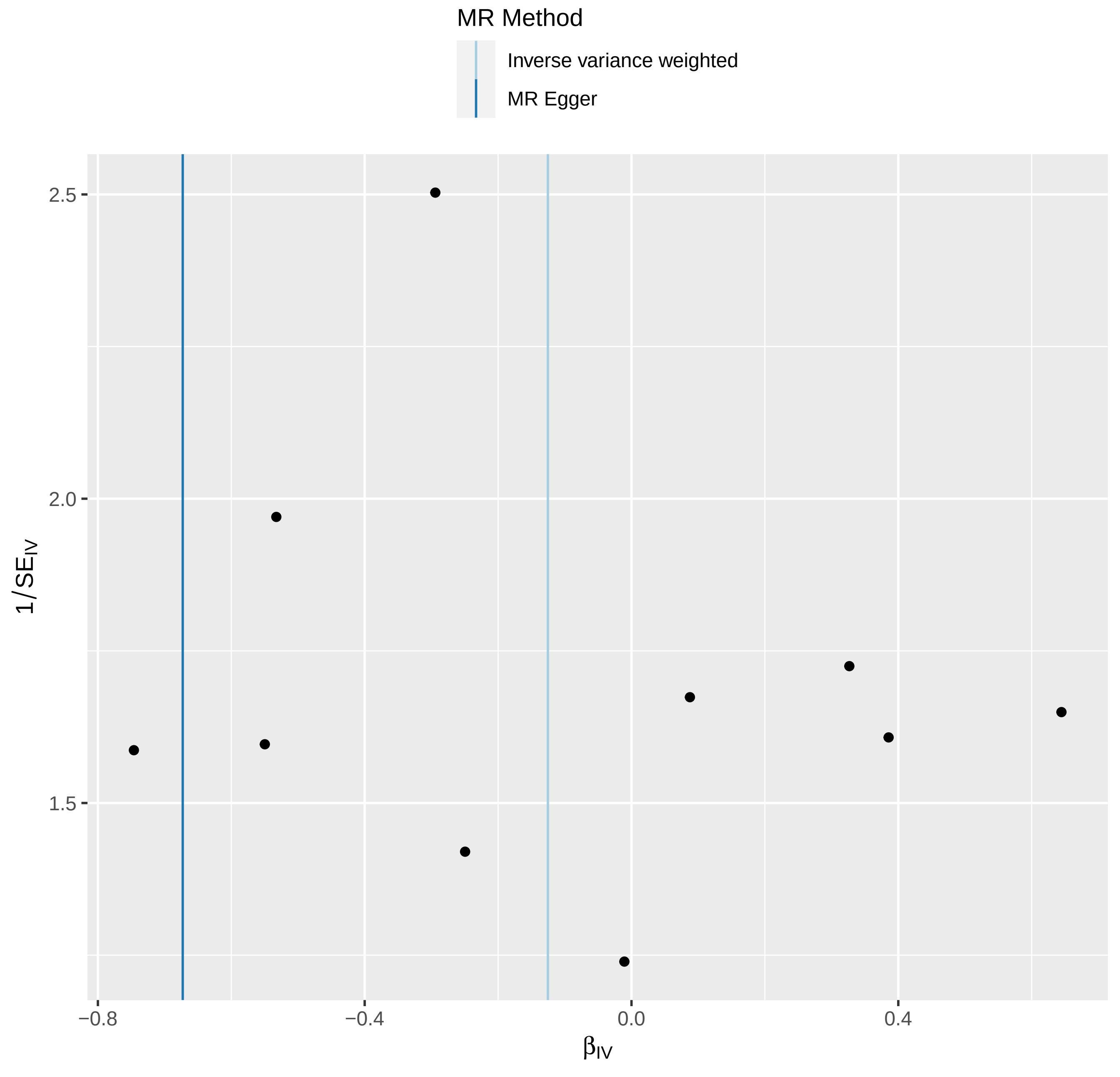

Batch 9 : Gut microbiota abundance (family Clostridiales vadin BB60 group id.11286) on Graves' disease

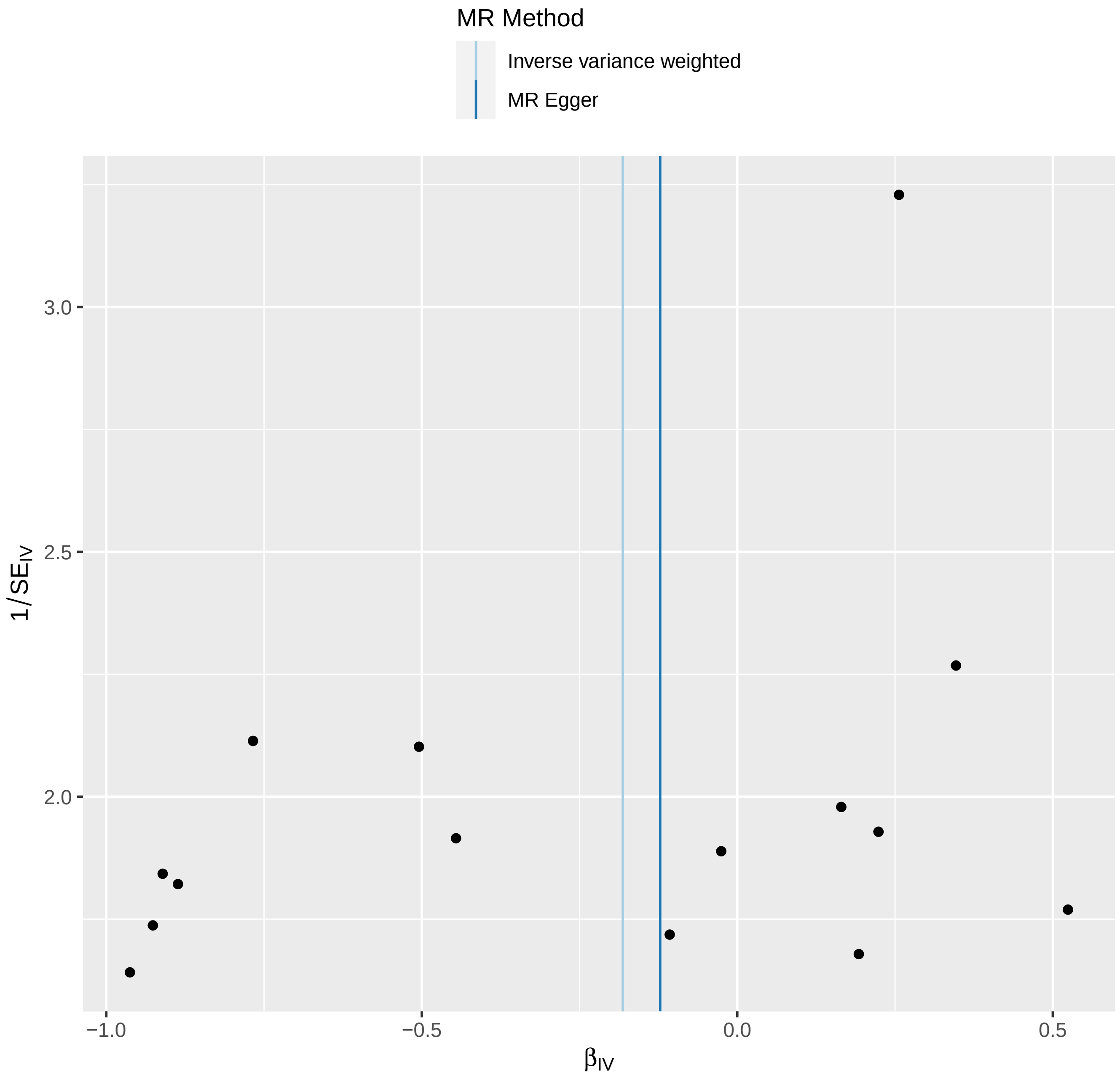

Batch 10 : Gut microbiota abundance (family Coriobacteriaceae id.811) on Graves' disease

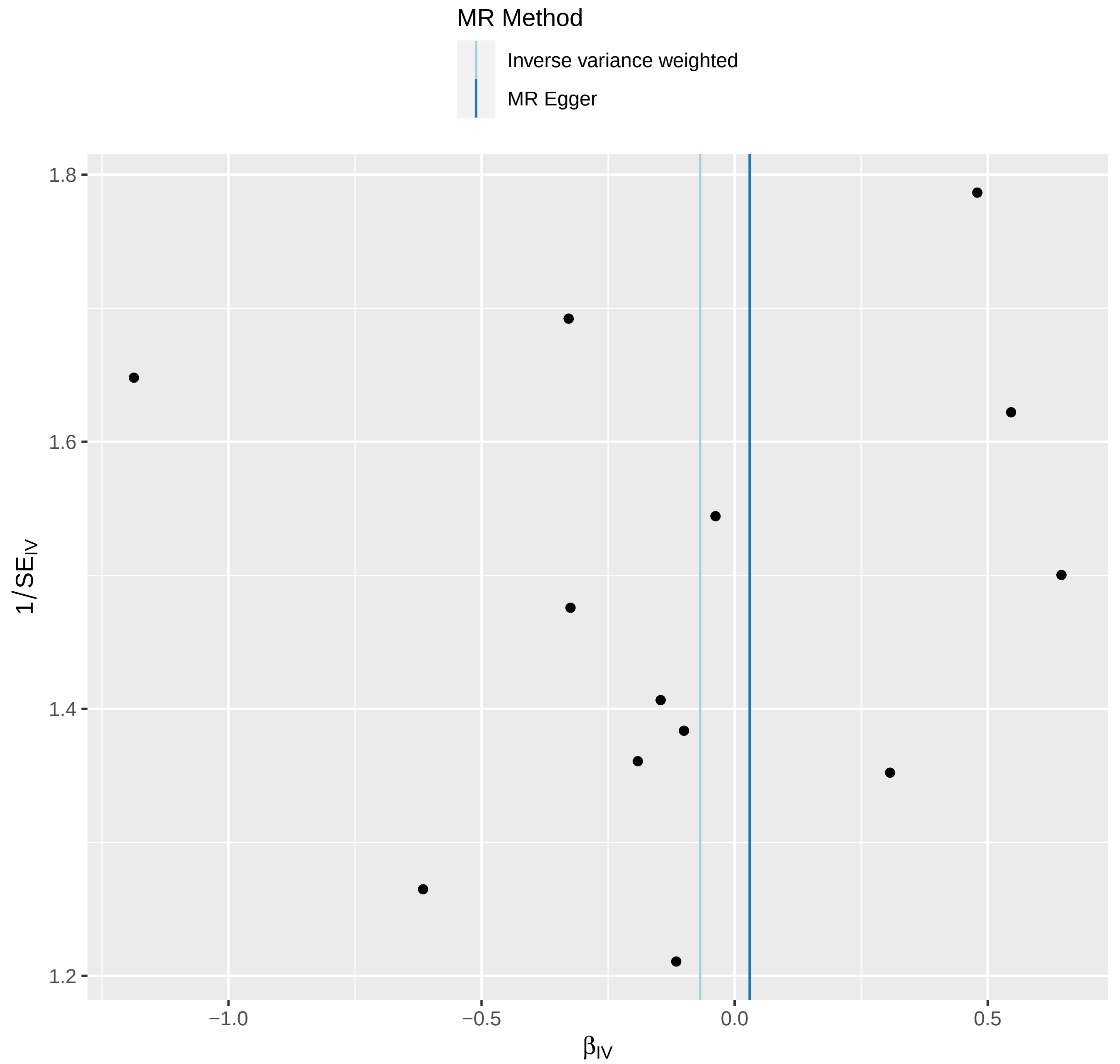

Batch 11 : Gut microbiota abundance (family Defluviitaleaceae id.1924) on Graves' disease

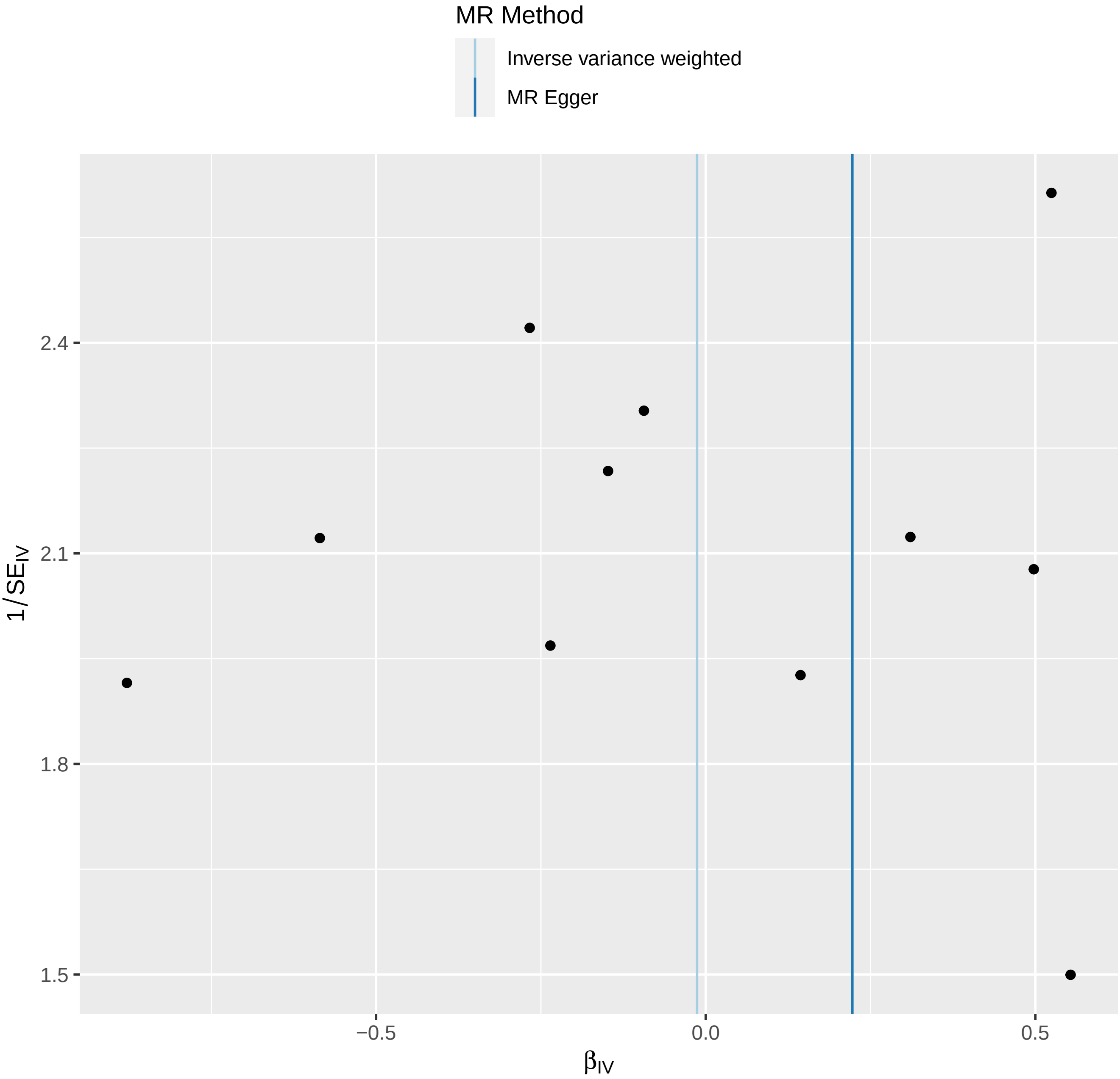

Batch 12 : Gut microbiota abundance (family Desulfovibrionaceae id.3169) on Graves' disease

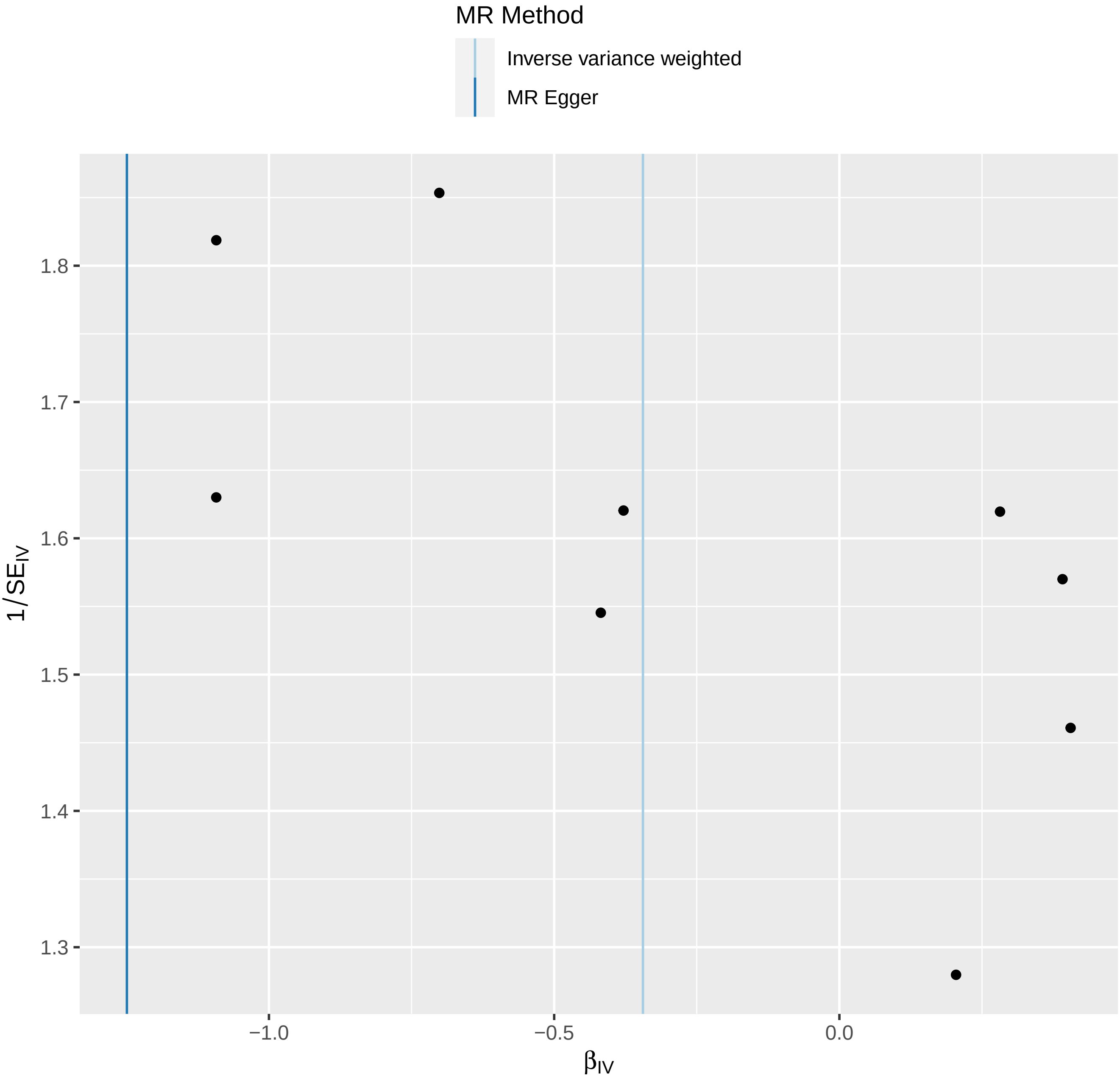

Batch 13 : Gut microbiota abundance (family Enterobacteriaceae id.3469) on Graves' disease

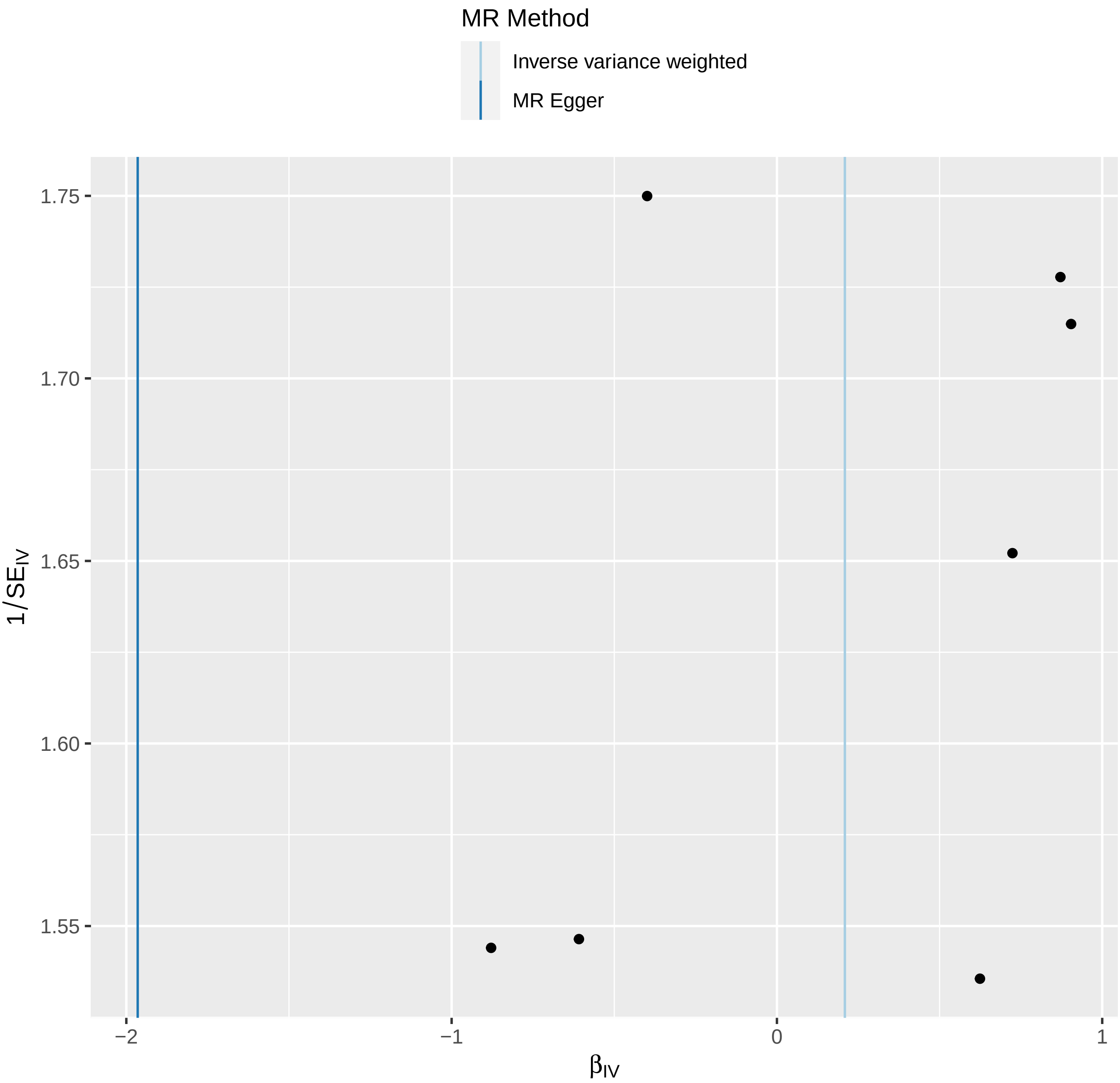

Batch 14 : Gut microbiota abundance (family Erysipelotrichaceae id.2149) on Graves' disease

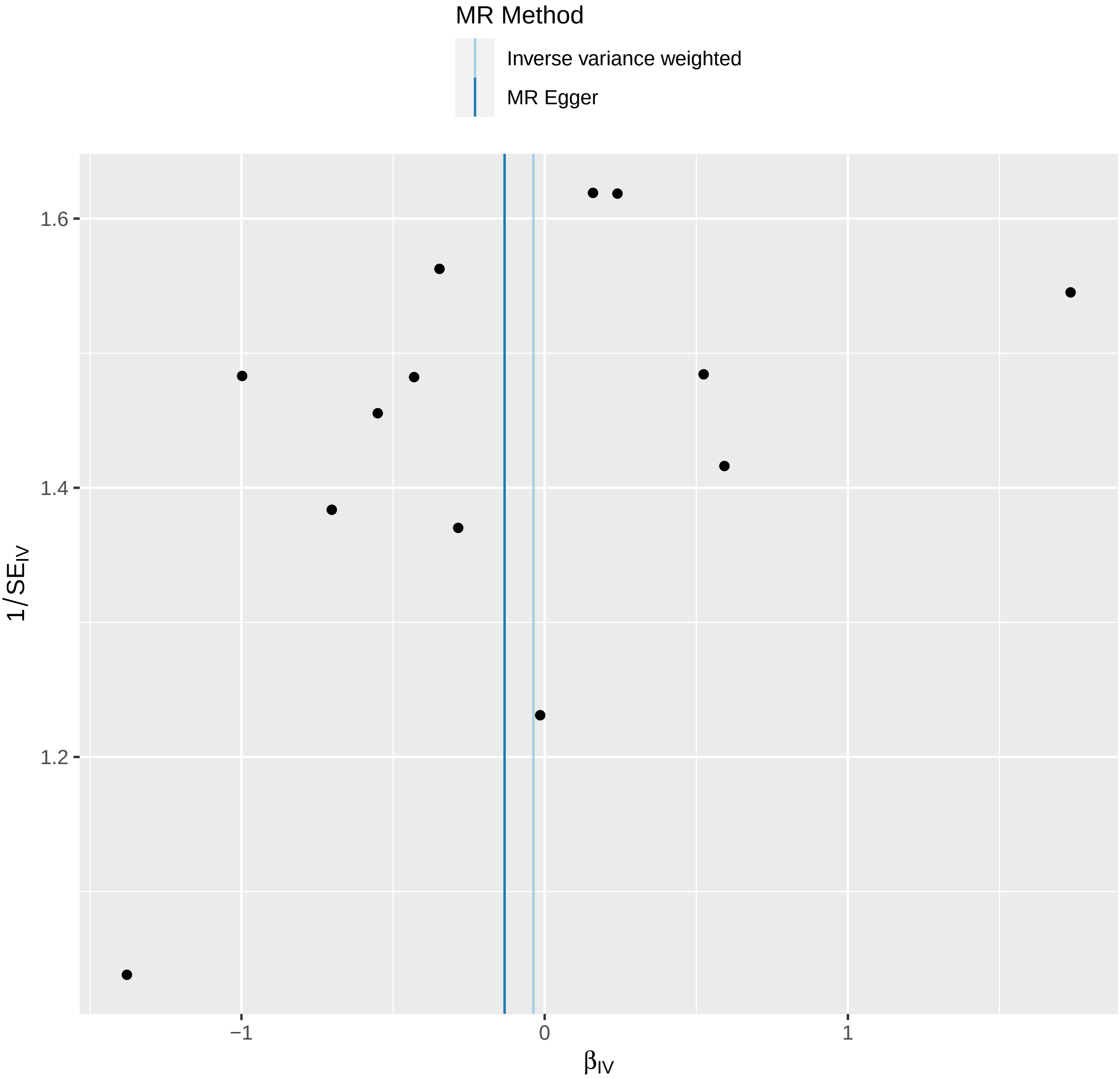

Batch 15 : Gut microbiota abundance (family Family XI id.1936) on Graves' disease

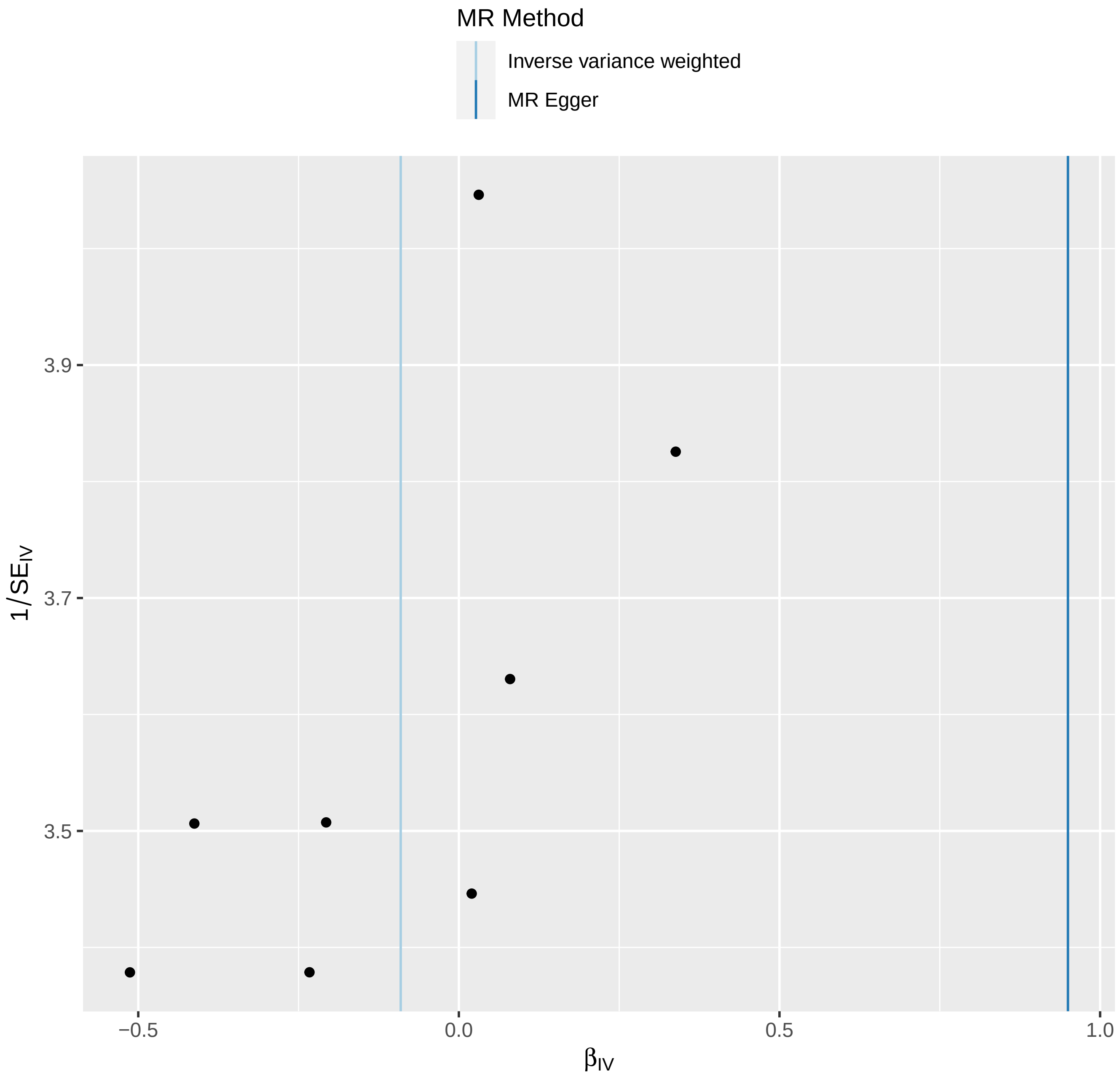

Batch 16 : Gut microbiota abundance (family Family XIII id.1957) on Graves' disease

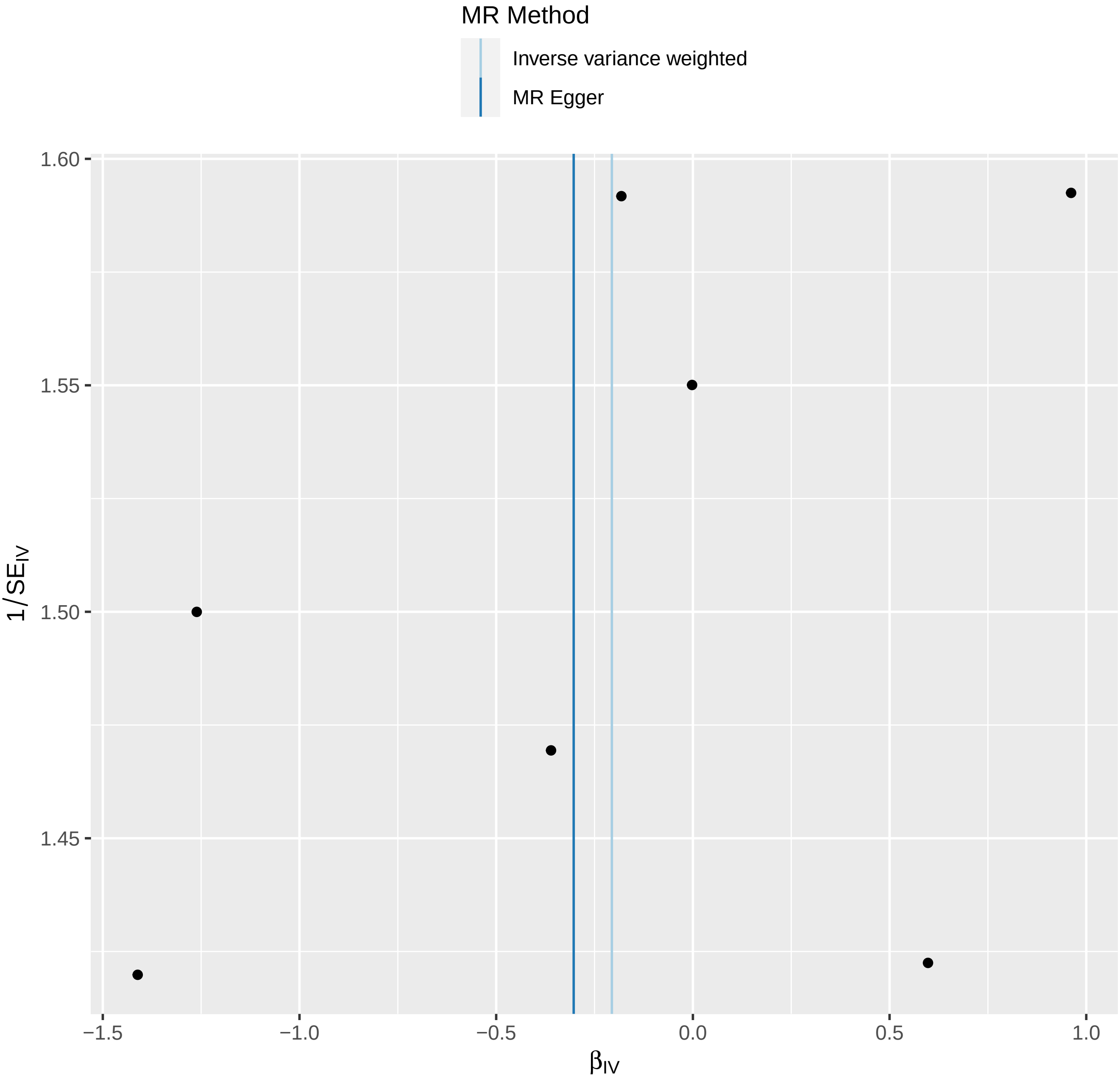

Batch 17 : Gut microbiota abundance (family Lachnospiraceae id.1987) on Graves' disease

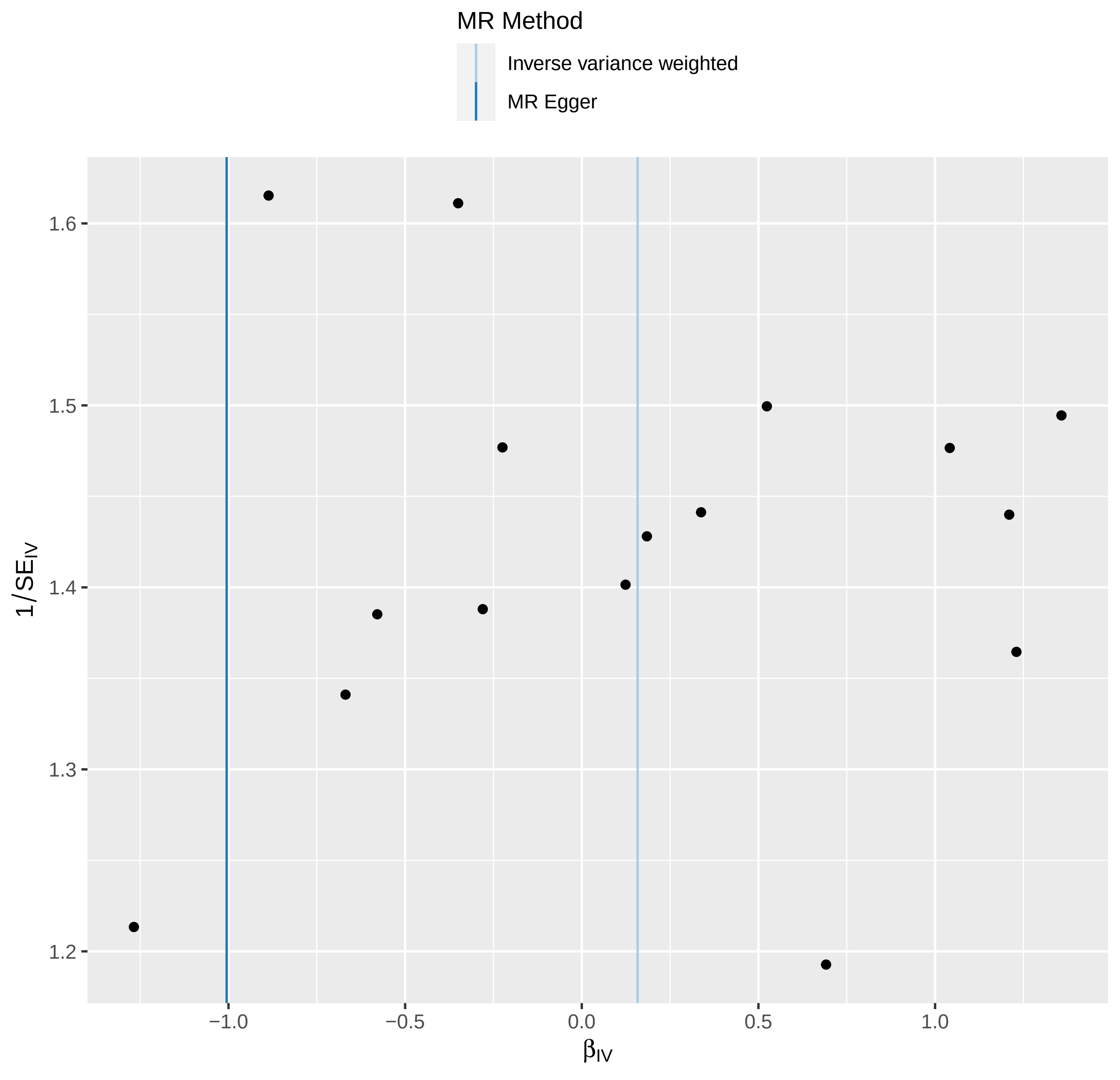

Batch 18 : Gut microbiota abundance (family Lactobacillaceae id.1836) on Graves' disease

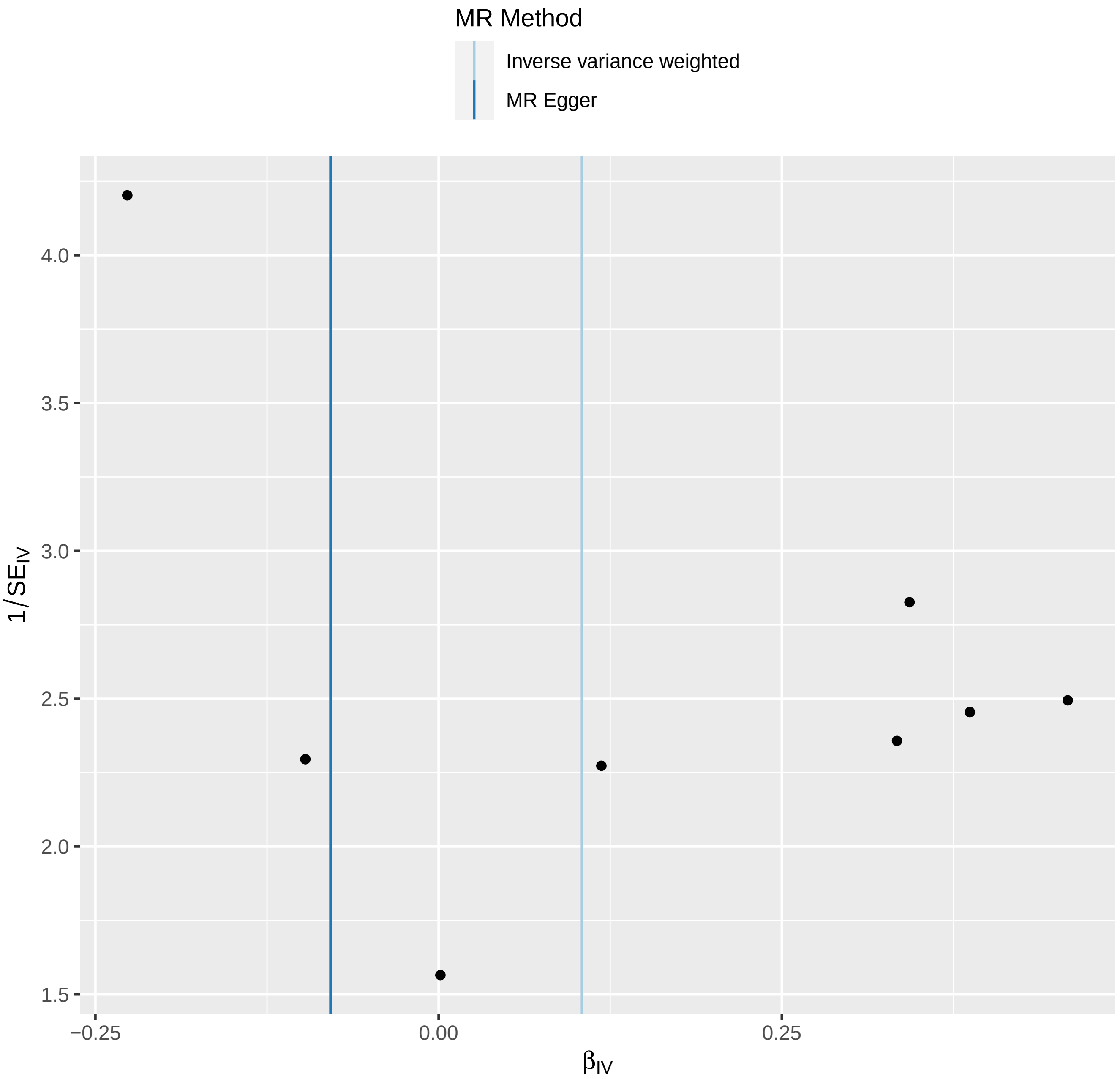

Batch 19 : Gut microbiota abundance (family Methanobacteriaceae id.121) on Graves' disease

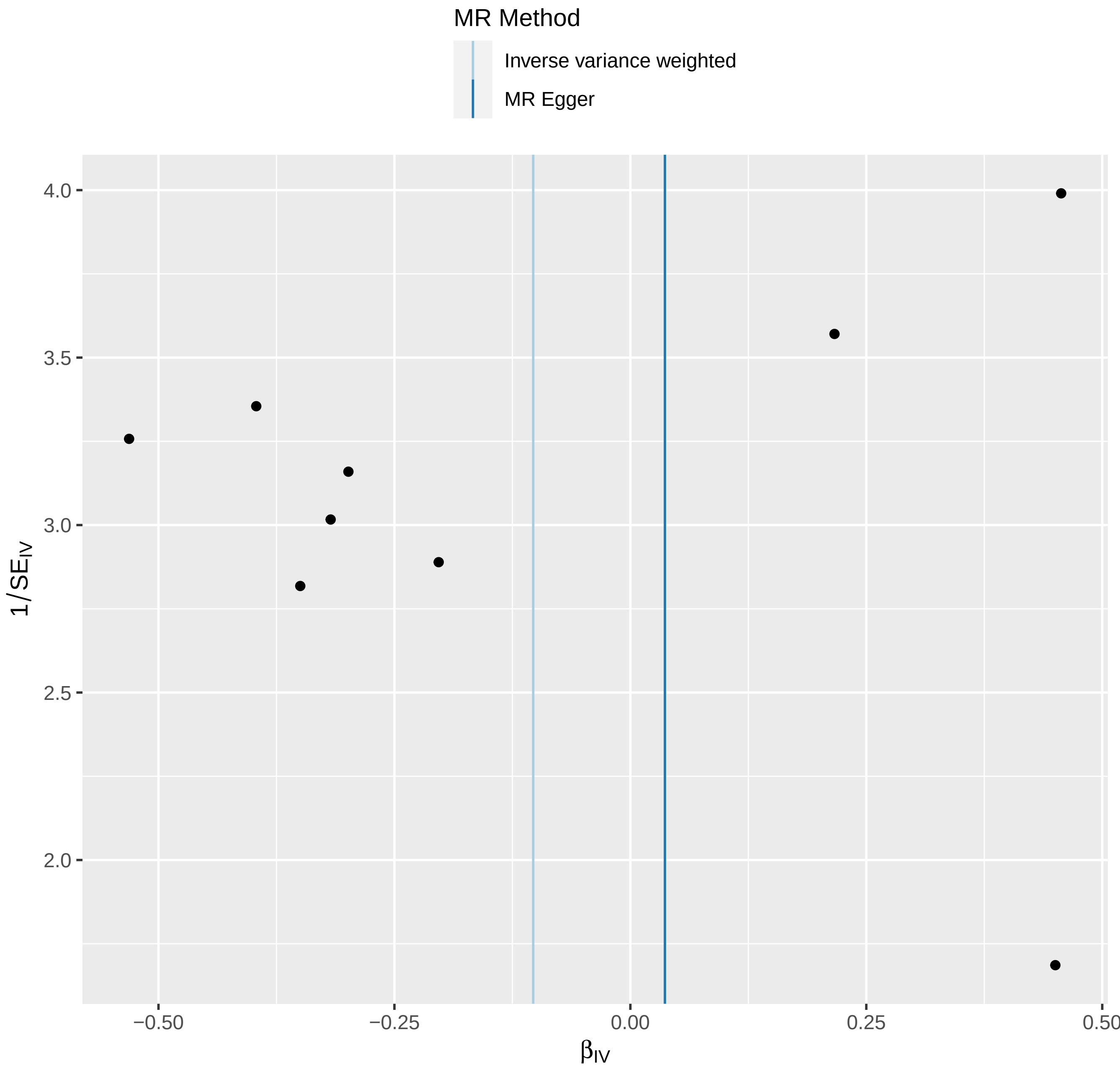

Batch 20 : Gut microbiota abundance (family Oxalobacteraceae id.2966) on Graves' disease

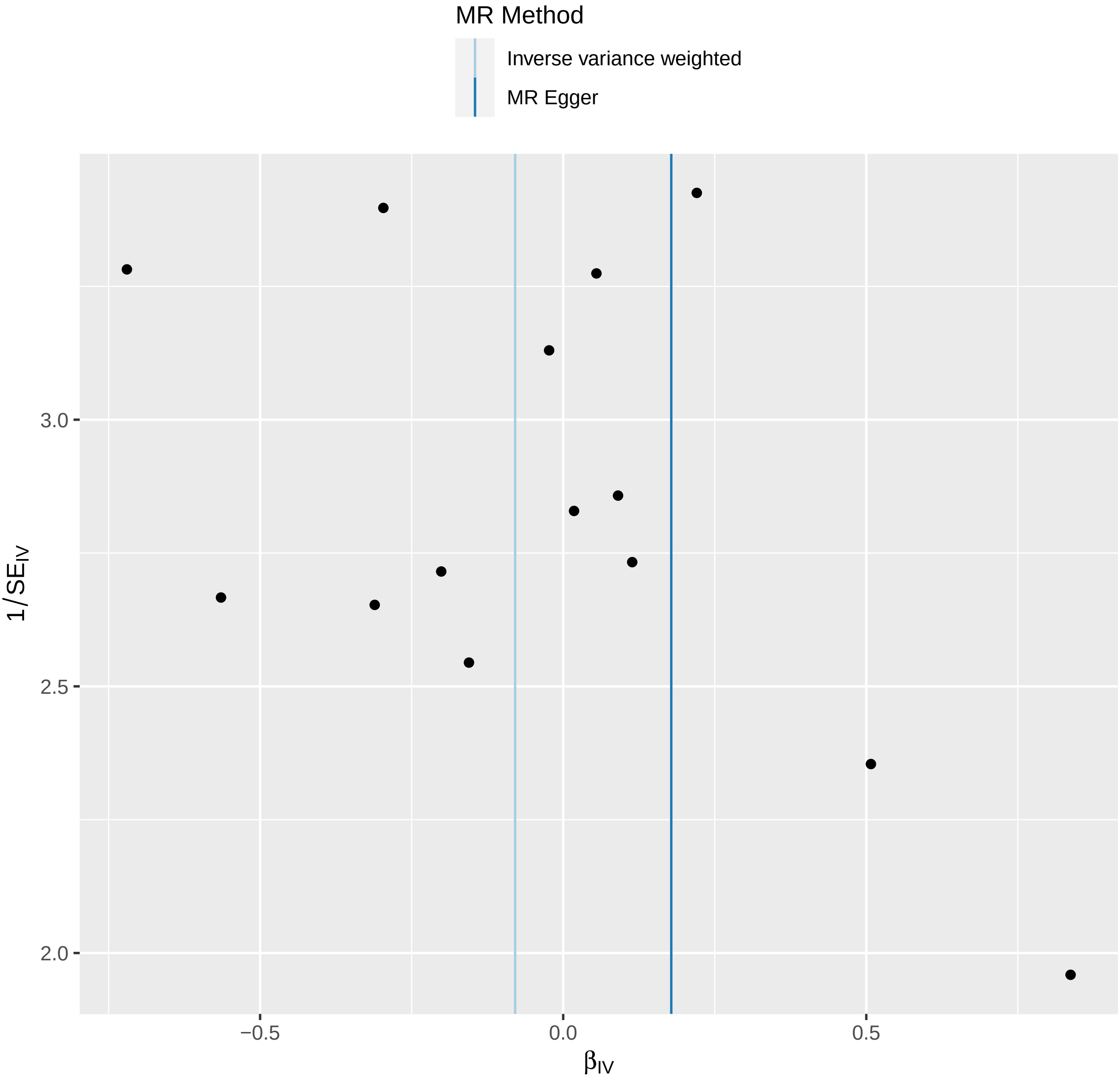

Batch 21 : Gut microbiota abundance (family Pasteurellaceae id.3689) on Graves' disease

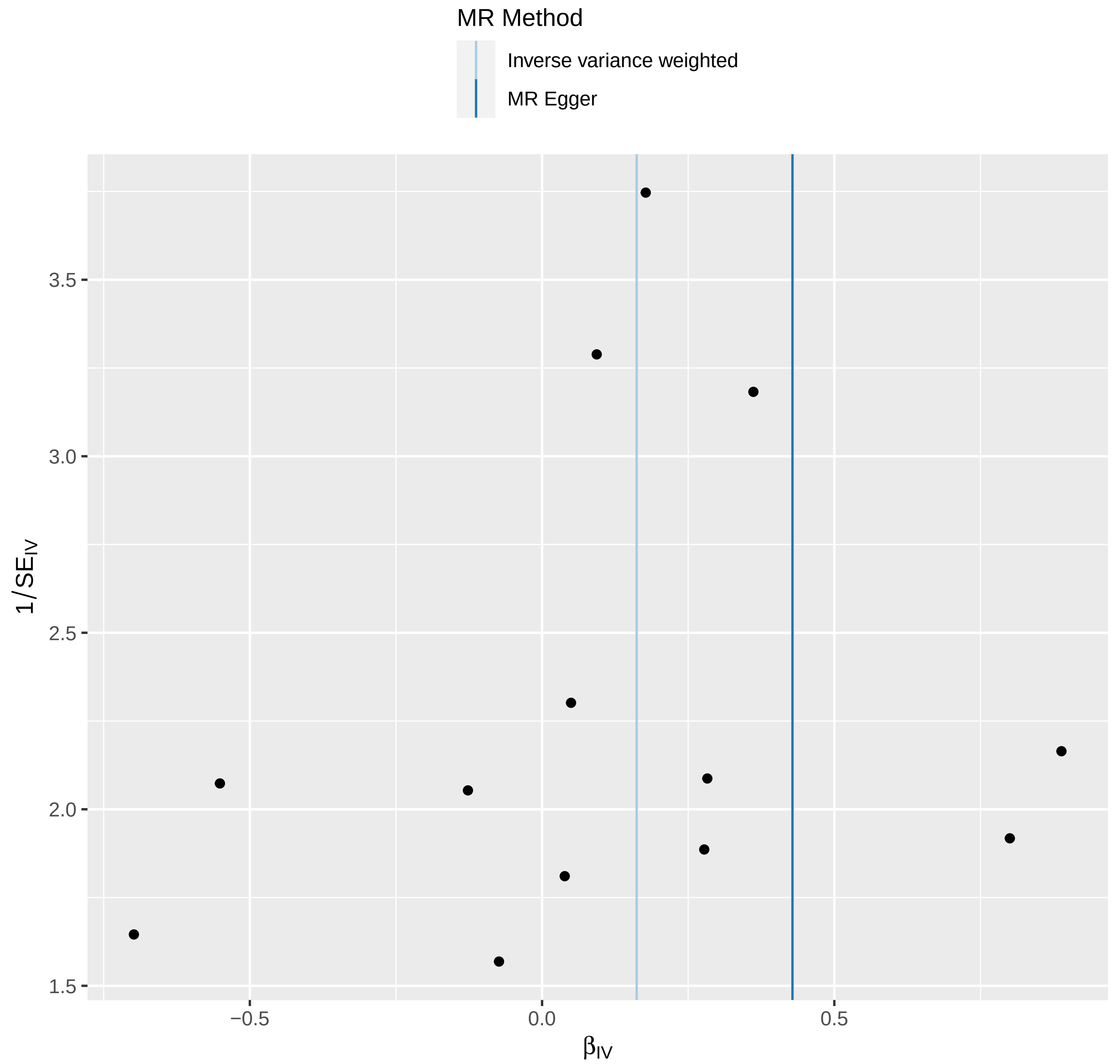

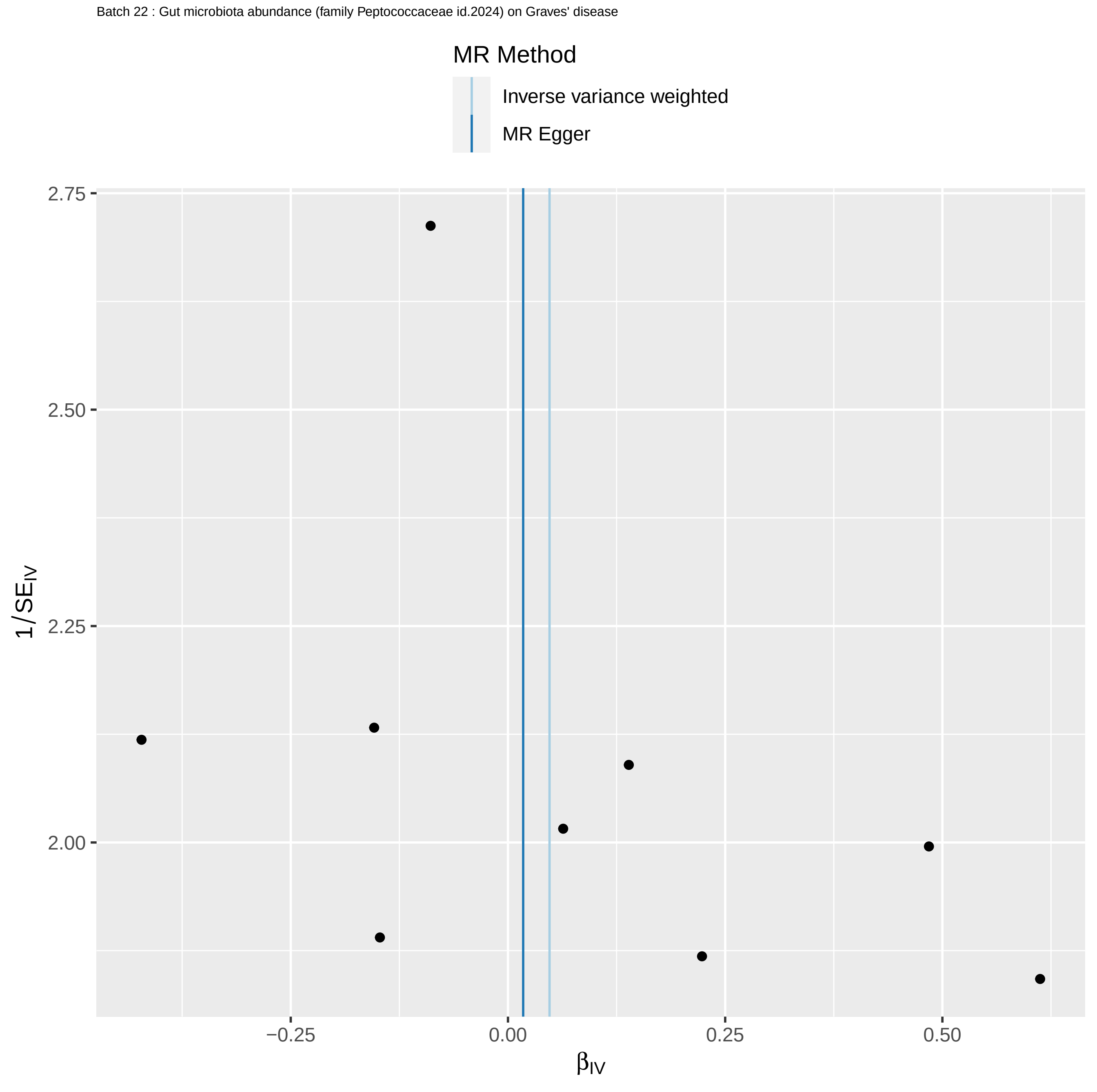

Batch 22 : Gut microbiota abundance (family Peptococcaceae id.2024) on Graves' disease
MR Method
Inverse variance weighted
MR Egger
2.75
2.50
2.25
2.00
1/SE_IV
−0.25
0.00
0.25
0.50
β_IV

Batch 23 : Gut microbiota abundance (family Peptostreptococcaceae id.2042) on Graves' disease

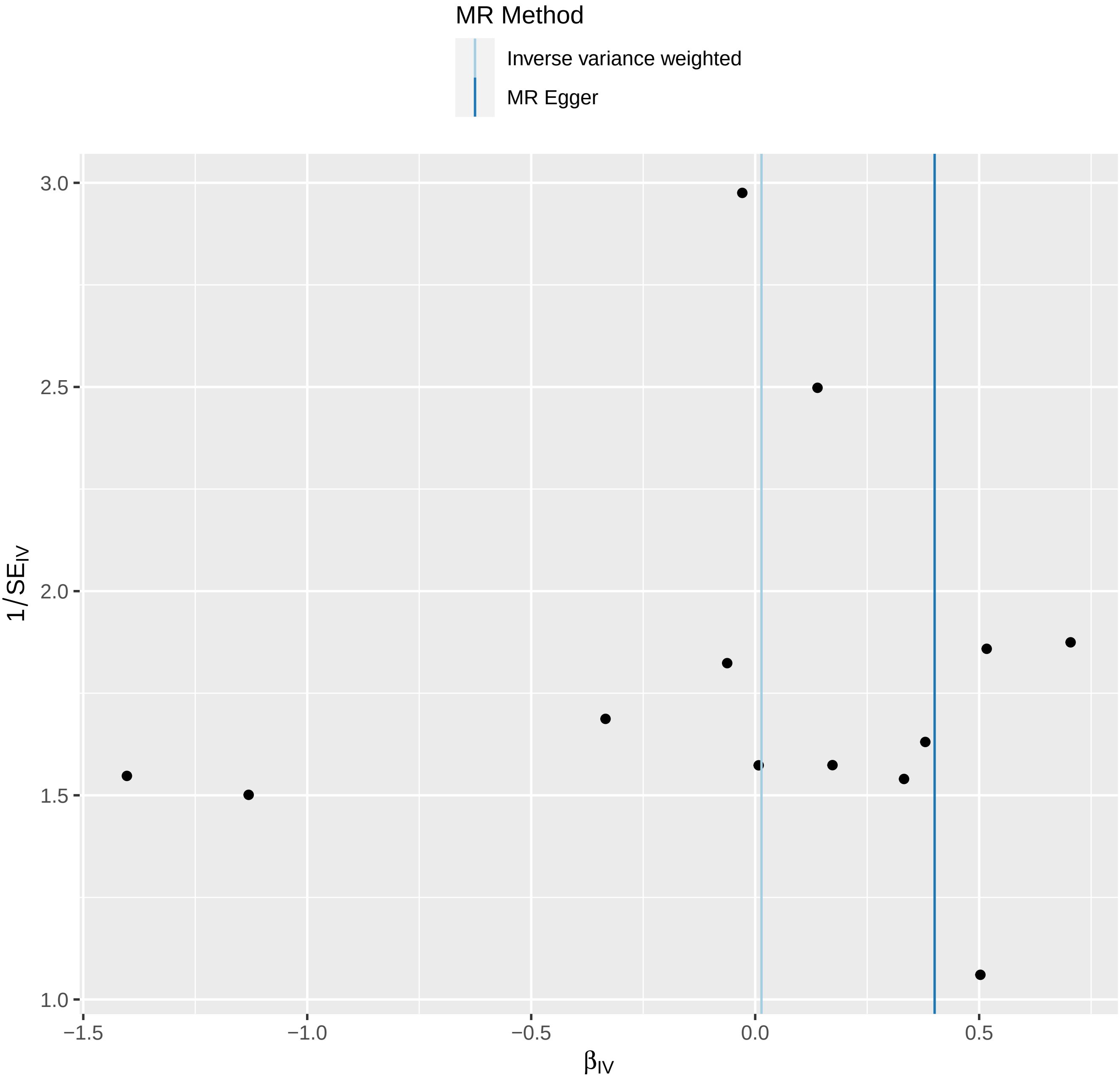

Batch 24 : Gut microbiota abundance (family Porphyromonadaceae id.943) on Graves' disease

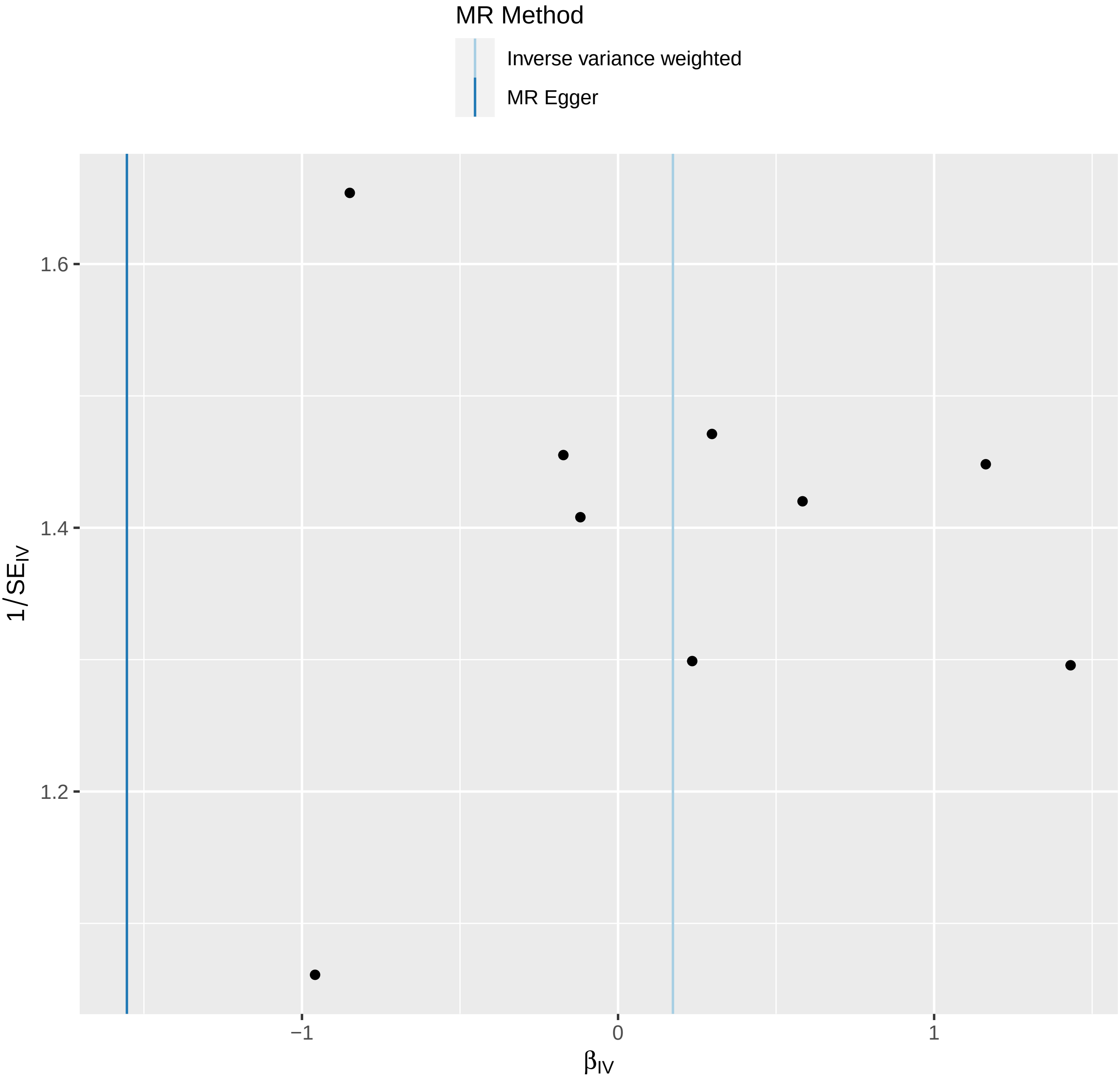

Batch 25 : Gut microbiota abundance (family Prevotellaceae id.960) on Graves' disease

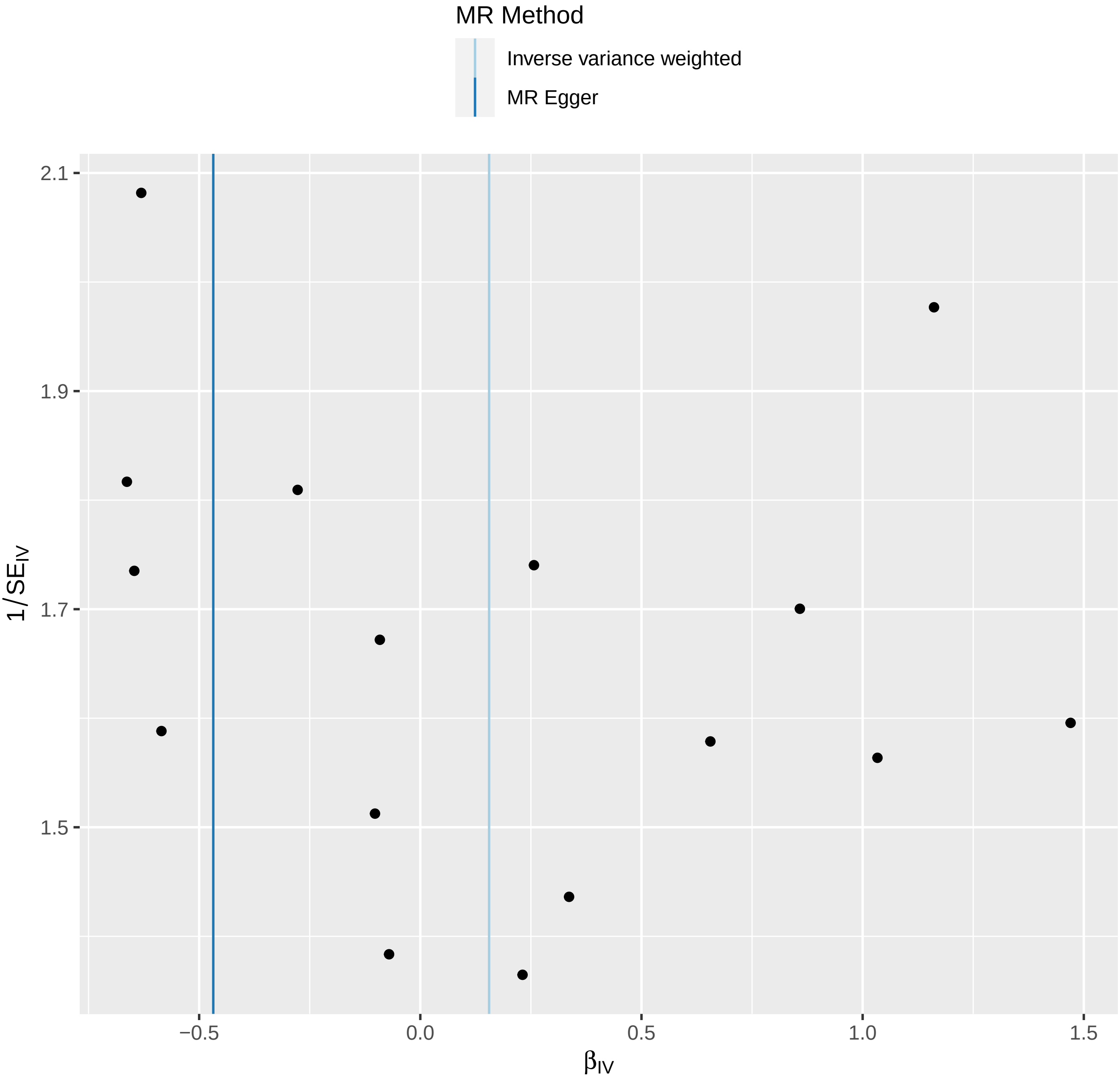

Batch 26 : Gut microbiota abundance (family Rhodospirillaceae id.2717) on Graves' disease

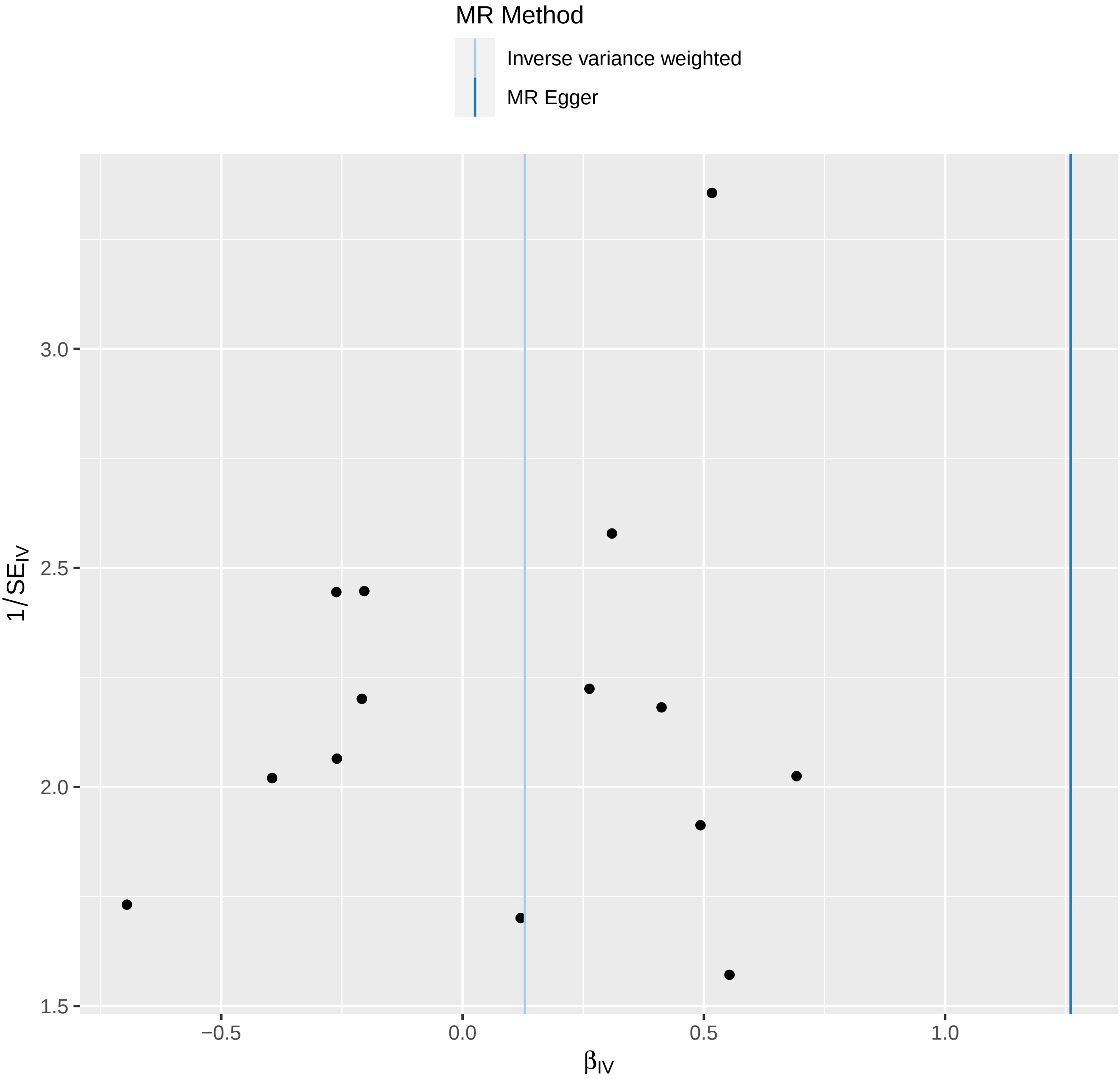

Batch 27 : Gut microbiota abundance (family Rikenellaceae id.967) on Graves' disease

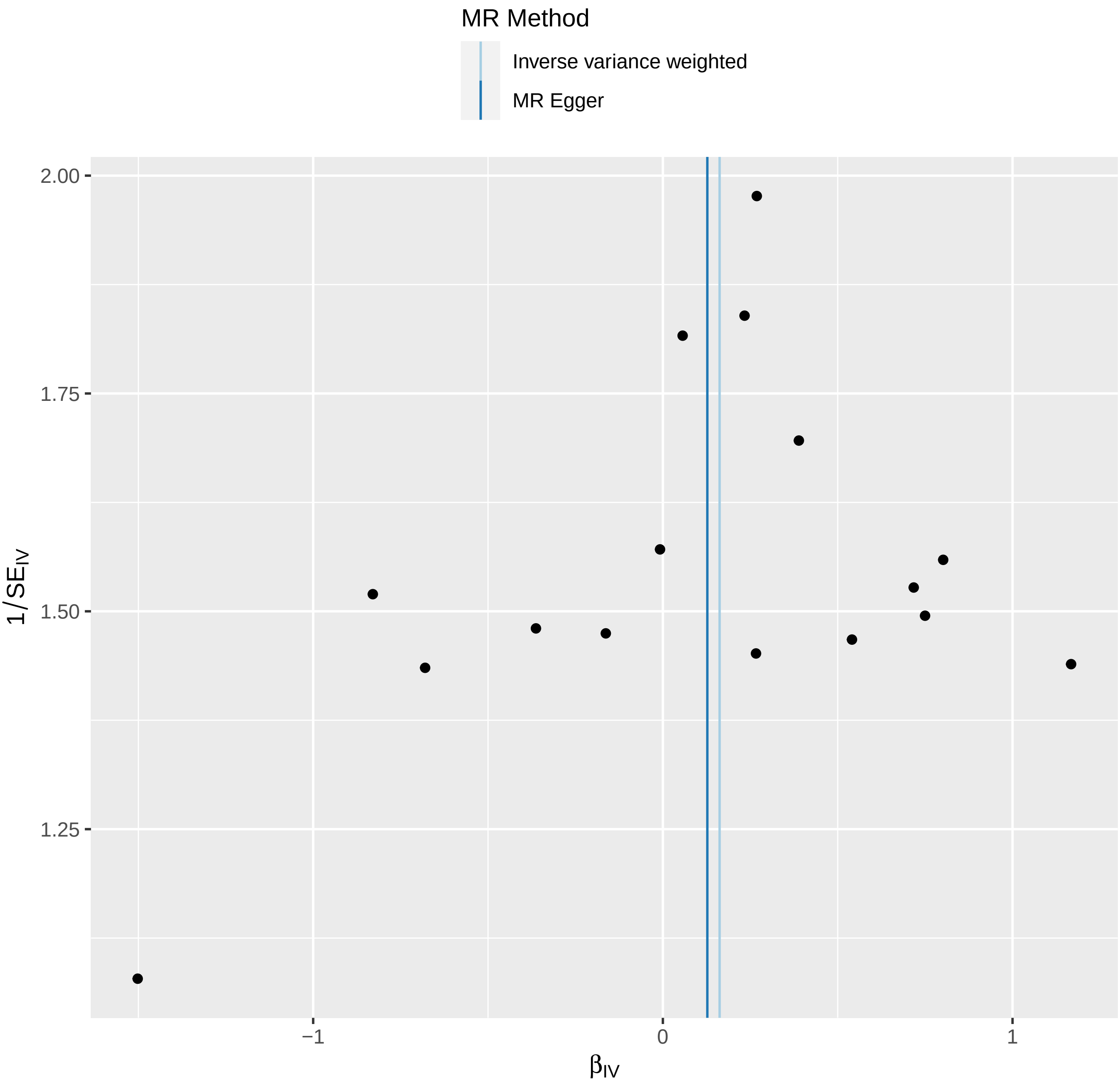

Batch 28 : Gut microbiota abundance (family Ruminococcaceae id.2050) on Graves' disease

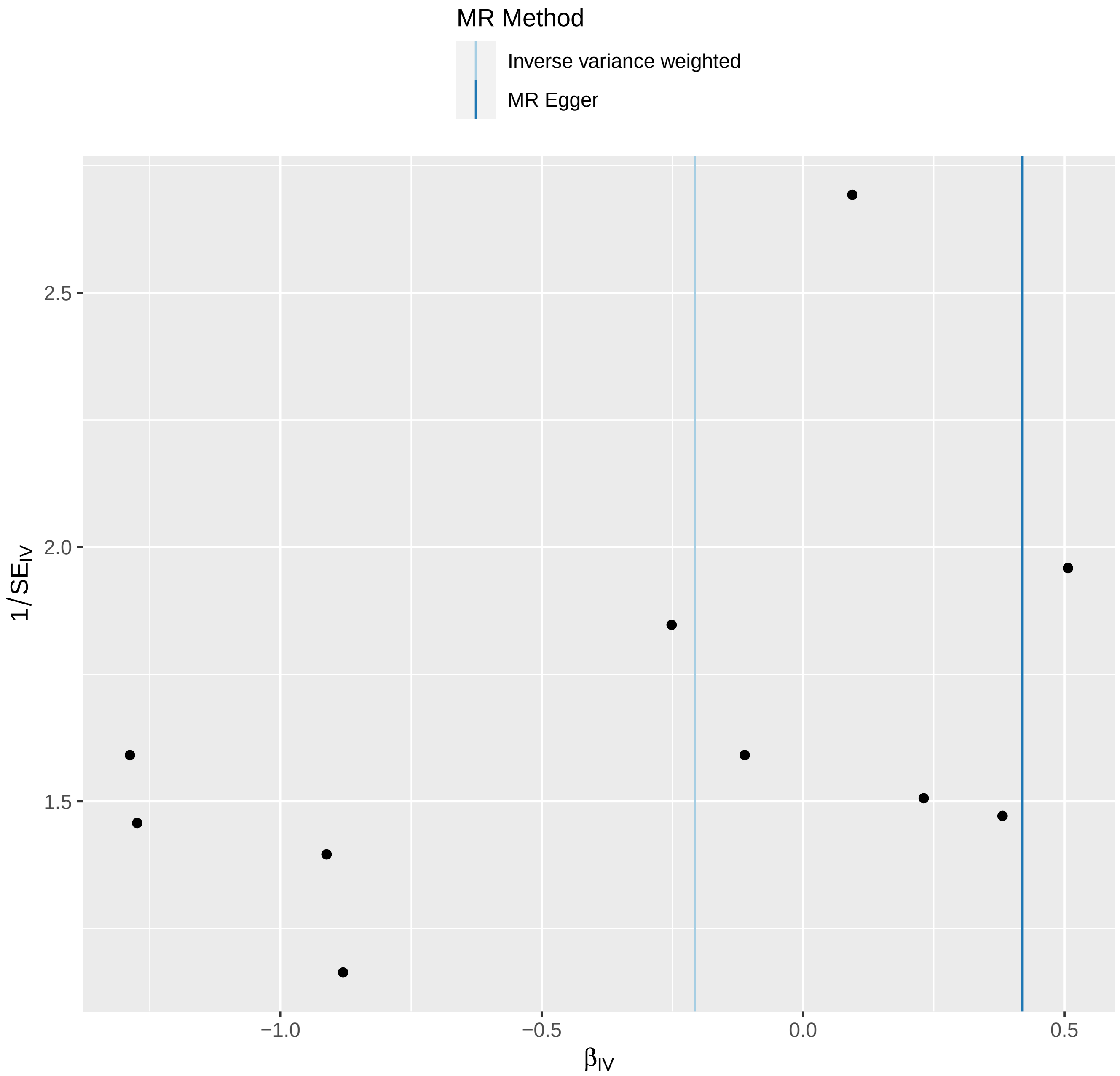

Batch 29 : Gut microbiota abundance (family Streptococcaceae id.1850) on Graves' disease

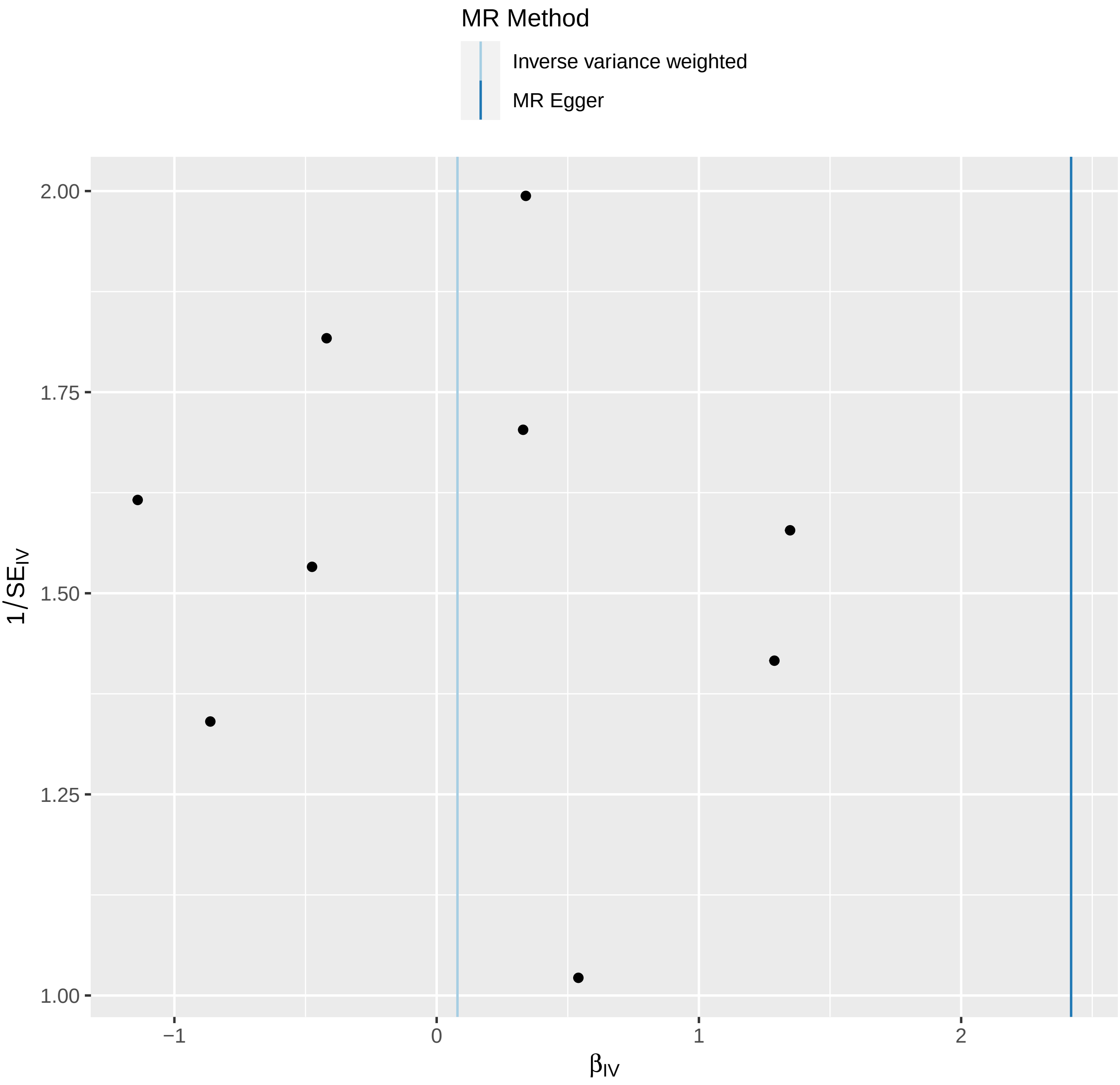

Batch 30 : Gut microbiota abundance (family Veillonellaceae id.2172) on Graves' disease

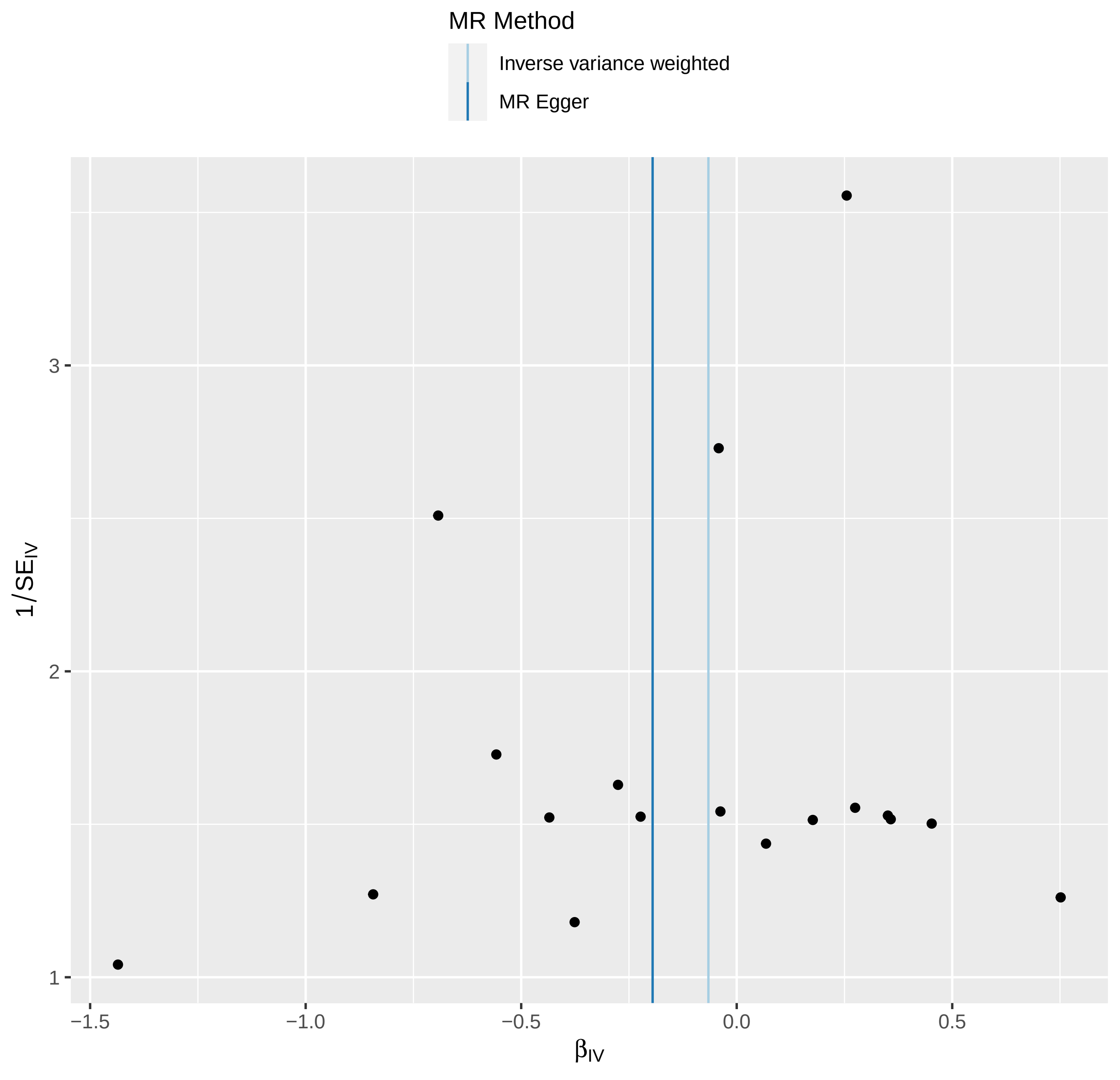

Batch 31 : Gut microbiota abundance (family Verrucomicrobiaceae id.4036) on Graves' disease

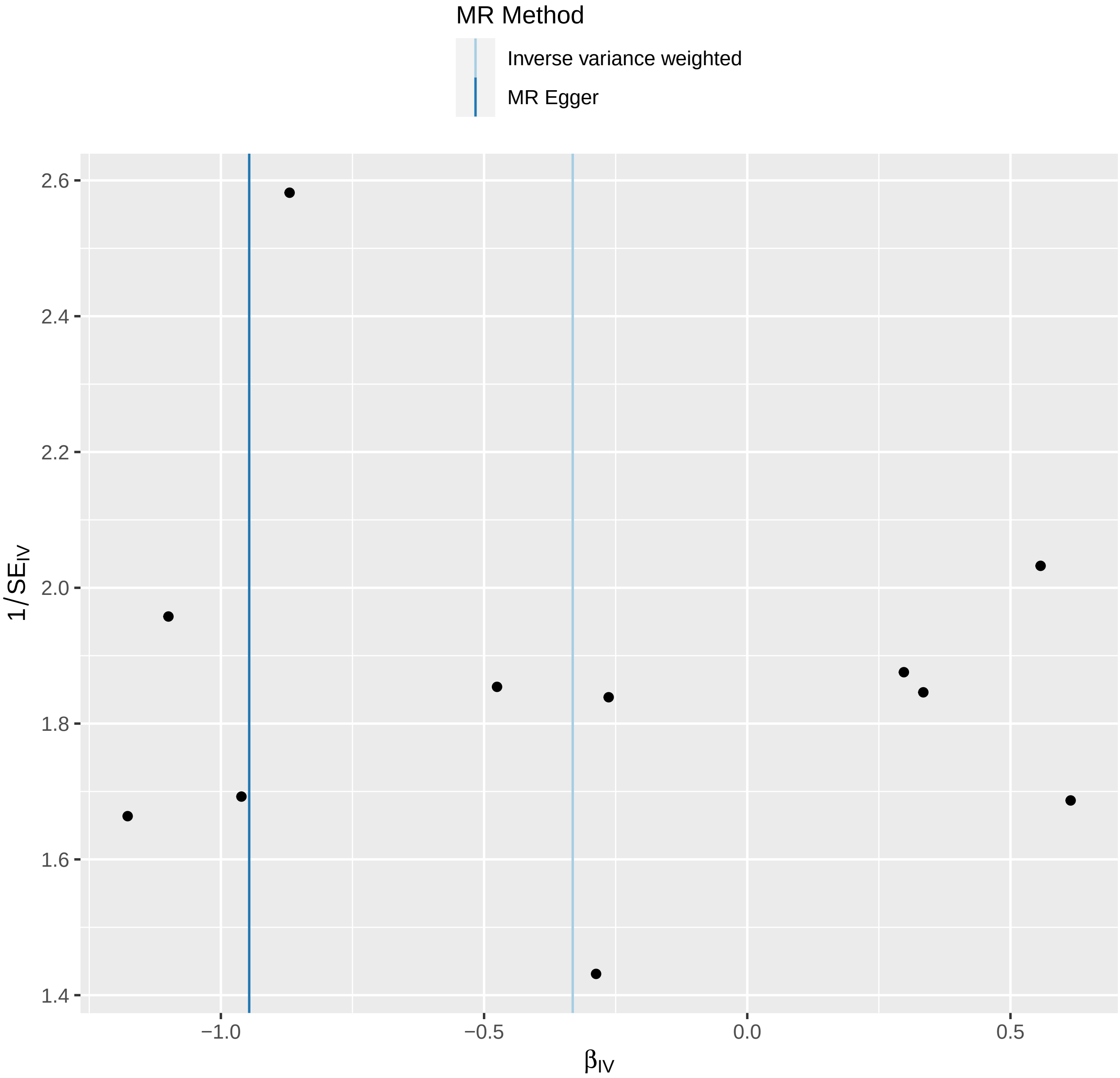

Batch 32 : Gut microbiota abundance (family Victivallaceae id.2255) on Graves' disease

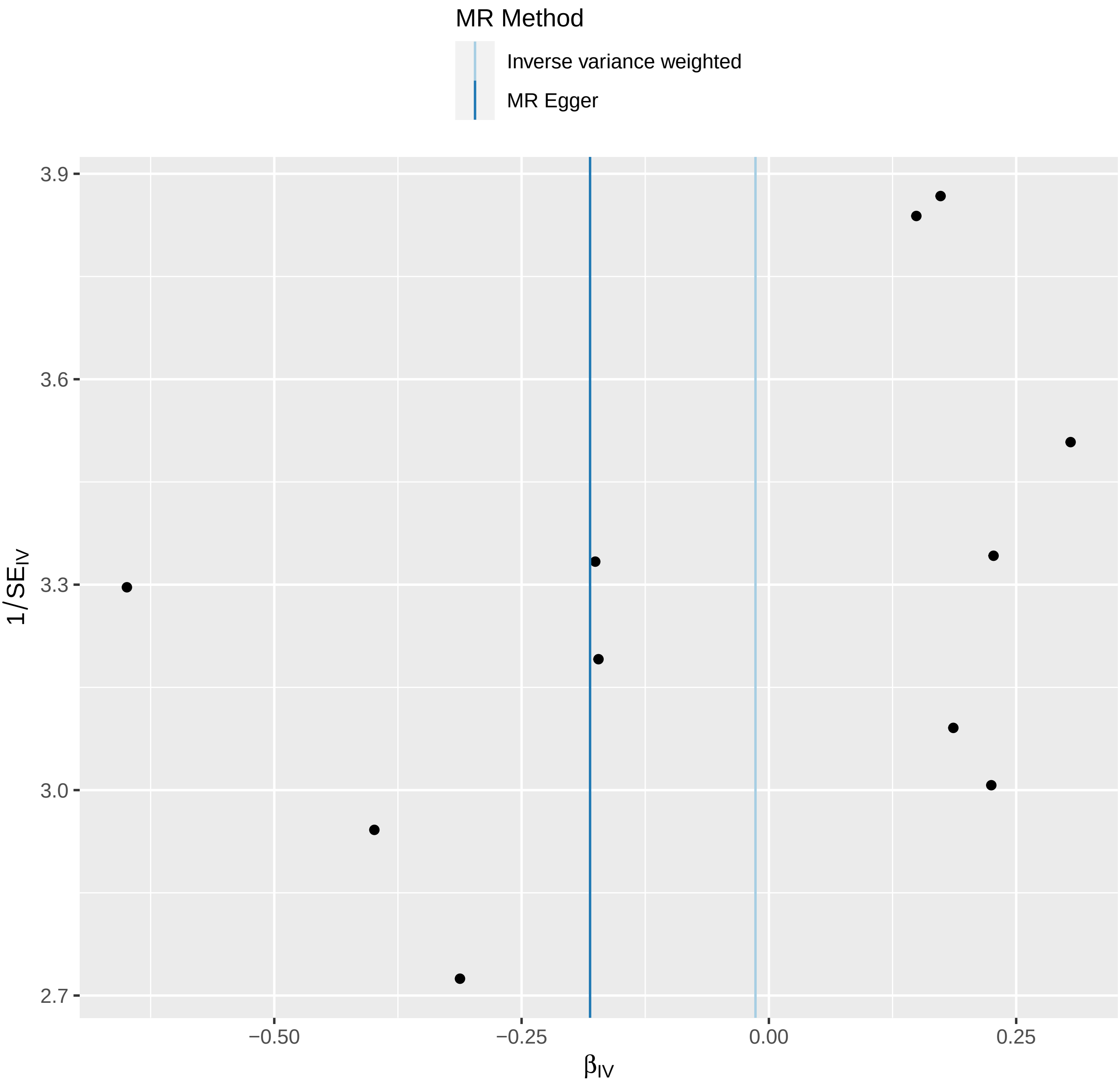

Batch 33 : Gut microbiota abundance (genus Actinomyces id.423) on Graves' disease

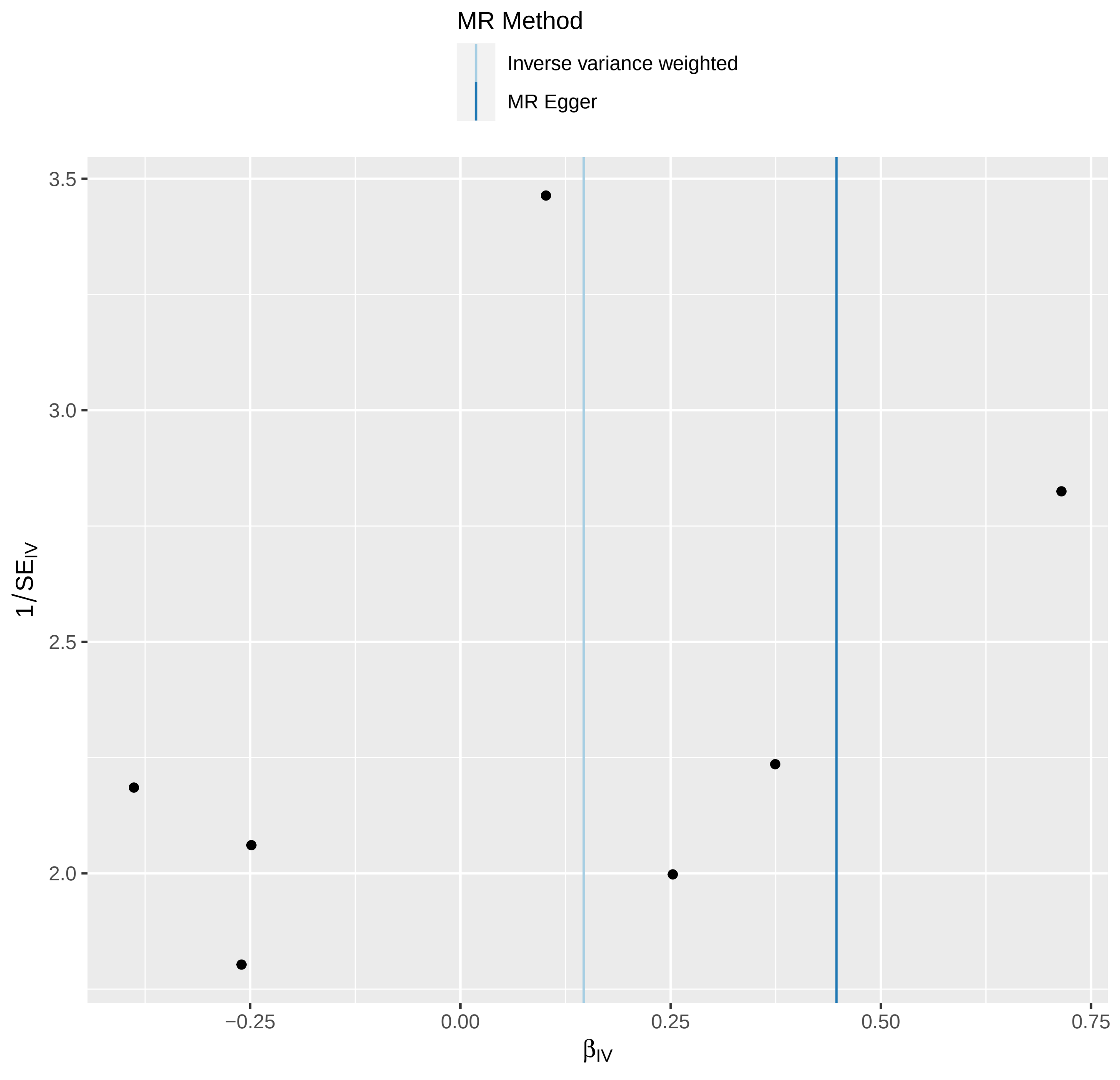

Batch 34 : Gut microbiota abundance (genus Adlercreutzia id.812) on Graves' disease

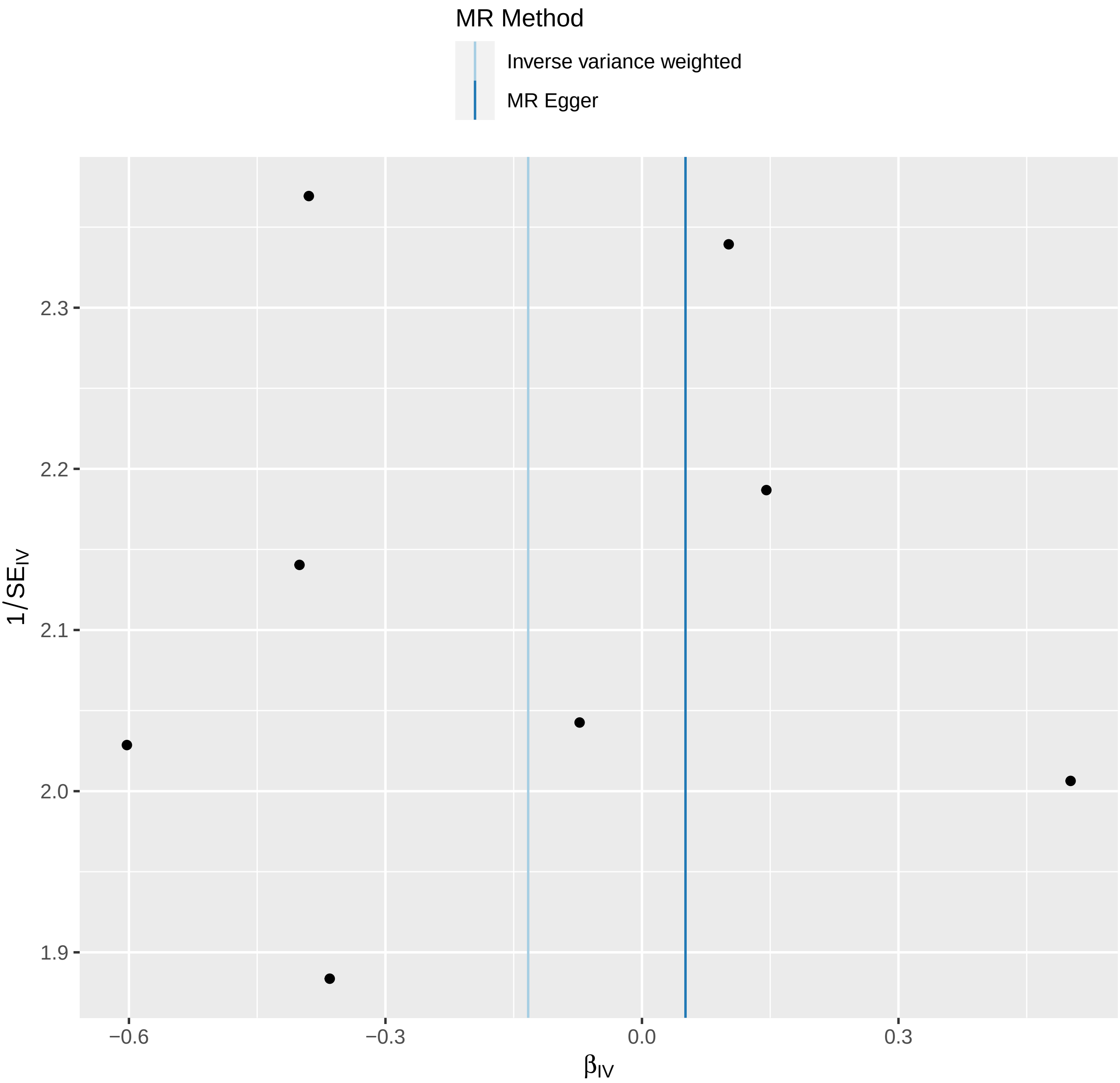

Batch 35 : Gut microbiota abundance (genus Akkermansia id.4037) on Graves' disease

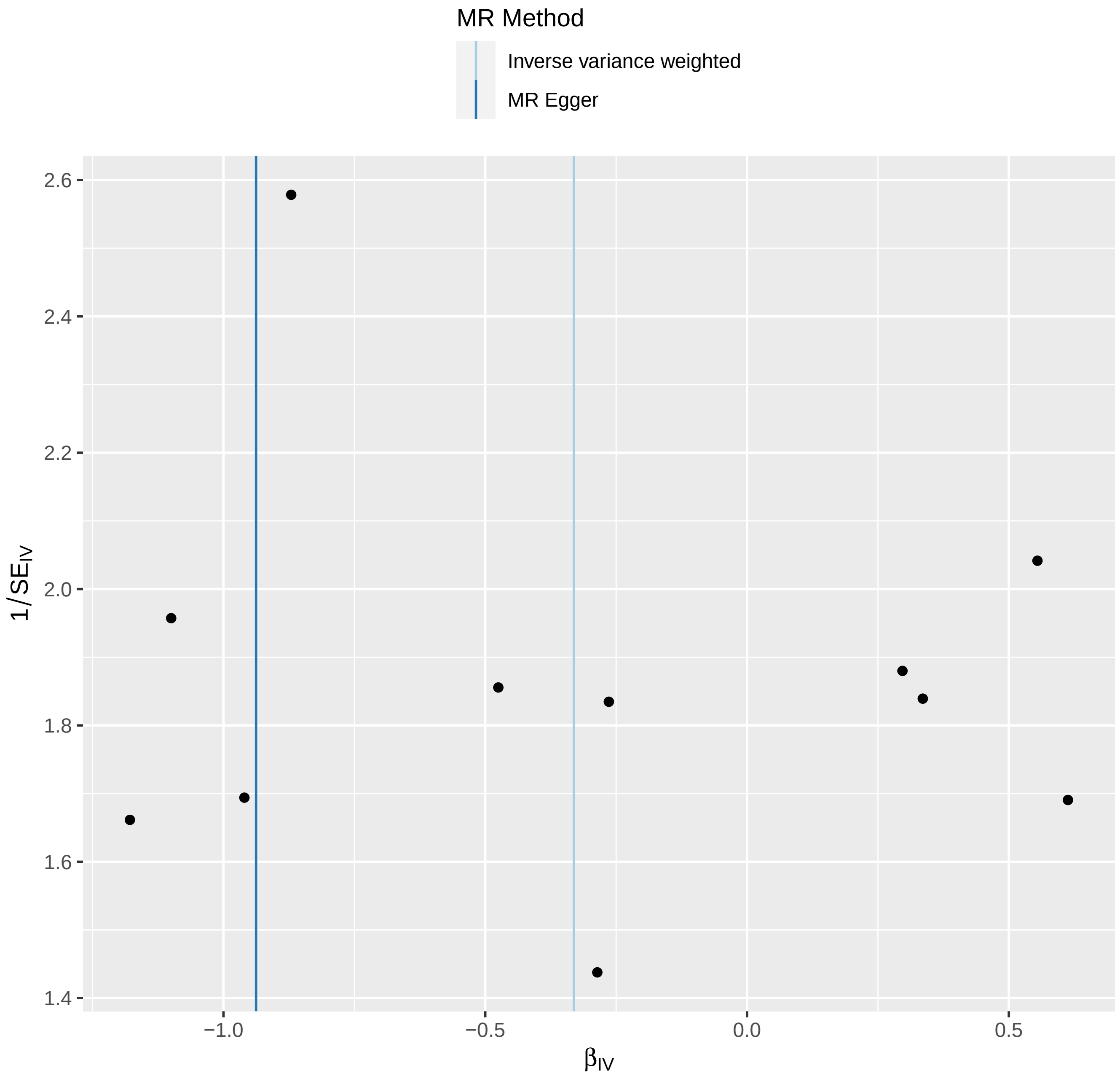

Batch 36 : Gut microbiota abundance (genus Alistipes id.968) on Graves' disease

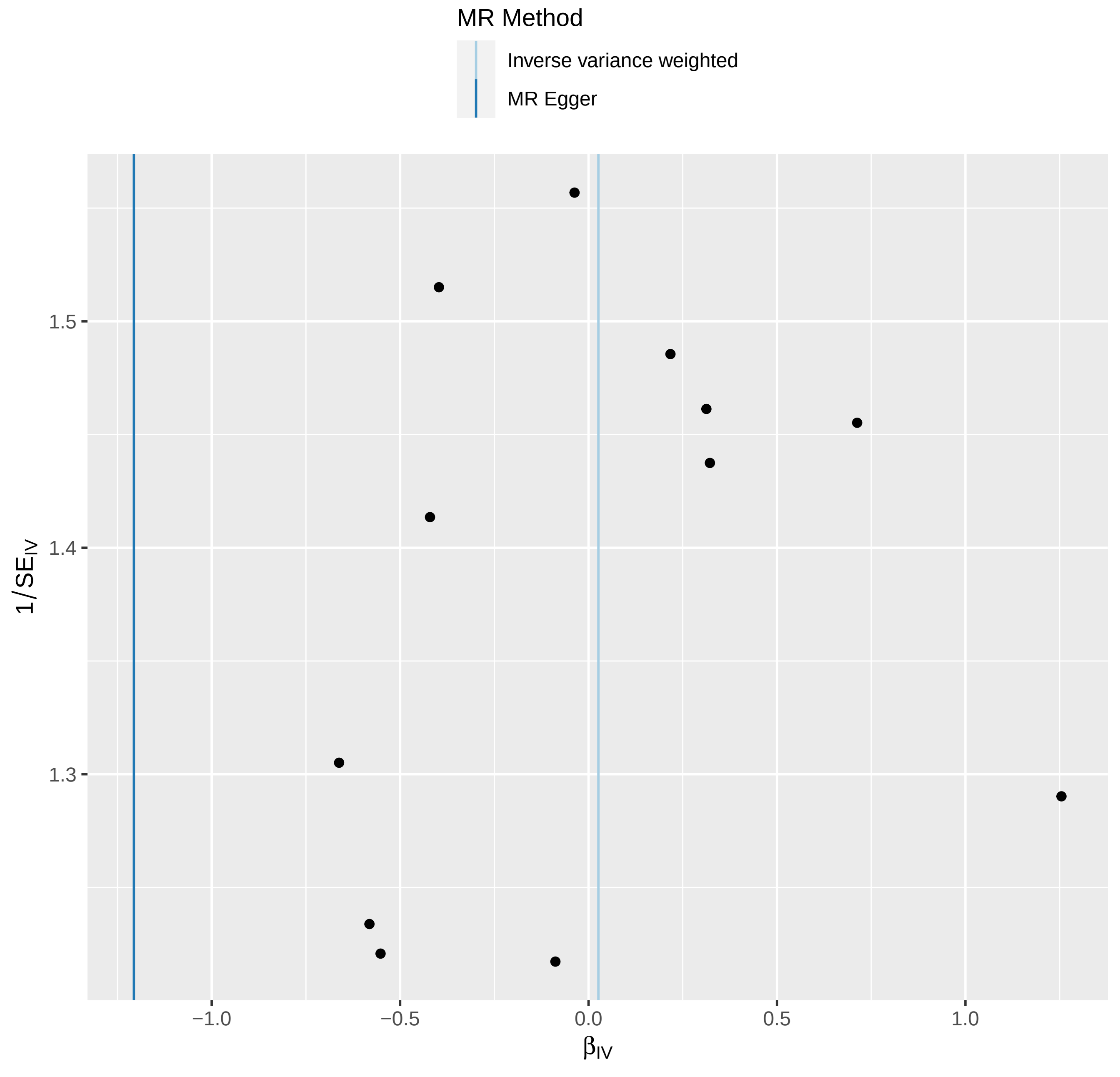

Batch 37 : Gut microbiota abundance (genus Allisonella id.2174) on Graves' disease

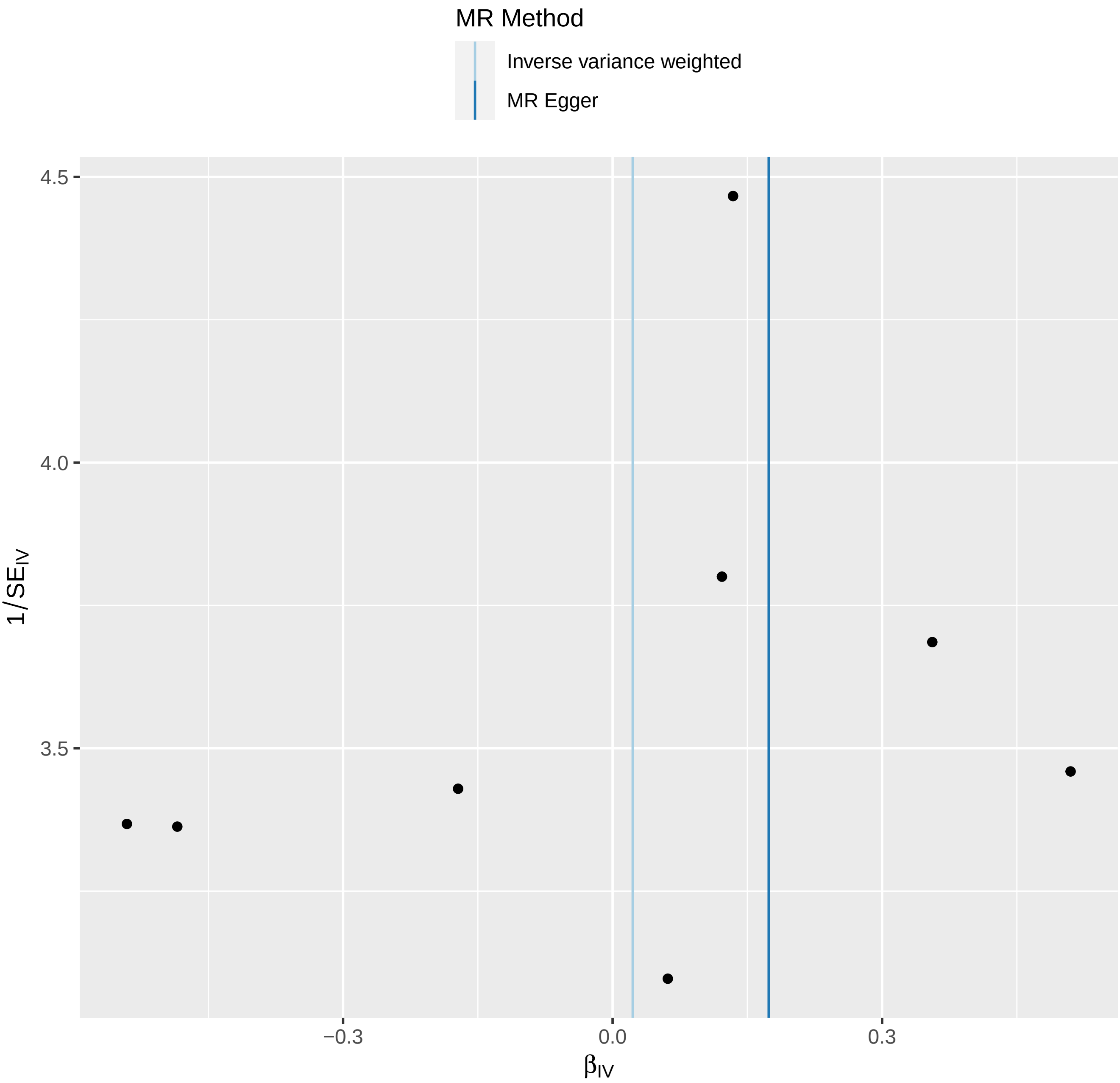

Batch 38 : Gut microbiota abundance (genus Alloprevotella id.961) on Graves' disease

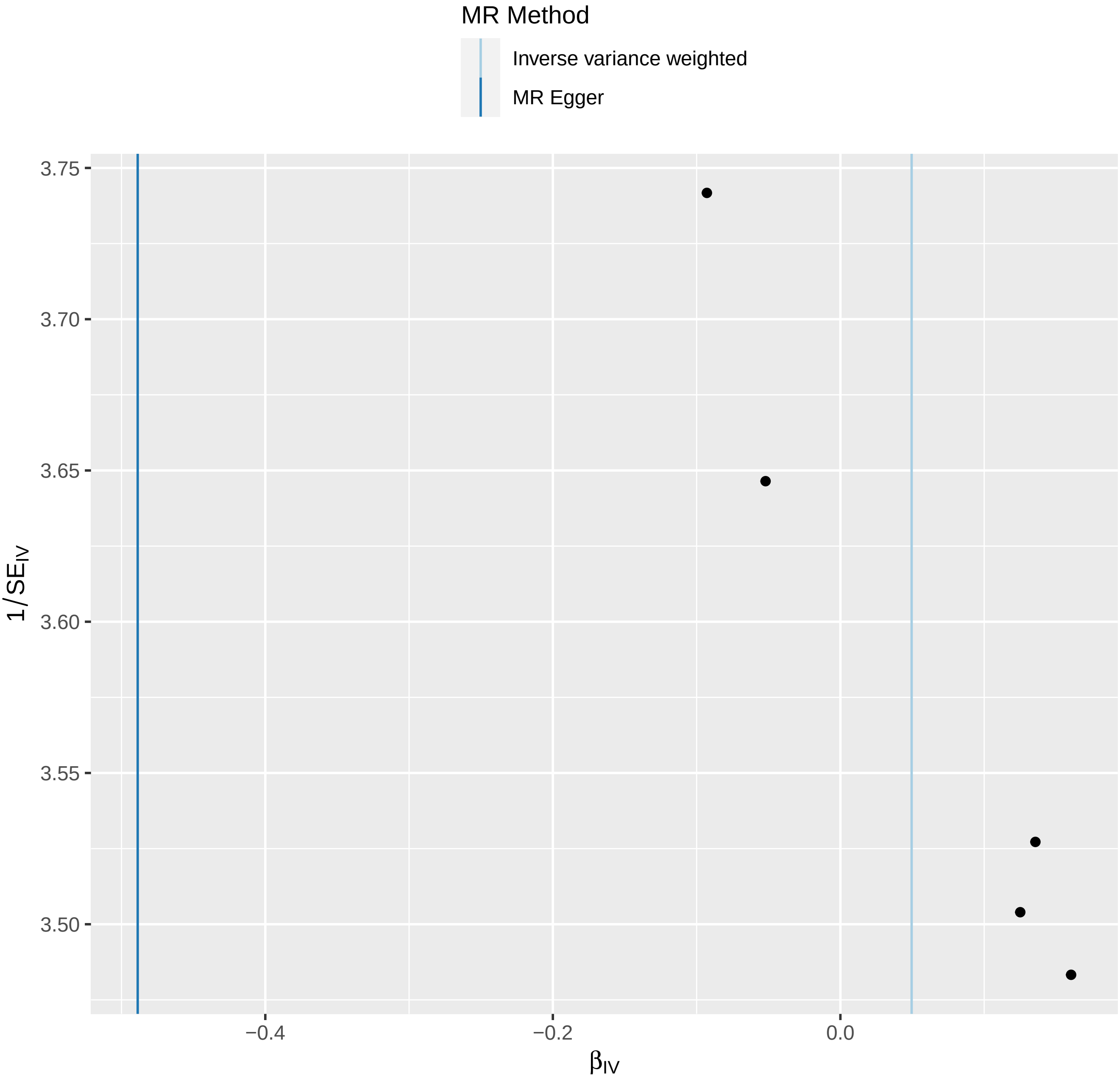

Batch 39 : Gut microbiota abundance (genus Anaerofilum id.2053) on Graves' disease

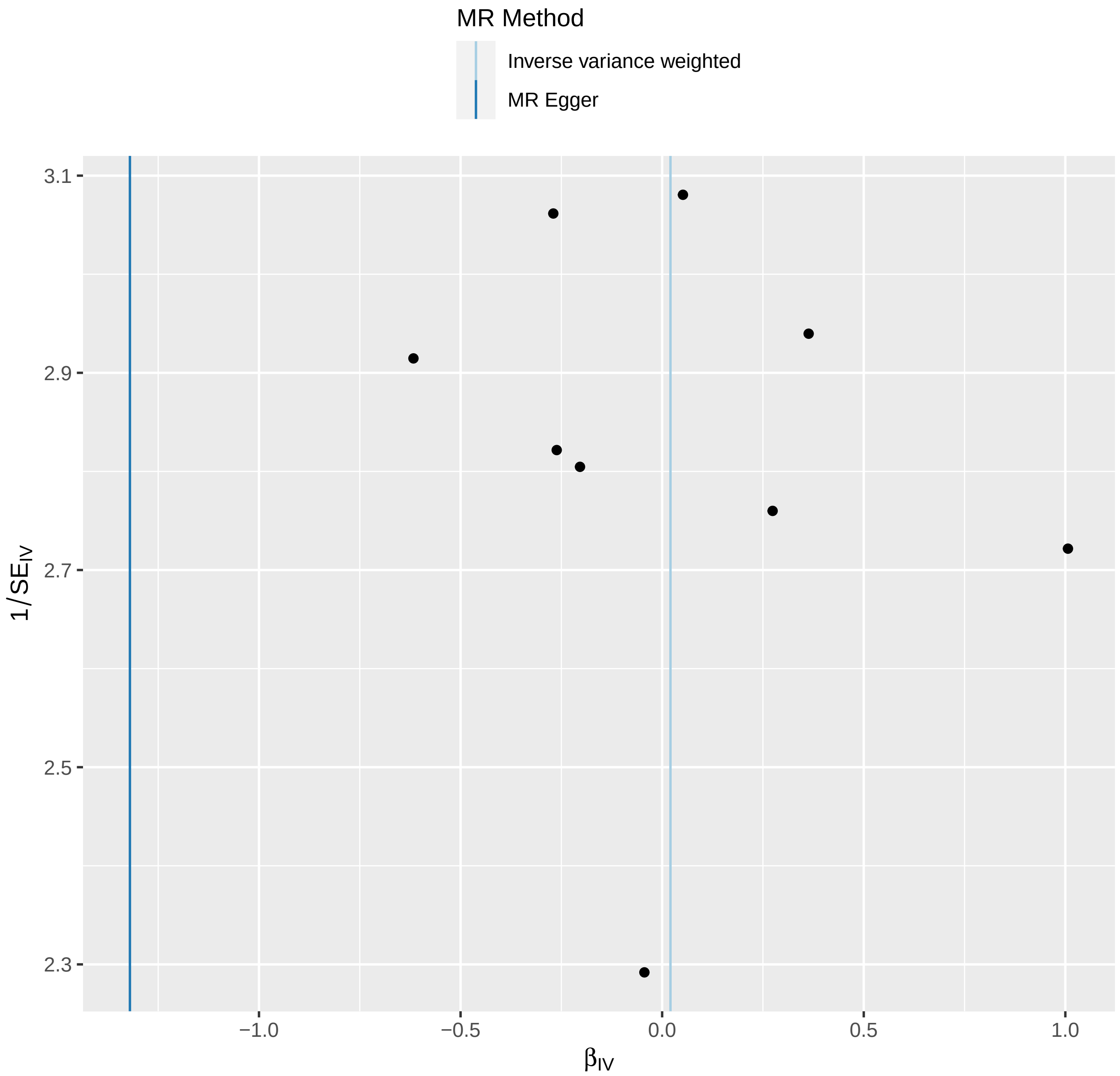

Batch 40 : Gut microbiota abundance (genus Anaerostipes id.1991) on Graves' disease

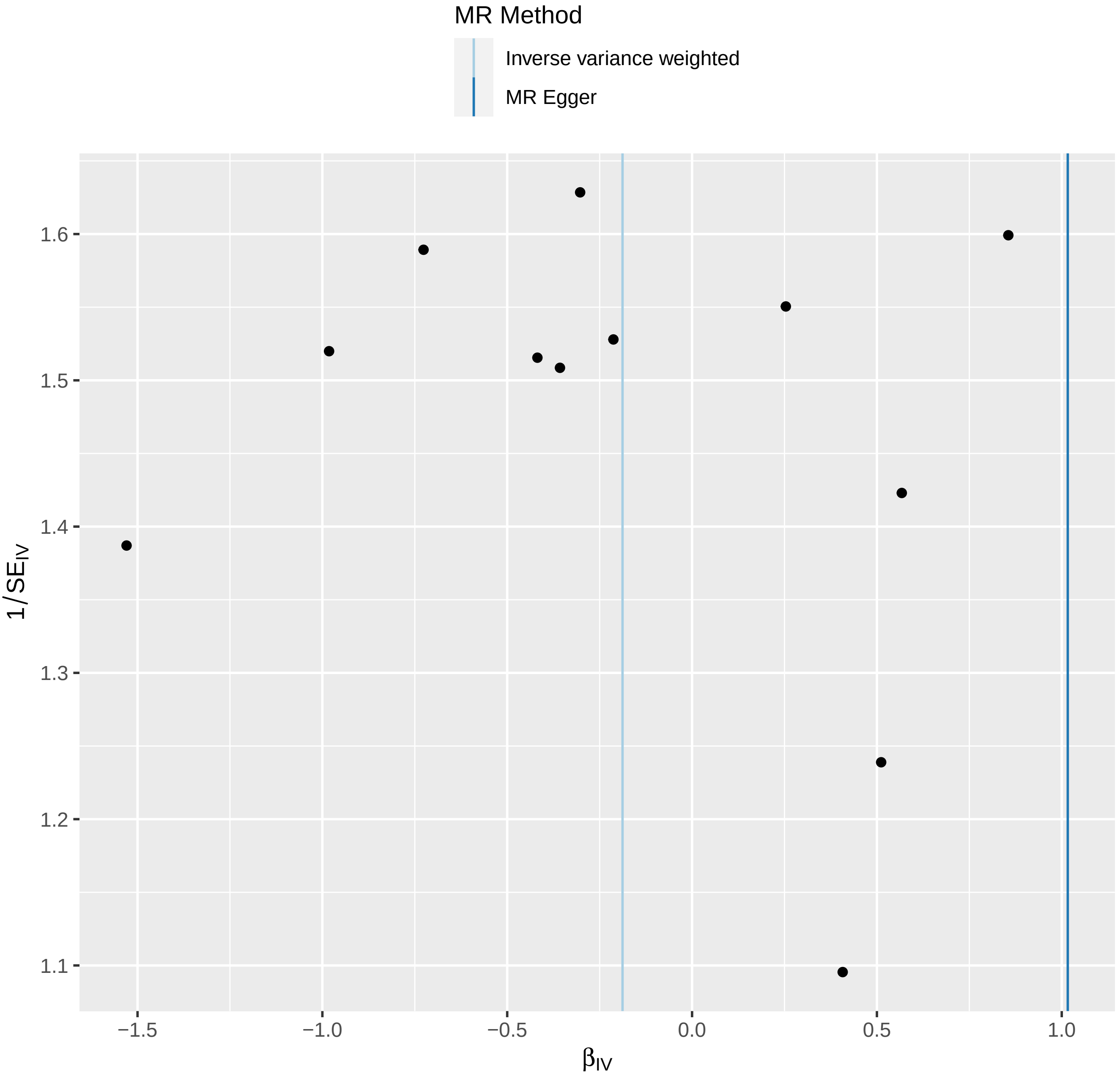

Batch 41 : Gut microbiota abundance (genus Anaerotruncus id.2054) on Graves' disease

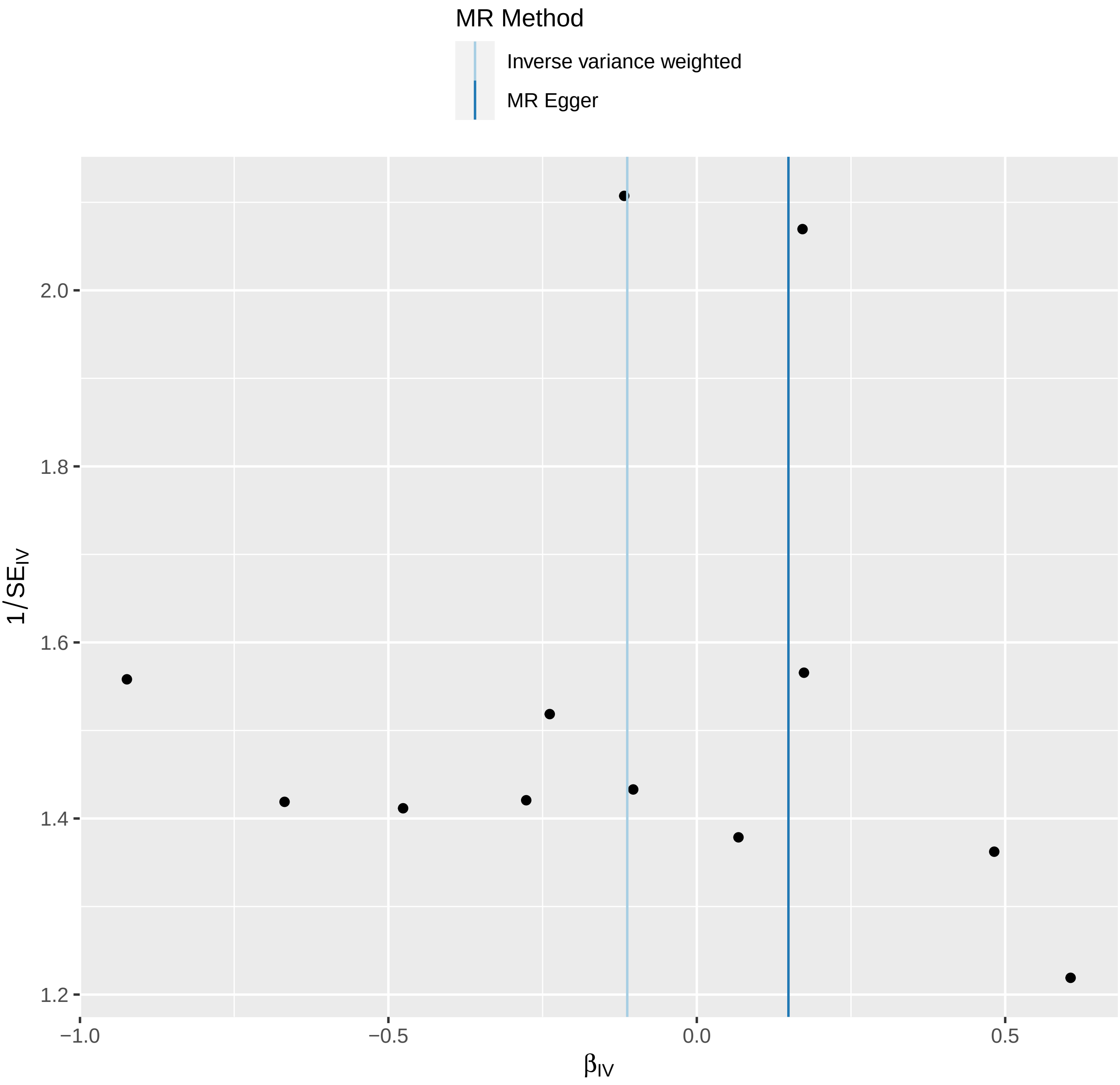

Batch 42 : Gut microbiota abundance (genus Bacteroides id.918) on Graves' disease

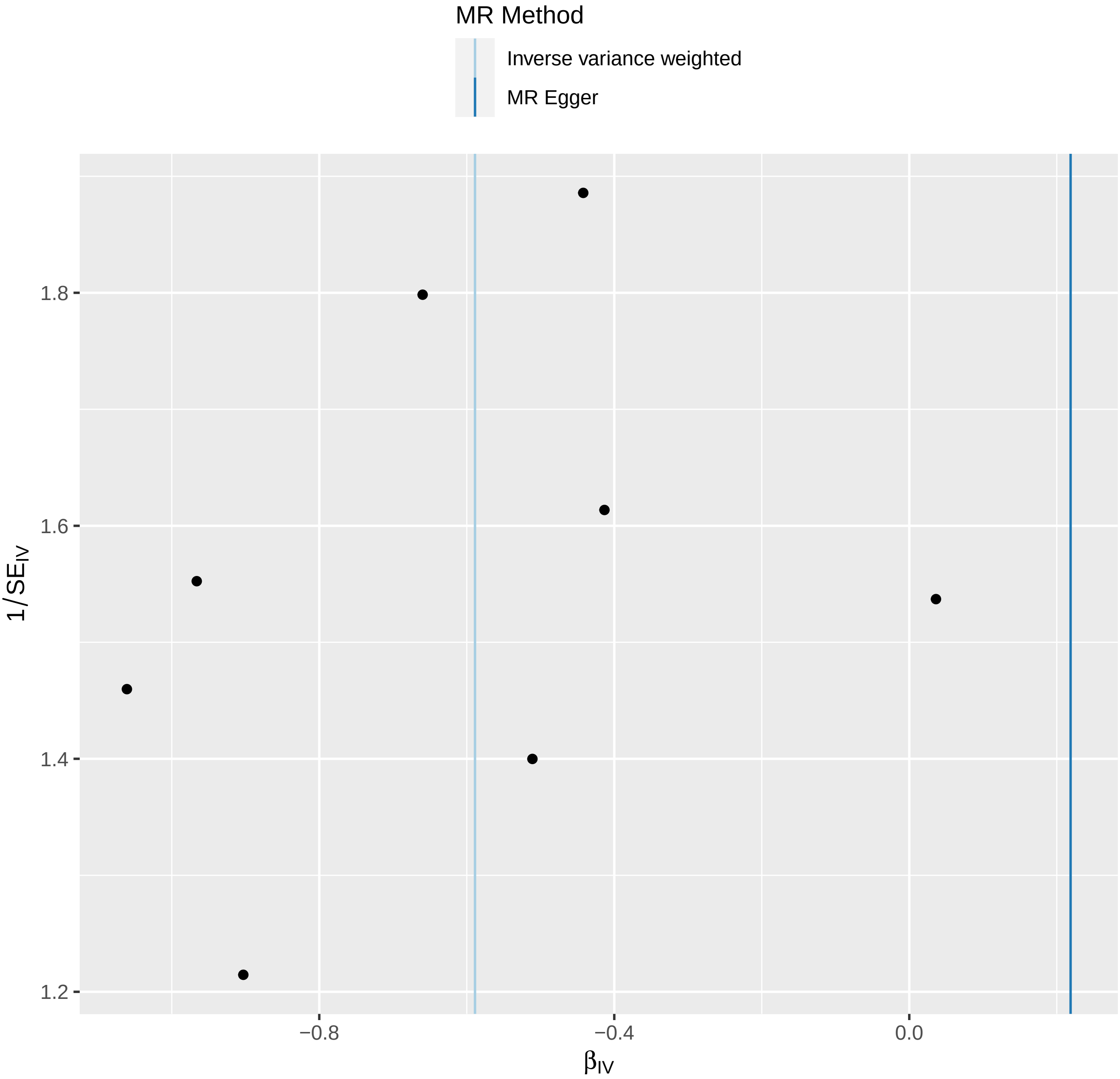

Batch 43 : Gut microbiota abundance (genus Barnesiella id.944) on Graves' disease

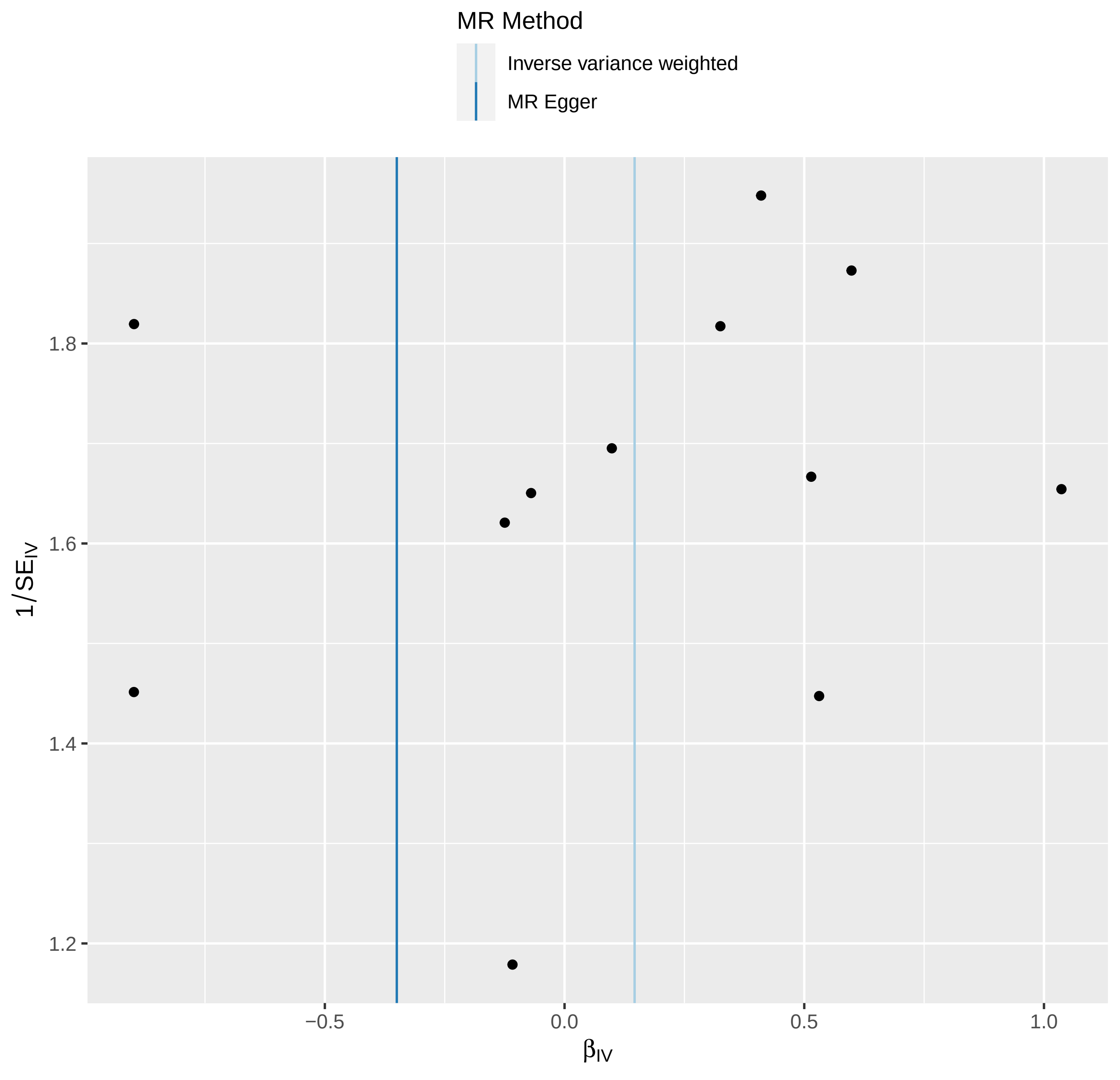

Batch 44 : Gut microbiota abundance (genus Bifidobacterium id.436) on Graves' disease

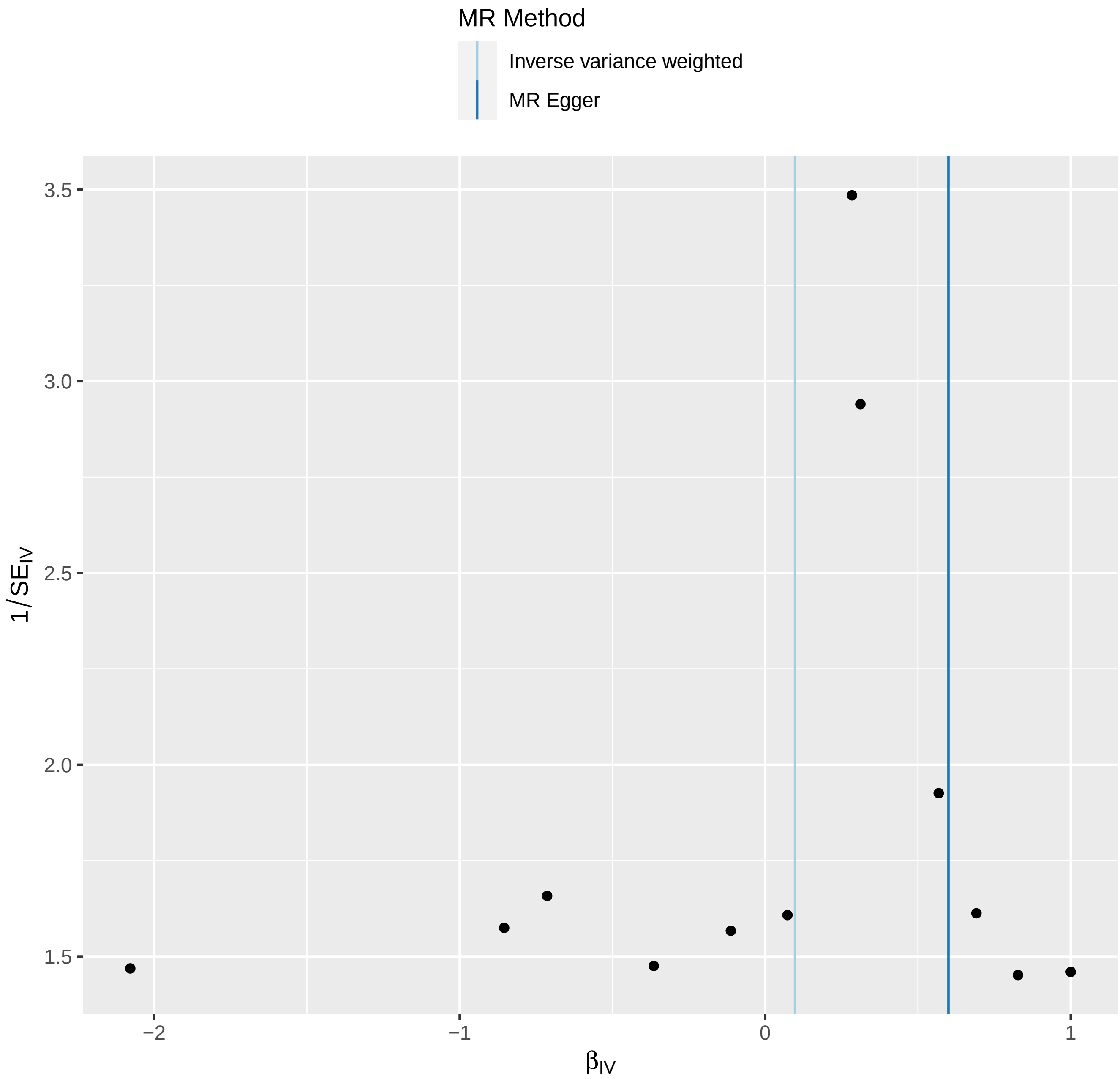

Batch 45 : Gut microbiota abundance (genus Bilophila id.3170) on Graves' disease

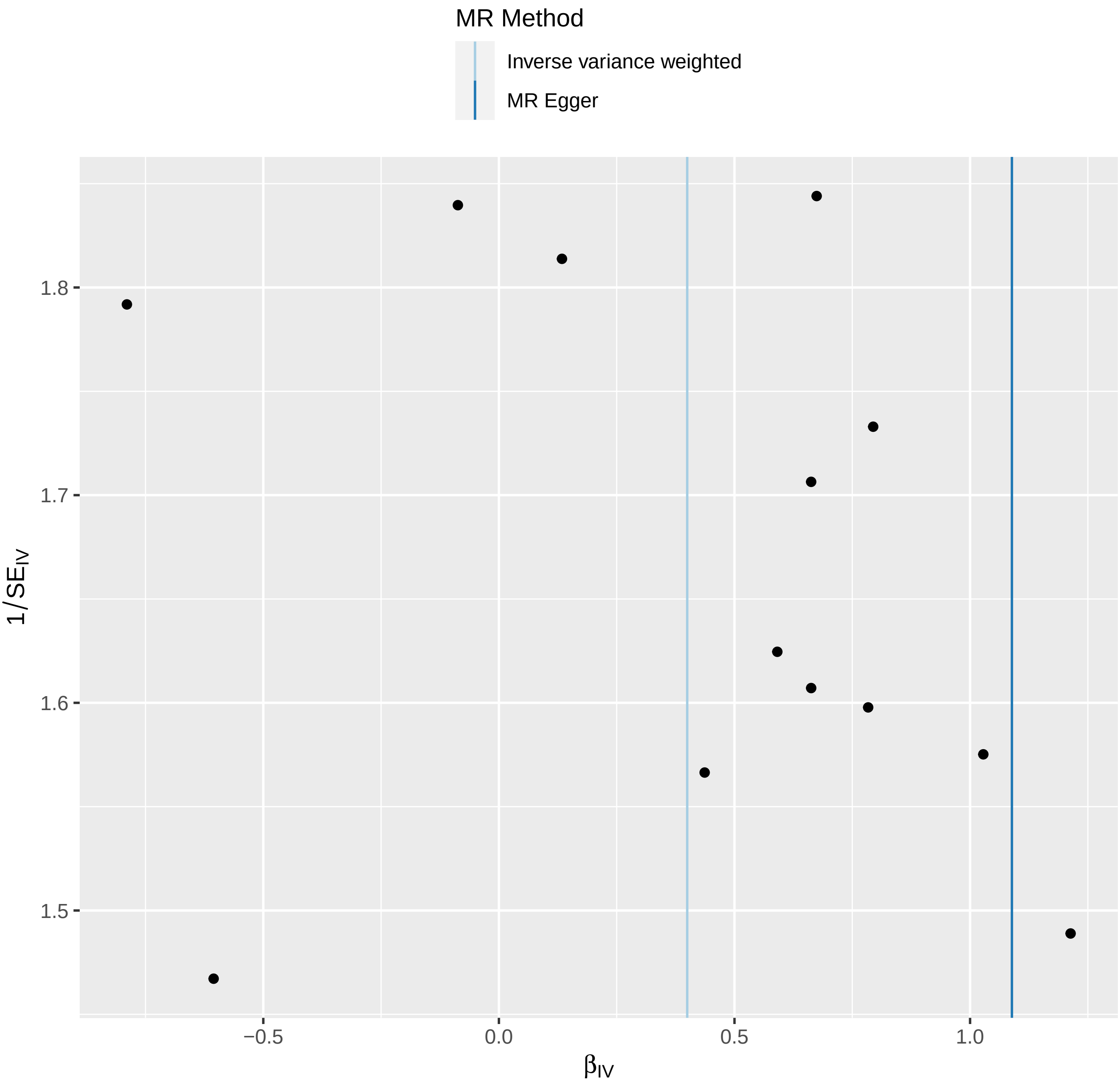

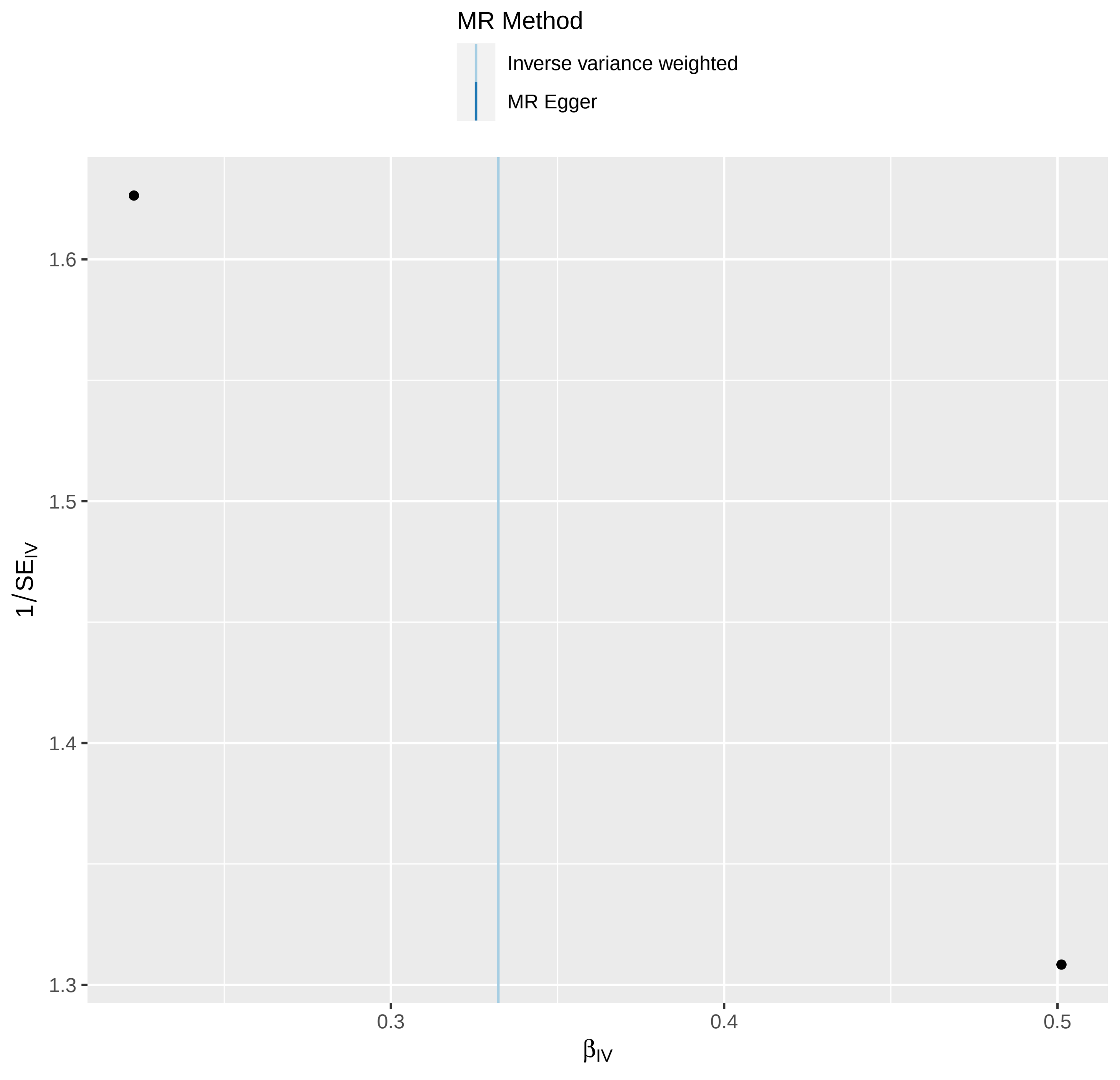
MR Method
Inverse variance weighted
MR Egger
1.6
1.5
1.4
1.3
1/SE_IV
0.3
0.4
0.5
β_IV

Batch 47 : Gut microbiota abundance (genus Butyricicoccus id.2055) on Graves' disease

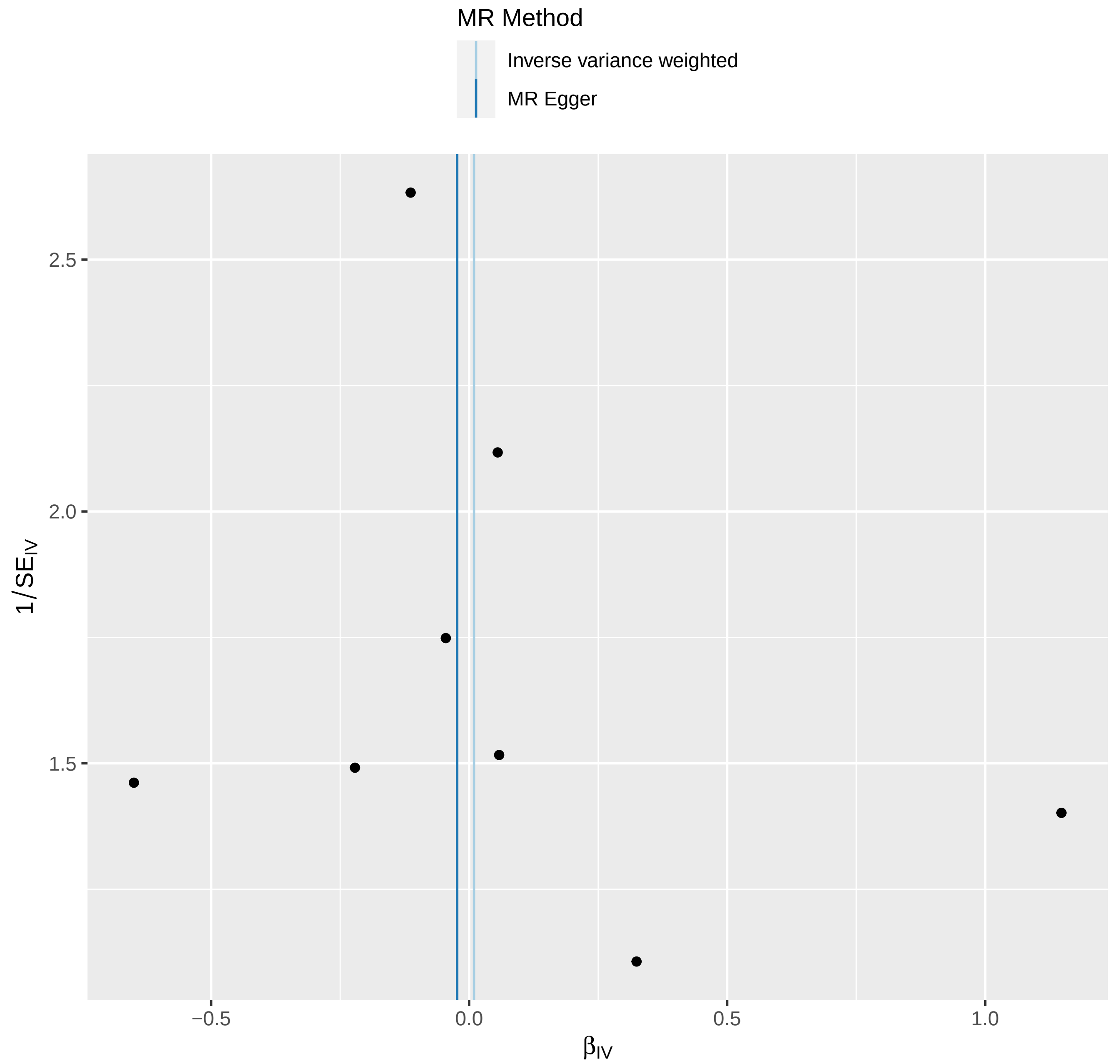

Batch 48 : Gut microbiota abundance (genus Butyricimonas id.945) on Graves' disease

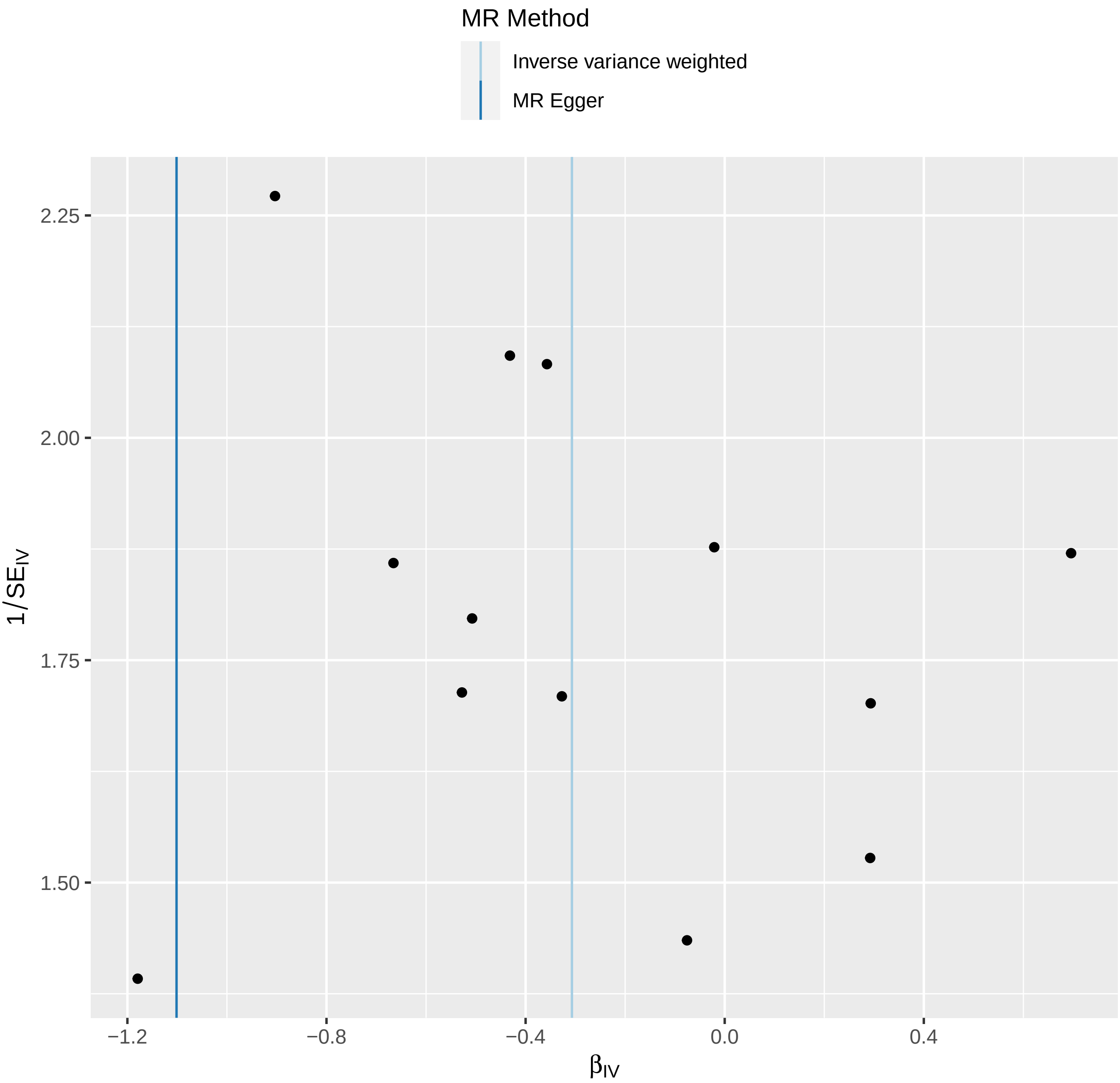

Batch 49 : Gut microbiota abundance (genus Butyrivibrio id.1993) on Graves' disease

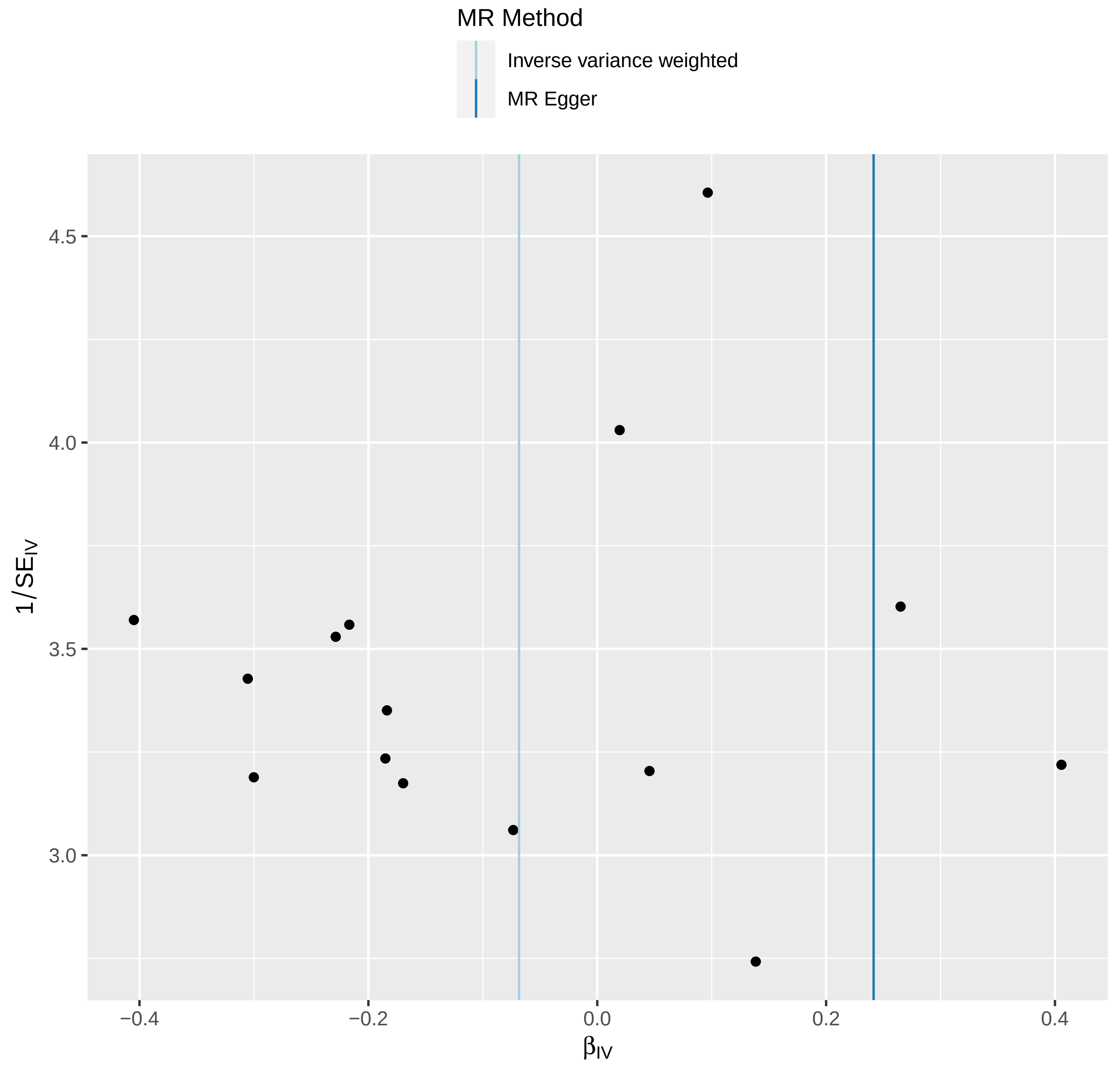

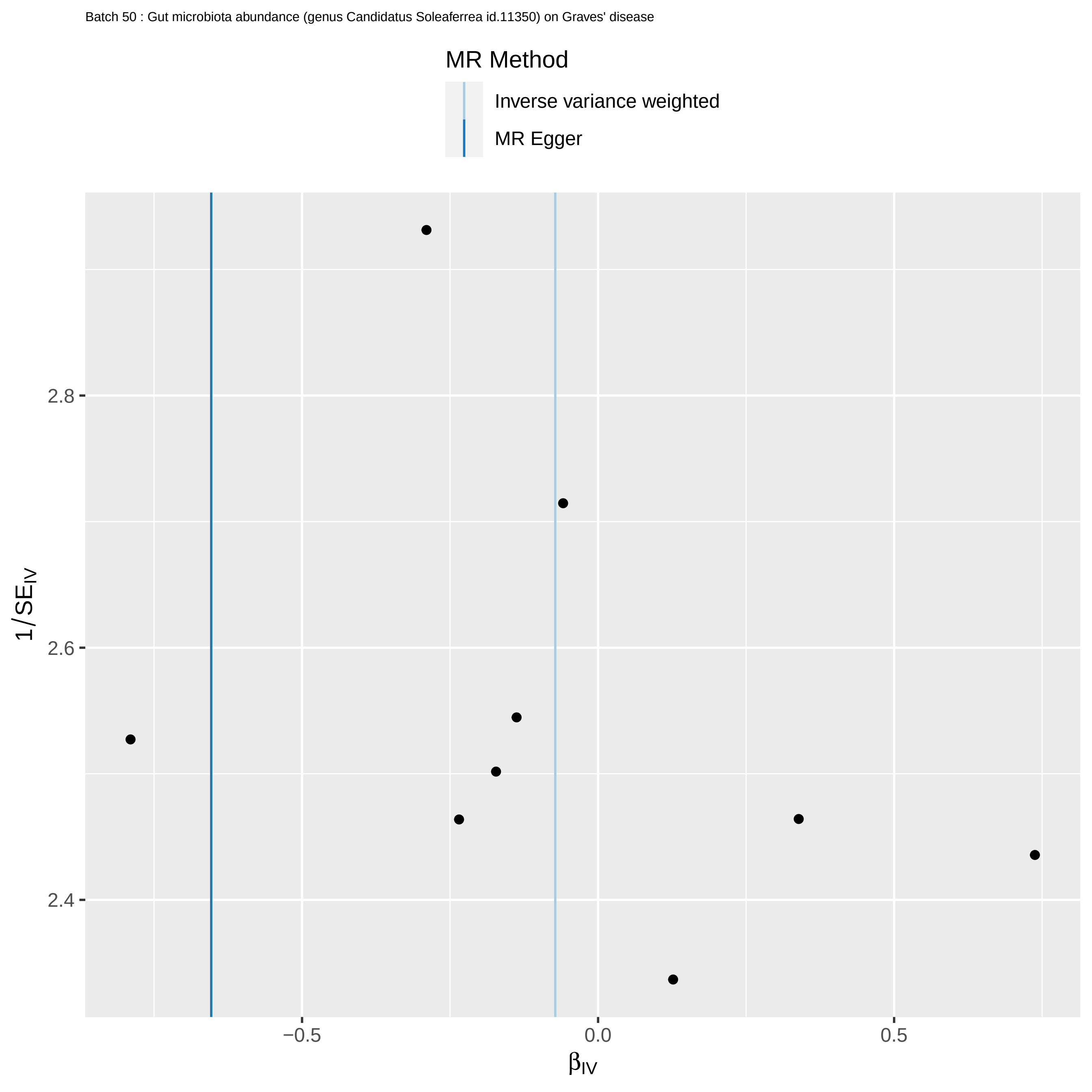
Batch 50 : Gut microbiota abundance (genus Candidatus Soleaferrea id.11350) on Graves' disease
MR Method
Inverse variance weighted
MR Egger
2.8
2.6
2.4
1/SE_IV
−0.5
0.0
0.5
β_IV

Batch 51 : Gut microbiota abundance (genus Catenibacterium id.2153) on Graves' disease

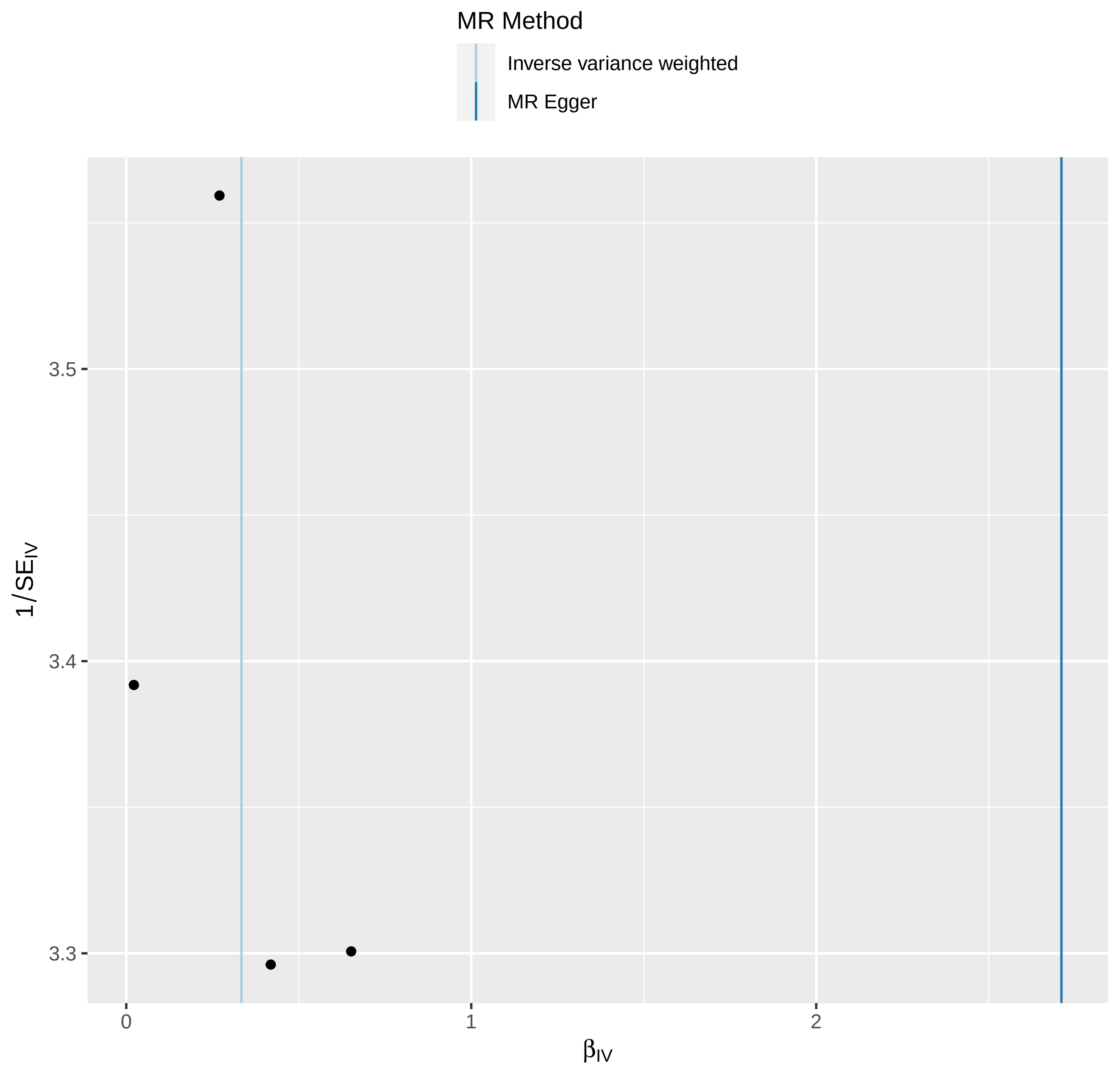

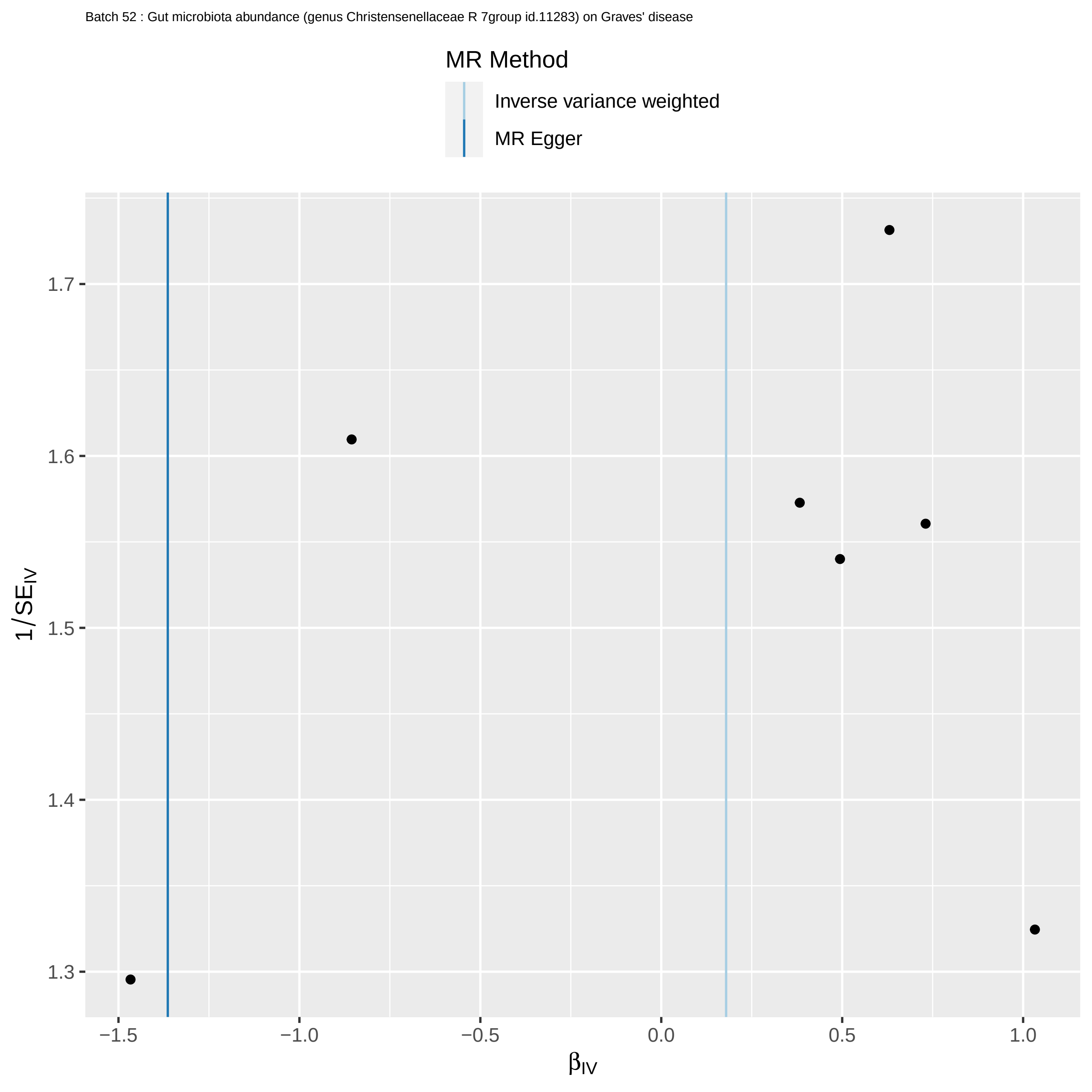

Batch 52 : Gut microbiota abundance (genus Christensenellaceae R 7group id.11283) on Graves' disease
MR Method
Inverse variance weighted
MR Egger
1.7
1.6
1.5
1.4
1.3
1/SE_IV
−1.5
−1.0
−0.5
0.0
0.5
1.0
β_IV

Batch 53 : Gut microbiota abundance (genus Clostridium innocuum group id.14397) on Graves' disease

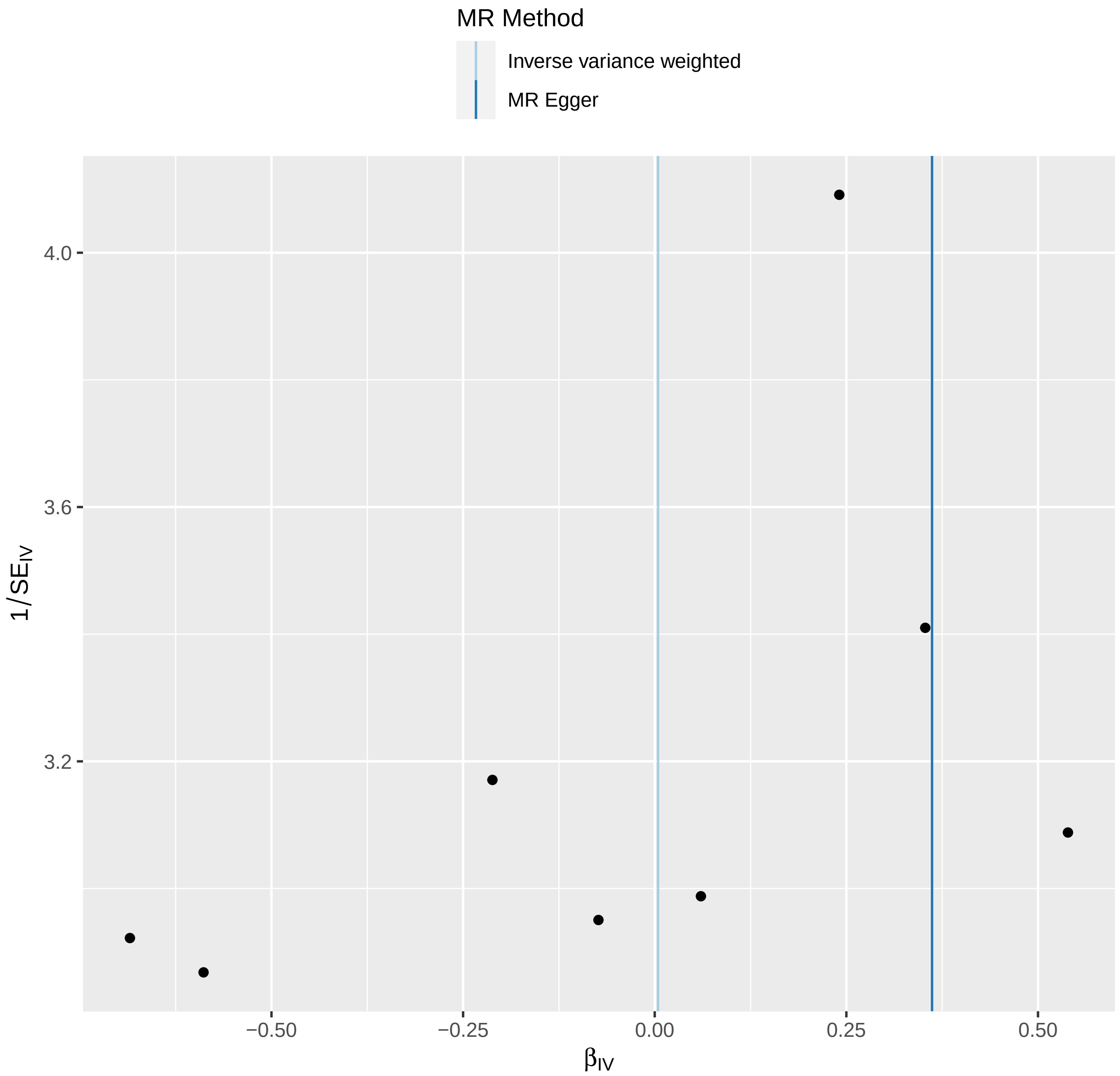

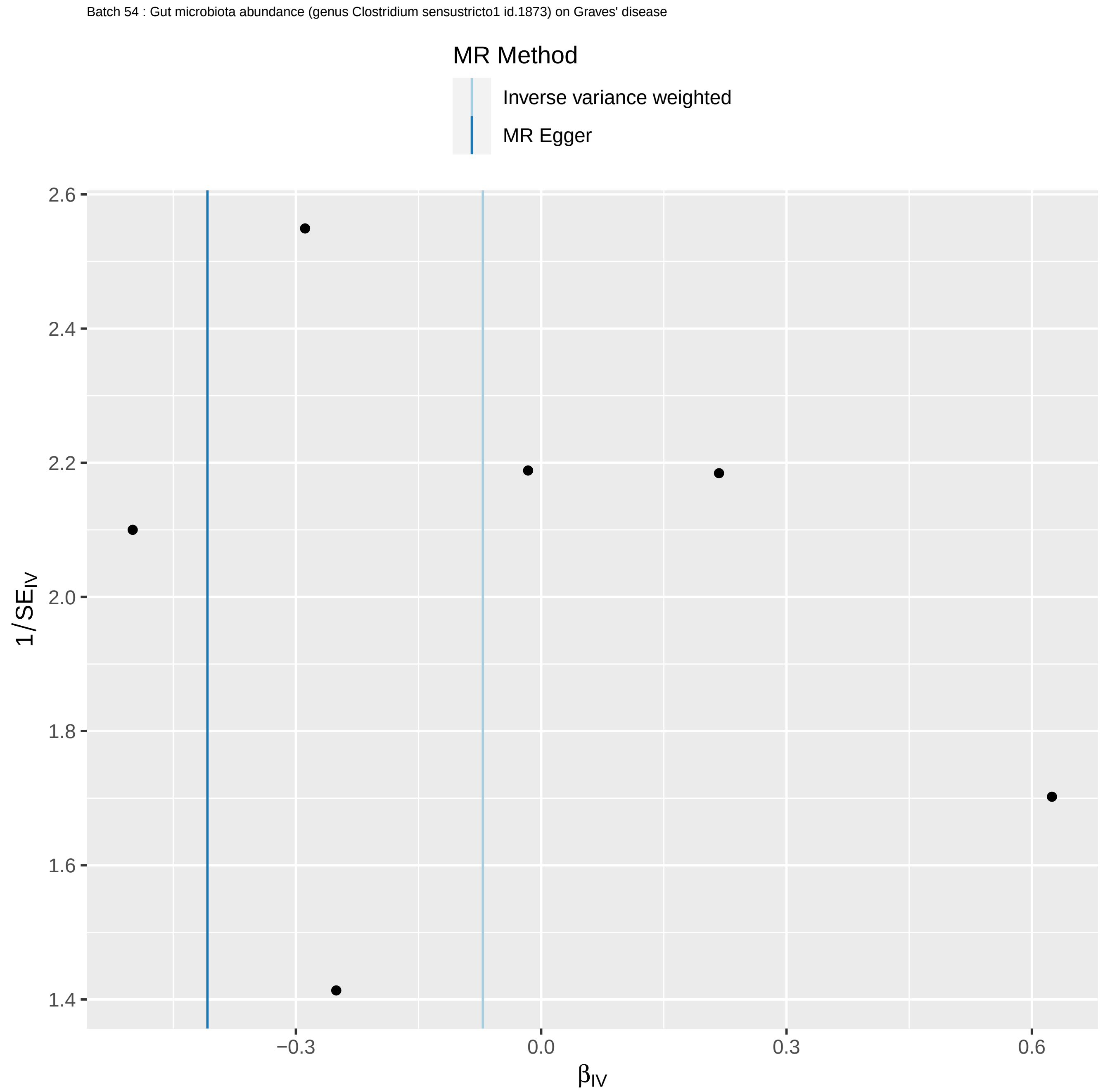

Batch 54 : Gut microbiota abundance (genus Clostridium sensustricto1 id.1873) on Graves' disease
MR Method
Inverse variance weighted
MR Egger
2.6
2.4
2.2
2.0
1.8
1.6
1.4
1/SE_IV
−0.3
0.0
0.3
0.6
β_IV

Batch 55 : Gut microbiota abundance (genus Collinsella id.815) on Graves' disease

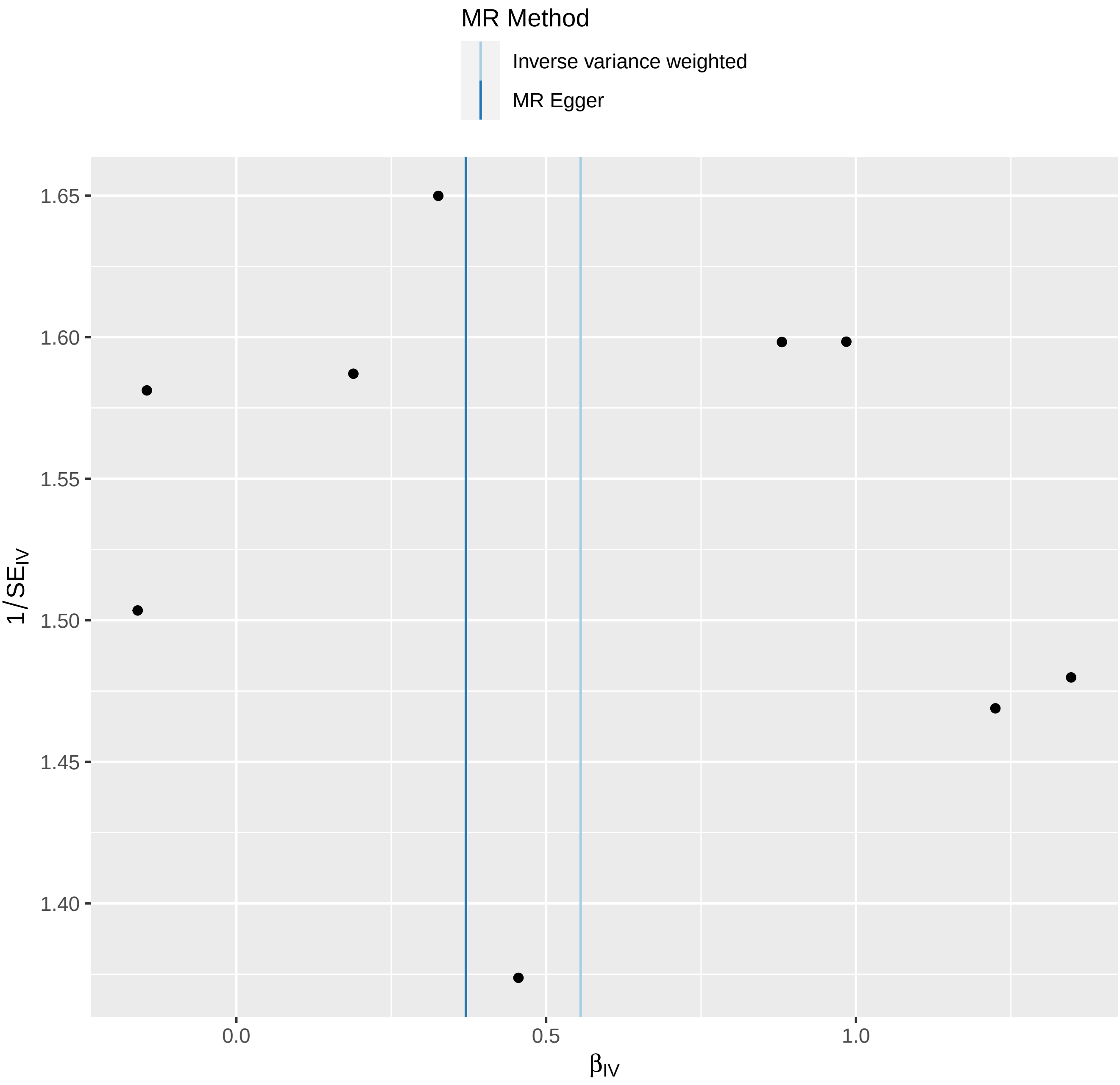

Batch 56 : Gut microbiota abundance (genus Coprobacter id.949) on Graves' disease

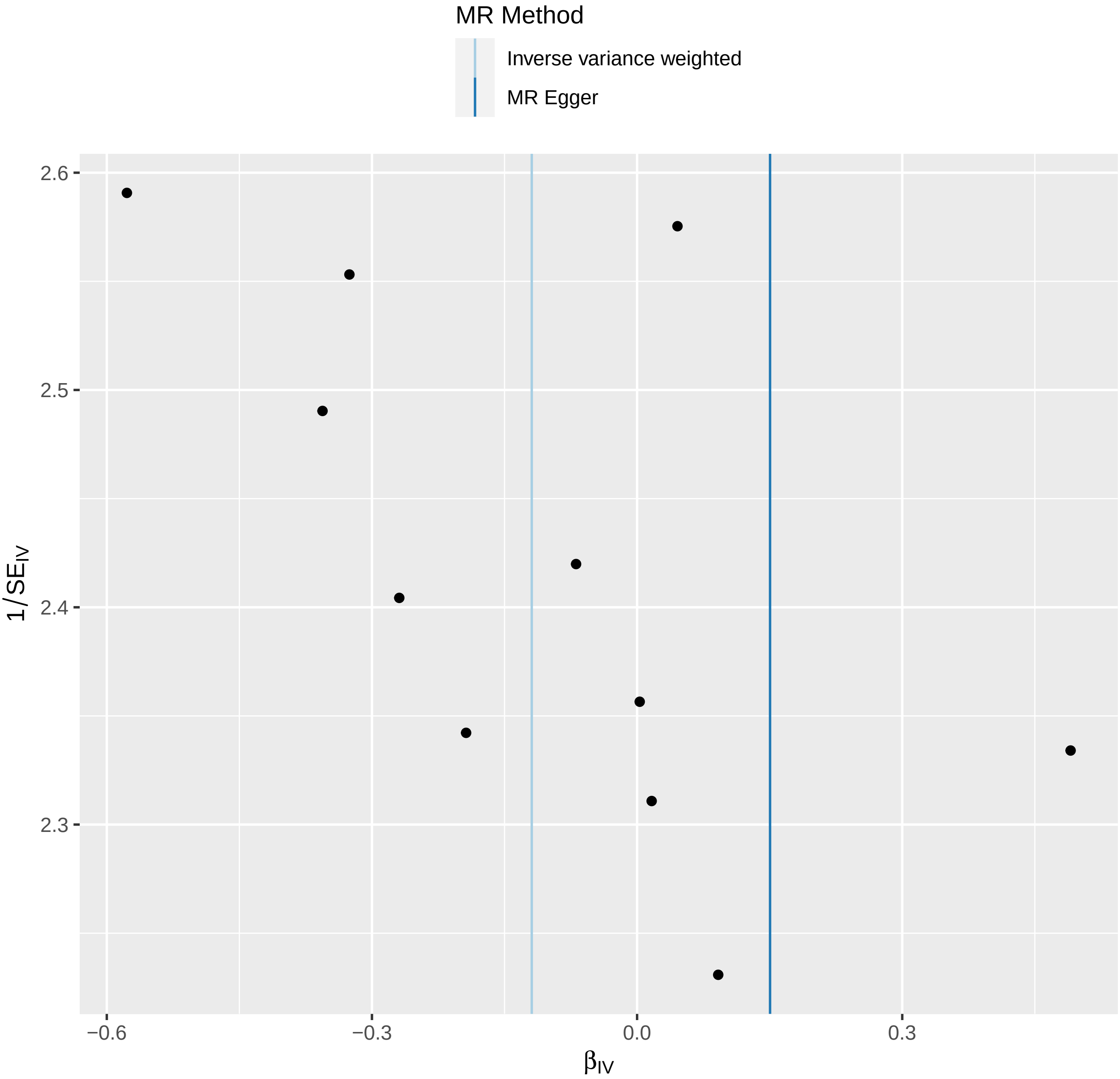

Batch 57 : Gut microbiota abundance (genus Coprococcus1 id.11301) on Graves' disease

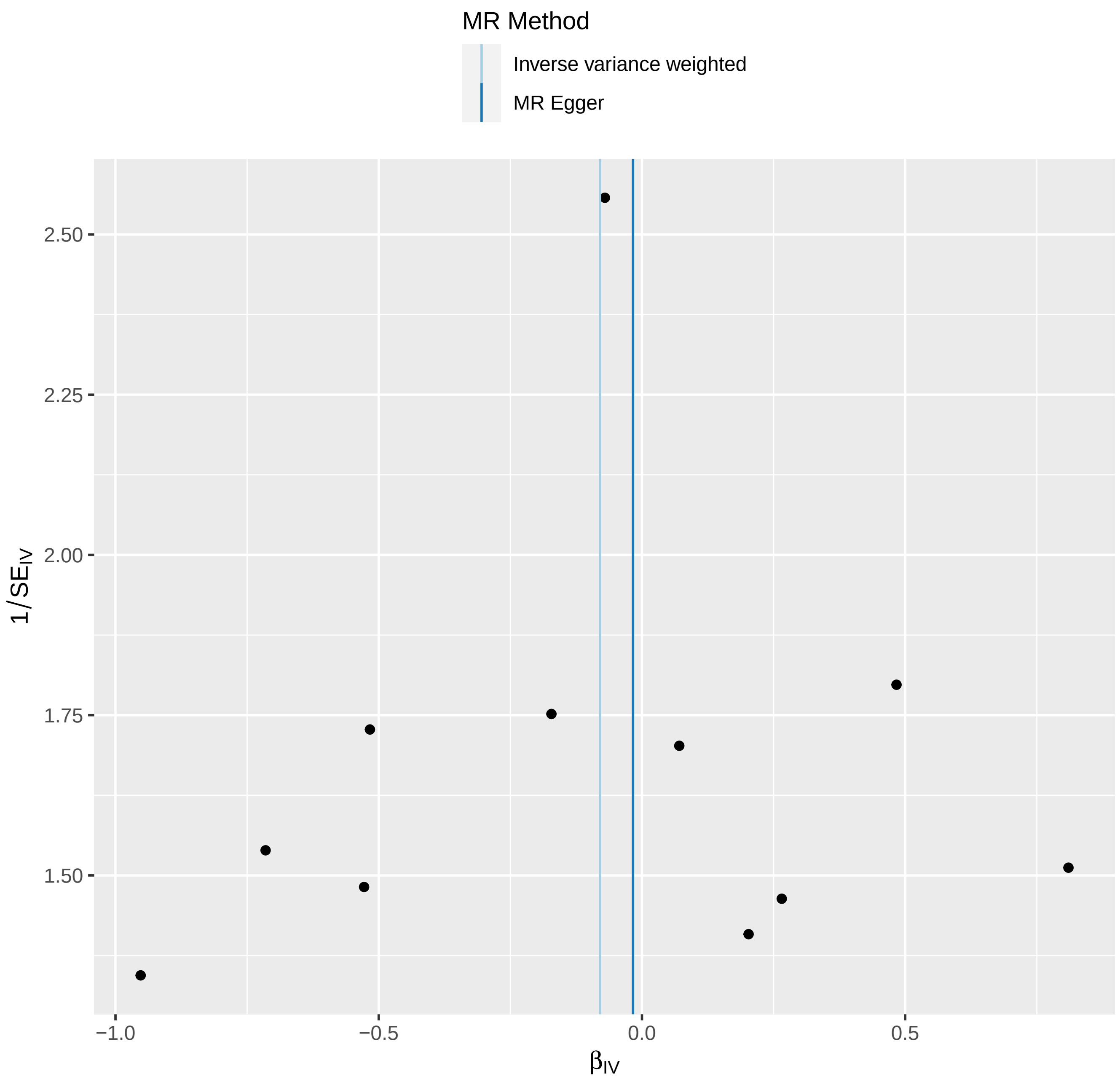

Batch 58 : Gut microbiota abundance (genus Coprococcus2 id.11302) on Graves' disease

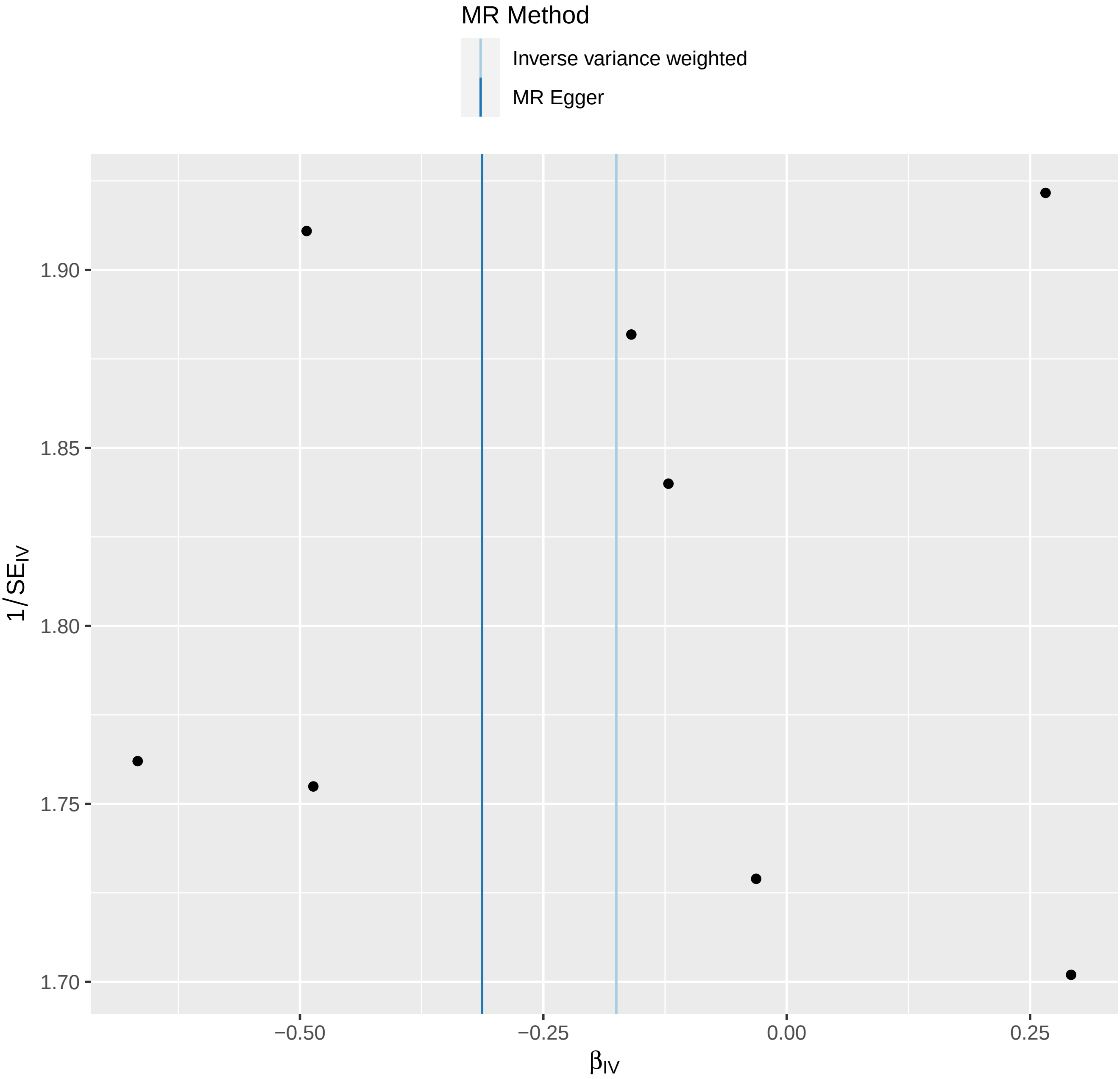

Batch 59 : Gut microbiota abundance (genus Coprococcus3 id.11303) on Graves' disease

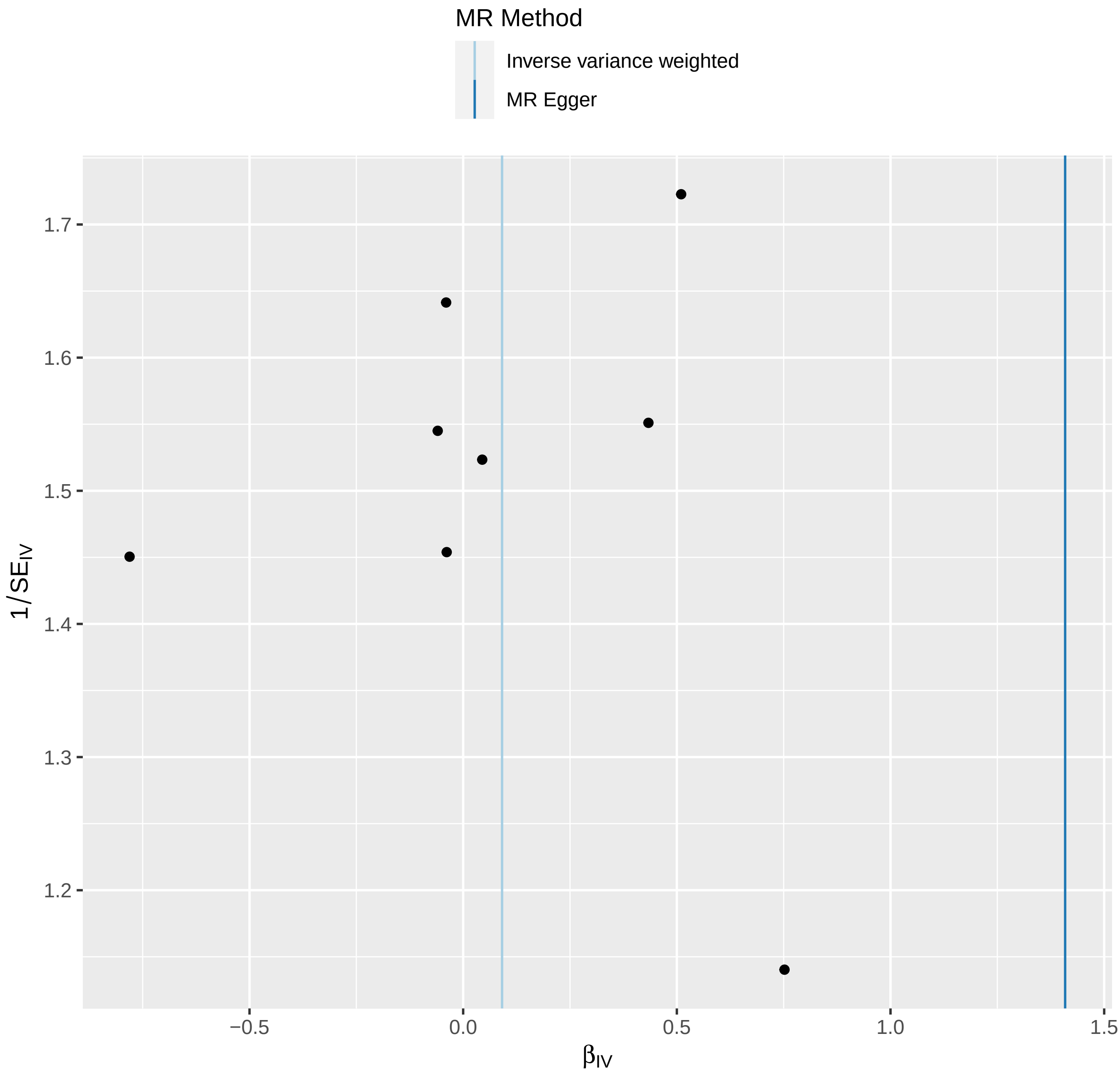

Batch 60 : Gut microbiota abundance (genus Defluviitaleaceae UCG011 id.11287) on Graves' disease

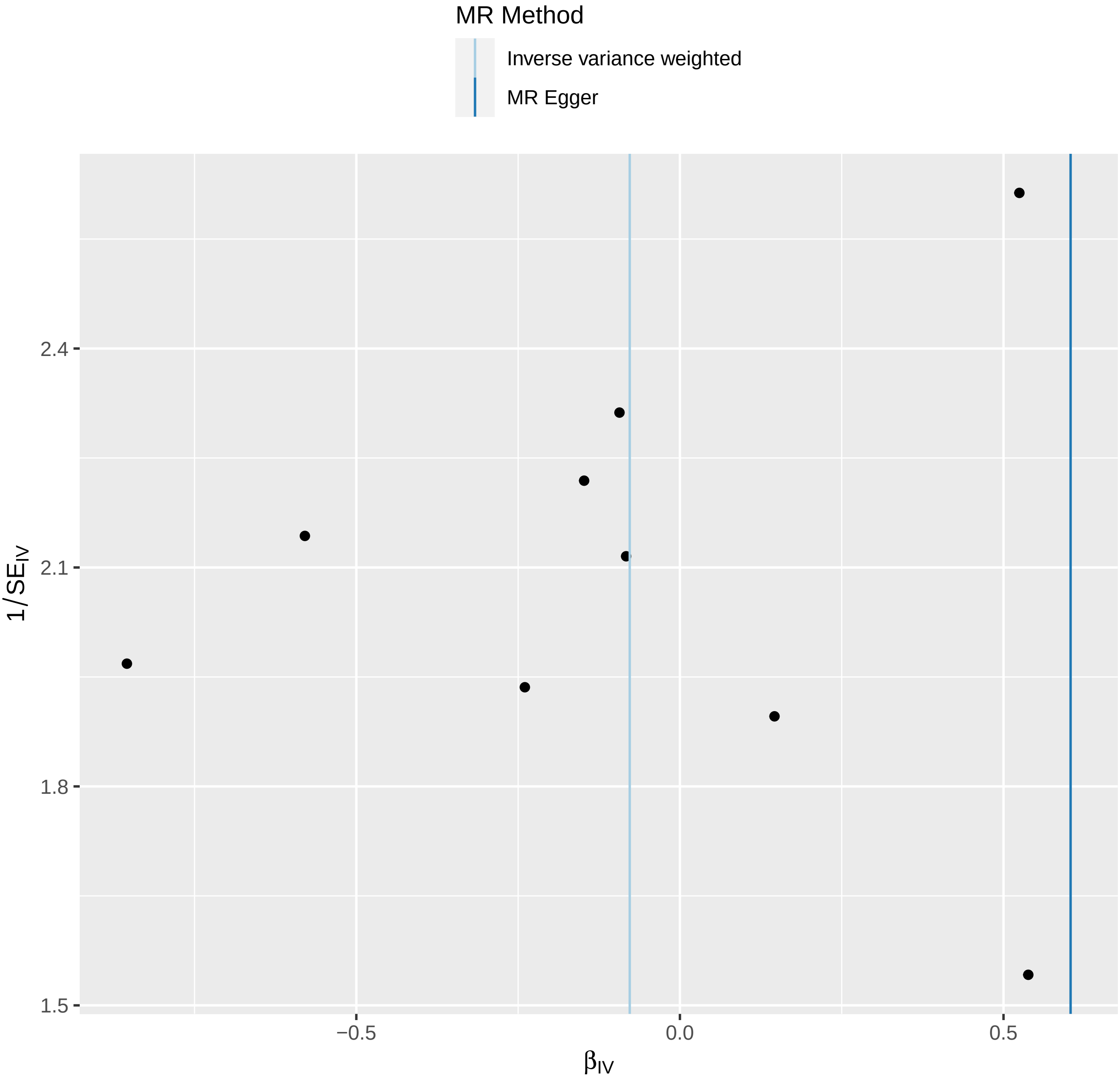

Batch 61 : Gut microbiota abundance (genus Desulfovibrio id.3173) on Graves' disease

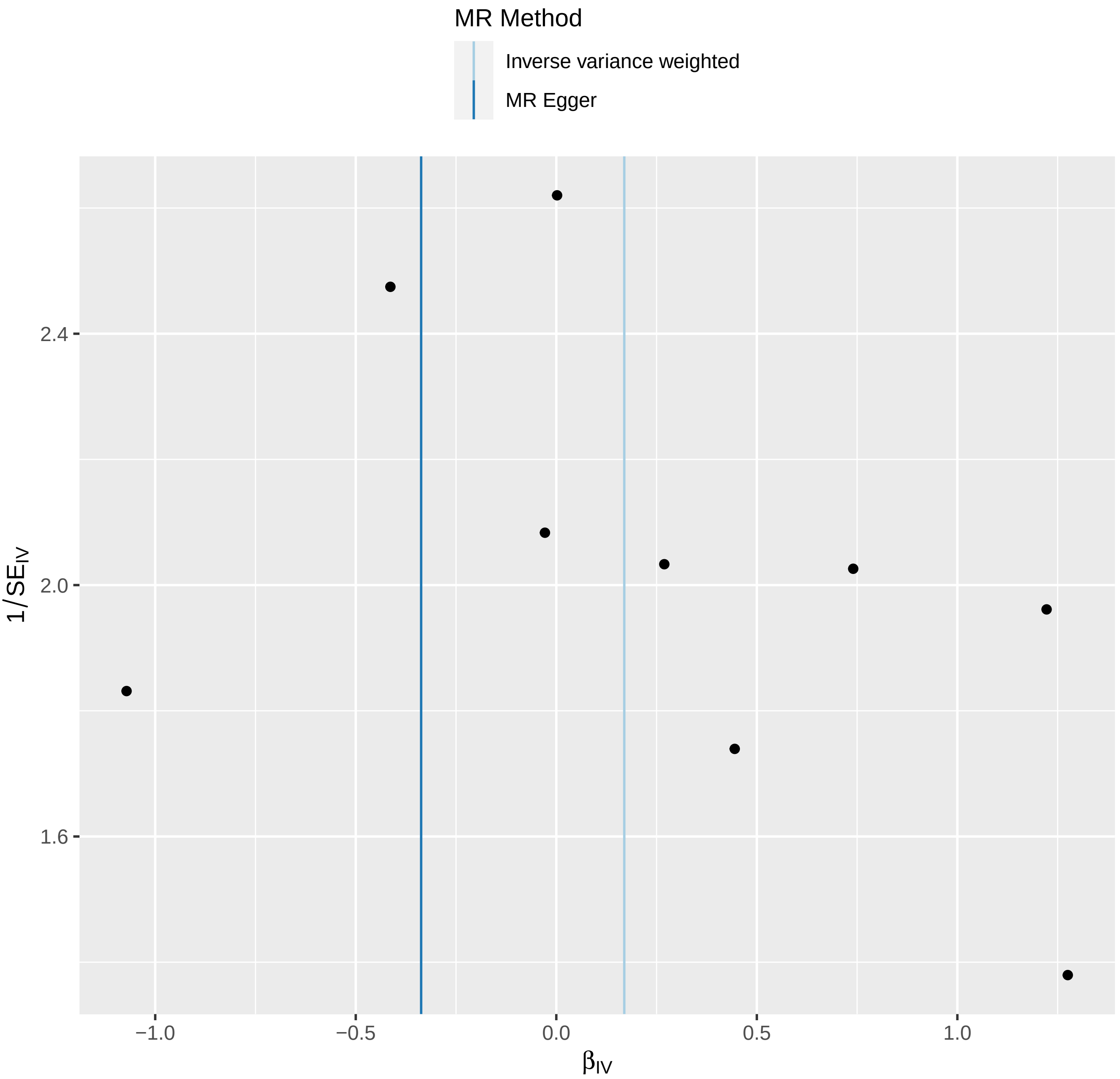

Batch 62 : Gut microbiota abundance (genus Dialister id.2183) on Graves' disease

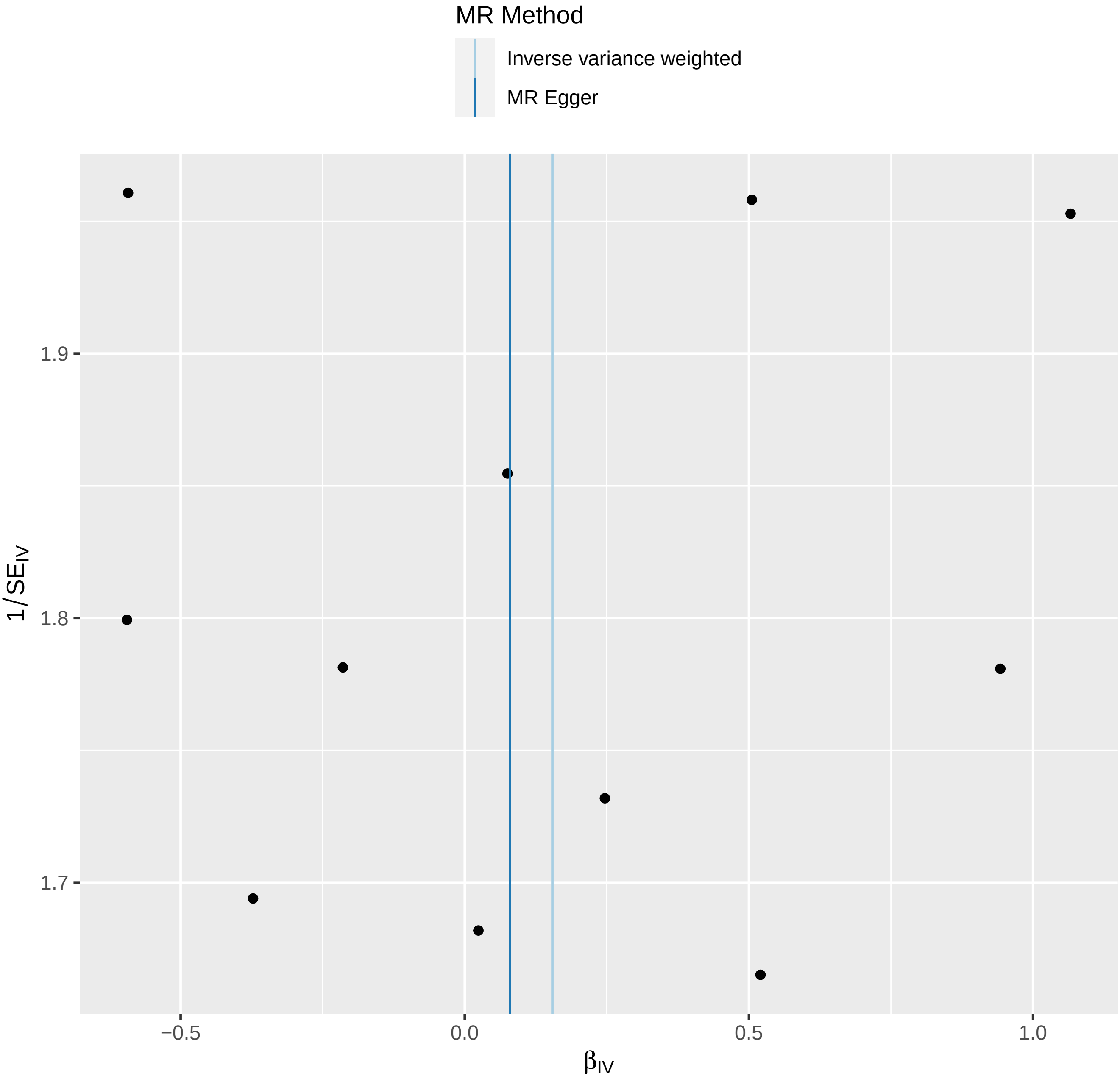

Batch 63 : Gut microbiota abundance (genus Dorea id.1997) on Graves' disease

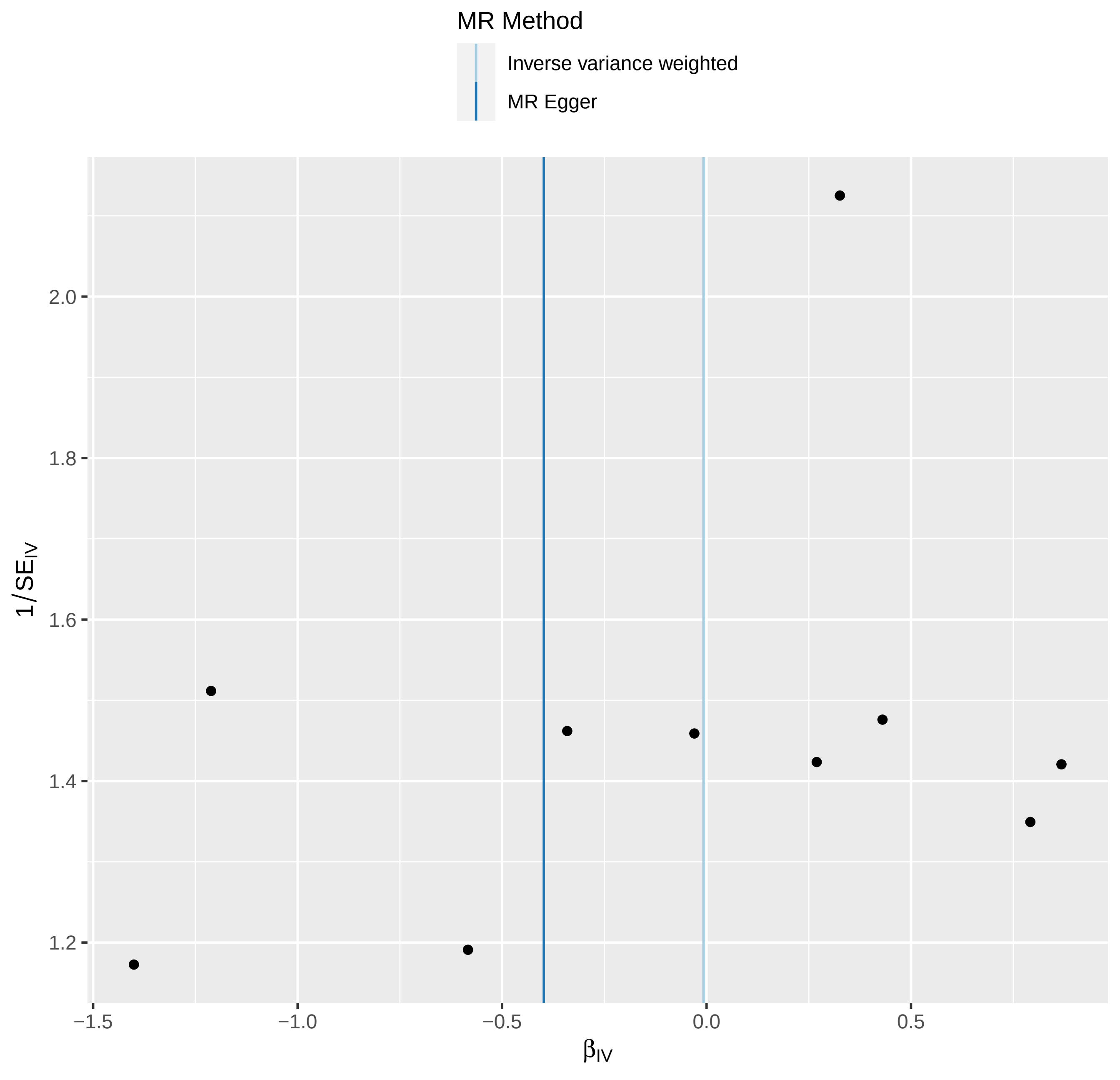

Batch 64 : Gut microbiota abundance (genus Eggerthella id.819) on Graves' disease

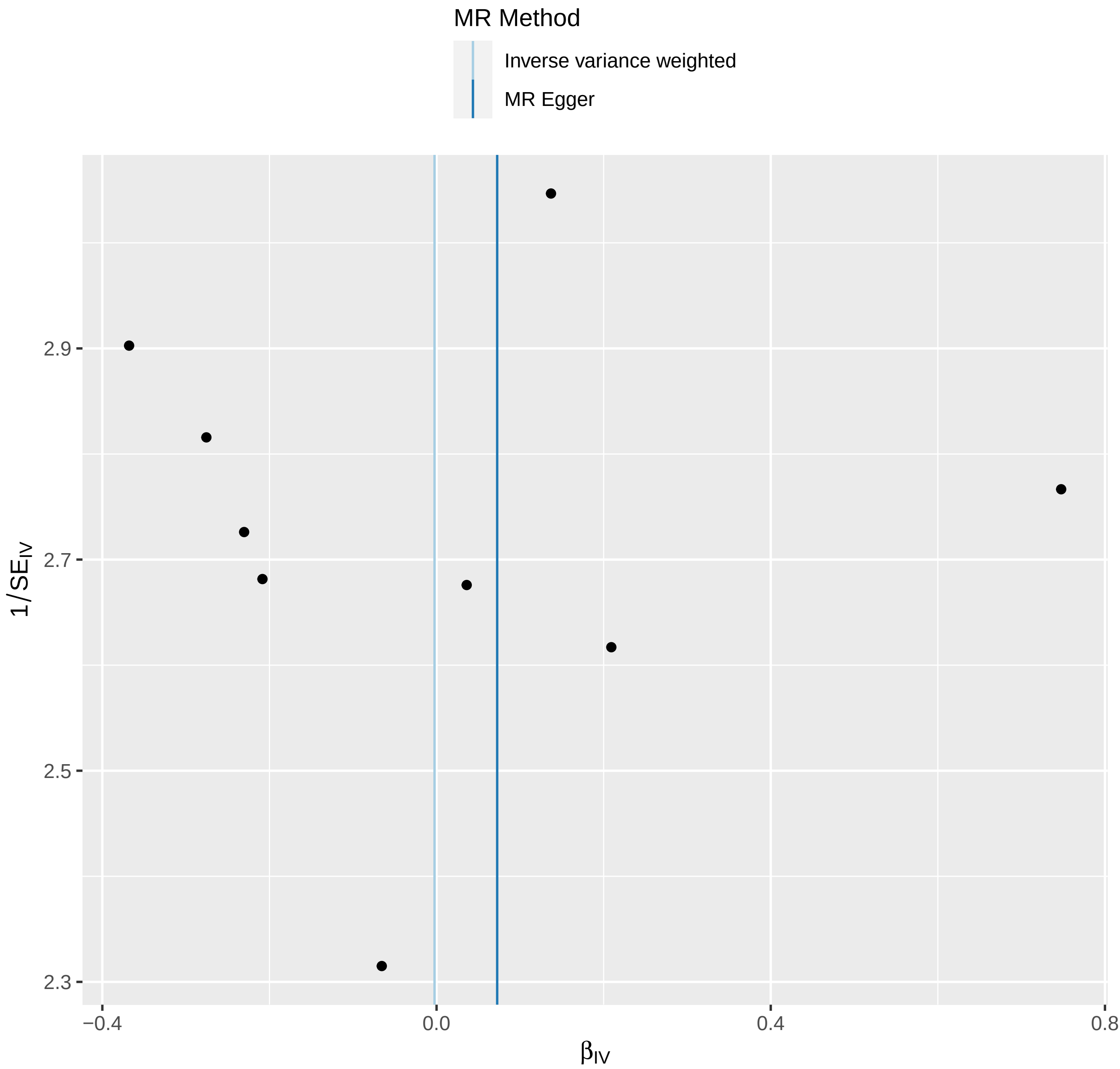

Batch 65 : Gut microbiota abundance (genus Eisenbergiella id.11304) on Graves' disease

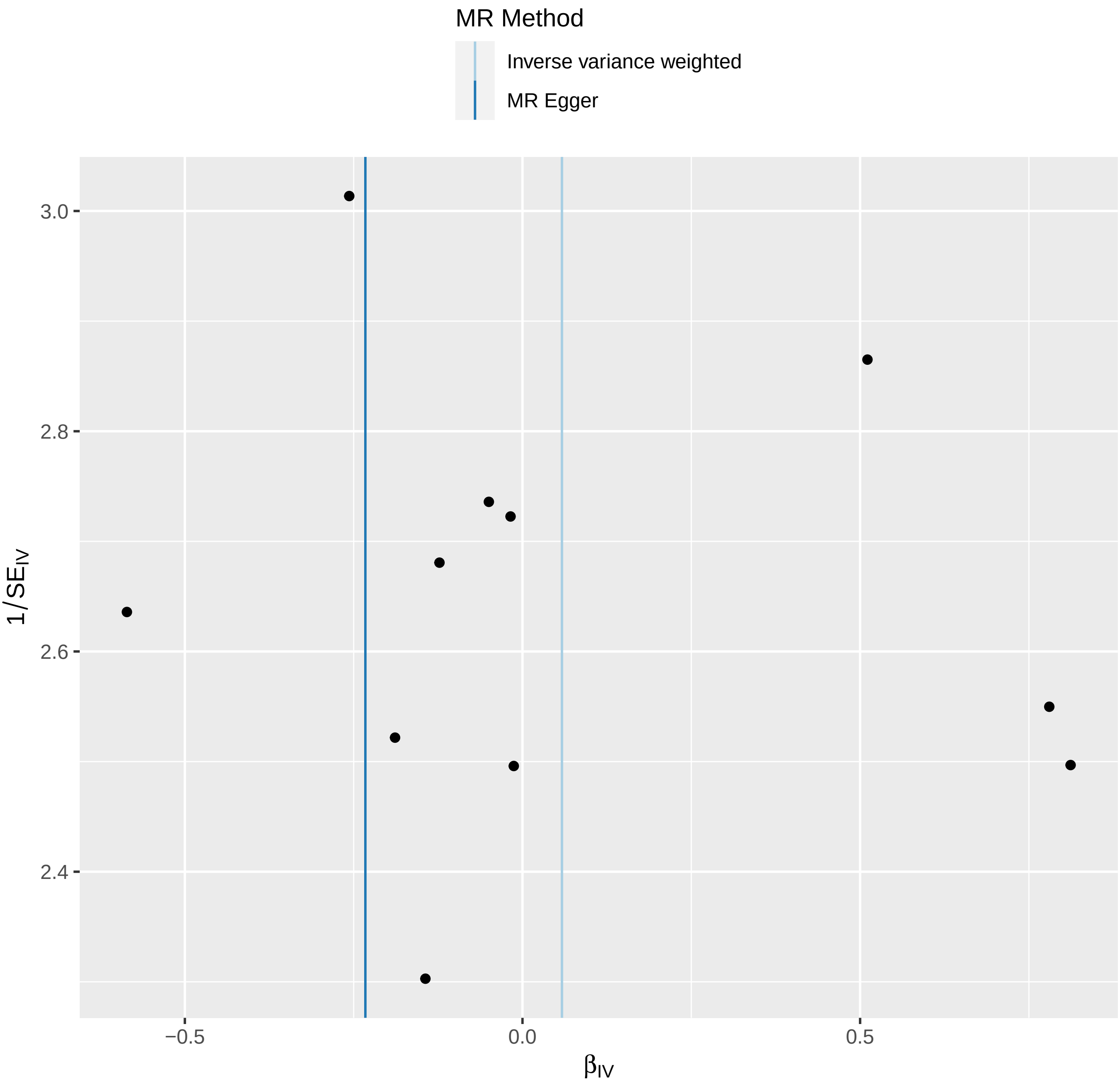

Batch 66 : Gut microbiota abundance (genus Enterorhabdus id.820) on Graves' disease

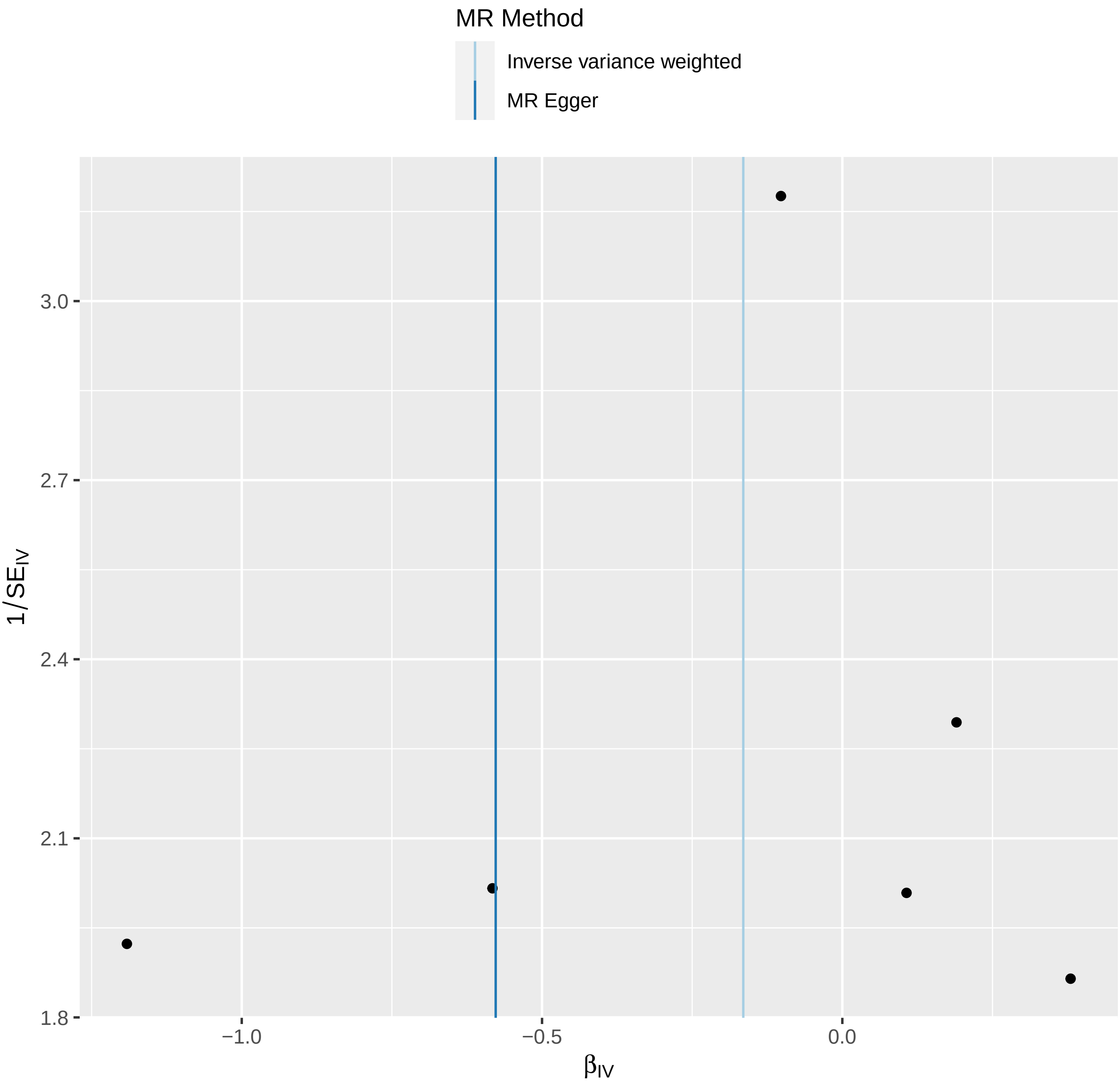

Batch 67 : Gut microbiota abundance (genus Erysipelatoclostridium id.11381) on Graves' disease

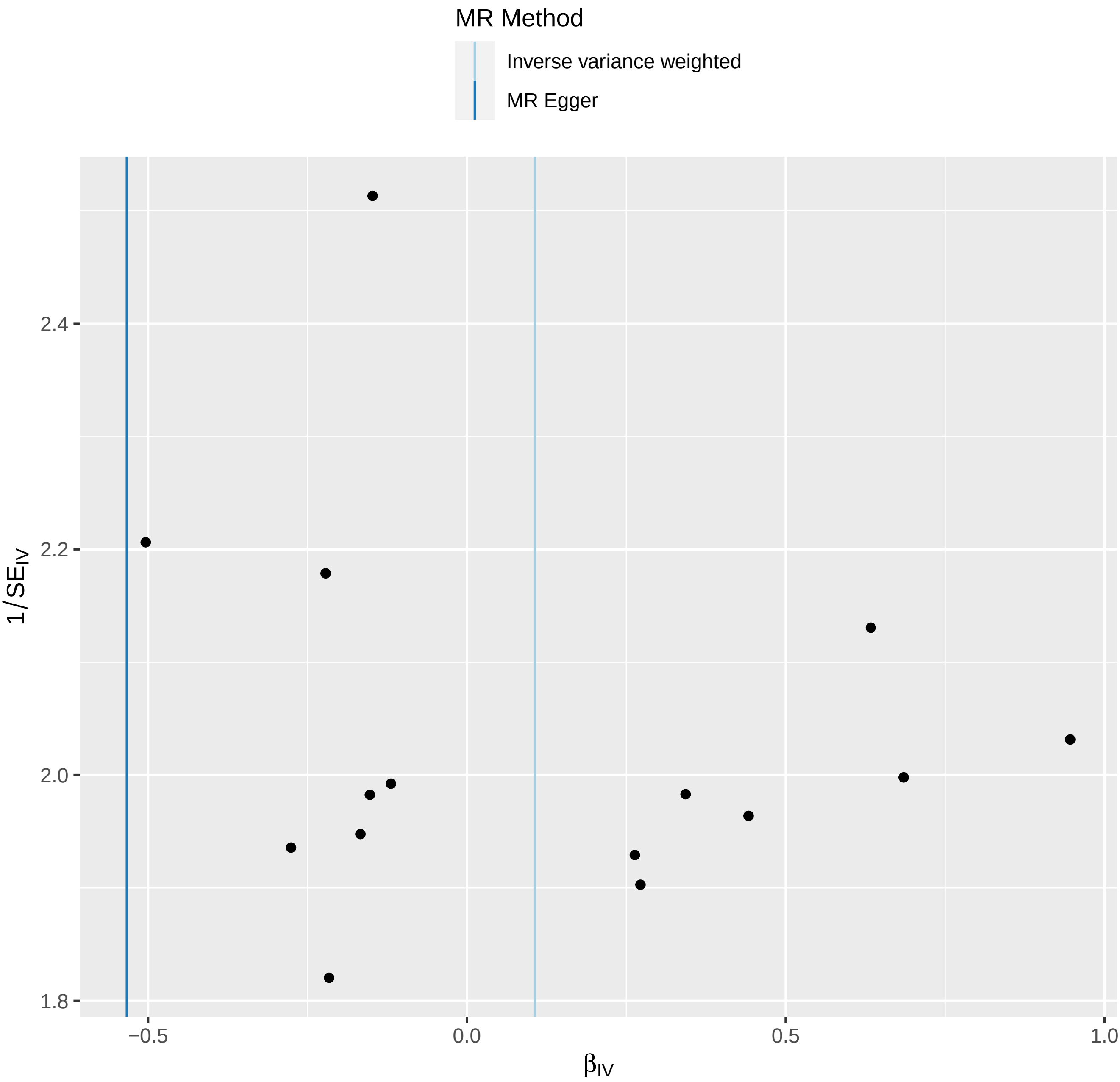

Batch 69 : Gut microbiota abundance (genus Escherichia Shigella id.3504) on Graves' disease

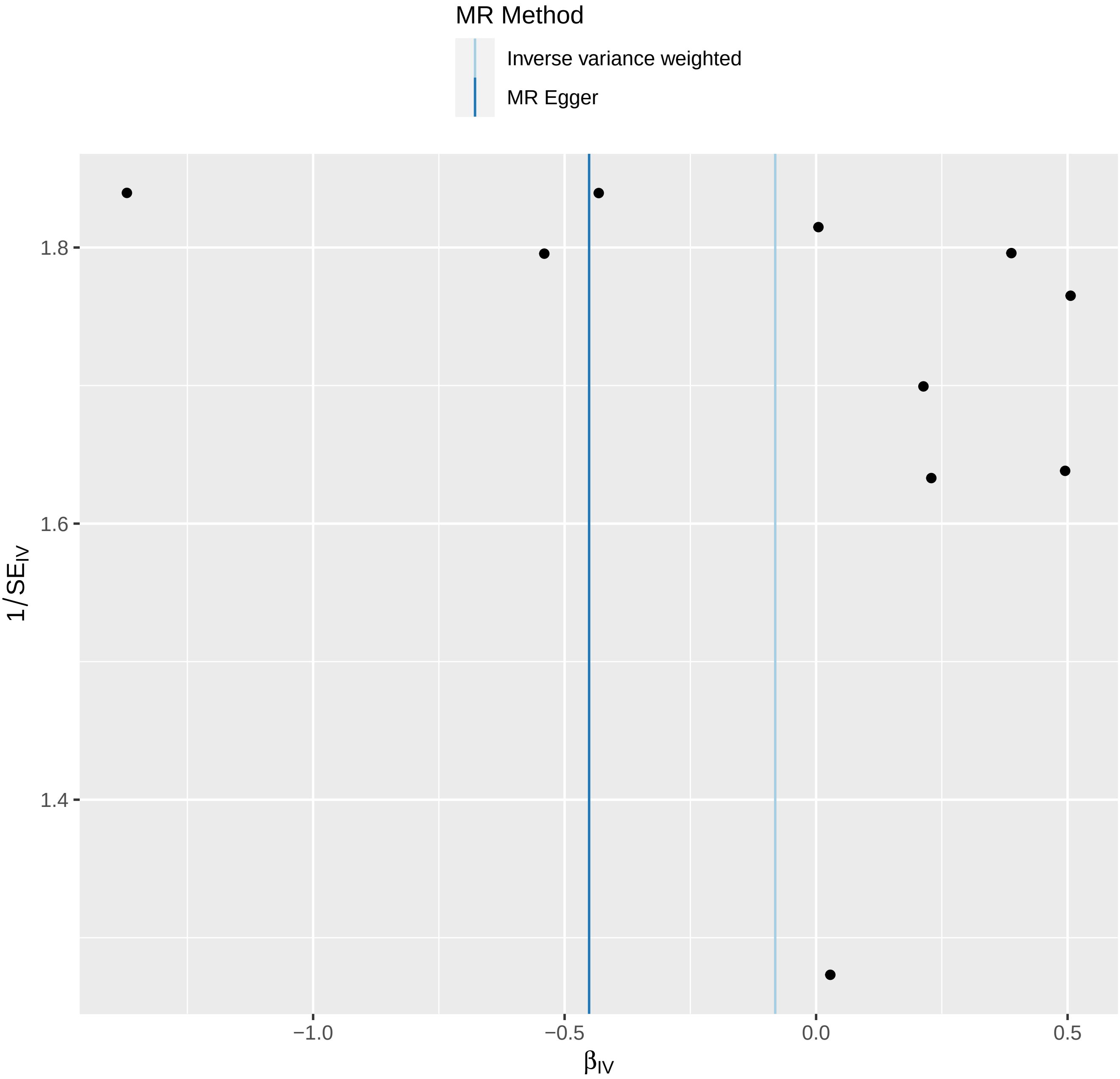

Batch 70 : Gut microbiota abundance (genus Eubacterium brachy group id.11296) on Graves' disease

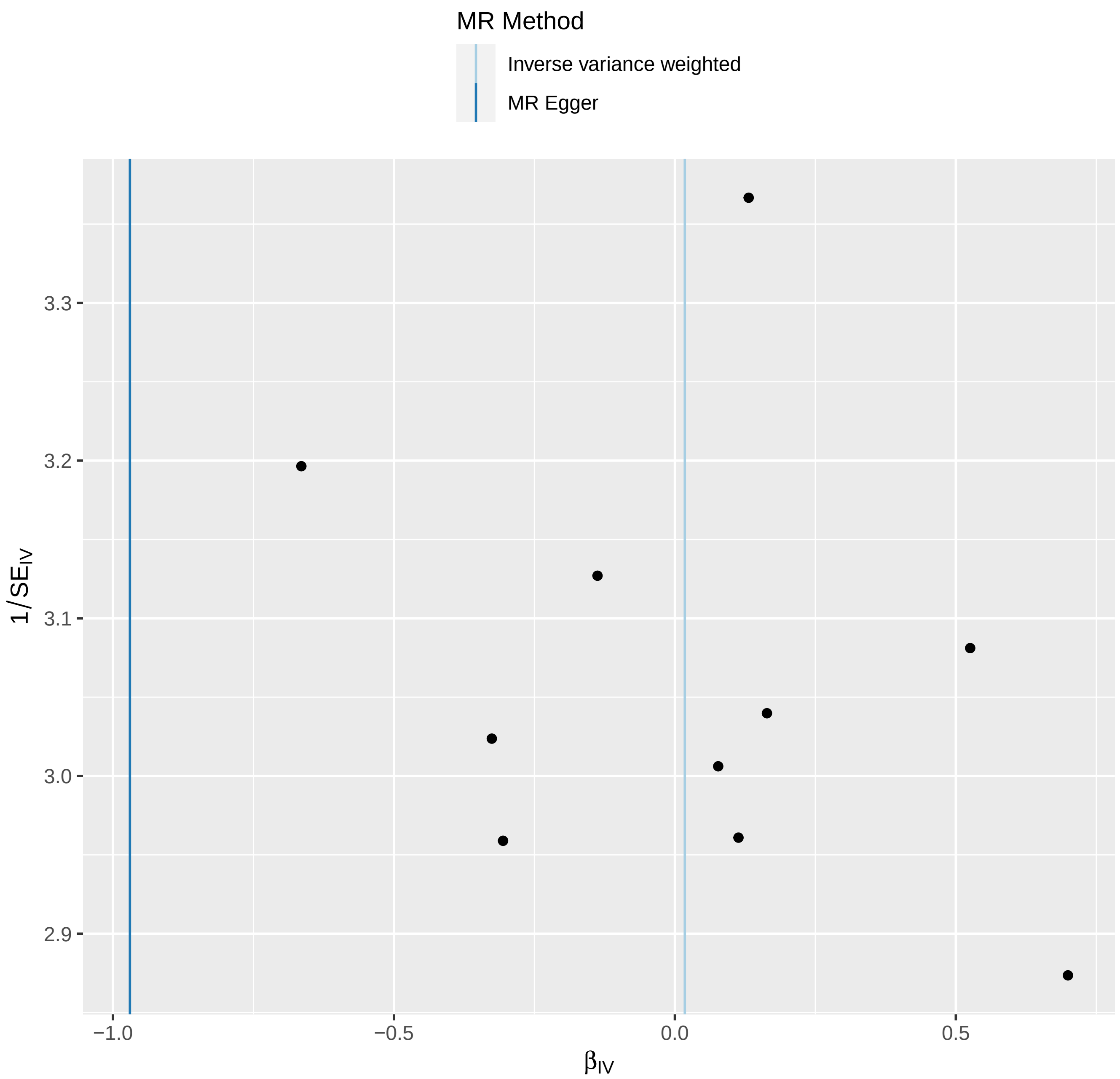

Batch 71 : Gut microbiota abundance (genus Eubacterium coprostanoligenes group id.11375) on Graves' disease

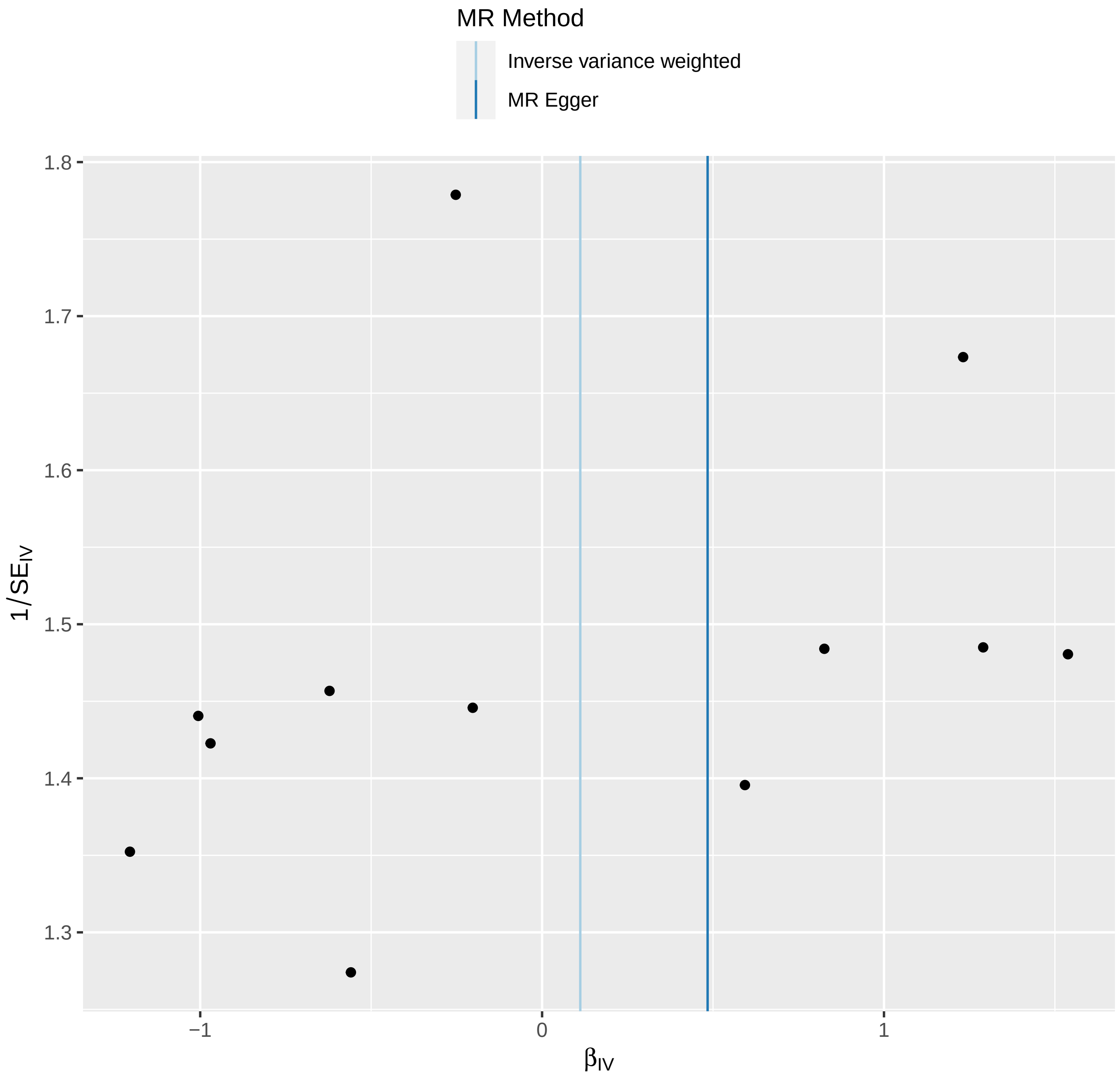

Batch 72 : Gut microbiota abundance (genus Eubacterium eligens group id.14372) on Graves' disease

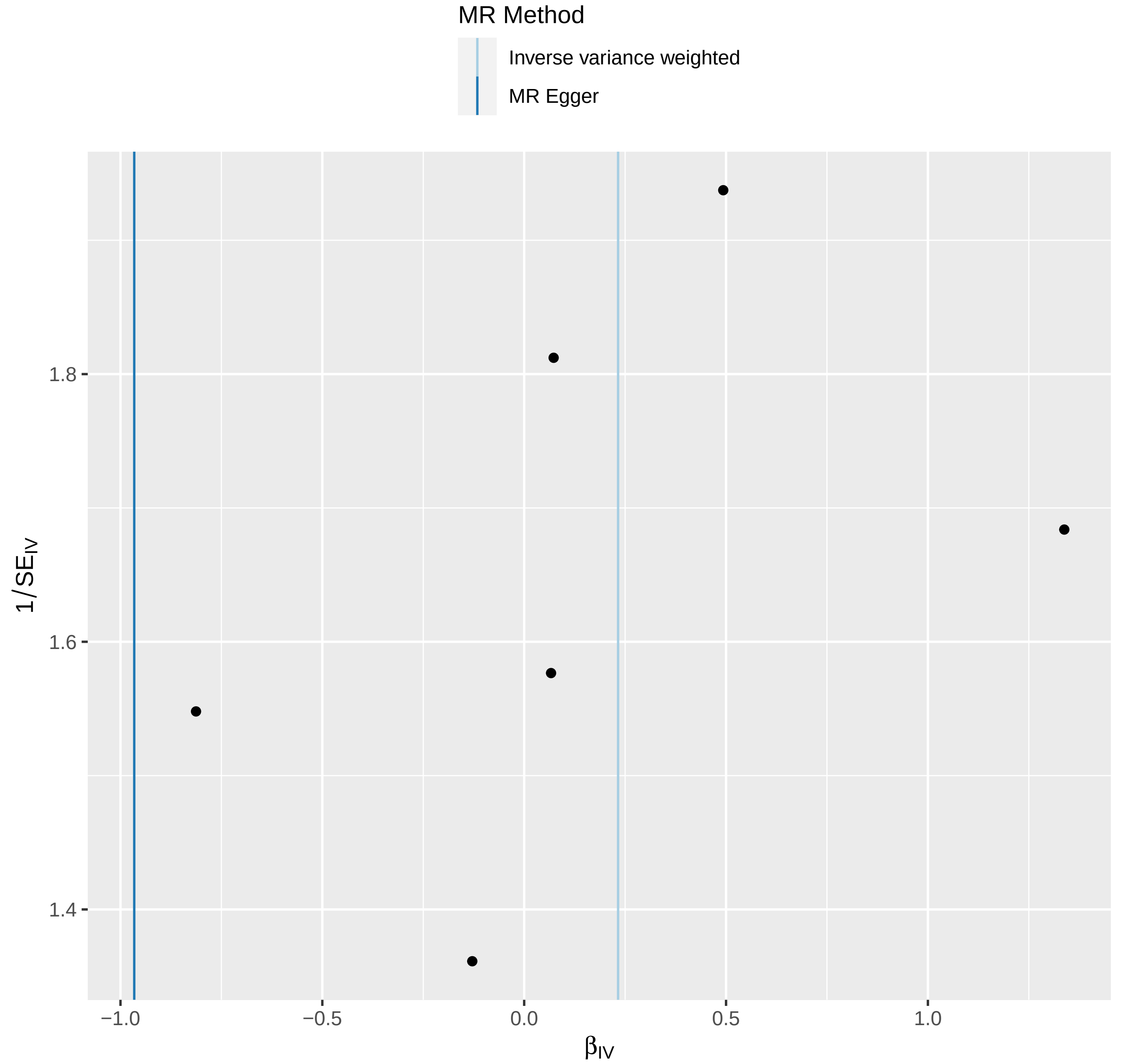

Batch 73 : Gut microbiota abundance (genus Eubacterium fissicatena group id.14373) on Graves' disease

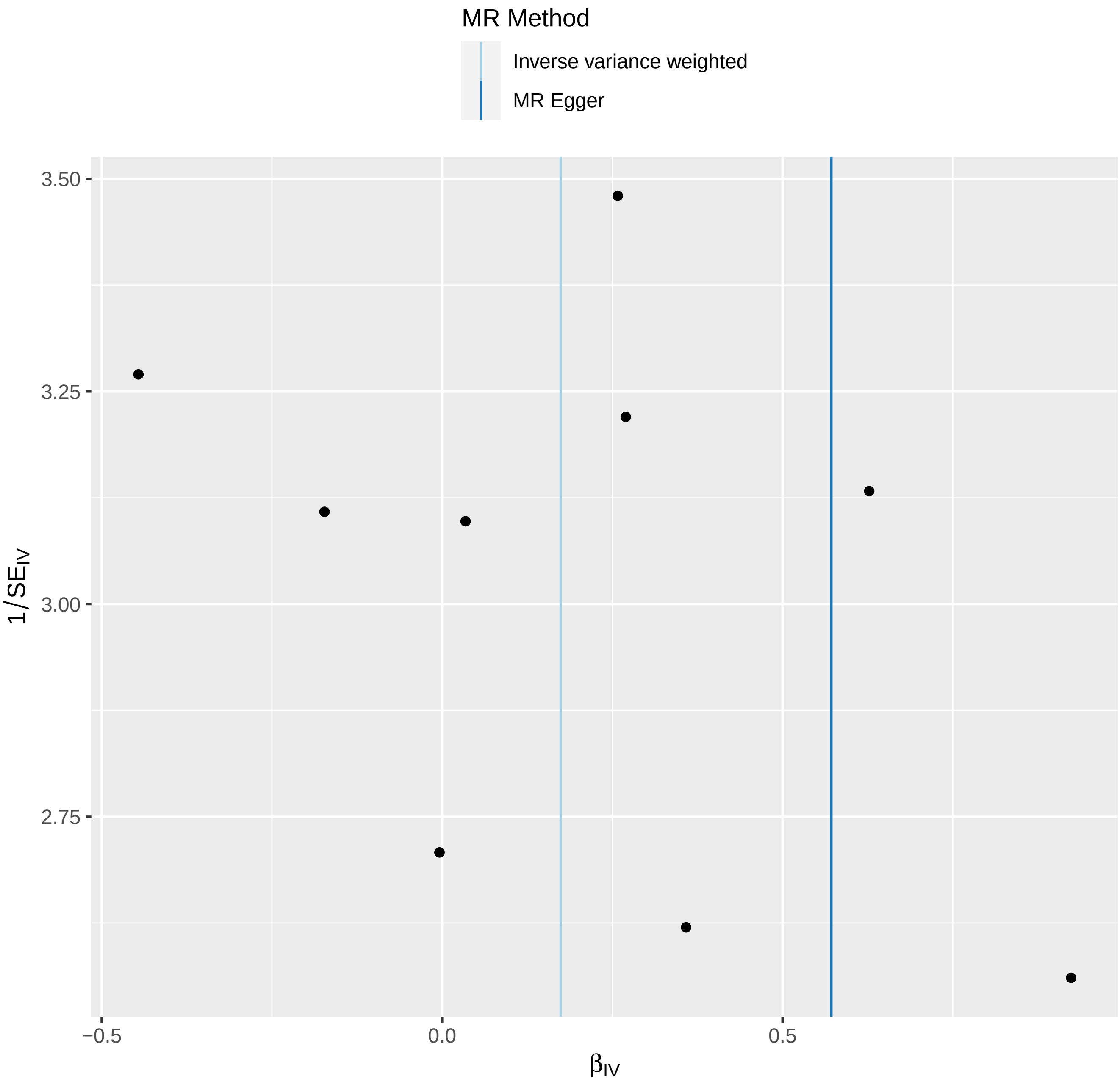

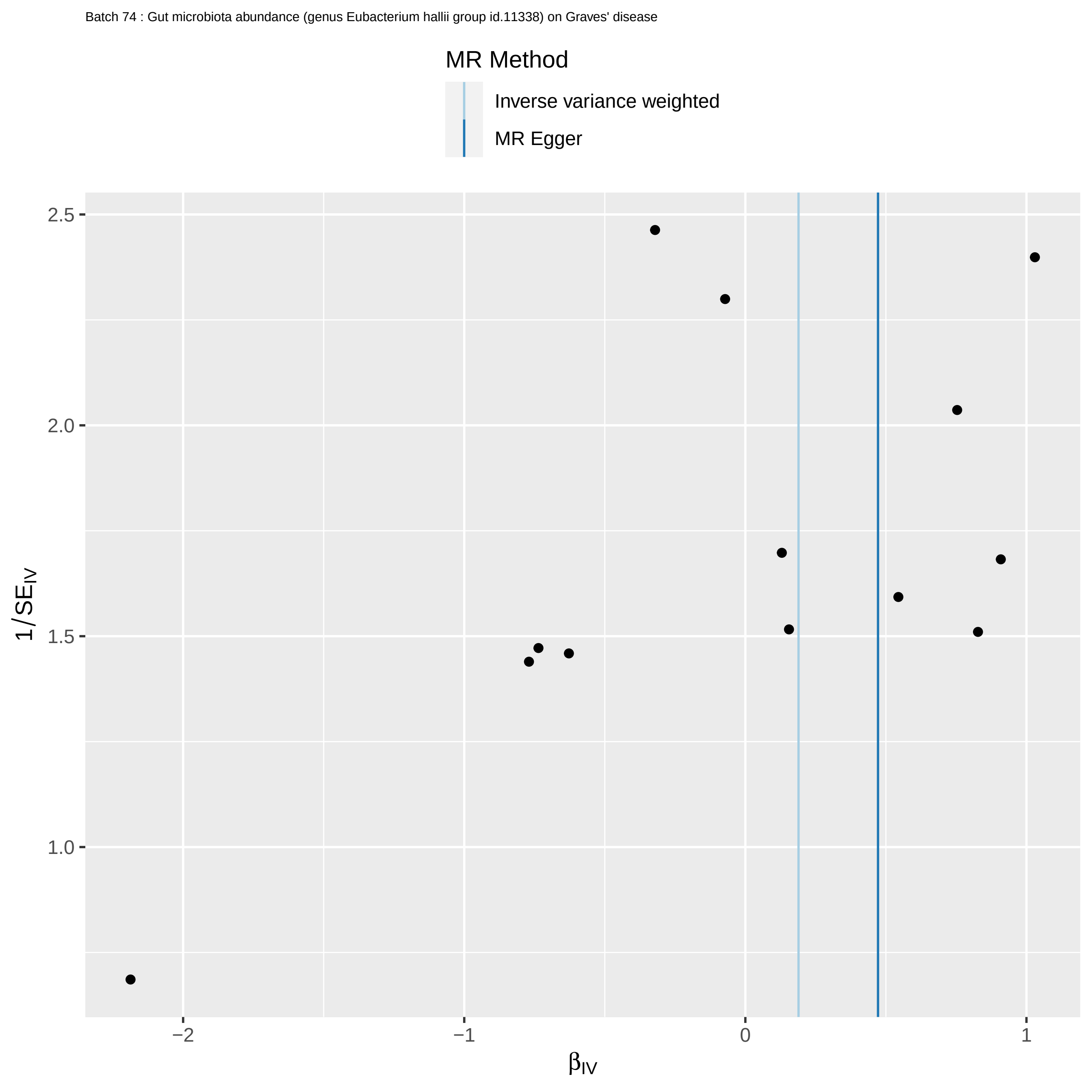

Batch 74 : Gut microbiota abundance (genus Eubacterium hallii group id.11338) on Graves' disease
MR Method
Inverse variance weighted
MR Egger
2.5
2.0
1.5
1.0
1/SE_IV
−2
−1
0
1
β_IV

Batch 75 : Gut microbiota abundance (genus Eubacterium nodatum group id.11297) on Graves' disease

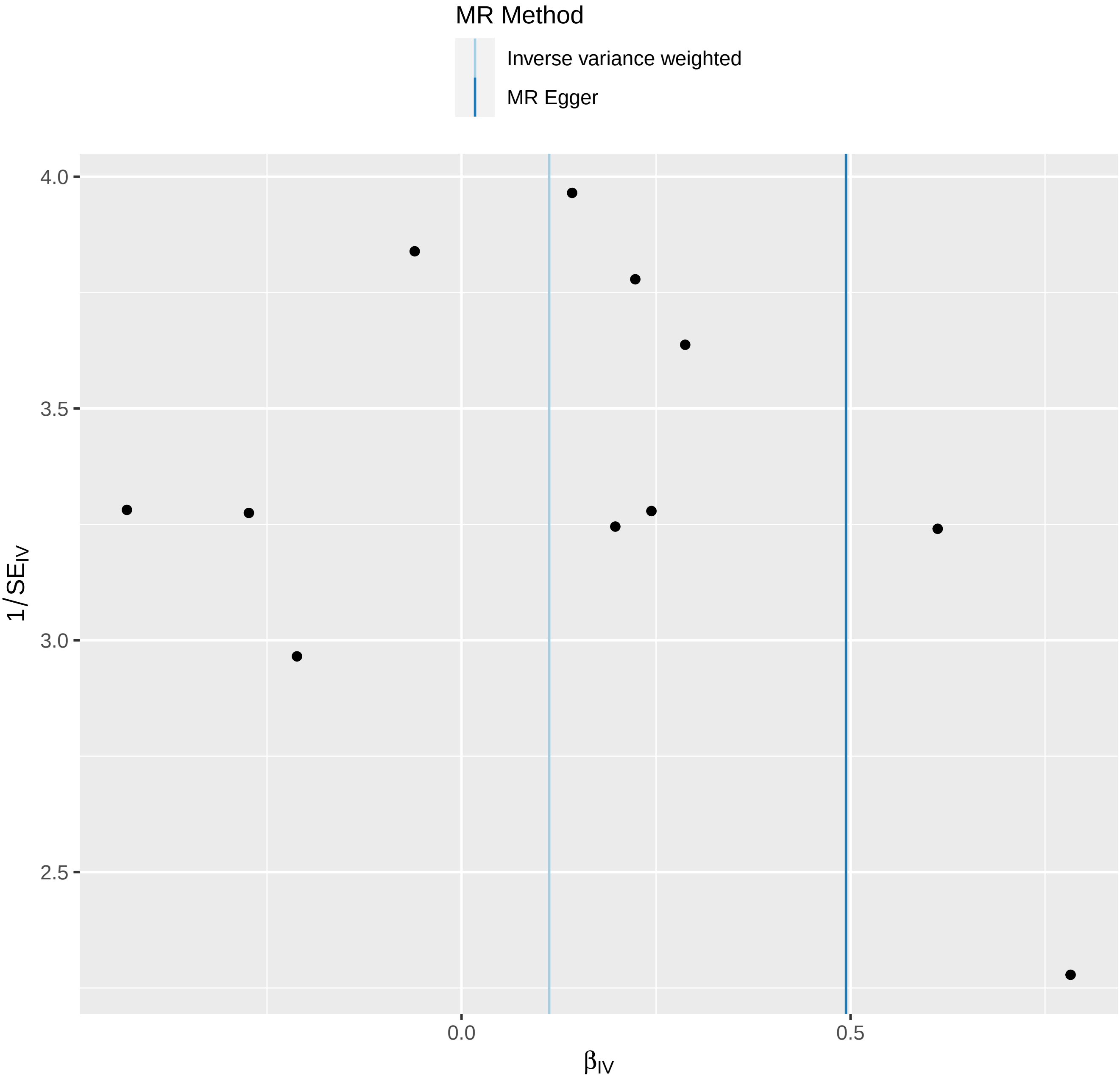

Batch 76 : Gut microbiota abundance (genus Eubacterium oxidoreducens group id.11339) on Graves' disease

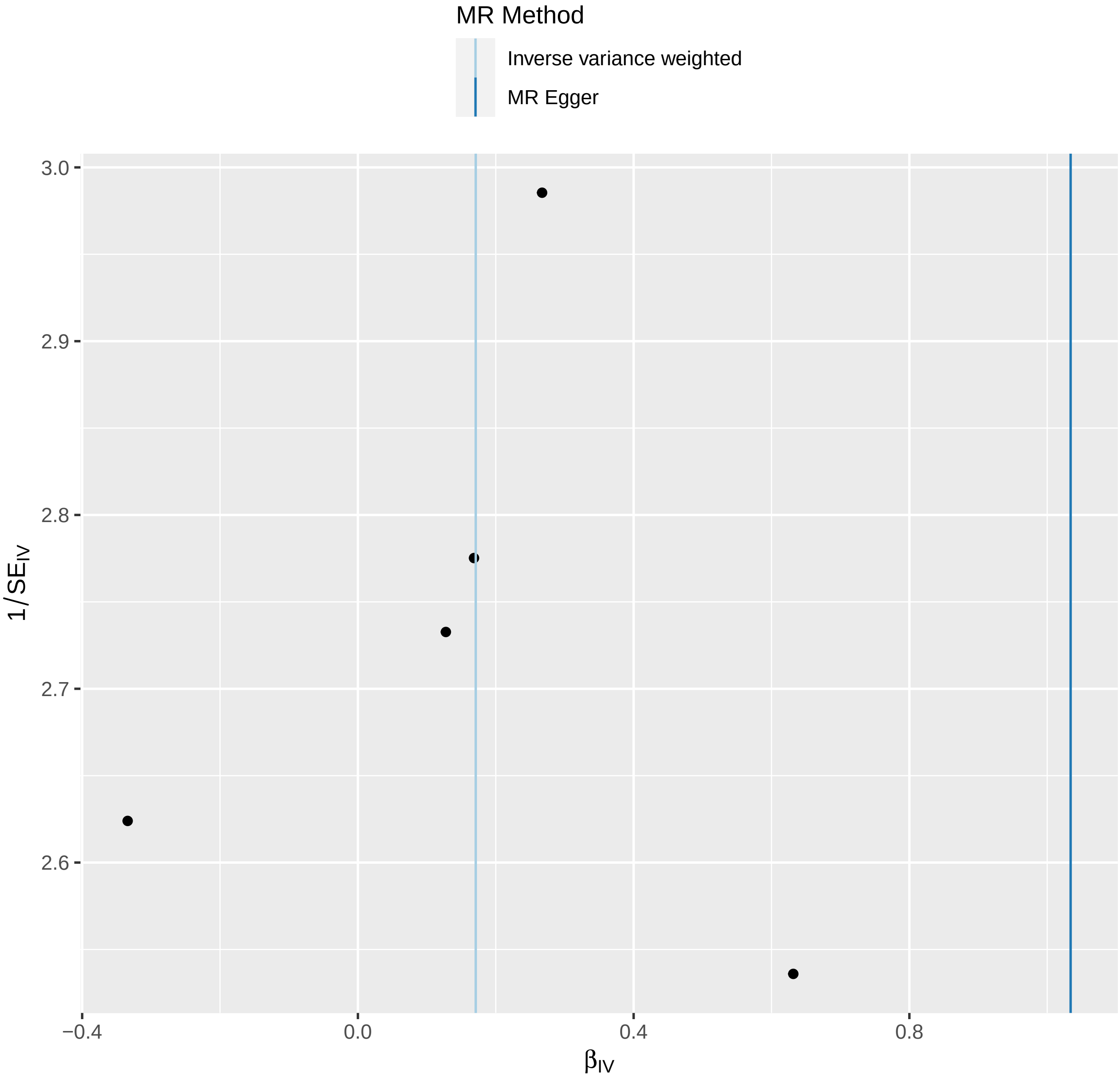

Batch 77 : Gut microbiota abundance (genus Eubacterium rectale group id.14374) on Graves' disease

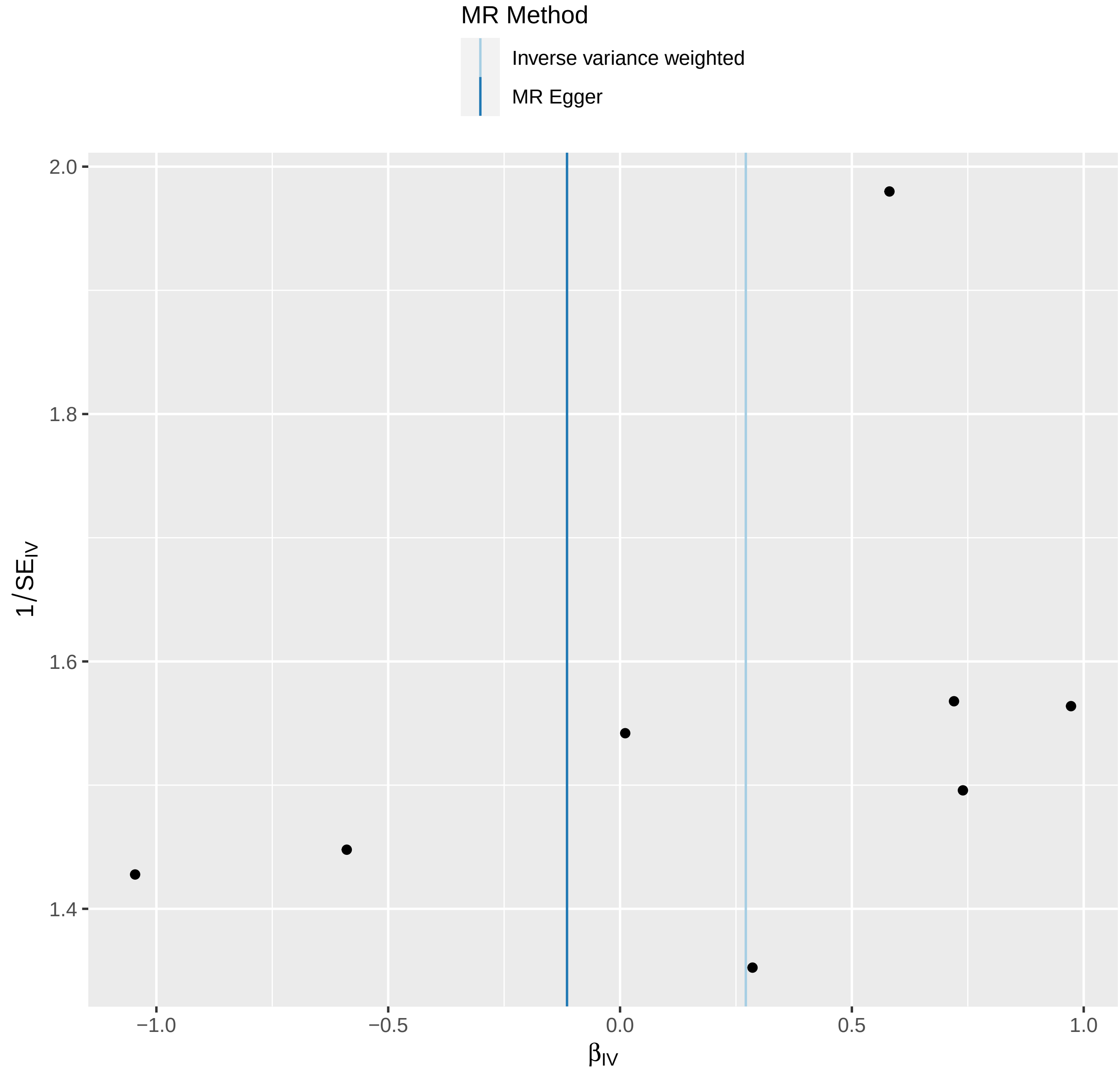

Batch 78 : Gut microbiota abundance (genus Eubacterium ruminantium group id.11340) on Graves' disease

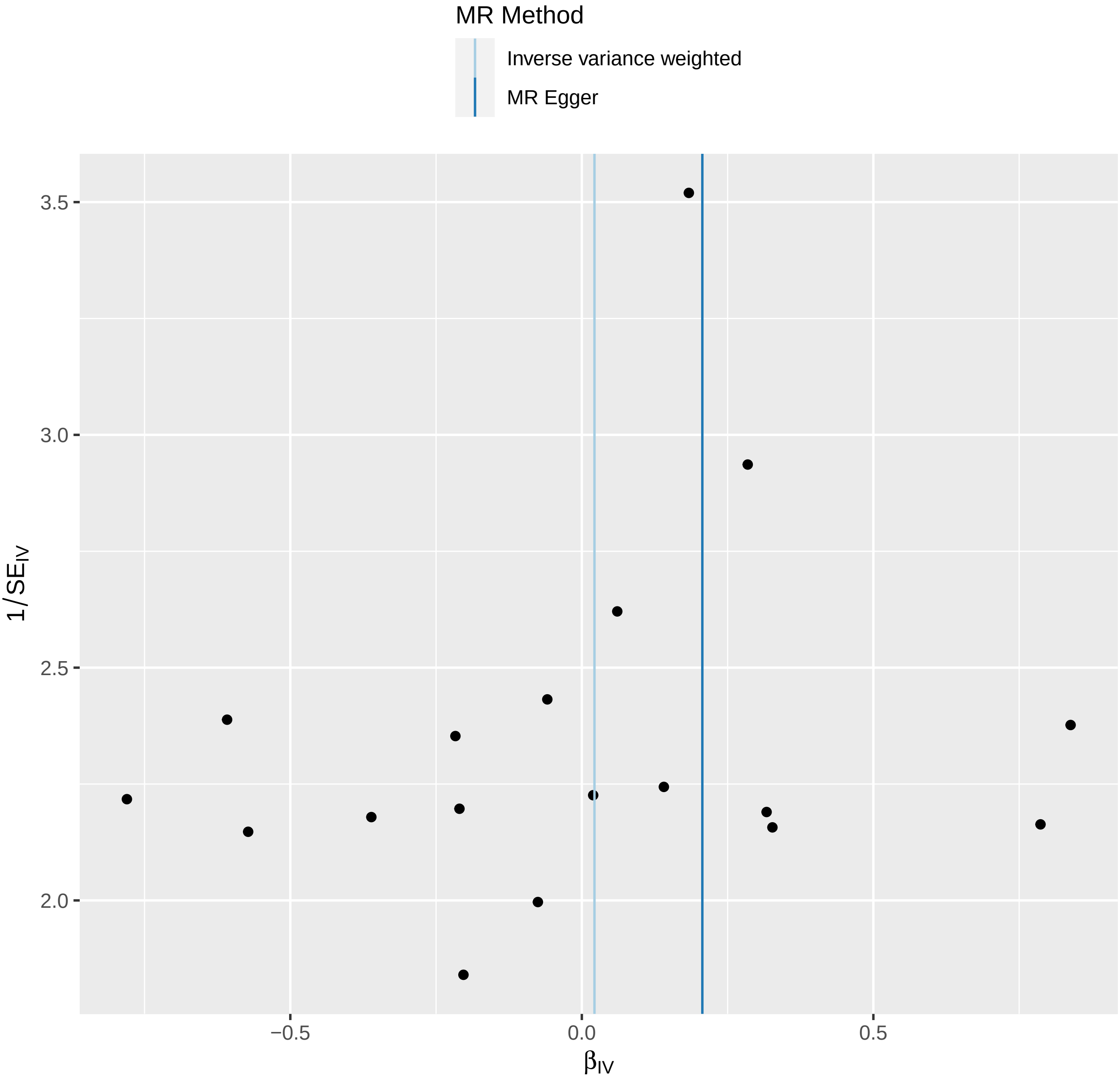

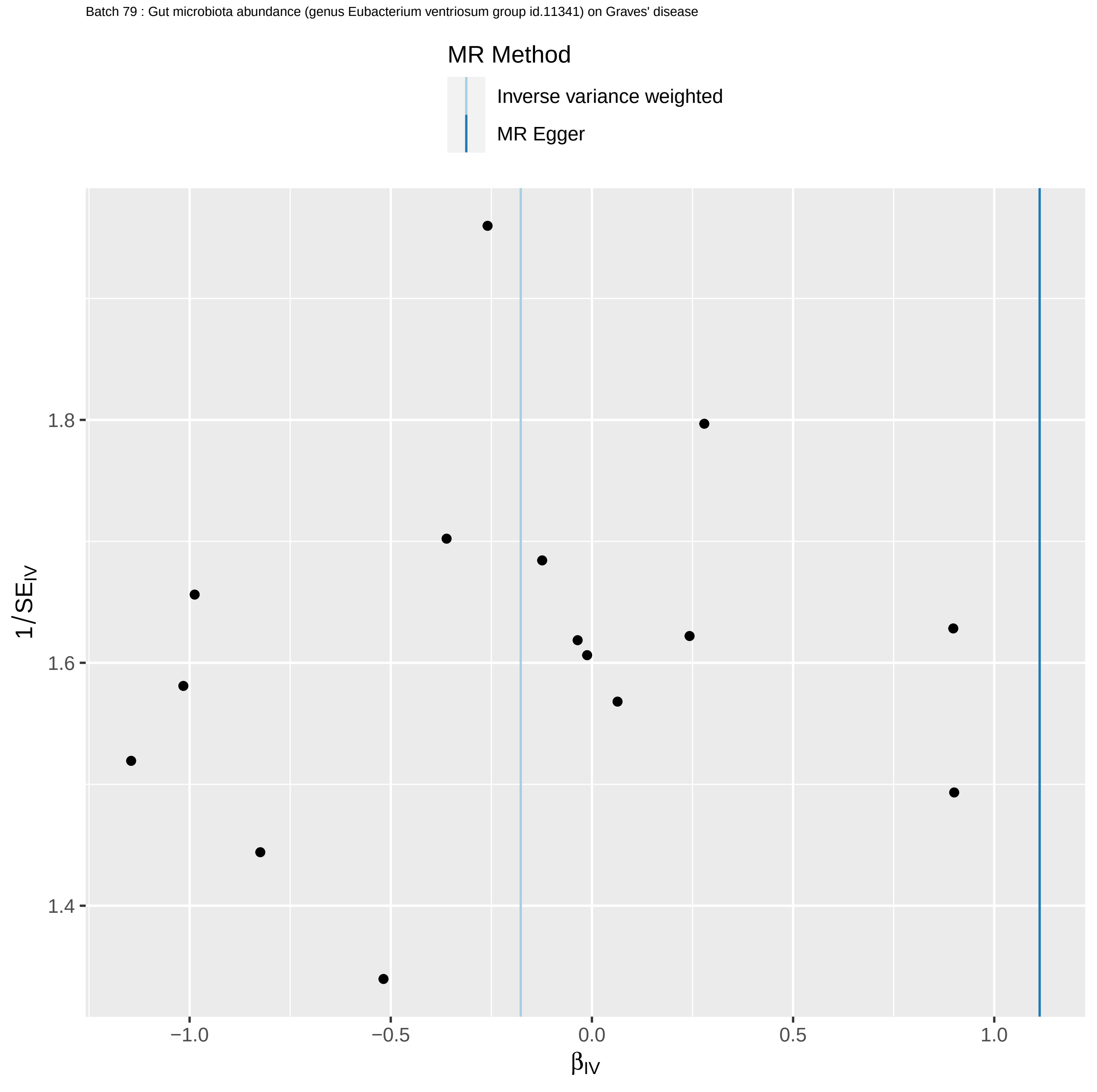

Batch 79 : Gut microbiota abundance (genus Eubacterium ventriosum group id.11341) on Graves' disease
MR Method
Inverse variance weighted
MR Egger
1.8
1.6
1.4
1/SE_IV
−1.0
−0.5
0.0
0.5
1.0
β_IV

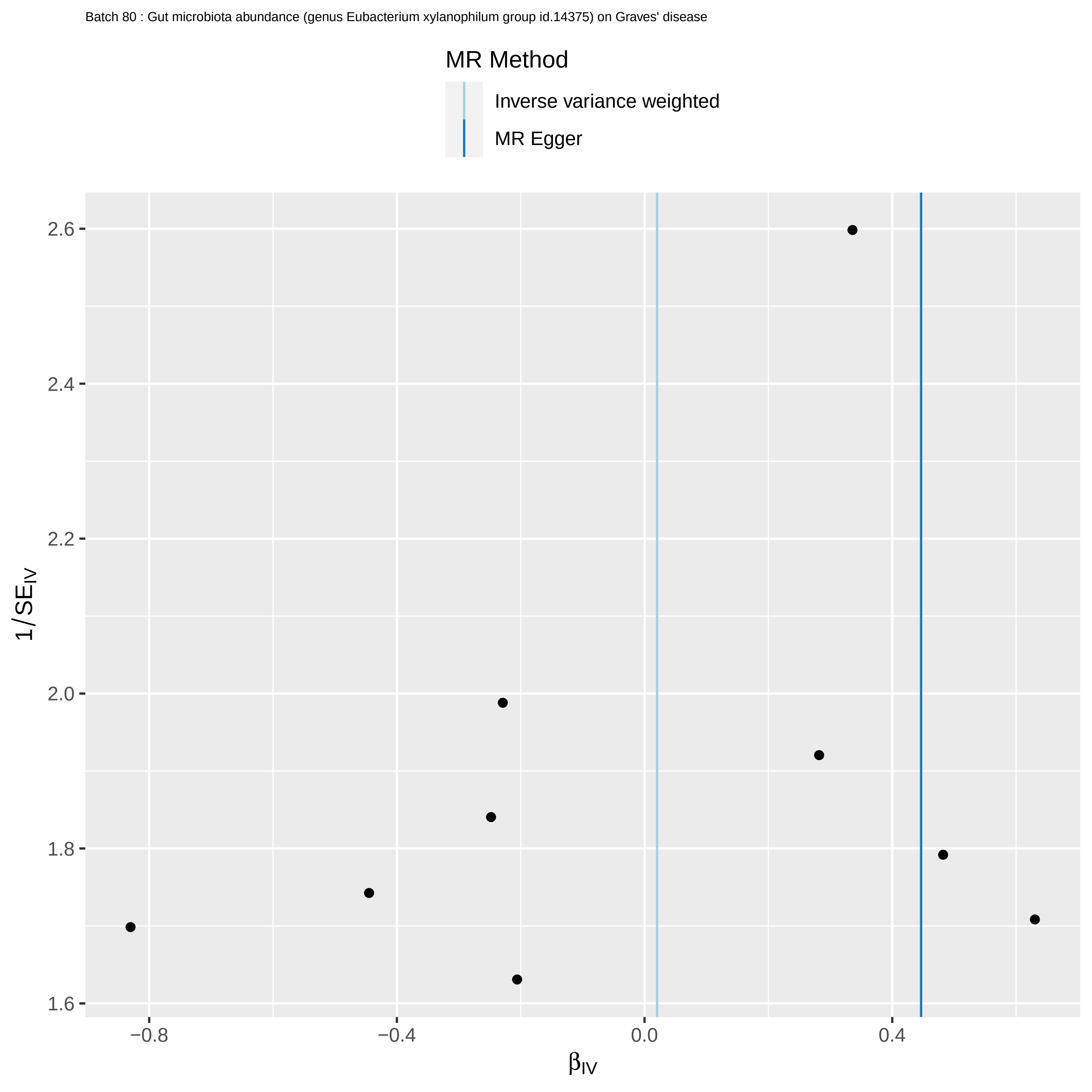

Batch 80 : Gut microbiota abundance (genus Eubacterium xylanophilum group id.14375) on Graves' disease
MR Method
Inverse variance weighted
MR Egger
2.6
2.4
2.2
2.0
1.8
1.6
1/SE_IV
−0.8
−0.4
0.0
0.4
β_IV

Batch 81 : Gut microbiota abundance (genus Faecalibacterium id.2057) on Graves' disease

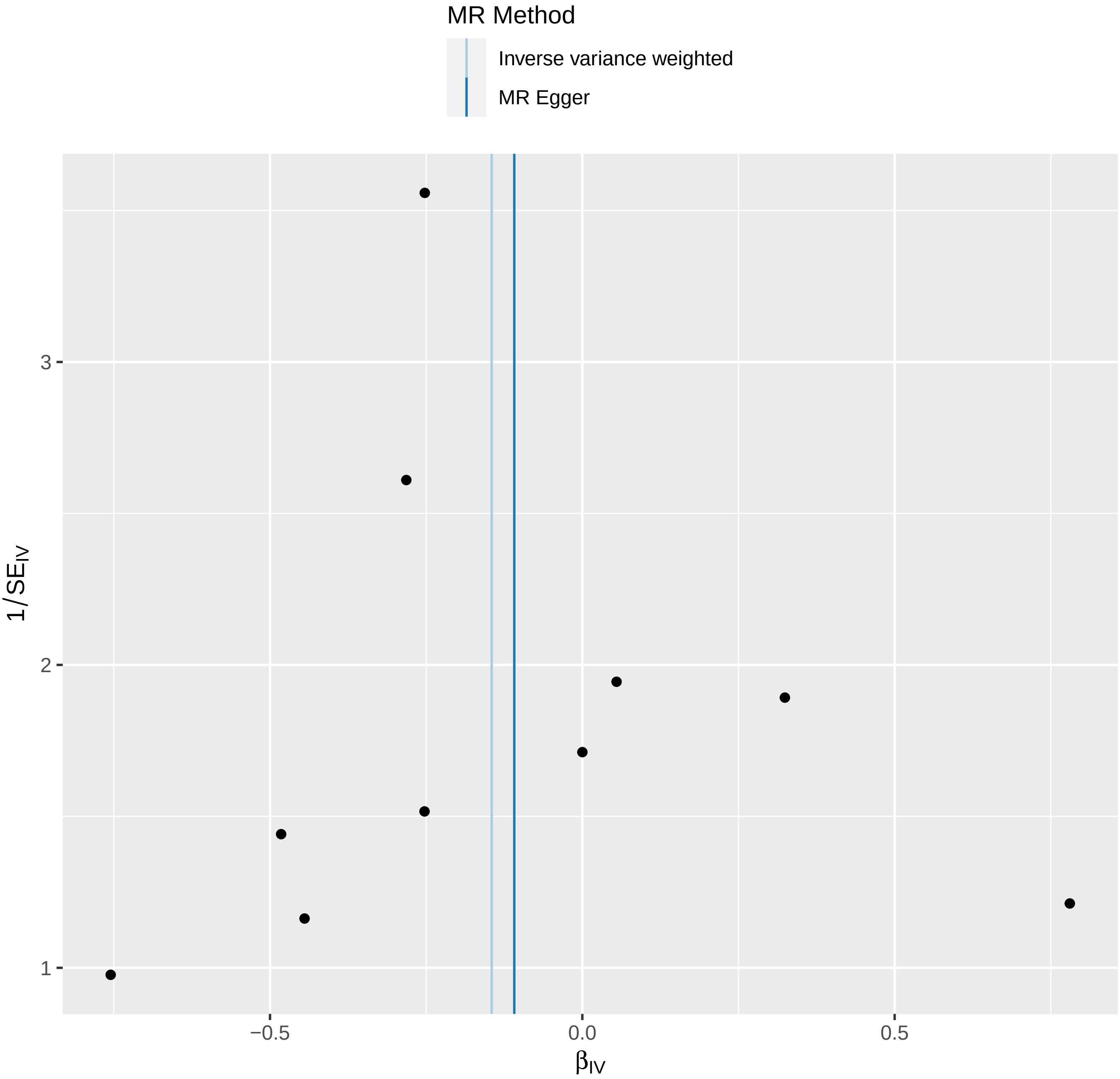

Batch 82 : Gut microbiota abundance (genus Family XIII AD3011 group id.11293) on Graves' disease

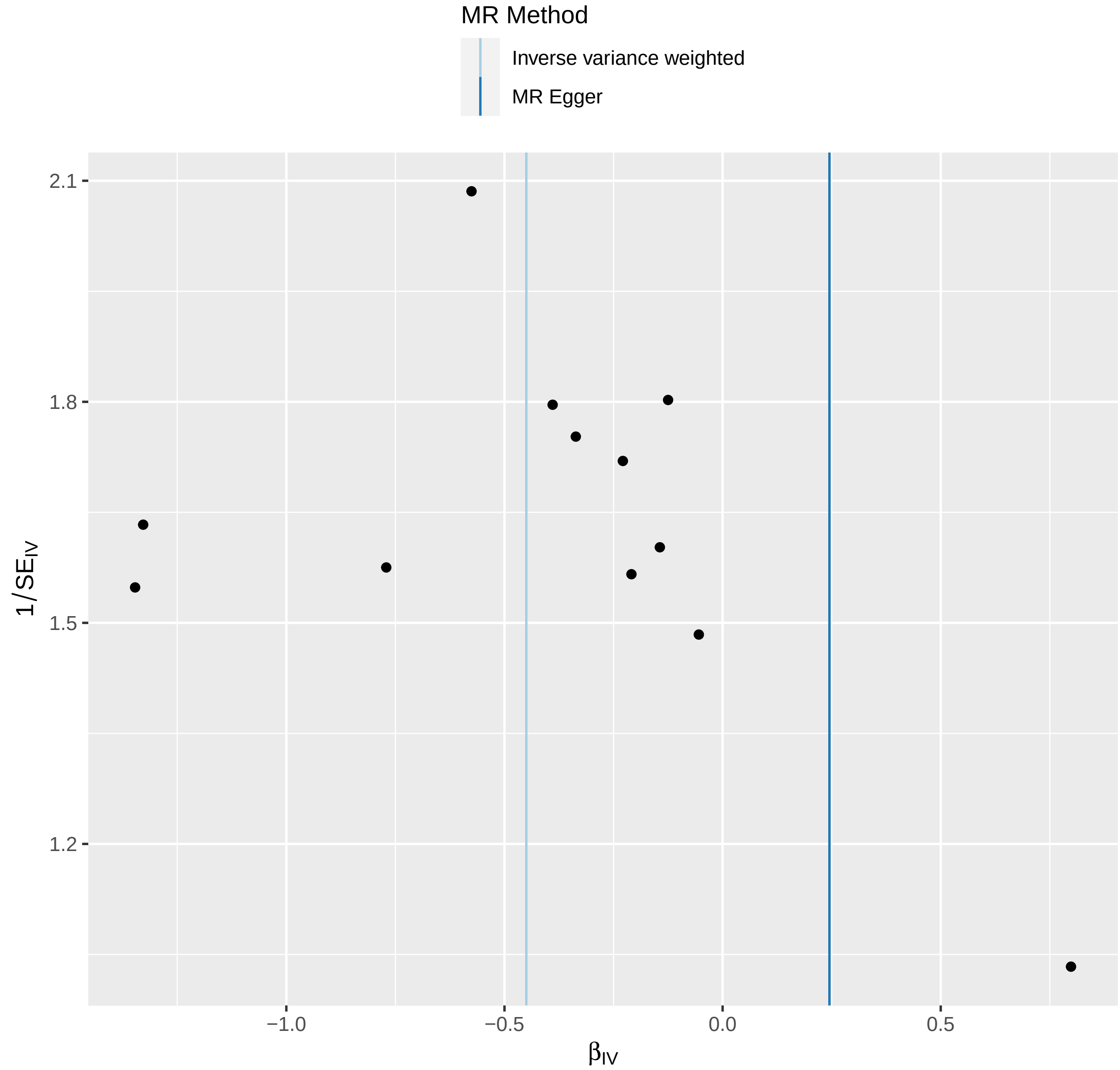

Batch 83 : Gut microbiota abundance (genus Family XIII UCG001 id.11294) on Graves' disease

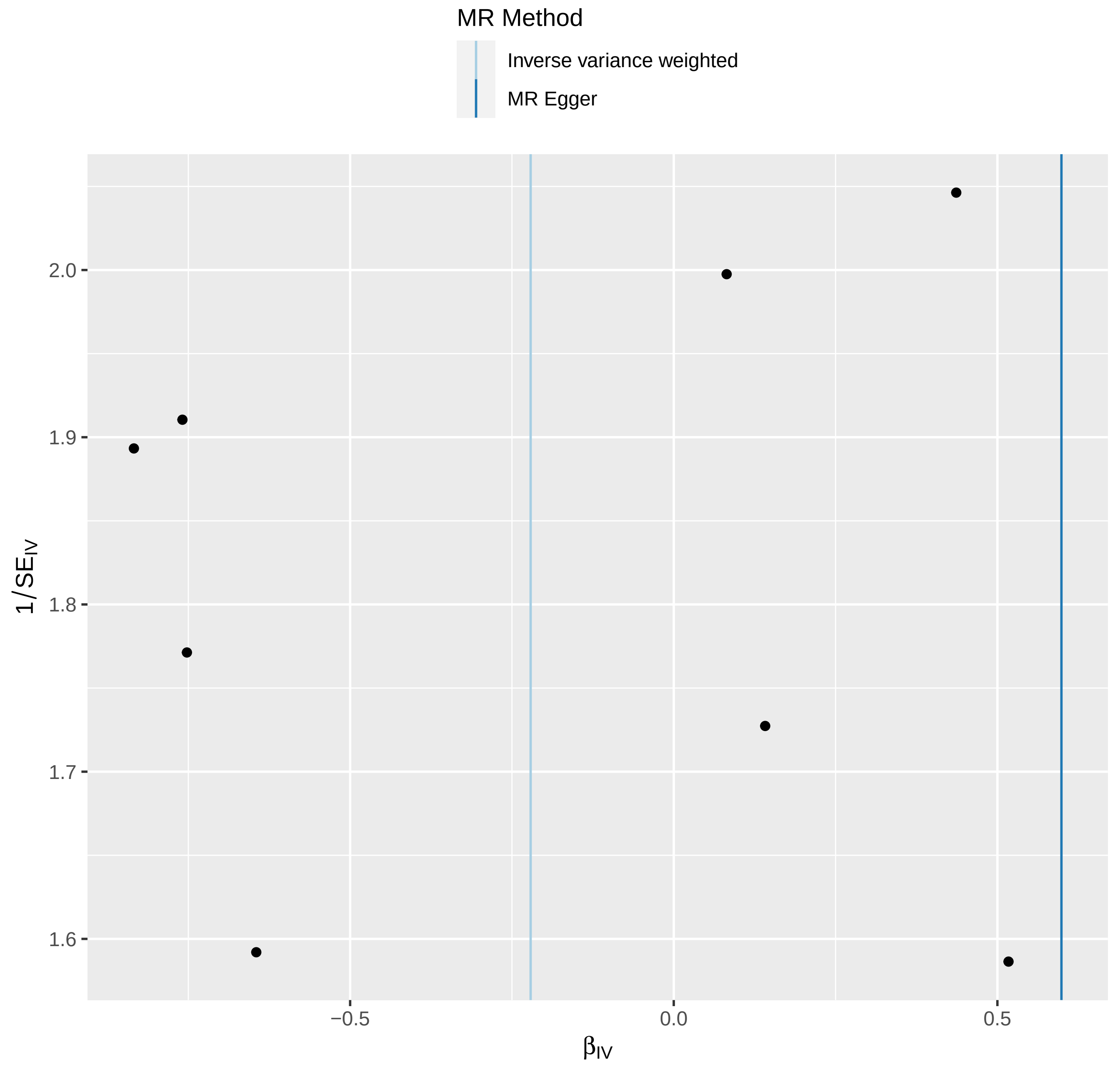

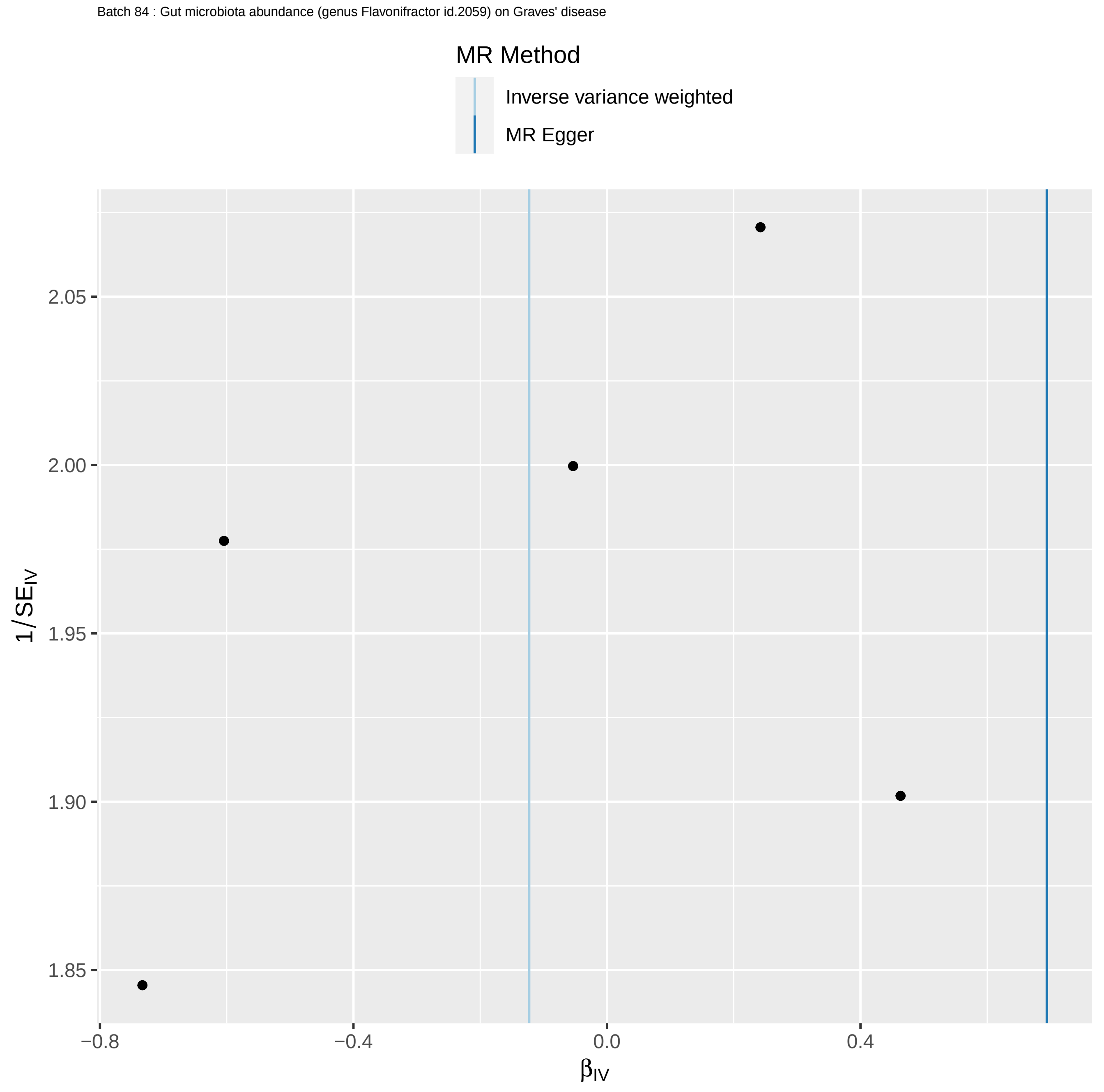
Batch 84 : Gut microbiota abundance (genus Flavonifractor id.2059) on Graves' disease
MR Method
Inverse variance weighted
MR Egger
2.05
2.00
1.95
1.90
1.85
1/SE_IV
−0.8
−0.4
0.0
0.4
β_IV

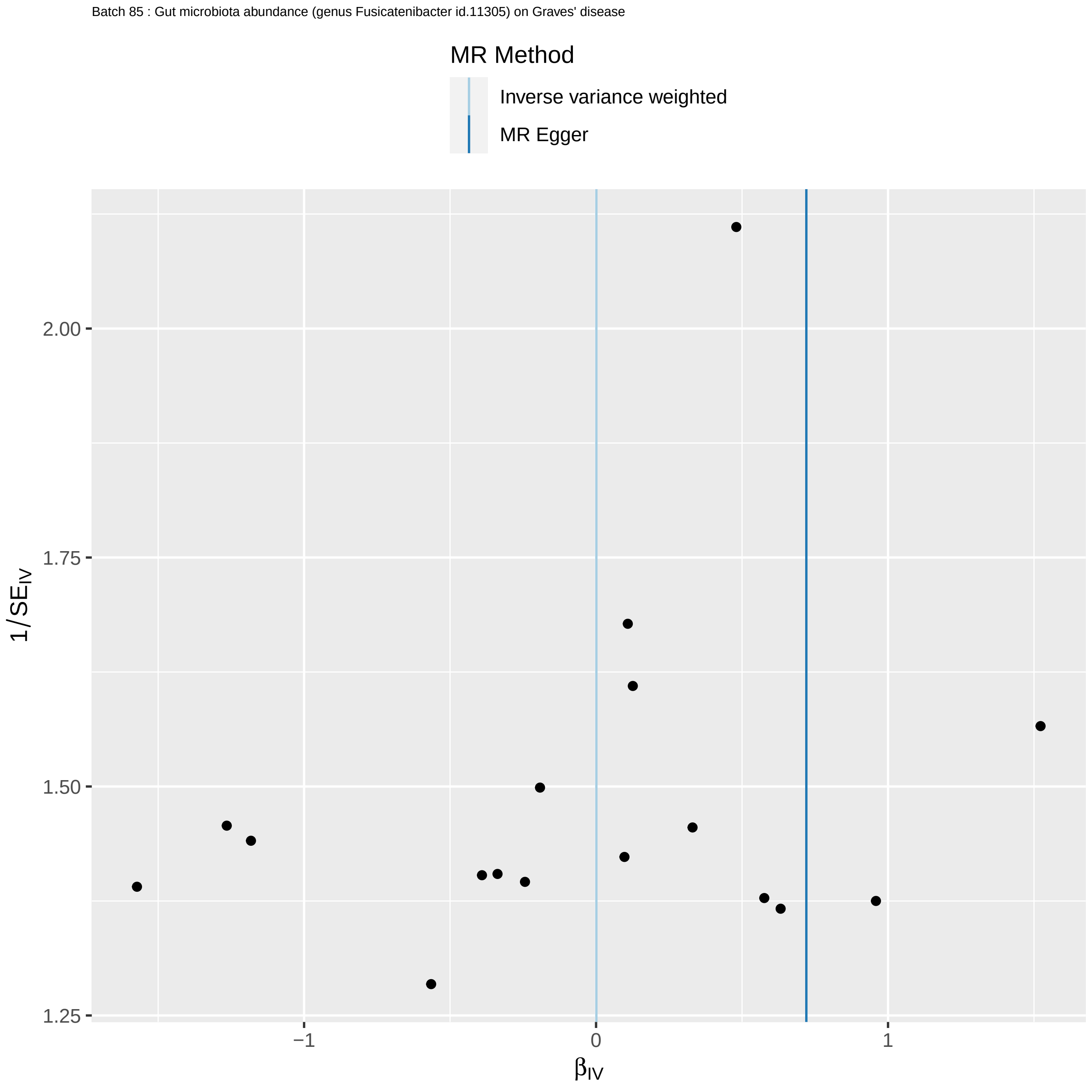
Batch 85 : Gut microbiota abundance (genus Fusicatenibacter id.11305) on Graves' disease
MR Method
Inverse variance weighted
MR Egger
2.00
1.75
1.50
1.25
1/SE_IV
−1
0
1
β_IV

Batch 86 : Gut microbiota abundance (genus Gordonibacter id.821) on Graves' disease

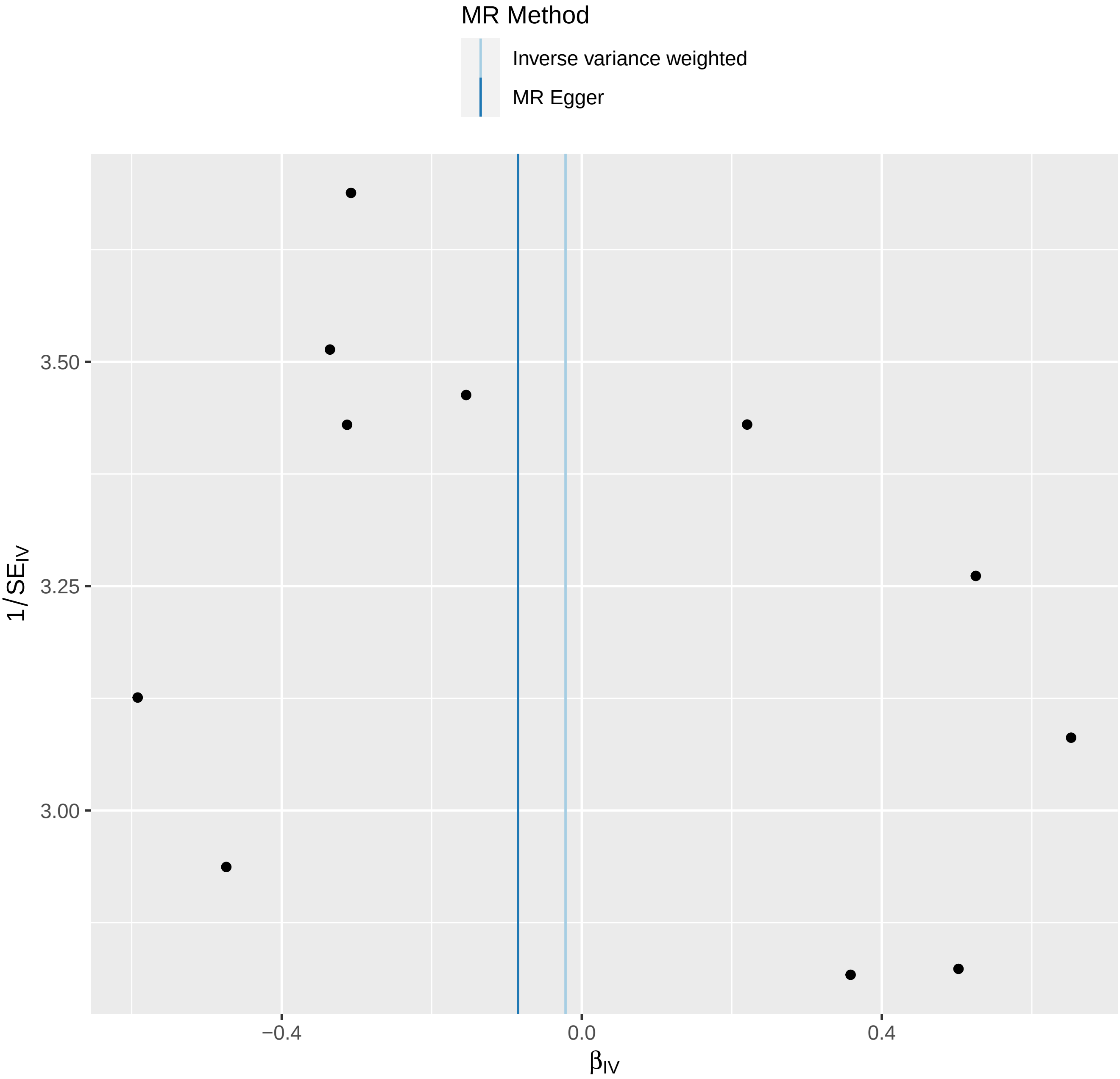

Batch 87 : Gut microbiota abundance (genus Haemophilus id.3698) on Graves' disease

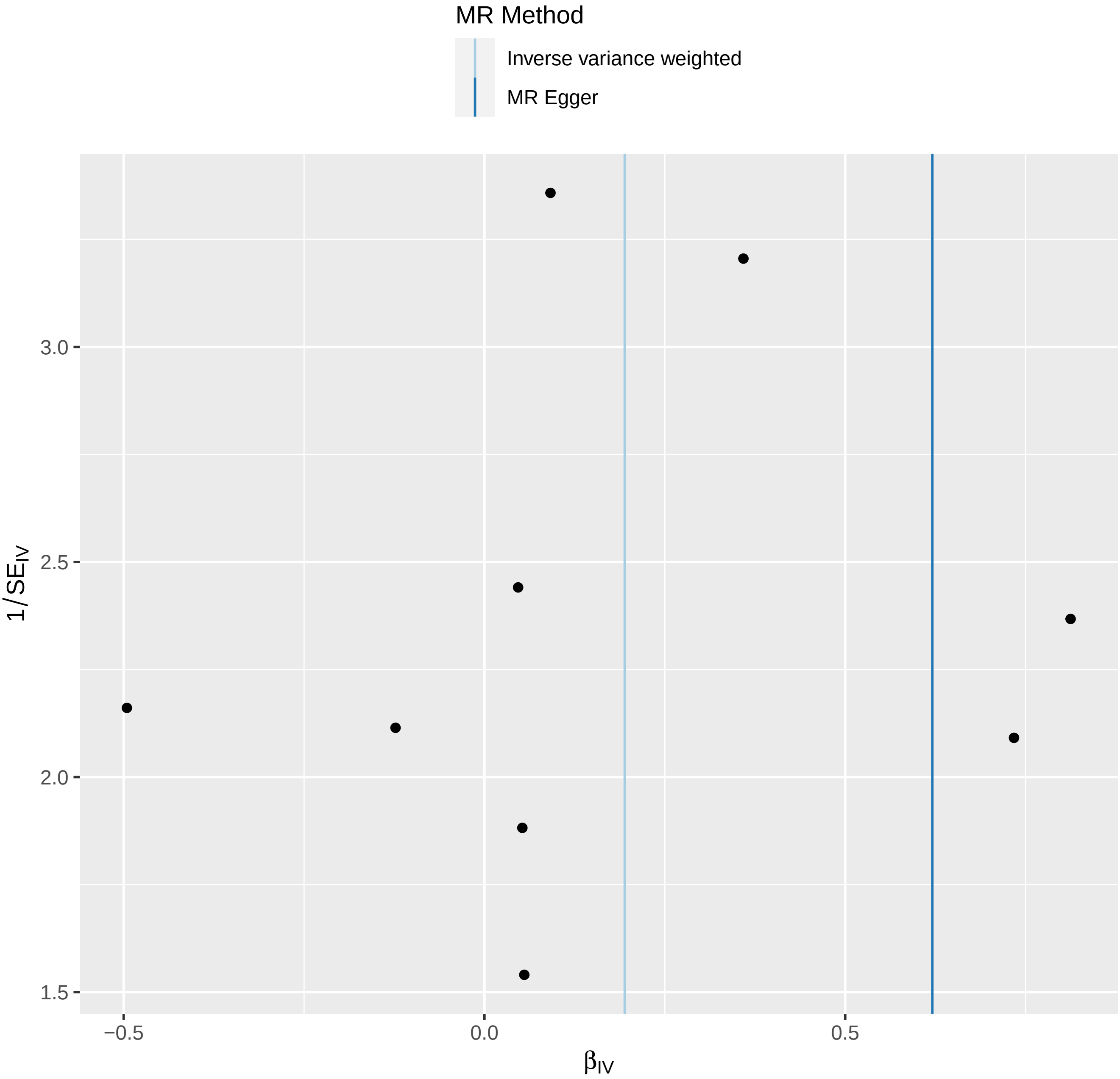

Batch 88 : Gut microbiota abundance (genus Holdemanella id.11393) on Graves' disease

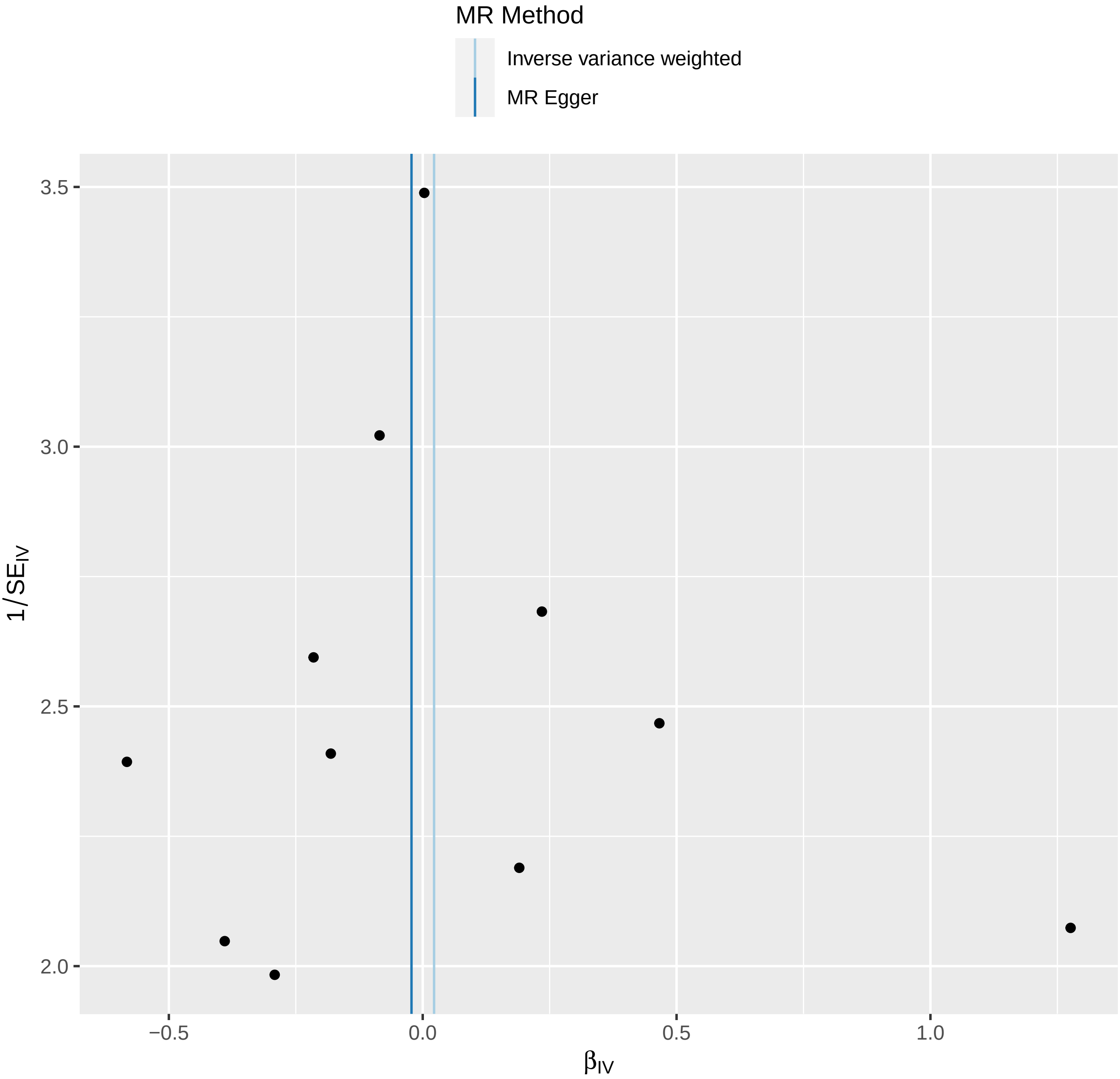

Batch 89 : Gut microbiota abundance (genus Holdemania id.2157) on Graves' disease

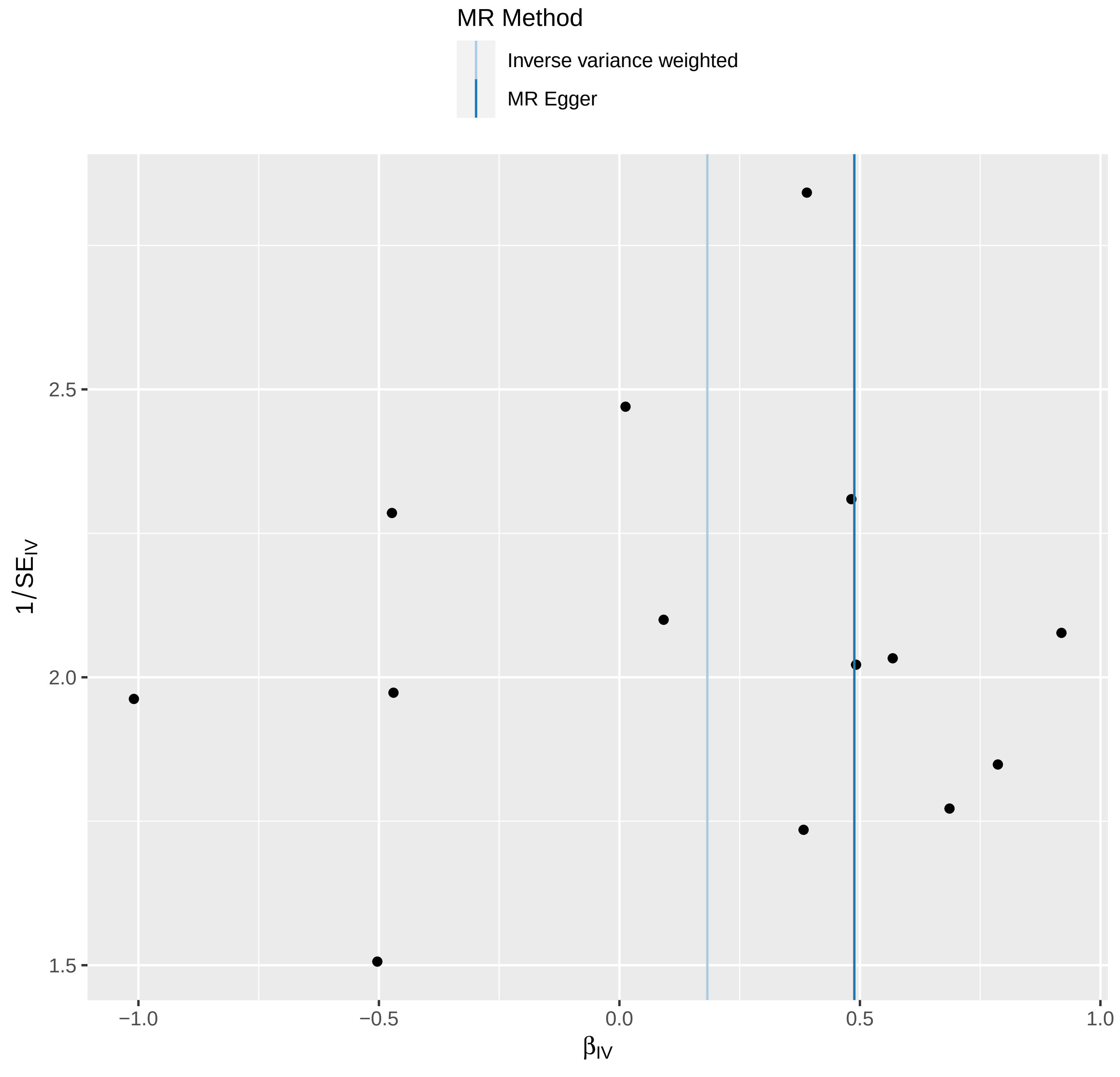

Batch 90 : Gut microbiota abundance (genus Howardella id.2000) on Graves' disease

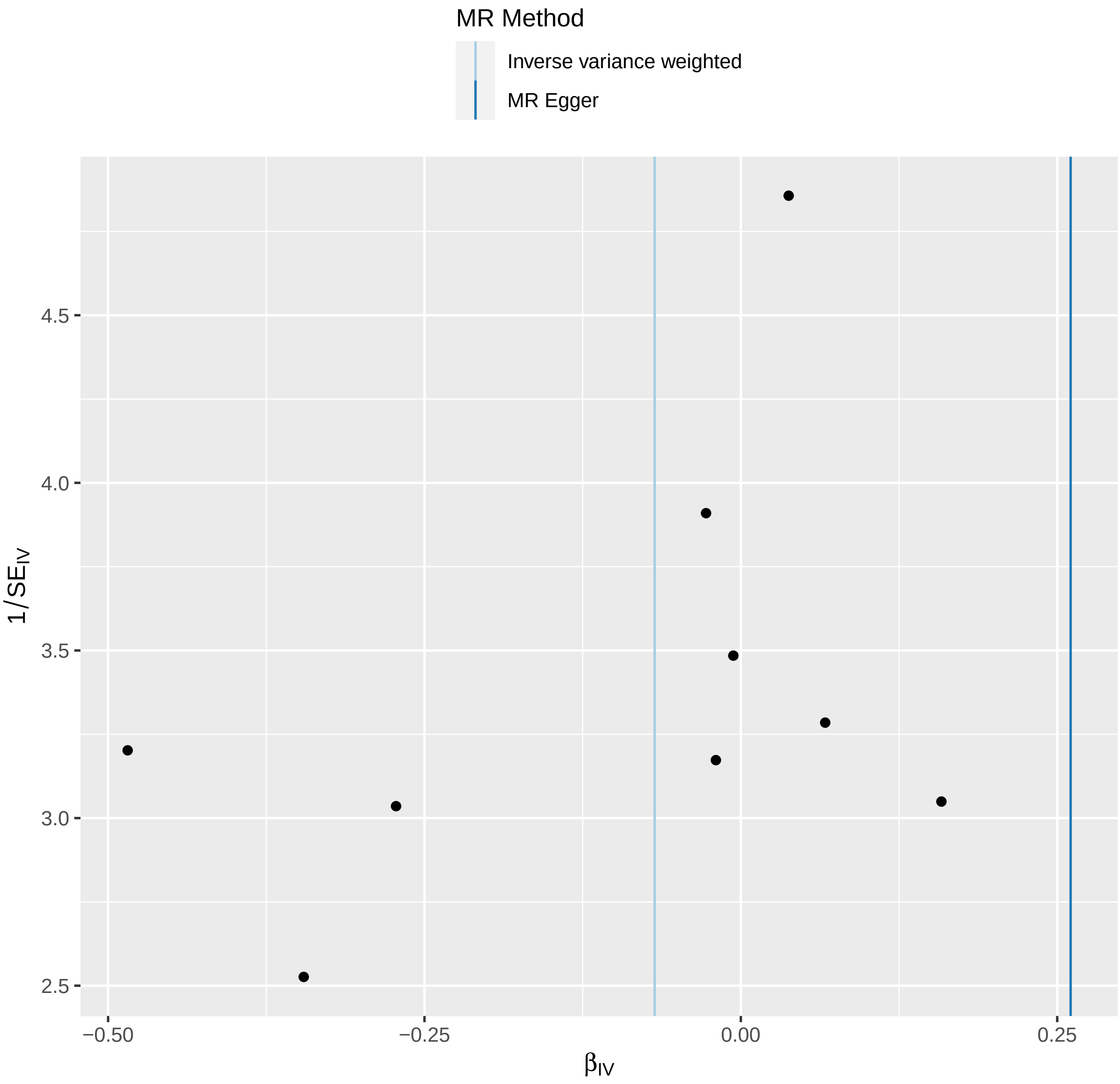

Batch 91 : Gut microbiota abundance (genus Hungatella id.11306) on Graves' disease

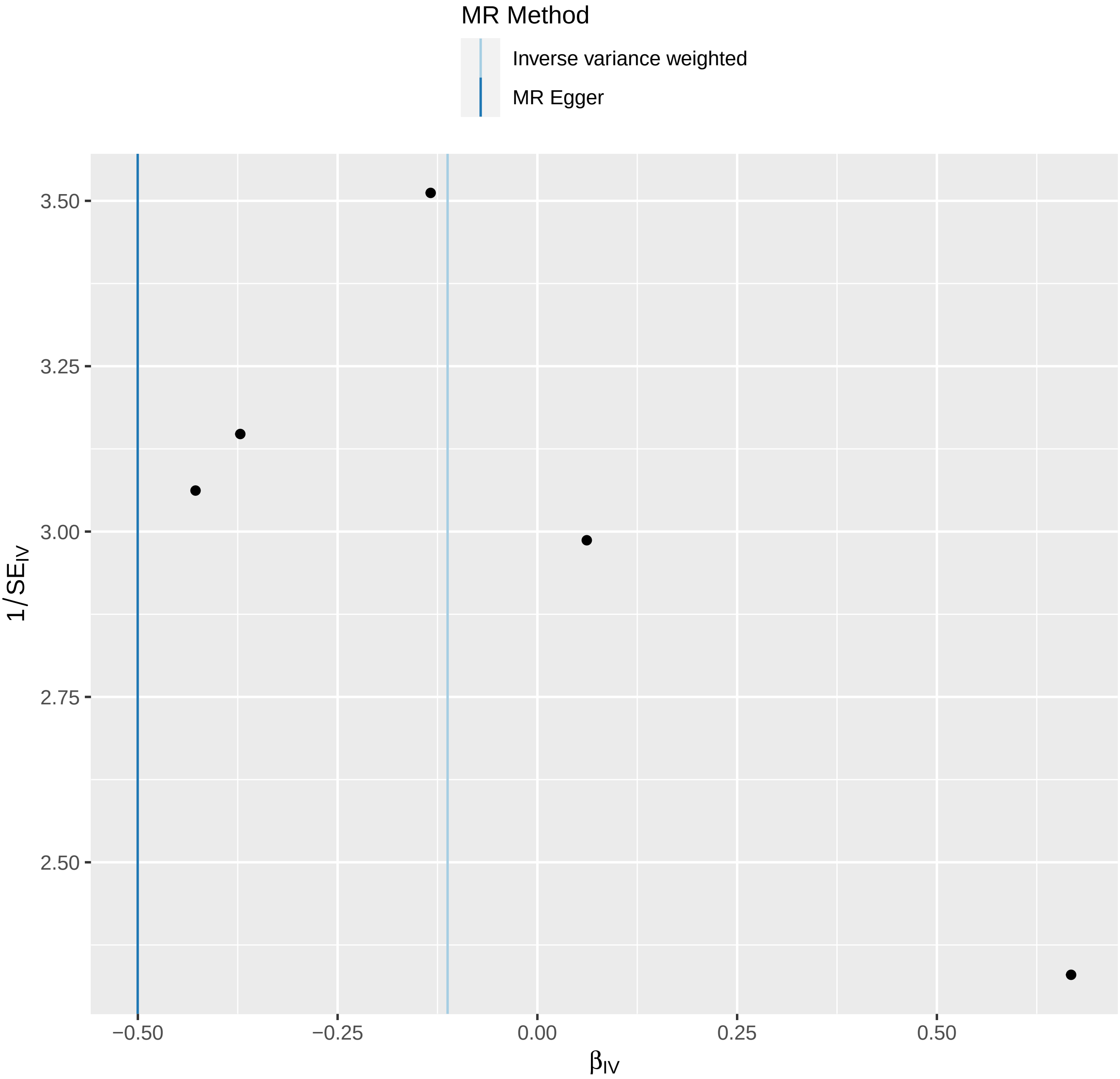

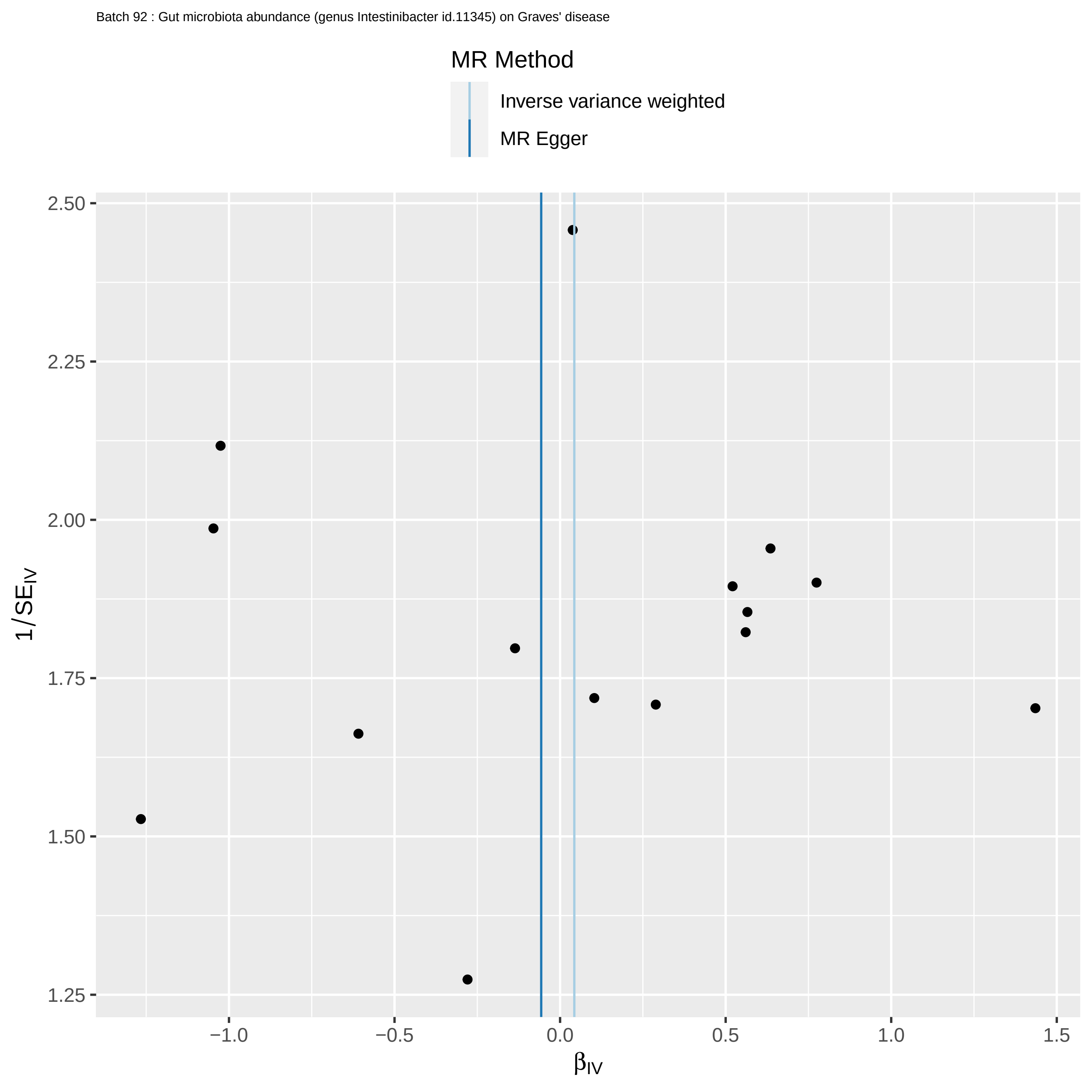

Batch 92 : Gut microbiota abundance (genus Intestinibacter id.11345) on Graves' disease
MR Method
Inverse variance weighted
MR Egger
2.50
2.25
2.00
1.75
1.50
1.25
1/SE_IV
−1.0
−0.5
0.0
0.5
1.0
1.5
β_IV

Batch 93 : Gut microbiota abundance (genus Intestinimonas id.2062) on Graves' disease

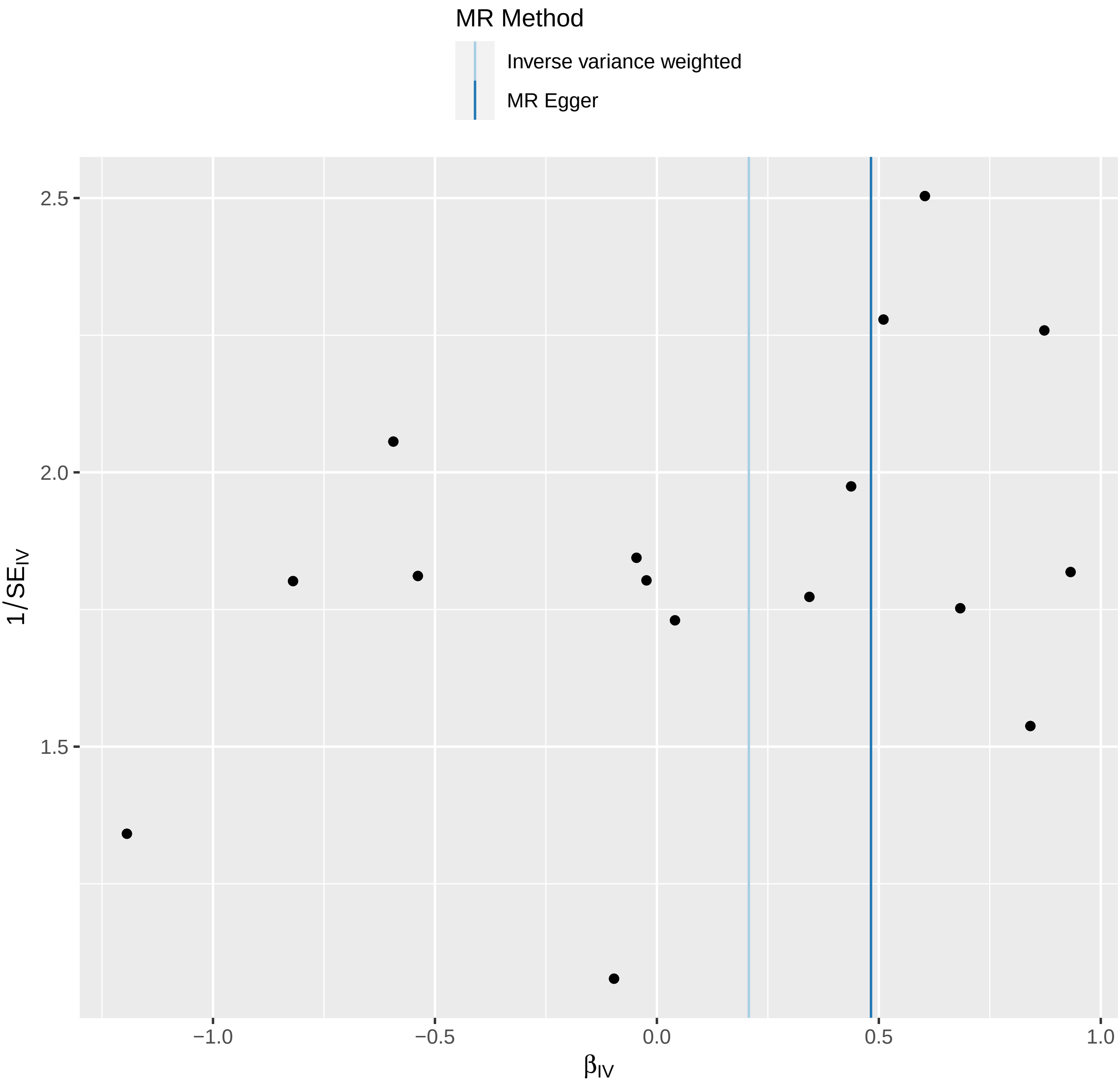

Batch 94 : Gut microbiota abundance (genus Lachnoclostridium id.11308) on Graves' disease

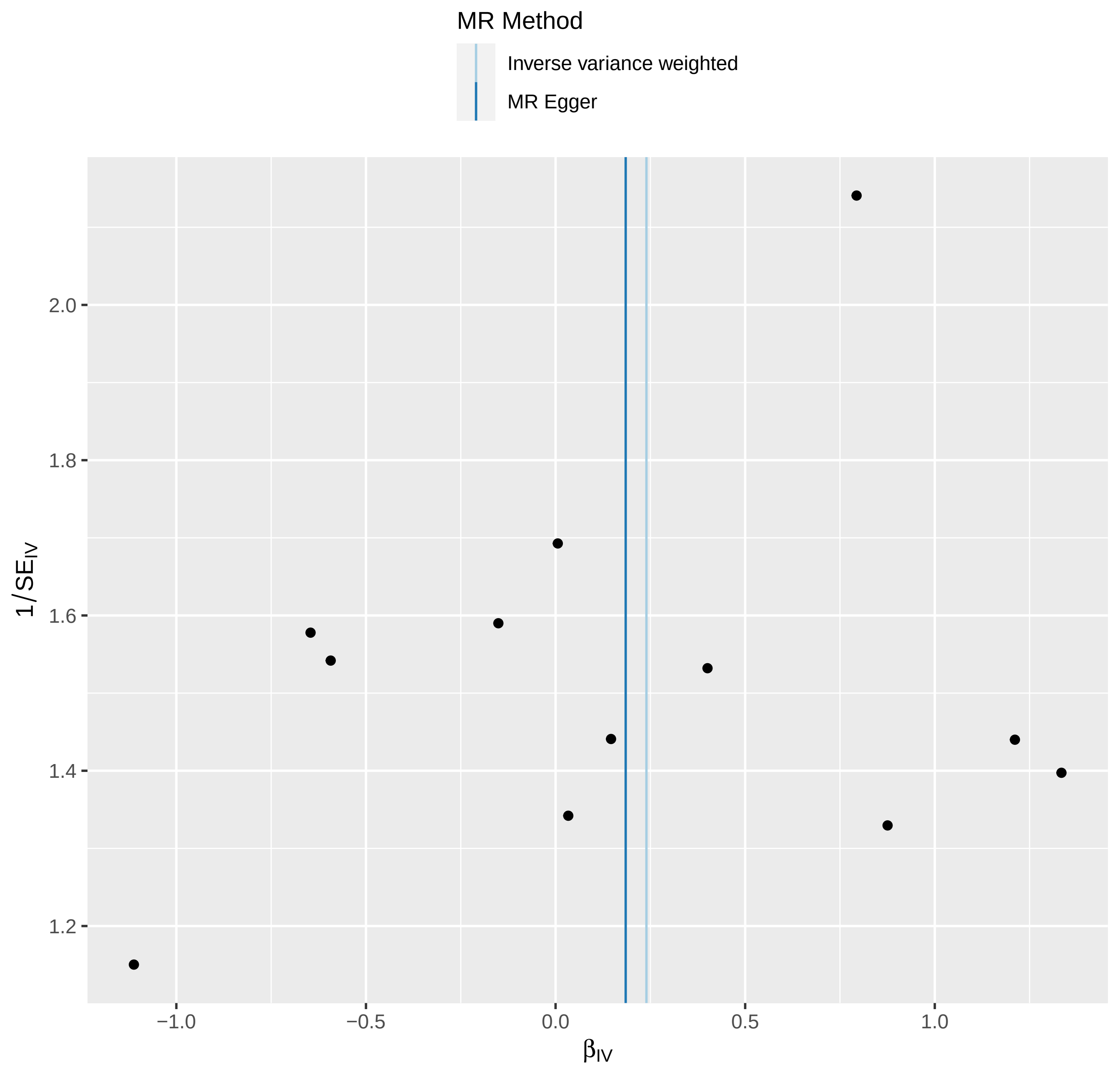

Batch 95 : Gut microbiota abundance (genus Lachnospiraceae FCS020 group id.11314) on Graves' disease

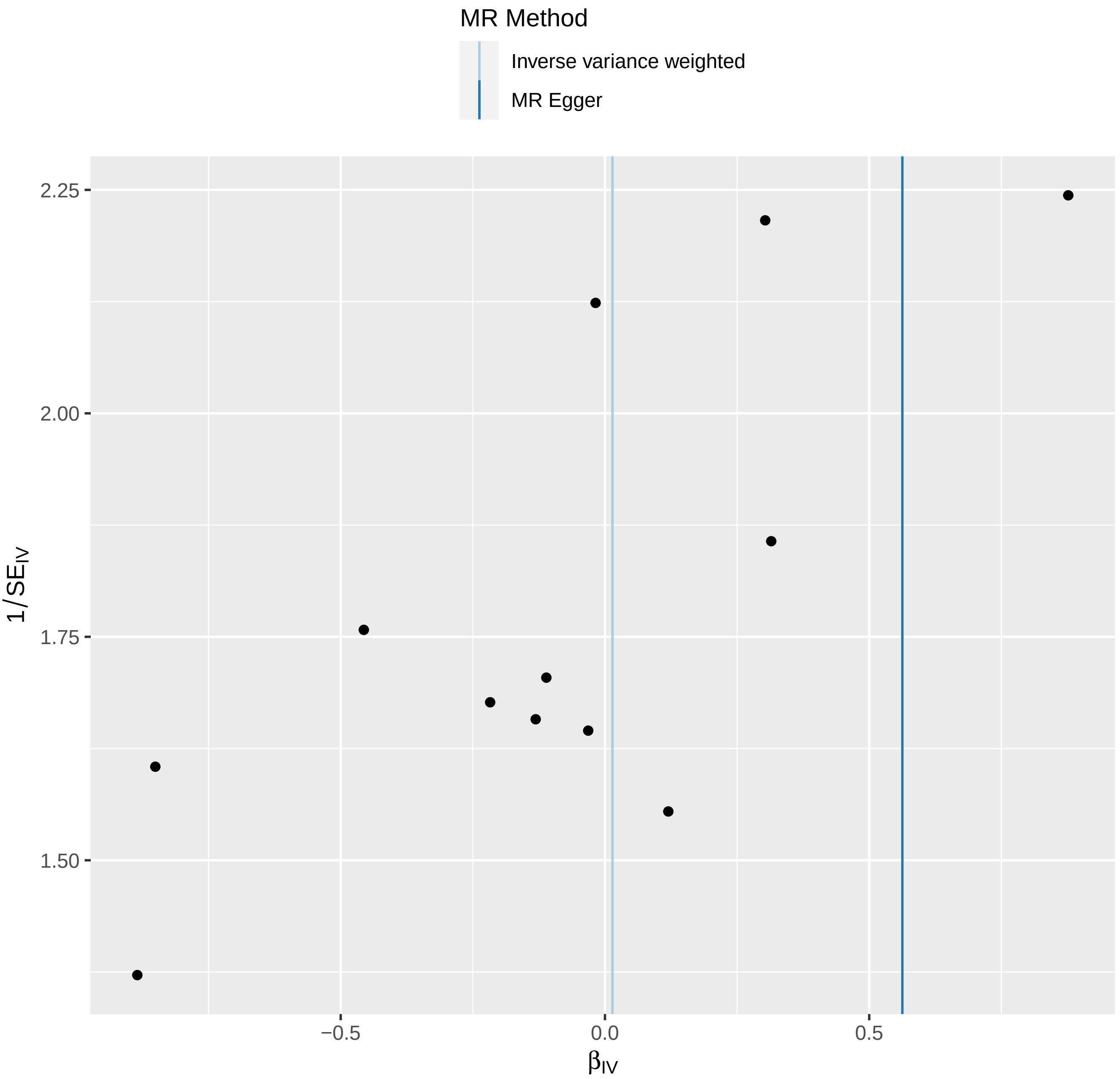

Batch 96 : Gut microbiota abundance (genus Lachnospiraceae NC2004 group id.11316) on Graves' disease

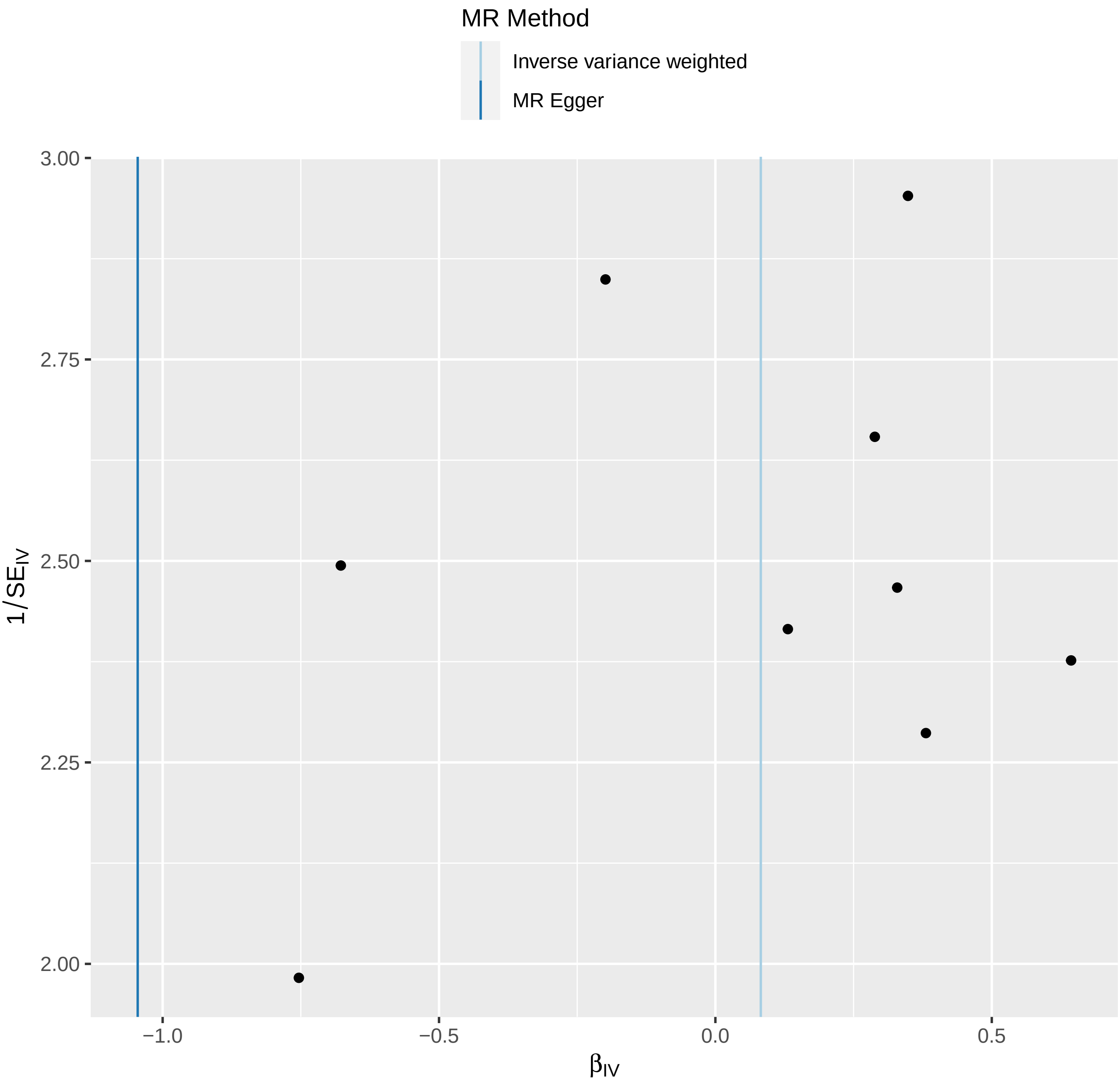

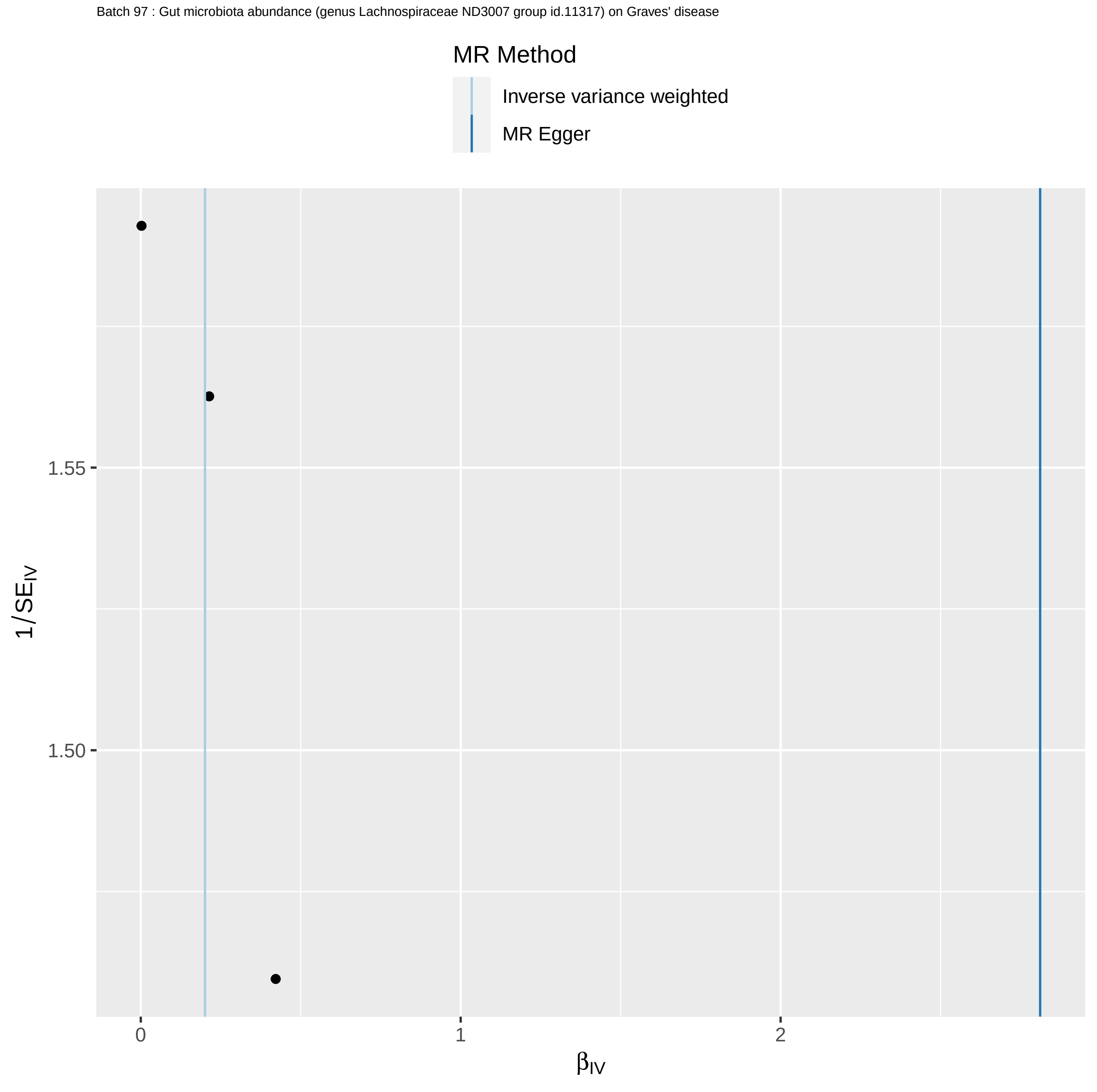
Batch 97 : Gut microbiota abundance (genus Lachnospiraceae ND3007 group id.11317) on Graves' disease
MR Method
Inverse variance weighted
MR Egger
1.55
1.50
1/SE_IV
0
1
2
β_IV

Batch 98 : Gut microbiota abundance (genus Lachnospiraceae NK4A136 group id.11319) on Graves' disease

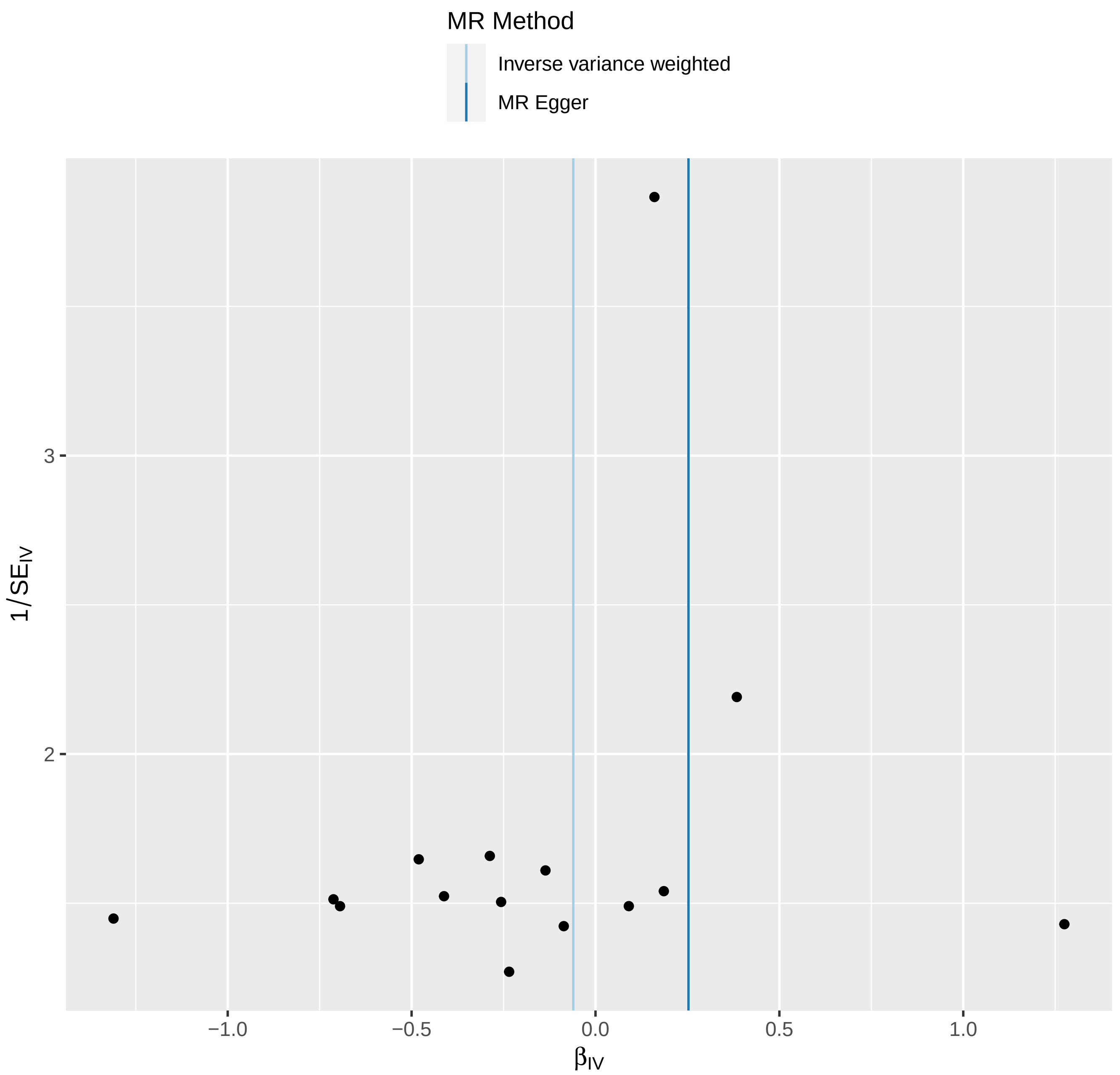

Batch 99 : Gut microbiota abundance (genus Lachnospiraceae UCG001 id.11321) on Graves' disease

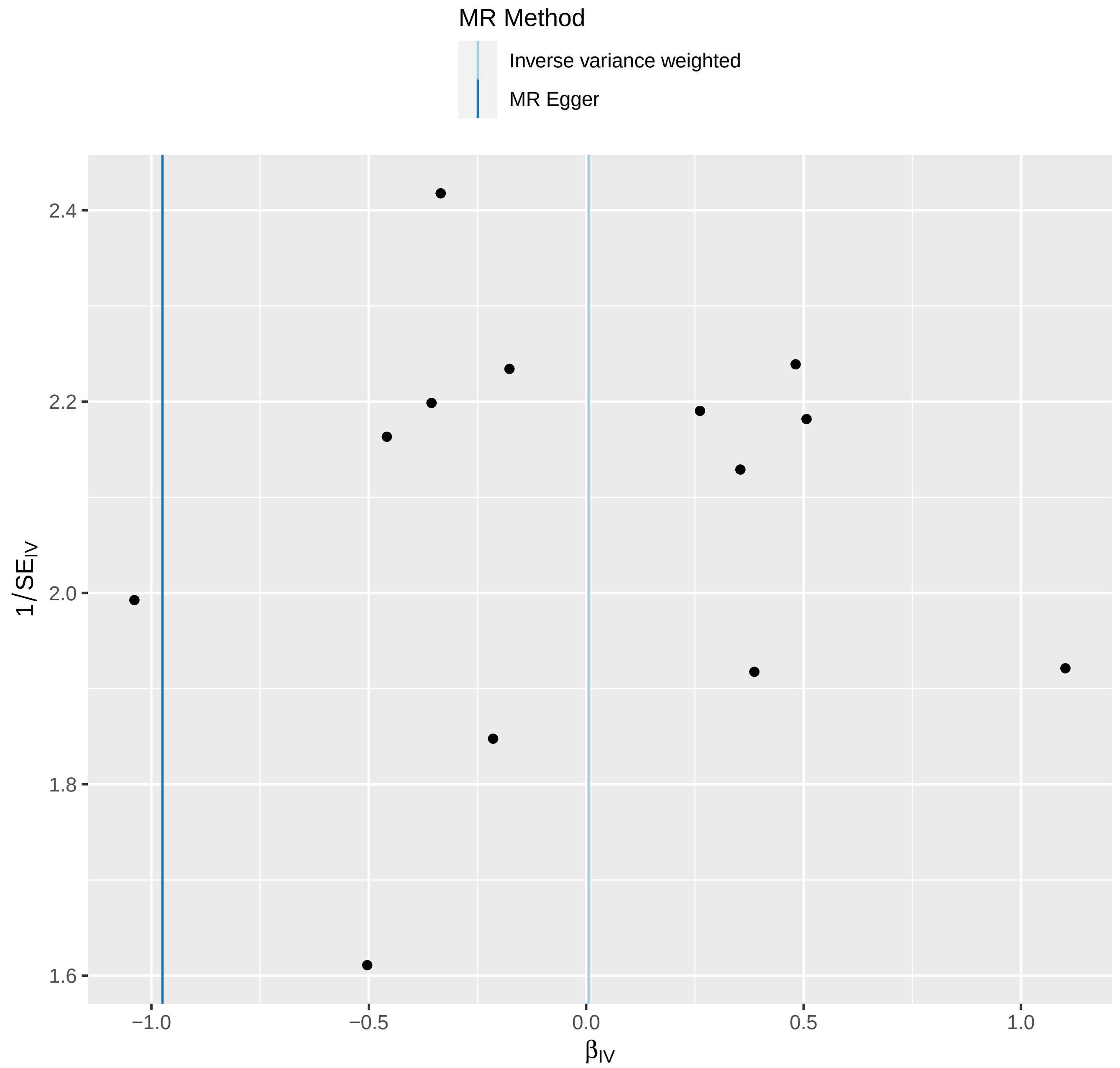

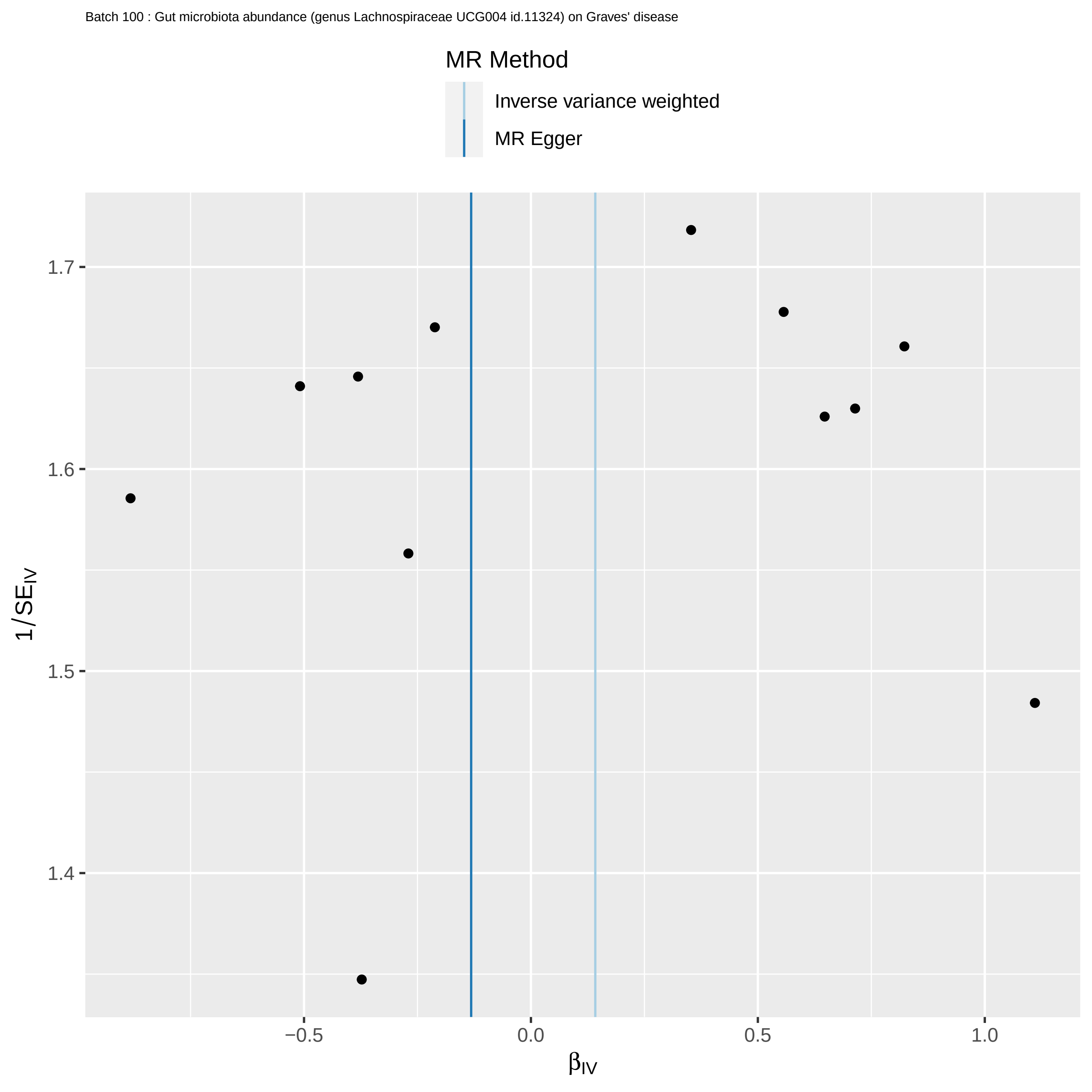

Batch 100 : Gut microbiota abundance (genus Lachnospiraceae UCG004 id.11324) on Graves' disease
MR Method
Inverse variance weighted
MR Egger
1.7
1.6
1.5
1.4
1/SE$_{IV}$
−0.5
0.0
0.5
1.0
β_{IV}

Batch 101 : Gut microbiota abundance (genus Lachnospiraceae UCG008 id.11328) on Graves' disease

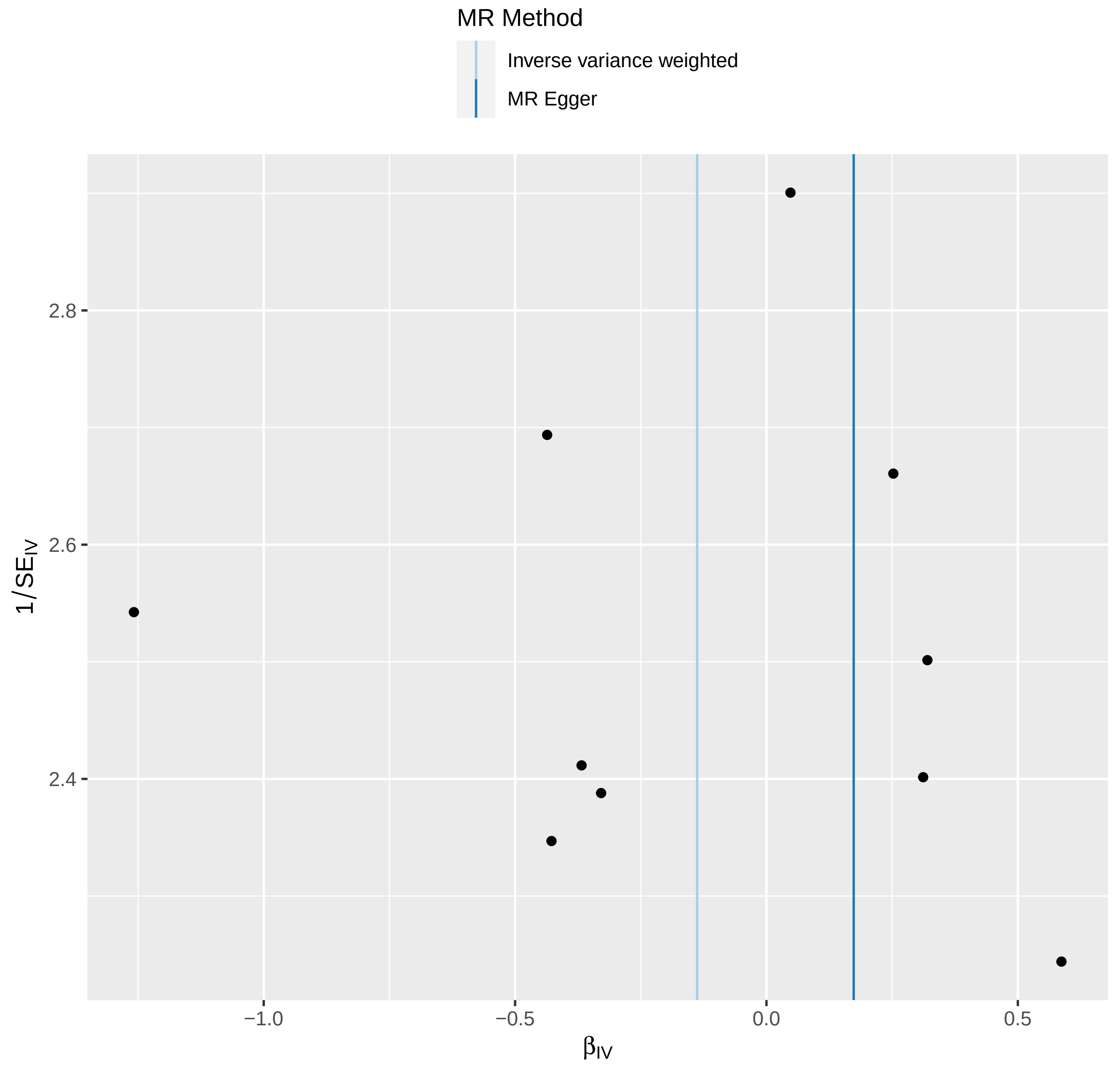

Batch 102 : Gut microbiota abundance (genus Lachnospiraceae UCG010 id.11330) on Graves' disease

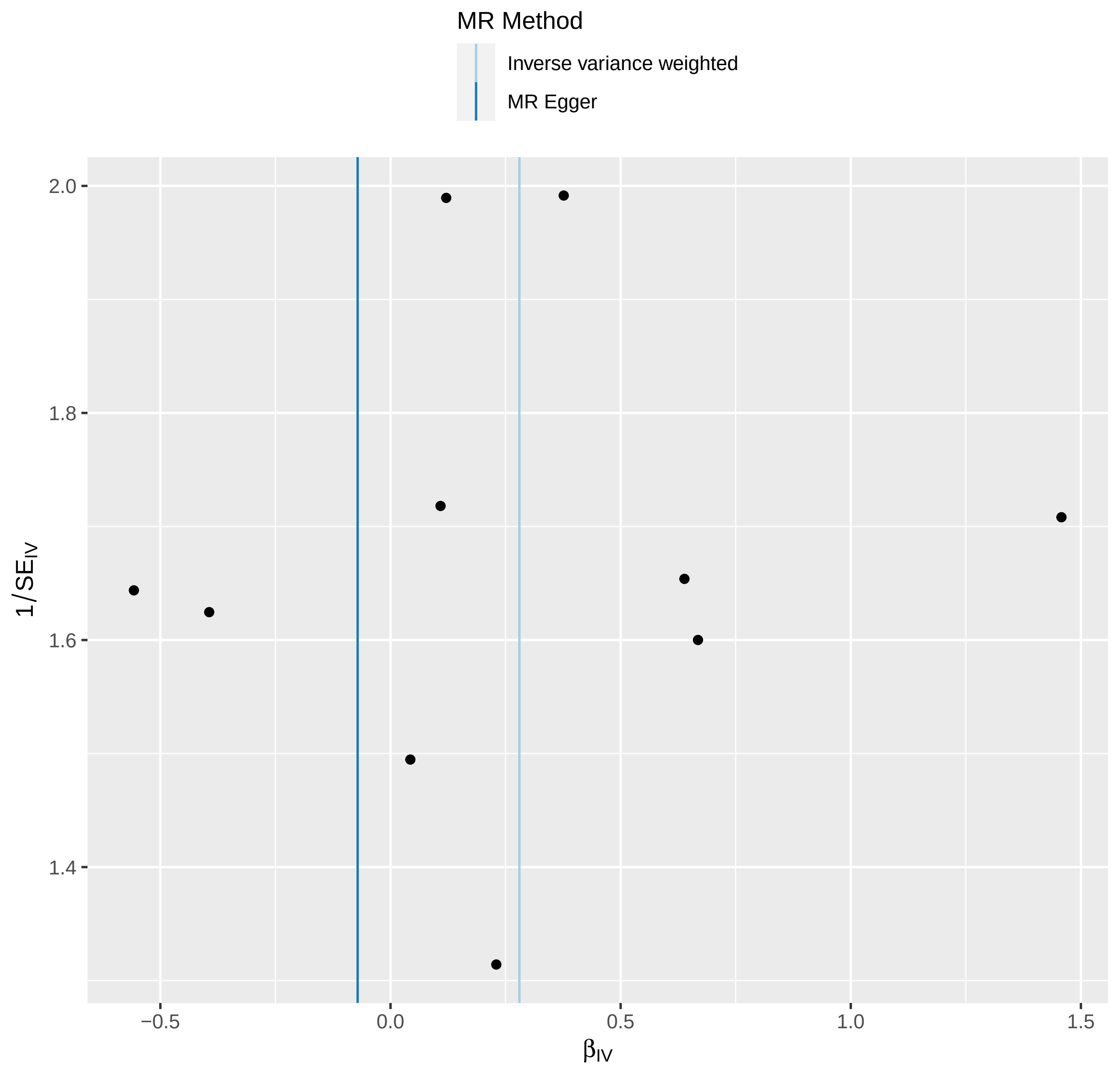

Batch 104 : Gut microbiota abundance (genus Lactobacillus id.1837) on Graves' disease

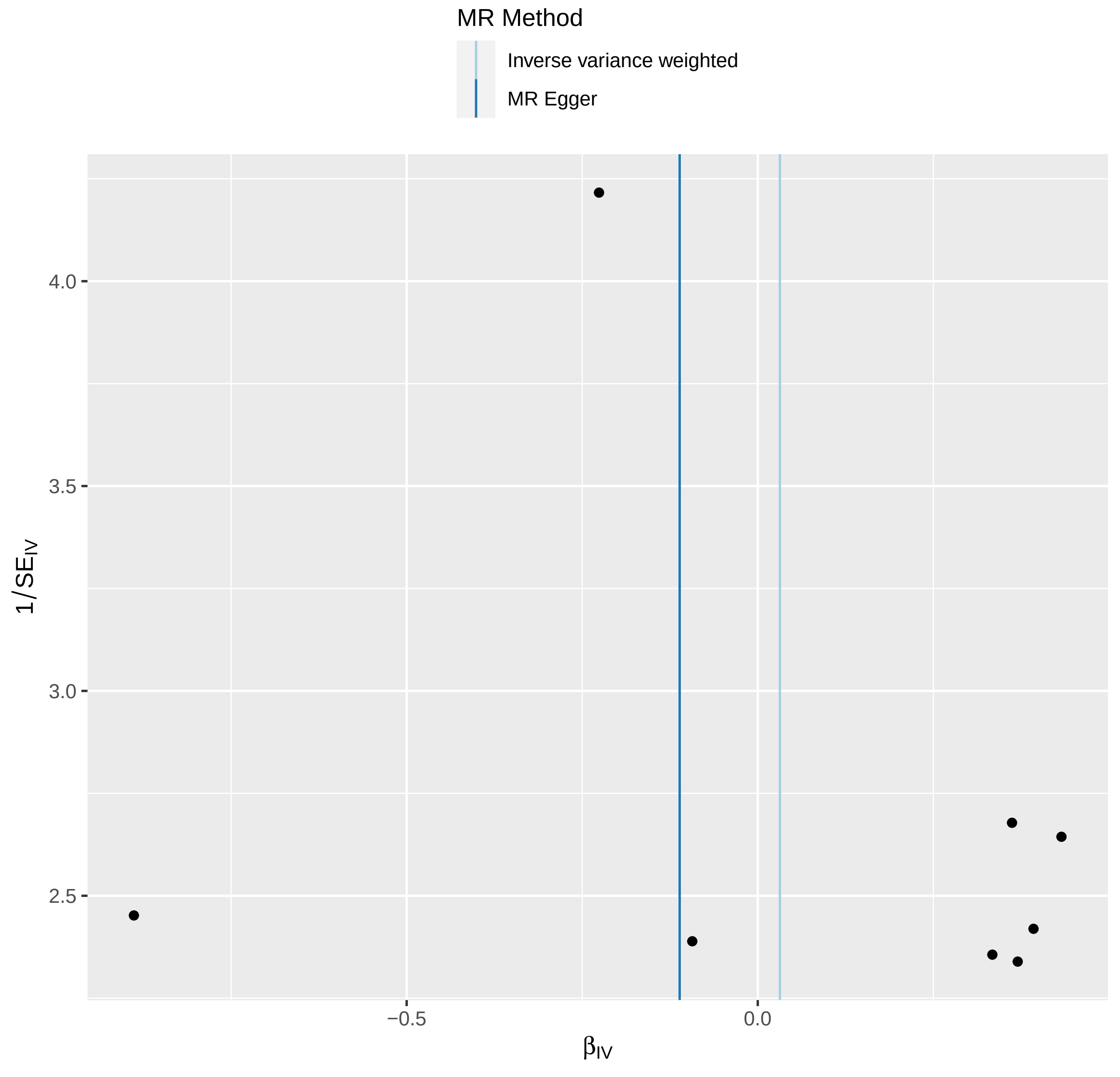

Batch 105 : Gut microbiota abundance (genus Lactococcus id.1851) on Graves' disease

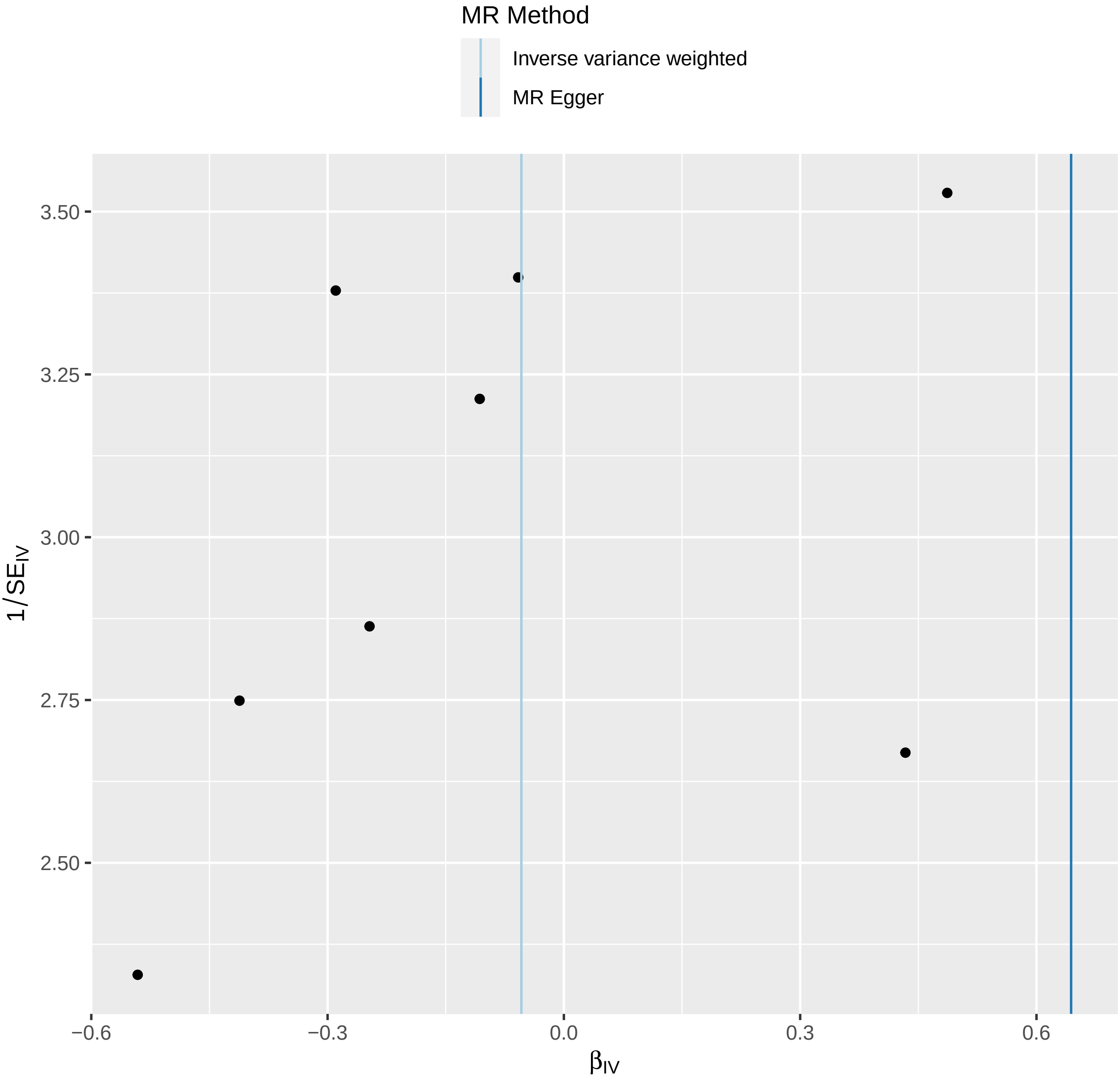

Batch 106 : Gut microbiota abundance (genus Marvinbryantia id.2005) on Graves' disease

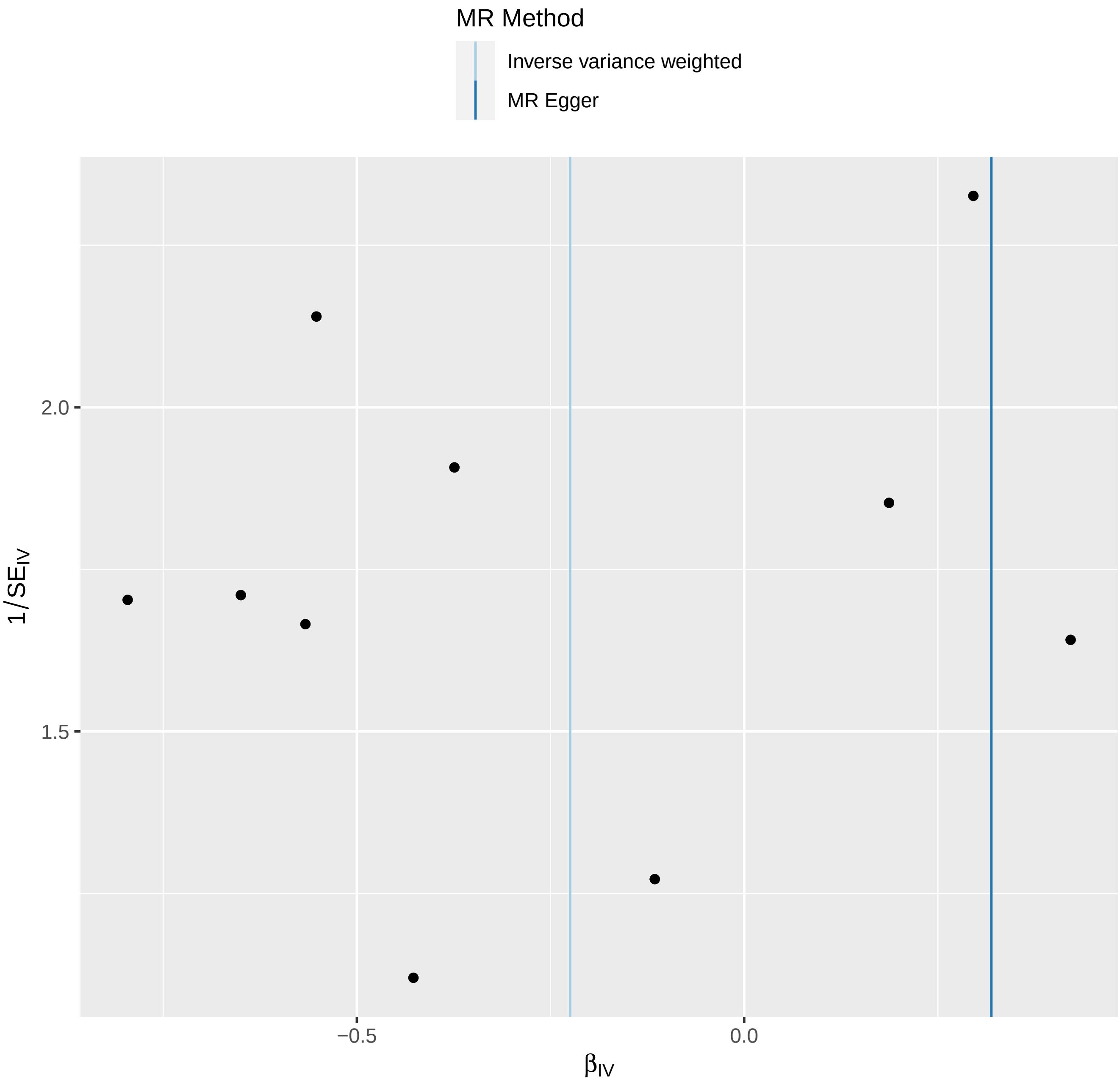

Batch 107 : Gut microbiota abundance (genus Methanobrevibacter id.123) on Graves' disease

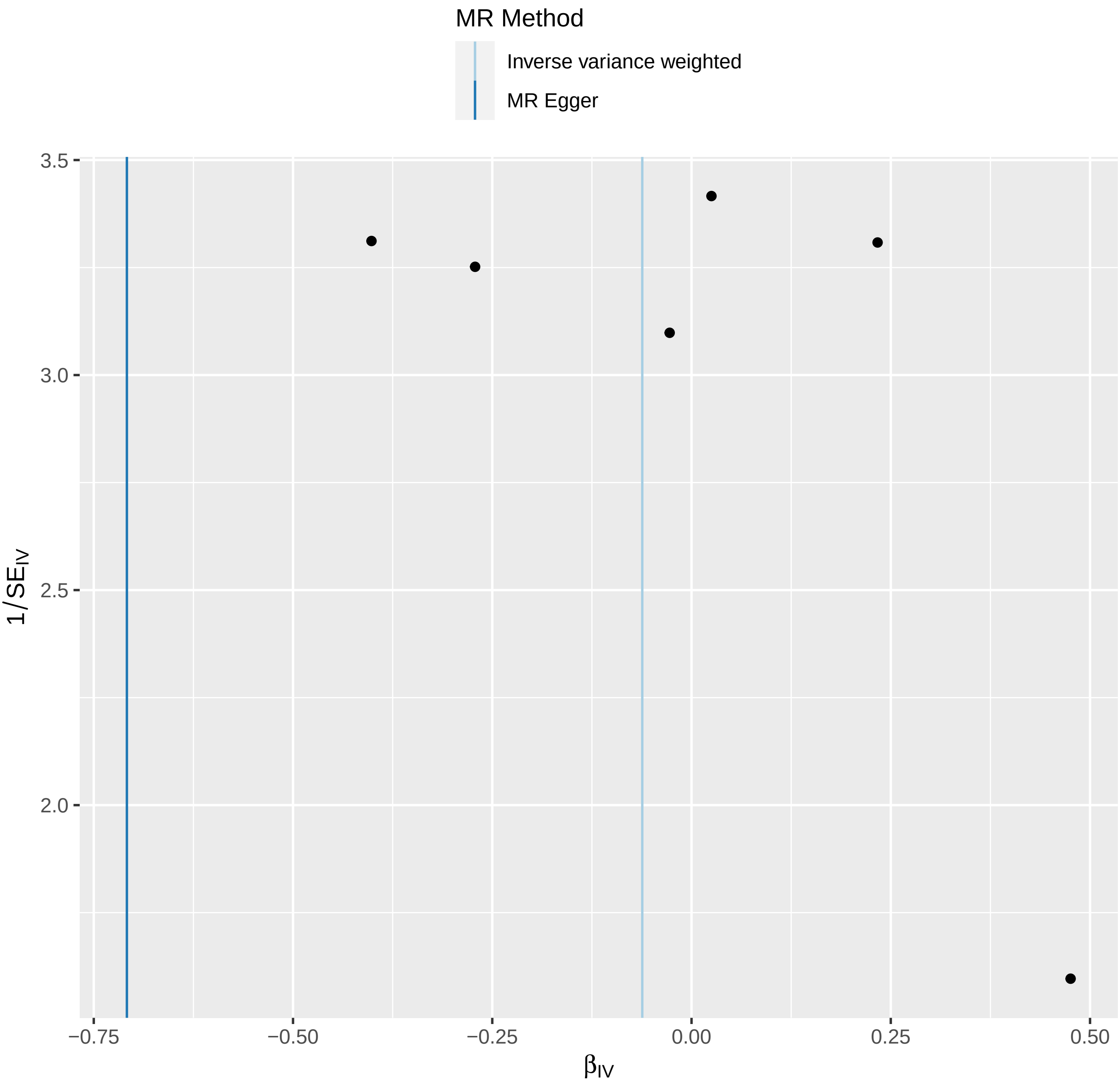

Batch 108 : Gut microbiota abundance (genus Odoribacter id.952) on Graves' disease

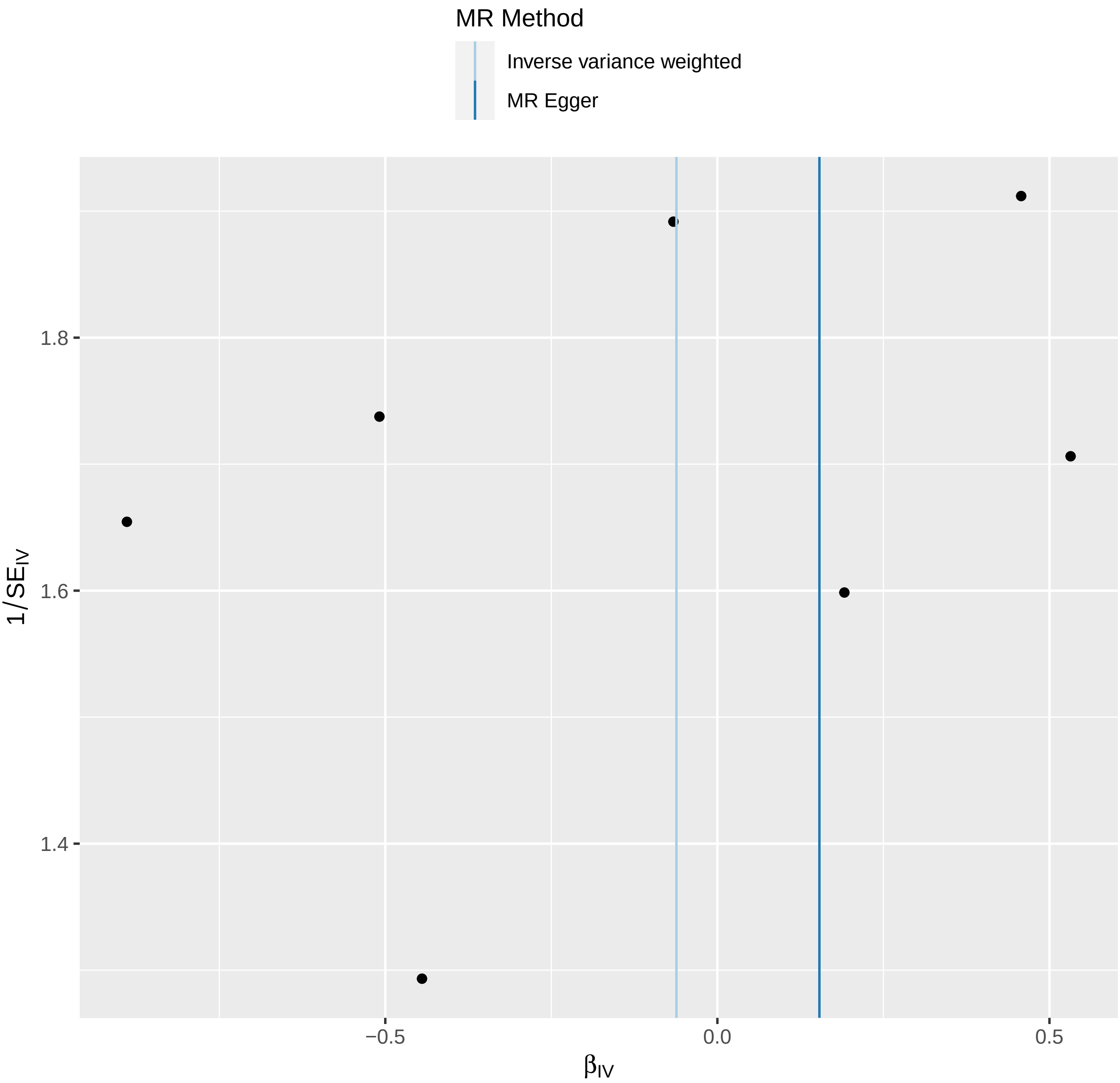

Batch 109 : Gut microbiota abundance (genus Olsenella id.822) on Graves' disease

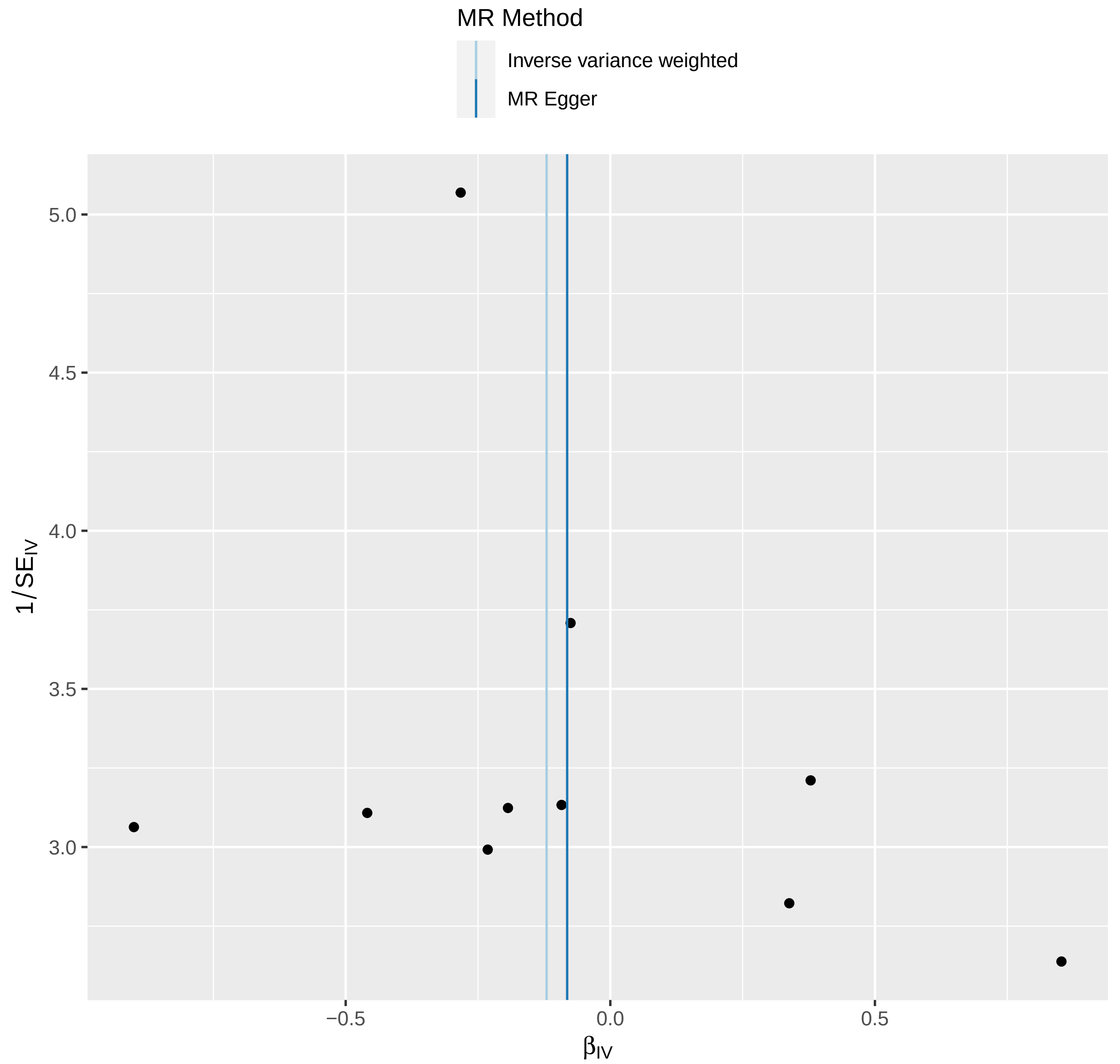

Batch 110 : Gut microbiota abundance (genus Oscillibacter id.2063) on Graves' disease

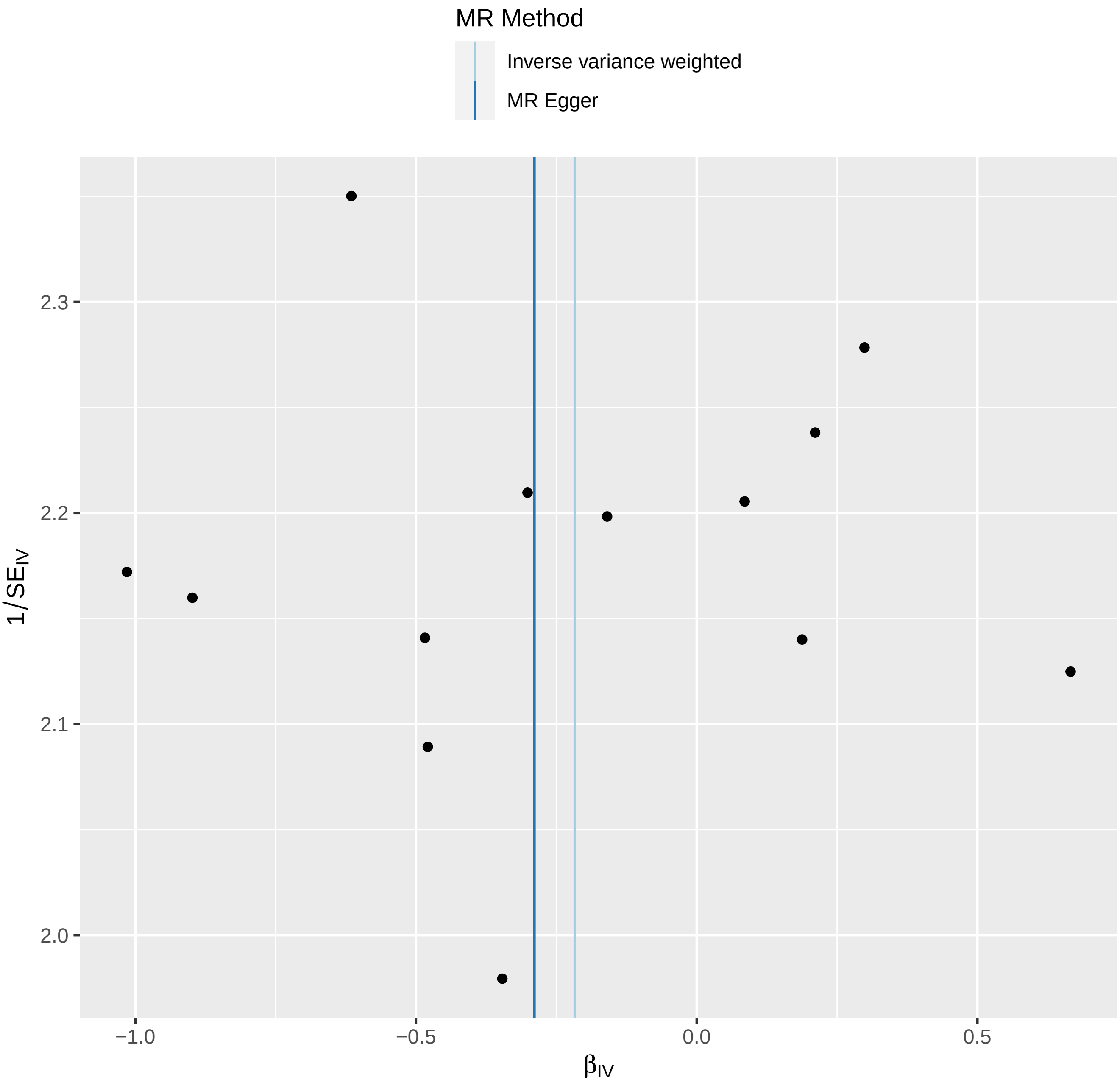

Batch 111 : Gut microbiota abundance (genus Oscillospira id.2064) on Graves' disease

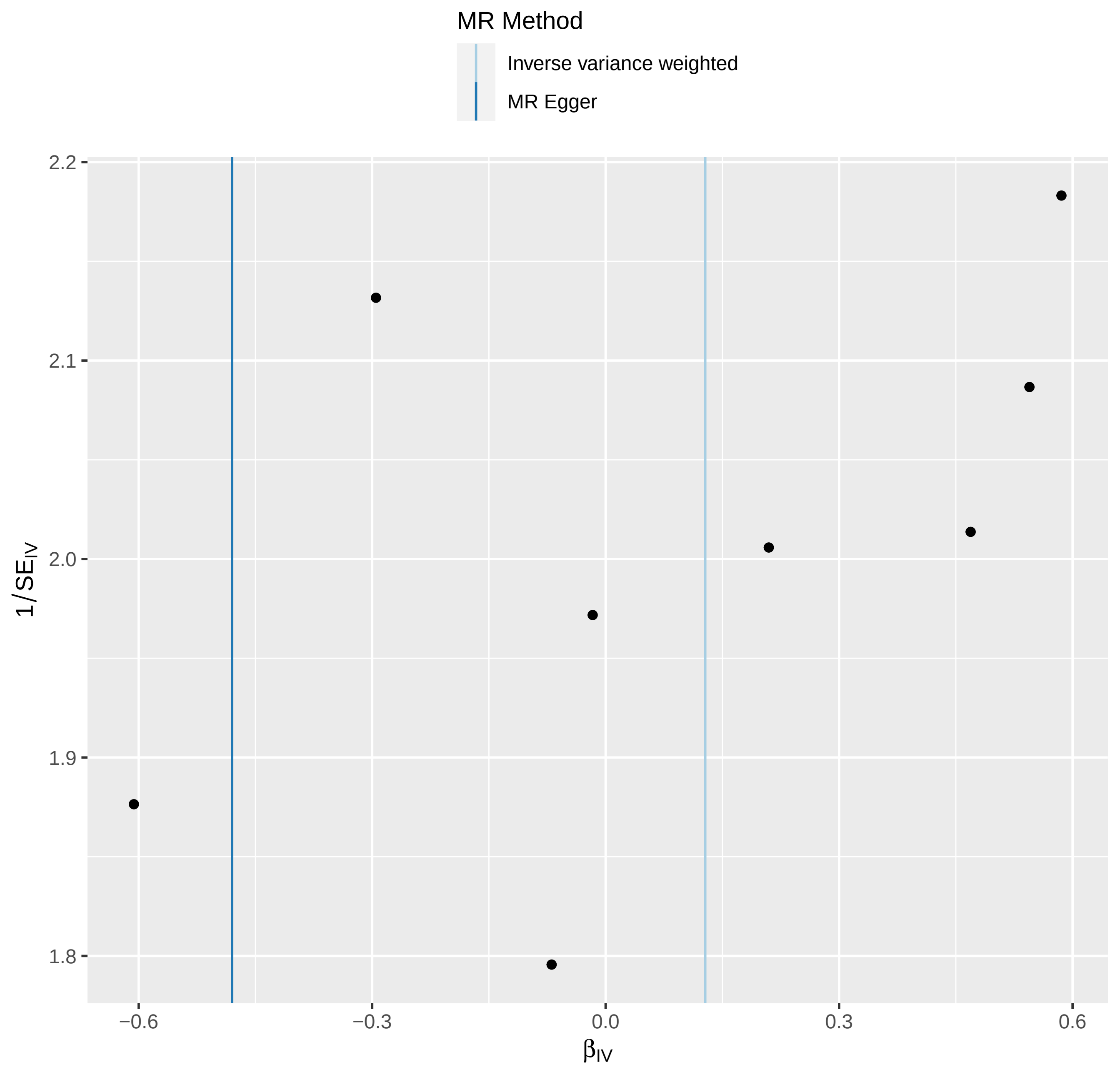

Batch 112 : Gut microbiota abundance (genus Oxalobacter id.2978) on Graves' disease

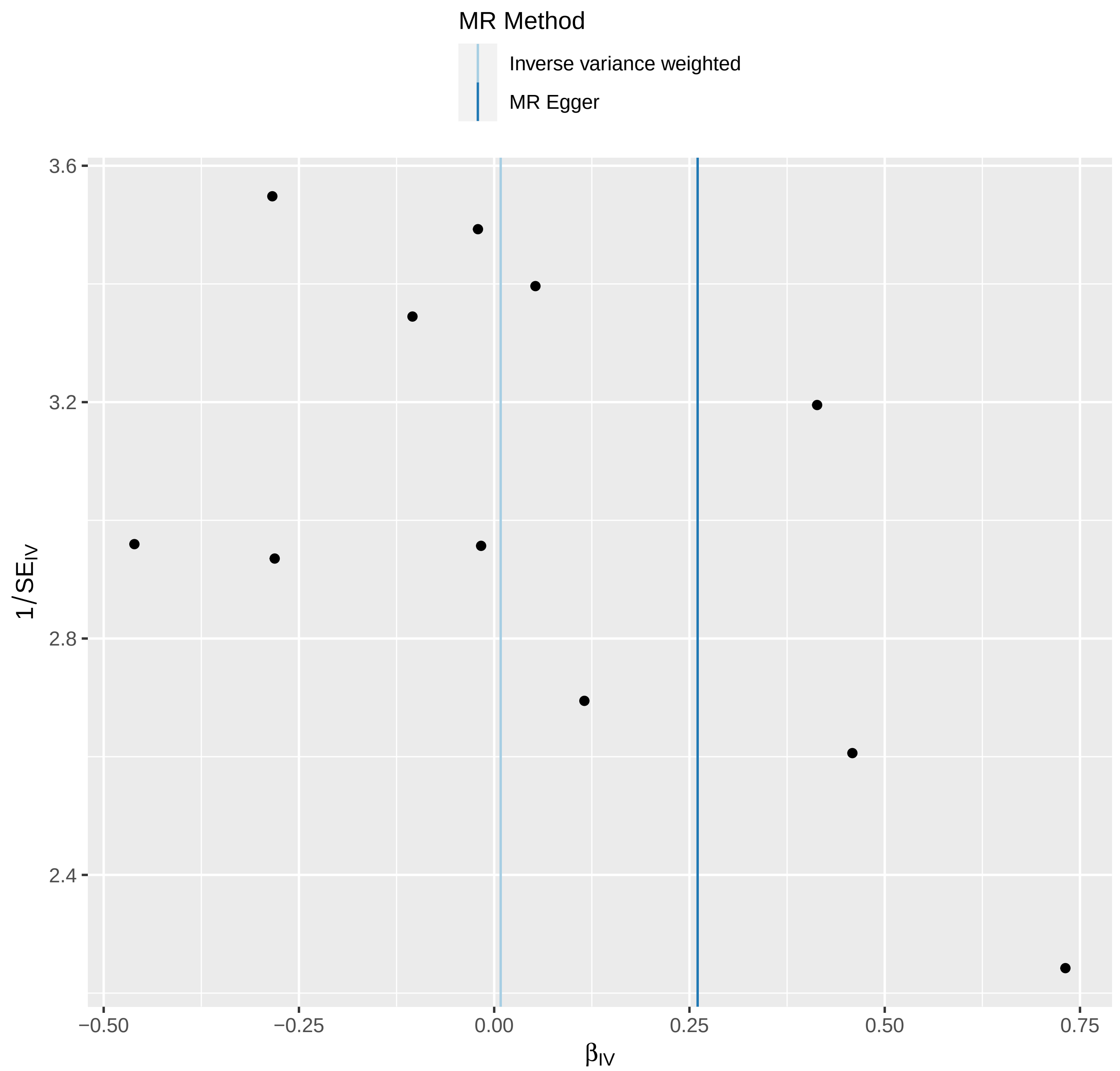

Batch 113 : Gut microbiota abundance (genus Parabacteroides id.954) on Graves' disease

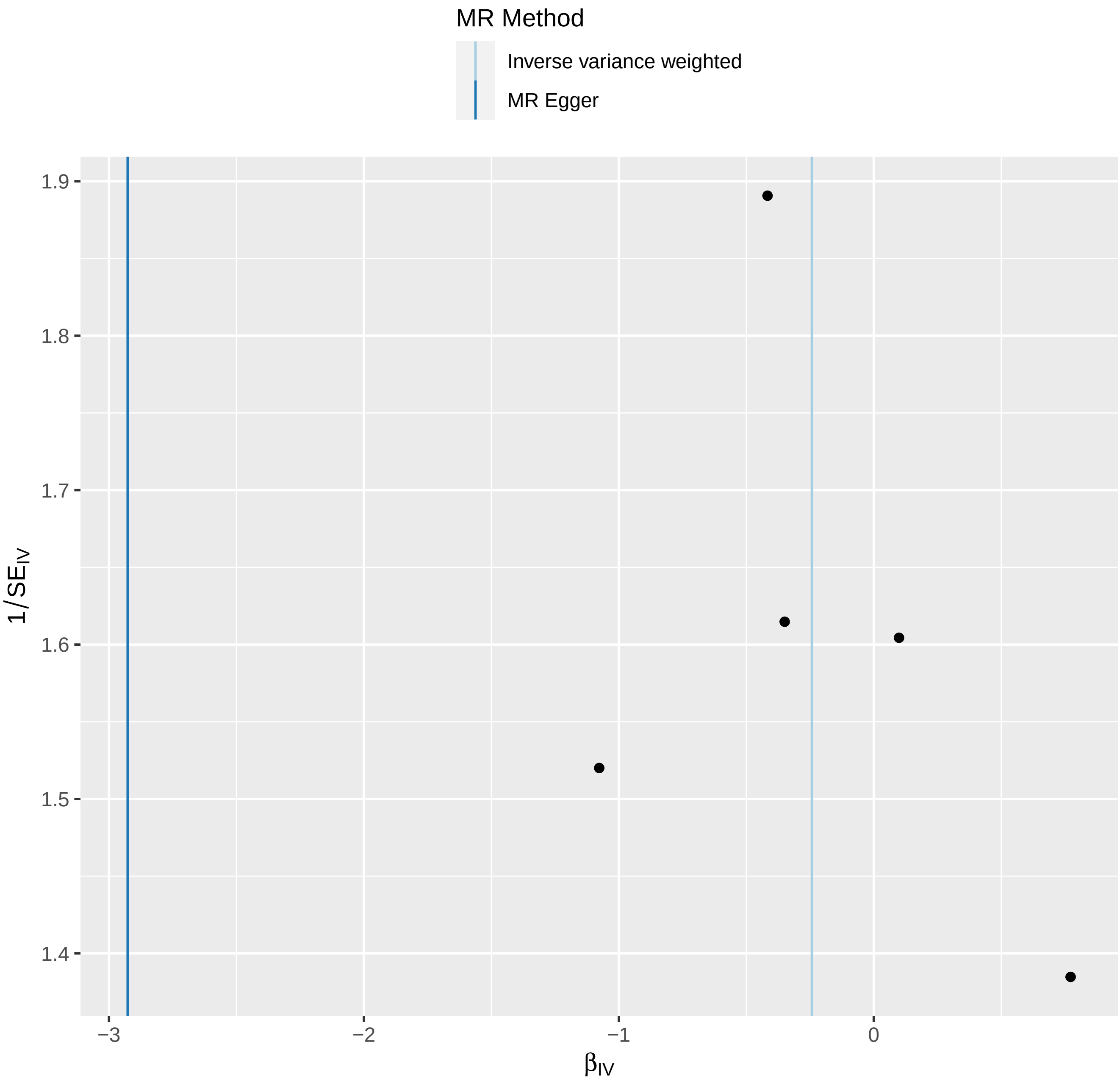

Batch 114 : Gut microbiota abundance (genus Paraprevotella id.962) on Graves' disease

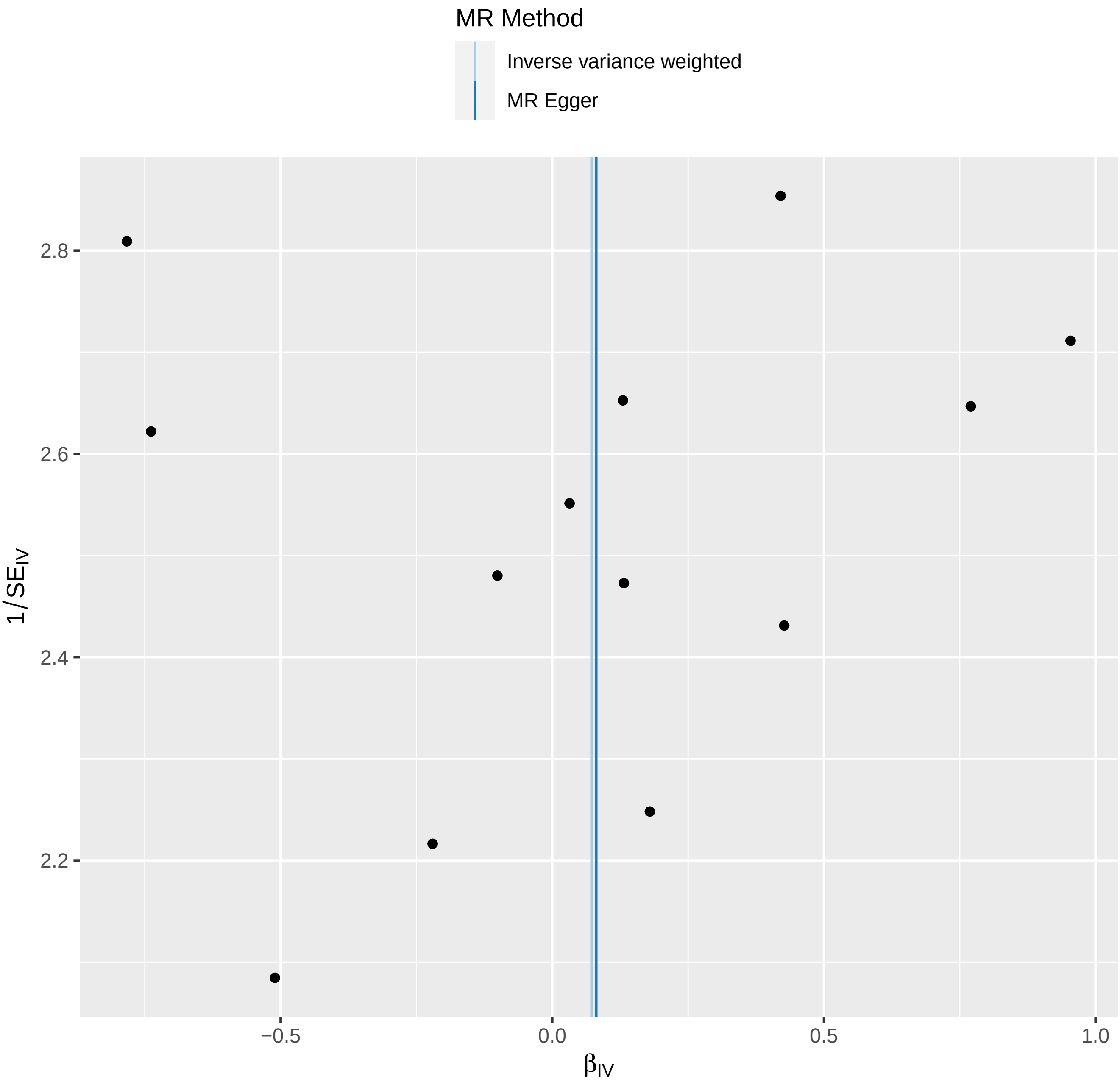

Batch 115 : Gut microbiota abundance (genus Parasutterella id.2892) on Graves' disease

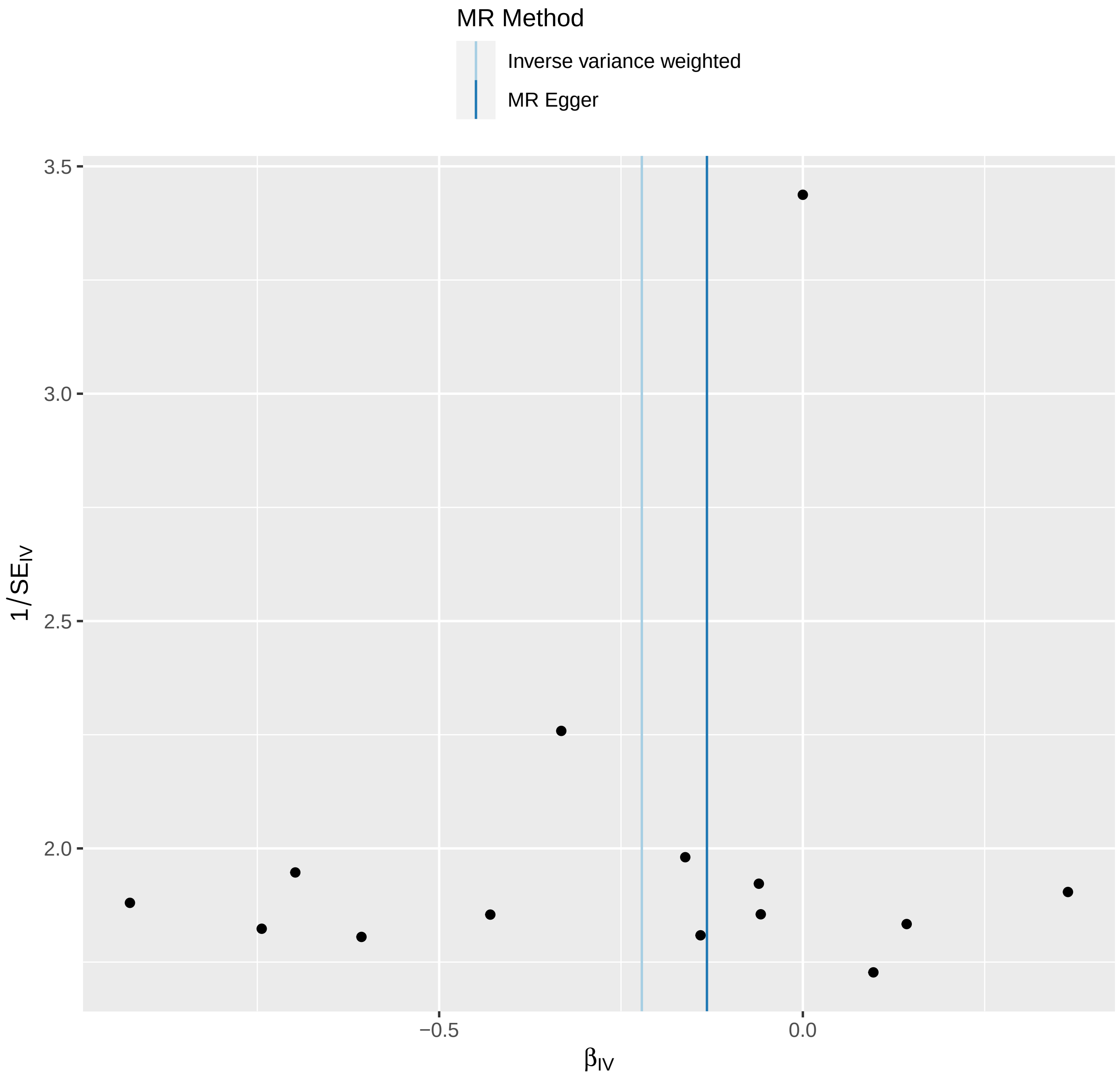

Batch 116 : Gut microbiota abundance (genus Peptococcus id.2037) on Graves' disease

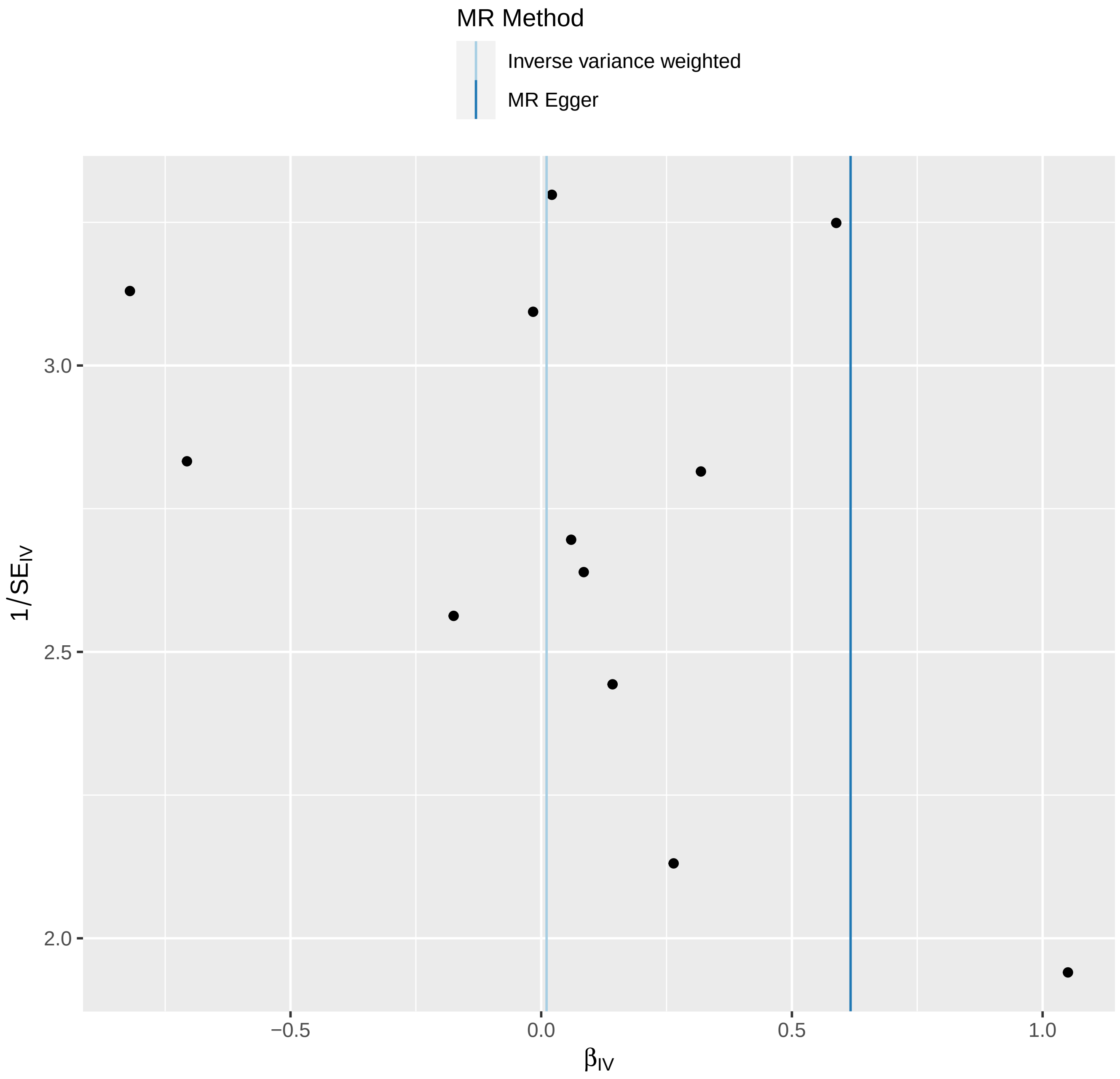

Batch 117 : Gut microbiota abundance (genus Phascolarctobacterium id.2168) on Graves' disease

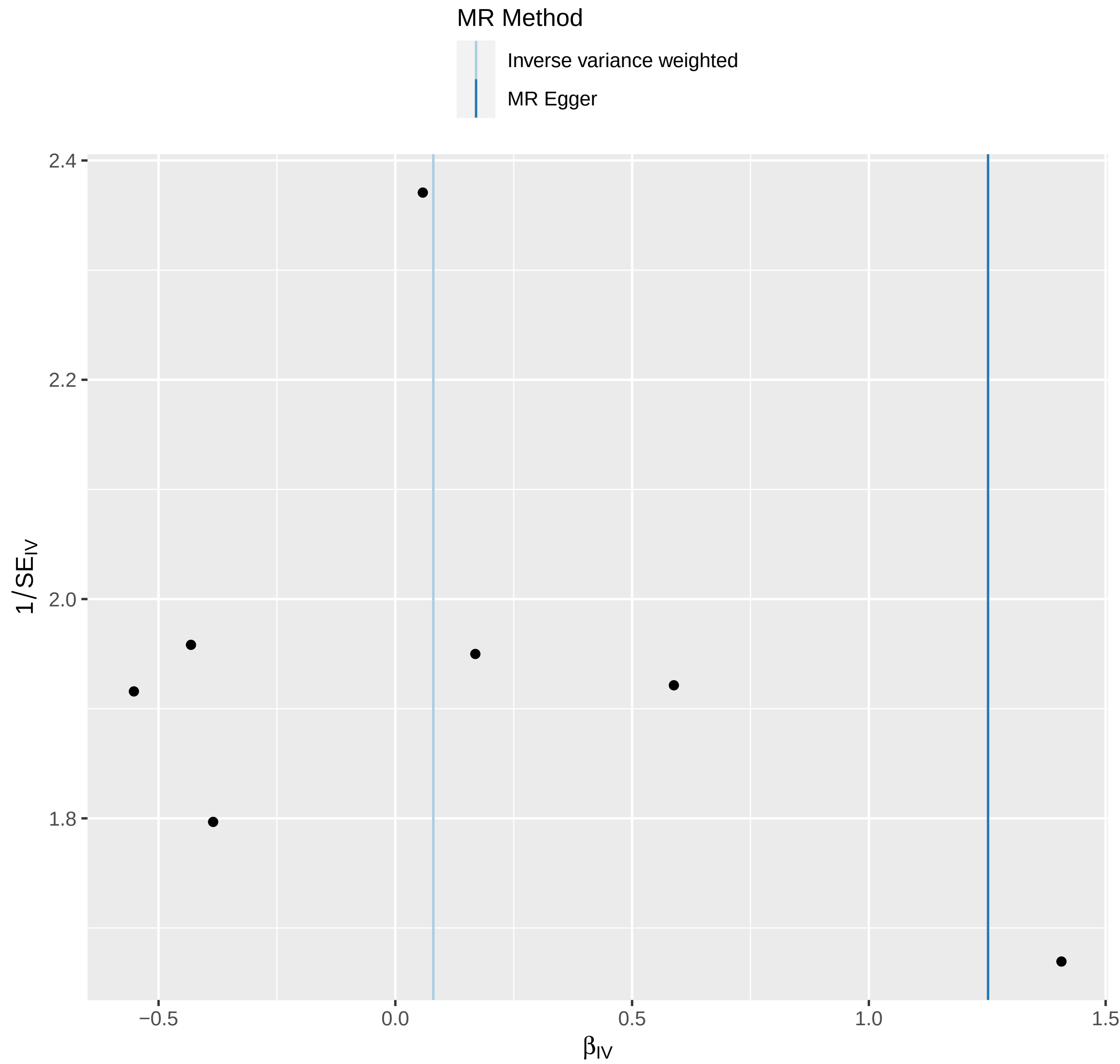

Batch 118 : Gut microbiota abundance (genus Prevotella7 id.11182) on Graves' disease

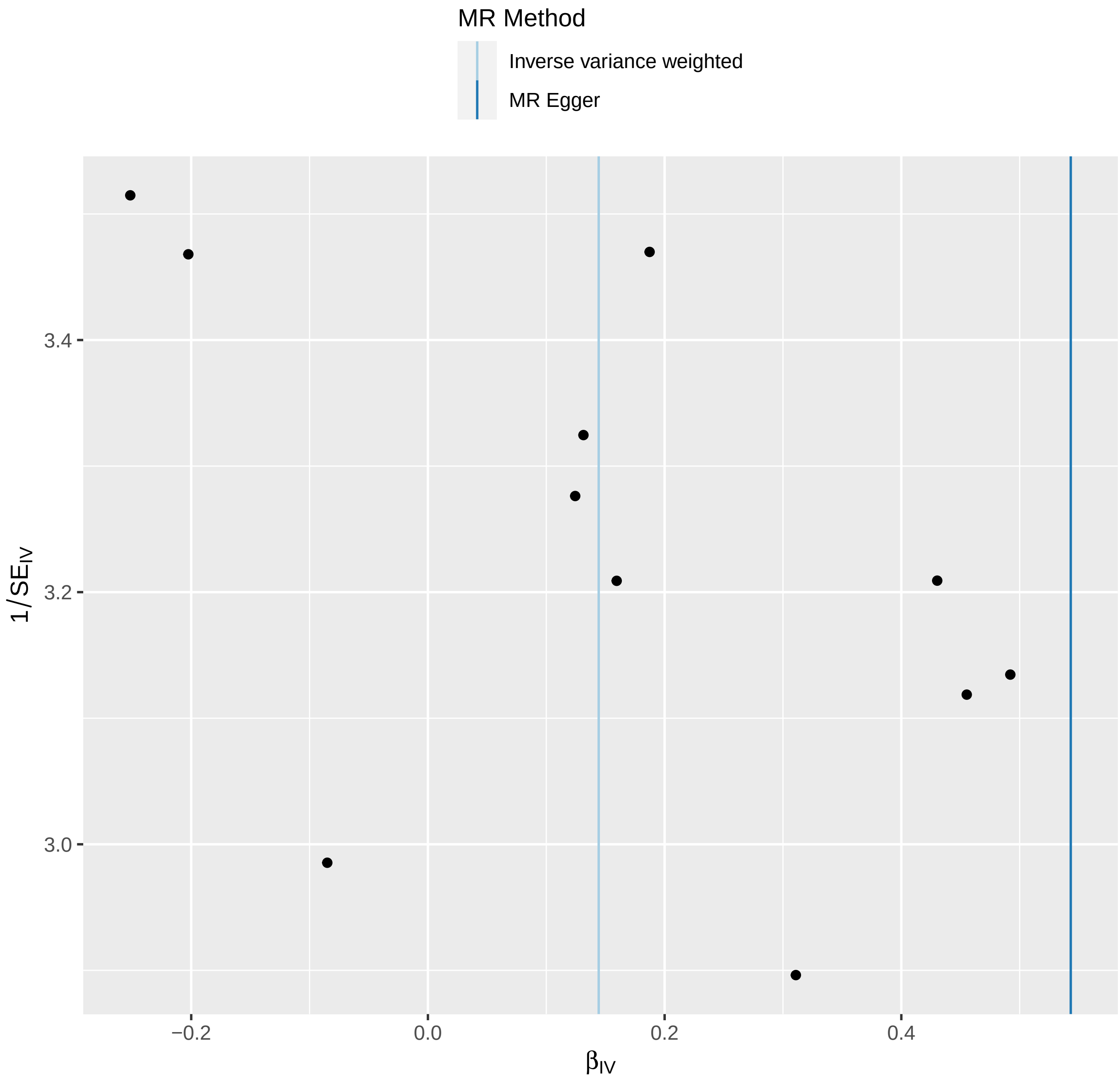

Batch 119 : Gut microbiota abundance (genus Prevotella9 id.11183) on Graves' disease

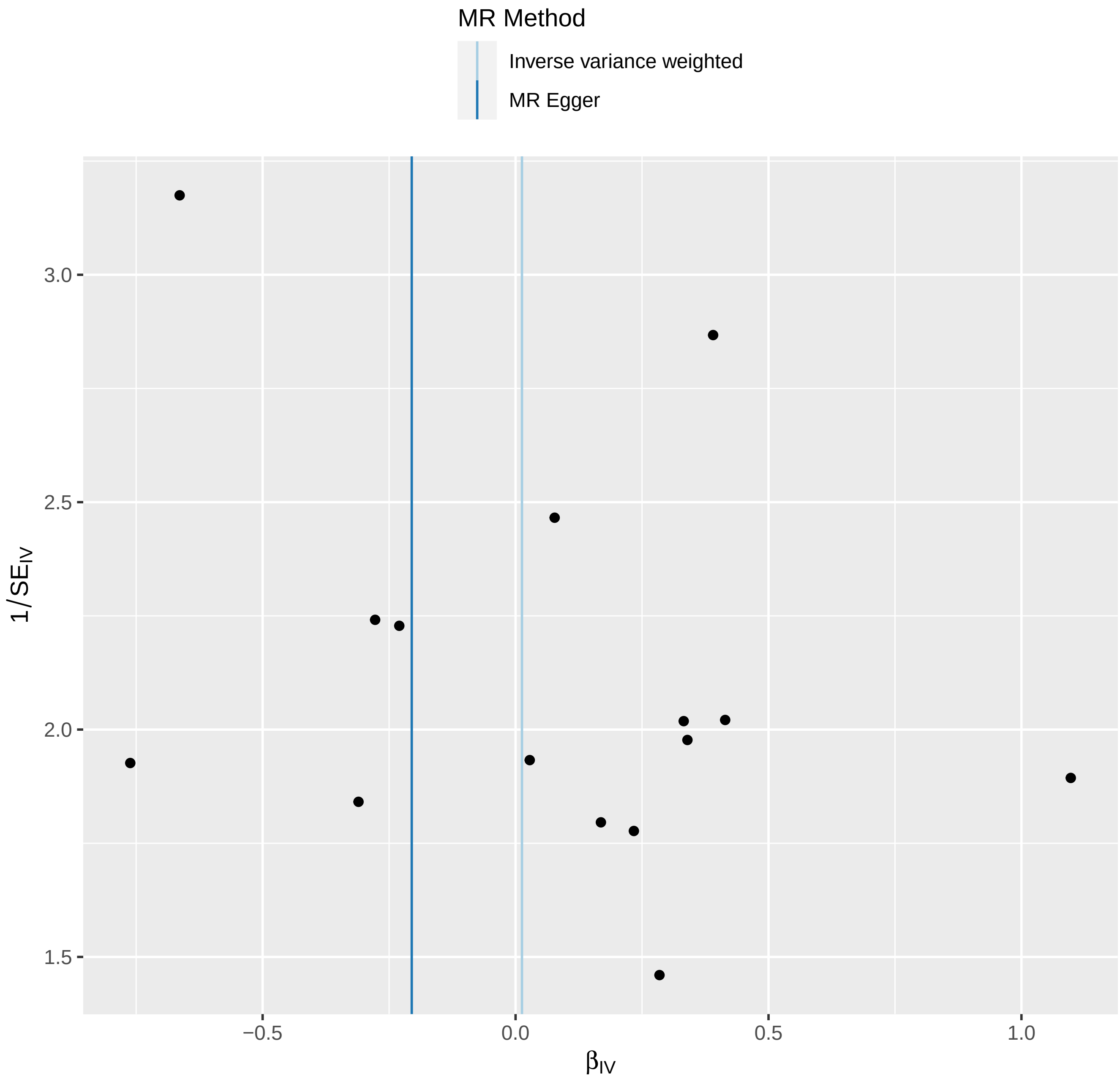

Batch 120 : Gut microbiota abundance (genus Rikenellaceae RC9 gut group id.11191) on Graves' disease

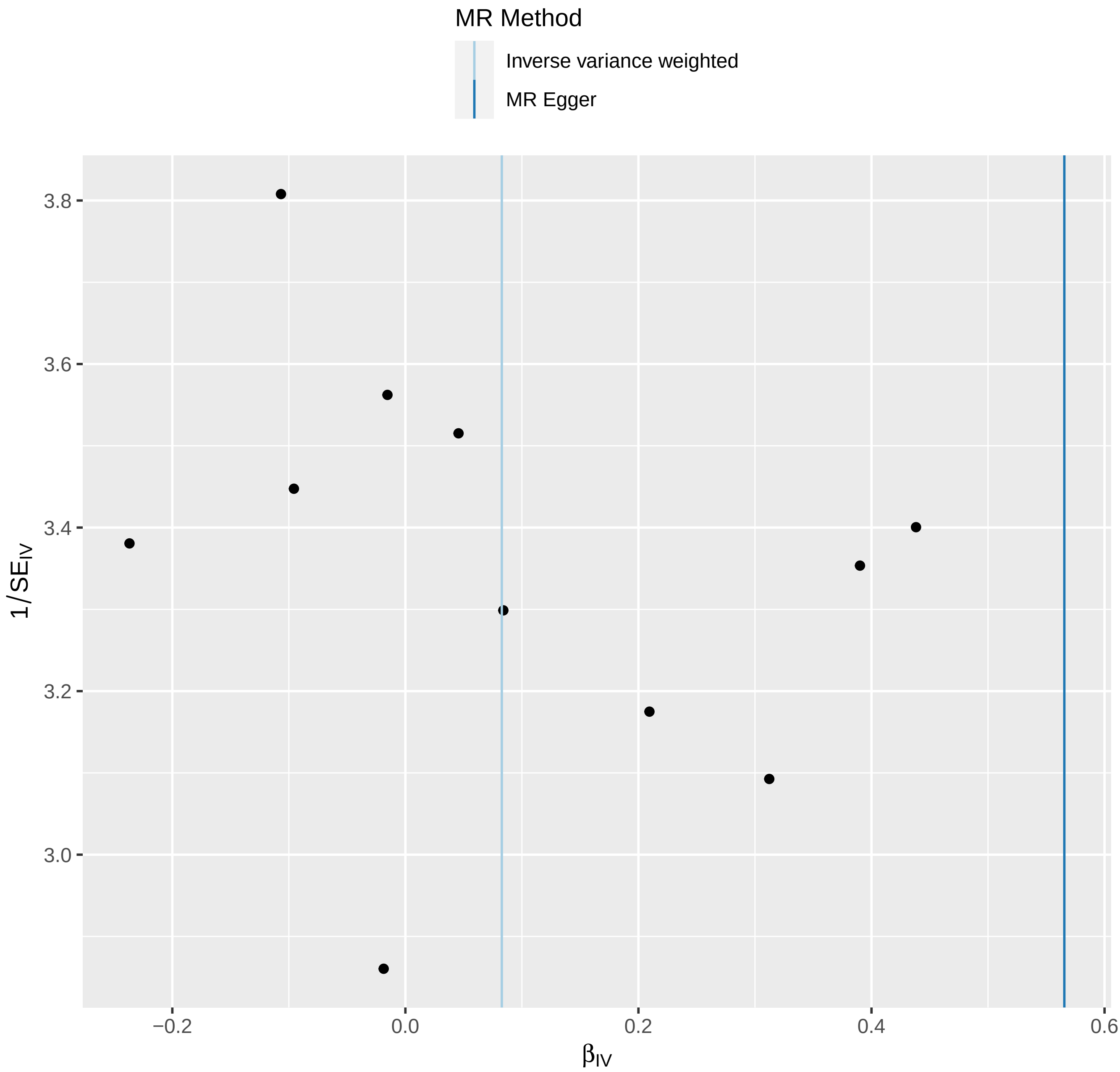

Batch 121 : Gut microbiota abundance (genus Romboutsia id.11347) on Graves' disease

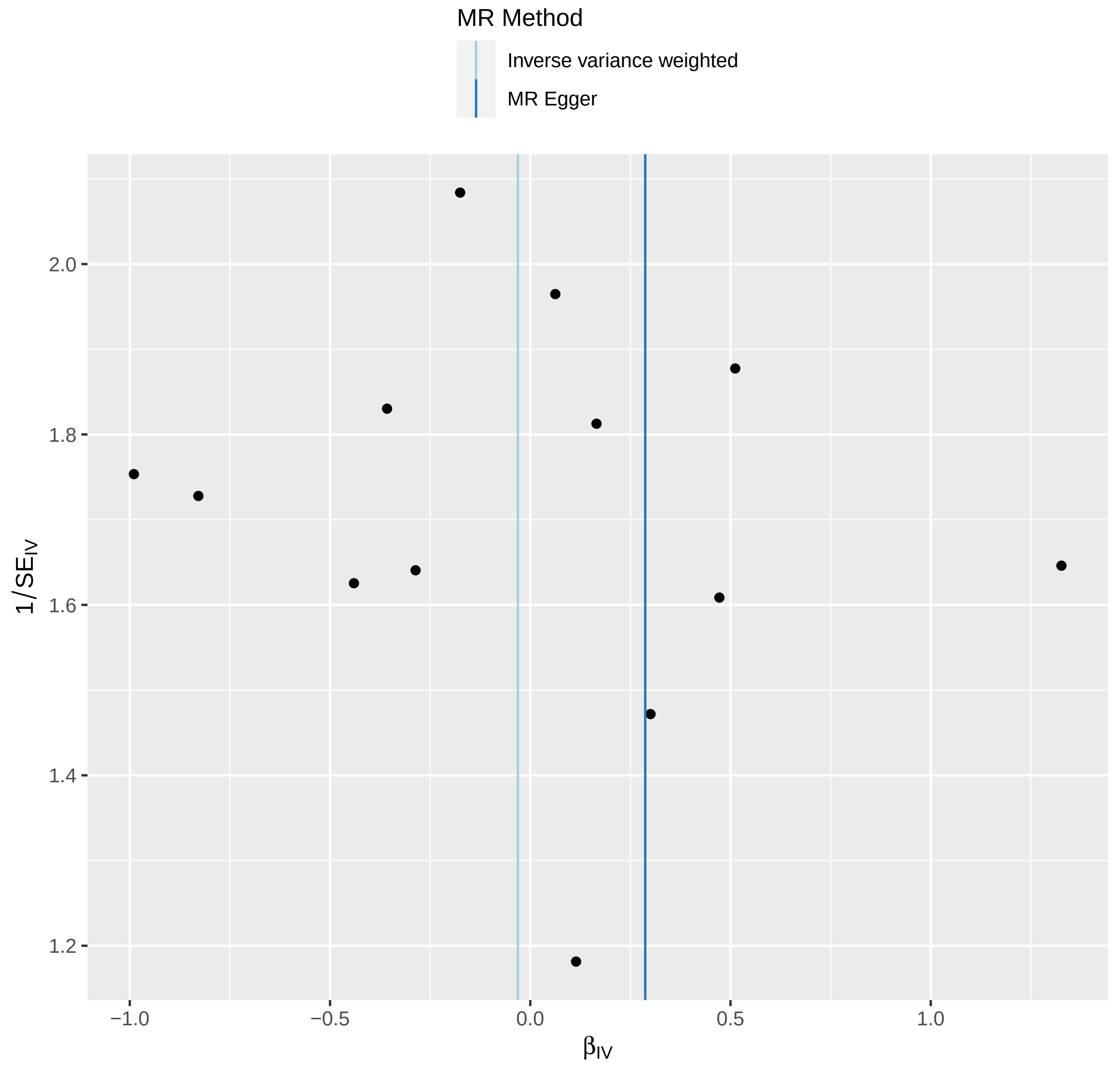

Batch 122 : Gut microbiota abundance (genus Roseburia id.2012) on Graves' disease

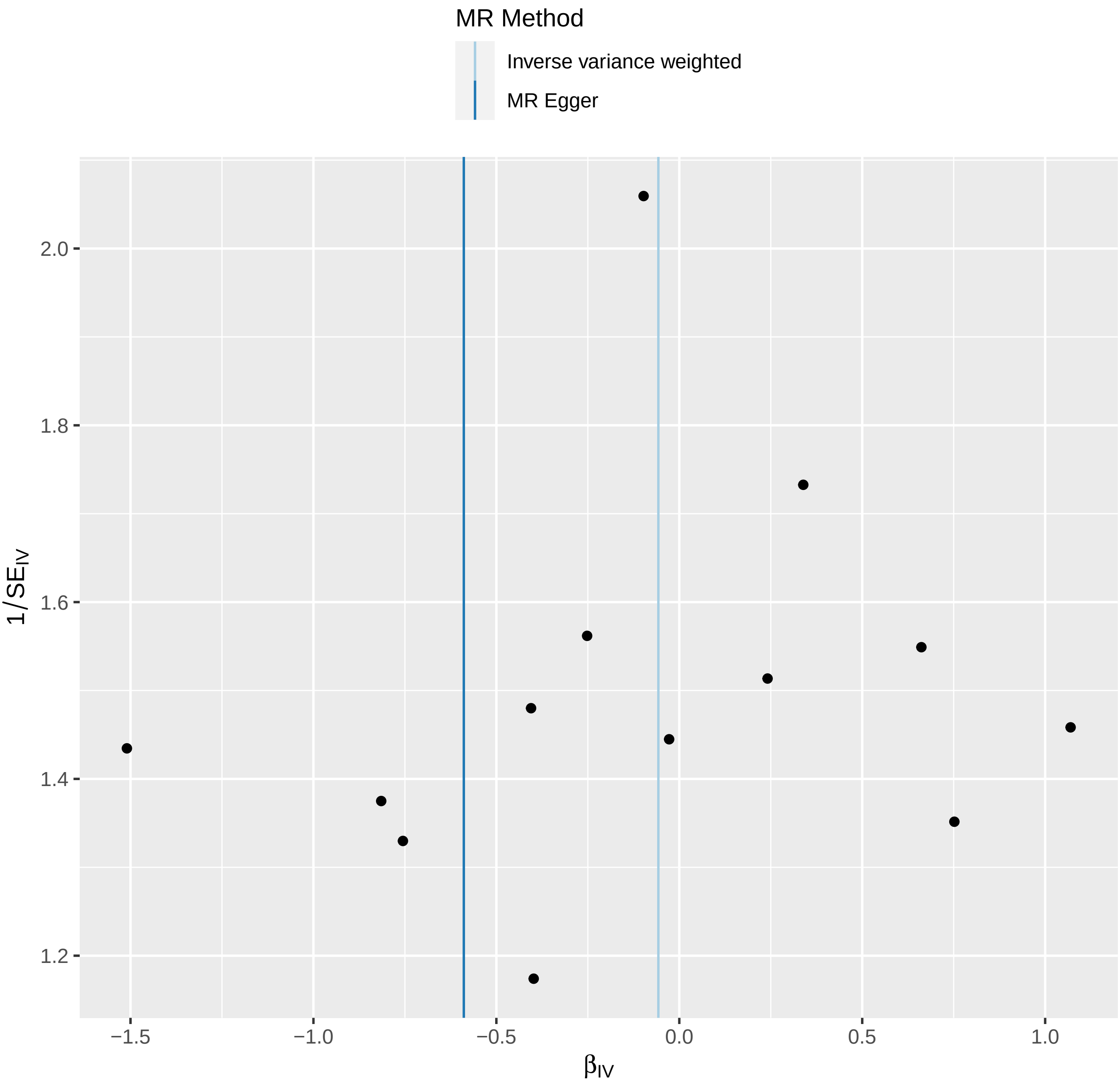

Batch 123 : Gut microbiota abundance (genus Ruminiclostridium5 id.11355) on Graves' disease

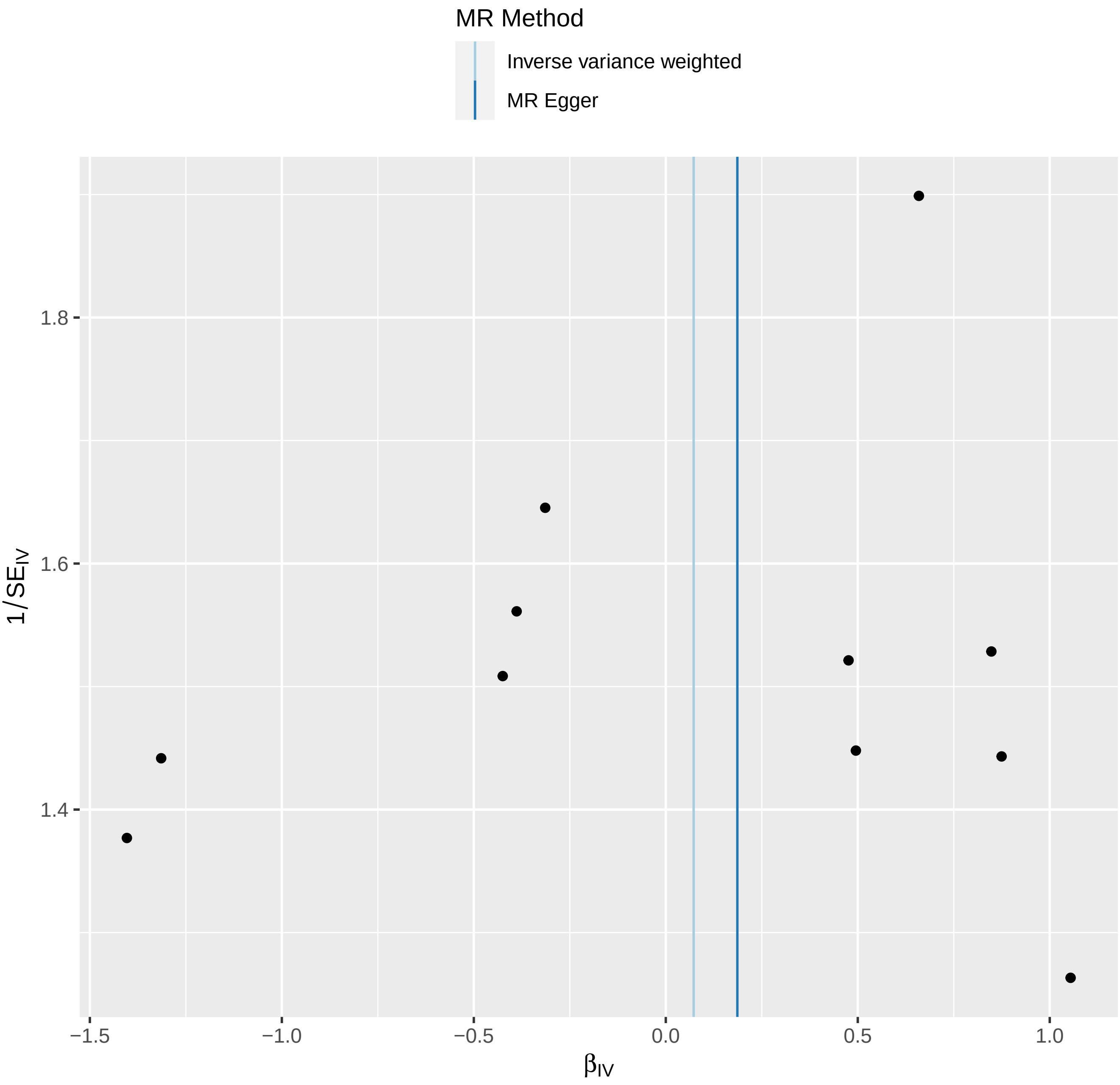

Batch 124 : Gut microbiota abundance (genus Ruminiclostridium6 id.11356) on Graves' disease

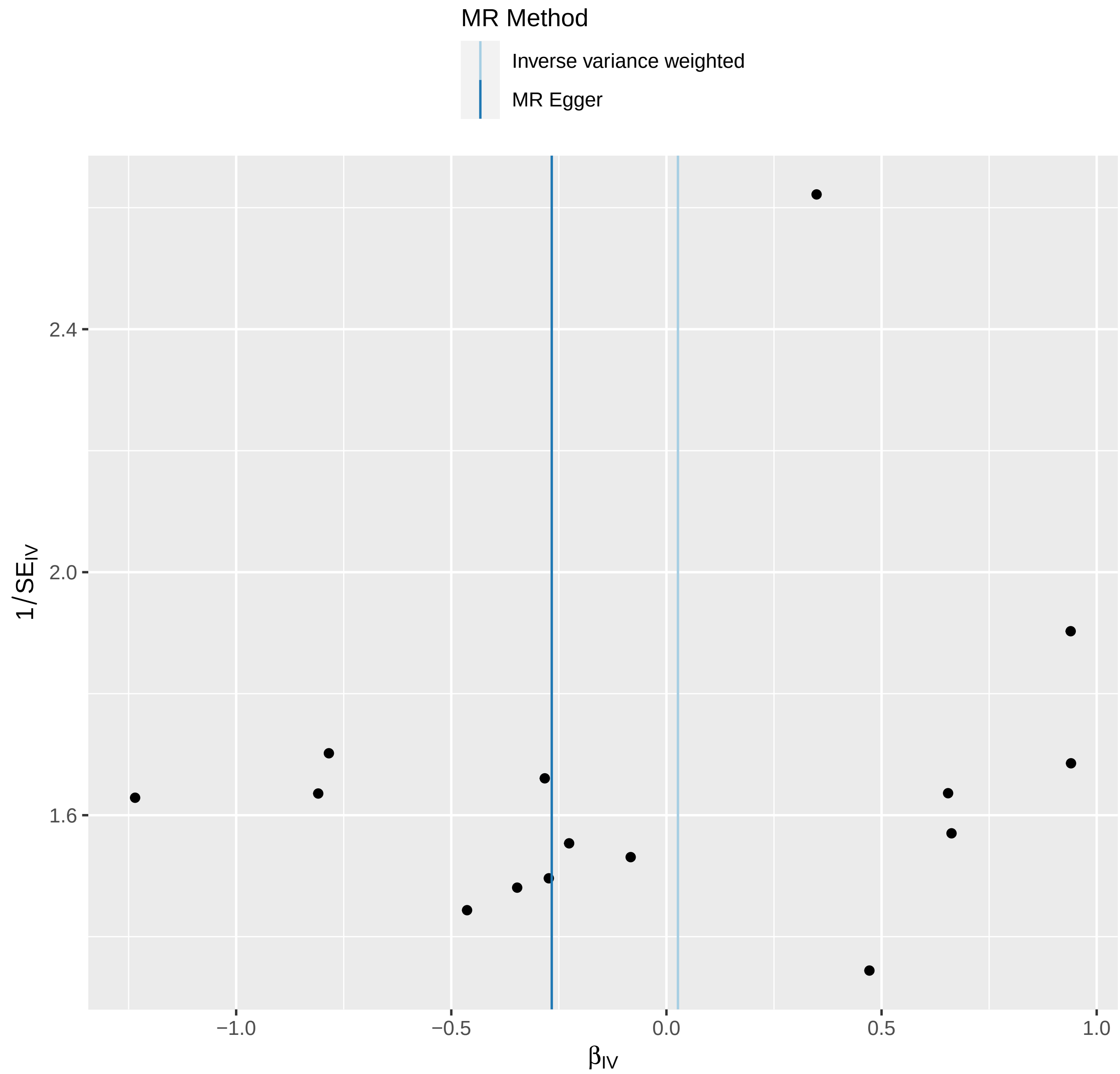

Batch 125 : Gut microbiota abundance (genus Ruminiclostridium9 id.11357) on Graves' disease

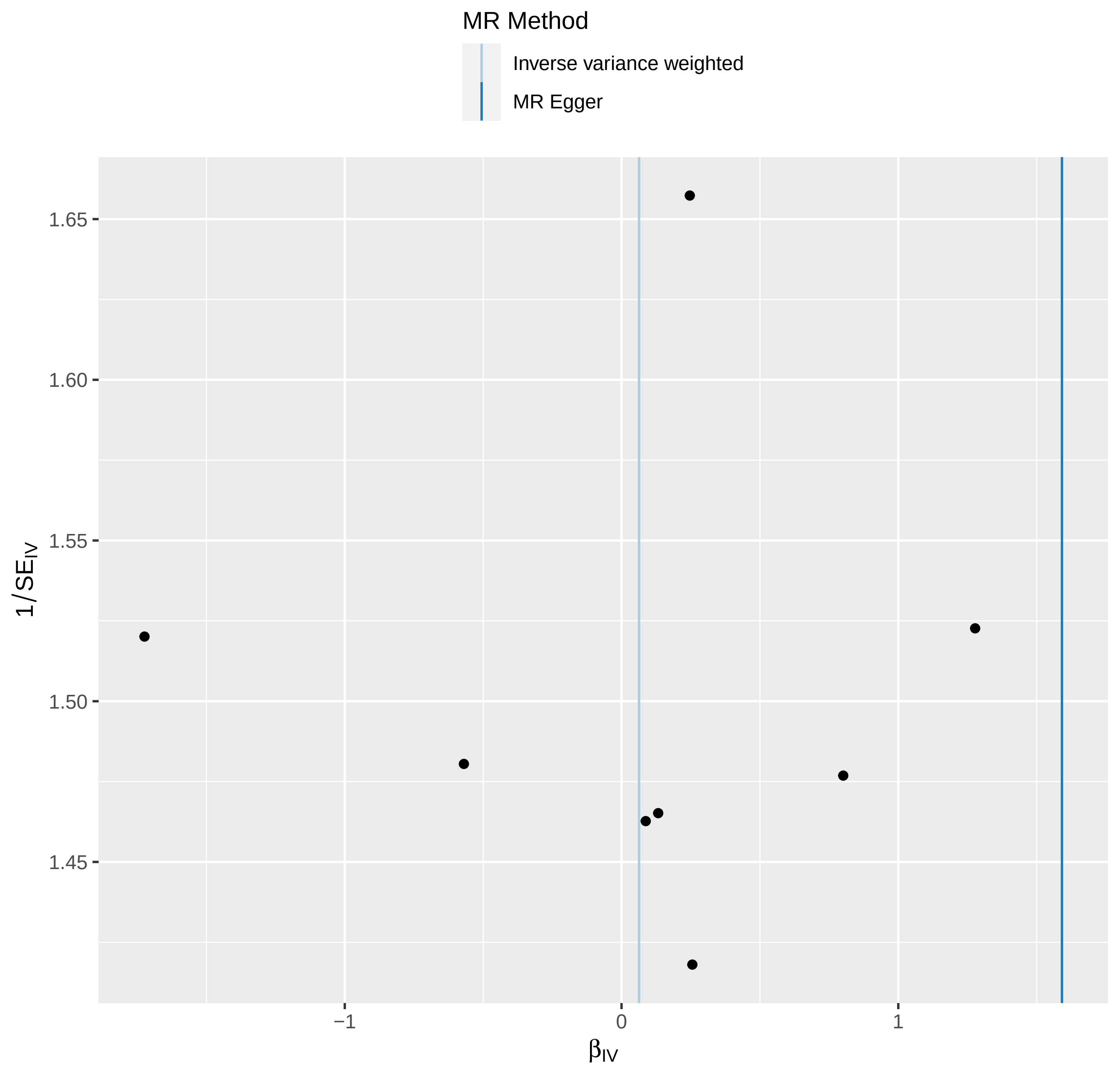

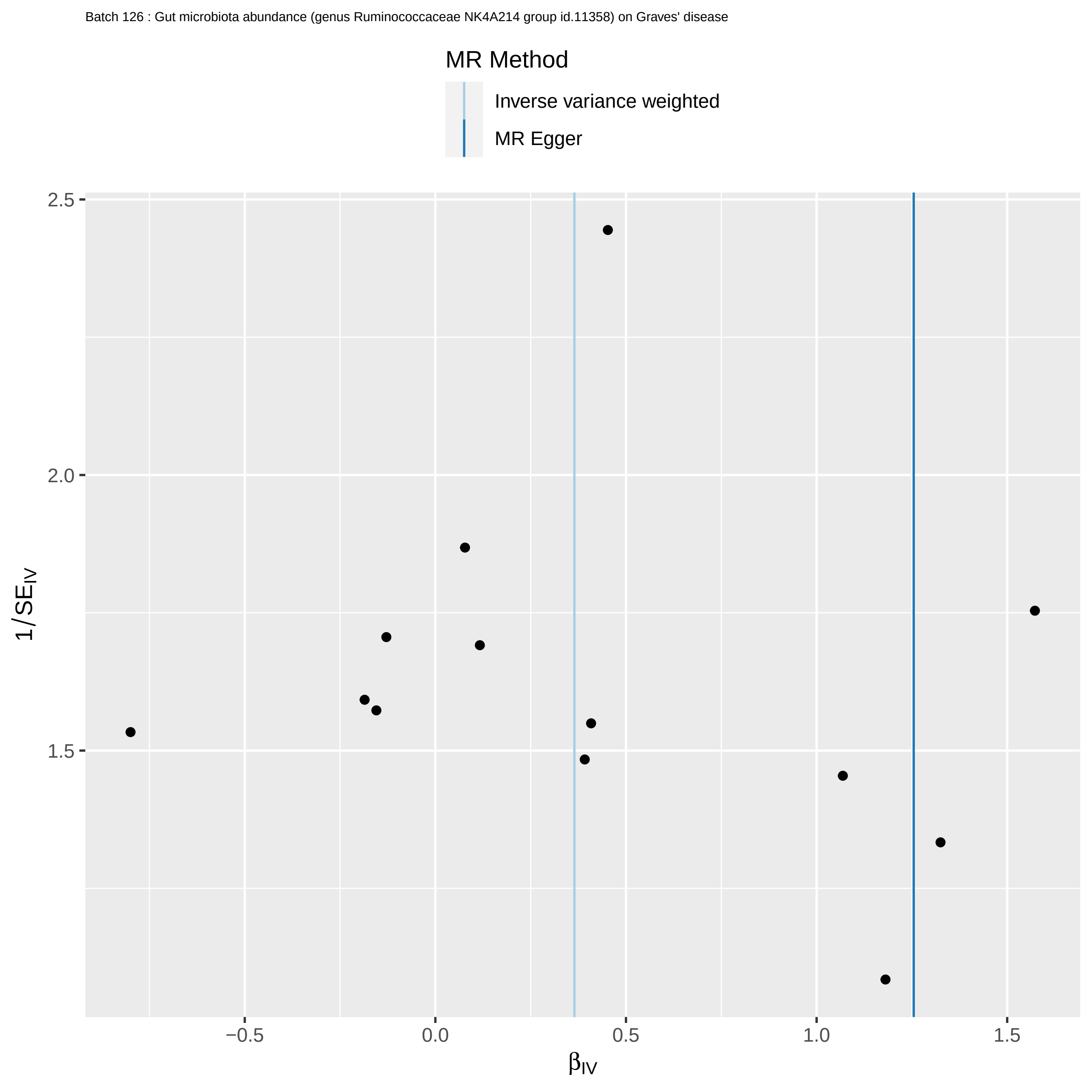

Batch 126 : Gut microbiota abundance (genus Ruminococcaceae NK4A214 group id.11358) on Graves' disease
MR Method
Inverse variance weighted
MR Egger
2.5
2.0
1.5
1/SE_IV
−0.5
0.0
0.5
1.0
1.5
β_IV

Batch 127 : Gut microbiota abundance (genus Ruminococcaceae UCG002 id.11360) on Graves' disease

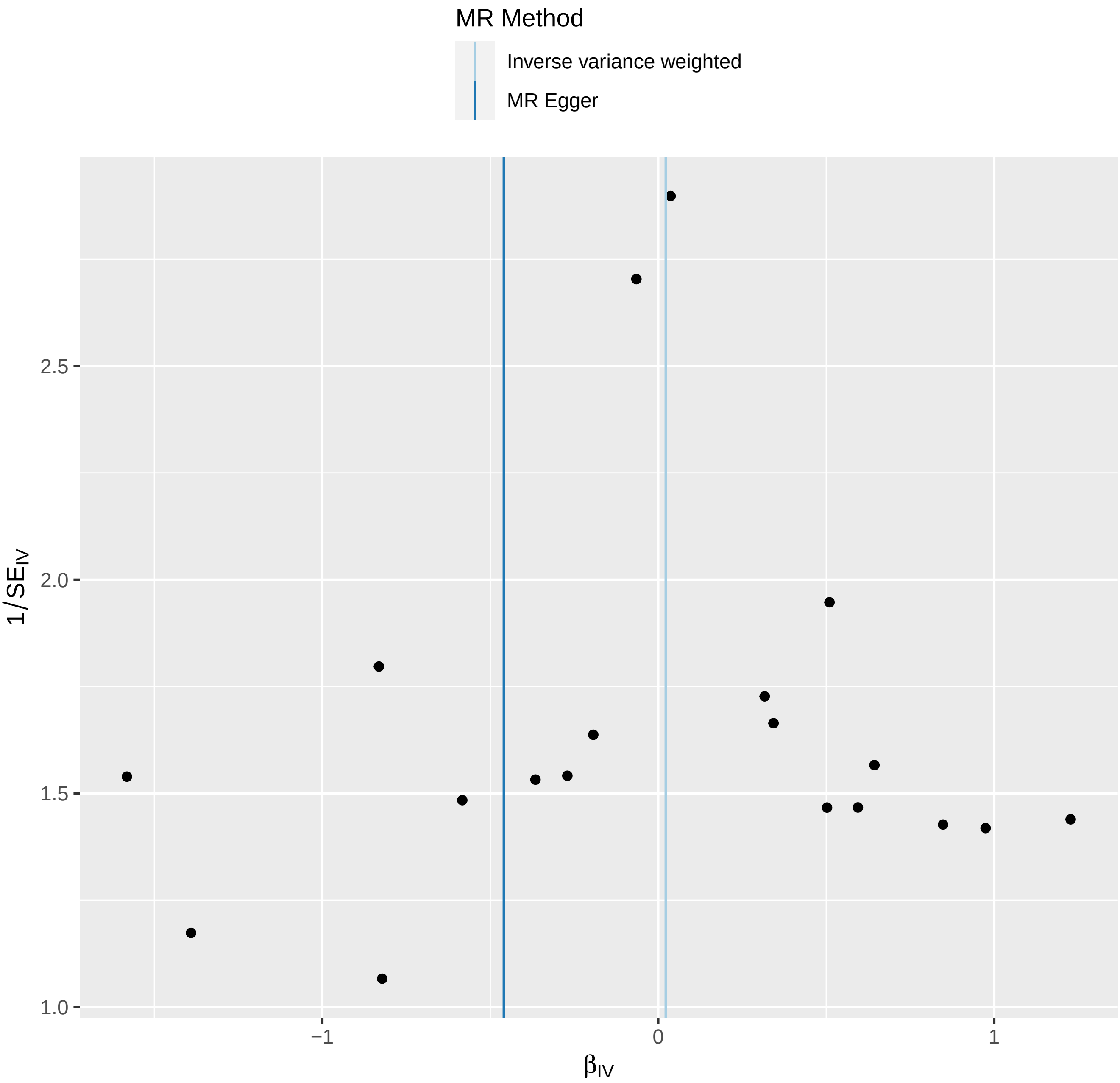

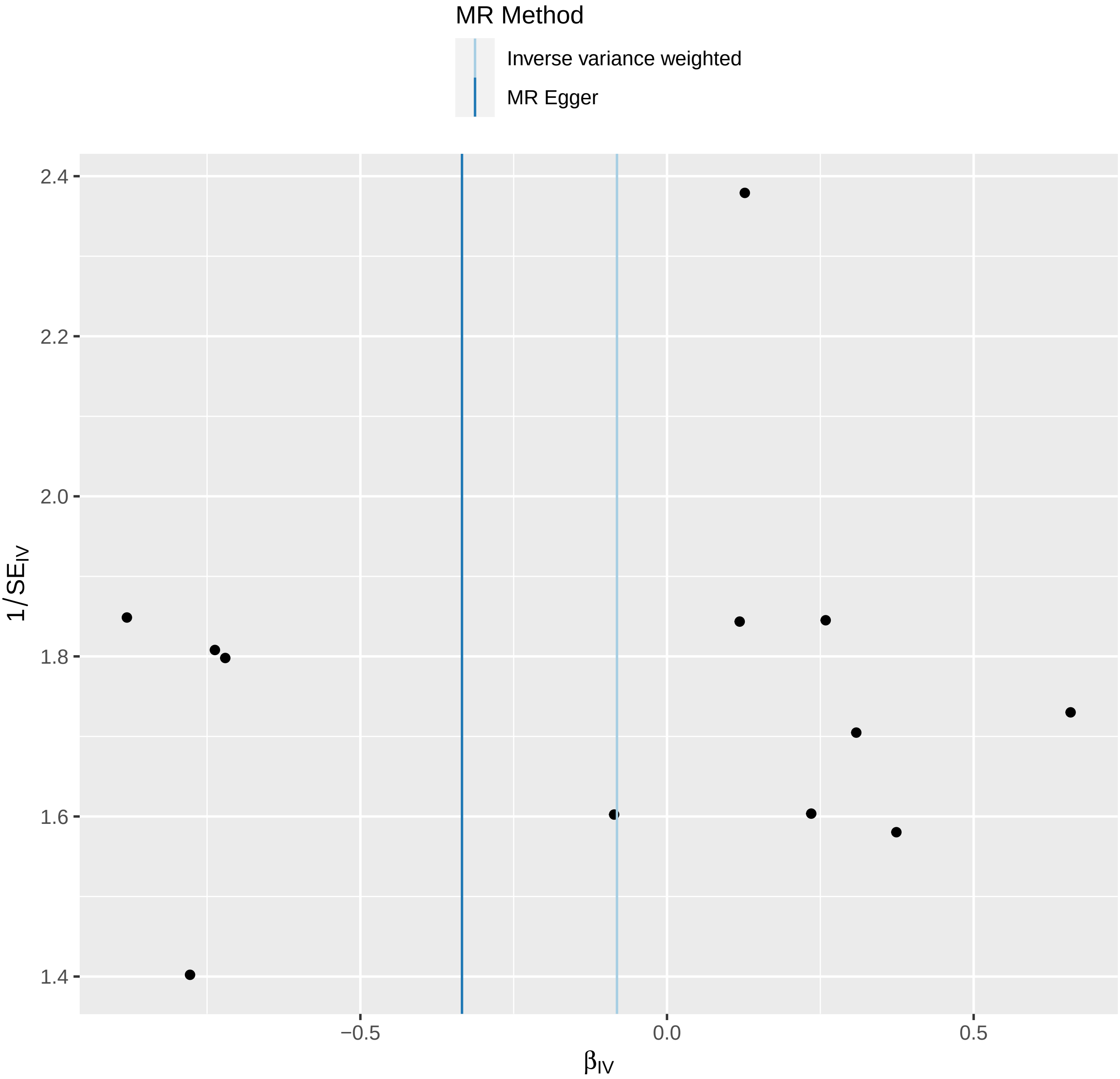

MR Method
Inverse variance weighted
MR Egger
2.4
2.2
2.0
1.8
1.6
1.4
1/SE_IV
−0.5
0.0
0.5
β_IV

Batch 129 : Gut microbiota abundance (genus Ruminococcaceae UCG004 id.11362) on Graves' disease

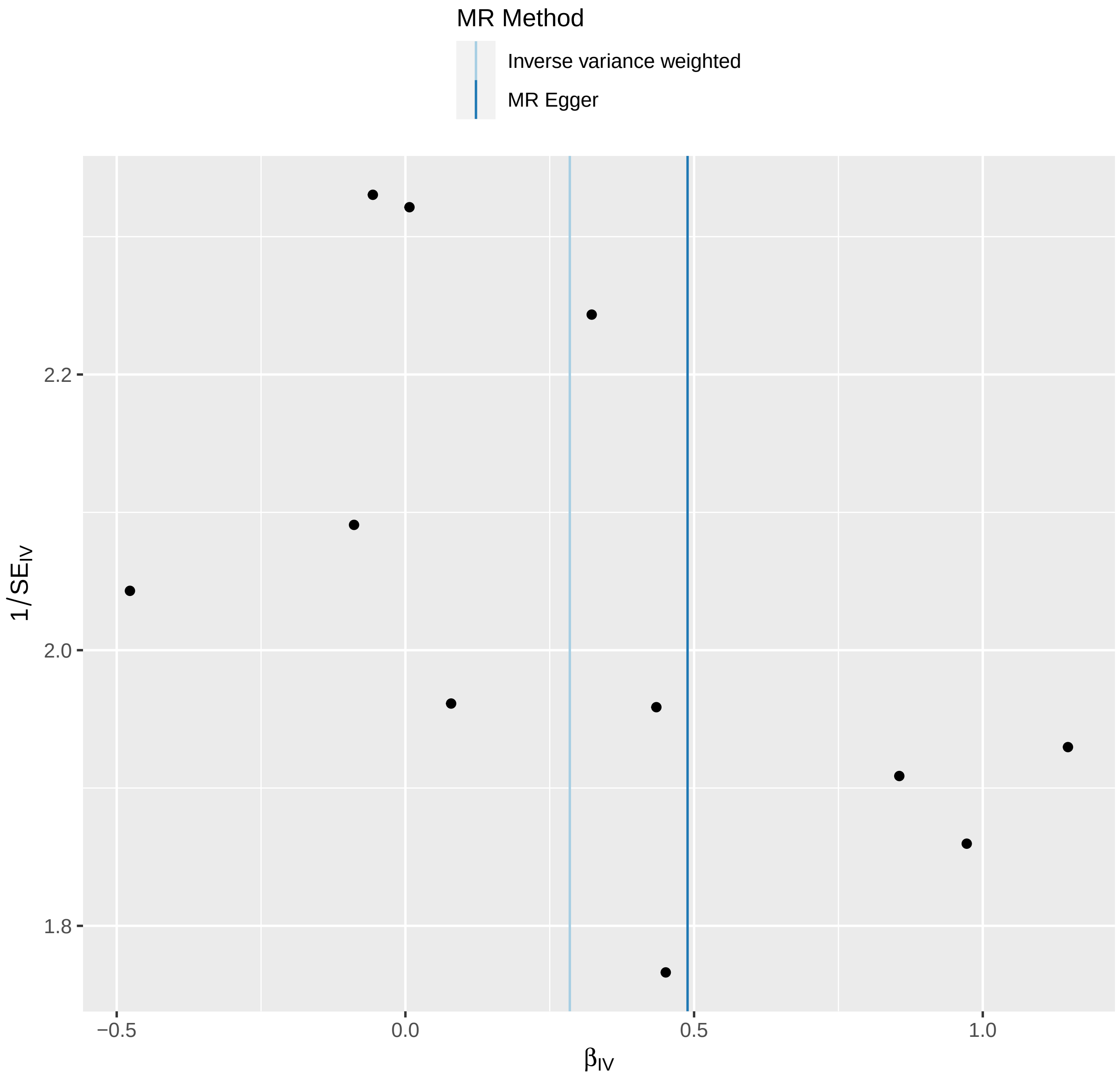

Batch 130 : Gut microbiota abundance (genus Ruminococcaceae UCG005 id.11363) on Graves' disease

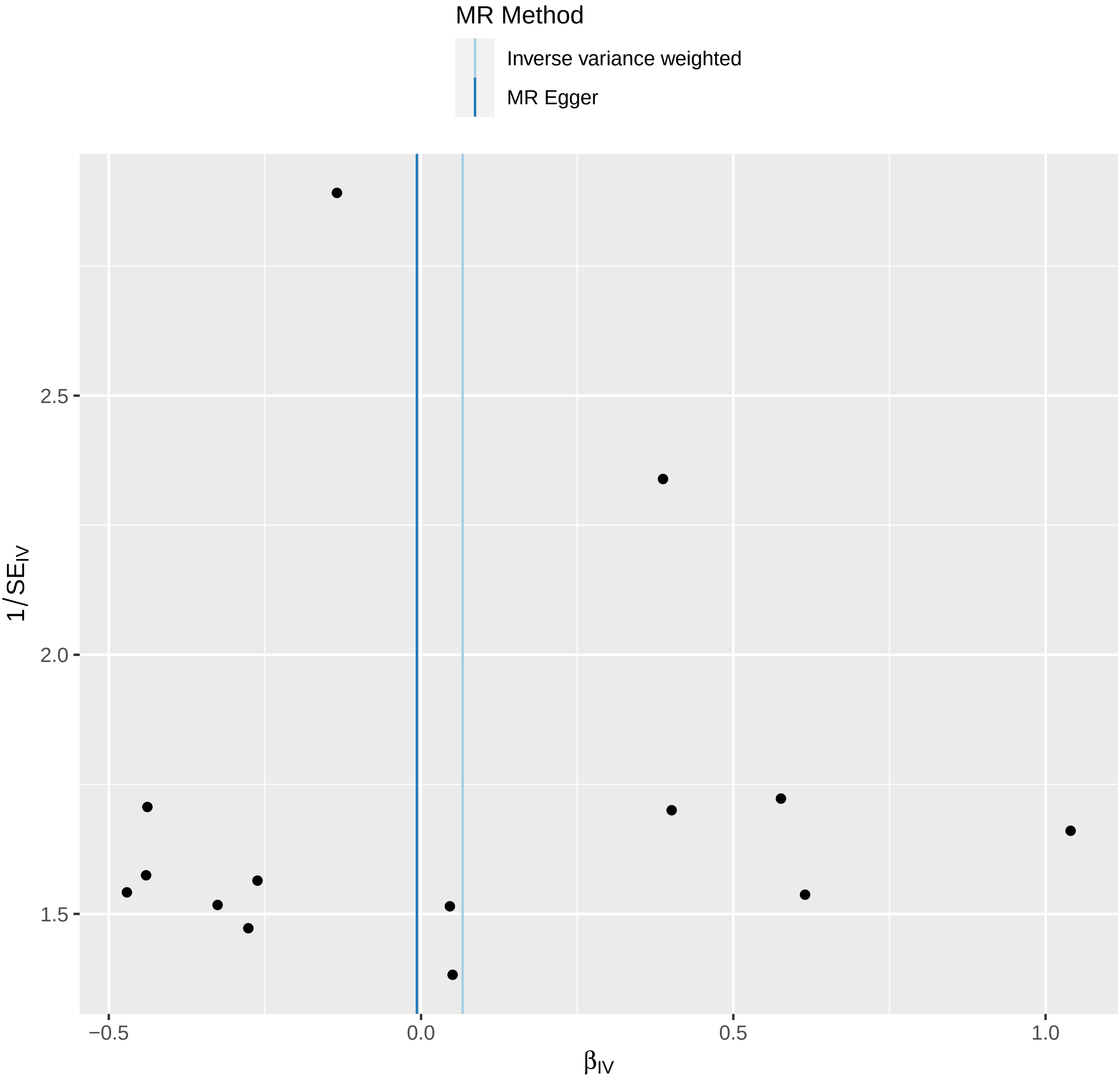

Batch 131 : Gut microbiota abundance (genus Ruminococcaceae UCG009 id.11366) on Graves' disease

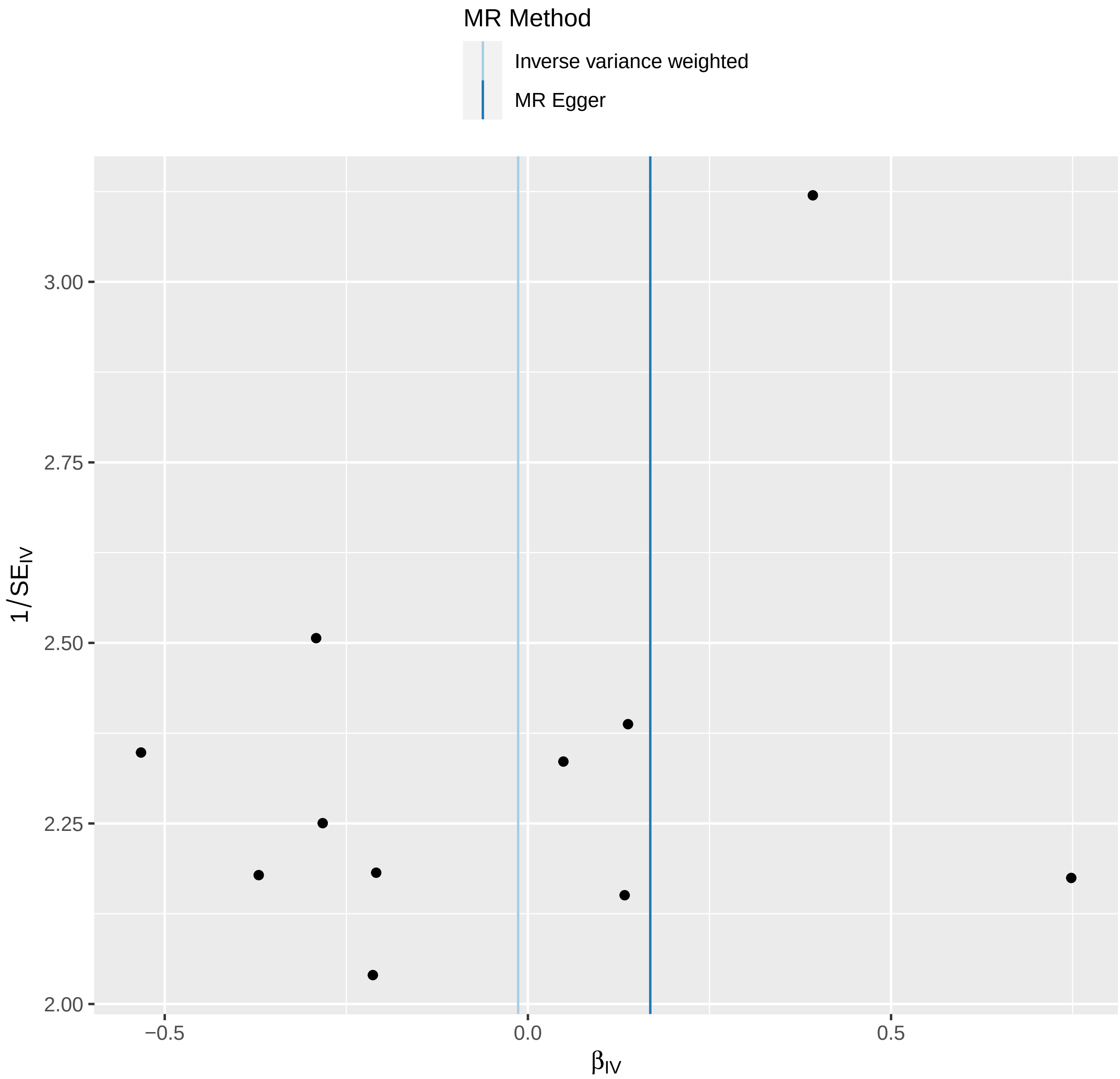

Batch 132 : Gut microbiota abundance (genus Ruminococcaceae UCG010 id.11367) on Graves' disease

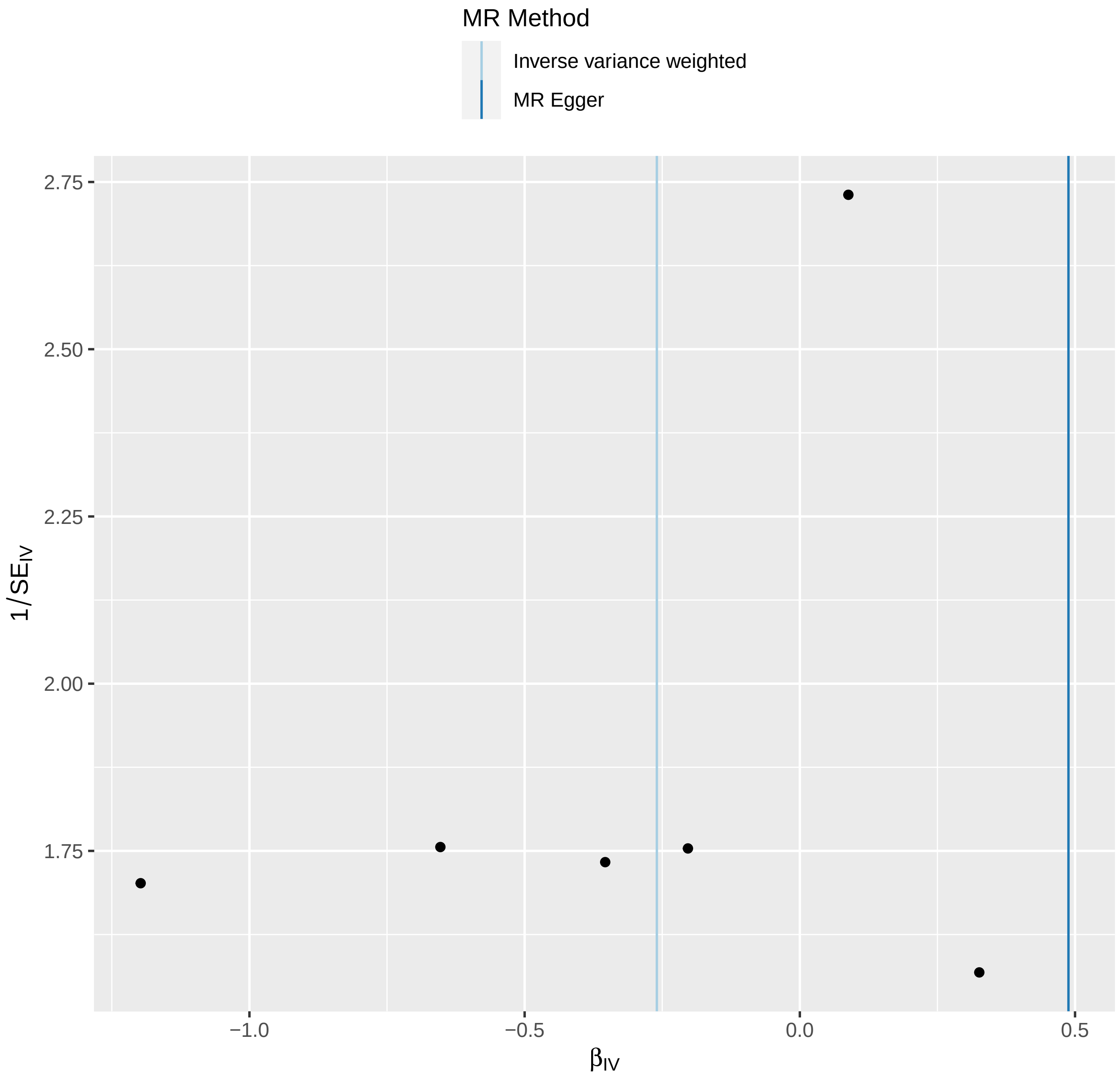

Batch 133 : Gut microbiota abundance (genus Ruminococcaceae UCG011 id.11368) on Graves' disease

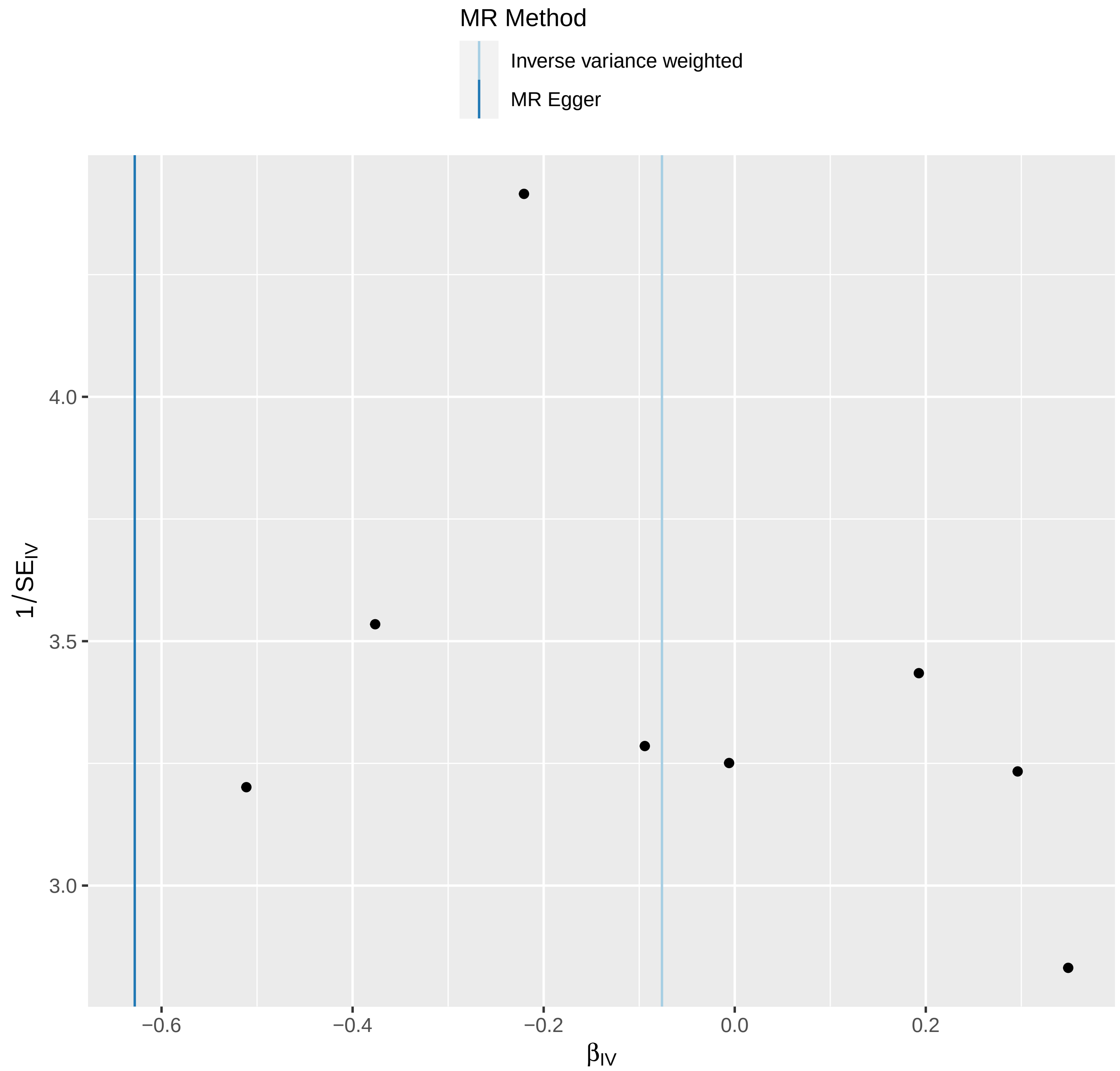

Batch 134 : Gut microbiota abundance (genus Ruminococcaceae UCG013 id.11370) on Graves' disease

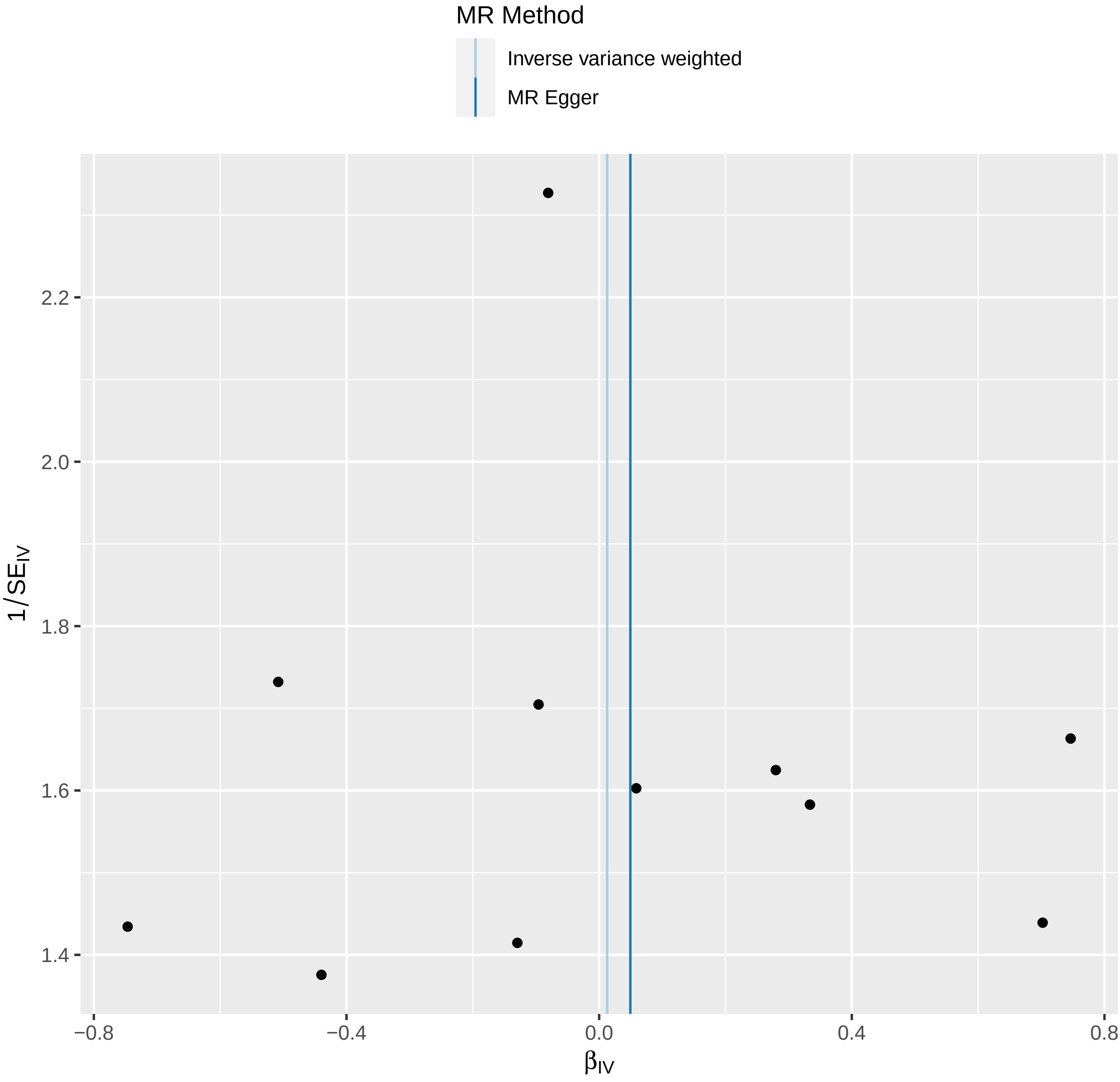

Batch 135 : Gut microbiota abundance (genus Ruminococcaceae UCG014 id.11371) on Graves' disease

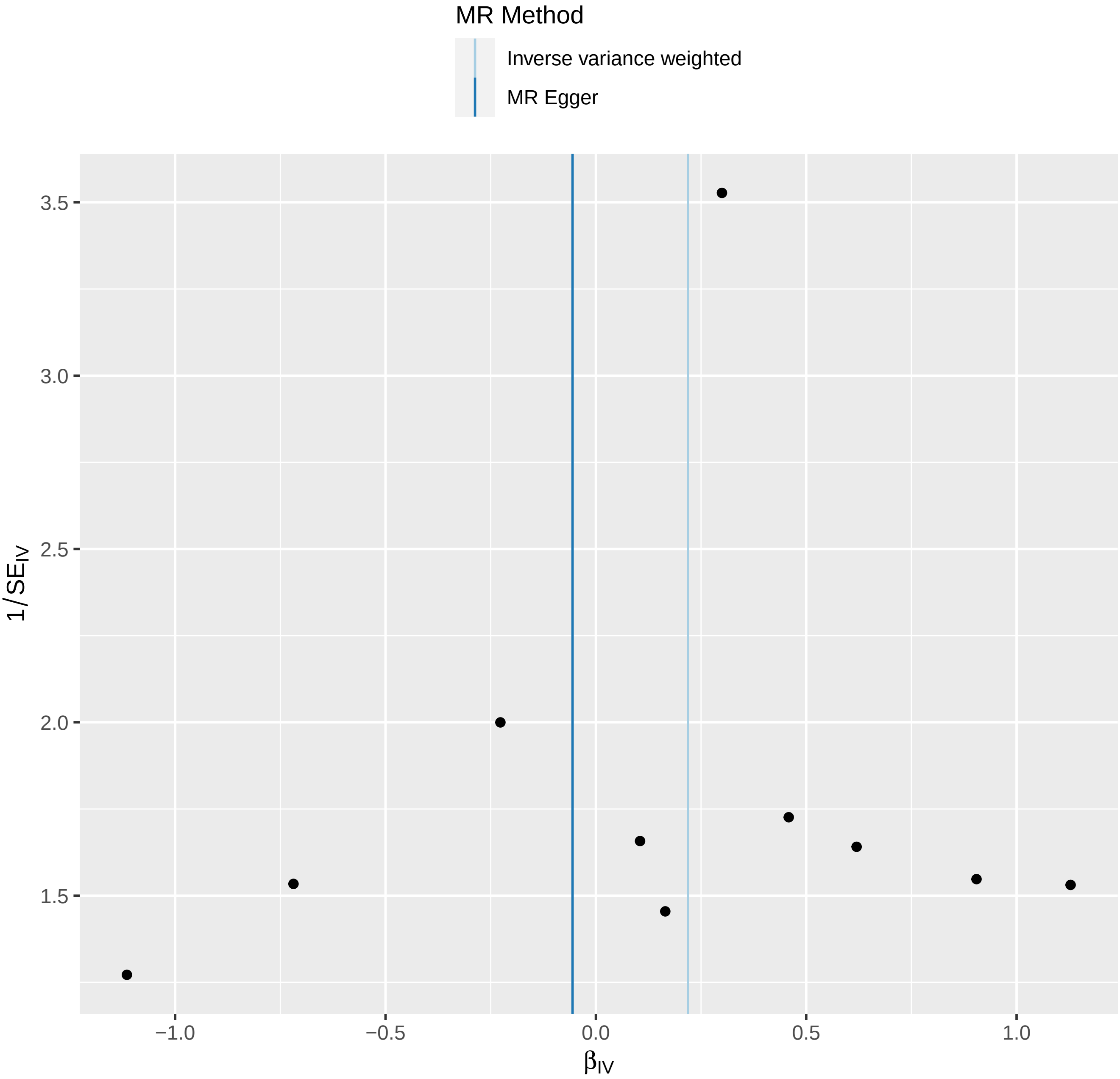

Batch 136 : Gut microbiota abundance (genus Ruminococcus1 id.11373) on Graves' disease

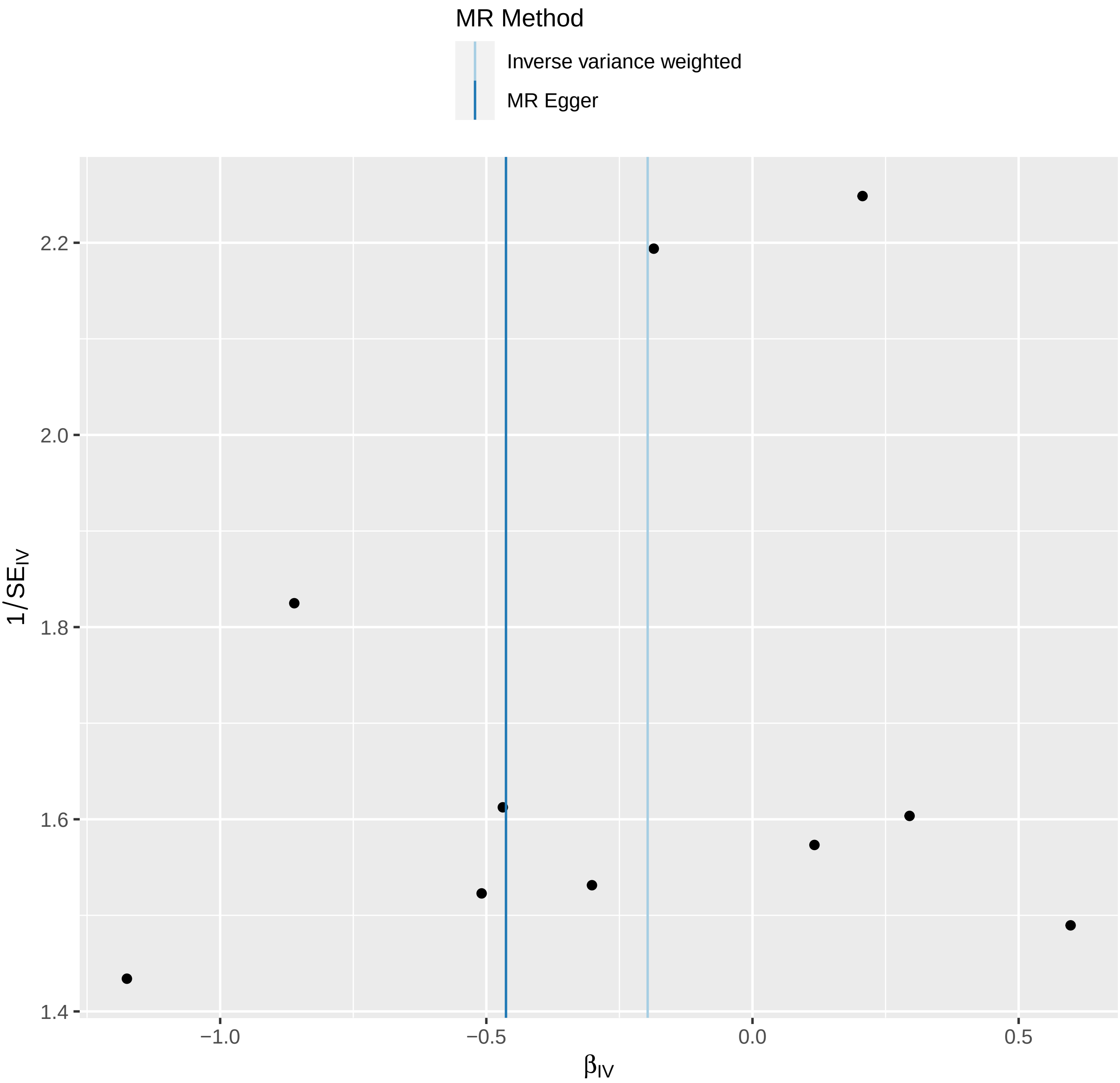

Batch 137 : Gut microbiota abundance (genus Ruminococcus2 id.11374) on Graves' disease

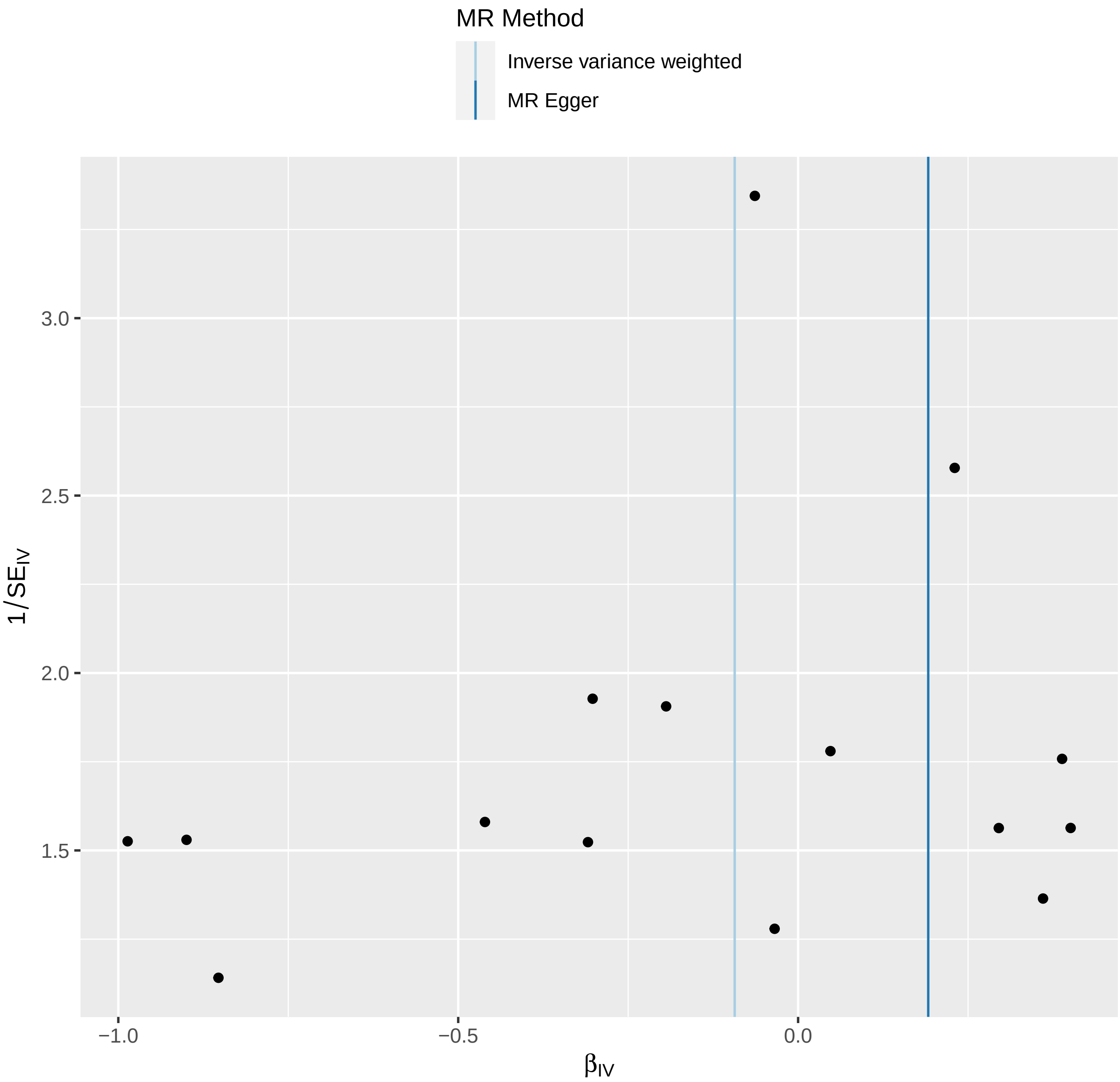

Batch 138 : Gut microbiota abundance (genus Ruminococcus gauvreauii group id.11342) on Graves' disease

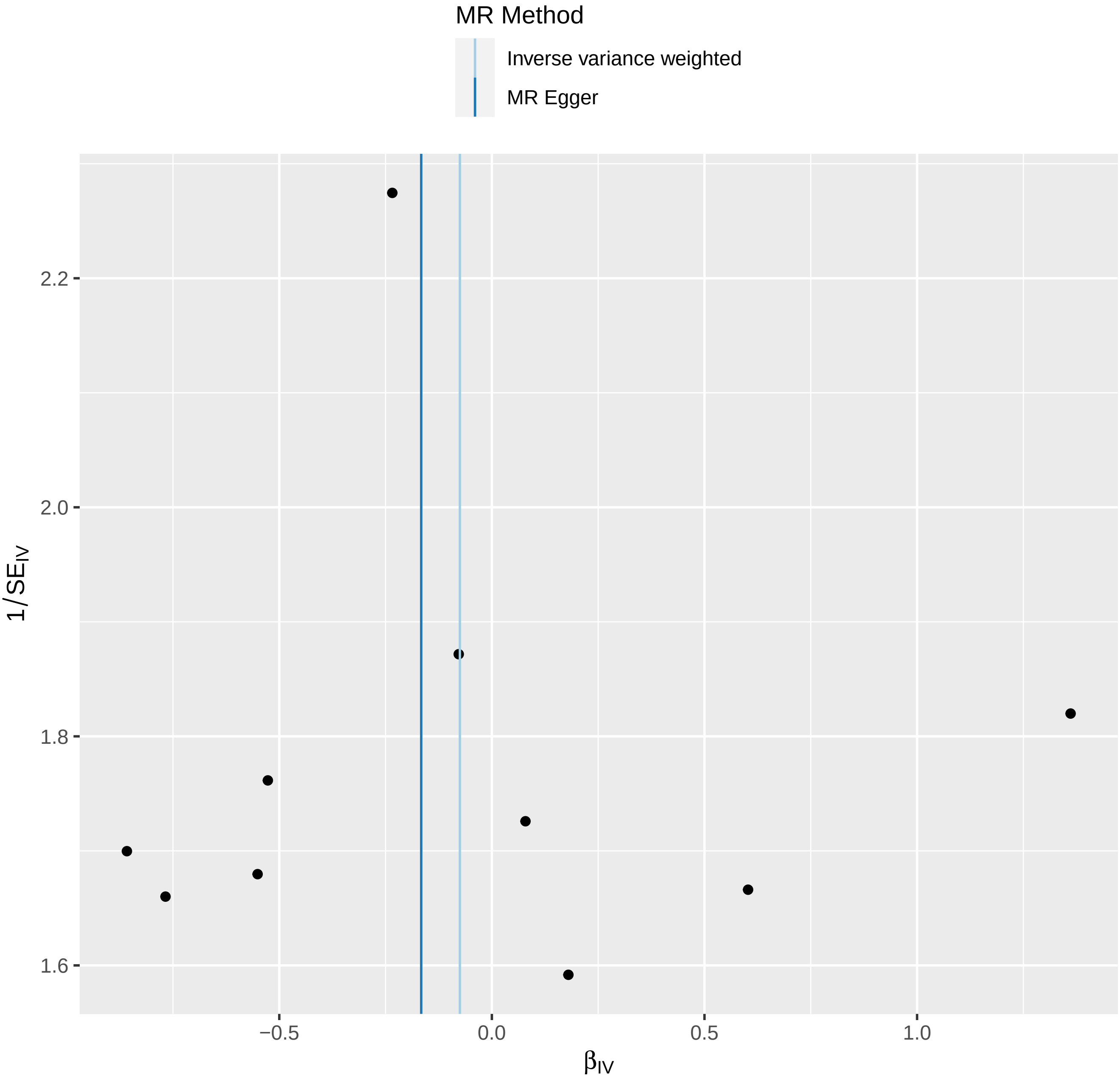

Batch 139 : Gut microbiota abundance (genus Ruminococcus gnavus group id.14376) on Graves' disease

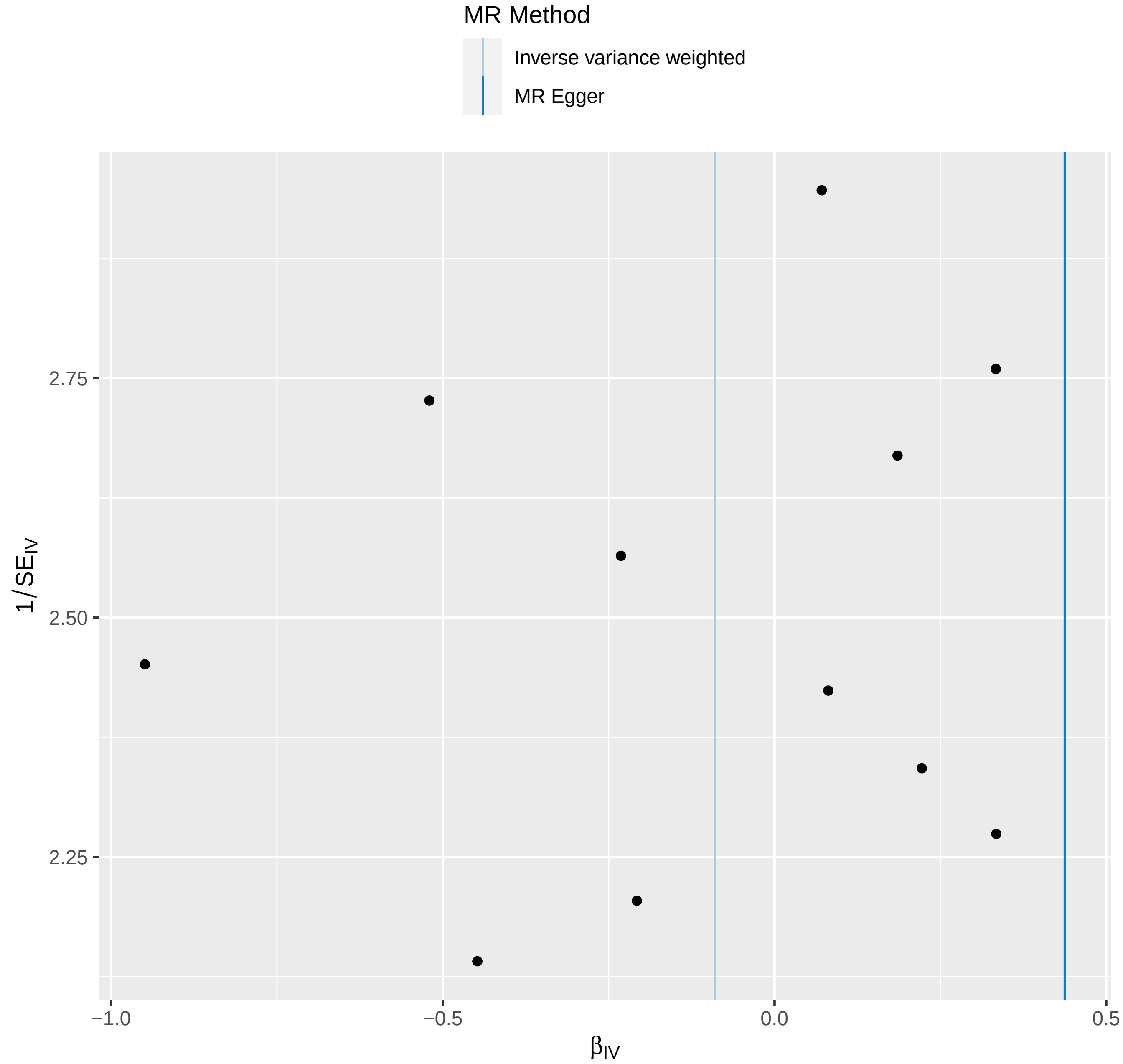

Batch 140 : Gut microbiota abundance (genus Ruminococcus torques group id.14377) on Graves' disease

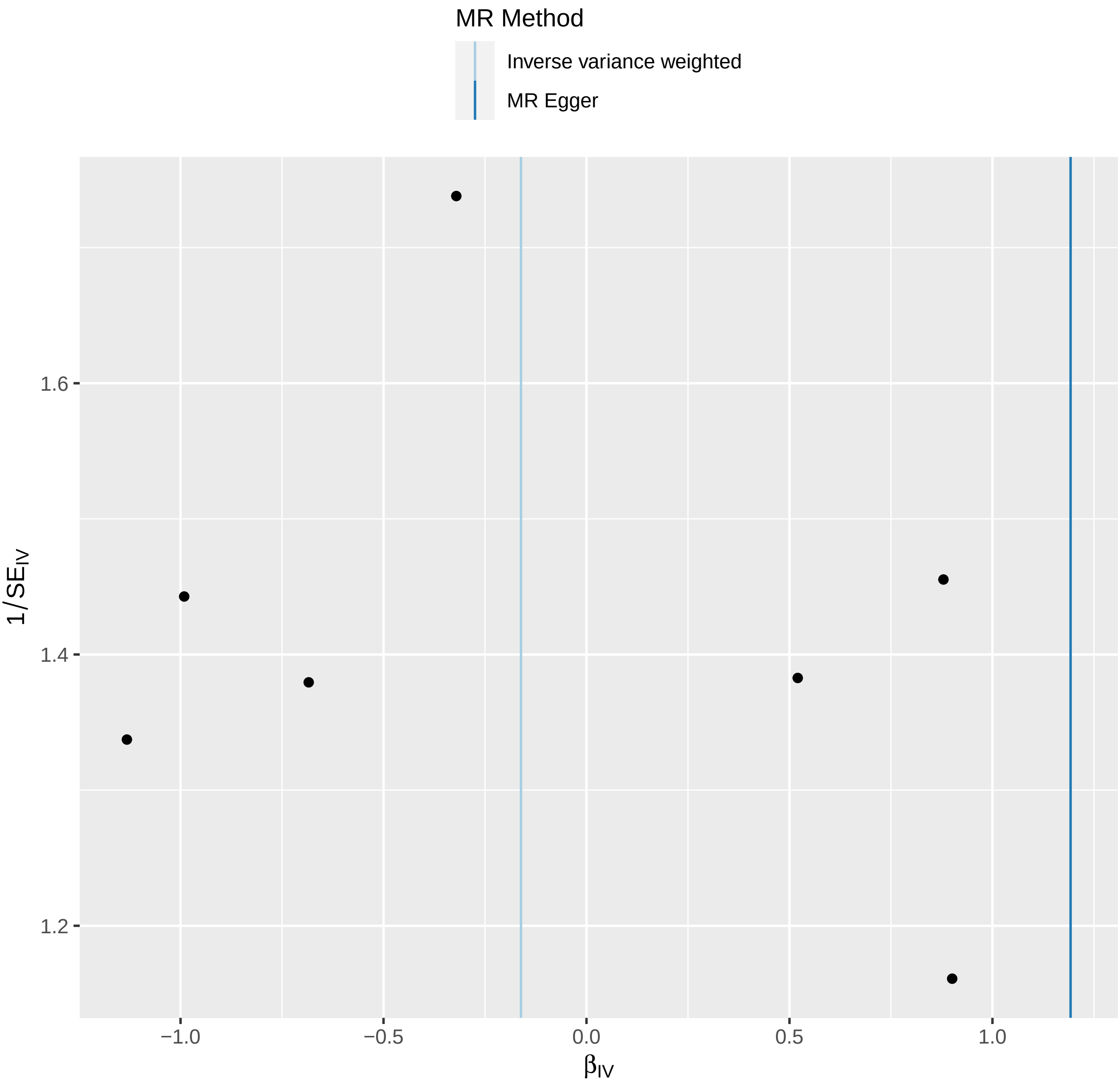

Batch 141 : Gut microbiota abundance (genus Sellimonas id.14369) on Graves' disease

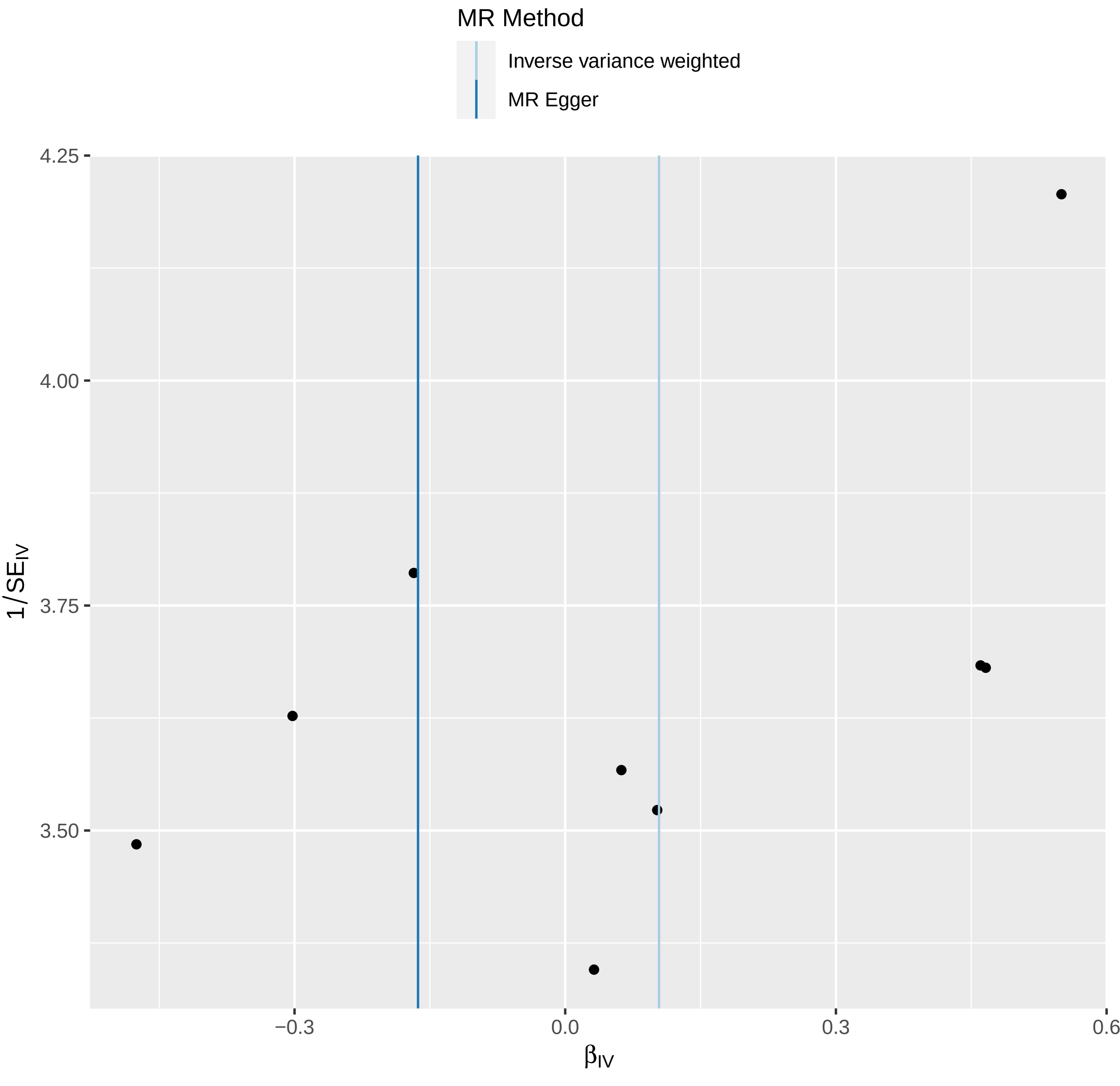

Batch 142 : Gut microbiota abundance (genus Senegalimassilia id.11160) on Graves' disease

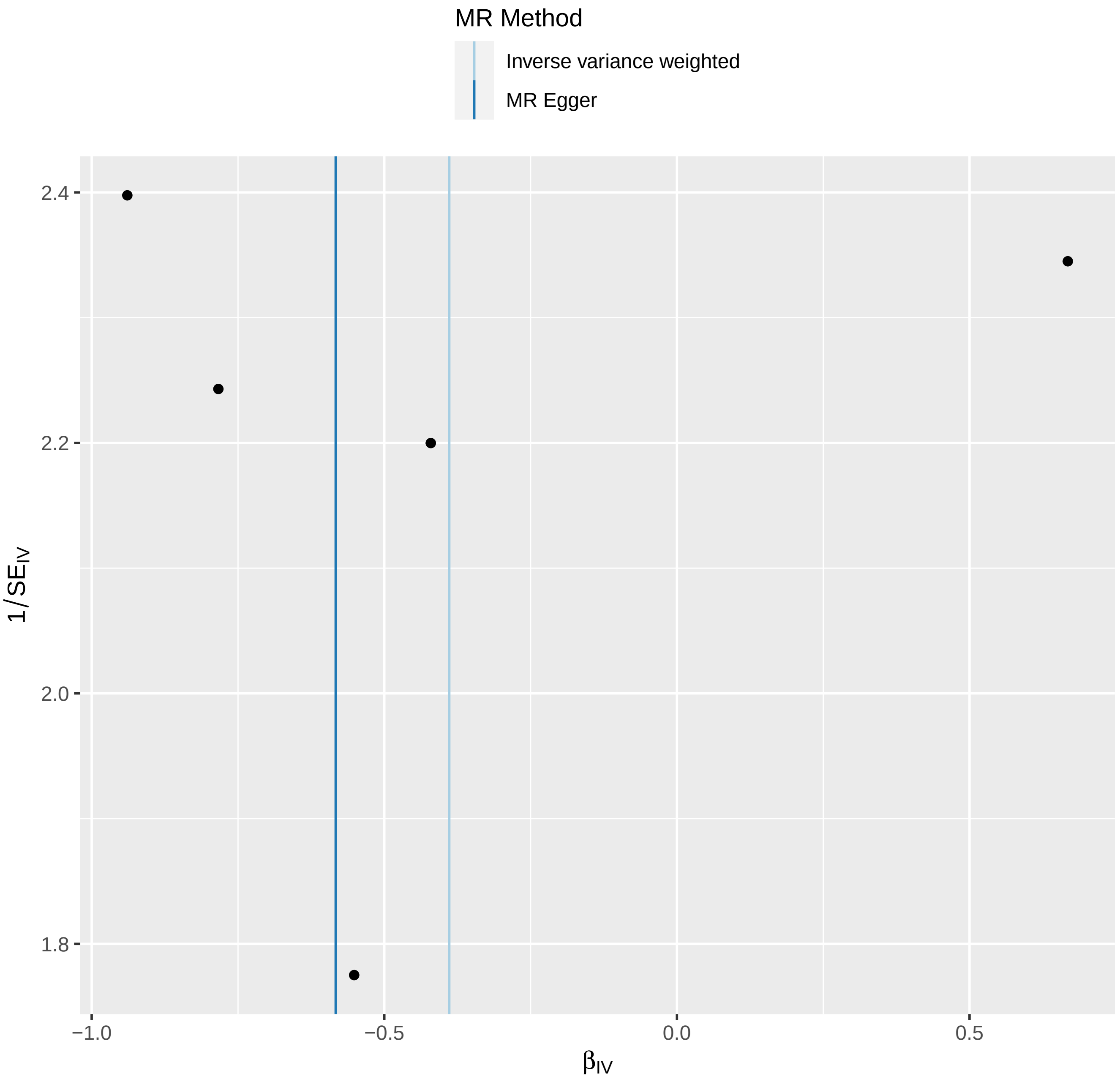

Batch 143 : Gut microbiota abundance (genus Slackia id.825) on Graves' disease

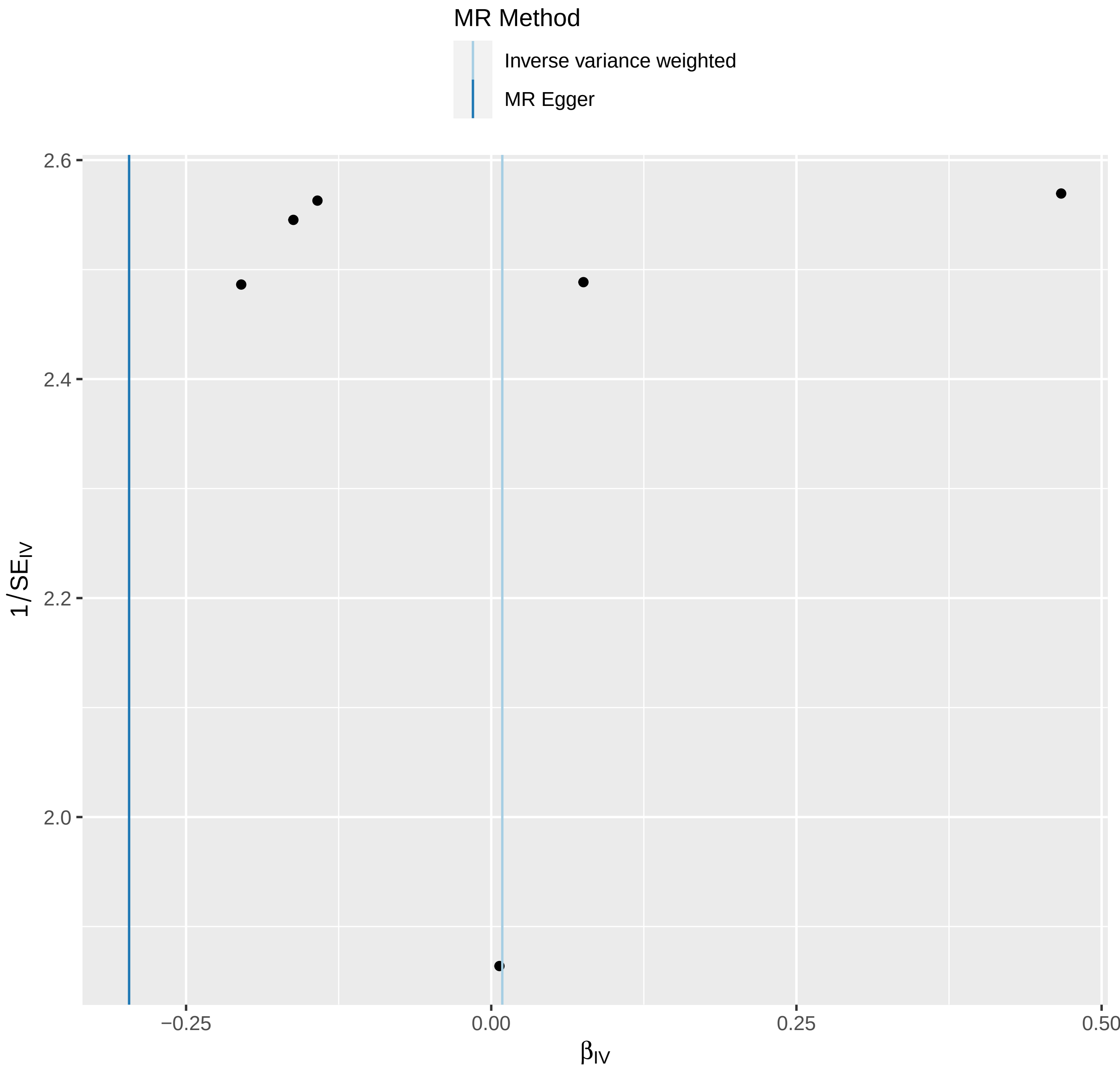

Batch 144 : Gut microbiota abundance (genus Streptococcus id.1853) on Graves' disease

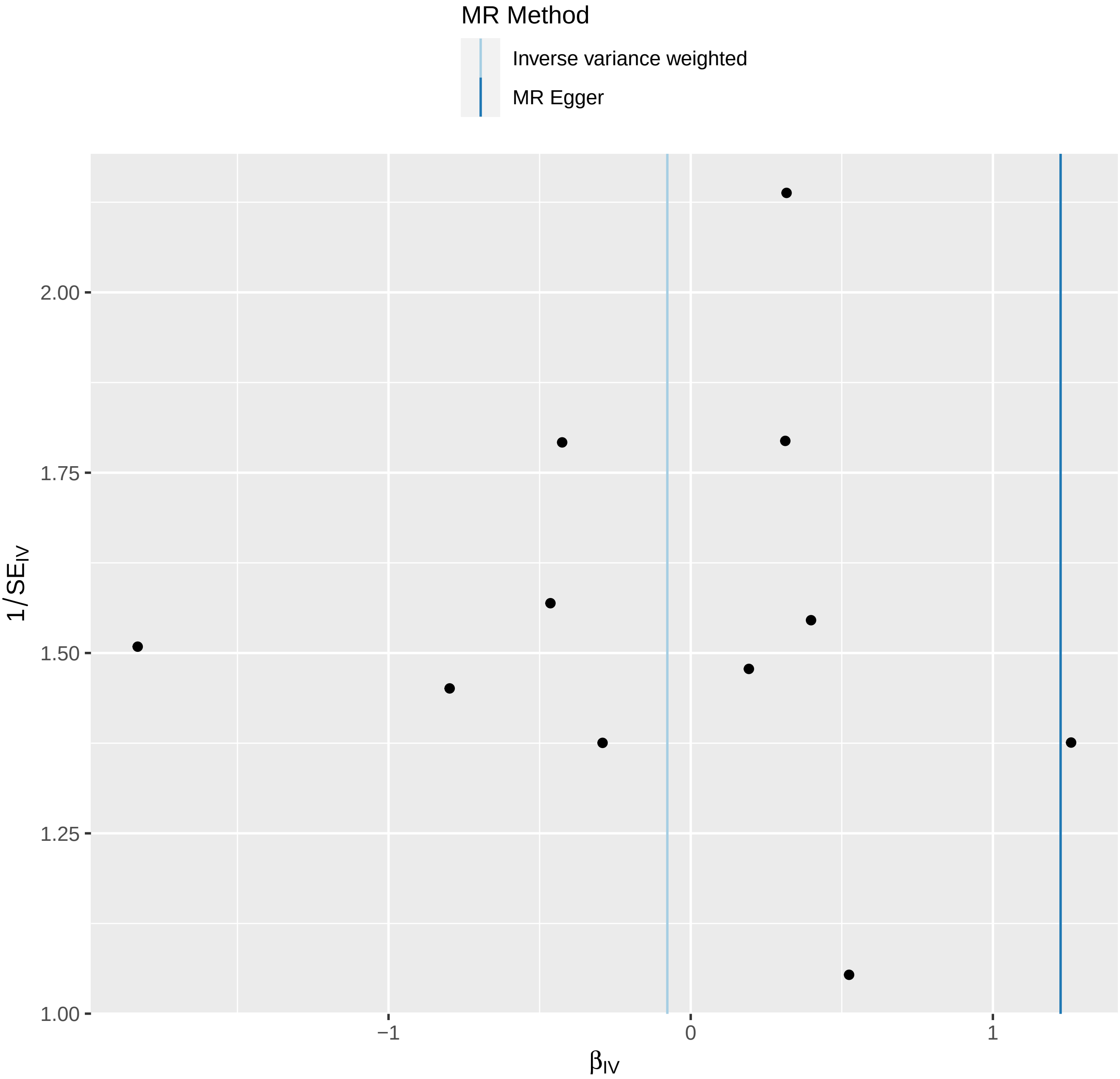

Batch 145 : Gut microbiota abundance (genus Subdoligranulum id.2070) on Graves' disease

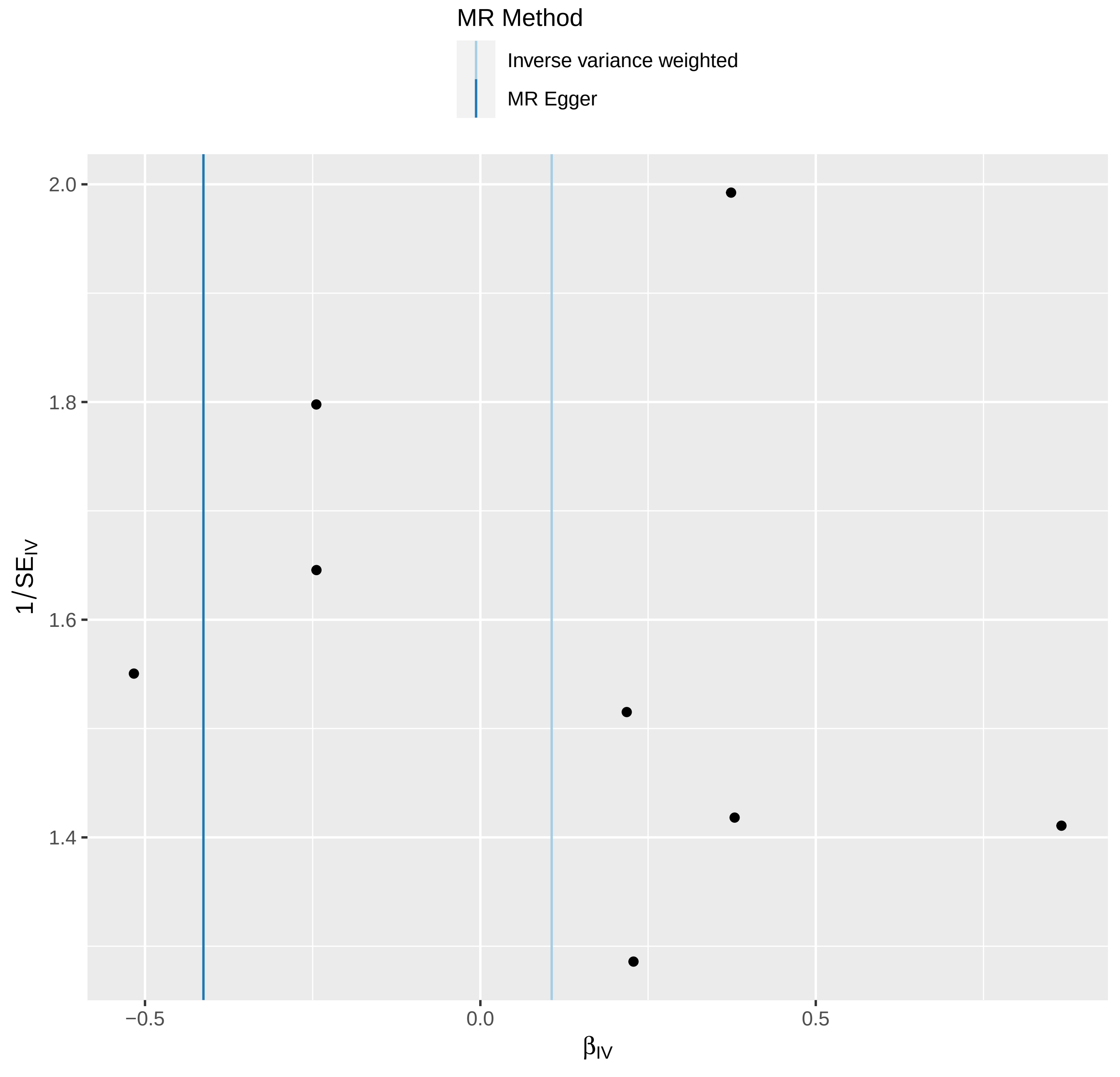

Batch 146 : Gut microbiota abundance (genus Sutterella id.2896) on Graves' disease

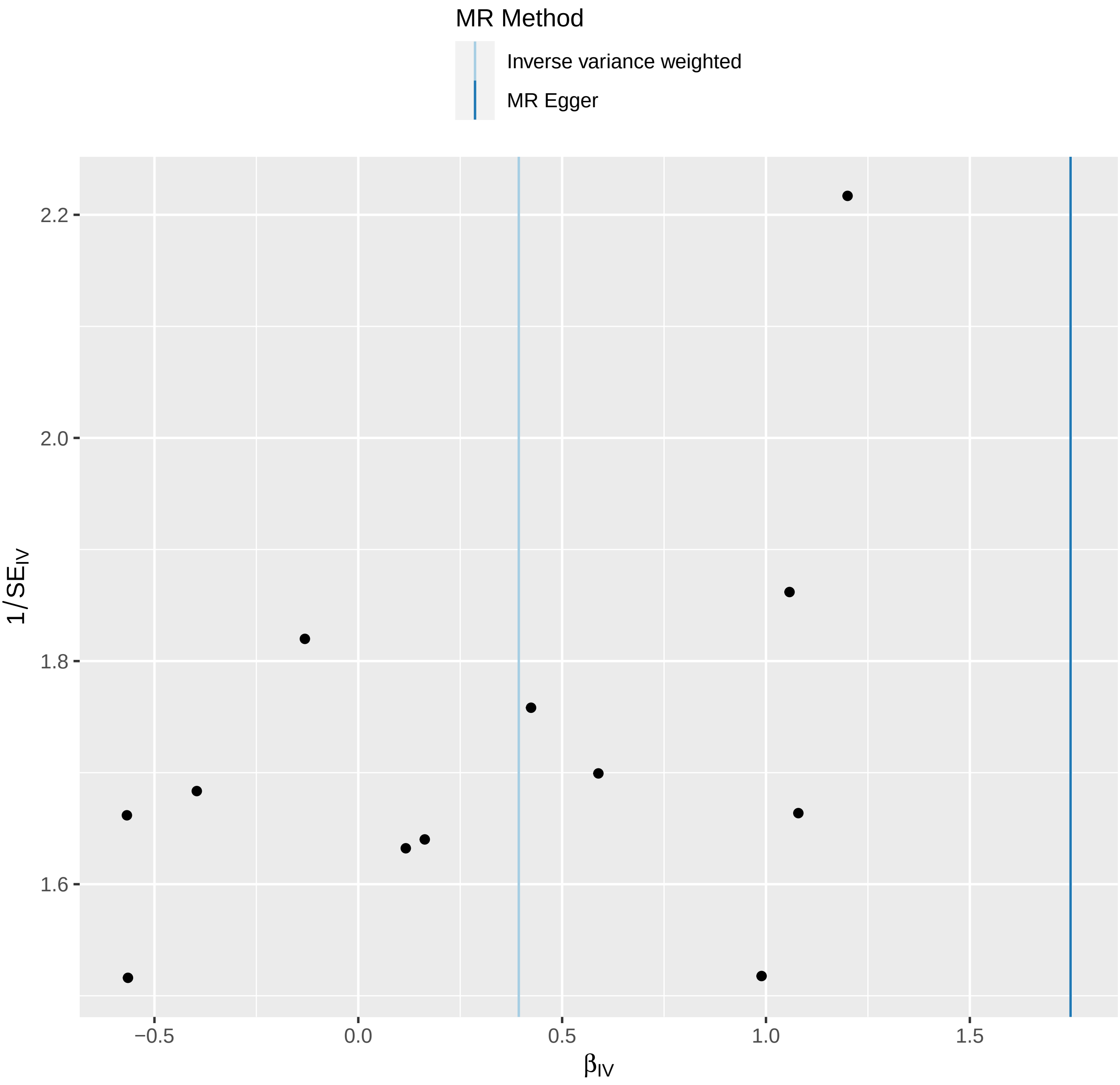

Batch 147 : Gut microbiota abundance (genus Terrisporobacter id.11348) on Graves' disease

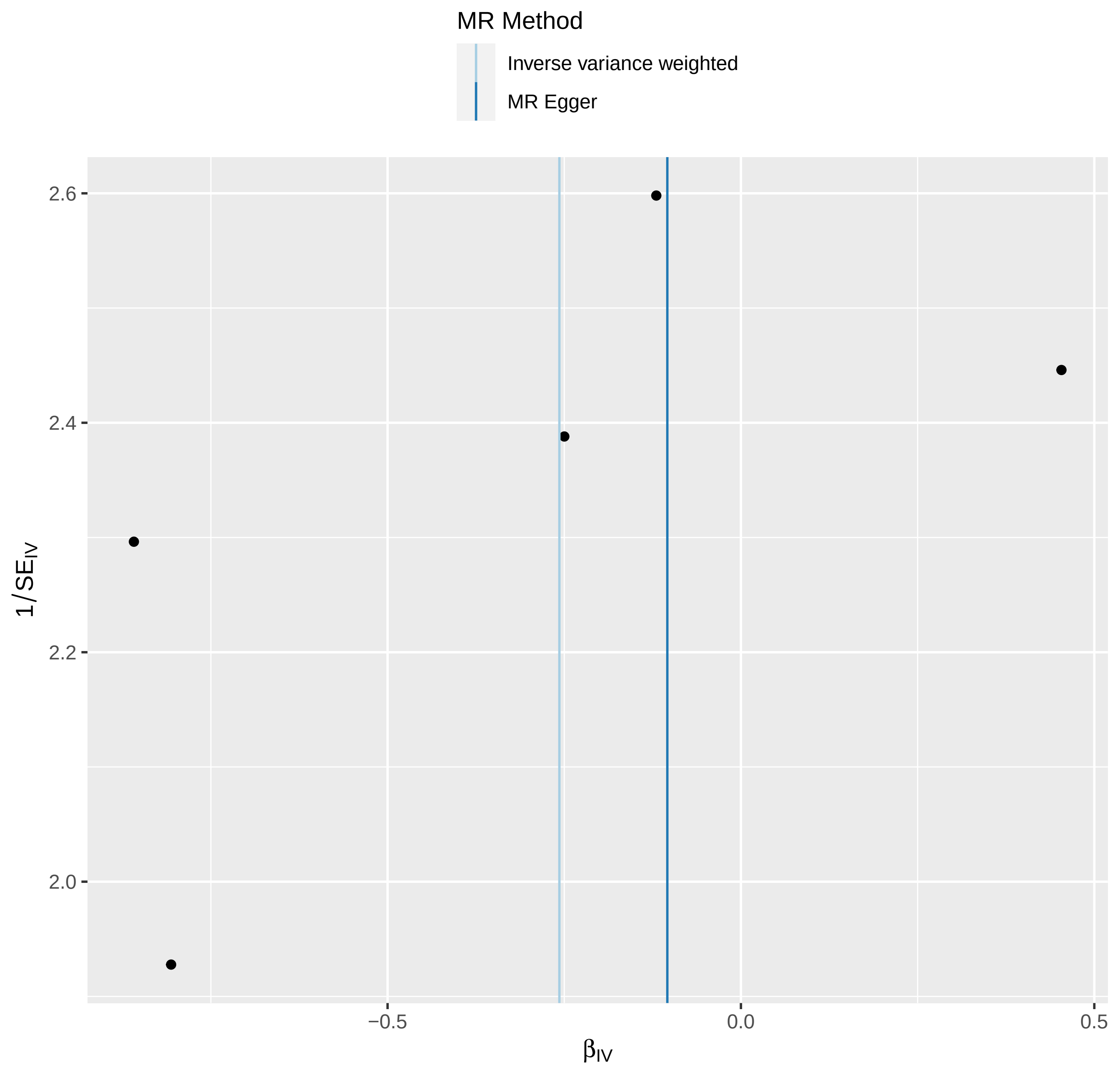

Batch 148 : Gut microbiota abundance (genus Turicibacter id.2162) on Graves' disease

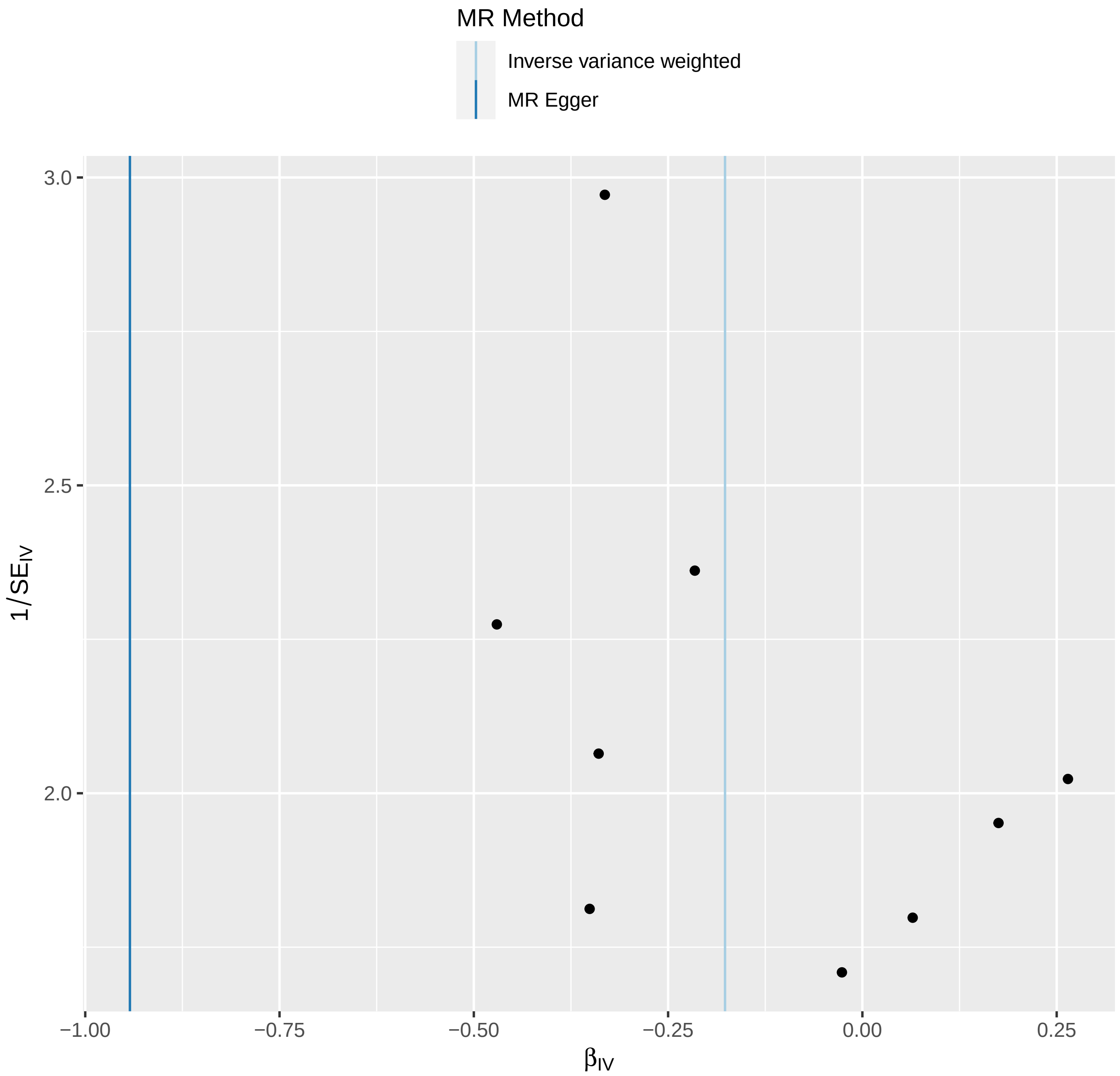

Batch 149 : Gut microbiota abundance (genus Tyzzerella3 id.11335) on Graves' disease

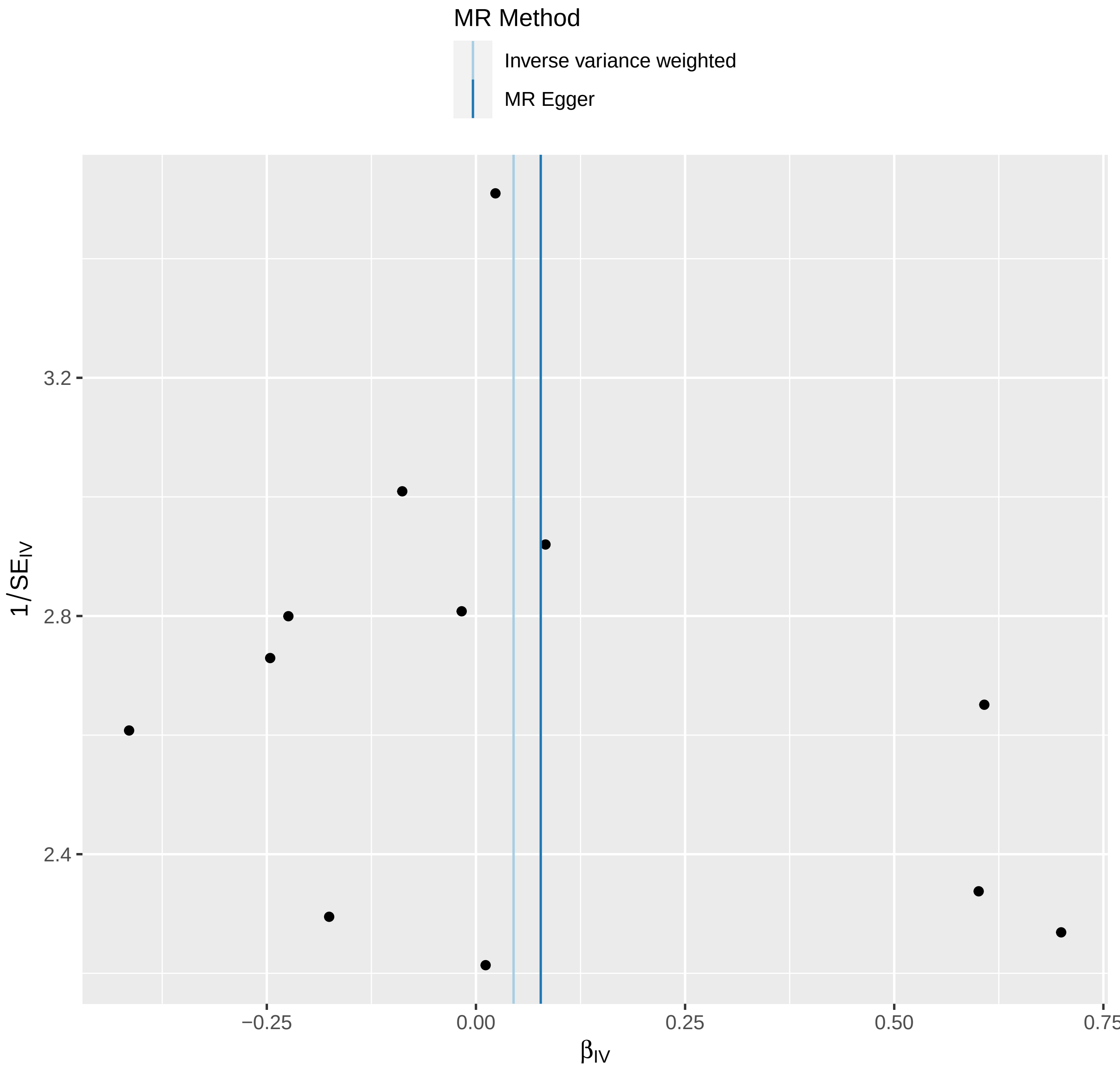

Batch 150 : Gut microbiota abundance (genus Veillonella id.2198) on Graves' disease

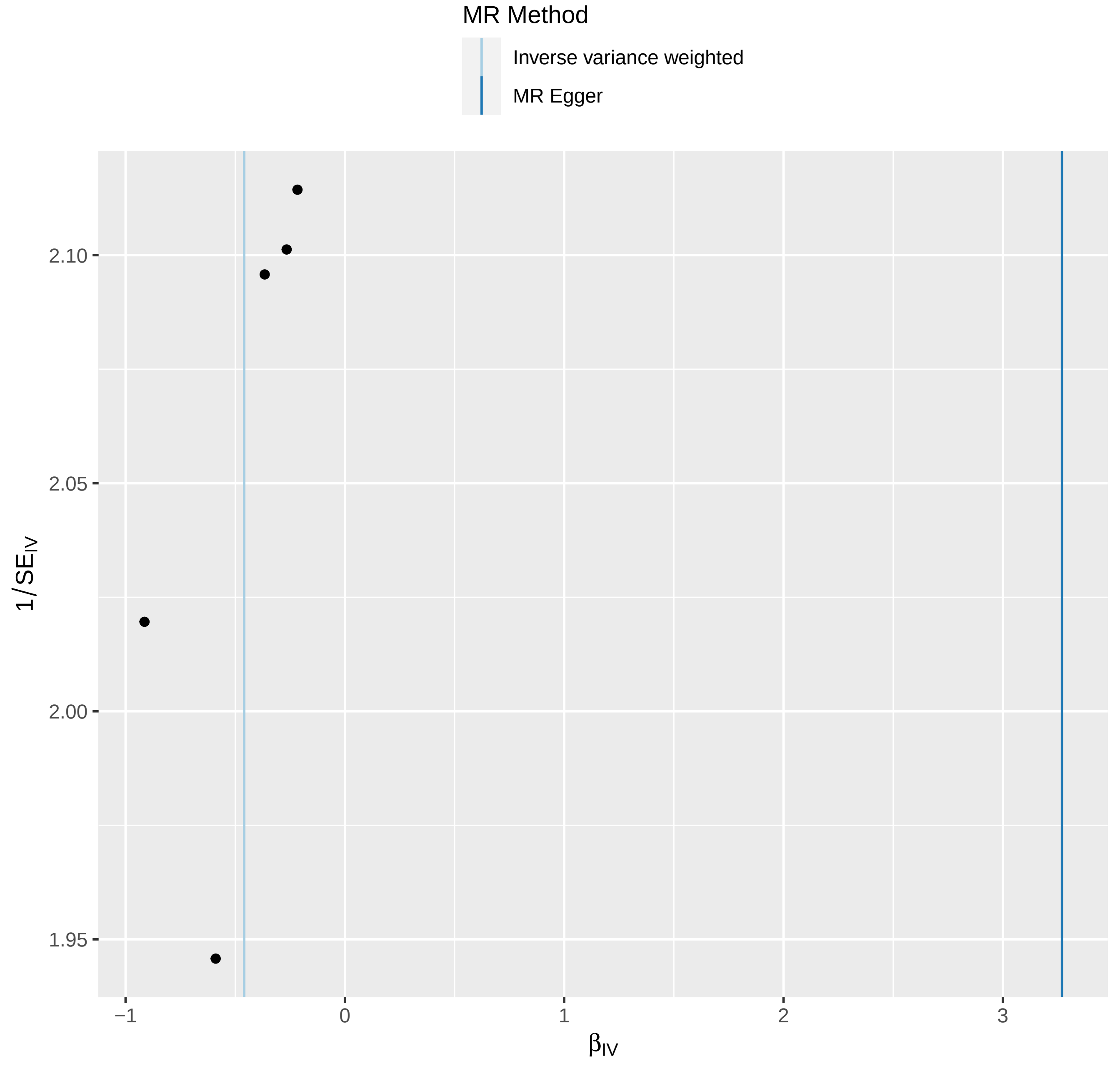

Supplement: Supplementary file 4 [file DataSheet_4.pdf]
